# Epigenetic diversity of genes with copy number variations among natural populations of the three‐spined stickleback

**DOI:** 10.1111/eva.13753

**Published:** 2024-07-14

**Authors:** Frédéric J. J. Chain, Britta S. Meyer, Melanie J. Heckwolf, Sören Franzenburg, Christophe Eizaguirre, Thorsten B. H. Reusch

**Affiliations:** ^1^ Department of Biological Sciences University of Massachusetts Lowell Lowell Massachusetts USA; ^2^ Marine Evolutionary Ecology GEOMAR Helmholtz Centre for Ocean Research Kiel Kiel Germany; ^3^ Institute of Clinical Molecular Biology, Kiel University Kiel Germany; ^4^ School of Biological and Behavioural Sciences Queen Mary University of London London UK; ^5^ Present address: Research Unit for Evolutionary Immunogenomics, Department of Biology University of Hamburg Hamburg Germany; ^6^ Present address: Fish Ecology and Evolution, Leibniz Centre for Tropical Marine Research Bremen Germany

**Keywords:** adaptive differentiation, copy number variations (CNVs), DNA methylation, epigenetic regulation, gene duplication, stickleback

## Abstract

Duplicated genes provide the opportunity for evolutionary novelty and adaptive divergence. In many cases, having more gene copies increases gene expression, which might facilitate adaptation to stressful or novel environments. Conversely, overexpression or misexpression of duplicated genes can be detrimental and subject to negative selection. In this scenario, newly duplicate genes may evade purifying selection if they are epigenetically silenced, at least temporarily, leading them to persist in populations as copy number variations (CNVs). In animals and plants, younger gene duplicates tend to have higher levels of DNA methylation and lower levels of gene expression, suggesting epigenetic regulation could promote the retention of gene duplications via expression repression or silencing. Here, we test the hypothesis that DNA methylation variation coincides with young duplicate genes that are segregating as CNVs in six populations of the three‐spined stickleback that span a salinity gradient from 4 to 30 PSU. Using reduced‐representation bisulfite sequencing, we found DNA methylation and CNV differentiation outliers rarely overlapped. Whereas lineage‐specific genes and young duplicates were found to be highly methylated, just two gene CNVs showed a significant association between promoter methylation level and copy number, suggesting that DNA methylation might not interact with CNVs in our dataset. If most new duplications are regulated for dosage by epigenetic mechanisms, our results do not support a strong contribution from DNA methylation soon after duplication. Instead, our results are consistent with a preference to duplicate genes that are already highly methylated.

## 1 INTRODUCTION

Gene duplication has long been recognized as an important process contributing to functional innovation, adaptation, and even speciation (Ohno, 1970; Taylor et al., 2001; Ting et al., 2004). In its most basic form, gene duplication can increase its transcriptional output, which selection might favor, for example, when organisms face new environmental challenges (Kondrashov, 2012; Riehle et al., 2001). The survival of a duplicated gene without modification by mutation or regulation is, however, expected to be rare, because duplication events that increase dosage are likely to adversely affect fitness (Adler et al., 2014) or to be deleterious if not counteracted by regulatory mechanisms that prevent overexpression (Ascencio et al., 2021; Qian et al., 2010). Temporary gene silencing of a newly duplicated gene could therefore allow mutations to arise without adversely affecting the organism. For example, epigenetic regulation of gene expression has been proposed as a rapid mechanism for adjusting dosage effects following gene duplication, at least for a subset of genes that would benefit from repression (Kenchanmane Raju et al., 2023; Rodin & Riggs, 2003). The evidence for this hypothesis remains elusive, as few studies have measured the degree to which epigenetic regulation affects newly duplicated genes across diverse environmental conditions, particularly those segregating as copy number variations (CNVs) in natural populations.

Variations in epigenetic modifications like DNA methylation are important contributors to phenotypic plasticity and evolutionary changes in natural populations (Chapelle & Silvestre, 2022; Heckwolf et al., 2020; Hu & Barrett, 2022; Sagonas et al., 2020). Moreover, epigenetic diversity itself may enable and drive adaptation and even speciation (Flores et al., 2013; Lamka et al., 2022; Vernaz et al., 2022; Weiner & Katz, 2021). Despite ontogenic remodeling, some DNA methylation marks may be heritable (Heckwolf et al., 2020; Villicaña & Bell, 2021; Zhang & Sirard, 2021), making them potentially important regulators at the onset of duplicate gene evolution. Promoter DNA methylation can reduce expression of duplicate genes in animals (Chang & Liao, 2012), and there is evidence from various species that DNA methylation levels, as measured by the percent methylation at each CpG site, are higher among young duplicate genes compared to older genes (Dyson & Goodisman, 2020; Fang et al., 2018; Huang & Chain, 2021; Keller & Yi, 2014; Wang et al., 2016; Zhong et al., 2016). The association between DNA methylation and the repression of duplicate gene expression has been suggested to play an important role in their retention via dosage maintenance and their subsequent diversification (Chang & Liao, 2012; Kenchanmane Raju et al., 2023; Rodin & Riggs, 2003). In support of this, young duplicated genes in stickleback have low expression and high DNA methylation levels (Huang & Chain, 2021), suggesting rapid repression of extra gene copies after duplication or preferential duplication of loci that already have high levels of methylation. However, previous studies did not directly compare DNA methylation levels among individuals with known differences in gene copy number, because the CNV data and DNA methylation data came from different sets of individuals. Since none of the individuals surveyed had both their genome and epigenome sequenced, it remains uncertain whether the observed population‐wide variation in DNA methylation levels among duplicate genes is a result of gene duplication, a contributing factor to duplication, or merely coincidental. What is missing is an analysis of DNA methylation levels in individuals that carry extra gene copies versus individuals without duplications to establish the direct relationship between variation in DNA methylation levels and gene duplication. It is therefore of interest to characterize the epigenetic dynamics of recent gene duplicates that are still copy number polymorphic within populations (i.e., CNVs), by surveying new duplications and their DNA methylation levels in the same individuals.

The three‐spined stickleback (*Gasterosteus aculeatus*) is a compelling study system for answering questions about the interplay between DNA methylation and CNVs in the context of rapid and adaptive evolution: there exist differentiated populations that have repeatedly adapted to various environmental conditions, offering an opportunity to simultaneously quantify divergences in DNA methylation levels and copy number variations. In sticklebacks, genome‐wide profiles of DNA methylation can vary by temperature, salinity, and infection (Artemov et al., 2017; Fellous et al., 2022; Heckwolf et al., 2020; Metzger & Schulte, 2018; Sagonas et al., 2020; Smith et al., 2015). The study of CNVs allows us to capture recently emerged duplicated genes that are still polymorphic in their copy numbers among populations, of which there are hundreds in sticklebacks (Chain et al., 2014). In this study, we describe the genome‐wide distribution of copy number differences among six populations of the three‐spined stickleback in the Baltic and North Sea across a salinity gradient, in relation to DNA methylation levels and the extent of methylation divergence across the genome. We expected some CNVs to be involved in population differentiation, and that higher gene copy number would be associated with higher levels of DNA methylation in gene promoters.

## 2 METHODS

In the current study, we use Whole Genome Sequencing (WGS) and Reduced Representation Bisulfite Sequencing (RBBS) data that builds on and expands the DNA methylation sequencing dataset by Heckwolf et al. (2020), as described below. We provide a summary of the methods used in that study, which analyzed 3 out of the 6 populations in the current study, as well as the data integration and additional analyses performed here.

### 2.1 Stickleback samples

**FIGURE 1:**
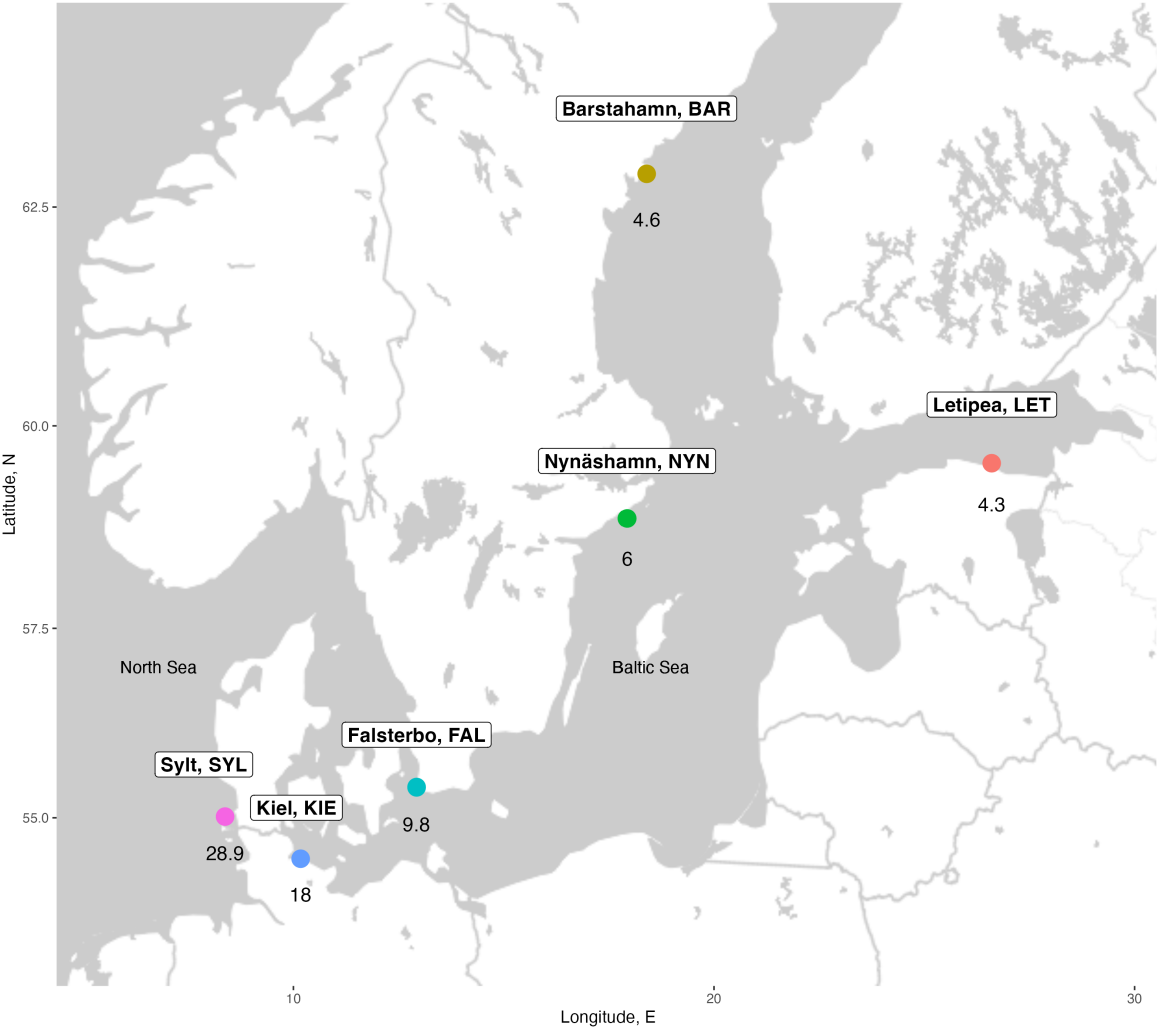
Six sampling locations for three‐spined stickleback along a salinity gradient in the Baltic Sea and North Sea. The number below each location represents the practical salinity unit (PSU).

Ninety‐six three‐spined stickleback fish (*Gasterosteus aculeatus*) were collected from five different sites in the Baltic Sea region and one from the North Sea in September 2014, and preserved in RNAlater solution at −20°C. The sites were selected based on their geographical location and unique ecological niches with different salinity levels (4–30 PSU) in Germany, Sweden, and Estonia (Figure 1). We therefore refer to each site as having different populations.

### 2.2 DNA extraction

DNA extraction and purification from gills (RRBS, WGS) and muscle or fins (WGS) was performed using the DNeasy Blood and Tissue Kit (QIAGEN) and the NucleoSpin gDNA Clean‐up (Macherey‐Nagel). Individual sex was determined using sex‐specific polymorphism in the isocitrate dehydrogenase gene through PCR (Heckwolf et al., 2020).

### 2.3 Whole genome sequencing: Library preparation and sequence processing

The library preparation for whole genome sequencing (WGS) using 150 bp paired‐end sequencing, as well as the sequence quality assessment, data filtering (trimming, removing duplicates), and mapping against the reference genome (Broad/gasAcu1) were all executed in accordance with the protocols outlined in the study conducted by Heckwolf et al. (2020). Here, we analyzed copy number variation in all 96 individuals from six populations, in comparison to the study by Heckwolf et al. (2020) that analyzed single nucleotide polymorphisms from three populations (KIE, NYN, and SYL). After data filtering, we had an average genome‐wide read depth of 13.84× (SD = 2.02).

### 2.4 Copy number variation analysis

Copy number variations (CNVs) were identified among the samples using a combination of variant callers that detect CNVs using read depth (CNVnator) (Abyzov et al., 2011), paired reads (Breakdancer) (Chen et al., 2009), split reads (pindel) (Ye et al., 2009), and both paired reads and split reads (delly and duppy) (Rausch et al., 2012). These methods have been previously used for detecting CNVs in sticklebacks with high validation success (Chain et al., 2014). CNVnator was used with 500 bp windows, and the rest of the tools were run with default settings. CNV calls from Breakdancer were then filtered based on a maximum coverage of 50x per sample and a minimum of 4 read pairs supporting each call. CNV calls from delly and duppy were filtered for mapping quality above 20 and a minimum of 2 supporting reads, but a maximum of 500 kb in length (the largest CNV from CNVnator was inferred as 480 kb). Bedtools v2.30.0 (Quinlan & Hall, 2010) was used to evaluate the overlap of variant calls among all programs, and only those CNVs calls from CNVnator that had overlaps of at least 50% of their length with calls from another program were kept for further analysis. This resulted in a total of 3102 deletions and 891 duplications across the 96 samples.

CNV regions were further filtered to remove potential false positives by aggregating normalized read depth signals across individuals (Chain et al., 2014). The normalized read depth for each sample was extracted using CNVnator and centered to 2 such that most CNVs have a median of 2 to represent diploid loci. CNV regions passed filtering when they met the following conditions: (1) at least one individual had an average normalized depth of coverage above 1.5× and another below 2.5× (to remove low‐coverage regions and high‐coverage regions), (2) the individual with the highest normalized depth of coverage was at least 1× higher than at least one other individual, and (3) two individuals had a difference in normalized coverage greater than 0.45 (to remove regions without distinct differences among individuals). While the initial CNV cutoff was set to 1.5× to call deletions and 2.5× to call duplications, in 30% of cases, these cutoffs were adjusted manually based on read depth variance across samples and genomic regions, guided by the k‐means clustering method kmeansruns with a krange of 2:10 in the R package fpc v.2.2‐9 (Hennig, 2020). When clusters did not match genotype expectations, the CNVs were excluded.

We detected a total of 547 deletions and 604 duplications that are polymorphic (i.e., CNVs) among the 96 stickleback individuals (Table S1). This included 33 CNVs on the sex chromosome (chrXIX: 19 deletions, 9 duplications, and 5 CNVs with both deletions and duplications) and 38 CNVs on the unassembled chromosome (chrUn: 13 deletions, 23 duplications, and 2 CNVs with both deletions and duplications). There were 27 CNVs that were sex‐specific, including 18 male‐specific “heterozygous deletions” on the sex chromosome that follows hemizygous expectations, and 9 male‐specific duplications that correspond to putative duplications that occur on the Y‐chromosome (Table S2) as previously reported in sticklebacks (Peichel et al., 2020). These sex‐specific CNVs were shared across all six populations, and were excluded from all subsequent analyses. A principal component analysis (PCA) was performed each for deletions and duplications using the prcomp function in R version 3.4.1 (R Core Team, 2020). The Mann–Whitney *U* test was used to measure statistical differences between the lengths of CNV deletions and duplications, and the frequency of CNVs between populations.

Each sample was assigned a copy number and genotype based on deviations from the expected diploid read depth of 2. Genes overlapping CNVs were determined using Bedtools and were classified as full gene CNVs if the overlap was at least 90% of the gene length (to account for inaccurate breakpoints from CNVnator), and partial gene CNVs for less than 90% overlap. Gene read depth for each sample was estimated using CNVnator to exclude genes that did not meet the read depth criteria for CNV regions as described above. For each CNV and gene CNV, *V*_st_ analyses were performed between pairwise populations to identify differentiation in copy numbers that could be indicative of local adaptation (Chain et al., 2014). *V*_st_ was calculated as (*V*_tot_ – *V*_pop_)/*V*_tot_, where *V*_tot_ is the total variance in copy number across all individuals and *V*_pop_ is the average variance within populations. Bi‐allelic CNVs were used to calculate population frequencies of deletions and duplications. The intersection of (shared) CNVs was visualized using UpSetR (Gehlenborg, 2019).

### 2.5 Reduced representation bisulfite sequencing: Library preparation and sequence processing

To explore cytosine methylation among the 96 individuals, we conducted Reduced Representation Bisulfite Sequencing (RRBS) of 100 bp single‐end reads using the MspI restriction enzyme in accordance with the procedures detailed in Heckwolf et al. (2020); in that study, Heckwolf et al. (2020) analyzed a subset of this RRBS dataset (three out of six populations: KIE, NYN, and SYL) with a specific focus on intergenerational stability versus inducibility. Consequently, the same quality assessment, data filtering, mapping, and methylation analysis methods were used, albeit with twice the number of individuals and populations used in the present study.

We sequenced a total of 11.2 million (SD = 2.5 million) raw reads per individual. Mapping was conducted with Bismark v0.17.0 (Krueger & Andrews, 2011), which resulted in an average of 5.6 million (SD = 1.6 million) mapped reads per individual and 58.8% (SD = 4.2%) mapping efficiency. Nine individuals were excluded from further analysis due to an insufficient number of reads (<5 million reads), low mapping efficiency (<52%), and deviations from the expected proportion of bases per position (referred to as “per base sequence content”) for RRBS libraries. The methylation calls of the remaining 87 individuals were subsequently processed using the R‐Bioconductor package “methylKit” version 1.7.4 (Akalin et al., 2012). On average, we sequenced 3,469,538 (SD = 253,317) distinct CpG sites per individual. Next, we performed data cleaning to mitigate potential artifacts: we excluded CpG sites exhibiting extremely high coverage likely stemming from a PCR bias (99.9th percentile) and those with low coverage (<10 reads per CpG site), resulting in 1,266,006 (SD = 276,576) CpG sites per individual, and we implemented read coverage normalization to reduce the risk of systematic oversampling of reads in specific samples. Following these filtering steps, we merged the 87 samples using the “unite” function implemented in methylkit with the “min.per.group” parameter, allowing ≤30% missing data while requiring CpGs to be covered by ≥11 samples per population.

After running these steps from the methylKit package, we applied a correction method to account for potential errors associated with single nucleotide polymorphisms (SNPs). We excluded 18,397 positions affected by C‐to‐T and G‐to‐A SNPs as identified from the 96 genomes using custom‐written scripts (and considering those with a genotype quality of 20 and a minimum allele frequency of 0.005) along with packages from methylKit (Akalin et al., 2012) and GenomicRanges (Lawrence et al., 2013), resulting in 507,267 sites. Lastly, we excluded CpG sites from the sex chromosomes (chromosome 19), resulting in a dataset with 495,635 CpG sites.

### 2.6 DNA methylation analysis and identification of differentially methylated sites

To compare DNA methylation in gene CNVs, specifically among individuals that have duplications versus those that do not, we estimated the percentage of cytosine methylation at each CpG site. Methylation levels were assigned to a gene by taking the mean percent methylation of CpG sites in the promoter region or the gene body (regions defined below). For any CNV duplication that exists as a single locus in the reference genome, DNA methylation levels represent an average across the copies. This is because we are unable to differentiate between each copy when reads map to the same genomic location. Due to this, if DNA methylation levels increase following duplication in at least one copy, we would expect a greater proportion of reads to be methylated although the levels of methylation are averaged across copies; in a scenario where one copy is completely methylated (or unmethylated) while the other is not, the methylation level will not reach 100% (or 0%).

Using the readTranscriptFeatures function and UCSC genePred files, we annotated CpGs sites based on their location relative to genes, including gene bodies (exons and introns excluding promoters), intergenic regions, and promoter sites defined by positions within 1500 bp upstream and 500 bp downstream of transcription start sites (Heckwolf et al., 2020) – resulting in 103,451 promoter CpGs. Pearson coefficients were used to measure the correlation of DNA methylation level among samples and to measure the correlation between the promoter methylation level of each gene (mean of all CpGs of one promoter) and its copy number (i.e., the same gene in different individuals for which some have duplication and some do not), with significance determined after applying the Benjamini–Hochberg FDR correction. Promoter methylation‐level differences were compared between genes with CNVs (gene CNVs) and without CNVs (non‐CNVs), and between CNV deletions and CNV duplications using Mann–Whitney *U* tests. These analyses were repeated to include gene body DNA methylation levels in addition to promoter regions. A chi‐square test was also used to compare the distribution of low‐frequency CNVs (<0.05% among all individuals) in genes with versus without promoter methylation data. While we summarized DNA methylation values into biologically relevant regions (promoters and gene bodies) for a one‐to‐one value comparison with gene CNVs, the population‐wide variability in DNA methylation is presented at the highest resolution for RRBS: base‐pair resolution. We used the “calculateDiffMeth” and “getMethylDiff” functions within methylKit to identify differentially methylated sites (DMS) between populations in a pairwise fashion. Our criteria included a minimum methylation difference of 15% and a *q*‐value of 0.0125, the same thresholds that were used in Heckwolf et al. (2020). Similar to CNVs, a PCA was performed on the methylation levels at DMS after excluding sites without methylation information from all included individuals, leaving us with 2085 sites. Permutation tests of 1000 randomly selected regions equal to the lengths of CNVs were used to evaluate the probability of overlapping with DMS.

### 2.7 Gene annotations and gene ontology enrichment analysis

Using the Ensembl comparative genomics framework and gene trees within the Ensembl database (Herrero et al., 2016), genes were categorized into three different gene types based on whether they had orthologs but no paralogs (singletons; *n* = 4134), paralogs that emerged before the diversification of sticklebacks (paralogs; *n* = 14,039), or young “lineage‐specific genes” (LSGs; *n* = 4283) that included genes with recently duplicated genes within the stickleback lineage and/or genes with no detectable orthologs. The average promoter methylation level of LSGs was compared with non‐LSGs using a Mann–Whitney *U* test with the expectation that younger genes are more methylated. Gene ontology terms were also acquired via Ensembl and used for gene ontology enrichment in topGO version 2.50.0 (Alexa et al., 2006). Functions were determined to be enriched among sets of genes based on FDR‐corrected *p*‐values <0.05 from the weight01 algorithm. R scripts for the analysis and visualization of DNA methylation and CNV data are available on github.com/BEEGlab/CNV‐methylation‐with‐RRBS.

## 3 RESULTS

### 3.1 CNVs across a salinity gradient in Baltic stickleback populations

**FIGURE 2:**
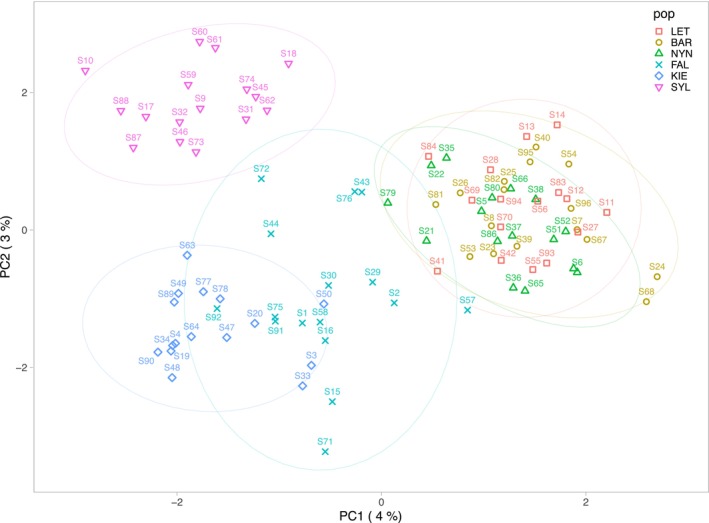
Principal component analysis (PCA) of all CNVs (duplications and deletions) based on presence/absence across 96 individuals. Ellipses represent a 95% confidence level around the samples from each population.

In total, 521 deletion CNVs and 594 duplication CNVs were identified among 96 stickleback individuals after excluding sex‐specific CNVs. The proportion of CNV singletons (CNVs in a single individual) for deletions (52%) was lower than for duplications (66%), resulting in deletions being shared among individuals more often than duplications (Figure 1). There were more deletions shared among all populations (151) and five out of 6 populations (30) compared to duplications (27 and 17, respectively; Figure S2). Based on CNV abundance and distribution, we found that populations at low salinity levels in the northern range of the Baltic (LET, BAR, NYN) clustered together while the populations at higher salinity levels in the southern range largely separated the Baltic (FAL, KIE) from the North Sea (SYL) populations (Figure 2; Figure S3).

Despite comparable numbers of deletions and duplications, deletions were on average smaller than duplications (mean deletion length: 7102 bp; mean duplication length: 11,930 bp; Mann–Whitney *U* Test *p* < 0.001). This resulted in many more genes overlapping with duplications; there were 436 CNV duplications with gene overlaps compared to 190 CNV deletions. We found 963 genes overlapping CNVs either fully (92 in deletions, 353 in duplications) or partially (161 in deletions, 465 in duplications). After filtering for read depth within each gene coordinate to reduce false positives (see methods), we retained 454 CNVs overlapping 442 genes (Table S3). Each fish has on average 89 deletions and 23 duplications, involving on average 10 gene deletions and 15 gene duplications (Table S4). Individuals from higher salinities in the southern range of the Baltic and North Sea had more CNVs (Mann–Whitney *U* test *p* < 0.001) and gene CNVs (Mann–Whitney *U* test *p* < 0.001; Table 1). There was relatively little overlap with gene CNVs found in other natural populations of stickleback from different environments; 10 overlaps out of 211 (5%) from Lowe et al. (2018), 62 out of 1865 (3%) from Chain et al. (2014), and 0 overlaps out of 24 from Hirase et al. (2014). Gene CNVs found only in our study populations were mostly singletons (79%) but did include 8 gene duplications at >10% frequency (Table S5), potentially representing young duplications that have rapidly spread through the Baltic. CNV frequency distributions were similar across populations, but whereas CNV deletions overlapping genes were found at proportionally lower frequencies than deletion CNVs overall in each population, CNV duplications overlapping genes were found at proportionally higher frequencies than duplication CNVs overall (FDR‐corrected Mann–Whitney *U* test *q* = 0.026 (LET), 0.004 (BAR), 0.004 (NYN), 0.014 (FAL), 0.005 (KIE), 0.026 (SYL); Figure S4).

**TABLE 1:** Number of CNVs per population, with nonsingleton private CNVs in between parentheses.

Population	PSU (practical salinity units)	Deletions	Duplications	Gene deletions	Gene duplications
LET	4.3	250 (1)	135 (2)	18	77
BAR	4.6	260 (2)	143 (3)	23	93
NYN	6.0	267 (2)	148 (2)	24	90
FAL	9.8	290 (2)	177 (9)	26	118
KIE	18.0	277 (8)	192 (13)	24	149
SYL	28.9	252 (4)	169 (7)	24	115

### 3.2 Pairwise gene copy number differences among populations

**FIGURE 3:**
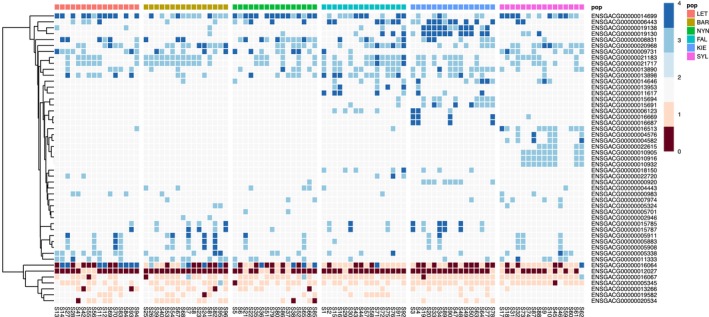
Heatmap of gene copy number within the top 2% highest *V*_st_ values. *V*_st_ is analogous to *F*_st_, measuring the genetic differentiation in CNVs between populations. Heatmap colors display the estimated copy number per individual (column) per gene (row), where 0 is a homozygous deletion, 1 is a heterozygous deletion, 2 is no CNV, and >2 represents duplications. Individuals are separated by population of origin, from lowest PSU (left) to highest (right).

**FIGURE 4:**
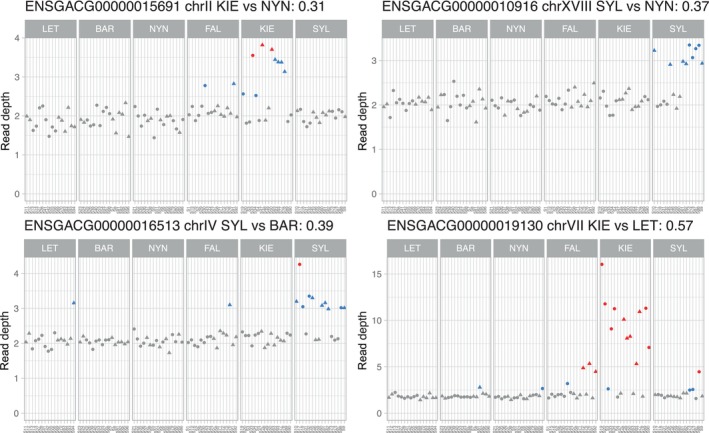
Read depth across individuals for select complete gene duplications with high *V*_st_. Read depth is normalized to two to represent a diploid copy number. The title for each gene includes the Ensembl ID, the chromosome, the populations between which there is the highest pairwise *V*_st_ value, and the corresponding *V*_st_ estimate. The genes are ENSGACG00000015691: (*adma*) adrenomedullin a, ENSGACG00000010916: (*rps12*) ribosomal protein S12, ENSGACG00000016513: (*tdo2a*) tryptophan 2,3‐dioxygenase a, and ENSGACG00000019130: (*ptgr1*) prostaglandin reductase 1. Circles in the plot represent females, triangles males, and the colors represent differences in the estimated copy number.

To determine population differentiation at CNVs that could reflect adaptive divergence in copy numbers along the genome, the *V*_st_ metric was used. While there were no fixed differences in CNVs among populations, genome‐wide *V*_st_ scans of CNVs and gene CNVs displayed several private CNVs at intermediate frequencies between populations (Figure S5). Of the 49 gene CNVs with the highest population differentiation as measured by *V*_st_ (top 2% of all pairwise *V*_st_ values; *V*_st_ > 0.1), most (94%) were duplications (Figure 3) and were found at intermediate to higher frequencies in southern populations (Table S6). The genes with the highest CNV differentiation included genes duplicated primarily in individuals from KIE or SYL (Figure 4) such as adrenomedullin a (*adma*; ENSGACG00000015691), adenosine monophosphate deaminase 3a (*ampd3a*; ENSGACG00000015694; partial duplication), RAD18 E3 ubiquitin protein ligase (*rad18*; ENSGACG00000000920; partial duplication), peptidylprolyl isomerase H (*ppih*; ENSGACG00000006443; partial duplication), two within‐species paralogs of prostaglandin reductase 1 (*ptgr1*; ENSGACG00000019130 and ENSGACG00000019138; the latter is a partial duplication), tryptophan 2,3‐dioxygenase a (*tdo2a*; ENSGACG00000016513), epoxide hydrolase 5 (*ephx5*; ENSGACG00000004582), and linked genes on chrXVIII that include EYA transcriptional coactivator and phosphatase 4 (*eya4*; ENSGACG00000010932; partial duplication), ribosomal protein S12 (*rps12*; ENSGACG00000010916), and an uncharacterized gene (ENSGACG00000010905) similar to *CD5L* and *SSC5D* (scavenger receptor cysteine rich family member with 5 domains). Gene enrichment analysis detected two enriched gene ontology functions (FDR < 0.05) among the CNVs with the highest *V*_st_ (2‐alkenal reductase [NAD(P)+] activity and 15‐oxoprostaglandin 13‐oxidase activity) but with only two annotated genes each. Similarly, gene CNVs overall had three enriched terms: tryptophan catabolic process (4 annotated genes), “de novo” NAD biosynthetic process from tryptophan (3 annotated genes), and anthranilate metabolic process (3 annotated genes). There was no functional enrichment among CNVs that were exclusive to, or shared among, only high‐ or low‐salinity populations. Visualization of read depth across samples for each gene CNV can be found in File S1. The abundance of gene CNVs among these stickleback genomes allowed us to investigate the potential role of epigenetic silencing in regulating extra gene copies using DNA methylation data from the same individuals.

### 3.3 Methylation levels and differentially methylated sites do not associate with CNVs

**FIGURE 5:**
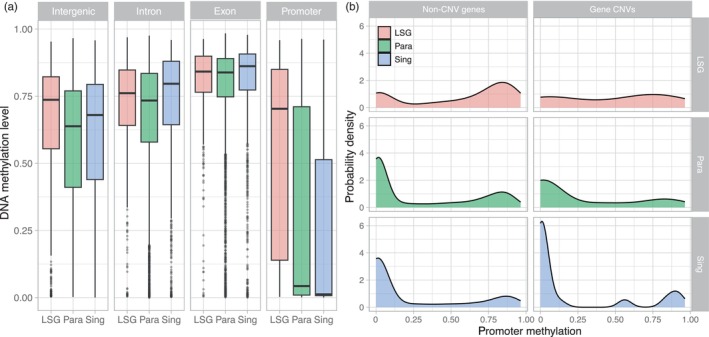
DNA methylation‐level distributions by gene type and location. (a) Boxplots of lineage‐specific genes and duplications (LSG), older paralogs (Para), and singleton genes (Sing) by genomic region. (b) Promoter methylation density distributions for non‐CNV genes and gene CNVs, separated by LSG, Para, and Sing.

We measured DNA methylation levels of genes as defined by the average percent methylation at CpG sites, with values between 0 (no methylated sites) and 1 (all sites were 100% methylated). Promoter DNA methylation was on average much lower (mean ± SD = 0.31 ± 0.36) than exons (0.78 ± 0.21), introns (0.68 ± 0.22), and intergenic regions (0.58 ± 0.26; all Mann–Whitney *U* test *p* < 0.001). DNA methylation level in promoters was highly correlated among samples regardless of an individual's origin or salinity exposure (all pairwise Pearson correlation values >0.96). To determine if younger genes and CNVs are preferentially methylated as was found in a previous study using different stickleback populations (Huang & Chain, 2021), gene promoter methylation level was stratified by gene type—lineage‐specific genes (LSGs), old paralogs, or singletons (see methods)—and by CNV presence among Baltic populations. We found that lineage‐specific genes displayed higher levels of promoter methylation (0.55 ± 0.35) compared to older genes (0.30 ± 0.36; Mann–Whitney *U* test *p* < 0.001; Figure 5), but that gene CNVs had similar levels of promoter methylation (0.27 ± 0.36) as non‐CNVs (0.32 ± 0.36; Mann–Whitney *U* test *p* = 0.097). While this latter result differs from earlier findings, it is noteworthy that over 30% of the gene CNVs used in the previous study by Huang and Chain (2021) are LSGs, which are highly methylated, compared to 11% of the gene CNVs in our study. Indeed, we found that the genes from Huang and Chain (2021) that are CNVs in non‐Baltic populations also have significantly higher DNA methylation levels in our study compared to non‐CNVs (Mann–Whitney *U* test *p* < 0.001).

With RRBS data, less than half (8143) of all protein‐coding genes (including 140 out of 442 gene CNVs) had sufficient coverage of CpG sites in their promoters, with on average 12.5 sites per promoter compared to the 77 average CpGs in promoter's genome‐wide based on the reference genome. This list of genes retrieved excluded some gene CNVs with the highest *V*_st_ (population frequency differences) values (including all genes from Figure 4) and was overrepresented with CNVs segregating at low population frequencies (<0.05%; chi‐squared *p* = 0.001). For each remaining gene CNV, we calculated the correlation between promoter DNA methylation level and copy number across samples. This analysis was used to determine, per gene, whether an increase in copy number is associated with more or less DNA methylation. We found little to no significant association overall; the mean correlation of all genes was zero (there were relatively equal numbers of genes with positive and negative correlations), with four genes having an absolute Pearson correlation of 0.3 or greater, two of which were statistically significant after FDR correction (*nsmfb* and *SLC16A2*; Figure S6). When we consider environmental differences among populations by measuring the correlations between promoter methylation and CNVs within each population separately, we find an additional significant correlation with the *setd7* gene (SET domain‐containing 7 histone lysine methyltransferase). Expanding the analysis to include all CpG sites in gene bodies (exons and introns) increased the range of sites and genes analyzed (272 gene CNVs) but did not change the overall patterns.

**FIGURE 6:**
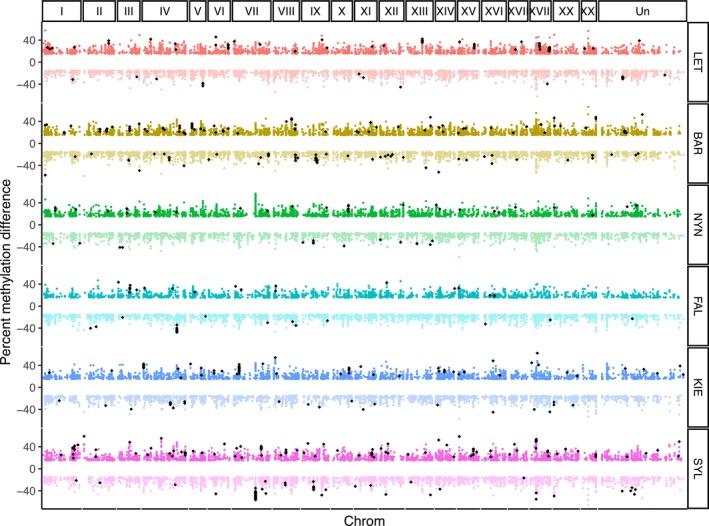
DMS scans across the genome are separated by chromosome. Each circular point represents a CpG site that is differentiated between two populations; for each focal population (labels on the right), 5 pairwise comparisons were carried out. DMS is colored by focal population, with darker shadings indicating higher methylation in the focal population. Black diamonds represent population‐specific DMS (found in all 5 pairwise comparisons).

**FIGURE 7:**
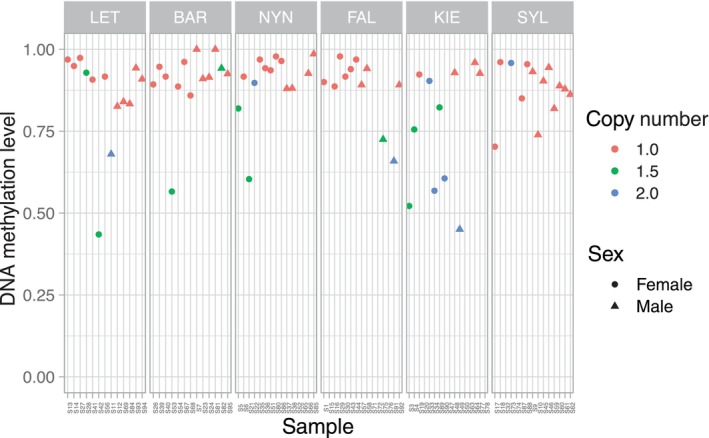
The gene peptidylprolyl isomerase H (*ppih*) shows differentiation in copy number and variation in gene methylation levels among populations. Individuals with higher copy numbers tend to have lower levels of DNA methylation.

Finally, we identified differentially methylated CpG sites (DMS) across the genome between pairwise populations to determine whether there is an association between methylation divergence and CNV divergence. In total we identified 13,691 unique DMS genome‐wide between at least two populations (Figure 6), including 544 population‐specific DMS (Table S7). Similar to CNVs, a PCA of DMS partly separates low‐salinity populations in the northern range versus high‐salinity populations in the southern range along the first principal component (Figure S7). While there was some overlap among DMS and CNVs, there was no enrichment of DMS among CNVs (259 overlaps in 114 CNVs, *p* = 0.11, permutation test) nor gene CNVs (101 overlaps in 45 genes, *p* = 0.42, permutation test). We found no gene ontology enrichment among these gene CNVs with DMS. There were 35 DMS occurring within promoters of 12 gene CNVs, and 66 DMS in gene bodies of 33 gene CNVs. The overlapping CNVs are at low population frequencies, deleted or duplicated in one or two individuals with the exception of four genes: an uncharacterized gene (ENSGACG00000018948) duplicated in three individuals, *il‐15ra* (ENSGACG00000019173) duplicated in each population in a total of 15 individuals (Table S3), and the genes *ptgr1* and *ppih* that both have a high frequency of partial gene duplication in the KIE population (Table S8). The gene *ptgr1* (prostaglandin reductase 1), duplicated in 12 out of 16 KIE individuals and with an average *V*_st_ of 0.48, contains one DMS in the gene body with a higher level of methylation in KIE versus NYN but not between other populations. This gene is involved in arachidonic acid metabolism and is also found in high copy numbers in freshwater fish species (Ishikawa et al., 2022). The gene *ppih* (peptidylproyly isomerase H) also has the highest duplication frequency in KIE (11 out of 16 individuals) and an average *V*_st_ of 0.19, and one DMS in the gene body with higher methylation level in KIE compared to BAR, FAL, and SYL. With *ppih*, the individuals with higher copy numbers have significantly lower levels of gene body DNA methylation (Pearson correlation of −0.62, *q* < 0.001; Figure 7), but promoter region CpGs of this gene were not captured by our RRBS approach.

## 4 DISCUSSION

### 4.1 Little evidence of DNA methylation regulation of CNVs

Our analyses of DNA methylation among CNVs did not detect an association between promoter methylation levels and gene copy number. While an increase in gene activity via gene duplication can be adaptive, as described for expansions of amylase genes across populations and species (Pajic et al., 2019; Perry et al., 2007), we expect that in most cases extra transcriptional activity is detrimental to the fitness of individuals. Repressing expression could therefore protect CNVs from purifying selection and allow otherwise harmful duplications and deletions to spread through a population (Rodin & Riggs, 2003). Epigenetic regulation, for example via DNA methylation of gene promoters, could provide a rapid regulatory response to such gene copy number changes. We therefore would predict that most duplicate genes lacking repression would soon be removed or remain at low population frequency because of detrimental overexpression, while repressed genes would have the opportunity to rise in population frequency due to being shielded from purifying selection against misregulation.

It was previously shown in sticklebacks that gene CNVs had on average higher promoter methylation levels and lower expression when compared with genes that are not CNVs, raising the possibility that DNA methylation modulates the expression of a large proportion of recently duplicated and deleted genes, or that genes with elevated methylation levels might be more permissive to subsequent copy number variation (Huang & Chain, 2021). In contrast to this, while we show that natural populations of stickleback in the Baltic and North Sea possess hundreds of copy number variable genes, the gene CNVs that we investigated had similar promoter methylation levels as non‐CNVs. It is possible that part of this difference can be attributed to the large proportion of CNVs at low frequency among the genes included in our promoter methylation analysis (e.g., only 14 genes are found in more than two individuals). It was previously observed that CNVs segregating at higher frequencies have higher levels of DNA methylation (Huang & Chain, 2021), possibly indicative of methylation accumulation over time following gene duplication. Another associated explanation stems from the types of genes detected as CNVs between studies; there were proportionally three times fewer CNVs of lineage‐specific genes in our study, which are genes that have high levels of promoter methylation regardless of CNV status as found here and previously (Huang & Chain, 2021). This suggests that there is a subset of evolutionarily young genes that are preferentially highly methylated in sticklebacks, but these are not necessarily segregating (or captured) as CNVs in all populations. In other words, it is possible that non‐Baltic CNVs have higher promoter methylation levels on average as a consequence of being lineage‐specific genes, rather than because they are CNVs. This aligns with the idea that DNA methylation may in some cases enable duplication persistence as CNVs, but that this process might occur over longer time periods and not necessarily change immediately post‐duplication.

In addition to comparisons made among genes, here our data combined methylation and genomic copy number data from the same individuals. This allowed us to directly measure DNA methylation levels in individuals with one versus multiple copies of a gene to test whether the presence of duplications correlates with changes in promoter DNA methylation. However, we found no consistent differences in DNA methylation levels between individuals with different gene copy numbers. That we did not detect a correlation between the number of copies of a gene and promoter methylation could suggest no relationship between gene copy number and hyper‐ or hypo‐methylation, at least for the part of the genome we investigated. This is unexpected if DNA methylation plays a role in the early repression of polymorphic gene duplications. As this study and others have found that young duplicate genes are highly methylated compared to older genes (Huang & Chain, 2021; Keller & Yi, 2014; Zhong et al., 2016), we propose that hypermethylation does not occur immediately after duplication, at least for the CNVs in the populations surveyed. Instead, this hypermethylation process may occur over longer timescales, while any early repression may be the result of other heritable transcriptional and post‐transcriptional repressors such as histone modifications or small RNAs. Over time, histone modifications can recruit DNA methylation and interact with DNA methyltransferases to facilitate stable gene repression (Cedar & Bergman, 2009; Rose & Klose, 2014). Research in different stickleback populations revealed that 39% of gene CNVs have a significant positive correlation between copy number and gene expression (Huang et al., 2019), demonstrating that there is ample expression regulation on the rest of the CNVs that prevent dosage effects. While this suggests that there could be >50% of CNVs that are regulated for dosage, we find only two gene CNVs (1.5%) with a relationship between copy number and promoter methylation levels. Our findings in this study suggest that most of the 140 polymorphic duplicate genes that were captured by our RRBS approach may not be regulated soon after duplication by promoter DNA methylation but could be regulated via some alternative (epigenetic) mechanisms like histone modifications or noncoding RNAs, or simply not downregulated at all.

### 4.2 Genetic copy number and DNA methylation variation are most prominent in high salinity

The divergence in expression of duplicated genes has been linked to salinity adaptations in various organisms such as protists, plants, and fish including stickleback (Dalziel et al., 2014; Harding et al., 2017; Tian et al., 2022; Wang et al., 2022; Xu et al., 2023). In addition, it is plausible that duplicating salinity‐relevant genes enables toggling their expression levels, which could impart habitat‐specific advantages for a euryhaline species such as the three‐spined stickleback. While there were gene CNVs that overlapped with CNVs found in other marine and freshwater stickleback populations worldwide, most gene CNVs we found were specific to Baltic and North Sea populations in this study. These included CNVs at high frequencies and with signals of population differentiation that could be the result of local adaptation to salinity levels or other environmental factors specific to ecogeographical regions in the Baltic Sea. For example, adrenomedullin a, which was found duplicated in most individuals from KIE and two from FAL, is suggested to be involved in osmoregulation activity and potentially plays an important role in adaptation to high salinity levels in other euryhaline fishes (Ogoshi et al., 2008, 2015). That we do not find DNA methylation levels to increase with CNVs suggests that there could be a pervasive dosage response to gene duplication and deletion, whereby individuals with higher copy numbers have higher gene expression (Huang et al., 2019). This would be expected for a subset of genes with adaptive increases in expression following gene duplication. We report at least one case of population differentiation of a gene CNV (peptidylprolyl isomerase H) that displays nearly half the level of gene body DNA methylation in individuals with the duplication. If an increase in DNA methylation is associated with repression and the observed methylation pattern extends to the promoter region CpGs, which were not captured by our RRBS approach, it could suggest that one of the duplicate copies has lost epigenetic repression and may contribute to adaptive expression gains. Because methylation level is estimated based on reads mapping to the reference genome that has a single gene copy, DNA methylation level is averaged for duplicates mapping to the same locus such that having half the level of DNA methylation could be the result of one of the copies being unmethylated. Without transcriptional information from these same individuals, however, we cannot confirm the increased expression of this gene CNV, but the lack of repression via DNA methylation coupled with the high level of differentiation between populations is consistent with an adaptive duplication. A largely different set of genes than those with copy number changes display differential methylation among our study populations. Whereas it is possible that epigenetic differentiation accompanies or enables environmental adaptation (Vogt, 2023; Weiner & Katz, 2021), we find that such occurrences in these stickleback populations appear largely independent from CNVs for the portion of the genome investigated.

### 4.3 Potential explanations for the lack of association between DNA methylation and CNVs

Epigenetic variance has been argued as potentially preceding genetic diversification in sticklebacks, at least for single nucleotide polymorphisms (Ord et al., 2023). This phenomenon was not observed in our study of structural variation in portions of the stickleback genome that we investigated. There could be a genuine lack of association between promoter methylation level and CNVs, despite previous observations suggesting otherwise using whole genome bisulfite data (Huang & Chain, 2021), which could instead be related to the propensity of CNVs that are lineage‐specific genes or segregating at high population frequencies. The higher levels of DNA methylation in promoters that we detect among lineage‐specific genes compared to older genes are consistent with previous studies that find that DNA methylation levels decrease with gene age (Dyson & Goodisman, 2020; Fang et al., 2018; Huang & Chain, 2021; Keller & Yi, 2014; Wang et al., 2016; Zhong et al., 2016). This could reflect the greater likelihood of duplicate genes surviving over time when they are already highly methylated prior to duplication, rather than hypermethylation of extra copies immediately after duplication (Huang & Chain, 2021). It could also be that DNA methylation is recruited to stabilize expression of gene duplications at later evolutionary stages as genes age, or that other epigenetic mechanisms like histone modifications play a larger role in balancing dosage post‐duplication (Chang & Liao, 2017).

There are, however, technical limitations that may have affected our ability to detect associations, for example, if we missed critical diagnostic CpG sites using RRBS data. Fewer than half of stickleback genes (40%) and one‐third of gene CNVs (32%) had promoter methylation information in our study, potentially affecting the detection of differences among individuals. Expanding our analyses to gene body methylation did not alter the overall patterns found from promoter methylation levels, although methylation levels in promoters are generally distinct from gene bodies in sticklebacks (Huang & Chain, 2021). Furthermore, DNA methylation of genes hundreds of Mb away can have impacts on CNVs (Shi et al., 2020), complicating the evaluation of promoter‐based analyses. But if our results are generally representative of promoter methylation of gene CNVs, it could suggest that DNA methylation plays a minimal role in regulating the earliest evolutionary stages of duplicate genes. To address these outstanding questions, advances in sequencing approaches enable investigating the complementary regulatory role of other epigenetic modifications on CNVs, and long‐read technologies may help distinguish both the transcriptional and epigenetic landscapes of each duplicate copy within the genome. Future studies of gene CNVs with both gene expression and epigenetic data from the same individuals are necessary to tease apart the varying influence that DNA methylation might, or might not, have on regulating the transcription of young and old duplicate genes. These types of investigations that include genomics, transcriptomics, and epigenomics all from the same individuals are currently rare, but essential to advance our understanding of CNV evolution and regulation.

## FUNDING INFORMATION

We express our gratitude for the financial support provided to the BAMBI project (grant agreement number: call 2012‐76) by BONUS; the joint Baltic Sea research program, funded by the European Union; the Federal Ministry of Education and Research in Germany (to C.E. and T.B.H.R., reference number 03F0680A). The research conducted by B.S.M. received support from the Excellence Cluster “The Future Ocean” (EXC 80), and F.J.J.C. was supported by the National Science Foundation (grant number 2144259).

## CONFLICT OF INTEREST STATEMENT

Melanie Heckwolf is an Editorial Board member of Evolutionary Applications and a co‐author of this article. To minimize bias, they were excluded from all editorial decision‐making related to the acceptance of this article for publication.

## ANIMAL WELFARE

All captures were conducted in compliance with legal authorizations granted by the relevant authorities, including The German Ministry of Energy Transition, Agriculture, Environment, Nature, and Digitalization in Schleswig‐Holstein (MELUR: V242‐7224.121‐19), the Estonian Ministry of the Environment (Keskkonnaministeerium ‐ eripüügiluba nr 28/2014), and the Swedish Sea and Water Authority (Havs och Vattenmyndigheten).

## Supporting information


Appendix S1

## Data Availability

The WGS raw reads are accessible from the BioProject PRJNA749309 and the RRBS data in the BioProjects PRJNA591803 and PRJNA743784.
