# Supplementary material for: Epigenetic diversity of genes with copy number variations among natural populations of the three‐spined stickleback
Source: Evol Appl. 2024 Jul 14;17(7):e13753. doi: 10.1111/eva.13753 (PMC11246597; doi:10.1111/eva.13753)
Supplement: Supplementary file 1 — Appendix S1 [file EVA-17-e13753-s001.zip › CNVBaltic_SupplFigures_final.docx]

**Figure S1:** CNV presence/absence frequency distributions separated by deletions (del) and duplications (dup).

**
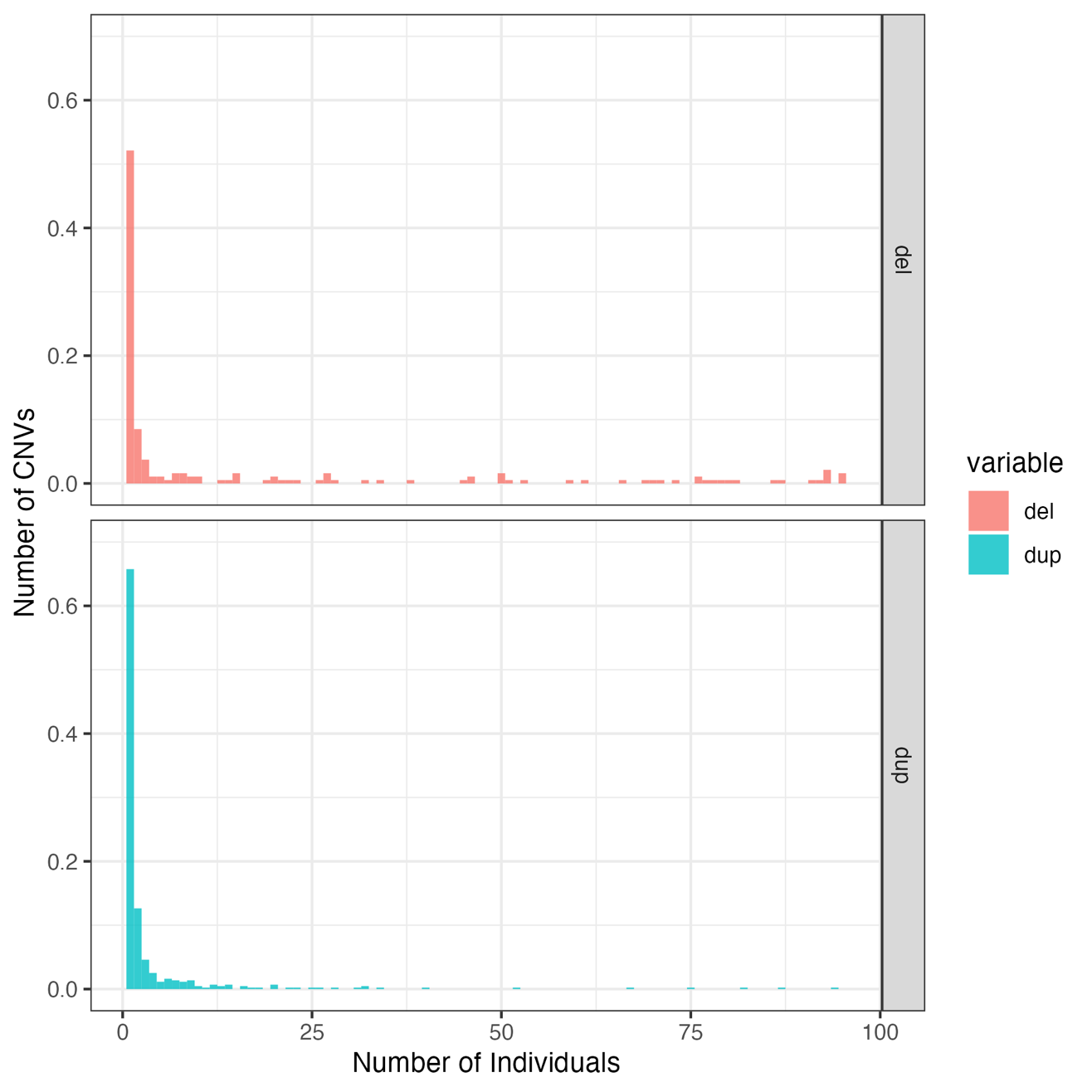
Figure S2:**  Upset plot showing the number of shared CNVs, both (A) deletions and (B) duplications across populations. The plots show the number of CNVs (y-axis) that overlap (are shared) among different populations, with the dots on the x-axis representing the populations in which a CNV is found.


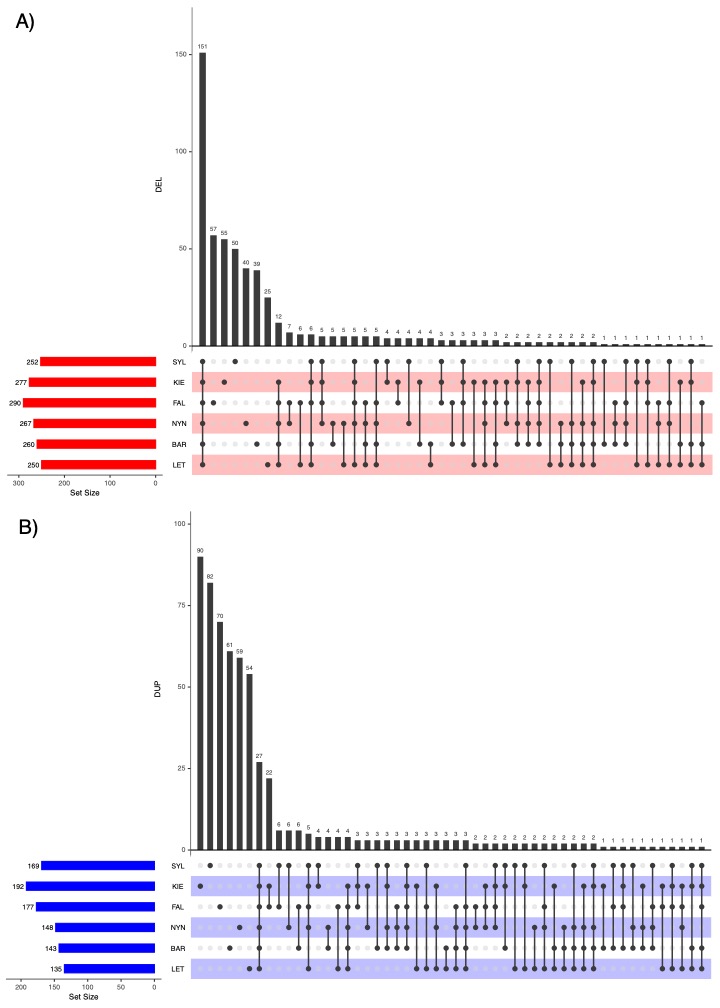
**Figure S3:** PCA (Principal Component Analysis) of all CNVs based on presence/absence across 96 individuals, separated by A) duplications and B) deletions. Ellipses represent a 95% confidence level around the samples from each population.


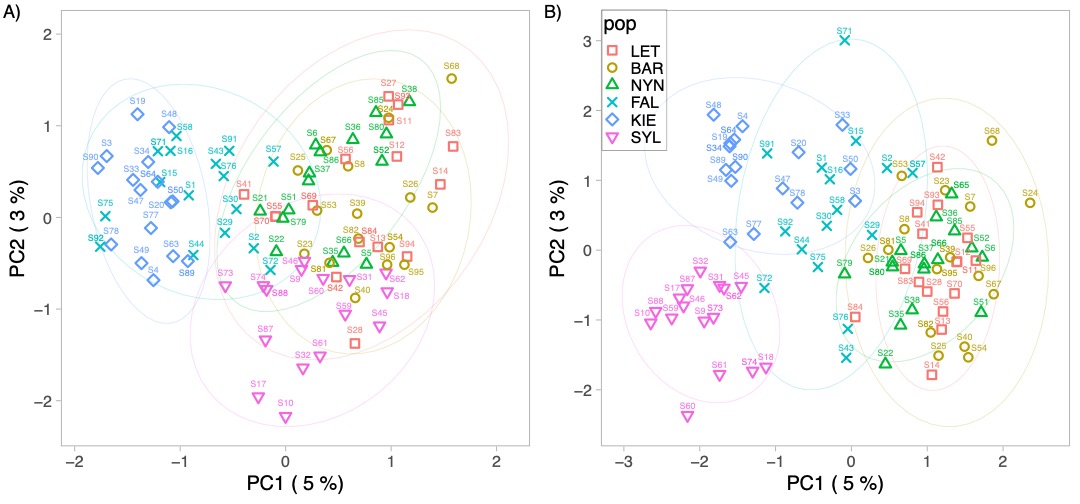
**Figure S4:** CNV frequency distribution of biallelic deletions (del) and duplications (dup) among populations for (A) all CNVs and (B) gene CNVs (CNVs overlapping genes).

**
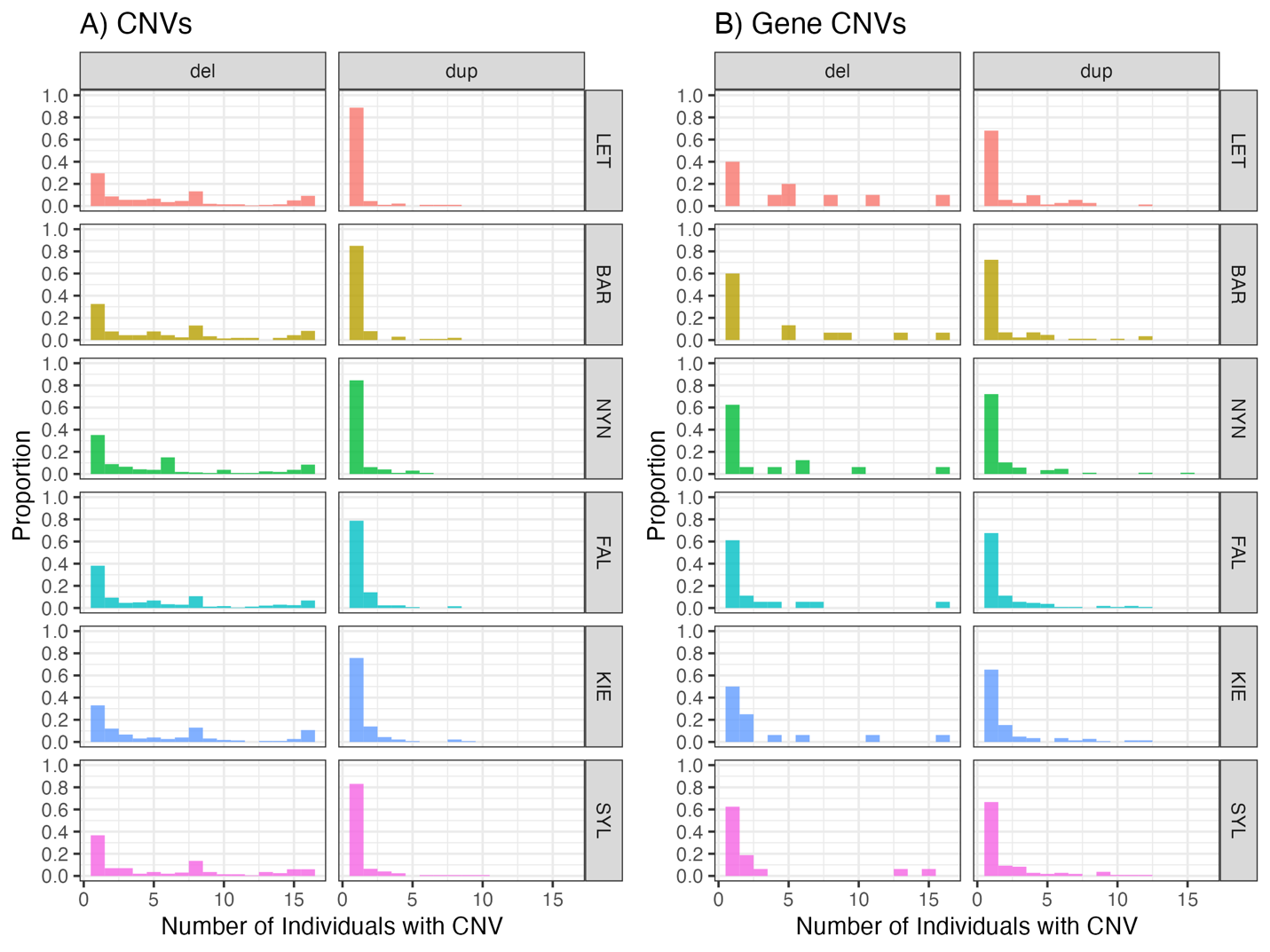
Figure S5**: Vst genome scans across all CNVs (A) pairwise and (B) averaged across all pairwise comparisons, as well as across all gene CNVs (C) pairwise and (D) average.

**Figure S6:** Volcano plots of the Pearson correlation values (cor) between gene copy number and (A) promoter DNA methylation level and (B) gene body + promoter DNA methylation level. After applying FDR correction, only two promoters (*nsmfb*: ENSGACG00000005324 and *SLC16A2*: ENSGACG00000017666) and seven gene bodies have significant associations between copy number and methylation level.

**Figure S7:** PCA (Principal Component Analysis) based on differentially methylated sites (DMS). After removing individuals with low sequencing quality and excluding sites without methylation information from all remaining 87 individuals, we were left with 2,085 sites. **
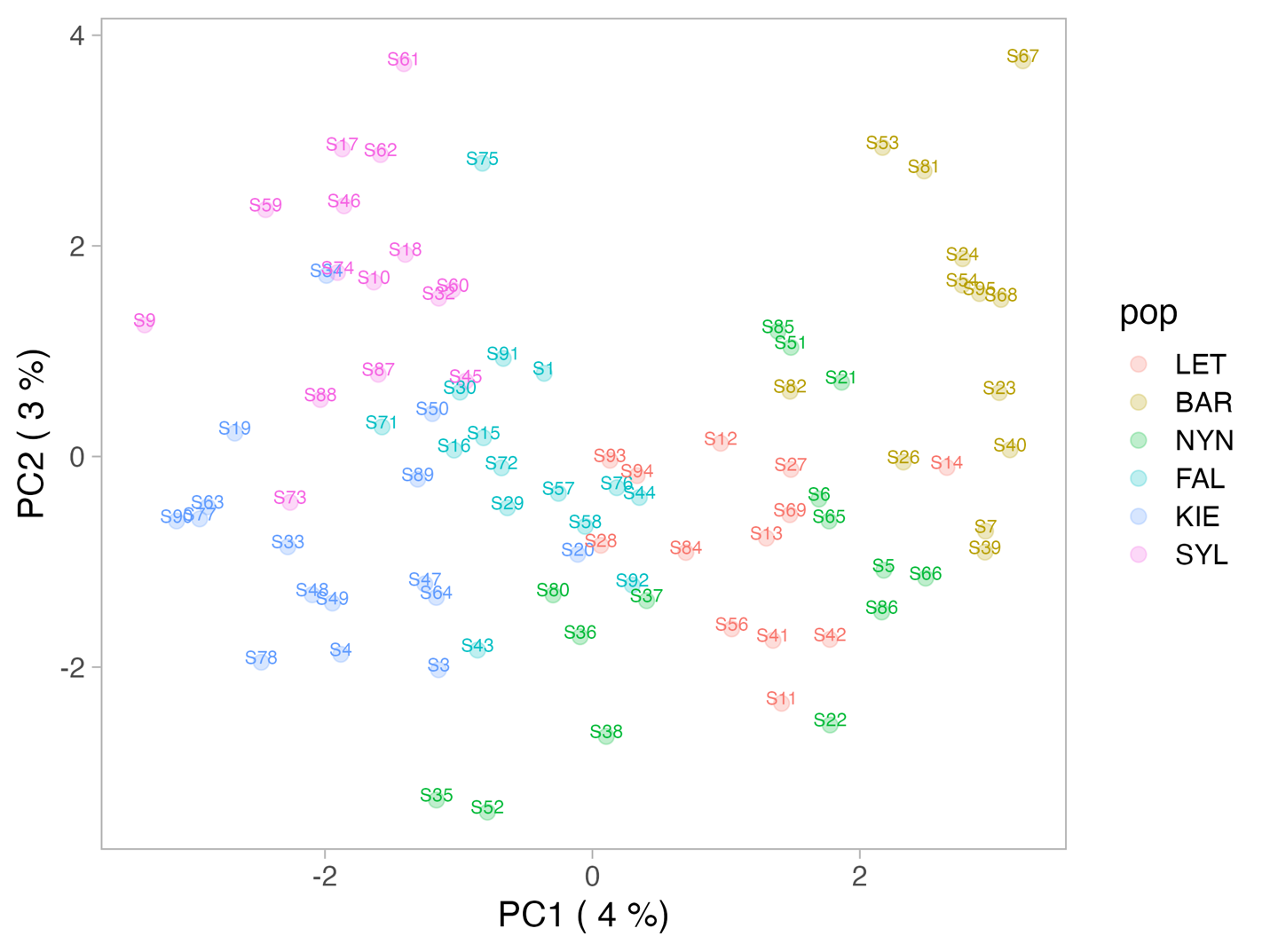
**
