# Supplementary material for: Epigenetic diversity of genes with copy number variations among natural populations of the three‐spined stickleback
Source: Evol Appl. 2024 Jul 14;17(7):e13753. doi: 10.1111/eva.13753 (PMC11246597; doi:10.1111/eva.13753)
Supplement: Supplementary file 1 — Appendix S1 [file SupplFile1.pdf › SupplFile1.pdf]

ENSGACG00000004408 chrI 42005 both

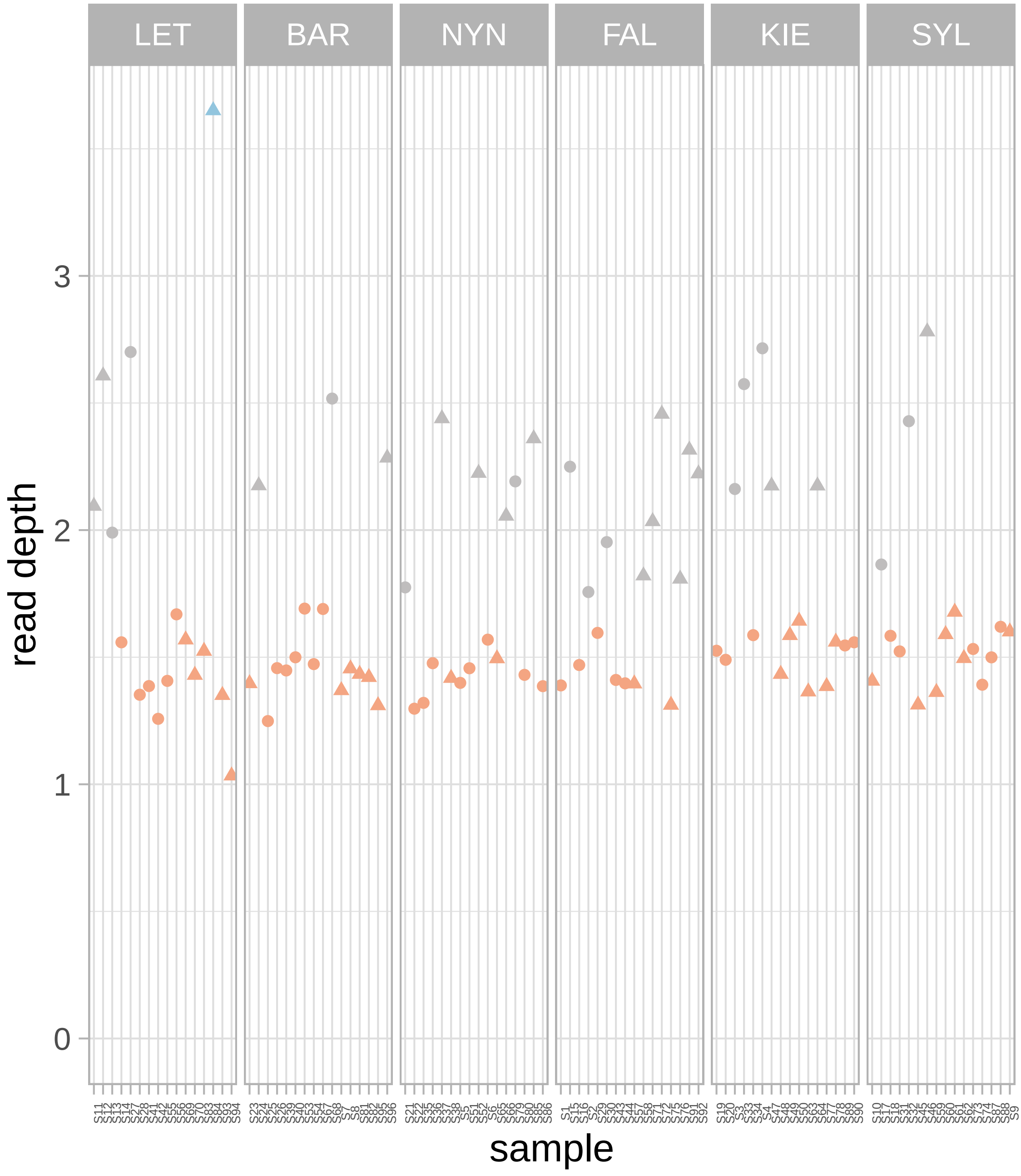

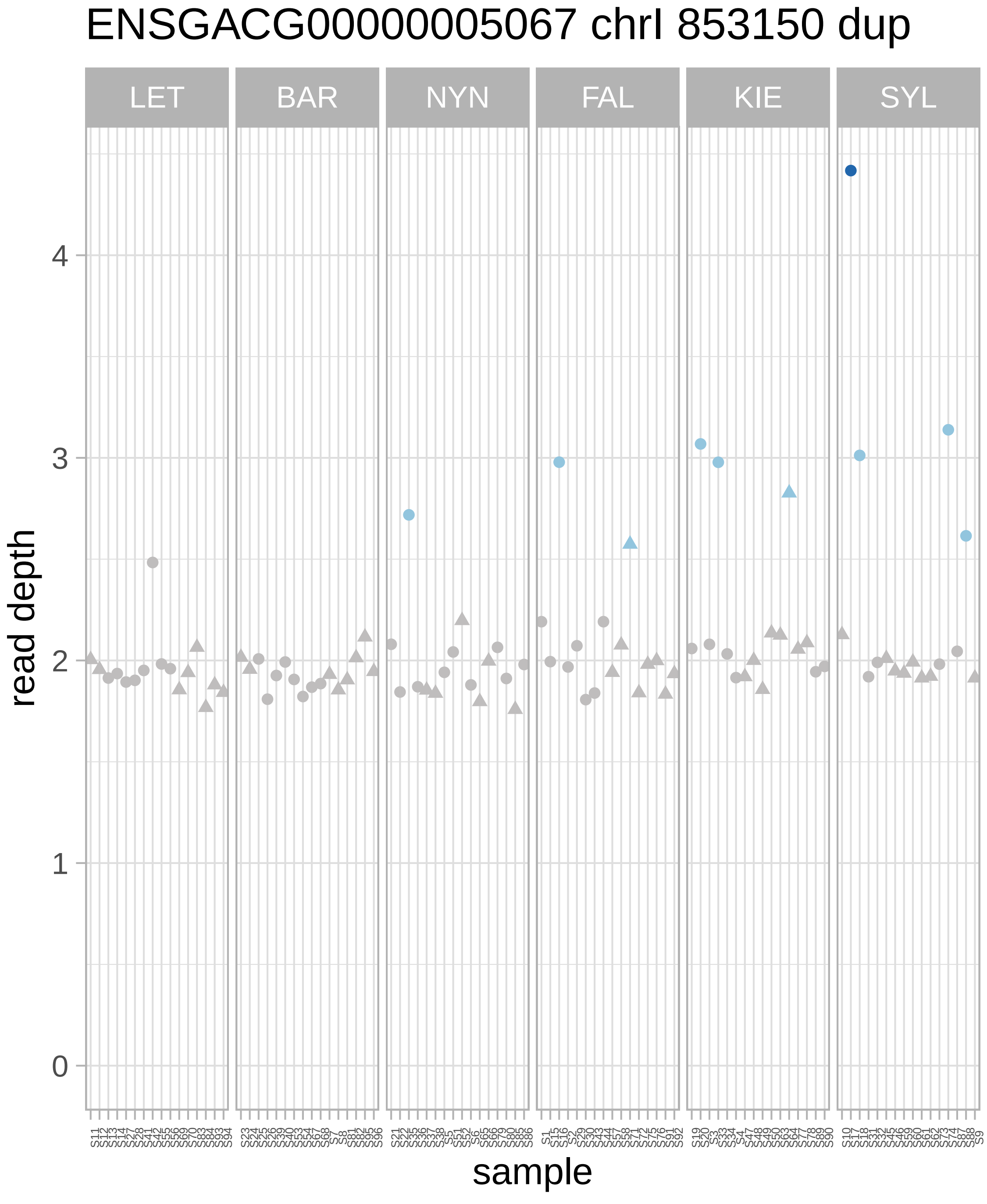
ENSGACG00000005067 chrI 853150 dup
LET
BAR
NYN
FAL
KIE
SYL
read depth
0
1
2
3
4
sample
S11 S12 S13 S14 S27 S28 S41 S42 S55 S56 S69 S70 S83 S84 S93 S94
S23 S24 S25 S26 S39 S40 S53 S54 S67 S68 S7 S8 S81 S82 S95 S96
S21 S22 S35 S36 S37 S38 S5 S51 S52 S6 S65 S66 S79 S80 S85 S86
S1 S15 S16 S2 S29 S30 S43 S44 S57 S58 S71 S72 S75 S76 S91 S92
S19 S20 S3 S33 S34 S4 S47 S48 S49 S50 S63 S64 S77 S78 S89 S90
S10 S17 S18 S31 S32 S45 S46 S59 S60 S61 S62 S73 S74 S87 S88 S9

ENSGACG00000005337 chrI 1234076 dup

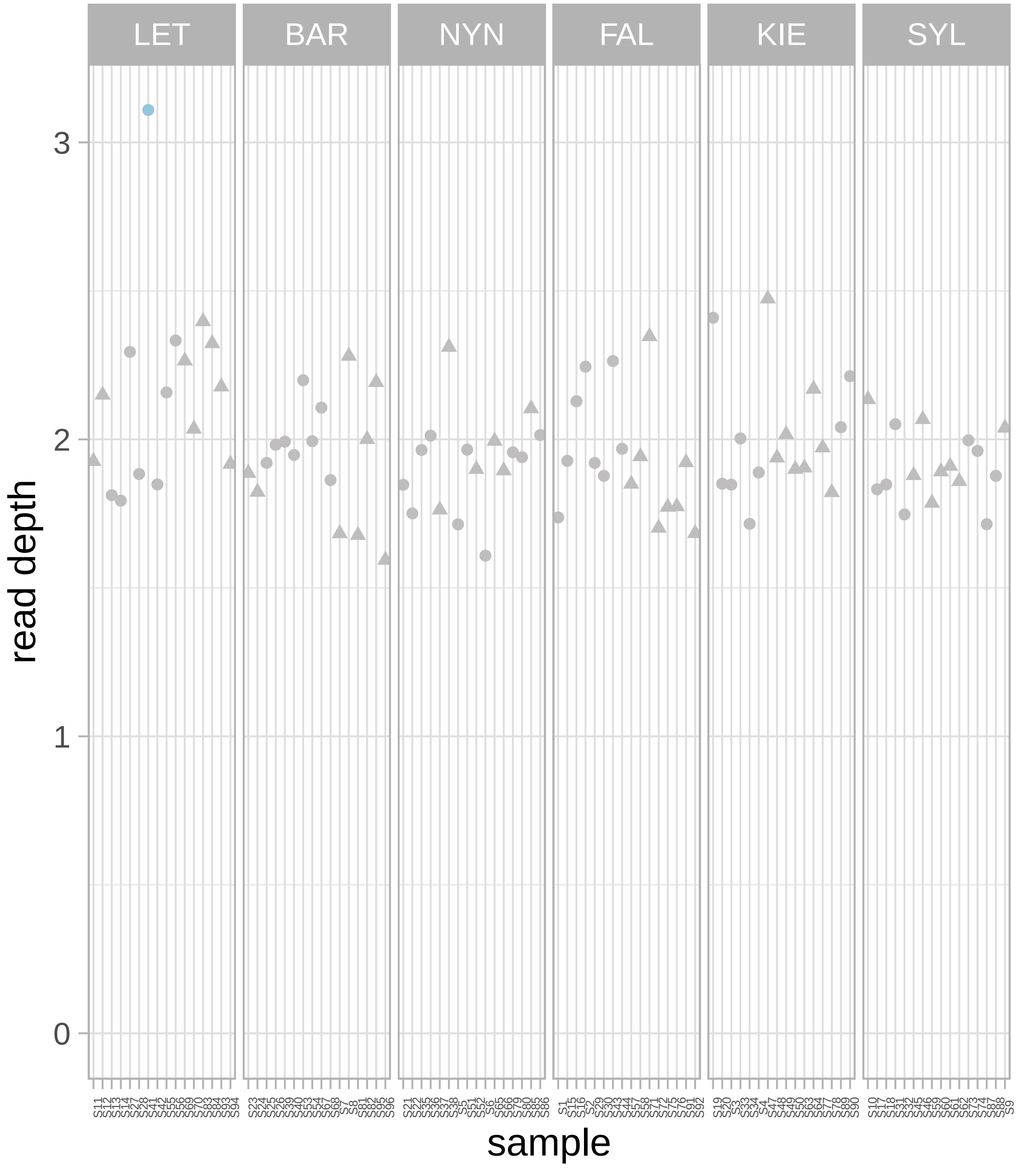

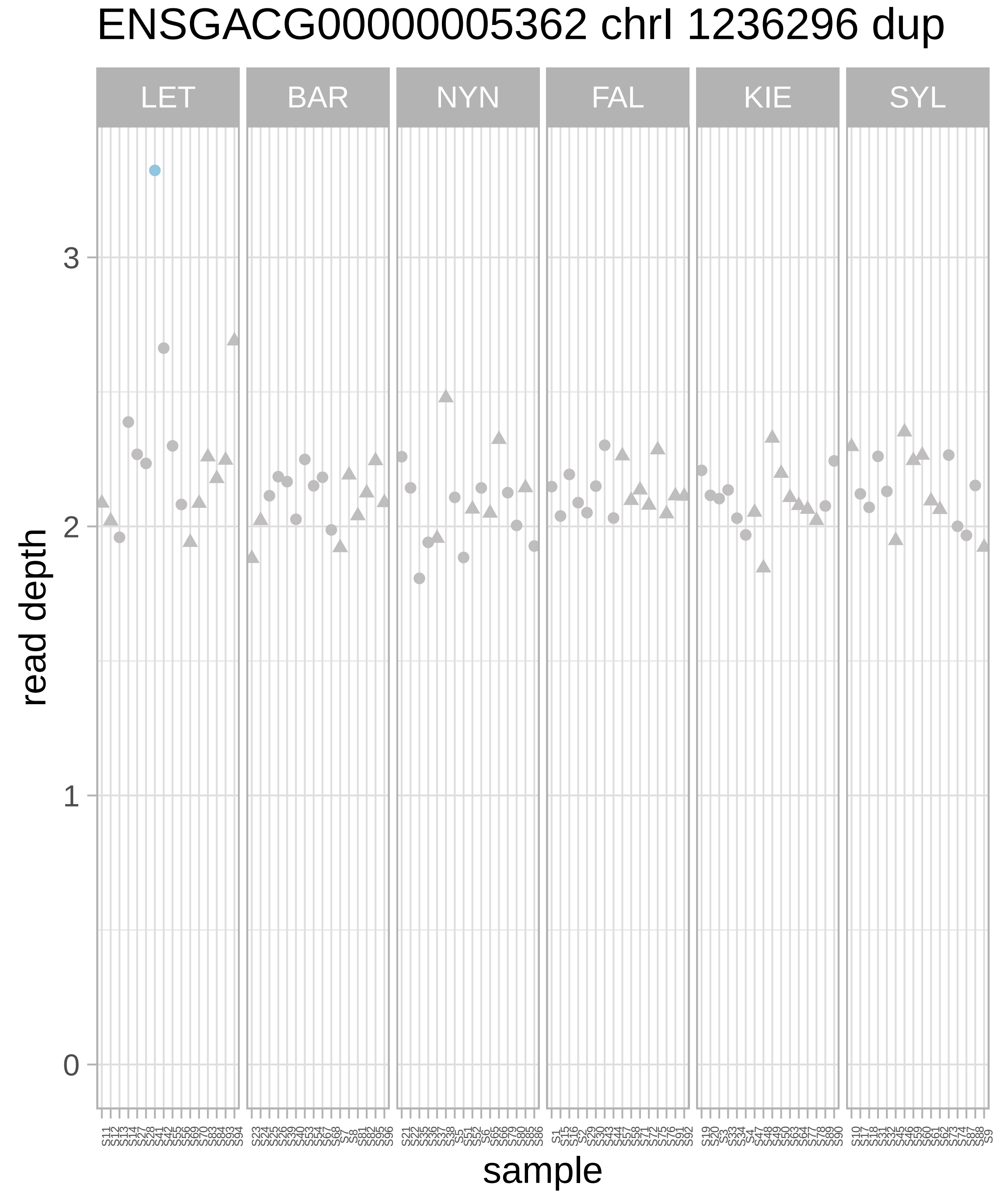
ENSGACG00000005362 chrI 1236296 dup
LET
BAR
NYN
FAL
KIE
SYL
read depth
0
1
2
3
sample

ENSGACG00000005375 chrI 1260570 dup

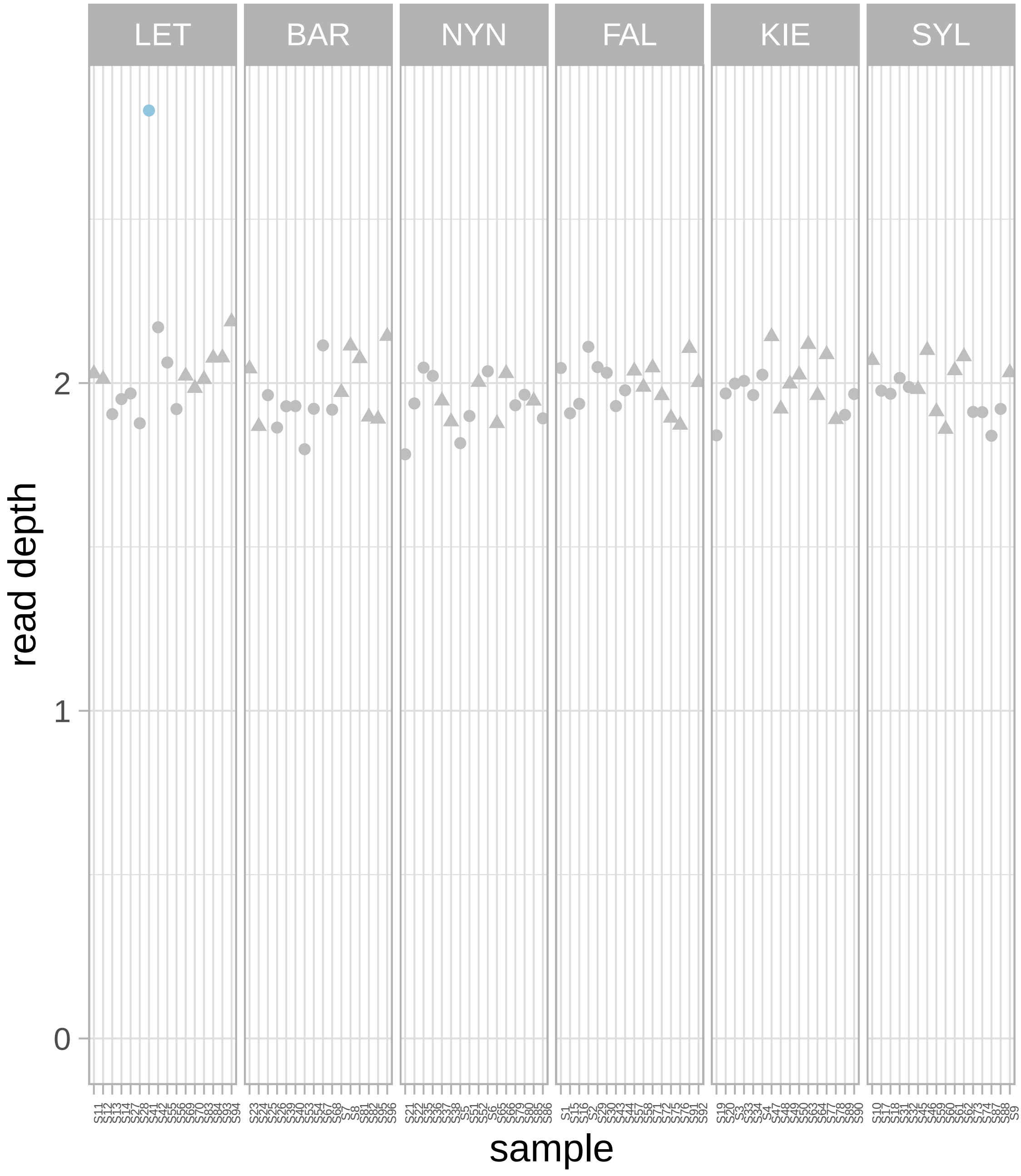

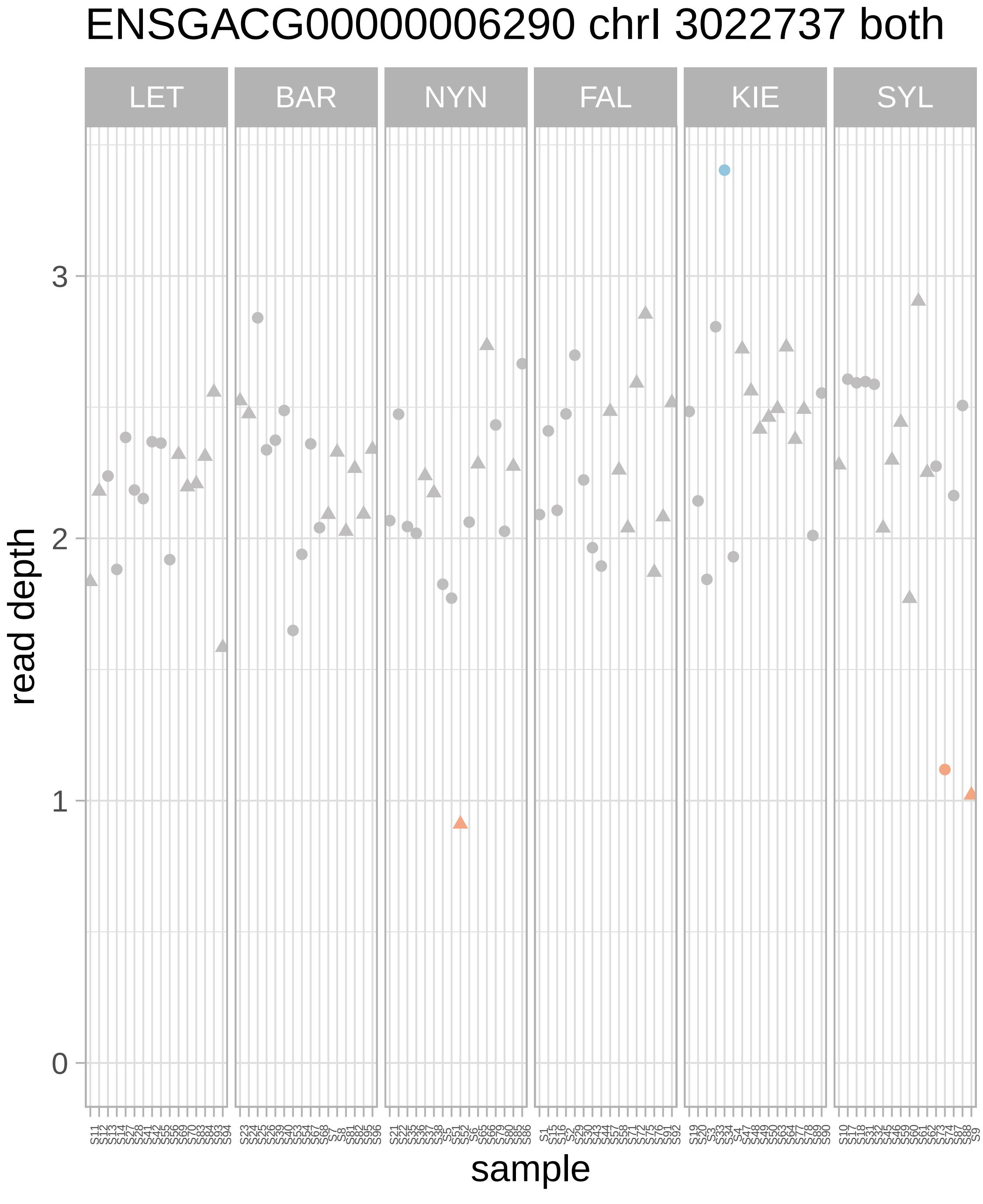
ENSGACG00000006290 chrI 3022737 both
LET
BAR
NYN
FAL
KIE
SYL
read depth
0
1
2
3
sample
S11 S12 S13 S14 S27 S28 S41 S42 S55 S56 S69 S70 S83 S84 S93 S94
S23 S24 S25 S26 S39 S40 S53 S54 S67 S68 S7 S8 S81 S82 S95 S96
S21 S22 S35 S36 S37 S38 S5 S51 S52 S6 S65 S66 S79 S80 S85 S86
S1 S15 S16 S2 S29 S30 S43 S44 S57 S58 S71 S72 S75 S76 S91 S92
S19 S20 S3 S33 S34 S4 S47 S48 S49 S50 S63 S64 S77 S78 S89 S90
S10 S17 S18 S31 S32 S45 S46 S59 S60 S61 S62 S73 S74 S87 S88 S9

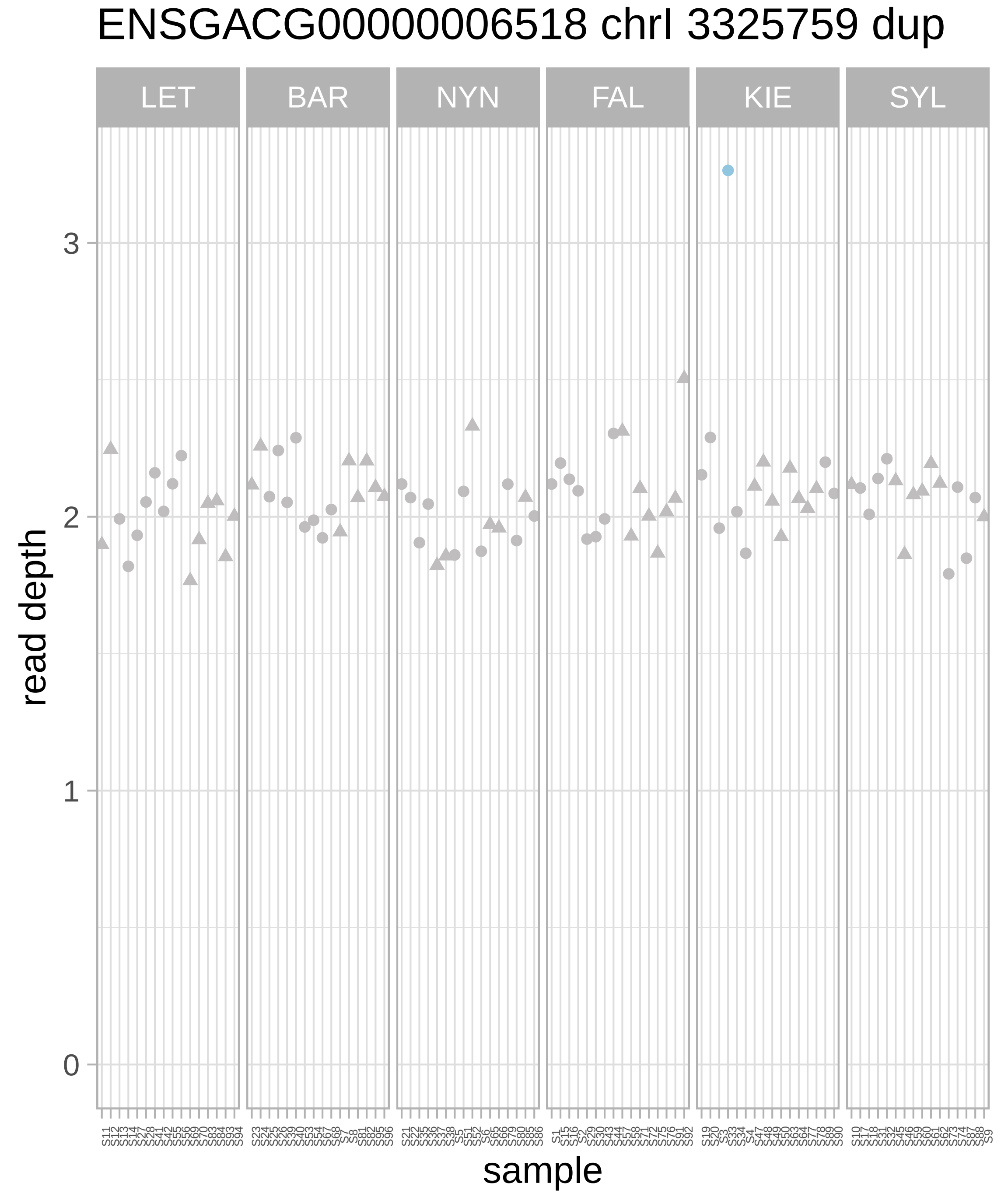

ENSGACG00000006518 chrI 3325759 dup
LET
BAR
NYN
FAL
KIE
SYL
read depth
0
1
2
3
sample

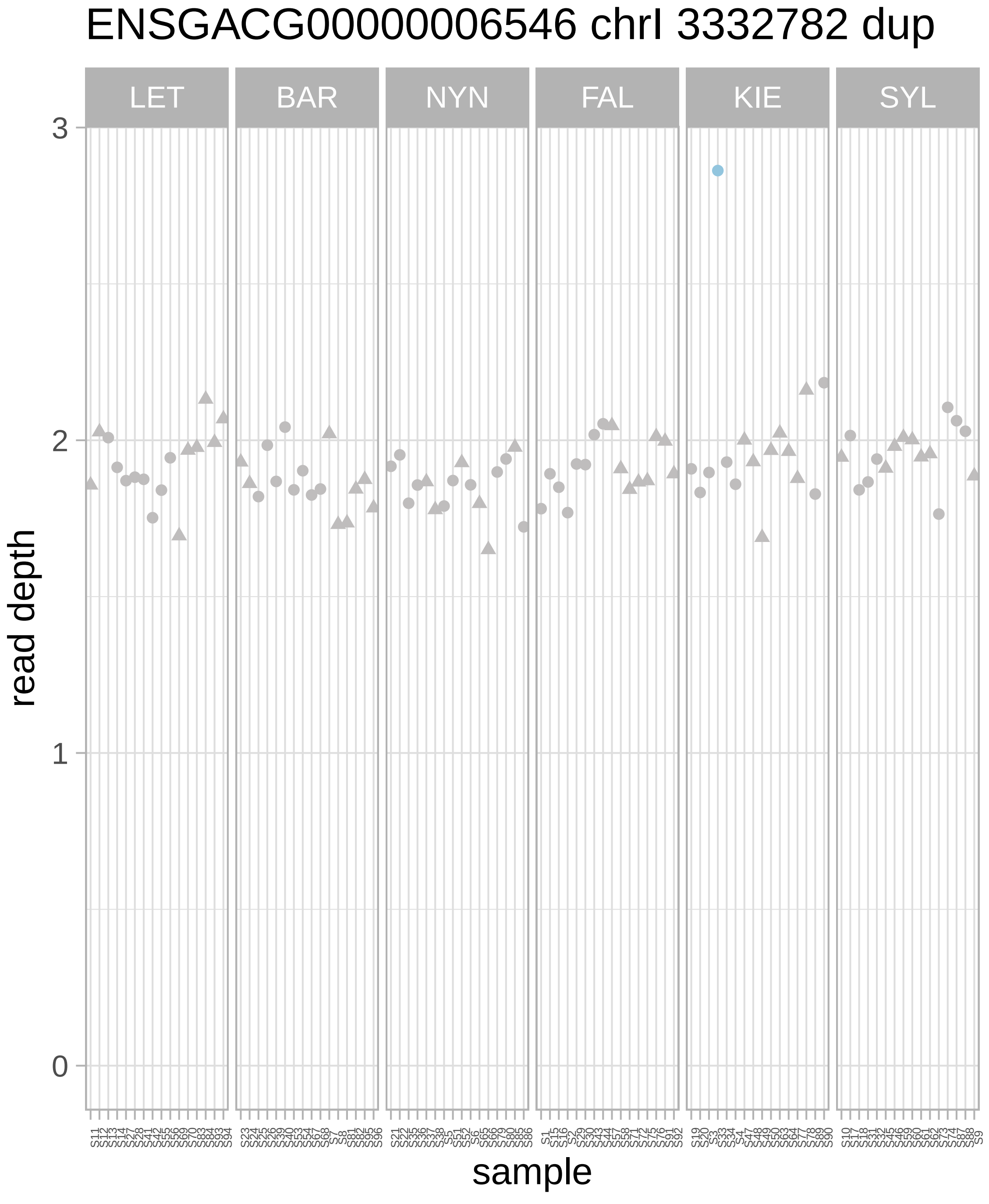
ENSGACG00000006546 chrI 3332782 dup
LET
BAR
NYN
FAL
KIE
SYL
read depth
3
2
1
0
sample
S11 S12 S13 S14 S27 S28 S41 S42 S55 S56 S69 S70 S83 S84 S93 S94
S23 S24 S25 S26 S39 S40 S53 S54 S67 S68 S7 S8 S81 S82 S95 S96
S21 S22 S35 S36 S37 S38 S5 S51 S52 S6 S65 S66 S79 S80 S85 S86
S1 S15 S16 S2 S29 S30 S43 S44 S57 S58 S71 S72 S75 S76 S91 S92
S19 S20 S3 S33 S34 S4 S47 S48 S49 S50 S63 S64 S77 S78 S89 S90
S10 S17 S18 S31 S32 S45 S46 S59 S60 S61 S62 S73 S74 S87 S88 S9

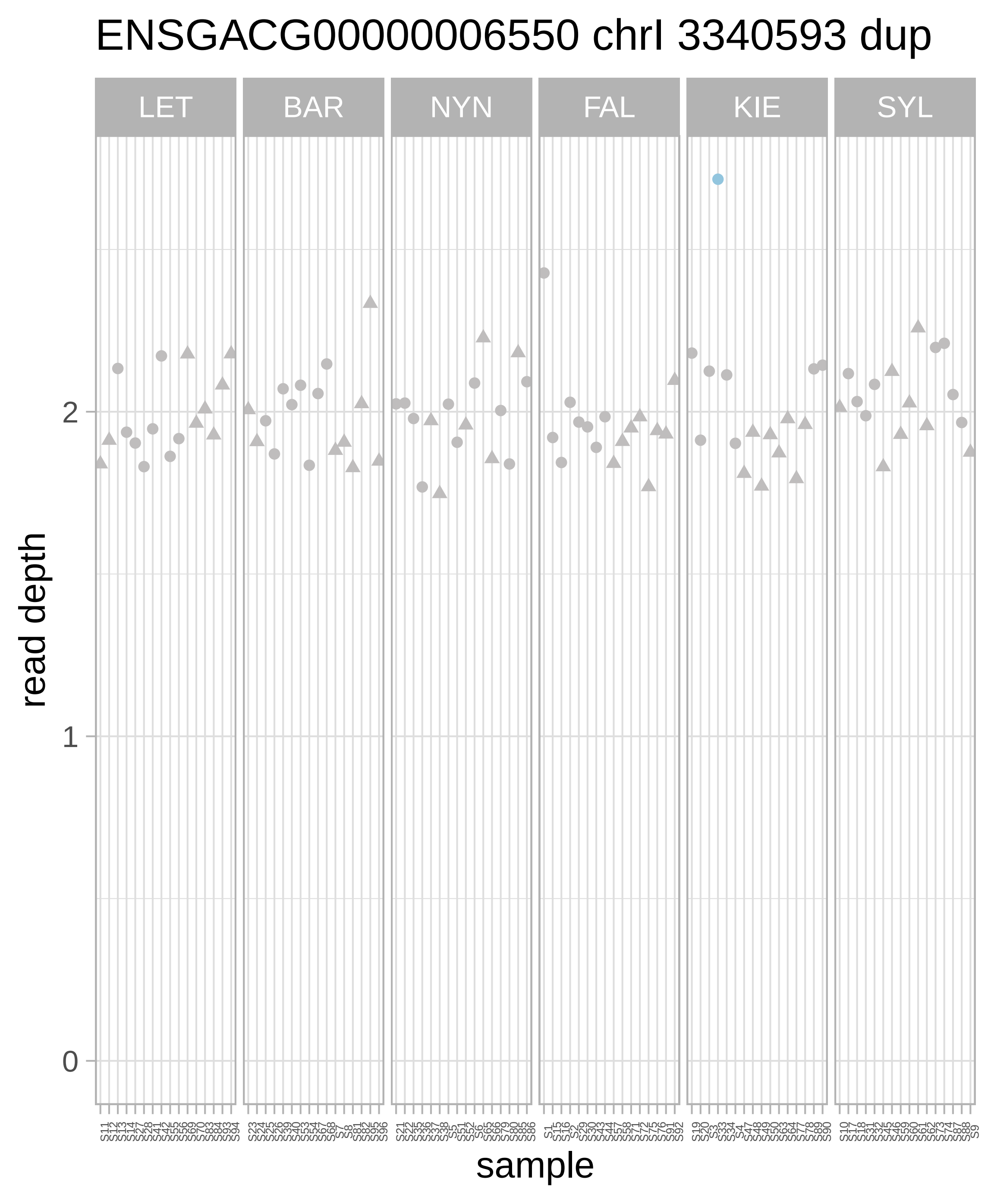
ENSGACG00000006550 chrI 3340593 dup
LET
BAR
NYN
FAL
KIE
SYL
read depth
0
1
2
sample
S11 S12 S13 S14 S27 S28 S41 S42 S55 S56 S69 S70 S83 S84 S93 S94
S23 S24 S25 S26 S39 S40 S53 S54 S67 S68 S7 S8 S81 S82 S95 S96
S21 S22 S35 S36 S37 S38 S5 S51 S52 S6 S65 S66 S79 S80 S85 S86
S1 S15 S16 S2 S29 S30 S43 S44 S57 S58 S71 S72 S75 S76 S91 S92
S19 S20 S3 S33 S34 S4 S47 S48 S49 S50 S63 S64 S77 S78 S89 S90
S10 S17 S18 S31 S32 S45 S46 S59 S60 S61 S62 S73 S74 S87 S88 S9

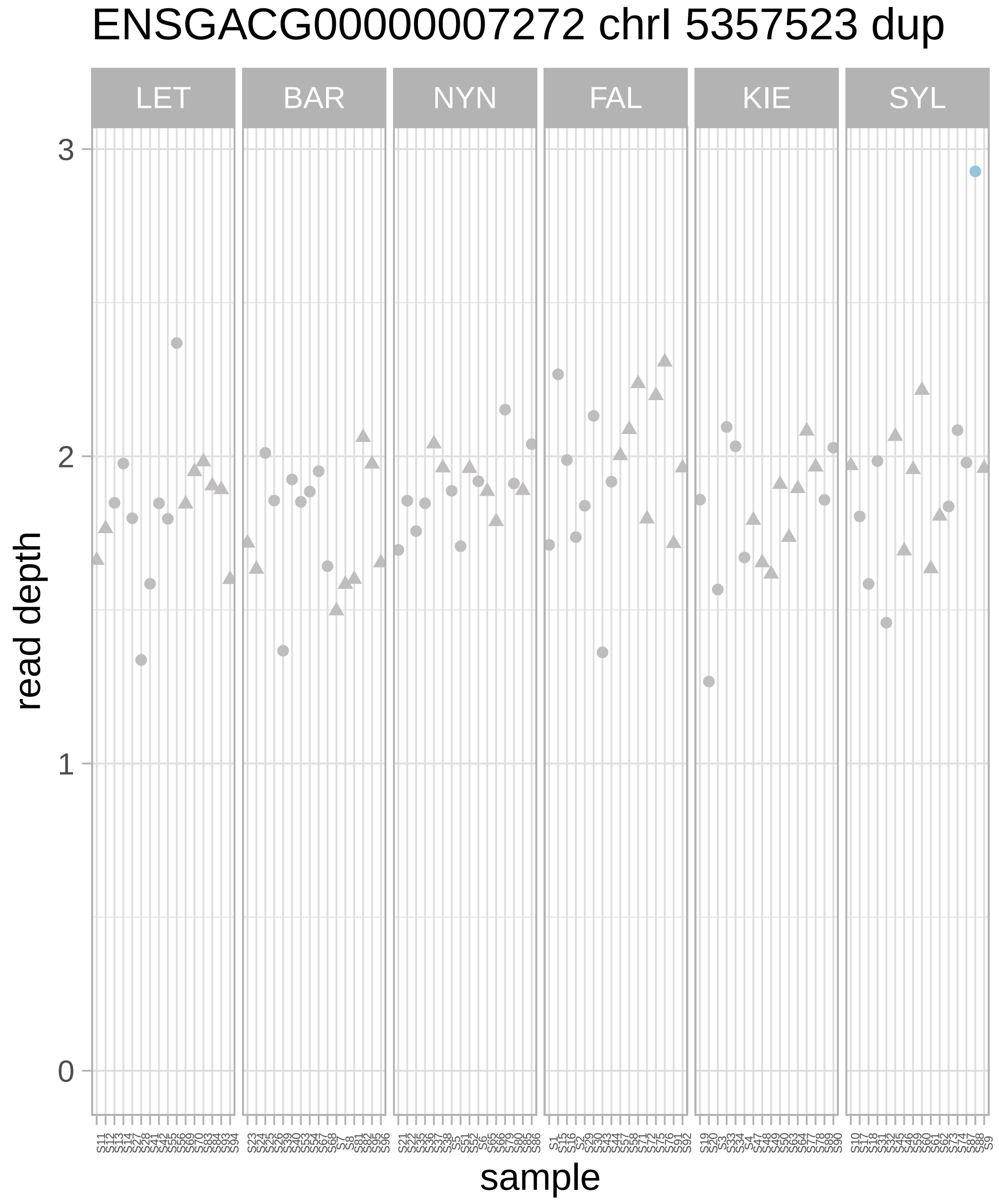
ENSGACG00000007272 chrI 5357523 dup
LET
BAR
NYN
FAL
KIE
SYL
read depth
3
2
1
0
S11 S12 S13 S14 S27 S28 S41 S42 S55 S56 S69 S70 S83 S84 S93 S94
S23 S24 S25 S26 S39 S40 S53 S54 S67 S68 S7 S8 S81 S82 S95 S96
S21 S22 S35 S36 S37 S38 S5 S51 S52 S6 S65 S66 S79 S80 S85 S86
S1 S15 S16 S2 S29 S30 S43 S44 S57 S58 S71 S72 S75 S76 S91 S92
S19 S20 S3 S33 S34 S4 S47 S48 S49 S50 S63 S64 S77 S78 S89 S90
S10 S17 S18 S31 S32 S45 S46 S59 S60 S61 S62 S73 S74 S87 S88 S9
sample

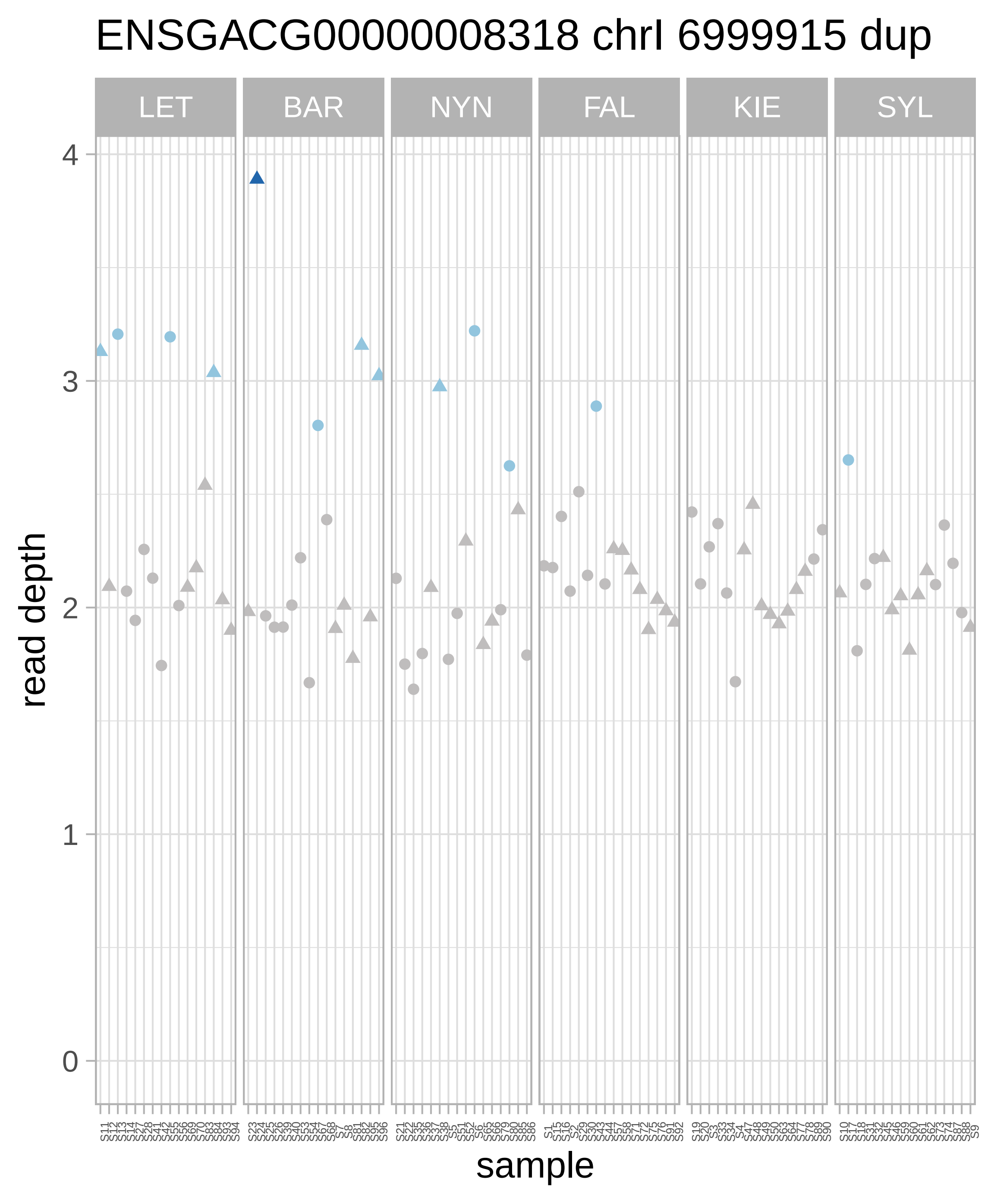
ENSGACG00000008318 chrI 6999915 dup
LET
BAR
NYN
FAL
KIE
SYL
4
3
2
1
0
read depth
sample

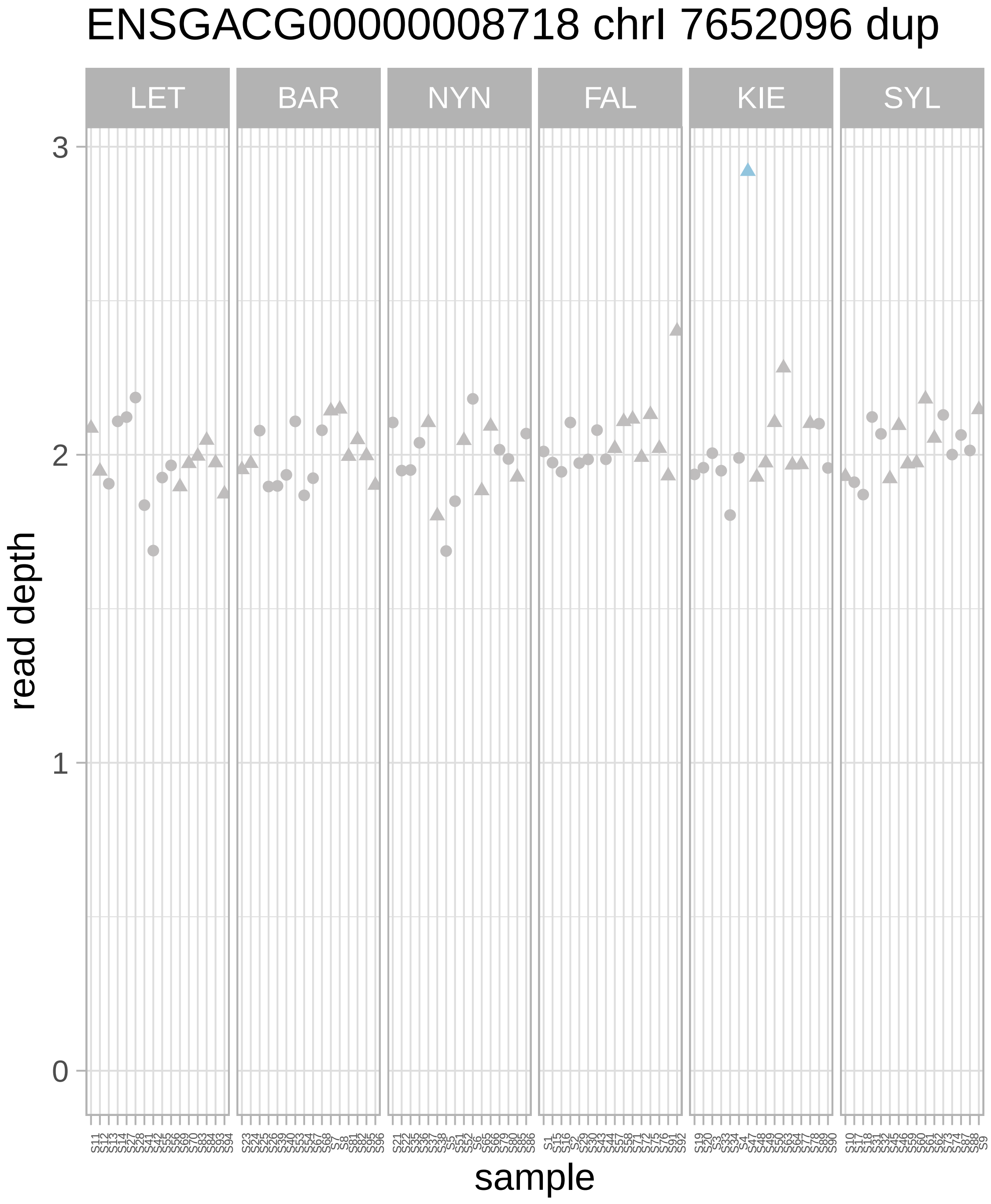
ENSGACG00000008718 chrI 7652096 dup
LET
BAR
NYN
FAL
KIE
SYL
read depth
0
1
2
3
S11 S12 S13 S14 S27 S28 S41 S42 S55 S56 S69 S70 S83 S84 S93 S94
S23 S24 S25 S26 S39 S40 S53 S54 S67 S68 S7 S8 S81 S82 S95 S96
S21 S22 S35 S36 S37 S38 S5 S51 S52 S6 S65 S66 S79 S80 S85 S86
S1 S15 S16 S2 S29 S30 S43 S44 S57 S58 S71 S72 S75 S76 S91 S92
S19 S20 S3 S33 S34 S4 S47 S48 S49 S50 S63 S64 S77 S78 S89 S90
S10 S17 S18 S31 S32 S45 S46 S59 S60 S61 S62 S73 S74 S87 S88 S9
sample

ENSGACG00000008738 chrI 7711423 dup

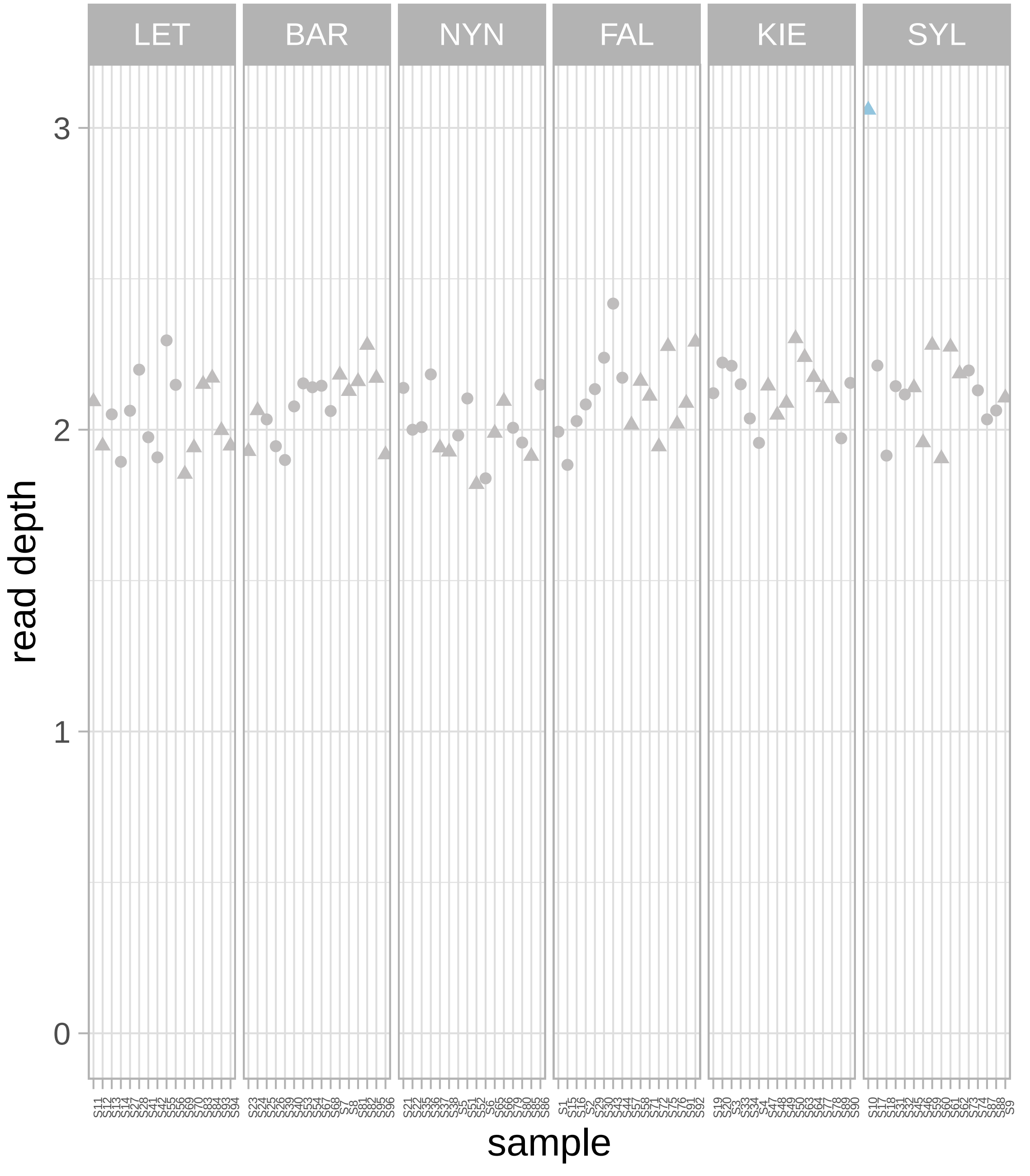

ENSGACG00000009158 chrI 8533756 dup

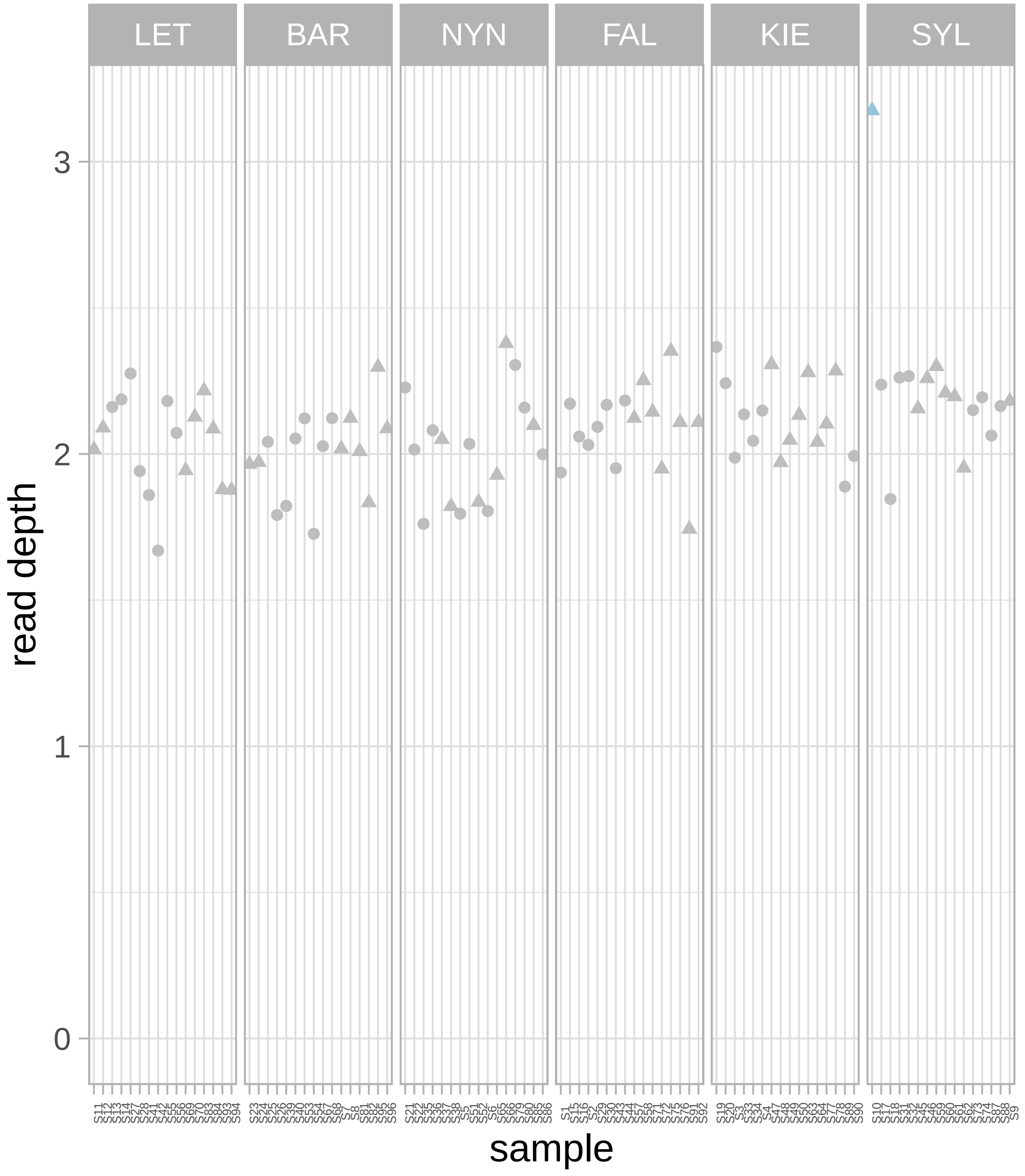

ENSGACG00000009165 chrI 8558660 dup

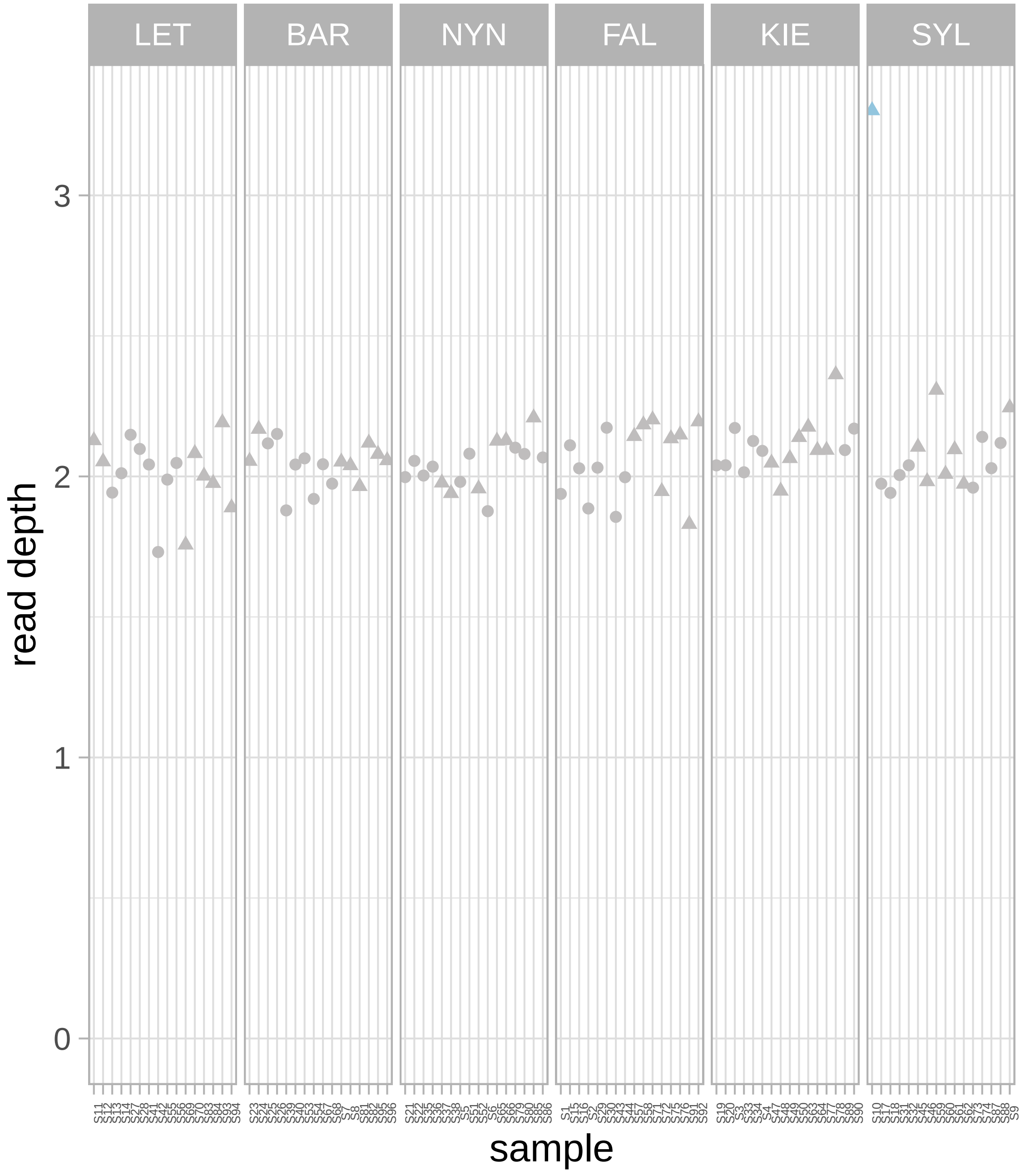

ENSGACG00000009171 chrI 8575329 dup

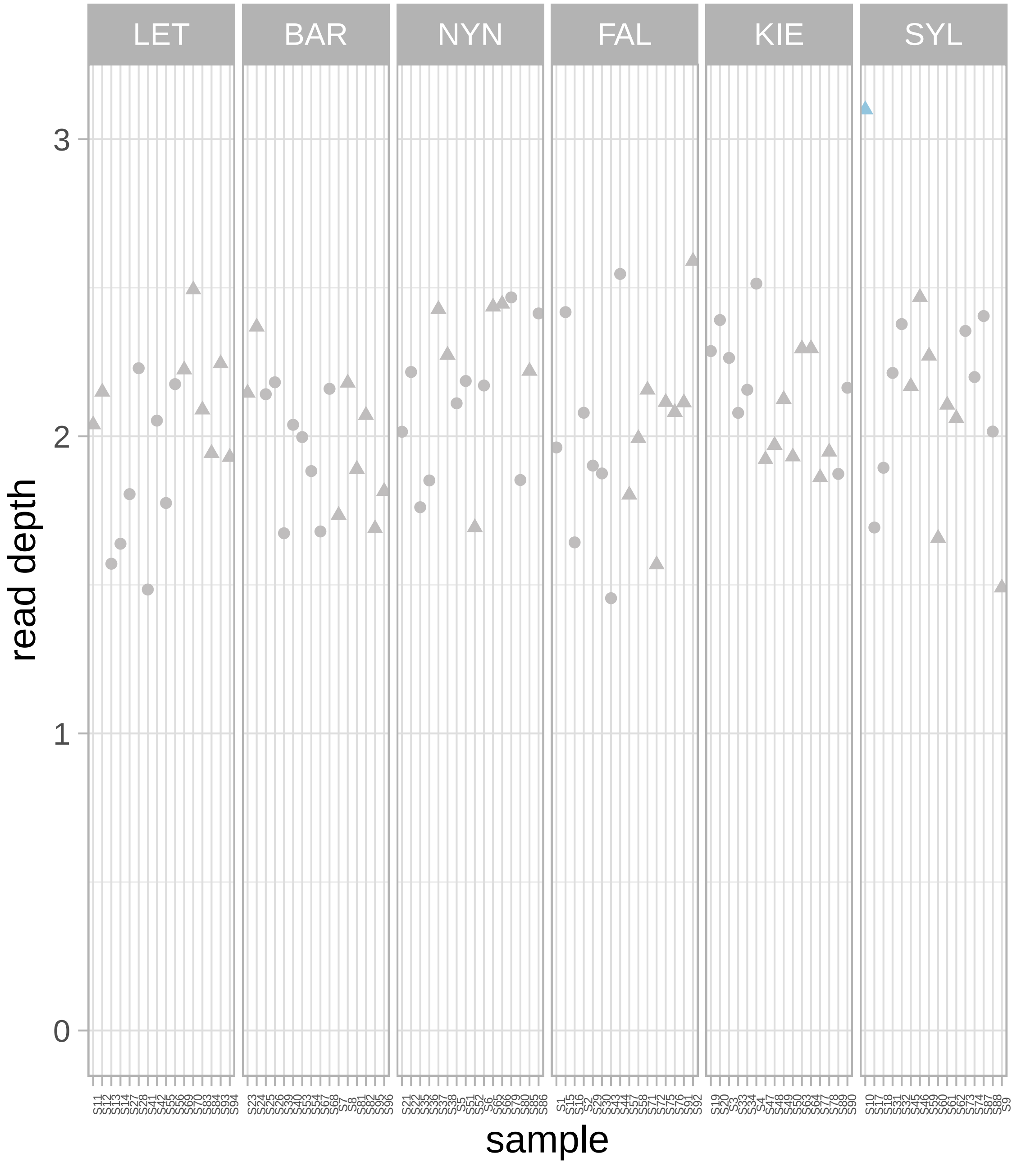

ENSGACG00000010313 chrI 10616414 dup

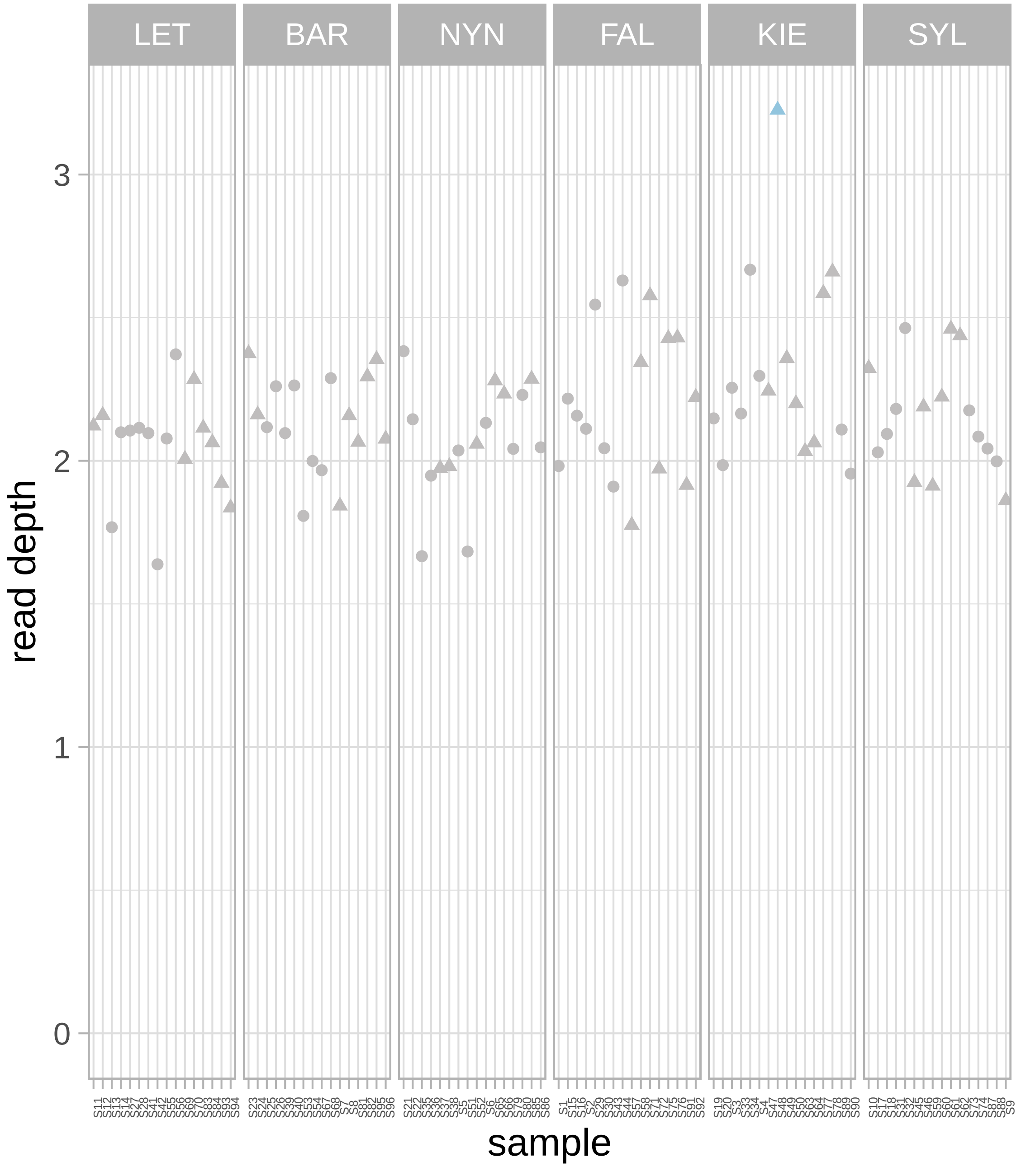

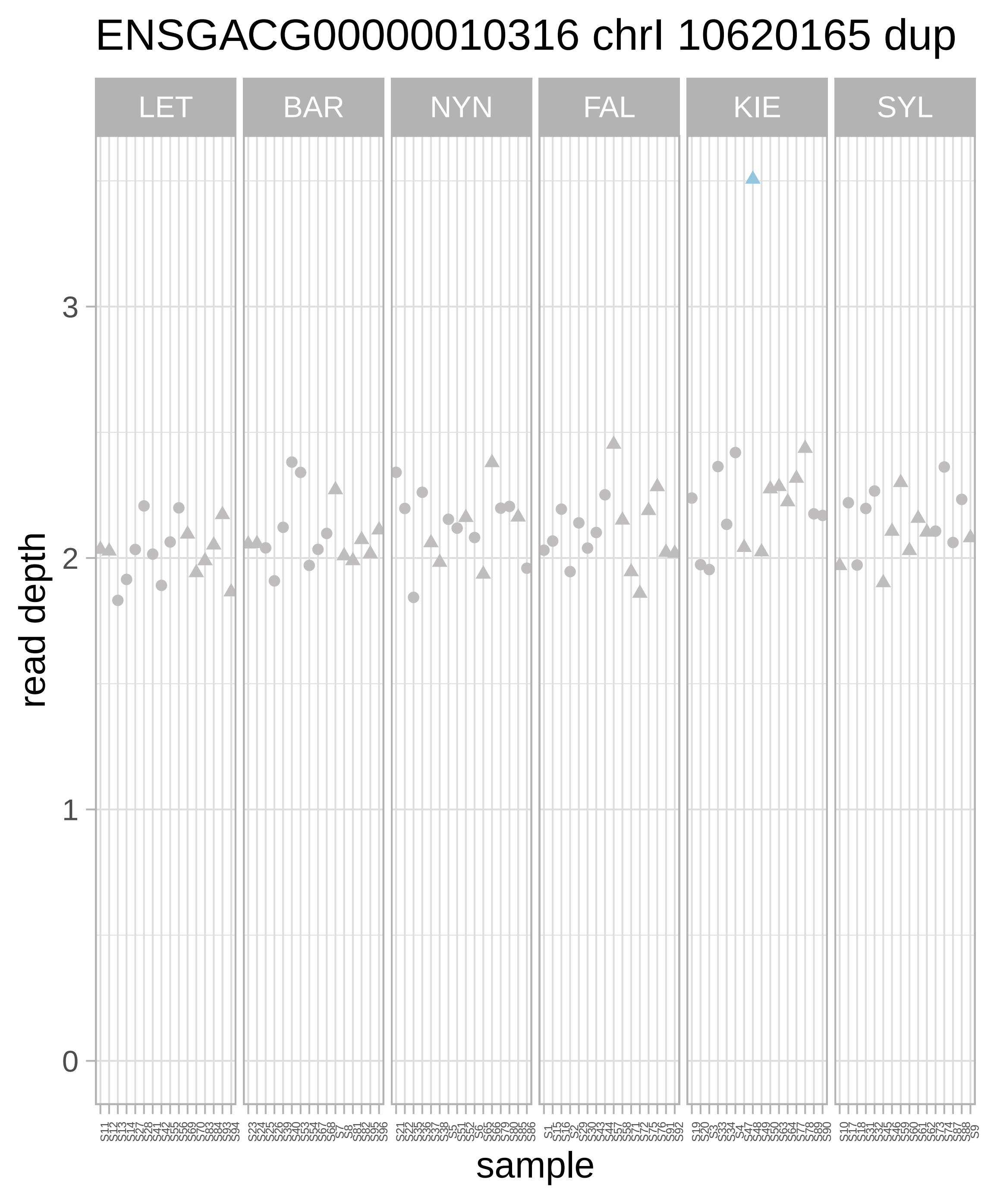
ENSGACG00000010316 chrI 10620165 dup
LET
BAR
NYN
FAL
KIE
SYL
read depth
0
1
2
3
sample

ENSGACG00000011281 chrI 12379162 dup

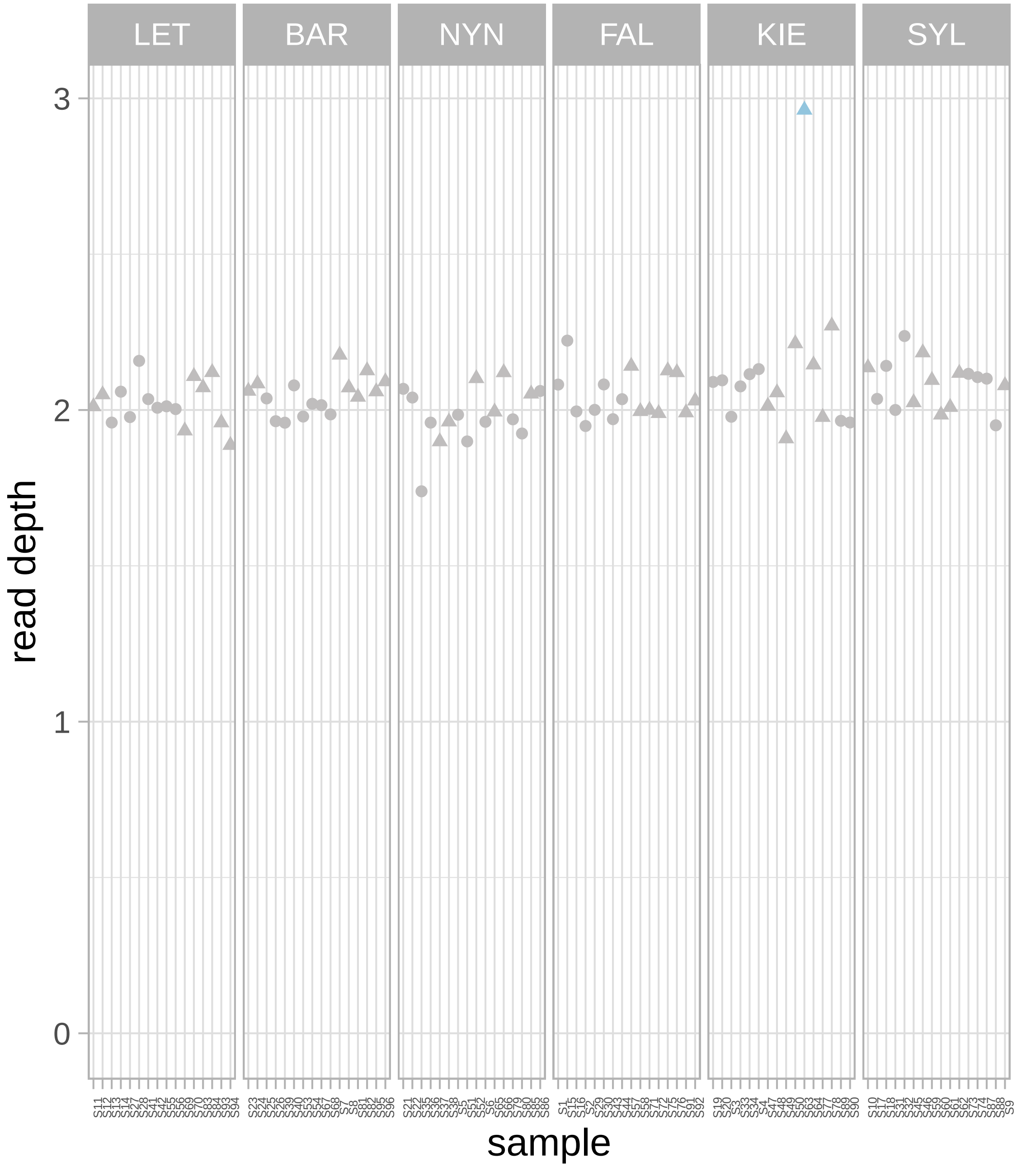

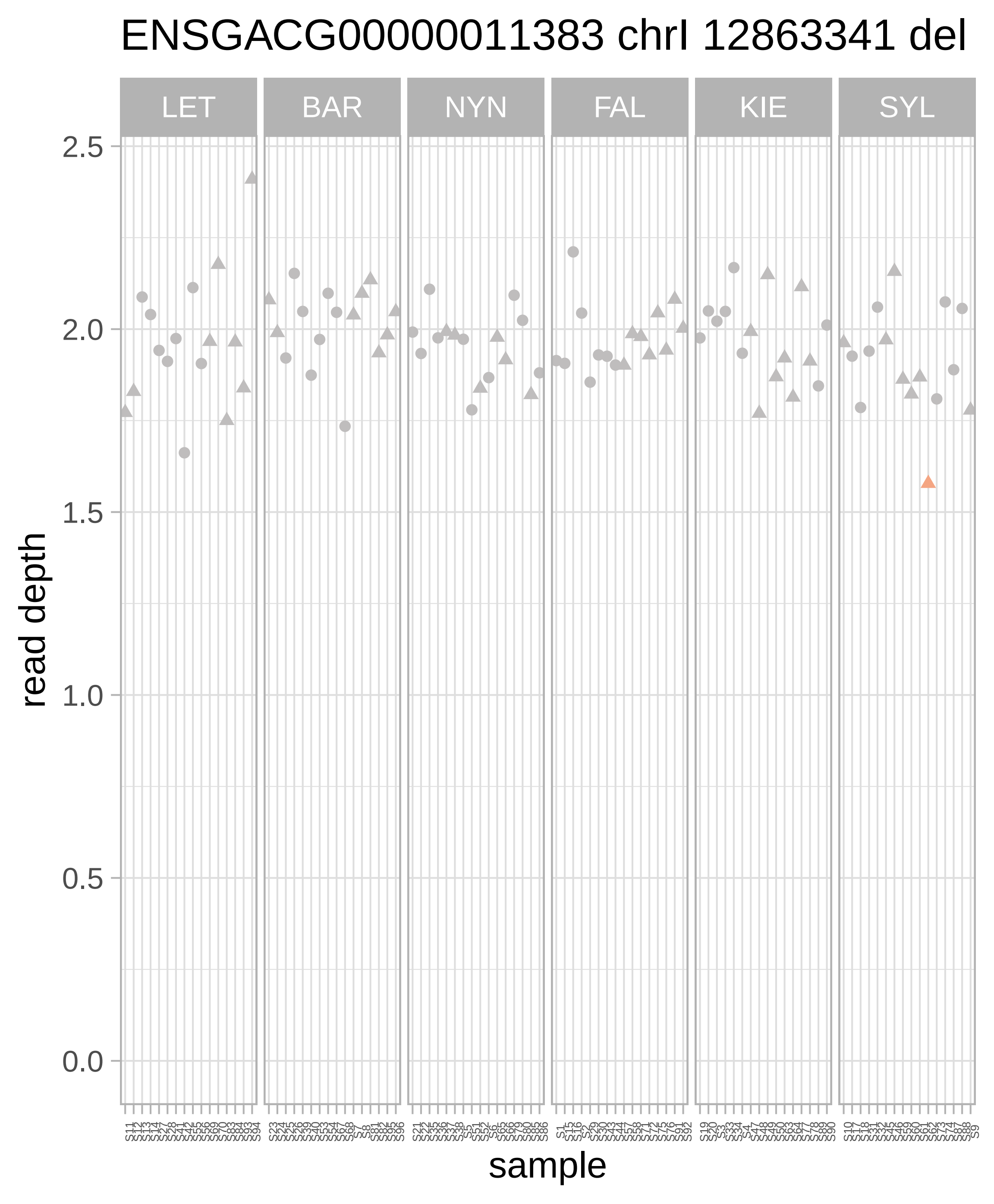

ENSGACG00000011383 chrI 12863341 del
LET
BAR
NYN
FAL
KIE
SYL
read depth
sample
0.0
0.5
1.0
1.5
2.0
2.5

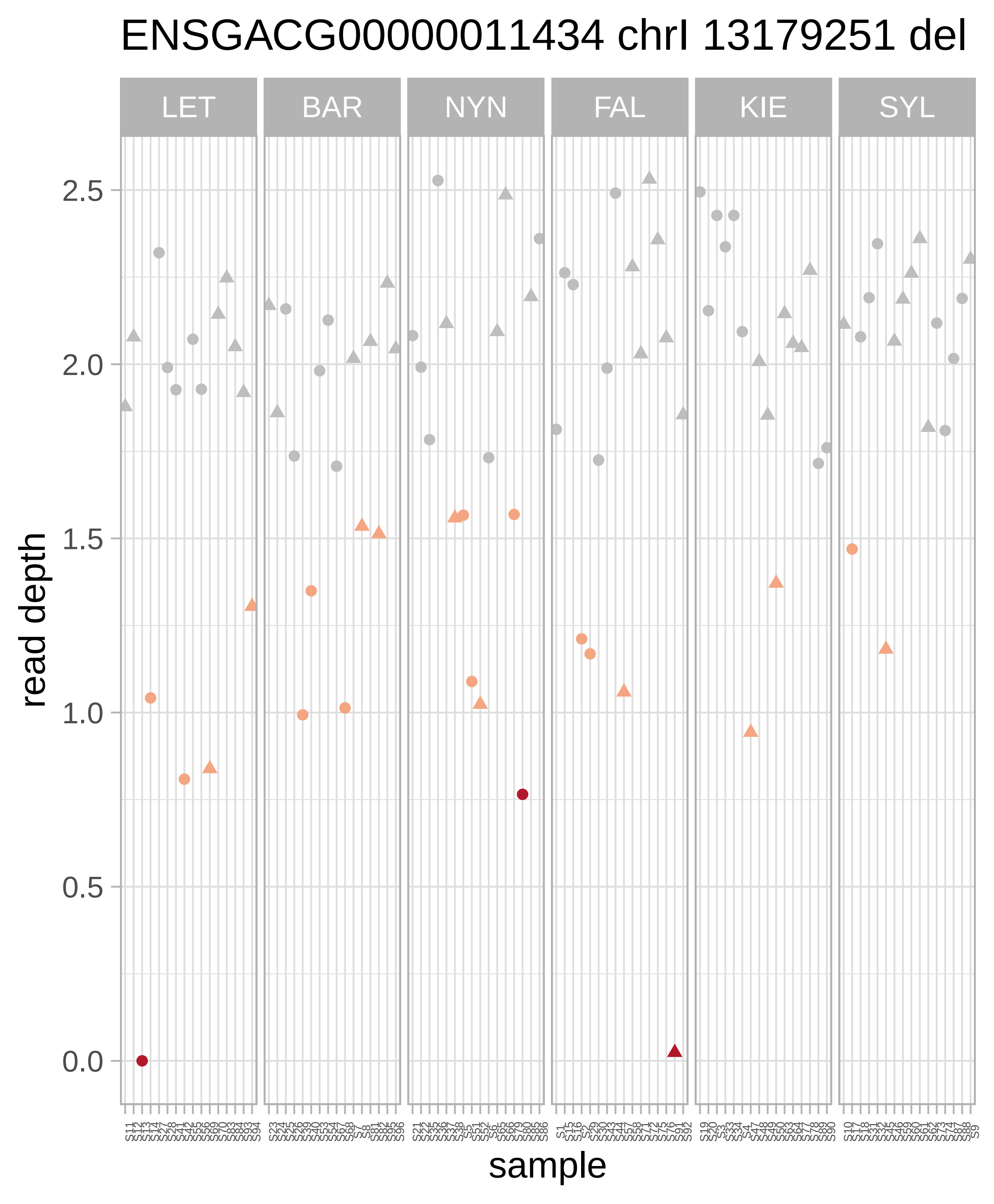
ENSGACG00000011434 chrI 13179251 del
LET
BAR
NYN
FAL
KIE
SYL
read depth
2.5
2.0
1.5
1.0
0.5
0.0
sample

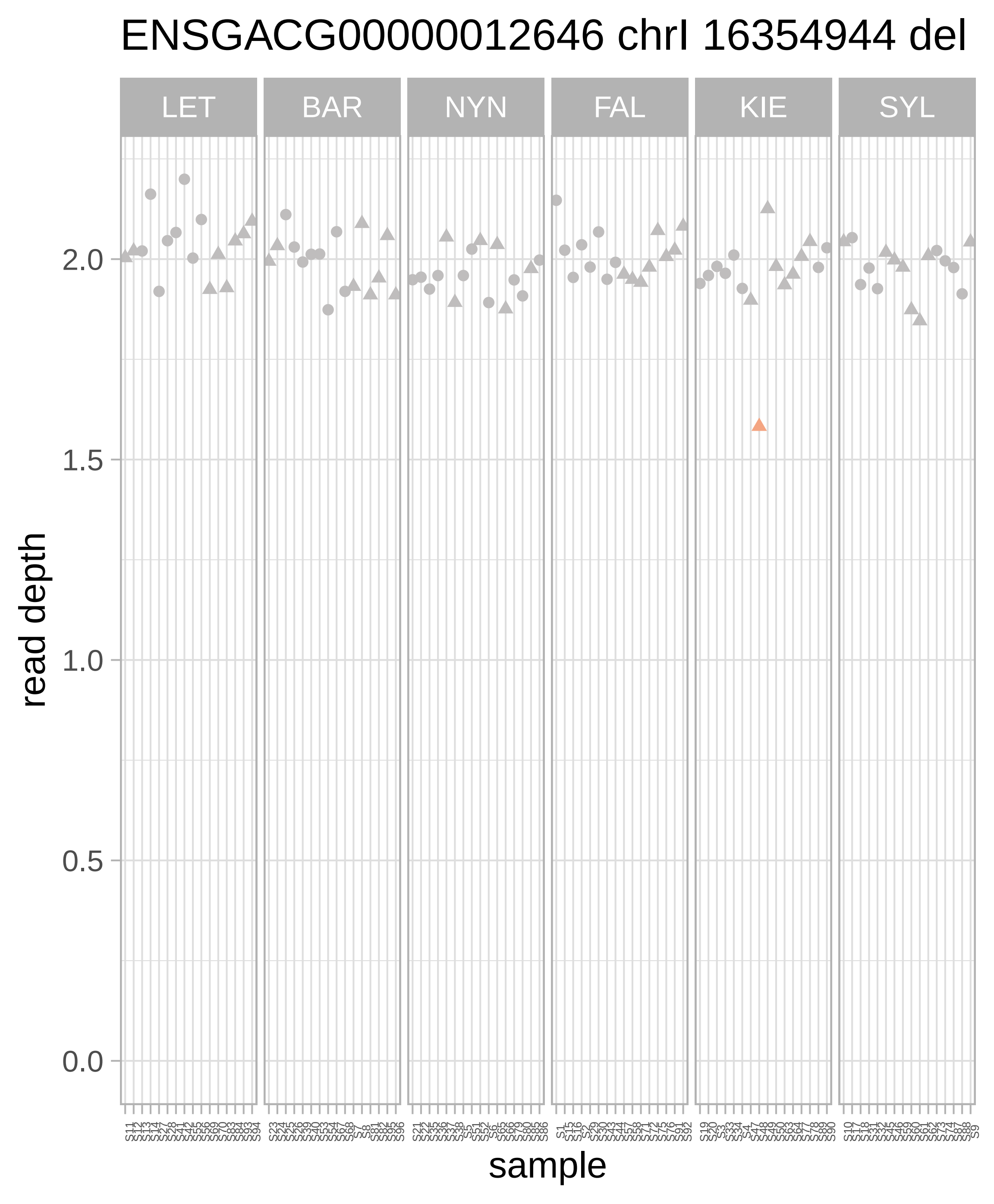
ENSGACG00000012646 chrI 16354944 del
LET
BAR
NYN
FAL
KIE
SYL
read depth
2.0
1.5
1.0
0.5
0.0
sample

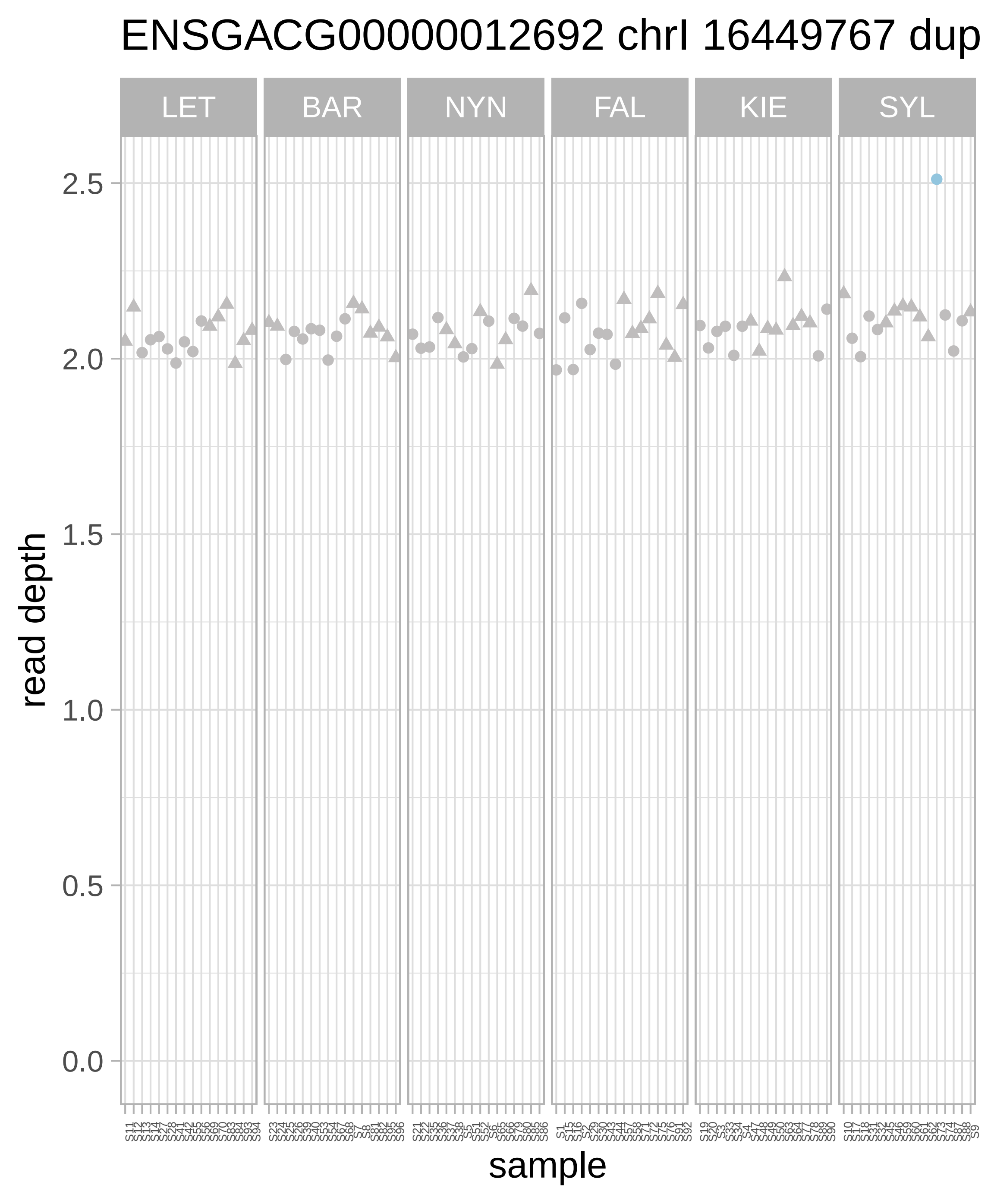
ENSGACG00000012692 chrI 16449767 dup
LET
BAR
NYN
FAL
KIE
SYL
read depth
2.5
2.0
1.5
1.0
0.5
0.0
S11 S12 S13 S14 S27 S28 S41 S42 S55 S56 S69 S70 S83 S84 S93 S94
S23 S24 S25 S26 S39 S40 S53 S54 S67 S68 S7 S8 S81 S82 S95 S96
S21 S22 S35 S36 S37 S38 S5 S51 S52 S6 S65 S66 S79 S80 S85 S86
S1 S15 S16 S2 S29 S30 S43 S44 S57 S58 S71 S72 S75 S76 S91 S92
S19 S20 S3 S33 S34 S4 S47 S48 S49 S50 S63 S64 S77 S78 S89 S90
S10 S17 S18 S31 S32 S45 S46 S59 S60 S61 S62 S73 S74 S87 S88 S9
sample

ENSGACG00000013452 chrI 18084023 dup

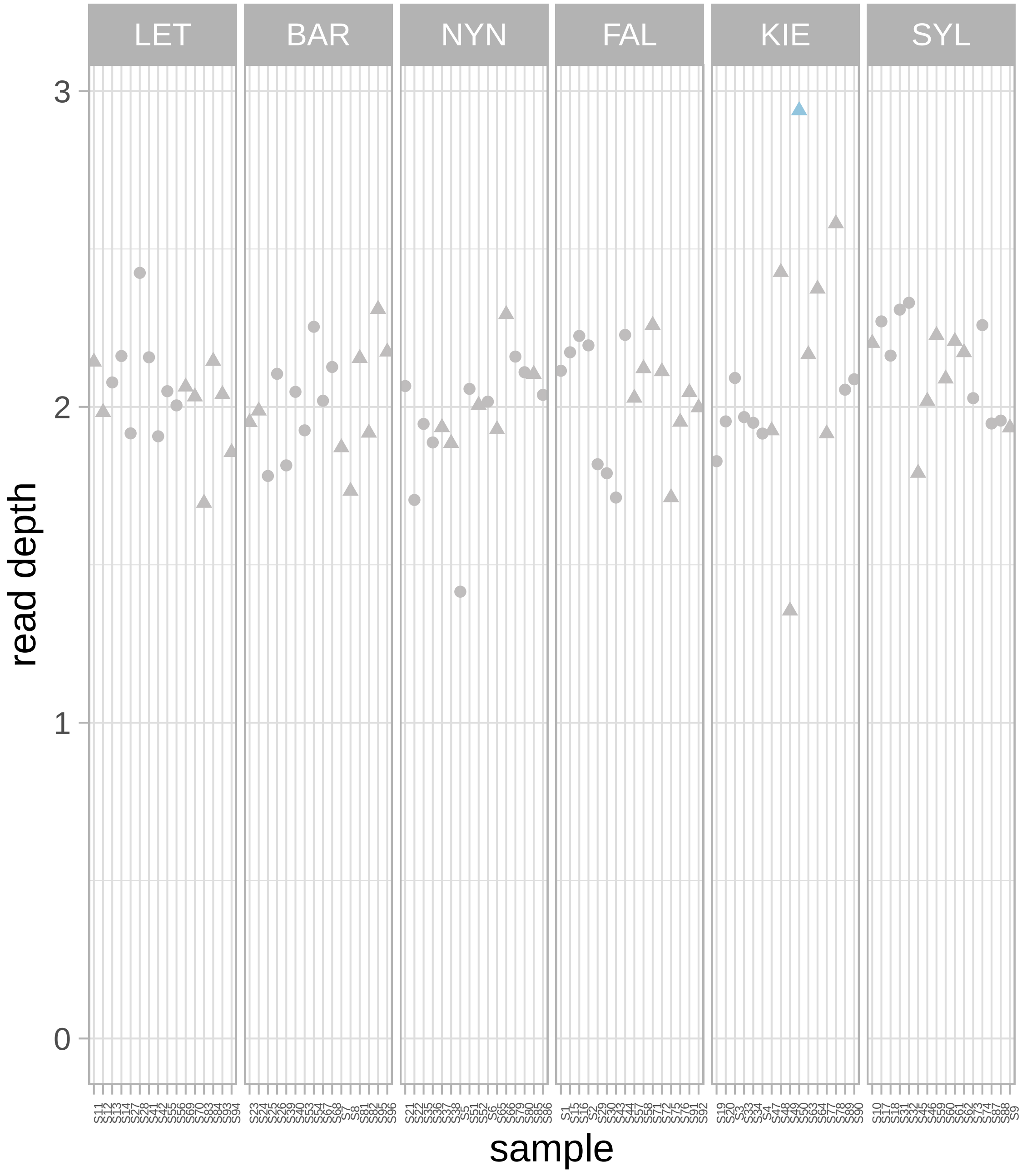

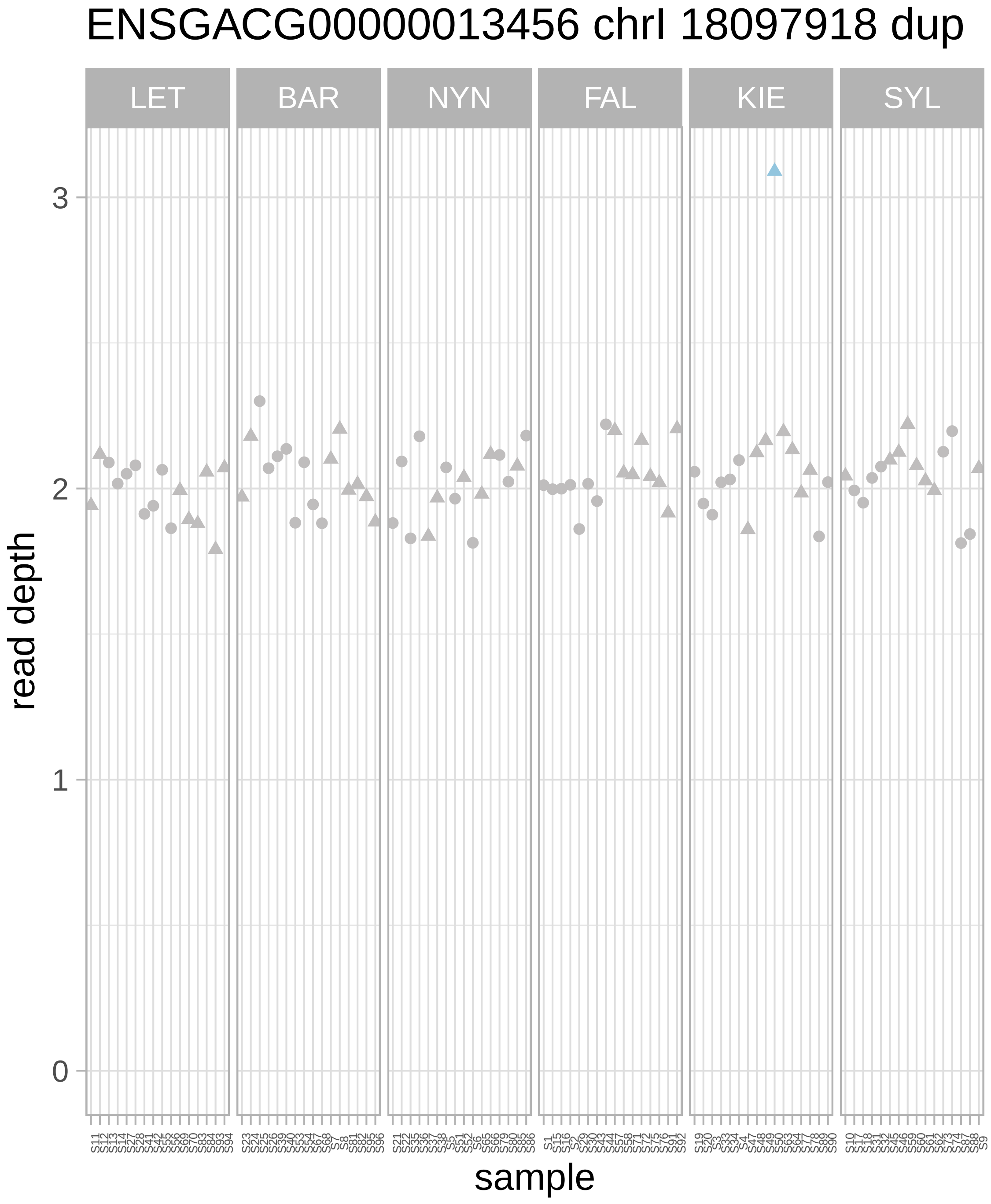
ENSGACG00000013456 chrI 18097918 dup
LET
BAR
NYN
FAL
KIE
SYL
read depth
3
2
1
0
sample

ENSGACG00000014203 chrI 21140857 dup

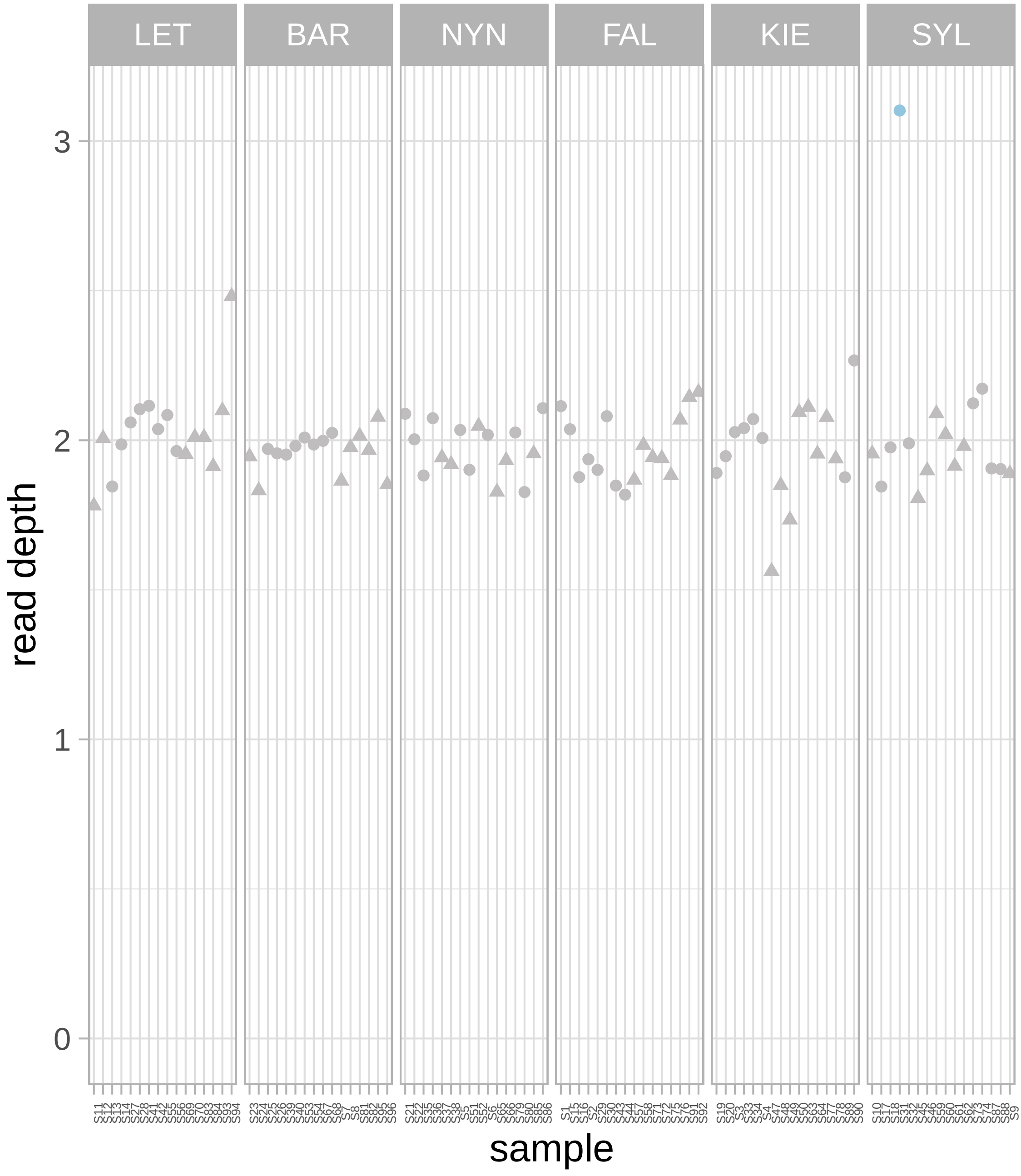

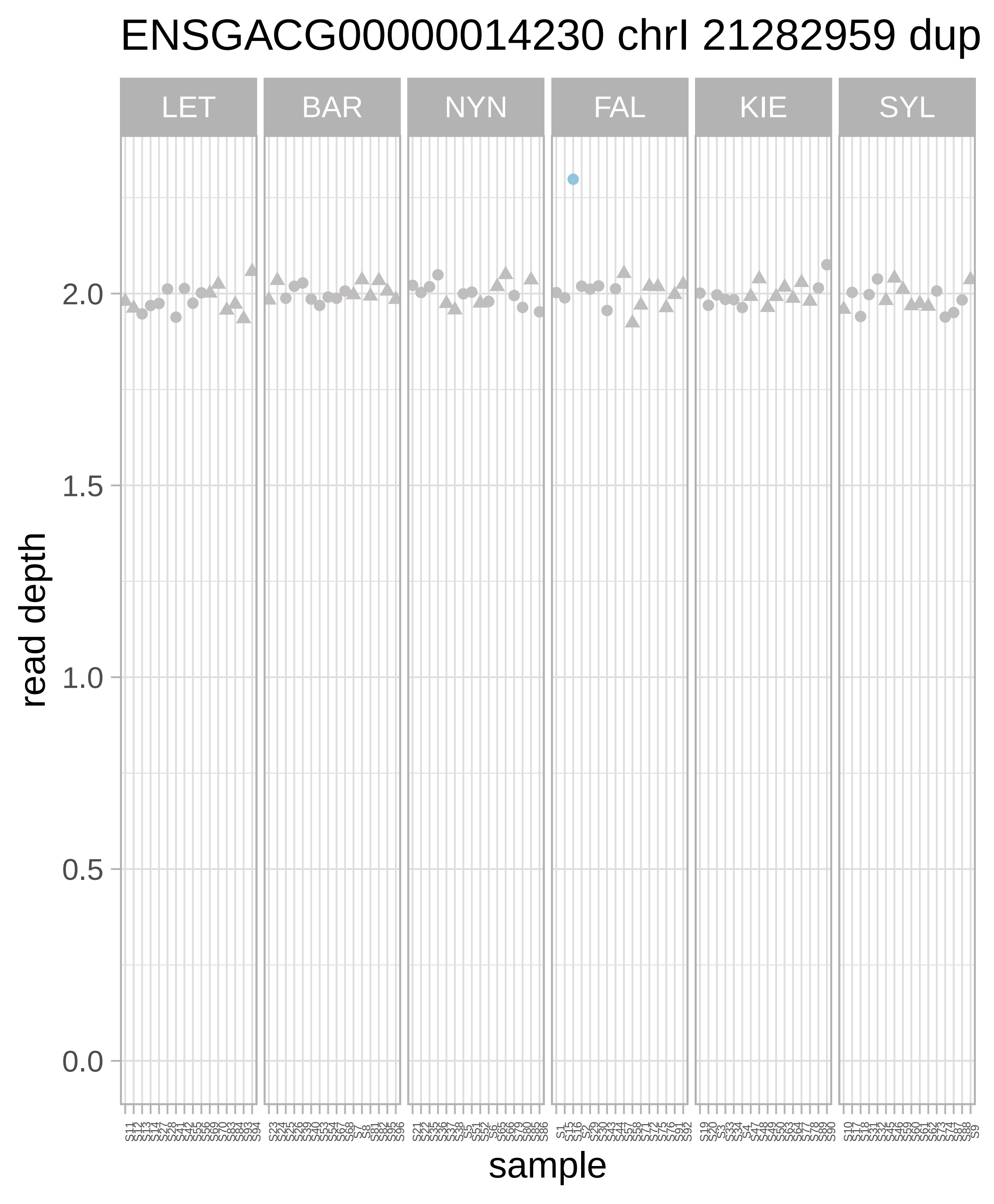

ENSGACG00000014230 chrI 21282959 dup
LET
BAR
NYN
FAL
KIE
SYL
read depth
0.0
0.5
1.0
1.5
2.0
sample

ENSGACG00000014436 chrI 22067856 dup

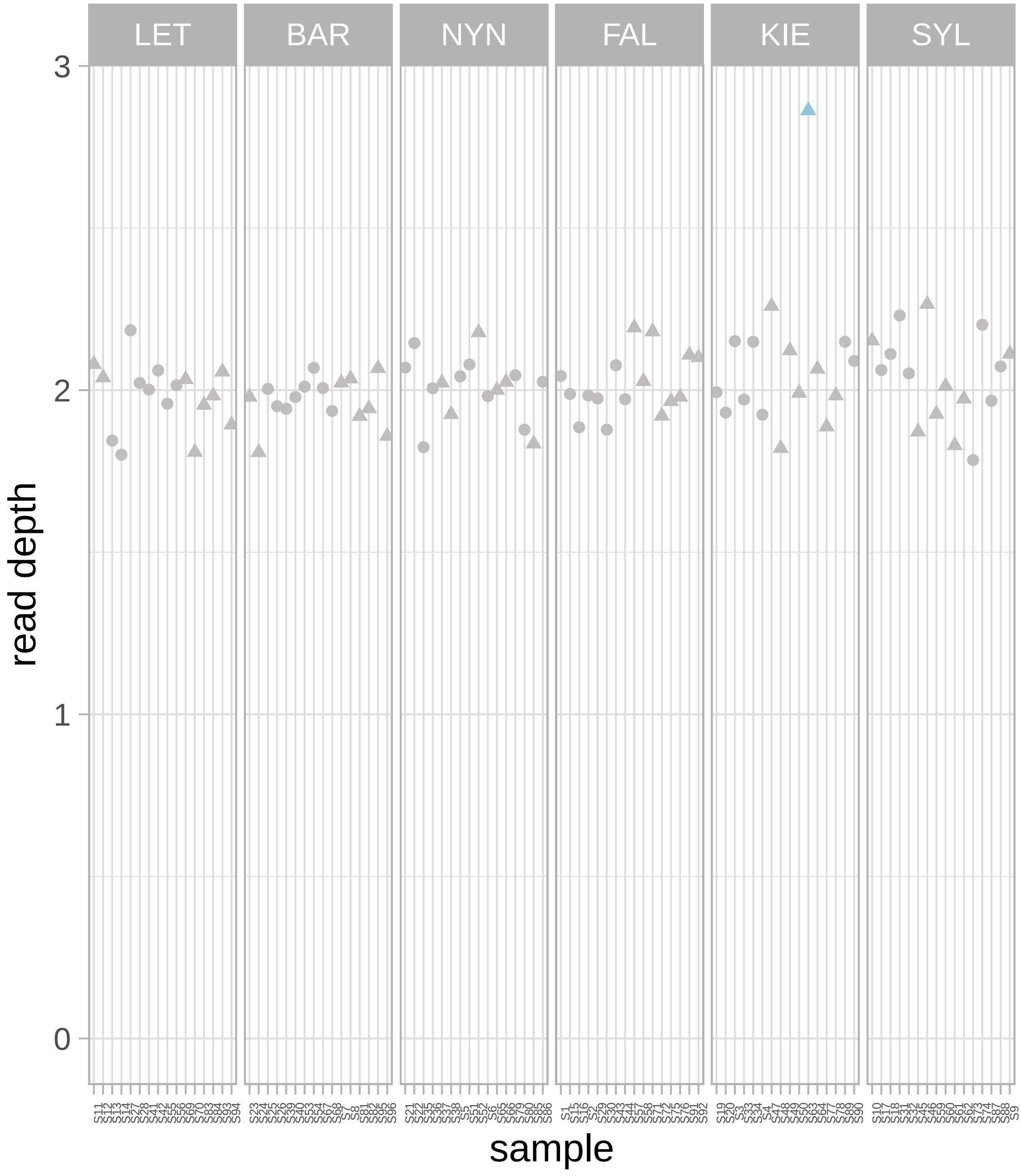

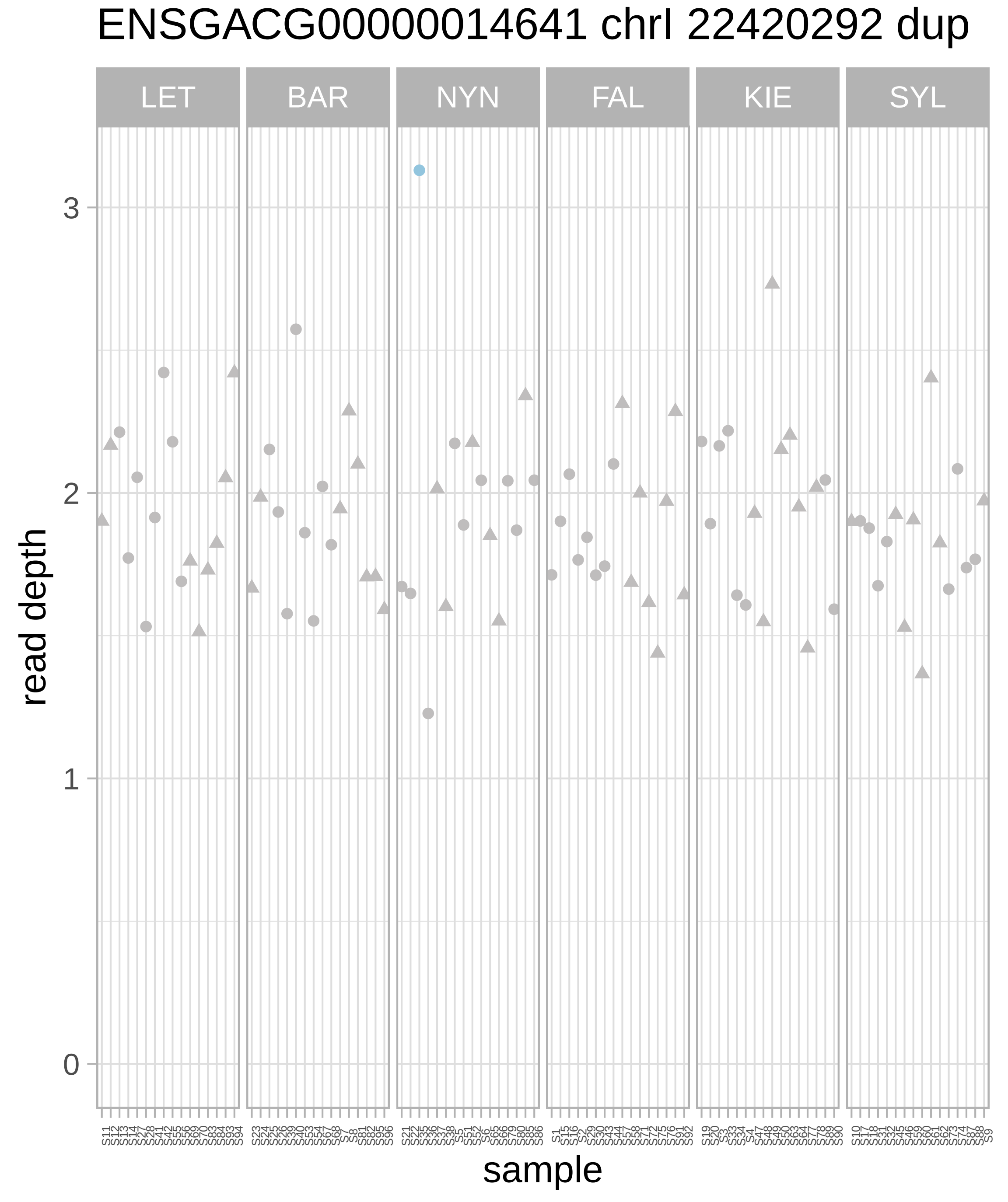
ENSGACG00000014641 chrI 22420292 dup
LET
BAR
NYN
FAL
KIE
SYL
read depth
0
1
2
3
sample

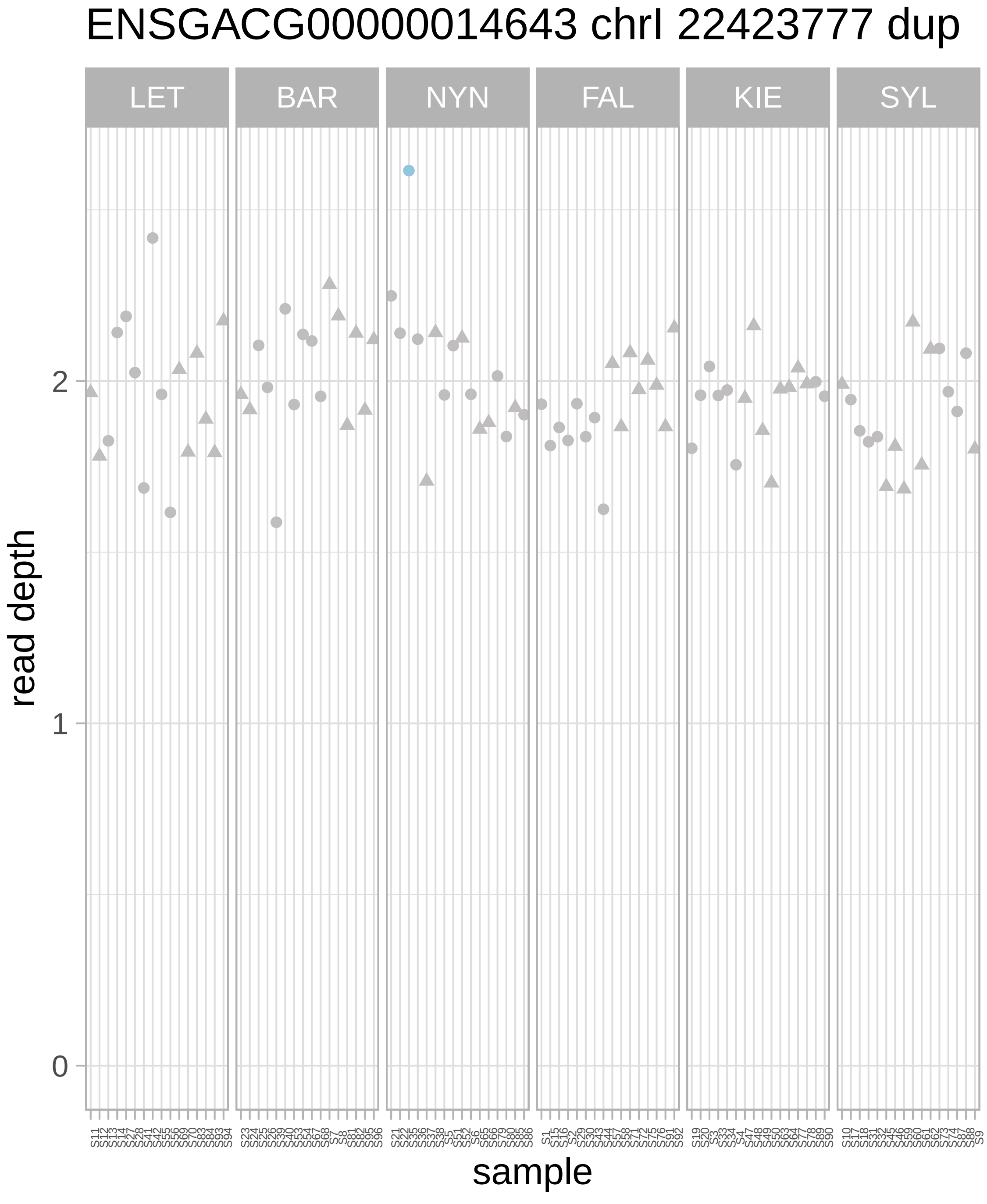

ENSGACG00000014643 chrI 22423777 dup
LET
BAR
NYN
FAL
KIE
SYL
read depth
2
1
0
sample

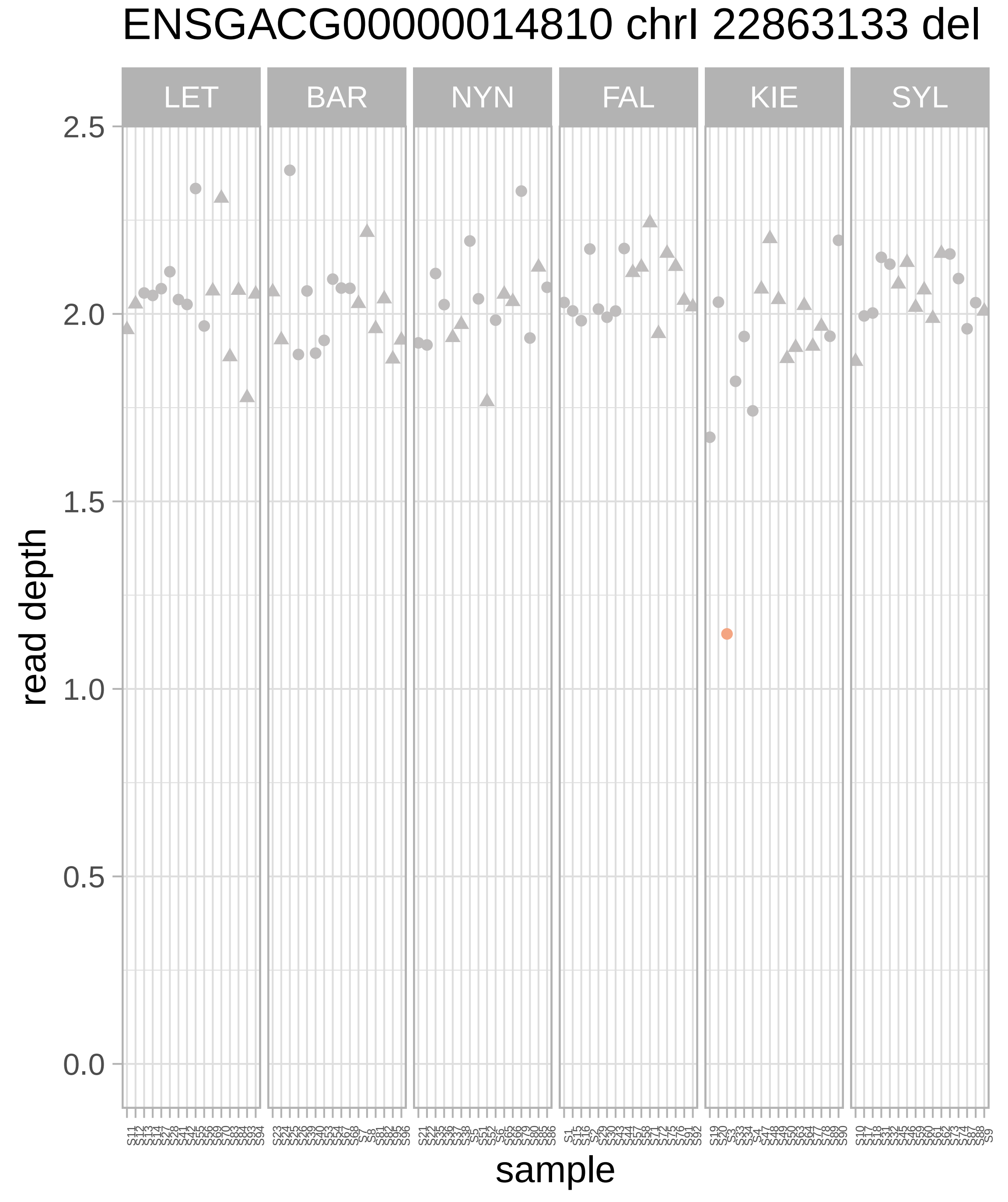
ENSGACG00000014810 chrI 22863133 del
LET
BAR
NYN
FAL
KIE
SYL
2.5
2.0
1.5
1.0
0.5
0.0
read depth
sample

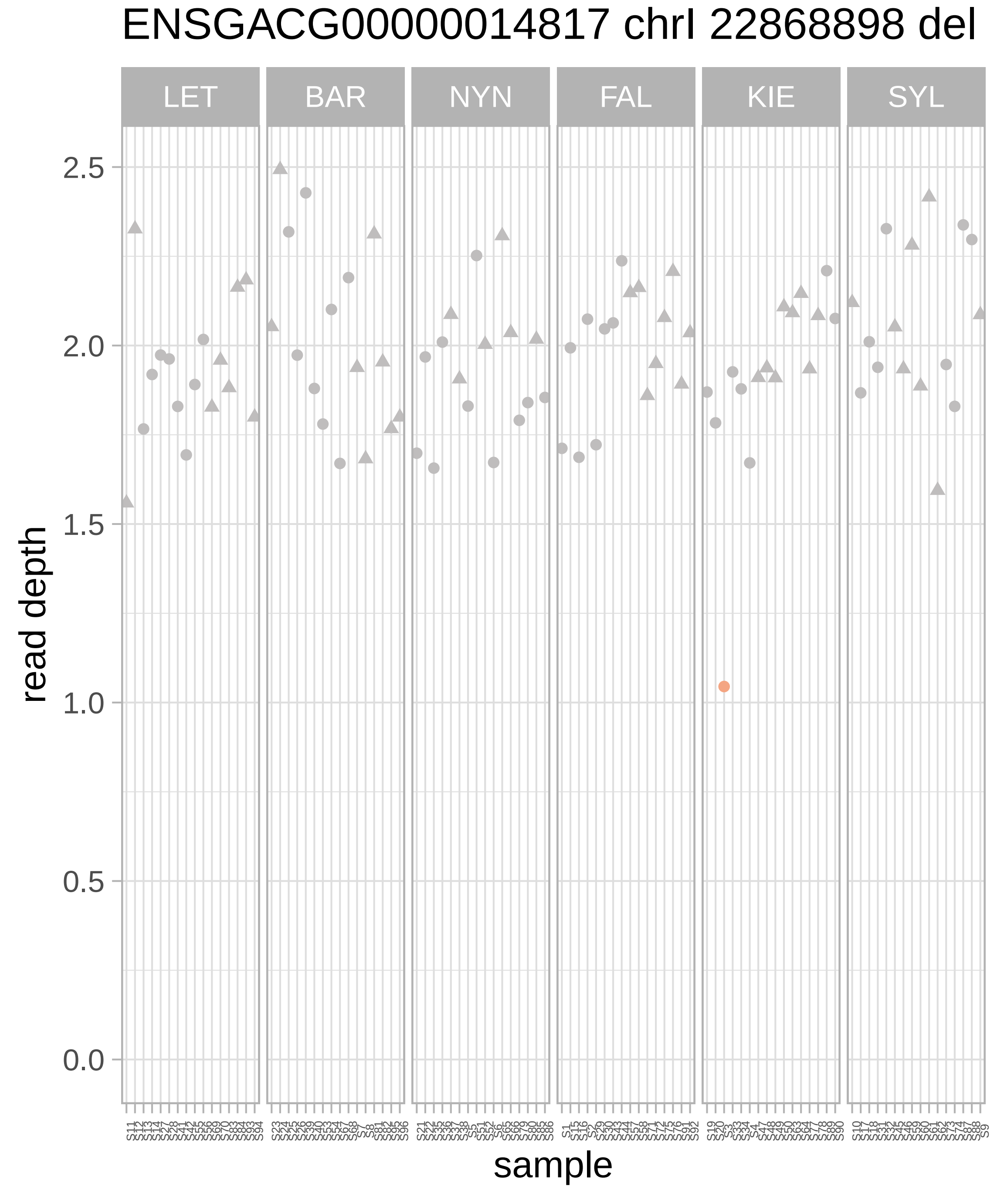
ENSGACG00000014817 chrI 22868898 del
LET
BAR
NYN
FAL
KIE
SYL
read depth
2.5
2.0
1.5
1.0
0.5
0.0
sample

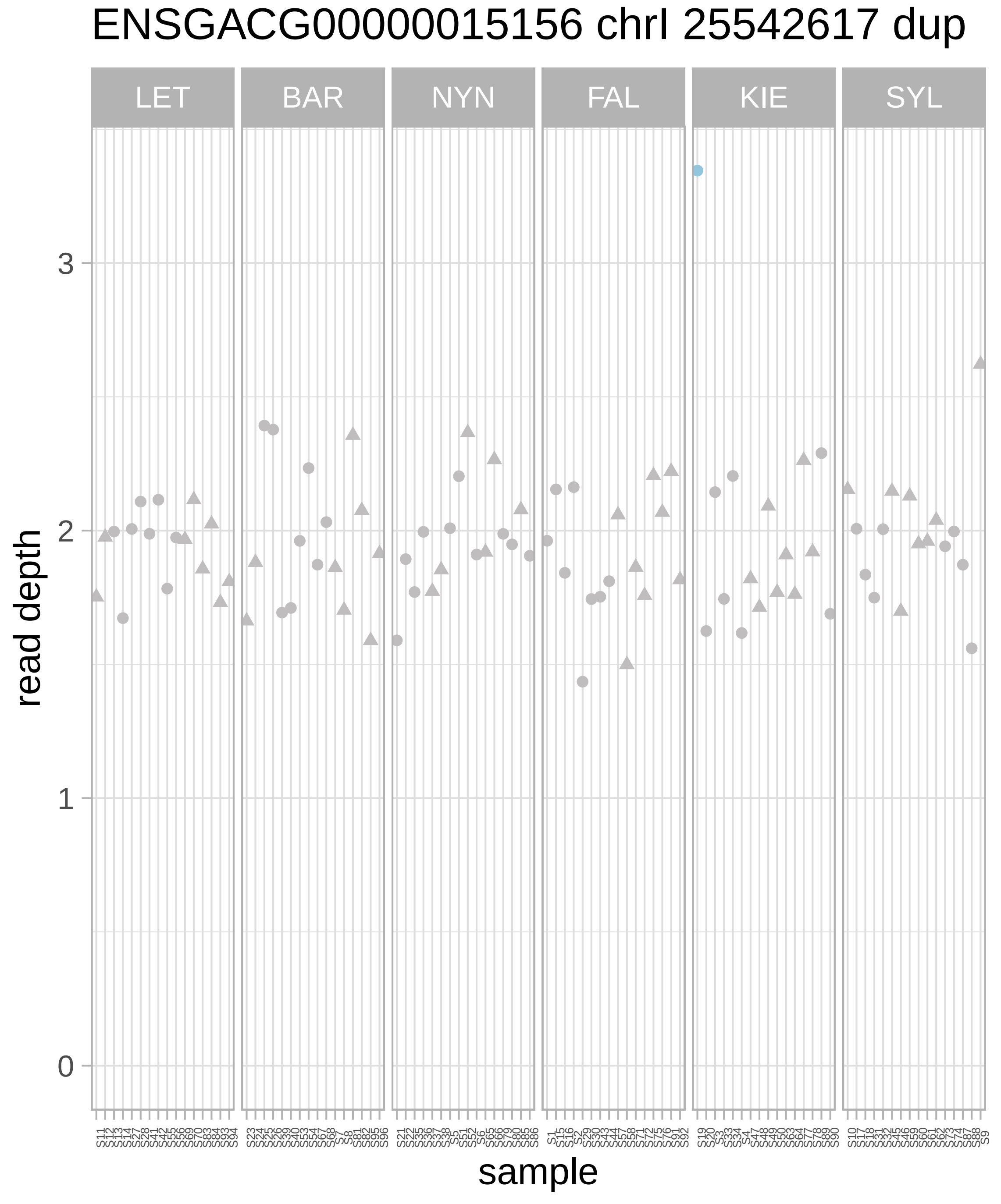
ENSGACG00000015156 chrI 25542617 dup
LET
BAR
NYN
FAL
KIE
SYL
read depth
0
1
2
3
sample

ENSGACG00000015159 chrI 25544366 dup

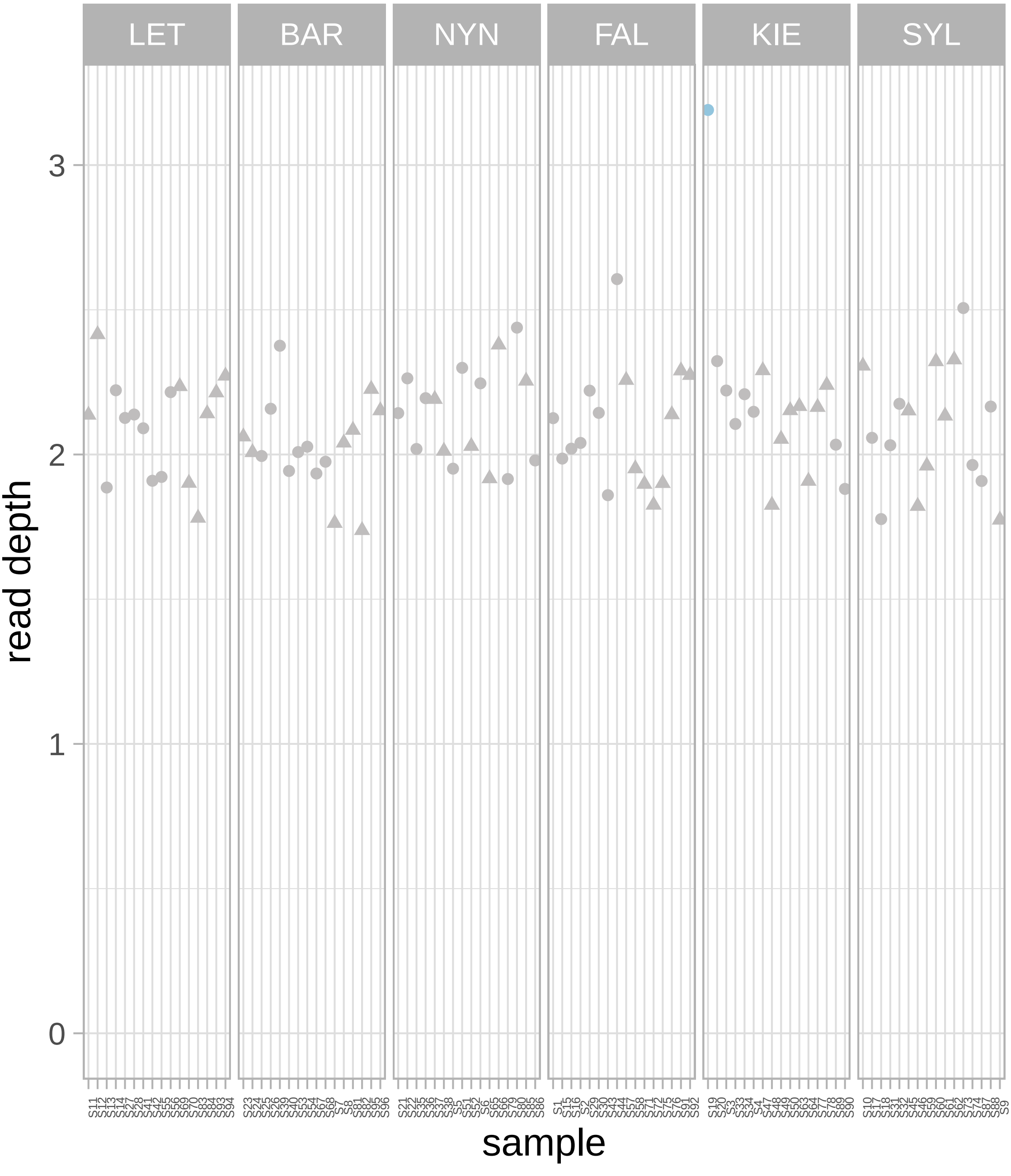

ENSGACG00000015162 chrI 25545112 dup

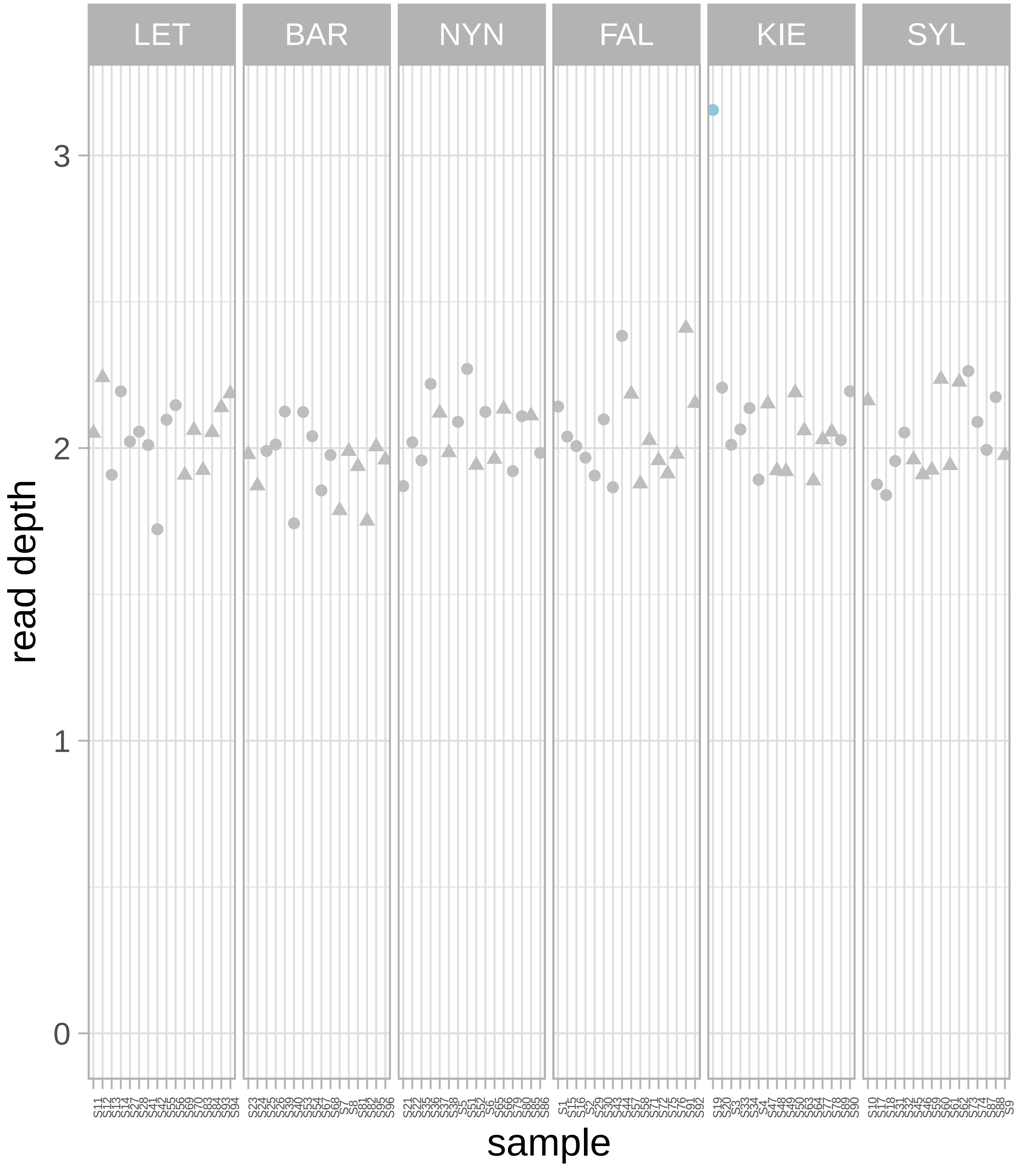

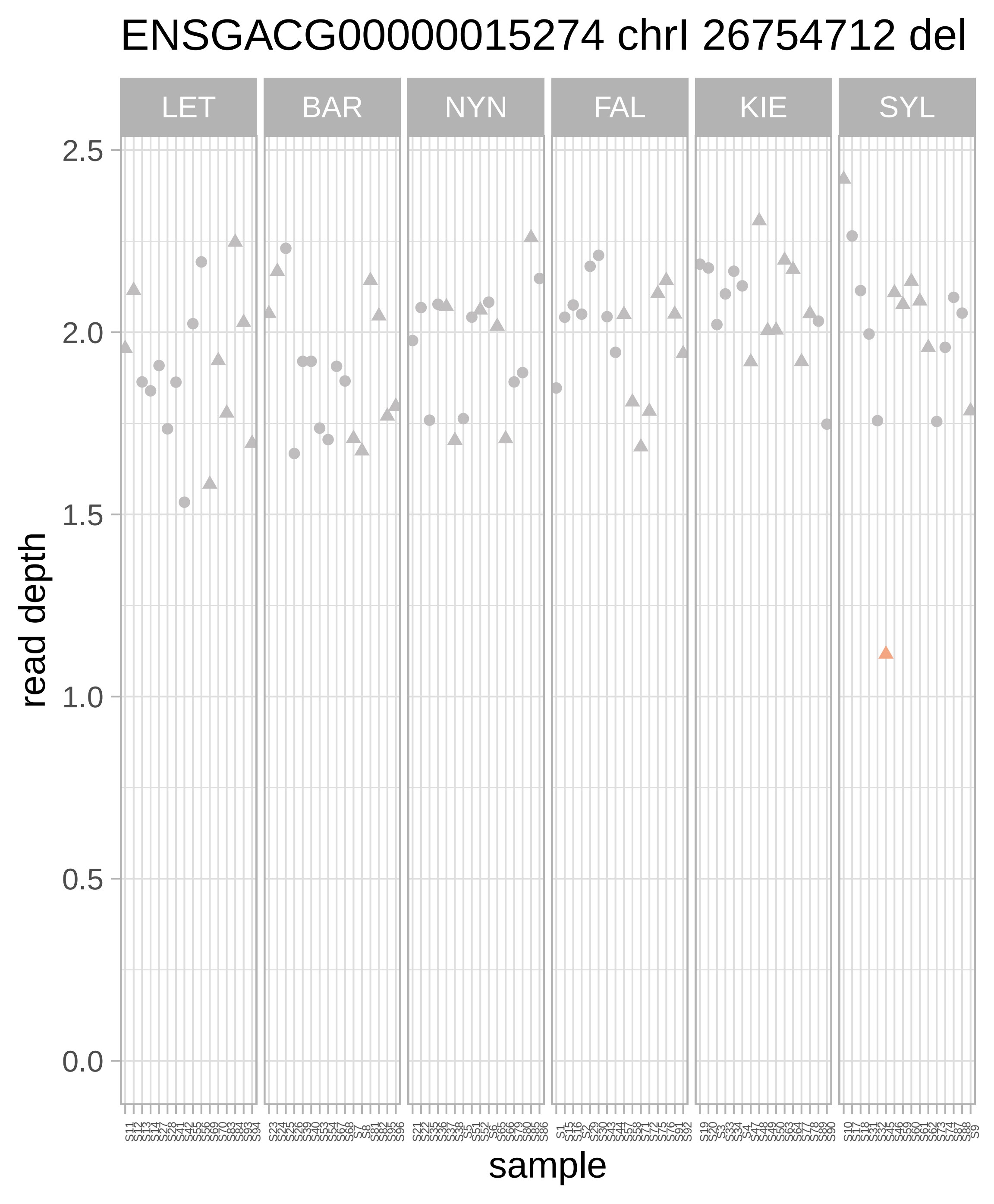
ENSGACG00000015274 chrI 26754712 del
LET
BAR
NYN
FAL
KIE
SYL
read depth
2.5
2.0
1.5
1.0
0.5
0.0
sample

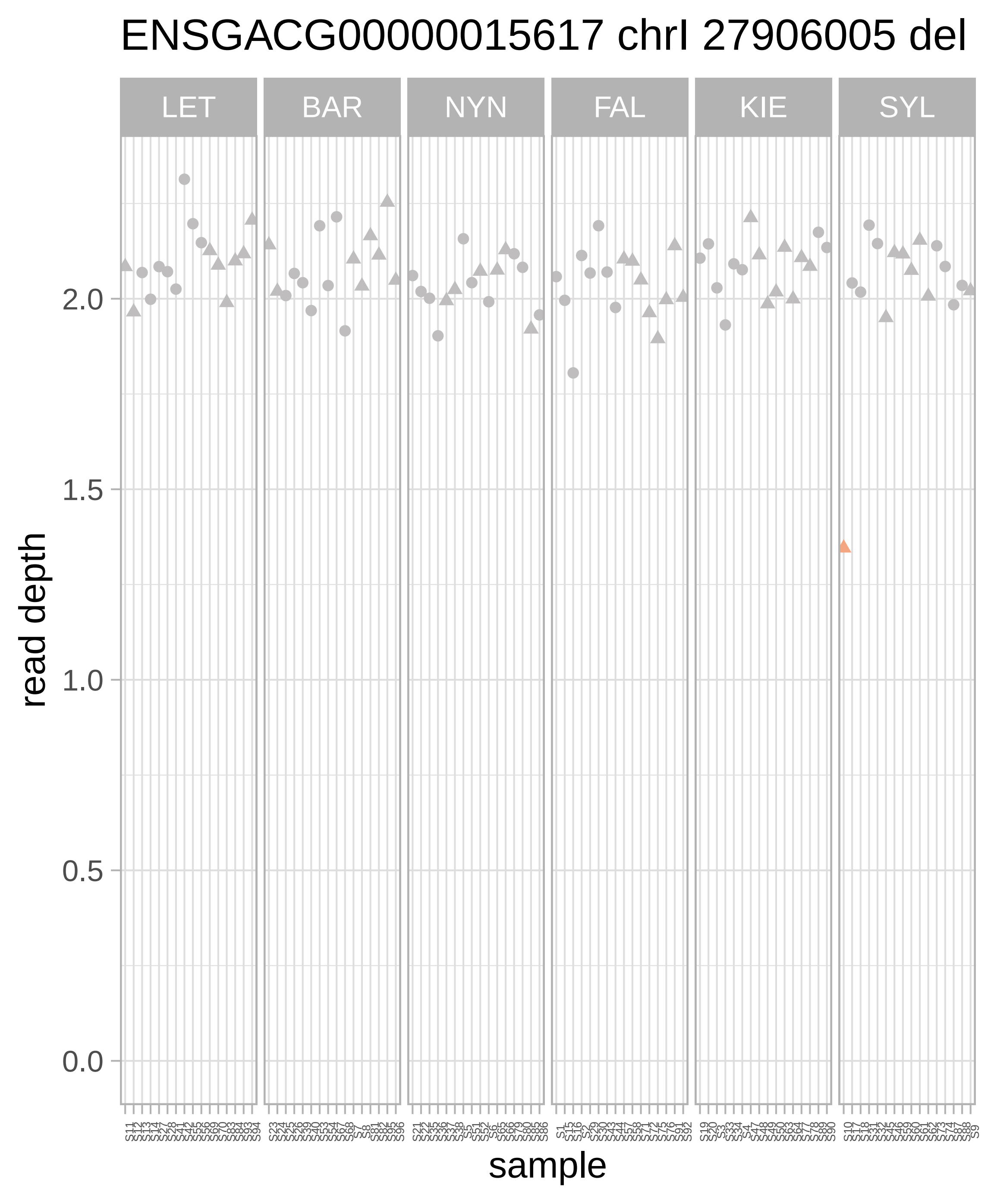
ENSGACG00000015617 chrI 27906005 del
LET
BAR
NYN
FAL
KIE
SYL
read depth
2.0
1.5
1.0
0.5
0.0
sample

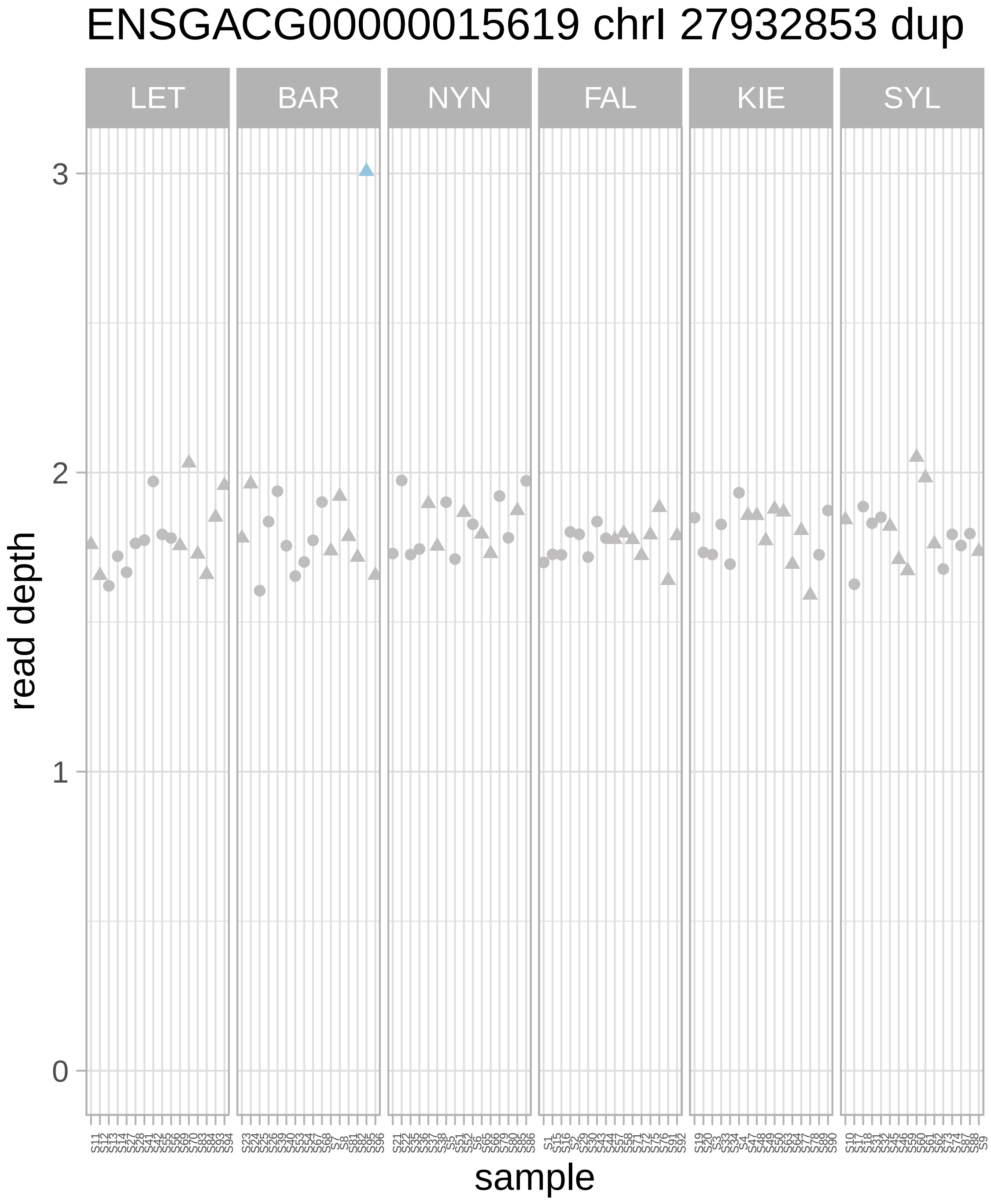
ENSGACG00000015619 chrI 27932853 dup
LET
BAR
NYN
FAL
KIE
SYL
read depth
3
2
1
0
sample

ENSGACG00000015622 chrI 27949094 dup

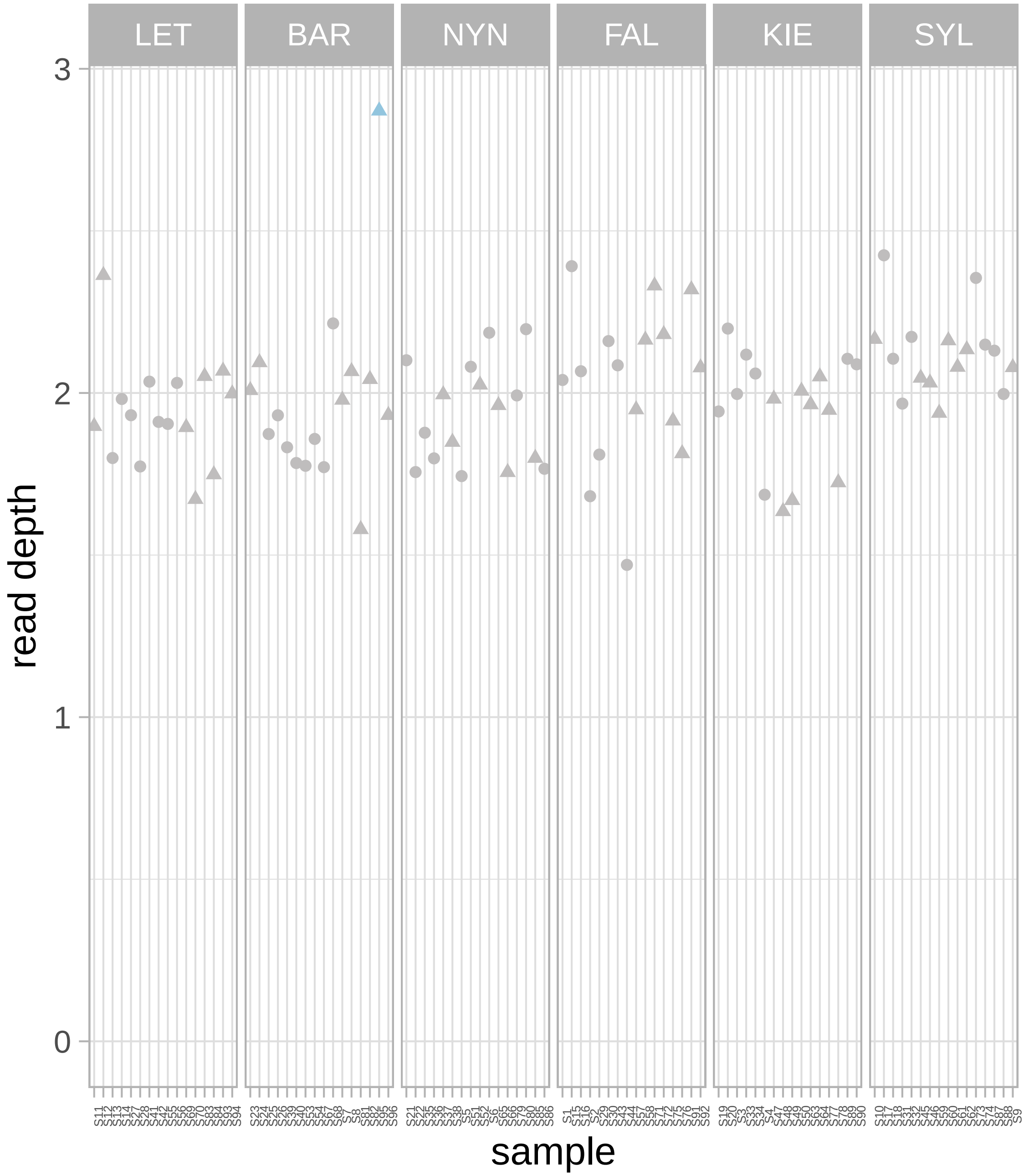

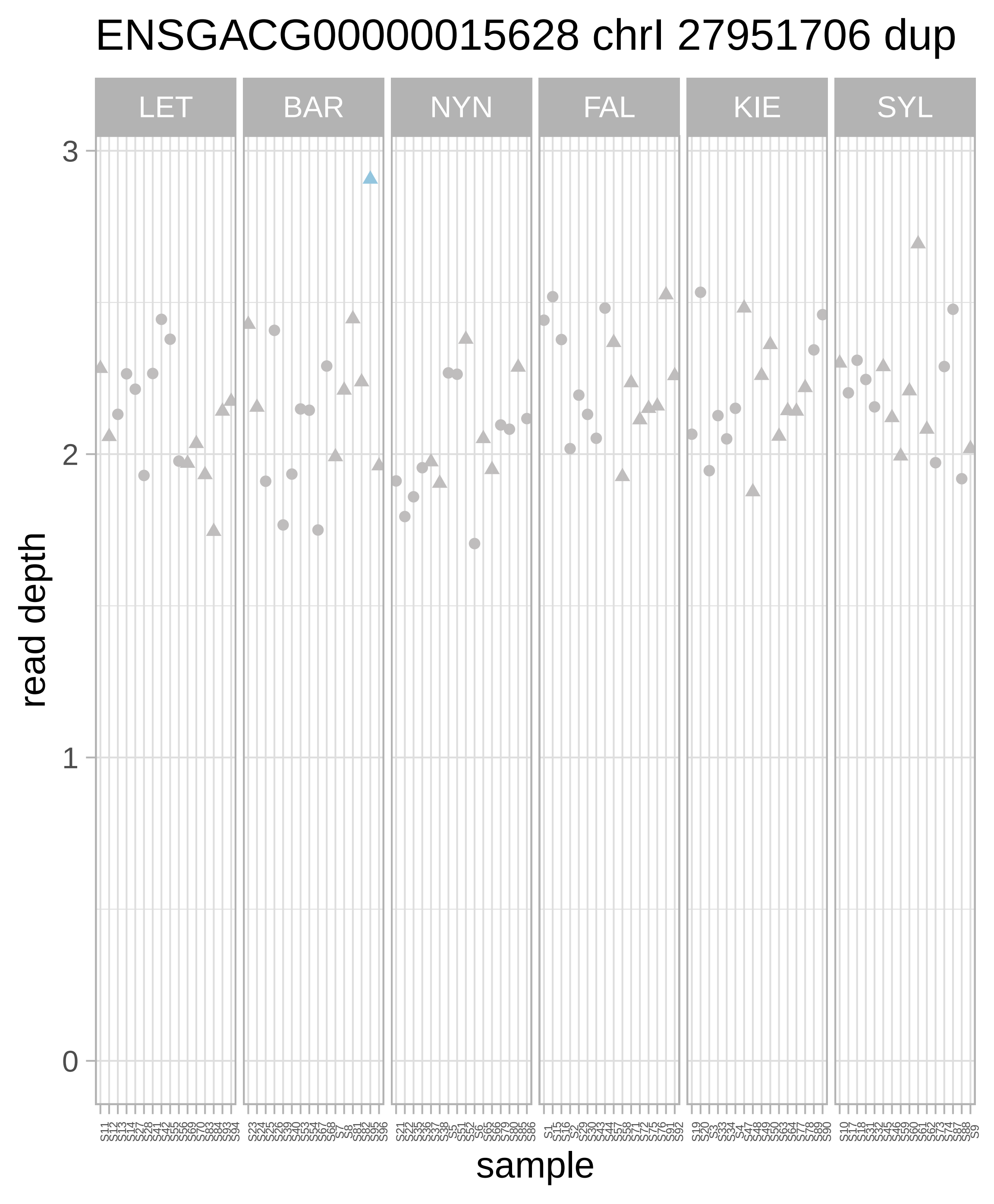
ENSGACG00000015628 chrI 27951706 dup
LET
BAR
NYN
FAL
KIE
SYL
read depth
0
1
2
3
sample

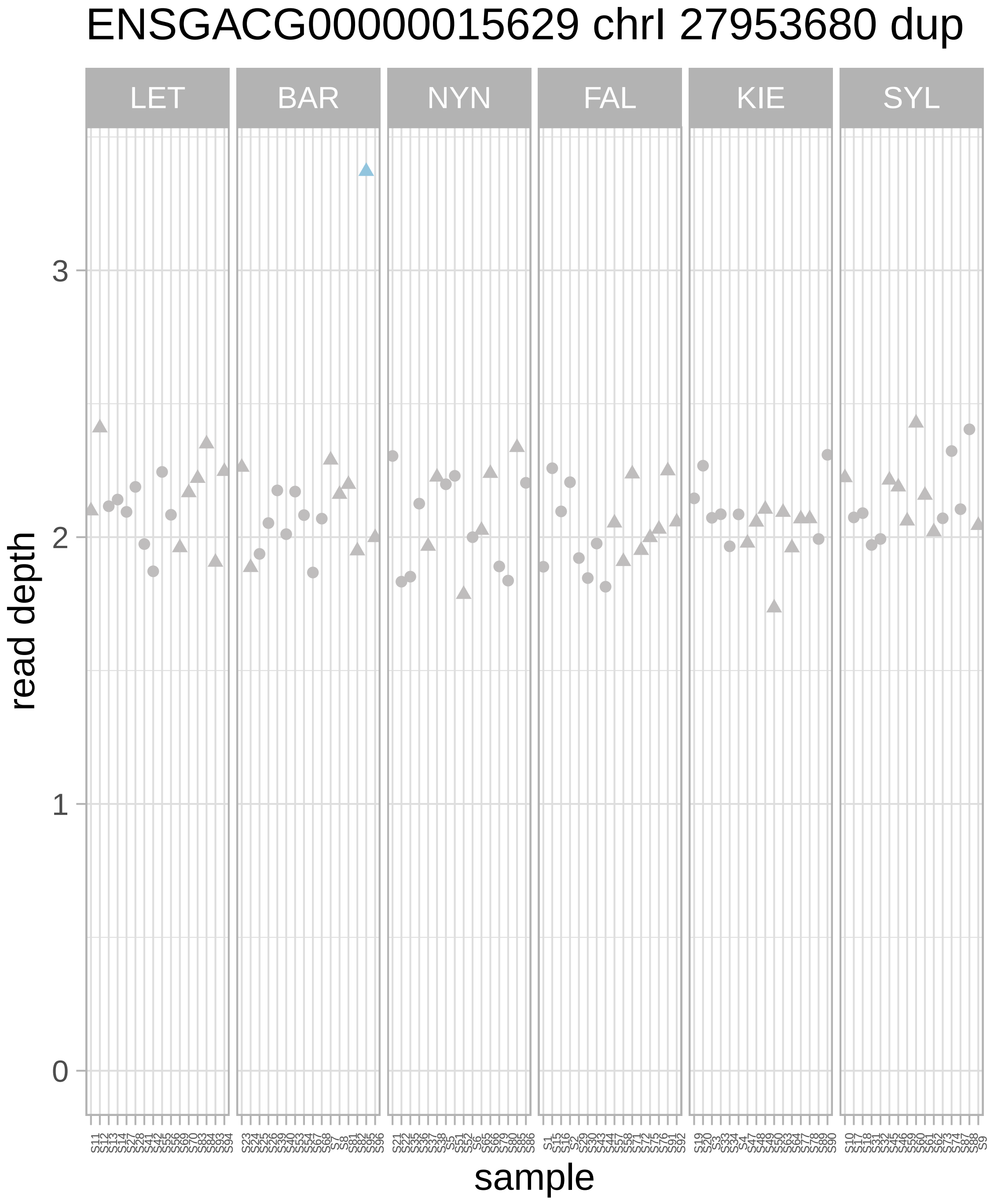
ENSGACG00000015629 chrI 27953680 dup
LET
BAR
NYN
FAL
KIE
SYL
read depth
0
1
2
3
S11 S12 S13 S14 S27 S28 S41 S42 S55 S56 S69 S70 S83 S84 S93 S94
S23 S24 S25 S26 S39 S40 S53 S54 S67 S68 S7 S8 S81 S82 S95 S96
S21 S22 S35 S36 S37 S38 S5 S51 S52 S6 S65 S66 S79 S80 S85 S86
S1 S15 S16 S2 S29 S30 S43 S44 S57 S58 S71 S72 S75 S76 S91 S92
S19 S20 S3 S33 S34 S4 S47 S48 S49 S50 S63 S64 S77 S78 S89 S90
S10 S17 S18 S31 S32 S45 S46 S59 S60 S61 S62 S73 S74 S87 S88 S9
sample

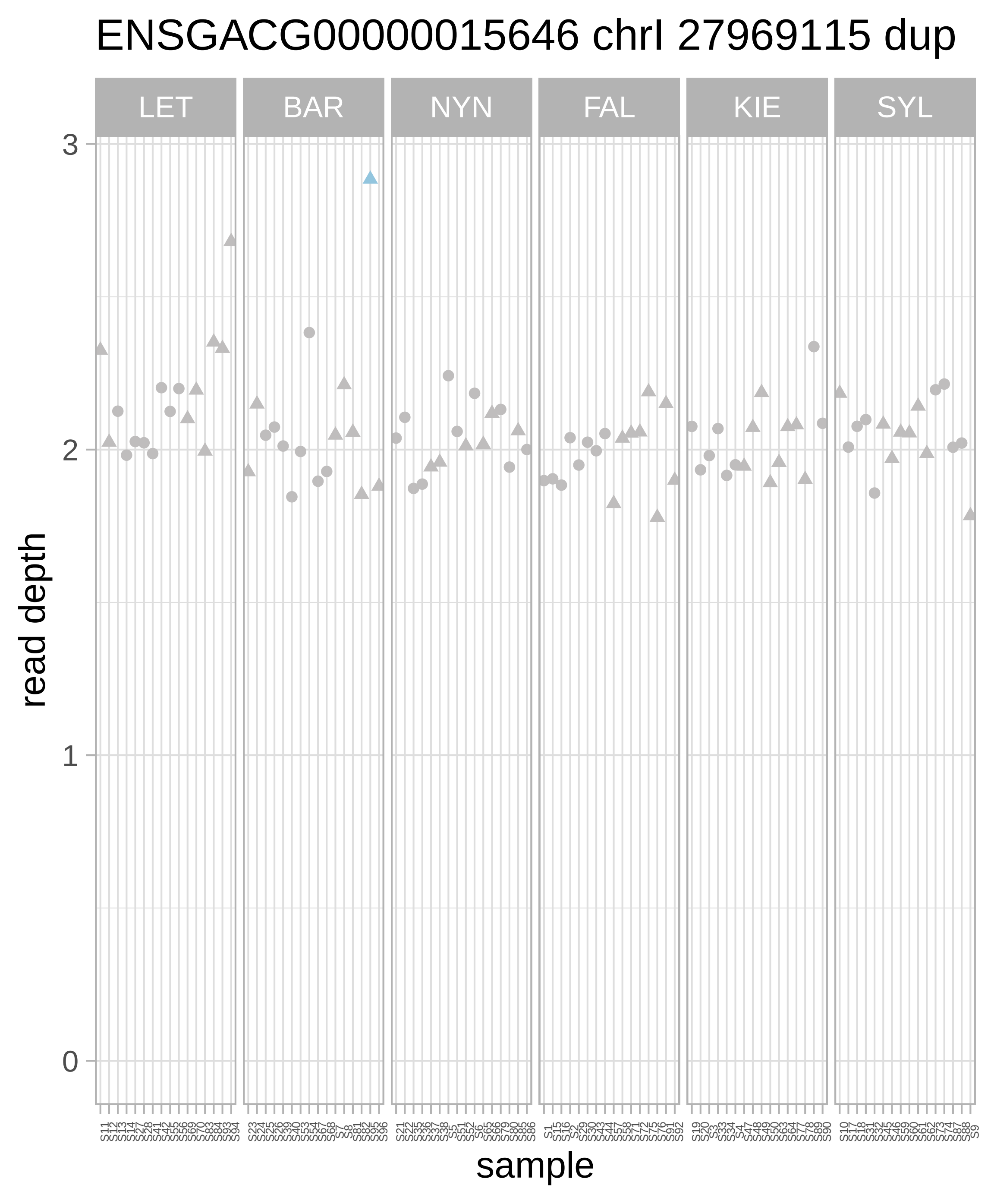
ENSGACG00000015646 chrI 27969115 dup
LET
BAR
NYN
FAL
KIE
SYL
read depth
0
1
2
3
sample

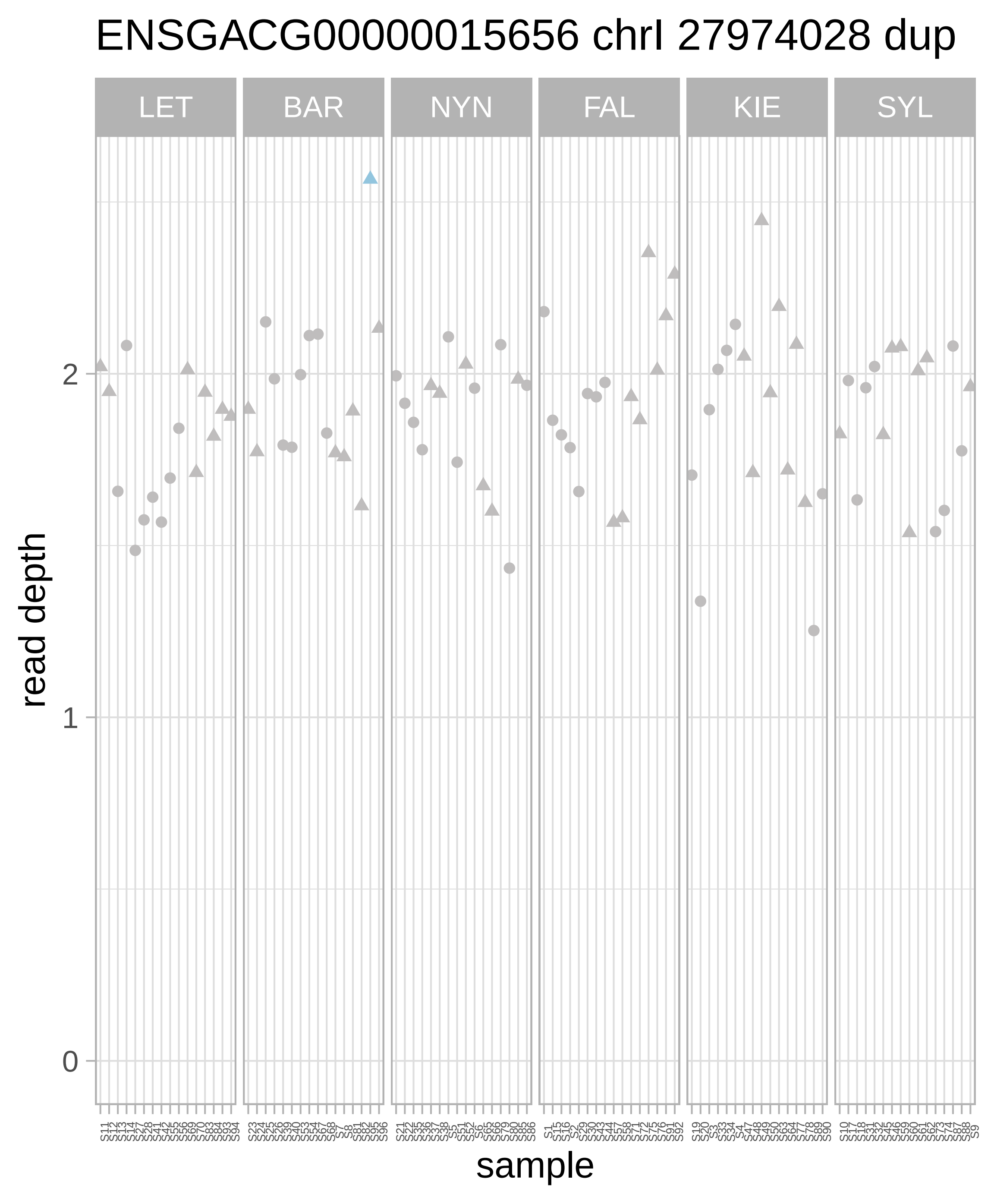
ENSGACG00000015656 chrI 27974028 dup
LET
BAR
NYN
FAL
KIE
SYL
read depth
2
1
0
sample

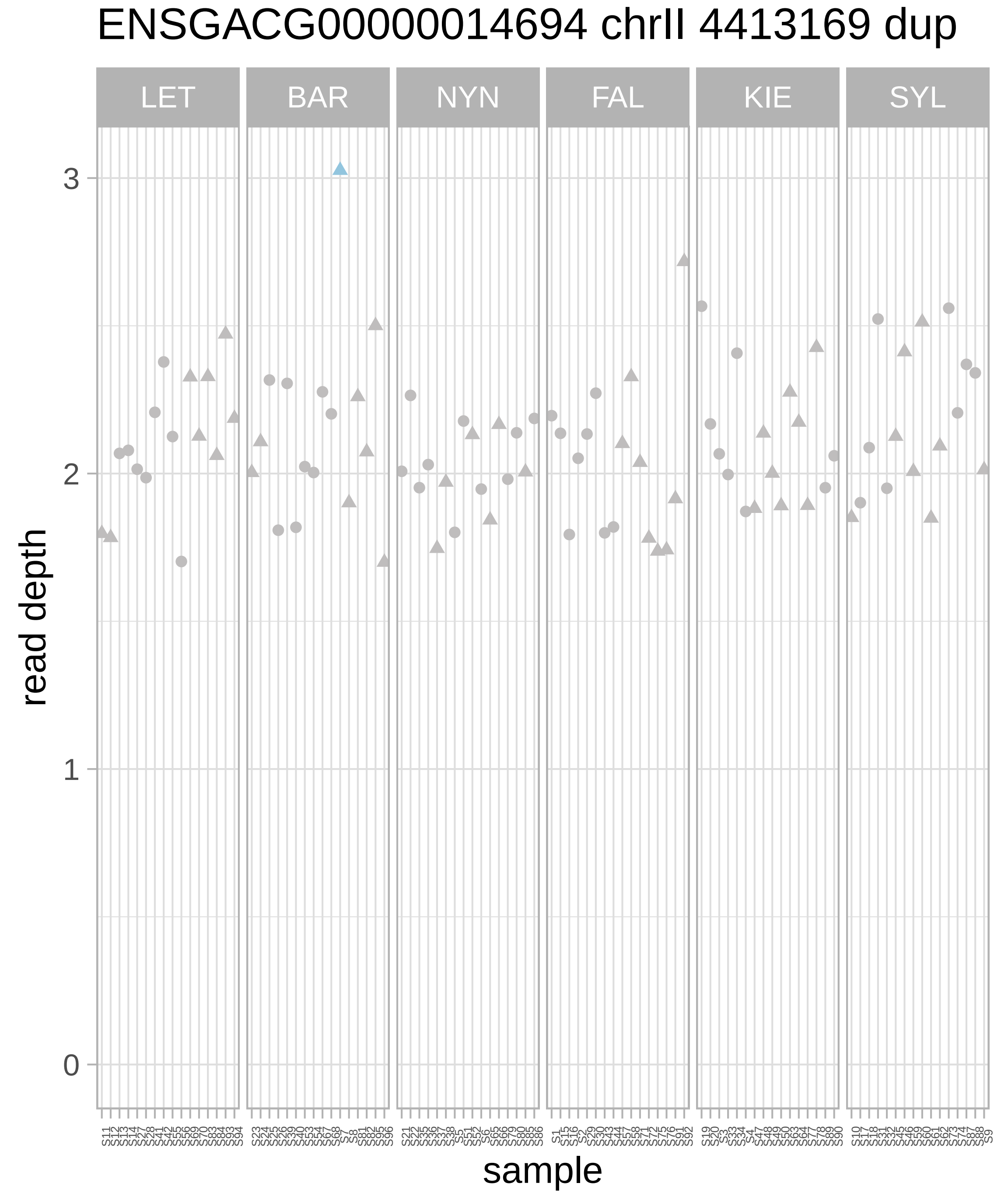
ENSGACG00000014694 chrII 4413169 dup
LET
BAR
NYN
FAL
KIE
SYL
read depth
0
1
2
3
sample

ENSGACG00000014815 chrII 5087410 dup

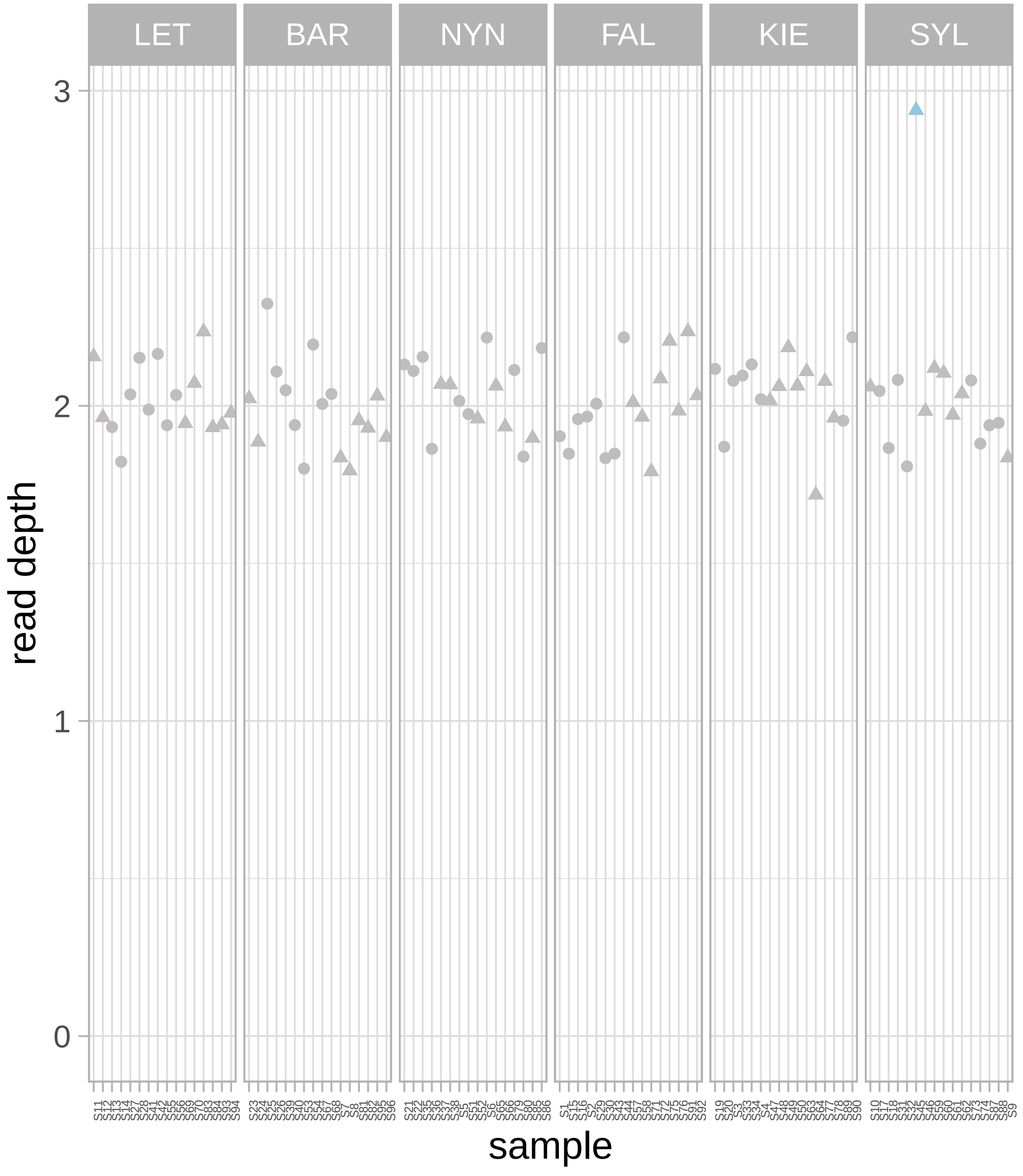

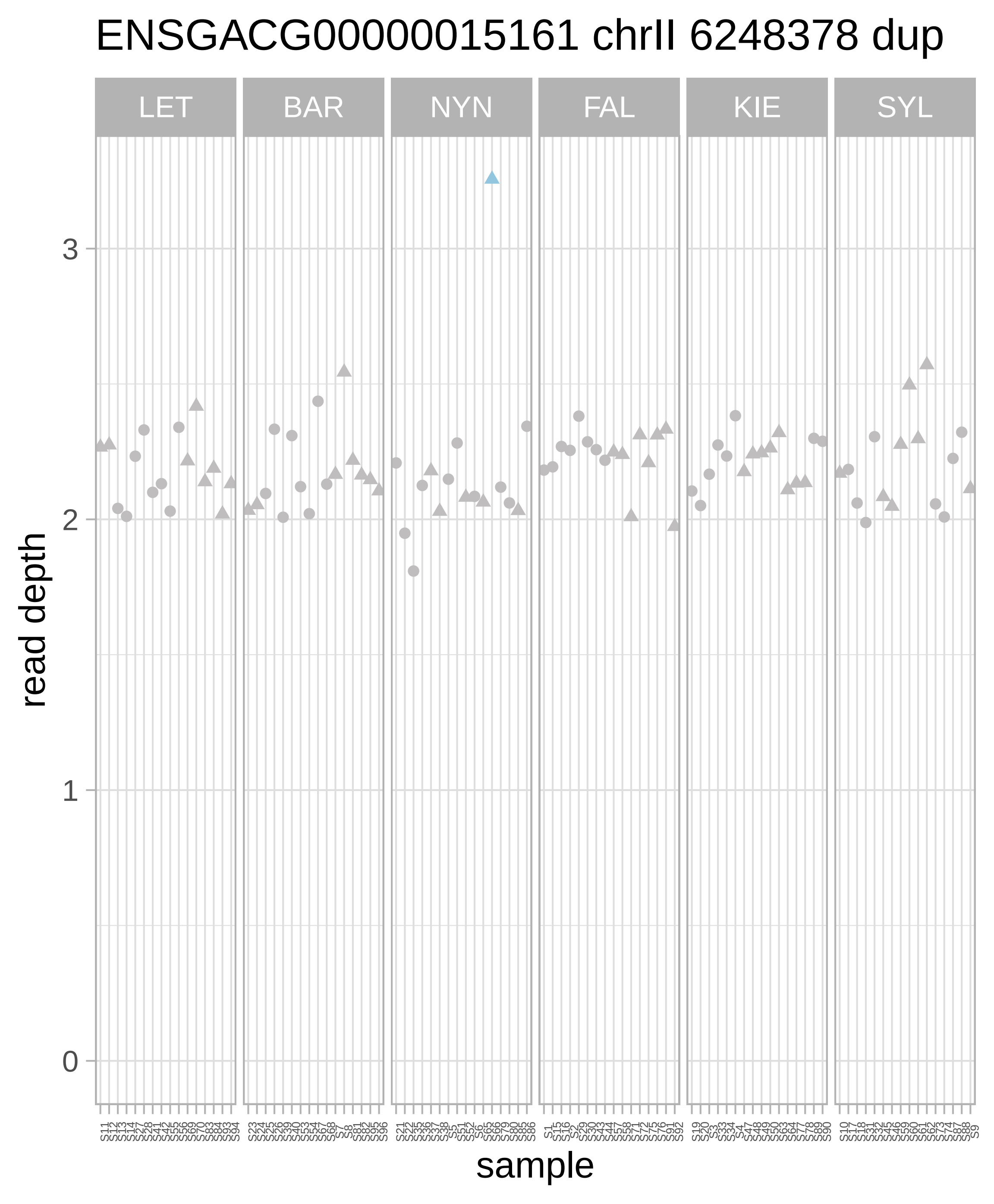
ENSGACG00000015161 chrII 6248378 dup
LET
BAR
NYN
FAL
KIE
SYL
3
2
1
0
read depth
sample

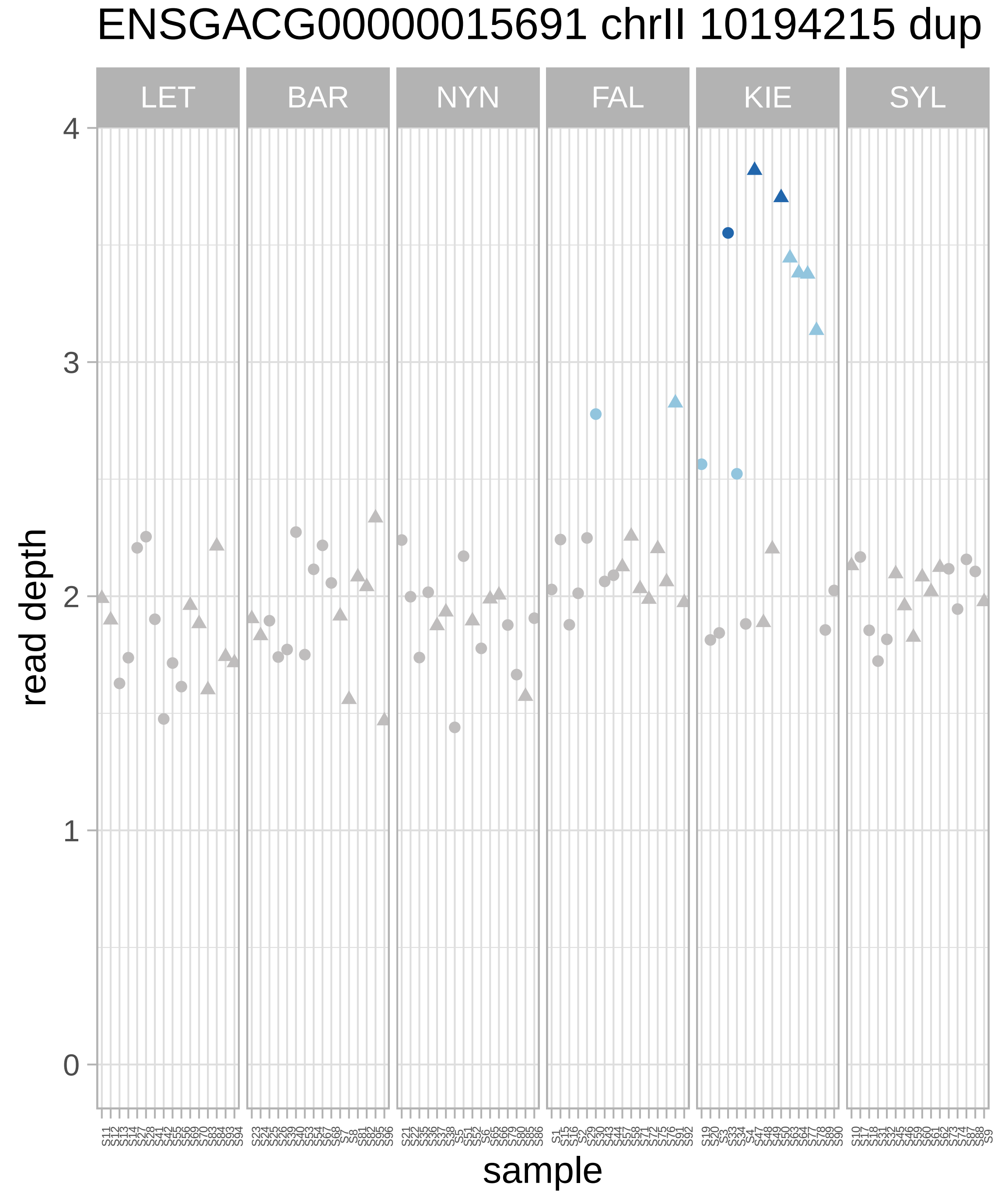
ENSGACG00000015691 chrII 10194215 dup
LET
BAR
NYN
FAL
KIE
SYL
read depth
sample
0
1
2
3
4

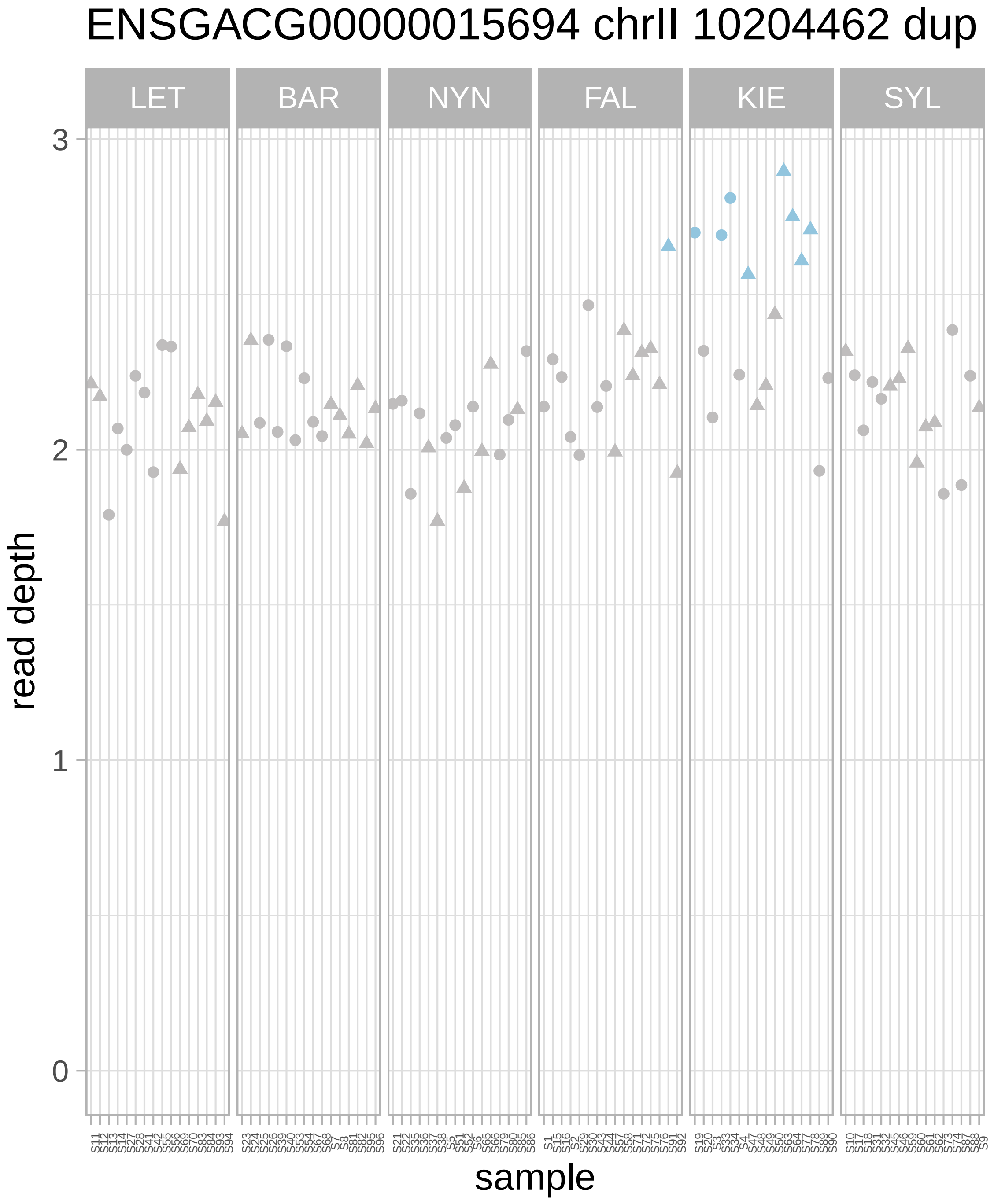
ENSGACG00000015694 chrII 10204462 dup
LET
BAR
NYN
FAL
KIE
SYL
read depth
0
1
2
3
sample

ENSGACG00000015725 chrII 10451835 dup

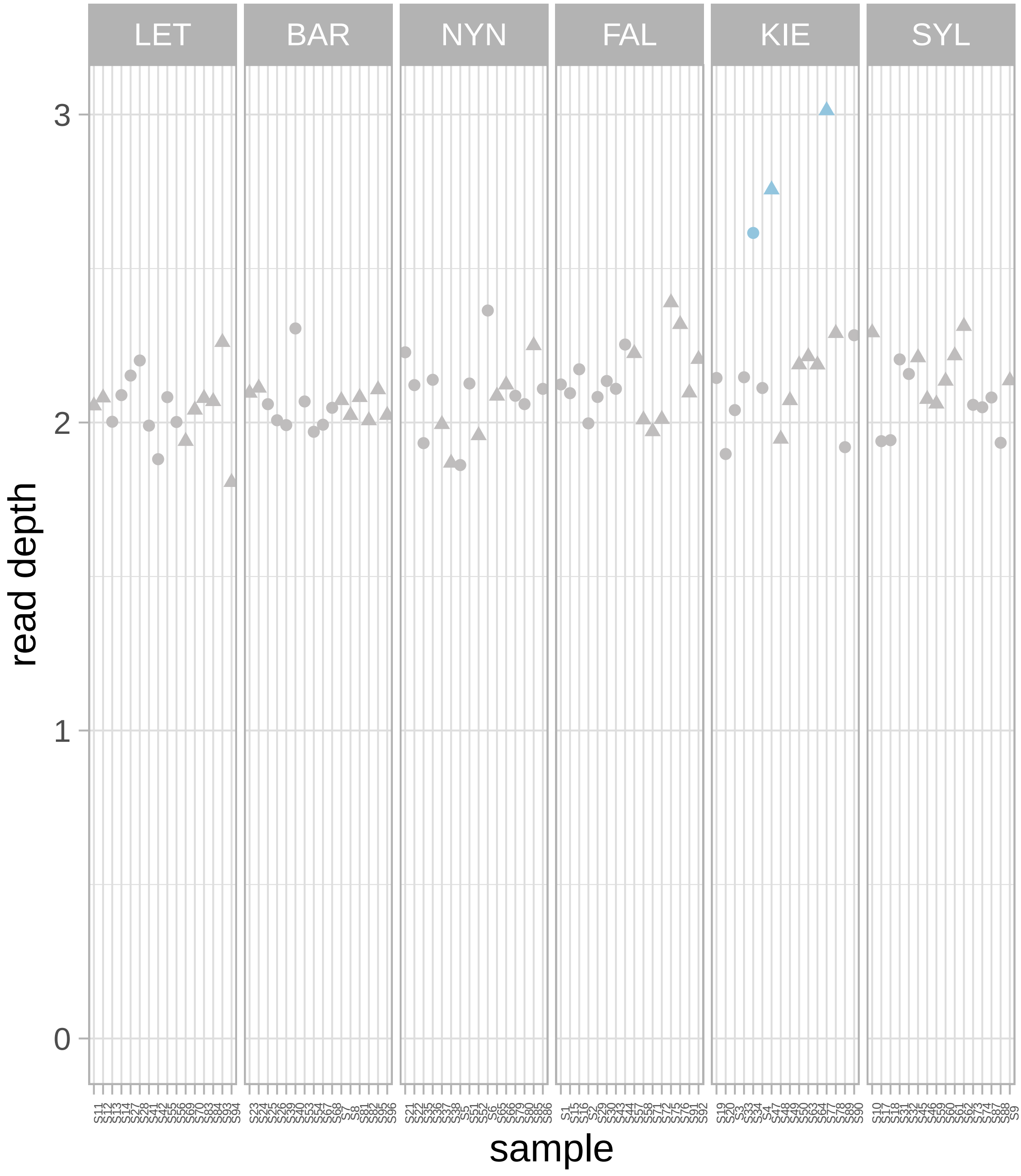

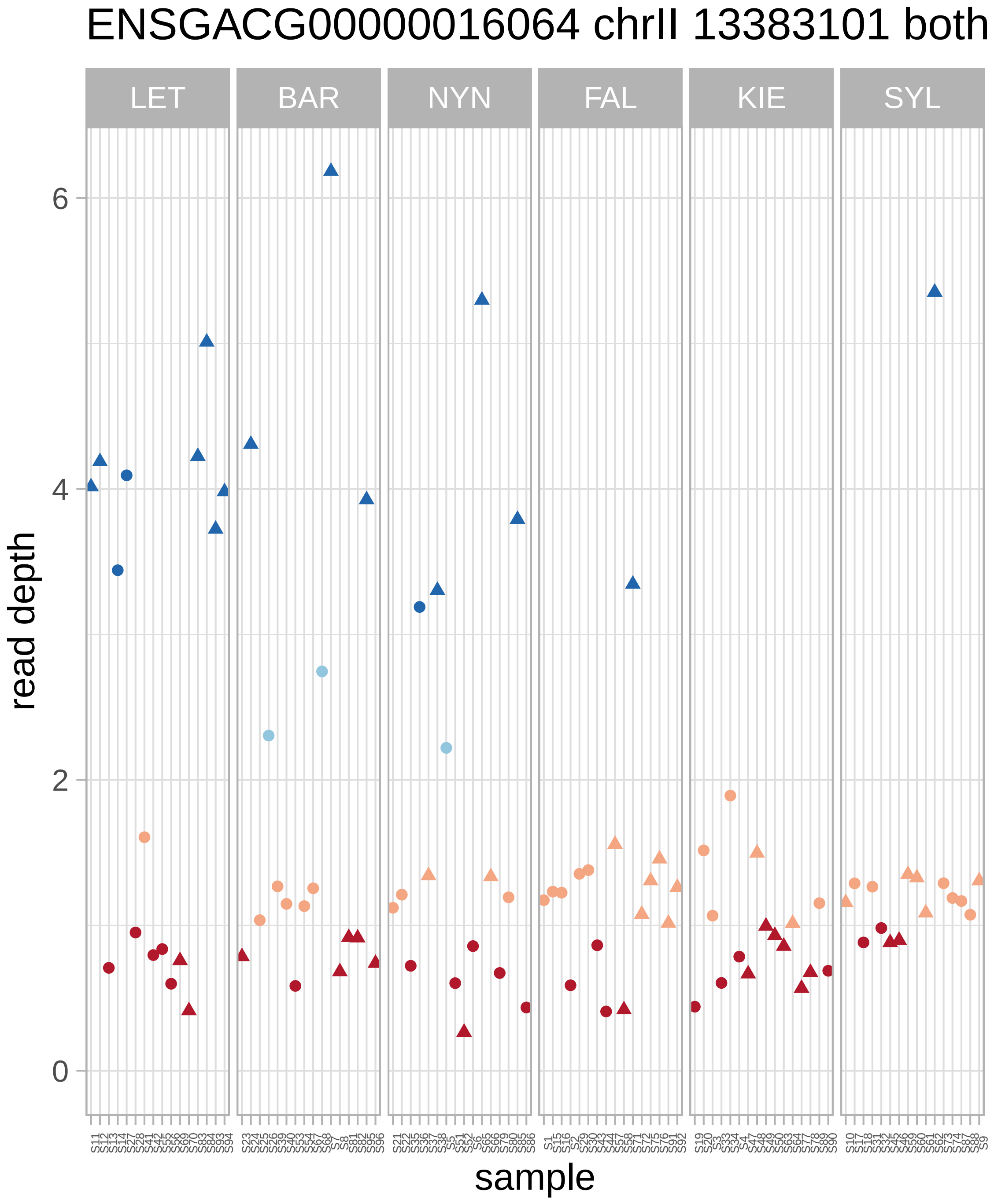
ENSGACG00000016064 chrII 13383101 both
LET
BAR
NYN
FAL
KIE
SYL
read depth
0
2
4
6
sample

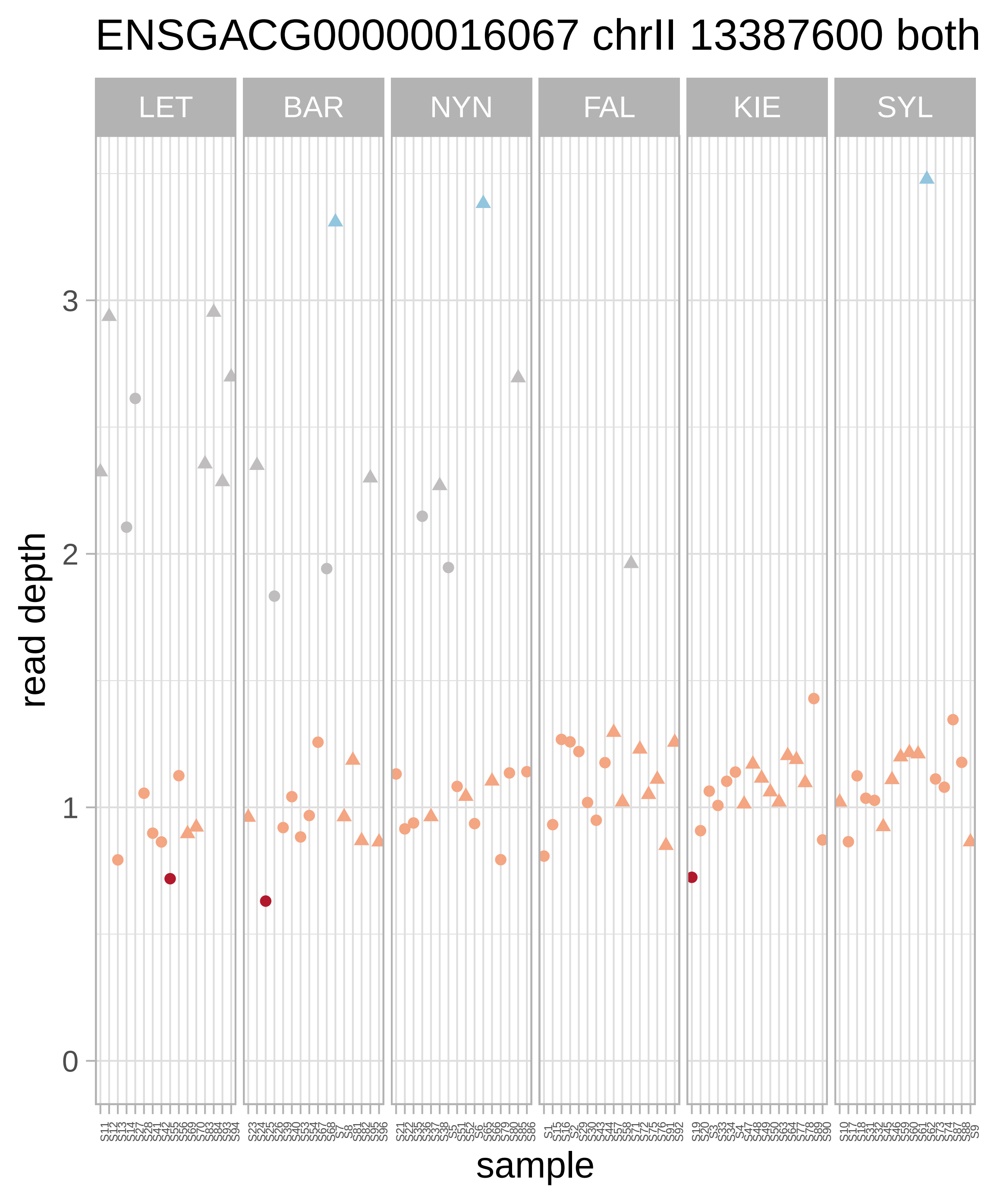
ENSGACG00000016067 chrII 13387600 both
LET
BAR
NYN
FAL
KIE
SYL
read depth
3
2
1
0
sample

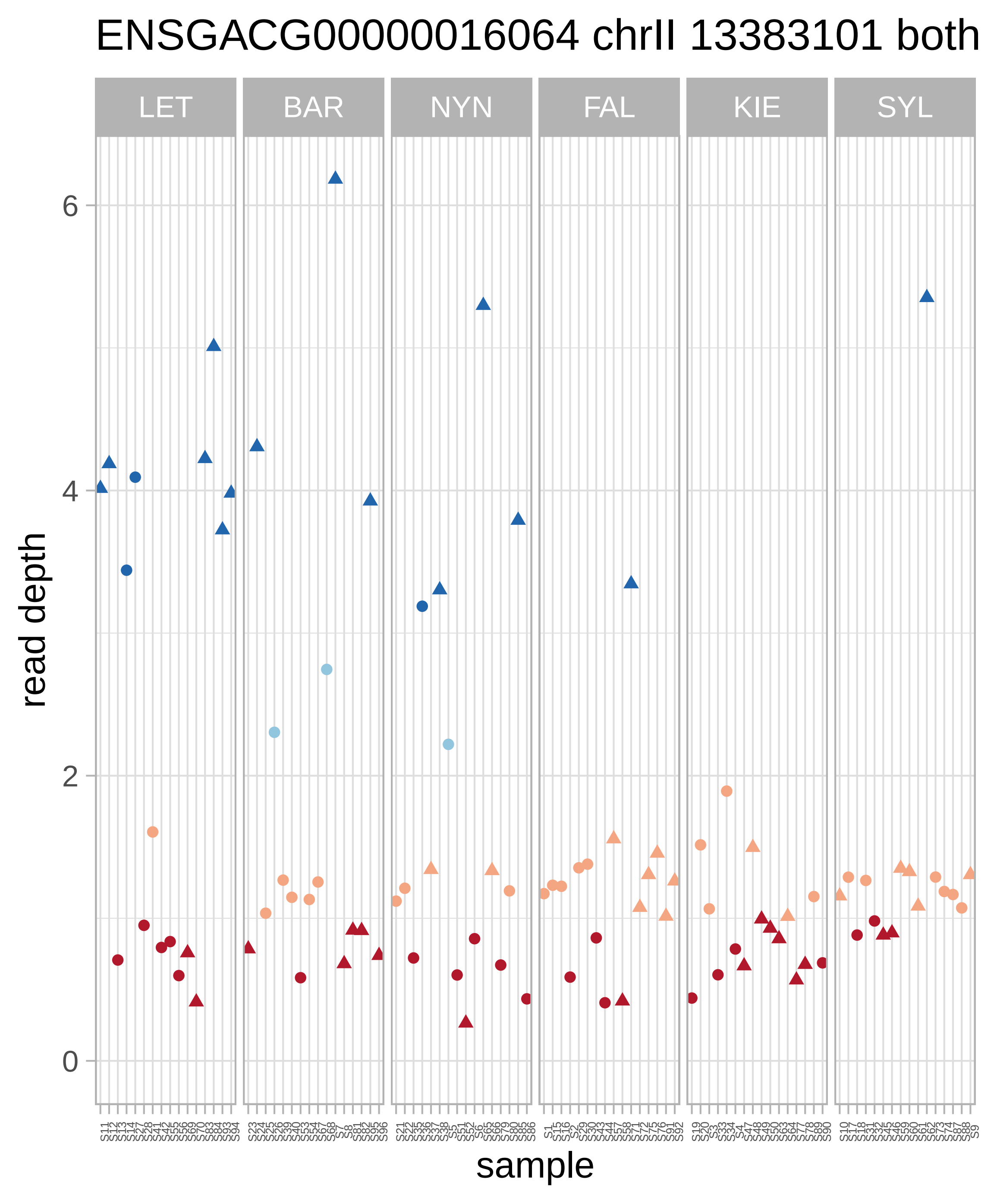
ENSGACG00000016064 chrII 13383101 both
LET
BAR
NYN
FAL
KIE
SYL
6
4
2
0
read depth
sample

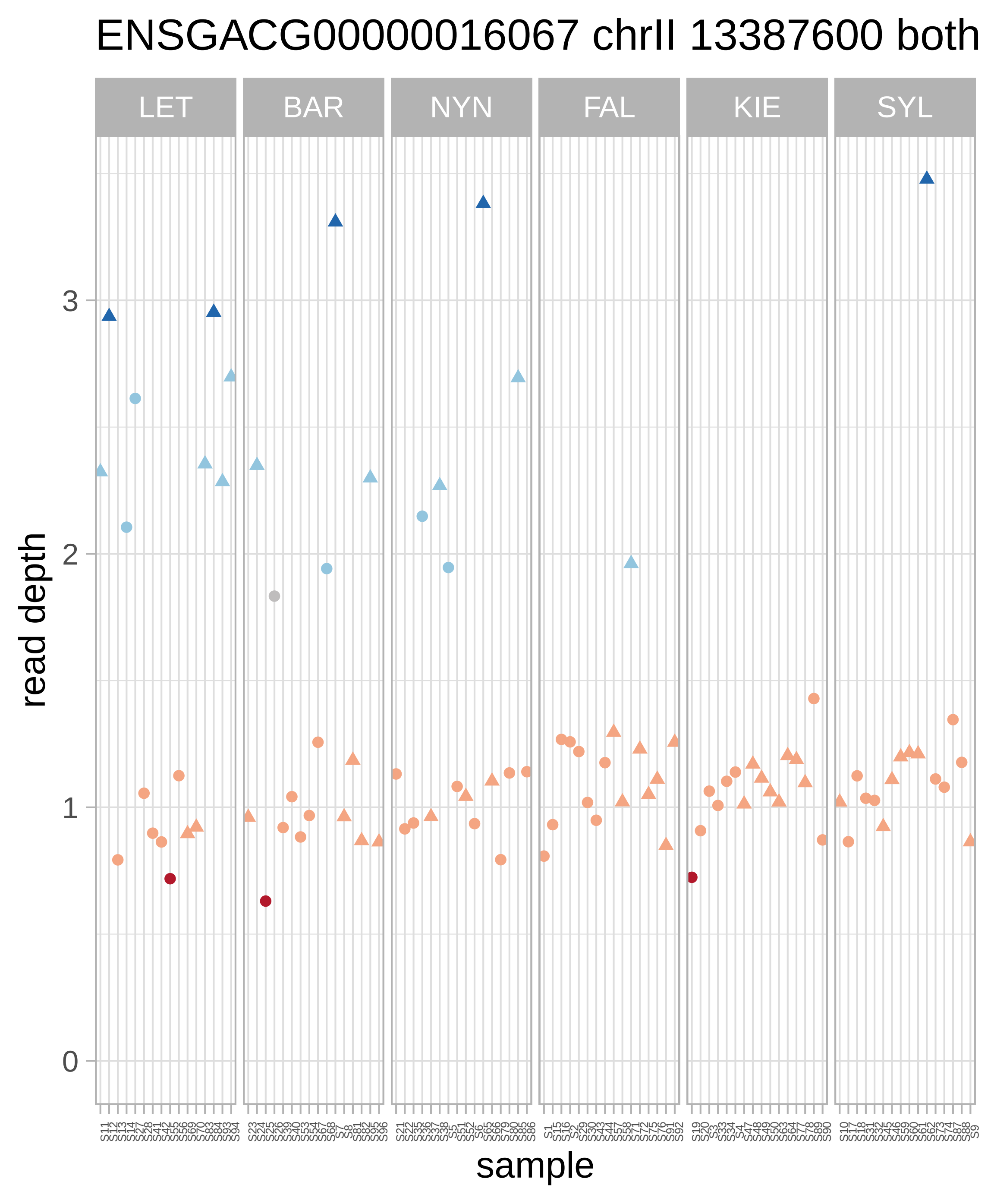
ENSGACG00000016067 chrII 13387600 both
LET
BAR
NYN
FAL
KIE
SYL
read depth
0
1
2
3
sample

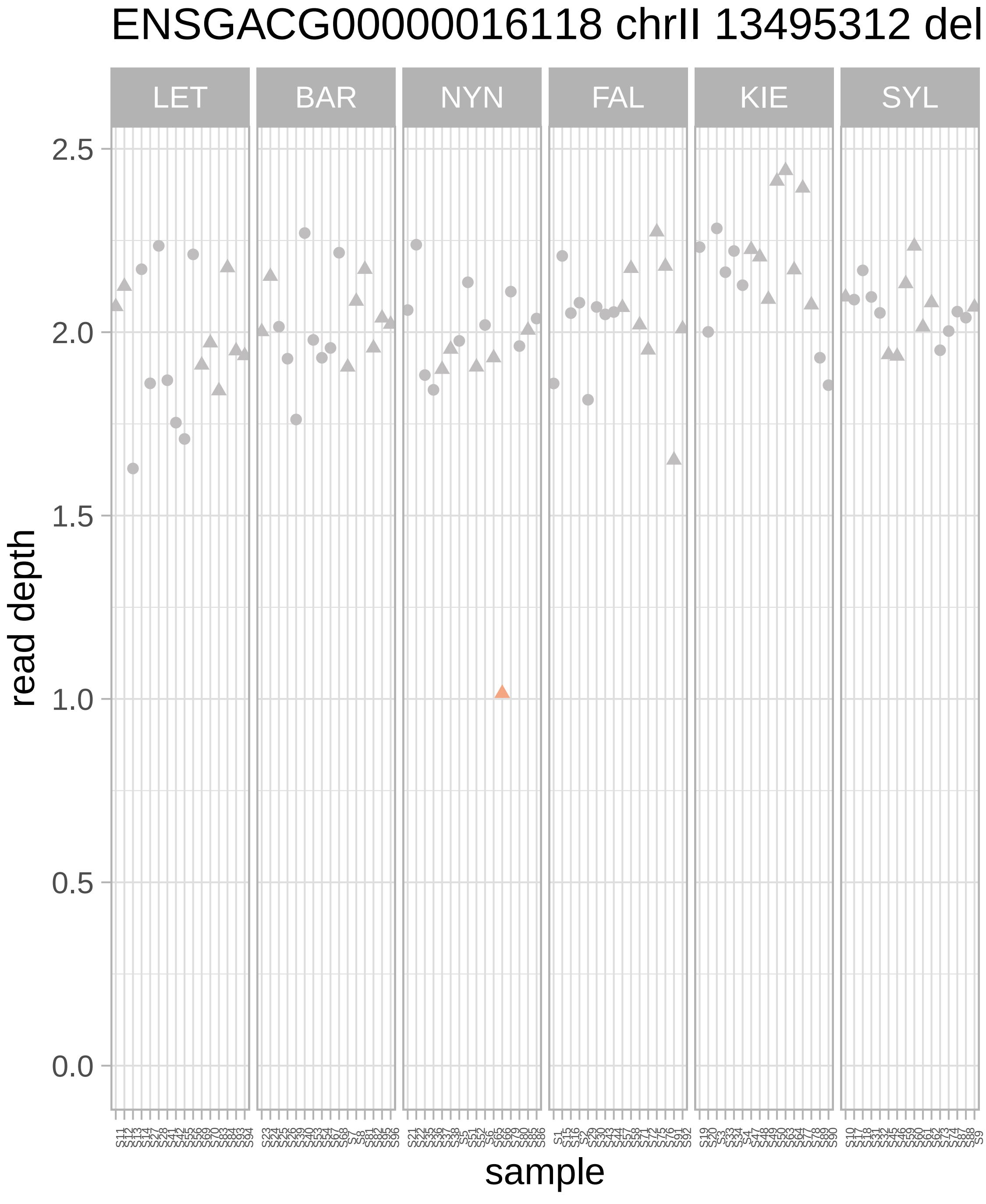
ENSGACG00000016118 chrII 13495312 del
LET
BAR
NYN
FAL
KIE
SYL
read depth
2.5
2.0
1.5
1.0
0.5
0.0
sample

ENSGACG00000016327 chrII 14627366 dup

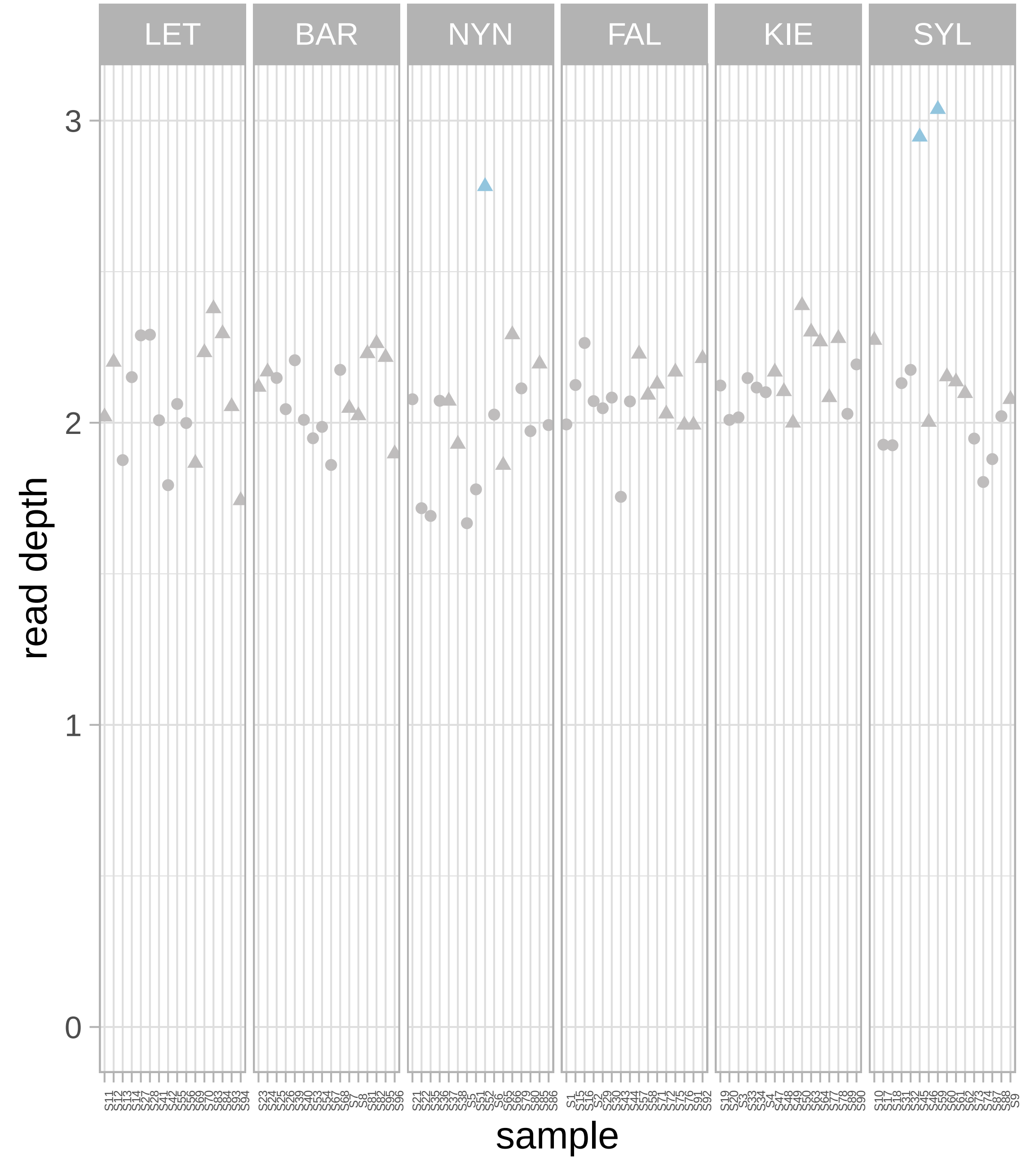

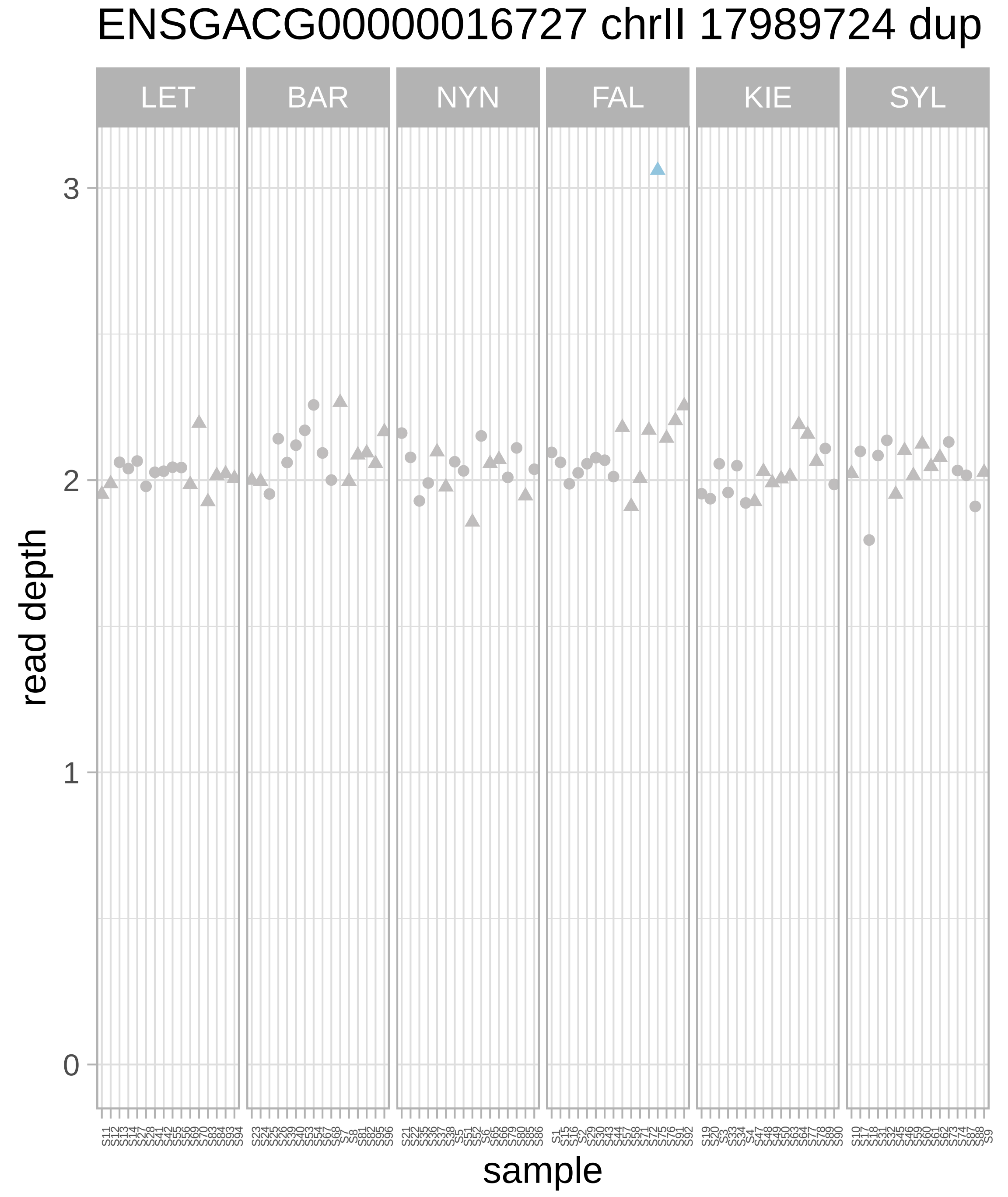
ENSGACG00000016727 chrII 17989724 dup
LET
BAR
NYN
FAL
KIE
SYL
read depth
0
1
2
3
sample

ENSGACG00000017168 chrII 21170824 dup

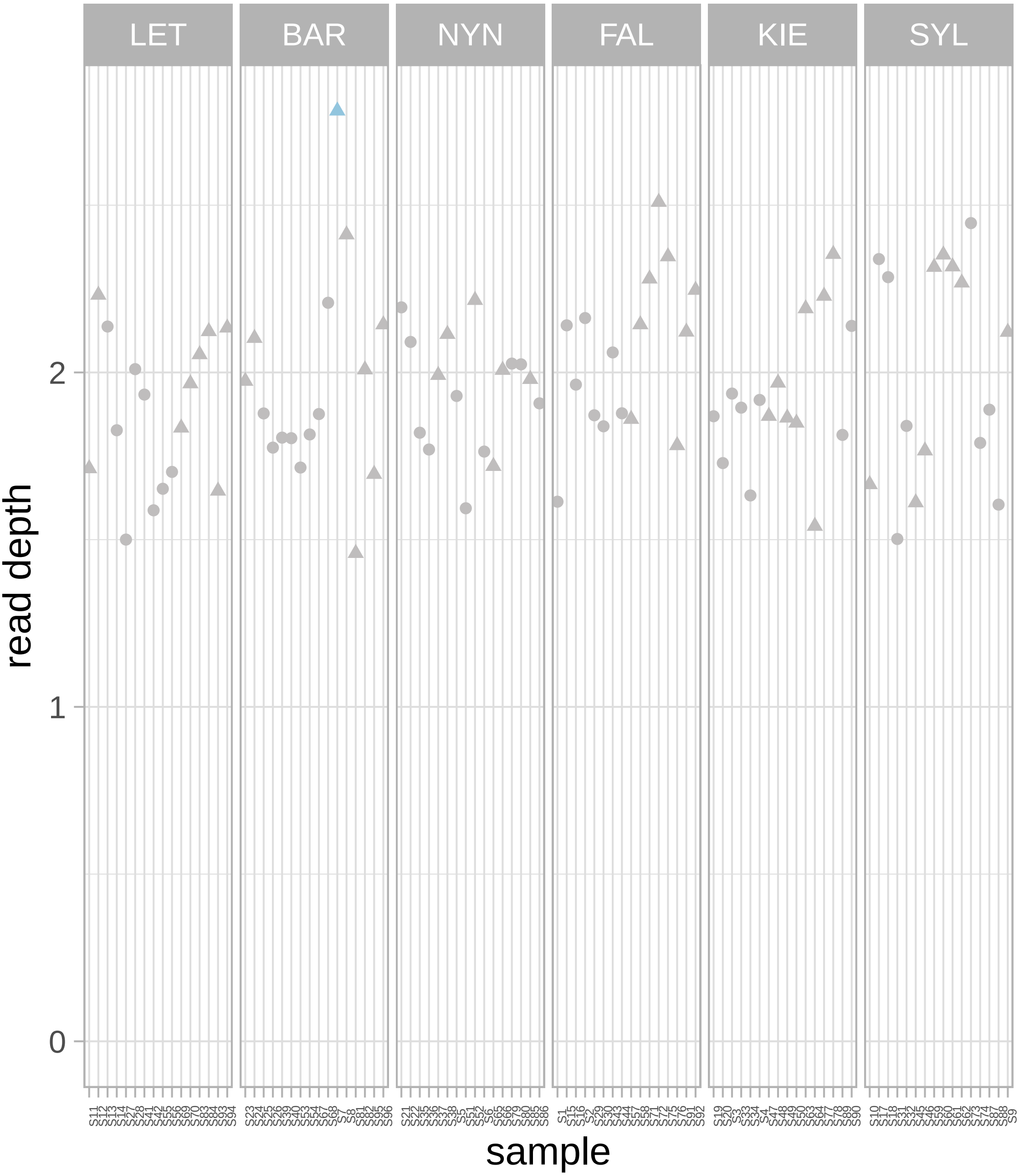

ENSGACG00000017337 chrII 21867140 dup

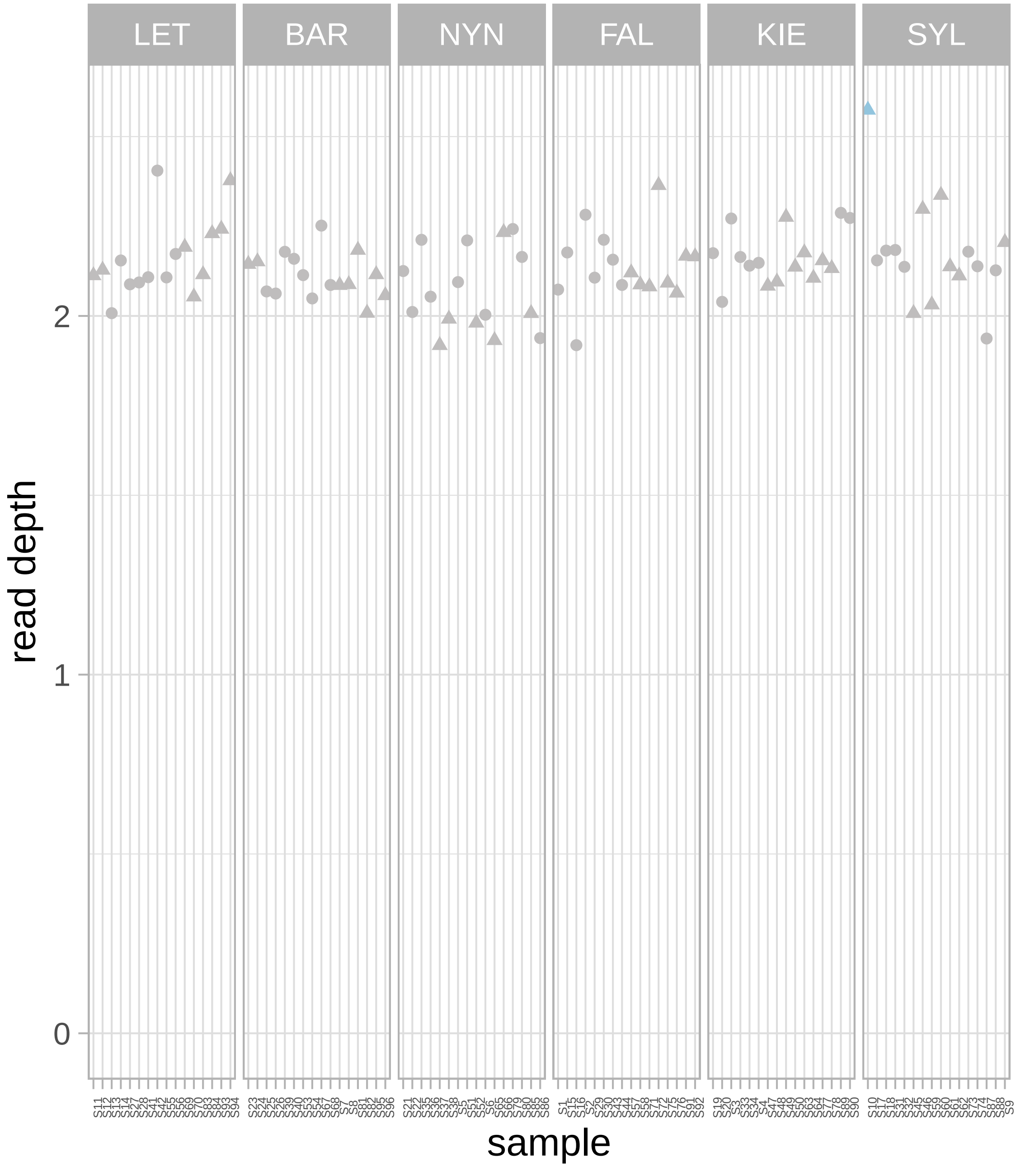

ENSGACG00000017631 chrII 23095751 dup

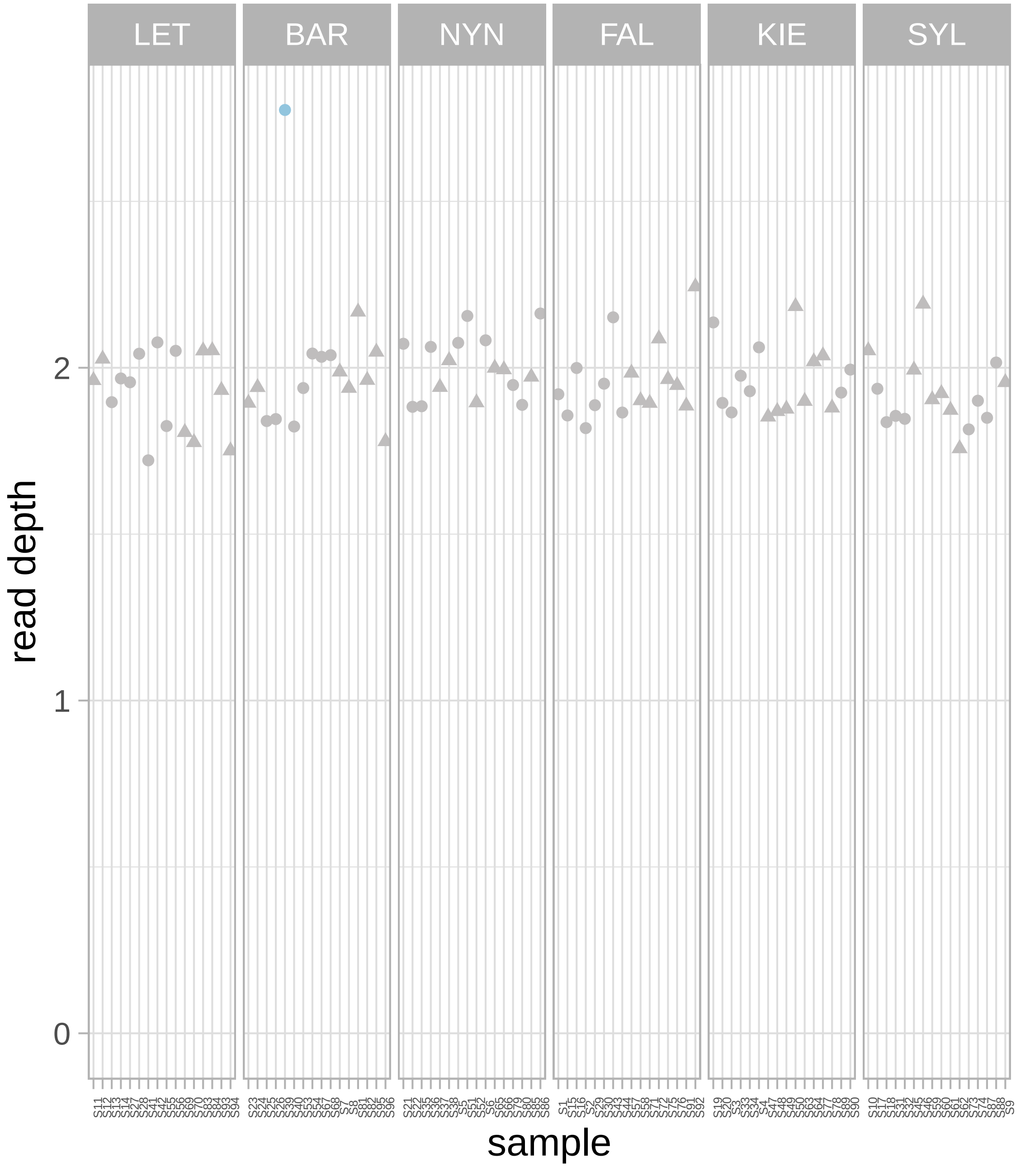

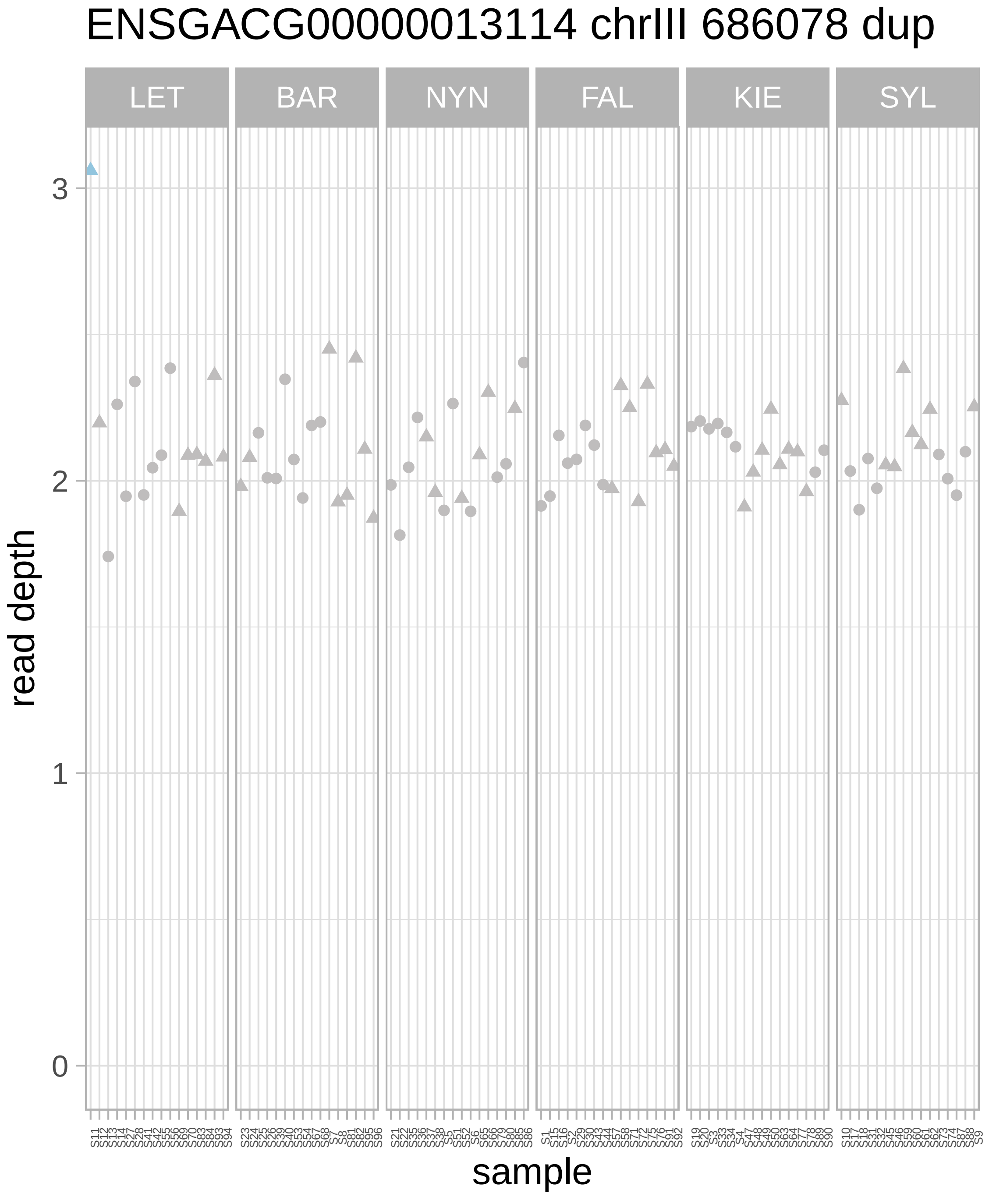
ENSGACG00000013114 chrIII 686078 dup
LET
BAR
NYN
FAL
KIE
SYL
read depth
3
2
1
0
sample

ENSGACG00000014043 chrIII 2763221 dup

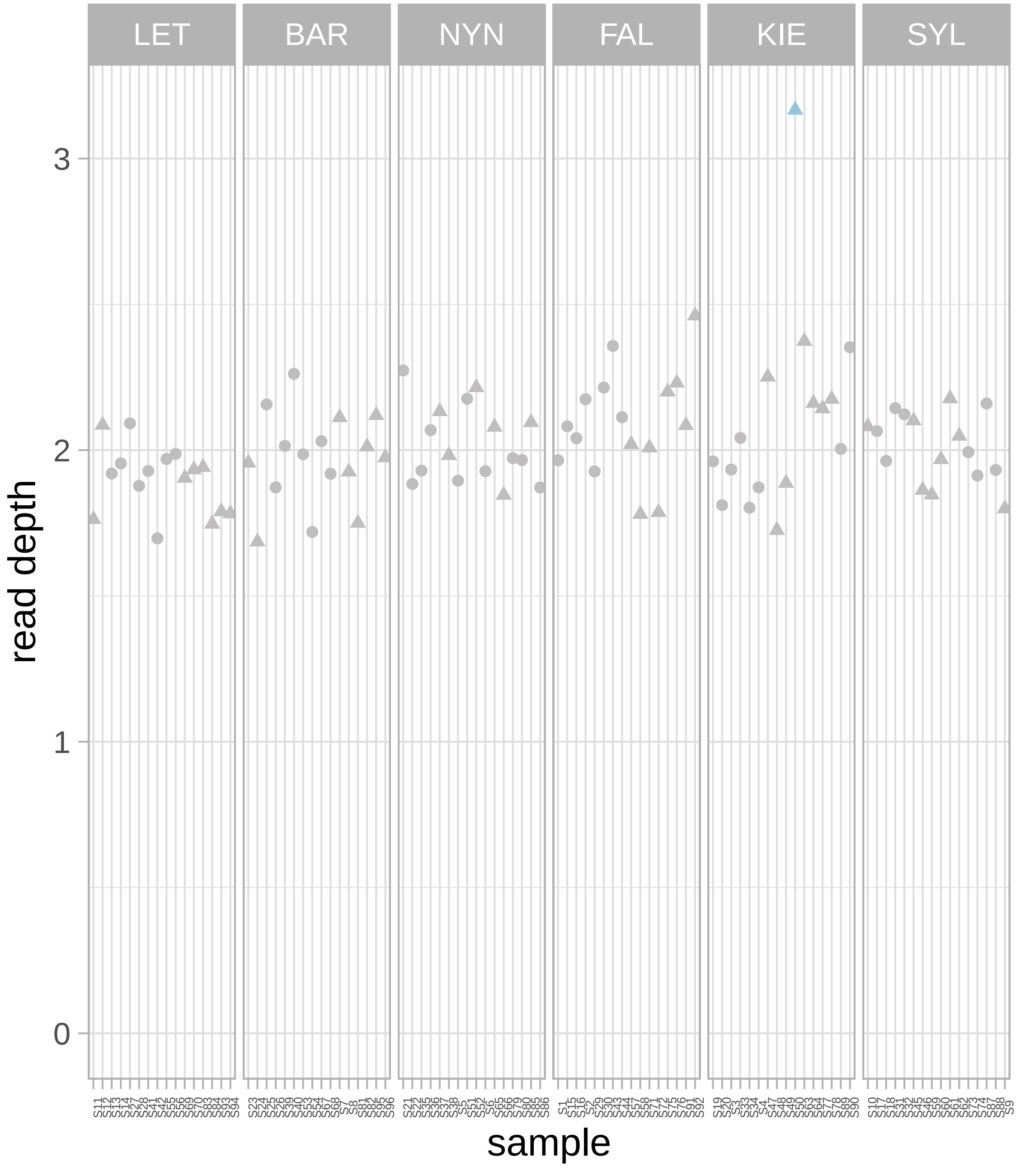

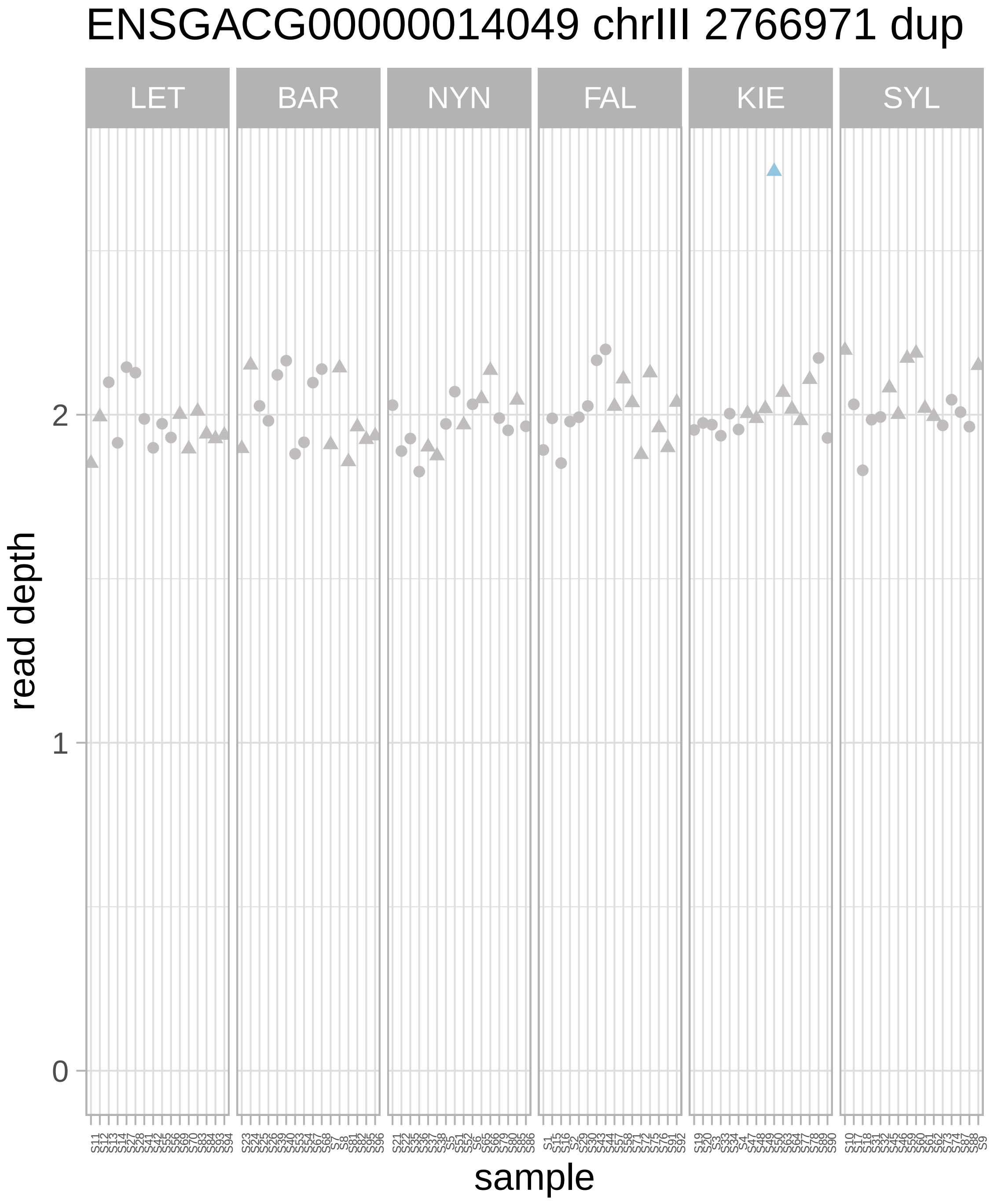
ENSGACG00000014049 chrIII 2766971 dup
LET
BAR
NYN
FAL
KIE
SYL
read depth
0
1
2
S11 S12 S13 S14 S27 S28 S41 S42 S55 S56 S69 S70 S83 S84 S93 S94
S23 S24 S25 S26 S39 S40 S53 S54 S67 S68 S7 S8 S81 S82 S95 S96
S21 S22 S35 S36 S37 S38 S5 S51 S52 S6 S65 S66 S79 S80 S85 S86
S1 S15 S16 S2 S29 S30 S43 S44 S57 S58 S71 S72 S75 S76 S91 S92
S19 S20 S3 S33 S34 S4 S47 S48 S49 S50 S63 S64 S77 S78 S89 S90
S10 S17 S18 S31 S32 S45 S46 S59 S60 S61 S62 S73 S74 S87 S88 S9
sample

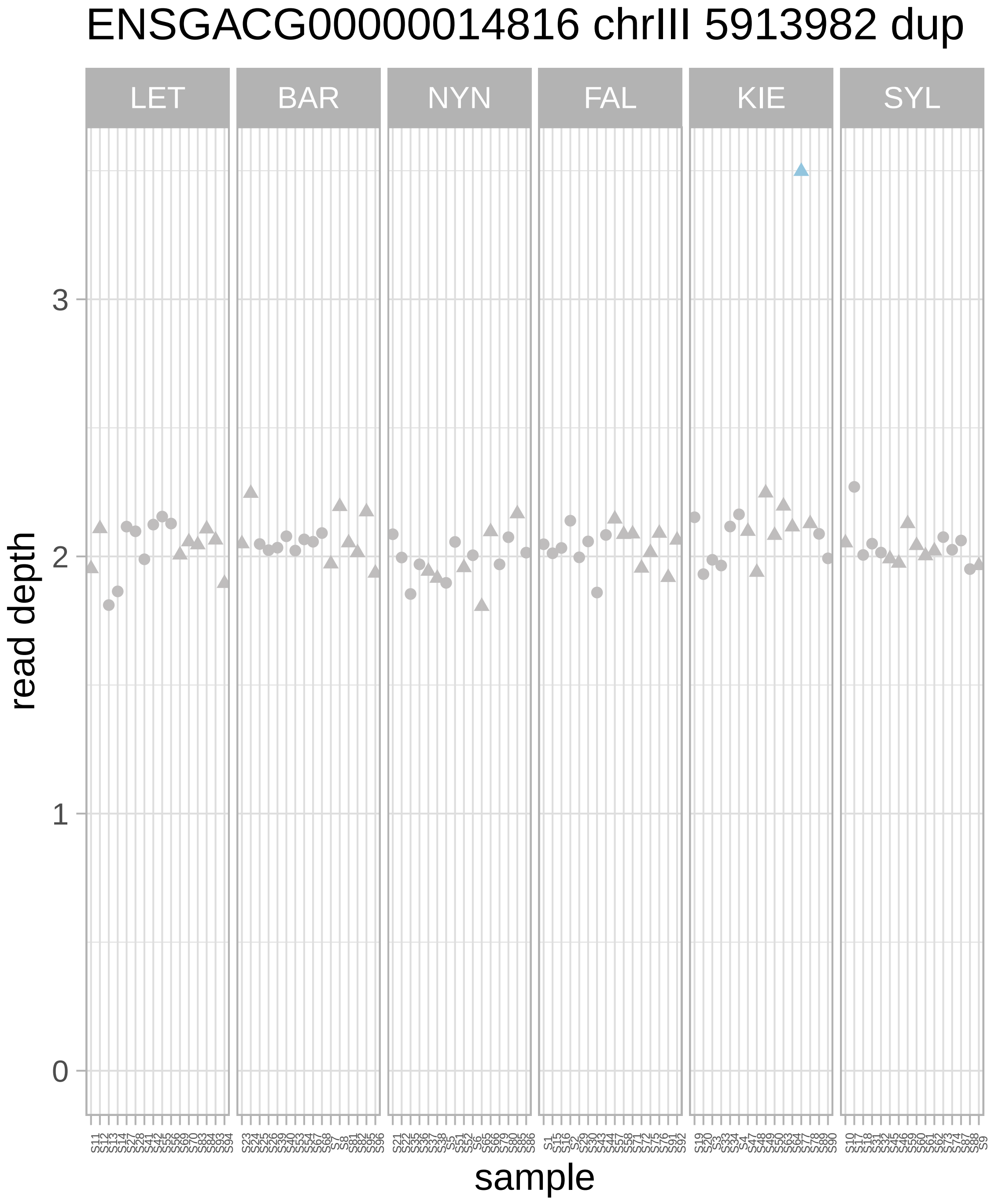

ENSGACG00000014816 chrIII 5913982 dup
LET
BAR
NYN
FAL
KIE
SYL
read depth
0
1
2
3
sample

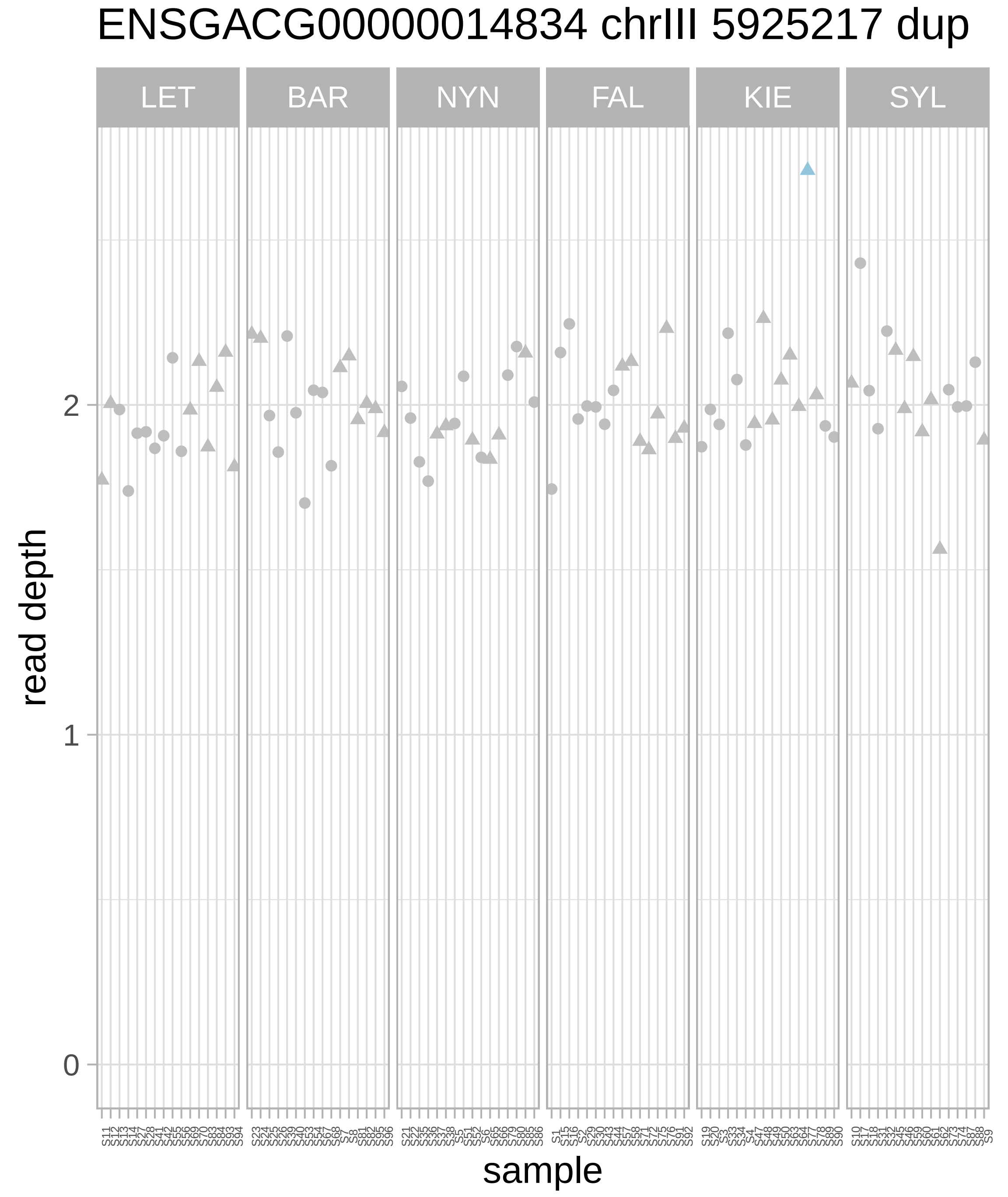

ENSGACG00000014834 chrIII 5925217 dup
LET
BAR
NYN
FAL
KIE
SYL
read depth
0
1
2
sample

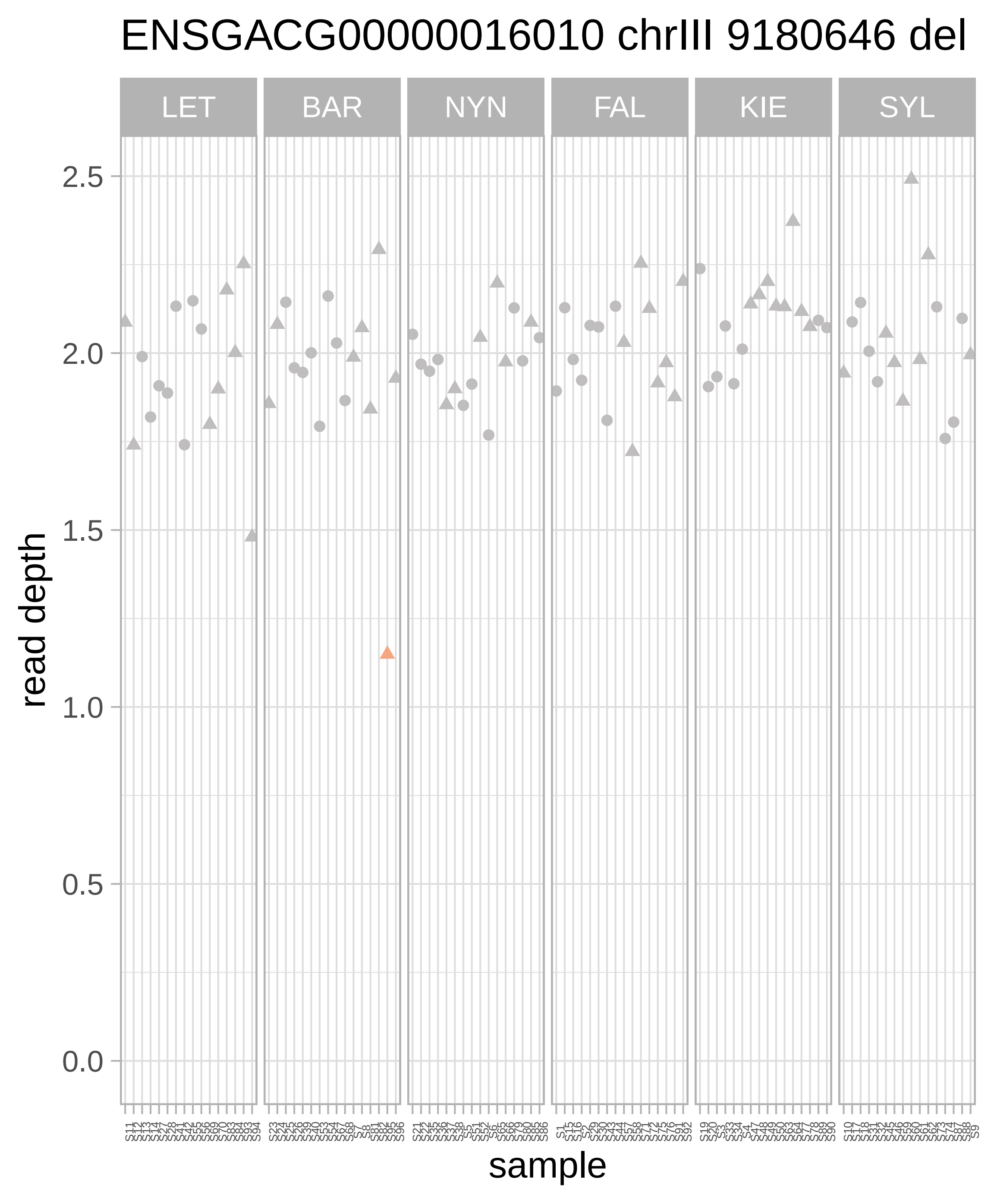
ENSGACG00000016010 chrIII 9180646 del
LET
BAR
NYN
FAL
KIE
SYL
2.5
2.0
1.5
1.0
0.5
0.0
read depth
sample

ENSGACG00000016114 chrIII 9425037 dup

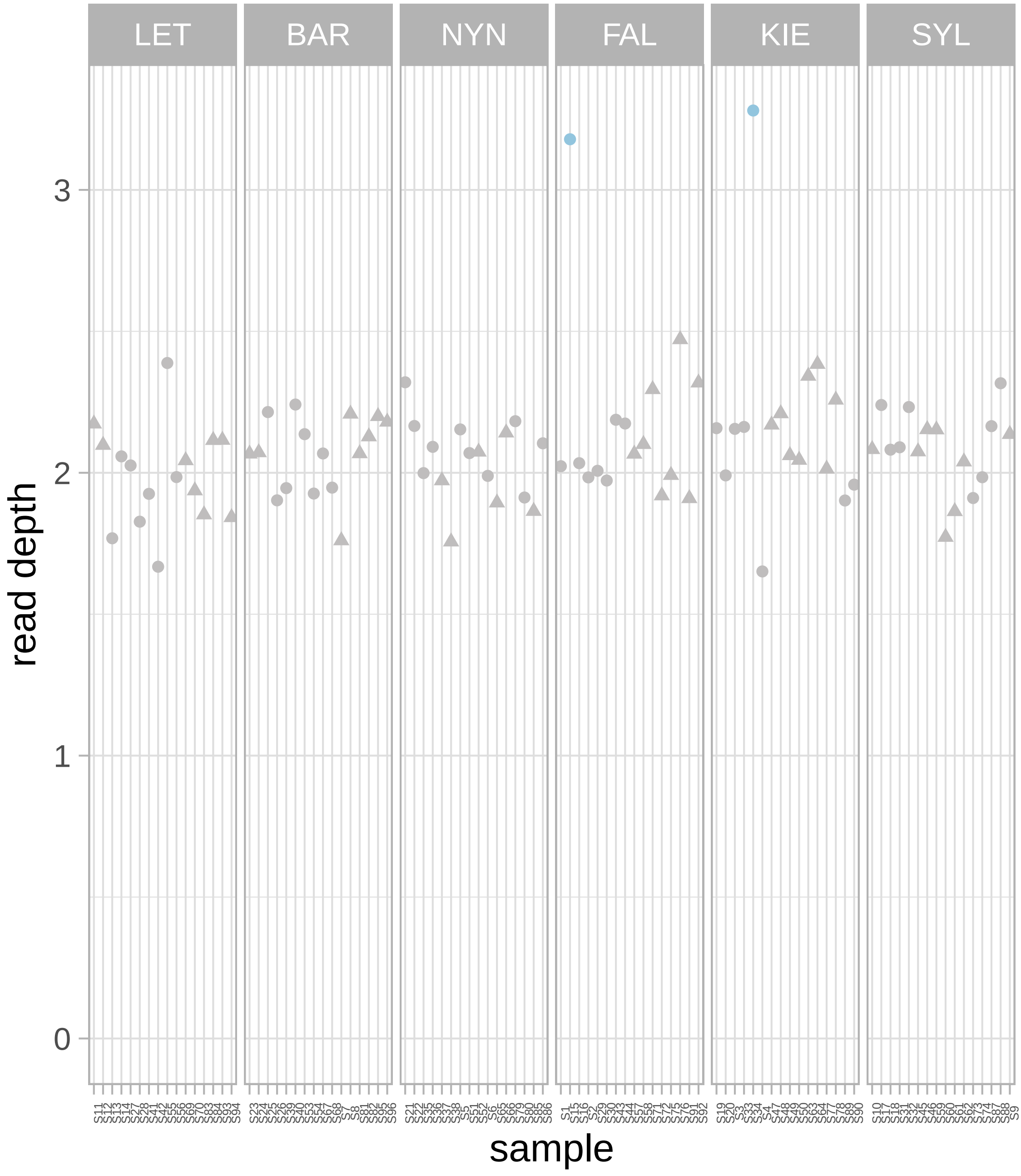

ENSGACG00000017016 chrIII 12615591 dup

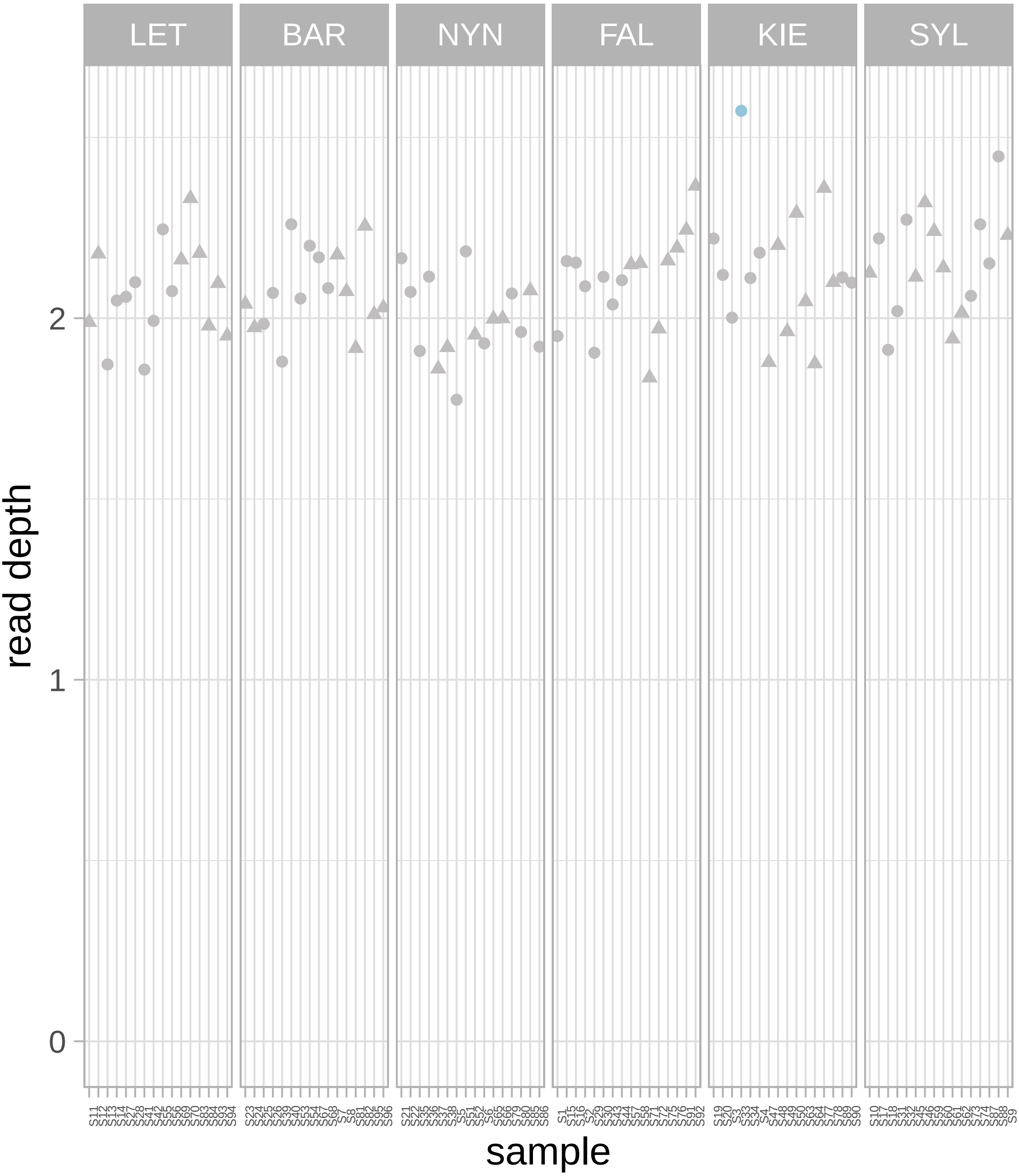

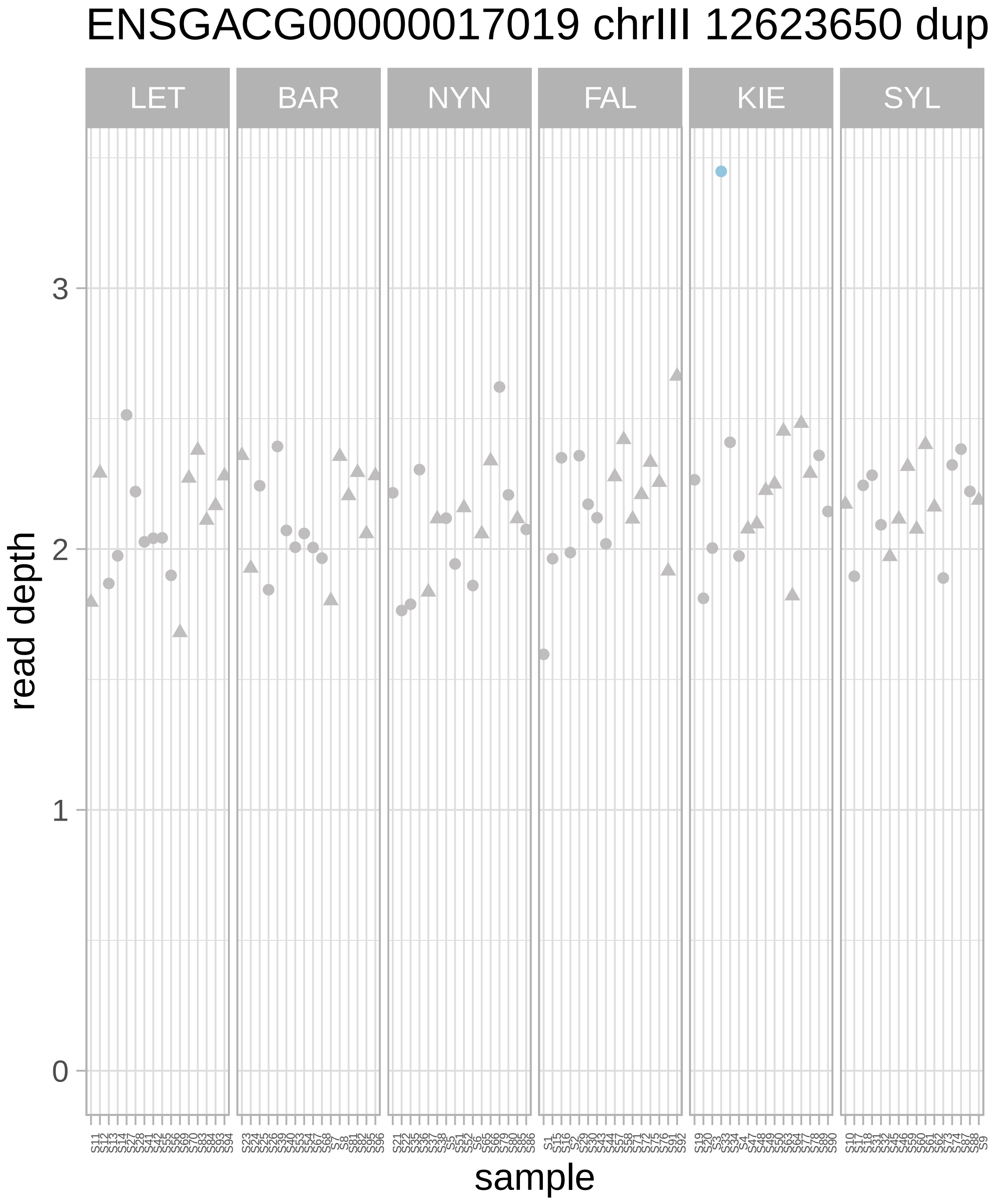
ENSGACG00000017019 chrIII 12623650 dup
LET
BAR
NYN
FAL
KIE
SYL
read depth
3
2
1
0
sample

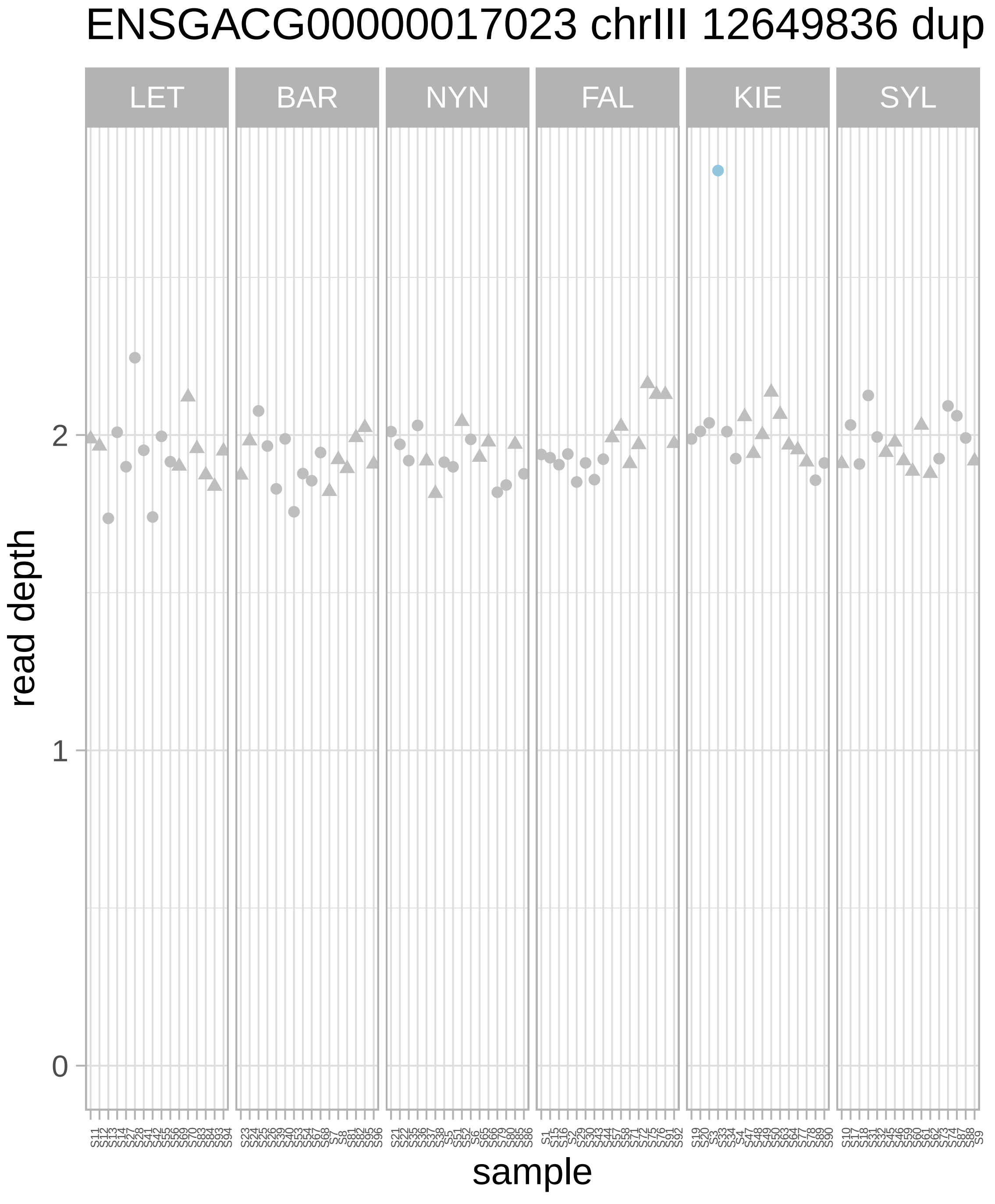
ENSGACG00000017023 chrIII 12649836 dup
LET
BAR
NYN
FAL
KIE
SYL
read depth
0
1
2
S11 S12 S13 S14 S27 S28 S41 S42 S55 S56 S69 S70 S83 S84 S93 S94
S23 S24 S25 S26 S39 S40 S53 S54 S67 S68 S7 S8 S81 S82 S95 S96
S21 S22 S35 S36 S37 S38 S5 S51 S52 S6 S65 S66 S79 S80 S85 S86
S1 S15 S16 S2 S29 S30 S43 S44 S57 S58 S71 S72 S75 S76 S91 S92
S19 S20 S3 S33 S34 S4 S47 S48 S49 S50 S63 S64 S77 S78 S89 S90
S10 S17 S18 S31 S32 S45 S46 S59 S60 S61 S62 S73 S74 S87 S88 S9
sample

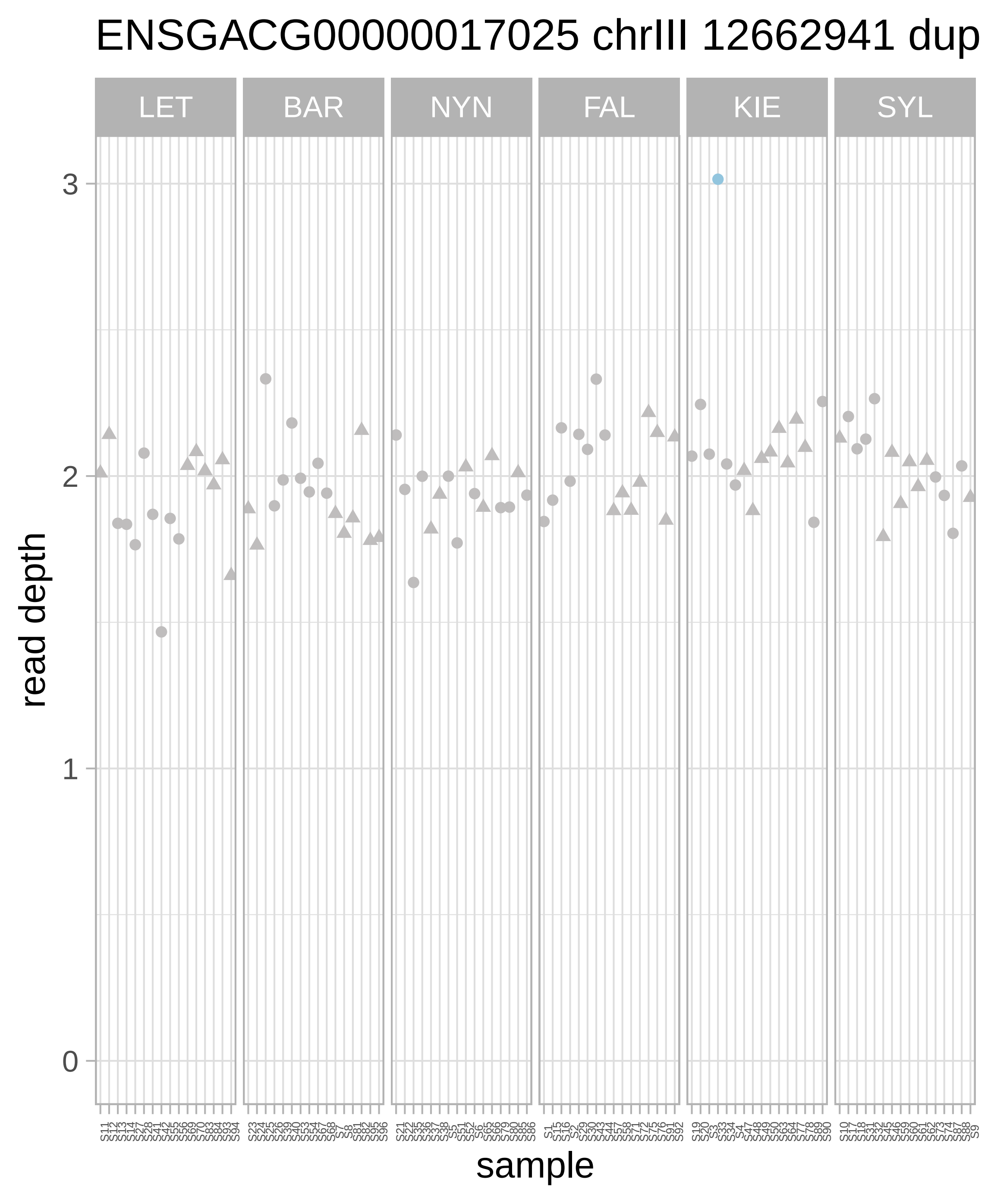
ENSGACG00000017025 chrIII 12662941 dup
LET
BAR
NYN
FAL
KIE
SYL
read depth
3
2
1
0
sample

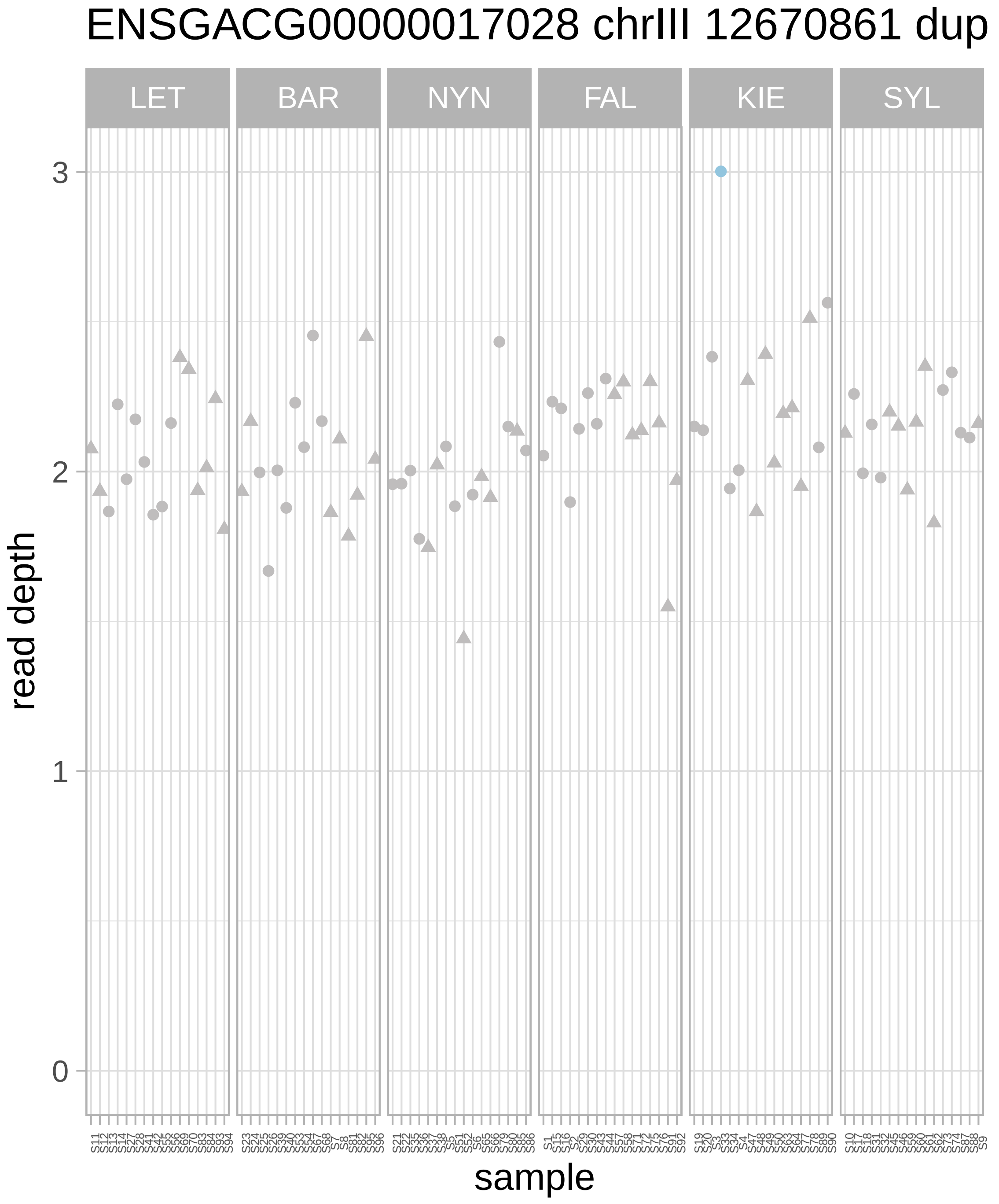
ENSGACG00000017028 chrIII 12670861 dup
LET
BAR
NYN
FAL
KIE
SYL
read depth
0
1
2
3
sample

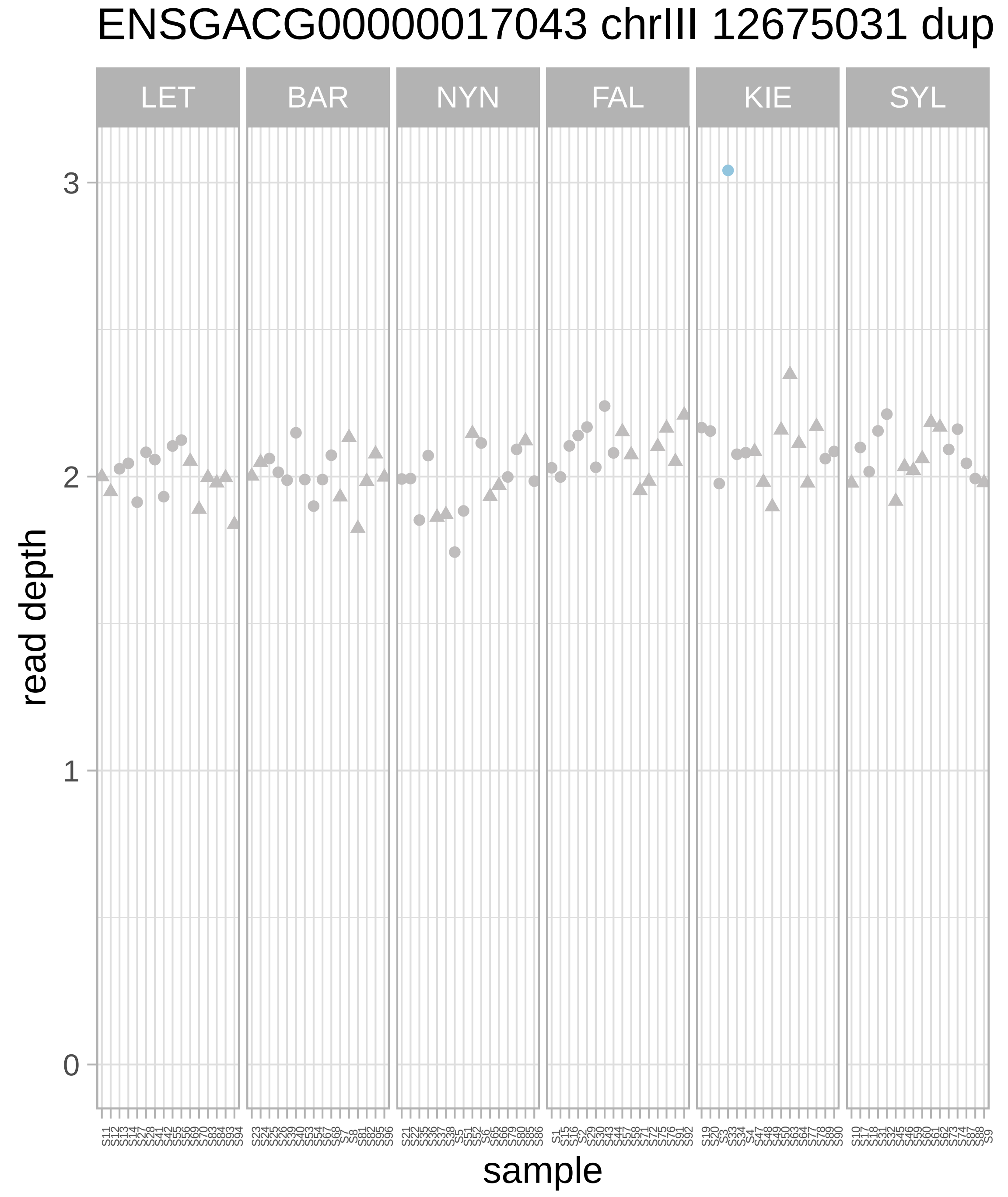
ENSGACG00000017043 chrIII 12675031 dup
LET
BAR
NYN
FAL
KIE
SYL
read depth
3
2
1
0
sample

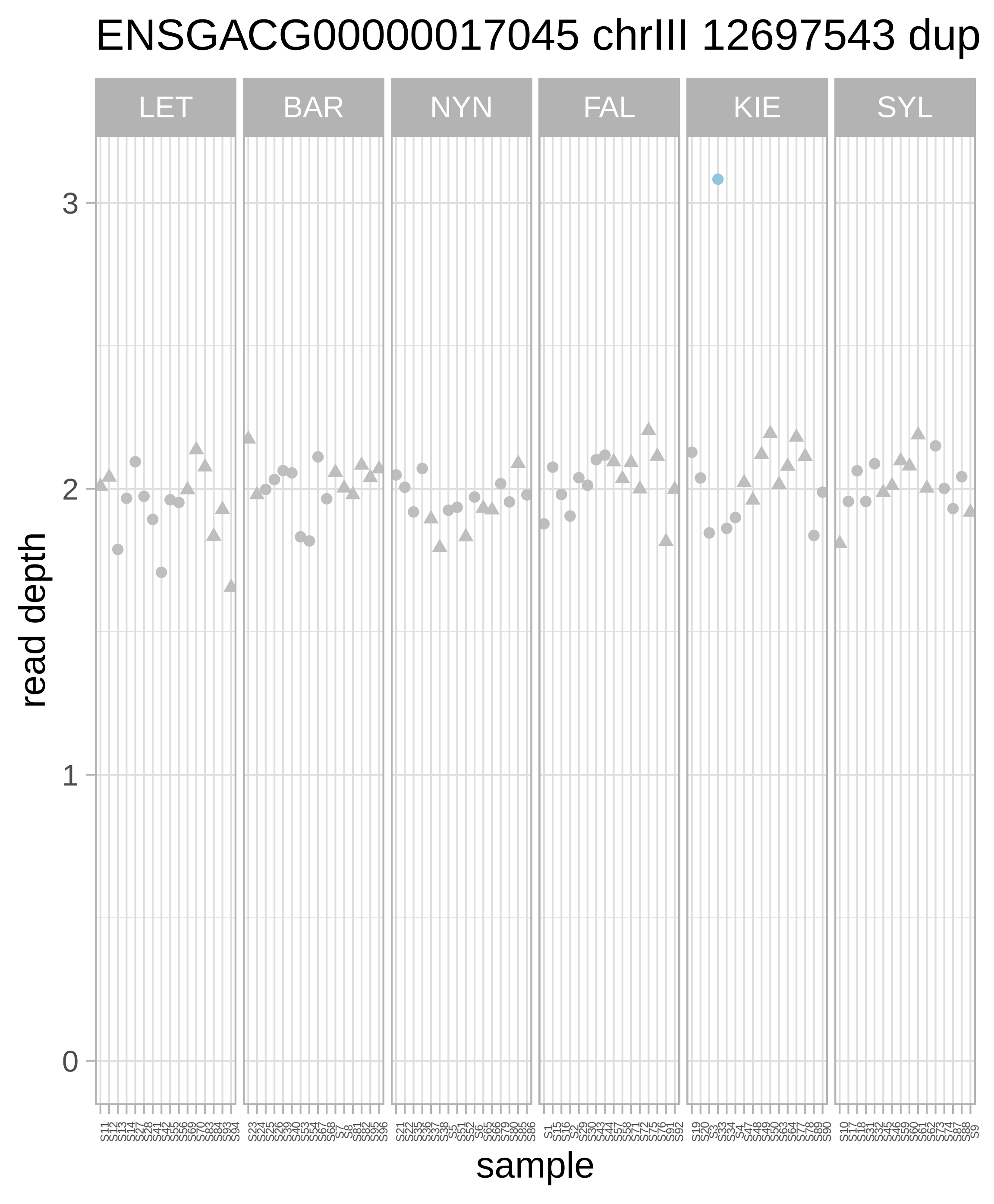

ENSGACG00000017045 chrIII 12697543 dup
LET
BAR
NYN
FAL
KIE
SYL
read depth
0
1
2
3
sample

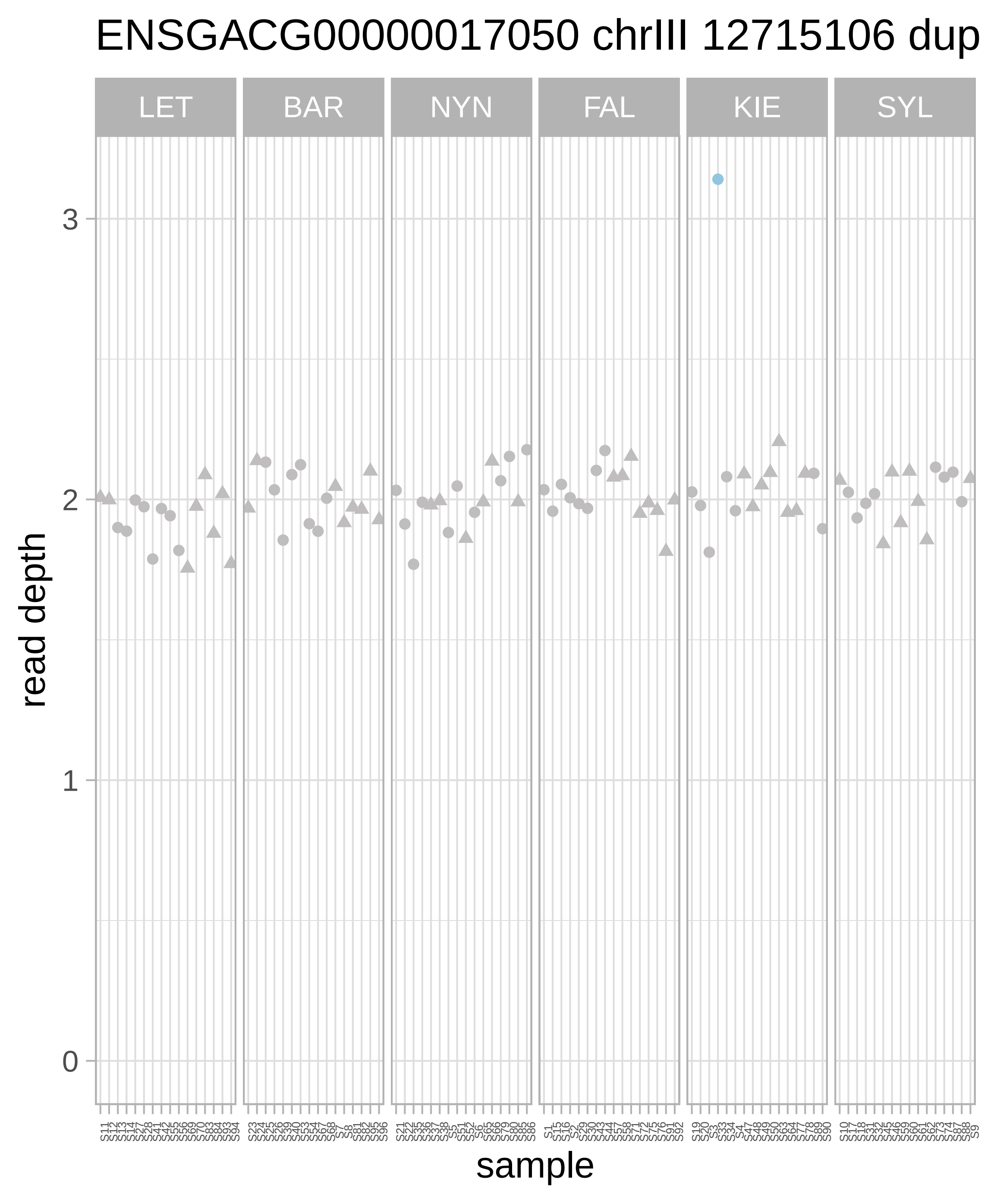
ENSGACG00000017050 chrIII 12715106 dup
LET
BAR
NYN
FAL
KIE
SYL
read depth
0
1
2
3
sample
S11 S12 S13 S14 S27 S28 S41 S42 S55 S56 S69 S70 S83 S84 S93 S94
S23 S24 S25 S26 S39 S40 S53 S54 S67 S68 S7 S8 S81 S82 S95 S96
S21 S22 S35 S36 S37 S38 S5 S51 S52 S6 S65 S66 S79 S80 S85 S86
S1 S15 S16 S2 S29 S30 S43 S44 S57 S58 S71 S72 S75 S76 S91 S92
S19 S20 S3 S33 S34 S4 S47 S48 S49 S50 S63 S64 S77 S78 S89 S90
S10 S17 S18 S31 S32 S45 S46 S59 S60 S61 S62 S73 S74 S87 S88 S9

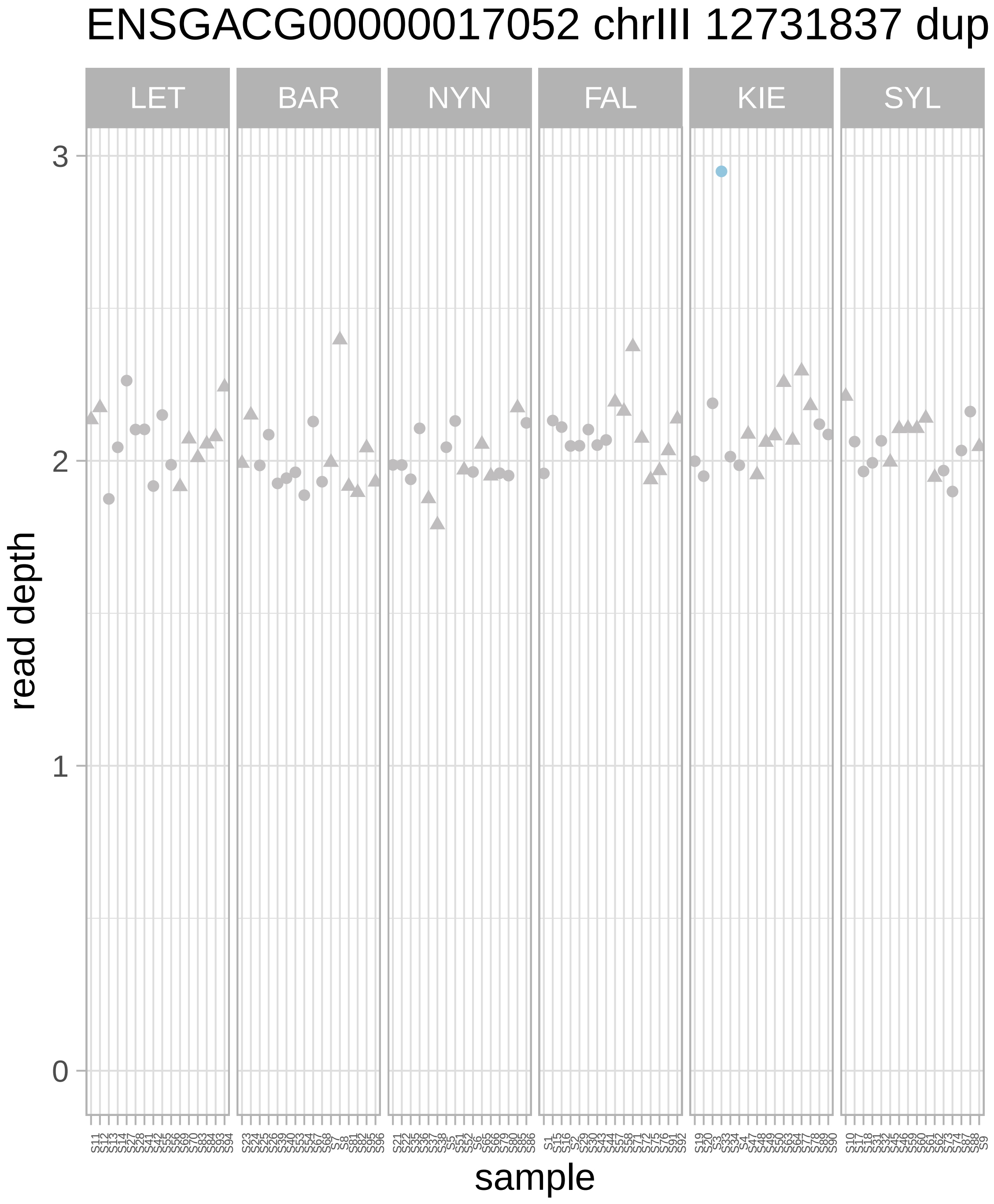
ENSGACG00000017052 chrIII 12731837 dup
LET
BAR
NYN
FAL
KIE
SYL
read depth
3
2
1
0
S11 S12 S13 S14 S27 S28 S41 S42 S55 S56 S69 S70 S83 S84 S93 S94
S23 S24 S25 S26 S39 S40 S53 S54 S67 S68 S7 S8 S81 S82 S95 S96
S21 S22 S35 S36 S37 S38 S5 S51 S52 S6 S65 S66 S79 S80 S85 S86
S1 S15 S16 S2 S29 S30 S43 S44 S57 S58 S71 S72 S75 S76 S91 S92
S19 S20 S3 S33 S34 S4 S47 S48 S49 S50 S63 S64 S77 S78 S89 S90
S10 S17 S18 S31 S32 S45 S46 S59 S60 S61 S62 S73 S74 S87 S88 S9
sample

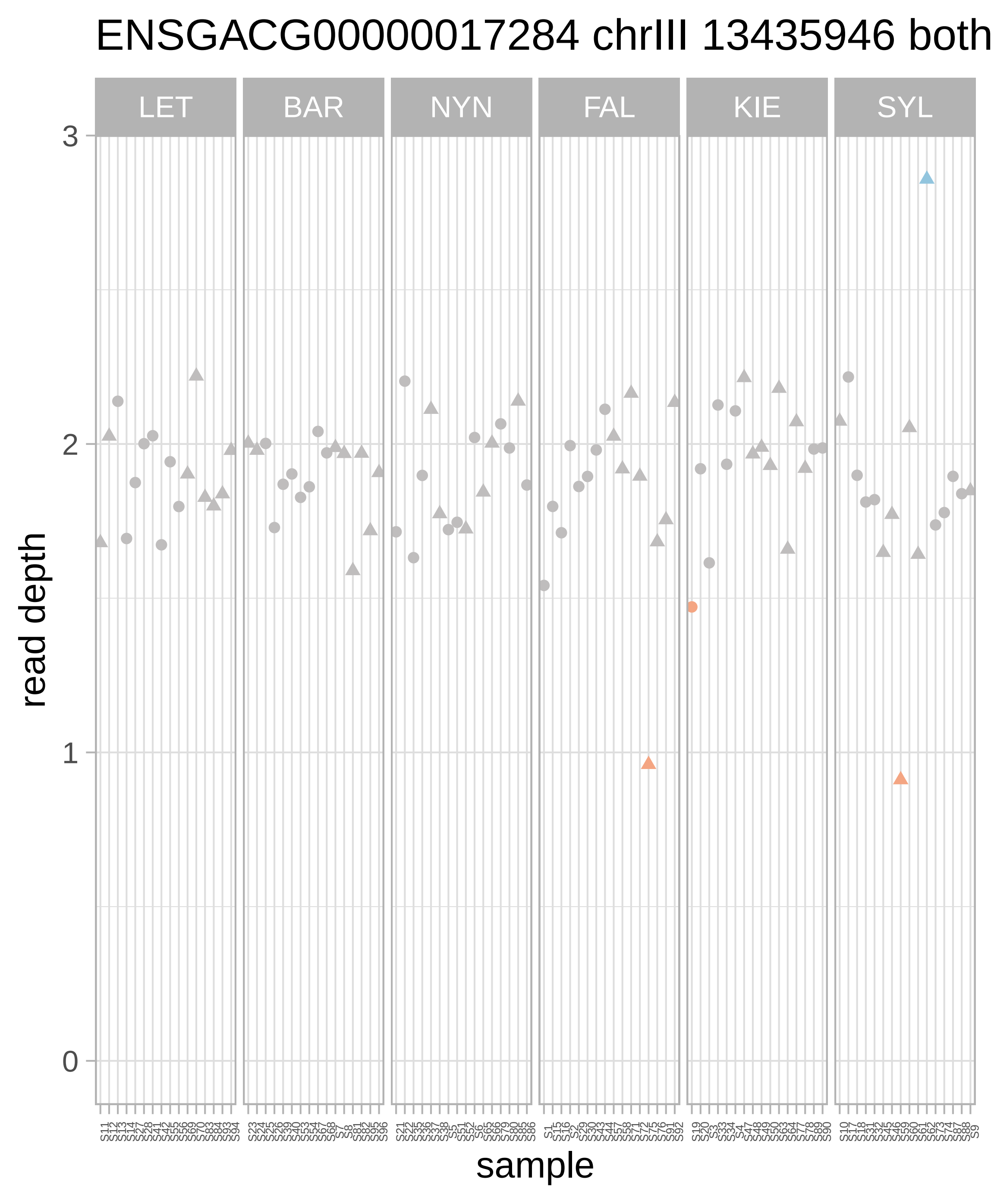

ENSGACG00000017284 chrIII 13435946 both
LET
BAR
NYN
FAL
KIE
SYL
read depth
0
1
2
3
sample

ENSGACG00000018003 chrIII 16775134 both

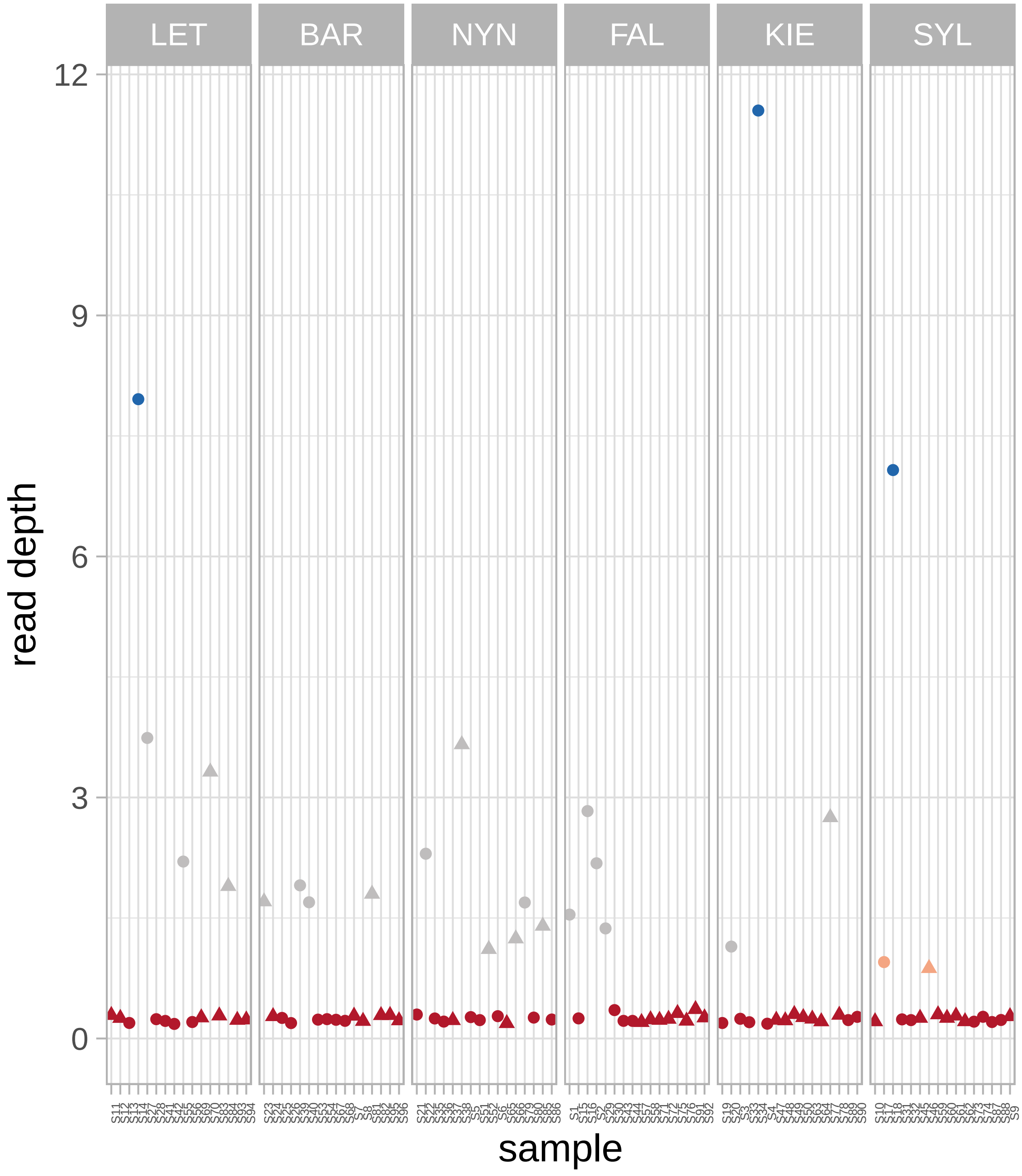

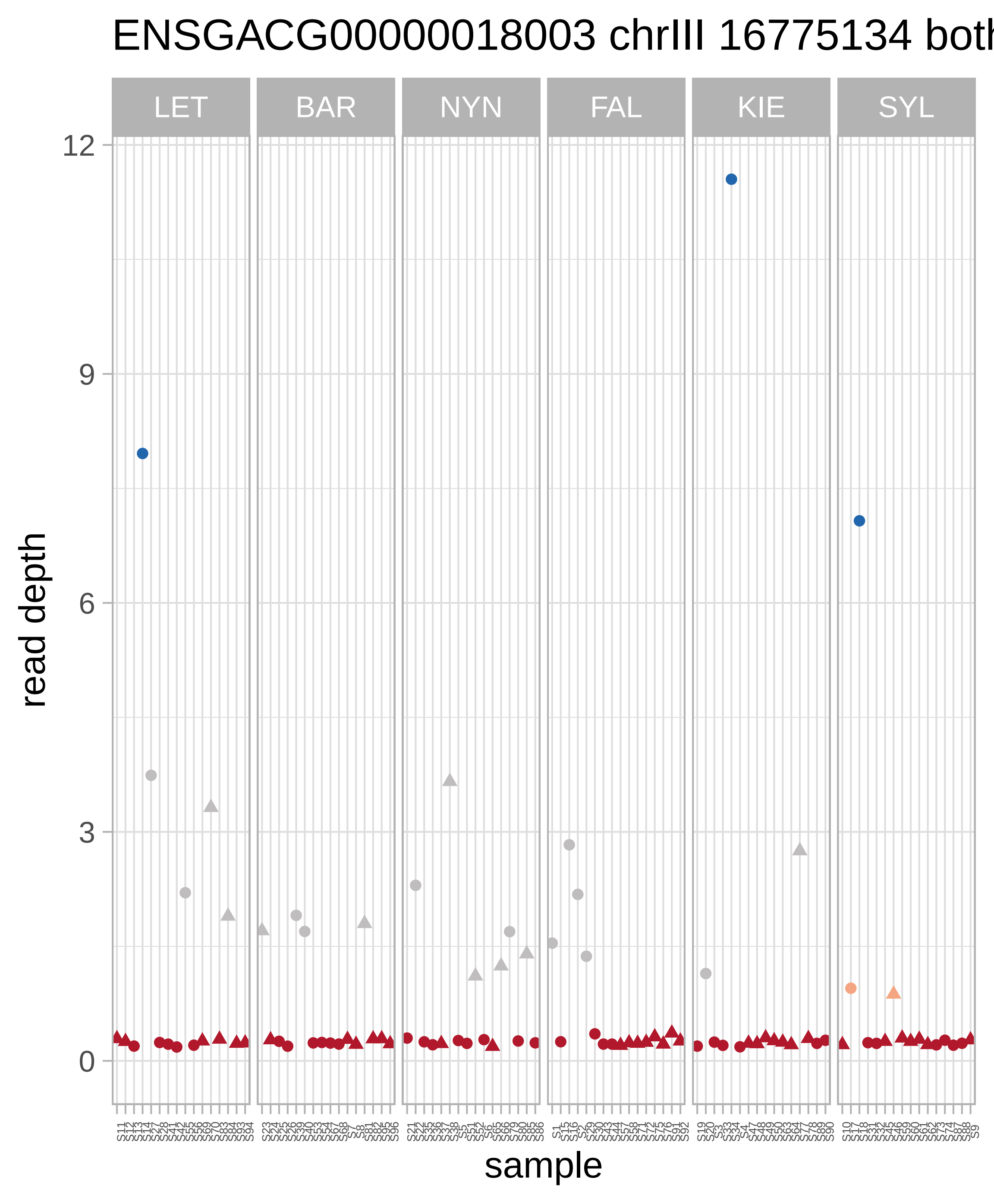
ENSGACG00000018003 chrIII 16775134 both
LET
BAR
NYN
FAL
KIE
SYL
read depth
12
9
6
3
0
sample

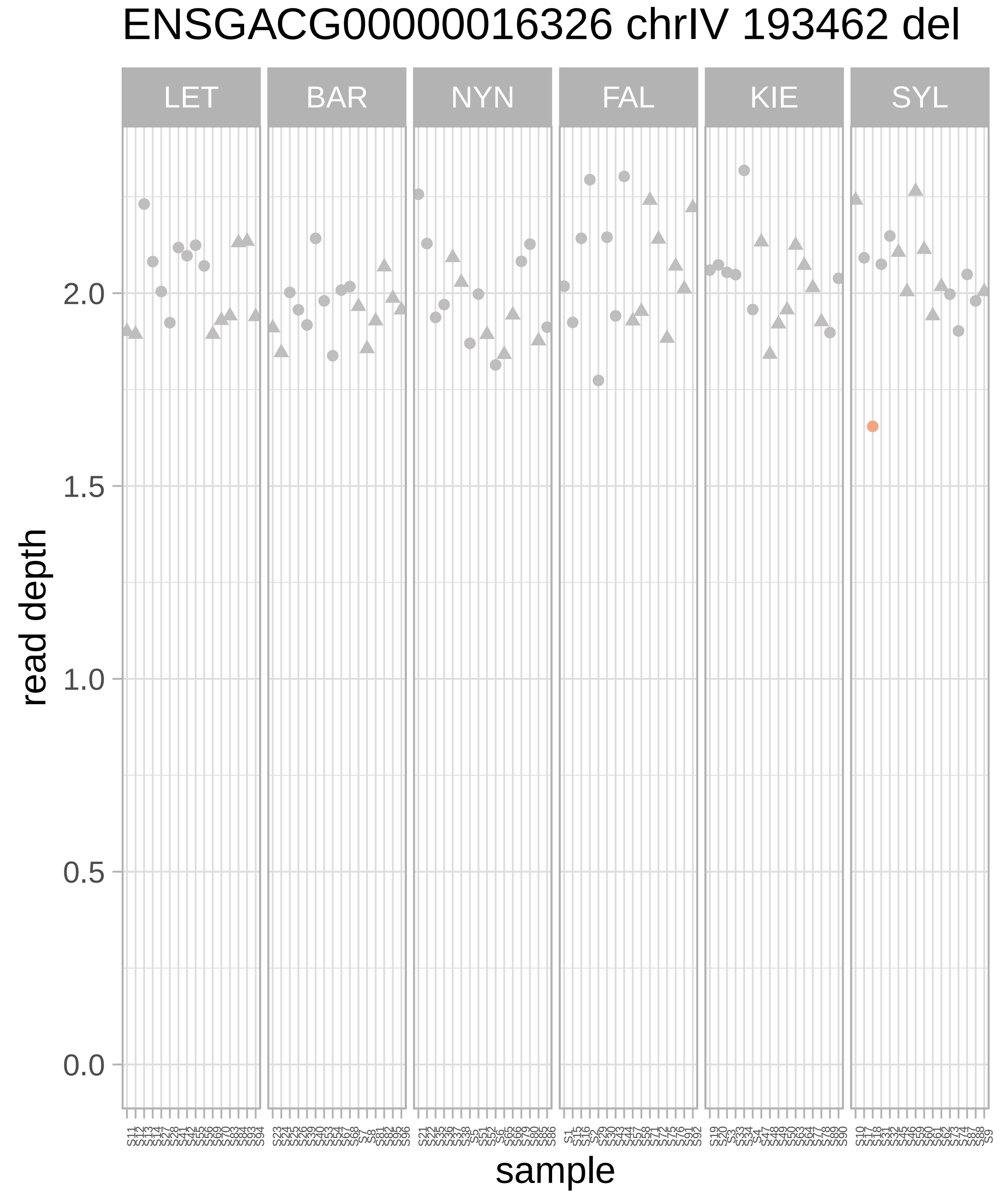
ENSGACG00000016326 chrIV 193462 del
LET
BAR
NYN
FAL
KIE
SYL
read depth
2.0
1.5
1.0
0.5
0.0
S11 S12 S13 S14 S27 S28 S41 S42 S55 S56 S69 S70 S83 S84 S93 S94
S23 S24 S25 S26 S39 S40 S53 S54 S67 S68 S7 S8 S81 S82 S95 S96
S21 S22 S35 S36 S37 S38 S5 S51 S52 S6 S65 S66 S79 S80 S85 S86
S1 S15 S16 S2 S29 S30 S43 S44 S57 S58 S71 S72 S75 S76 S91 S92
S19 S20 S3 S33 S34 S4 S47 S48 S49 S50 S63 S64 S77 S78 S89 S90
S10 S17 S18 S31 S32 S45 S46 S59 S60 S61 S62 S73 S74 S87 S88 S9
sample

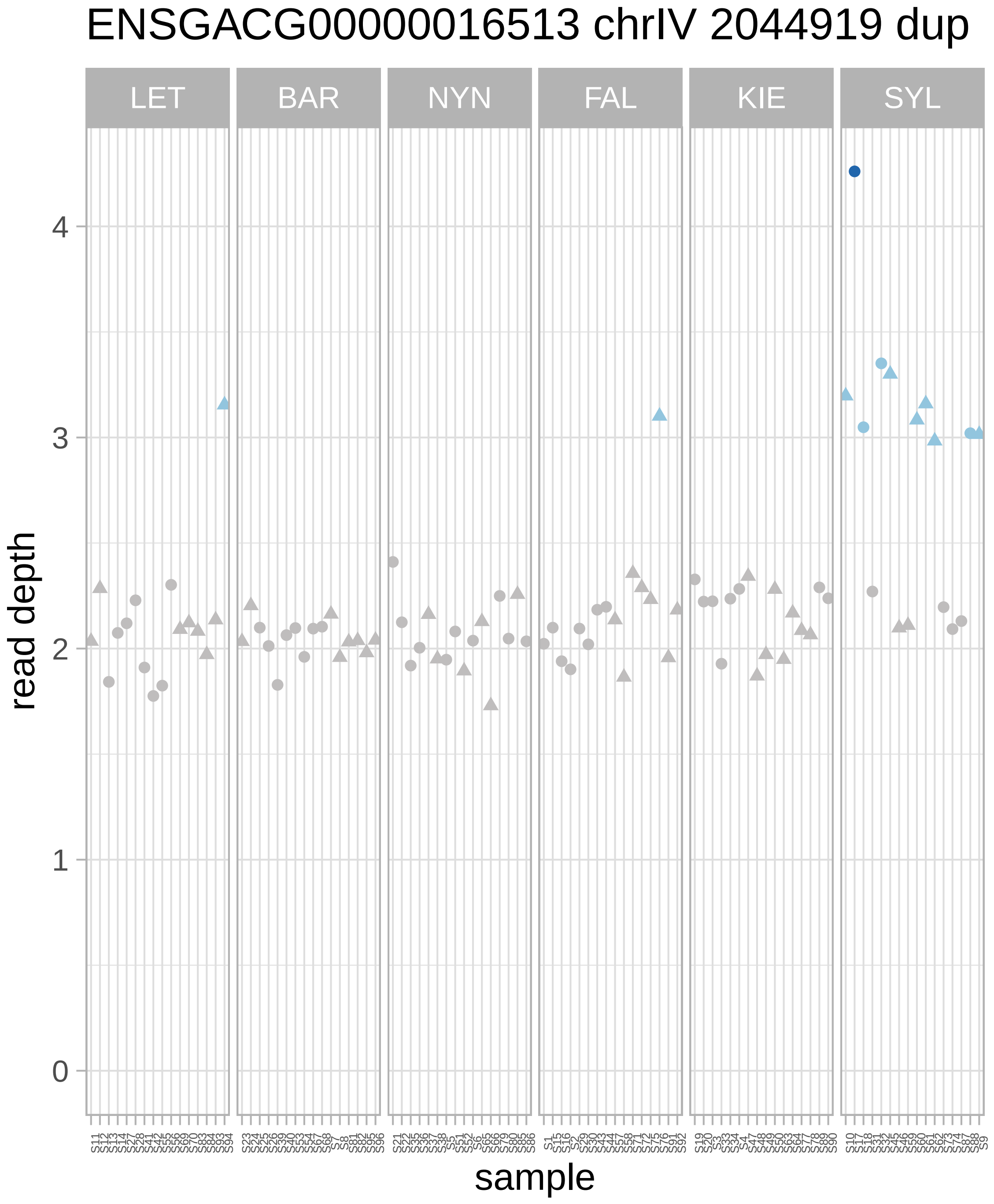
ENSGACG00000016513 chrIV 2044919 dup
LET
BAR
NYN
FAL
KIE
SYL
read depth
0
1
2
3
4
sample
S11 S12 S13 S14 S27 S28 S41 S42 S55 S56 S69 S70 S83 S84 S93 S94
S23 S24 S25 S26 S39 S40 S53 S54 S67 S68 S7 S8 S81 S82 S95 S96
S21 S22 S35 S36 S37 S38 S5 S51 S52 S6 S65 S66 S79 S80 S85 S86
S1 S15 S16 S2 S29 S30 S43 S44 S57 S58 S71 S72 S75 S76 S91 S92
S19 S20 S3 S33 S34 S4 S47 S48 S49 S50 S63 S64 S77 S78 S89 S90
S10 S17 S18 S31 S32 S45 S46 S59 S60 S61 S62 S73 S74 S87 S88 S9

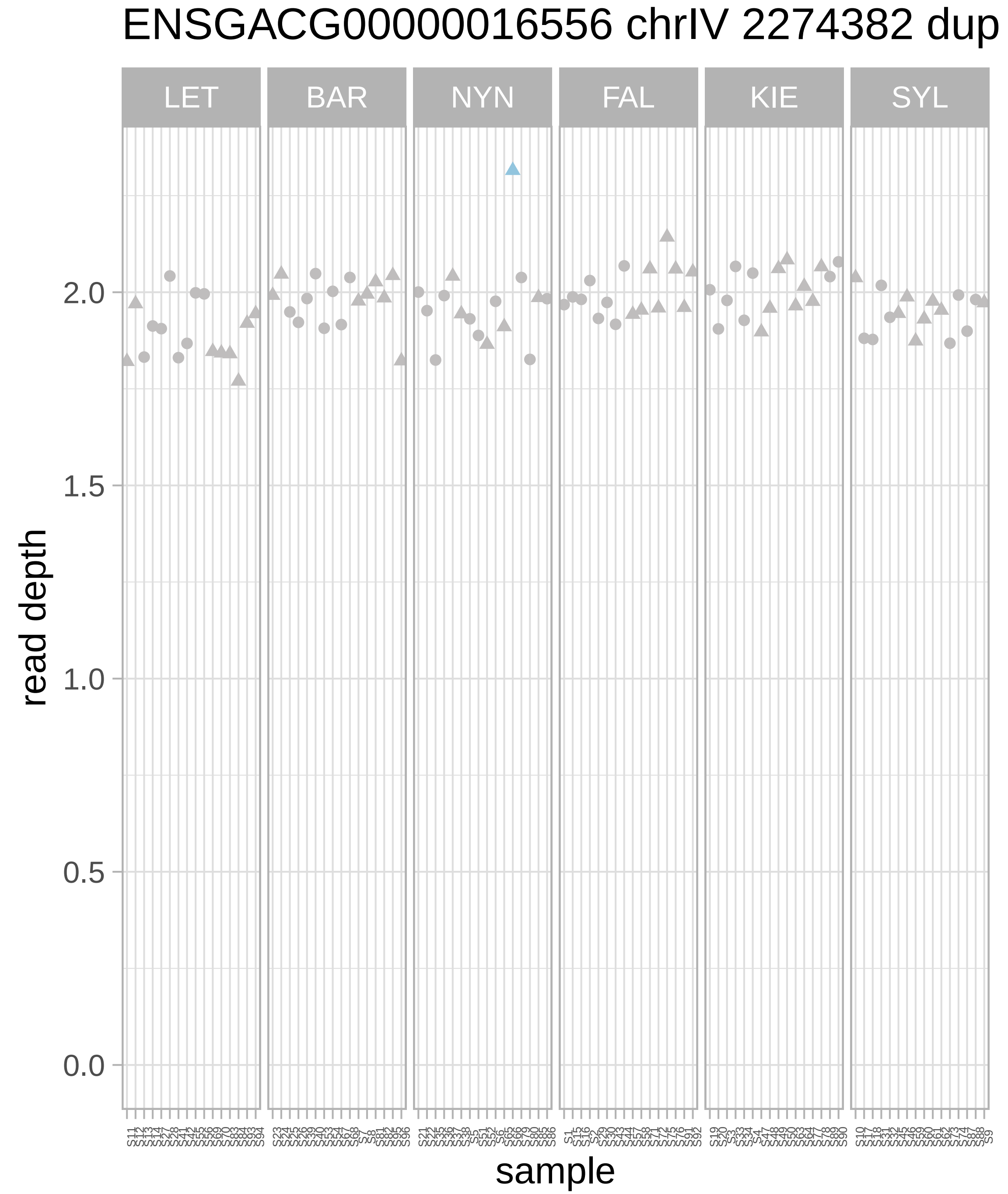

ENSGACG00000016556 chrIV 2274382 dup
LET
BAR
NYN
FAL
KIE
SYL
read depth
2.0
1.5
1.0
0.5
0.0
sample

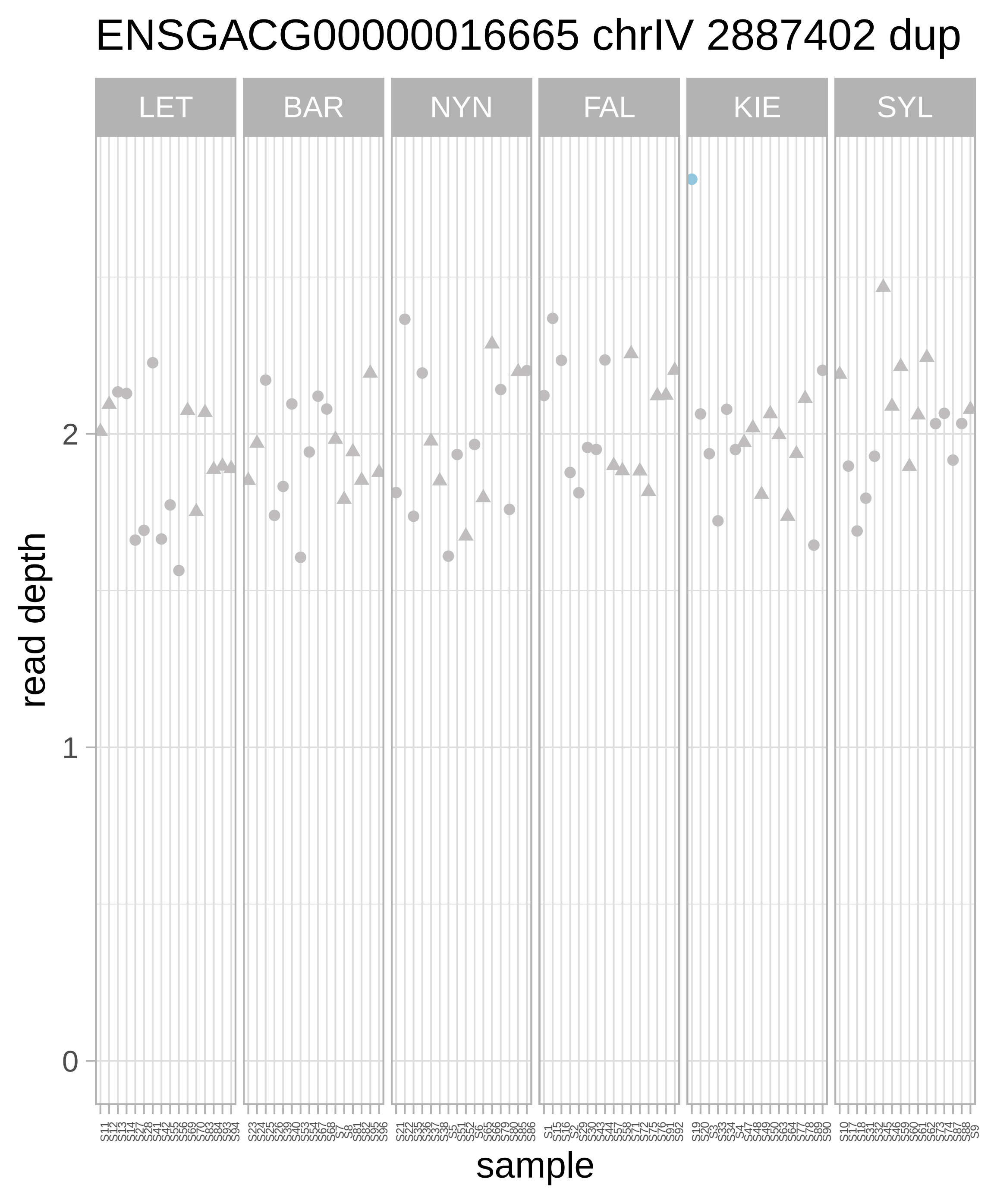
ENSGACG00000016665 chrIV 2887402 dup
LET
BAR
NYN
FAL
KIE
SYL
read depth
2
1
0
sample

ENSGACG00000016673 chrIV 2891431 dup

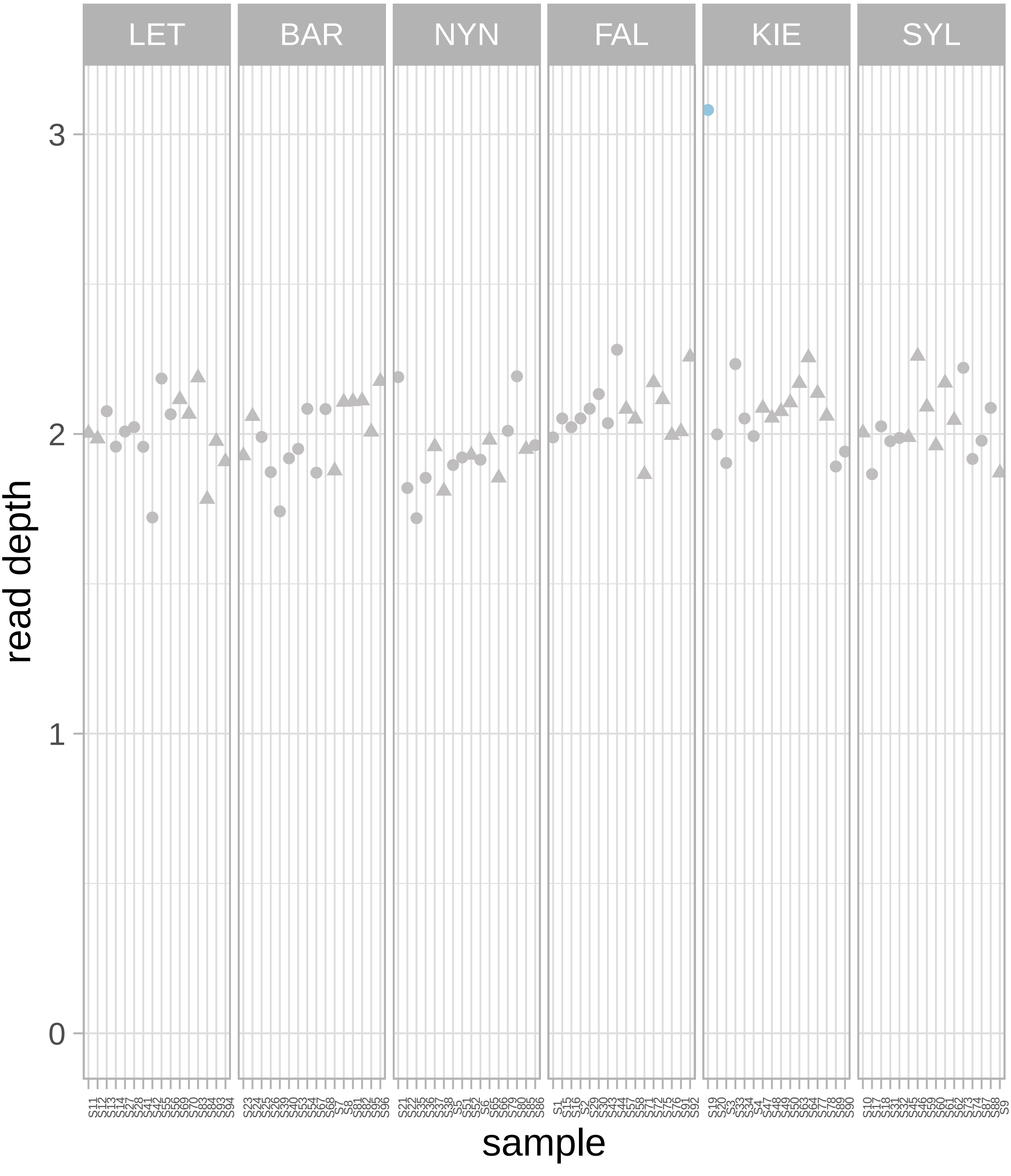

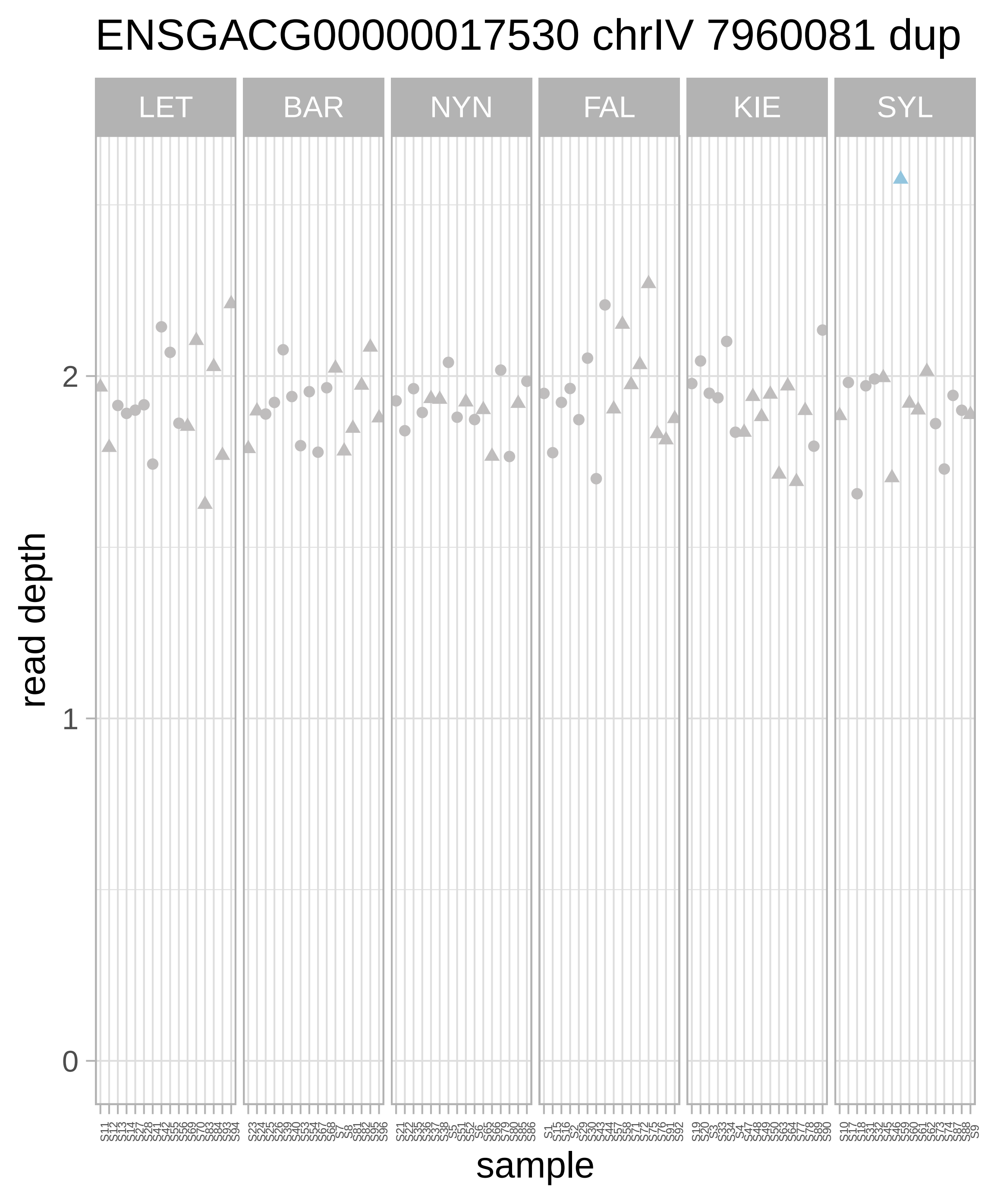
ENSGACG00000017530 chrIV 7960081 dup
LET
BAR
NYN
FAL
KIE
SYL
read depth
0
1
2
sample

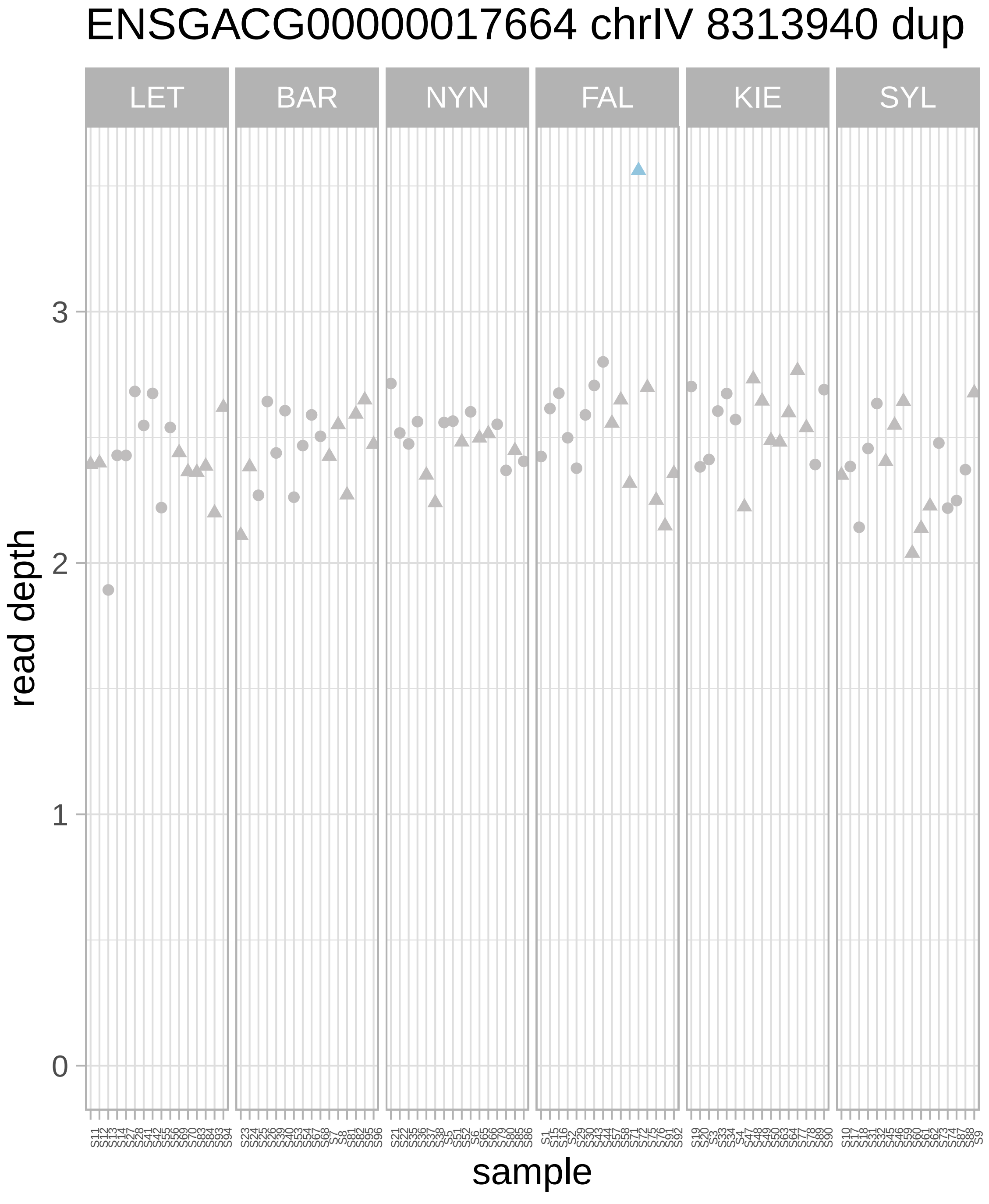
ENSGACG00000017664 chrIV 8313940 dup
LET
BAR
NYN
FAL
KIE
SYL
read depth
0
1
2
3
sample

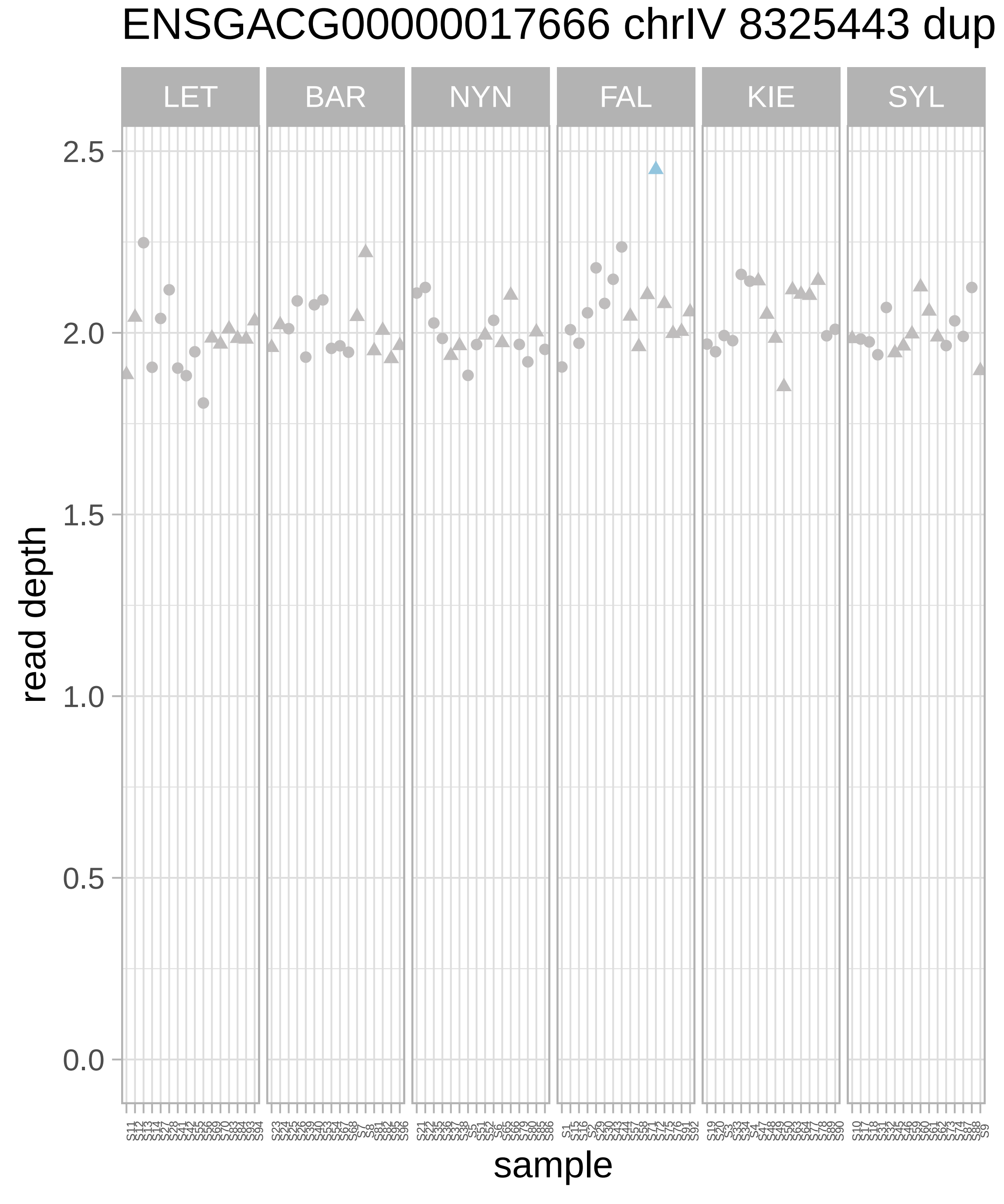
ENSGACG00000017666 chrIV 8325443 dup
LET
BAR
NYN
FAL
KIE
SYL
2.5
2.0
1.5
1.0
0.5
0.0
read depth
sample

ENSGACG00000017733 chrIV 9165337 dup

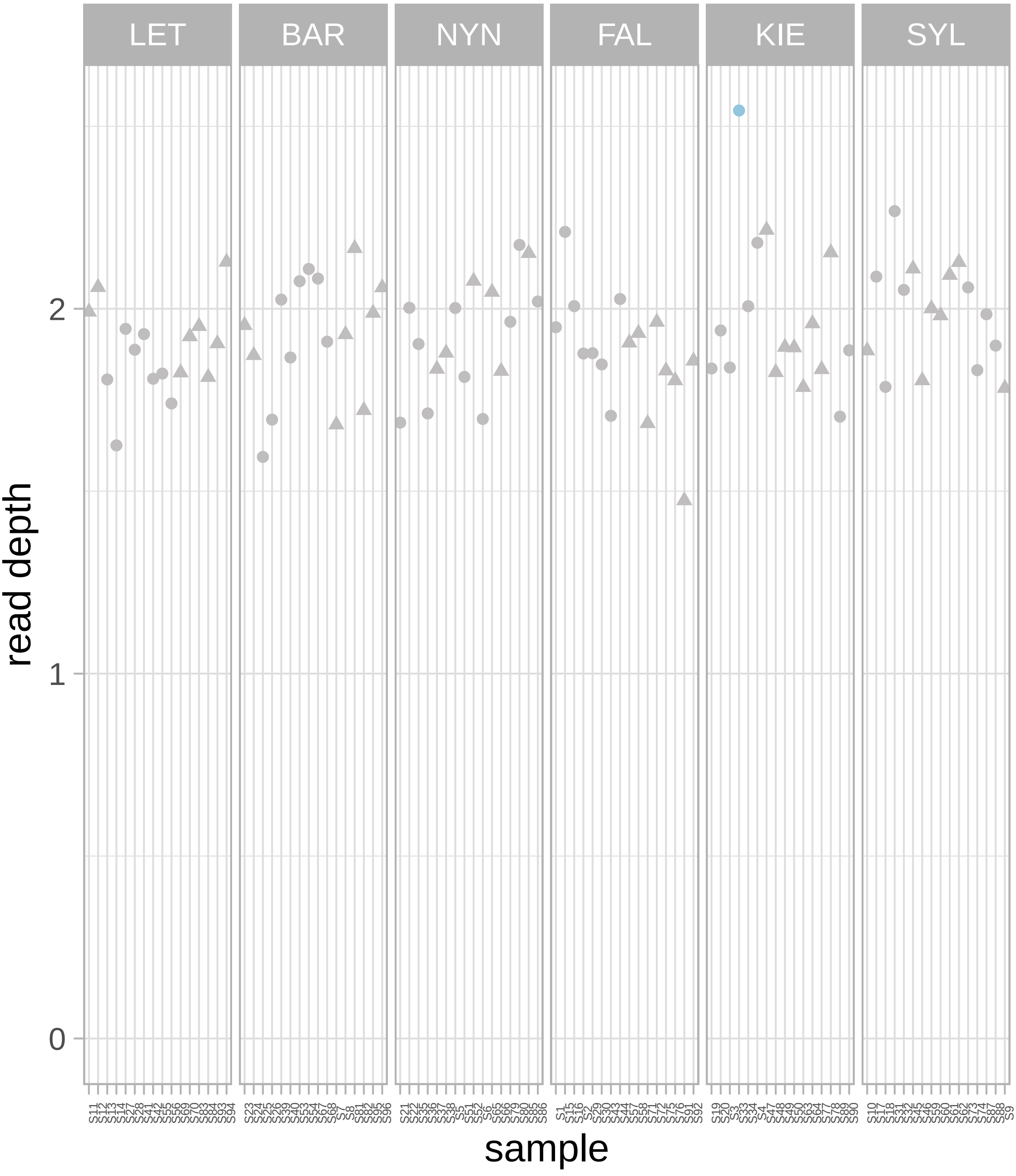

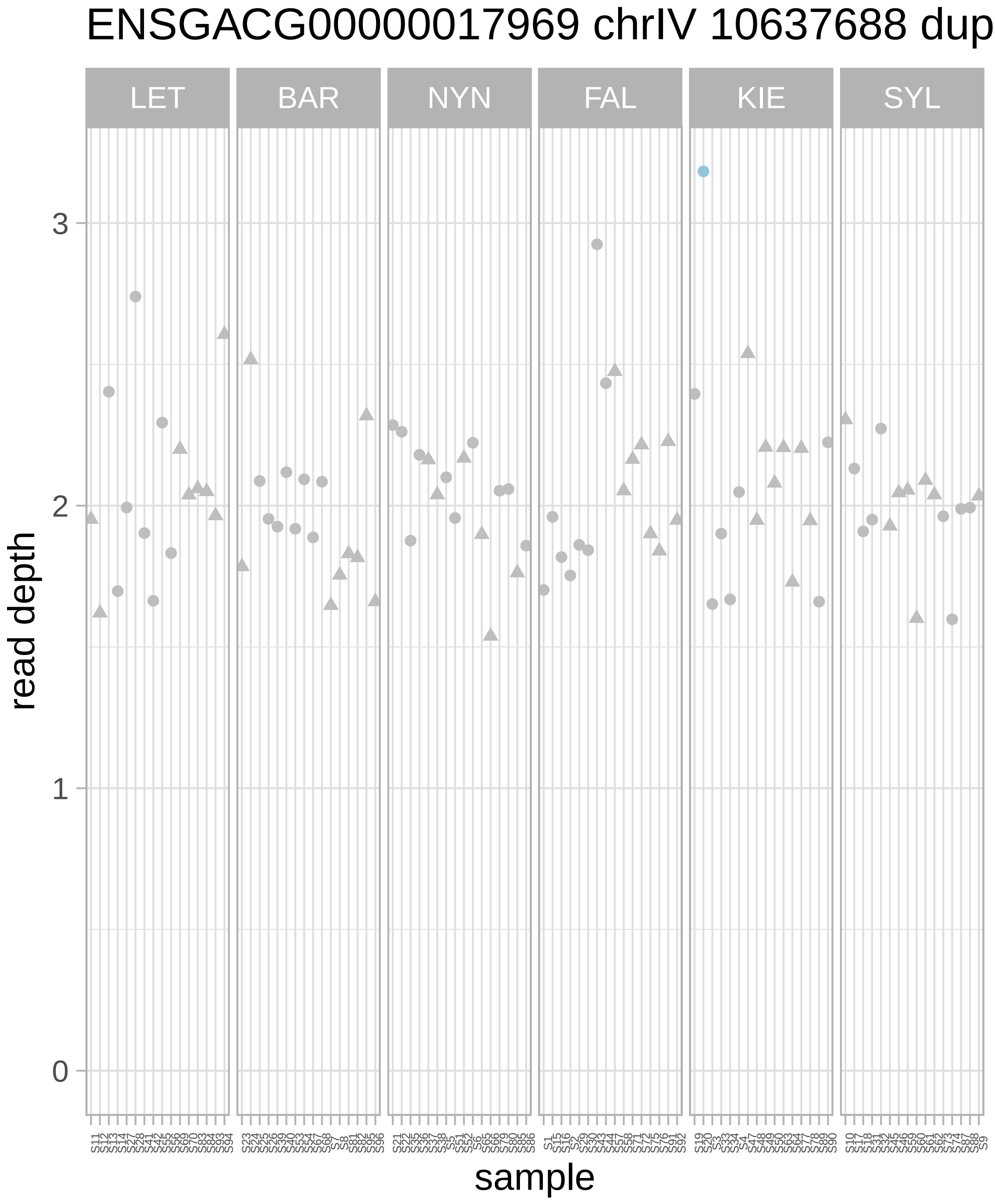

ENSGACG00000017969 chrIV 10637688 dup
LET
BAR
NYN
FAL
KIE
SYL
read depth
3
2
1
0
sample

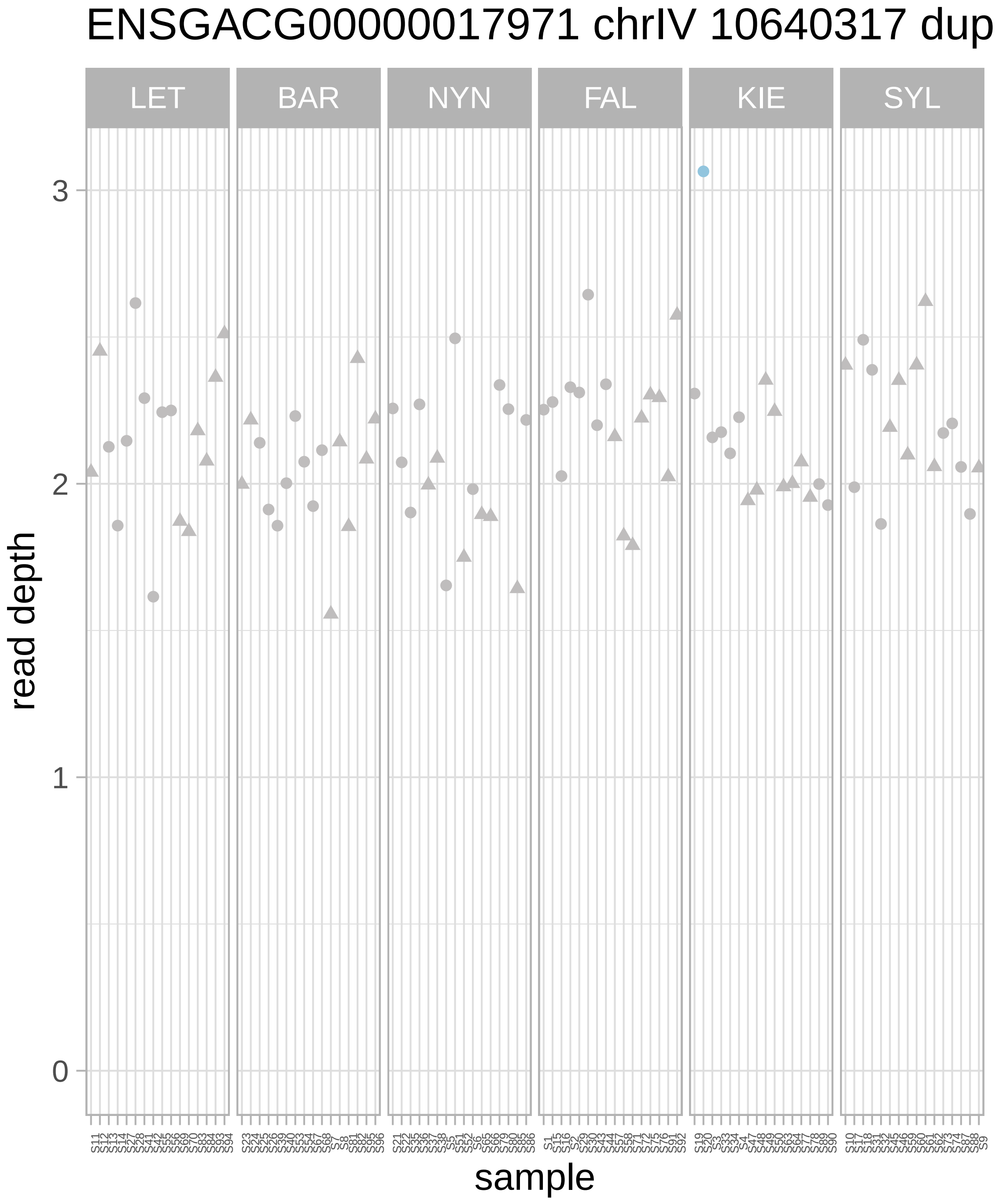
ENSGACG00000017971 chrIV 10640317 dup
LET
BAR
NYN
FAL
KIE
SYL
read depth
0
1
2
3
S11 S12 S13 S14 S27 S28 S41 S42 S55 S56 S69 S70 S83 S84 S93 S94
S23 S24 S25 S26 S39 S40 S53 S54 S67 S68 S7 S8 S81 S82 S95 S96
S21 S22 S35 S36 S37 S38 S5 S51 S52 S6 S65 S66 S79 S80 S85 S86
S1 S15 S16 S2 S29 S30 S43 S44 S57 S58 S71 S72 S75 S76 S91 S92
S19 S20 S3 S33 S34 S4 S47 S48 S49 S50 S63 S64 S77 S78 S89 S90
S10 S17 S18 S31 S32 S45 S46 S59 S60 S61 S62 S73 S74 S87 S88 S9
sample

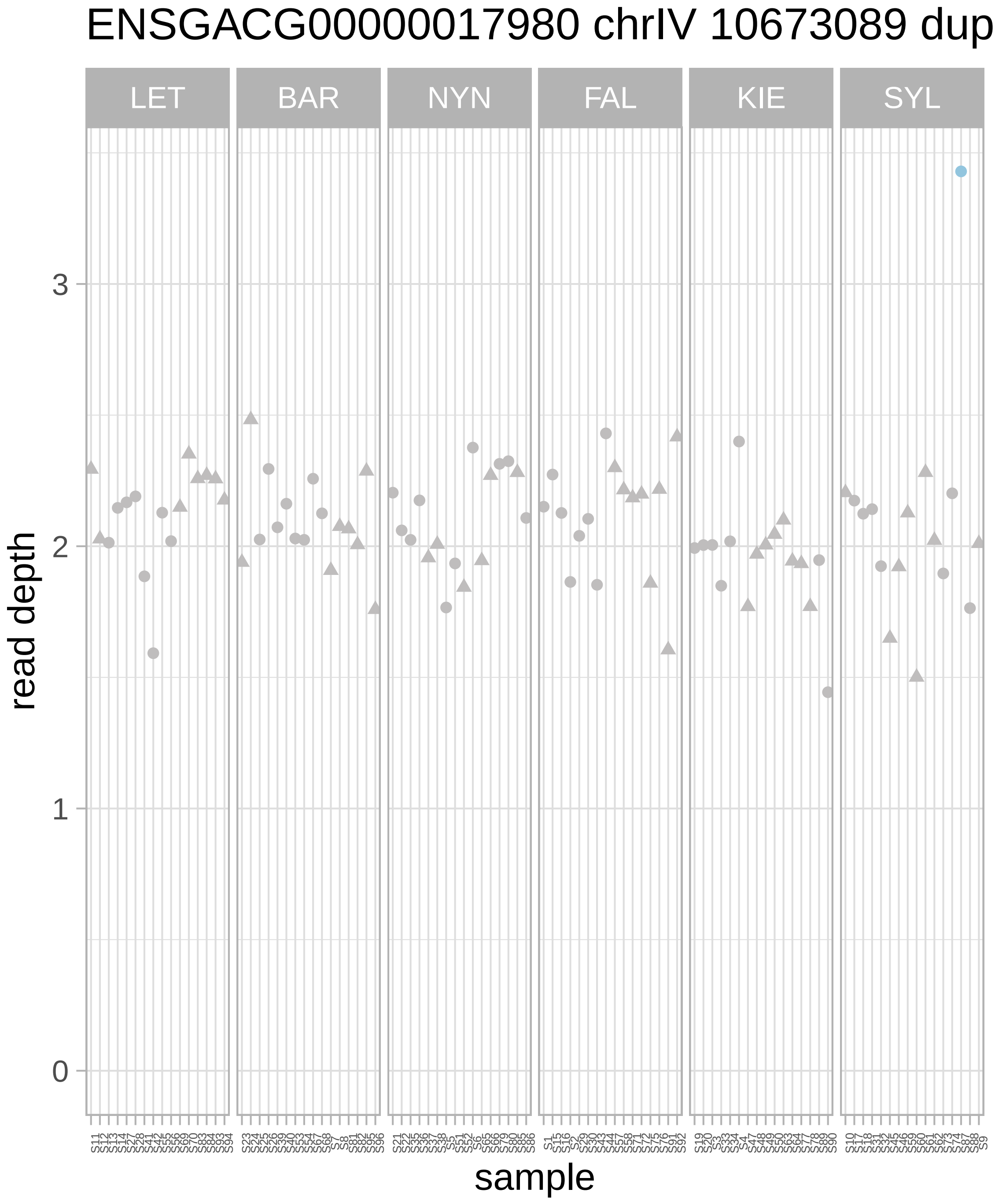
ENSGACG00000017980 chrIV 10673089 dup
LET
BAR
NYN
FAL
KIE
SYL
read depth
0
1
2
3
S11 S12 S13 S14 S27 S28 S41 S42 S55 S56 S69 S70 S83 S84 S93 S94
S23 S24 S25 S26 S39 S40 S53 S54 S67 S68 S7 S8 S81 S82 S95 S96
S21 S22 S35 S36 S37 S38 S5 S51 S52 S6 S65 S66 S79 S80 S85 S86
S1 S15 S16 S2 S29 S30 S43 S44 S57 S58 S71 S72 S75 S76 S91 S92
S19 S20 S3 S33 S34 S4 S47 S48 S49 S50 S63 S64 S77 S78 S89 S90
S10 S17 S18 S31 S32 S45 S46 S59 S60 S61 S62 S73 S74 S87 S88 S9
sample

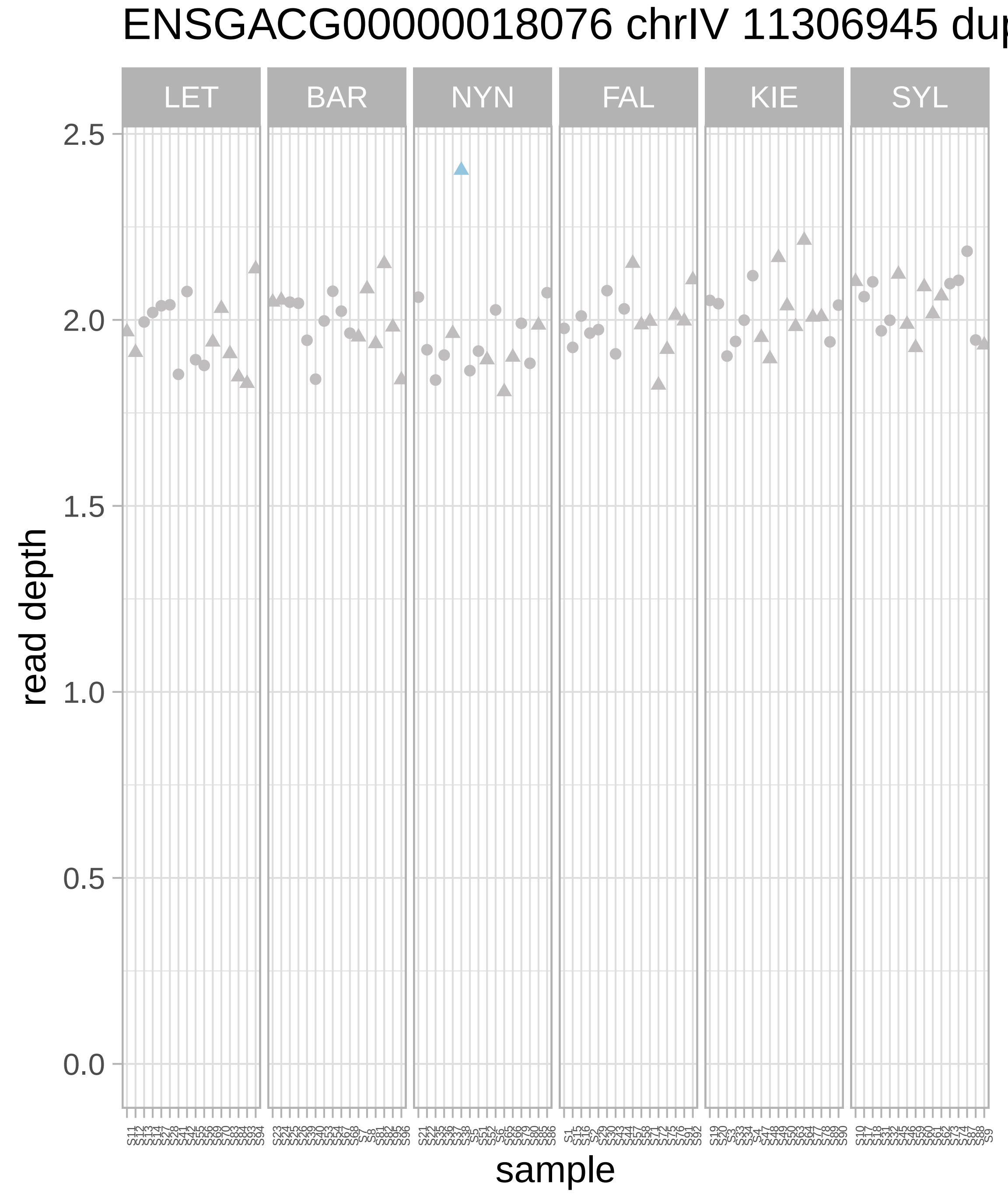
ENSGACG00000018076 chrIV 11306945 dup
LET
BAR
NYN
FAL
KIE
SYL
2.5
2.0
1.5
1.0
0.5
0.0
read depth
sample

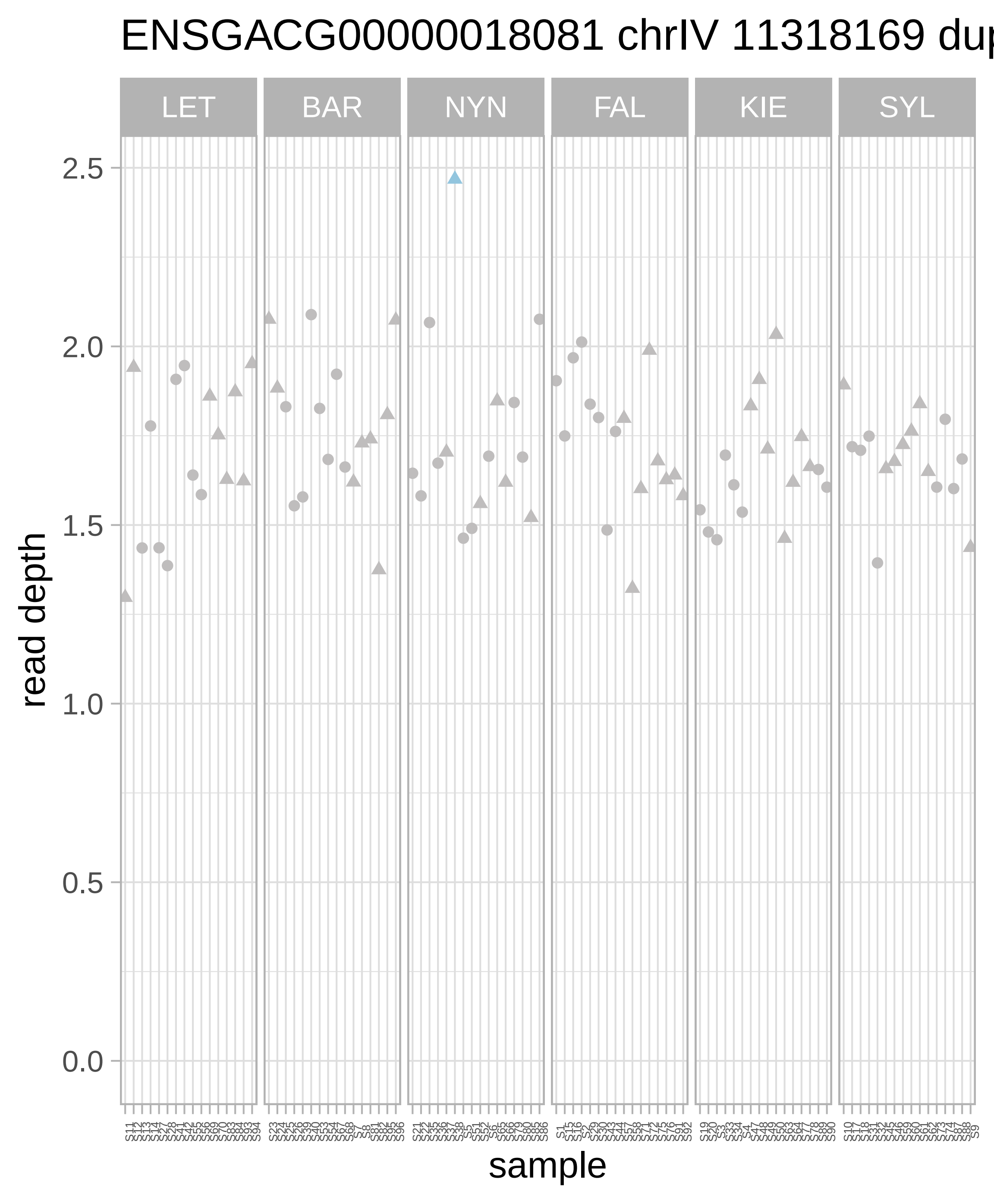

ENSGACG00000018081 chrIV 11318169 dup
LET
BAR
NYN
FAL
KIE
SYL
read depth
0.0
0.5
1.0
1.5
2.0
2.5
sample

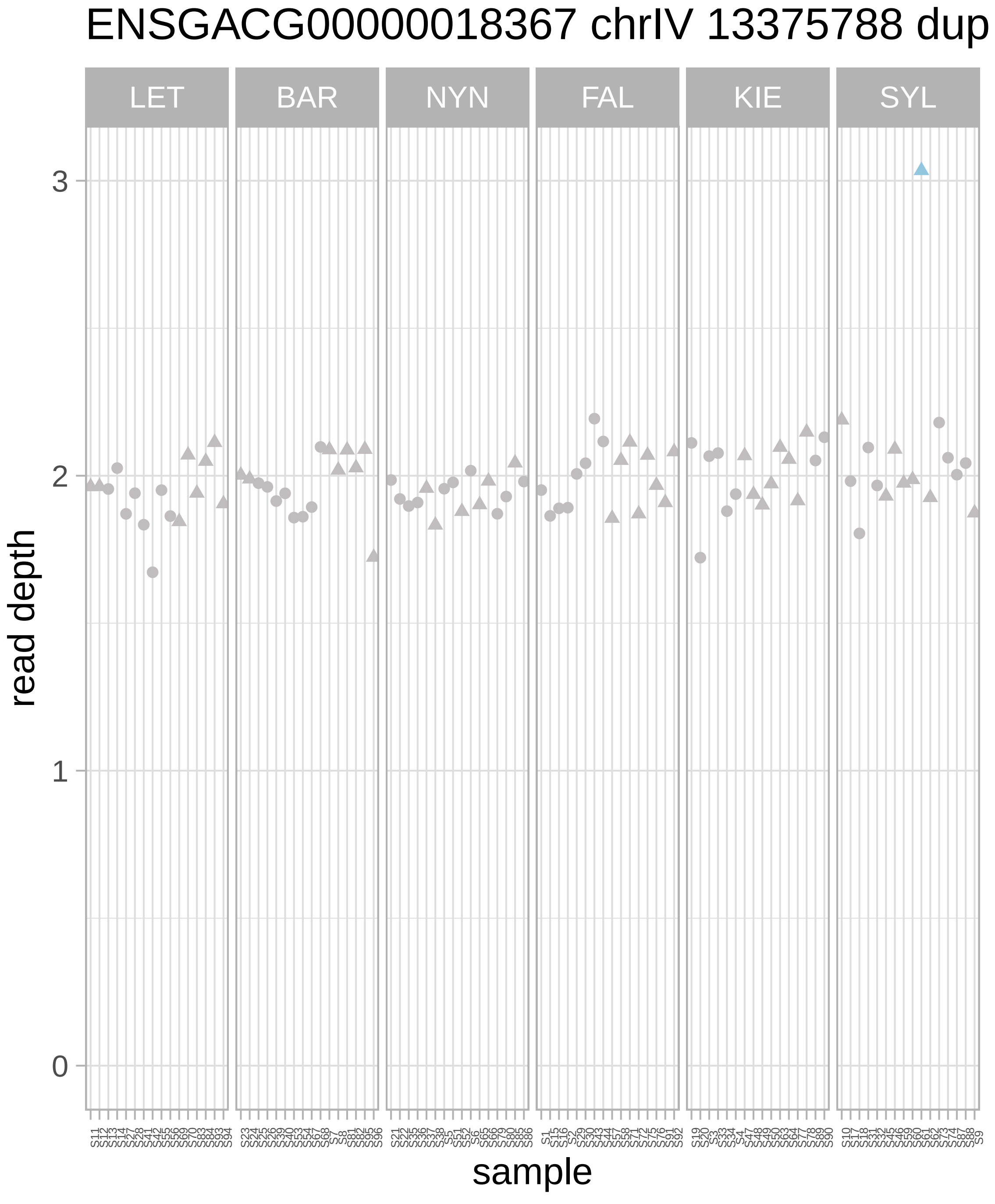
ENSGACG00000018367 chrIV 13375788 dup
LET
BAR
NYN
FAL
KIE
SYL
read depth
0
1
2
3
sample

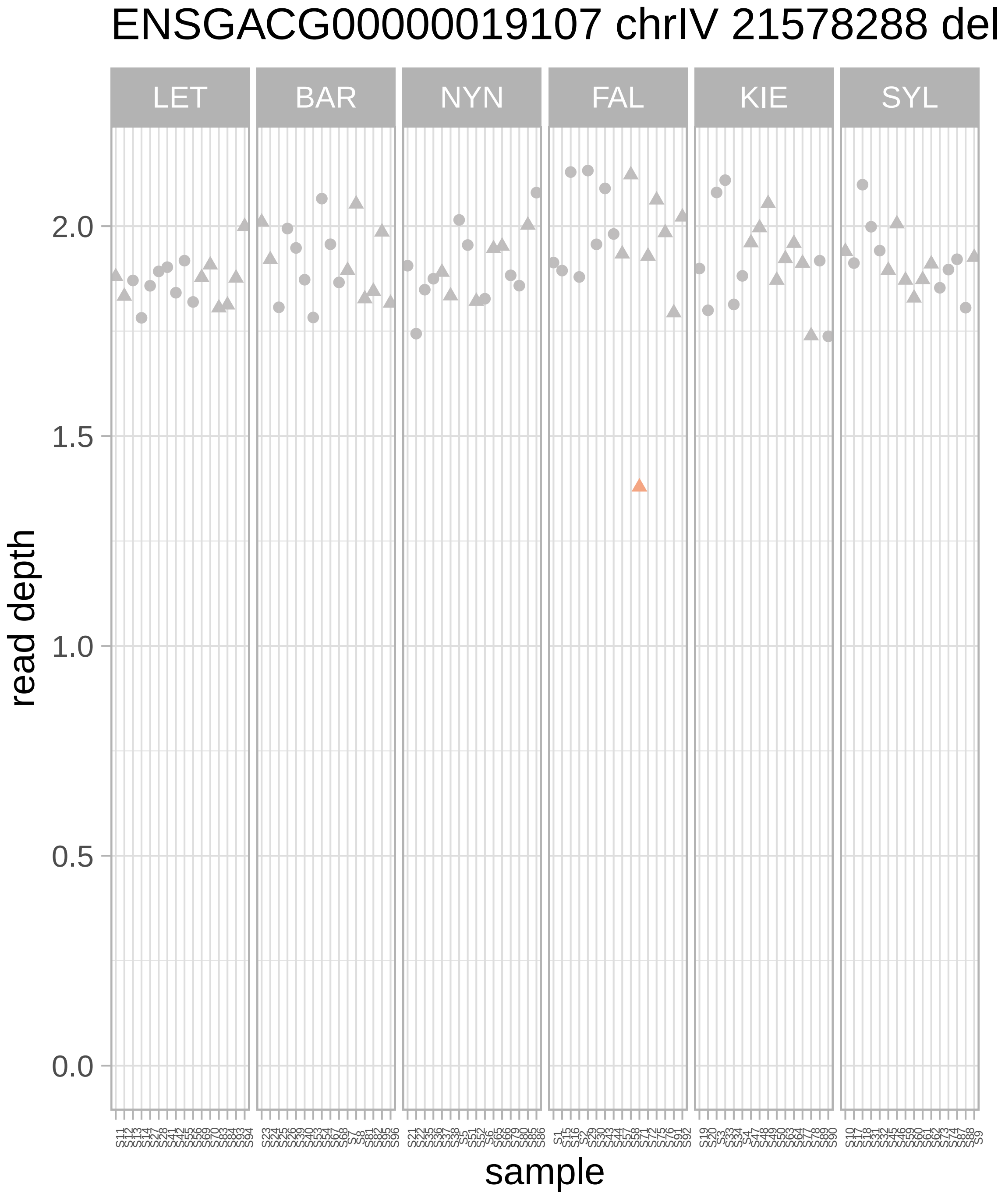
ENSGACG00000019107 chrIV 21578288 del
LET
BAR
NYN
FAL
KIE
SYL
read depth
0.0
0.5
1.0
1.5
2.0
S11 S12 S13 S14 S27 S28 S41 S42 S55 S56 S69 S70 S83 S84 S93 S94
S23 S24 S25 S26 S39 S40 S53 S54 S67 S68 S7 S8 S81 S82 S95 S96
S21 S22 S35 S36 S37 S38 S5 S51 S52 S6 S65 S66 S79 S80 S85 S86
S1 S15 S16 S2 S29 S30 S43 S44 S57 S58 S71 S72 S75 S76 S91 S92
S19 S20 S3 S33 S34 S4 S47 S48 S49 S50 S63 S64 S77 S78 S89 S90
S10 S17 S18 S31 S32 S45 S46 S59 S60 S61 S62 S73 S74 S87 S88 S9
sample

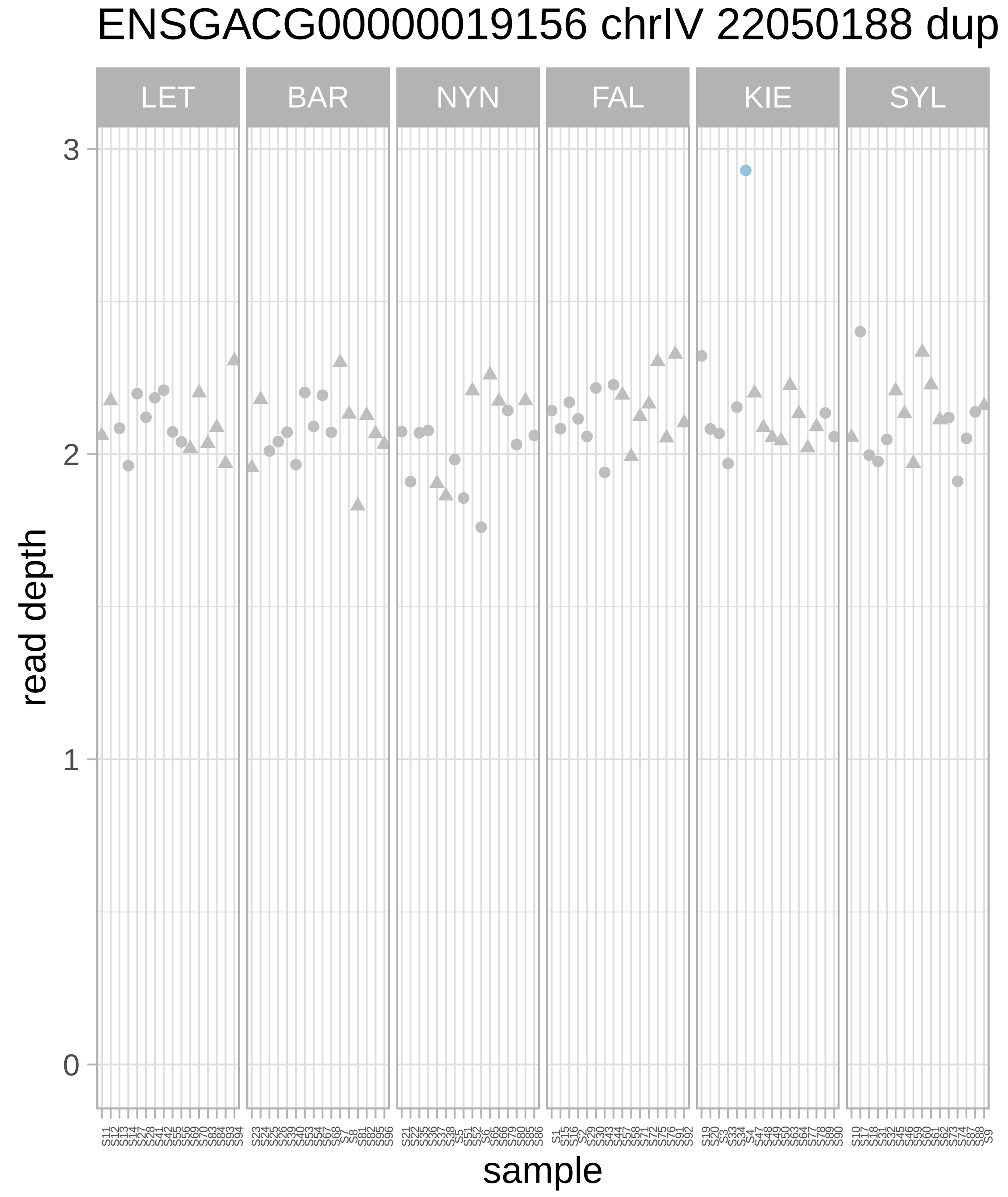
ENSGACG00000019156 chrIV 22050188 dup
LET
BAR
NYN
FAL
KIE
SYL
3
2
1
0
read depth
sample

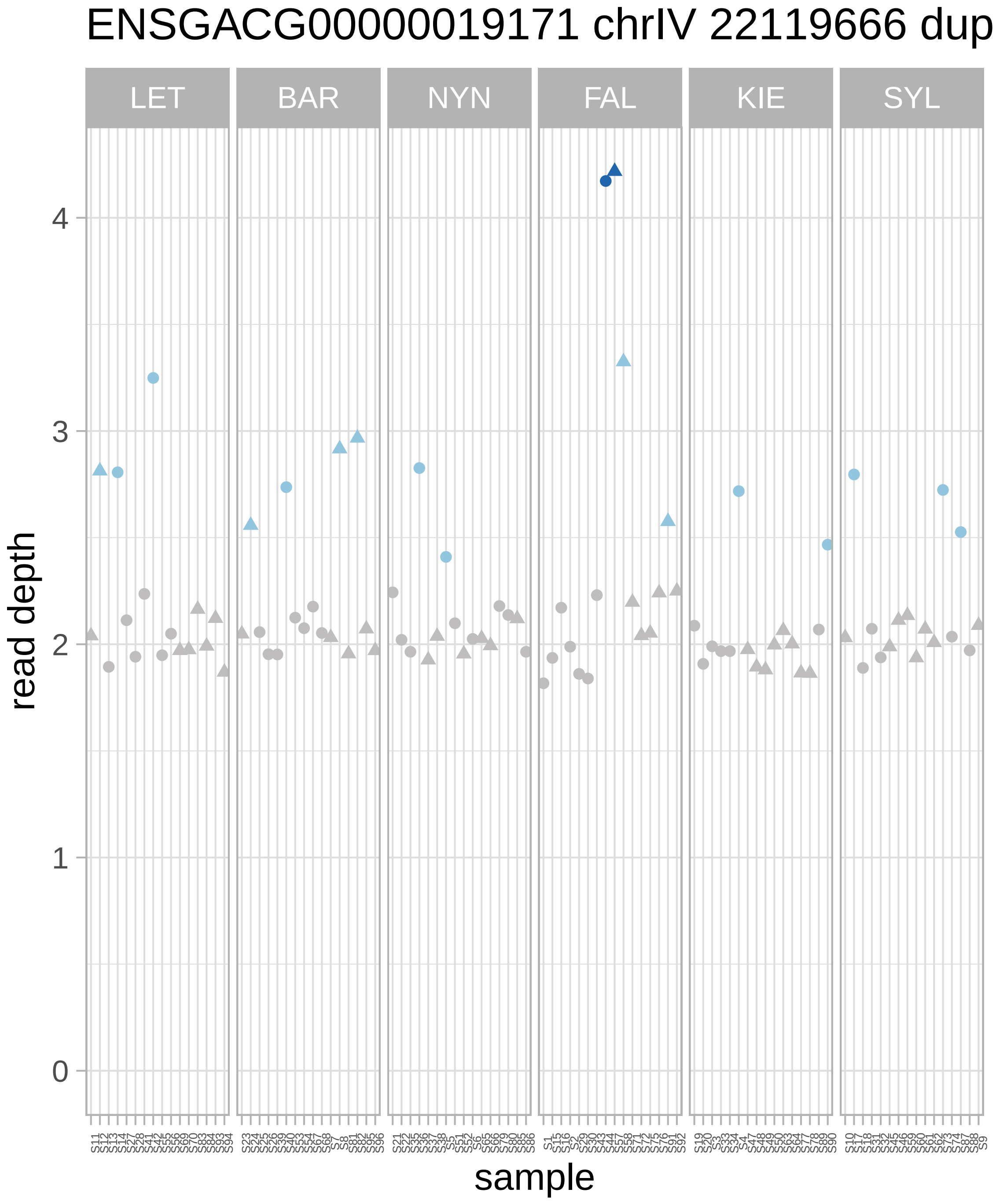
ENSGACG00000019171 chrIV 22119666 dup
LET
BAR
NYN
FAL
KIE
SYL
read depth
0
1
2
3
4
S11 S12 S13 S14 S27 S28 S41 S42 S55 S56 S69 S70 S83 S84 S93 S94
S23 S24 S25 S26 S39 S40 S53 S54 S67 S68 S7 S8 S81 S82 S95 S96
S21 S22 S35 S36 S37 S38 S5 S51 S52 S6 S65 S66 S79 S80 S85 S86
S1 S15 S16 S2 S29 S30 S43 S44 S57 S58 S71 S72 S75 S76 S91 S92
S19 S20 S3 S33 S34 S4 S47 S48 S49 S50 S63 S64 S77 S78 S89 S90
S10 S17 S18 S31 S32 S45 S46 S59 S60 S61 S62 S73 S74 S87 S88 S9
sample

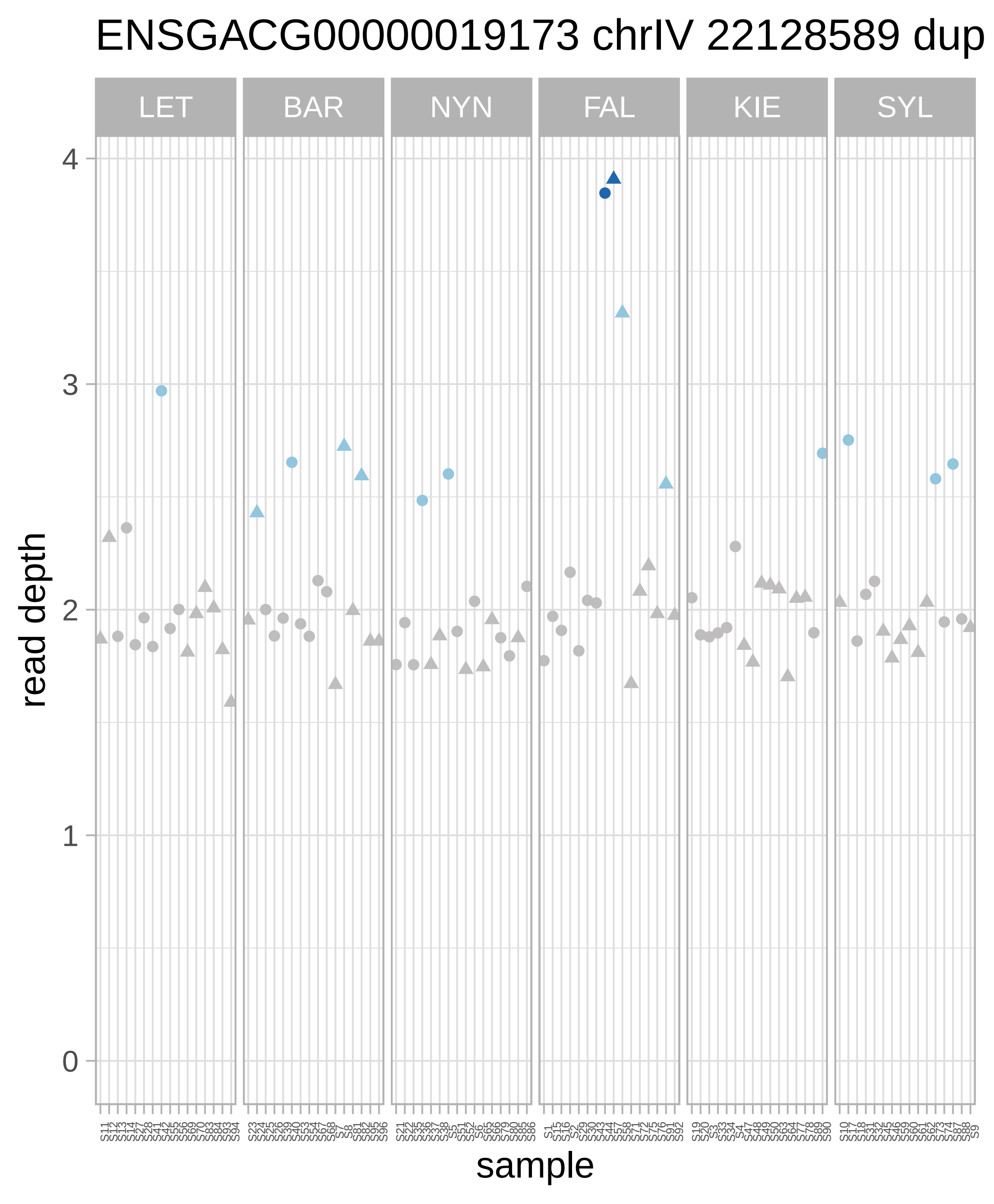
ENSGACG00000019173 chrIV 22128589 dup
LET
BAR
NYN
FAL
KIE
SYL
read depth
0
1
2
3
4
sample

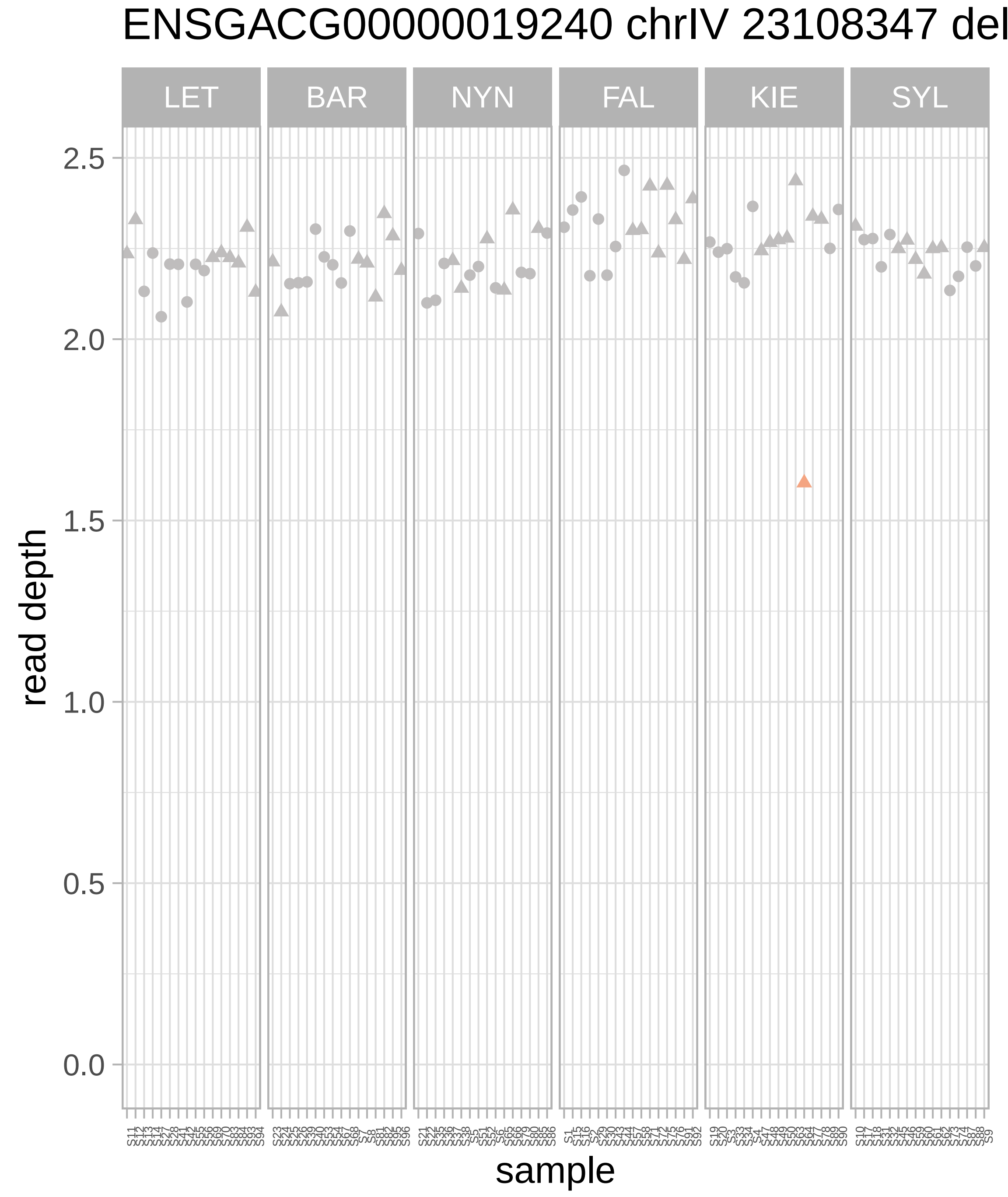

ENSGACG00000019240 chrIV 23108347 del
LET
BAR
NYN
FAL
KIE
SYL
read depth
2.5
2.0
1.5
1.0
0.5
0.0
S11 S12 S13 S14 S27 S28 S41 S42 S55 S56 S69 S70 S83 S84 S93 S94
S23 S24 S25 S26 S39 S40 S53 S54 S67 S68 S7 S8 S81 S82 S95 S96
S21 S22 S35 S36 S37 S38 S5 S51 S52 S6 S65 S66 S79 S80 S85 S86
S1 S15 S16 S2 S29 S30 S43 S44 S57 S58 S71 S72 S75 S76 S91 S92
S19 S20 S3 S33 S34 S4 S47 S48 S49 S50 S63 S64 S77 S78 S89 S90
S10 S17 S18 S31 S32 S45 S46 S59 S60 S61 S62 S73 S74 S87 S88 S9
sample

ENSGACG00000019292 chrIV 23828127 dup

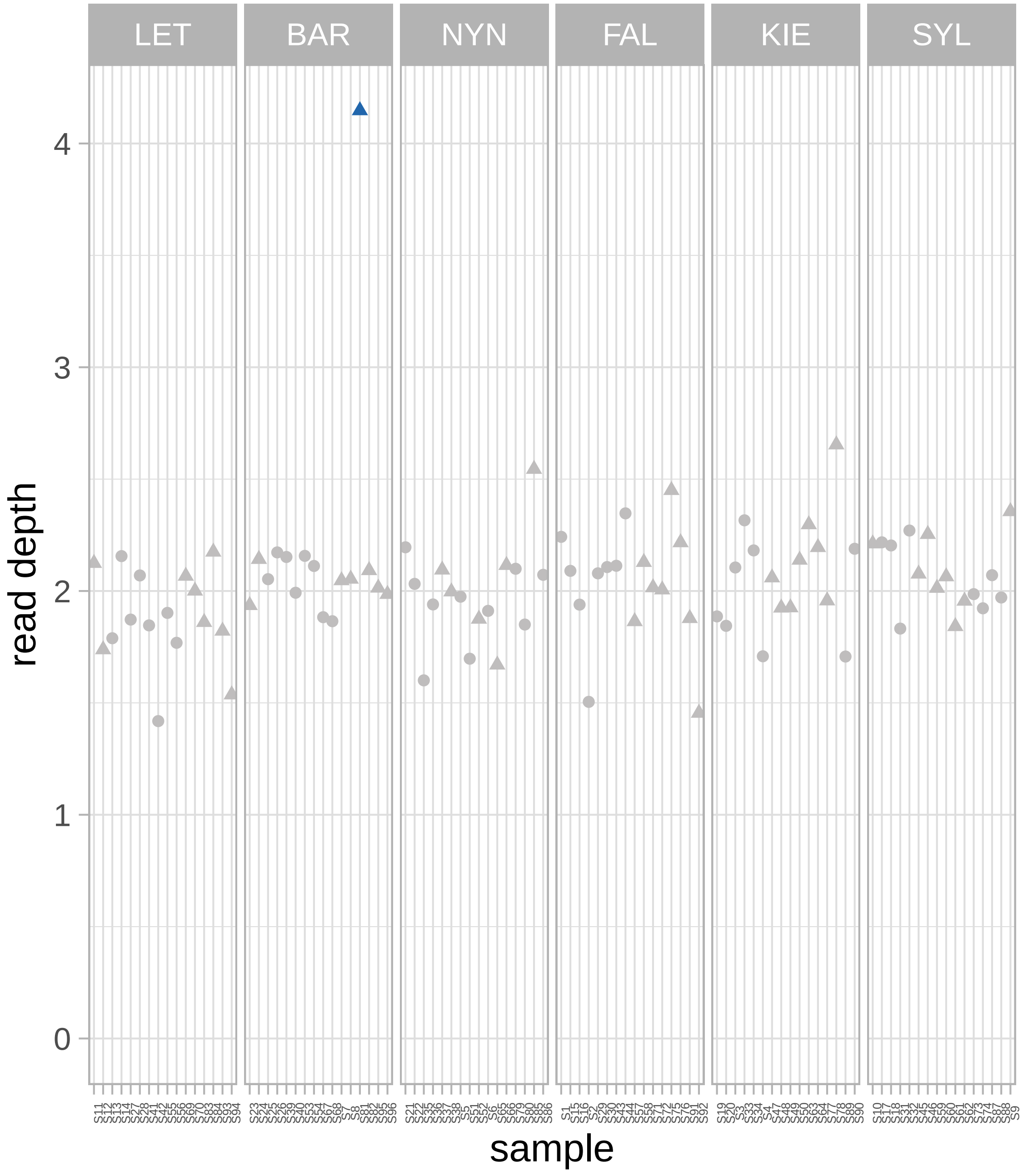

ENSGACG00000019373 chrIV 24621730 dup

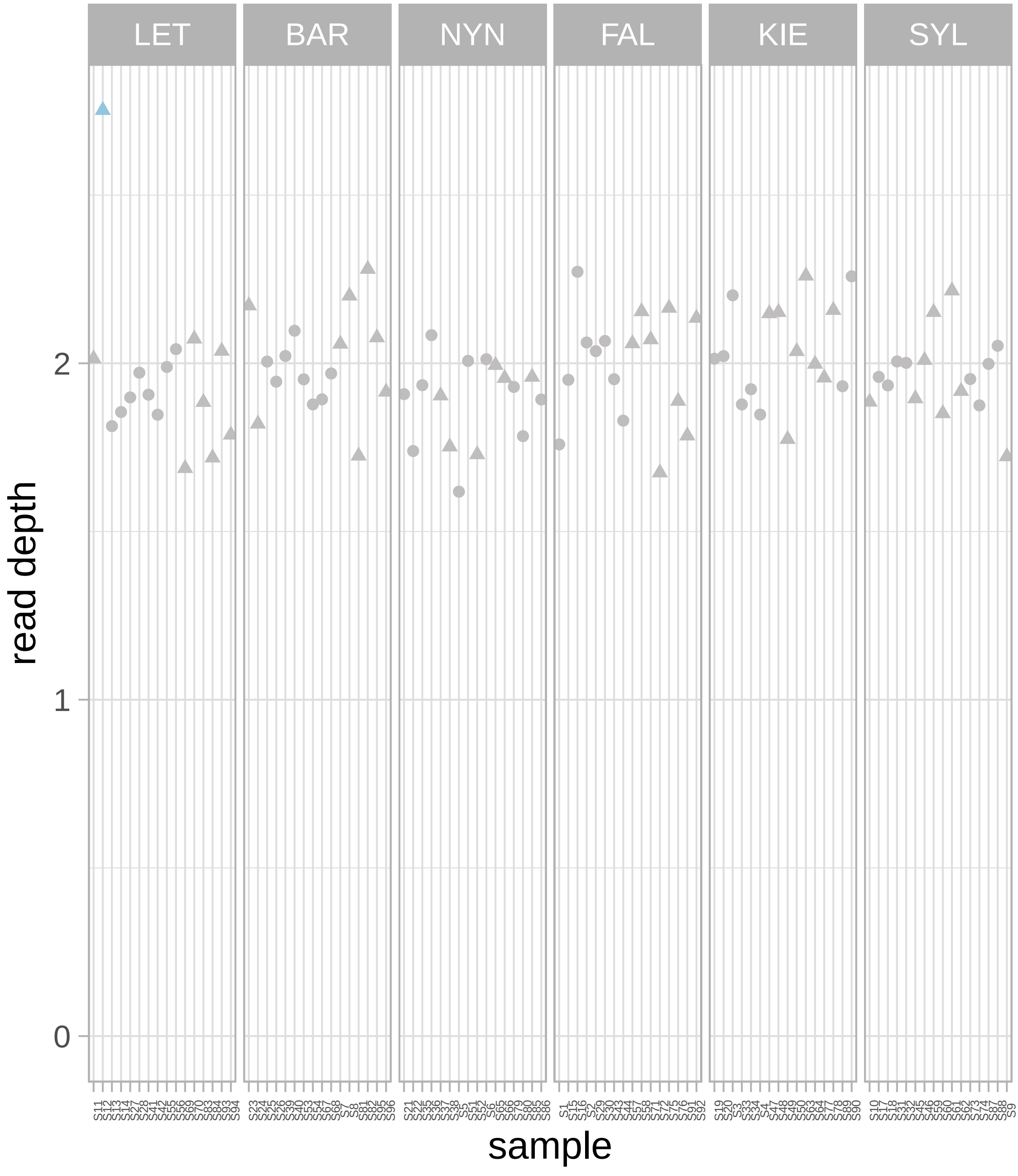

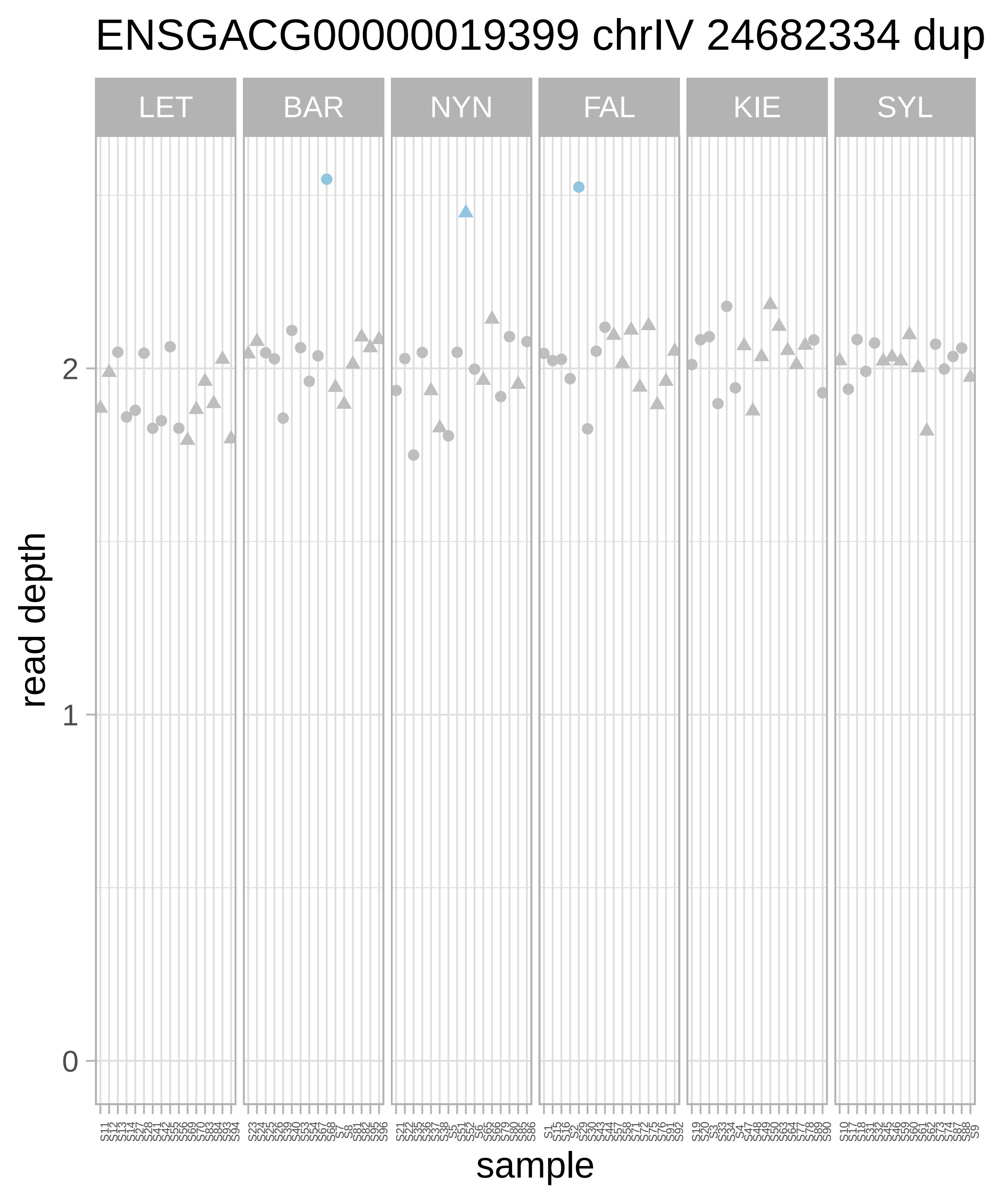
ENSGACG00000019399 chrIV 24682334 dup
LET
BAR
NYN
FAL
KIE
SYL
read depth
0
1
2
sample

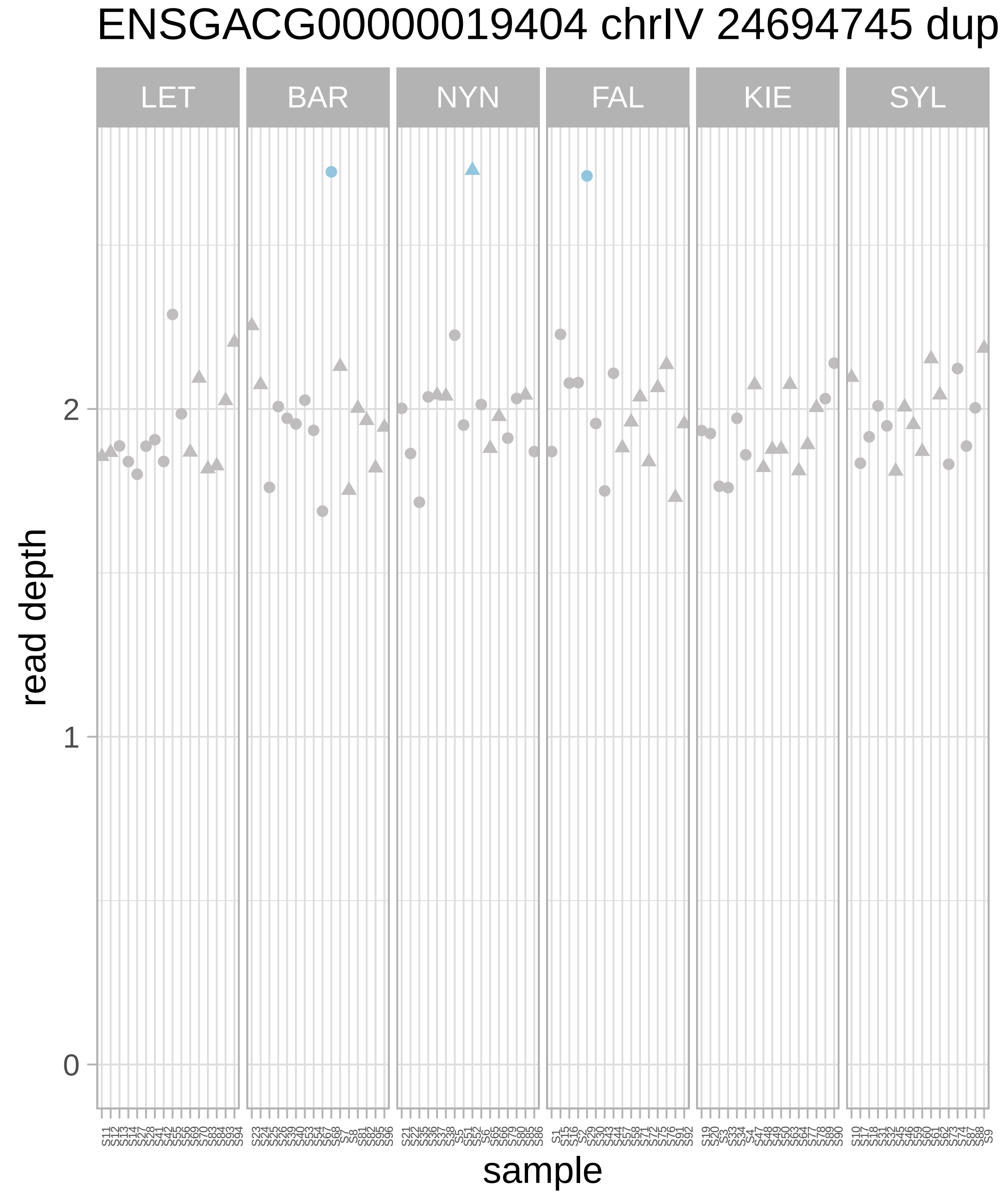
ENSGACG00000019404 chrIV 24694745 dup
LET
BAR
NYN
FAL
KIE
SYL
read depth
0
1
2
sample
S11 S12 S13 S14 S27 S28 S41 S42 S55 S56 S69 S70 S83 S84 S93 S94
S23 S24 S25 S26 S39 S40 S53 S54 S67 S68 S7 S8 S81 S82 S95 S96
S21 S22 S35 S36 S37 S38 S5 S51 S52 S6 S65 S66 S79 S80 S85 S86
S1 S15 S16 S2 S29 S30 S43 S44 S57 S58 S71 S72 S75 S76 S91 S92
S19 S20 S3 S33 S34 S4 S47 S48 S49 S50 S63 S64 S77 S78 S89 S90
S10 S17 S18 S31 S32 S45 S46 S59 S60 S61 S62 S73 S74 S87 S88 S9

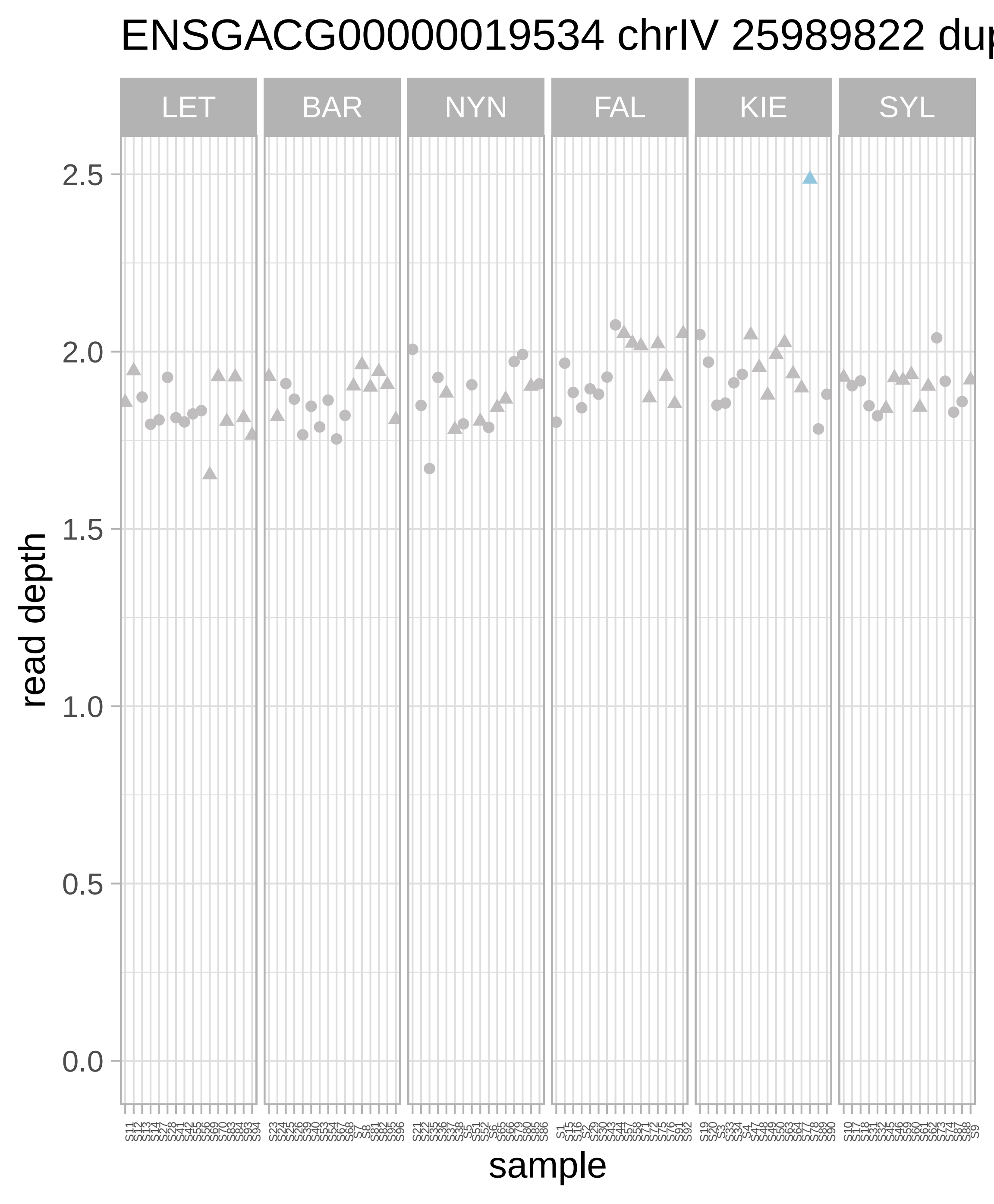
ENSGACG00000019534 chrIV 25989822 dup
LET
BAR
NYN
FAL
KIE
SYL
2.5
2.0
1.5
1.0
0.5
0.0
read depth
sample

ENSGACG00000019588 chrIV 26317519 dup

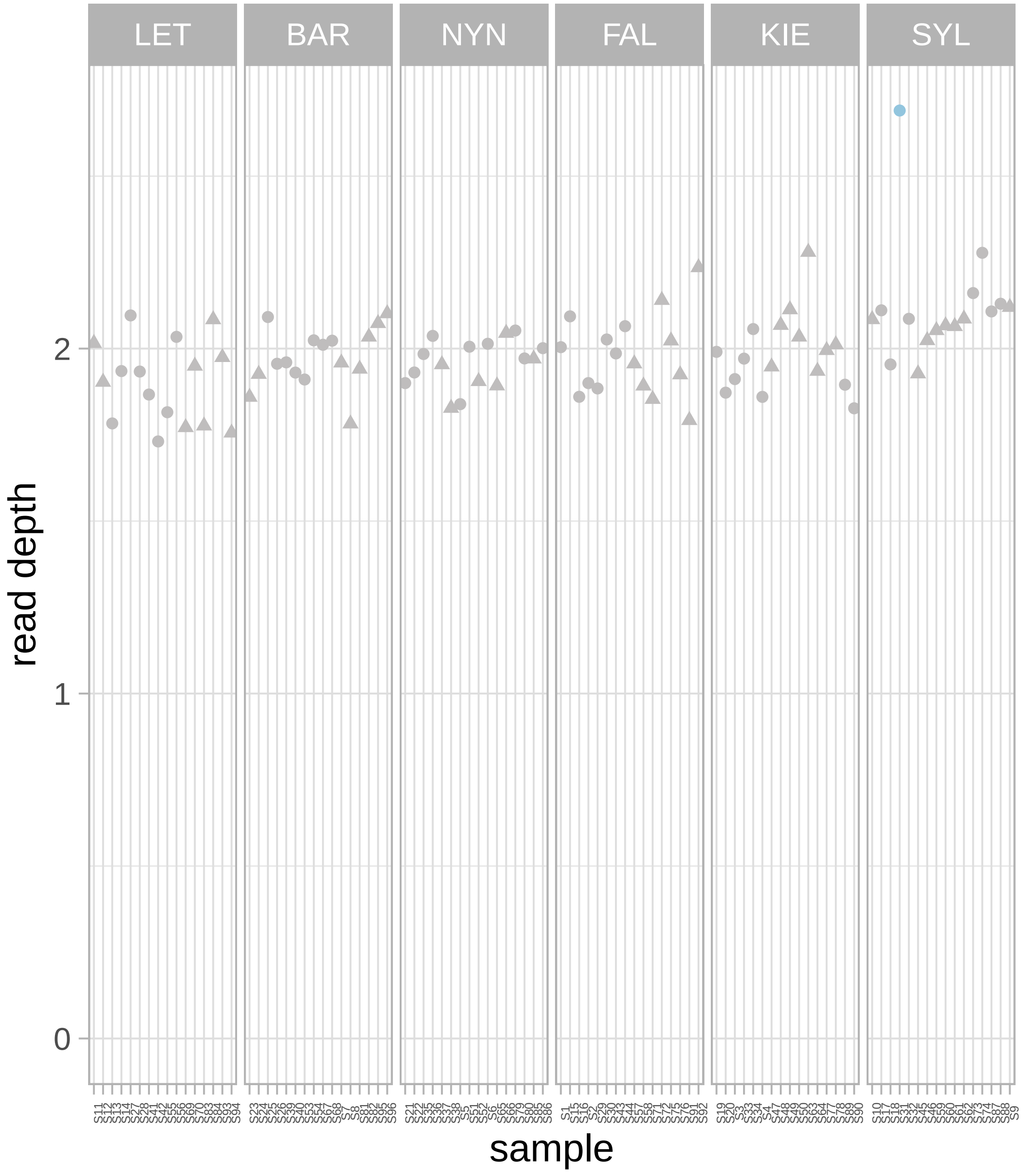

ENSGACG00000019621 chrIV 27574929 dup

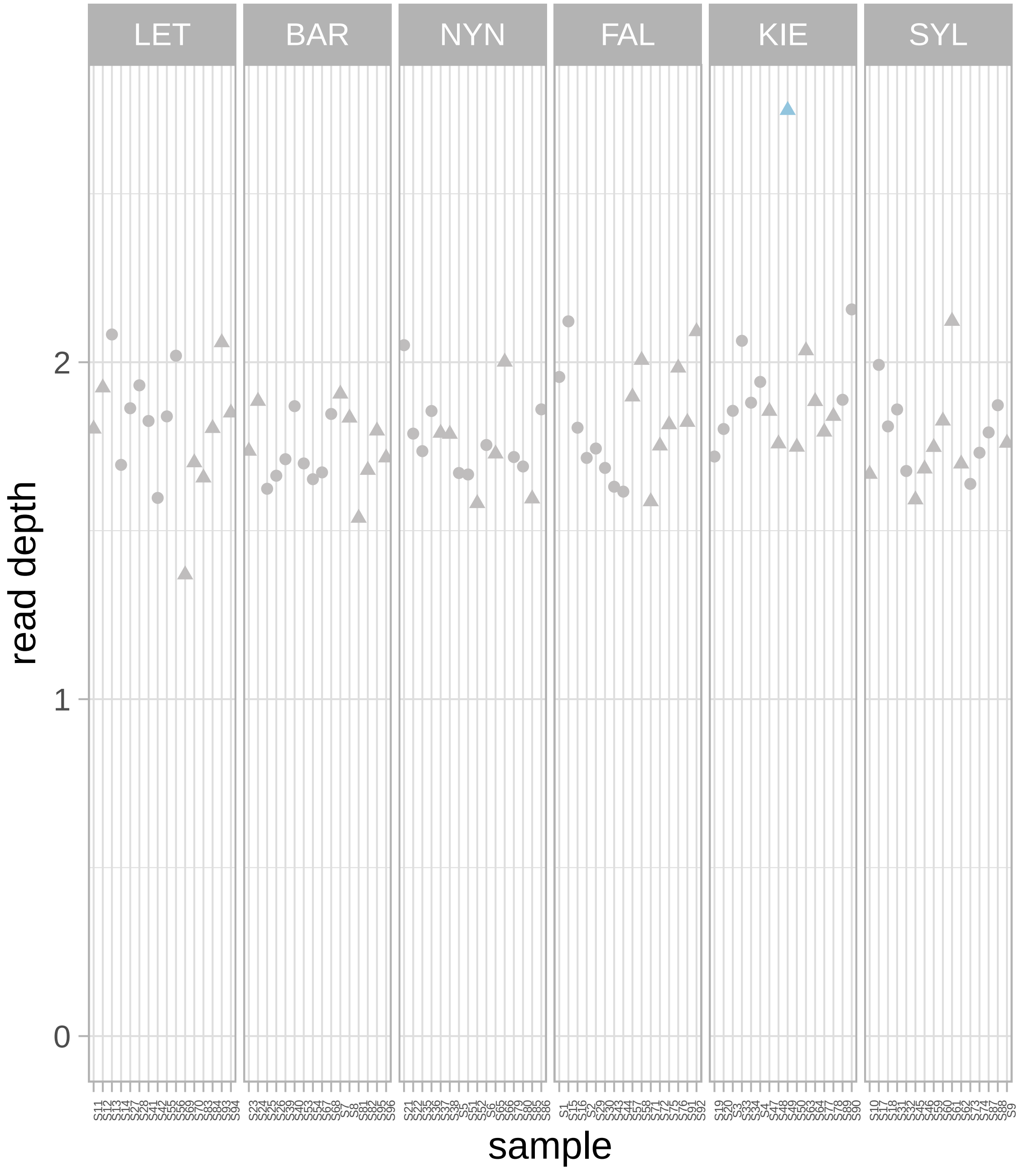

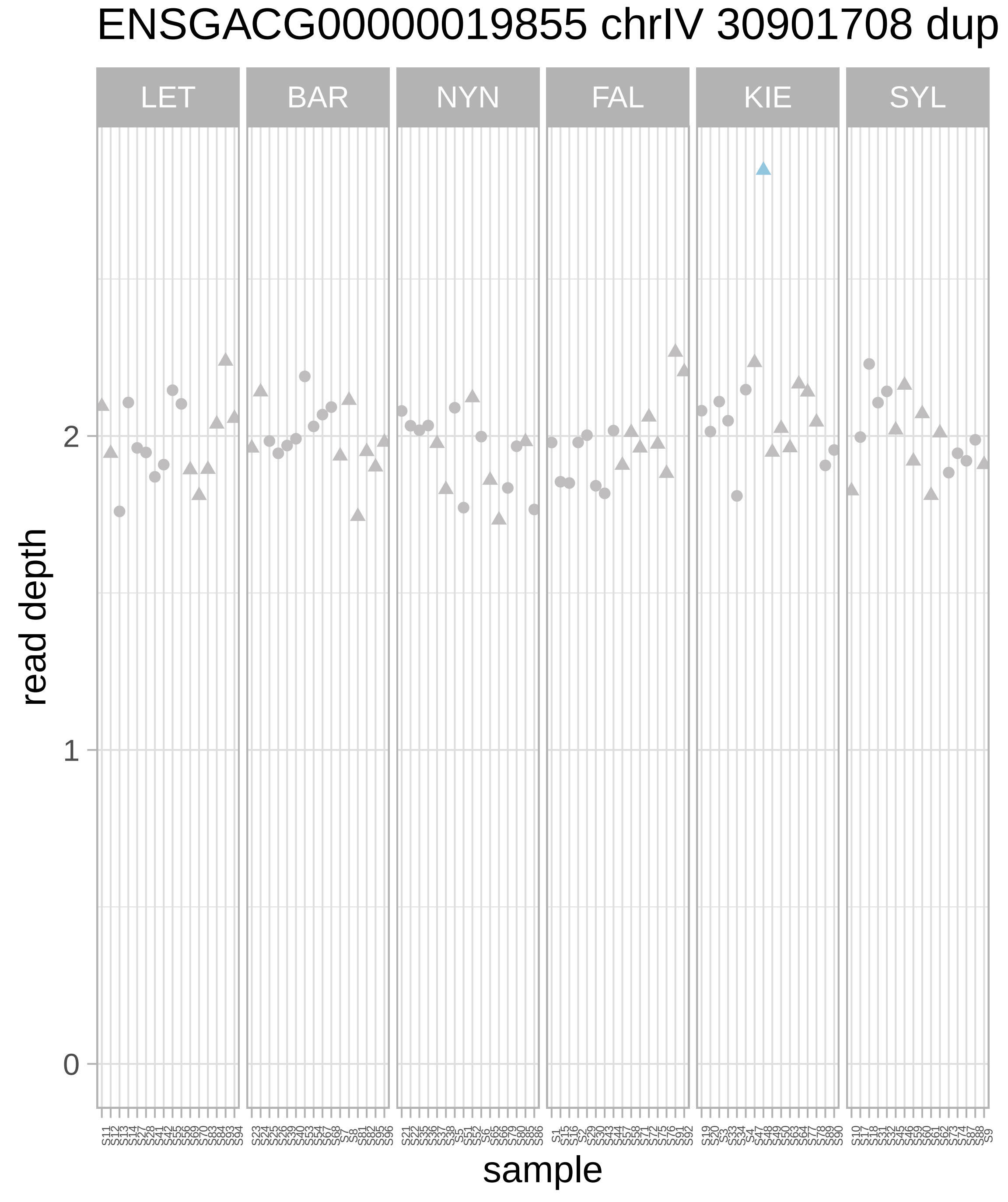
ENSGACG00000019855 chrIV 30901708 dup
LET
BAR
NYN
FAL
KIE
SYL
read depth
2
1
0
sample

ENSGACG00000019984 chrIV 31577732 dup

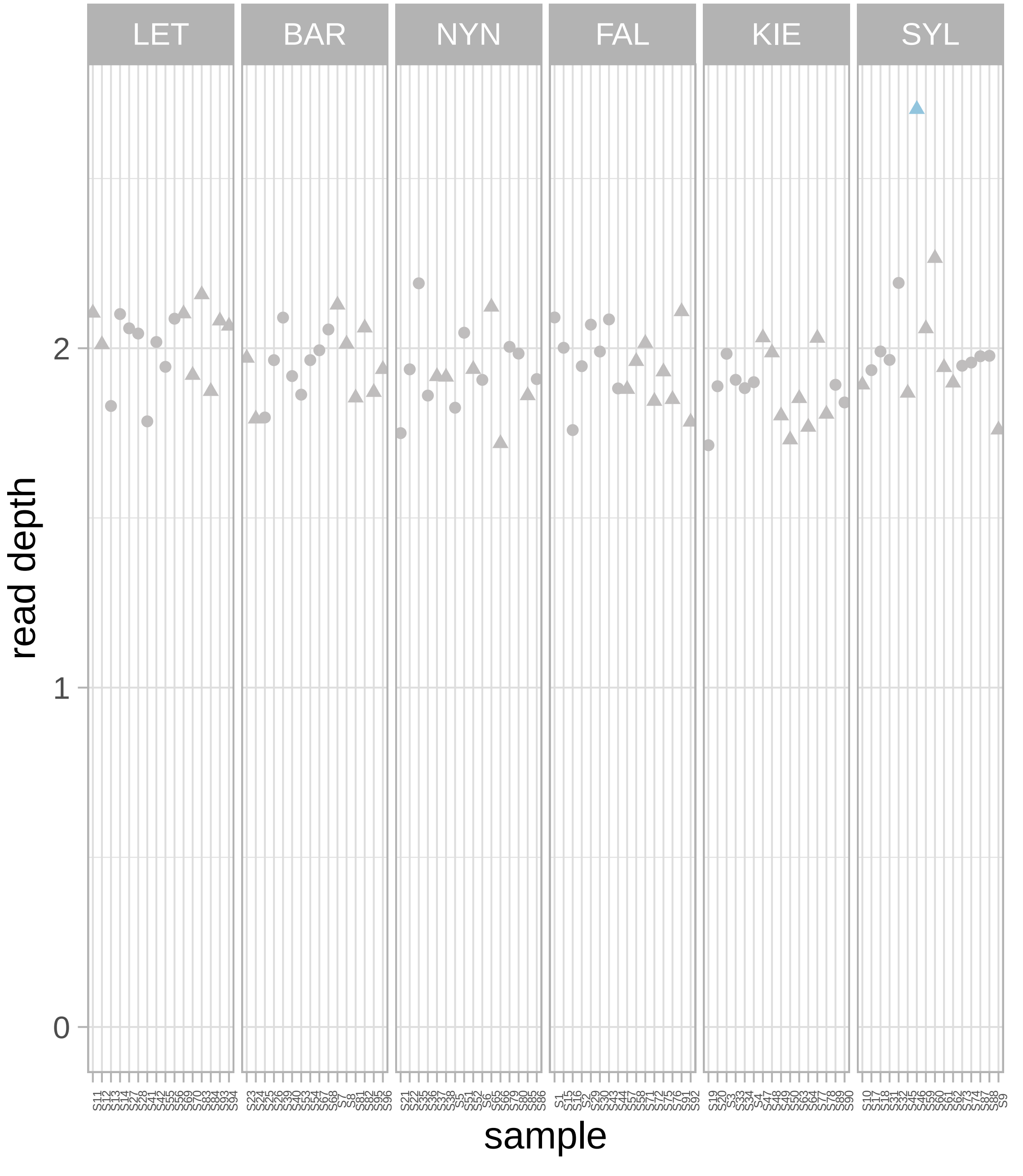

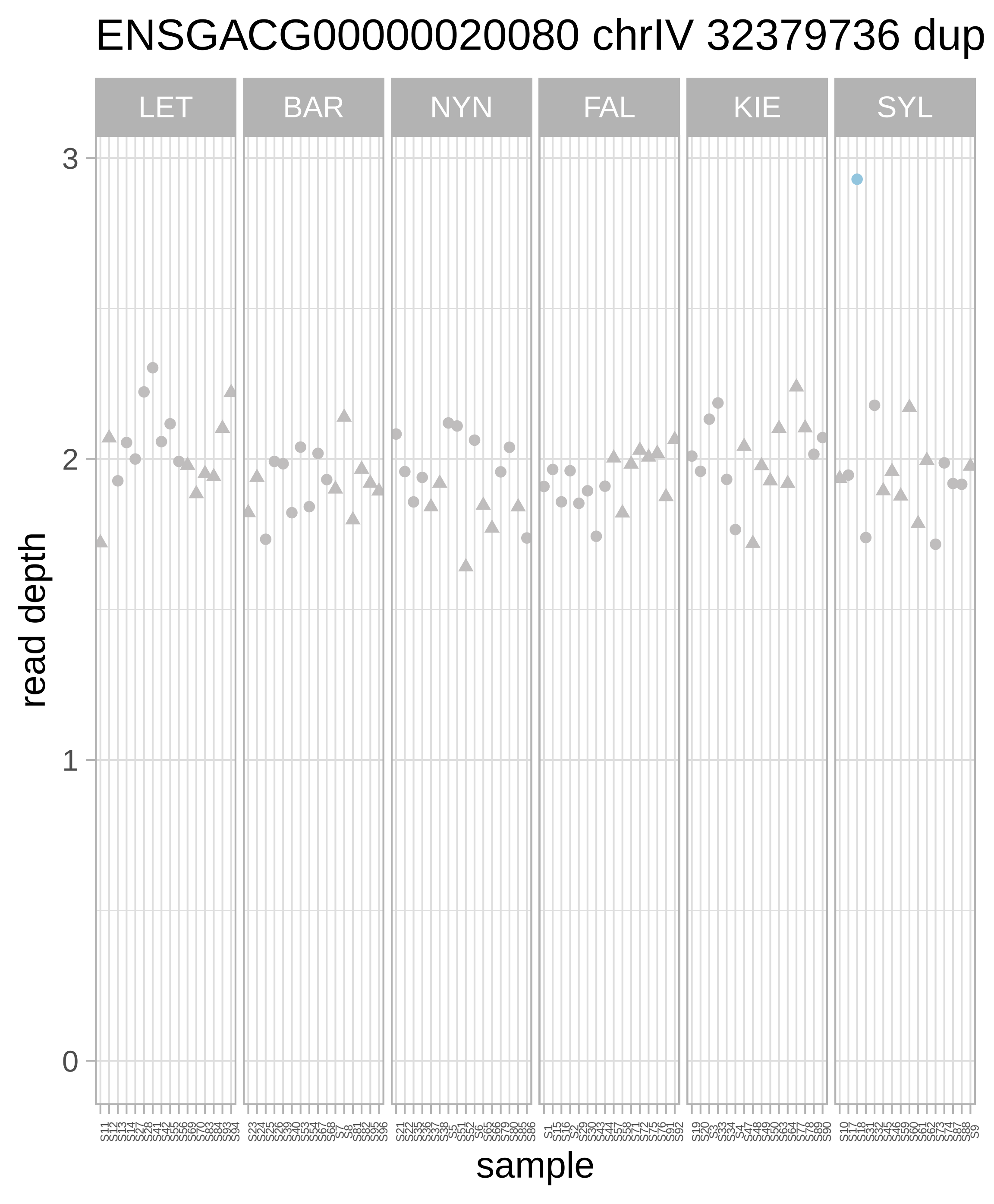
ENSGACG00000020080 chrIV 32379736 dup
LET
BAR
NYN
FAL
KIE
SYL
read depth
0
1
2
3
sample

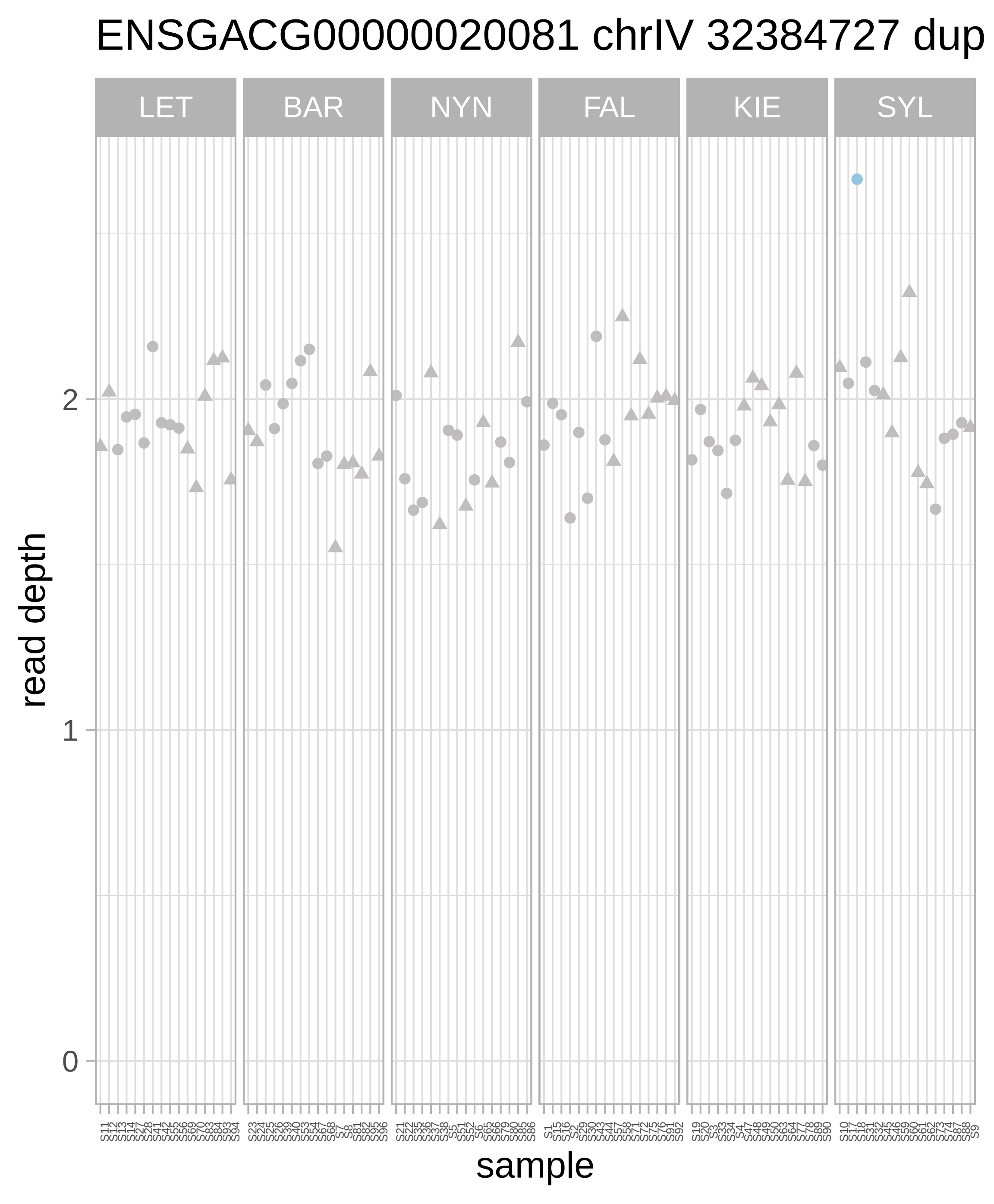
ENSGACG00000020081 chrIV 32384727 dup
LET
BAR
NYN
FAL
KIE
SYL
read depth
0
1
2
sample

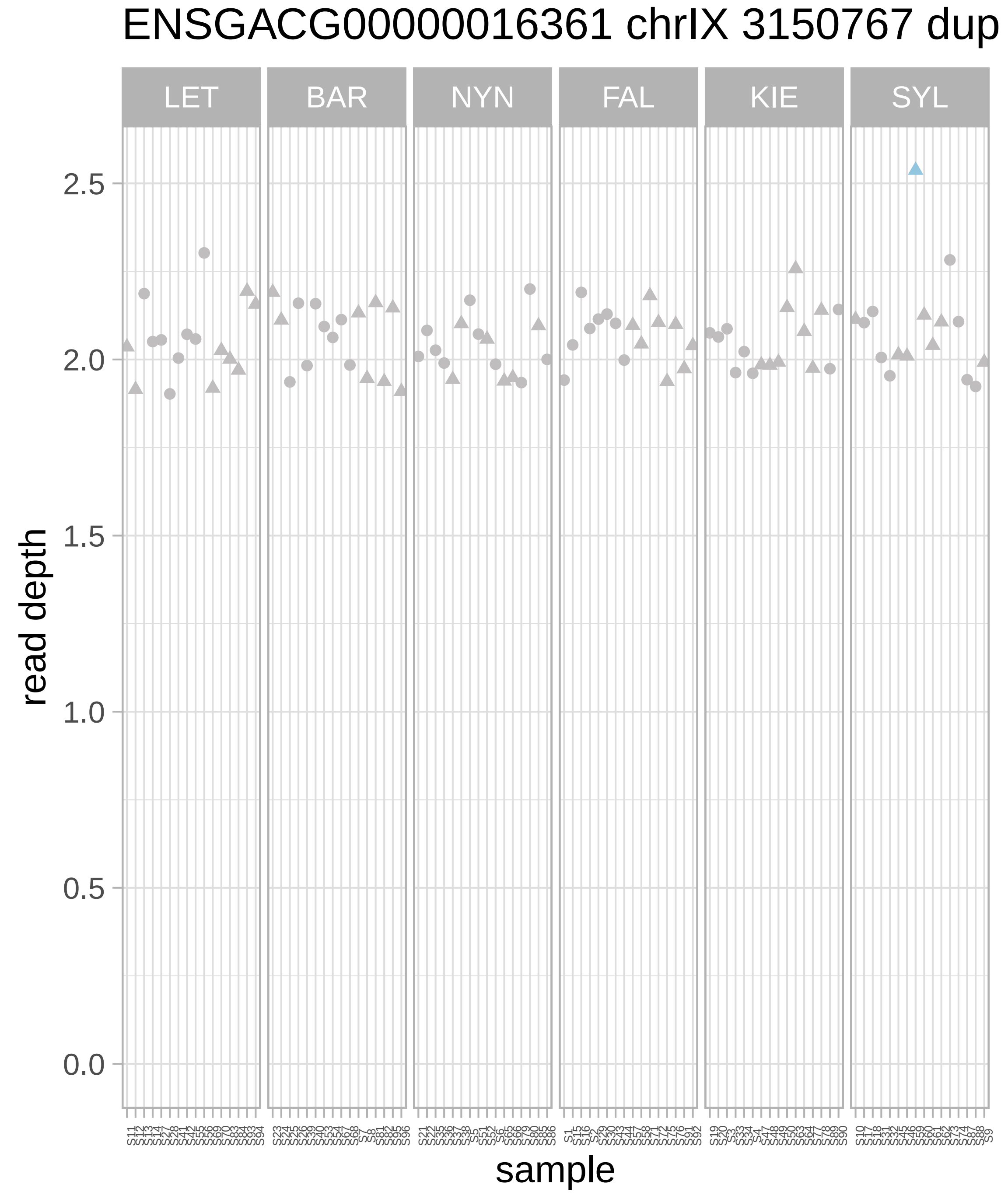

ENSGACG00000016361 chrIX 3150767 dup
LET
BAR
NYN
FAL
KIE
SYL
read depth
2.5
2.0
1.5
1.0
0.5
0.0
sample

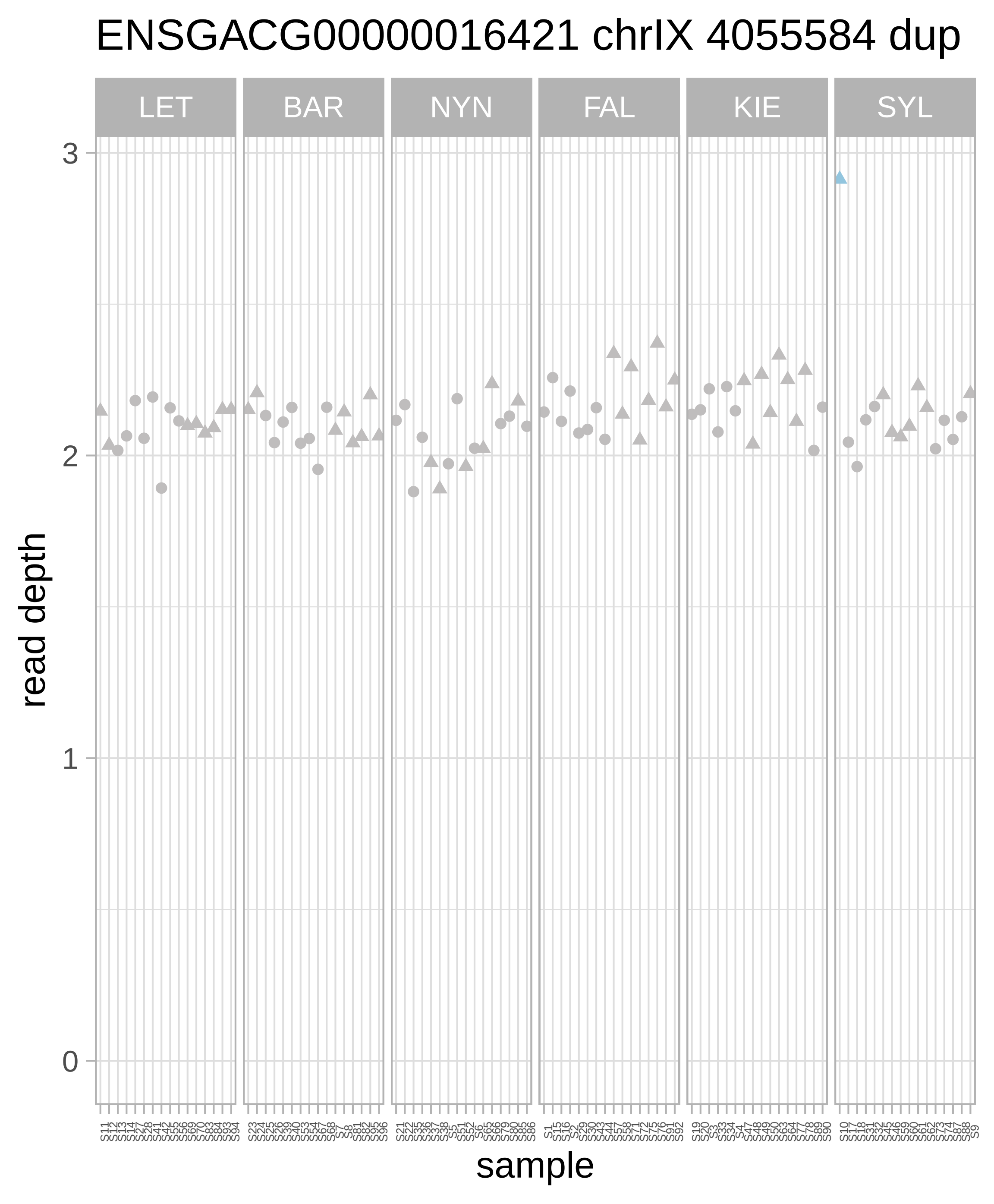
ENSGACG00000016421 chrIX 4055584 dup
LET
BAR
NYN
FAL
KIE
SYL
read depth
sample
0
1
2
3

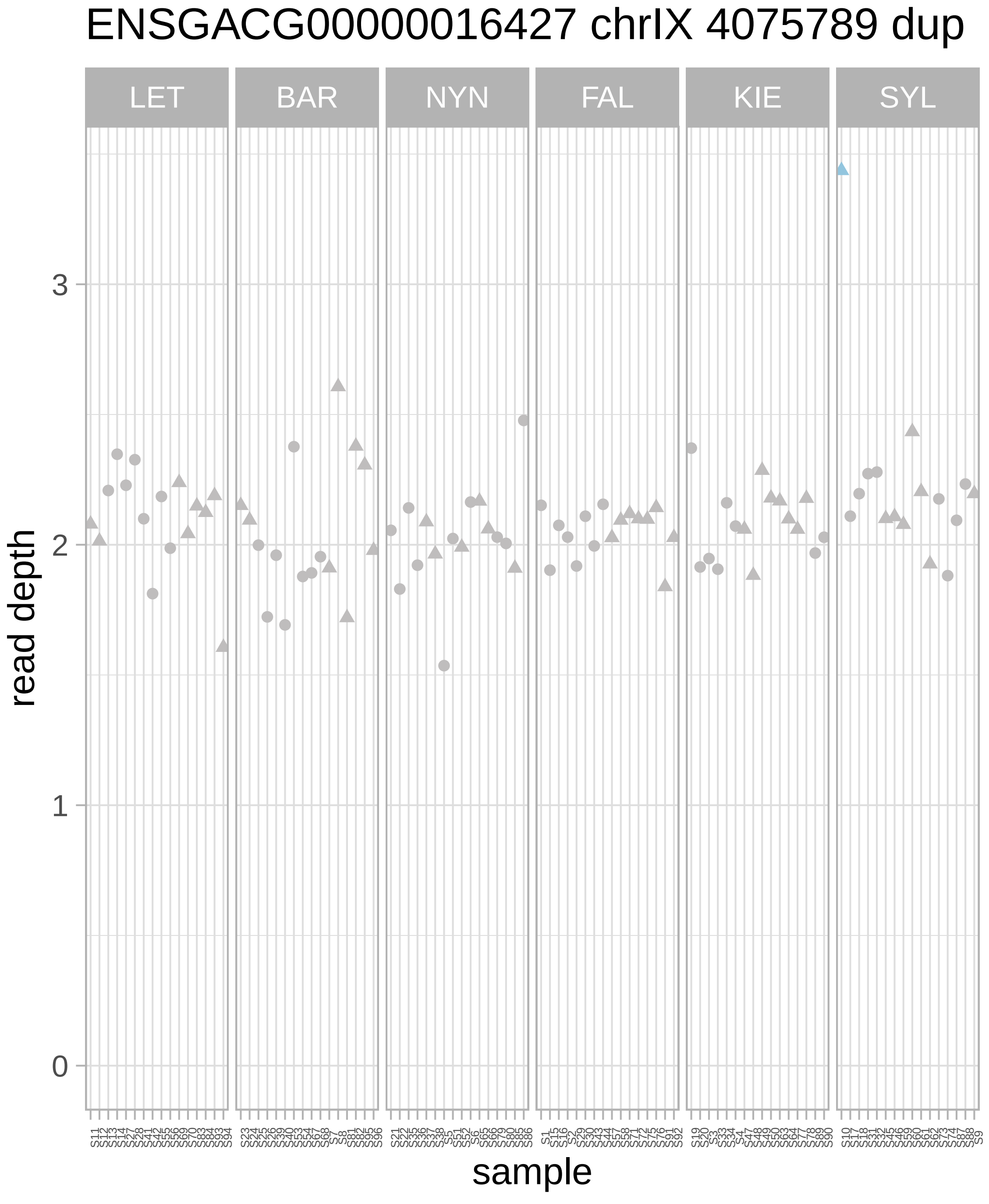
ENSGACG00000016427 chrIX 4075789 dup
LET
BAR
NYN
FAL
KIE
SYL
read depth
0
1
2
3
sample
S11 S12 S13 S14 S27 S28 S41 S42 S55 S56 S69 S70 S83 S84 S93 S94
S23 S24 S25 S26 S39 S40 S53 S54 S67 S68 S7 S8 S81 S82 S95 S96
S21 S22 S35 S36 S37 S38 S5 S51 S52 S6 S65 S66 S79 S80 S85 S86
S1 S15 S16 S2 S29 S30 S43 S44 S57 S58 S71 S72 S75 S76 S91 S92
S19 S20 S3 S33 S34 S4 S47 S48 S49 S50 S63 S64 S77 S78 S89 S90
S10 S17 S18 S31 S32 S45 S46 S59 S60 S61 S62 S73 S74 S87 S88 S9

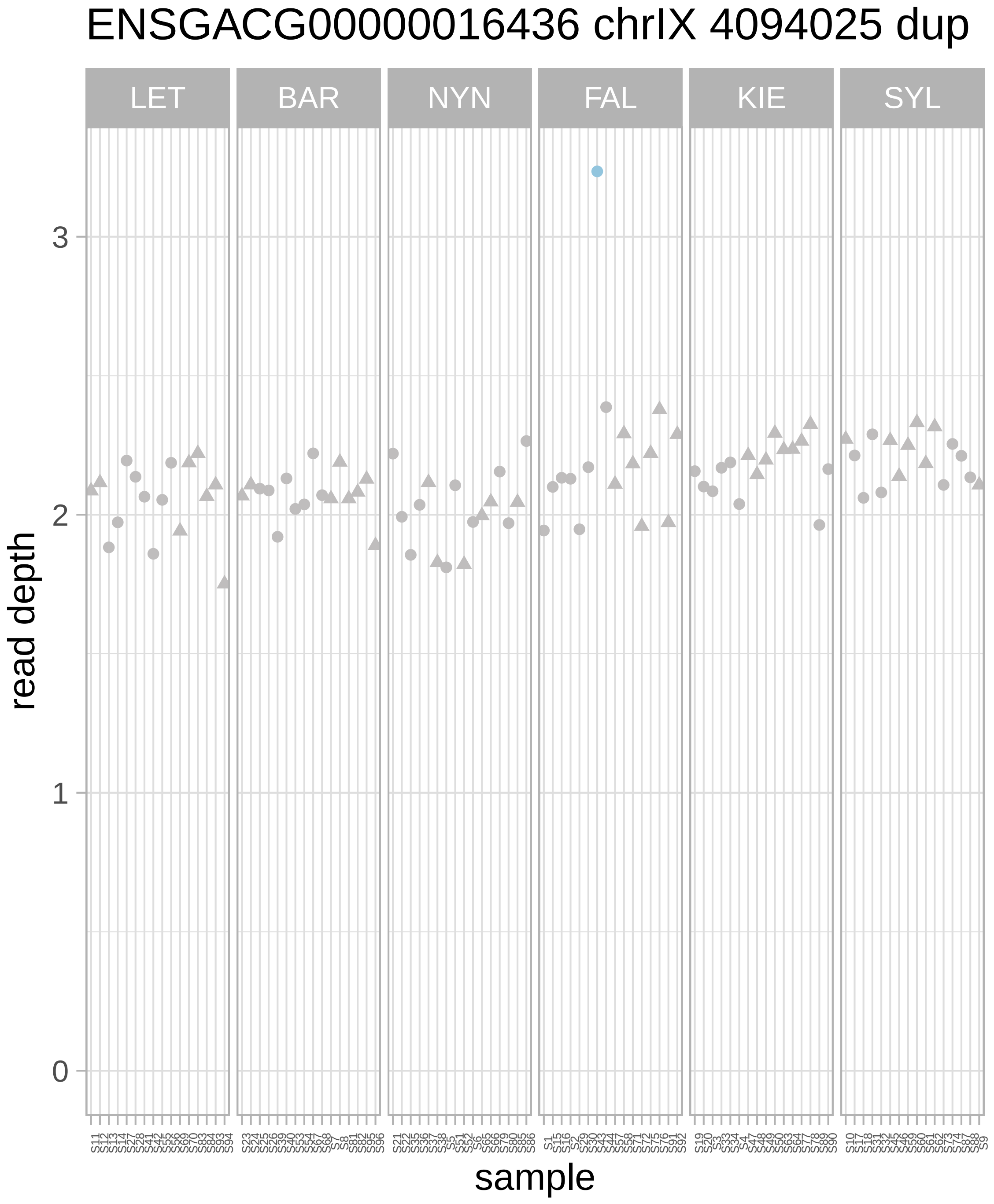
ENSGACG00000016436 chrIX 4094025 dup
LET
BAR
NYN
FAL
KIE
SYL
read depth
3
2
1
0
sample

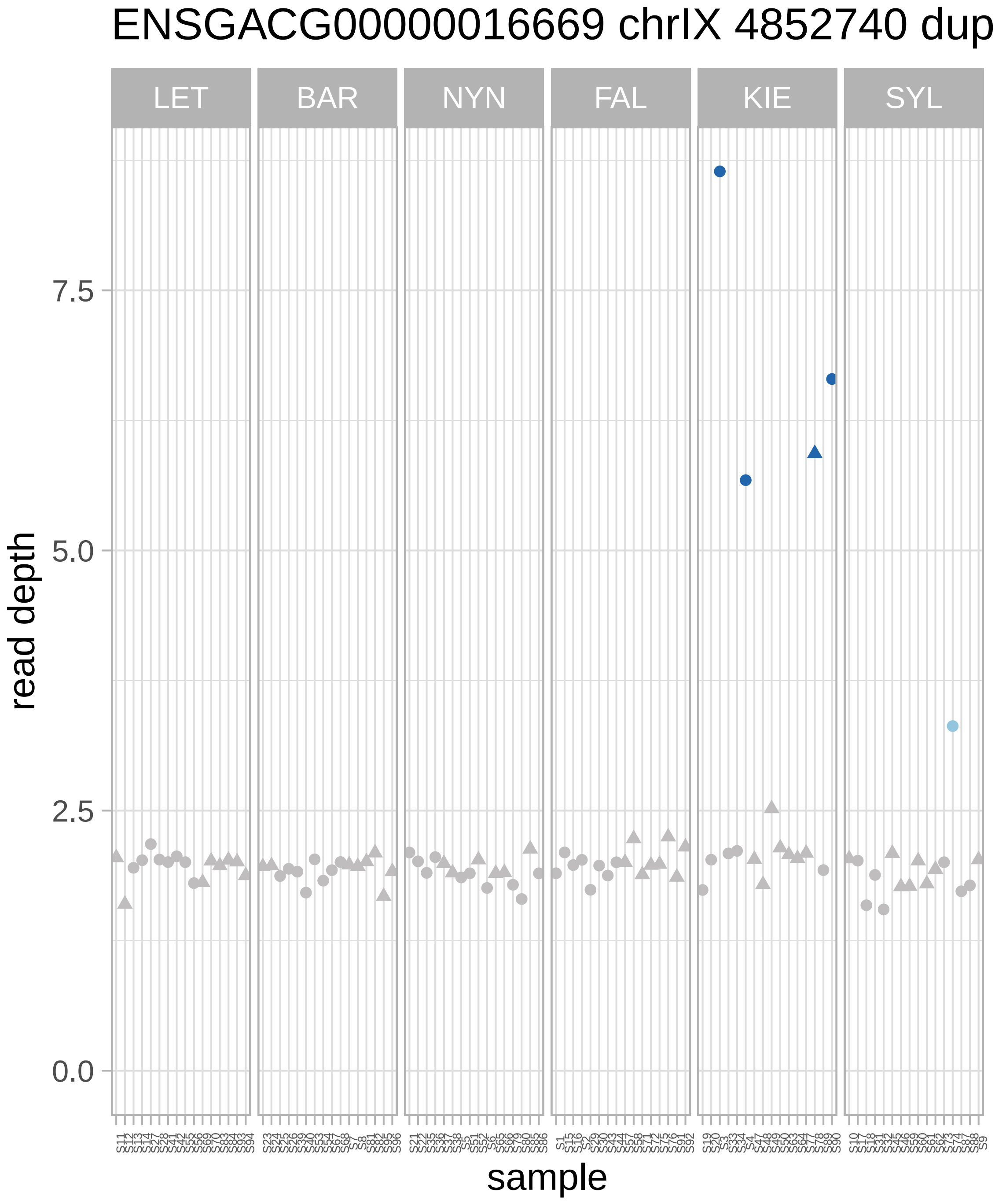
ENSGACG00000016669 chrIX 4852740 dup
LET
BAR
NYN
FAL
KIE
SYL
read depth
7.5
5.0
2.5
0.0
sample

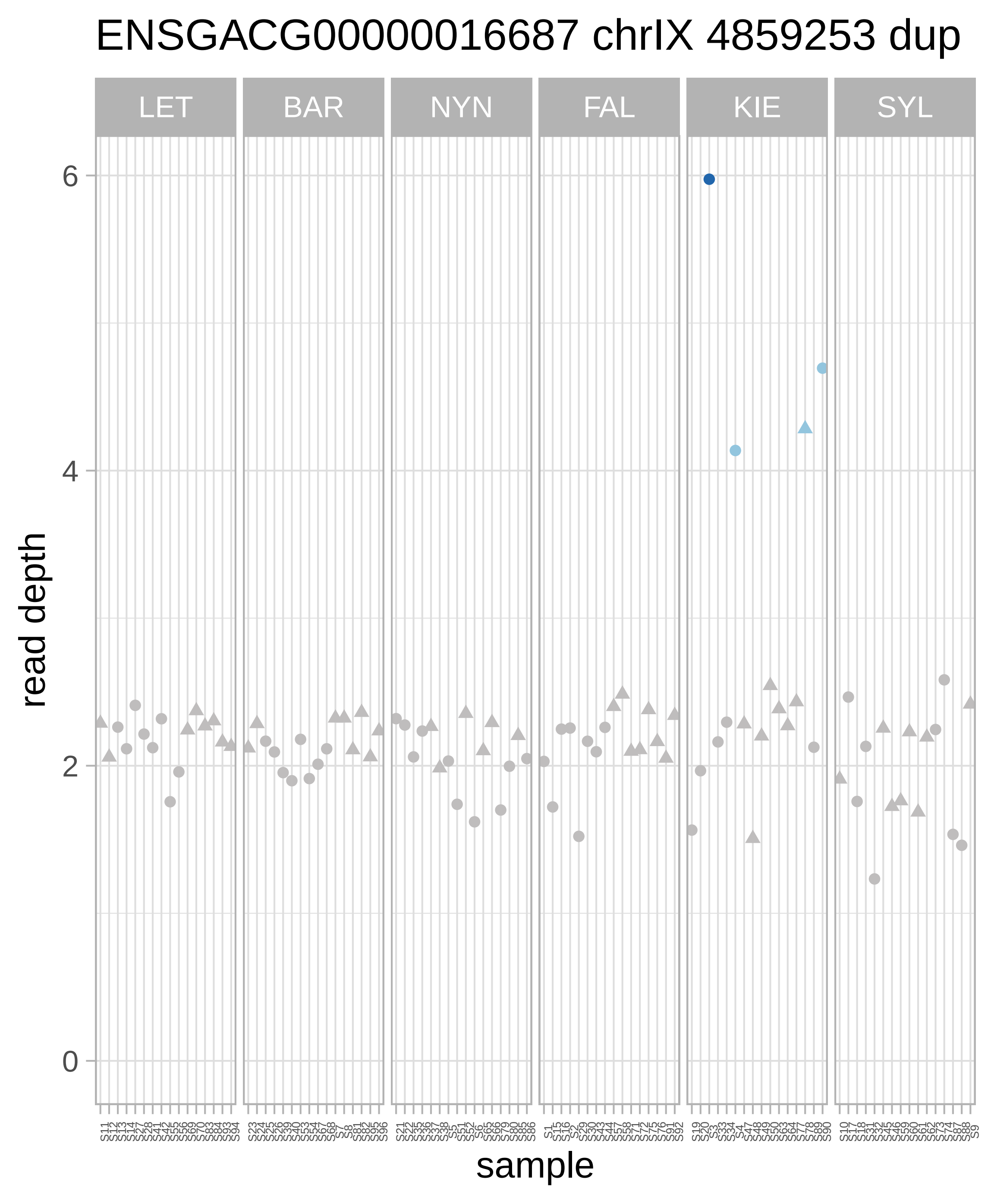
ENSGACG00000016687 chrIX 4859253 dup
LET
BAR
NYN
FAL
KIE
SYL
read depth
0
2
4
6
sample

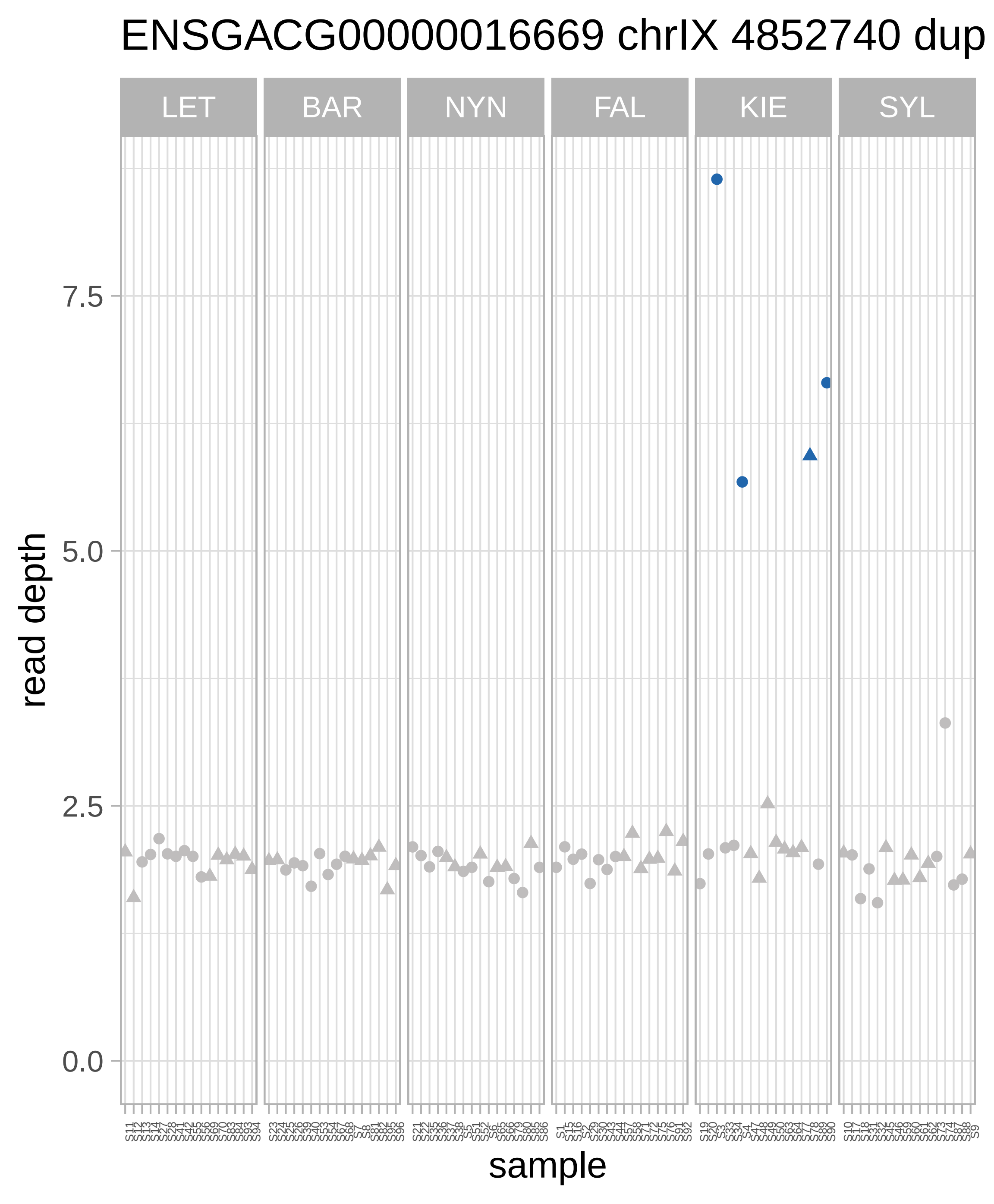
ENSGACG00000016669 chrIX 4852740 dup
LET
BAR
NYN
FAL
KIE
SYL
read depth
7.5
5.0
2.5
0.0
sample

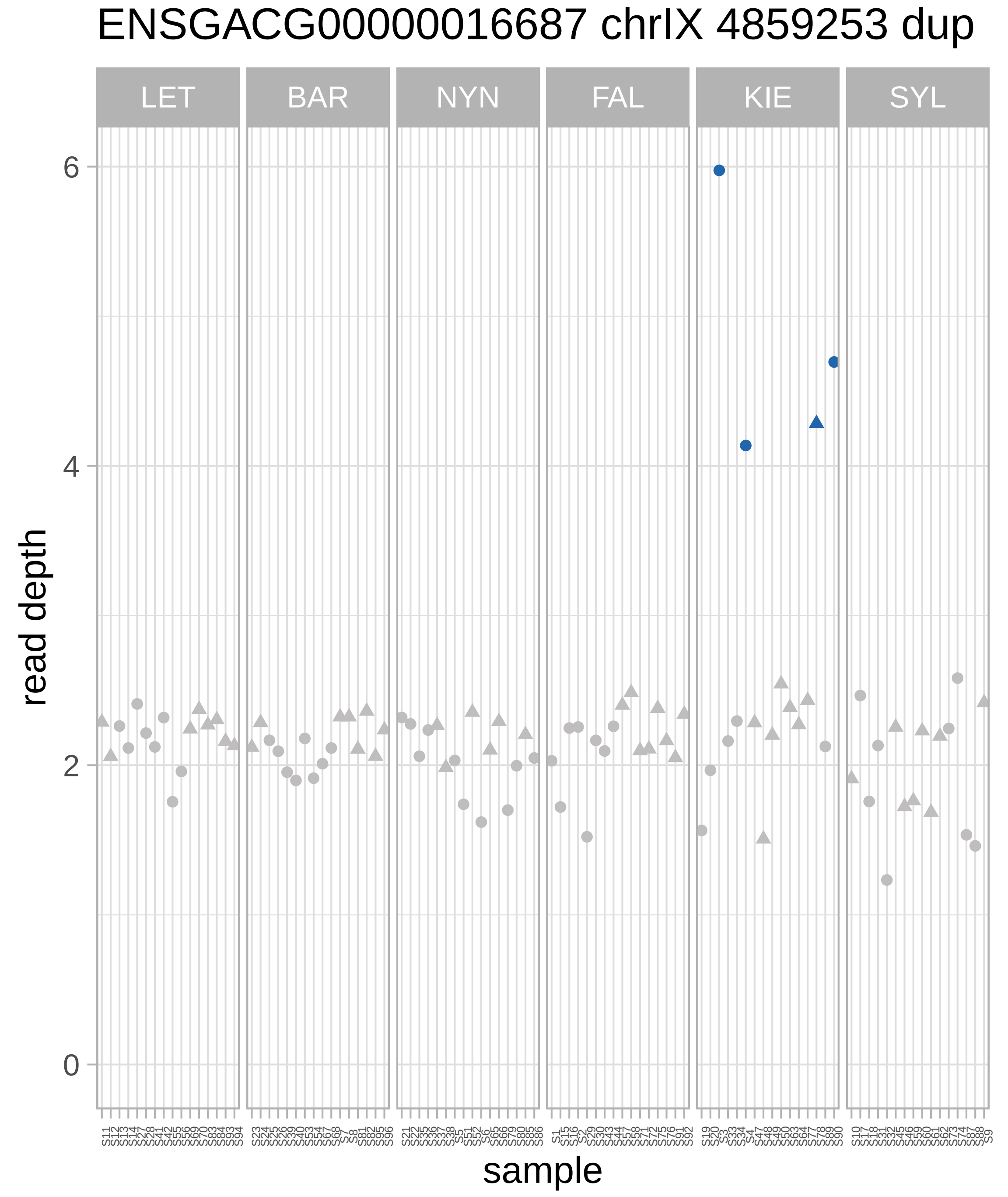

ENSGACG00000016687 chrIX 4859253 dup
LET
BAR
NYN
FAL
KIE
SYL
read depth
0
2
4
6
S11 S12 S13 S14 S27 S28 S41 S42 S55 S56 S69 S70 S83 S84 S93 S94
S23 S24 S25 S26 S39 S40 S53 S54 S67 S68 S7 S8 S81 S82 S95 S96
S21 S22 S35 S36 S37 S38 S5 S51 S52 S6 S65 S66 S79 S80 S85 S86
S1 S15 S16 S2 S29 S30 S43 S44 S57 S58 S71 S72 S75 S76 S91 S92
S19 S20 S3 S33 S34 S4 S47 S48 S49 S50 S63 S64 S77 S78 S89 S90
S10 S17 S18 S31 S32 S45 S46 S59 S60 S61 S62 S73 S74 S87 S88 S9
sample

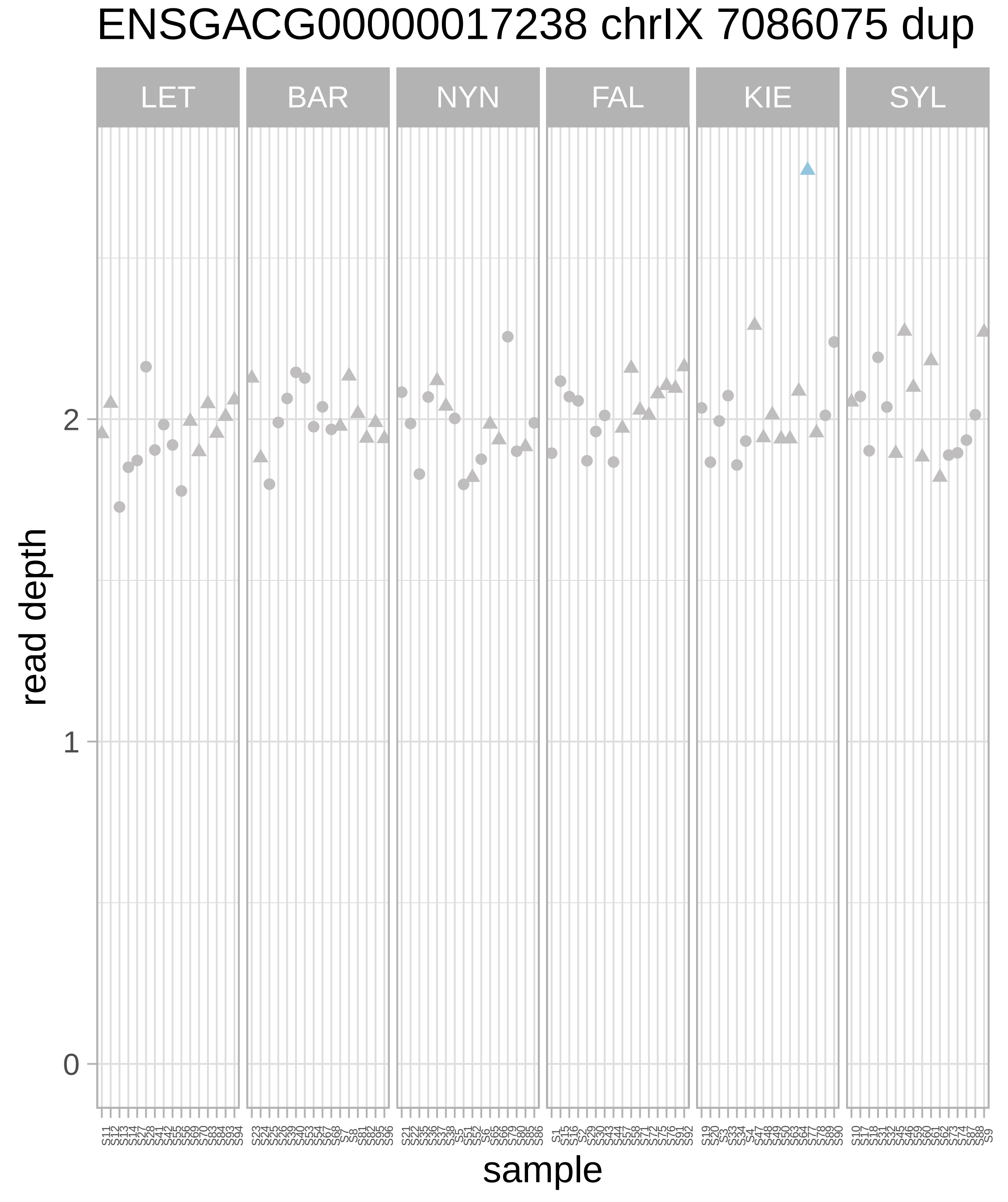
ENSGACG00000017238 chrIX 7086075 dup
LET
BAR
NYN
FAL
KIE
SYL
read depth
0
1
2
S11 S12 S13 S14 S27 S28 S41 S42 S55 S56 S69 S70 S83 S84 S93 S94
S23 S24 S25 S26 S39 S40 S53 S54 S67 S68 S7 S8 S81 S82 S95 S96
S21 S22 S35 S36 S37 S38 S5 S51 S52 S6 S65 S66 S79 S80 S85 S86
S1 S15 S16 S2 S29 S30 S43 S44 S57 S58 S71 S72 S75 S76 S91 S92
S19 S20 S3 S33 S34 S4 S47 S48 S49 S50 S63 S64 S77 S78 S89 S90
S10 S17 S18 S31 S32 S45 S46 S59 S60 S61 S62 S73 S74 S87 S88 S9
sample

ENSGACG00000017248 chrIX 7095599 dup

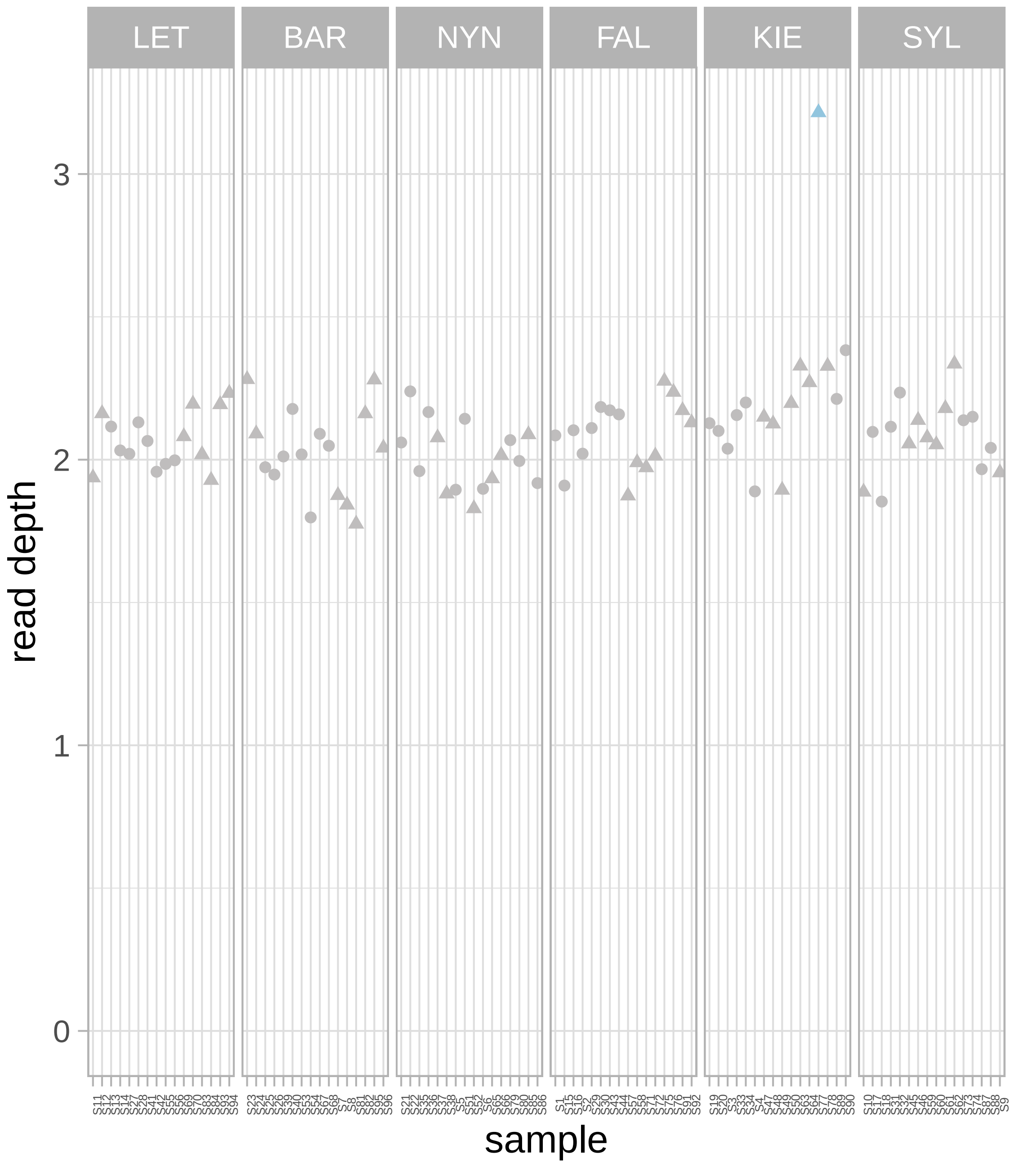

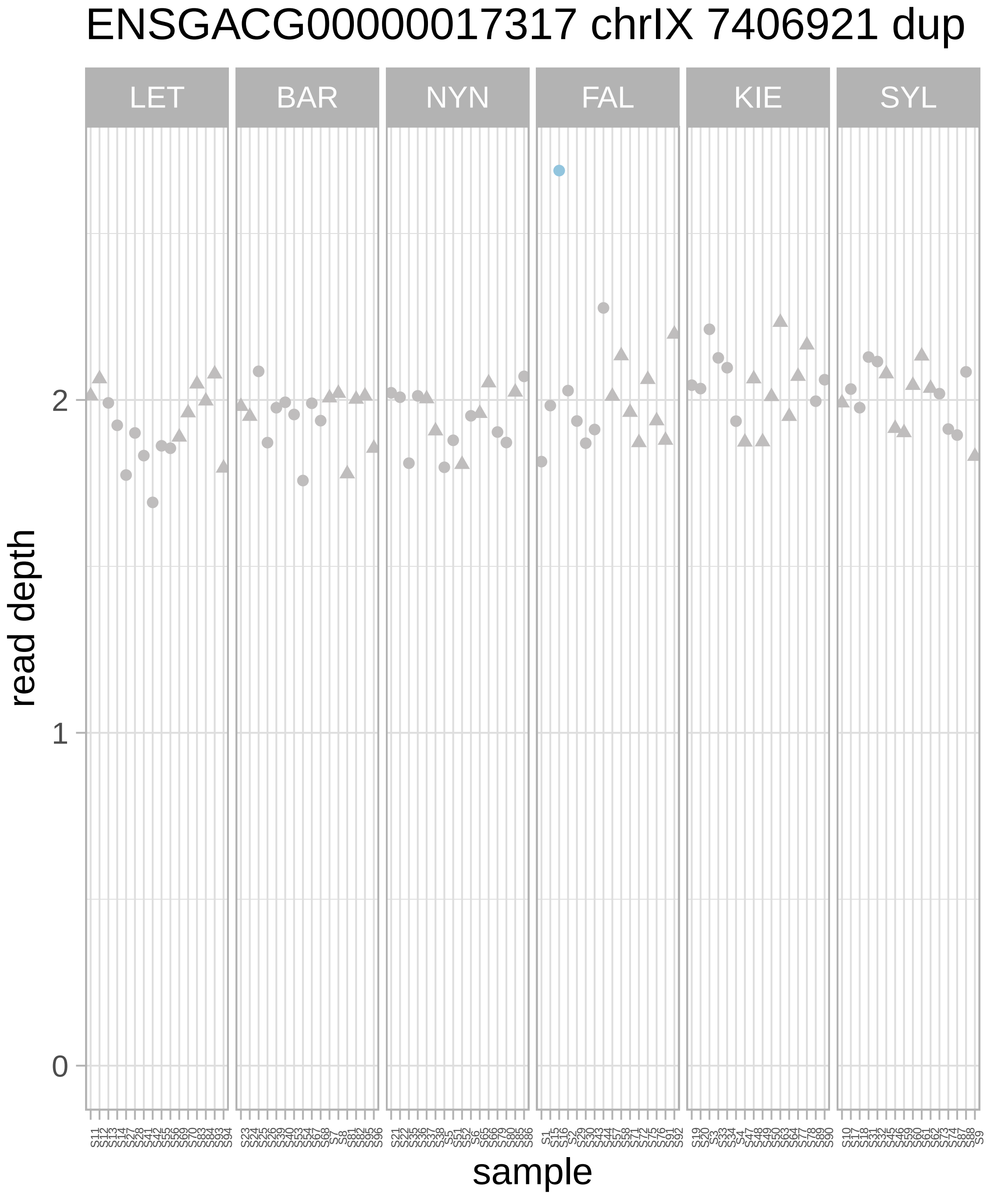

ENSGACG00000017317 chrIX 7406921 dup
LET
BAR
NYN
FAL
KIE
SYL
read depth
0
1
2
sample
S11 S12 S13 S14 S27 S28 S41 S42 S55 S56 S69 S70 S83 S84 S93 S94
S23 S24 S25 S26 S39 S40 S53 S54 S67 S68 S7 S8 S81 S82 S95 S96
S21 S22 S35 S36 S37 S38 S5 S51 S52 S6 S65 S66 S79 S80 S85 S86
S1 S15 S16 S2 S29 S30 S43 S44 S57 S58 S71 S72 S75 S76 S91 S92
S19 S20 S3 S33 S34 S4 S47 S48 S49 S50 S63 S64 S77 S78 S89 S90
S10 S17 S18 S31 S32 S45 S46 S59 S60 S61 S62 S73 S74 S87 S88 S9

ENSGACG00000017322 chrIX 7420832 dup

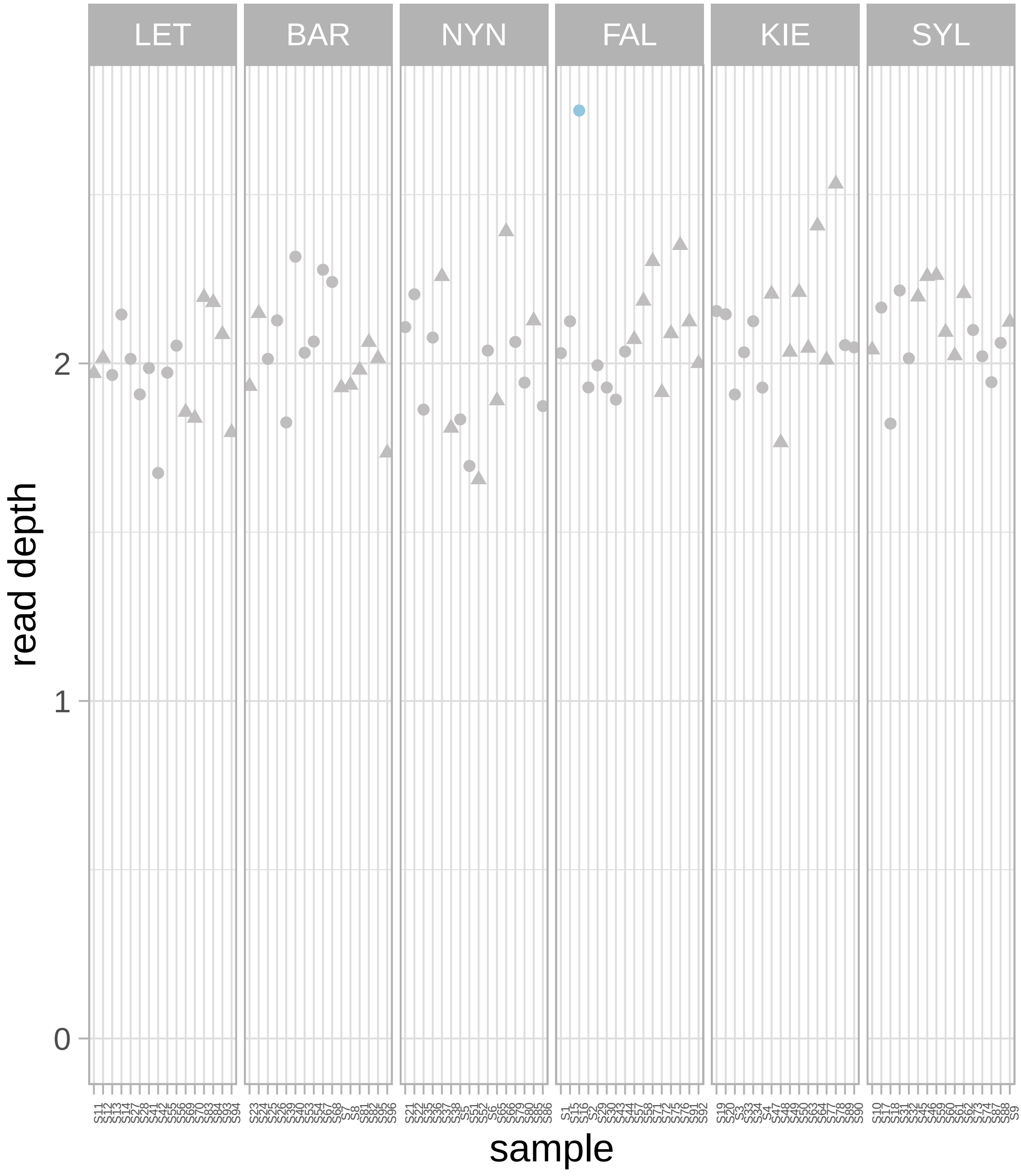

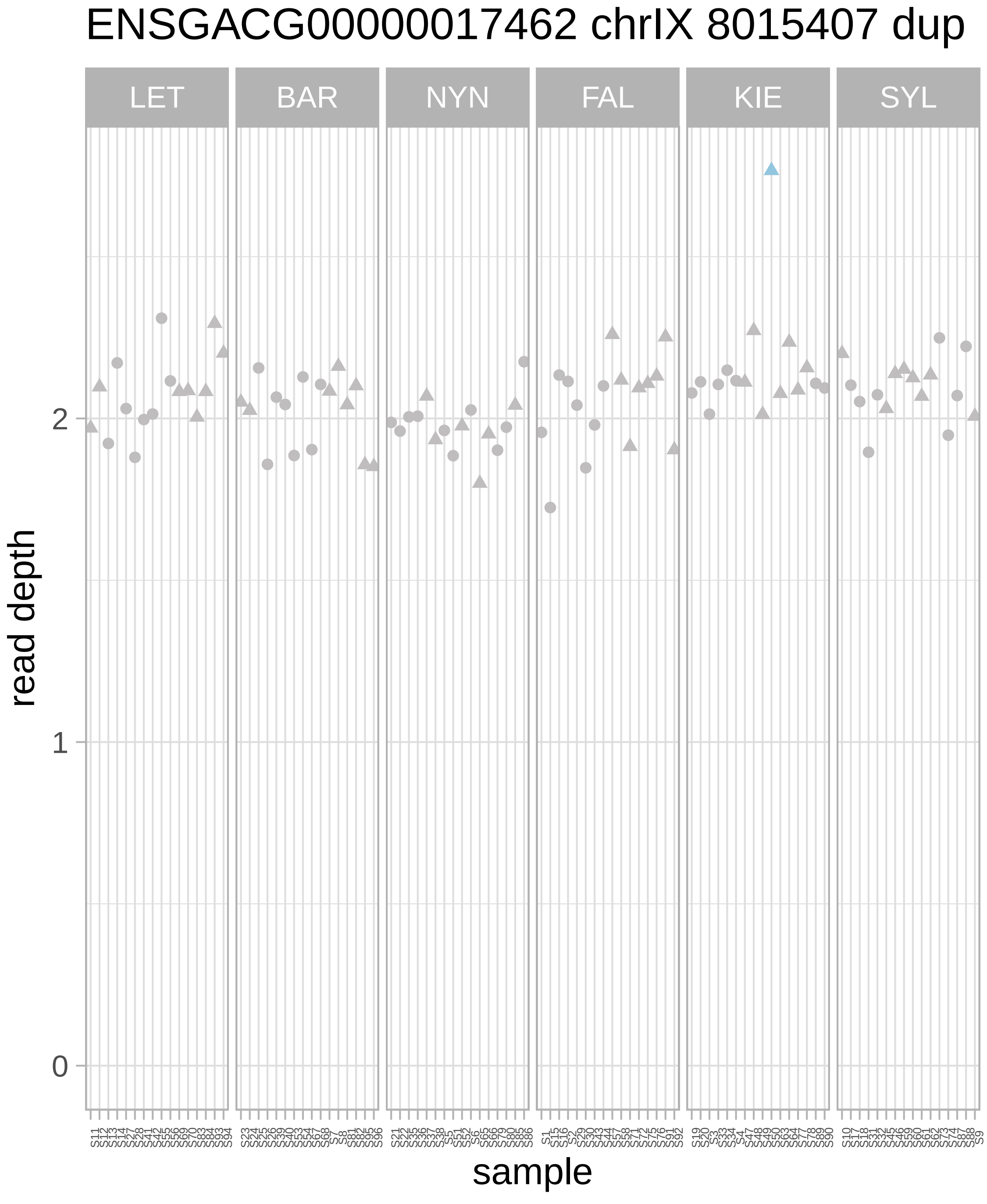
ENSGACG00000017462 chrIX 8015407 dup
LET
BAR
NYN
FAL
KIE
SYL
read depth
2
1
0
sample
S11 S12 S13 S14 S27 S28 S41 S42 S55 S56 S69 S70 S83 S84 S93 S94
S23 S24 S25 S26 S39 S40 S53 S54 S67 S68 S7 S8 S81 S82 S95 S96
S21 S22 S35 S36 S37 S38 S5 S51 S52 S6 S65 S66 S79 S80 S85 S86
S1 S15 S16 S2 S29 S30 S43 S44 S57 S58 S71 S72 S75 S76 S91 S92
S19 S20 S3 S33 S34 S4 S47 S48 S49 S50 S63 S64 S77 S78 S89 S90
S10 S17 S18 S31 S32 S45 S46 S59 S60 S61 S62 S73 S74 S87 S88 S9

ENSGACG00000017492 chrIX 8023545 dup

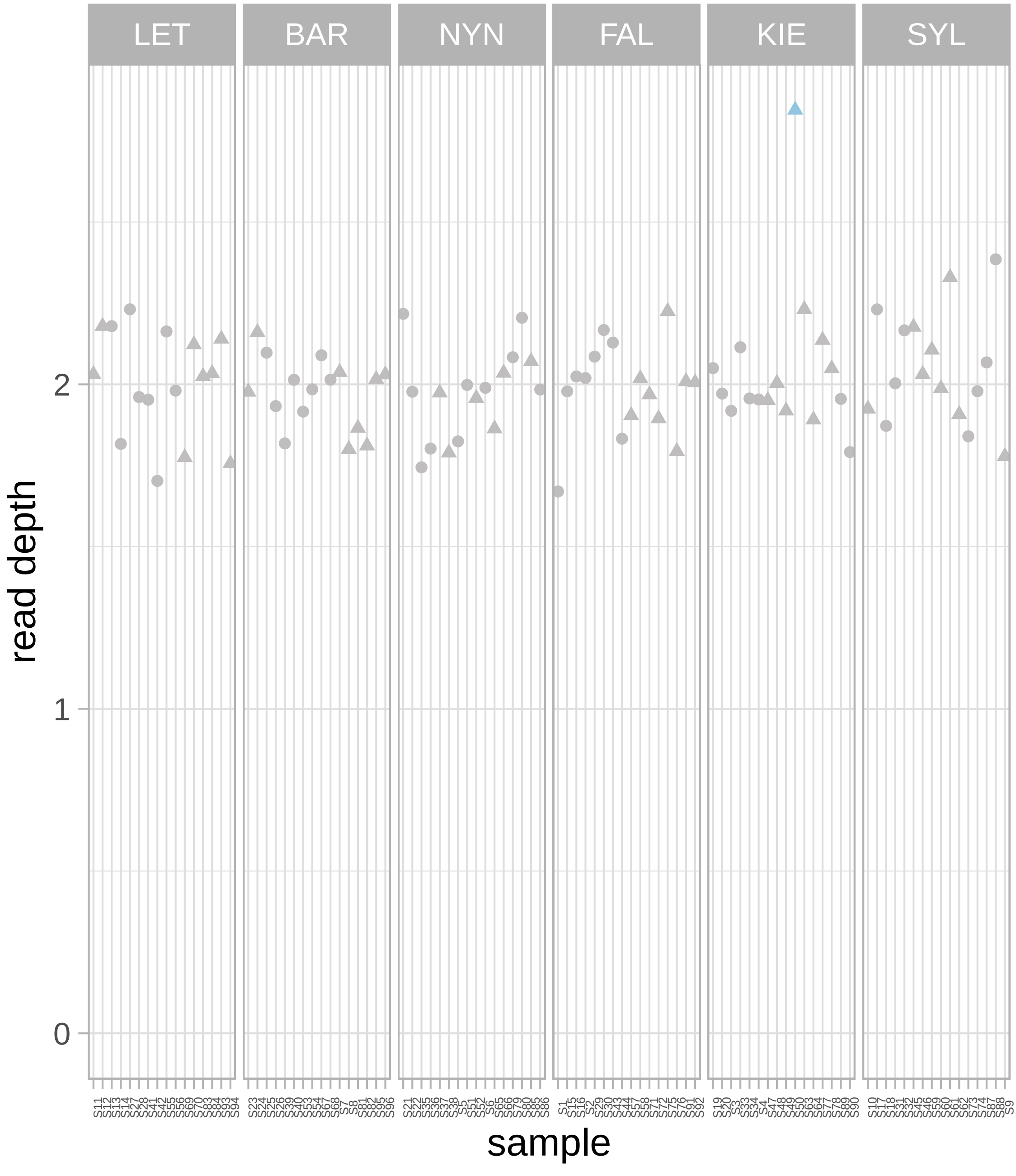

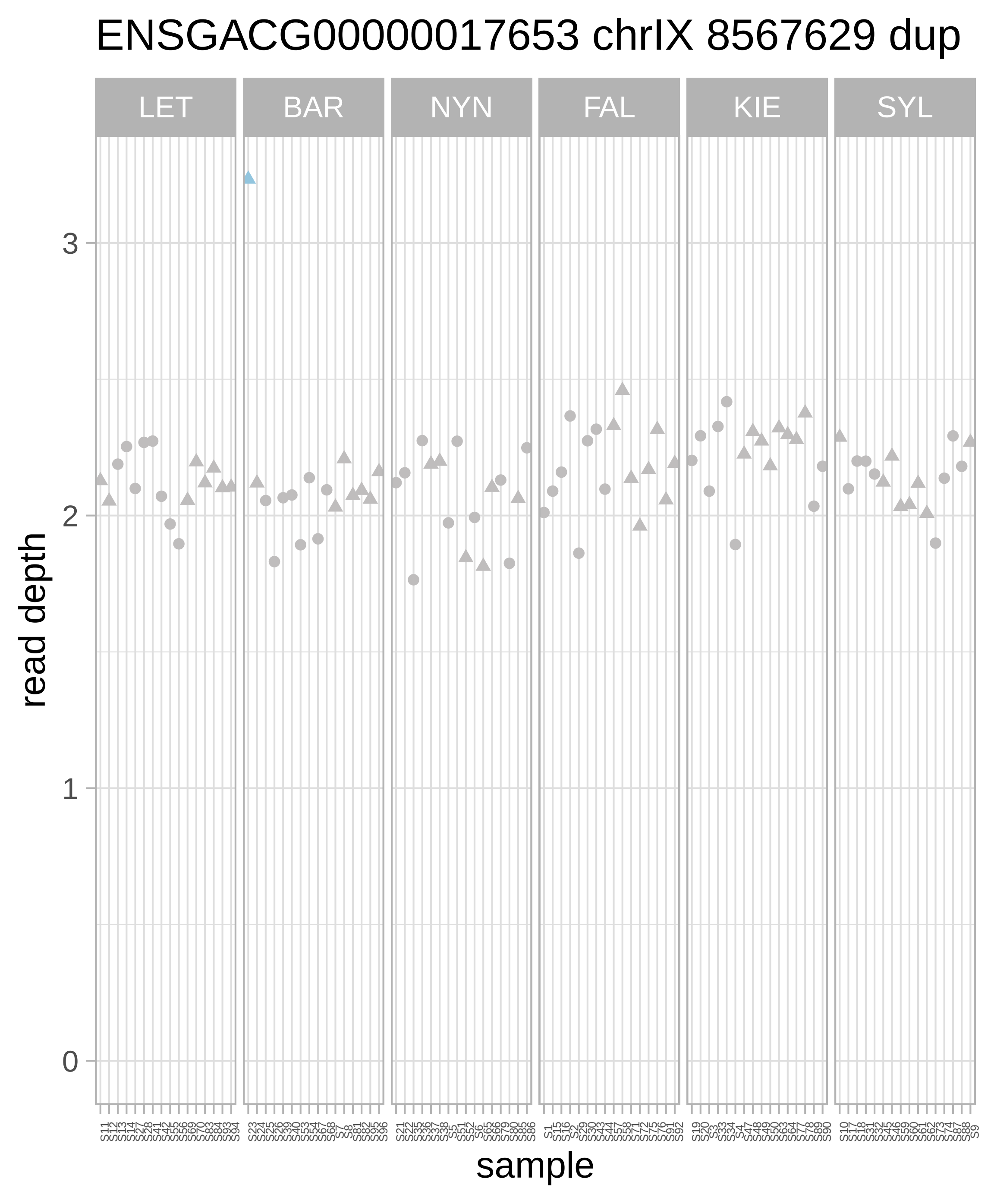
ENSGACG00000017653 chrIX 8567629 dup
LET
BAR
NYN
FAL
KIE
SYL
read depth
0
1
2
3
sample

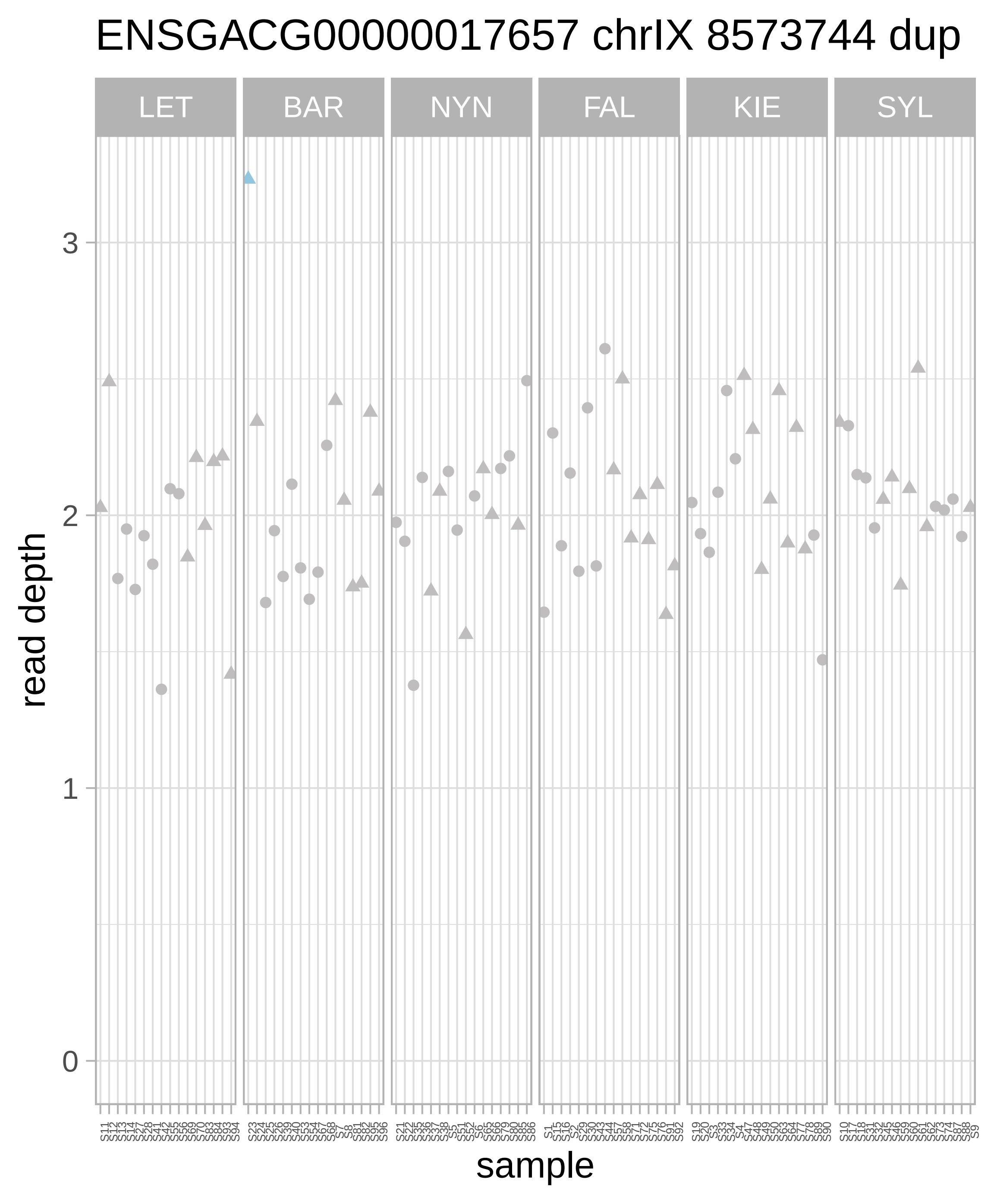
ENSGACG00000017657 chrIX 8573744 dup
LET
BAR
NYN
FAL
KIE
SYL
read depth
3
2
1
0
sample

ENSGACG00000017994 chrIX 9406132 del

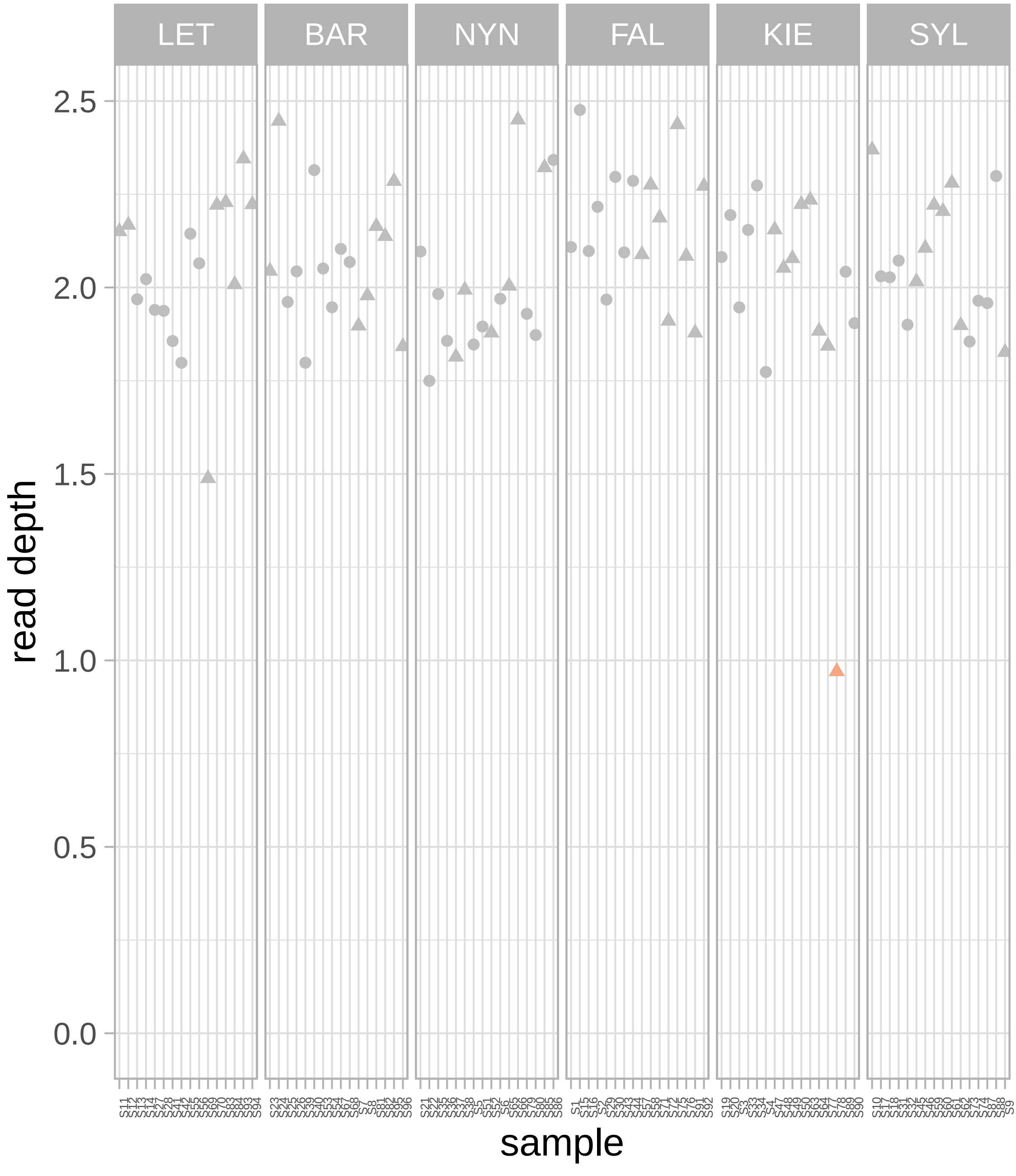

ENSGACG00000018160 chrIX 10171913 dup

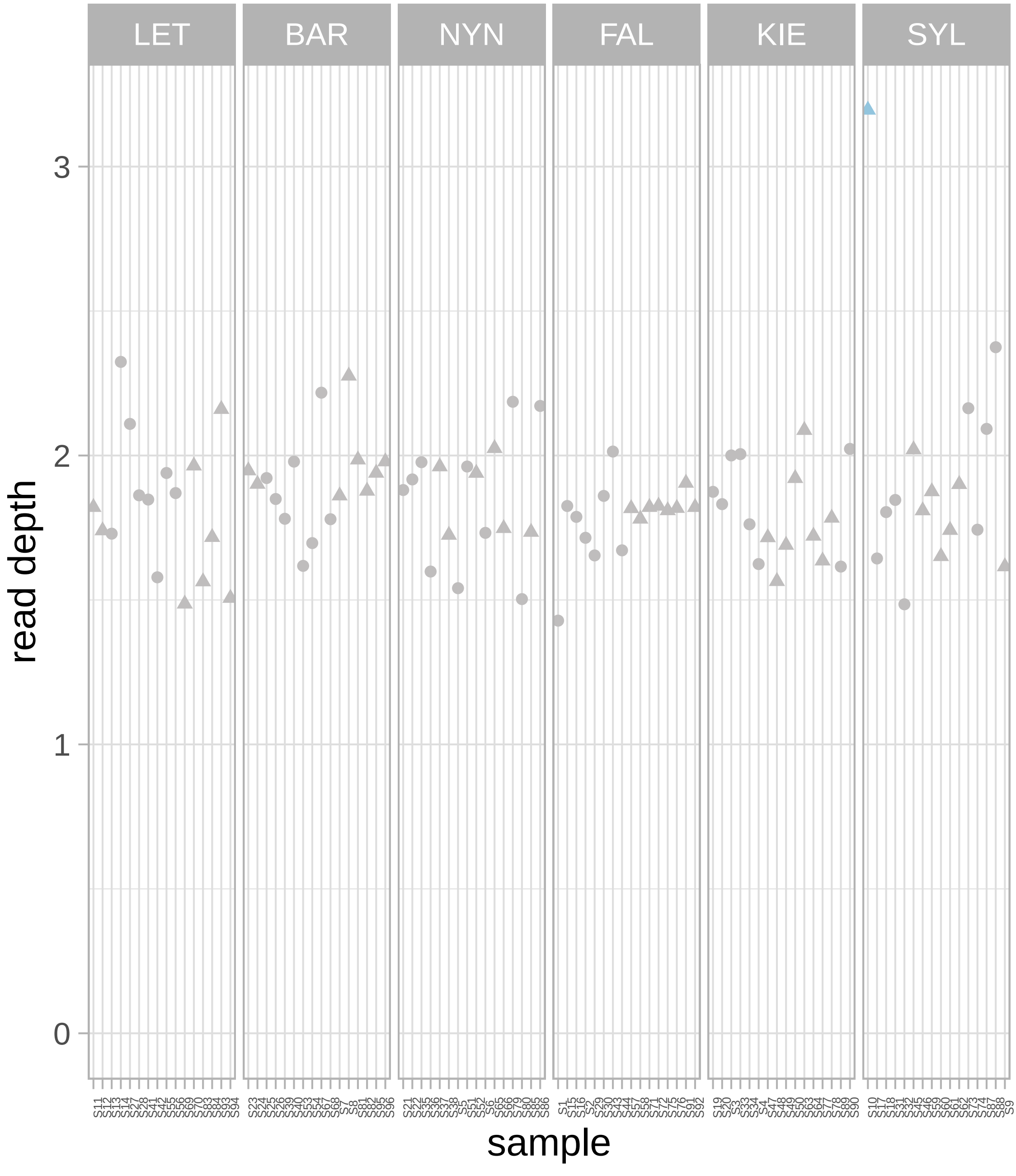

ENSGACG00000018208 chrIX 10461924 both

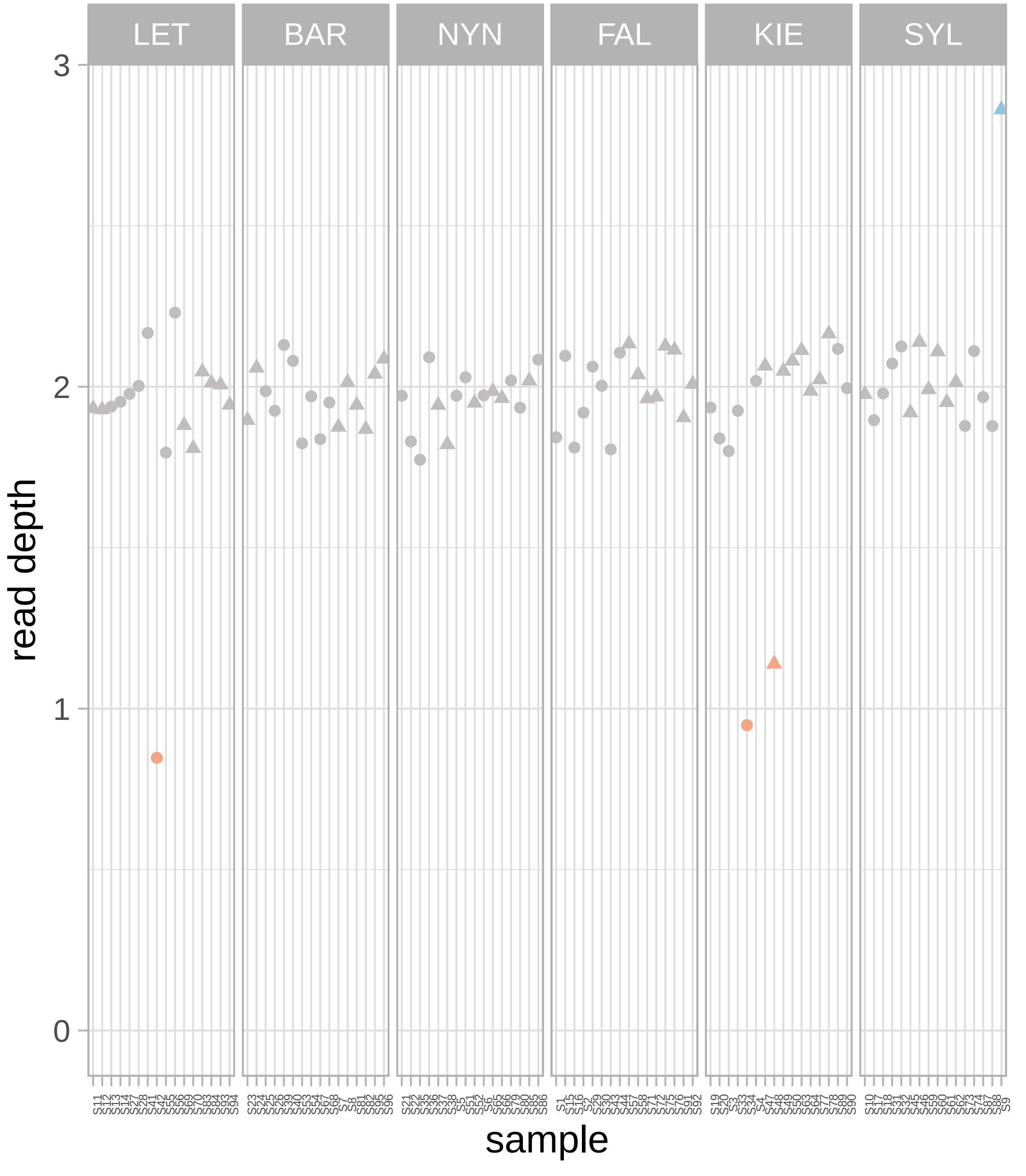

ENSGACG00000018530 chrIX 12938359 dup

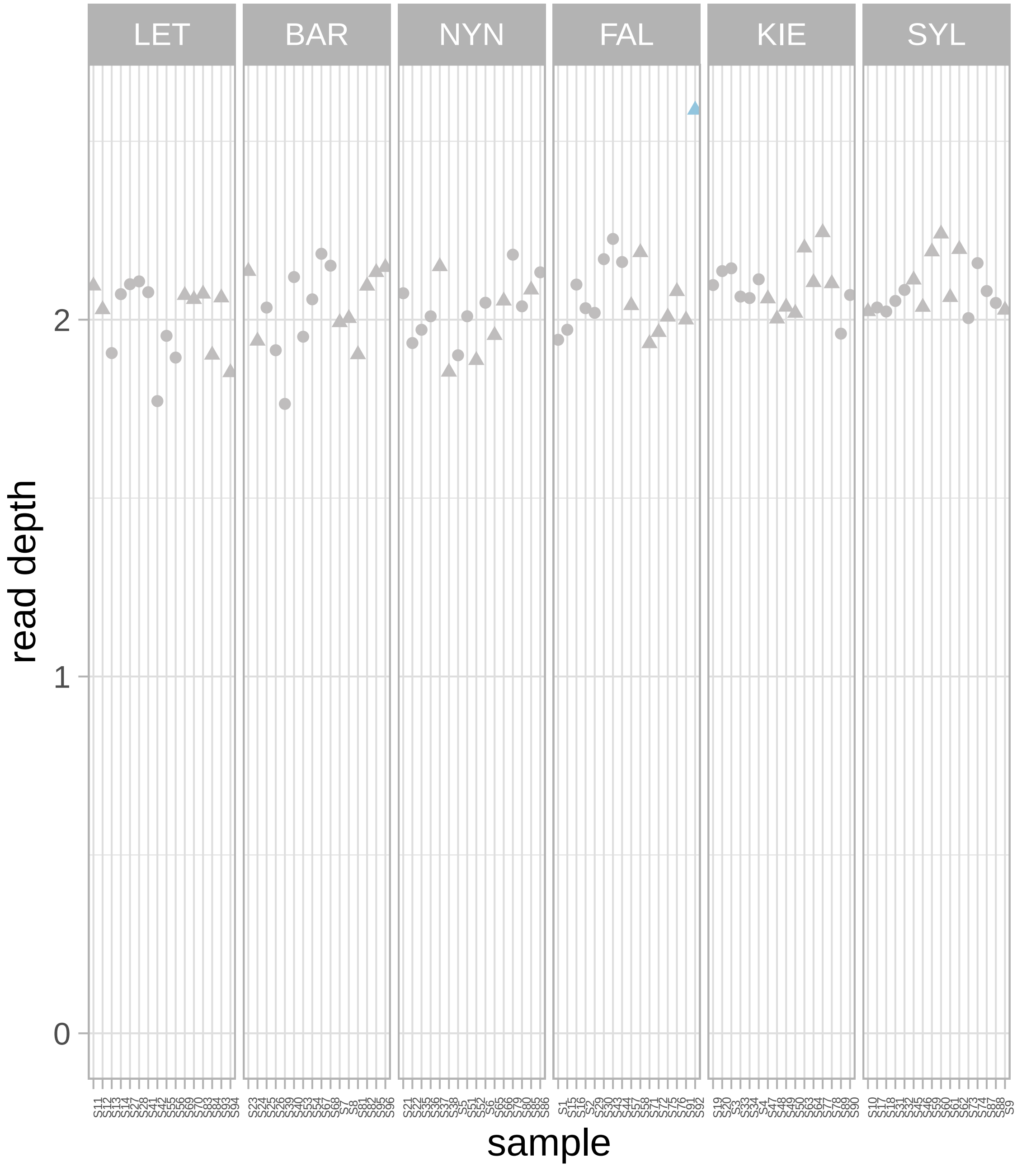

ENSGACG00000018535 chrIX 12976613 dup

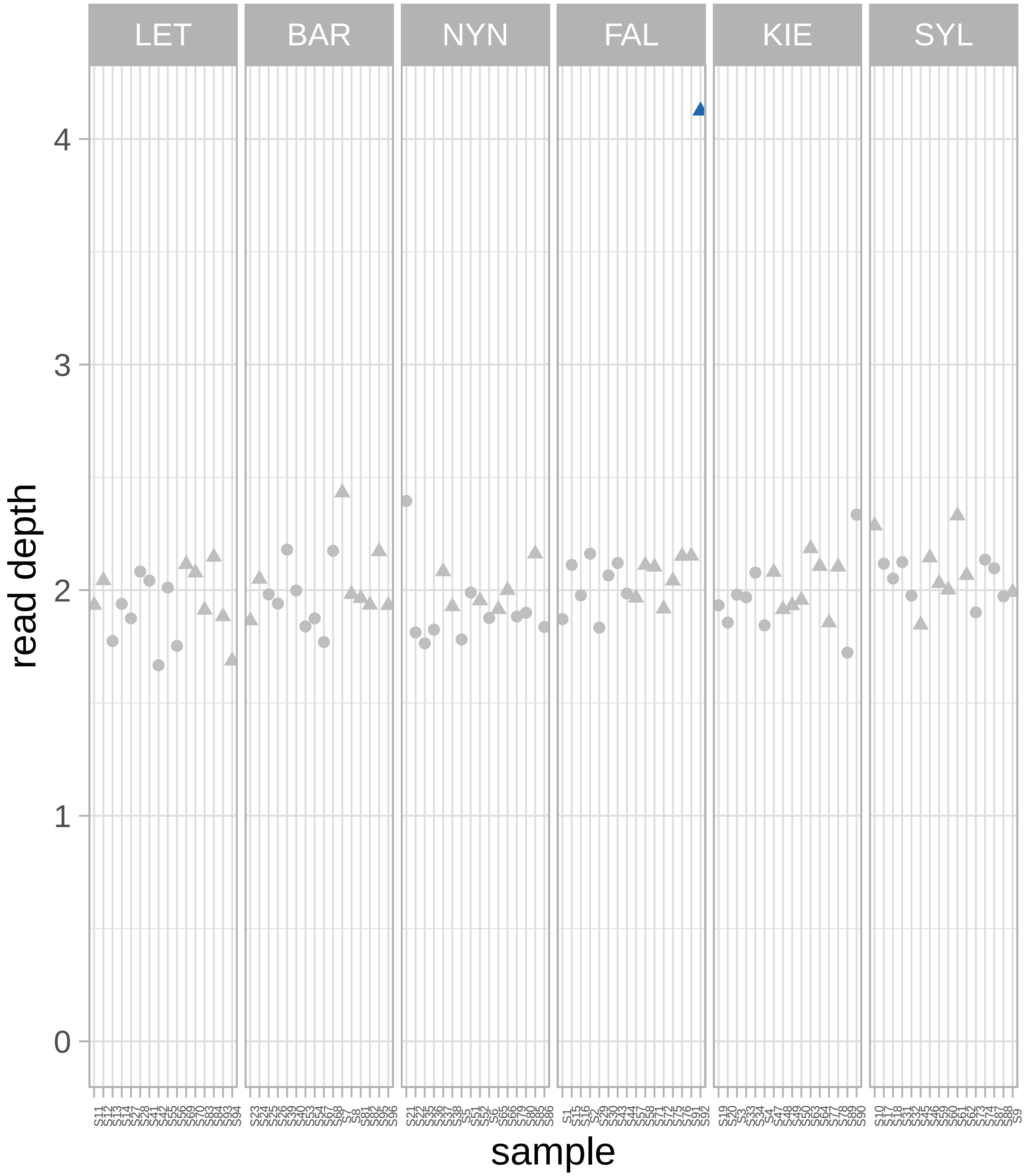

ENSGACG00000019054 chrIX 15288385 dup

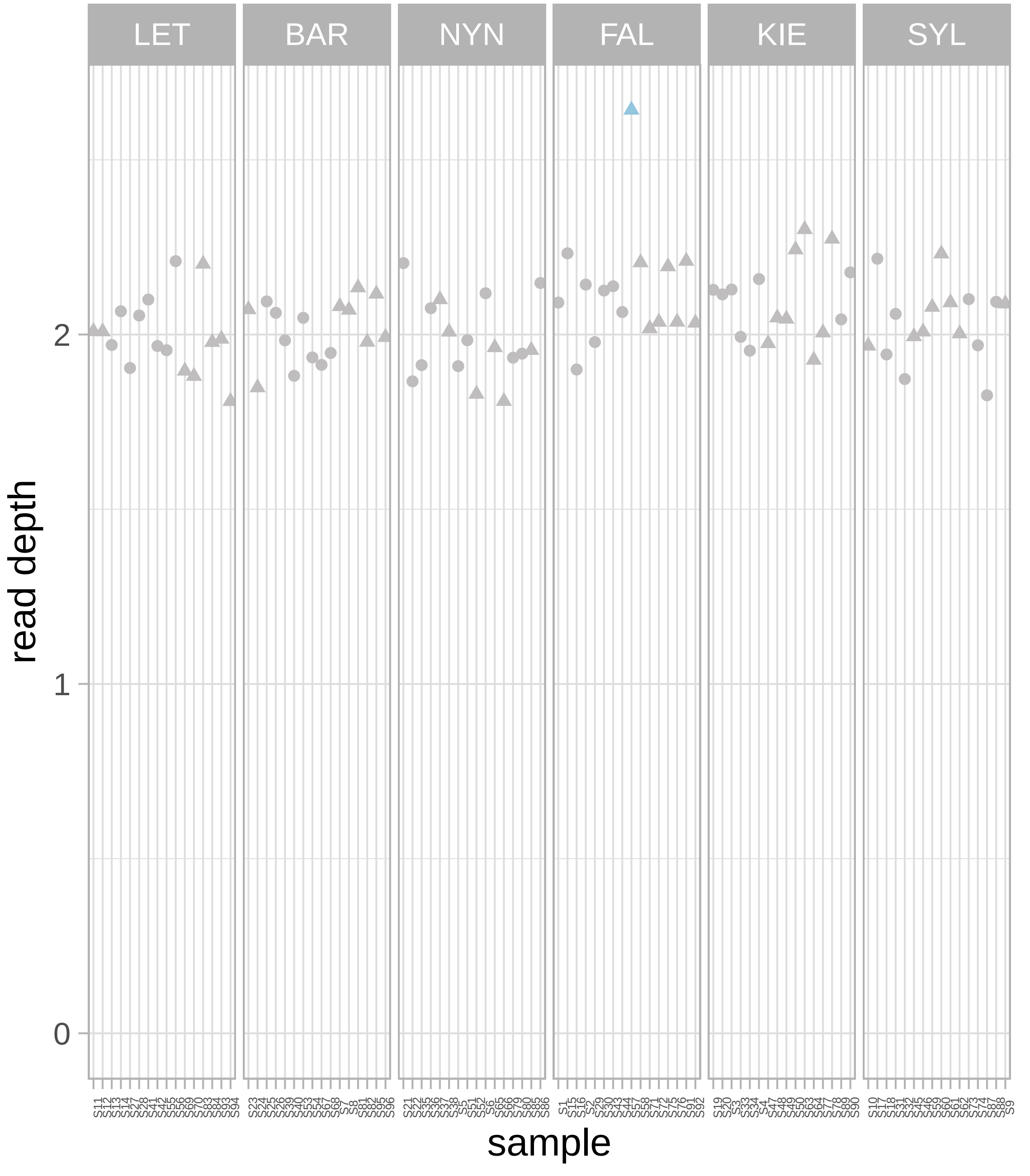

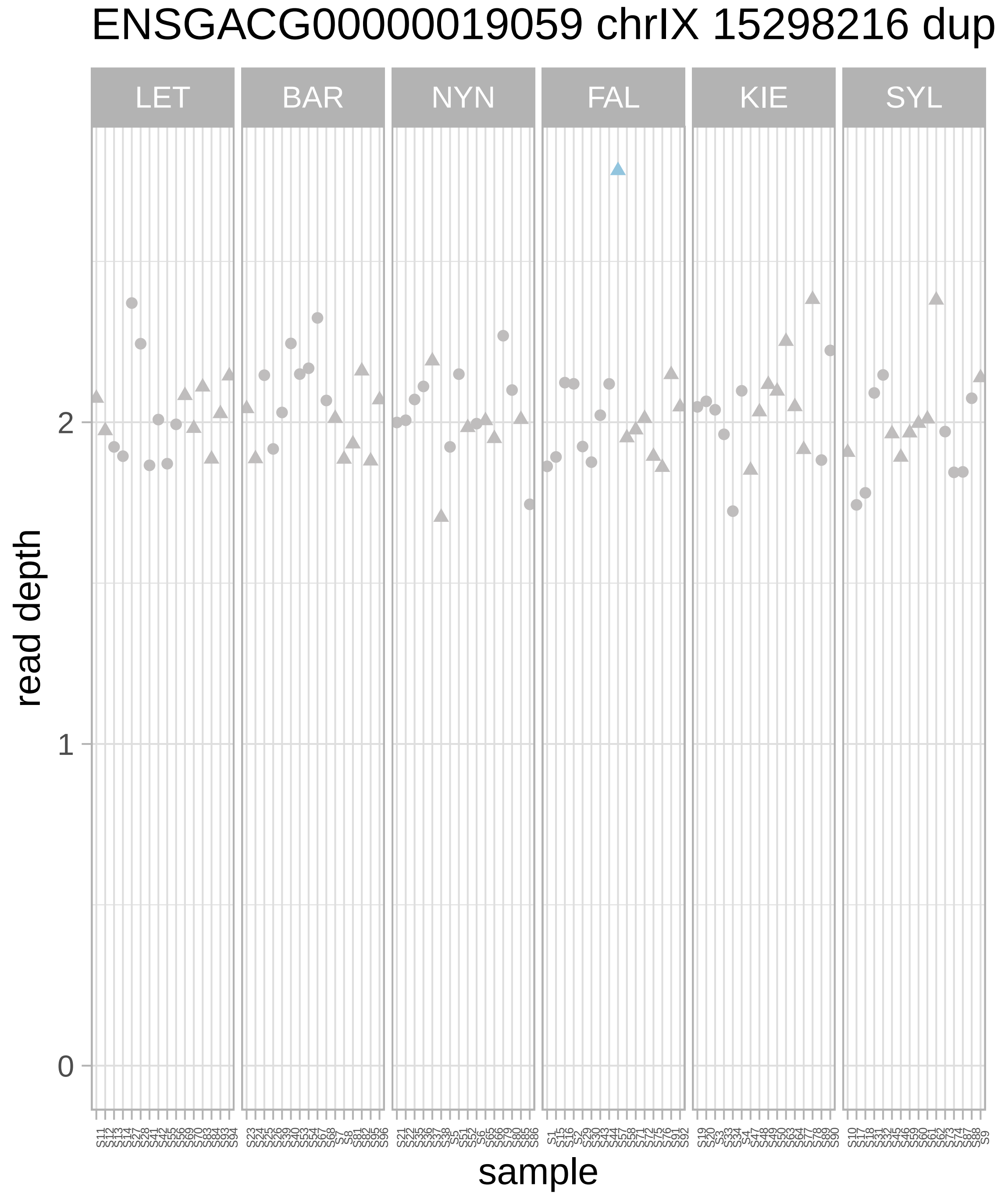
ENSGACG00000019059 chrIX 15298216 dup
LET
BAR
NYN
FAL
KIE
SYL
read depth
0
1
2
sample
S11 S12 S13 S14 S27 S28 S41 S42 S55 S56 S69 S70 S83 S84 S93 S94
S23 S24 S25 S26 S39 S40 S53 S54 S67 S68 S7 S8 S81 S82 S95 S96
S21 S22 S35 S36 S37 S38 S5 S51 S52 S6 S65 S66 S79 S80 S85 S86
S1 S15 S16 S2 S29 S30 S43 S44 S57 S58 S71 S72 S75 S76 S91 S92
S19 S20 S3 S33 S34 S4 S47 S48 S49 S50 S63 S64 S77 S78 S89 S90
S10 S17 S18 S31 S32 S45 S46 S59 S60 S61 S62 S73 S74 S87 S88 S9

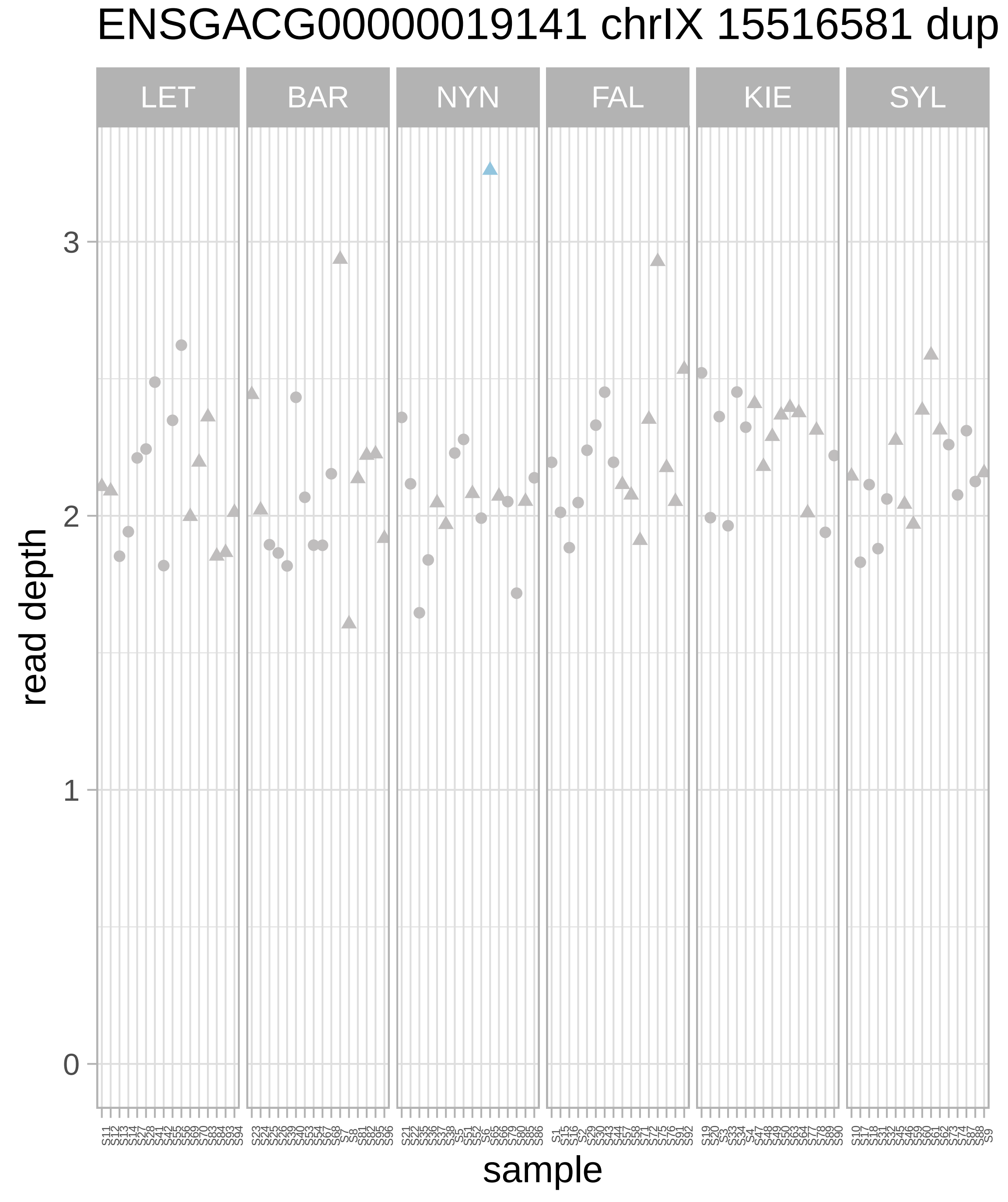
ENSGACG00000019141 chrIX 15516581 dup
LET
BAR
NYN
FAL
KIE
SYL
read depth
0
1
2
3
sample

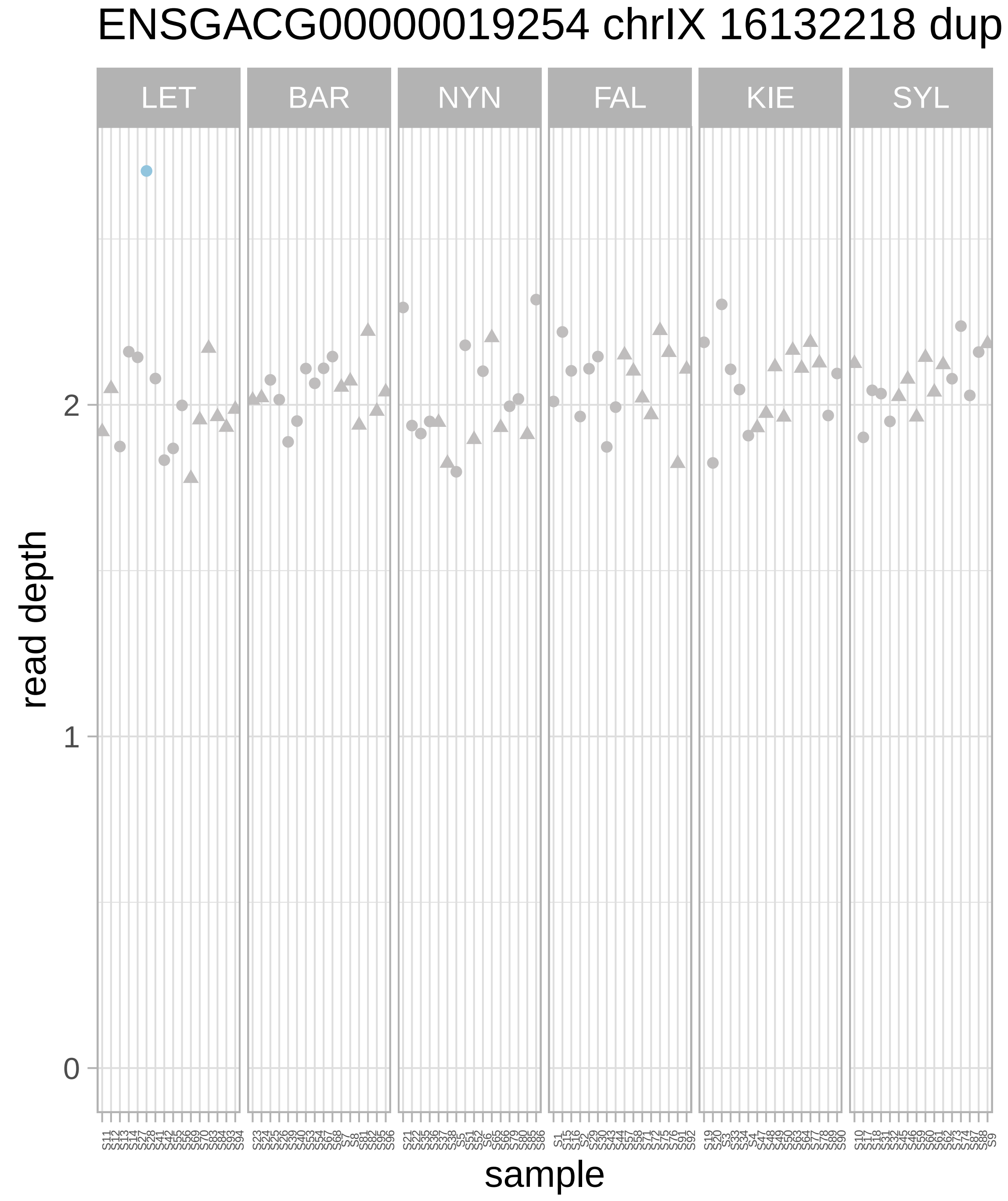
ENSGACG00000019254 chrIX 16132218 dup
LET
BAR
NYN
FAL
KIE
SYL
read depth
0
1
2
sample
S11 S12 S13 S14 S27 S28 S41 S42 S55 S56 S69 S70 S83 S84 S93 S94
S23 S24 S25 S26 S39 S40 S53 S54 S67 S68 S7 S8 S81 S82 S95 S96
S21 S22 S35 S36 S37 S38 S5 S51 S52 S6 S65 S66 S79 S80 S85 S86
S1 S15 S16 S2 S29 S30 S43 S44 S57 S58 S71 S72 S75 S76 S91 S92
S19 S20 S3 S33 S34 S4 S47 S48 S49 S50 S63 S64 S77 S78 S89 S90
S10 S17 S18 S31 S32 S45 S46 S59 S60 S61 S62 S73 S74 S87 S88 S9

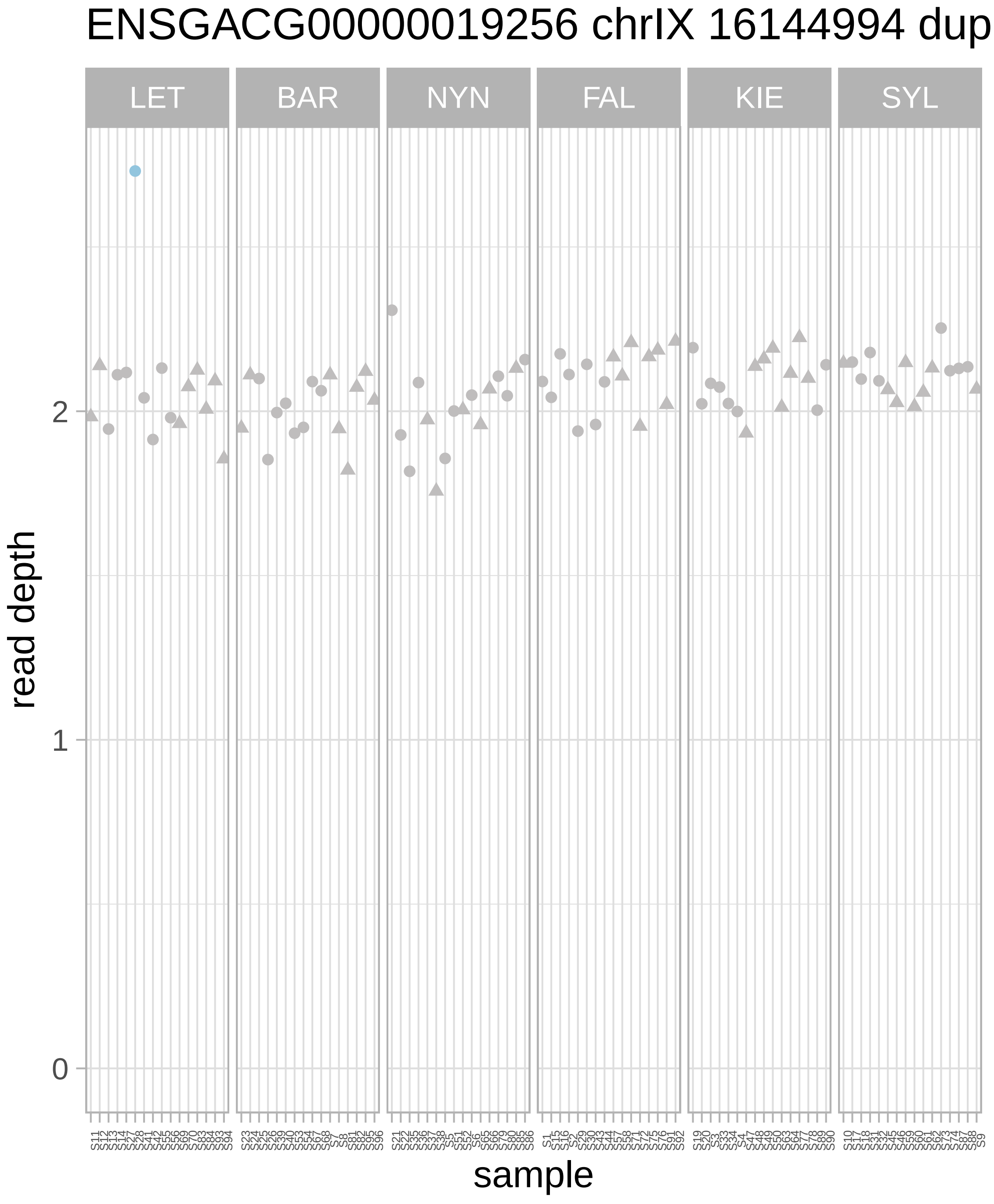
ENSGACG00000019256 chrIX 16144994 dup
LET
BAR
NYN
FAL
KIE
SYL
read depth
0
1
2
sample

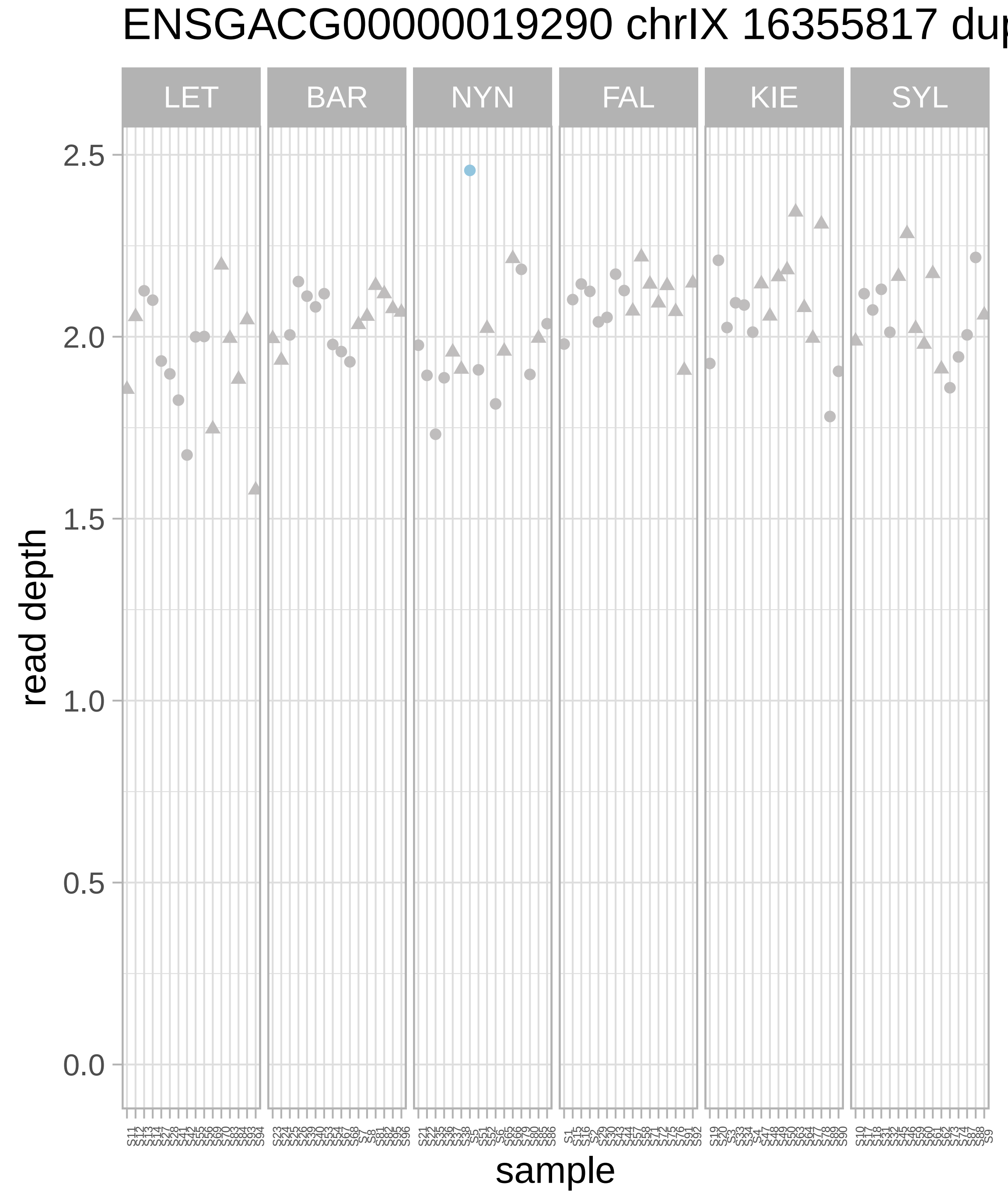
ENSGACG00000019290 chrIX 16355817 dup
LET
BAR
NYN
FAL
KIE
SYL
read depth
2.5
2.0
1.5
1.0
0.5
0.0
sample

ENSGACG00000019296 chrIX 16371697 dup

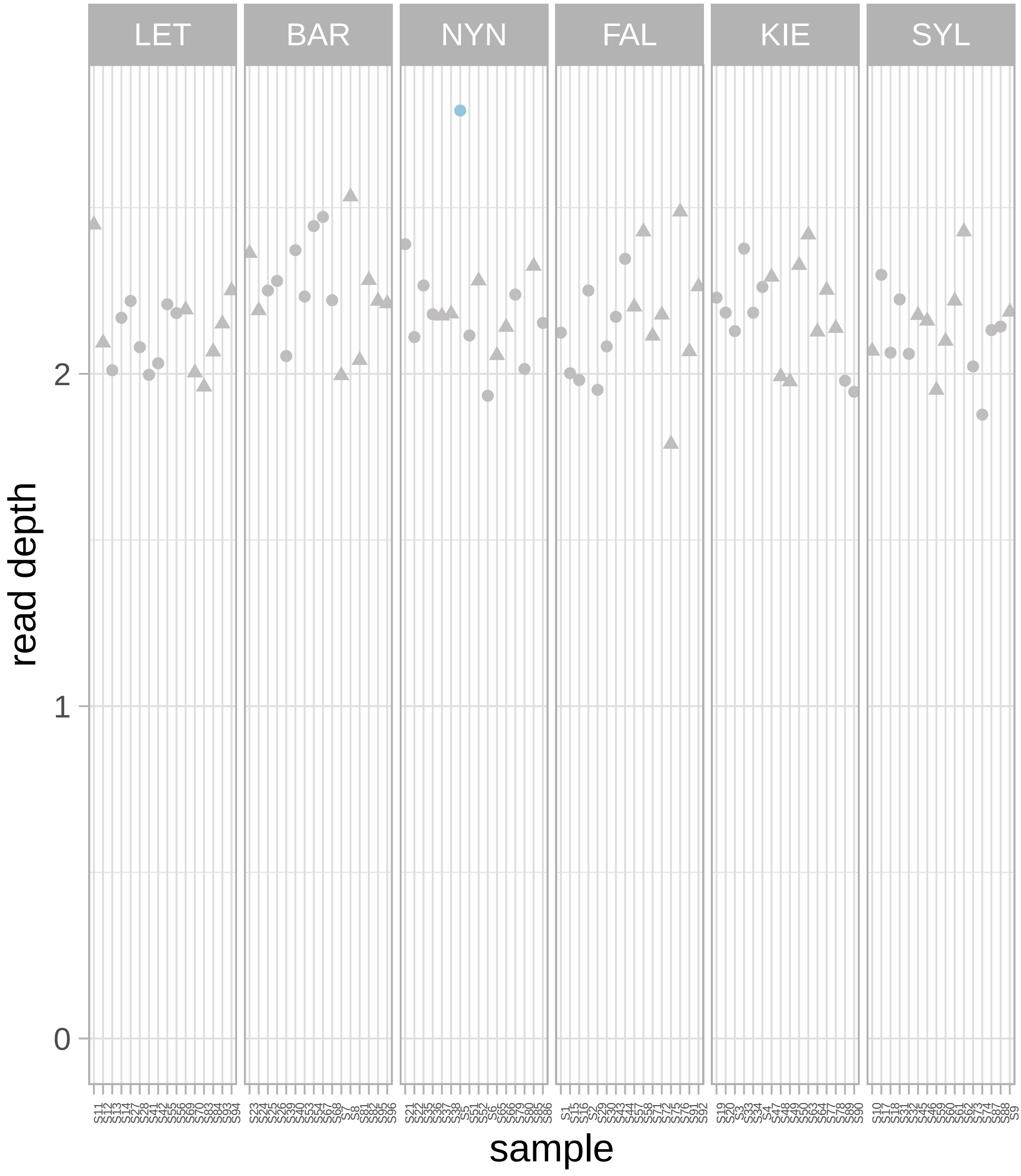

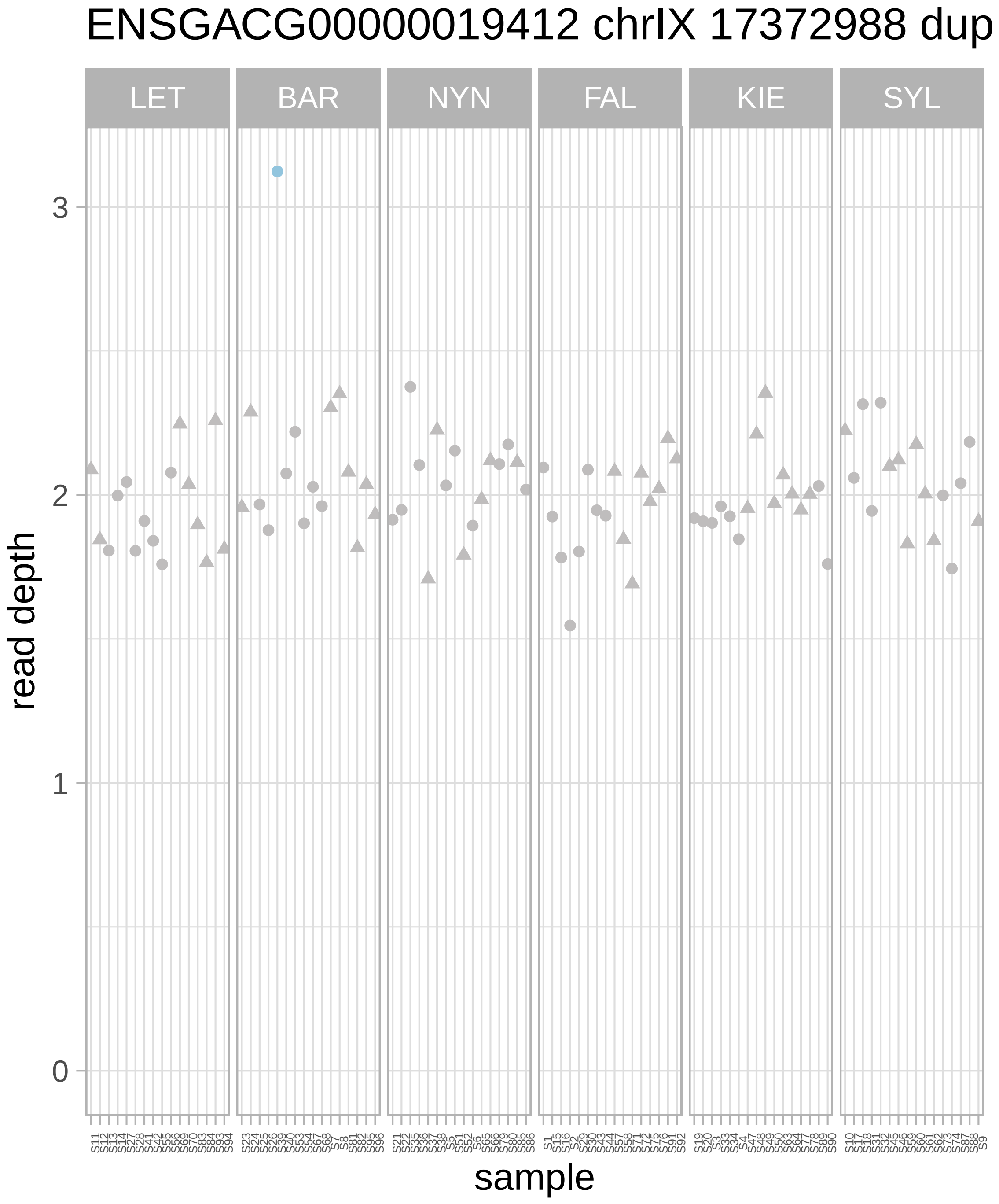
ENSGACG00000019412 chrIX 17372988 dup
LET
BAR
NYN
FAL
KIE
SYL
read depth
0
1
2
3
S11 S12 S13 S14 S27 S28 S41 S42 S55 S56 S69 S70 S83 S84 S93 S94
S23 S24 S25 S26 S39 S40 S53 S54 S67 S68 S7 S8 S81 S82 S95 S96
S21 S22 S35 S36 S37 S38 S5 S51 S52 S6 S65 S66 S79 S80 S85 S86
S1 S15 S16 S2 S29 S30 S43 S44 S57 S58 S71 S72 S75 S76 S91 S92
S19 S20 S3 S33 S34 S4 S47 S48 S49 S50 S63 S64 S77 S78 S89 S90
S10 S17 S18 S31 S32 S45 S46 S59 S60 S61 S62 S73 S74 S87 S88 S9
sample

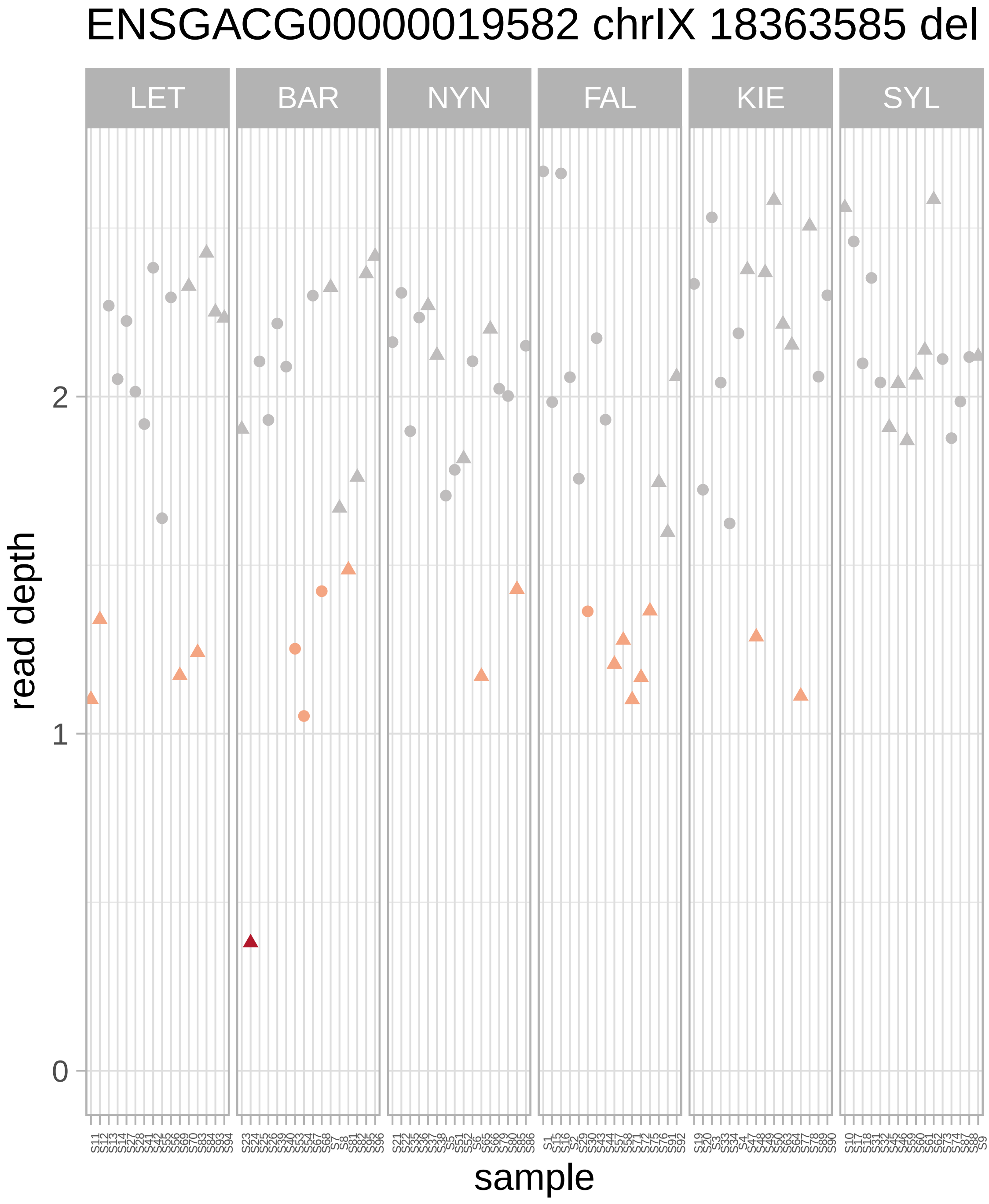
ENSGACG00000019582 chrIX 18363585 del
LET
BAR
NYN
FAL
KIE
SYL
read depth
0
1
2
S11 S12 S13 S14 S27 S28 S41 S42 S55 S56 S69 S70 S83 S84 S93 S94
S23 S24 S25 S26 S39 S40 S53 S54 S67 S68 S7 S8 S81 S82 S95 S96
S21 S22 S35 S36 S37 S38 S5 S51 S52 S6 S65 S66 S79 S80 S85 S86
S1 S15 S16 S2 S29 S30 S43 S44 S57 S58 S71 S72 S75 S76 S91 S92
S19 S20 S3 S33 S34 S4 S47 S48 S49 S50 S63 S64 S77 S78 S89 S90
S10 S17 S18 S31 S32 S45 S46 S59 S60 S61 S62 S73 S74 S87 S88 S9
sample

ENSGACG00000019595 chrIX 18428700 dup

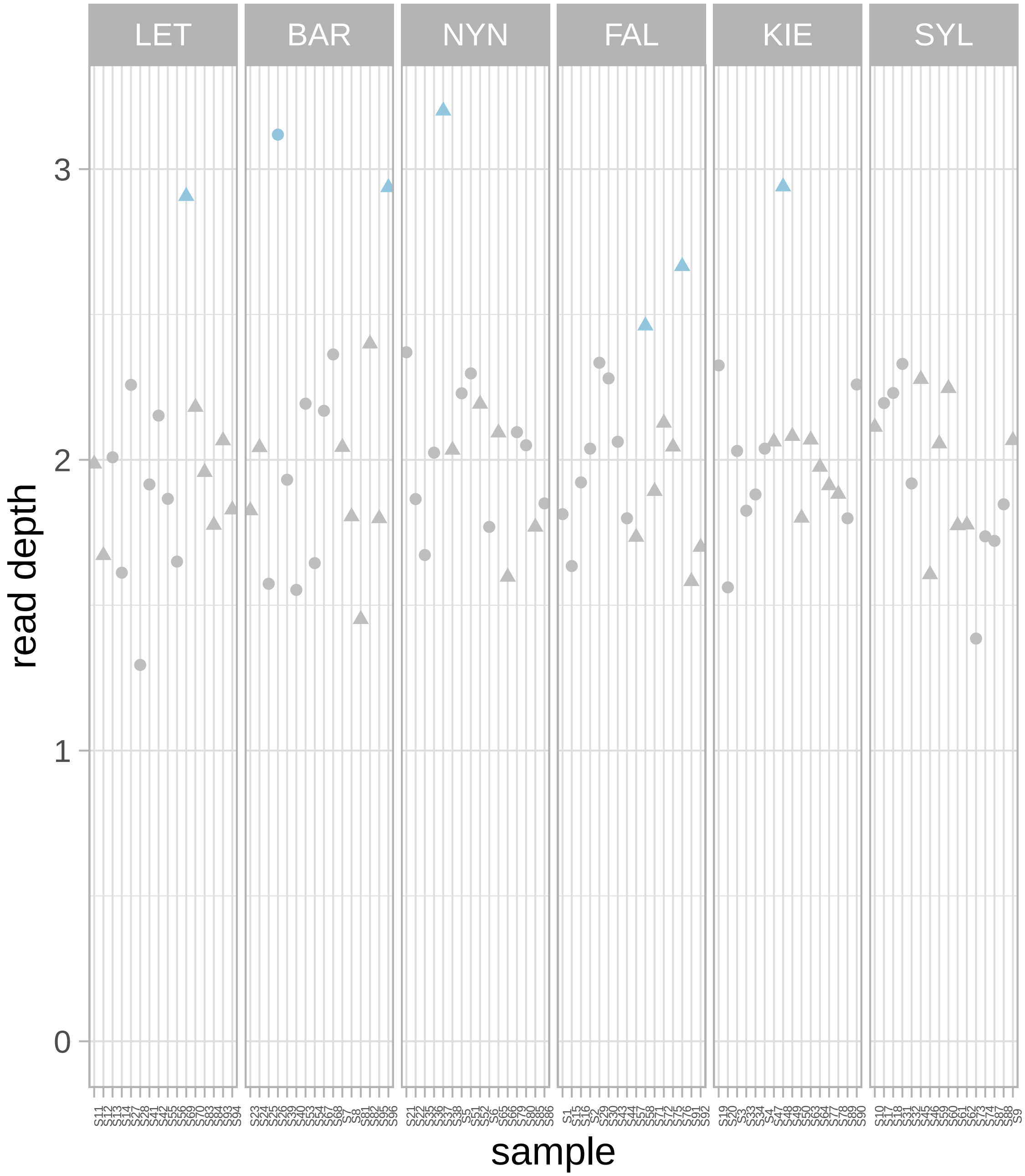

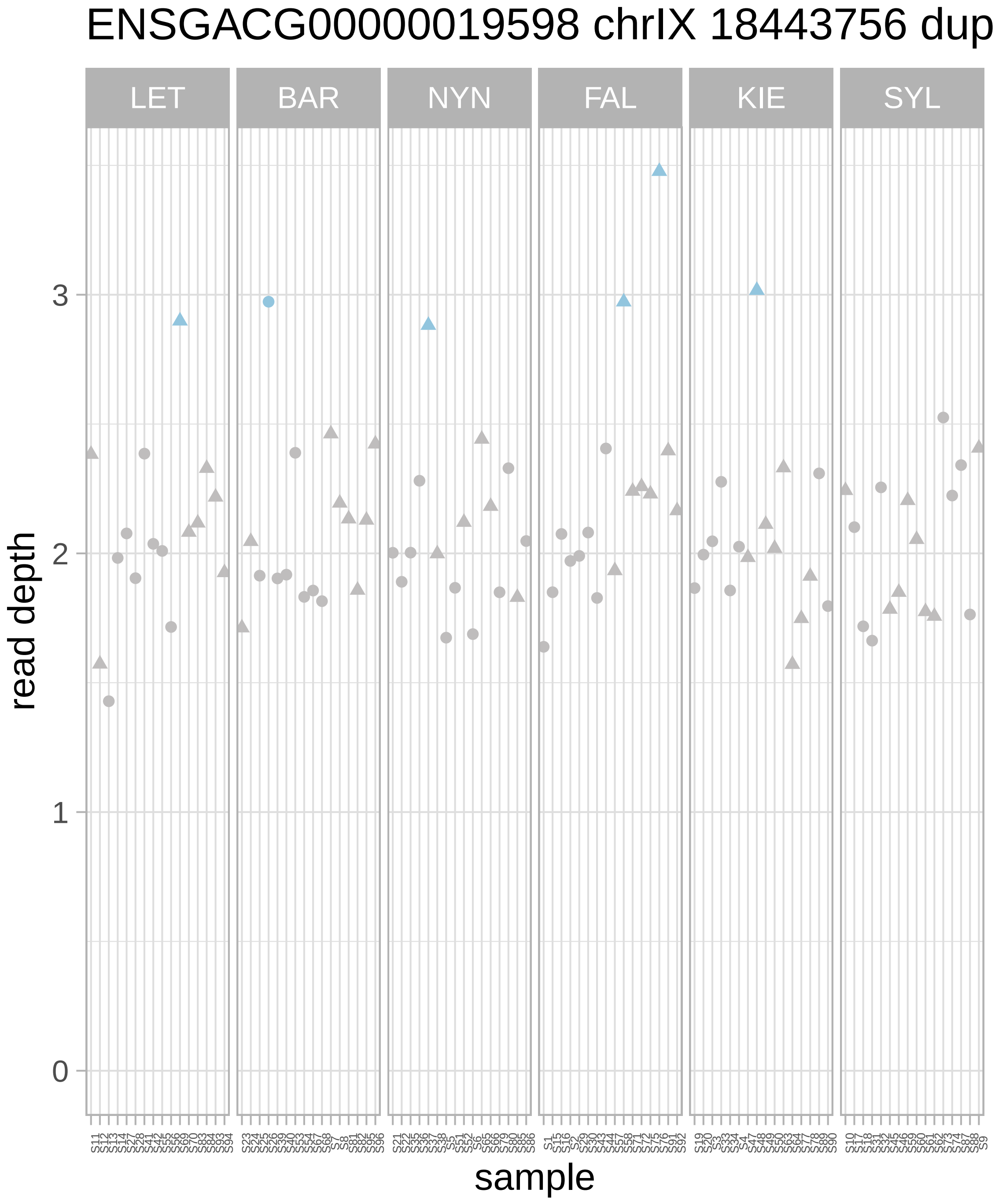
ENSGACG00000019598 chrIX 18443756 dup
LET
BAR
NYN
FAL
KIE
SYL
read depth
0
1
2
3
sample
S11 S12 S13 S14 S27 S28 S41 S42 S55 S56 S69 S70 S83 S84 S93 S94
S23 S24 S25 S26 S39 S40 S53 S54 S67 S68 S7 S8 S81 S82 S95 S96
S21 S22 S35 S36 S37 S38 S5 S51 S52 S6 S65 S66 S79 S80 S85 S86
S1 S15 S16 S2 S29 S30 S43 S44 S57 S58 S71 S72 S75 S76 S91 S92
S19 S20 S3 S33 S34 S4 S47 S48 S49 S50 S63 S64 S77 S78 S89 S90
S10 S17 S18 S31 S32 S45 S46 S59 S60 S61 S62 S73 S74 S87 S88 S9

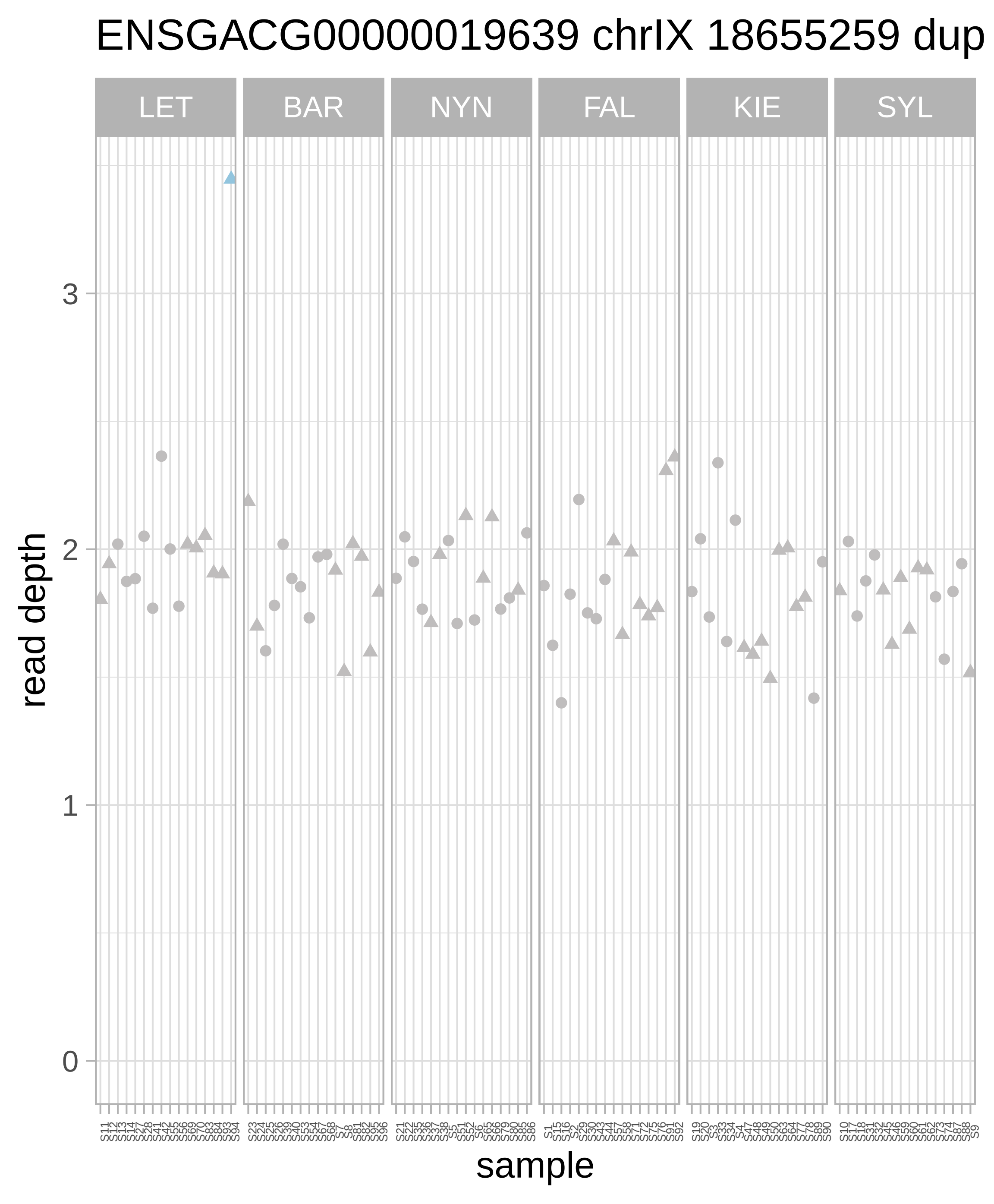
ENSGACG00000019639 chrIX 18655259 dup
LET
BAR
NYN
FAL
KIE
SYL
read depth
3
2
1
0
sample

ENSGACG00000019641 chrIX 18661206 dup

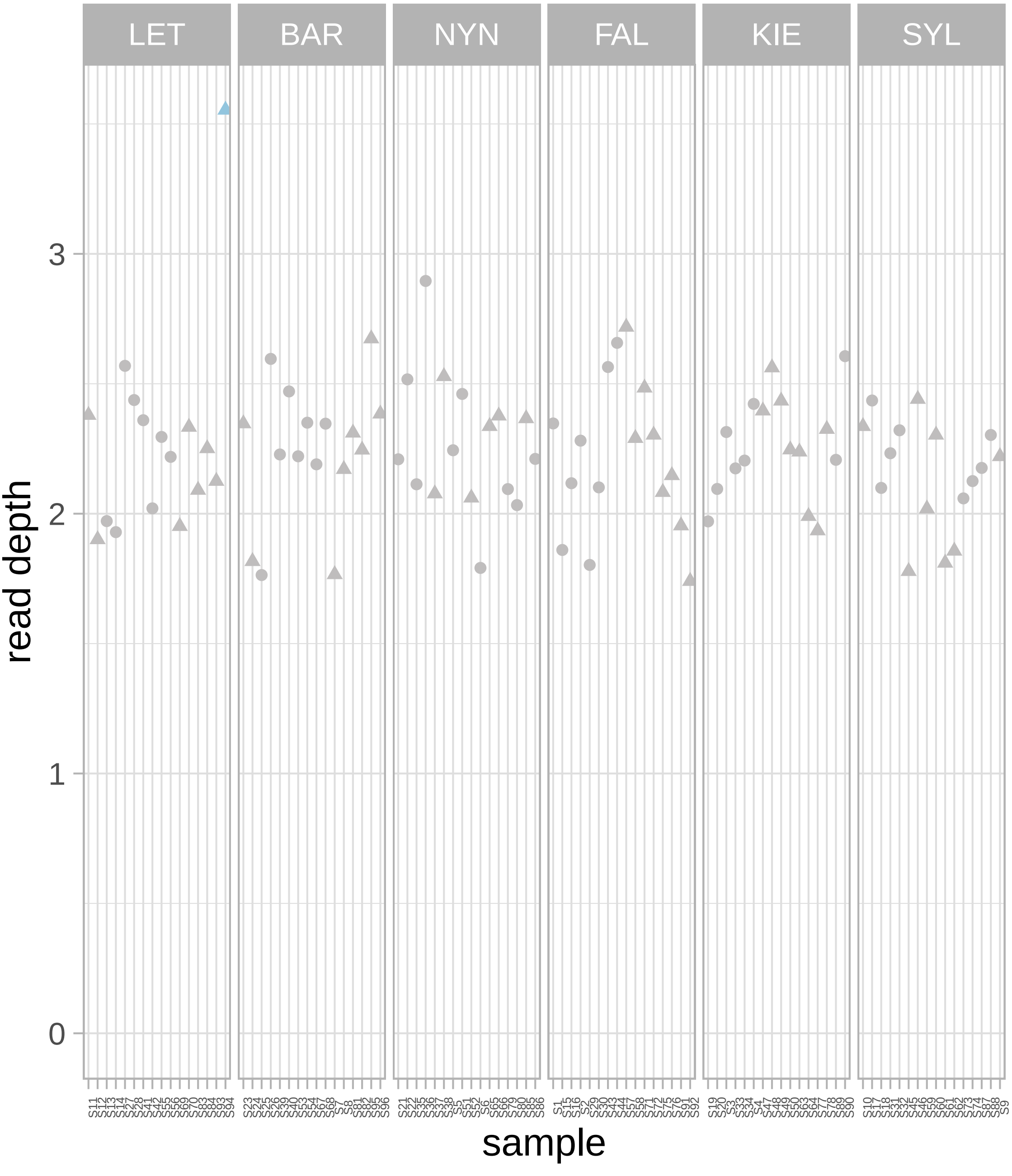

ENSGACG00000019780 chrIX 19478876 dup

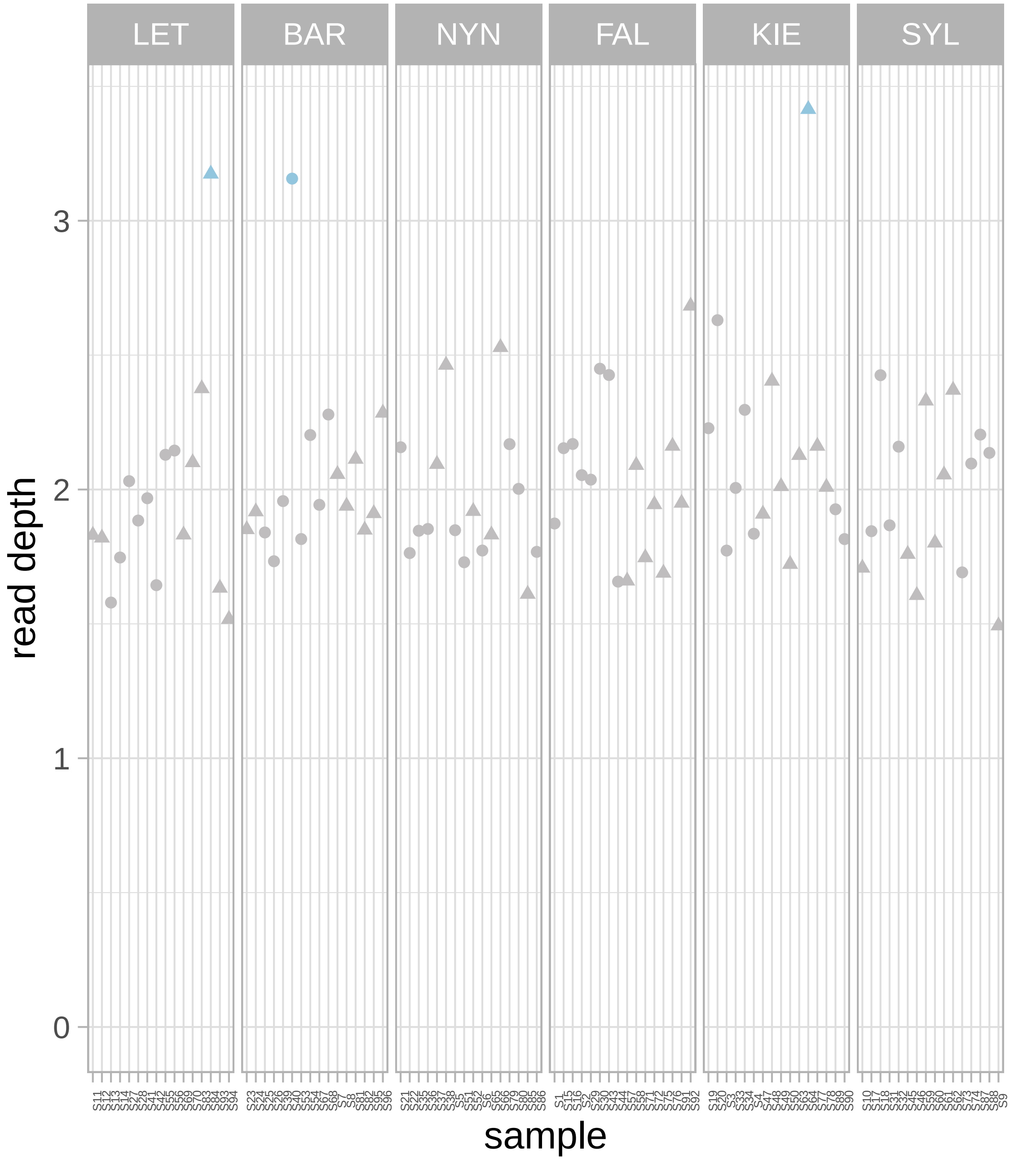

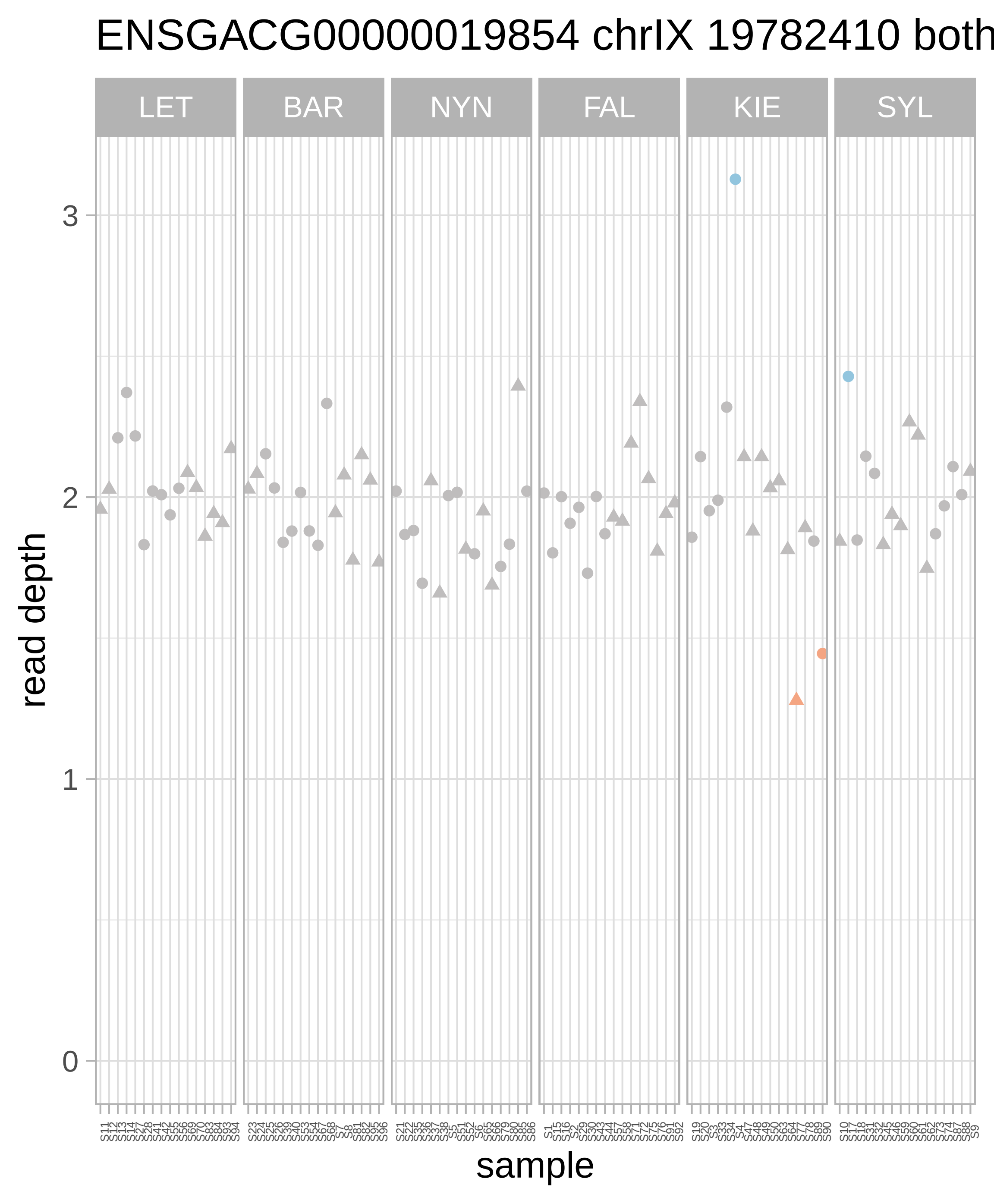
ENSGACG00000019854 chrIX 19782410 both
LET
BAR
NYN
FAL
KIE
SYL
read depth
0
1
2
3
sample

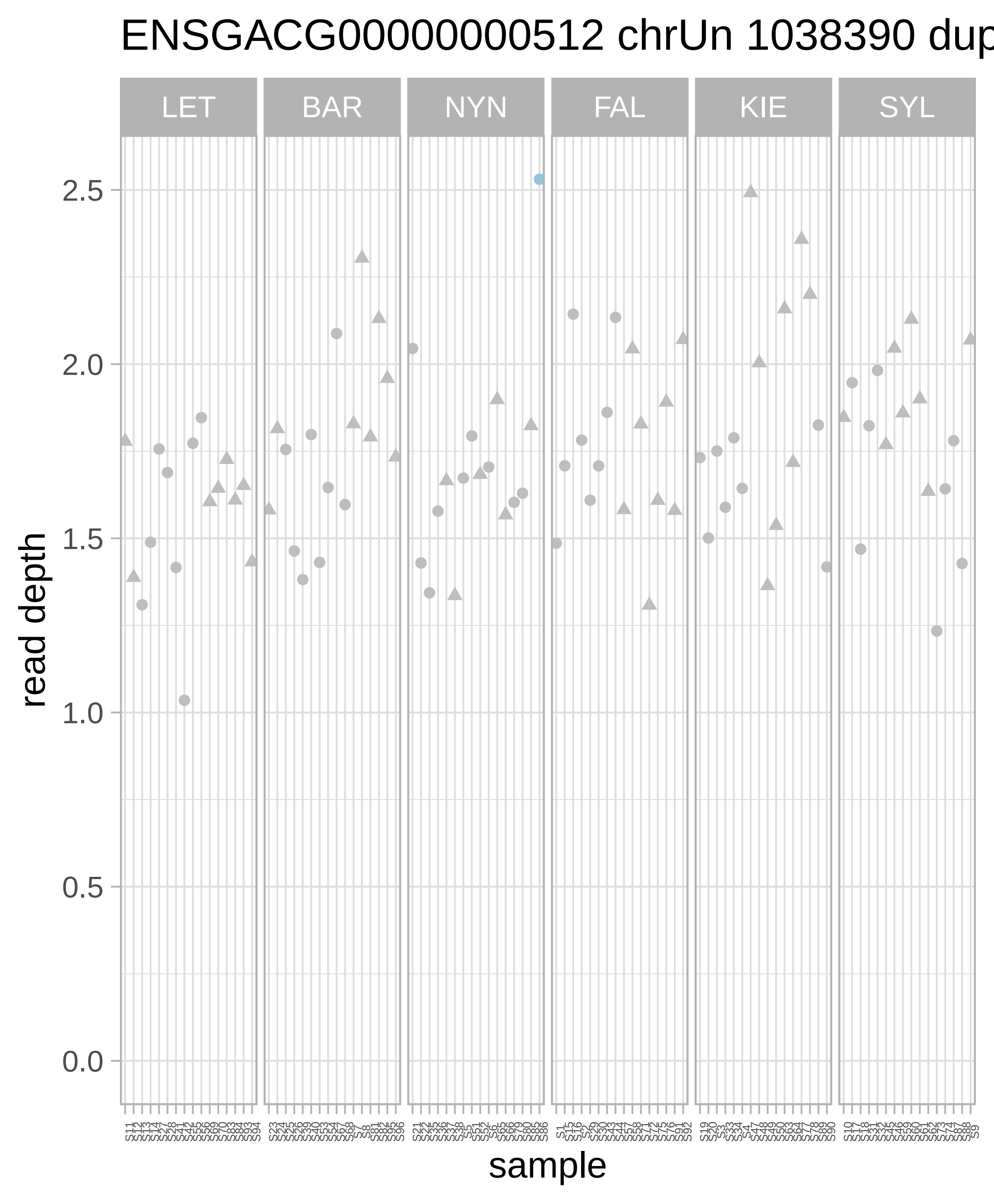

ENSGACG00000000512 chrUn 1038390 dup
LET
BAR
NYN
FAL
KIE
SYL
read depth
sample
0.0
0.5
1.0
1.5
2.0
2.5

ENSGACG00000000516 chrUn 1042506 dup

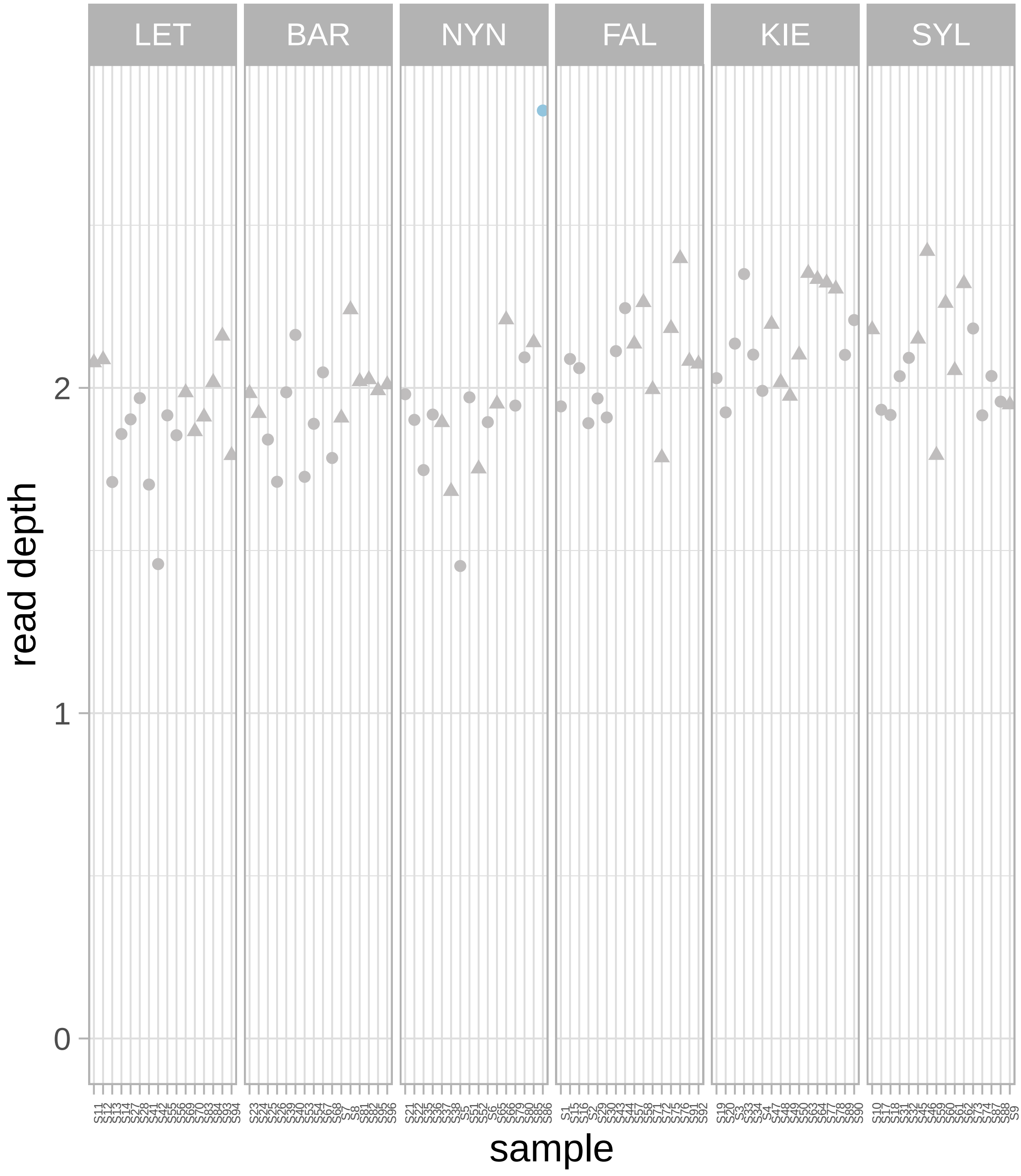

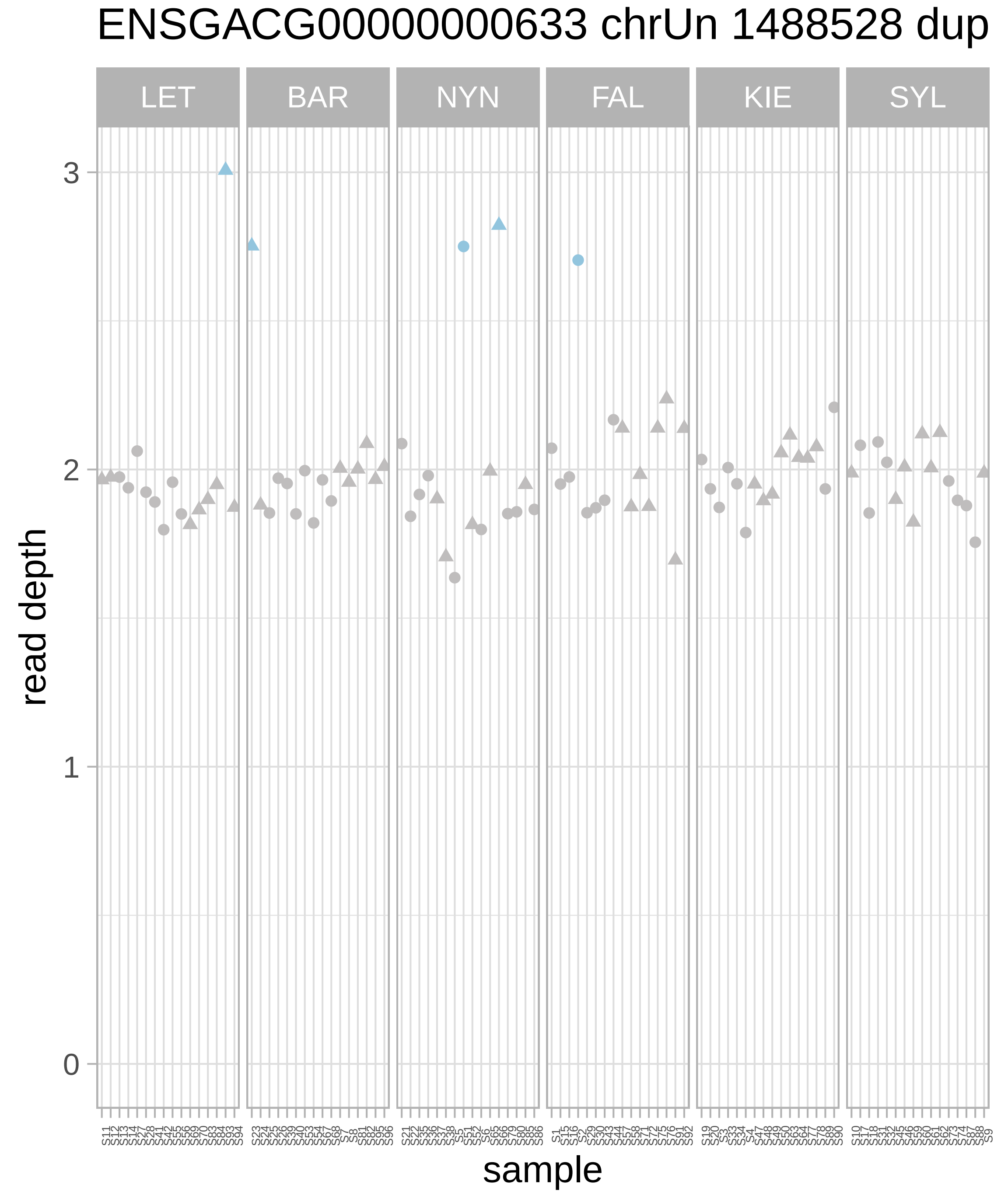
ENSGACG00000000633 chrUn 1488528 dup
LET
BAR
NYN
FAL
KIE
SYL
read depth
3
2
1
0
S11 S12 S13 S14 S27 S28 S41 S42 S55 S56 S69 S70 S83 S84 S93 S94
S23 S24 S25 S26 S39 S40 S53 S54 S67 S68 S7 S8 S81 S82 S95 S96
S21 S22 S35 S36 S37 S38 S5 S51 S52 S6 S65 S66 S79 S80 S85 S86
S1 S15 S16 S2 S29 S30 S43 S44 S57 S58 S71 S72 S75 S76 S91 S92
S19 S20 S3 S33 S34 S4 S47 S48 S49 S50 S63 S64 S77 S78 S89 S90
S10 S17 S18 S31 S32 S45 S46 S59 S60 S61 S62 S73 S74 S87 S88 S9
sample

ENSGACG00000000813 chrUn 2235630 dup

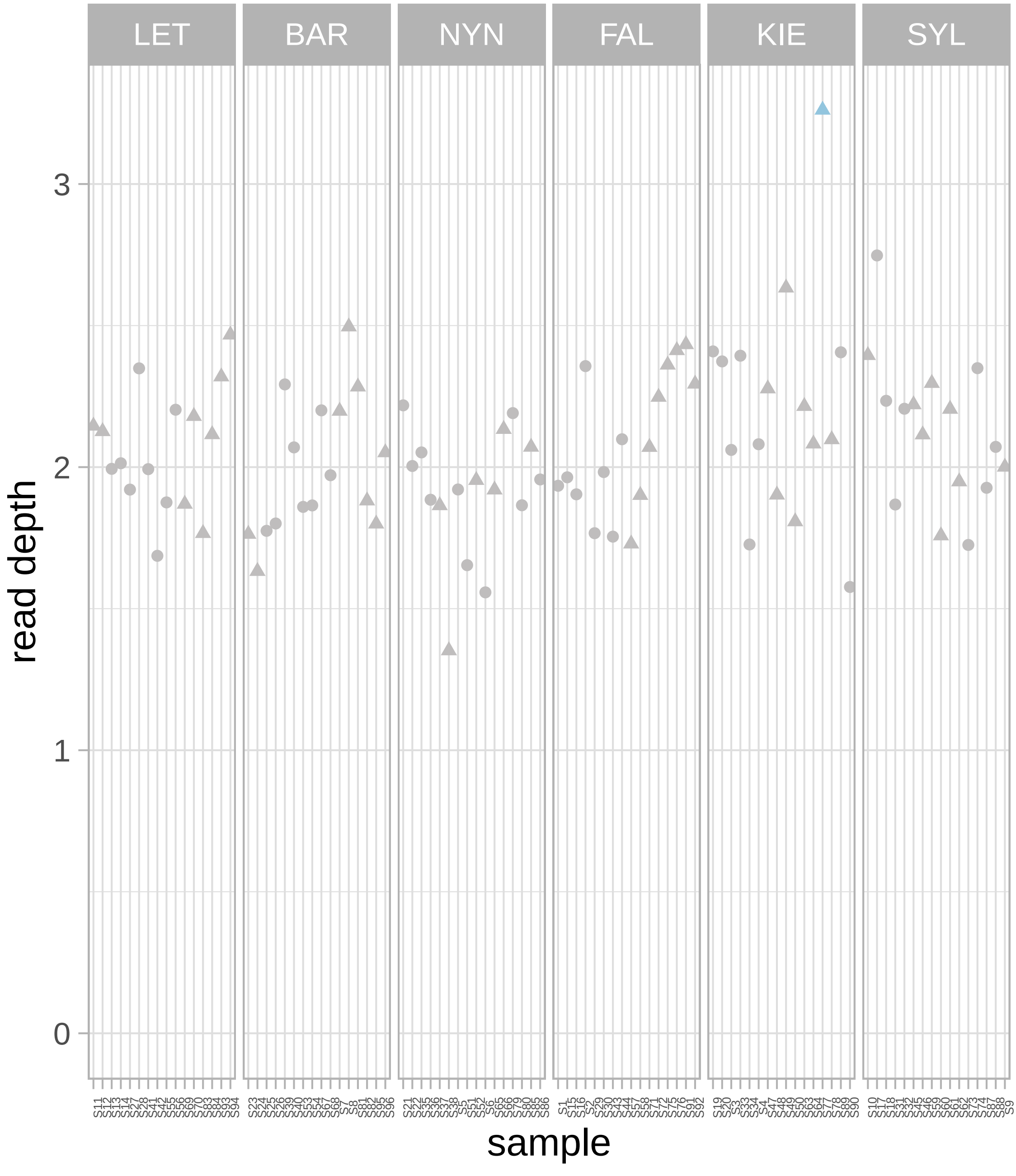

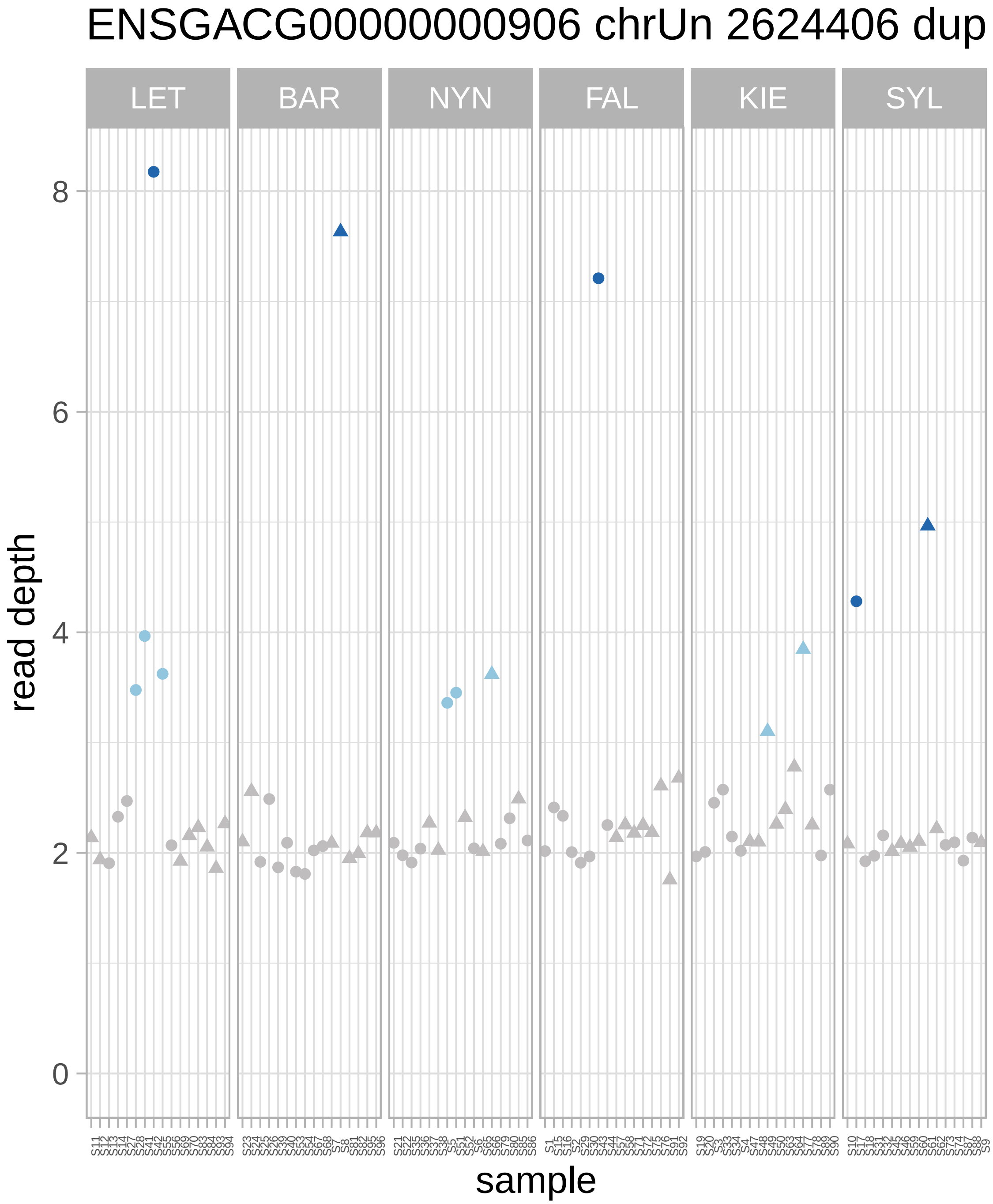
ENSGACG00000000906 chrUn 2624406 dup
LET
BAR
NYN
FAL
KIE
SYL
read depth
8
6
4
2
0
S11 S12 S13 S14 S27 S28 S41 S42 S55 S56 S69 S70 S83 S84 S93 S94
S23 S24 S25 S26 S39 S40 S53 S54 S67 S68 S7 S8 S81 S82 S95 S96
S21 S22 S35 S36 S37 S38 S5 S51 S52 S6 S65 S66 S79 S80 S85 S86
S1 S15 S16 S2 S29 S30 S43 S44 S57 S58 S71 S72 S75 S76 S91 S92
S19 S20 S3 S33 S34 S4 S47 S48 S49 S50 S63 S64 S77 S78 S89 S90
S10 S17 S18 S31 S32 S45 S46 S59 S60 S61 S62 S73 S74 S87 S88 S9
sample

ENSGACG00000000920 chrUn 2676591 dup

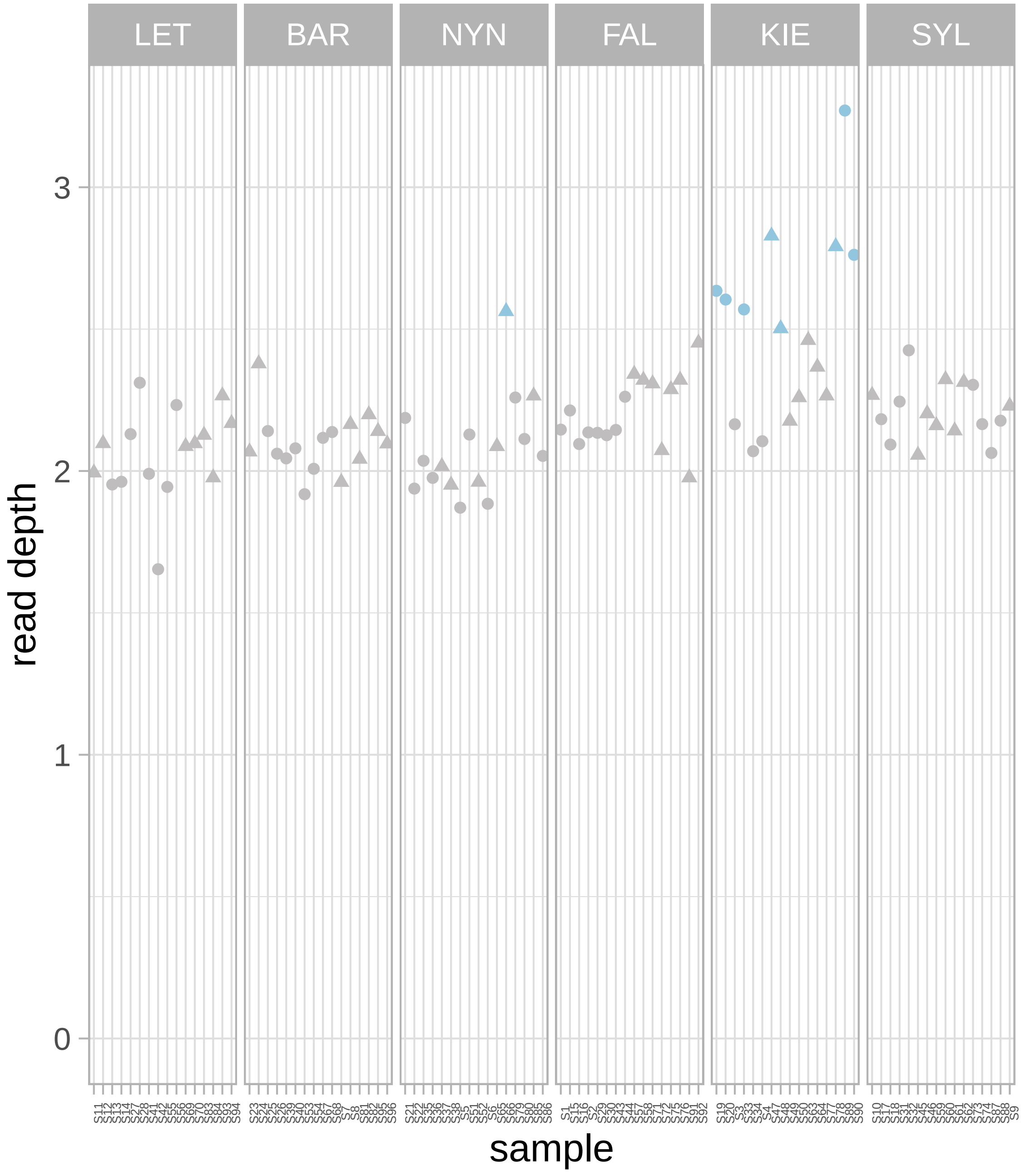

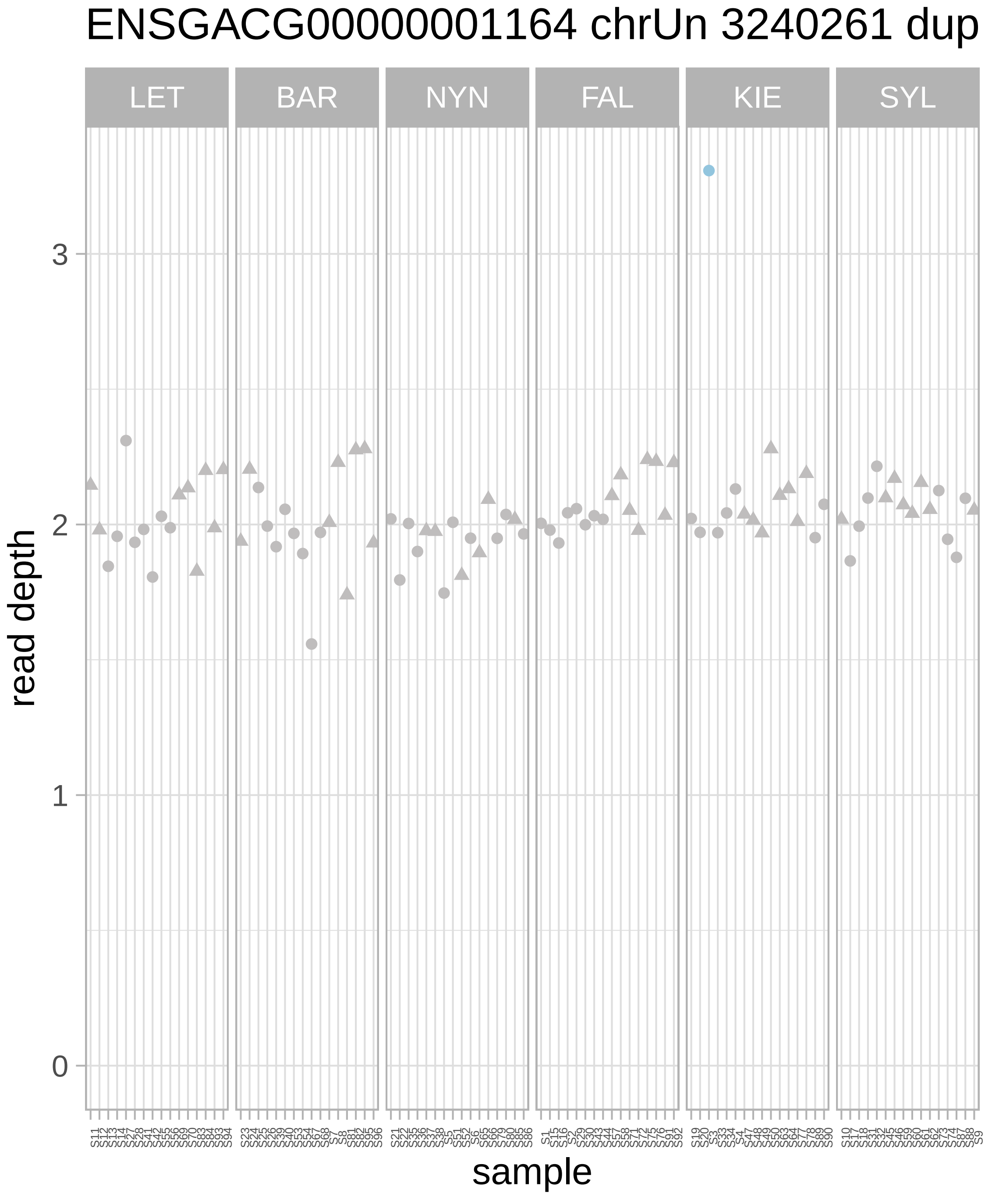
ENSGACG00000001164 chrUn 3240261 dup
LET
BAR
NYN
FAL
KIE
SYL
read depth
0
1
2
3
sample
S11 S12 S13 S14 S27 S28 S41 S42 S55 S56 S69 S70 S83 S84 S93 S94
S23 S24 S25 S26 S39 S40 S53 S54 S67 S68 S7 S8 S81 S82 S95 S96
S21 S22 S35 S36 S37 S38 S5 S51 S52 S6 S65 S66 S79 S80 S85 S86
S1 S15 S16 S2 S29 S30 S43 S44 S57 S58 S71 S72 S75 S76 S91 S92
S19 S20 S3 S33 S34 S4 S47 S48 S49 S50 S63 S64 S77 S78 S89 S90
S10 S17 S18 S31 S32 S45 S46 S59 S60 S61 S62 S73 S74 S87 S88 S9

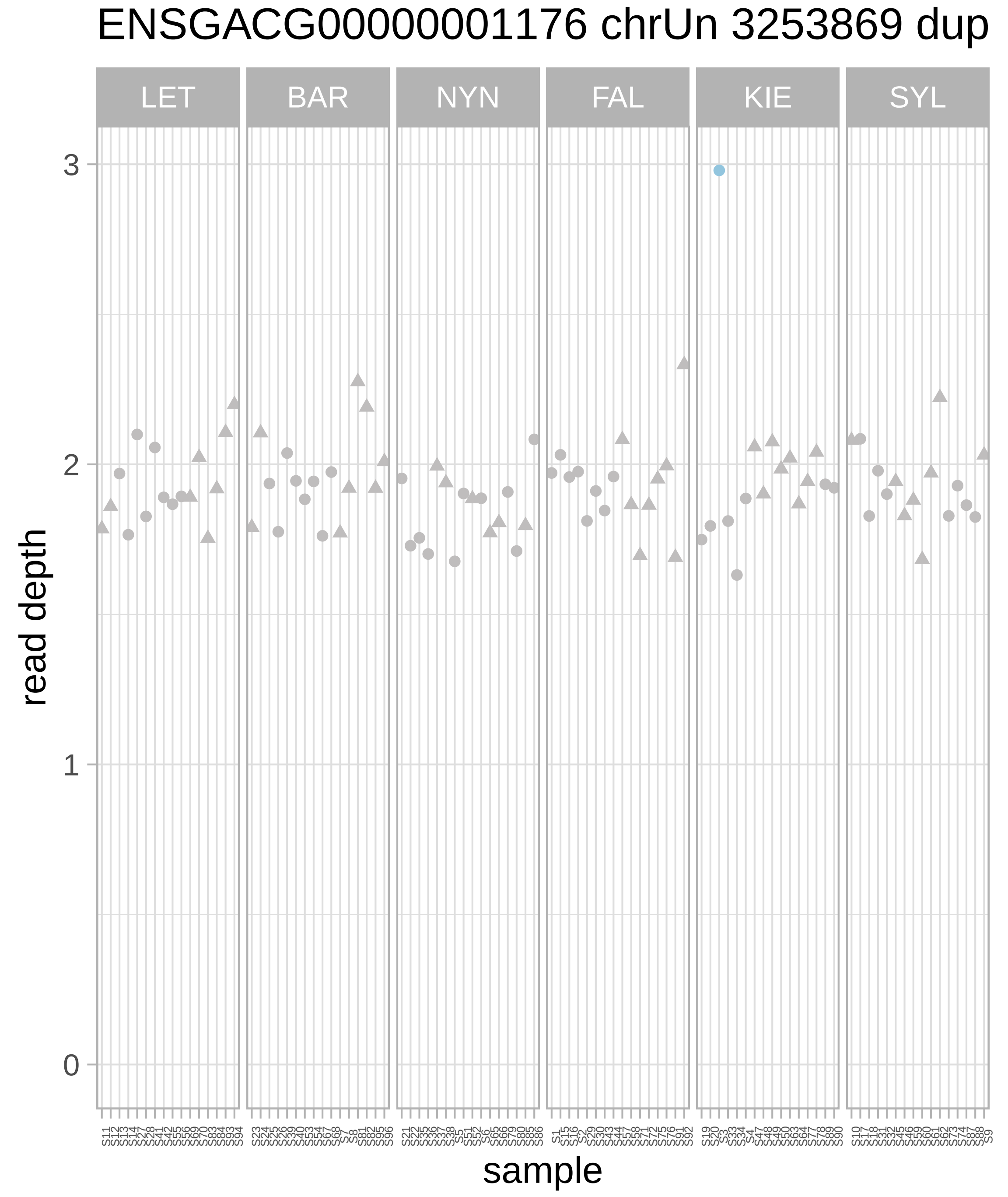
ENSGACG00000001176 chrUn 3253869 dup
LET
BAR
NYN
FAL
KIE
SYL
read depth
0
1
2
3
sample

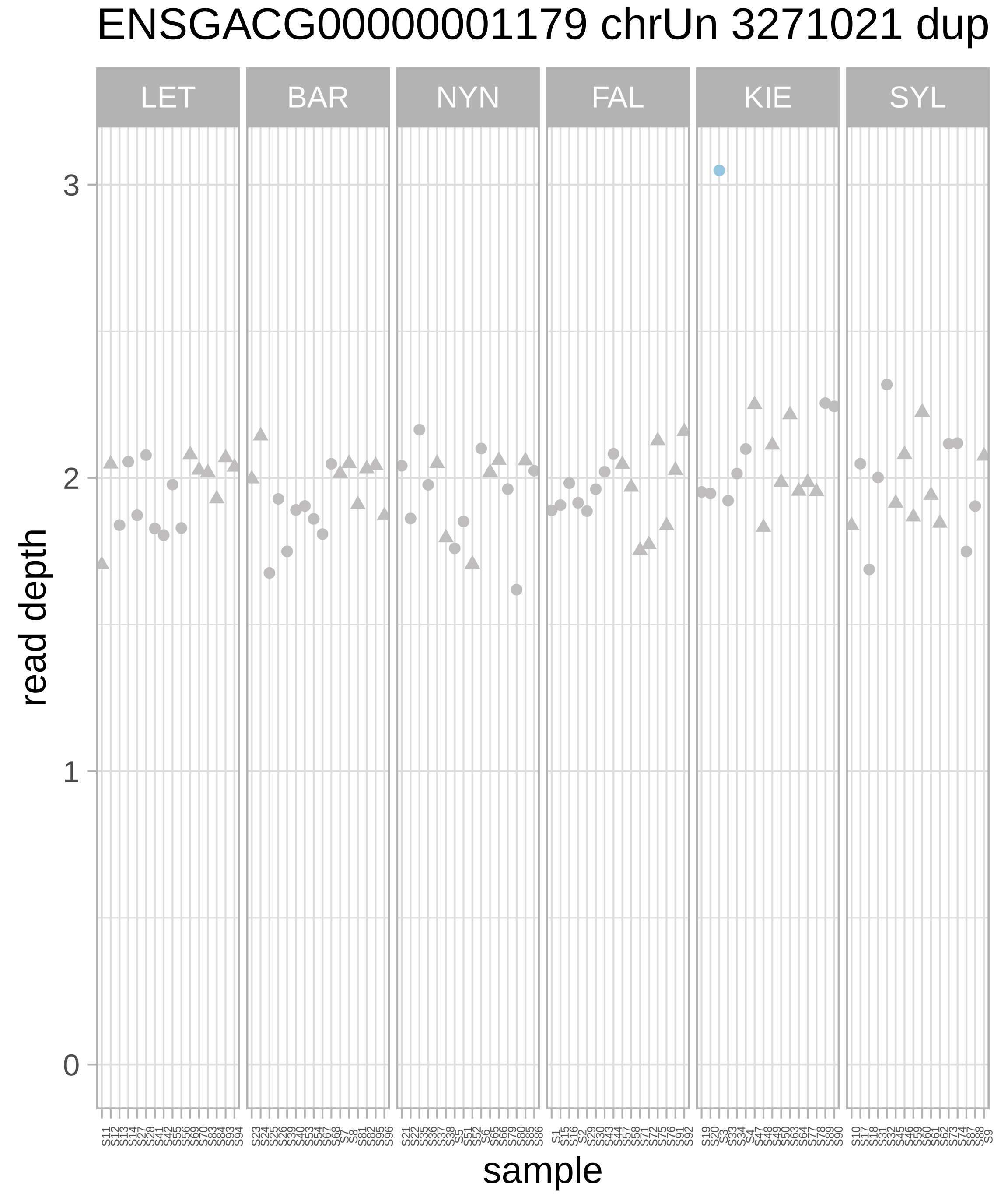
ENSGACG00000001179 chrUn 3271021 dup
LET
BAR
NYN
FAL
KIE
SYL
read depth
0
1
2
3
sample
S11 S12 S13 S14 S27 S28 S41 S42 S55 S56 S69 S70 S83 S84 S93 S94
S23 S24 S25 S26 S39 S40 S53 S54 S67 S68 S7 S8 S81 S82 S95 S96
S21 S22 S35 S36 S37 S38 S5 S51 S52 S6 S65 S66 S79 S80 S85 S86
S1 S15 S16 S2 S29 S30 S43 S44 S57 S58 S71 S72 S75 S76 S91 S92
S19 S20 S3 S33 S34 S4 S47 S48 S49 S50 S63 S64 S77 S78 S89 S90
S10 S17 S18 S31 S32 S45 S46 S59 S60 S61 S62 S73 S74 S87 S88 S9

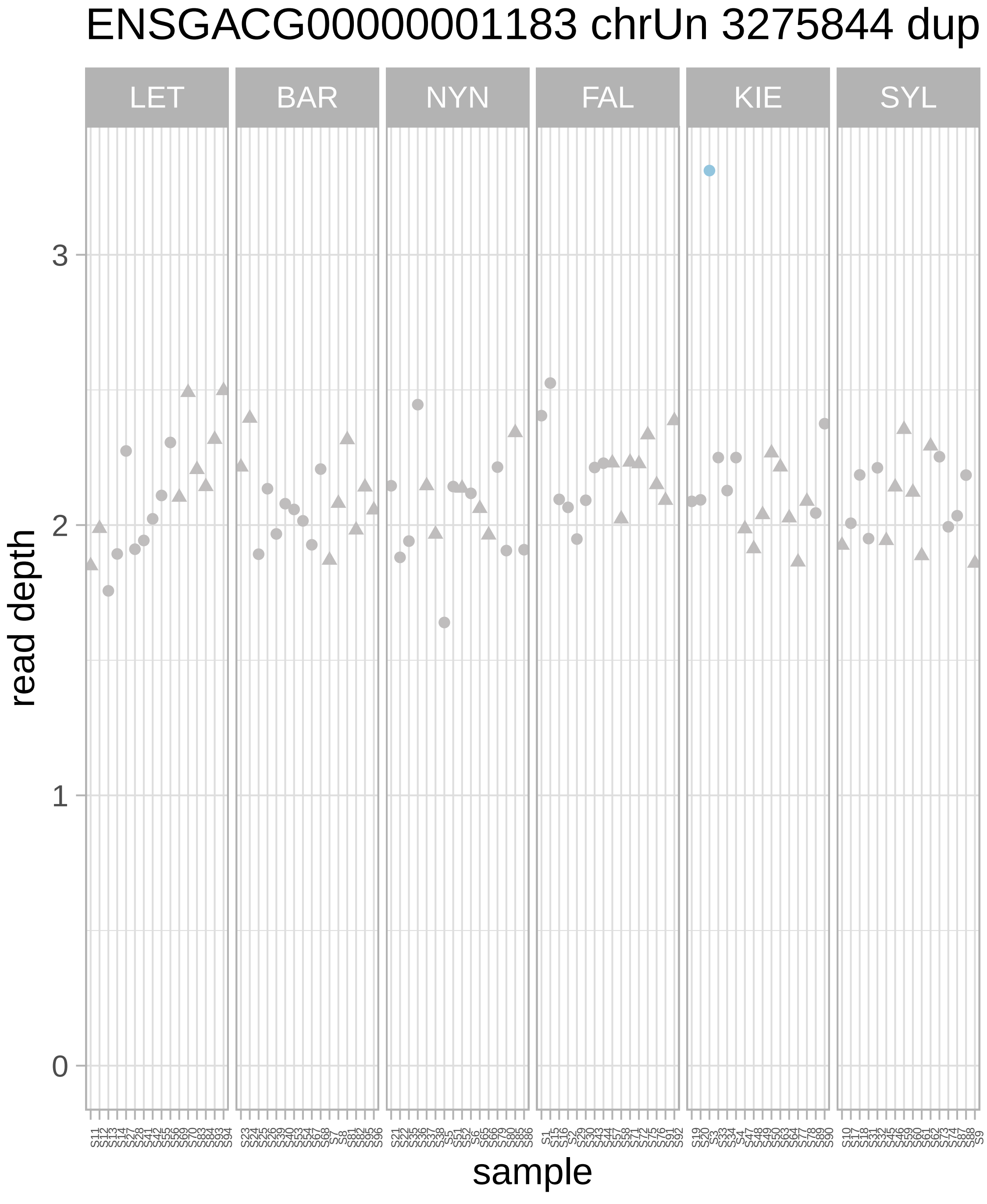
ENSGACG00000001183 chrUn 3275844 dup
LET
BAR
NYN
FAL
KIE
SYL
read depth
0
1
2
3
sample

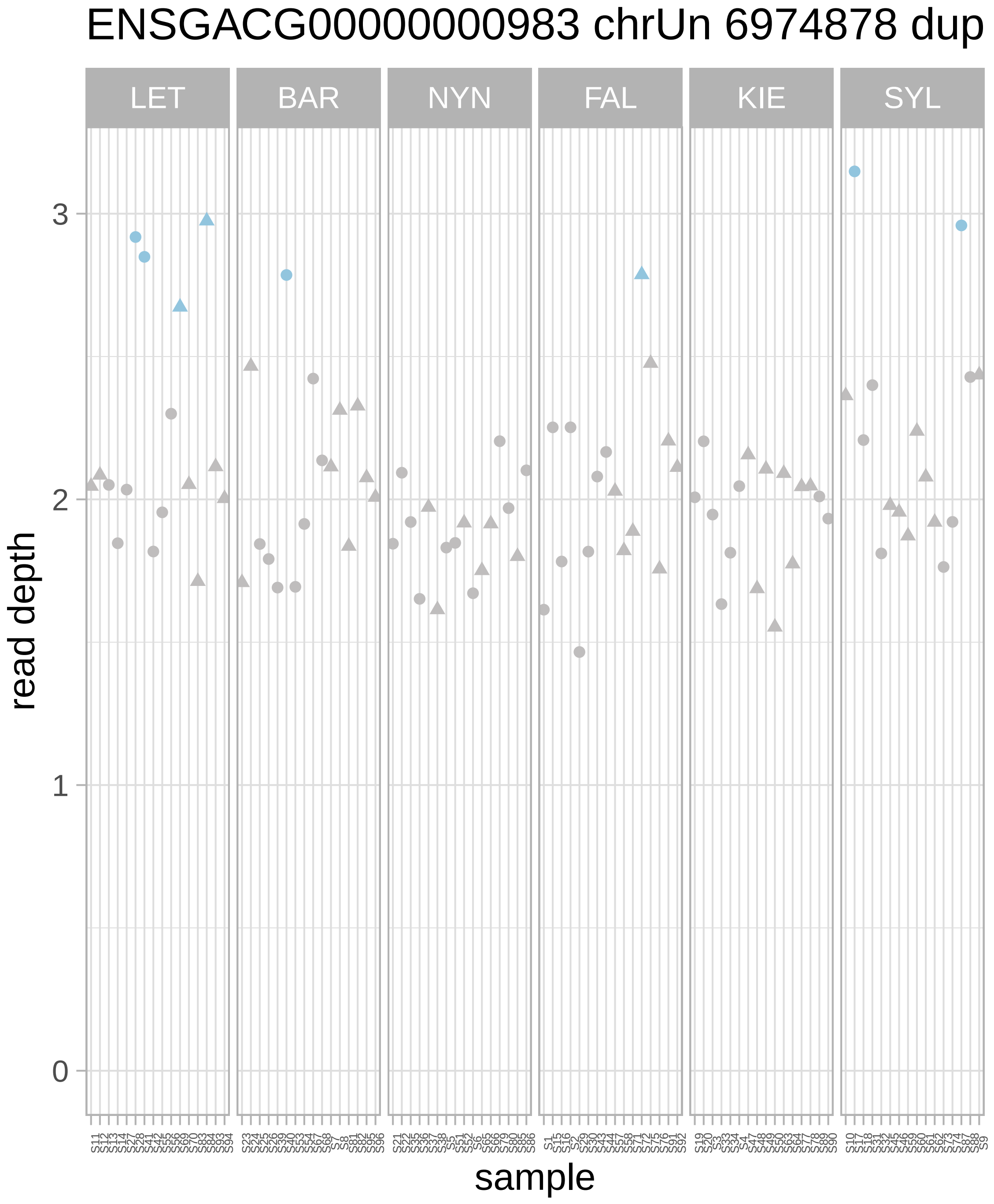

ENSGACG00000000983 chrUn 6974878 dup
LET
BAR
NYN
FAL
KIE
SYL
read depth
0
1
2
3
sample
S11 S12 S13 S14 S27 S28 S41 S42 S55 S56 S69 S70 S83 S84 S93 S94
S23 S24 S25 S26 S39 S40 S53 S54 S67 S68 S7 S8 S81 S82 S95 S96
S21 S22 S35 S36 S37 S38 S5 S51 S52 S6 S65 S66 S79 S80 S85 S86
S1 S15 S16 S2 S29 S30 S43 S44 S57 S58 S71 S72 S75 S76 S91 S92
S19 S20 S3 S33 S34 S4 S47 S48 S49 S50 S63 S64 S77 S78 S89 S90
S10 S17 S18 S31 S32 S45 S46 S59 S60 S61 S62 S73 S74 S87 S88 S9

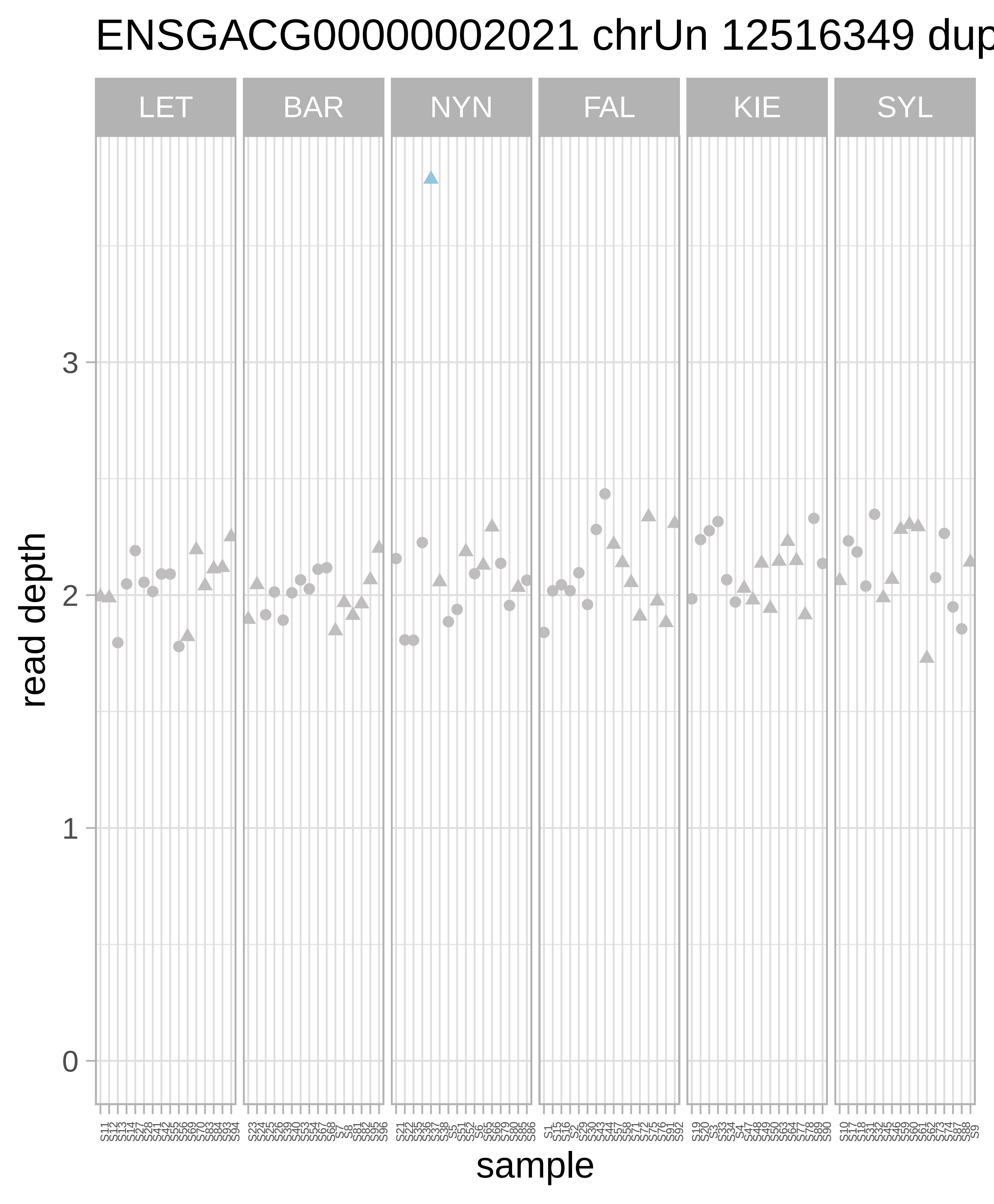
ENSGACG00000002021 chrUn 12516349 dup
LET
BAR
NYN
FAL
KIE
SYL
read depth
3
2
1
0
sample

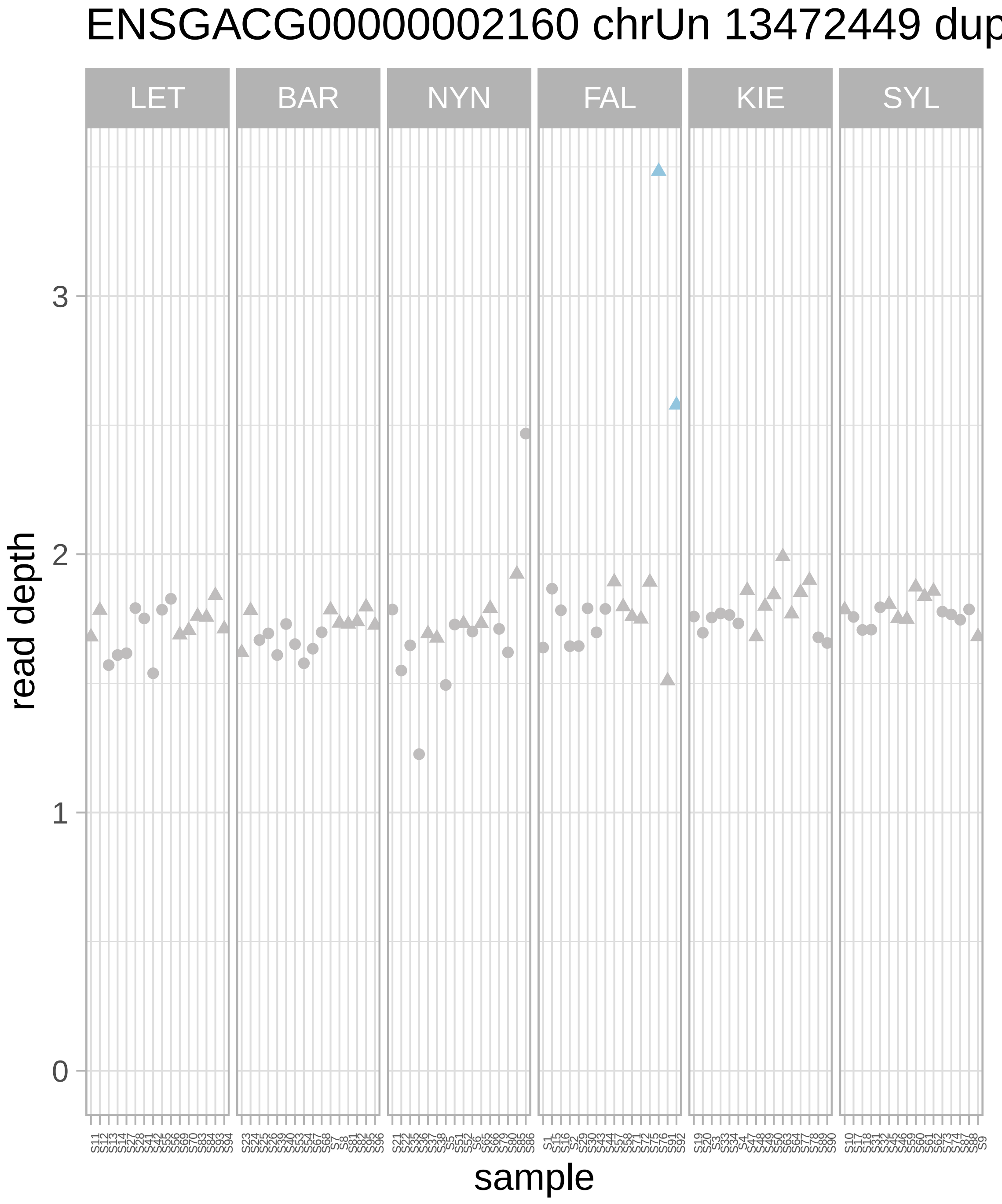
ENSGACG00000002160 chrUn 13472449 dup
LET
BAR
NYN
FAL
KIE
SYL
read depth
3
2
1
0
sample

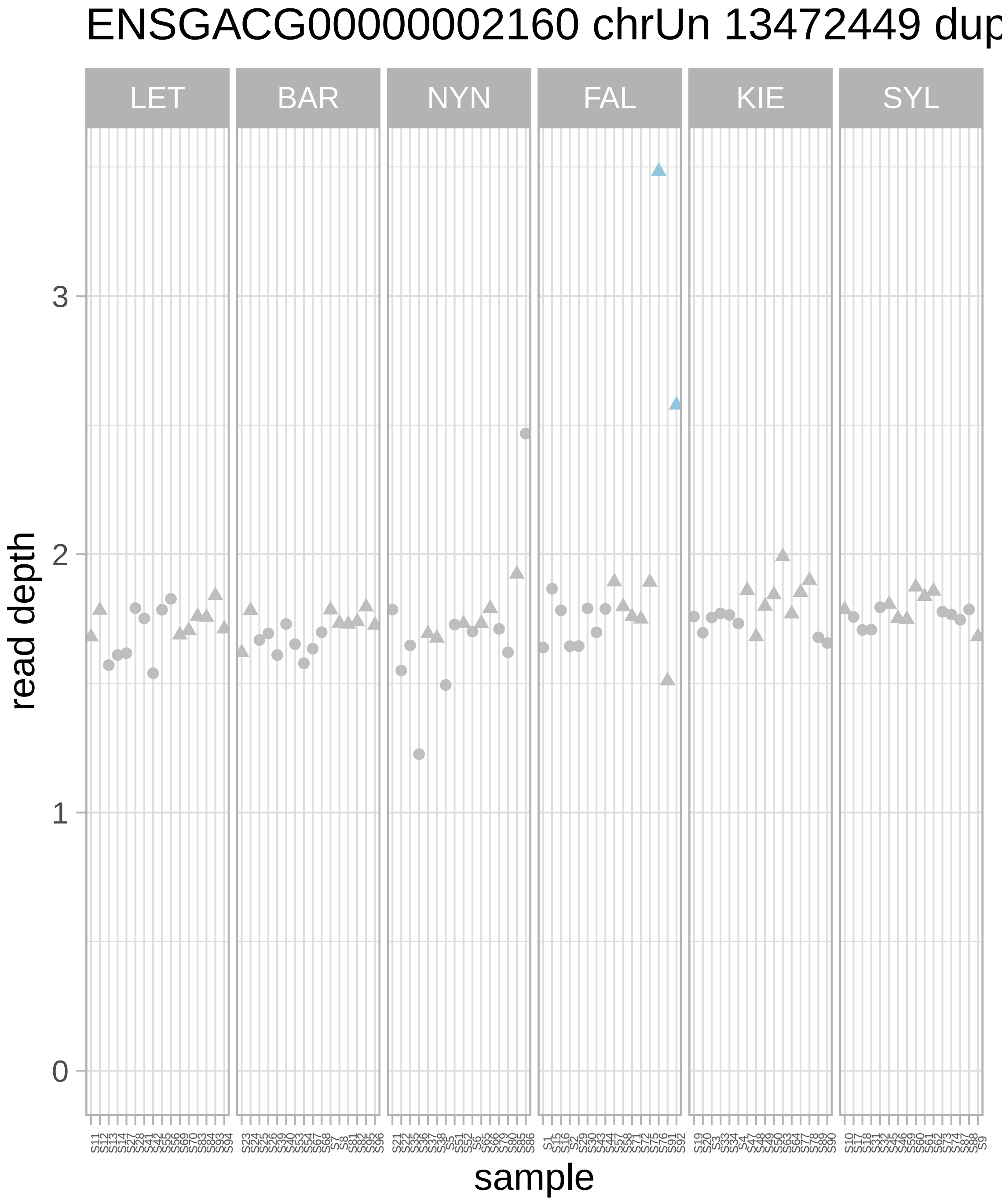
ENSGACG00000002160 chrUn 13472449 dup
LET
BAR
NYN
FAL
KIE
SYL
read depth
0
1
2
3
S11 S12 S13 S14 S27 S28 S41 S42 S55 S56 S69 S70 S83 S84 S93 S94
S23 S24 S25 S26 S39 S40 S53 S54 S67 S68 S7 S8 S81 S82 S95 S96
S21 S22 S35 S36 S37 S38 S5 S51 S52 S6 S65 S66 S79 S80 S85 S86
S1 S15 S16 S2 S29 S30 S43 S44 S57 S58 S71 S72 S75 S76 S91 S92
S19 S20 S3 S33 S34 S4 S47 S48 S49 S50 S63 S64 S77 S78 S89 S90
S10 S17 S18 S31 S32 S45 S46 S59 S60 S61 S62 S73 S74 S87 S88 S9
sample

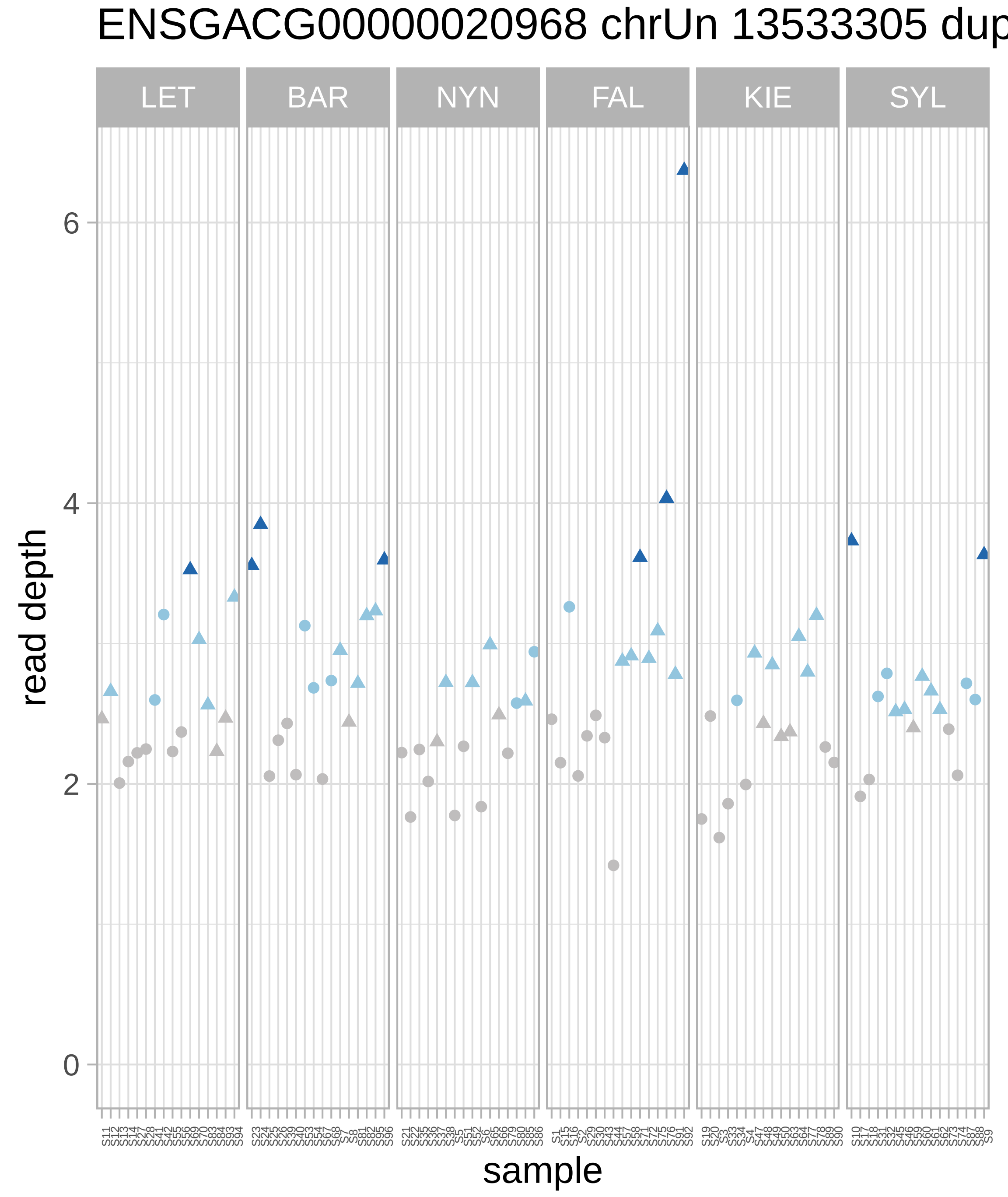
ENSGACG00000020968 chrUn 13533305 dup
LET
BAR
NYN
FAL
KIE
SYL
read depth
0
2
4
6
sample

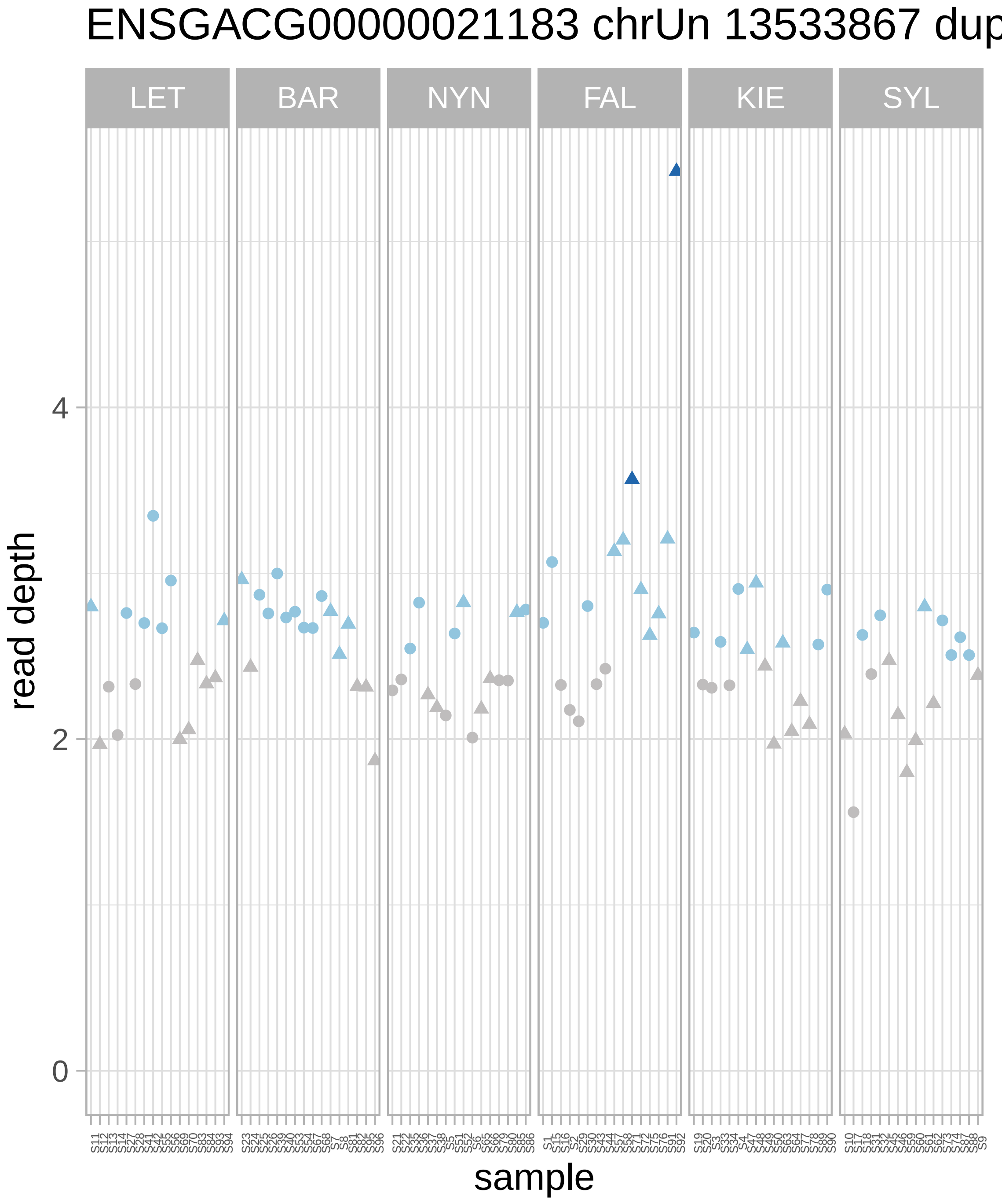
ENSGACG00000021183 chrUn 13533867 dup
LET
BAR
NYN
FAL
KIE
SYL
read depth
0
2
4
sample

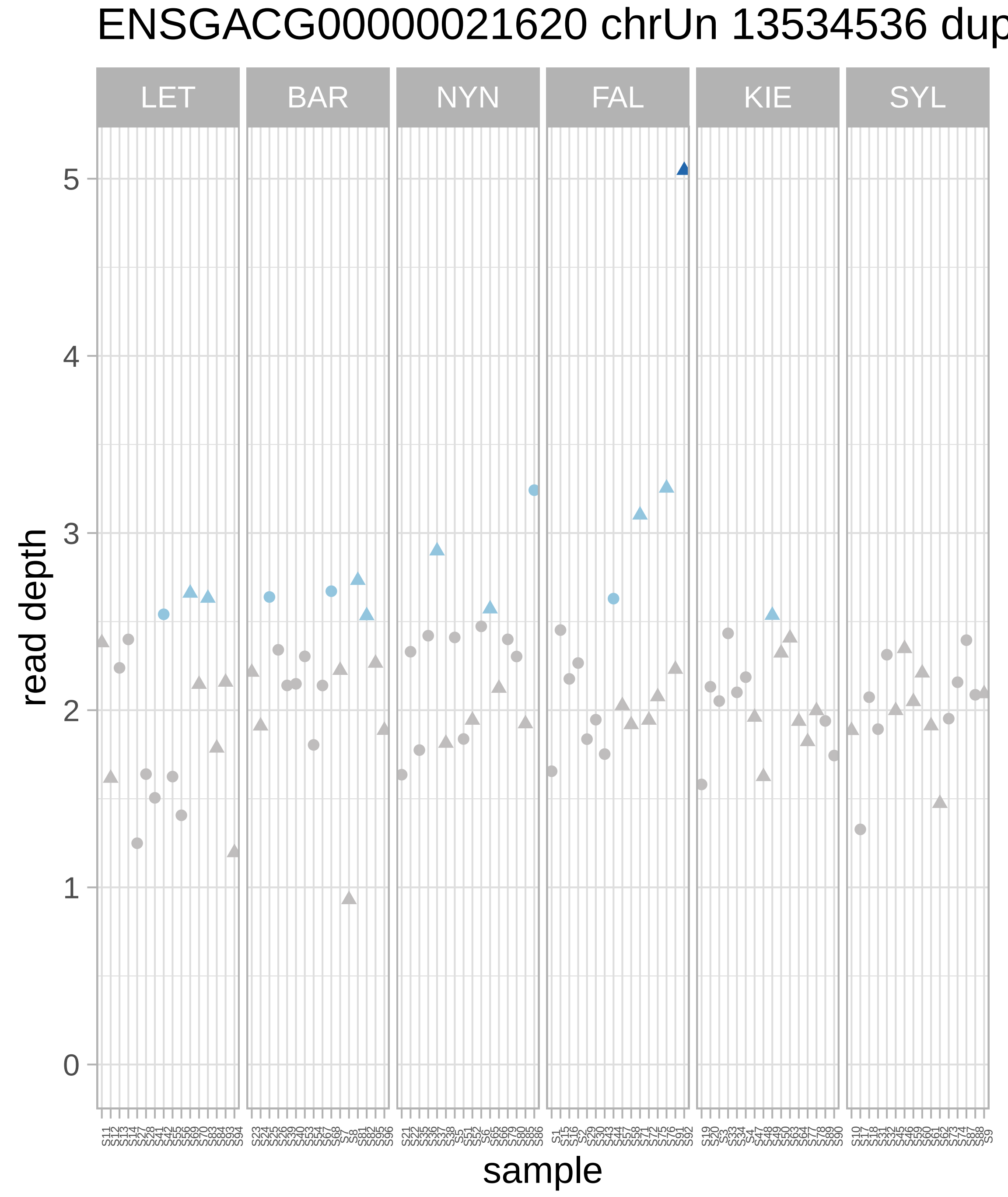
ENSGACG00000021620 chrUn 13534536 dup
LET
BAR
NYN
FAL
KIE
SYL
read depth
0
1
2
3
4
5
S11 S12 S13 S14 S27 S28 S41 S42 S55 S56 S69 S70 S83 S84 S93 S94
S23 S24 S25 S26 S39 S40 S53 S54 S67 S68 S7 S8 S81 S82 S95 S96
S21 S22 S35 S36 S37 S38 S5 S51 S52 S6 S65 S66 S79 S80 S85 S86
S1 S15 S16 S2 S29 S30 S43 S44 S57 S58 S71 S72 S75 S76 S91 S92
S19 S20 S3 S33 S34 S4 S47 S48 S49 S50 S63 S64 S77 S78 S89 S90
S10 S17 S18 S31 S32 S45 S46 S59 S60 S61 S62 S73 S74 S87 S88 S9
sample

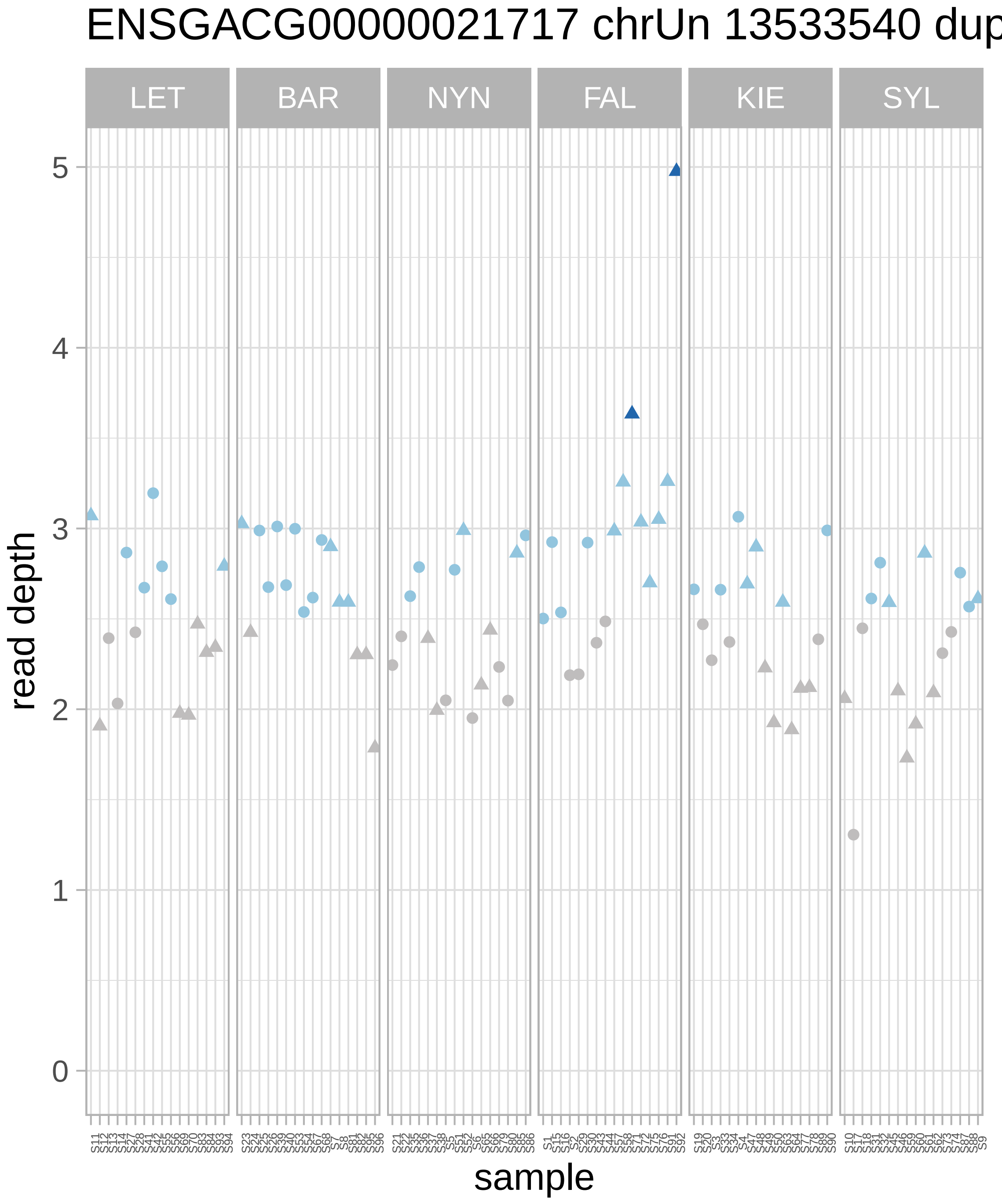
ENSGACG00000021717 chrUn 13533540 dup
LET
BAR
NYN
FAL
KIE
SYL
read depth
0
1
2
3
4
5
S11 S12 S13 S14 S27 S28 S41 S42 S55 S56 S69 S70 S83 S84 S93 S94
S23 S24 S25 S26 S39 S40 S53 S54 S67 S68 S7 S8 S81 S82 S95 S96
S21 S22 S35 S36 S37 S38 S5 S51 S52 S6 S65 S66 S79 S80 S85 S86
S1 S15 S16 S2 S29 S30 S43 S44 S57 S58 S71 S72 S75 S76 S91 S92
S19 S20 S3 S33 S34 S4 S47 S48 S49 S50 S63 S64 S77 S78 S89 S90
S10 S17 S18 S31 S32 S45 S46 S59 S60 S61 S62 S73 S74 S87 S88 S9
sample

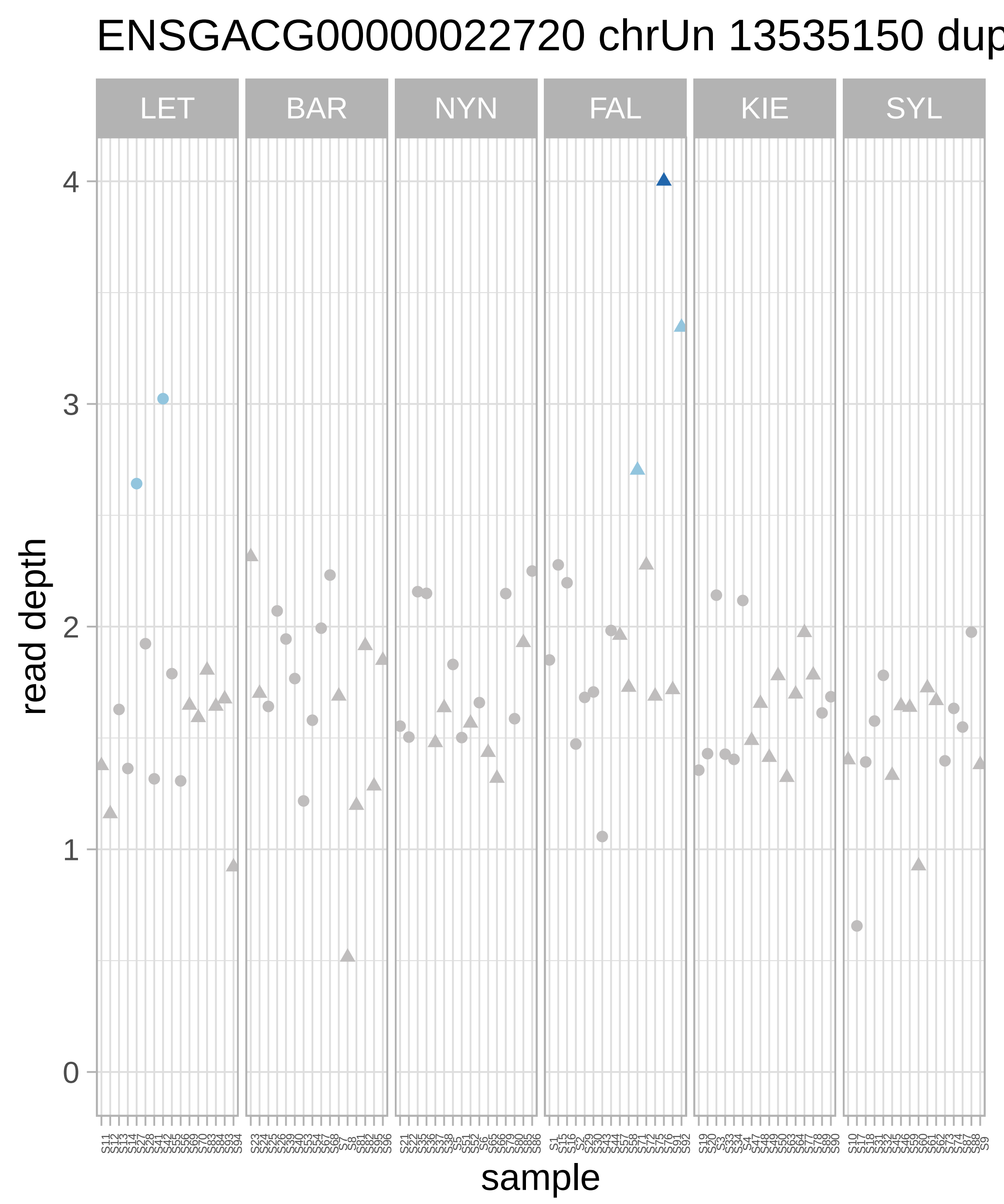

ENSGACG00000022720 chrUn 13535150 dup
LET
BAR
NYN
FAL
KIE
SYL
read depth
sample
0
1
2
3
4

ENSGACG00000020968 chrUn 13533305 dup

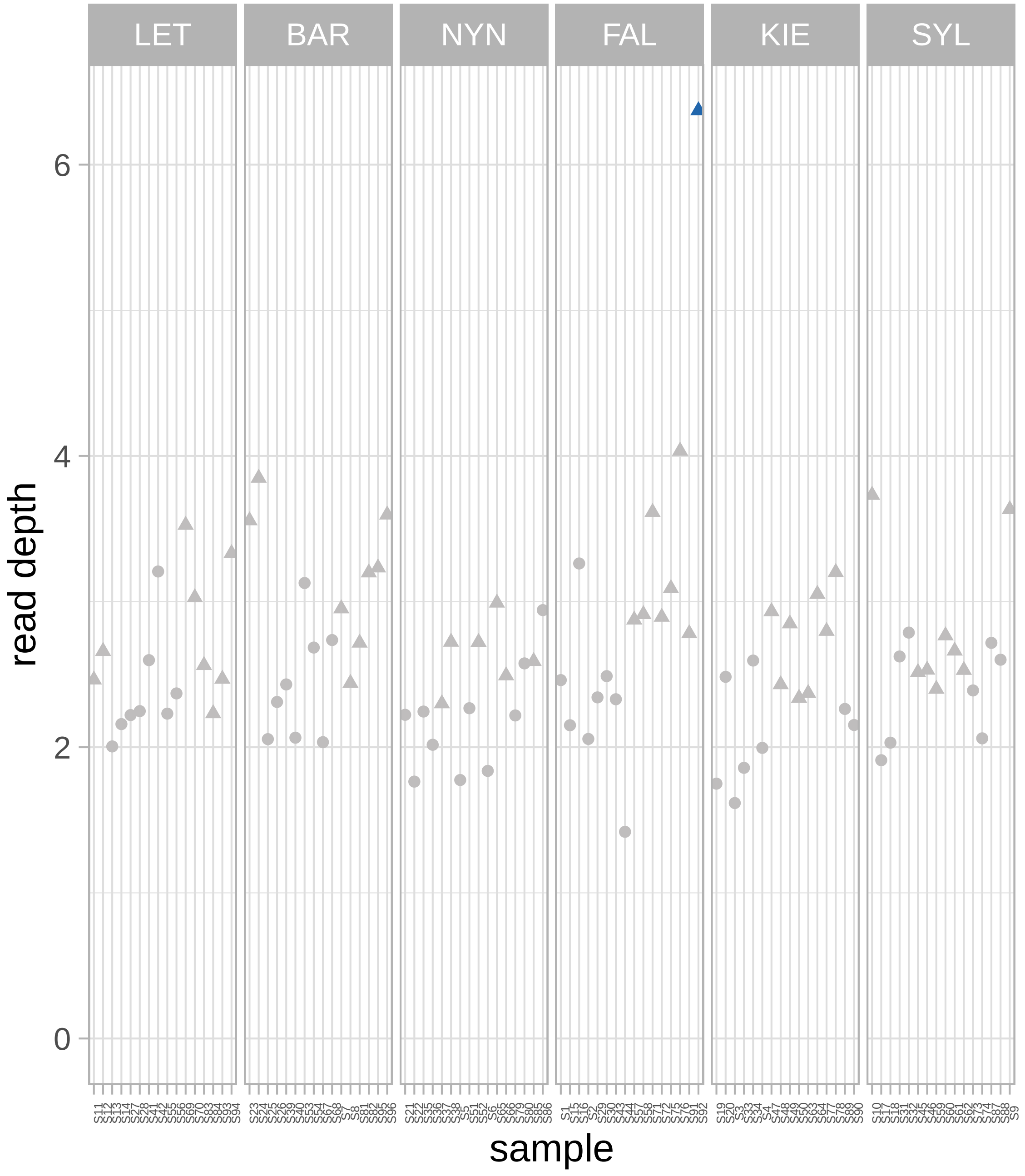

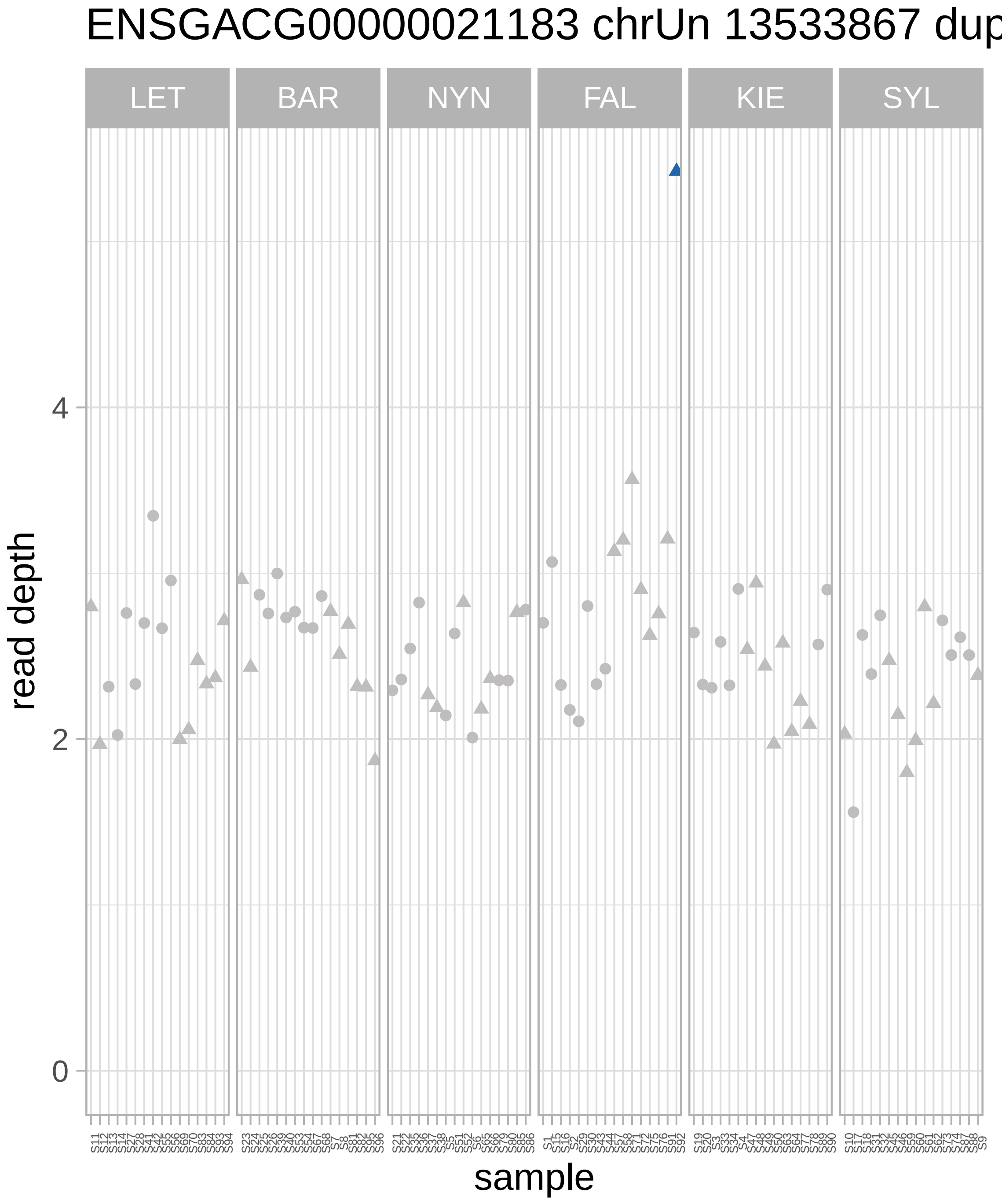
ENSGACG00000021183 chrUn 13533867 dup
LET
BAR
NYN
FAL
KIE
SYL
read depth
0
2
4
S11 S12 S13 S14 S27 S28 S41 S42 S55 S56 S69 S70 S83 S84 S93 S94
S23 S24 S25 S26 S39 S40 S53 S54 S67 S68 S7 S8 S81 S82 S95 S96
S21 S22 S35 S36 S37 S38 S5 S51 S52 S6 S65 S66 S79 S80 S85 S86
S1 S15 S16 S2 S29 S30 S43 S44 S57 S58 S71 S72 S75 S76 S91 S92
S19 S20 S3 S33 S34 S4 S47 S48 S49 S50 S63 S64 S77 S78 S89 S90
S10 S17 S18 S31 S32 S45 S46 S59 S60 S61 S62 S73 S74 S87 S88 S9
sample

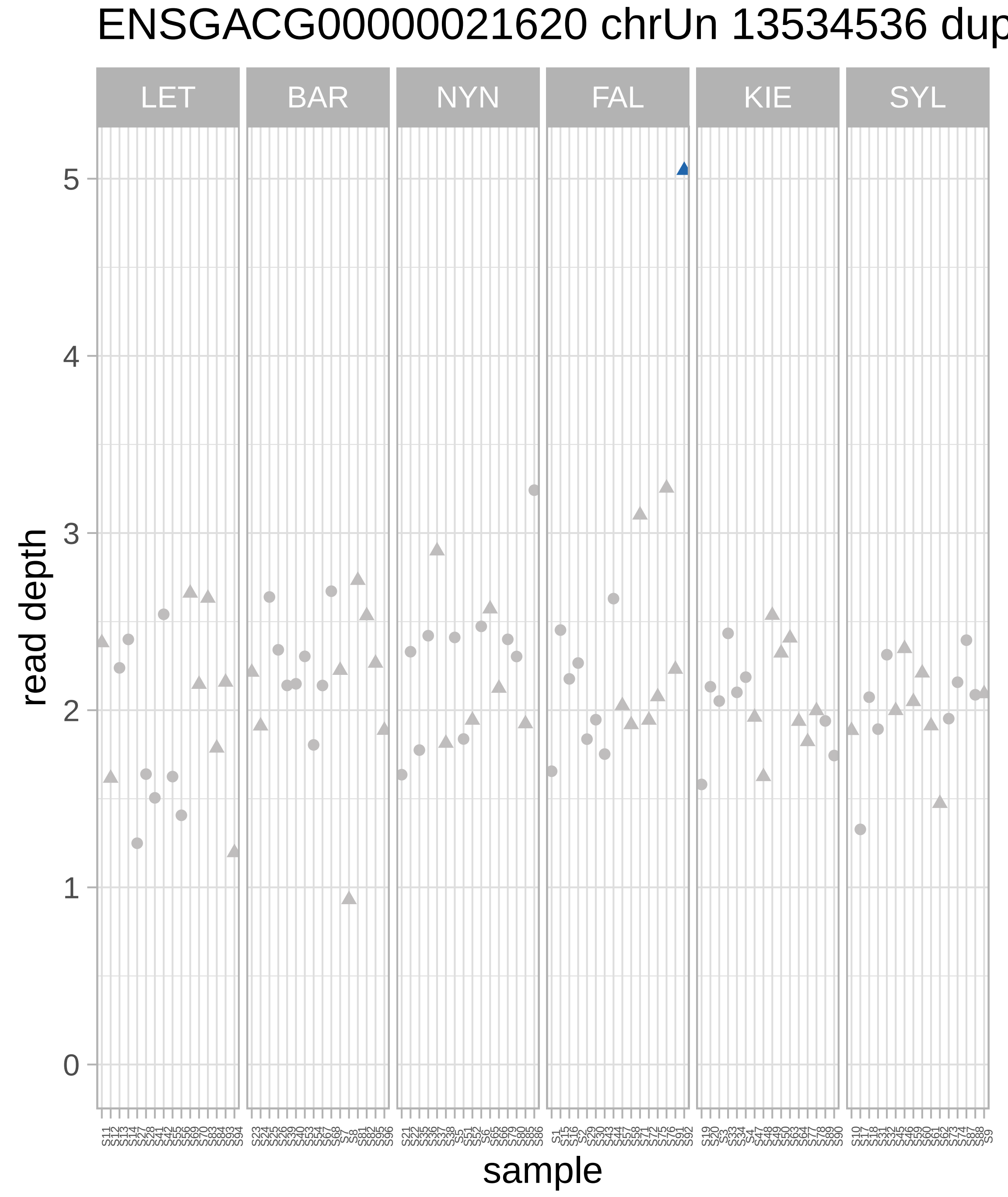
ENSGACG00000021620 chrUn 13534536 dup
LET
BAR
NYN
FAL
KIE
SYL
read depth
sample

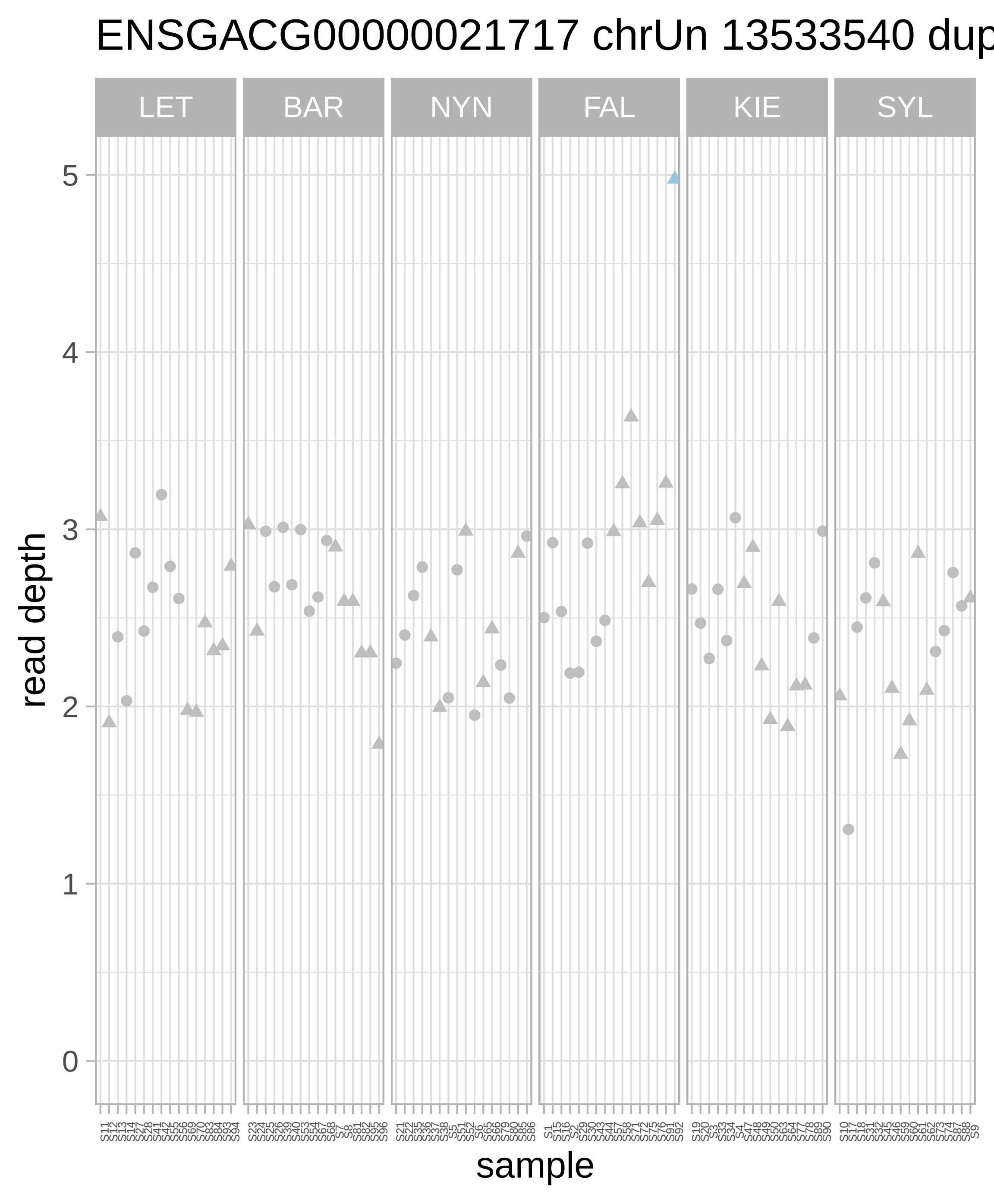
ENSGACG00000021717 chrUn 13533540 dup
LET
BAR
NYN
FAL
KIE
SYL
read depth
0
1
2
3
4
5
sample

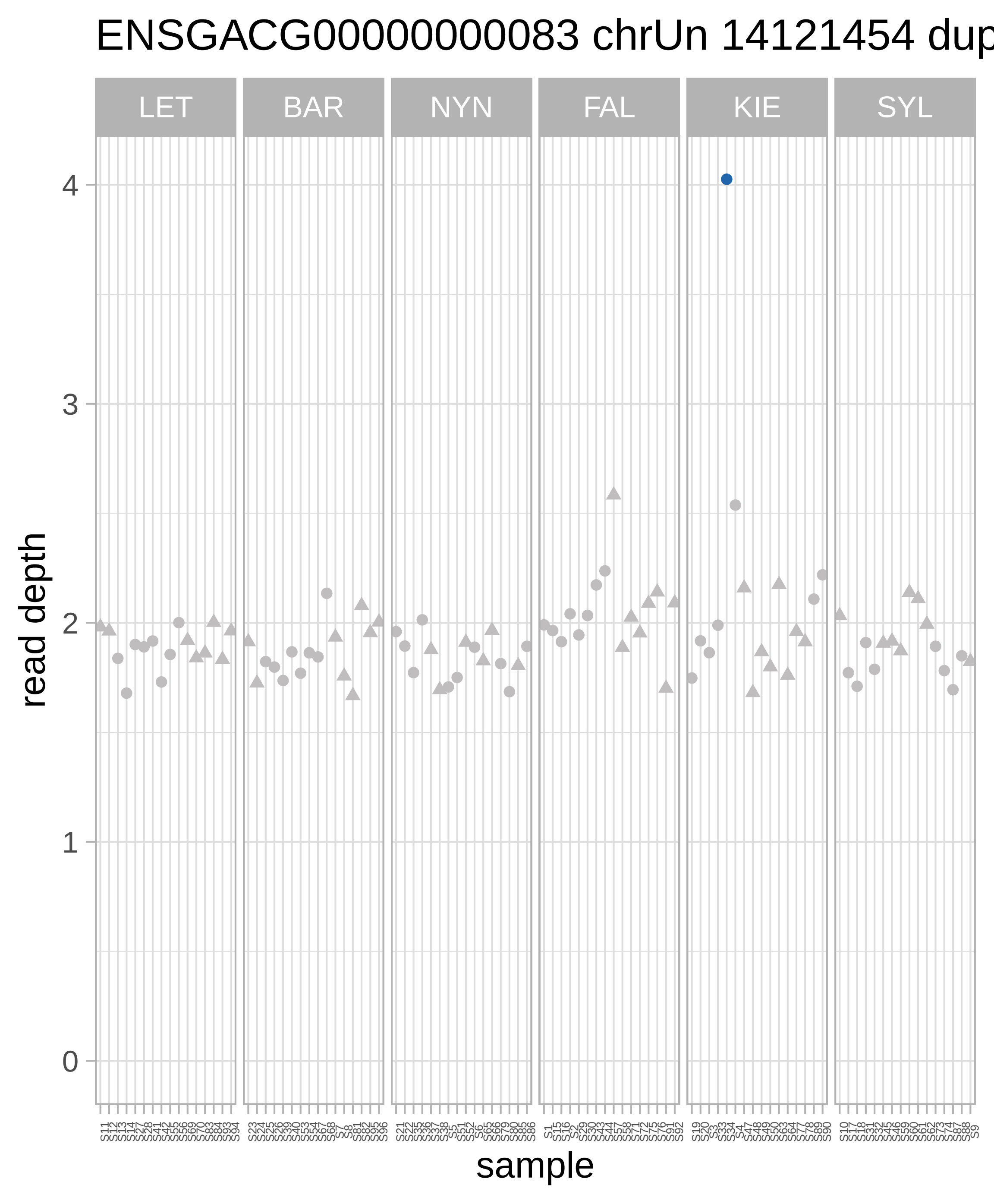
ENSGACG00000000083 chrUn 14121454 dup
LET
BAR
NYN
FAL
KIE
SYL
read depth
0
1
2
3
4
sample

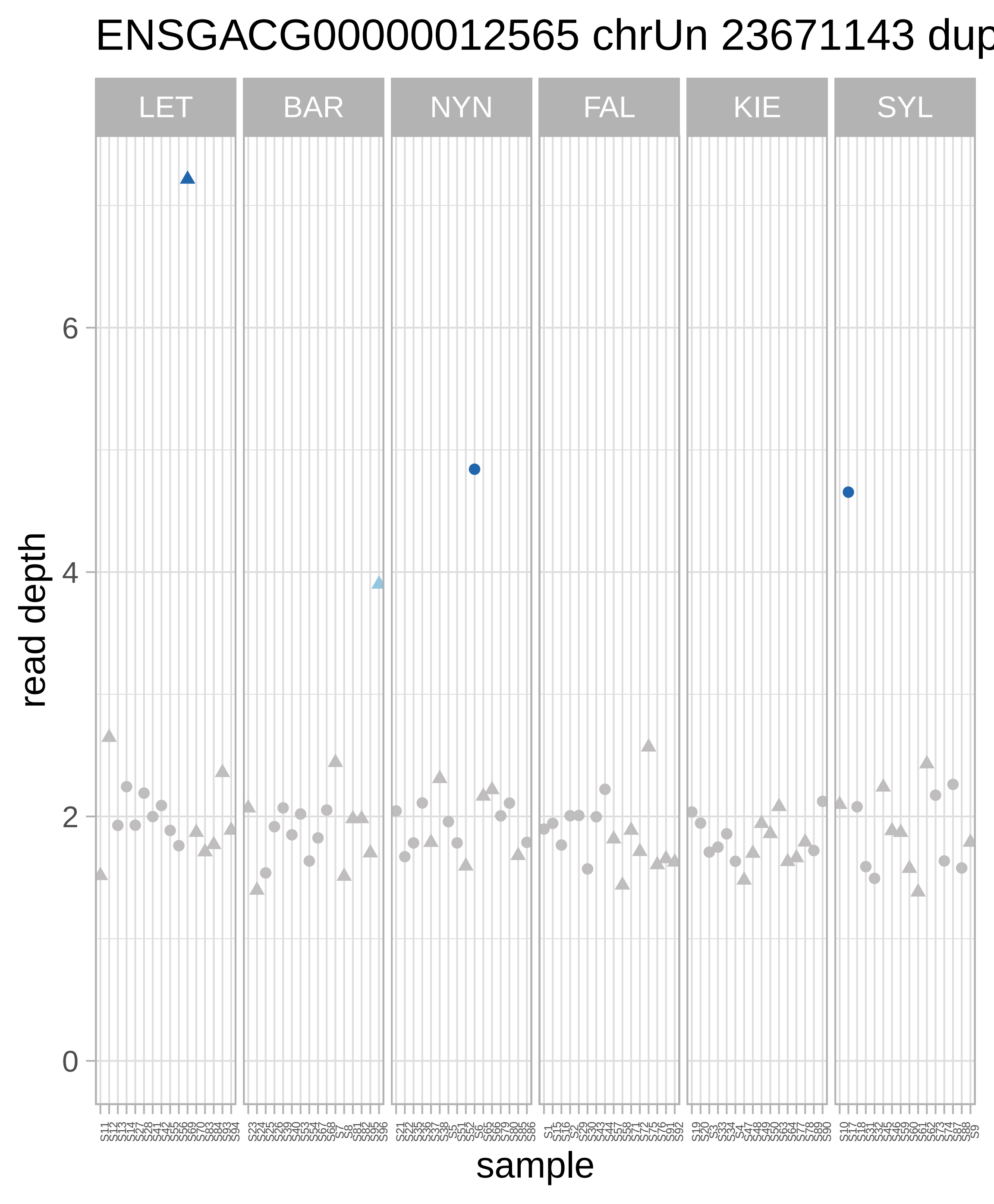
ENSGACG00000012565 chrUn 23671143 dup
LET
BAR
NYN
FAL
KIE
SYL
read depth
0
2
4
6
sample

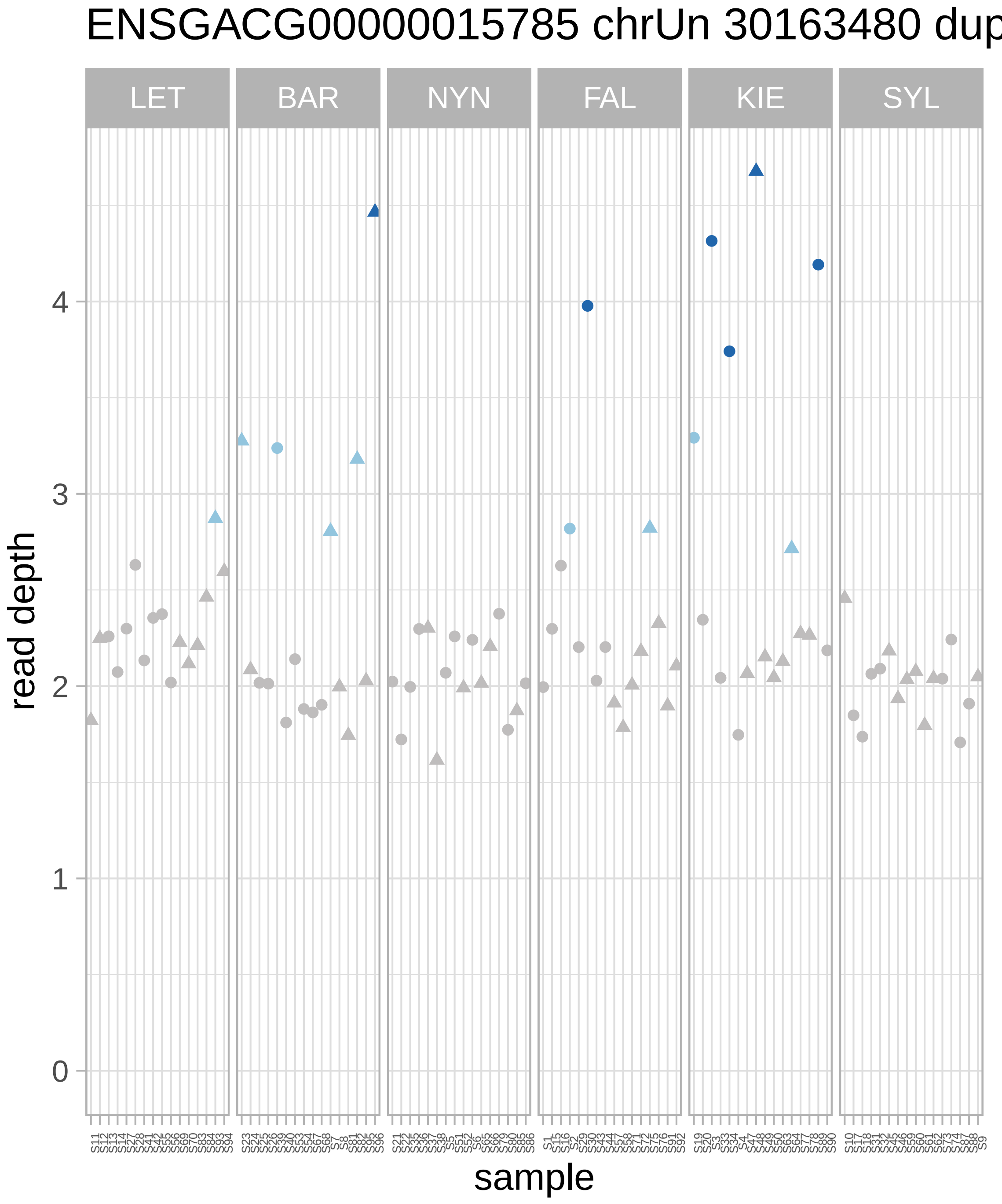

ENSGACG00000015785 chrUn 30163480 dup
LET
BAR
NYN
FAL
KIE
SYL
read depth
0
1
2
3
4
sample

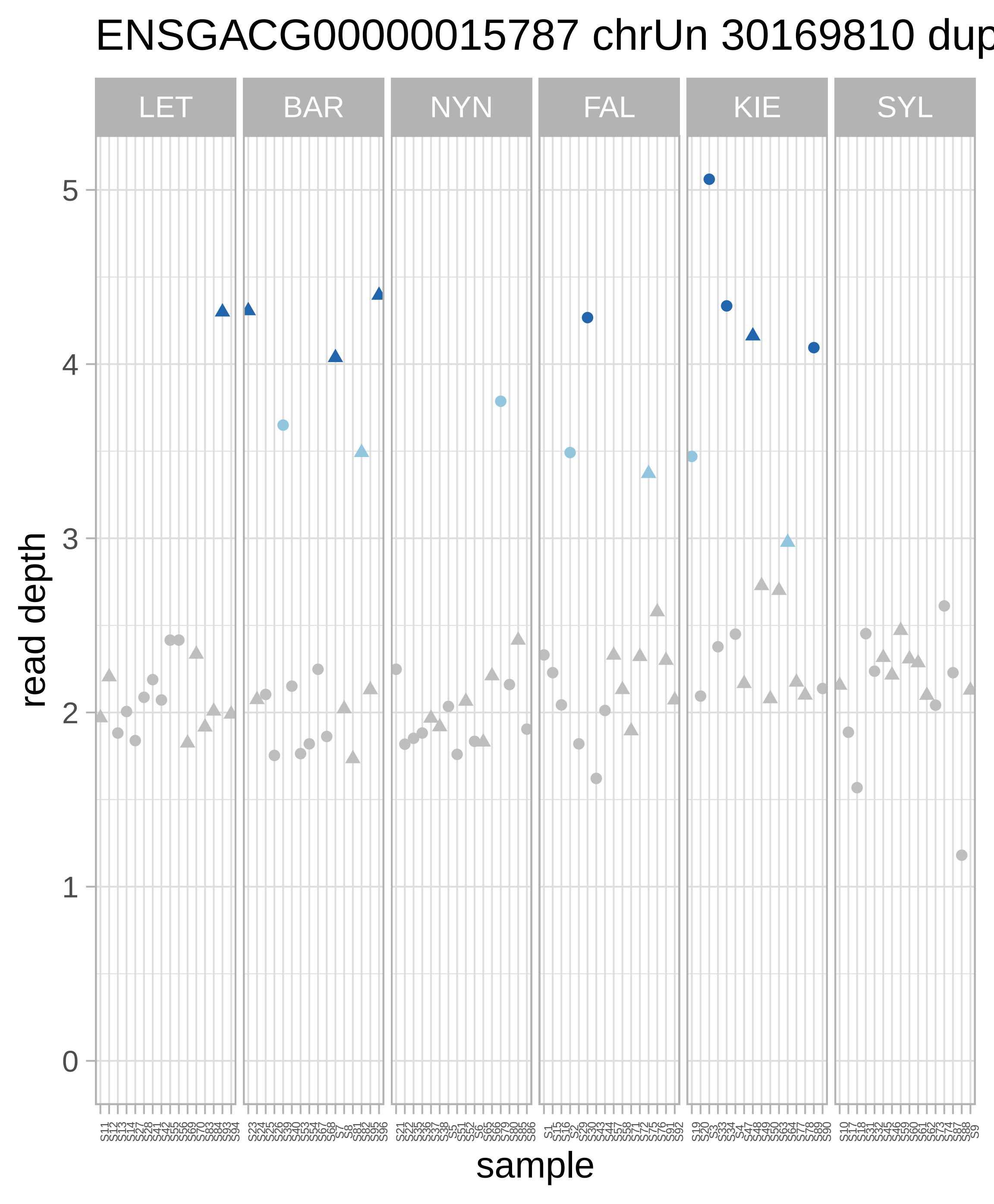
ENSGACG00000015787 chrUn 30169810 dup
LET
BAR
NYN
FAL
KIE
SYL
read depth
0
1
2
3
4
5
sample
S11 S12 S13 S14 S27 S28 S41 S42 S55 S56 S69 S70 S83 S84 S93 S94
S23 S24 S25 S26 S39 S40 S53 S54 S67 S68 S7 S8 S81 S82 S95 S96
S21 S22 S35 S36 S37 S38 S5 S51 S52 S6 S65 S66 S79 S80 S85 S86
S1 S15 S16 S2 S29 S30 S43 S44 S57 S58 S71 S72 S75 S76 S91 S92
S19 S20 S3 S33 S34 S4 S47 S48 S49 S50 S63 S64 S77 S78 S89 S90
S10 S17 S18 S31 S32 S45 S46 S59 S60 S61 S62 S73 S74 S87 S88 S9

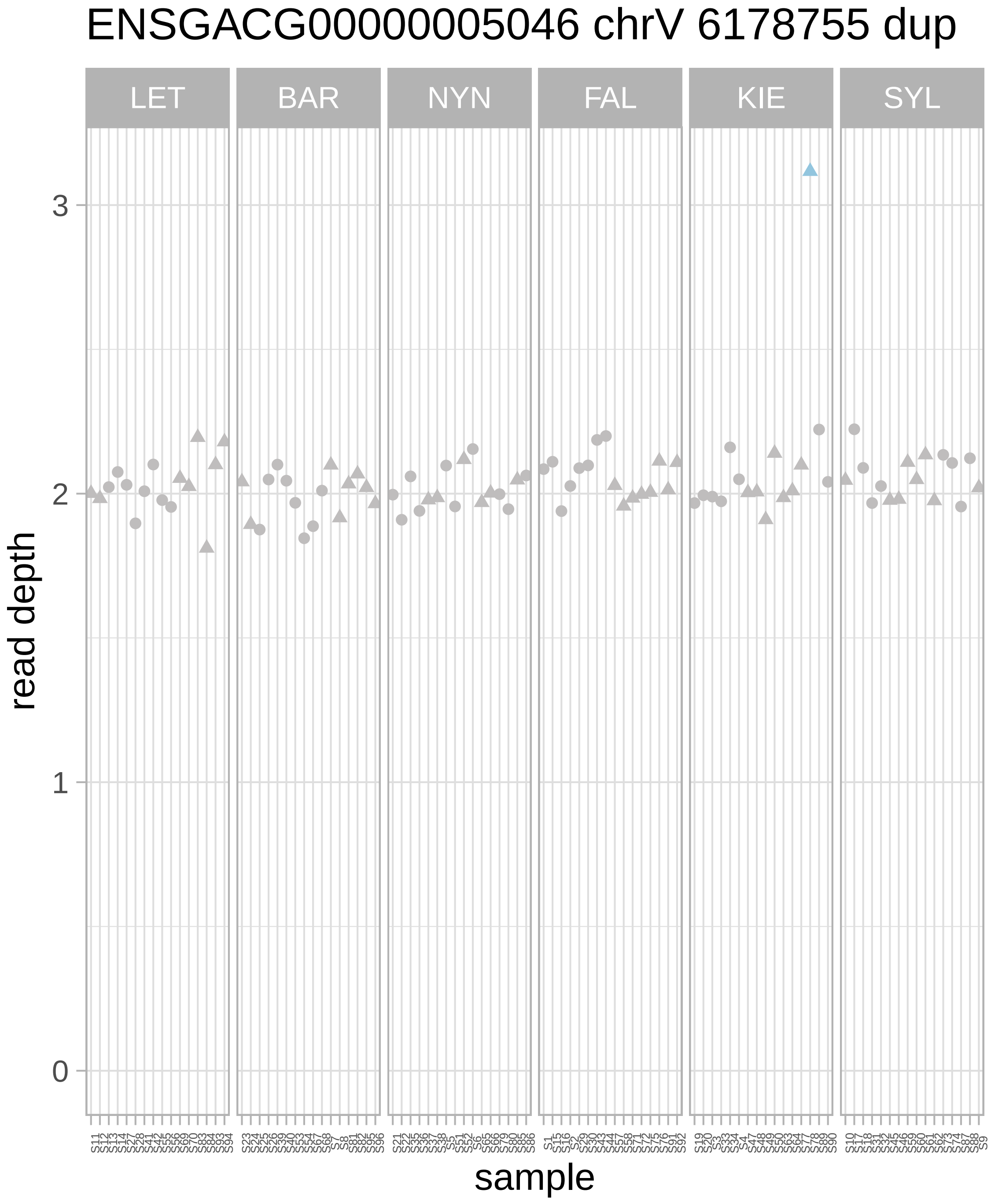
ENSGACG00000005046 chrV 6178755 dup
LET
BAR
NYN
FAL
KIE
SYL
read depth
3
2
1
0
S11 S12 S13 S14 S27 S28 S41 S42 S55 S56 S69 S70 S83 S84 S93 S94
S23 S24 S25 S26 S39 S40 S53 S54 S67 S68 S7 S8 S81 S82 S95 S96
S21 S22 S35 S36 S37 S38 S5 S51 S52 S6 S65 S66 S79 S80 S85 S86
S1 S15 S16 S2 S29 S30 S43 S44 S57 S58 S71 S72 S75 S76 S91 S92
S19 S20 S3 S33 S34 S4 S47 S48 S49 S50 S63 S64 S77 S78 S89 S90
S10 S17 S18 S31 S32 S45 S46 S59 S60 S61 S62 S73 S74 S87 S88 S9
sample

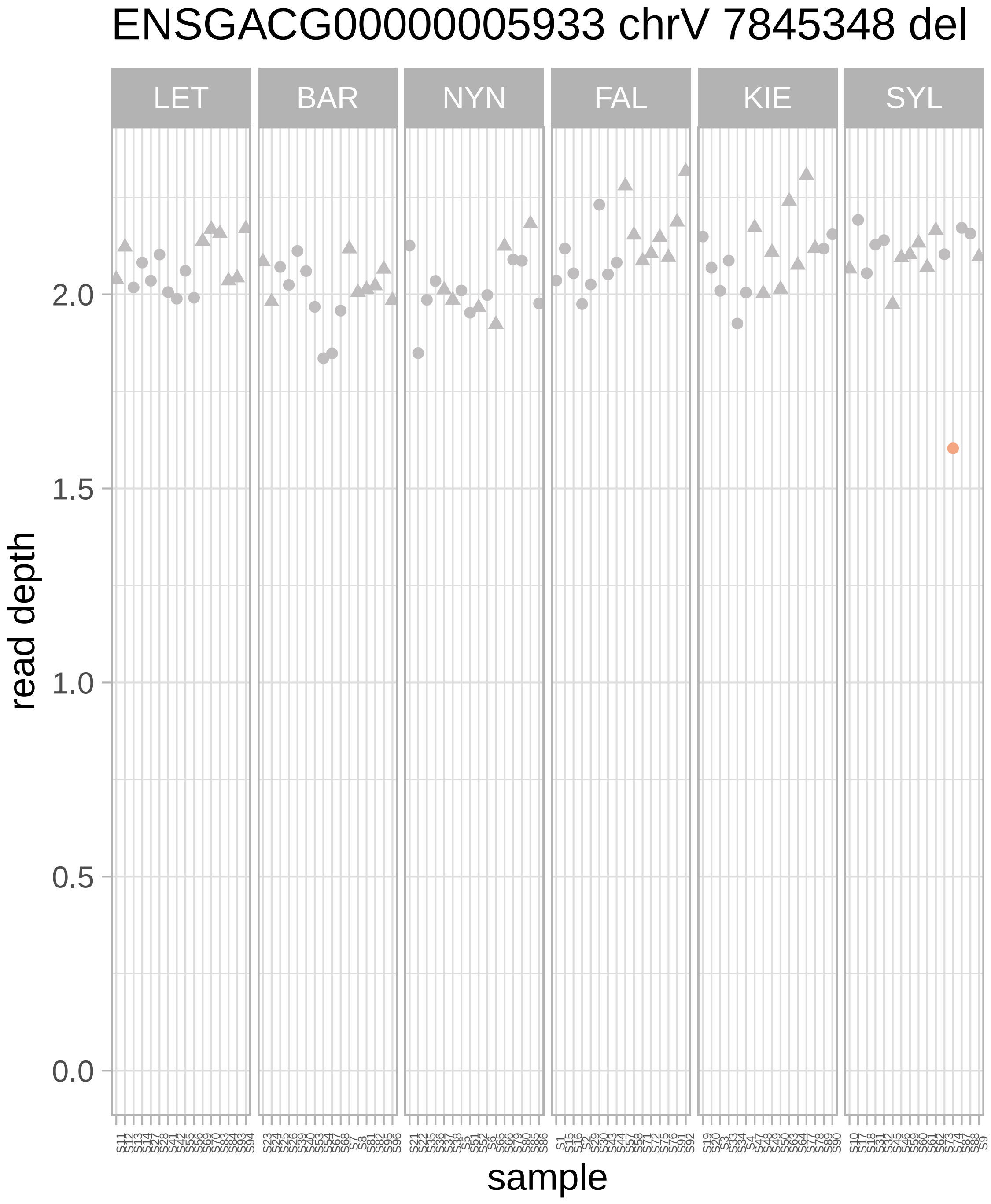
ENSGACG00000005933 chrV 7845348 del
LET
BAR
NYN
FAL
KIE
SYL
read depth
2.0
1.5
1.0
0.5
0.0
sample

ENSGACG00000006590 chrV 8464438 dup

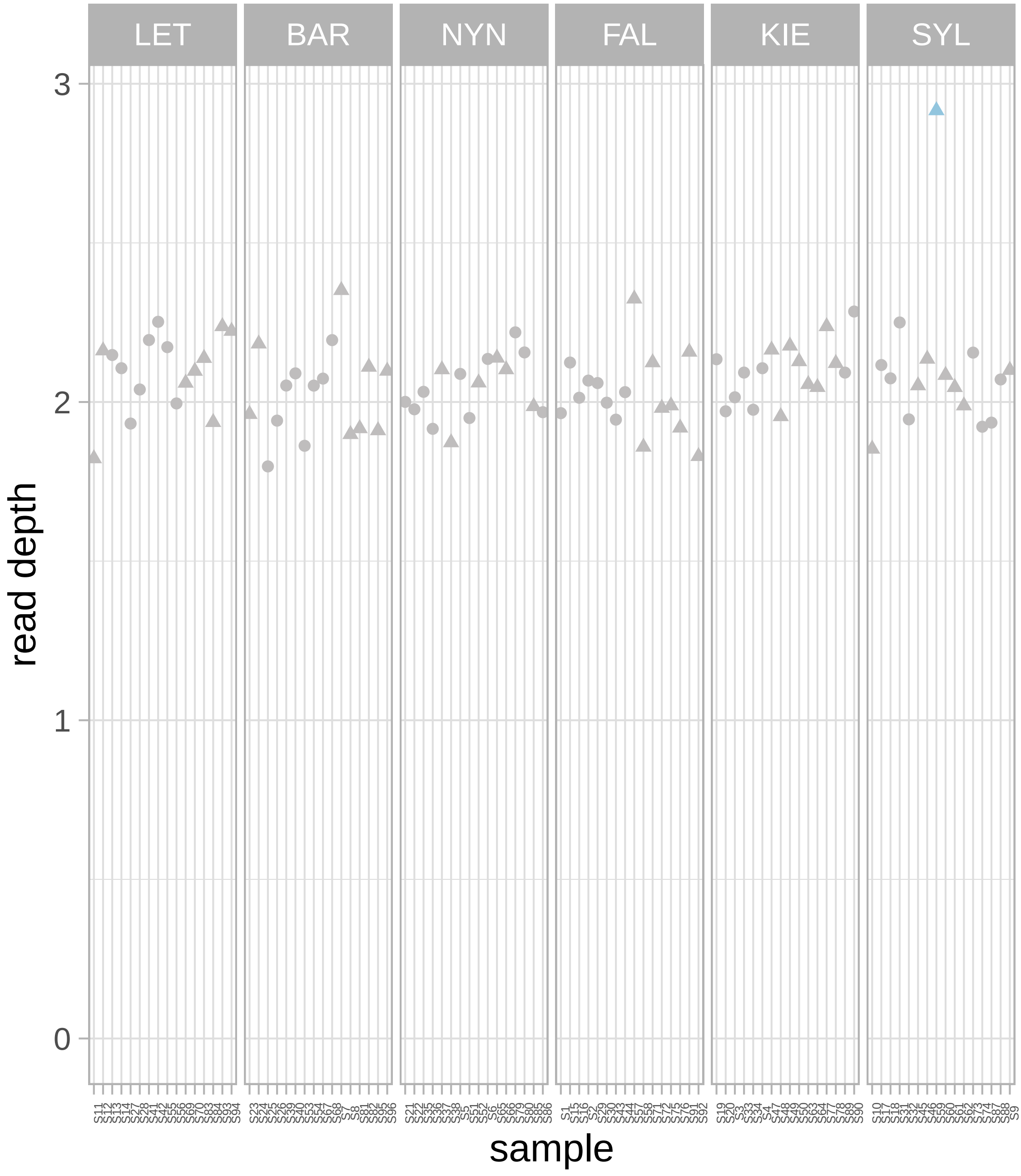

ENSGACG00000007901 chrV 9813450 dup

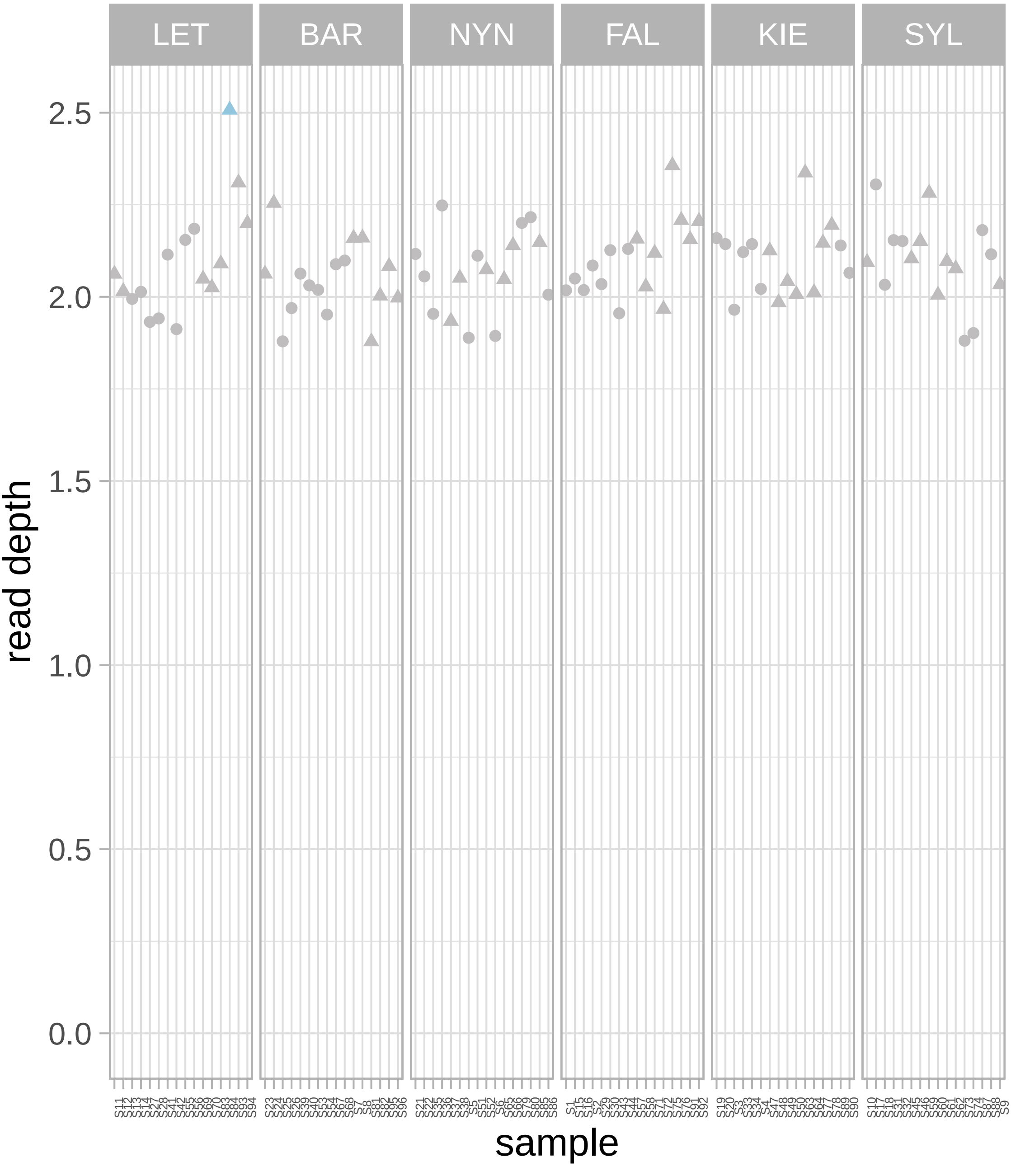

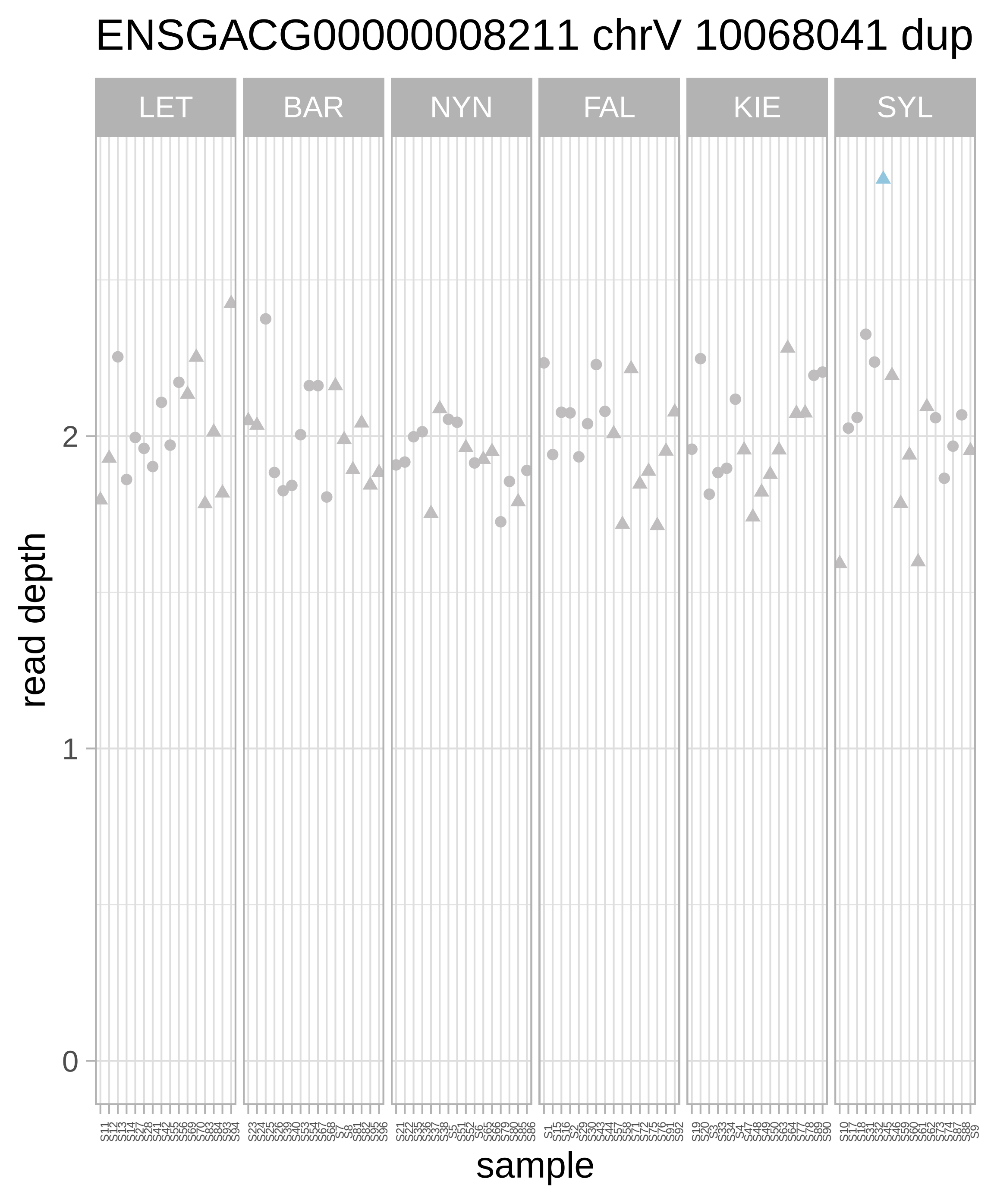
ENSGACG00000008211 chrV 10068041 dup
LET
BAR
NYN
FAL
KIE
SYL
read depth
0
1
2
sample

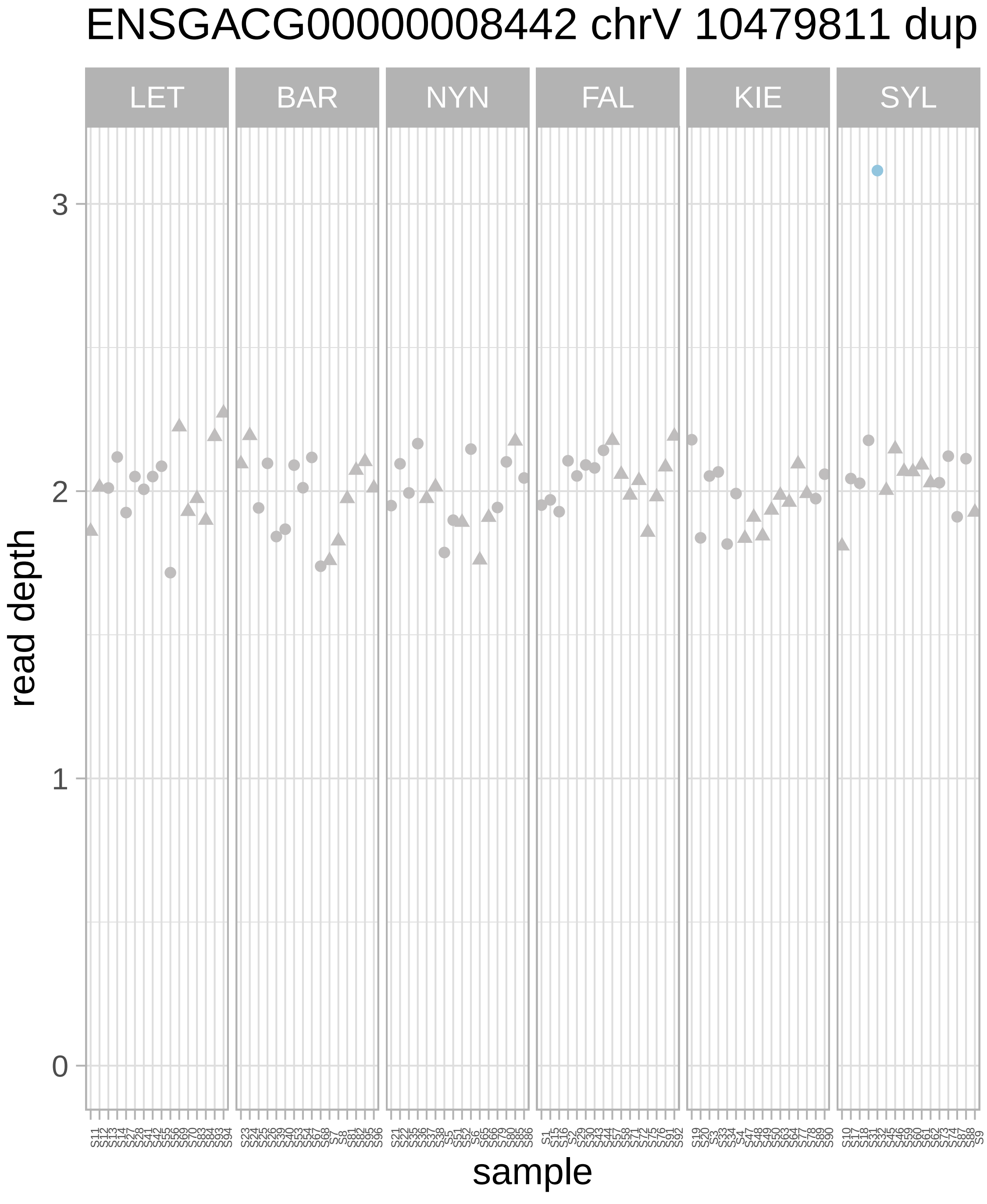

ENSGACG00000008442 chrV 10479811 dup
LET
BAR
NYN
FAL
KIE
SYL
read depth
0
1
2
3
S11 S12 S13 S14 S27 S28 S41 S42 S55 S56 S69 S70 S83 S84 S93 S94
S23 S24 S25 S26 S39 S40 S53 S54 S67 S68 S7 S8 S81 S82 S95 S96
S21 S22 S35 S36 S37 S38 S5 S51 S52 S6 S65 S66 S79 S80 S85 S86
S1 S15 S16 S2 S29 S30 S43 S44 S57 S58 S71 S72 S75 S76 S91 S92
S19 S20 S3 S33 S34 S4 S47 S48 S49 S50 S63 S64 S77 S78 S89 S90
S10 S17 S18 S31 S32 S45 S46 S59 S60 S61 S62 S73 S74 S87 S88 S9
sample

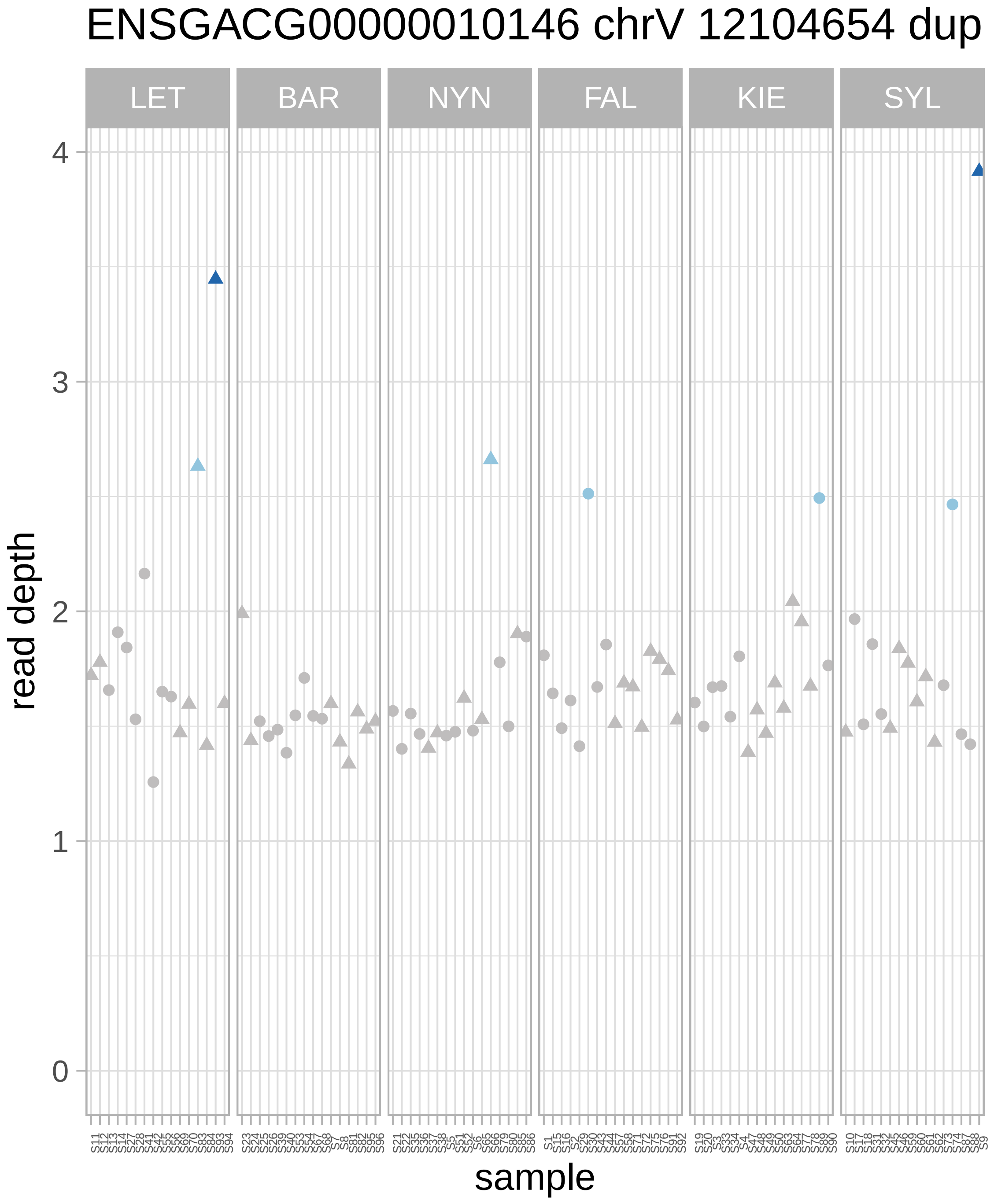

ENSGACG00000010146 chrV 12104654 dup
LET
BAR
NYN
FAL
KIE
SYL
read depth
0
1
2
3
4
S11 S12 S13 S14 S27 S28 S41 S42 S55 S56 S69 S70 S83 S84 S93 S94
S23 S24 S25 S26 S39 S40 S53 S54 S67 S68 S7 S8 S81 S82 S95 S96
S21 S22 S35 S36 S37 S38 S5 S51 S52 S6 S65 S66 S79 S80 S85 S86
S1 S15 S16 S2 S29 S30 S43 S44 S57 S58 S71 S72 S75 S76 S91 S92
S19 S20 S3 S33 S34 S4 S47 S48 S49 S50 S63 S64 S77 S78 S89 S90
S10 S17 S18 S31 S32 S45 S46 S59 S60 S61 S62 S73 S74 S87 S88 S9
sample

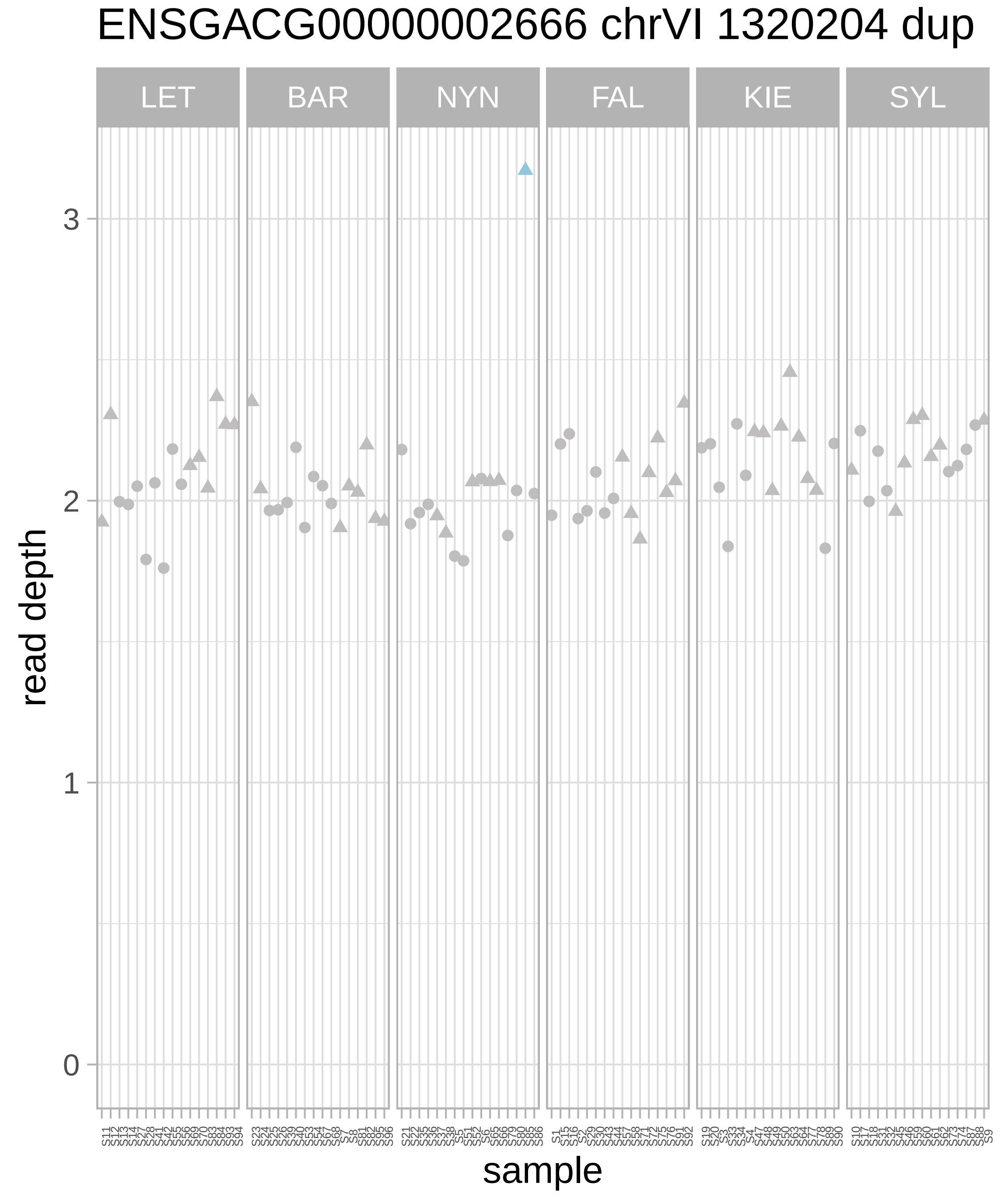

ENSGACG00000002666 chrVI 1320204 dup
LET
BAR
NYN
FAL
KIE
SYL
read depth
0
1
2
3
sample

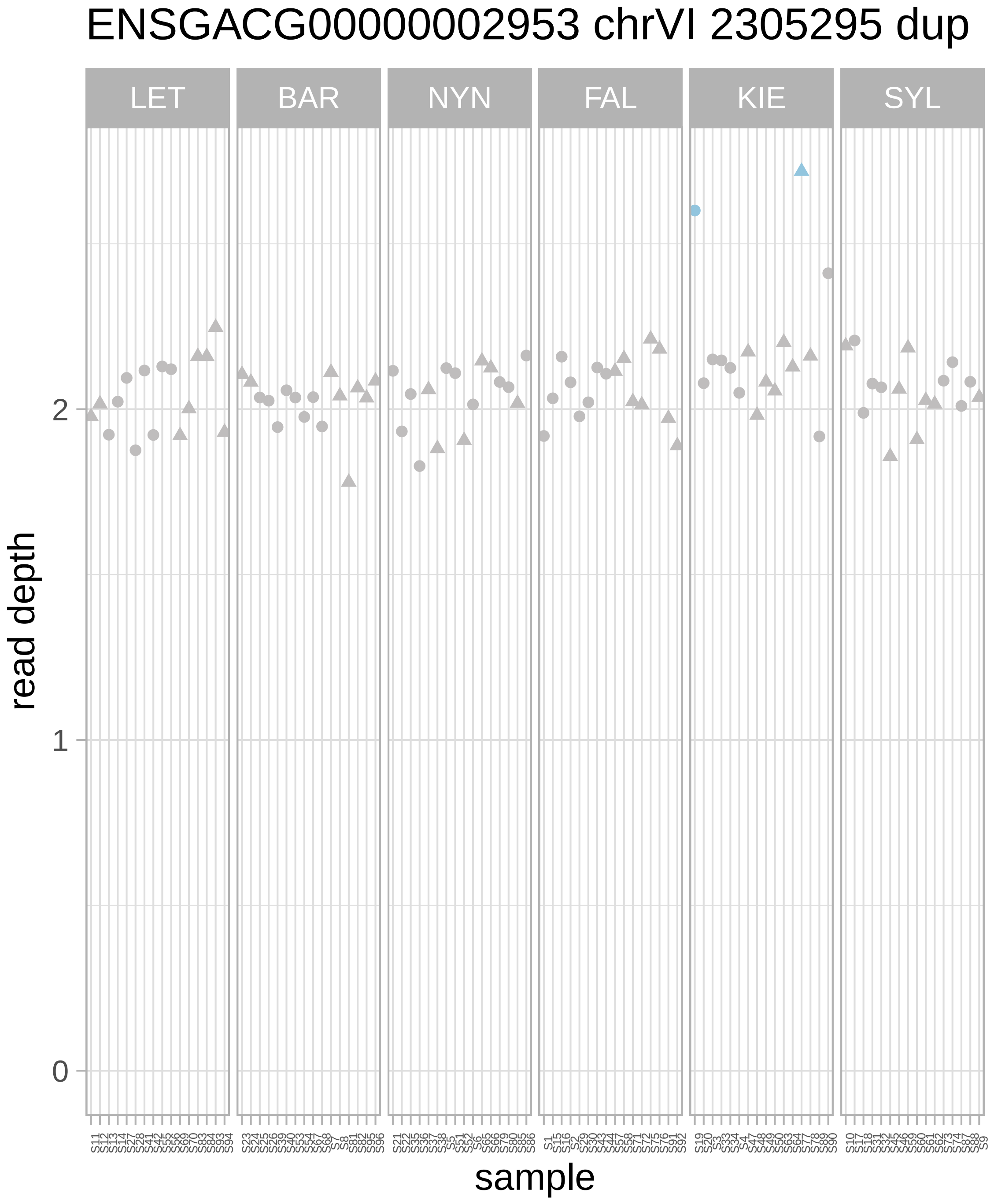
ENSGACG00000002953 chrVI 2305295 dup
LET
BAR
NYN
FAL
KIE
SYL
read depth
0
1
2
sample

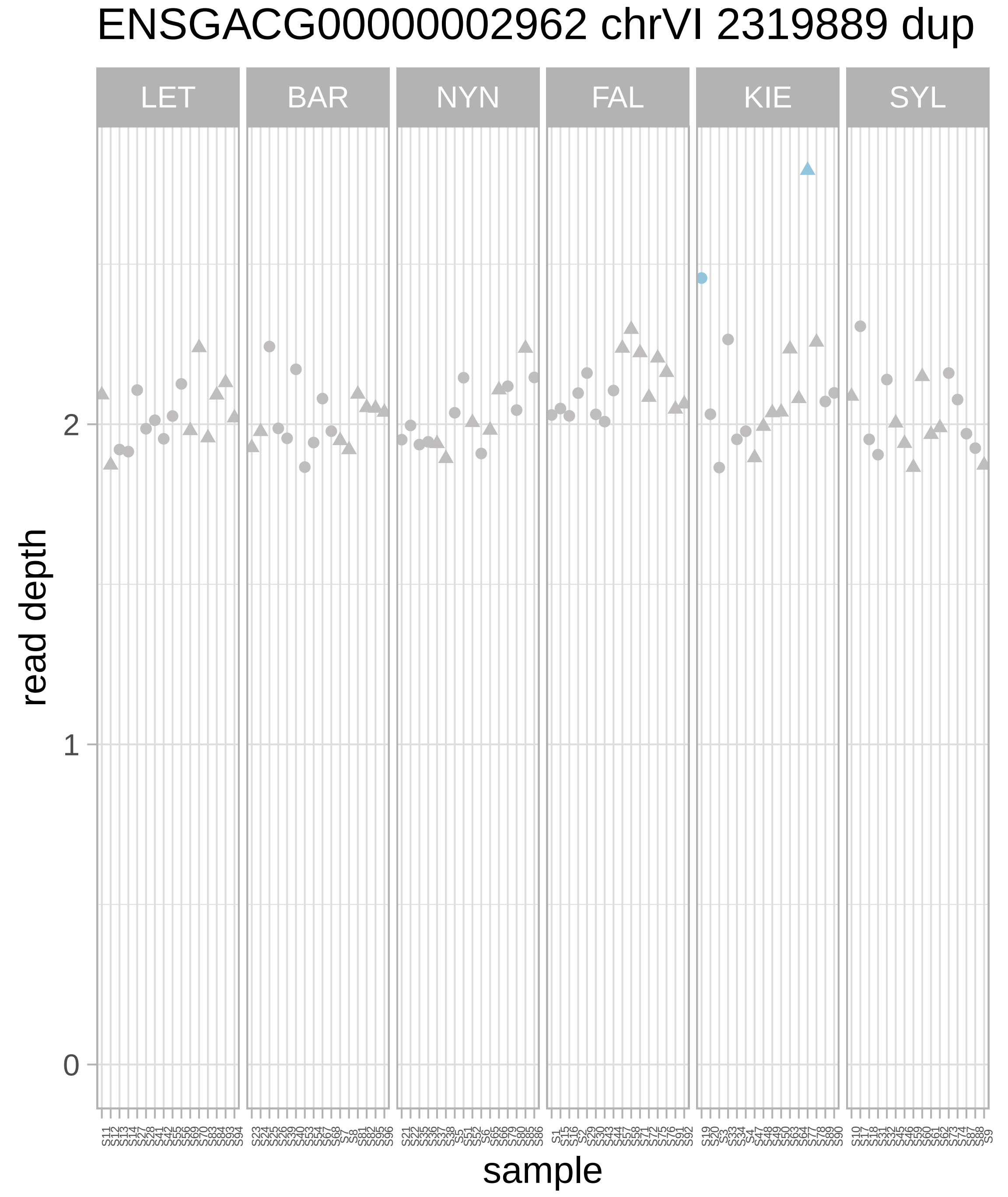

ENSGACG00000002962 chrVI 2319889 dup
LET
BAR
NYN
FAL
KIE
SYL
read depth
0
1
2
sample

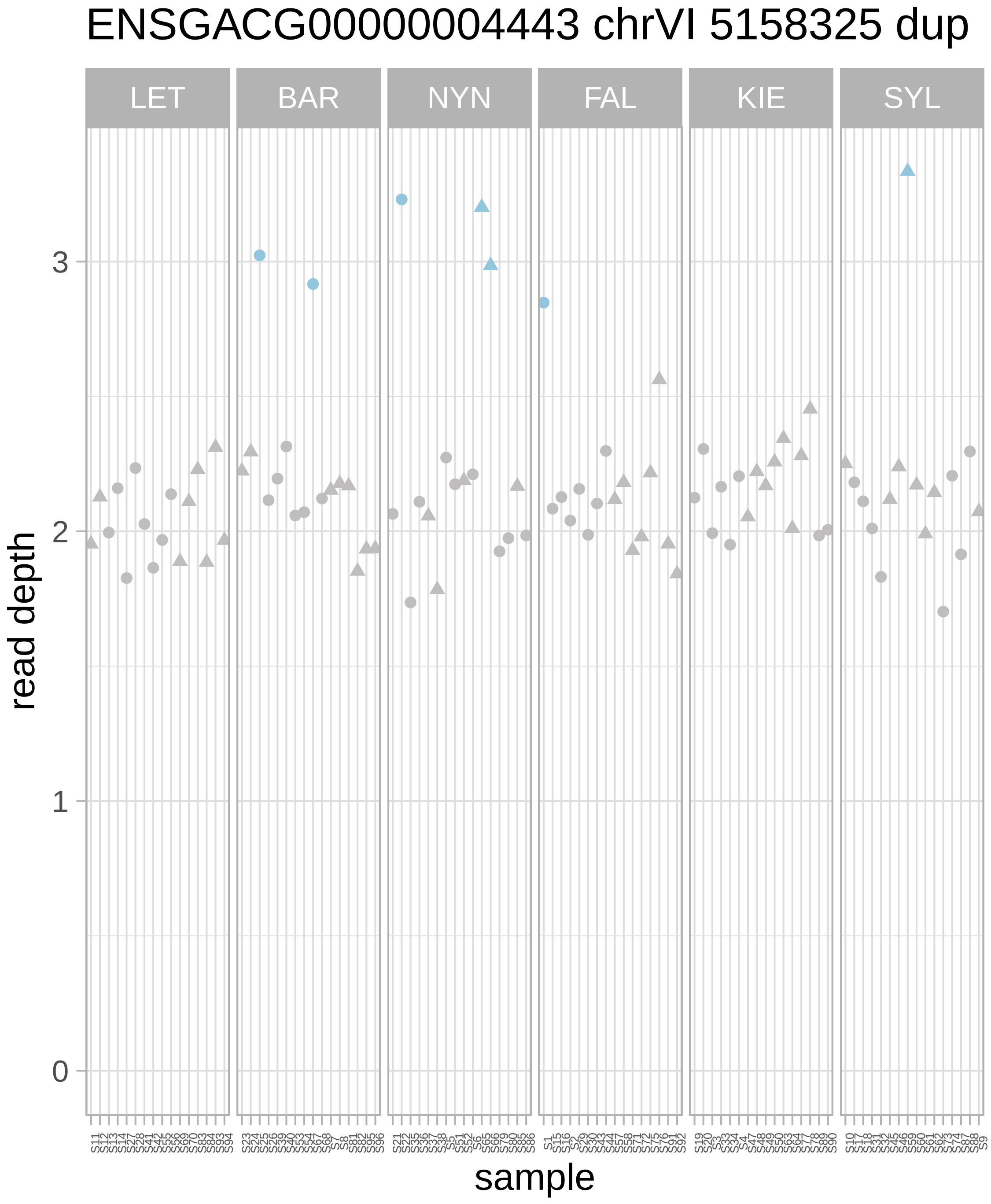
ENSGACG00000004443 chrVI 5158325 dup
LET
BAR
NYN
FAL
KIE
SYL
read depth
0
1
2
3
sample
S11 S12 S13 S14 S27 S28 S41 S42 S55 S56 S69 S70 S83 S84 S93 S94
S23 S24 S25 S26 S39 S40 S53 S54 S67 S68 S7 S8 S81 S82 S95 S96
S21 S22 S35 S36 S37 S38 S5 S51 S52 S6 S65 S66 S79 S80 S85 S86
S1 S15 S16 S2 S29 S30 S43 S44 S57 S58 S71 S72 S75 S76 S91 S92
S19 S20 S3 S33 S34 S4 S47 S48 S49 S50 S63 S64 S77 S78 S89 S90
S10 S17 S18 S31 S32 S45 S46 S59 S60 S61 S62 S73 S74 S87 S88 S9

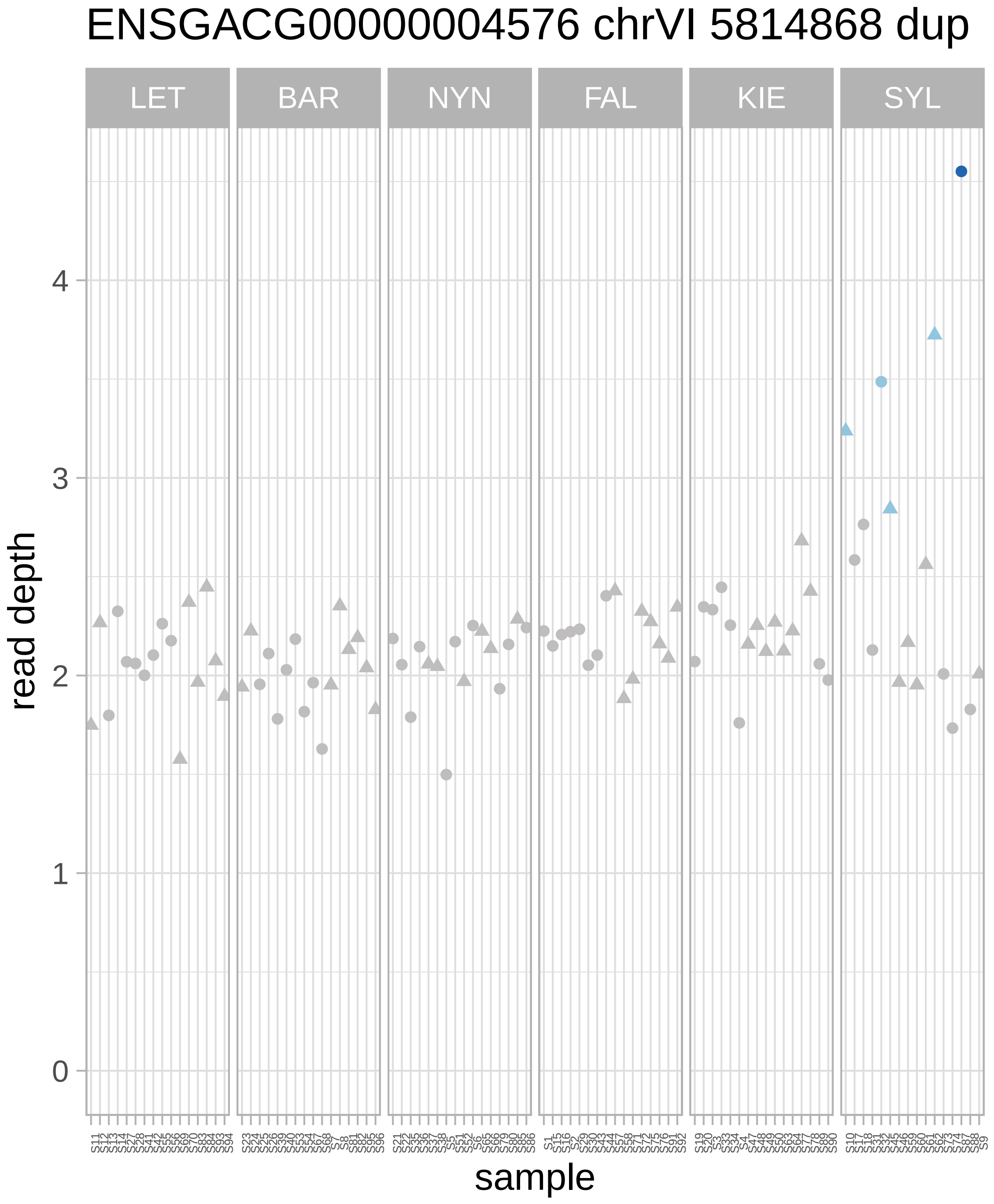
ENSGACG00000004576 chrVI 5814868 dup
LET
BAR
NYN
FAL
KIE
SYL
read depth
0
1
2
3
4
S11 S12 S13 S14 S27 S28 S41 S42 S55 S56 S69 S70 S83 S84 S93 S94
S23 S24 S25 S26 S39 S40 S53 S54 S67 S68 S7 S8 S81 S82 S95 S96
S21 S22 S35 S36 S37 S38 S5 S51 S52 S6 S65 S66 S79 S80 S85 S86
S1 S15 S16 S2 S29 S30 S43 S44 S57 S58 S71 S72 S75 S76 S91 S92
S19 S20 S3 S33 S34 S4 S47 S48 S49 S50 S63 S64 S77 S78 S89 S90
S10 S17 S18 S31 S32 S45 S46 S59 S60 S61 S62 S73 S74 S87 S88 S9
sample

ENSGACG00000004582 chrVI 5817186 dup

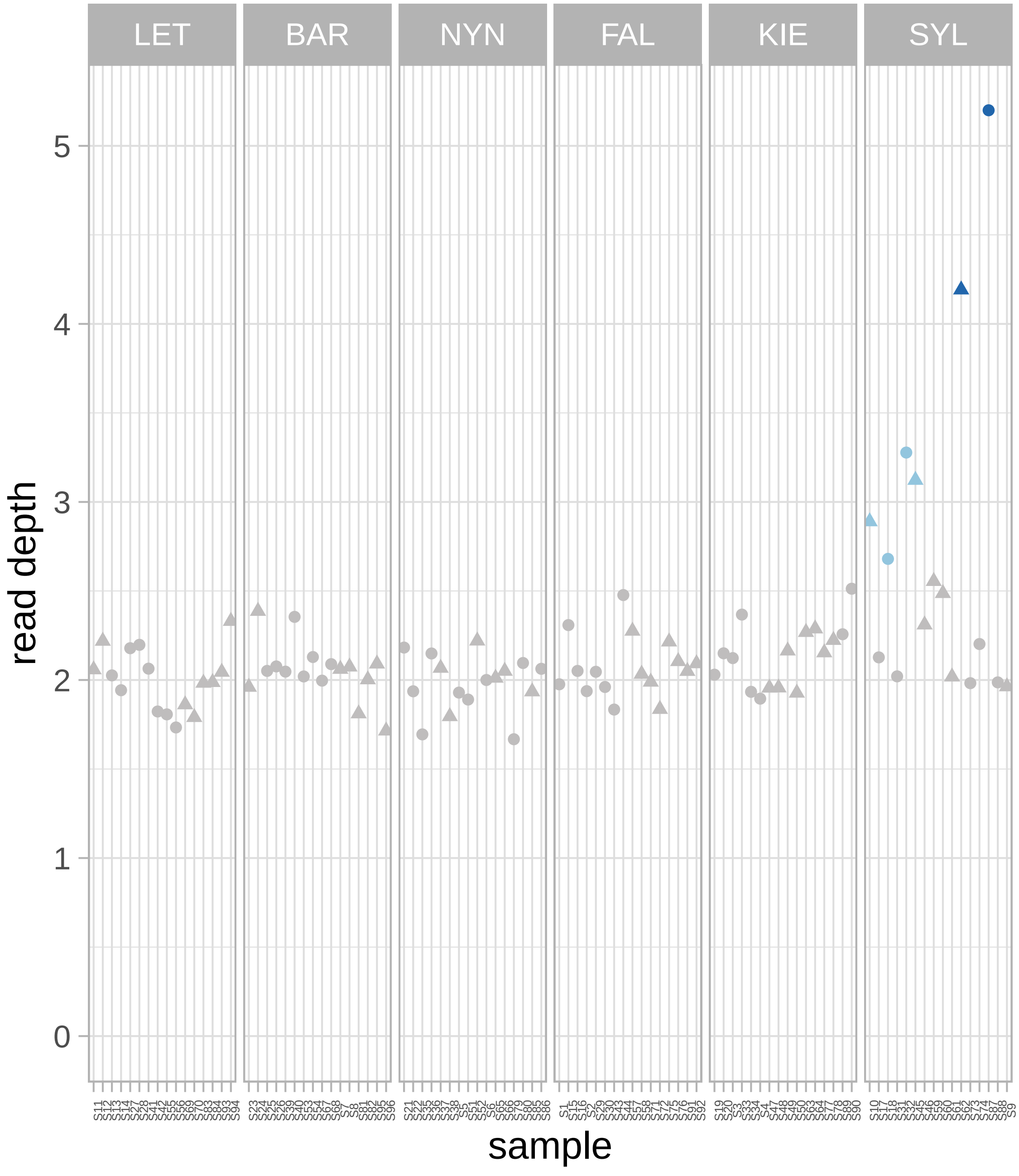

ENSGACG00000006746 chrVI 8006823 dup

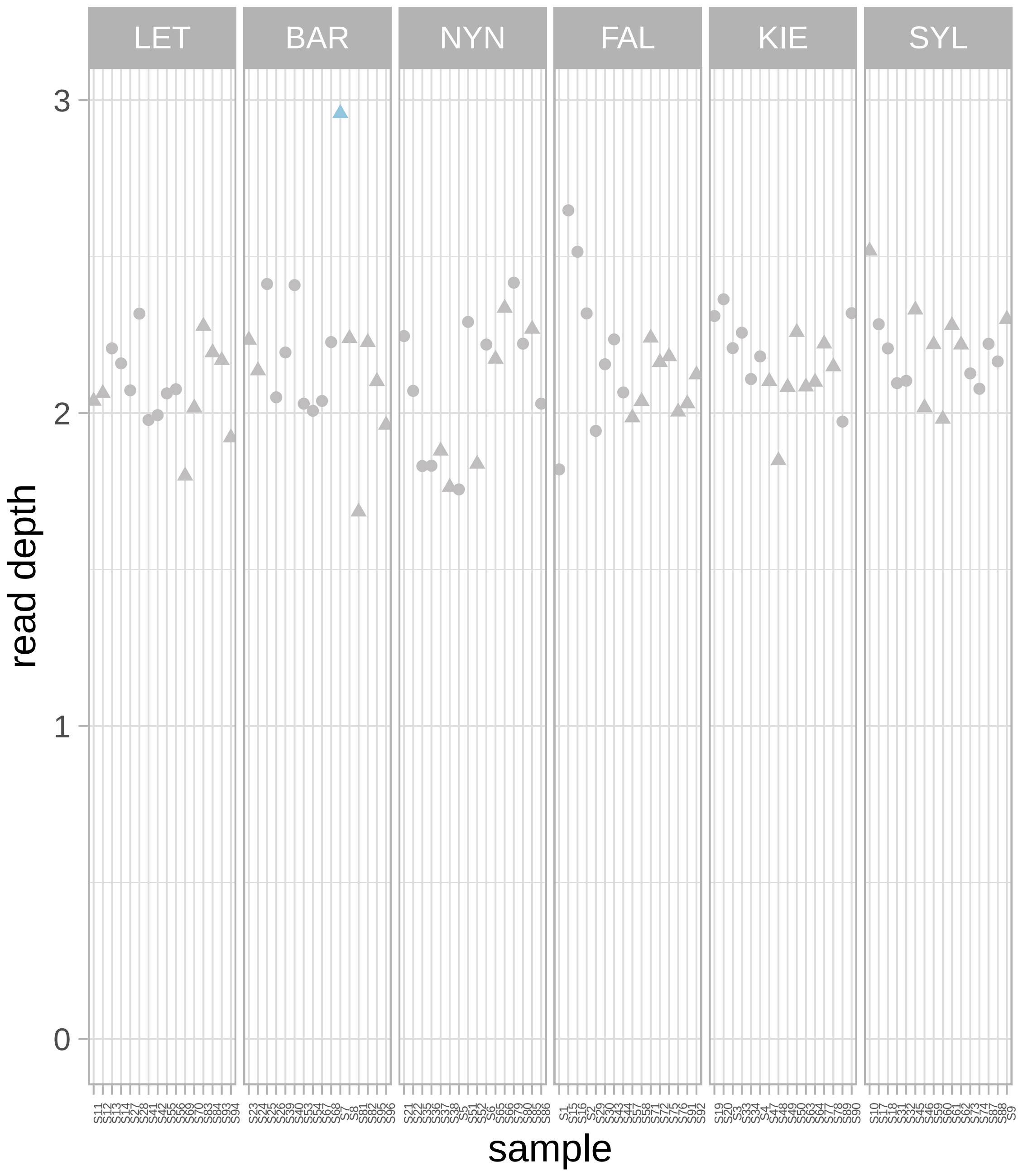

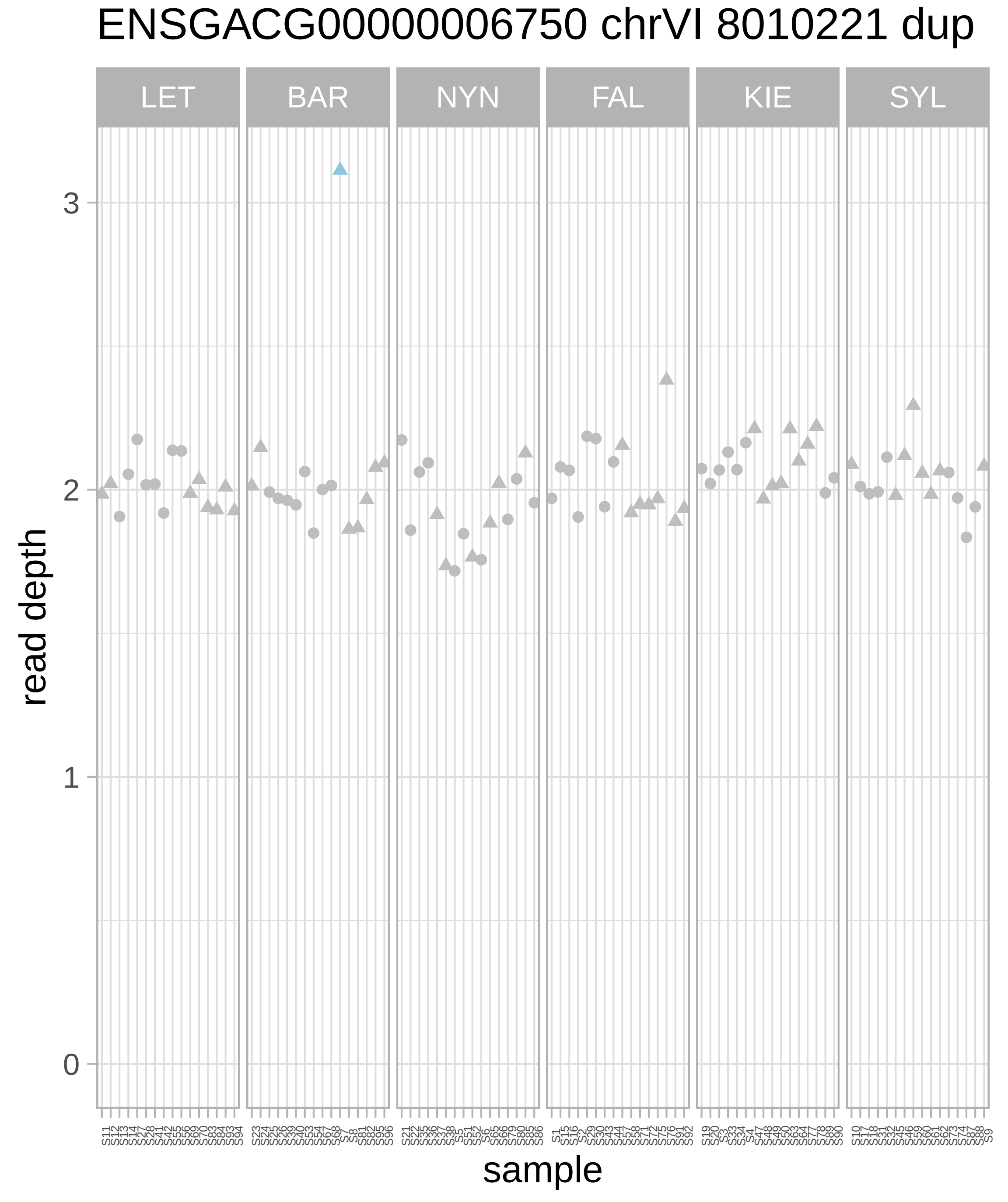
ENSGACG00000006750 chrVI 8010221 dup
LET
BAR
NYN
FAL
KIE
SYL
3
2
1
0
read depth
sample

ENSGACG00000006768 chrVI 8018384 dup

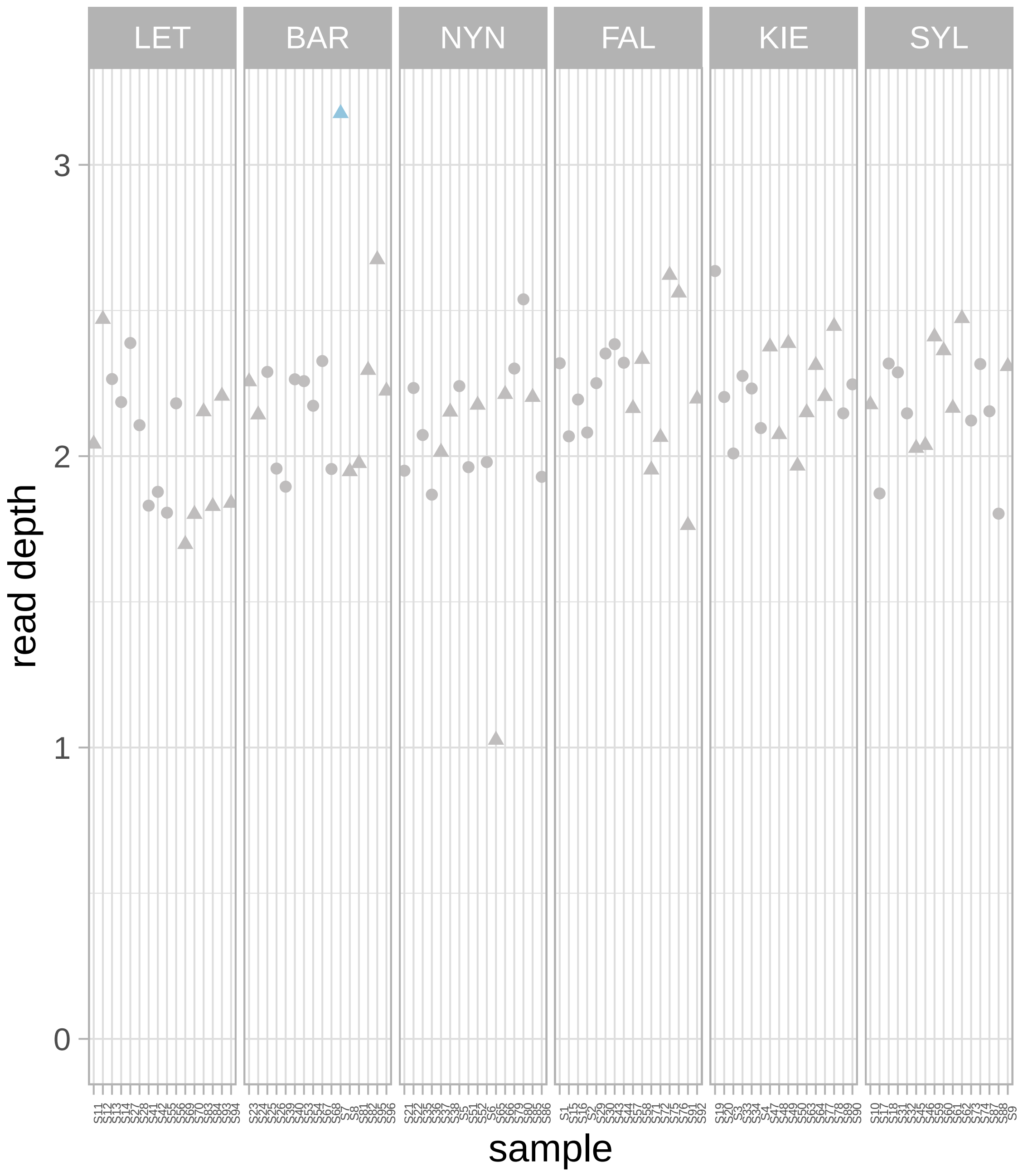

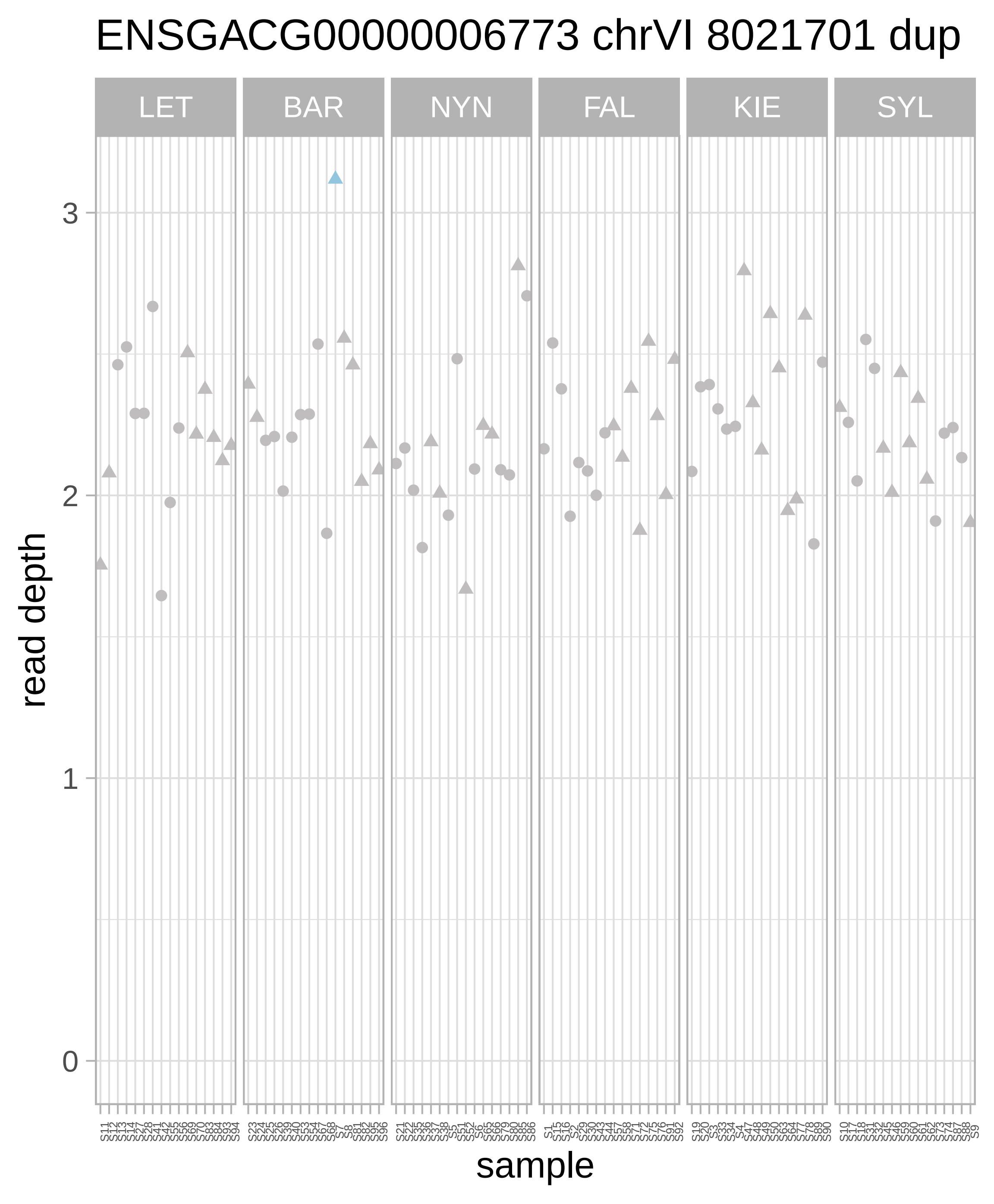
ENSGACG00000006773 chrVI 8021701 dup
LET
BAR
NYN
FAL
KIE
SYL
read depth
0
1
2
3
sample

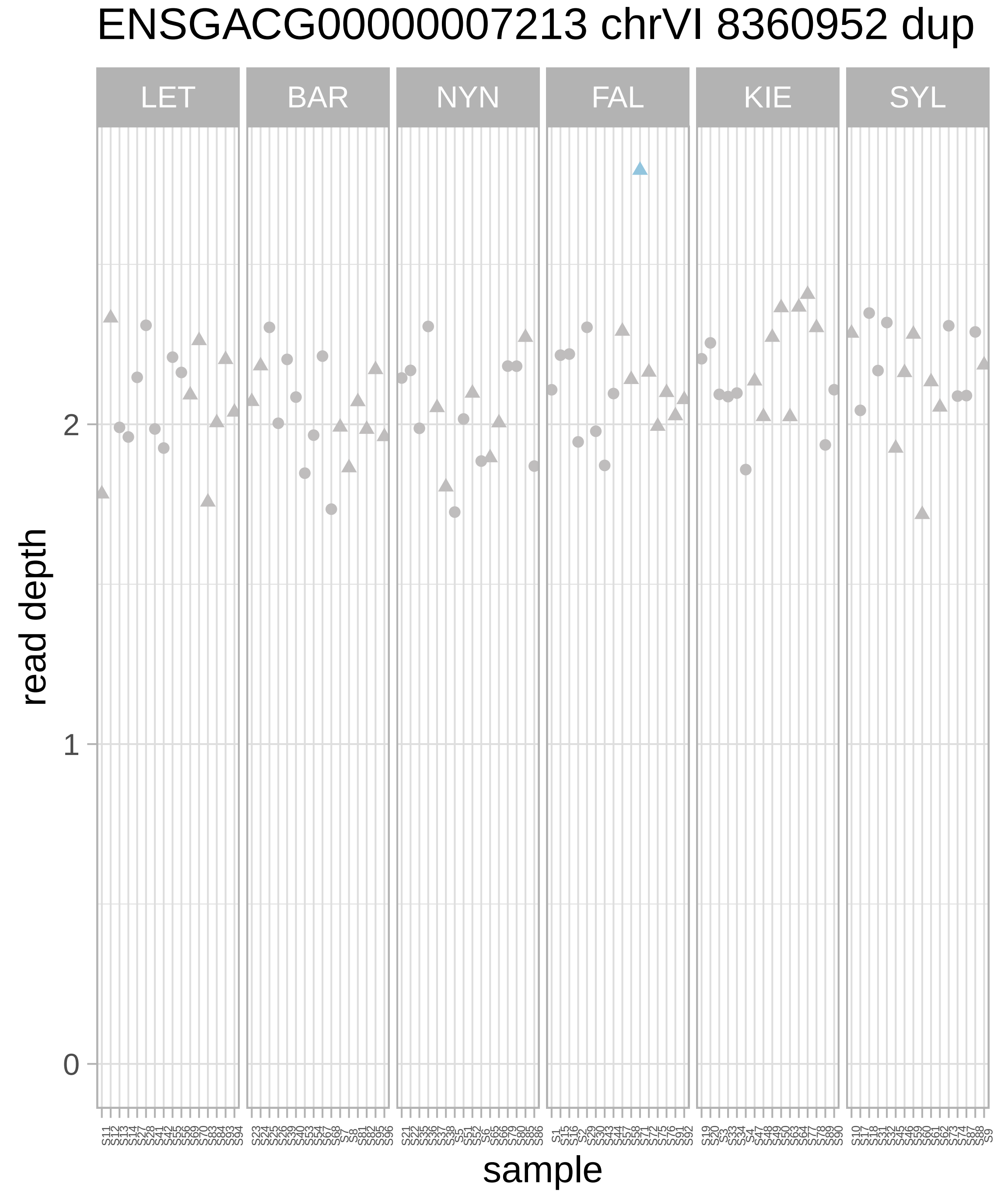
ENSGACG00000007213 chrVI 8360952 dup
LET
BAR
NYN
FAL
KIE
SYL
read depth
0
1
2
sample
S11 S12 S13 S14 S27 S28 S41 S42 S55 S56 S69 S70 S83 S84 S93 S94
S23 S24 S25 S26 S39 S40 S53 S54 S67 S68 S7 S8 S81 S82 S95 S96
S21 S22 S35 S36 S37 S38 S5 S51 S52 S6 S65 S66 S79 S80 S85 S86
S1 S15 S16 S2 S29 S30 S43 S44 S57 S58 S71 S72 S75 S76 S91 S92
S19 S20 S3 S33 S34 S4 S47 S48 S49 S50 S63 S64 S77 S78 S89 S90
S10 S17 S18 S31 S32 S45 S46 S59 S60 S61 S62 S73 S74 S87 S88 S9

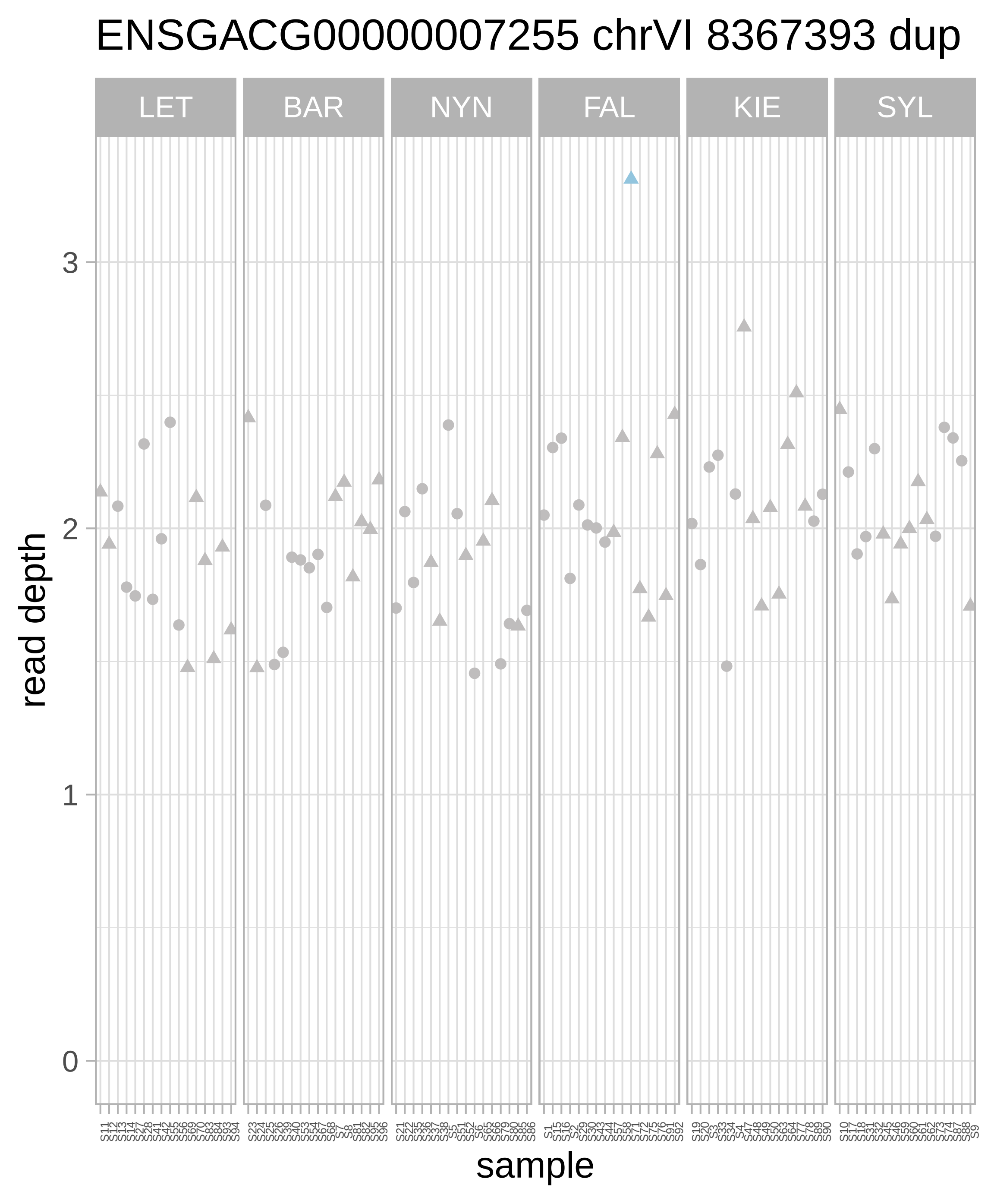

ENSGACG00000007255 chrVI 8367393 dup
LET
BAR
NYN
FAL
KIE
SYL
read depth
sample
0
1
2
3

ENSGACG00000007419 chrVI 8589741 dup

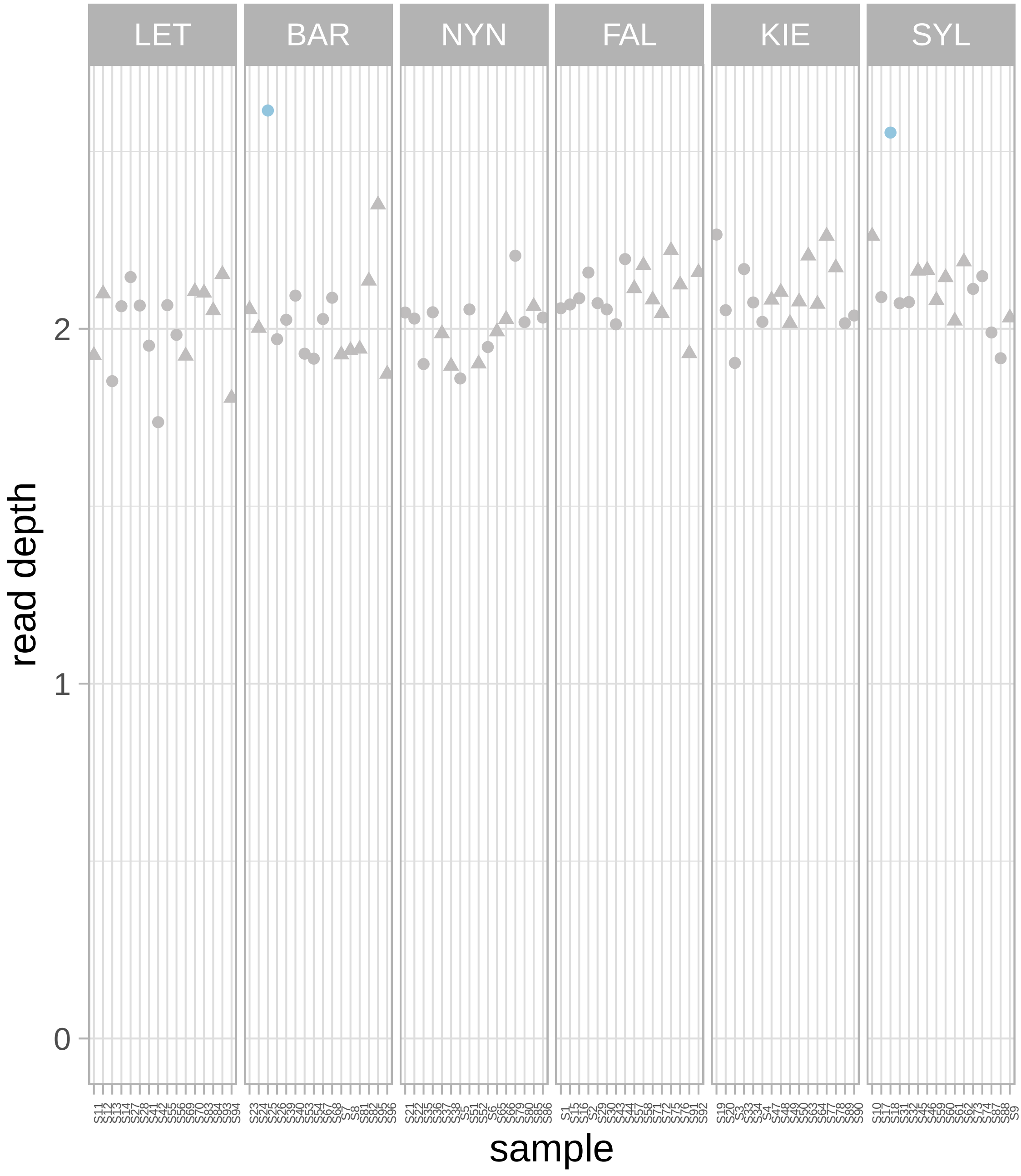

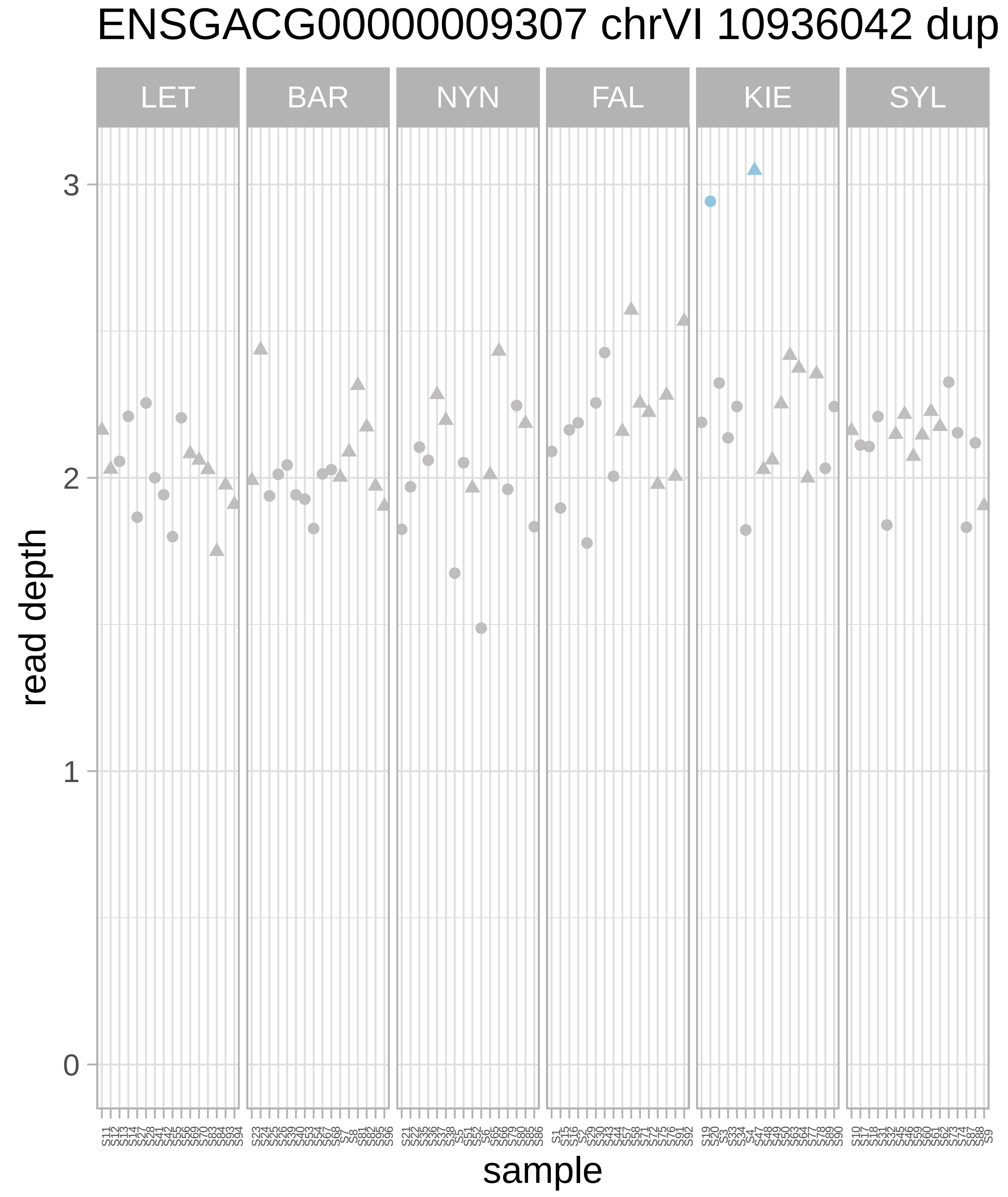

ENSGACG00000009307 chrVI 10936042 dup
LET
BAR
NYN
FAL
KIE
SYL
3
2
1
0
read depth
sample

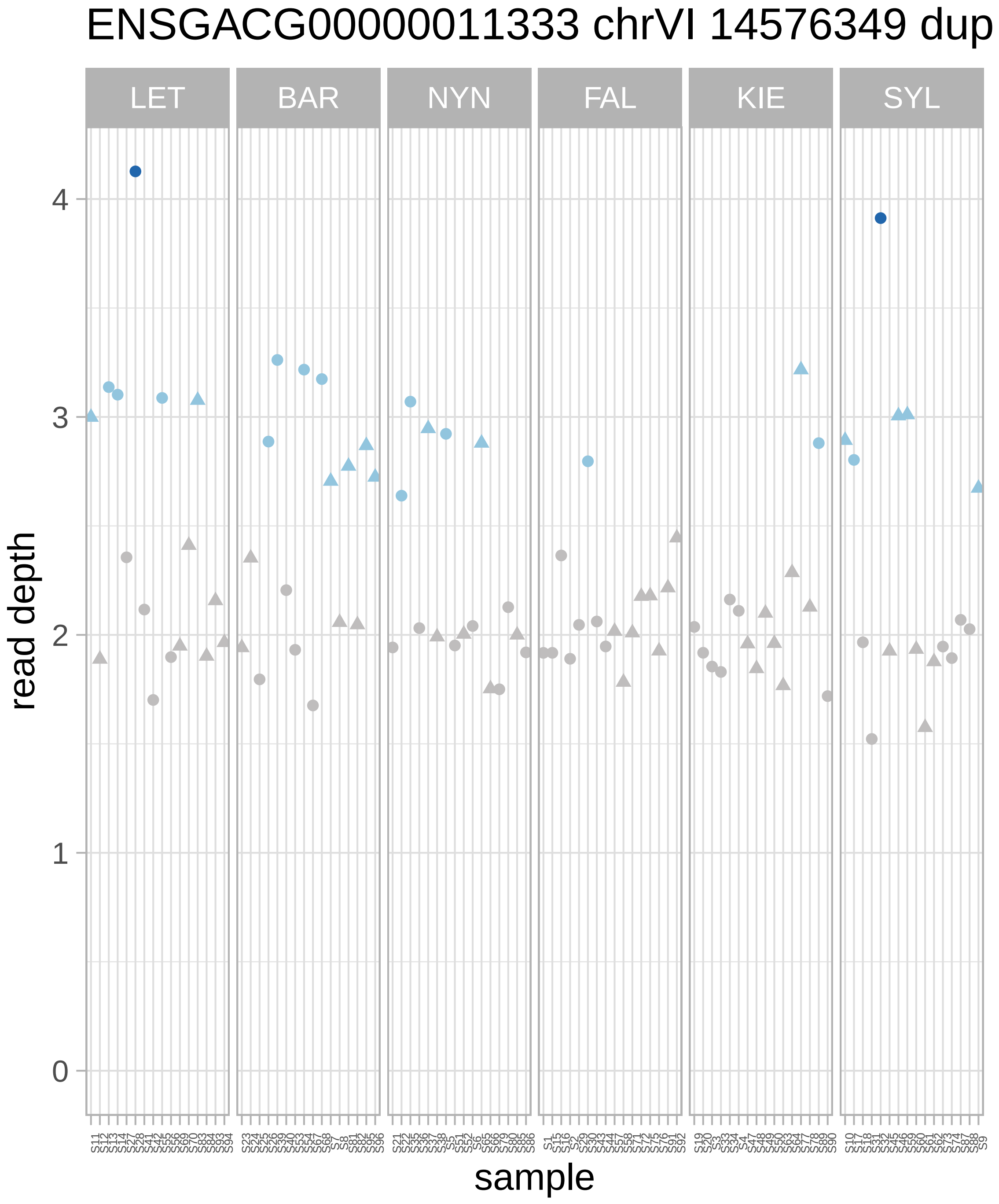
ENSGACG00000011333 chrVI 14576349 dup
LET
BAR
NYN
FAL
KIE
SYL
read depth
0
1
2
3
4
sample

ENSGACG00000011558 chrVI 15393127 dup

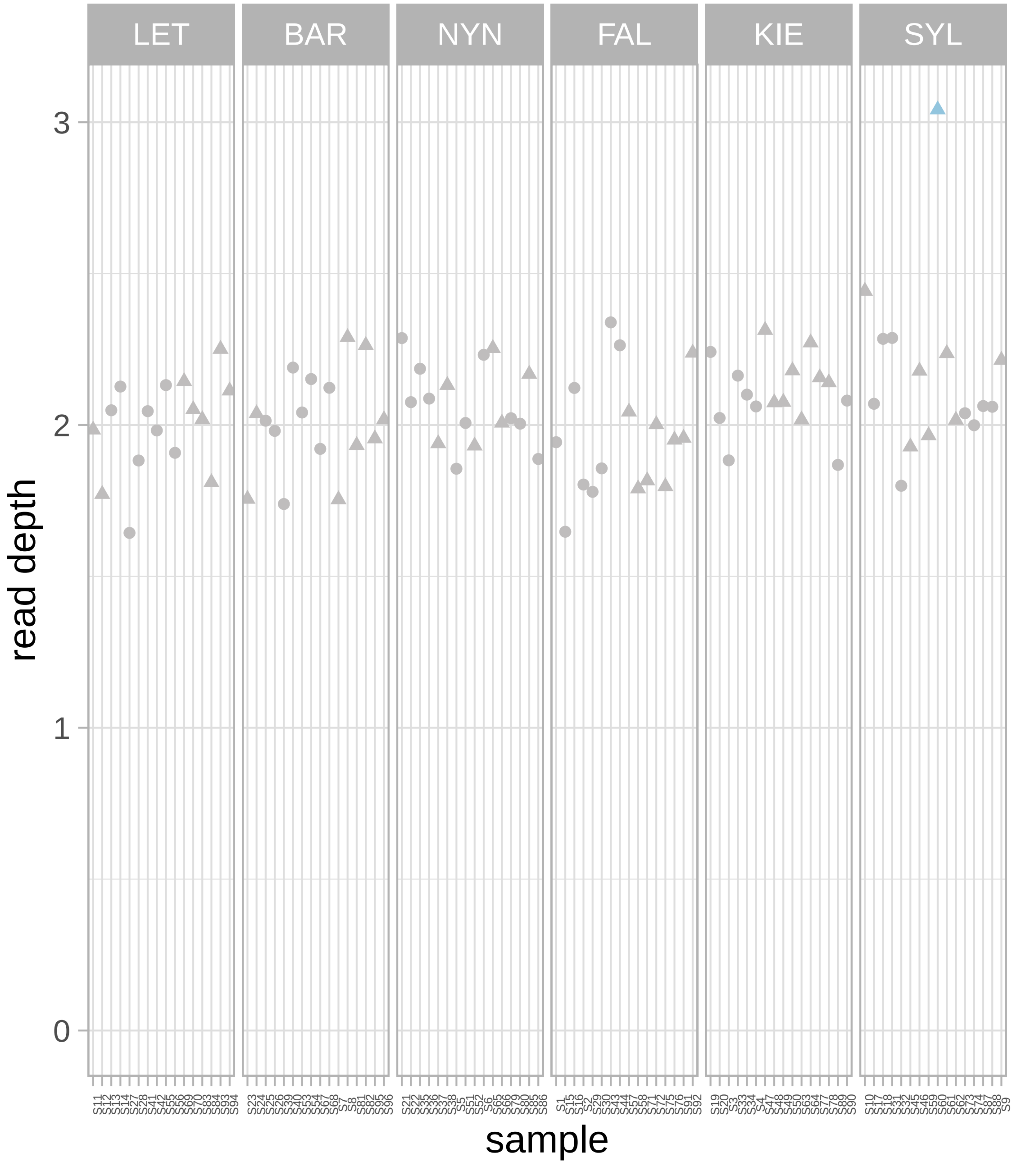

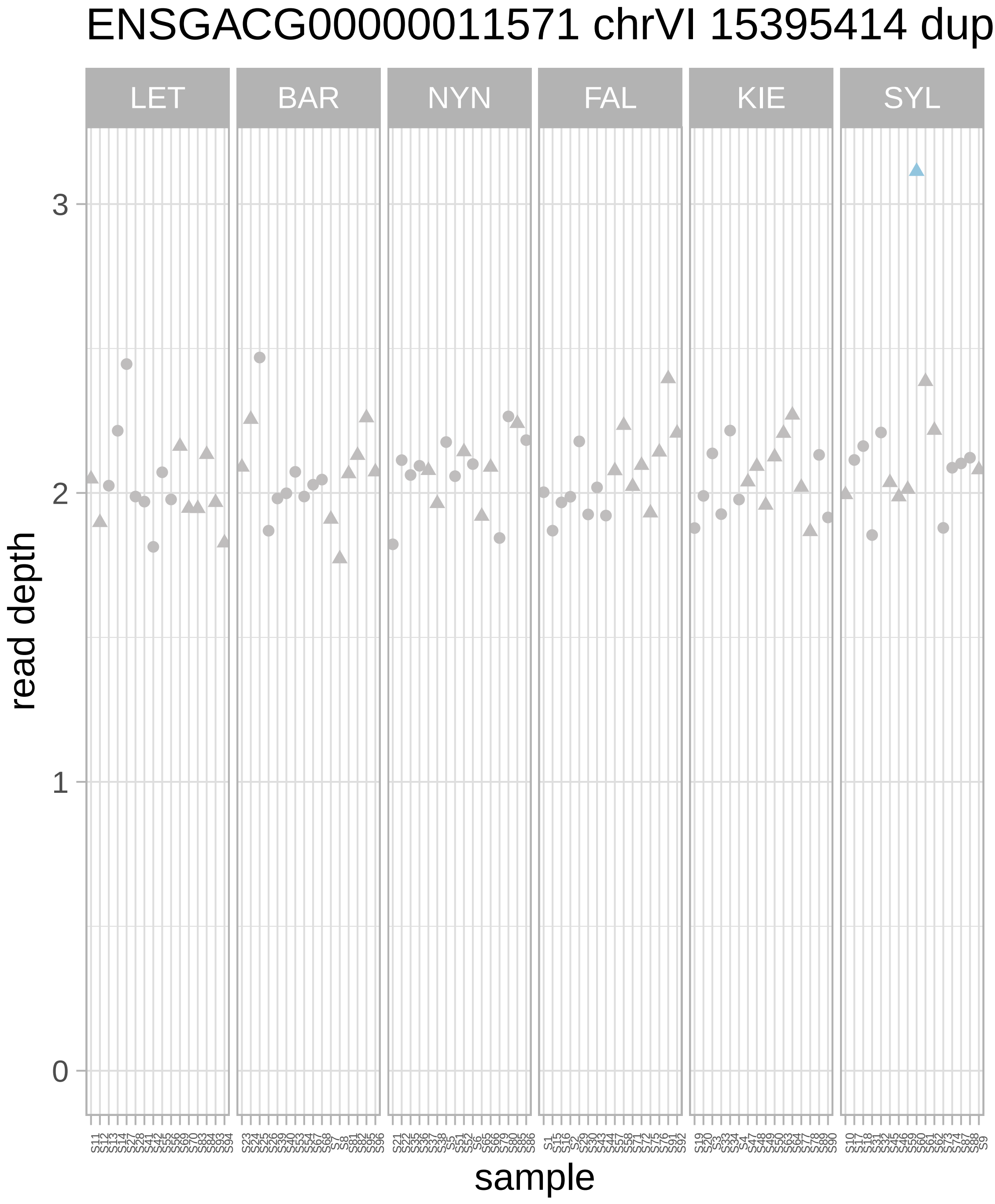
ENSGACG00000011571 chrVI 15395414 dup
LET
BAR
NYN
FAL
KIE
SYL
read depth
0
1
2
3
S11 S12 S13 S14 S27 S28 S41 S42 S55 S56 S69 S70 S83 S84 S93 S94
S23 S24 S25 S26 S39 S40 S53 S54 S67 S68 S7 S8 S81 S82 S95 S96
S21 S22 S35 S36 S37 S38 S5 S51 S52 S6 S65 S66 S79 S80 S85 S86
S1 S15 S16 S2 S29 S30 S43 S44 S57 S58 S71 S72 S75 S76 S91 S92
S19 S20 S3 S33 S34 S4 S47 S48 S49 S50 S63 S64 S77 S78 S89 S90
S10 S17 S18 S31 S32 S45 S46 S59 S60 S61 S62 S73 S74 S87 S88 S9
sample

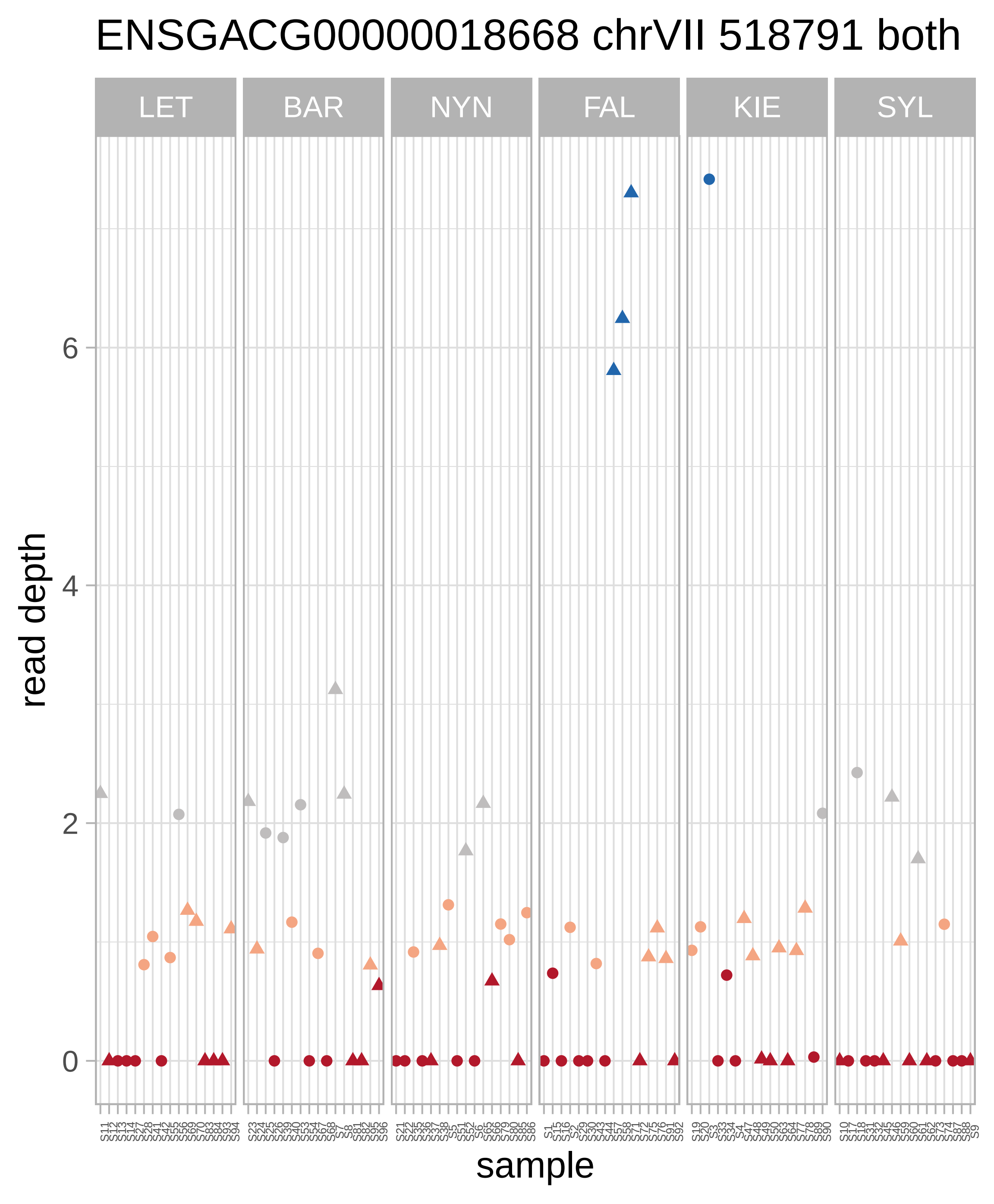
ENSGACG00000018668 chrVII 518791 both
LET
BAR
NYN
FAL
KIE
SYL
read depth
0
2
4
6
sample

ENSGACG00000018897 chrVII 1522986 dup

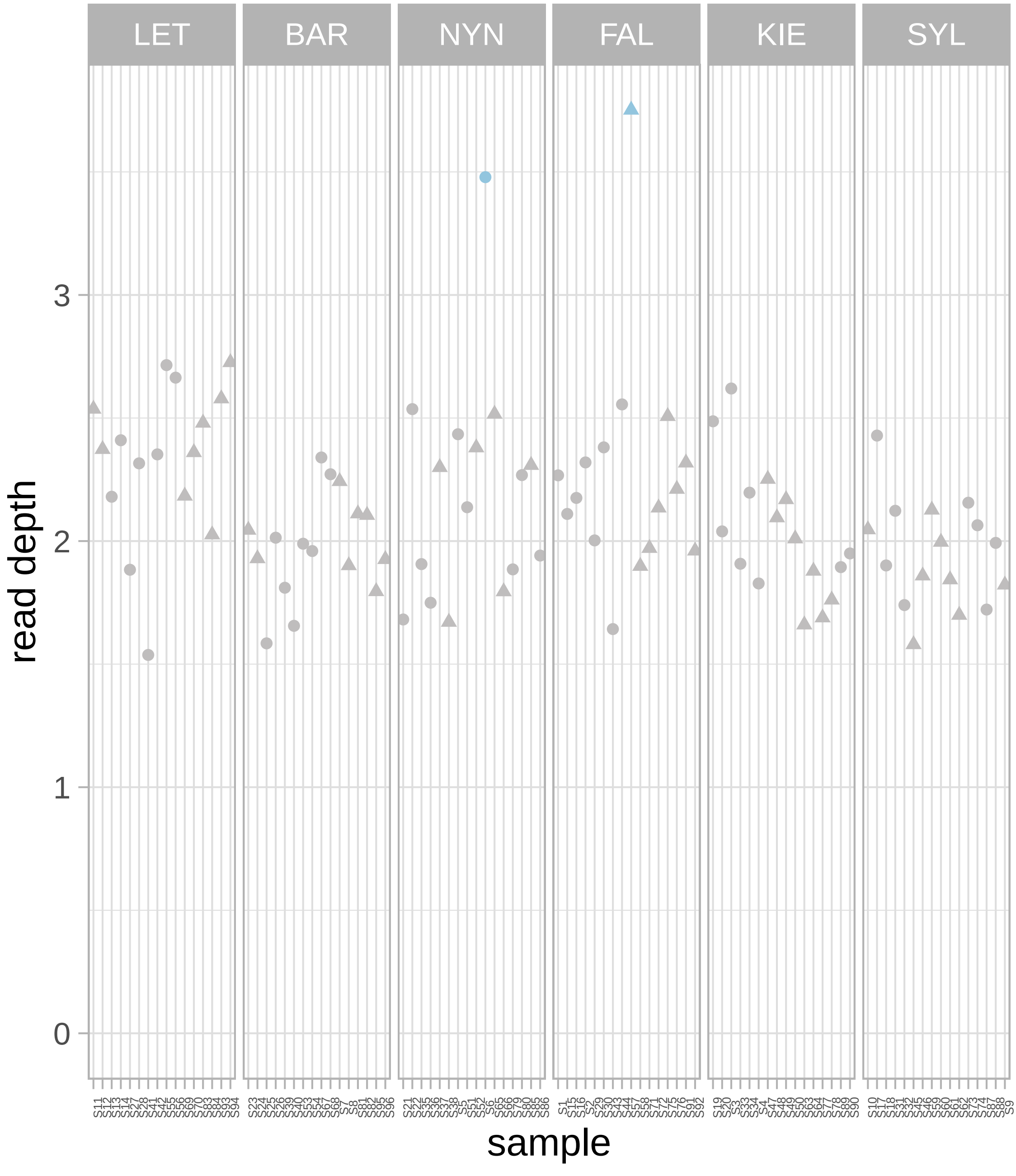

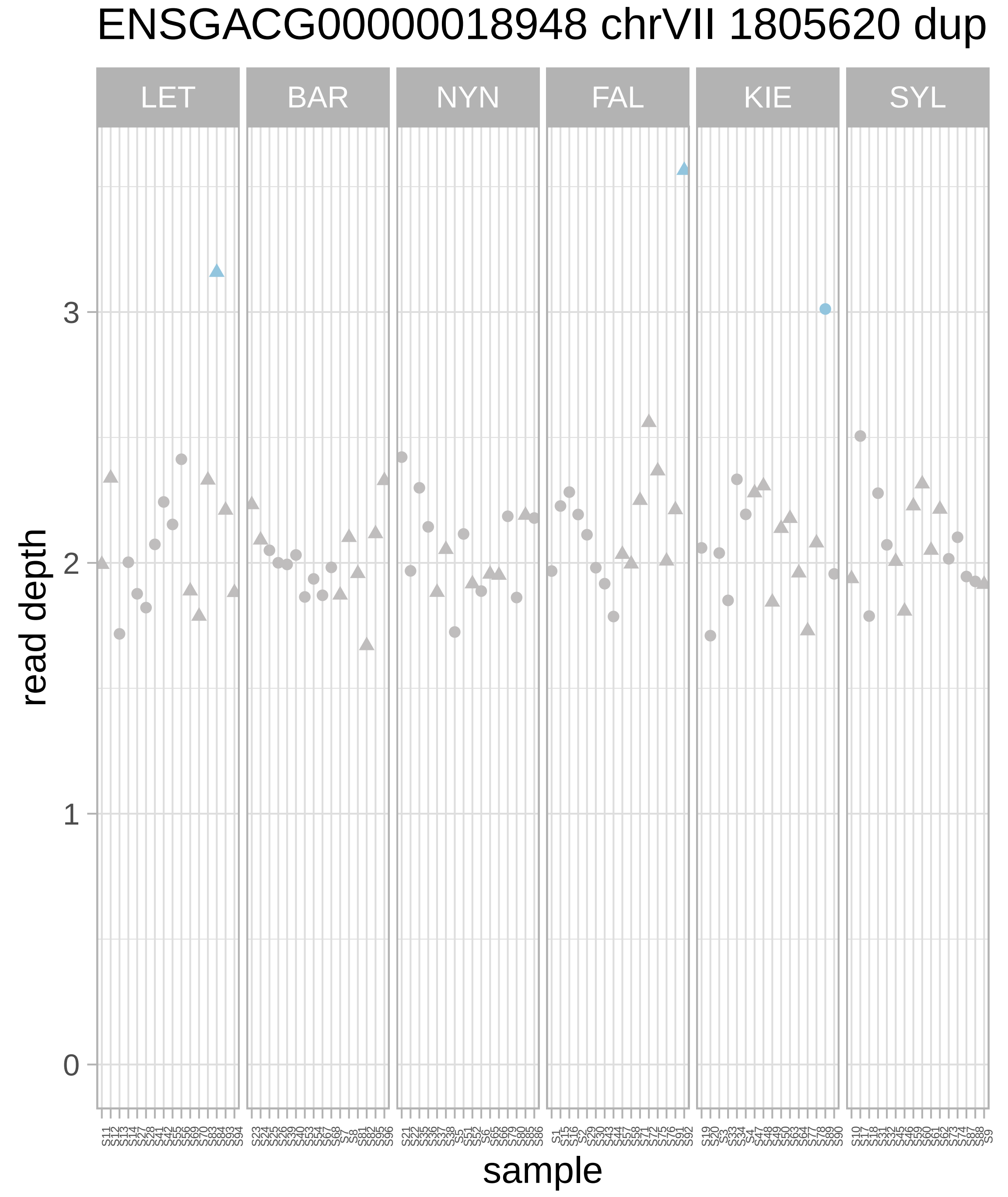
ENSGACG00000018948 chrVII 1805620 dup
LET
BAR
NYN
FAL
KIE
SYL
read depth
3
2
1
0
sample

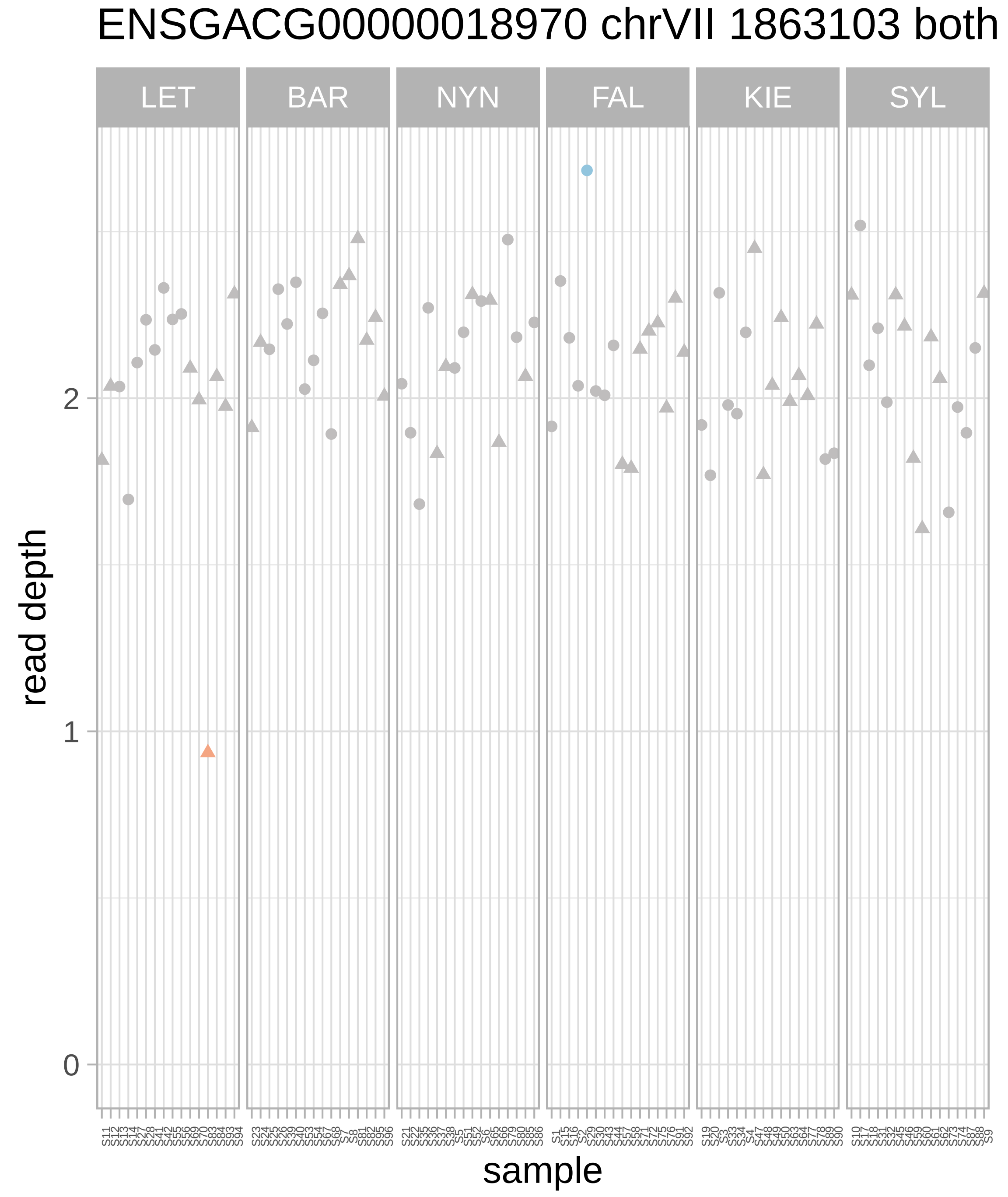
ENSGACG00000018970 chrVII 1863103 both
LET
BAR
NYN
FAL
KIE
SYL
read depth
0
1
2
S11 S12 S13 S14 S27 S28 S41 S42 S55 S56 S69 S70 S83 S84 S93 S94
S23 S24 S25 S26 S39 S40 S53 S54 S67 S68 S7 S8 S81 S82 S95 S96
S21 S22 S35 S36 S37 S38 S5 S51 S52 S6 S65 S66 S79 S80 S85 S86
S1 S15 S16 S2 S29 S30 S43 S44 S57 S58 S71 S72 S75 S76 S91 S92
S19 S20 S3 S33 S34 S4 S47 S48 S49 S50 S63 S64 S77 S78 S89 S90
S10 S17 S18 S31 S32 S45 S46 S59 S60 S61 S62 S73 S74 S87 S88 S9
sample

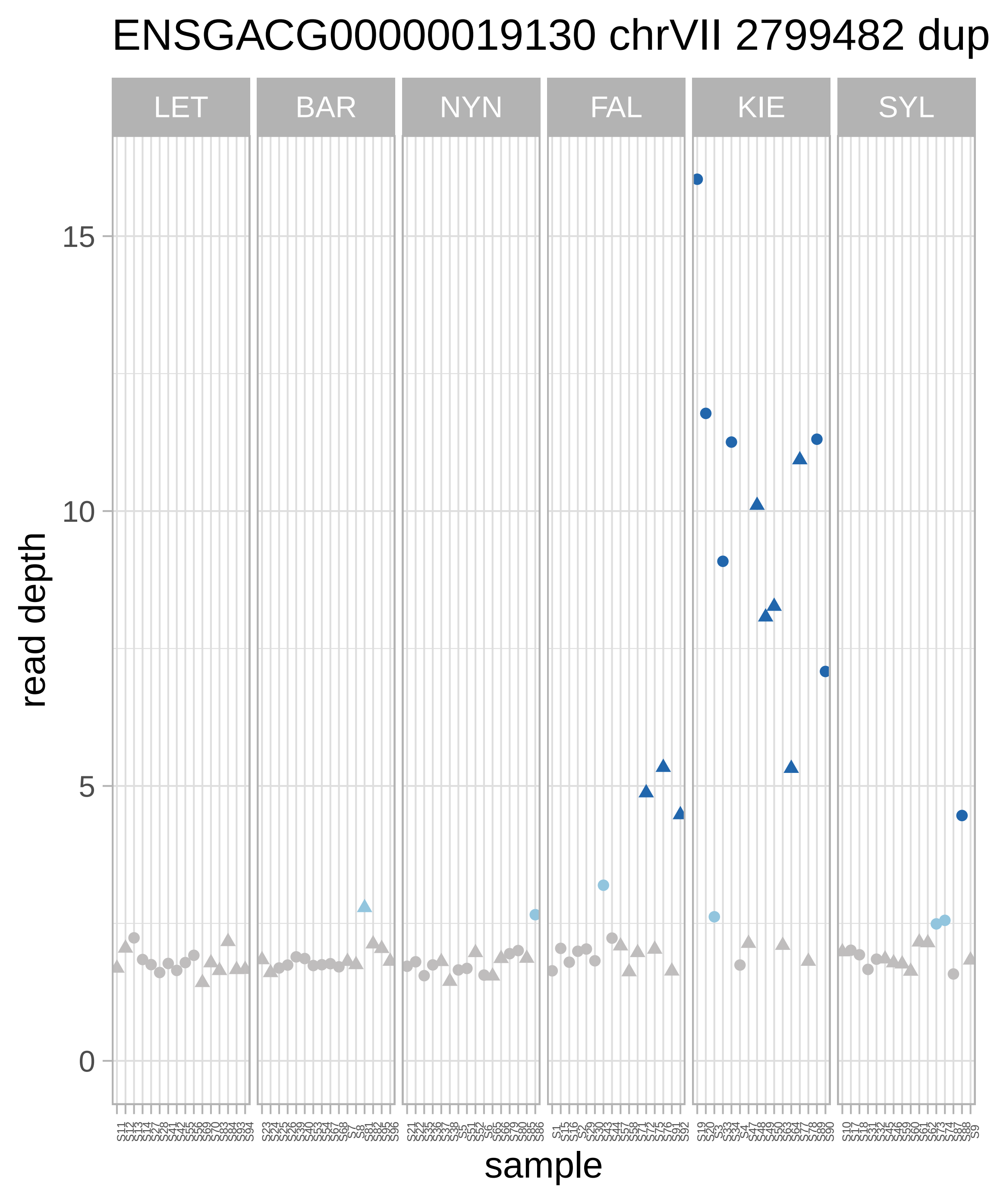
ENSGACG00000019130 chrVII 2799482 dup
LET
BAR
NYN
FAL
KIE
SYL
read depth
15
10
5
0
sample

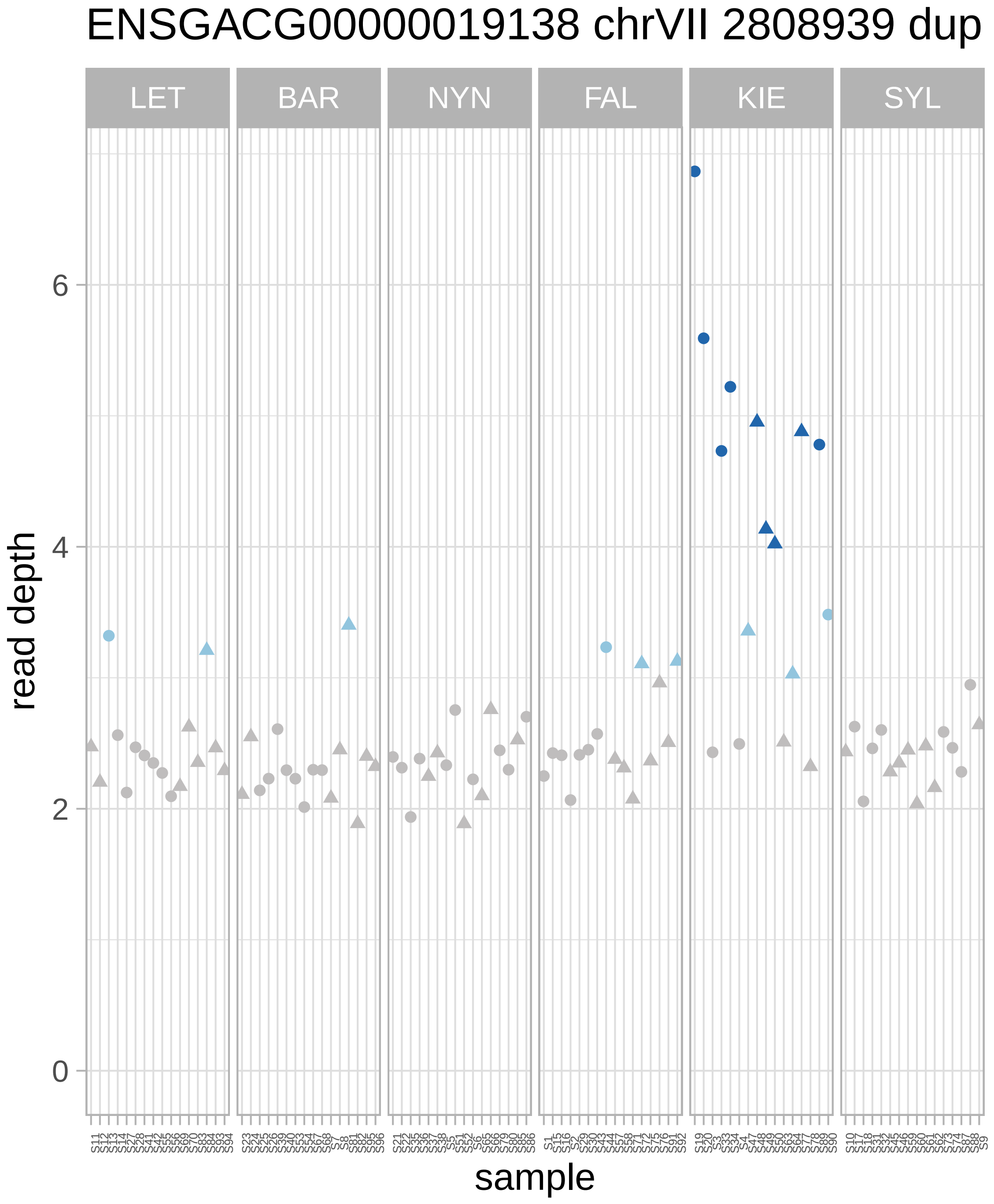
ENSGACG00000019138 chrVII 2808939 dup
LET
BAR
NYN
FAL
KIE
SYL
read depth
0
2
4
6
sample

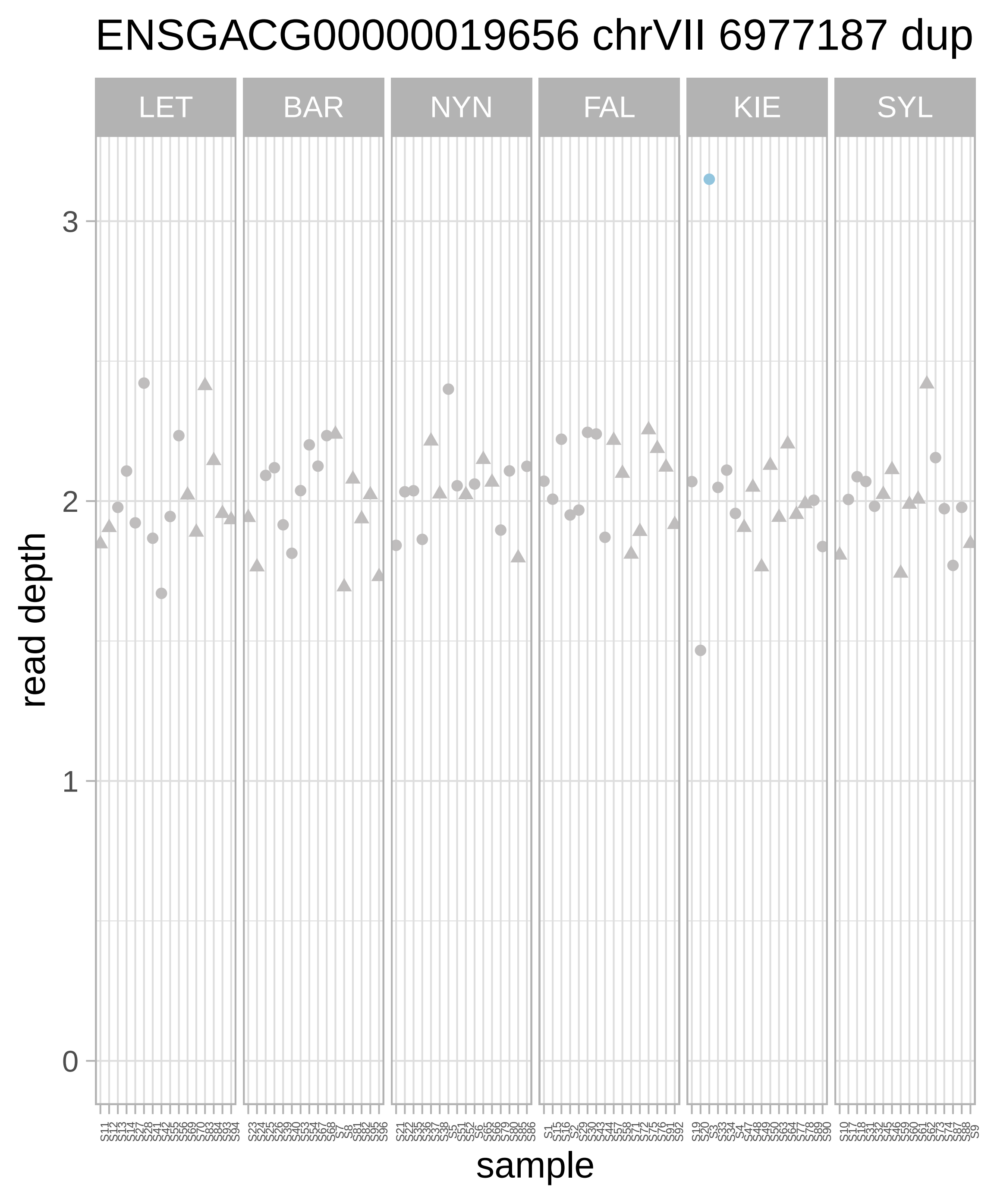
ENSGACG00000019656 chrVII 6977187 dup
LET
BAR
NYN
FAL
KIE
SYL
read depth
3
2
1
0
sample

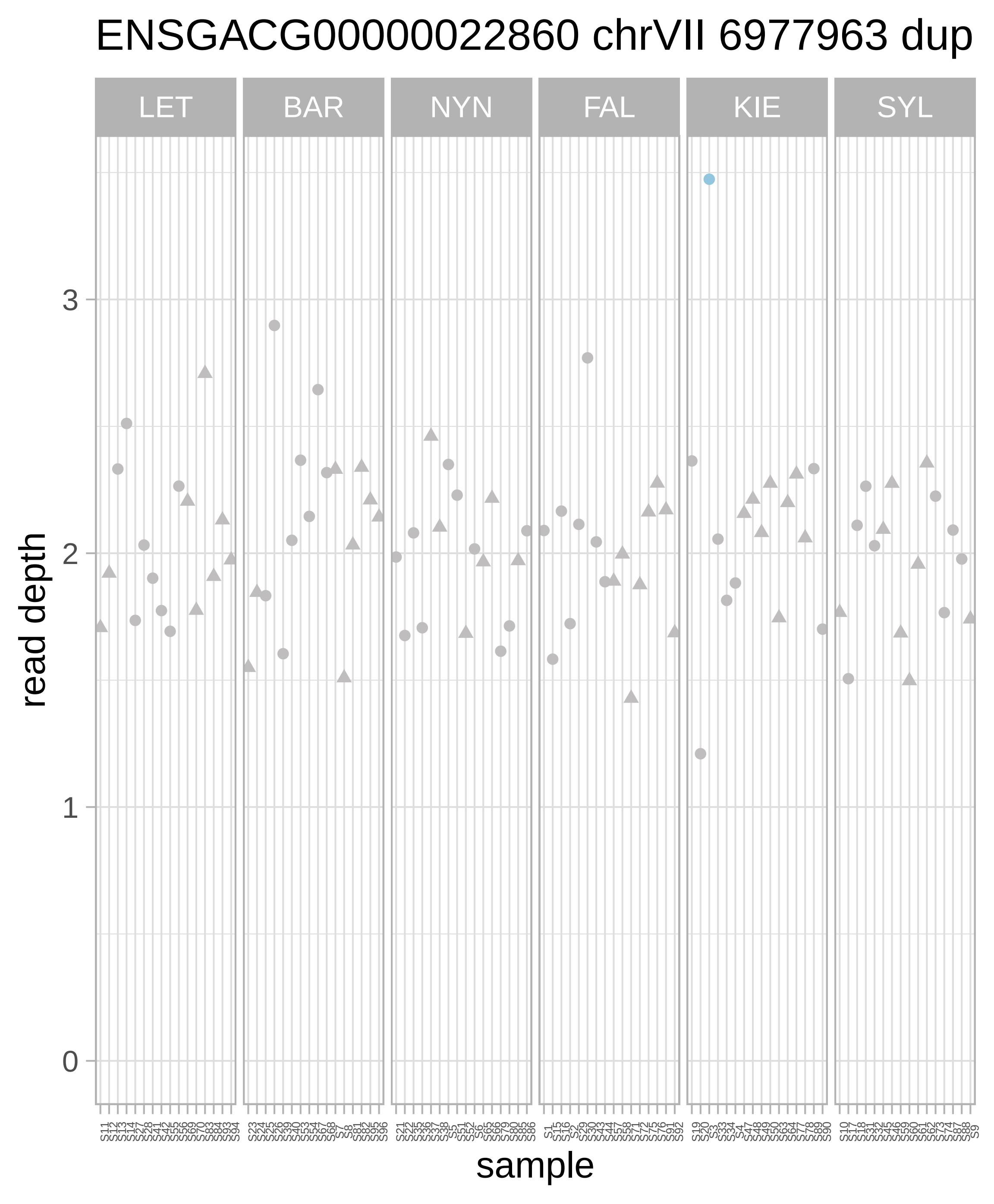
ENSGACG00000022860 chrVII 6977963 dup
LET
BAR
NYN
FAL
KIE
SYL
read depth
3
2
1
0
sample

ENSGACG00000019701 chrVII 7175722 dup

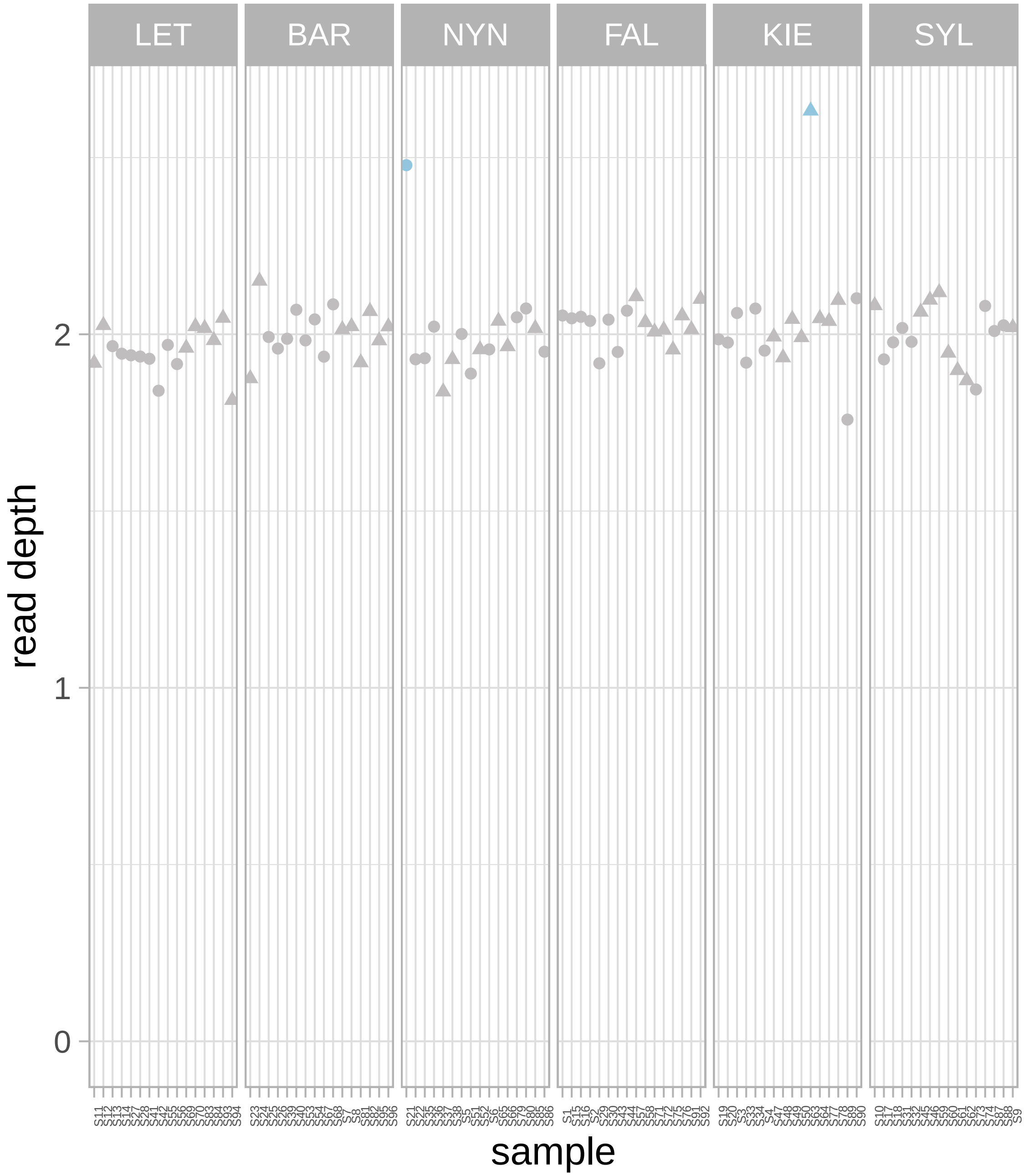

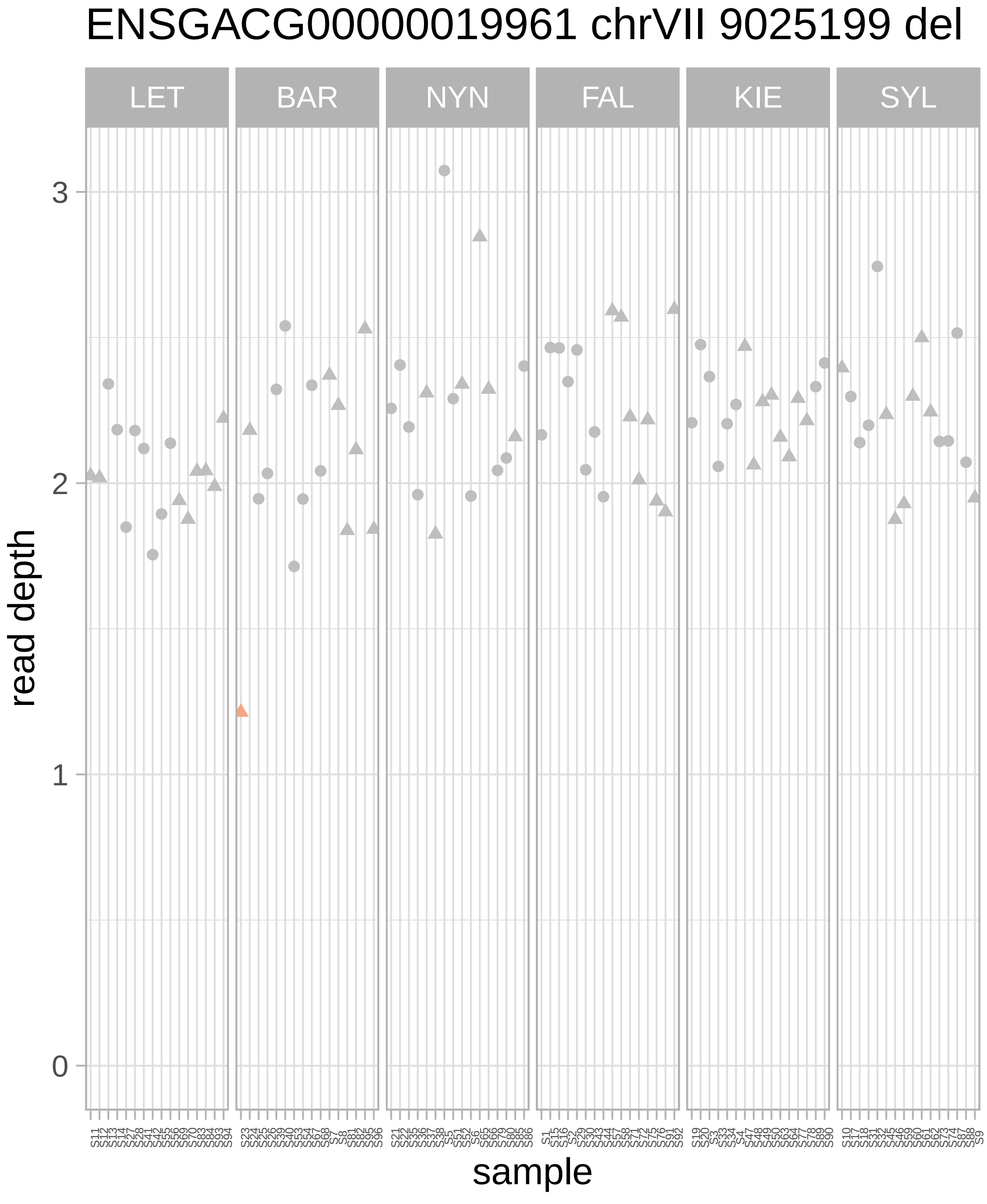
ENSGACG00000019961 chrVII 9025199 del
LET
BAR
NYN
FAL
KIE
SYL
read depth
0
1
2
3
sample

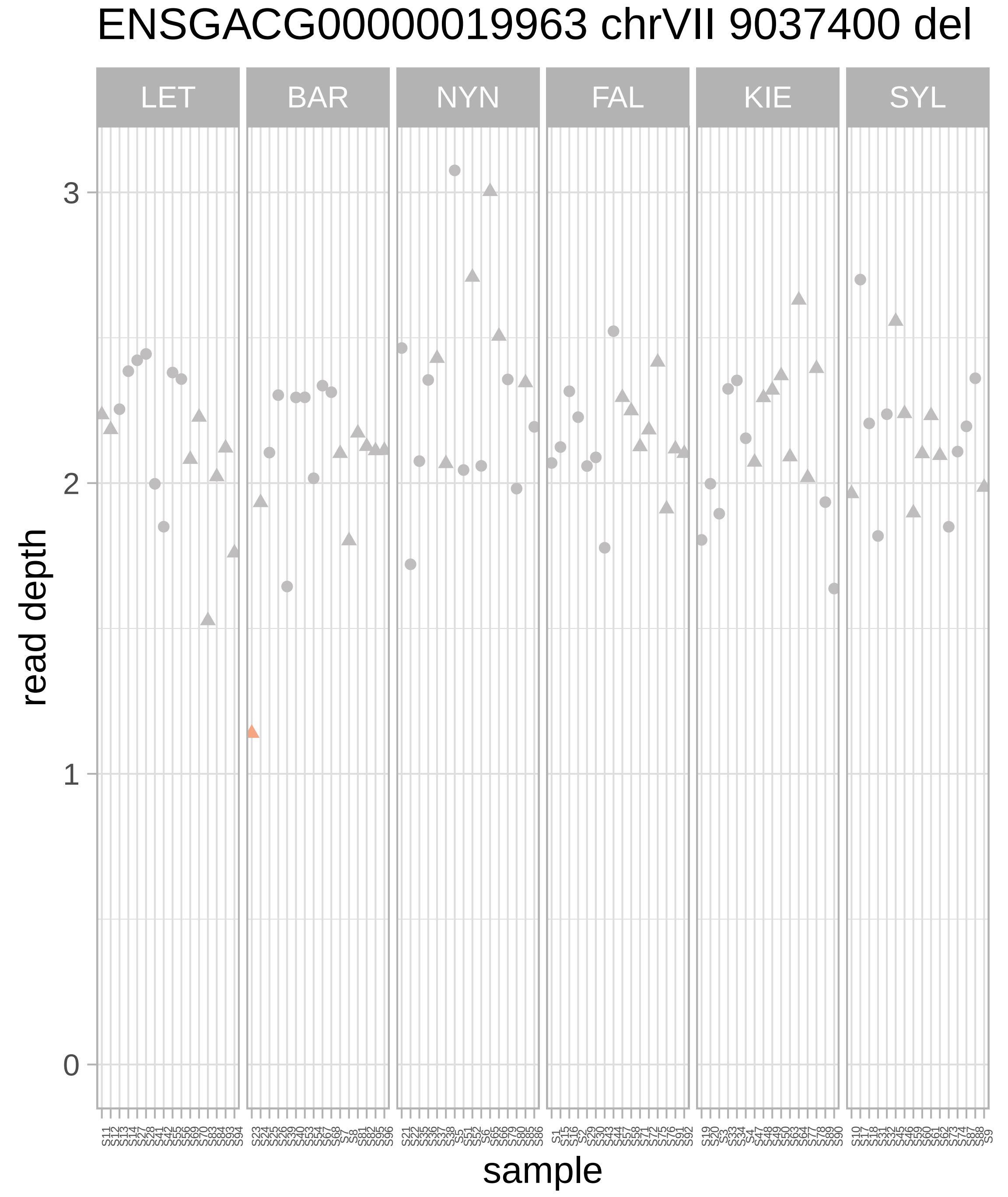
ENSGACG00000019963 chrVII 9037400 del
LET
BAR
NYN
FAL
KIE
SYL
read depth
0
1
2
3
S11 S12 S13 S14 S27 S28 S41 S42 S55 S56 S69 S70 S83 S84 S93 S94
S23 S24 S25 S26 S39 S40 S53 S54 S67 S68 S7 S8 S81 S82 S95 S96
S21 S22 S35 S36 S37 S38 S5 S51 S52 S6 S65 S66 S79 S80 S85 S86
S1 S15 S16 S2 S29 S30 S43 S44 S57 S58 S71 S72 S75 S76 S91 S92
S19 S20 S3 S33 S34 S4 S47 S48 S49 S50 S63 S64 S77 S78 S89 S90
S10 S17 S18 S31 S32 S45 S46 S59 S60 S61 S62 S73 S74 S87 S88 S9
sample

ENSGACG00000019964 chrVII 9049945 del

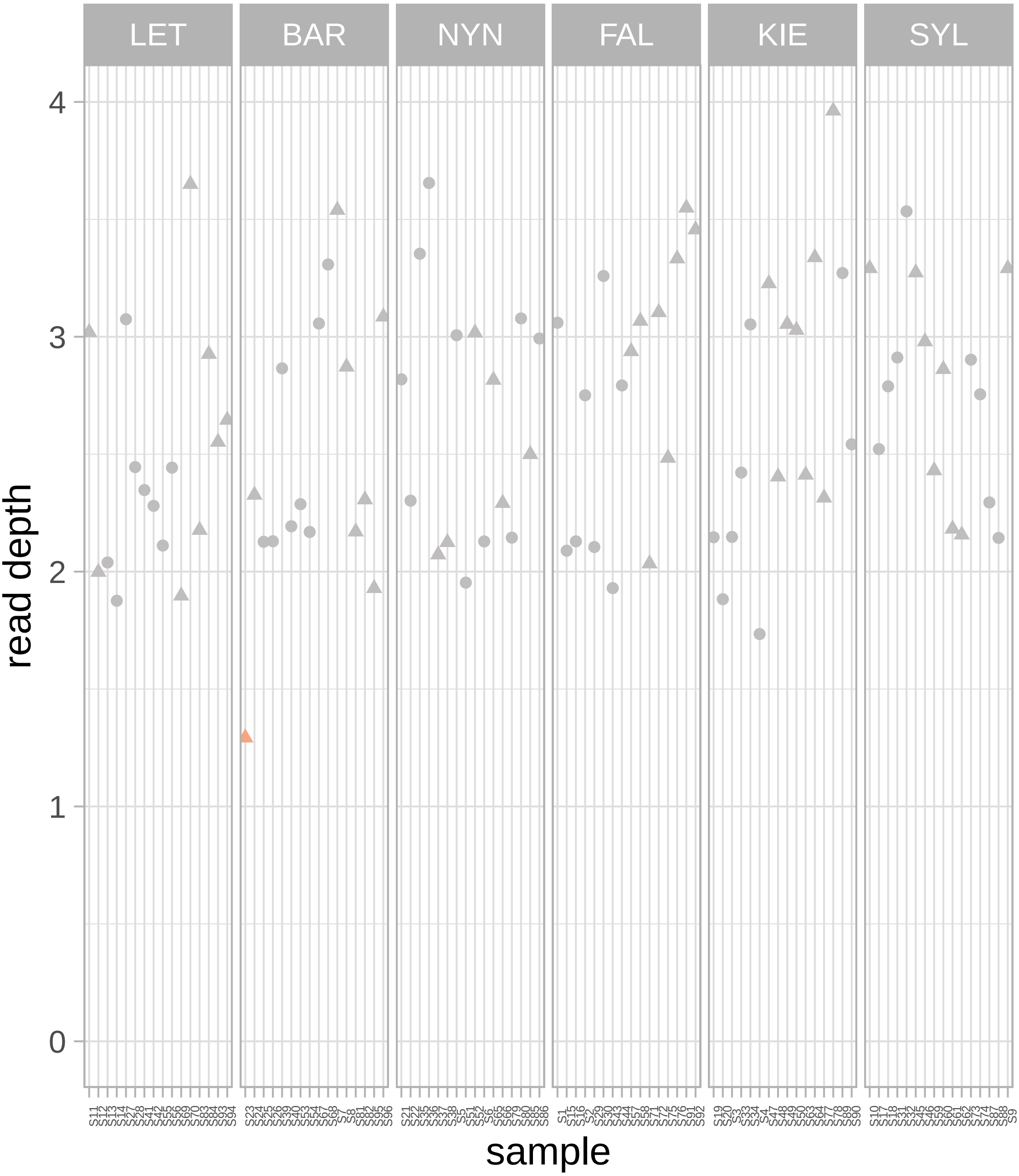

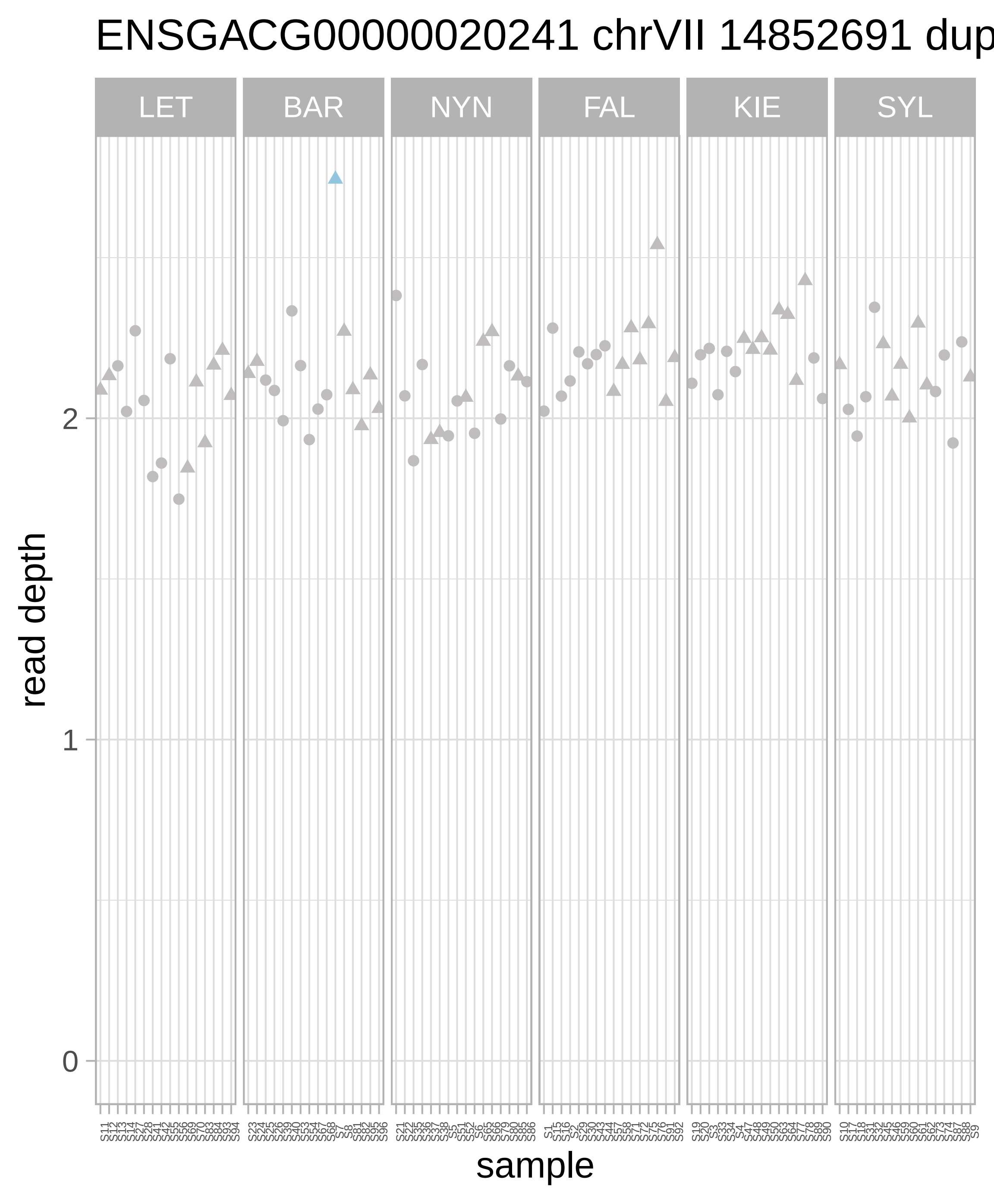
ENSGACG00000020241 chrVII 14852691 dup
LET
BAR
NYN
FAL
KIE
SYL
read depth
0
1
2
sample

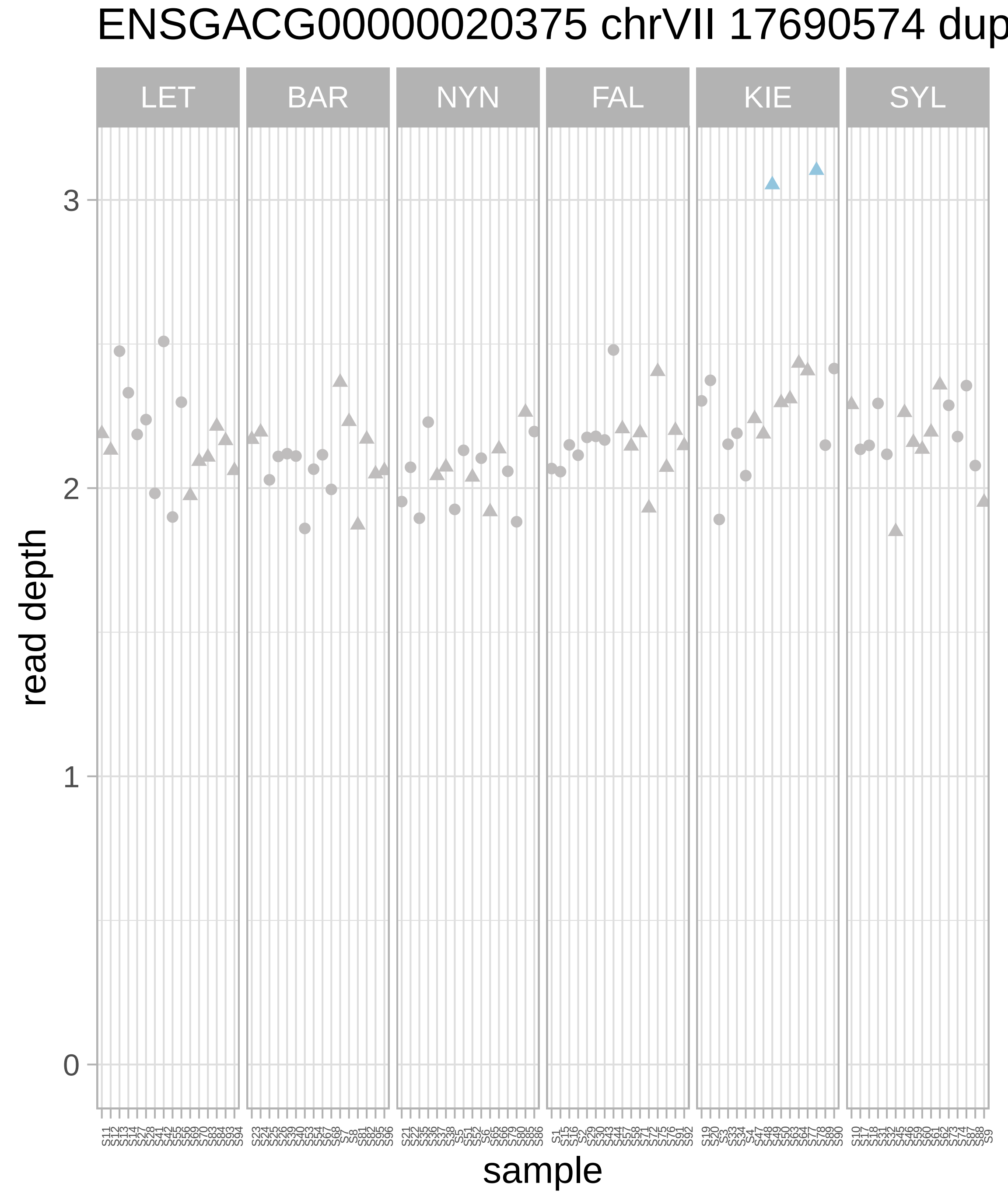
ENSGACG00000020375 chrVII 17690574 dup
LET
BAR
NYN
FAL
KIE
SYL
read depth
3
2
1
0
sample

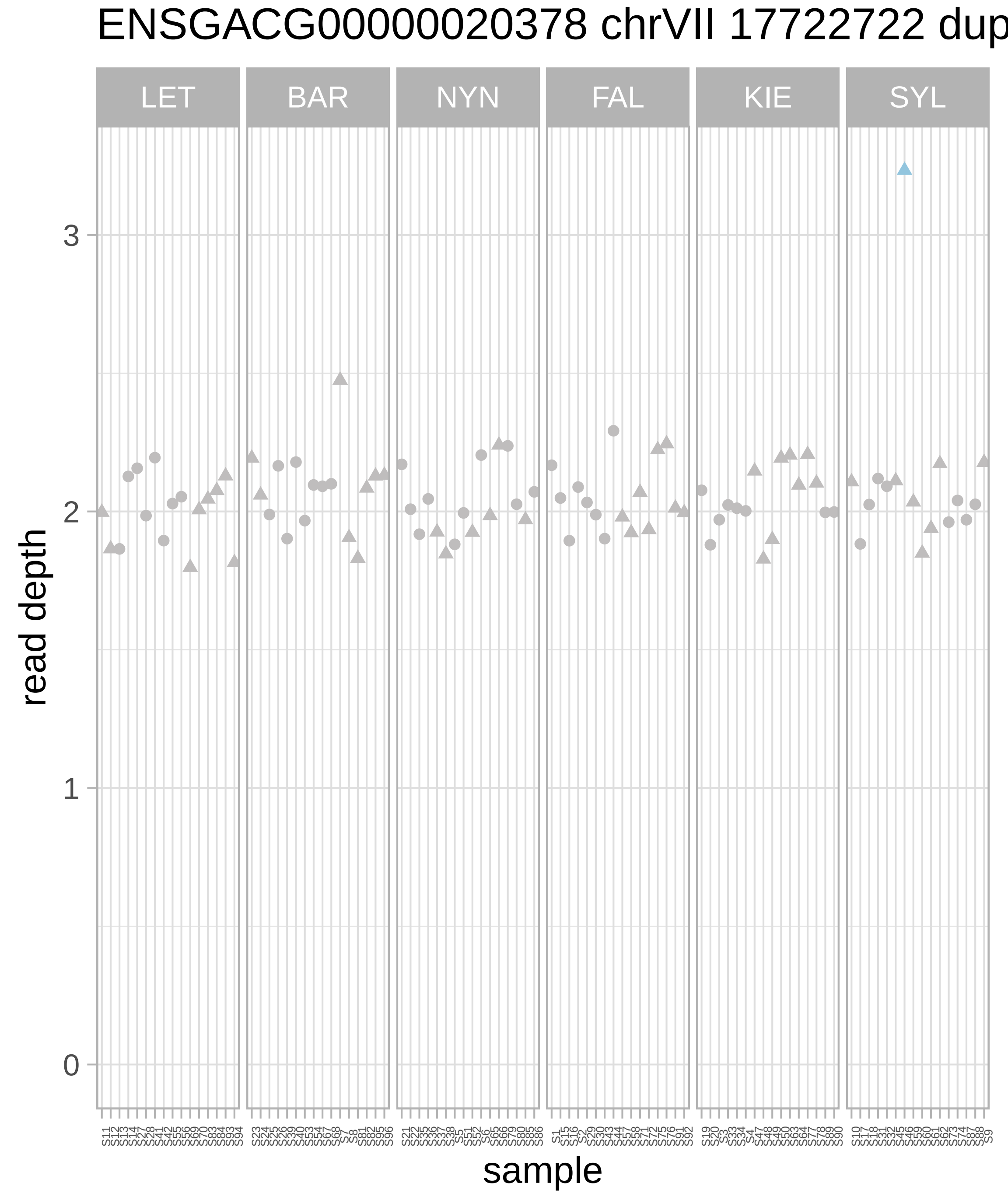
ENSGACG00000020378 chrVII 17722722 dup
LET
BAR
NYN
FAL
KIE
SYL
read depth
0
1
2
3
sample
S11 S12 S13 S14 S27 S28 S41 S42 S55 S56 S69 S70 S83 S84 S93 S94
S23 S24 S25 S26 S39 S40 S53 S54 S67 S68 S7 S8 S81 S82 S95 S96
S21 S22 S35 S36 S37 S38 S5 S51 S52 S6 S65 S66 S79 S80 S85 S86
S1 S15 S16 S2 S29 S30 S43 S44 S57 S58 S71 S72 S75 S76 S91 S92
S19 S20 S3 S33 S34 S4 S47 S48 S49 S50 S63 S64 S77 S78 S89 S90
S10 S17 S18 S31 S32 S45 S46 S59 S60 S61 S62 S73 S74 S87 S88 S9

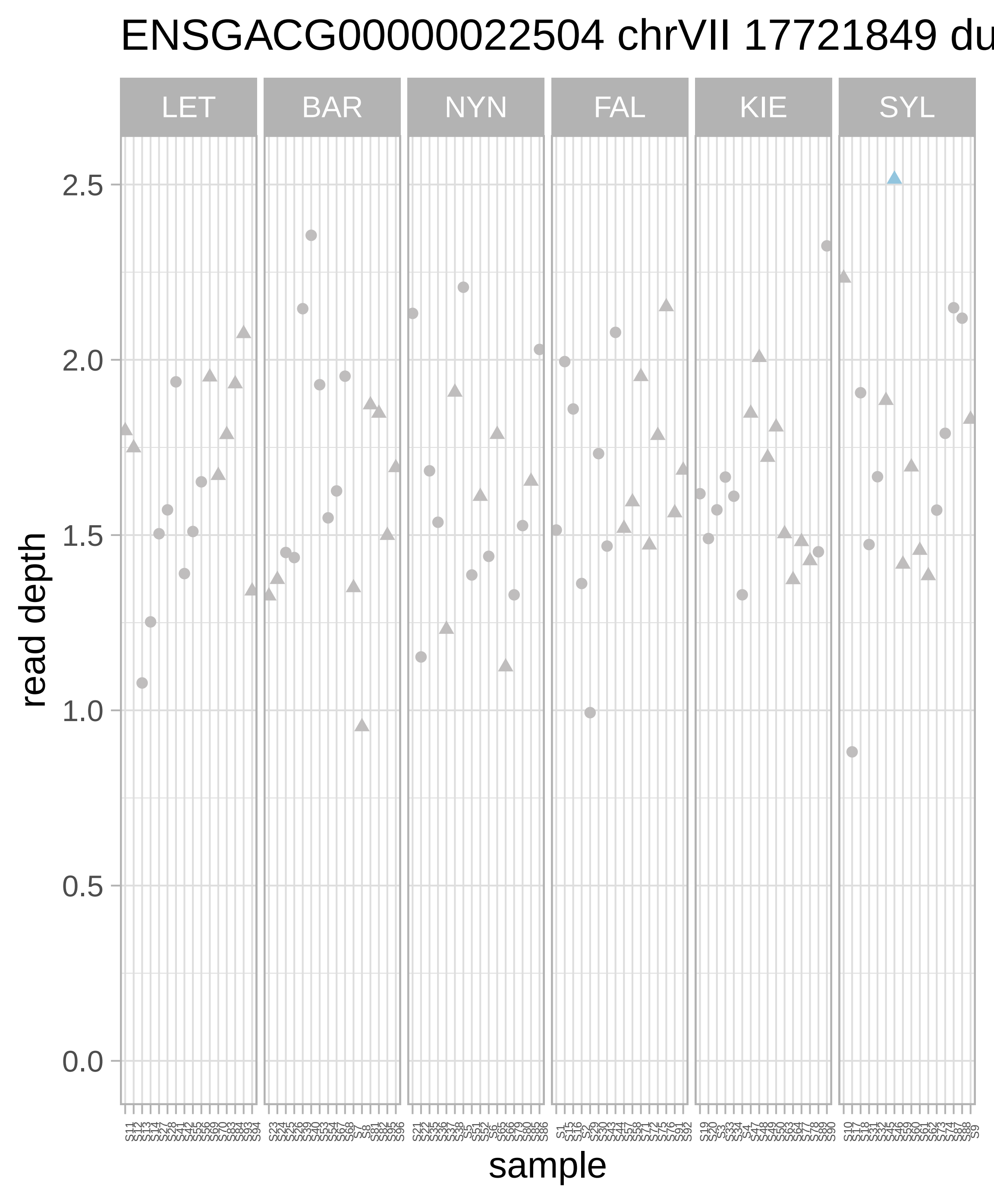

ENSGACG00000022504 chrVII 17721849 du
LET
BAR
NYN
FAL
KIE
SYL
read depth
2.5
2.0
1.5
1.0
0.5
0.0
sample

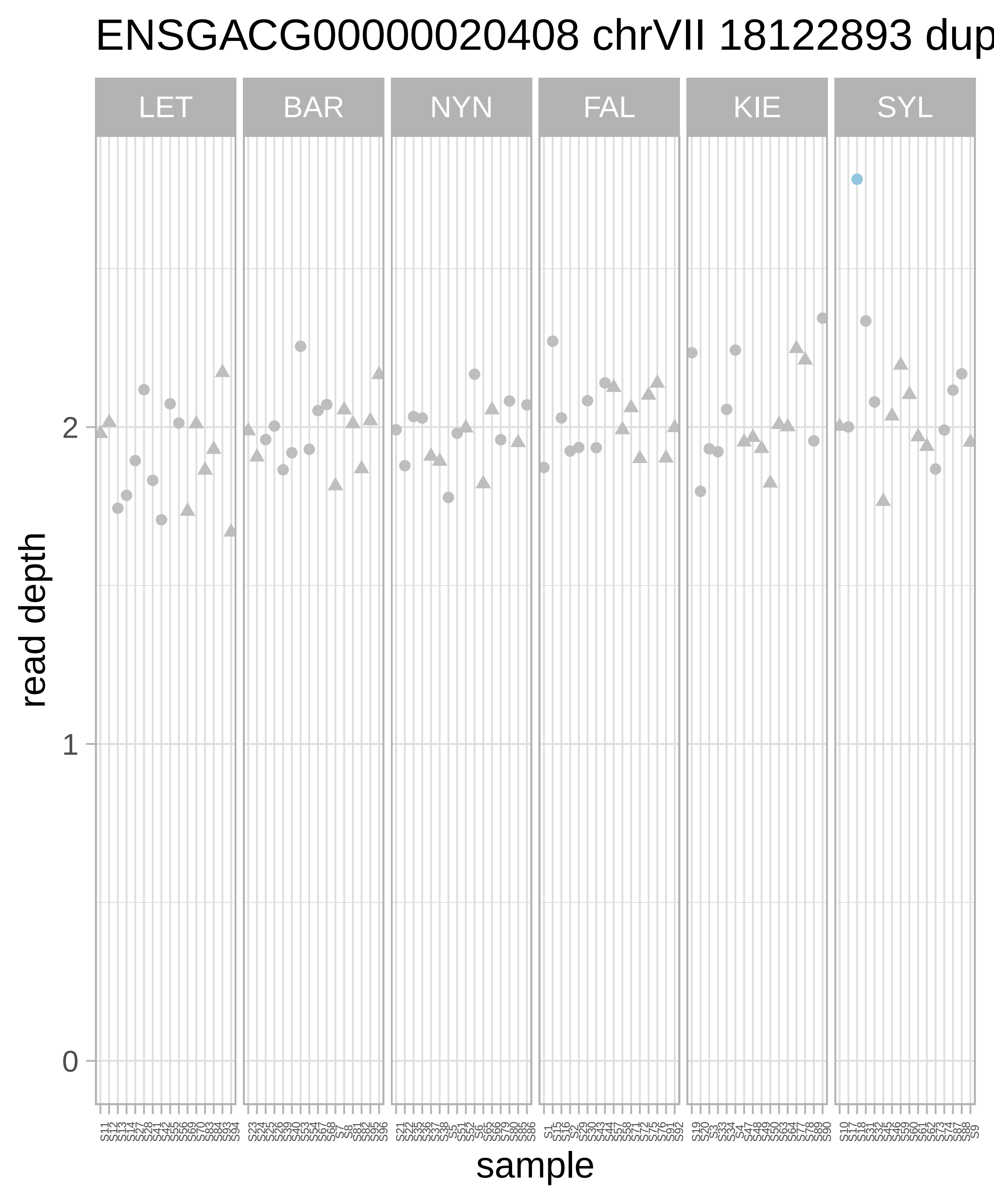
ENSGACG00000020408 chrVII 18122893 dup
LET
BAR
NYN
FAL
KIE
SYL
read depth
0
1
2
sample

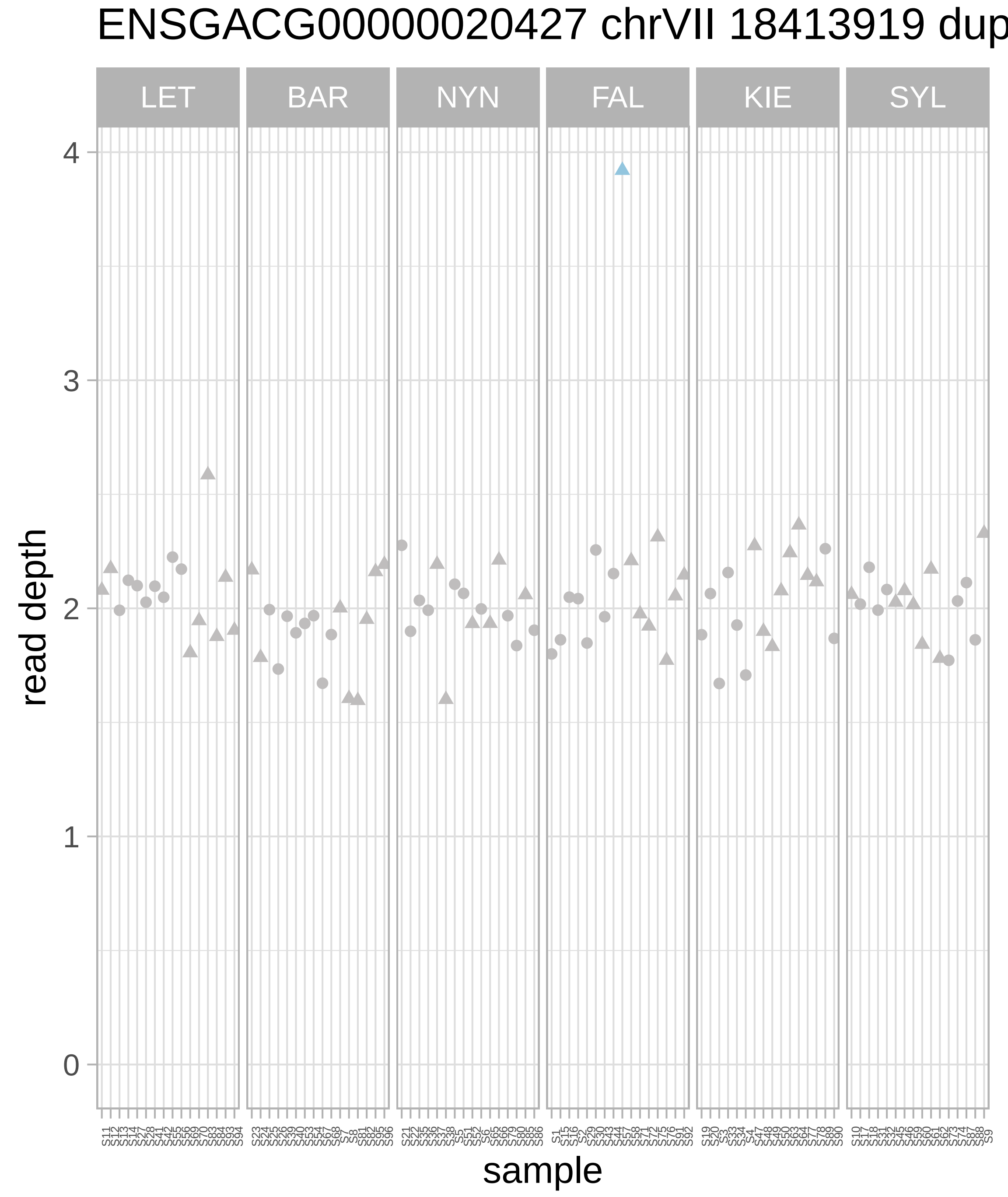
ENSGACG00000020427 chrVII 18413919 dup
LET
BAR
NYN
FAL
KIE
SYL
read depth
0
1
2
3
4
S11 S12 S13 S14 S27 S28 S41 S42 S55 S56 S69 S70 S83 S84 S93 S94
S23 S24 S25 S26 S39 S40 S53 S54 S67 S68 S7 S8 S81 S82 S95 S96
S21 S22 S35 S36 S37 S38 S5 S51 S52 S6 S65 S66 S79 S80 S85 S86
S1 S15 S16 S2 S29 S30 S43 S44 S57 S58 S71 S72 S75 S76 S91 S92
S19 S20 S3 S33 S34 S4 S47 S48 S49 S50 S63 S64 S77 S78 S89 S90
S10 S17 S18 S31 S32 S45 S46 S59 S60 S61 S62 S73 S74 S87 S88 S9
sample

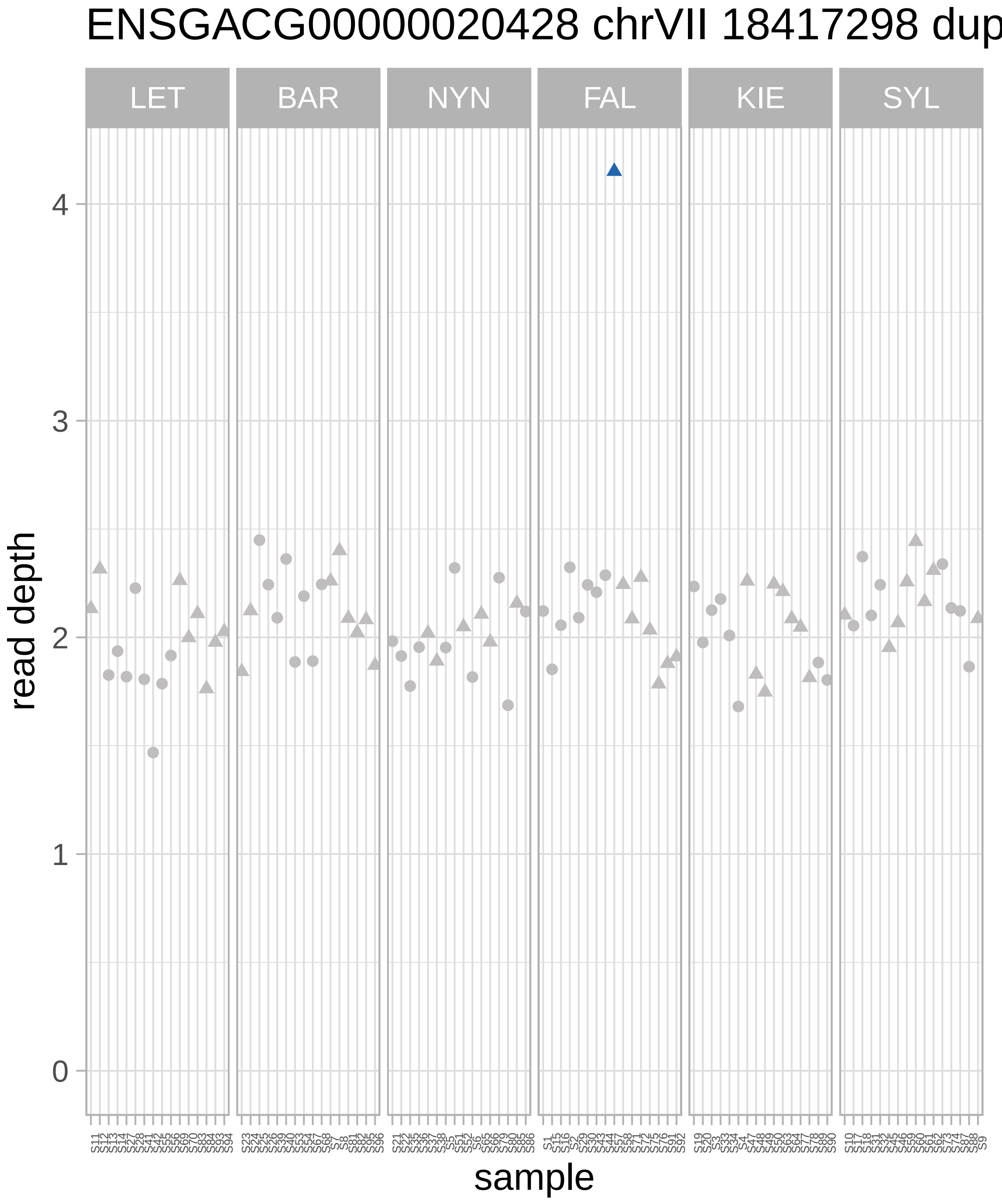
ENSGACG00000020428 chrVII 18417298 dup
LET
BAR
NYN
FAL
KIE
SYL
read depth
0
1
2
3
4
sample

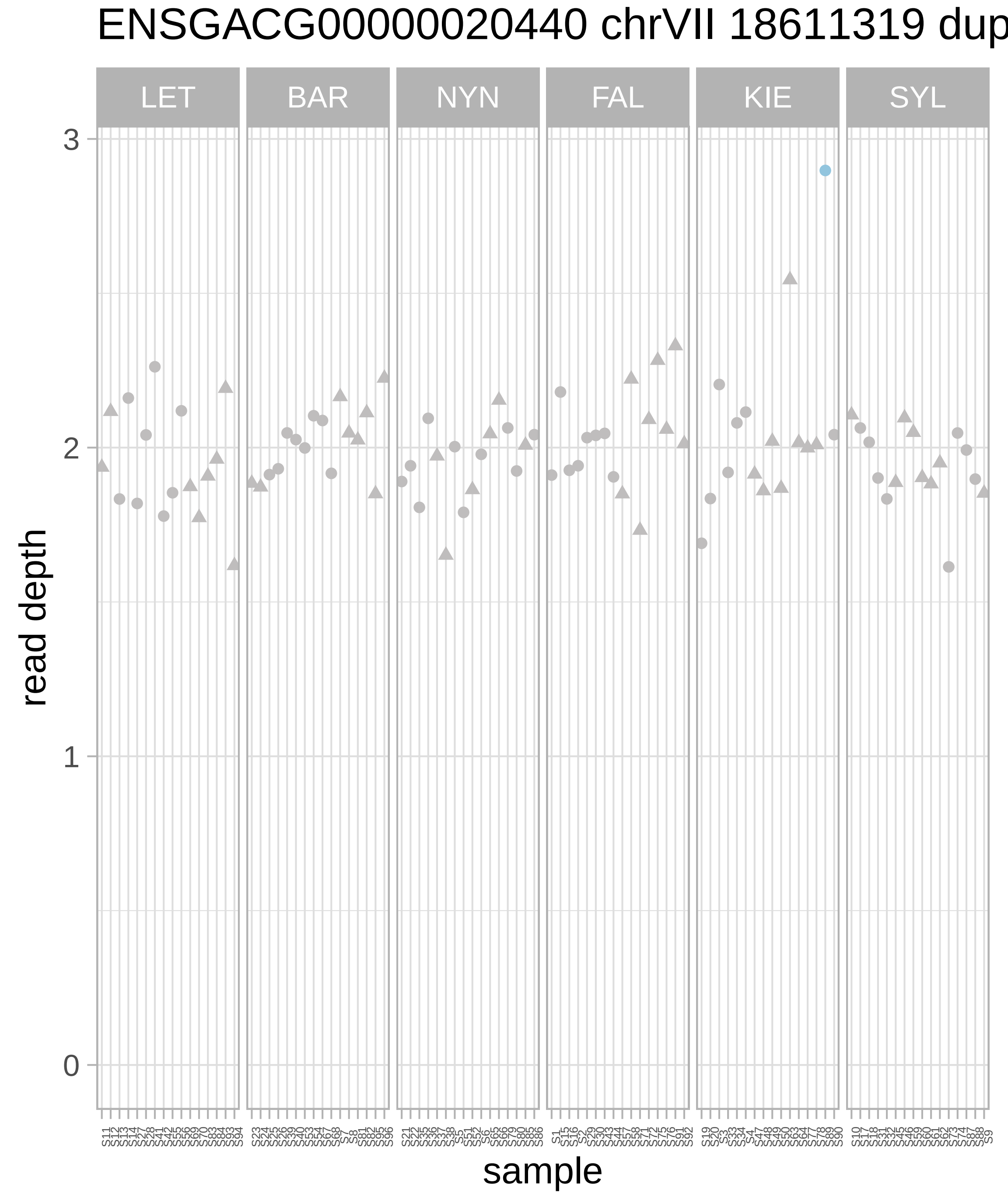

ENSGACG00000020440 chrVII 18611319 dup
LET
BAR
NYN
FAL
KIE
SYL
read depth
0
1
2
3
sample

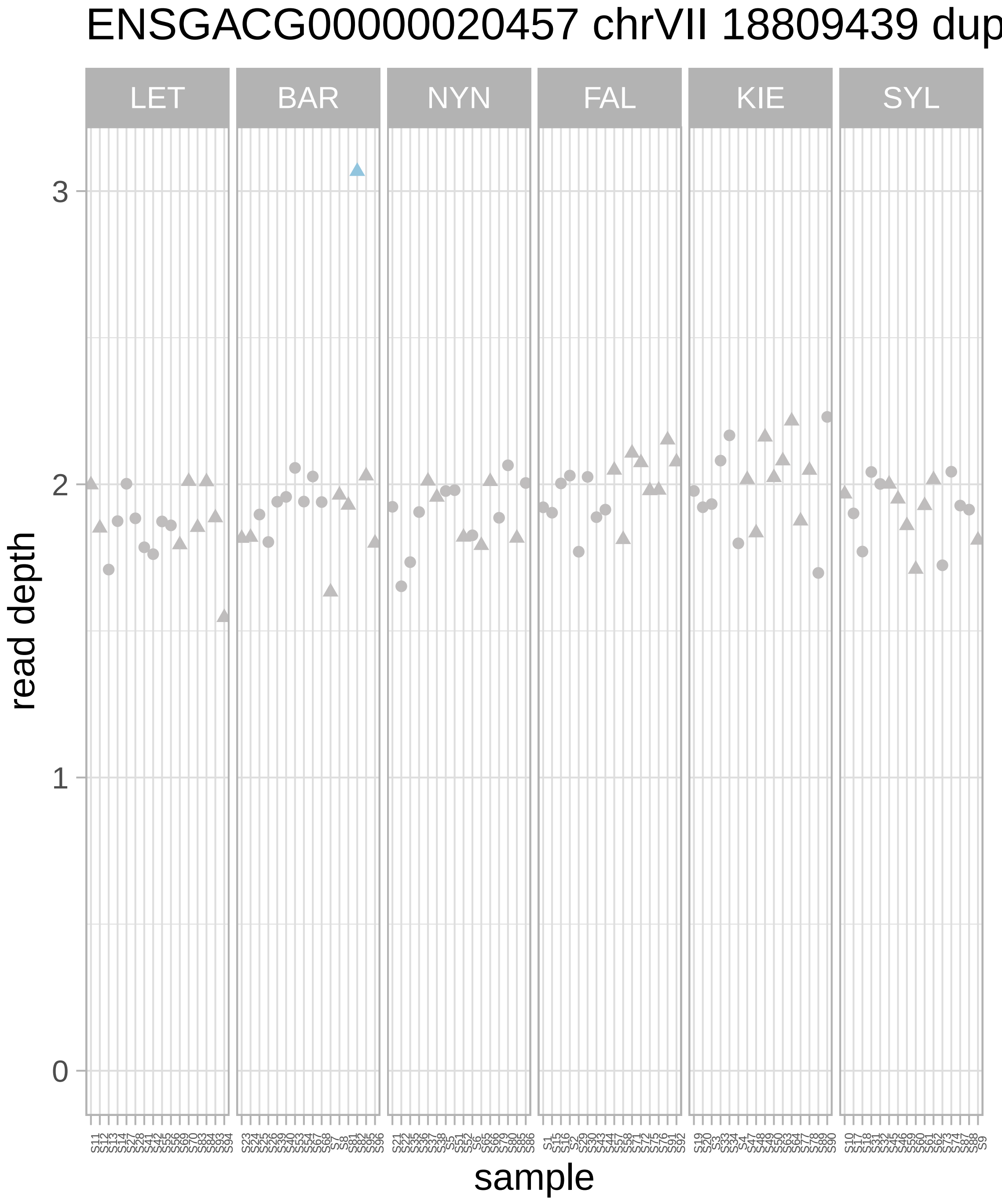
ENSGACG00000020457 chrVII 18809439 dup
LET
BAR
NYN
FAL
KIE
SYL
read depth
0
1
2
3
S11 S12 S13 S14 S27 S28 S41 S42 S55 S56 S69 S70 S83 S84 S93 S94
S23 S24 S25 S26 S39 S40 S53 S54 S67 S68 S7 S8 S81 S82 S95 S96
S21 S22 S35 S36 S37 S38 S5 S51 S52 S6 S65 S66 S79 S80 S85 S86
S1 S15 S16 S2 S29 S30 S43 S44 S57 S58 S71 S72 S75 S76 S91 S92
S19 S20 S3 S33 S34 S4 S47 S48 S49 S50 S63 S64 S77 S78 S89 S90
S10 S17 S18 S31 S32 S45 S46 S59 S60 S61 S62 S73 S74 S87 S88 S9
sample

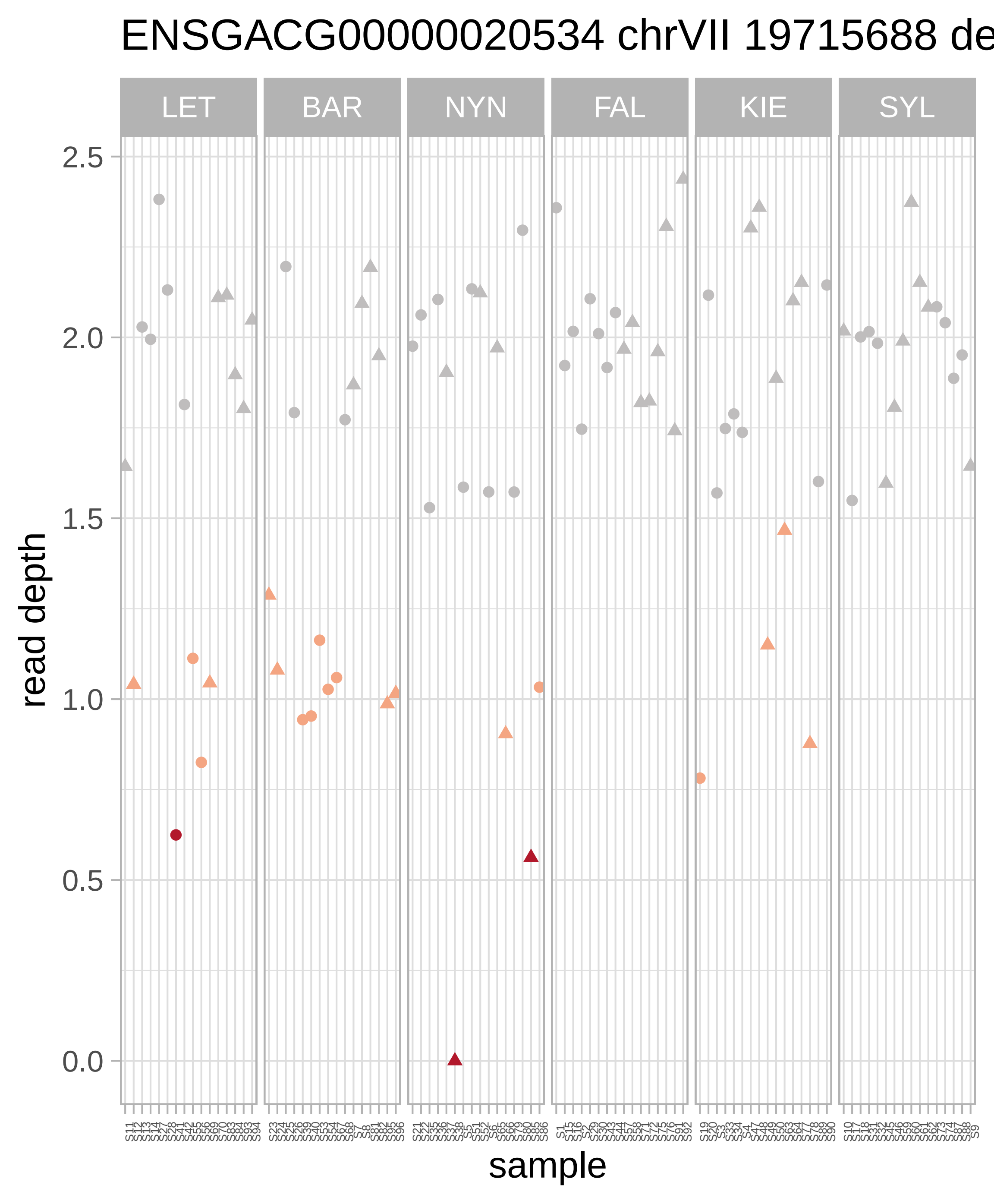
ENSGACG00000020534 chrVII 19715688 de
LET
BAR
NYN
FAL
KIE
SYL
read depth
2.5
2.0
1.5
1.0
0.5
0.0
sample

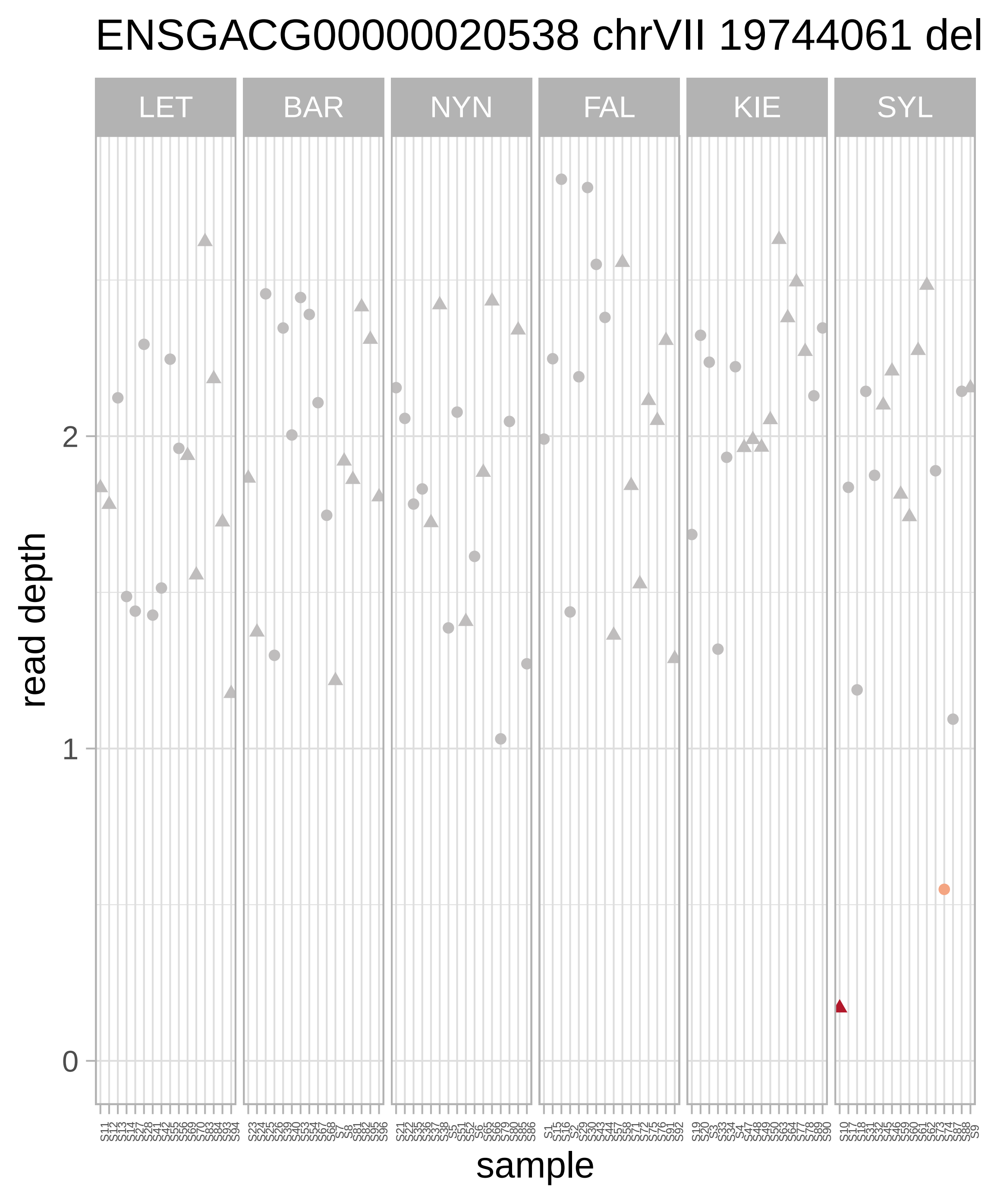
ENSGACG00000020538 chrVII 19744061 del
LET
BAR
NYN
FAL
KIE
SYL
read depth
0
1
2
sample

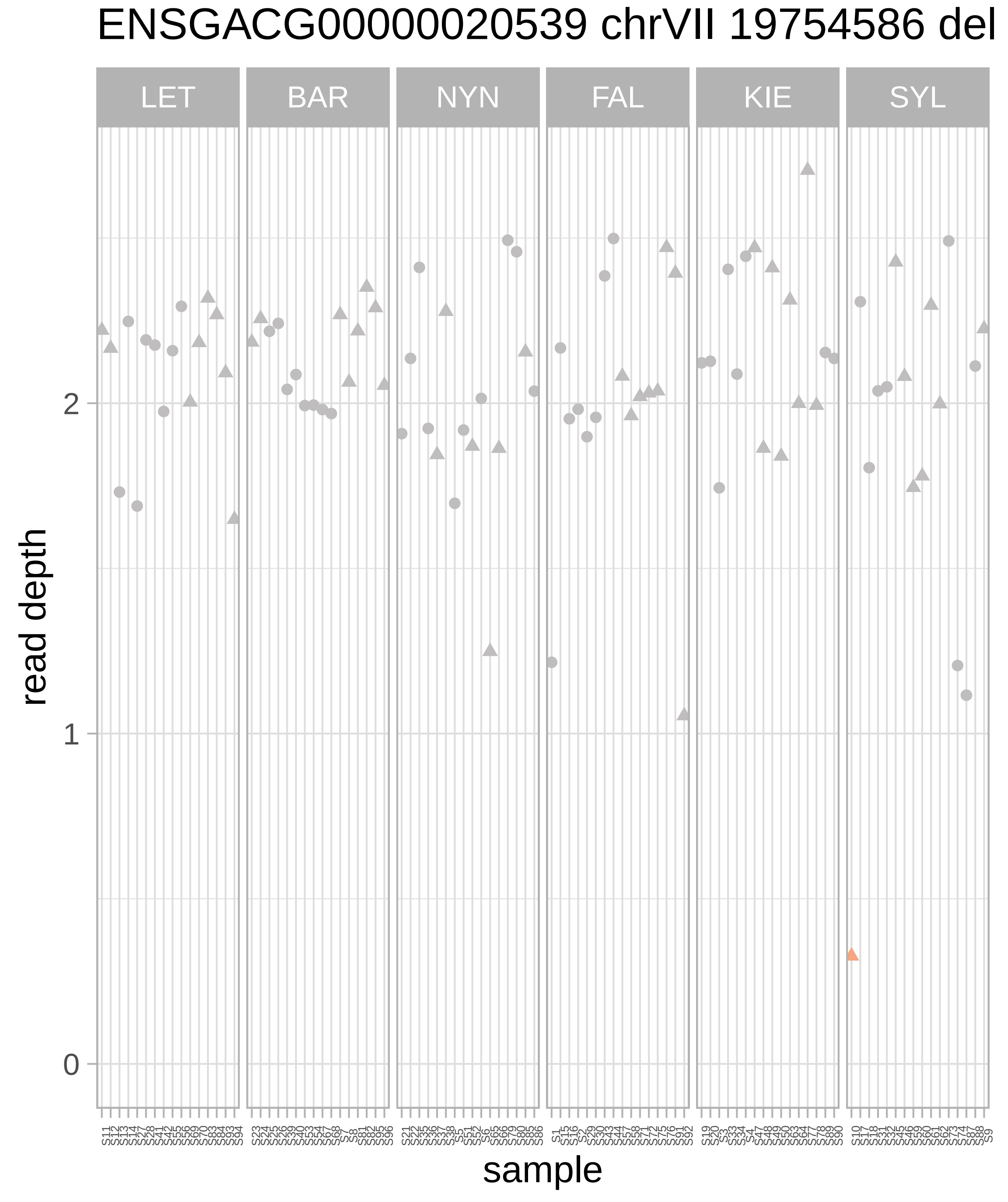
ENSGACG00000020539 chrVII 19754586 del
LET
BAR
NYN
FAL
KIE
SYL
read depth
0
1
2
sample
S11 S12 S13 S14 S27 S28 S41 S42 S55 S56 S69 S70 S83 S84 S93 S94
S23 S24 S25 S26 S39 S40 S53 S54 S67 S68 S7 S8 S81 S82 S95 S96
S21 S22 S35 S36 S37 S38 S5 S51 S52 S6 S65 S66 S79 S80 S85 S86
S1 S15 S16 S2 S29 S30 S43 S44 S57 S58 S71 S72 S75 S76 S91 S92
S19 S20 S3 S33 S34 S4 S47 S48 S49 S50 S63 S64 S77 S78 S89 S90
S10 S17 S18 S31 S32 S45 S46 S59 S60 S61 S62 S73 S74 S87 S88 S9

ENSGACG00000020749 chrVII 24008384 dup

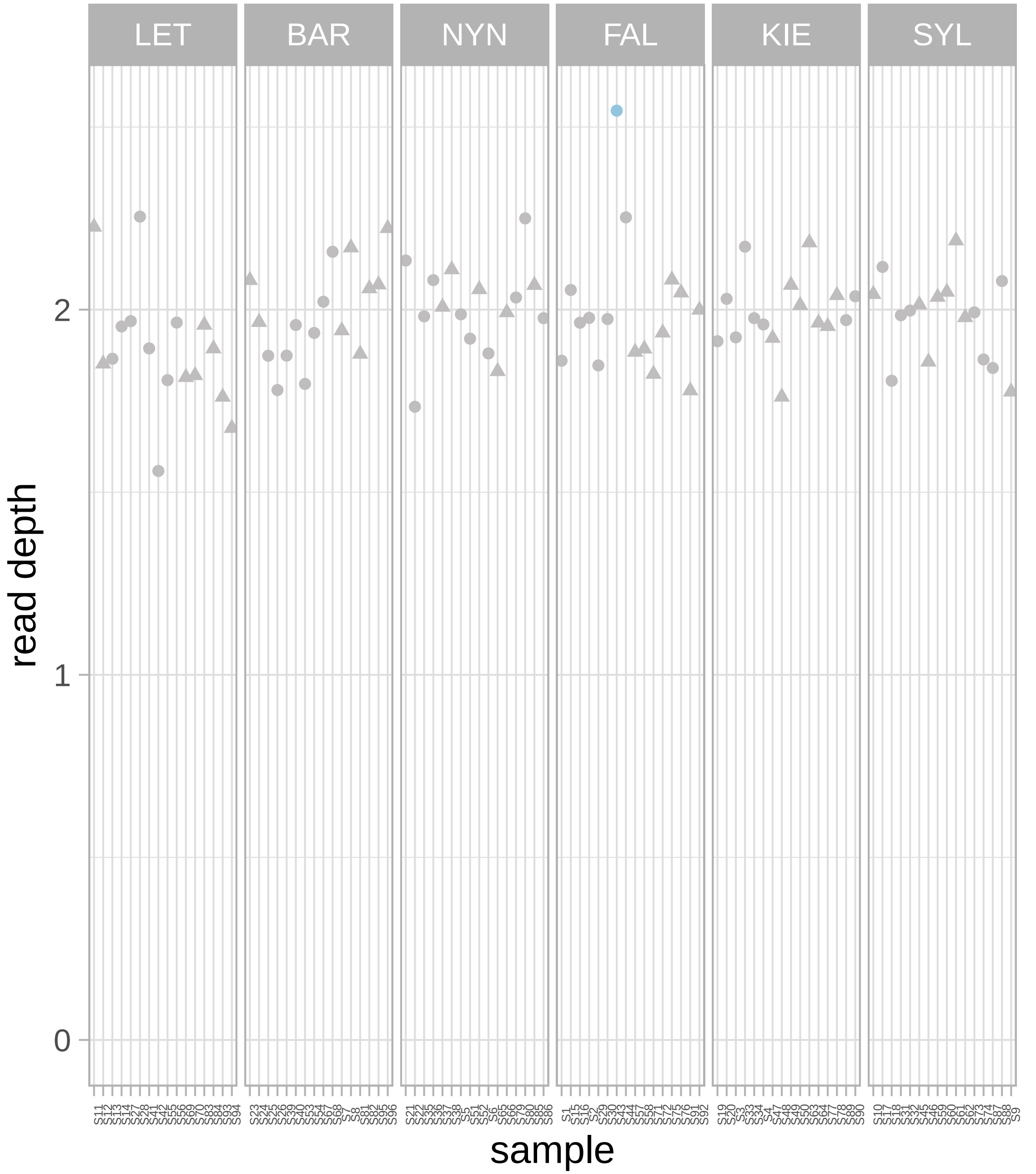

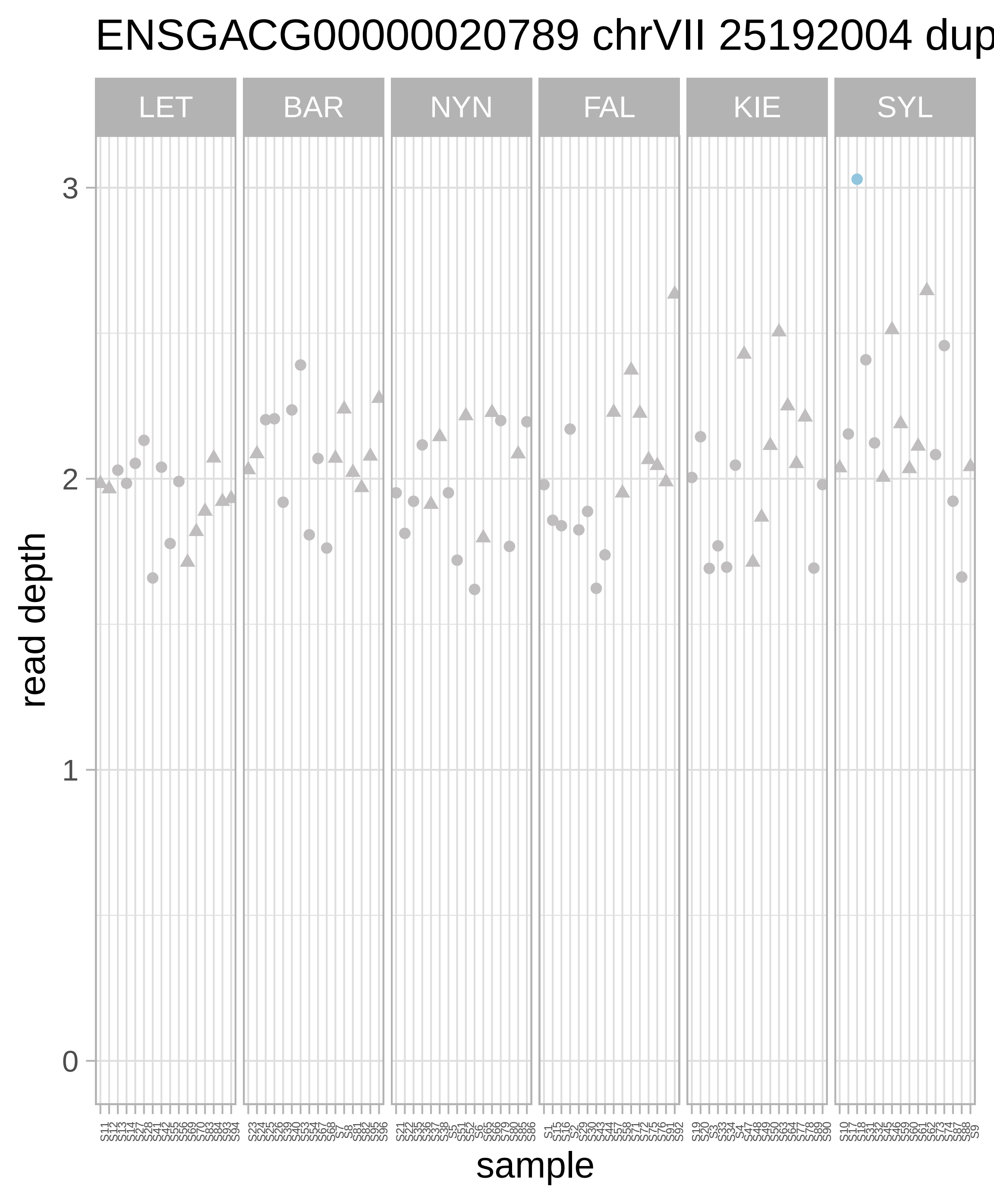
ENSGACG00000020789 chrVII 25192004 dup
LET
BAR
NYN
FAL
KIE
SYL
read depth
0
1
2
3
sample
S11 S12 S13 S14 S27 S28 S41 S42 S55 S56 S69 S70 S83 S84 S93 S94
S23 S24 S25 S26 S39 S40 S53 S54 S67 S68 S7 S8 S81 S82 S95 S96
S21 S22 S35 S36 S37 S38 S5 S51 S52 S6 S65 S66 S79 S80 S85 S86
S1 S15 S16 S2 S29 S30 S43 S44 S57 S58 S71 S72 S75 S76 S91 S92
S19 S20 S3 S33 S34 S4 S47 S48 S49 S50 S63 S64 S77 S78 S89 S90
S10 S17 S18 S31 S32 S45 S46 S59 S60 S61 S62 S73 S74 S87 S88 S9

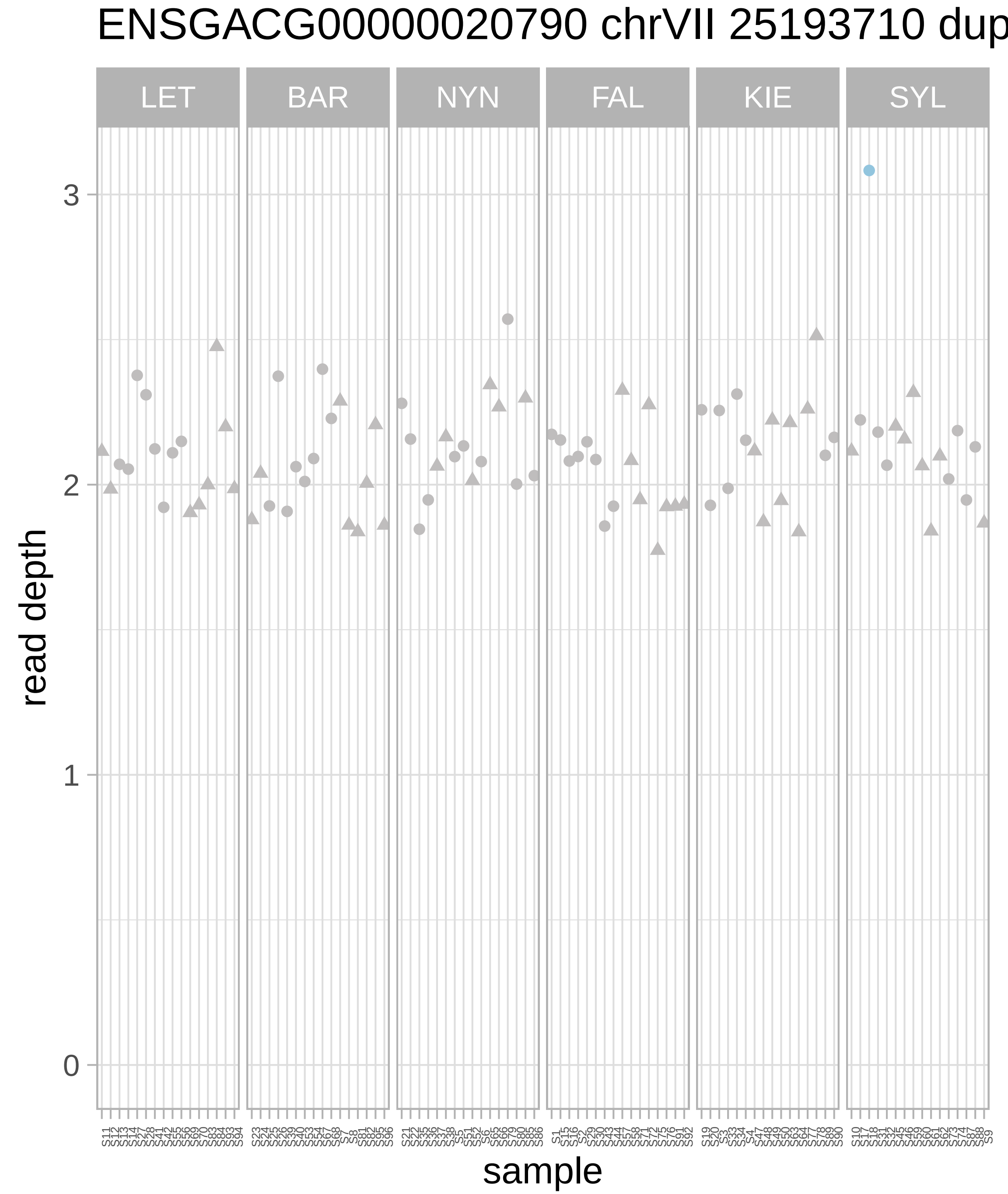
ENSGACG00000020790 chrVII 25193710 dup
LET
BAR
NYN
FAL
KIE
SYL
read depth
0
1
2
3
S11 S12 S13 S14 S27 S28 S41 S42 S55 S56 S69 S70 S83 S84 S93 S94
S23 S24 S25 S26 S39 S40 S53 S54 S67 S68 S7 S8 S81 S82 S95 S96
S21 S22 S35 S36 S37 S38 S5 S51 S52 S6 S65 S66 S79 S80 S85 S86
S1 S15 S16 S2 S29 S30 S43 S44 S57 S58 S71 S72 S75 S76 S91 S92
S19 S20 S3 S33 S34 S4 S47 S48 S49 S50 S63 S64 S77 S78 S89 S90
S10 S17 S18 S31 S32 S45 S46 S59 S60 S61 S62 S73 S74 S87 S88 S9
sample

ENSGACG00000002934 chrVIII 196884 dup

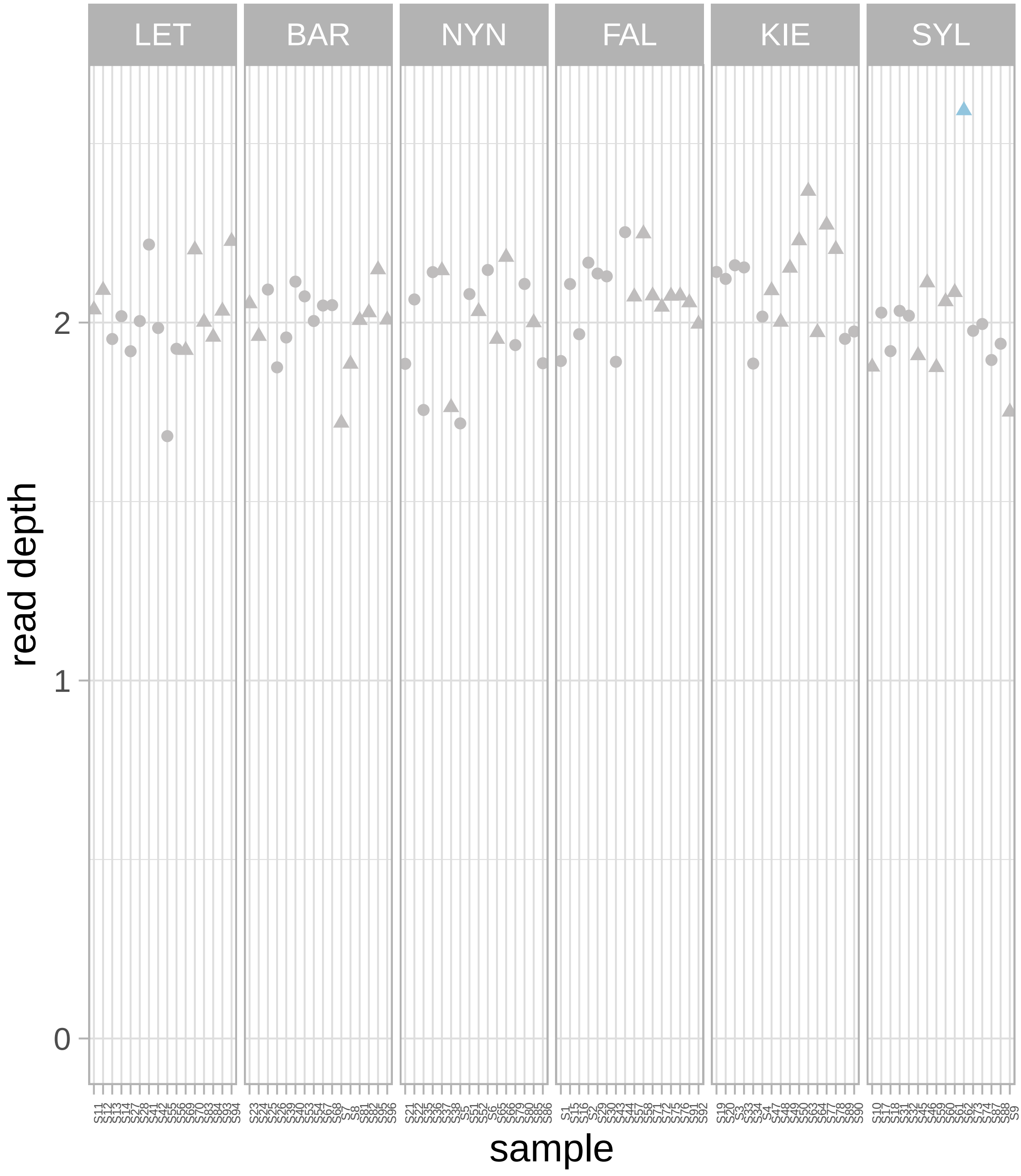

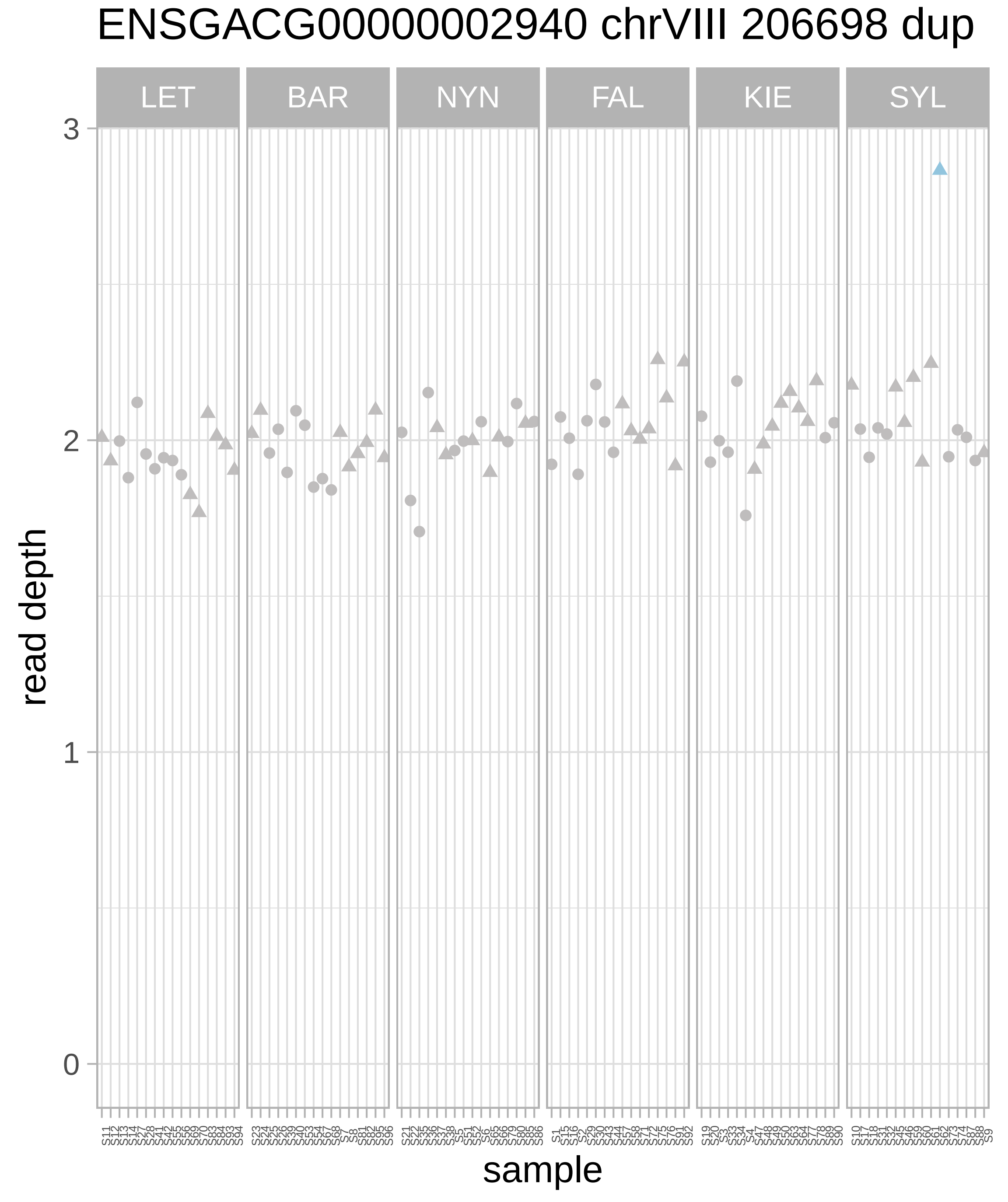
ENSGACG00000002940 chrVIII 206698 dup
LET
BAR
NYN
FAL
KIE
SYL
read depth
0
1
2
3
sample

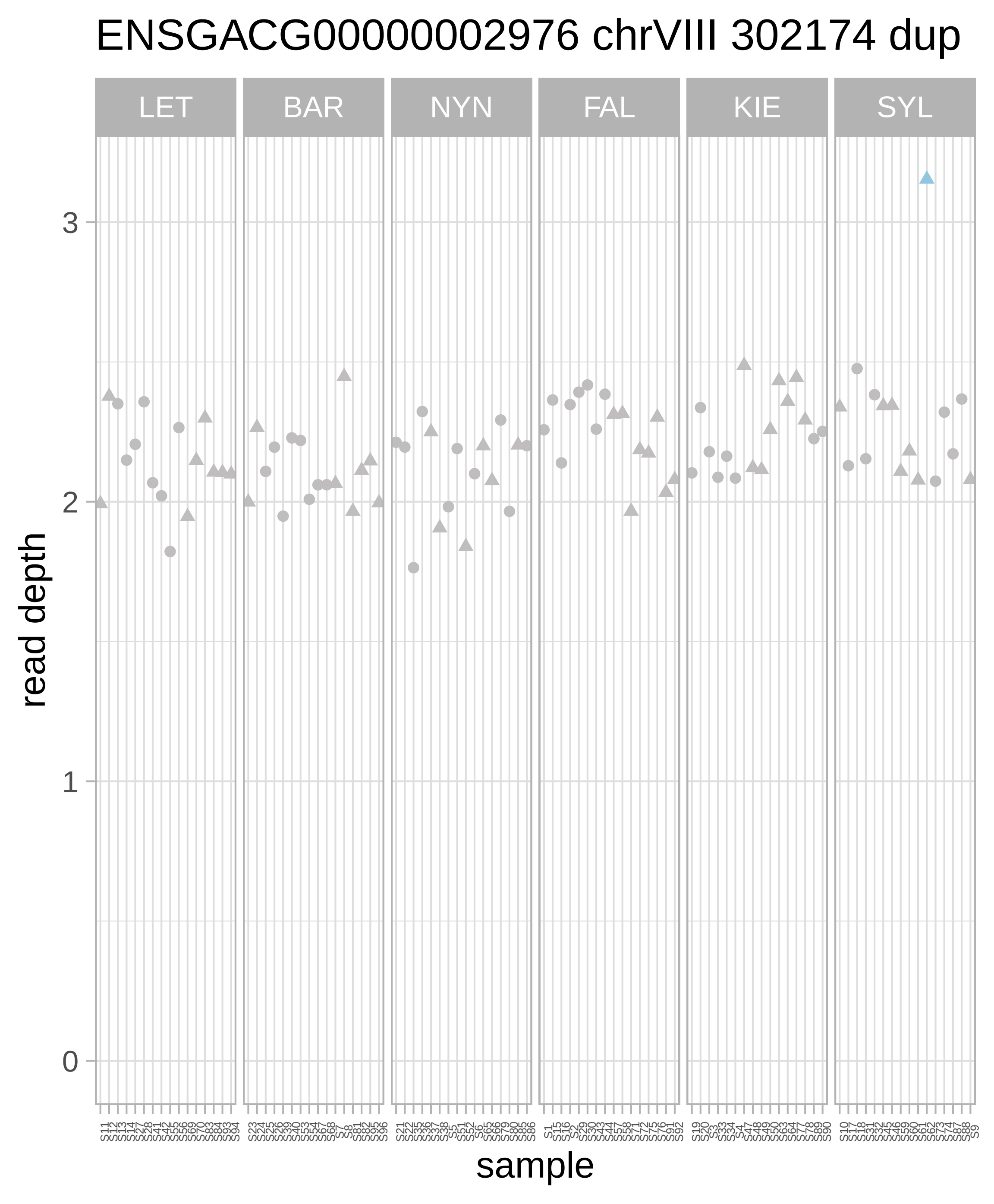

ENSGACG00000002976 chrVIII 302174 dup
LET
BAR
NYN
FAL
KIE
SYL
read depth
0
1
2
3
sample

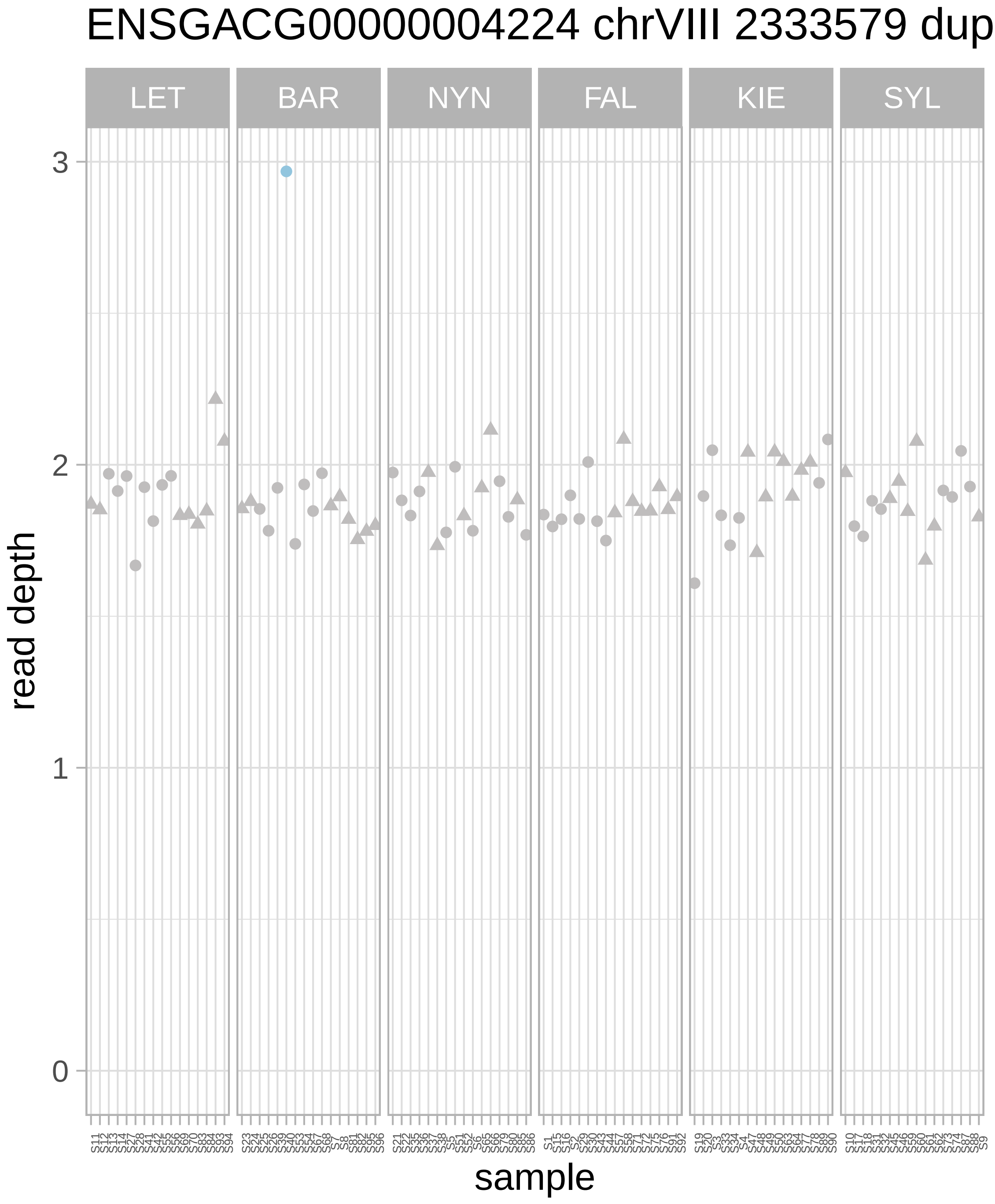
ENSGACG00000004224 chrVIII 2333579 dup
LET
BAR
NYN
FAL
KIE
SYL
read depth
3
2
1
0
sample

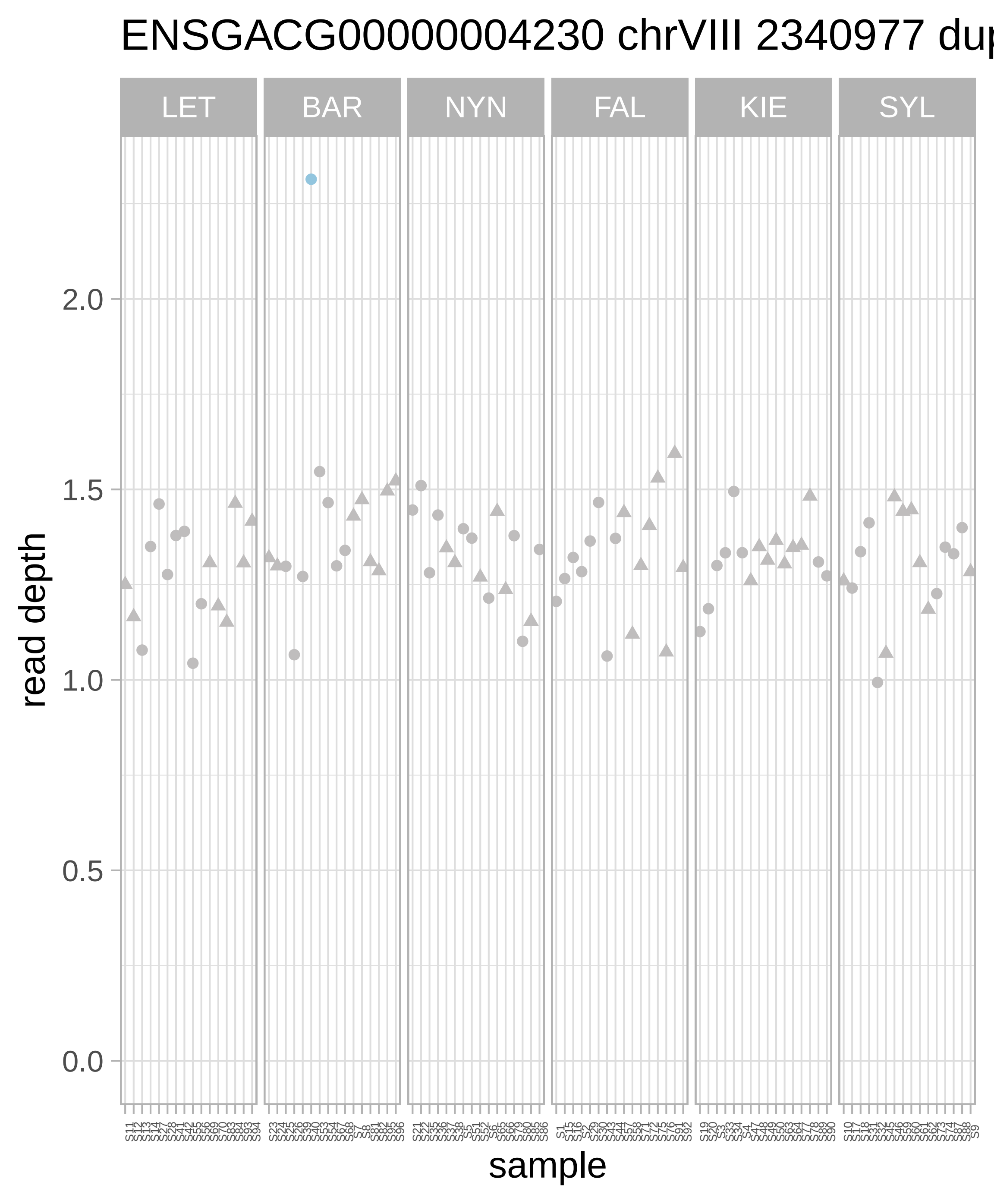
ENSGACG00000004230 chrVIII 2340977 dup
LET
BAR
NYN
FAL
KIE
SYL
read depth
2.0
1.5
1.0
0.5
0.0
sample

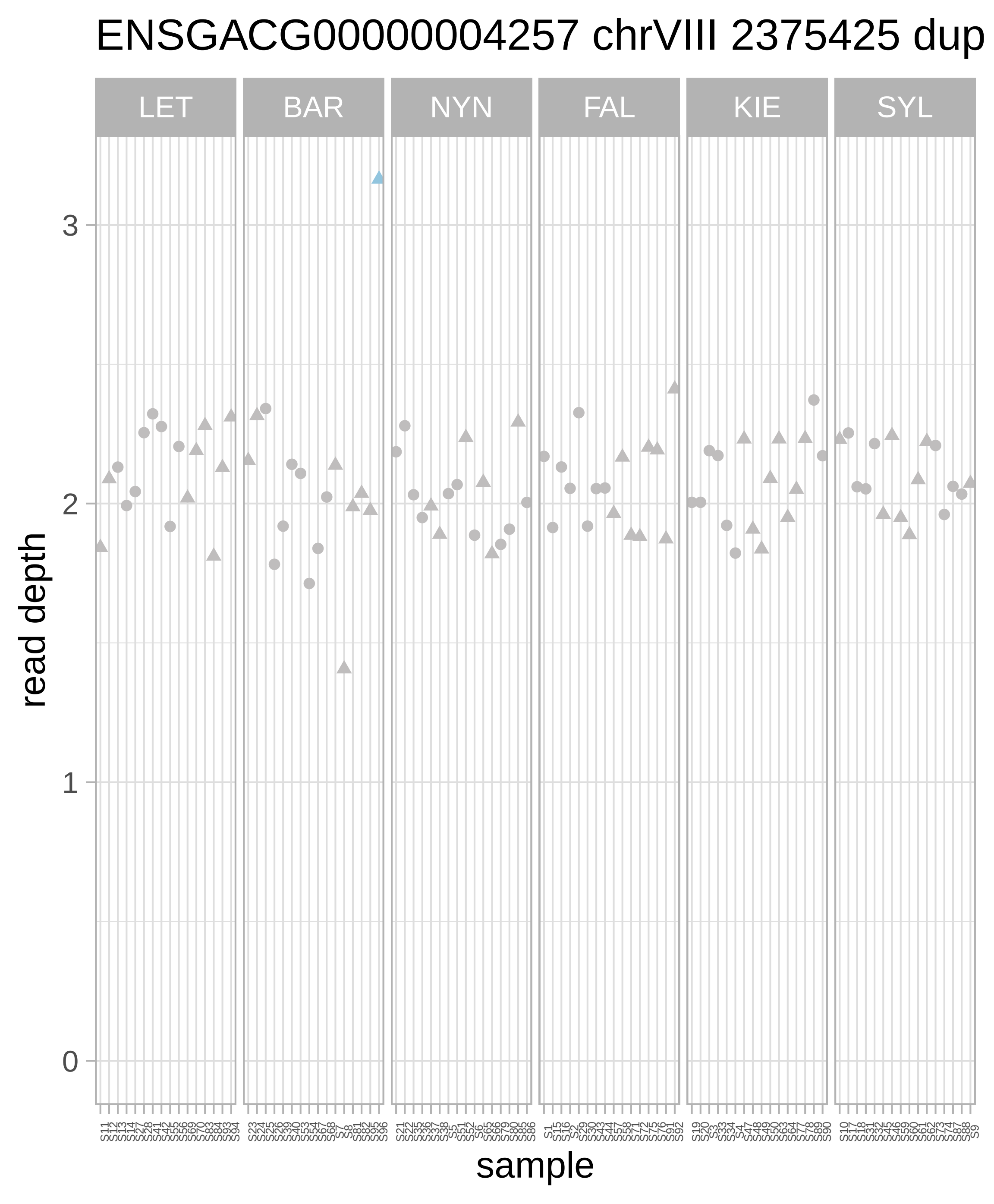
ENSGACG00000004257 chrVIII 2375425 dup
LET
BAR
NYN
FAL
KIE
SYL
read depth
sample
0
1
2
3

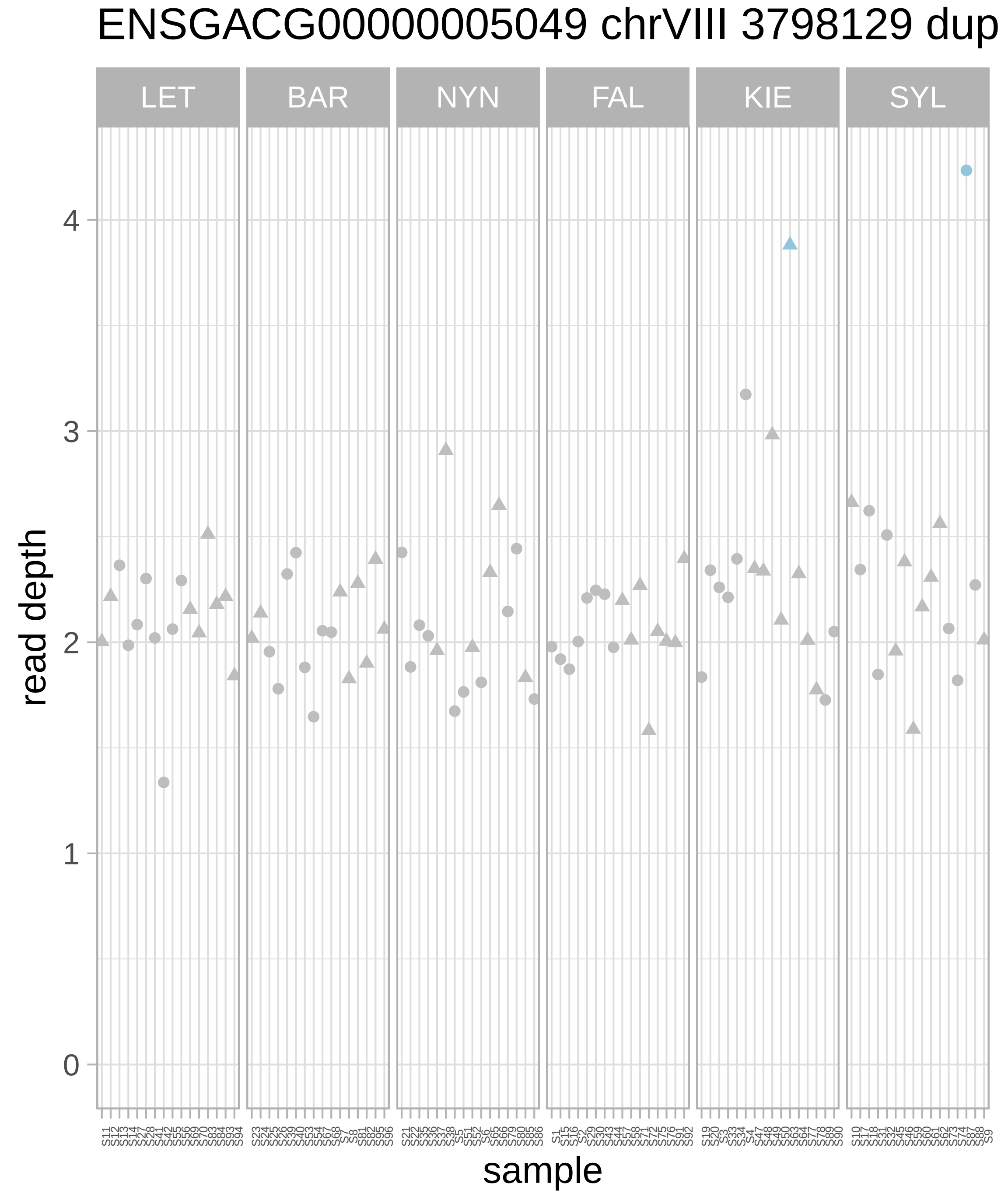
ENSGACG00000005049 chrVIII 3798129 dup
LET
BAR
NYN
FAL
KIE
SYL
read depth
0
1
2
3
4
sample

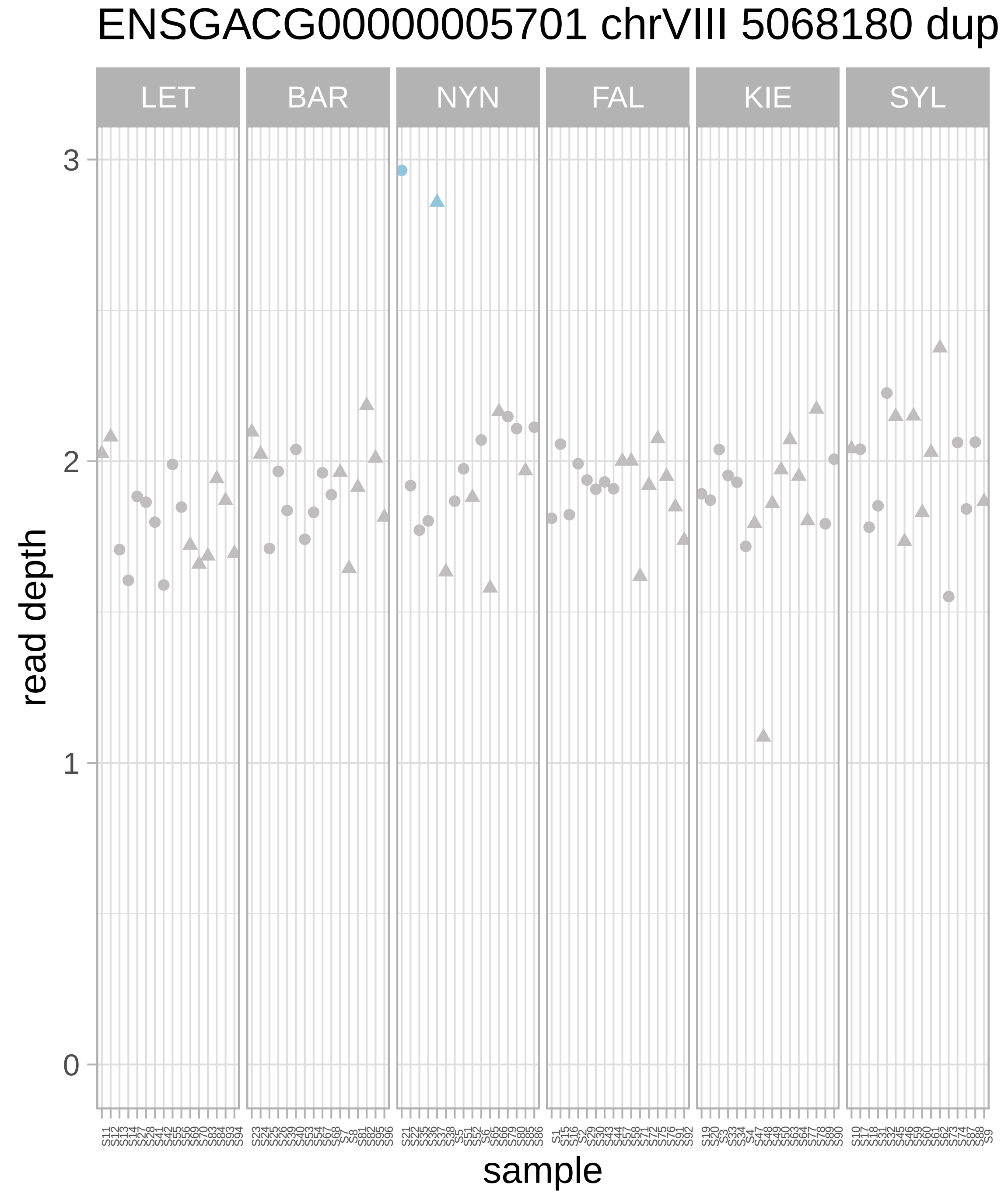
ENSGACG00000005701 chrVIII 5068180 dup
LET
BAR
NYN
FAL
KIE
SYL
3
2
1
0
read depth
S11 S12 S13 S14 S27 S28 S41 S42 S55 S56 S69 S70 S83 S84 S93 S94
S23 S24 S25 S26 S39 S40 S53 S54 S67 S68 S7 S8 S81 S82 S95 S96
S21 S22 S35 S36 S37 S38 S5 S51 S52 S6 S65 S66 S79 S80 S85 S86
S1 S15 S16 S2 S29 S30 S43 S44 S57 S58 S71 S72 S75 S76 S91 S92
S19 S20 S3 S33 S34 S4 S47 S48 S49 S50 S63 S64 S77 S78 S89 S90
S10 S17 S18 S31 S32 S45 S46 S59 S60 S61 S62 S73 S74 S87 S88 S9
sample

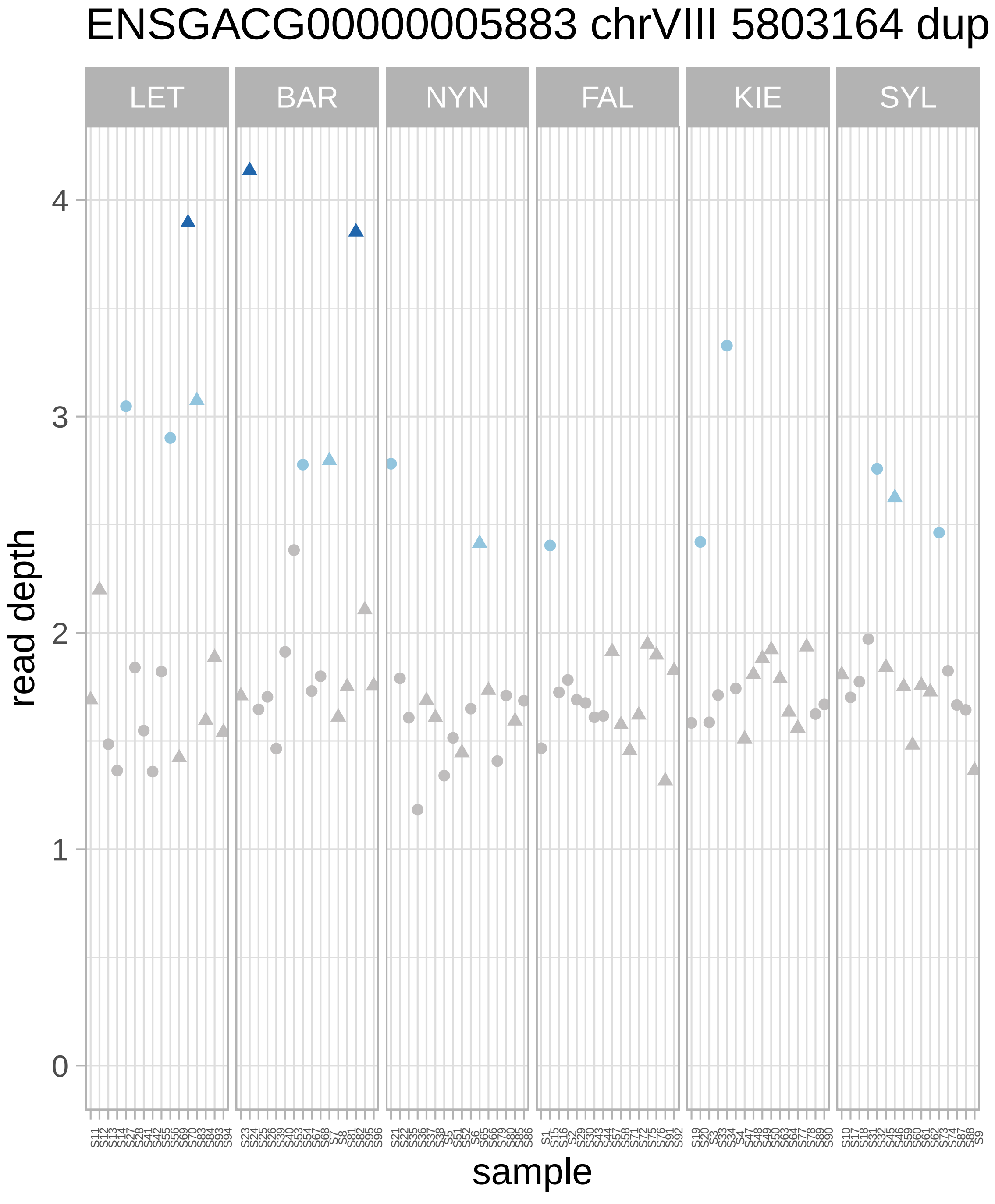
ENSGACG00000005883 chrVIII 5803164 dup
LET
BAR
NYN
FAL
KIE
SYL
read depth
0
1
2
3
4
sample

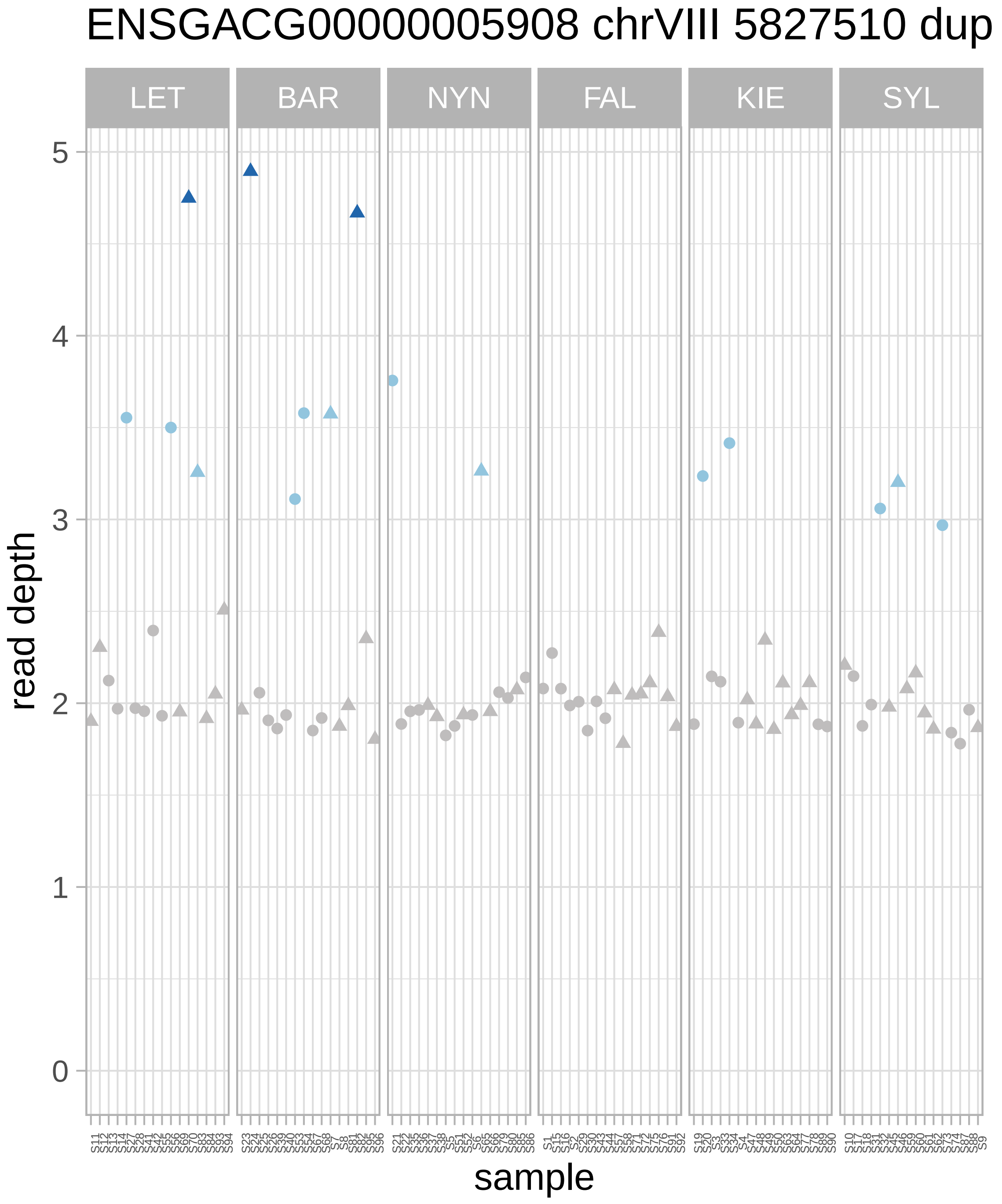
ENSGACG00000005908 chrVIII 5827510 dup
LET
BAR
NYN
FAL
KIE
SYL
read depth
0
1
2
3
4
5
S11 S12 S13 S14 S27 S28 S41 S42 S55 S56 S69 S70 S83 S84 S93 S94
S23 S24 S25 S26 S39 S40 S53 S54 S67 S68 S7 S8 S81 S82 S95 S96
S21 S22 S35 S36 S37 S38 S5 S51 S52 S6 S65 S66 S79 S80 S85 S86
S1 S15 S16 S2 S29 S30 S43 S44 S57 S58 S71 S72 S75 S76 S91 S92
S19 S20 S3 S33 S34 S4 S47 S48 S49 S50 S63 S64 S77 S78 S89 S90
S10 S17 S18 S31 S32 S45 S46 S59 S60 S61 S62 S73 S74 S87 S88 S9
sample

ENSGACG00000005911 chrVIII 5829401 dup

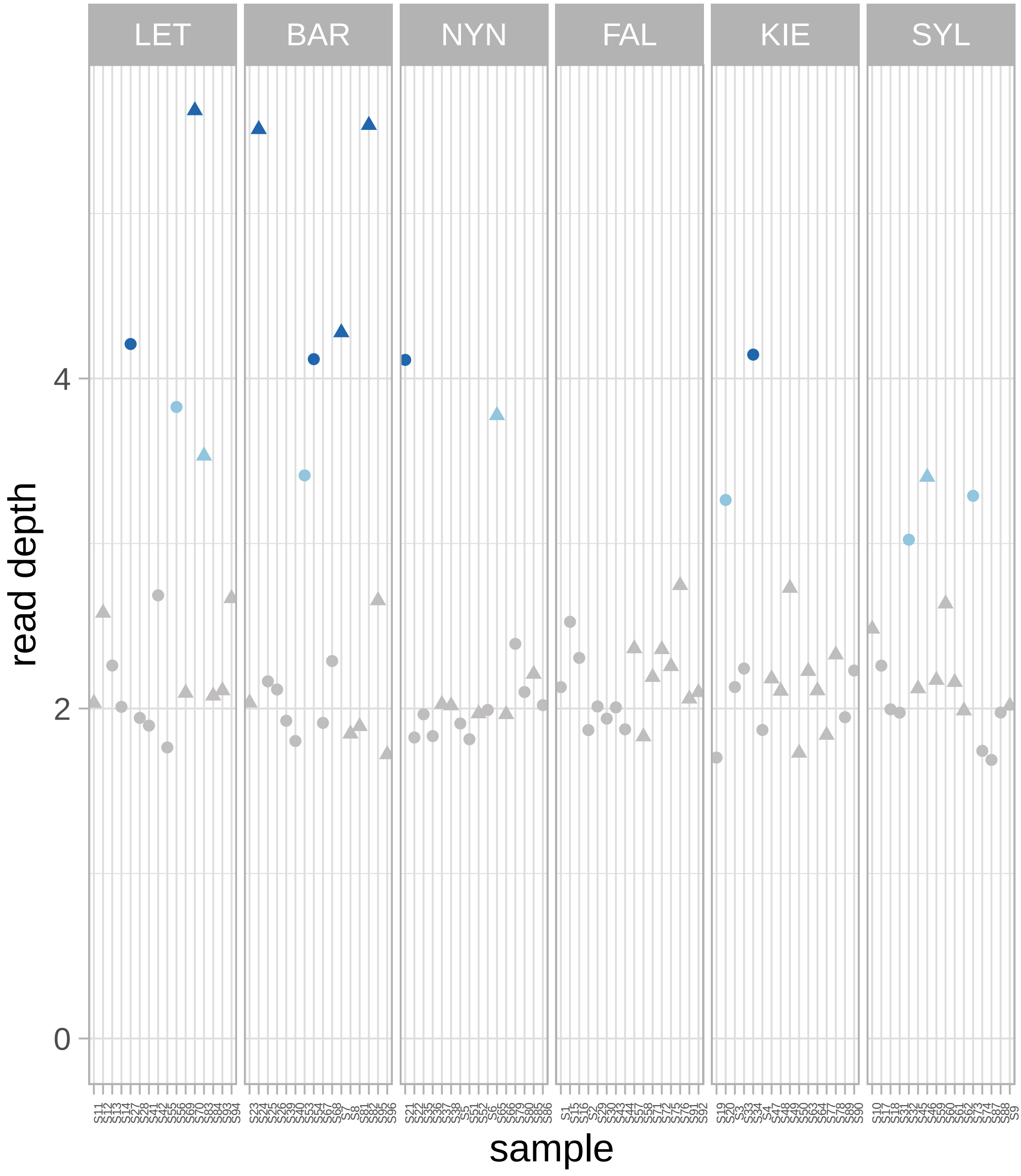

ENSGACG00000006285 chrVIII 6647308 dup

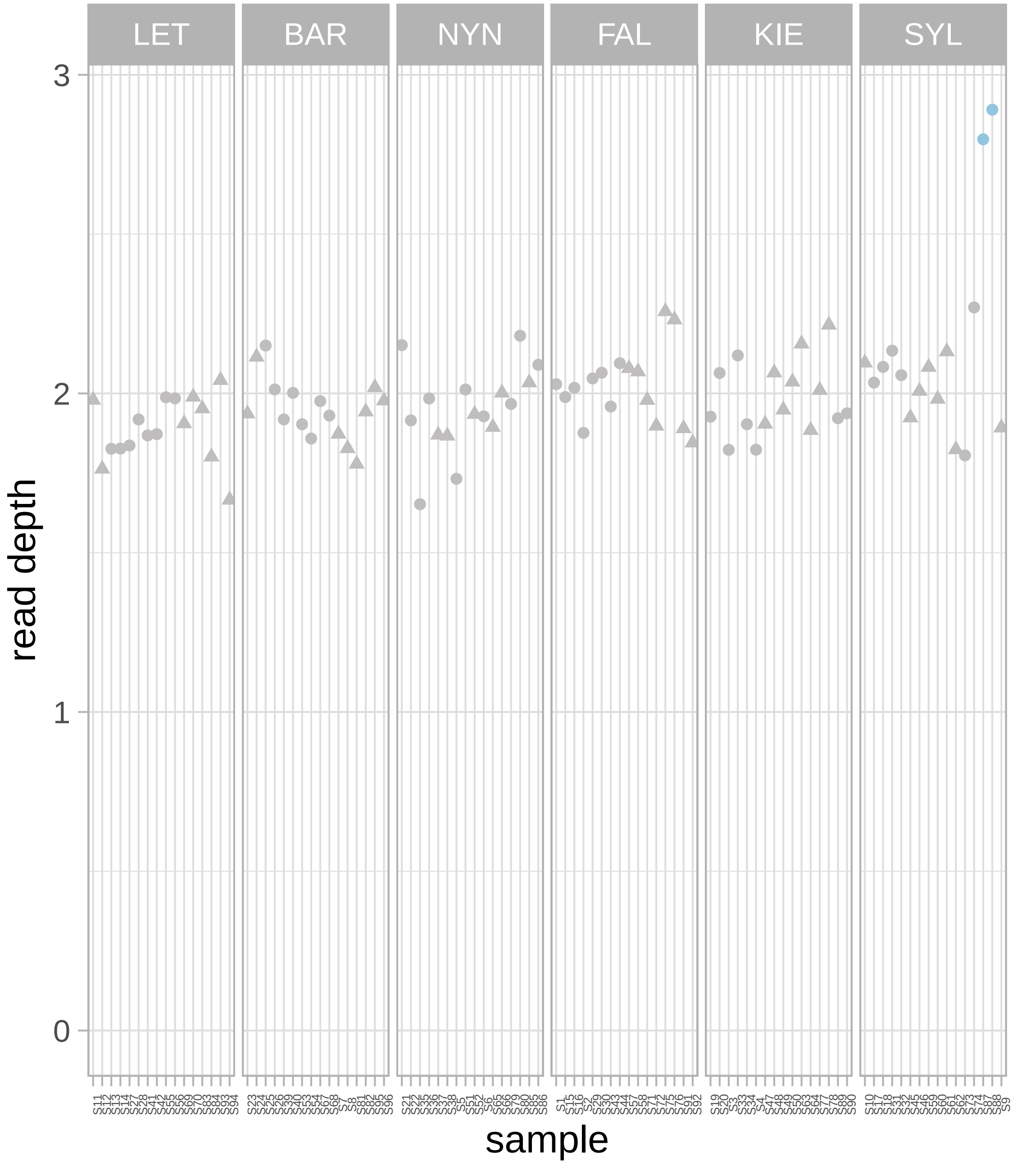

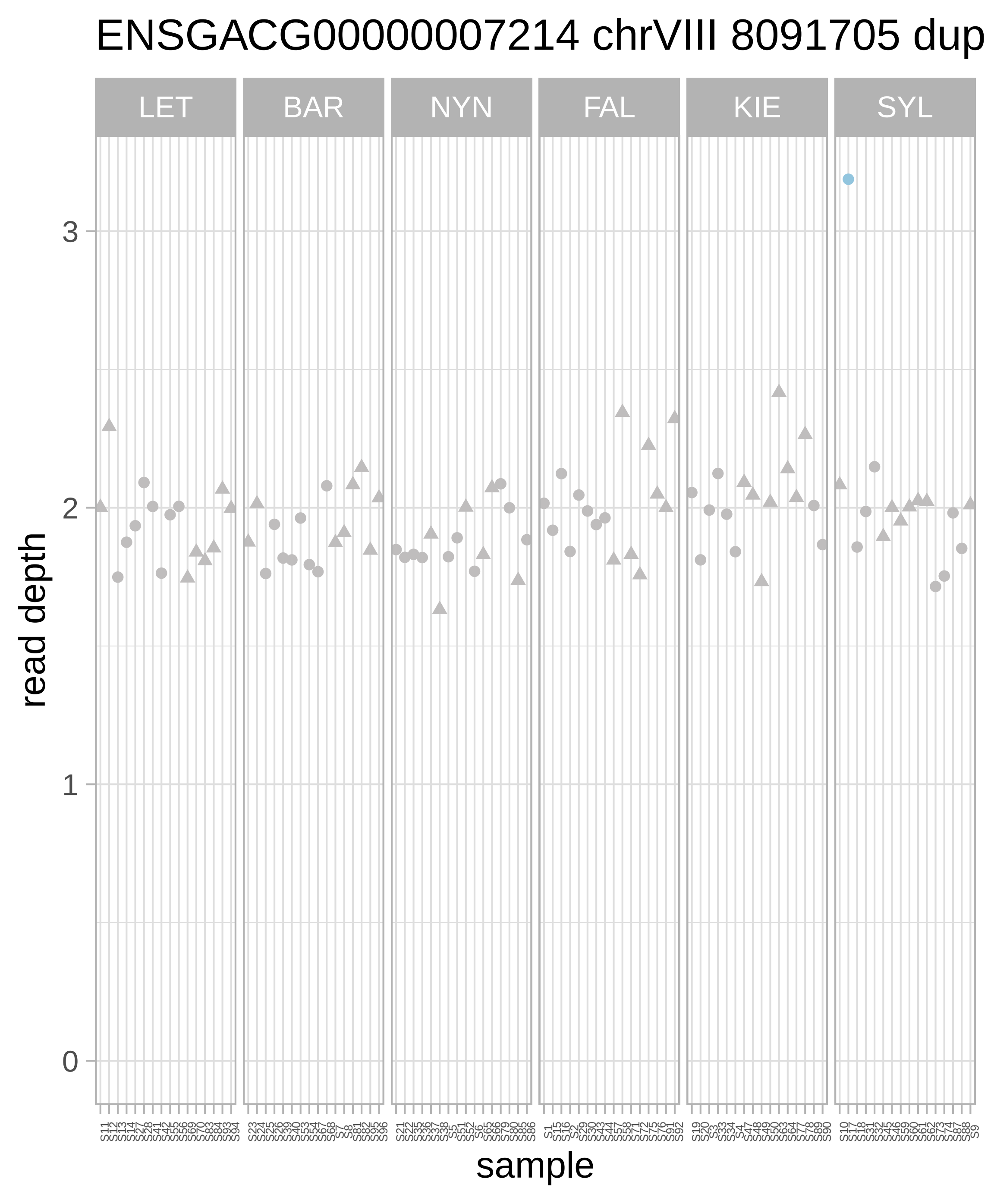
ENSGACG00000007214 chrVIII 8091705 dup
LET
BAR
NYN
FAL
KIE
SYL
read depth
0
1
2
3
sample

ENSGACG00000007240 chrVIII 8096513 dup

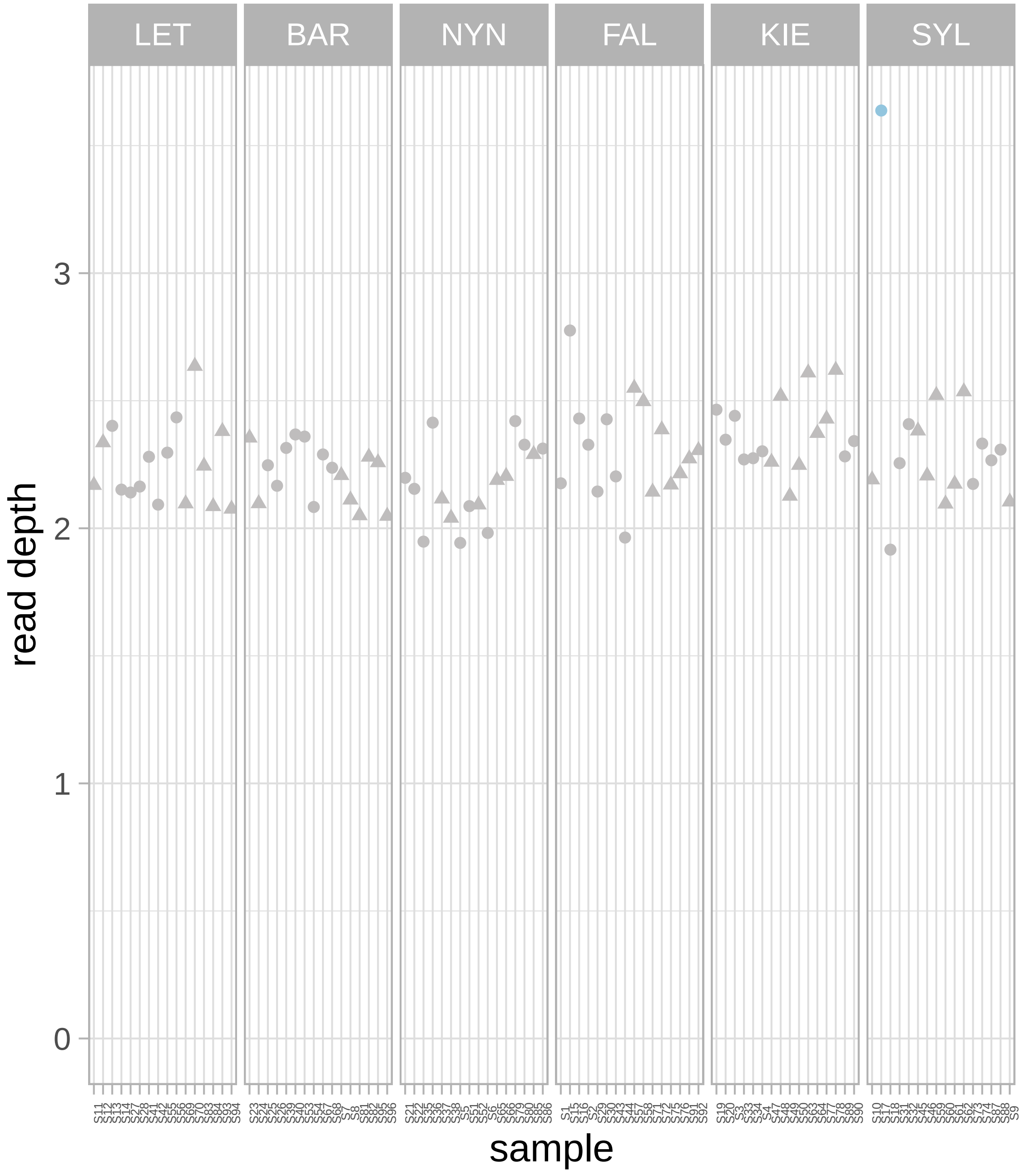

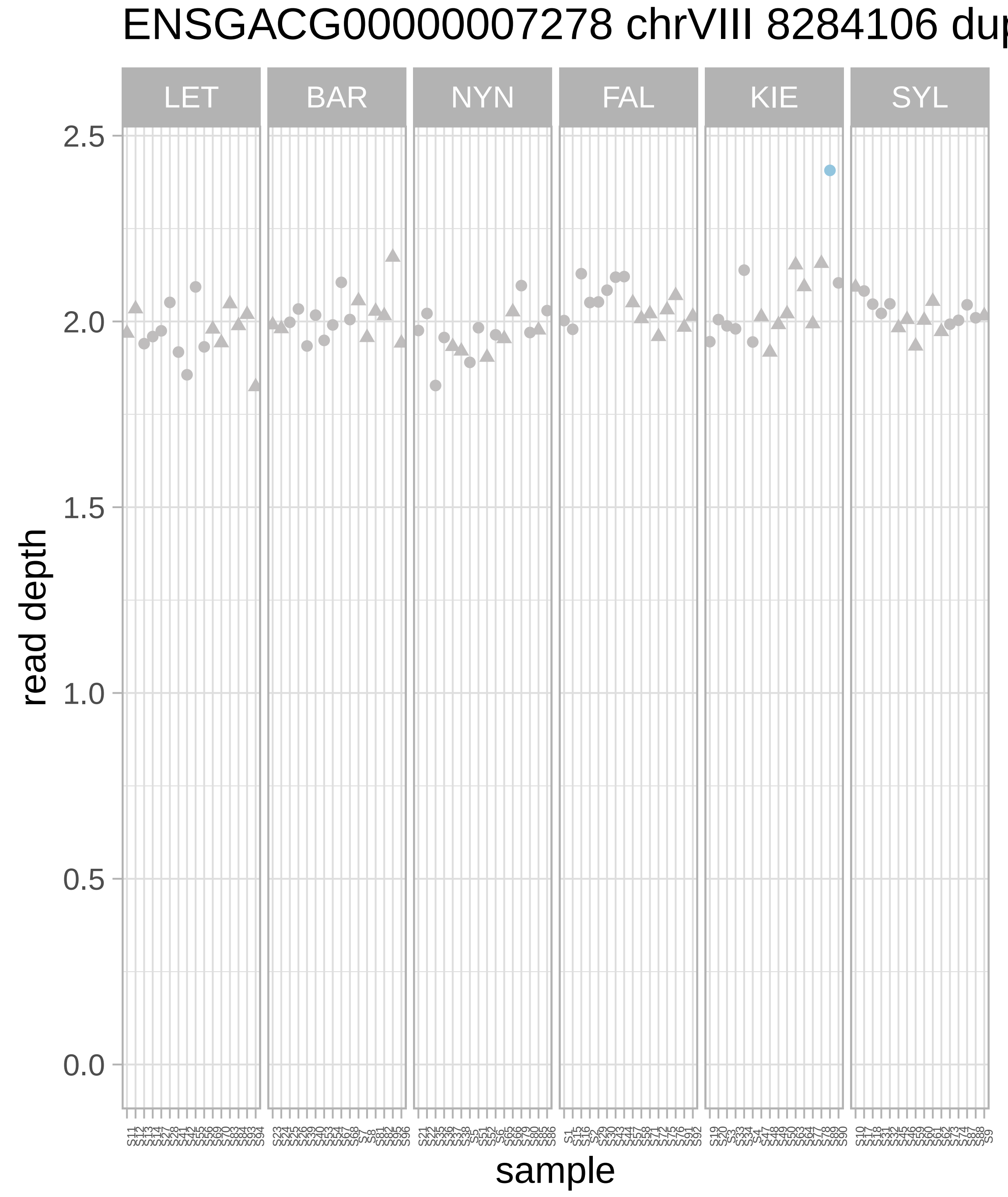
ENSGACG00000007278 chrVIII 8284106 dup
LET
BAR
NYN
FAL
KIE
SYL
read depth
2.5
2.0
1.5
1.0
0.5
0.0
sample

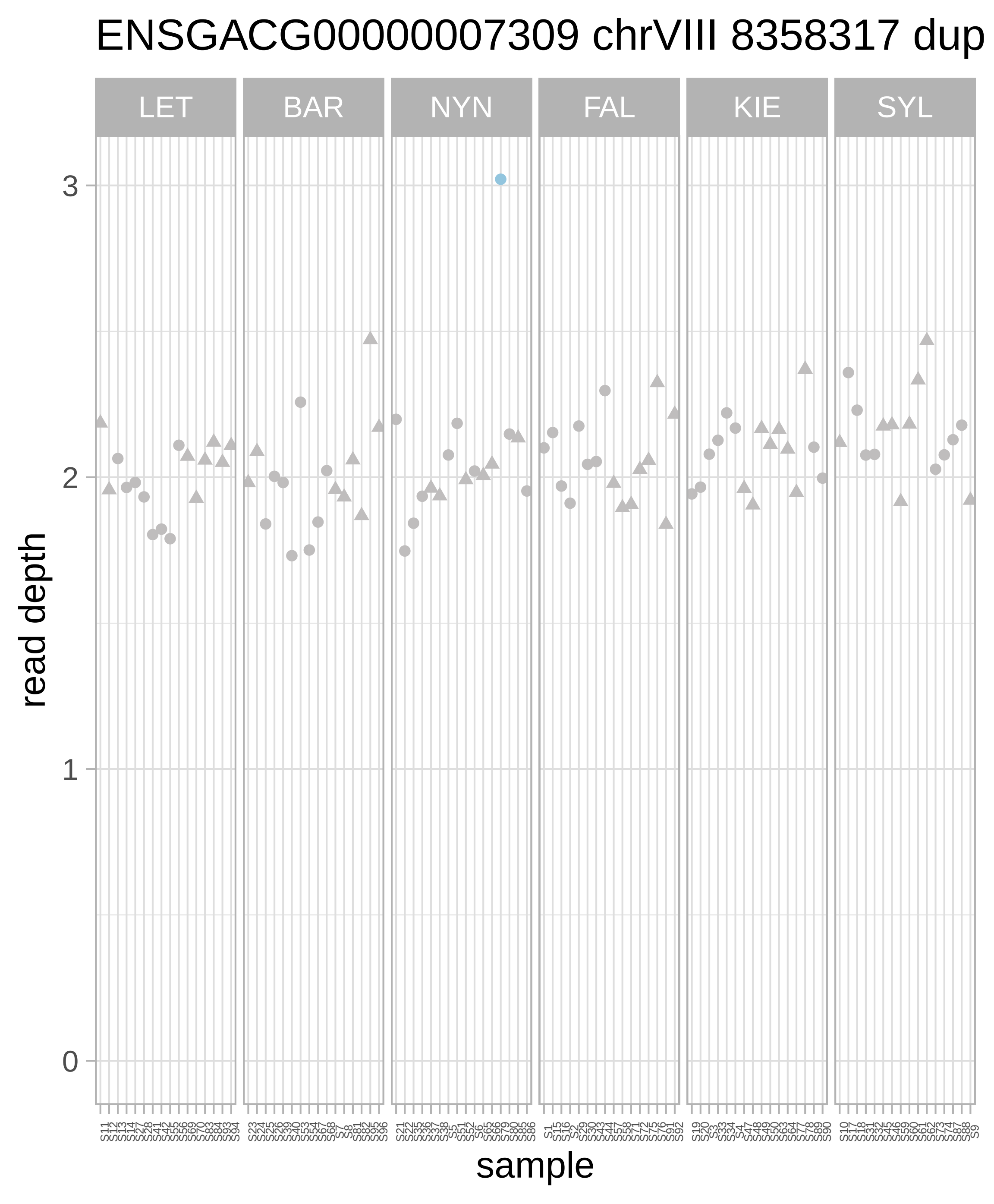

ENSGACG00000007309 chrVIII 8358317 dup
LET
BAR
NYN
FAL
KIE
SYL
read depth
sample
0
1
2
3

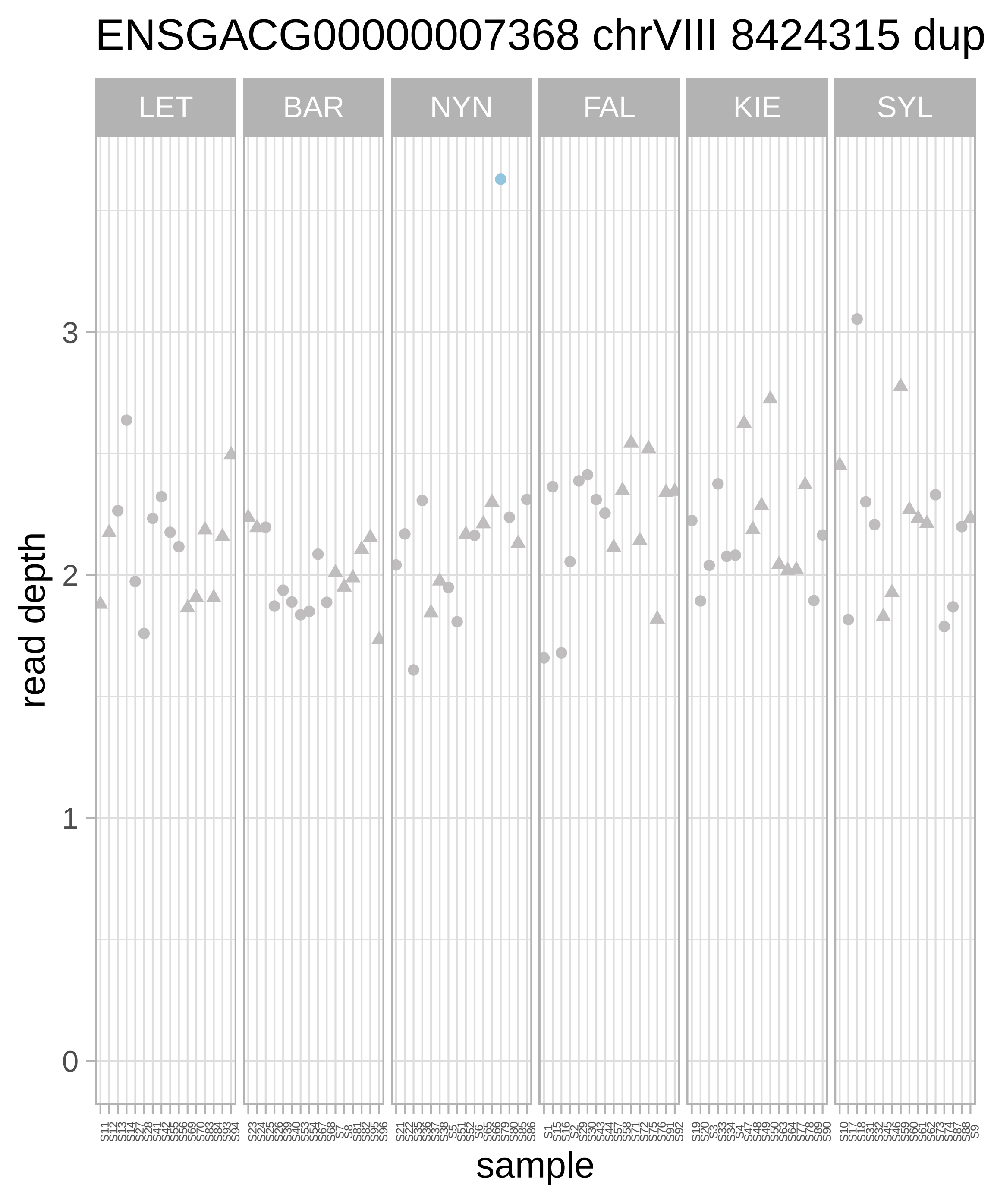
ENSGACG00000007368 chrVIII 8424315 dup
LET
BAR
NYN
FAL
KIE
SYL
read depth
3
2
1
0
sample

ENSGACG00000007974 chrVIII 9234256 dup

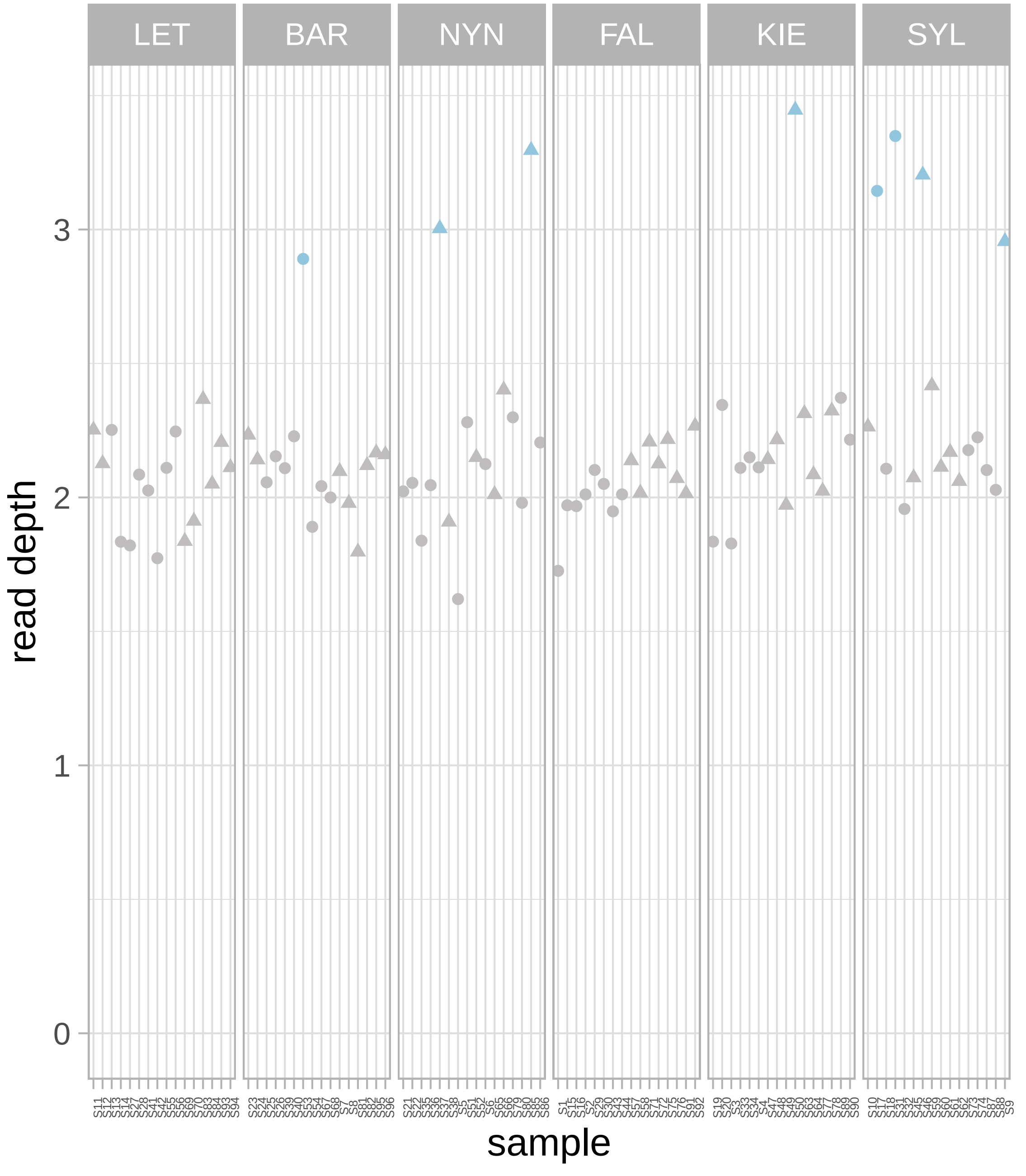

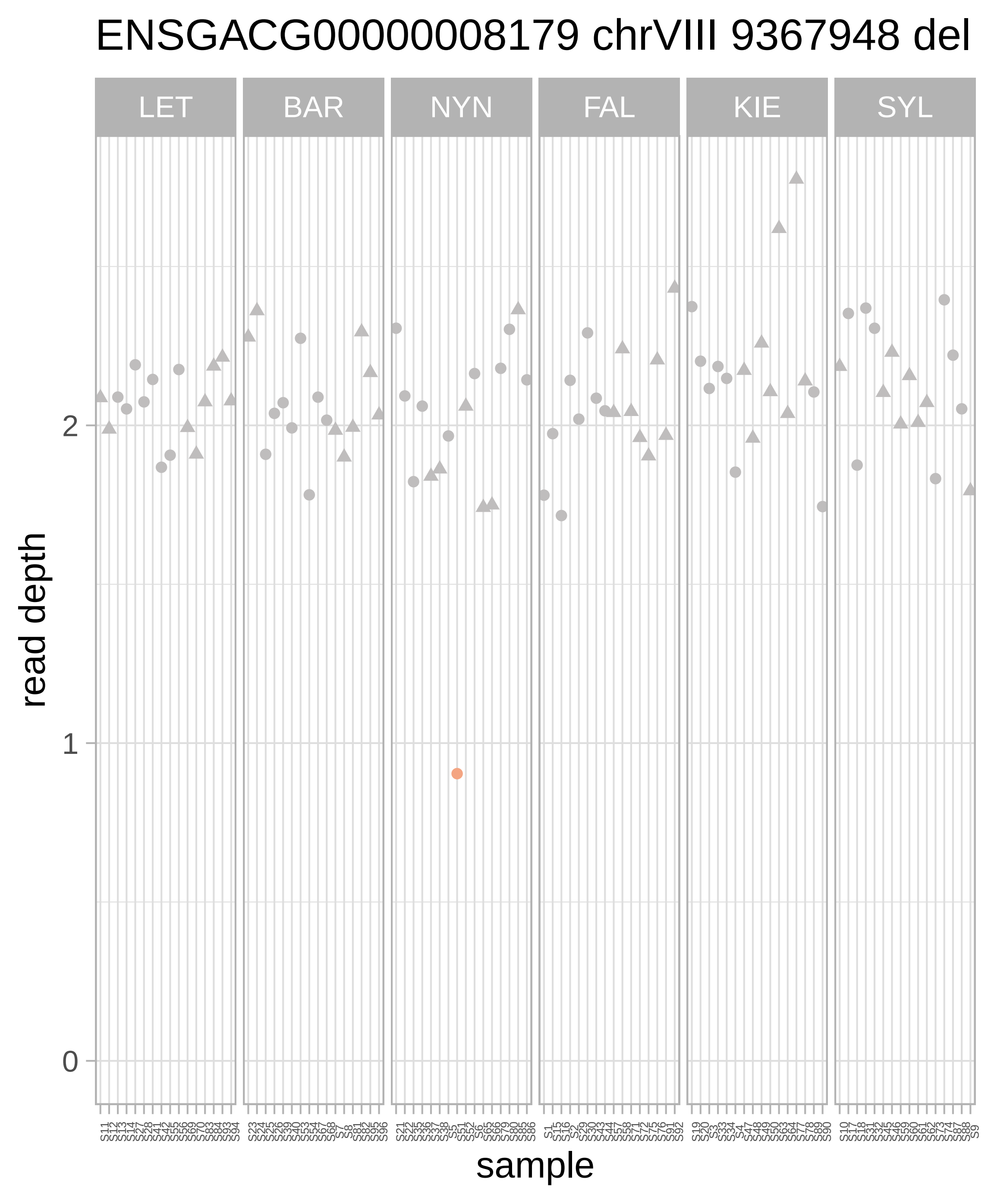
ENSGACG00000008179 chrVIII 9367948 del
LET
BAR
NYN
FAL
KIE
SYL
read depth
0
1
2
sample

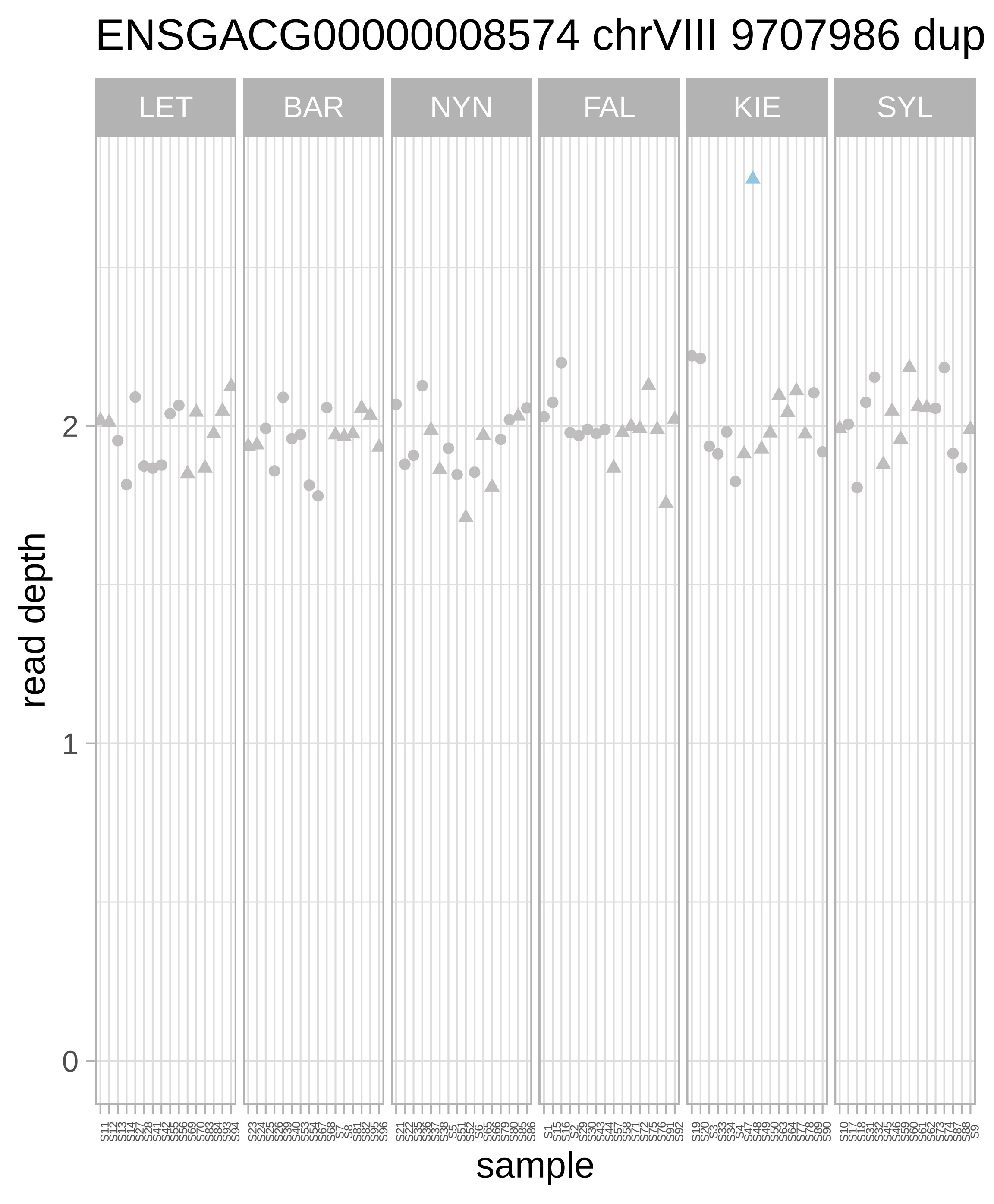

ENSGACG00000008574 chrVIII 9707986 dup
LET
BAR
NYN
FAL
KIE
SYL
read depth
0
1
2
sample
S11 S12 S13 S14 S27 S28 S41 S42 S55 S56 S69 S70 S83 S84 S93 S94
S23 S24 S25 S26 S39 S40 S53 S54 S67 S68 S7 S8 S81 S82 S95 S96
S21 S22 S35 S36 S37 S38 S5 S51 S52 S6 S65 S66 S79 S80 S85 S86
S1 S15 S16 S2 S29 S30 S43 S44 S57 S58 S71 S72 S75 S76 S91 S92
S19 S20 S3 S33 S34 S4 S47 S48 S49 S50 S63 S64 S77 S78 S89 S90
S10 S17 S18 S31 S32 S45 S46 S59 S60 S61 S62 S73 S74 S87 S88 S9

ENSGACG00000008858 chrVIII 10377571 dup

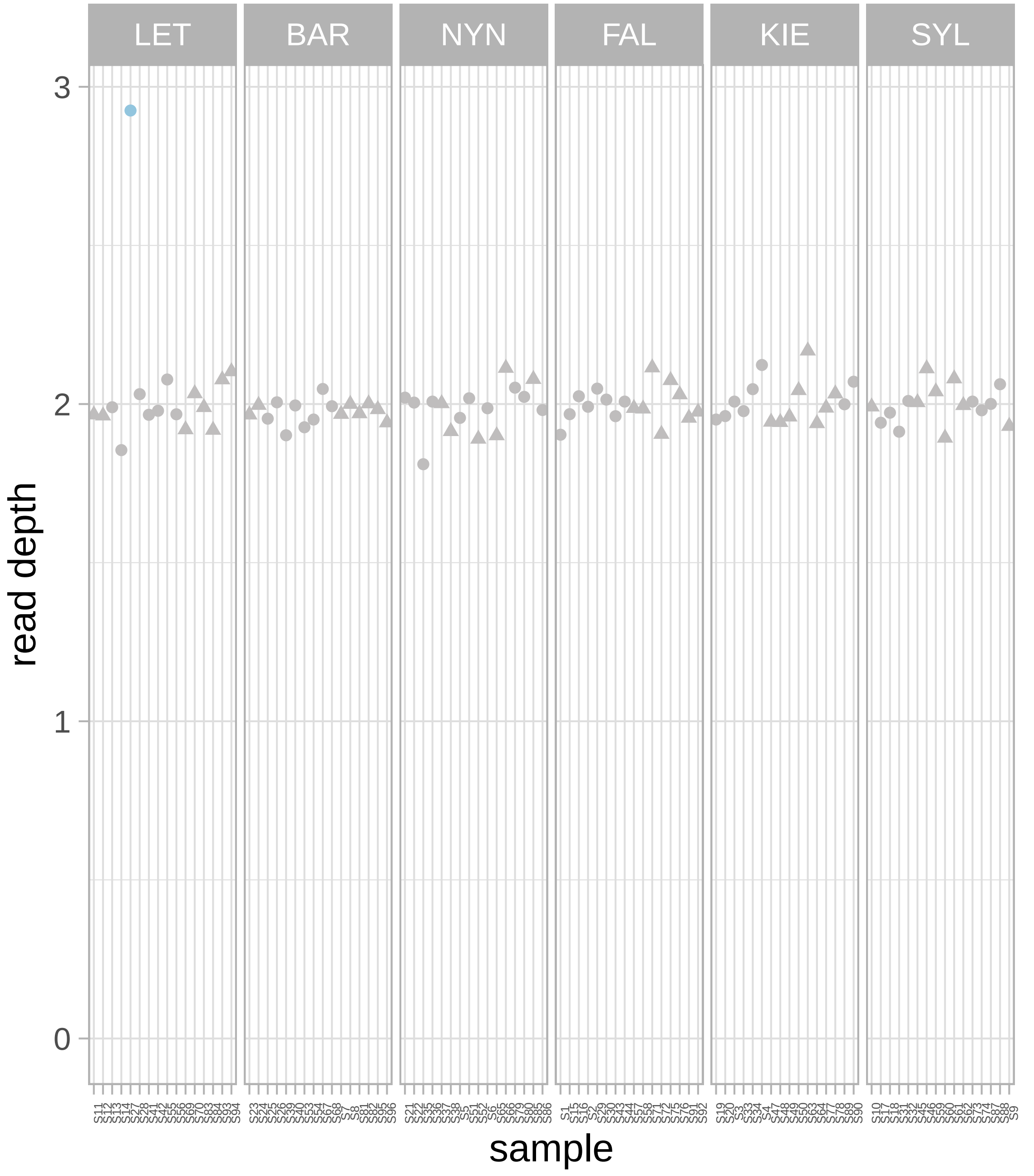

ENSGACG00000008863 chrVIII 10410857 dup

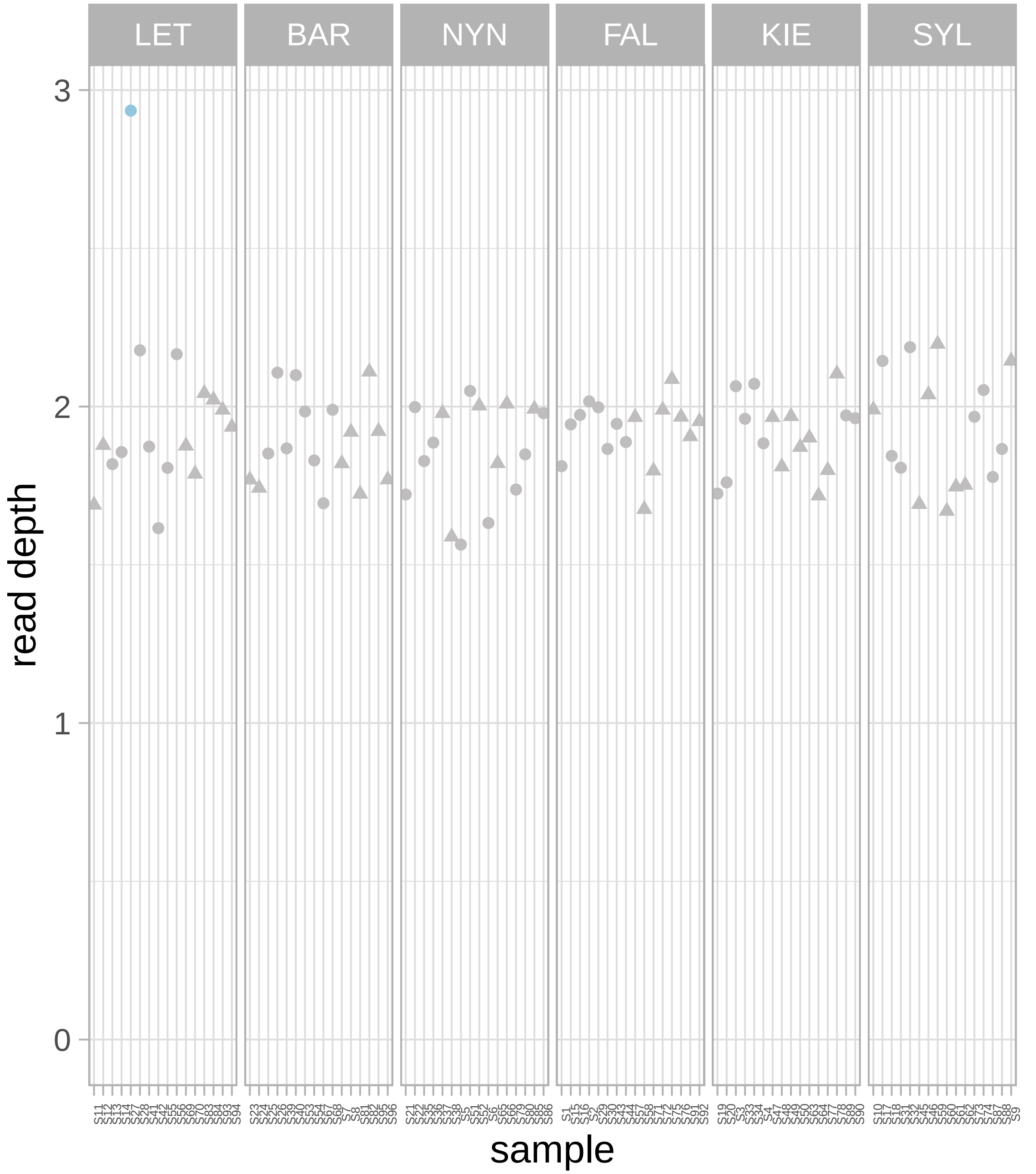

ENSGACG00000008878 chrVIII 10421882 dup

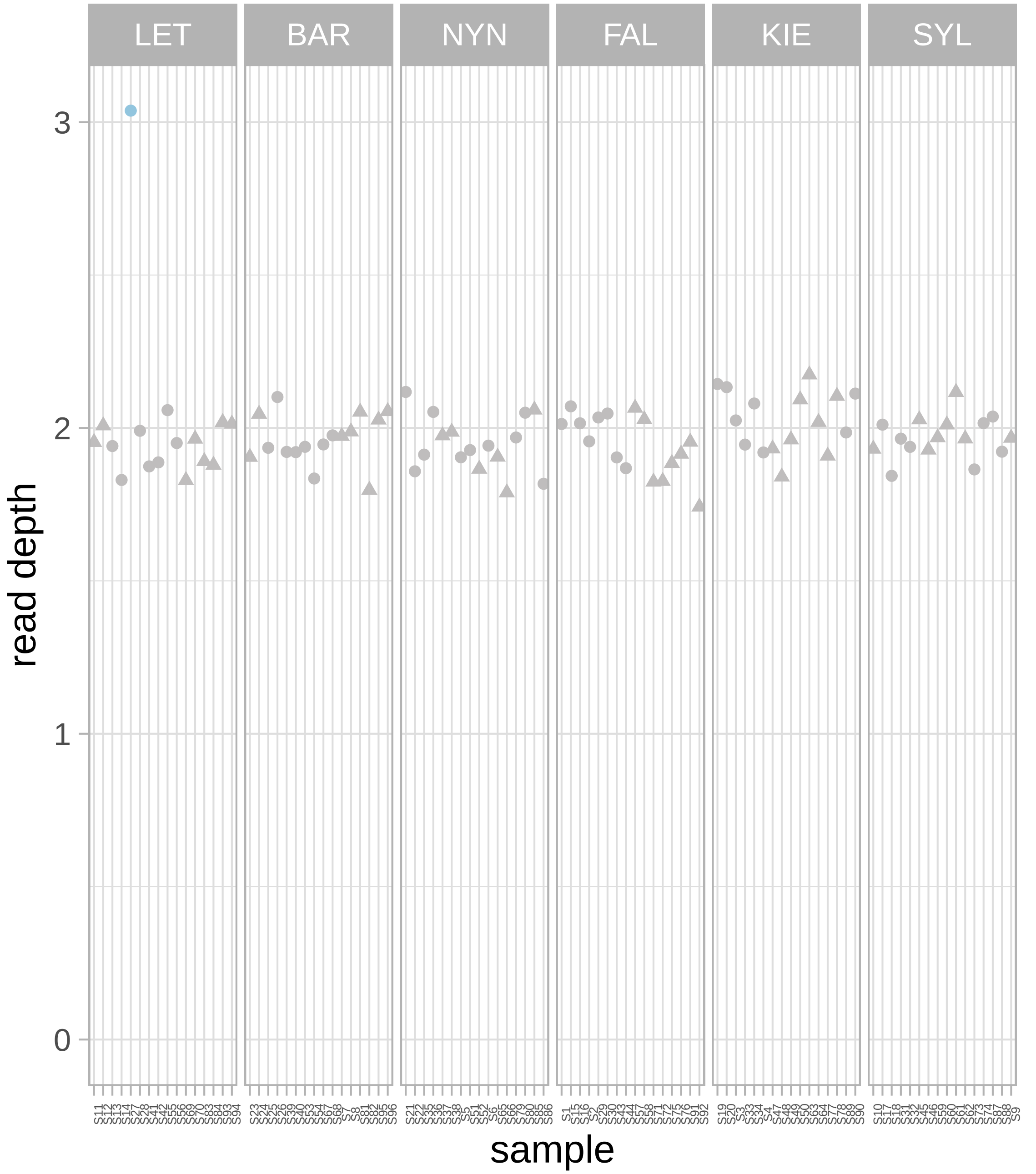

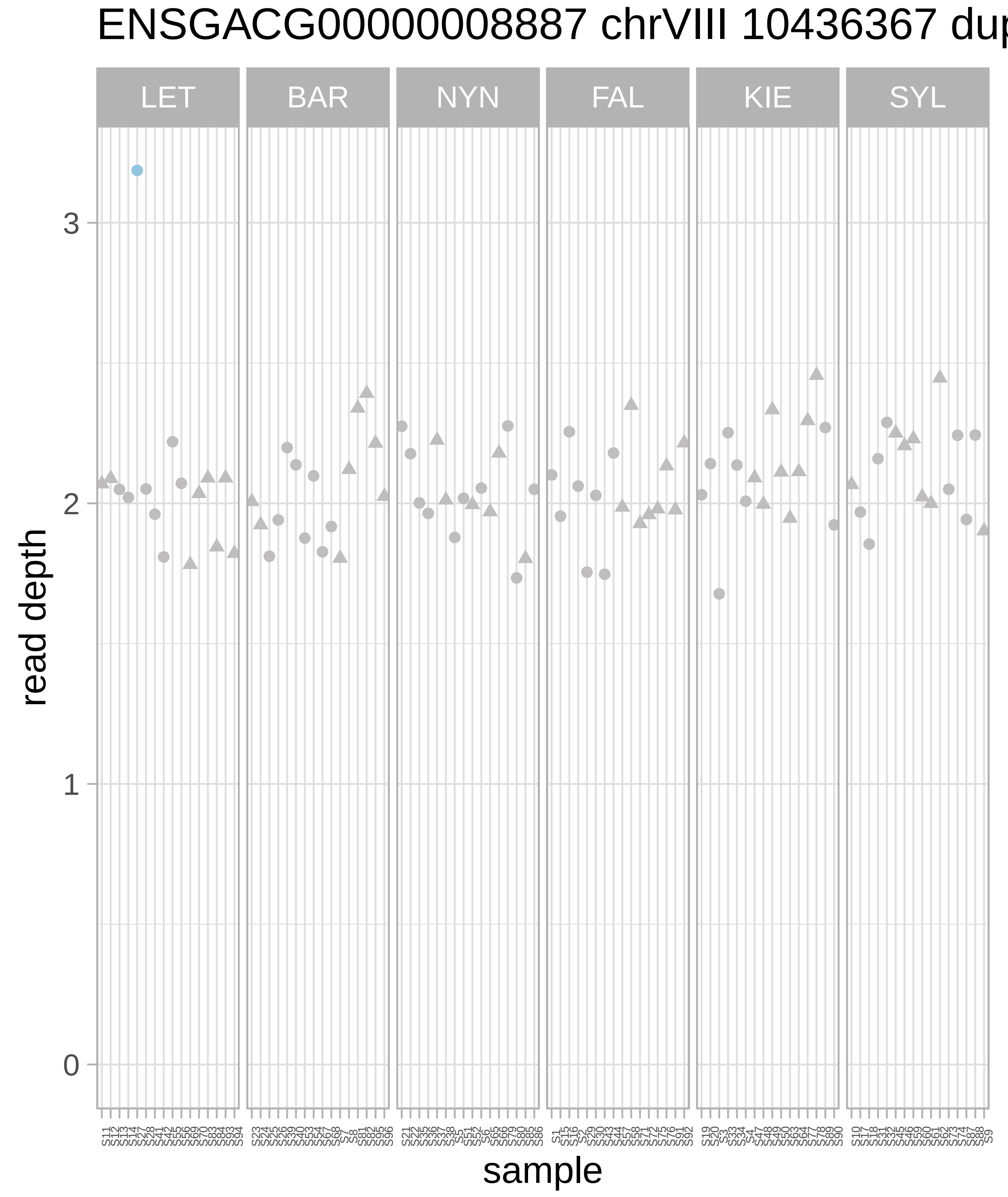
ENSGACG00000008887 chrVIII 10436367 dup
LET
BAR
NYN
FAL
KIE
SYL
read depth
3
2
1
0
sample

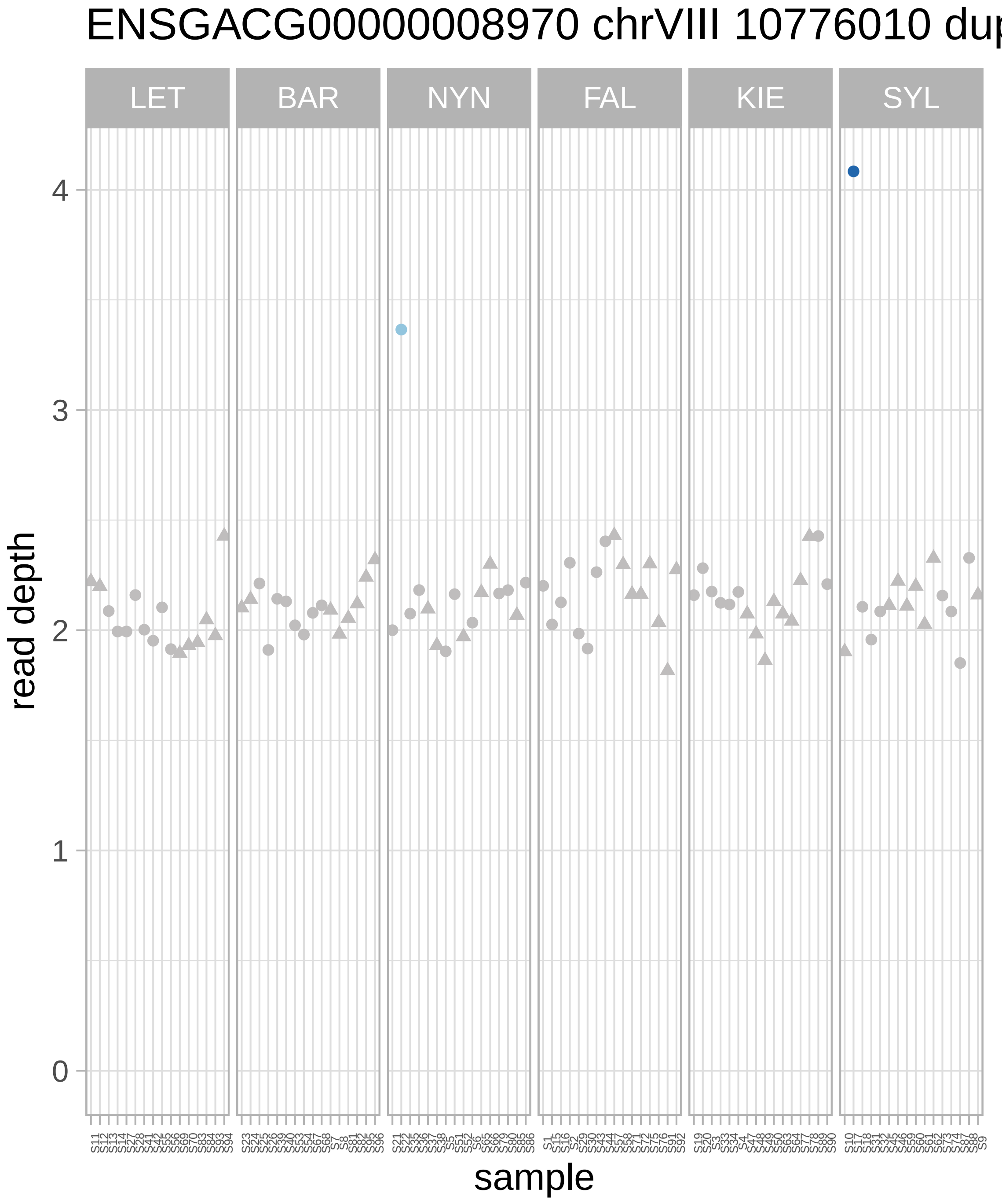
ENSGACG00000008970 chrVIII 10776010 dup
LET
BAR
NYN
FAL
KIE
SYL
read depth
0
1
2
3
4
sample

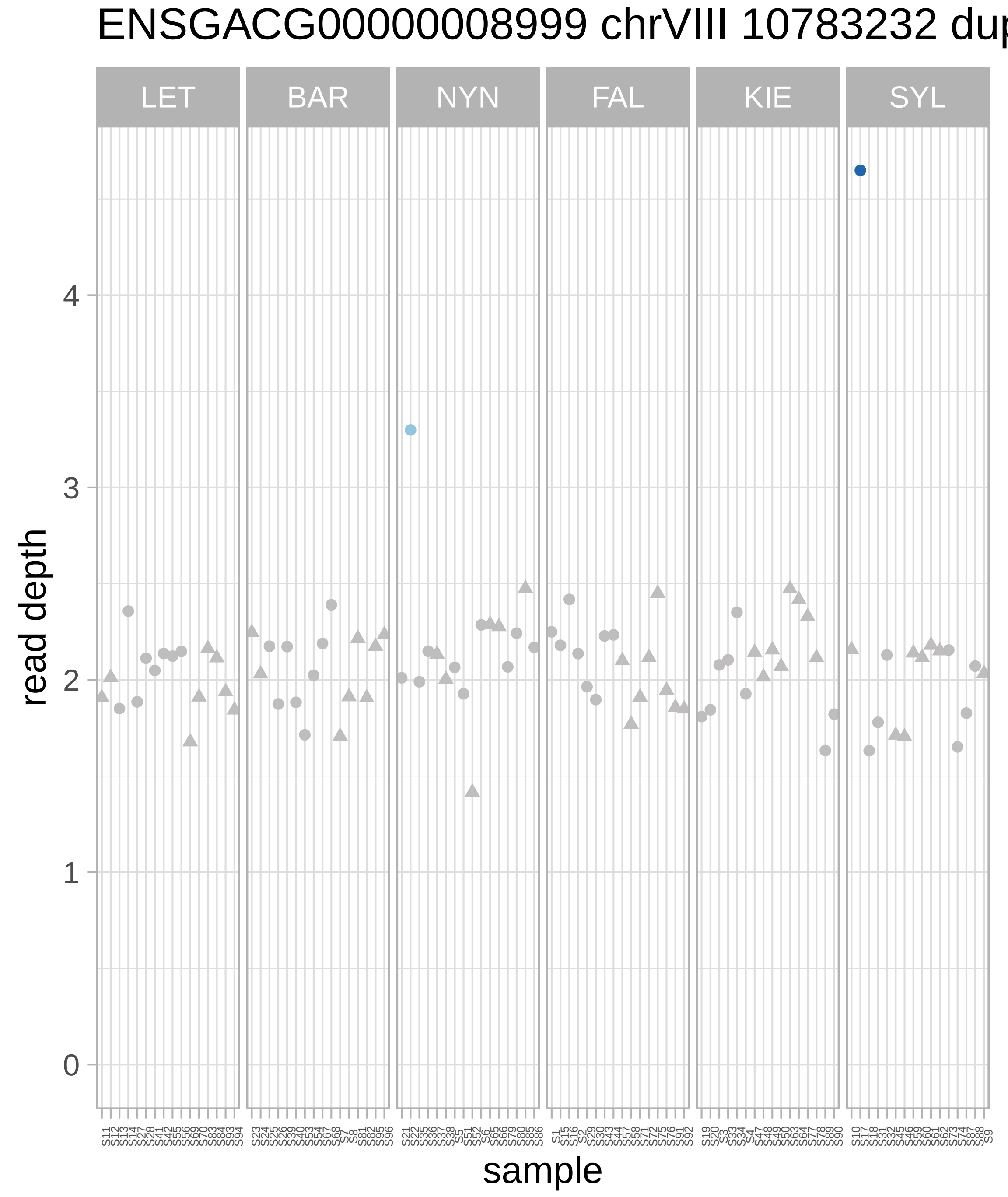
ENSGACG00000008999 chrVIII 10783232 dup
LET
BAR
NYN
FAL
KIE
SYL
read depth
0
1
2
3
4
sample

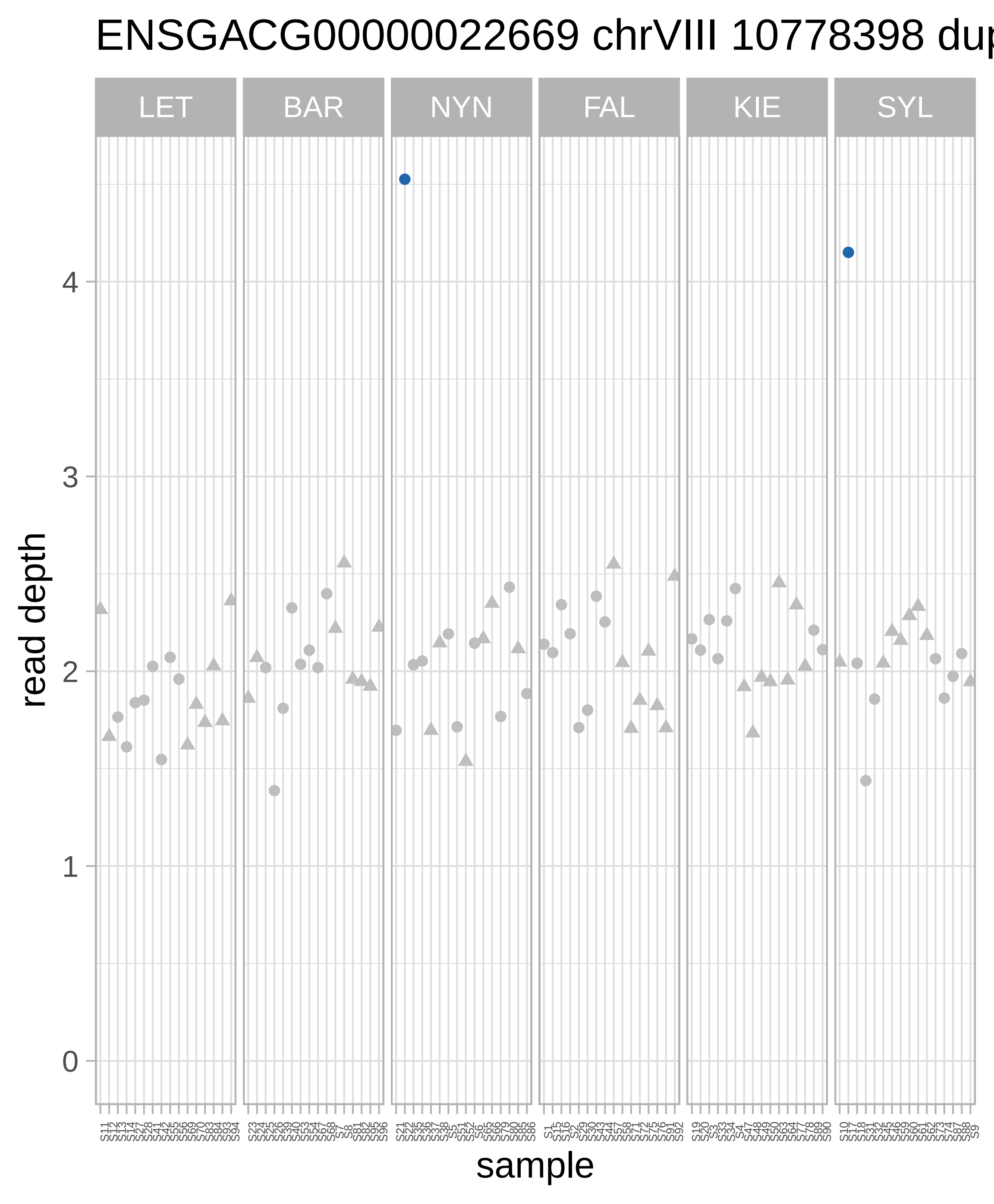

ENSGACG00000022669 chrVIII 10778398 dup
LET
BAR
NYN
FAL
KIE
SYL
read depth
0
1
2
3
4
sample

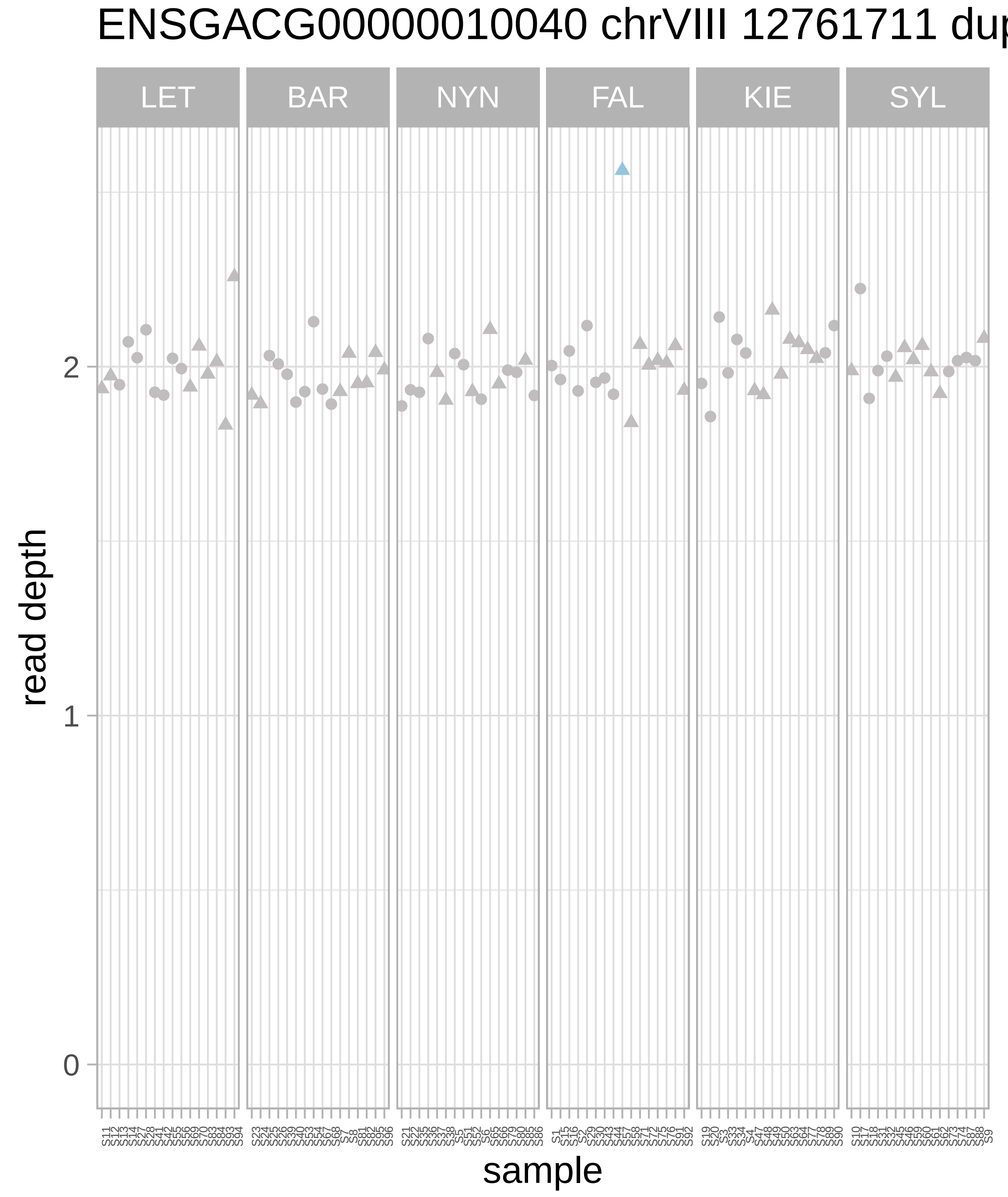
ENSGACG00000010040 chrVIII 12761711 dup
LET
BAR
NYN
FAL
KIE
SYL
read depth
0
1
2
sample
S11 S12 S13 S14 S27 S28 S41 S42 S55 S56 S69 S70 S83 S84 S93 S94
S23 S24 S25 S26 S39 S40 S53 S54 S67 S68 S7 S8 S81 S82 S95 S96
S21 S22 S35 S36 S37 S38 S5 S51 S52 S6 S65 S66 S79 S80 S85 S86
S1 S15 S16 S2 S29 S30 S43 S44 S57 S58 S71 S72 S75 S76 S91 S92
S19 S20 S3 S33 S34 S4 S47 S48 S49 S50 S63 S64 S77 S78 S89 S90
S10 S17 S18 S31 S32 S45 S46 S59 S60 S61 S62 S73 S74 S87 S88 S9

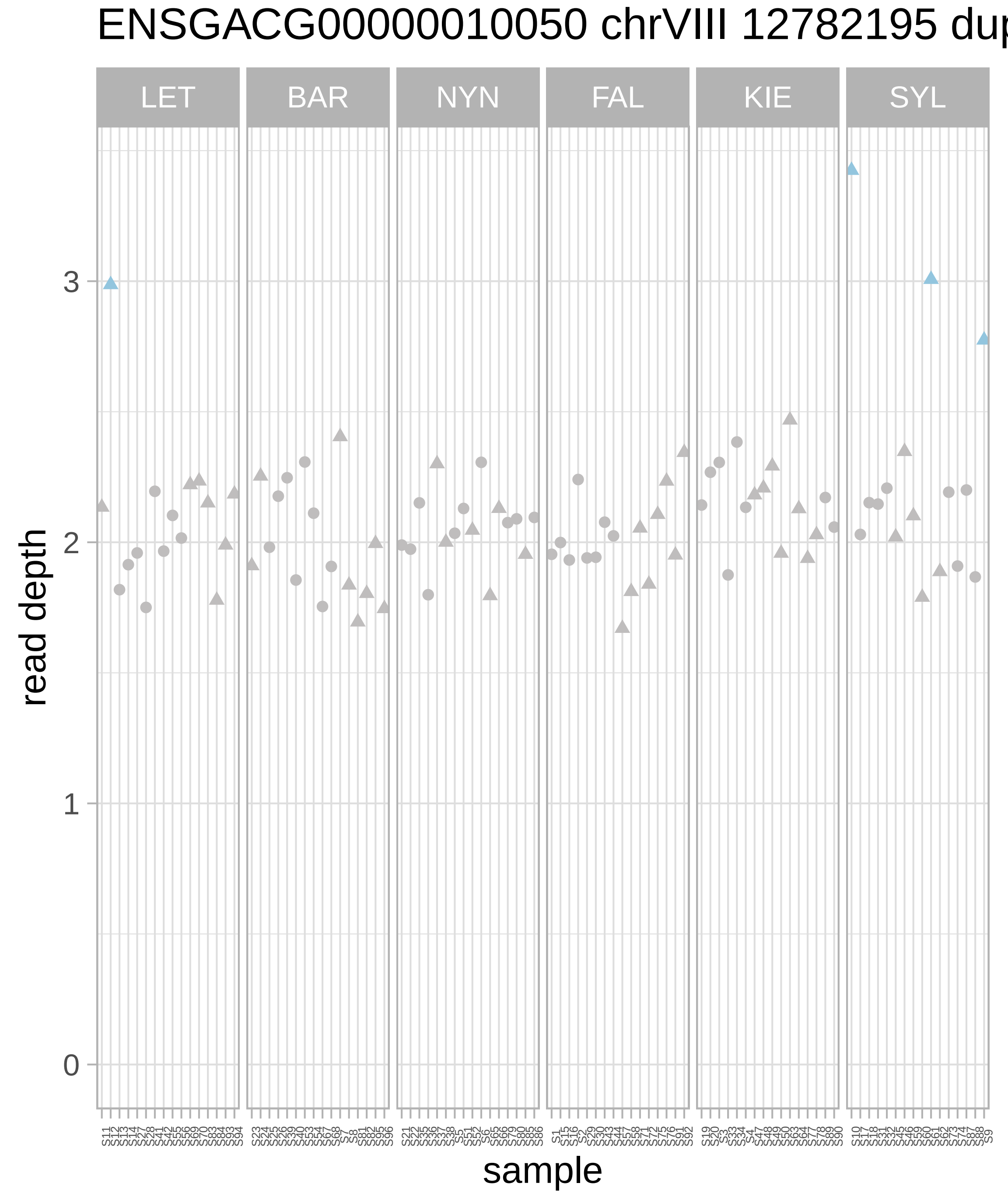
ENSGACG00000010050 chrVIII 12782195 dup
LET
BAR
NYN
FAL
KIE
SYL
read depth
3
2
1
0
sample

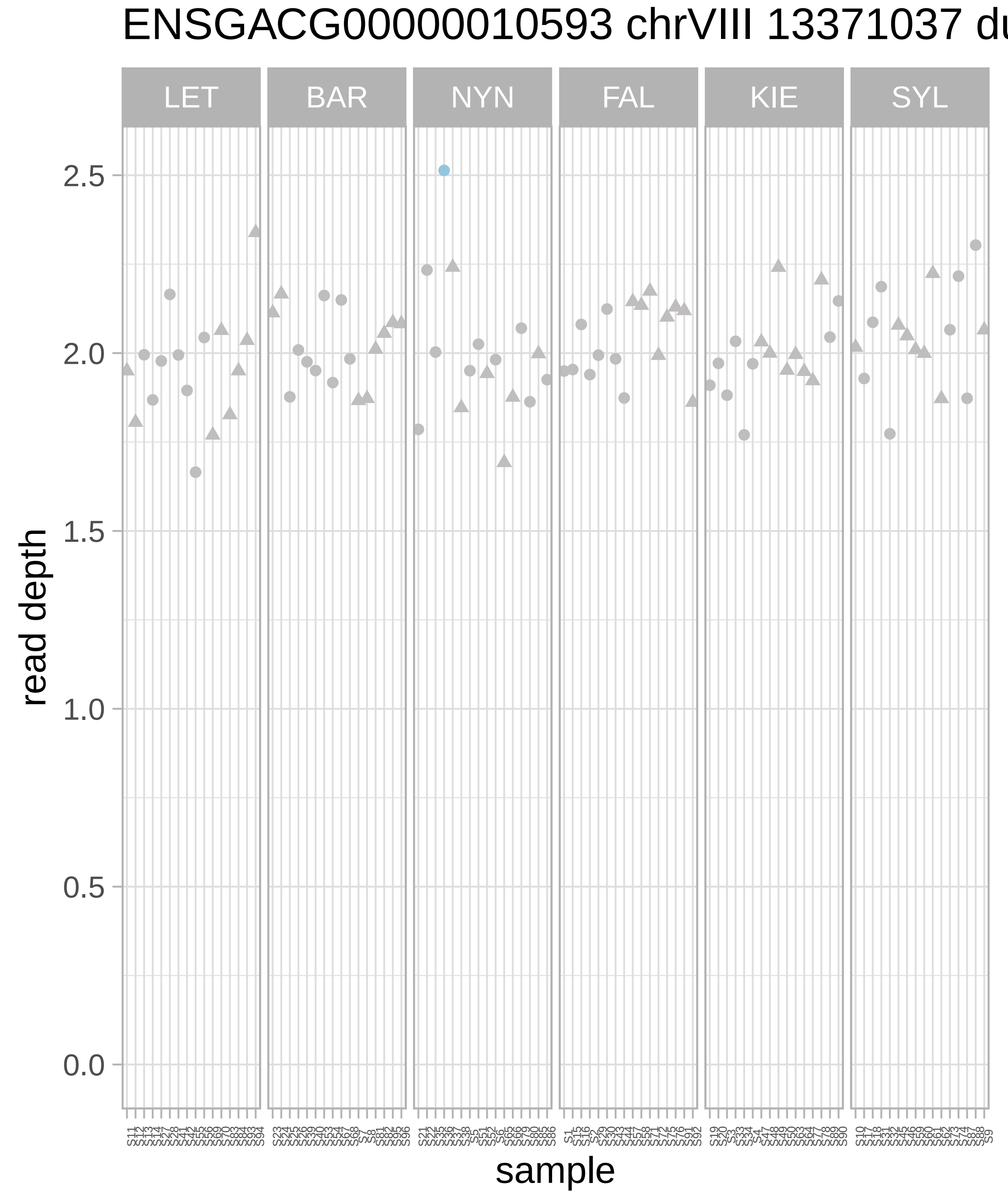
ENSGACG00000010593 chrVIII 13371037 du
LET
BAR
NYN
FAL
KIE
SYL
read depth
2.5
2.0
1.5
1.0
0.5
0.0
sample

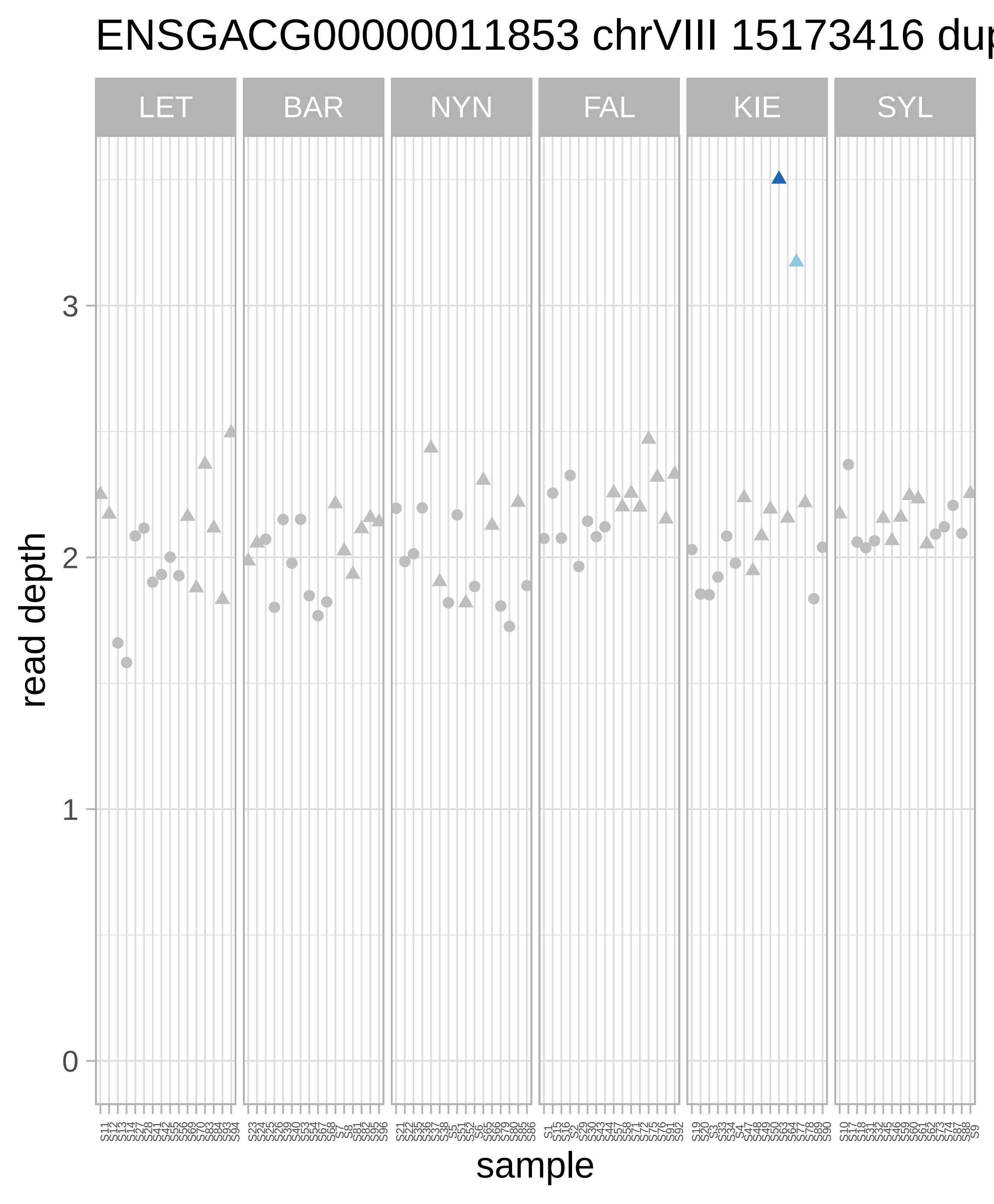
ENSGACG00000011853 chrVIII 15173416 dup
LET
BAR
NYN
FAL
KIE
SYL
read depth
0
1
2
3
sample

ENSGACG00000012639 chrVIII 15988071 del

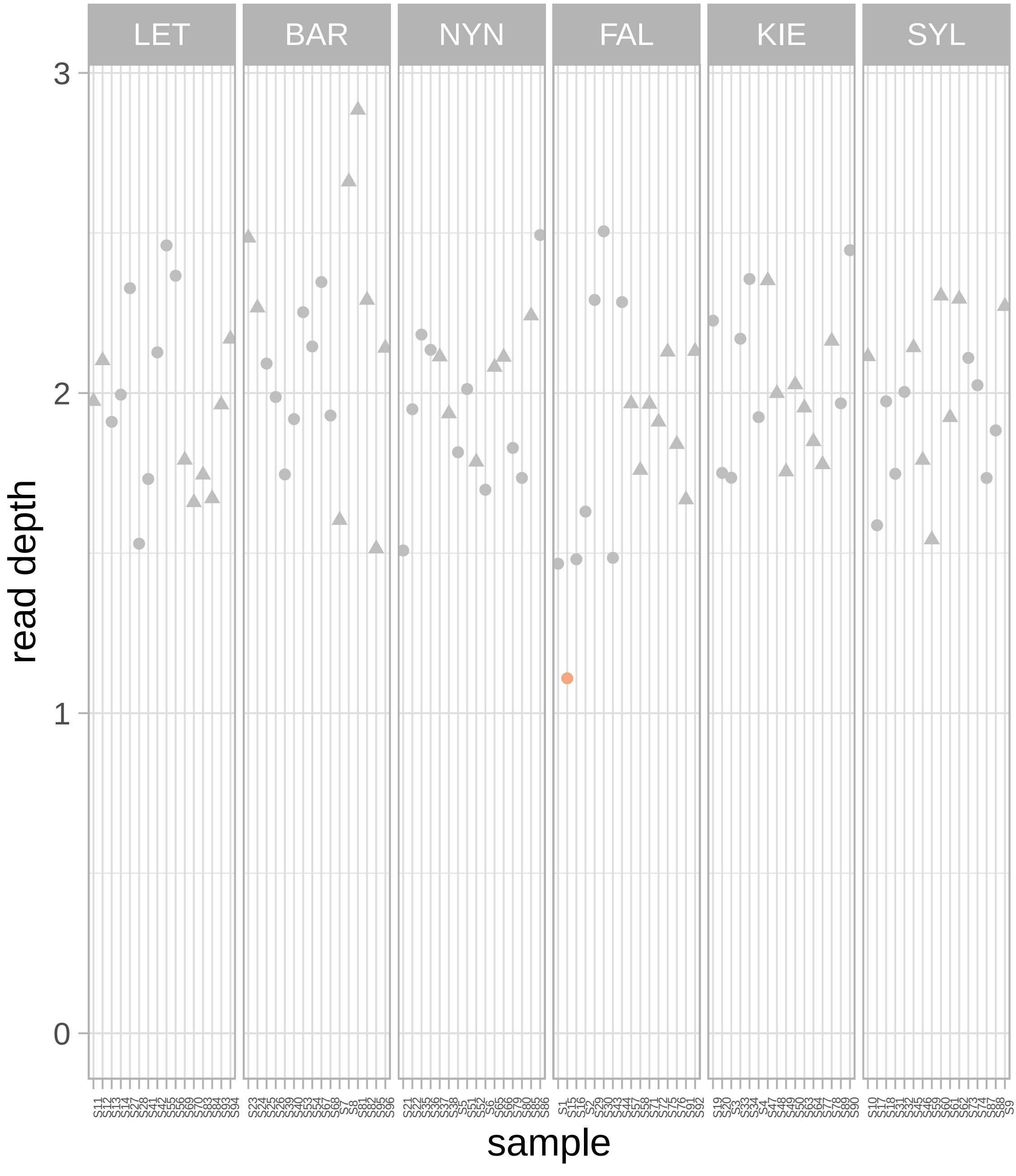

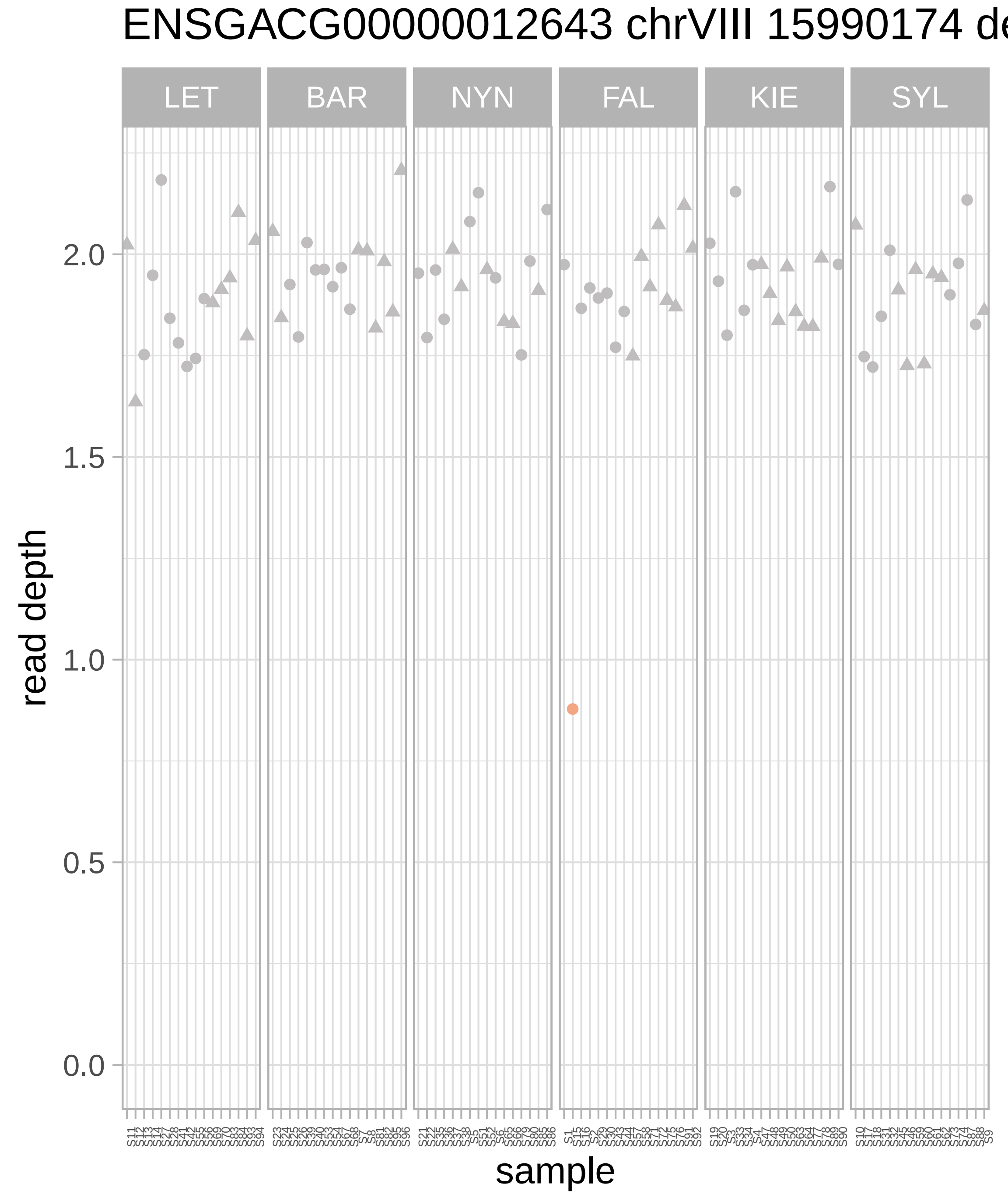
ENSGACG00000012643 chrVIII 15990174 de
LET
BAR
NYN
FAL
KIE
SYL
read depth
sample
0.0
0.5
1.0
1.5
2.0

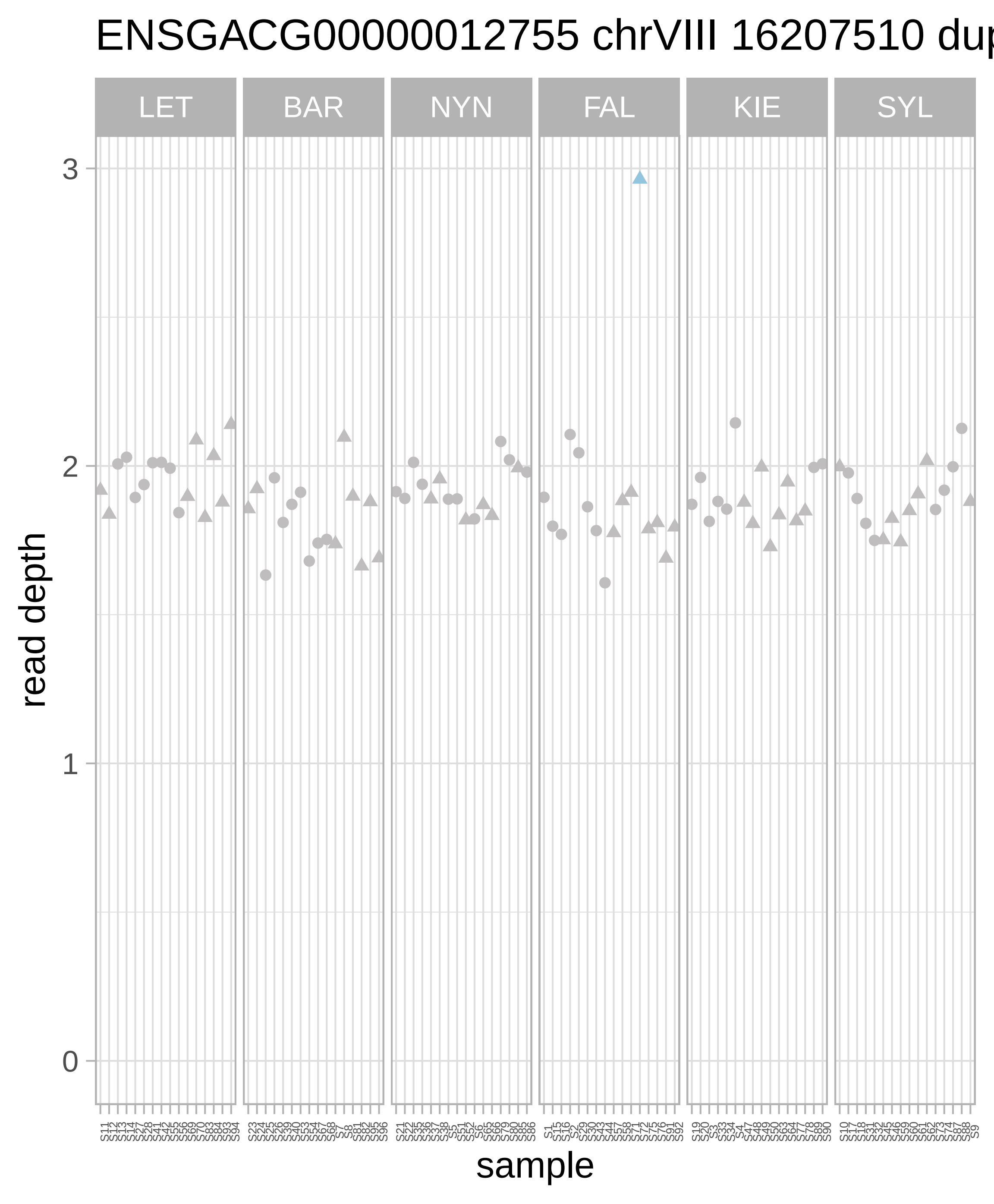

ENSGACG00000012755 chrVIII 16207510 dup
LET
BAR
NYN
FAL
KIE
SYL
read depth
0
1
2
3
sample

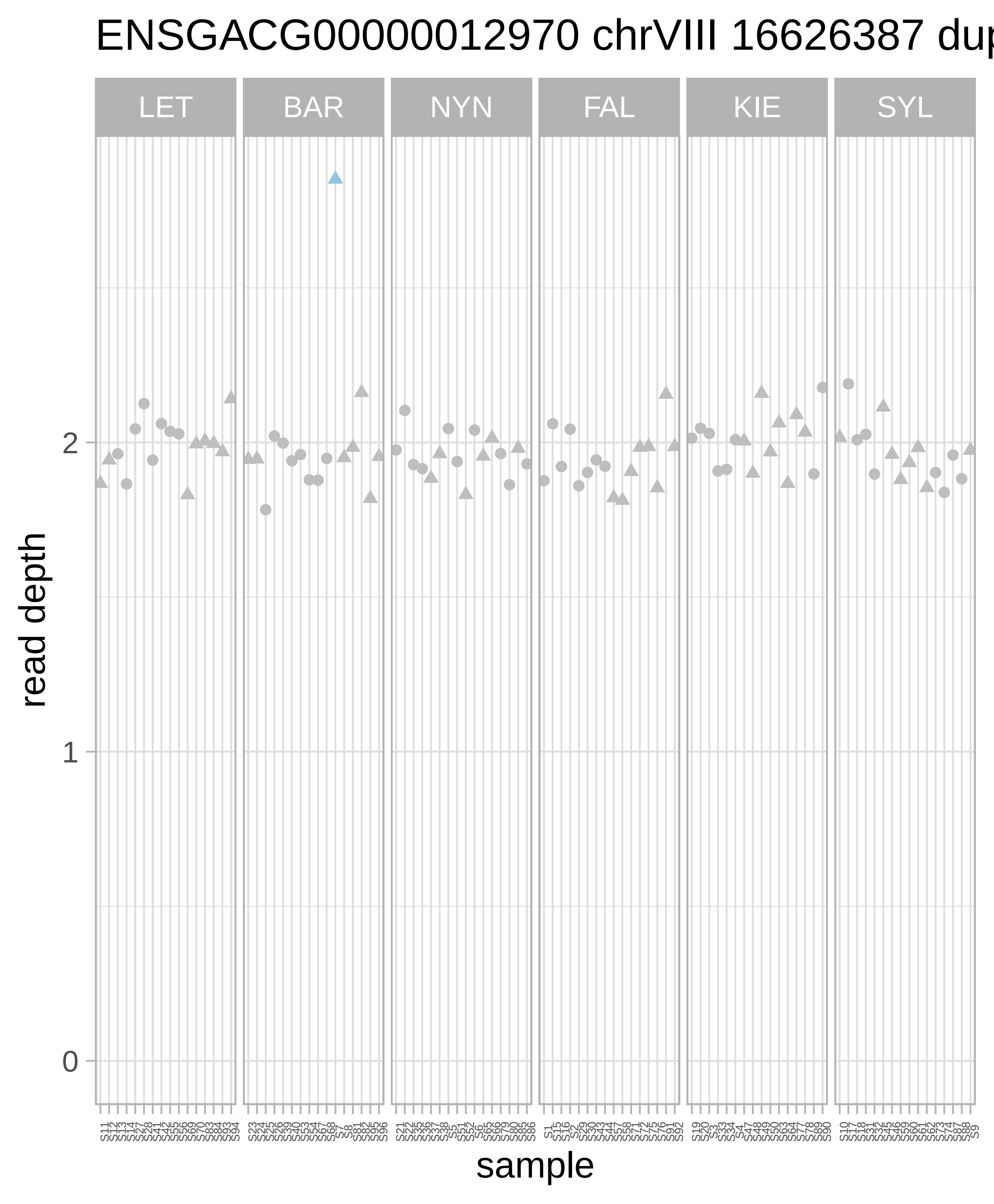
ENSGACG00000012970 chrVIII 16626387 dup
LET
BAR
NYN
FAL
KIE
SYL
read depth
0
1
2
S11 S12 S13 S14 S27 S28 S41 S42 S55 S56 S69 S70 S83 S84 S93 S94
S23 S24 S25 S26 S39 S40 S53 S54 S67 S68 S7 S8 S81 S82 S95 S96
S21 S22 S35 S36 S37 S38 S5 S51 S52 S6 S65 S66 S79 S80 S85 S86
S1 S15 S16 S2 S29 S30 S43 S44 S57 S58 S71 S72 S75 S76 S91 S92
S19 S20 S3 S33 S34 S4 S47 S48 S49 S50 S63 S64 S77 S78 S89 S90
S10 S17 S18 S31 S32 S45 S46 S59 S60 S61 S62 S73 S74 S87 S88 S9
sample

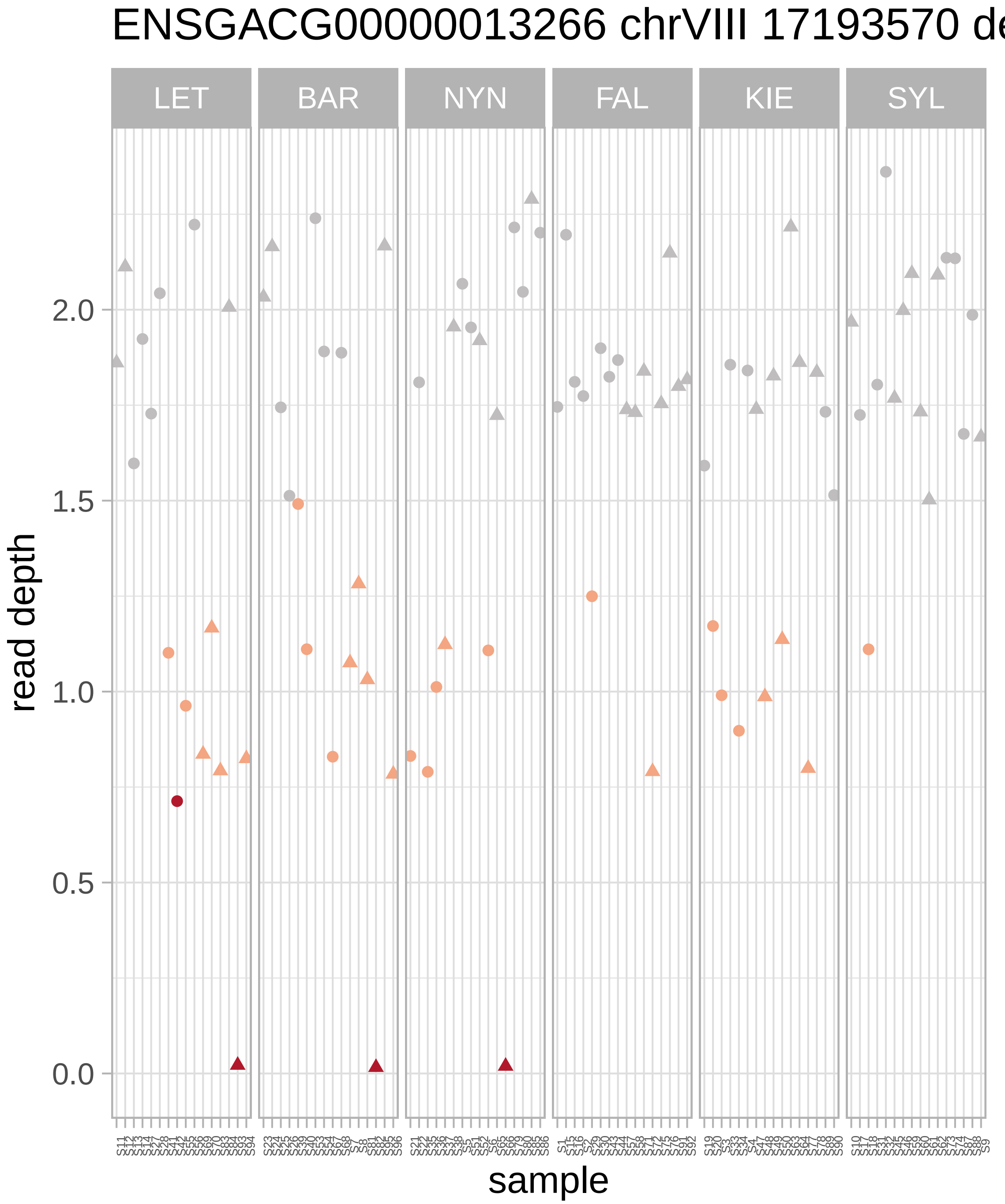
ENSGACG00000013266 chrVIII 17193570 de
LET
BAR
NYN
FAL
KIE
SYL
read depth
0.0
0.5
1.0
1.5
2.0
S11 S12 S13 S14 S27 S28 S41 S42 S55 S56 S69 S70 S83 S84 S93 S94
S23 S24 S25 S26 S39 S40 S53 S54 S67 S68 S7 S8 S81 S82 S95 S96
S21 S22 S35 S36 S37 S38 S5 S51 S52 S6 S65 S66 S79 S80 S85 S86
S1 S15 S16 S2 S29 S30 S43 S44 S57 S58 S71 S72 S75 S76 S91 S92
S19 S20 S3 S33 S34 S4 S47 S48 S49 S50 S63 S64 S77 S78 S89 S90
S10 S17 S18 S31 S32 S45 S46 S59 S60 S61 S62 S73 S74 S87 S88 S9
sample

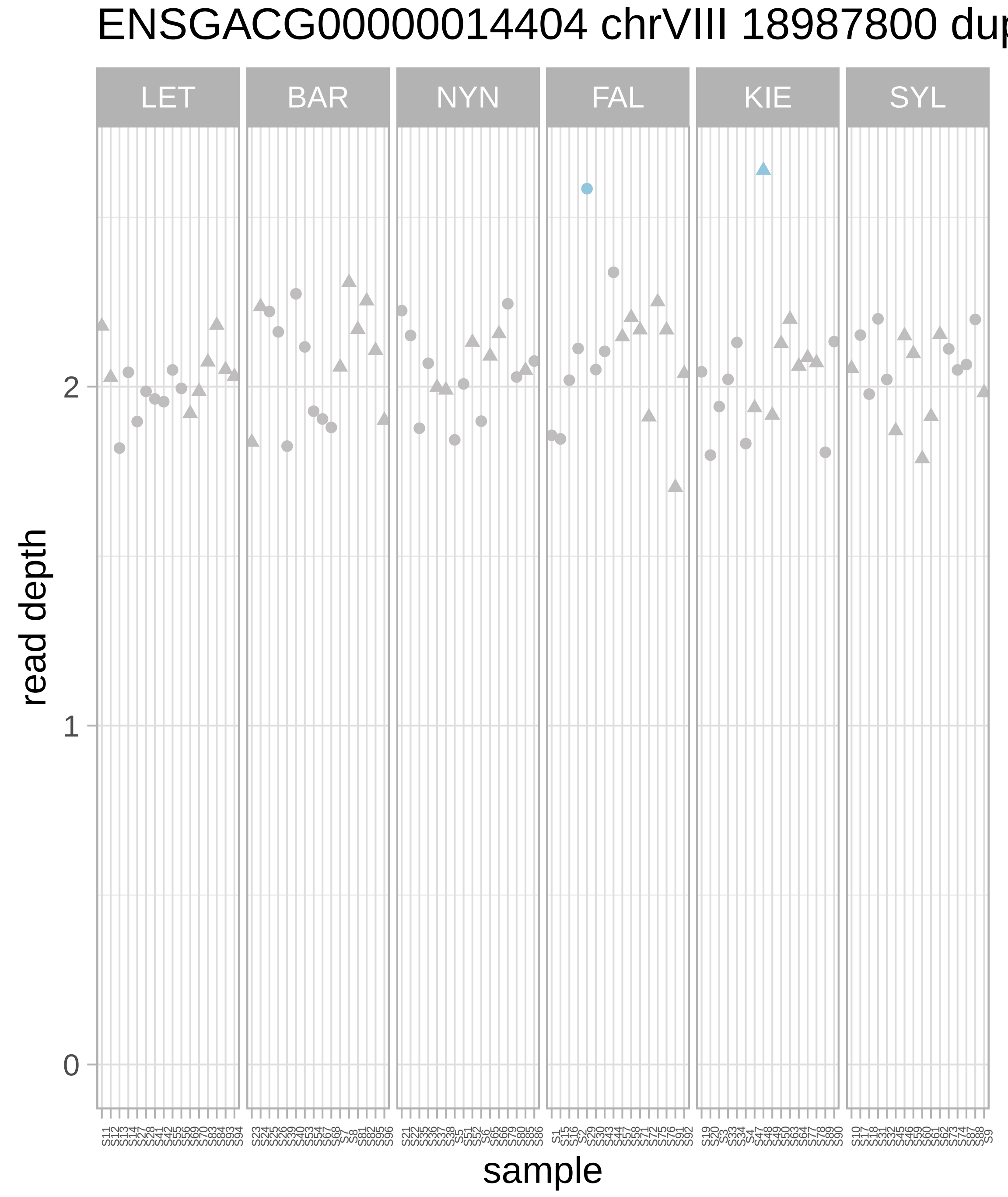
ENSGACG00000014404 chrVIII 18987800 dup
LET
BAR
NYN
FAL
KIE
SYL
read depth
0
1
2
sample

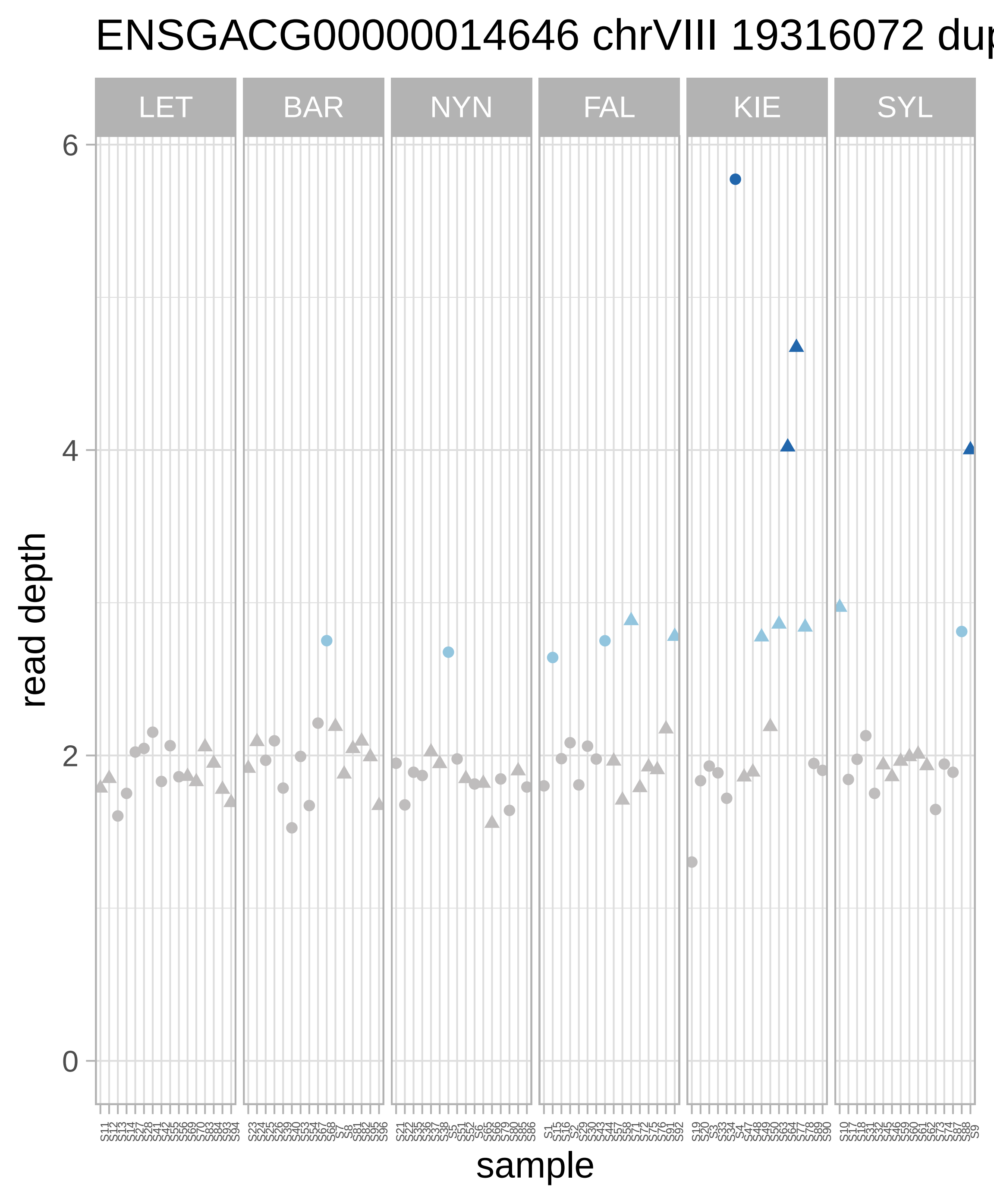
ENSGACG00000014646 chrVIII 19316072 dup
LET
BAR
NYN
FAL
KIE
SYL
read depth
0
2
4
6
sample

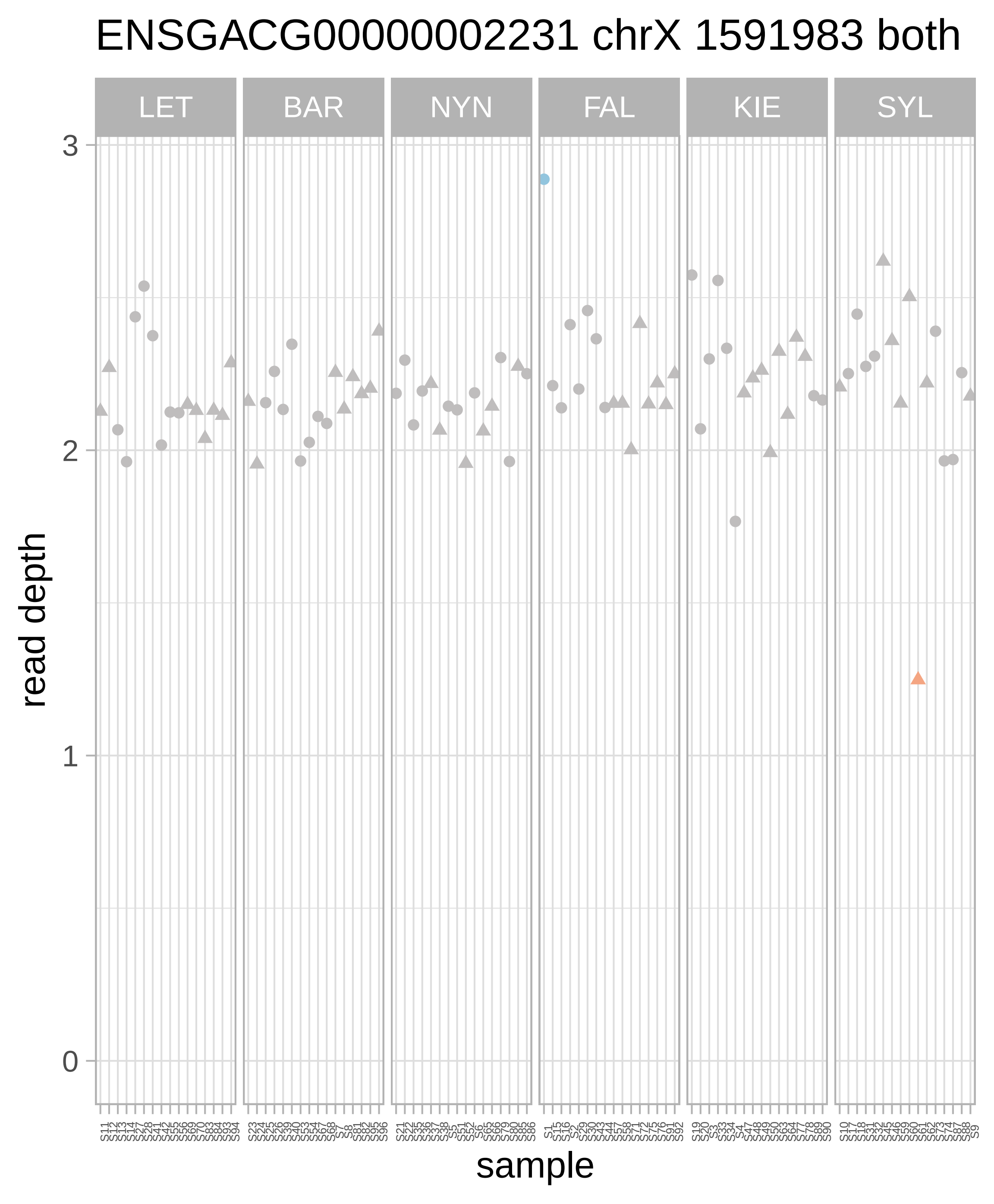
ENSGACG00000002231 chrX 1591983 both
LET
BAR
NYN
FAL
KIE
SYL
read depth
sample
0
1
2
3

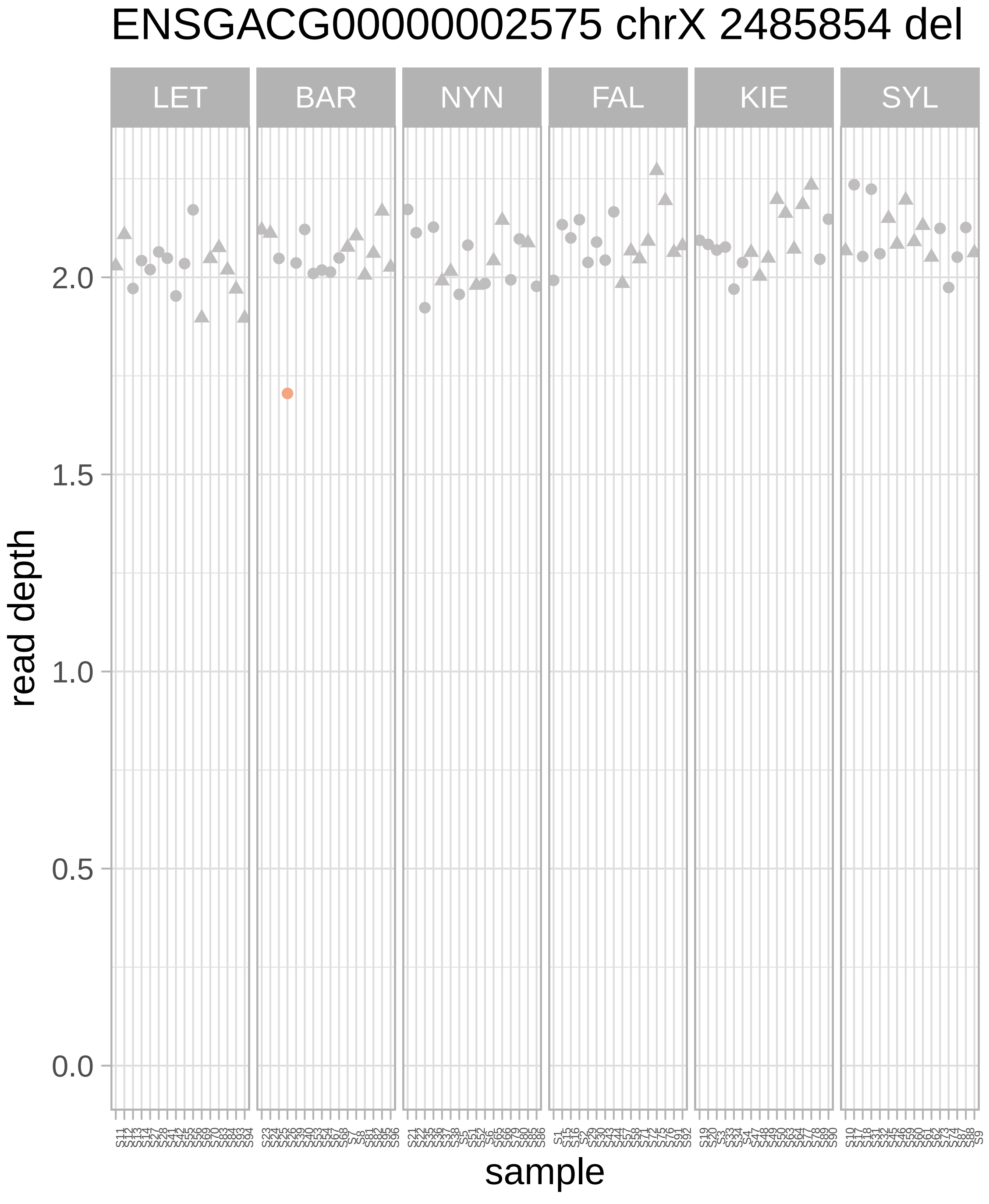
ENSGACG00000002575 chrX 2485854 del
LET
BAR
NYN
FAL
KIE
SYL
read depth
0.0
0.5
1.0
1.5
2.0
sample

ENSGACG00000002946 chrX 3330458 dup

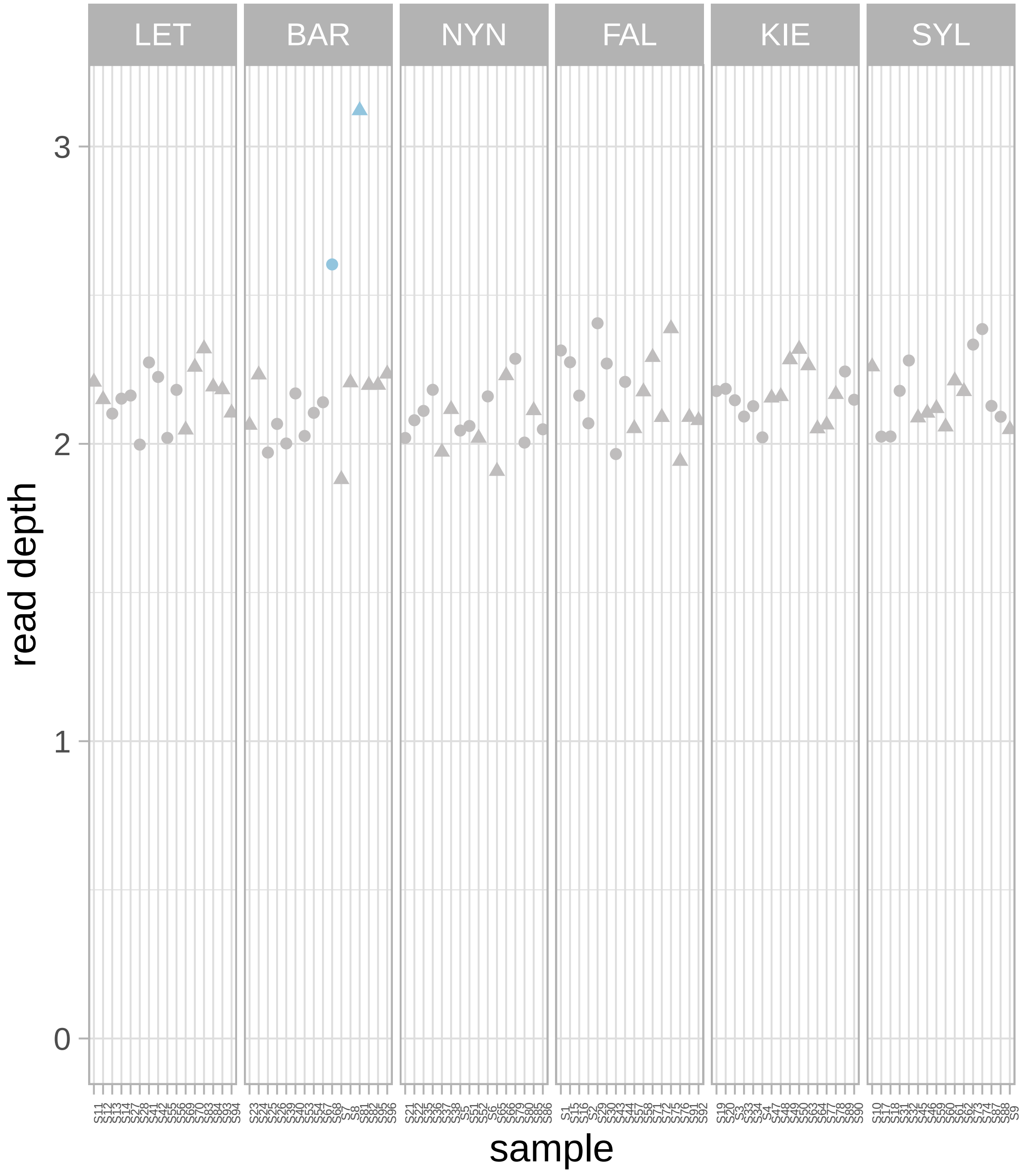

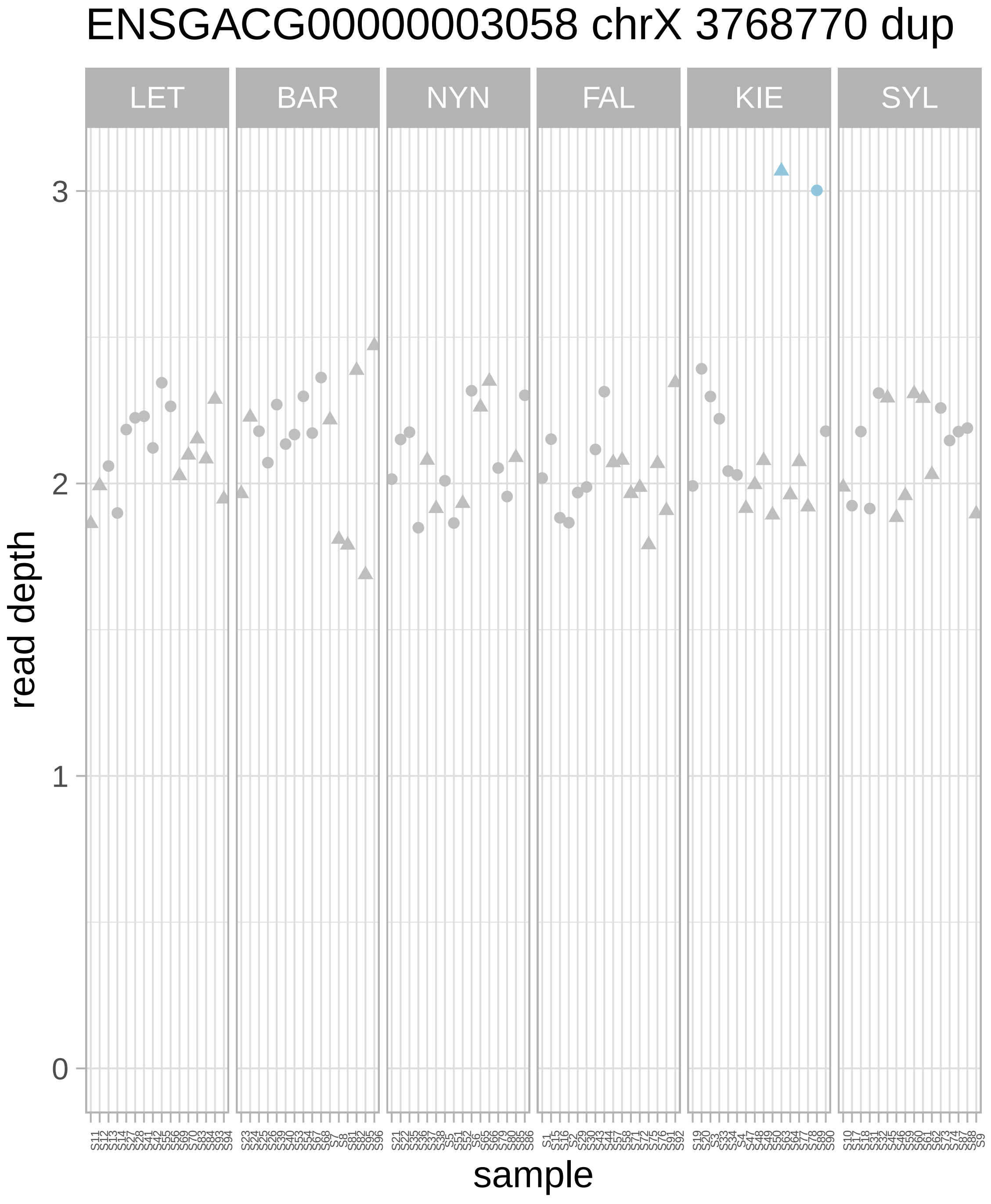
ENSGACG00000003058 chrX 3768770 dup
LET
BAR
NYN
FAL
KIE
SYL
read depth
0
1
2
3
S11 S12 S13 S14 S27 S28 S41 S42 S55 S56 S69 S70 S83 S84 S93 S94
S23 S24 S25 S26 S39 S40 S53 S54 S67 S68 S7 S8 S81 S82 S95 S96
S21 S22 S35 S36 S37 S38 S5 S51 S52 S6 S65 S66 S79 S80 S85 S86
S1 S15 S16 S2 S29 S30 S43 S44 S57 S58 S71 S72 S75 S76 S91 S92
S19 S20 S3 S33 S34 S4 S47 S48 S49 S50 S63 S64 S77 S78 S89 S90
S10 S17 S18 S31 S32 S45 S46 S59 S60 S61 S62 S73 S74 S87 S88 S9
sample

ENSGACG00000003061 chrX 3777496 dup

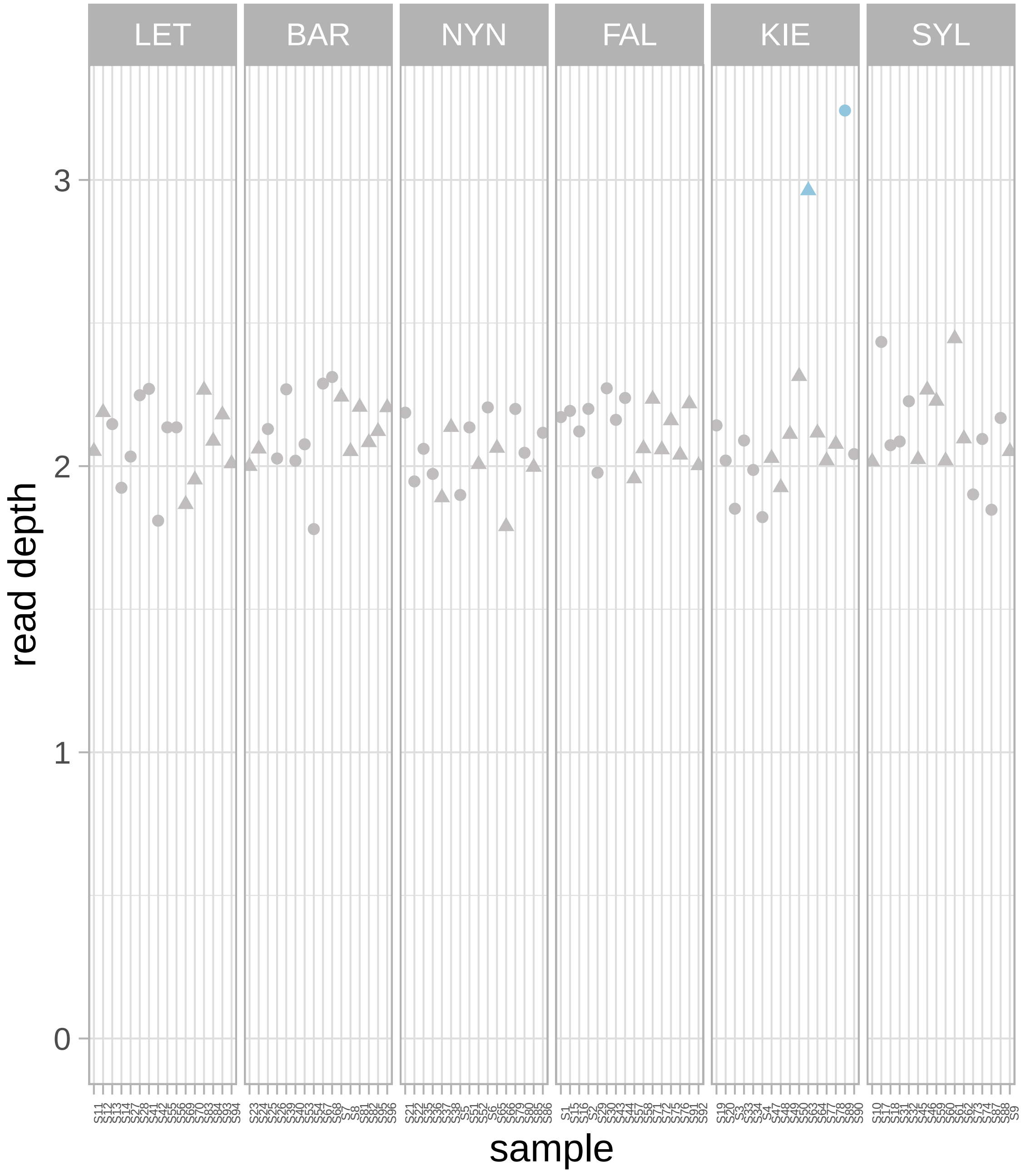

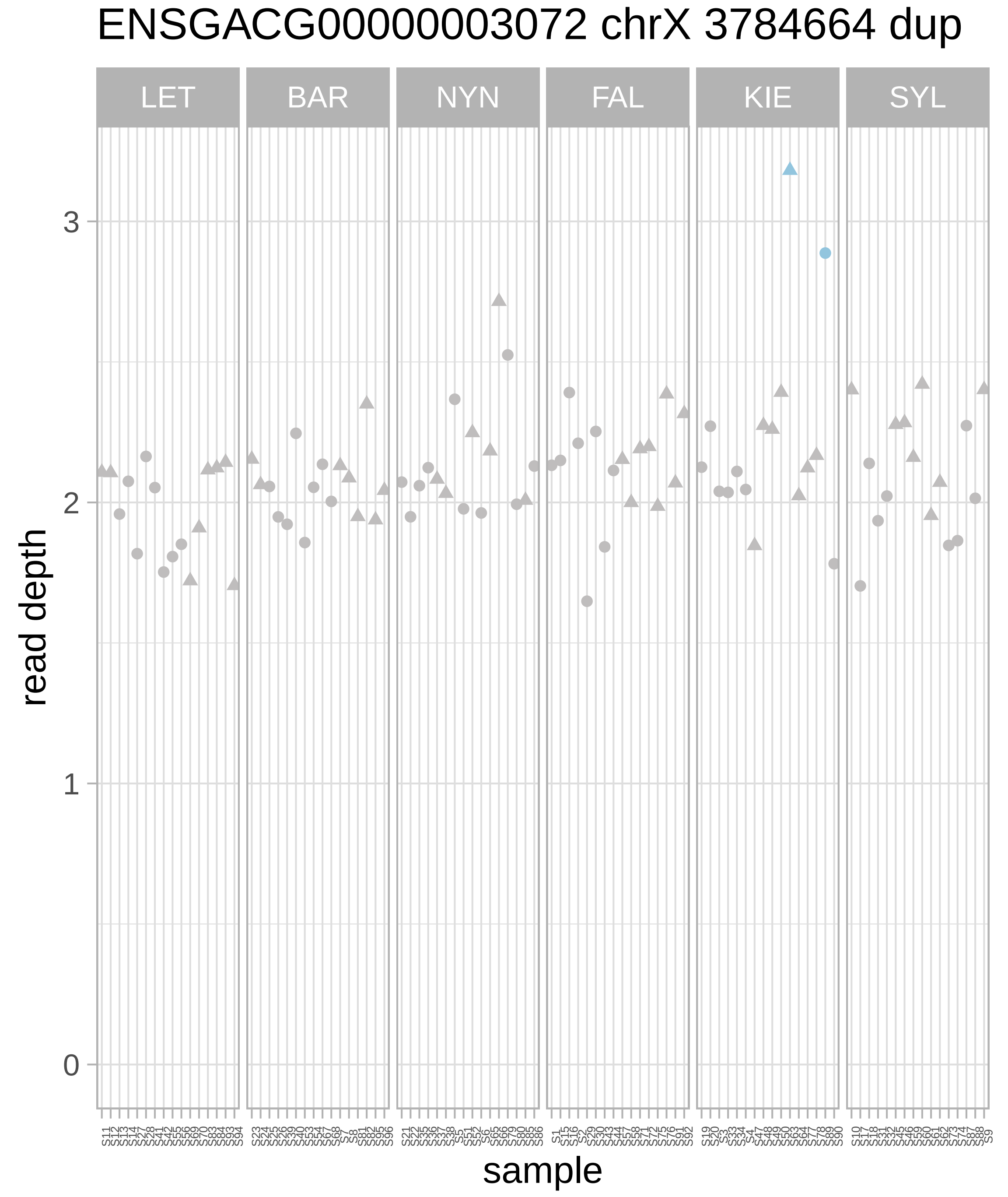
ENSGACG00000003072 chrX 3784664 dup
LET
BAR
NYN
FAL
KIE
SYL
3
2
1
0
read depth
sample

ENSGACG00000003074 chrX 3789001 dup

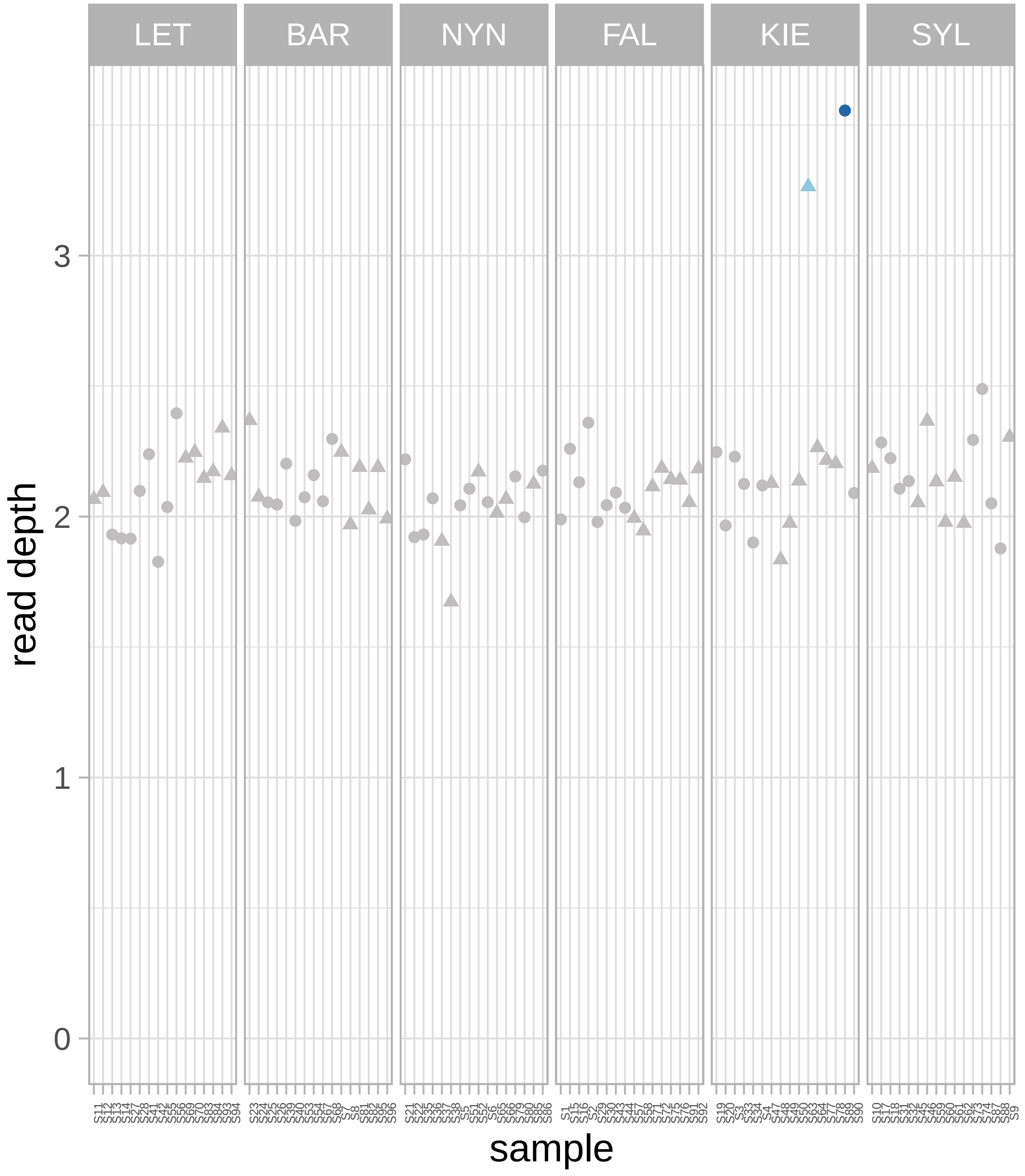

ENSGACG00000003078 chrX 3793645 dup

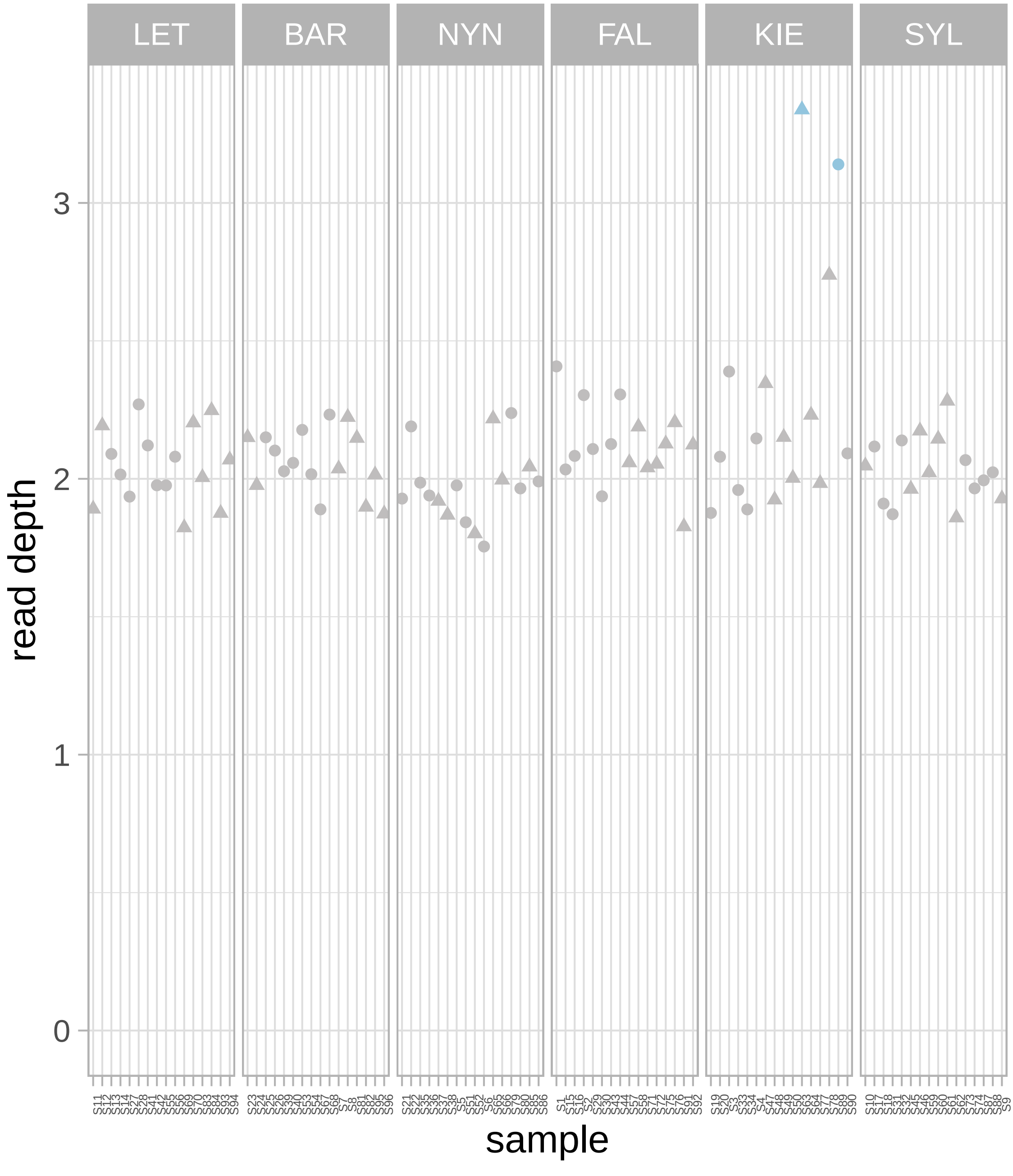

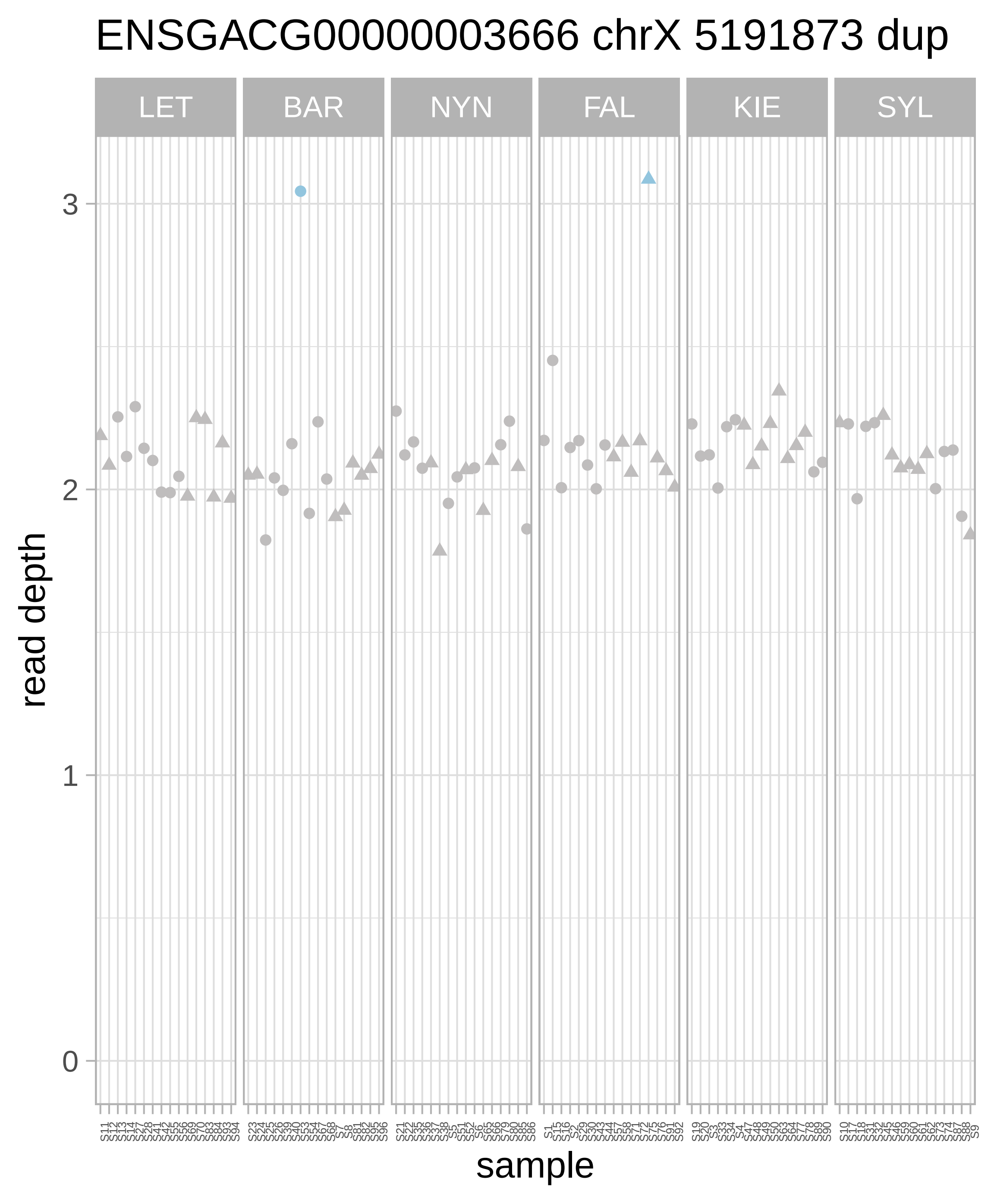

ENSGACG00000003666 chrX 5191873 dup
LET
BAR
NYN
FAL
KIE
SYL
read depth
sample
0
1
2
3

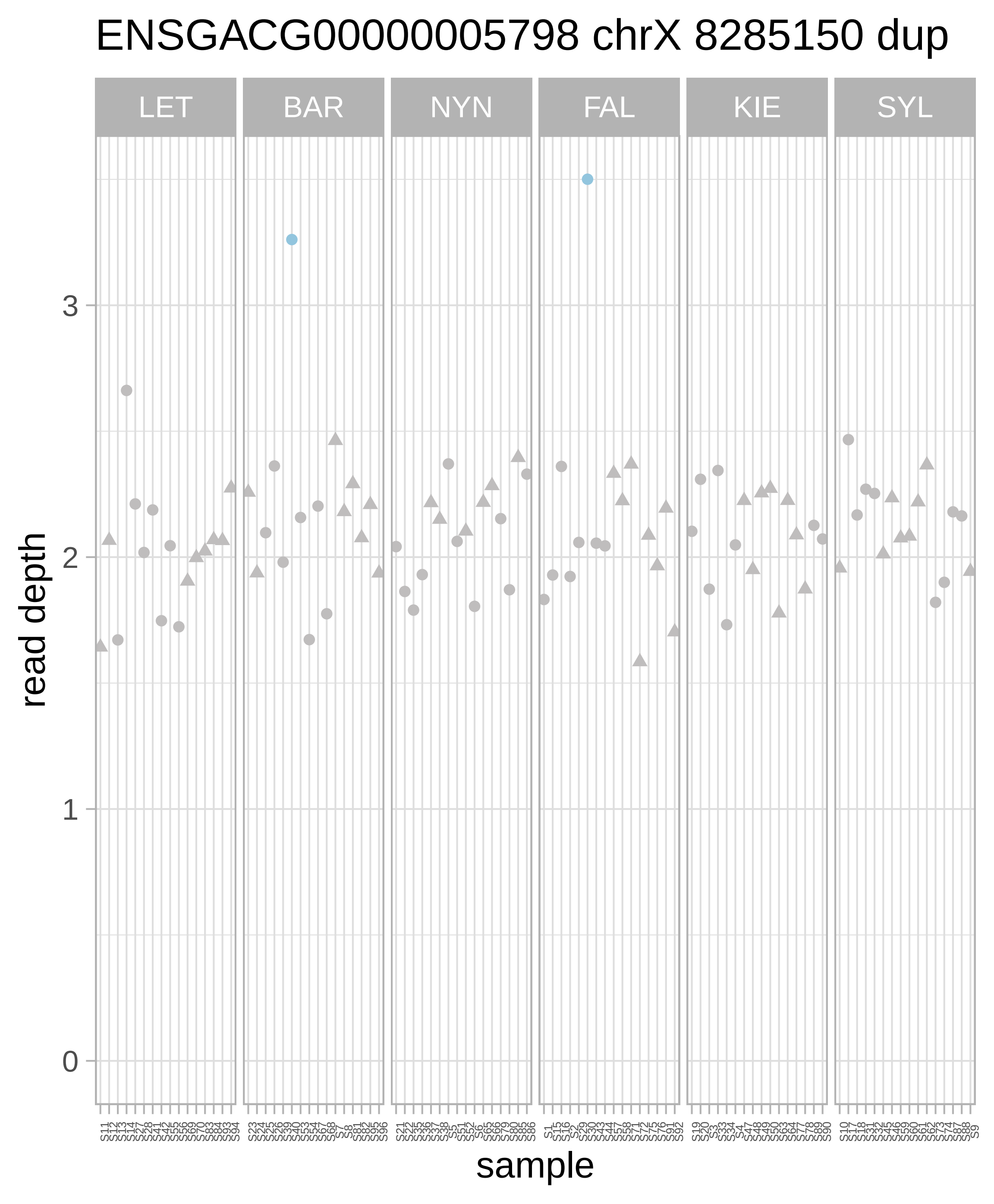
ENSGACG00000005798 chrX 8285150 dup
LET
BAR
NYN
FAL
KIE
SYL
read depth
0
1
2
3
sample

ENSGACG00000005801 chrX 8286556 dup

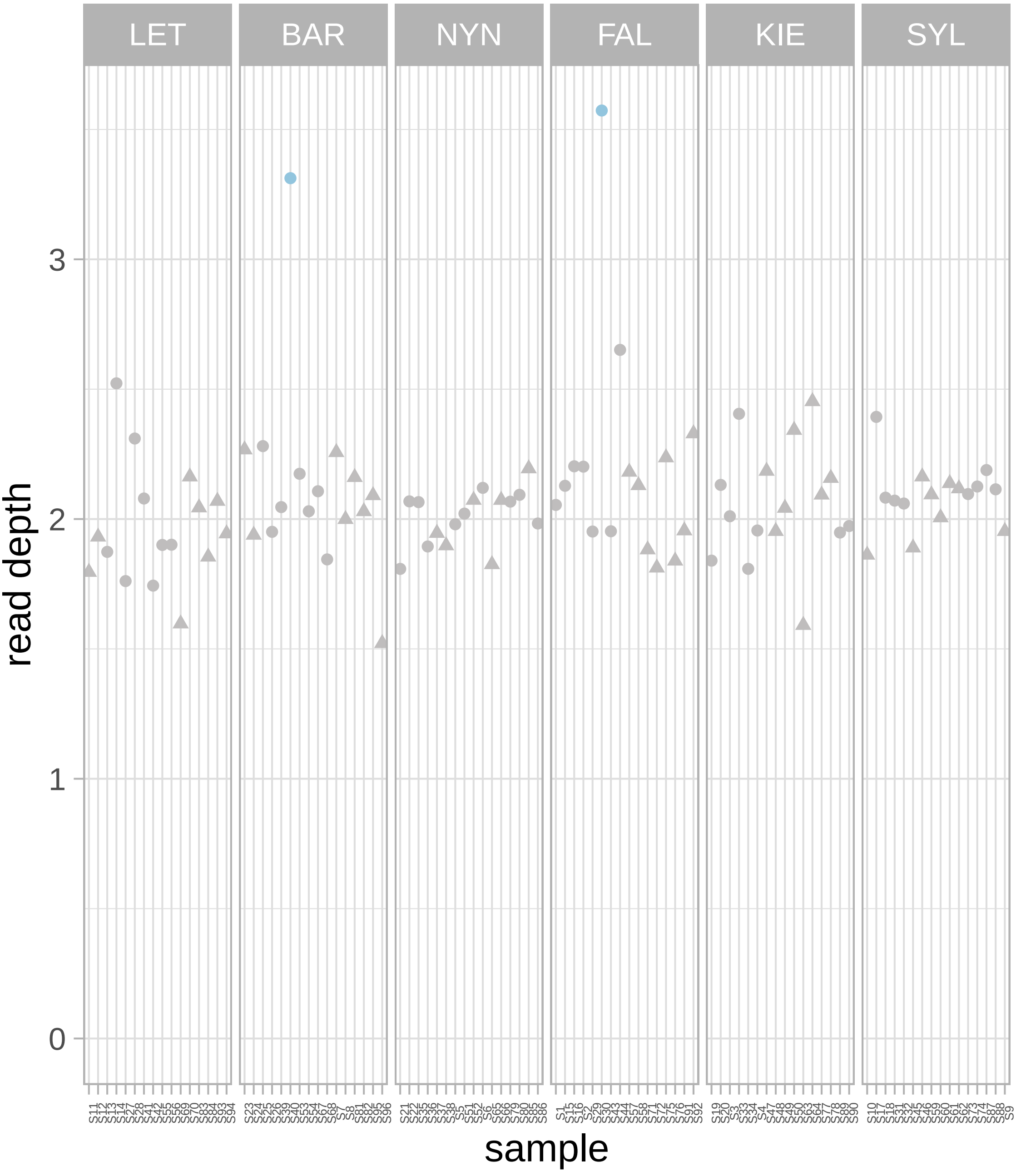

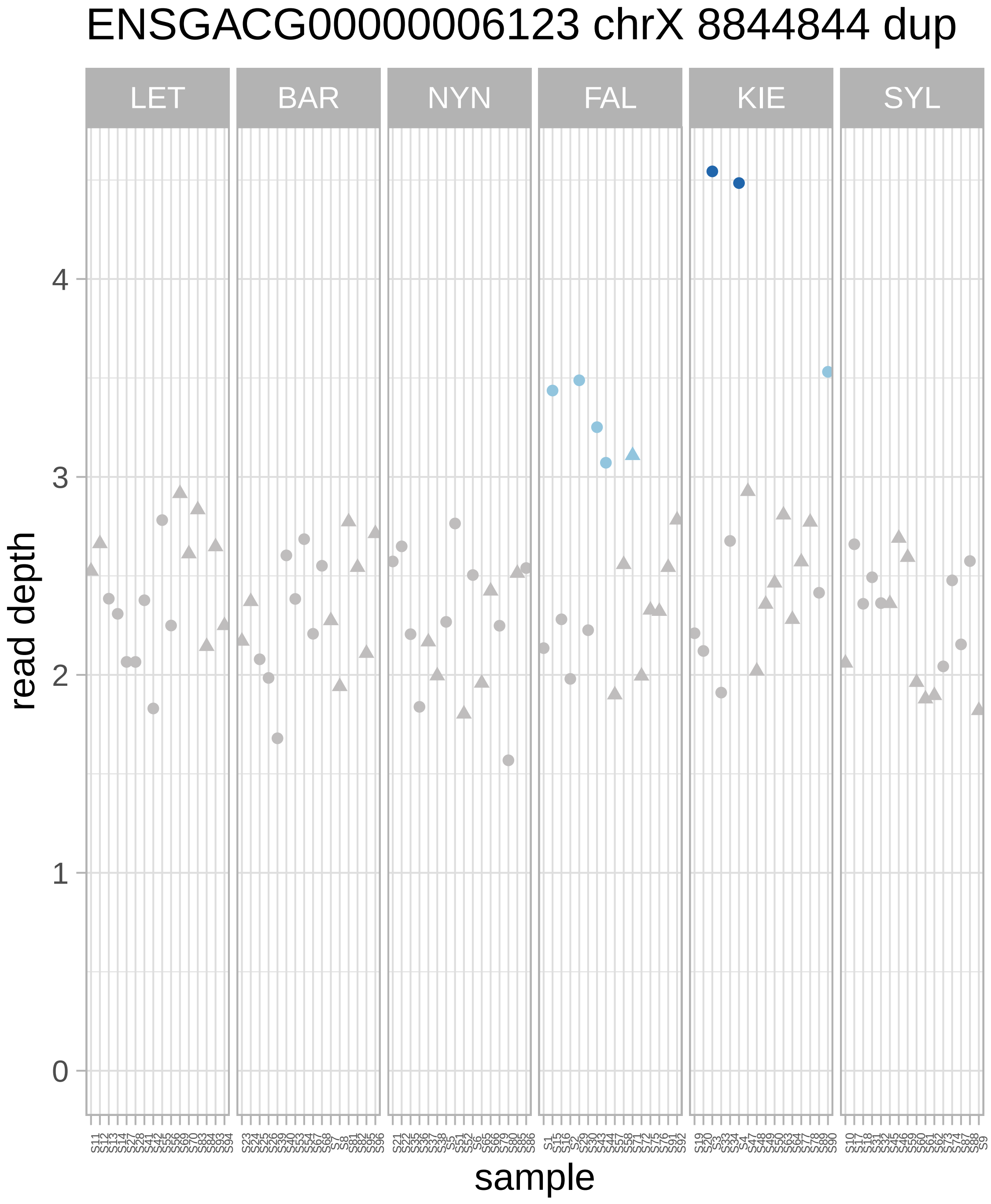
ENSGACG00000006123 chrX 8844844 dup
LET
BAR
NYN
FAL
KIE
SYL
read depth
0
1
2
3
4
sample

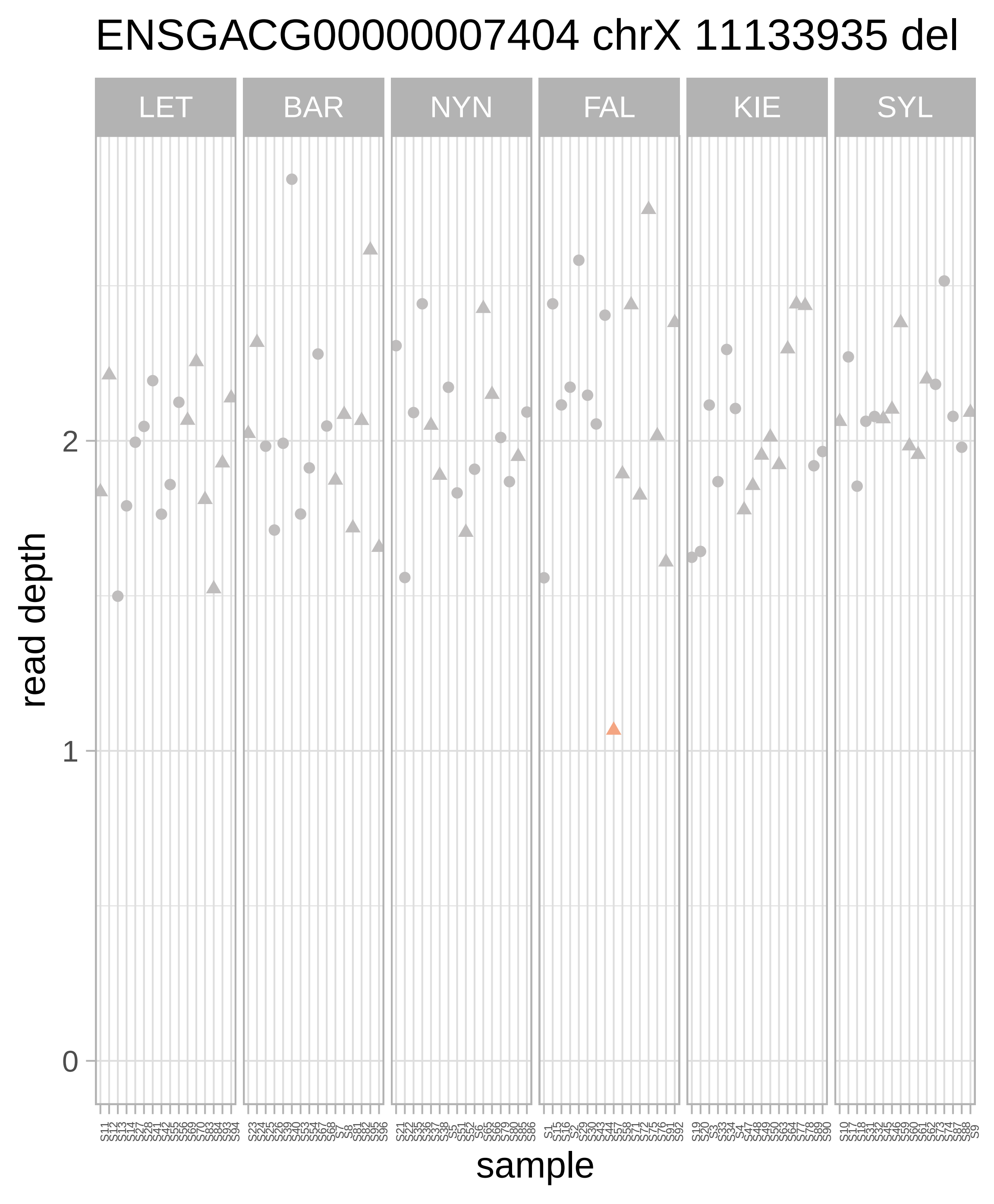
ENSGACG00000007404 chrX 11133935 del
LET
BAR
NYN
FAL
KIE
SYL
read depth
2
1
0
sample

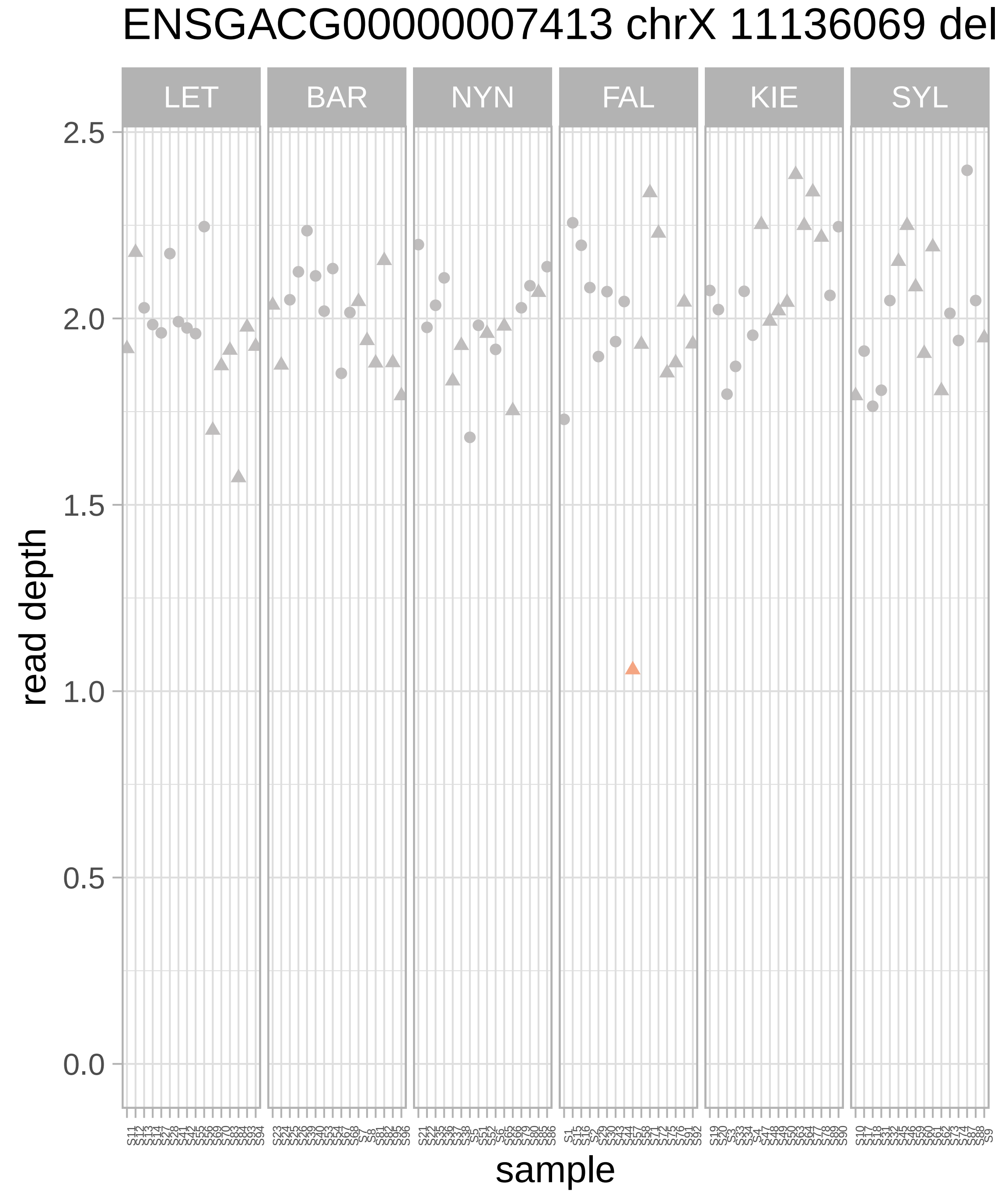
ENSGACG00000007413 chrX 11136069 del
LET
BAR
NYN
FAL
KIE
SYL
2.5
2.0
1.5
1.0
0.5
0.0
read depth
sample

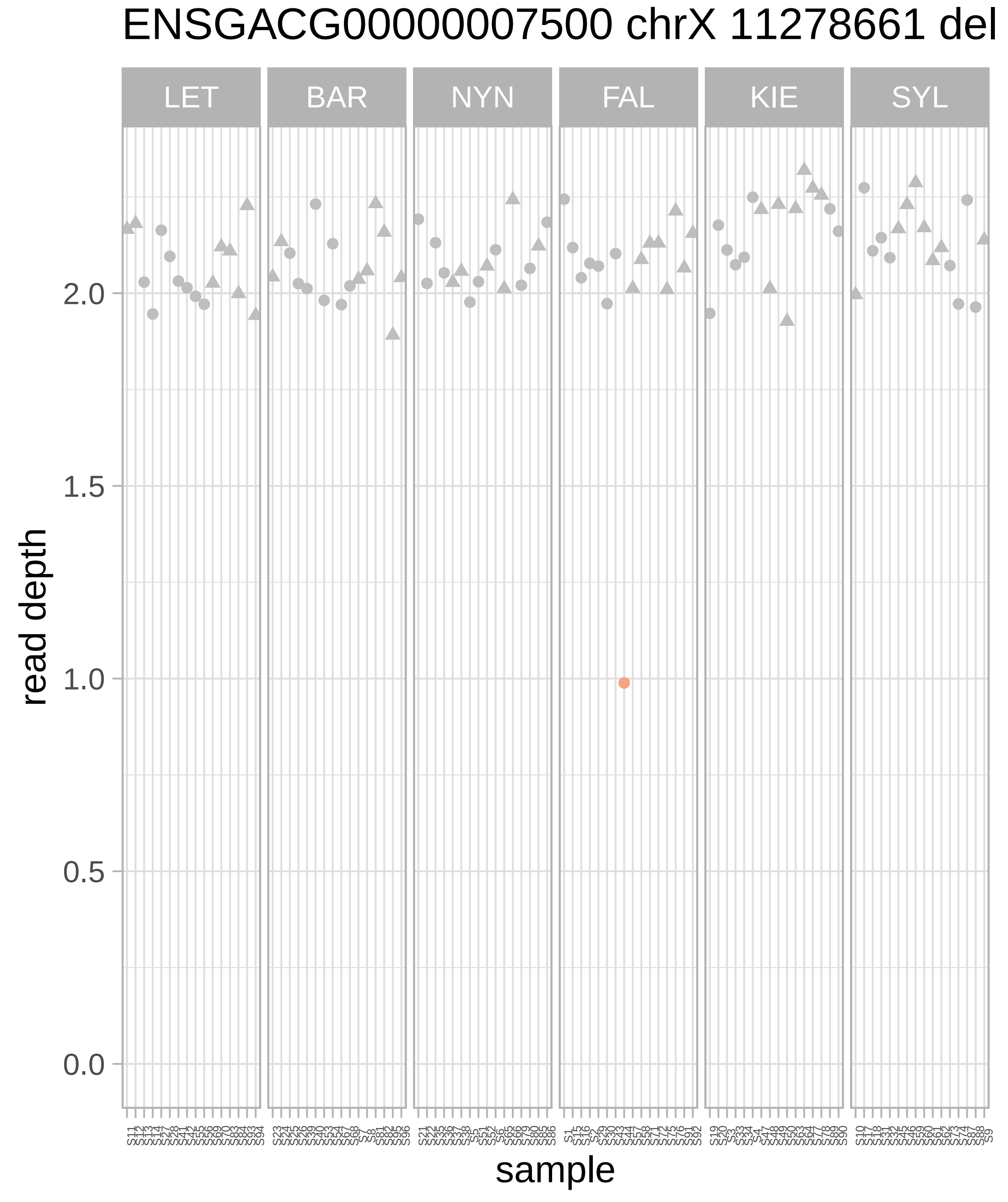
ENSGACG00000007500 chrX 11278661 del
LET
BAR
NYN
FAL
KIE
SYL
read depth
0.0
0.5
1.0
1.5
2.0
S11 S12 S13 S14 S27 S28 S41 S42 S55 S56 S69 S70 S83 S84 S93 S94
S23 S24 S25 S26 S39 S40 S53 S54 S67 S68 S7 S8 S81 S82 S95 S96
S21 S22 S35 S36 S37 S38 S5 S51 S52 S6 S65 S66 S79 S80 S85 S86
S1 S15 S16 S2 S29 S30 S43 S44 S57 S58 S71 S72 S75 S76 S91 S92
S19 S20 S3 S33 S34 S4 S47 S48 S49 S50 S63 S64 S77 S78 S89 S90
S10 S17 S18 S31 S32 S45 S46 S59 S60 S61 S62 S73 S74 S87 S88 S9
sample

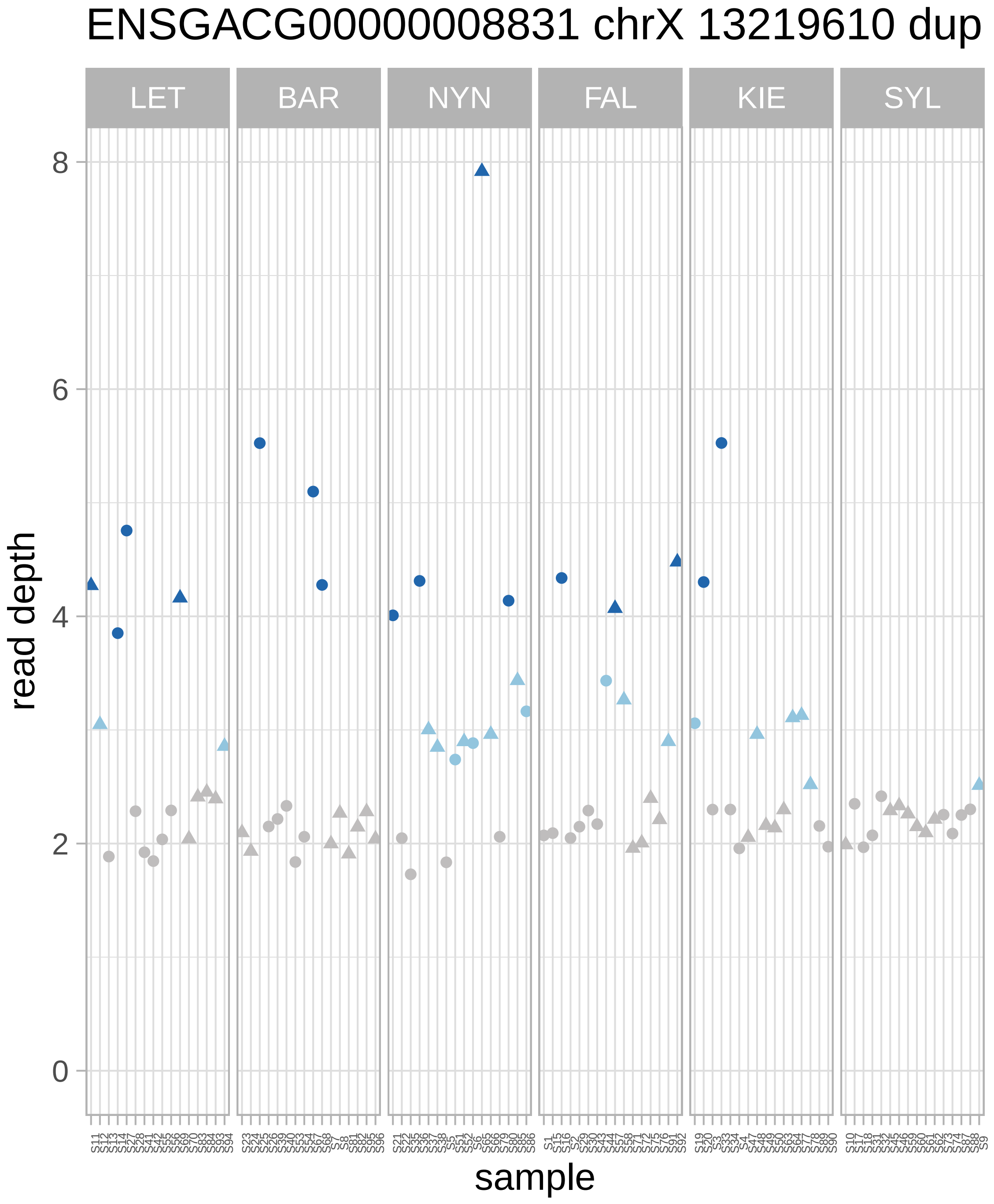
ENSGACG00000008831 chrX 13219610 dup
LET
BAR
NYN
FAL
KIE
SYL
read depth
8
6
4
2
0
sample

ENSGACG00000009240 chrX 14213304 dup

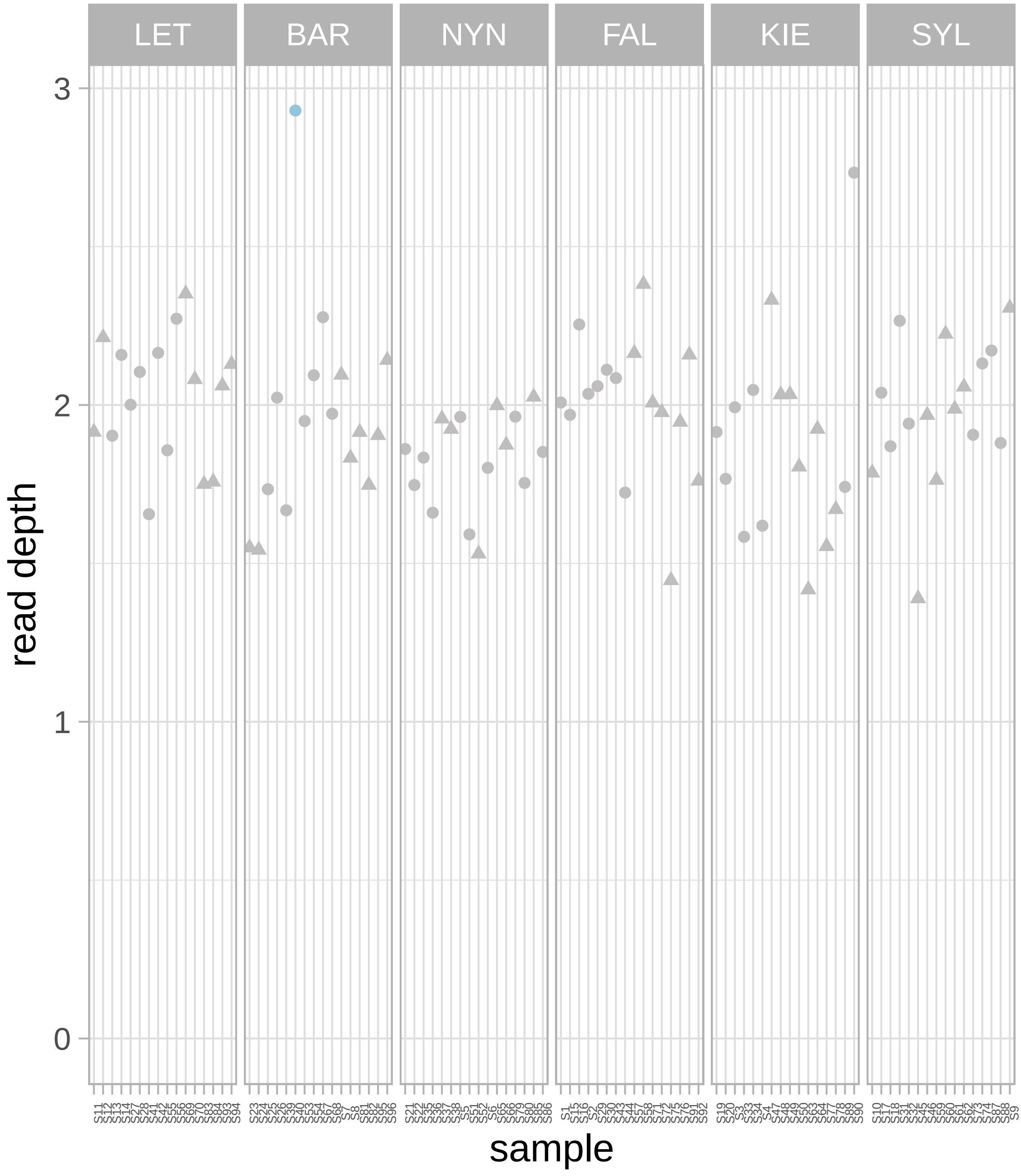

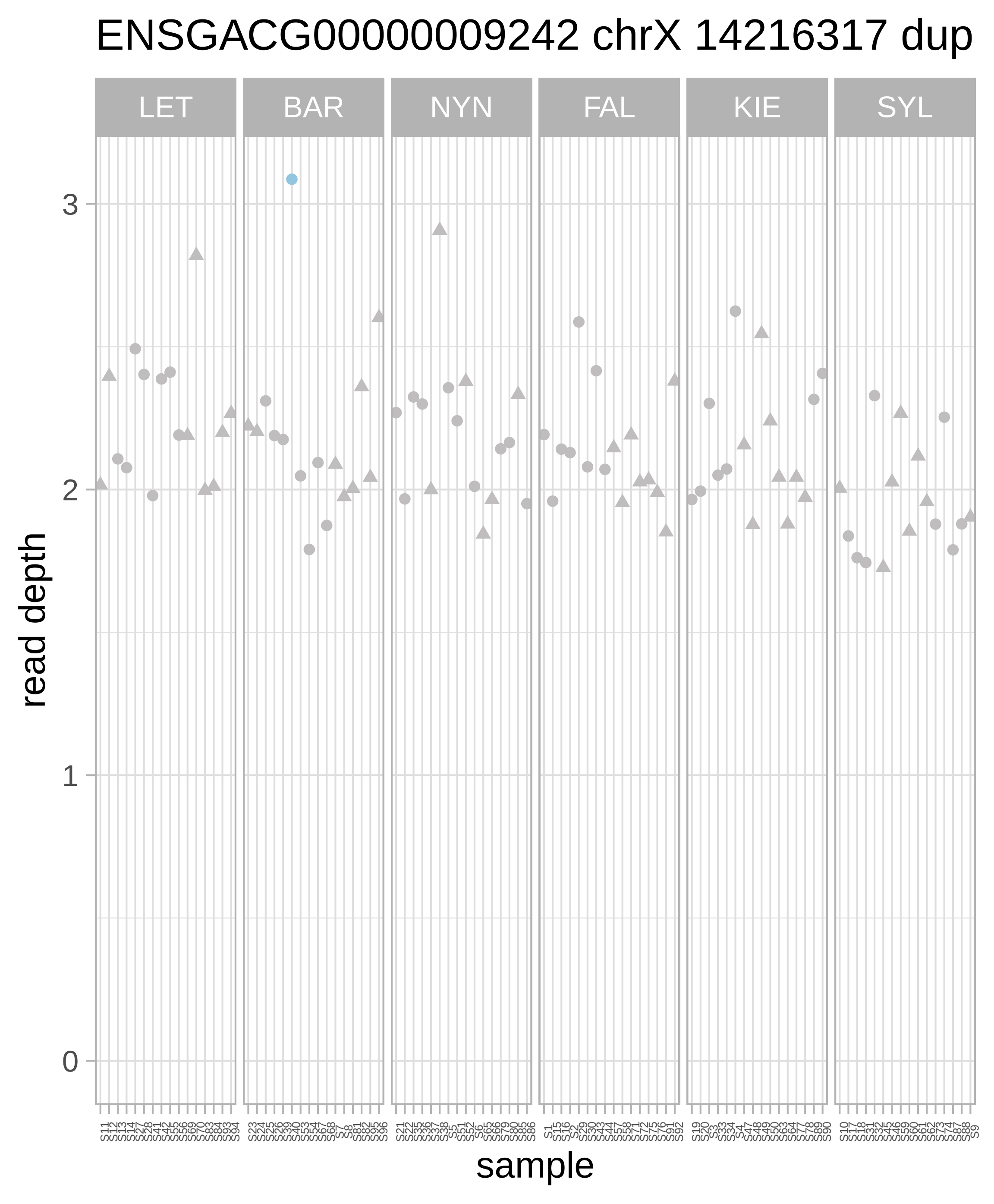
ENSGACG00000009242 chrX 14216317 dup
LET
BAR
NYN
FAL
KIE
SYL
read depth
sample
0
1
2
3

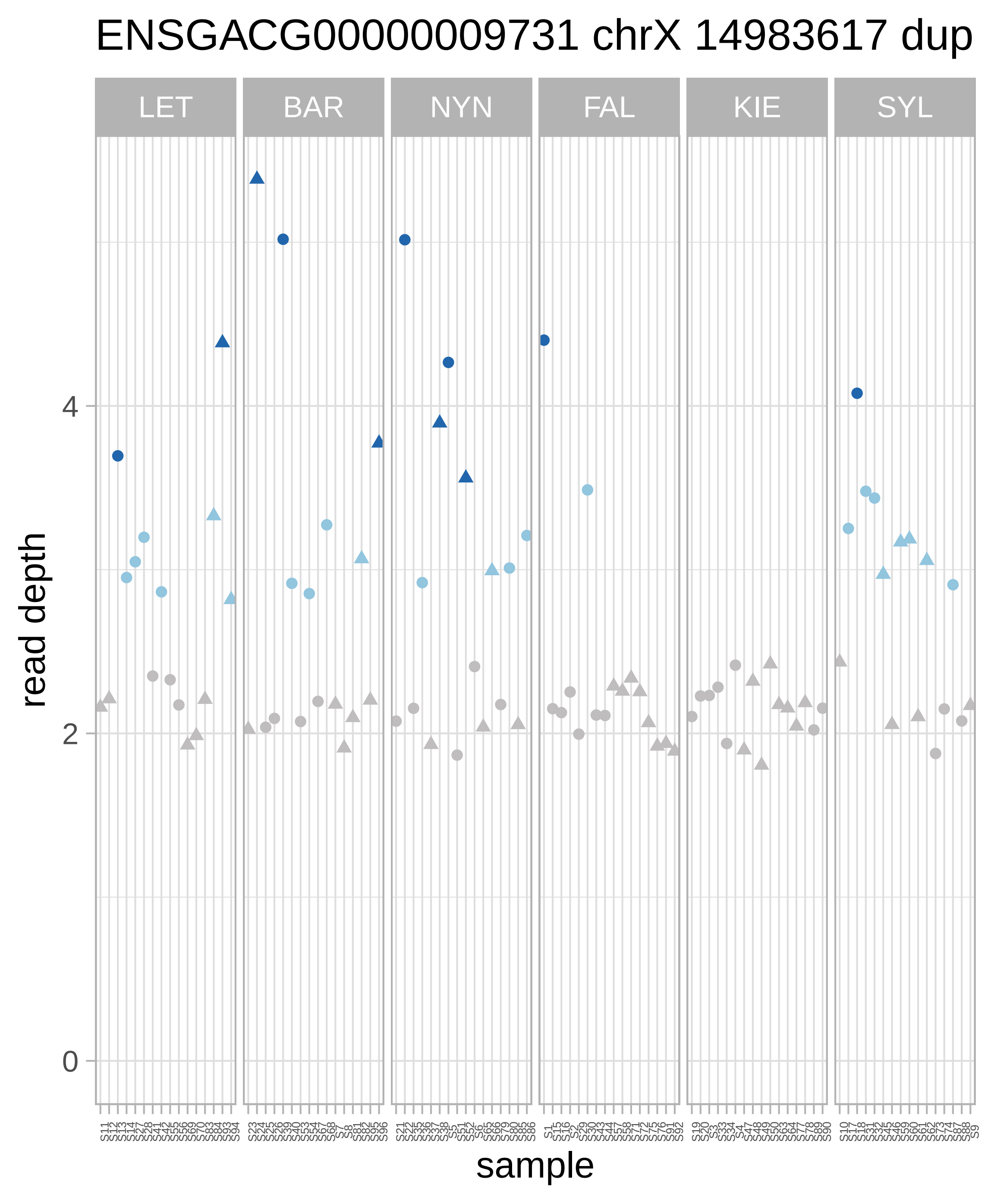
ENSGACG00000009731 chrX 14983617 dup
LET
BAR
NYN
FAL
KIE
SYL
read depth
0
2
4
sample

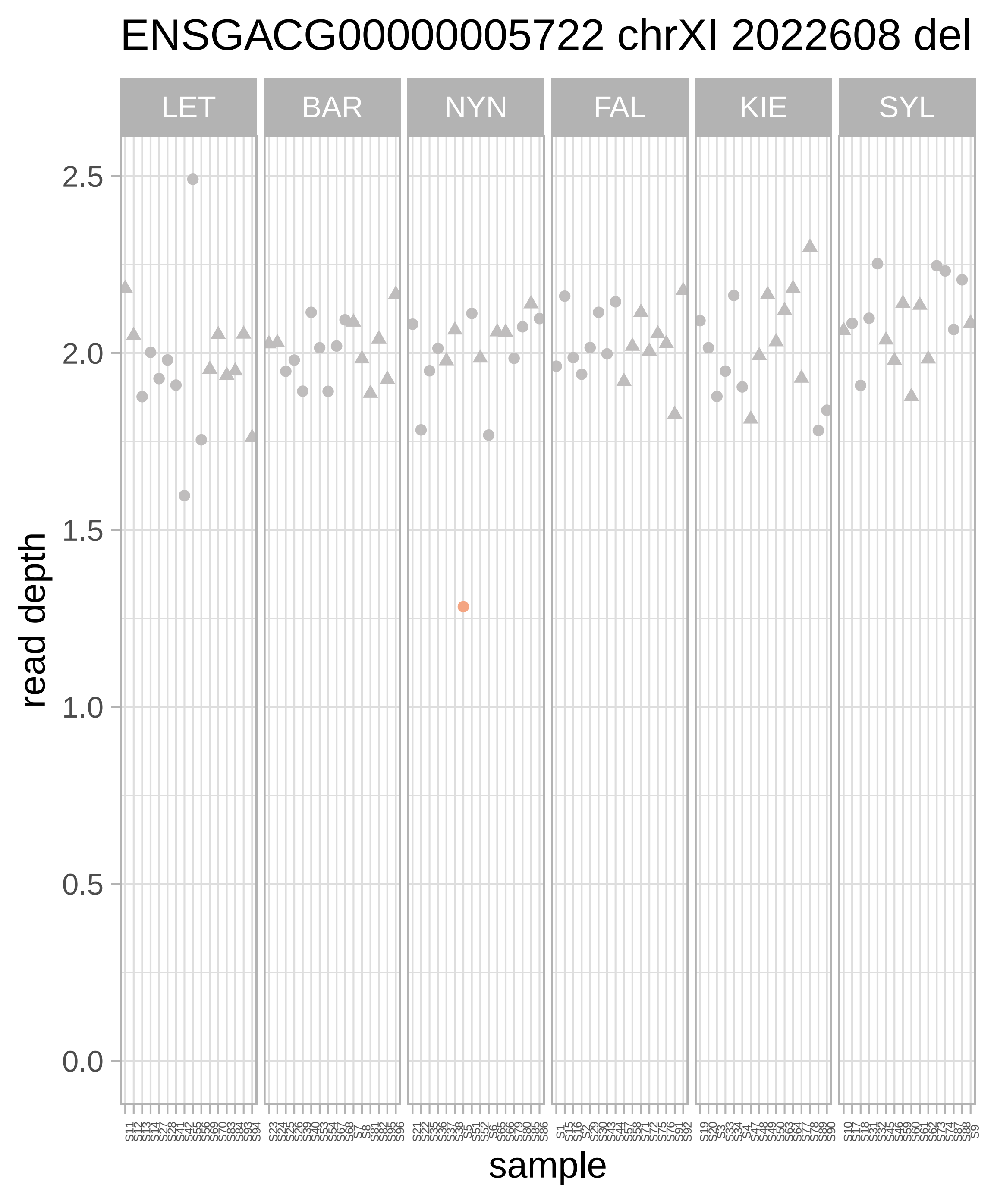
ENSGACG00000005722 chrXI 2022608 del
LET
BAR
NYN
FAL
KIE
SYL
read depth
2.5
2.0
1.5
1.0
0.5
0.0
sample

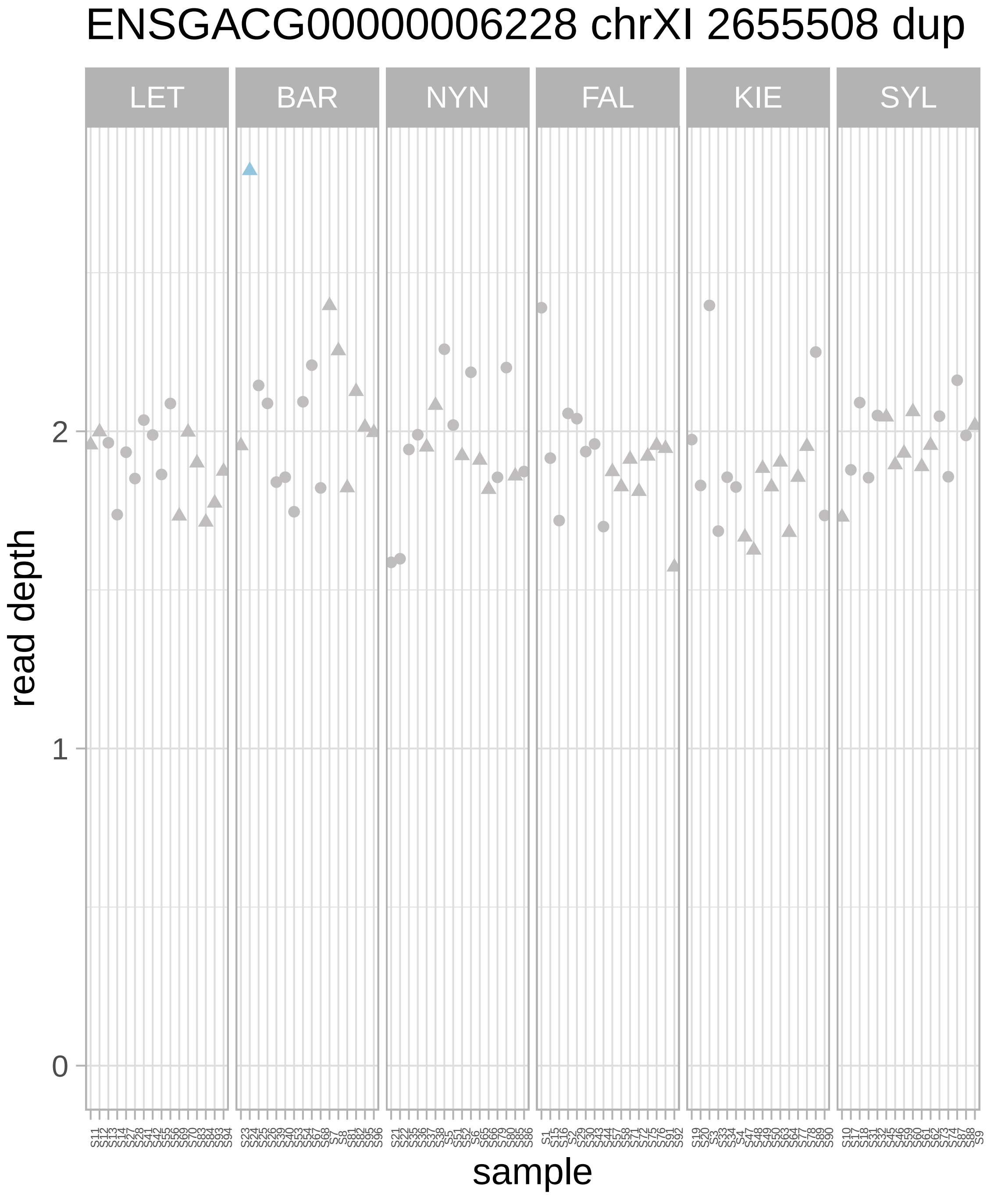
ENSGACG00000006228 chrXI 2655508 dup
LET
BAR
NYN
FAL
KIE
SYL
read depth
0
1
2
sample

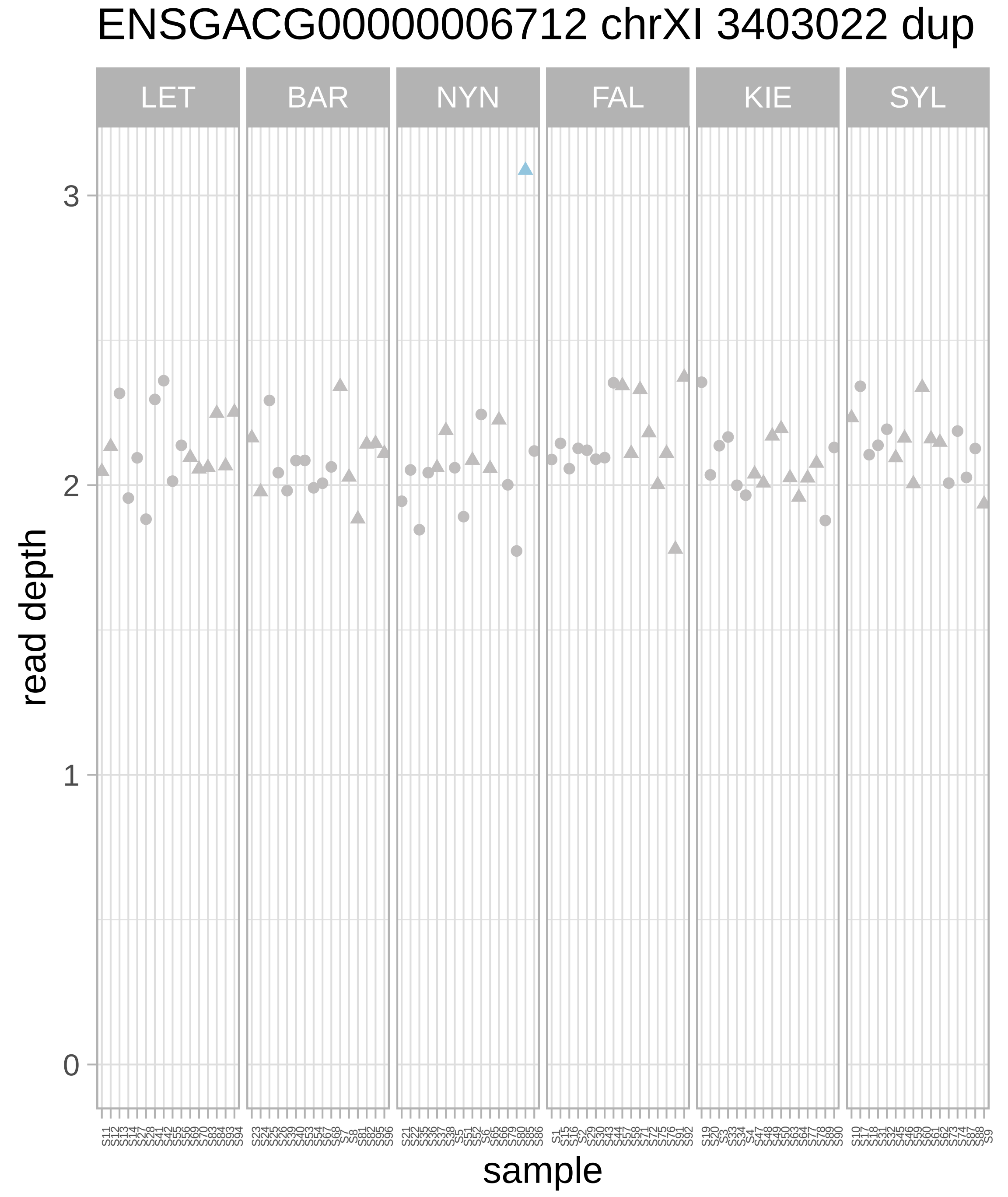

ENSGACG00000006712 chrXI 3403022 dup
LET
BAR
NYN
FAL
KIE
SYL
read depth
3
2
1
0
sample

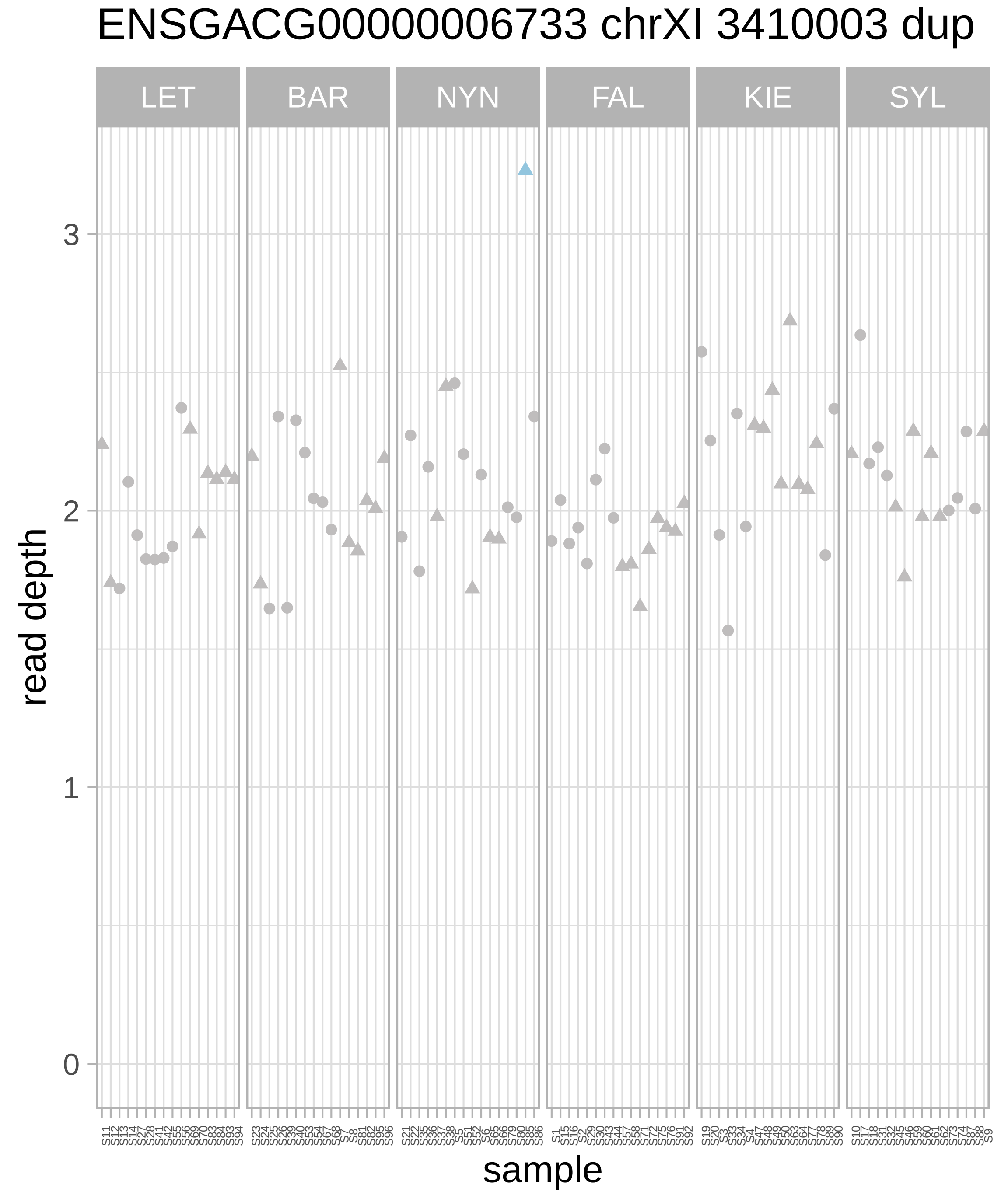
ENSGACG00000006733 chrXI 3410003 dup
LET
BAR
NYN
FAL
KIE
SYL
read depth
0
1
2
3
S11 S12 S13 S14 S27 S28 S41 S42 S55 S56 S69 S70 S83 S84 S93 S94
S23 S24 S25 S26 S39 S40 S53 S54 S67 S68 S7 S8 S81 S82 S95 S96
S21 S22 S35 S36 S37 S38 S5 S51 S52 S6 S65 S66 S79 S80 S85 S86
S1 S15 S16 S2 S29 S30 S43 S44 S57 S58 S71 S72 S75 S76 S91 S92
S19 S20 S3 S33 S34 S4 S47 S48 S49 S50 S63 S64 S77 S78 S89 S90
S10 S17 S18 S31 S32 S45 S46 S59 S60 S61 S62 S73 S74 S87 S88 S9
sample

ENSGACG00000007026 chrXI 3743298 dup

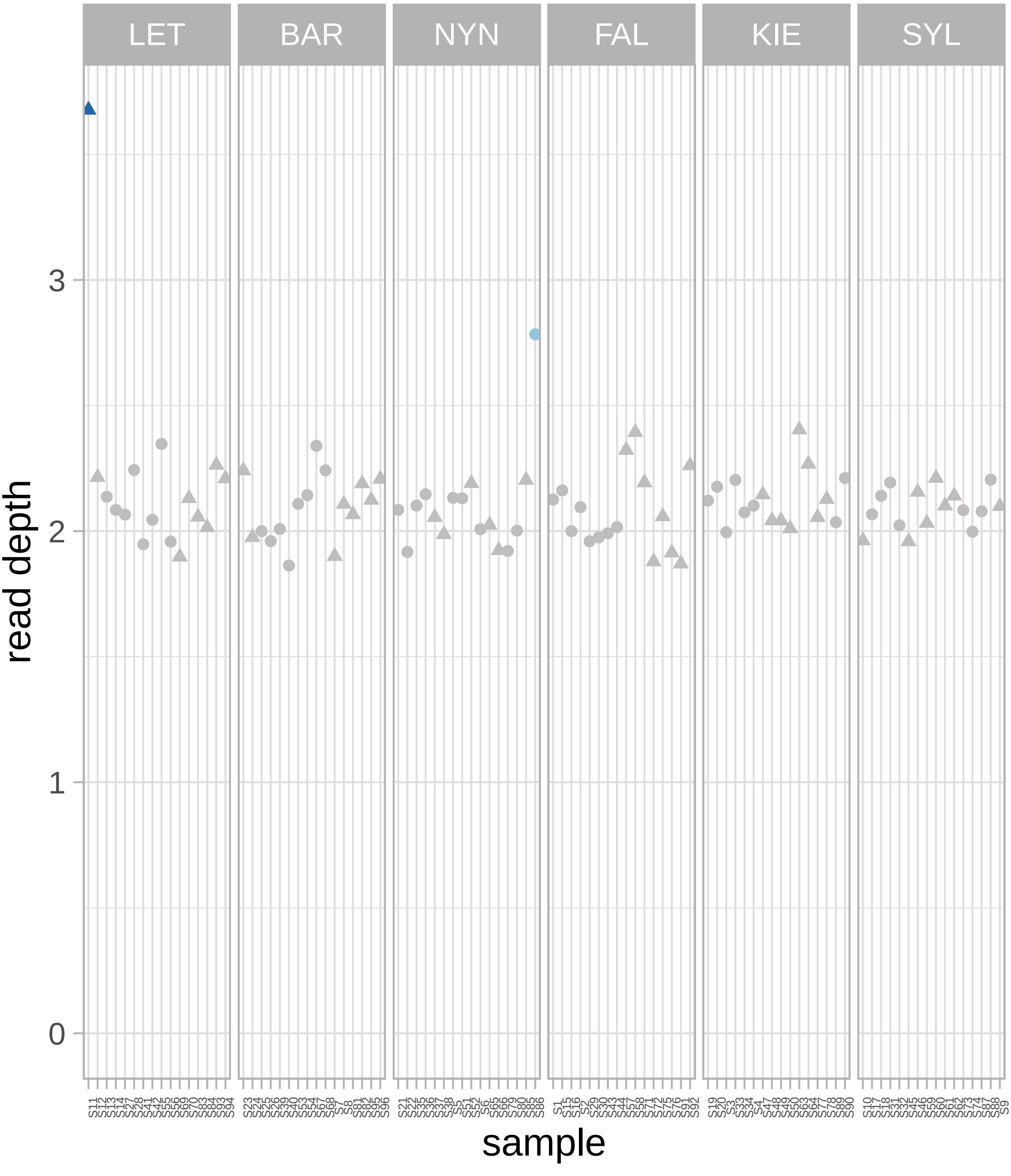

ENSGACG00000008904 chrXI 6106840 dup

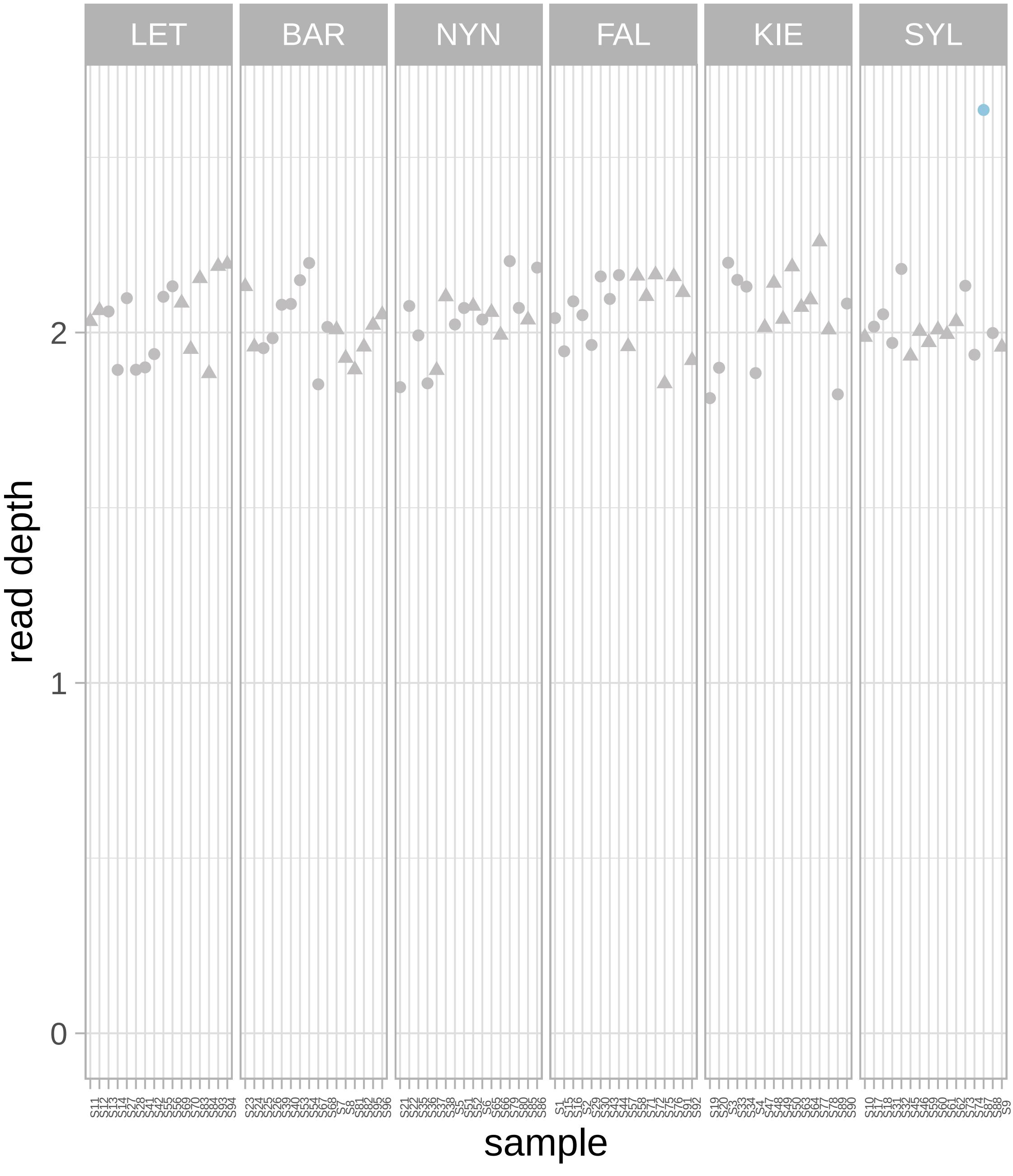

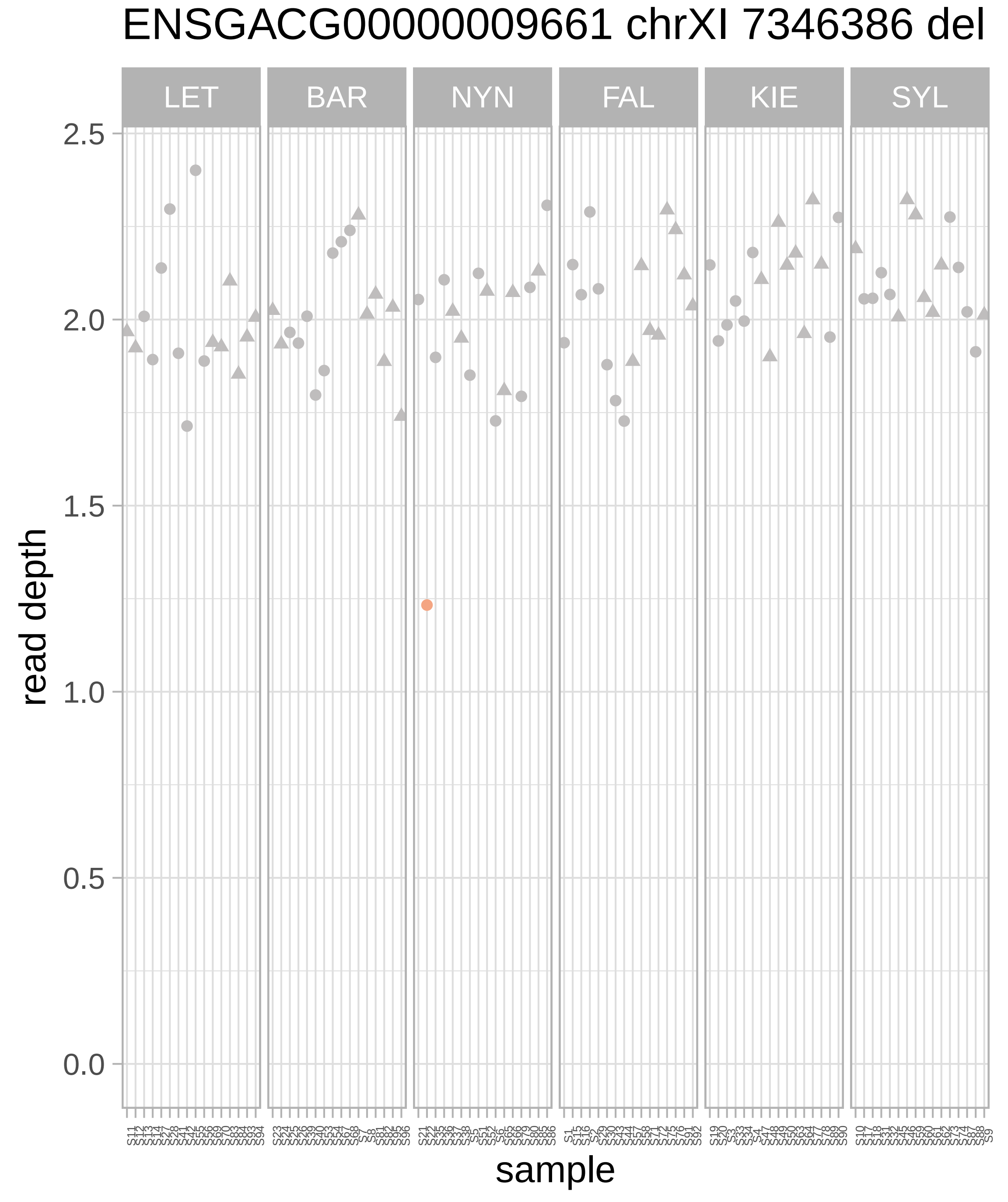
ENSGACG00000009661 chrXI 7346386 del
LET
BAR
NYN
FAL
KIE
SYL
read depth
2.5
2.0
1.5
1.0
0.5
0.0
S11 S12 S13 S14 S27 S28 S41 S42 S55 S56 S69 S70 S83 S84 S93 S94
S23 S24 S25 S26 S39 S40 S53 S54 S67 S68 S7 S8 S81 S82 S95 S96
S21 S22 S35 S36 S37 S38 S5 S51 S52 S6 S65 S66 S79 S80 S85 S86
S1 S15 S16 S2 S29 S30 S43 S44 S57 S58 S71 S72 S75 S76 S91 S92
S19 S20 S3 S33 S34 S4 S47 S48 S49 S50 S63 S64 S77 S78 S89 S90
S10 S17 S18 S31 S32 S45 S46 S59 S60 S61 S62 S73 S74 S87 S88 S9
sample

ENSGACG00000010948 chrXI 8890779 dup

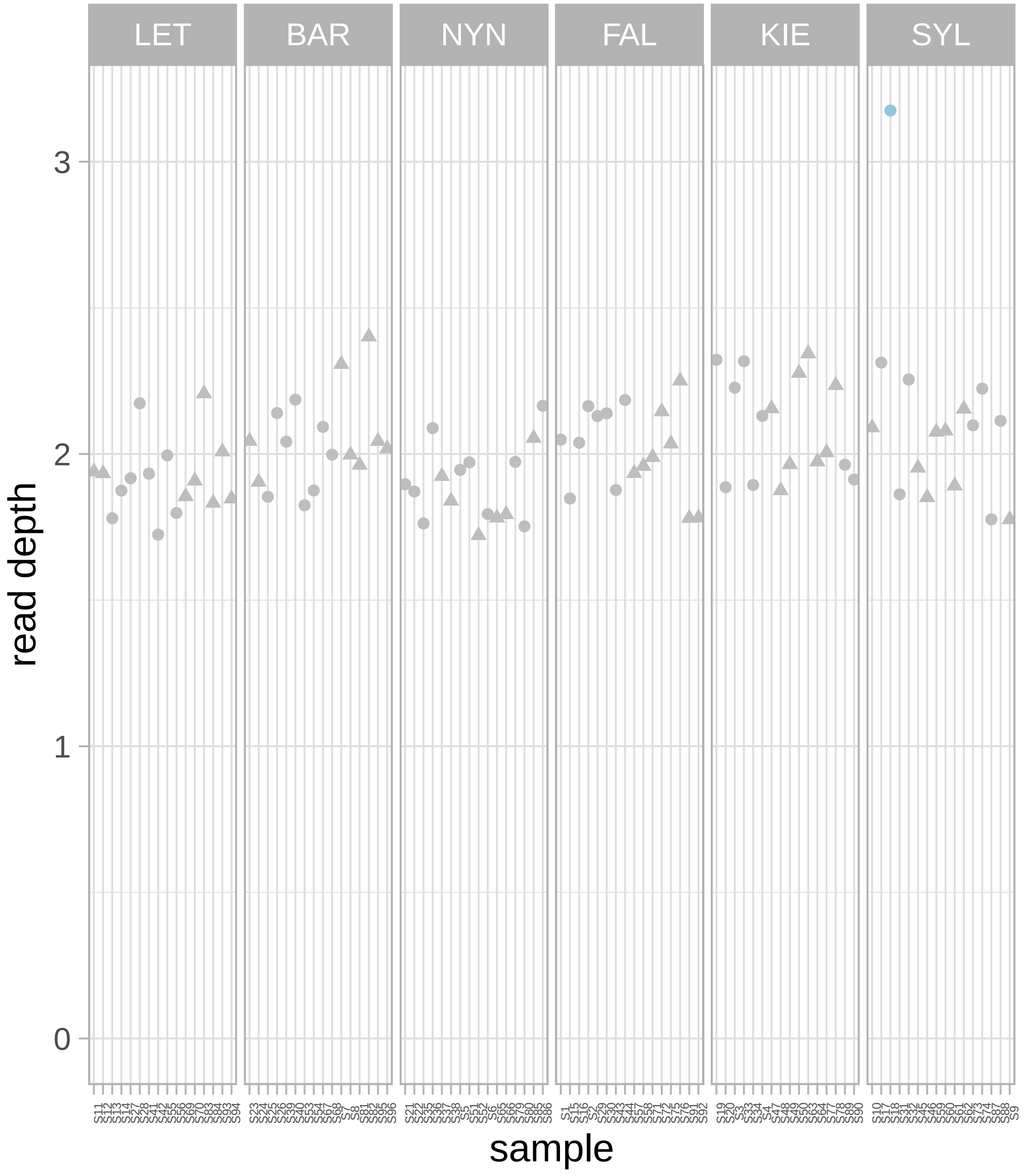

ENSGACG00000010955 chrXI 8896393 dup

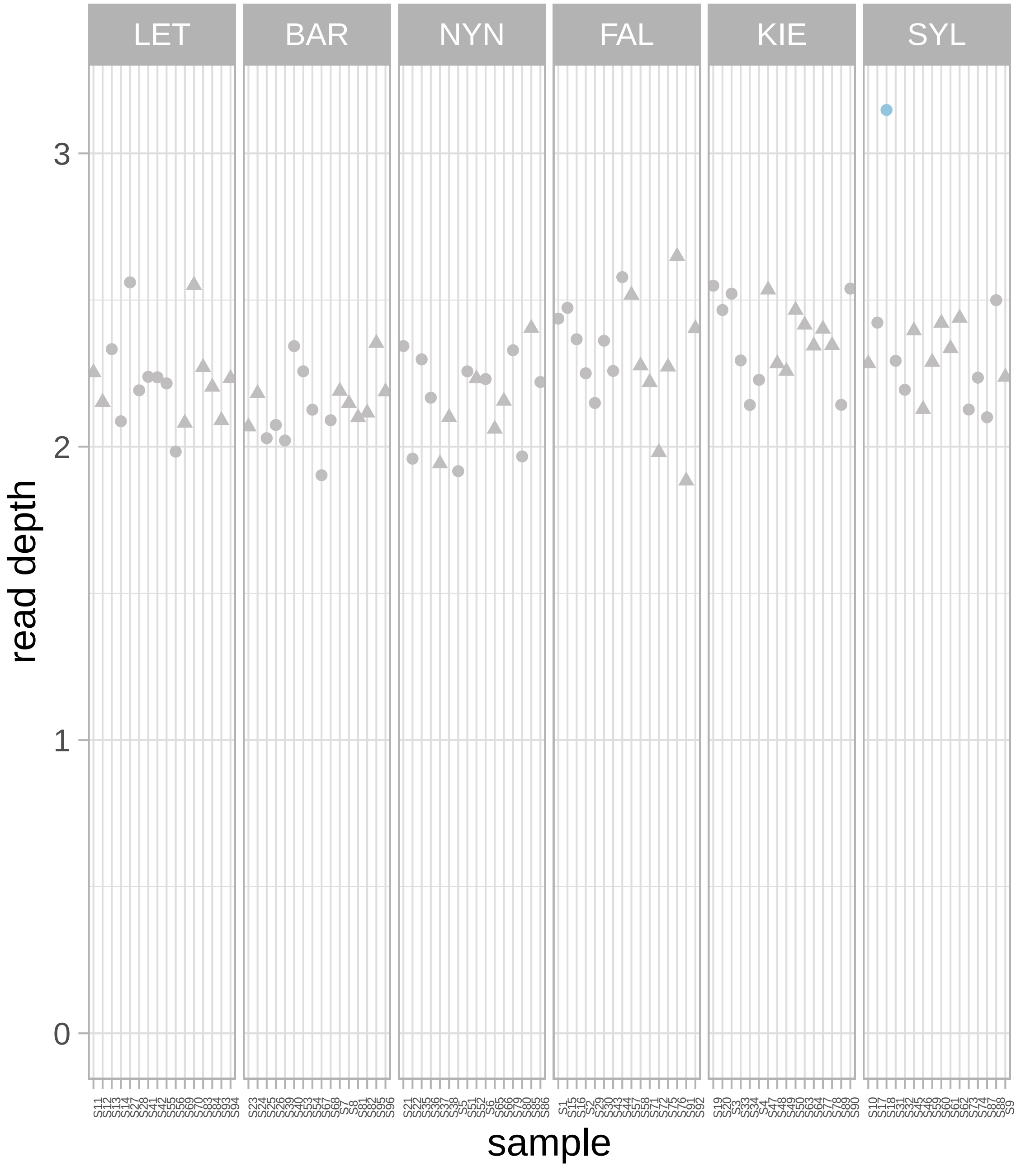

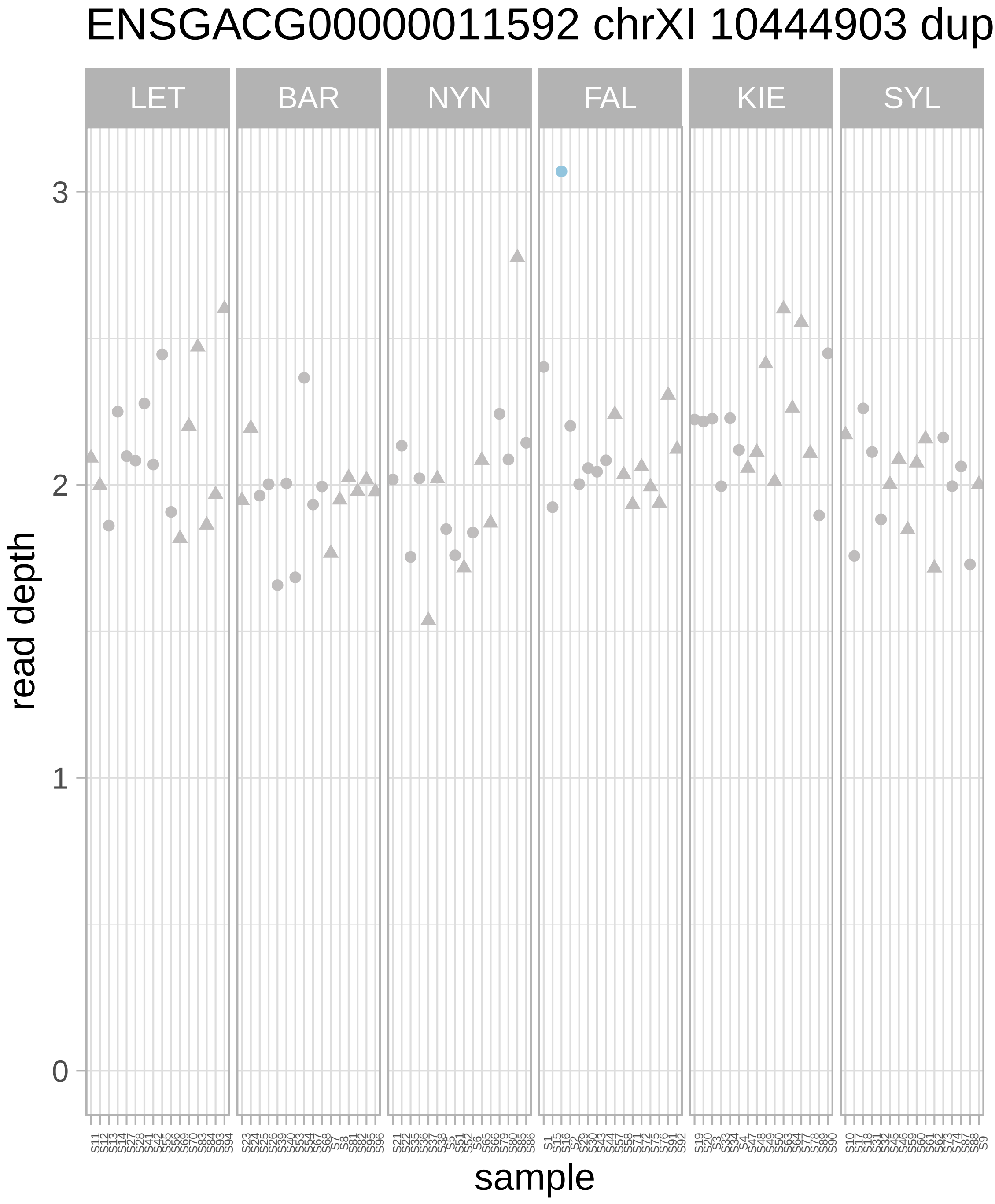
ENSGACG00000011592 chrXI 10444903 dup
LET
BAR
NYN
FAL
KIE
SYL
read depth
3
2
1
0
S11 S12 S13 S14 S27 S28 S41 S42 S55 S56 S69 S70 S83 S84 S93 S94
S23 S24 S25 S26 S39 S40 S53 S54 S67 S68 S7 S8 S81 S82 S95 S96
S21 S22 S35 S36 S37 S38 S5 S51 S52 S6 S65 S66 S79 S80 S85 S86
S1 S15 S16 S2 S29 S30 S43 S44 S57 S58 S71 S72 S75 S76 S91 S92
S19 S20 S3 S33 S34 S4 S47 S48 S49 S50 S63 S64 S77 S78 S89 S90
S10 S17 S18 S31 S32 S45 S46 S59 S60 S61 S62 S73 S74 S87 S88 S9
sample

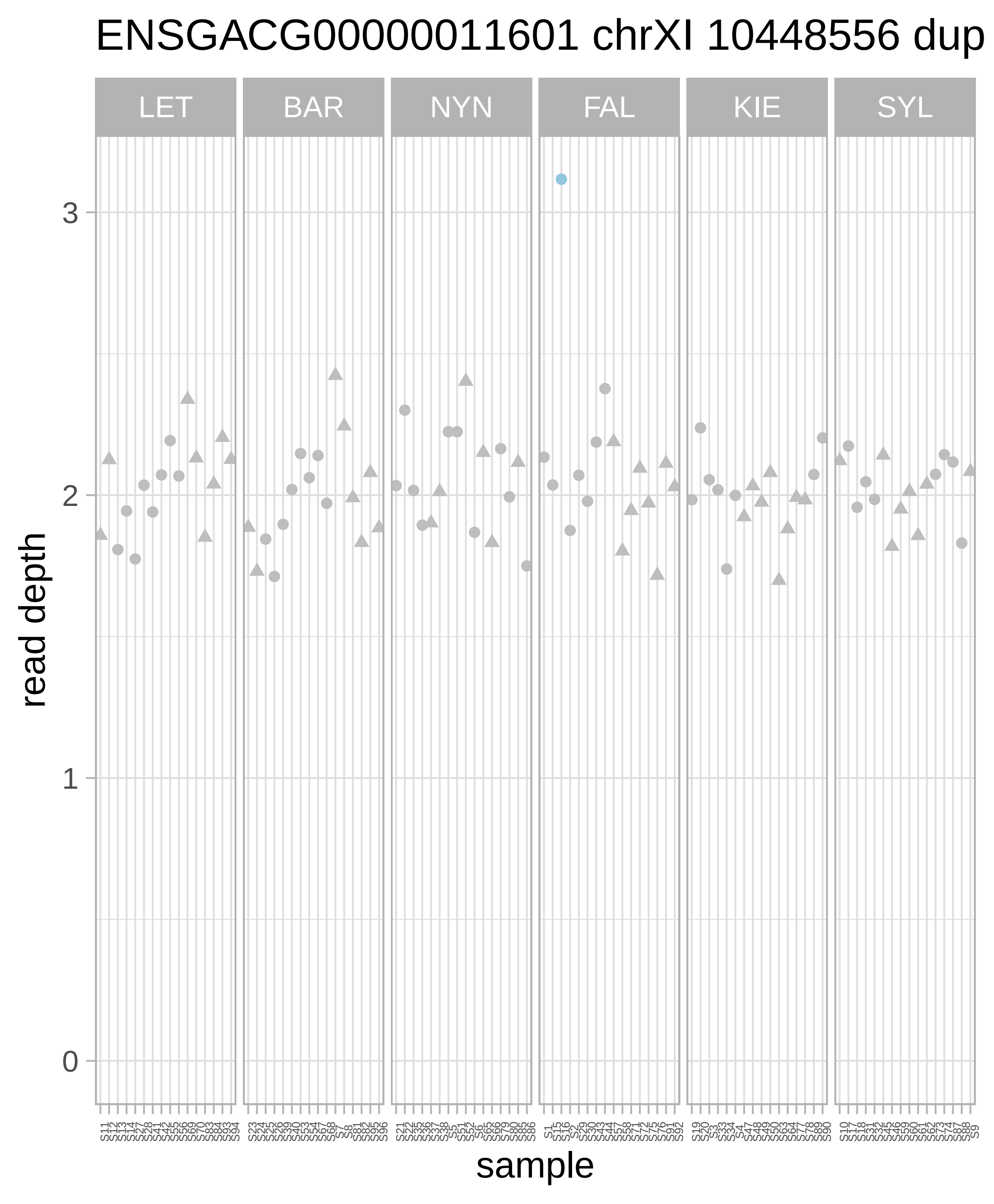
ENSGACG00000011601 chrXI 10448556 dup
LET
BAR
NYN
FAL
KIE
SYL
read depth
0
1
2
3
sample

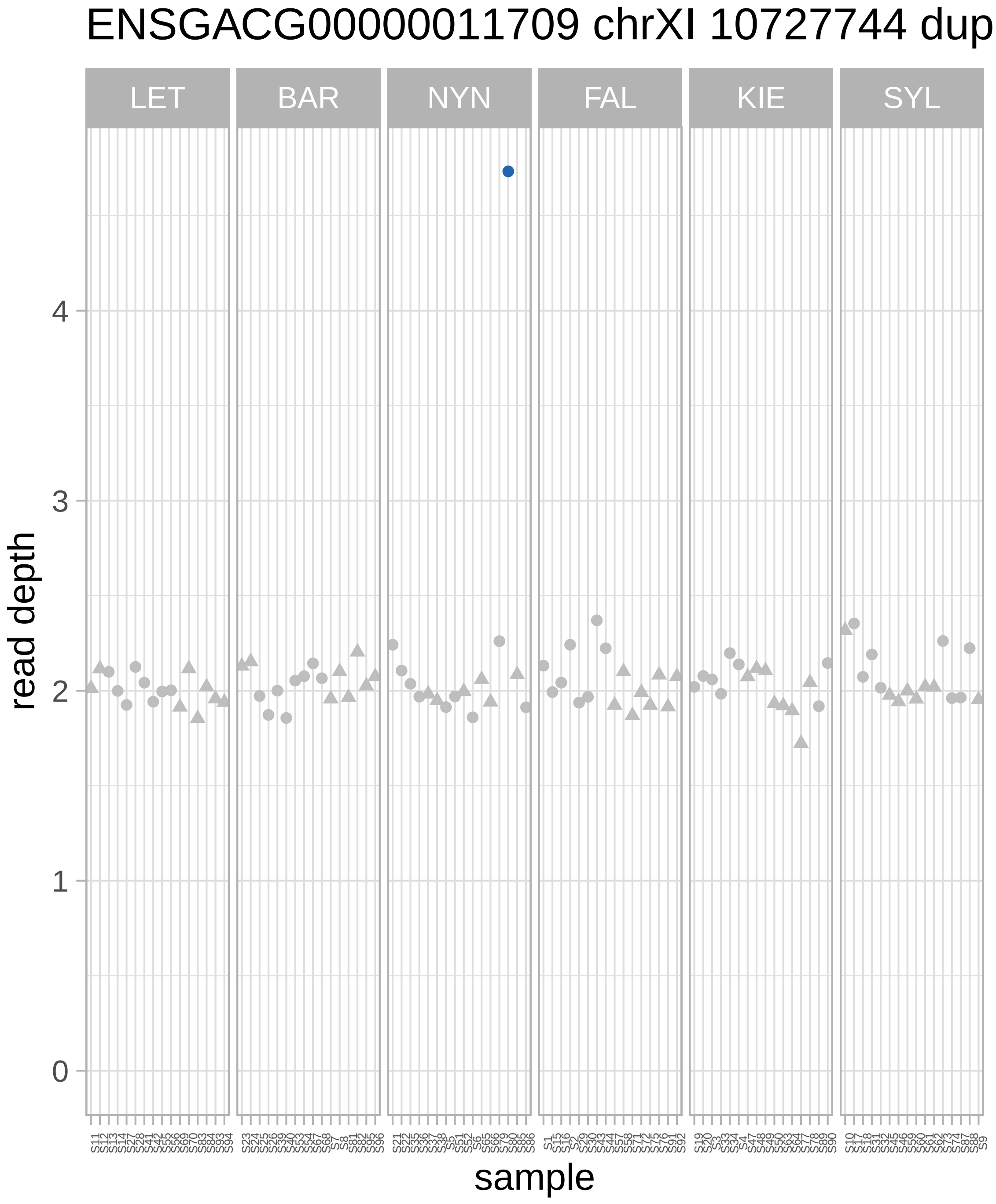
ENSGACG00000011709 chrXI 10727744 dup
LET
BAR
NYN
FAL
KIE
SYL
read depth
0
1
2
3
4
sample

ENSGACG00000012896 chrXI 12567979 dup

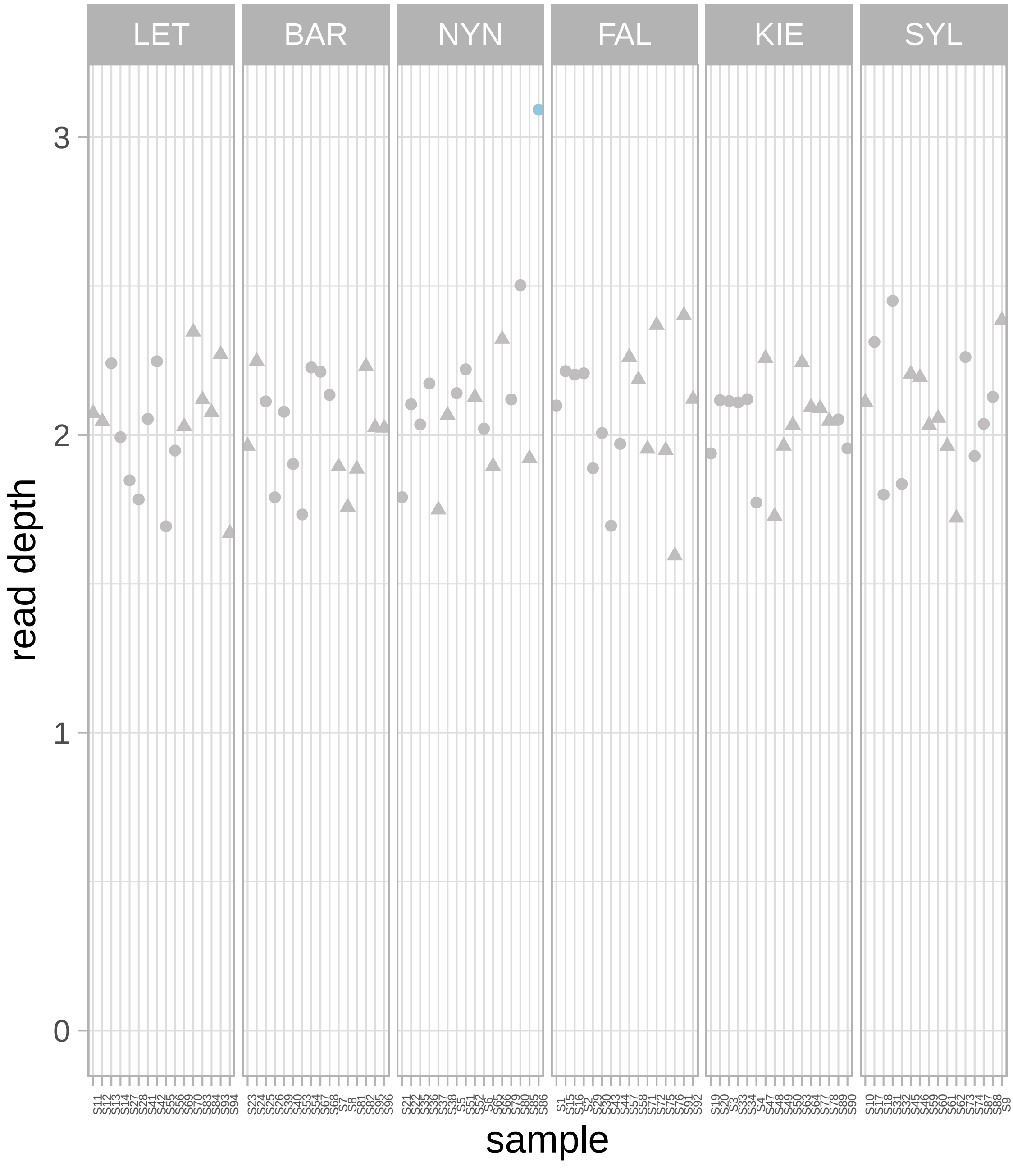

ENSGACG00000012911 chrXI 12570915 dup

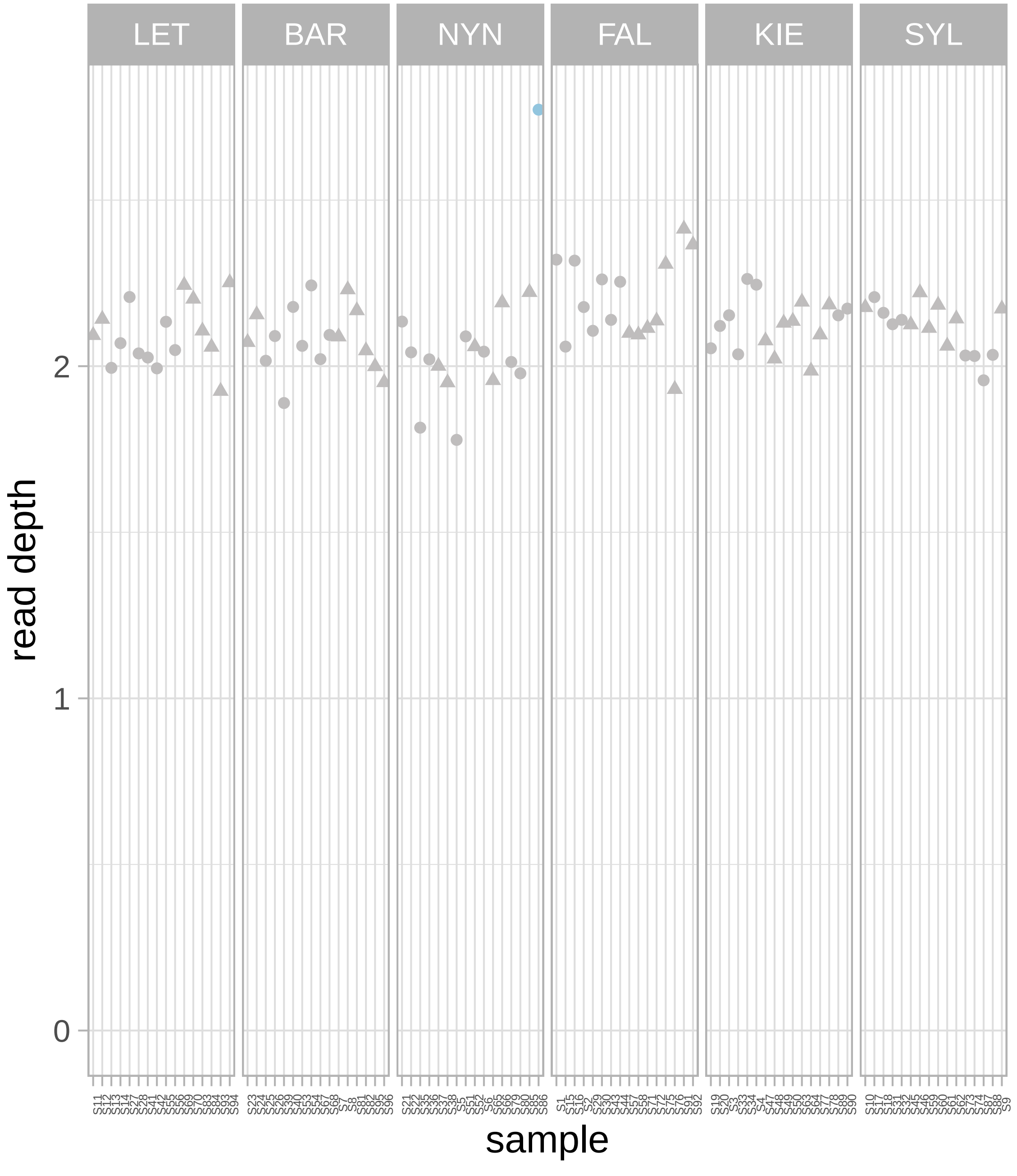

ENSGACG00000013462 chrXI 13050298 dup

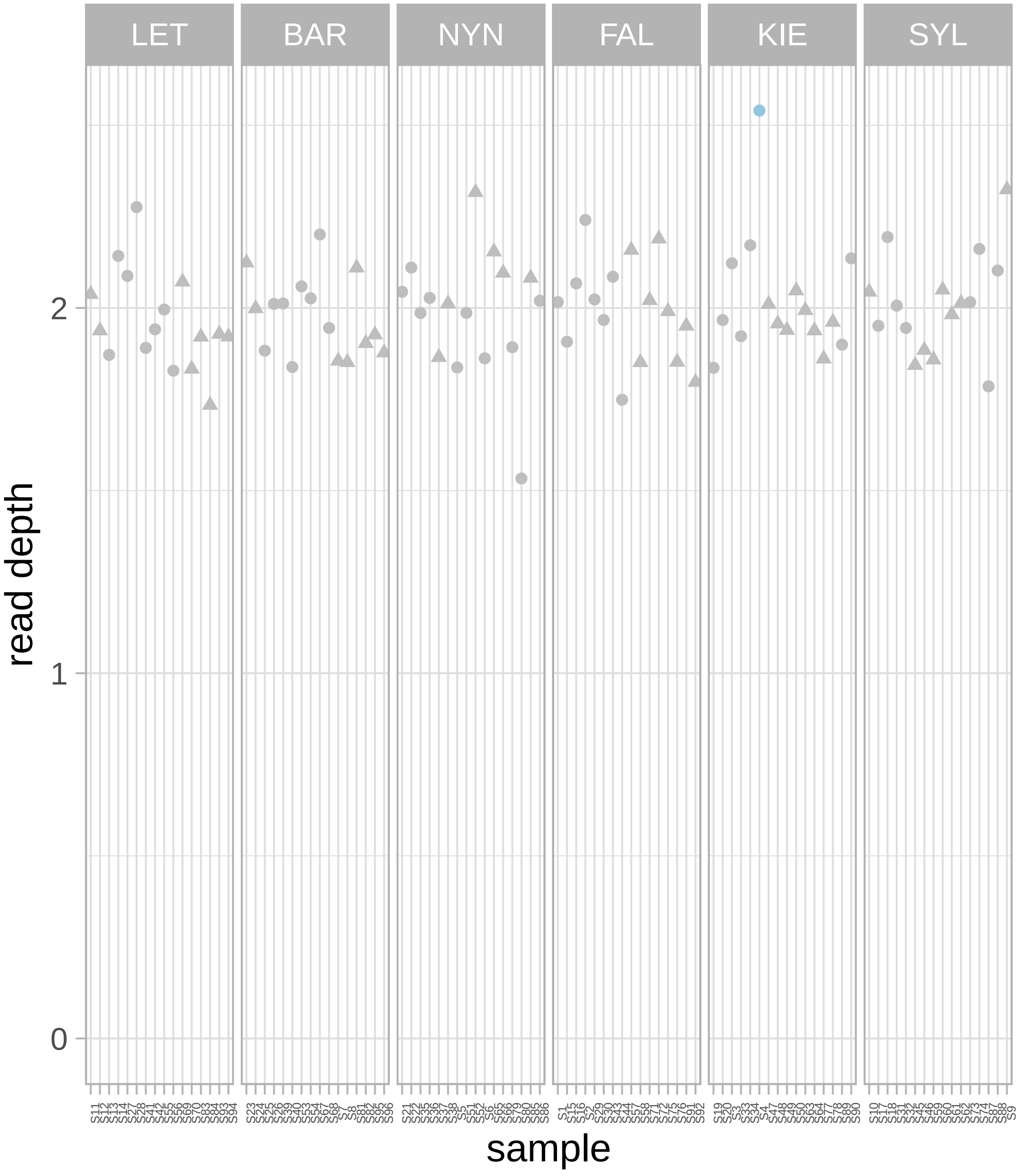

ENSGACG00000013762 chrXI 13483374 dup

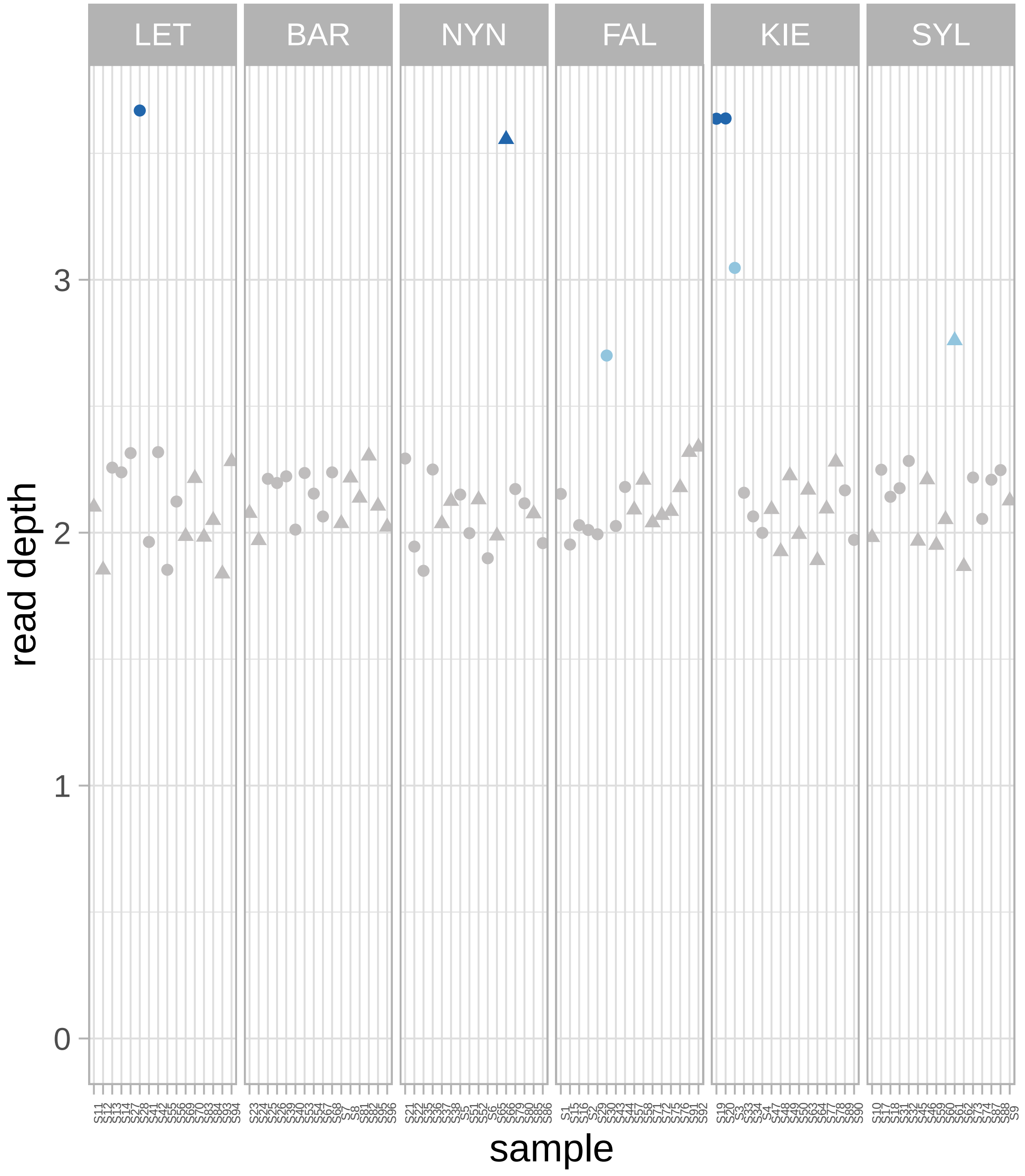

ENSGACG00000003056 chrXII 1404687 dup

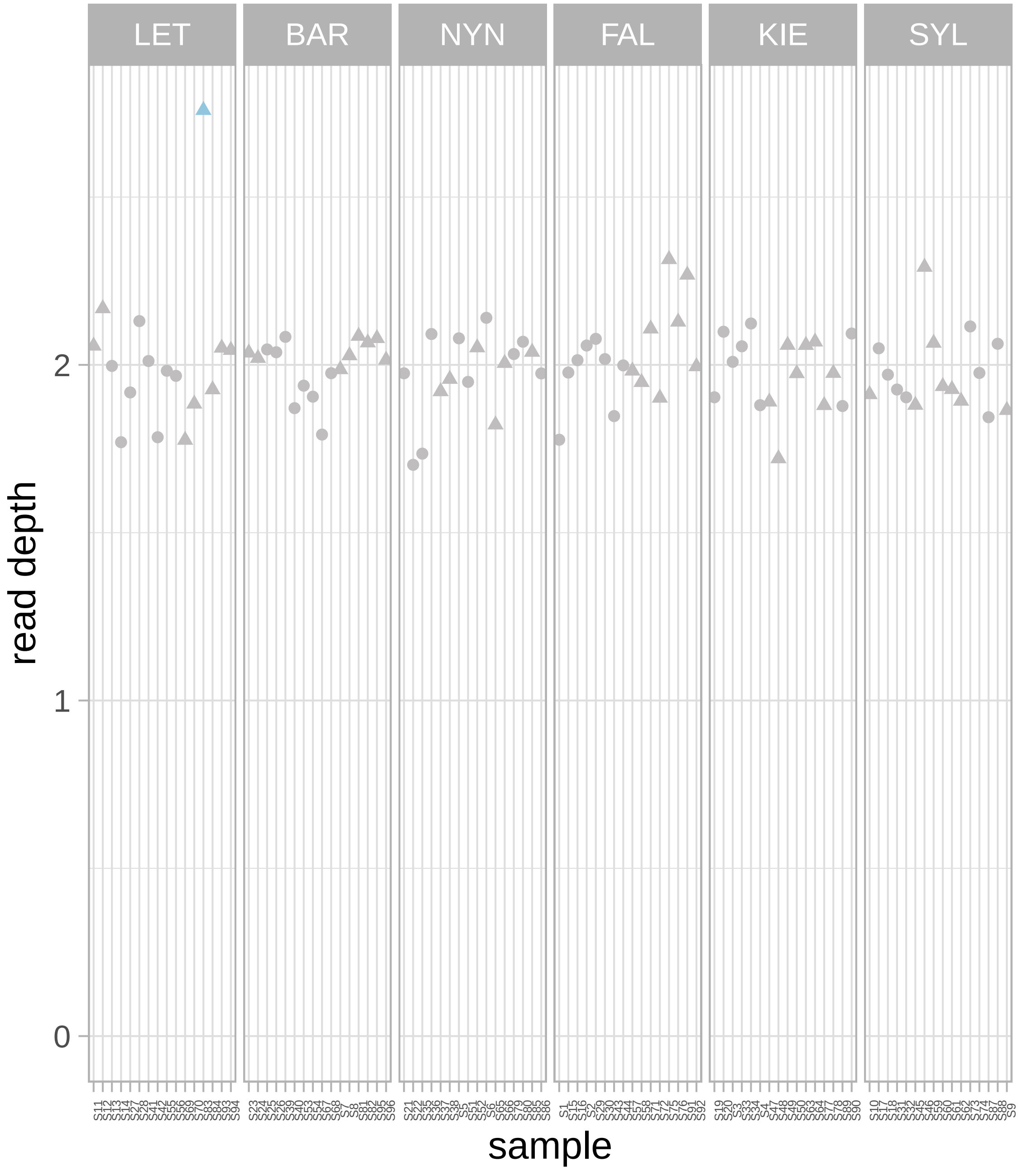

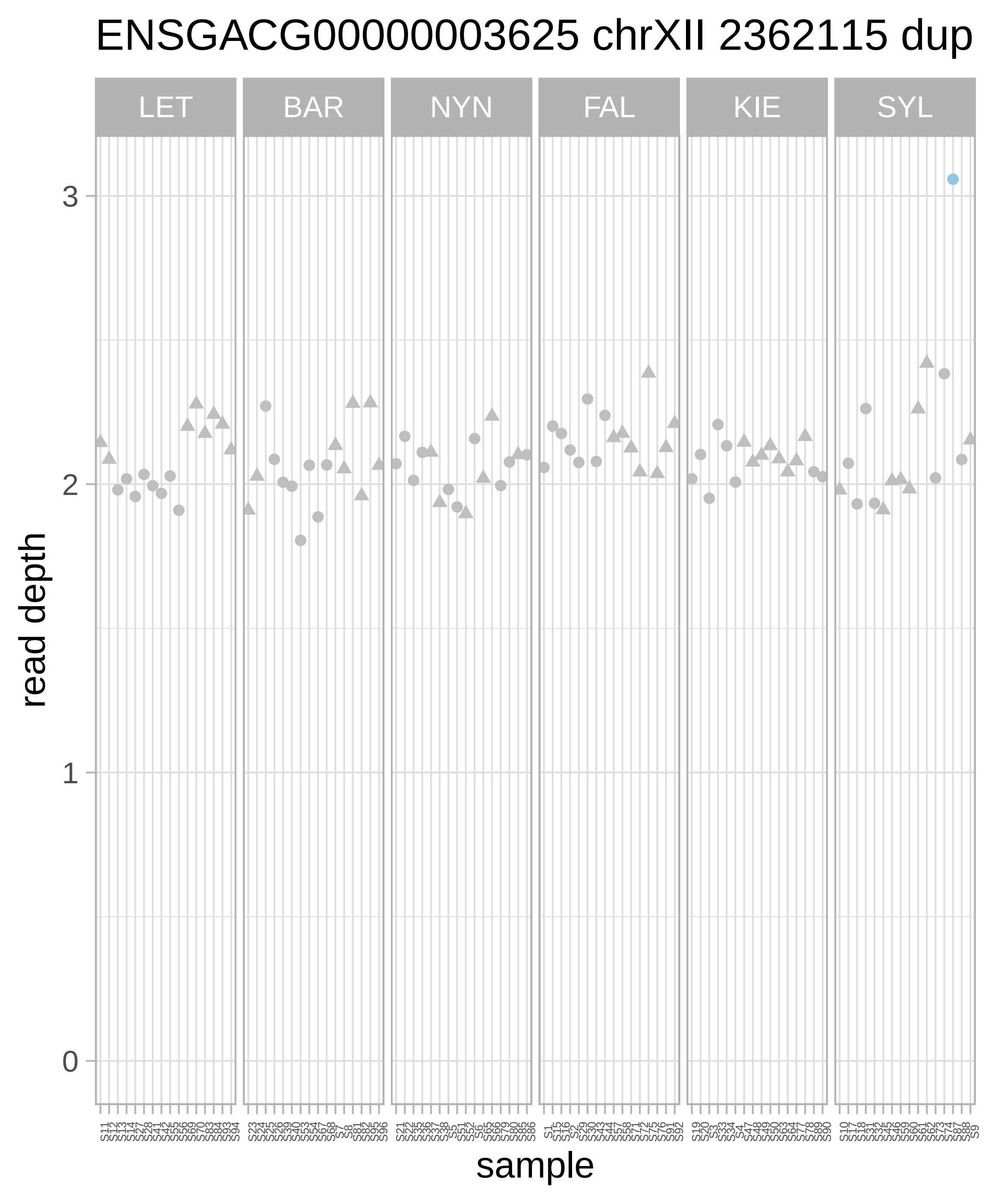
ENSGACG00000003625 chrXII 2362115 dup
LET
BAR
NYN
FAL
KIE
SYL
read depth
0
1
2
3
S11 S12 S13 S14 S27 S28 S41 S42 S55 S56 S69 S70 S83 S84 S93 S94
S23 S24 S25 S26 S39 S40 S53 S54 S67 S68 S7 S8 S81 S82 S95 S96
S21 S22 S35 S36 S37 S38 S5 S51 S52 S6 S65 S66 S79 S80 S85 S86
S1 S15 S16 S2 S29 S30 S43 S44 S57 S58 S71 S72 S75 S76 S91 S92
S19 S20 S3 S33 S34 S4 S47 S48 S49 S50 S63 S64 S77 S78 S89 S90
S10 S17 S18 S31 S32 S45 S46 S59 S60 S61 S62 S73 S74 S87 S88 S9
sample

ENSGACG00000004144 chrXII 3528805 del

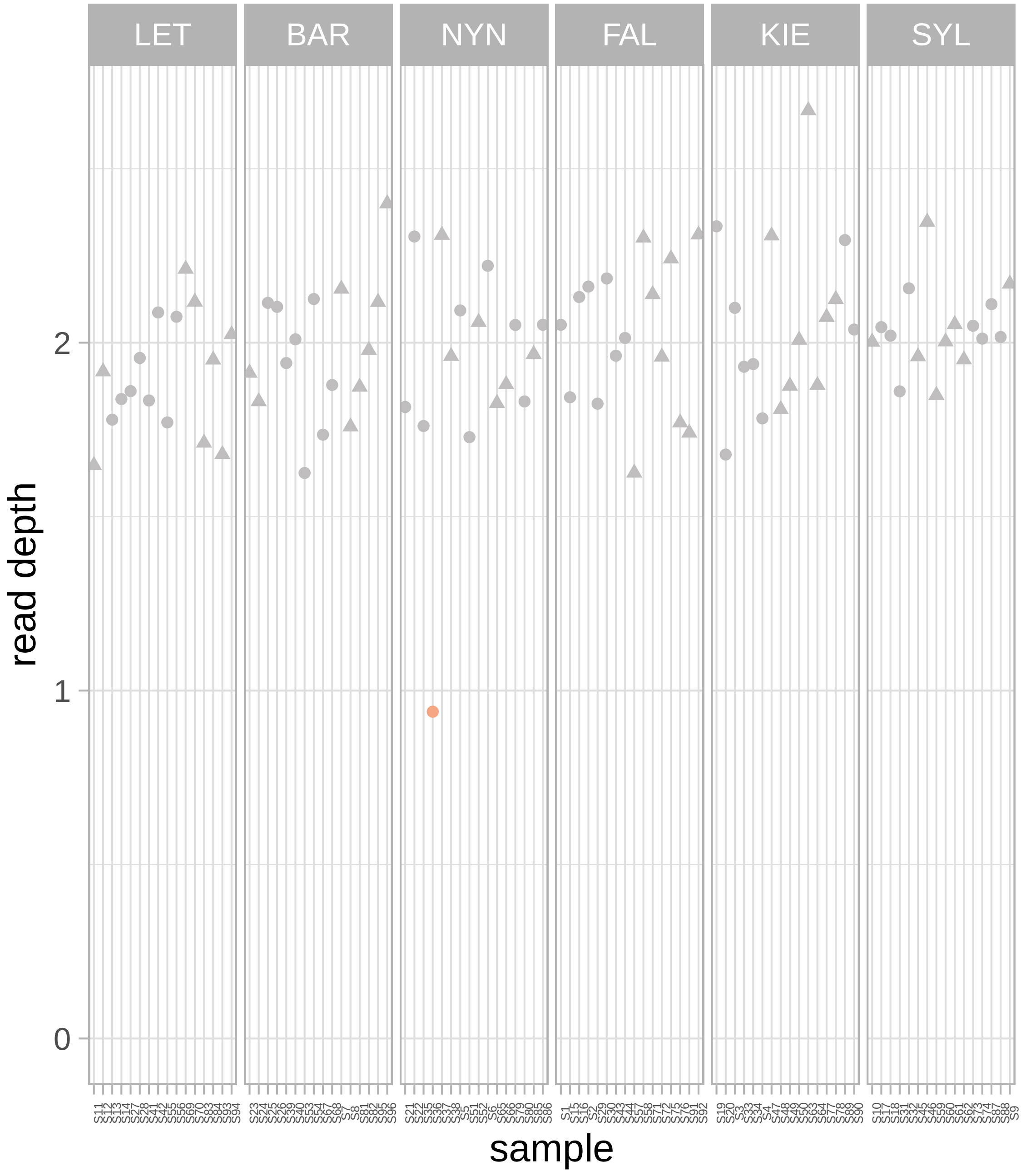

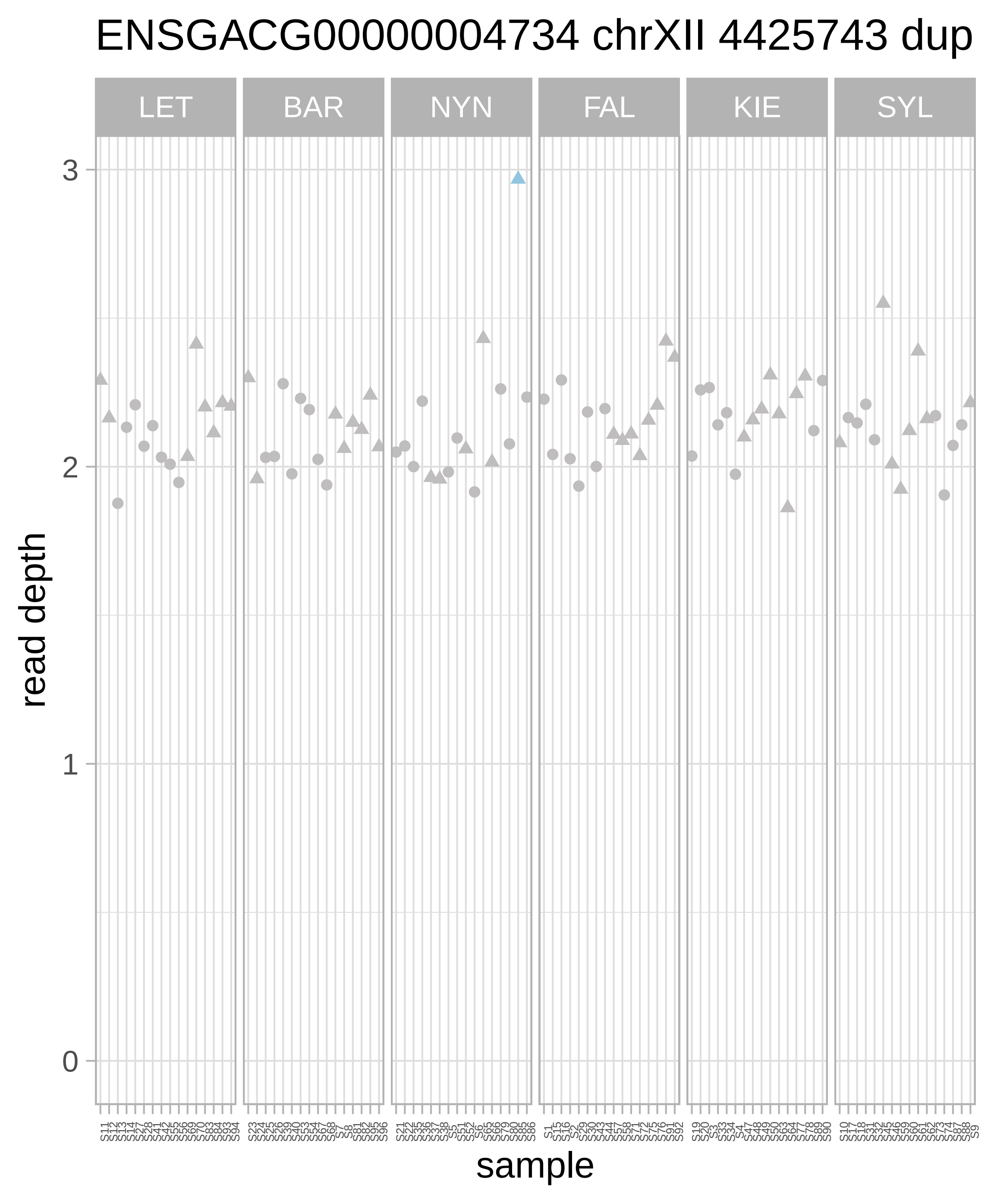
ENSGACG00000004734 chrXII 4425743 dup
LET
BAR
NYN
FAL
KIE
SYL
read depth
0
1
2
3
S11 S12 S13 S14 S27 S28 S41 S42 S55 S56 S69 S70 S83 S84 S93 S94
S23 S24 S25 S26 S39 S40 S53 S54 S67 S68 S7 S8 S81 S82 S95 S96
S21 S22 S35 S36 S37 S38 S5 S51 S52 S6 S65 S66 S79 S80 S85 S86
S1 S15 S16 S2 S29 S30 S43 S44 S57 S58 S71 S72 S75 S76 S91 S92
S19 S20 S3 S33 S34 S4 S47 S48 S49 S50 S63 S64 S77 S78 S89 S90
S10 S17 S18 S31 S32 S45 S46 S59 S60 S61 S62 S73 S74 S87 S88 S9
sample

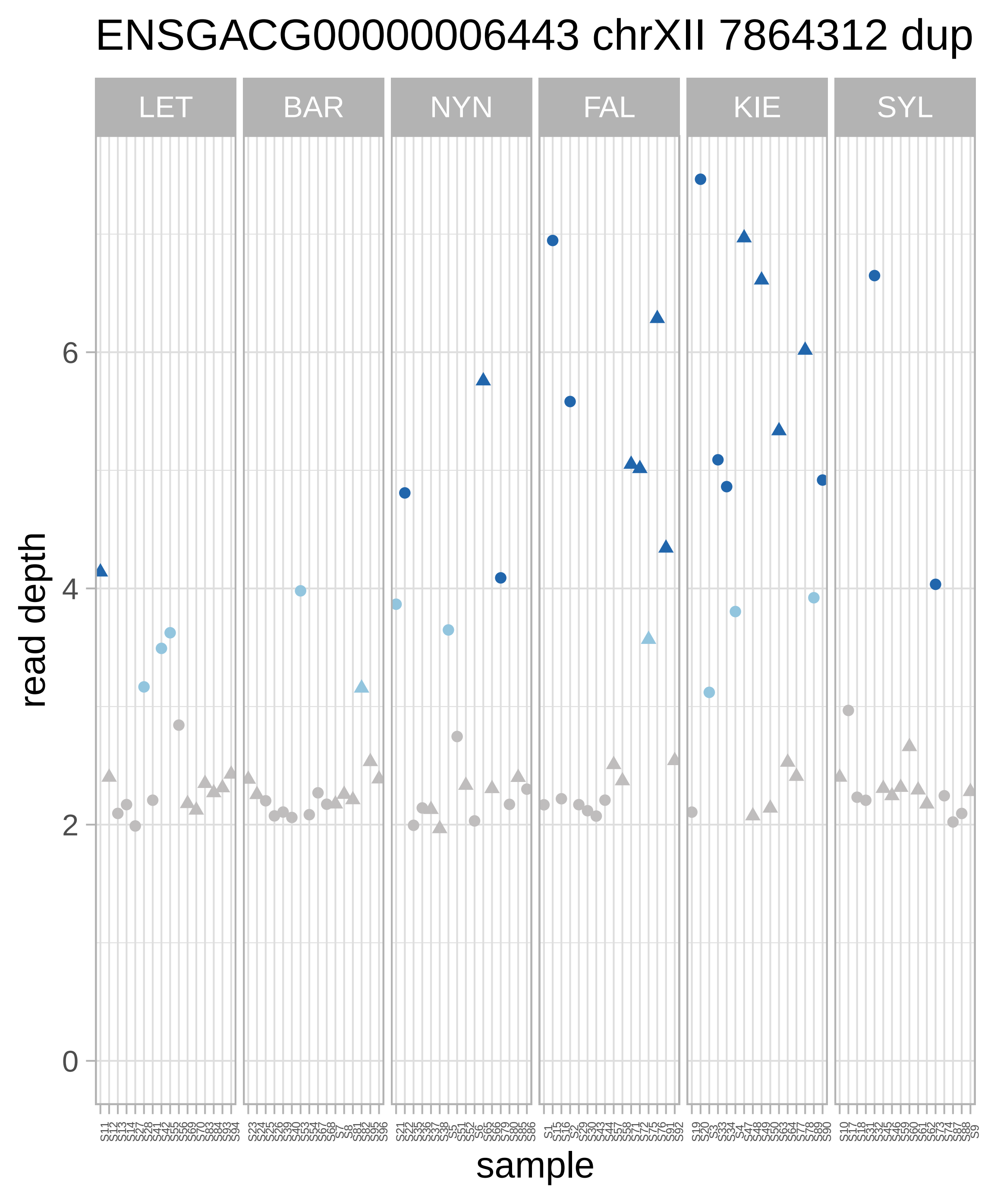
ENSGACG00000006443 chrXII 7864312 dup
LET
BAR
NYN
FAL
KIE
SYL
read depth
0
2
4
6
sample

ENSGACG00000006506 chrXII 8028024 dup

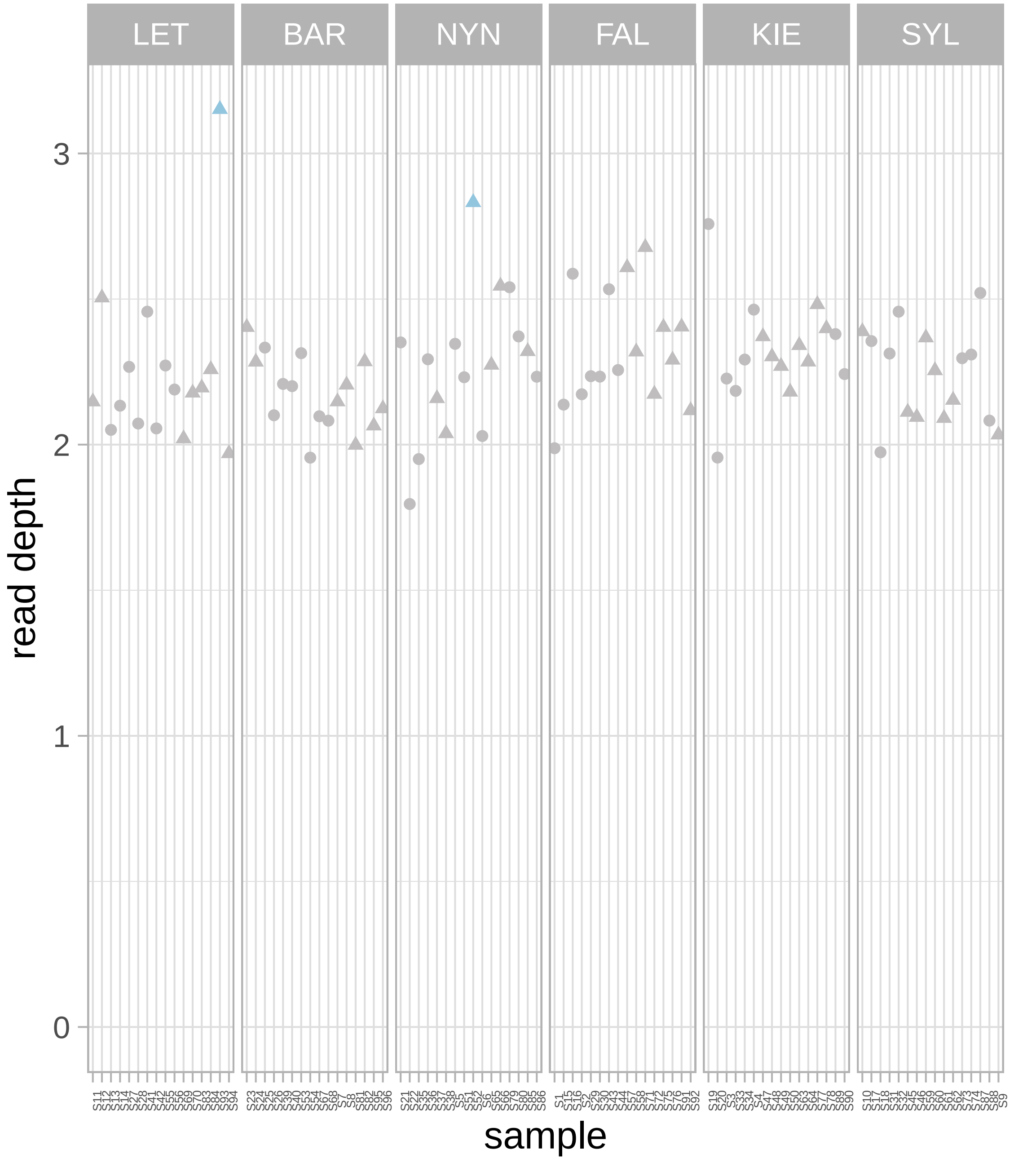

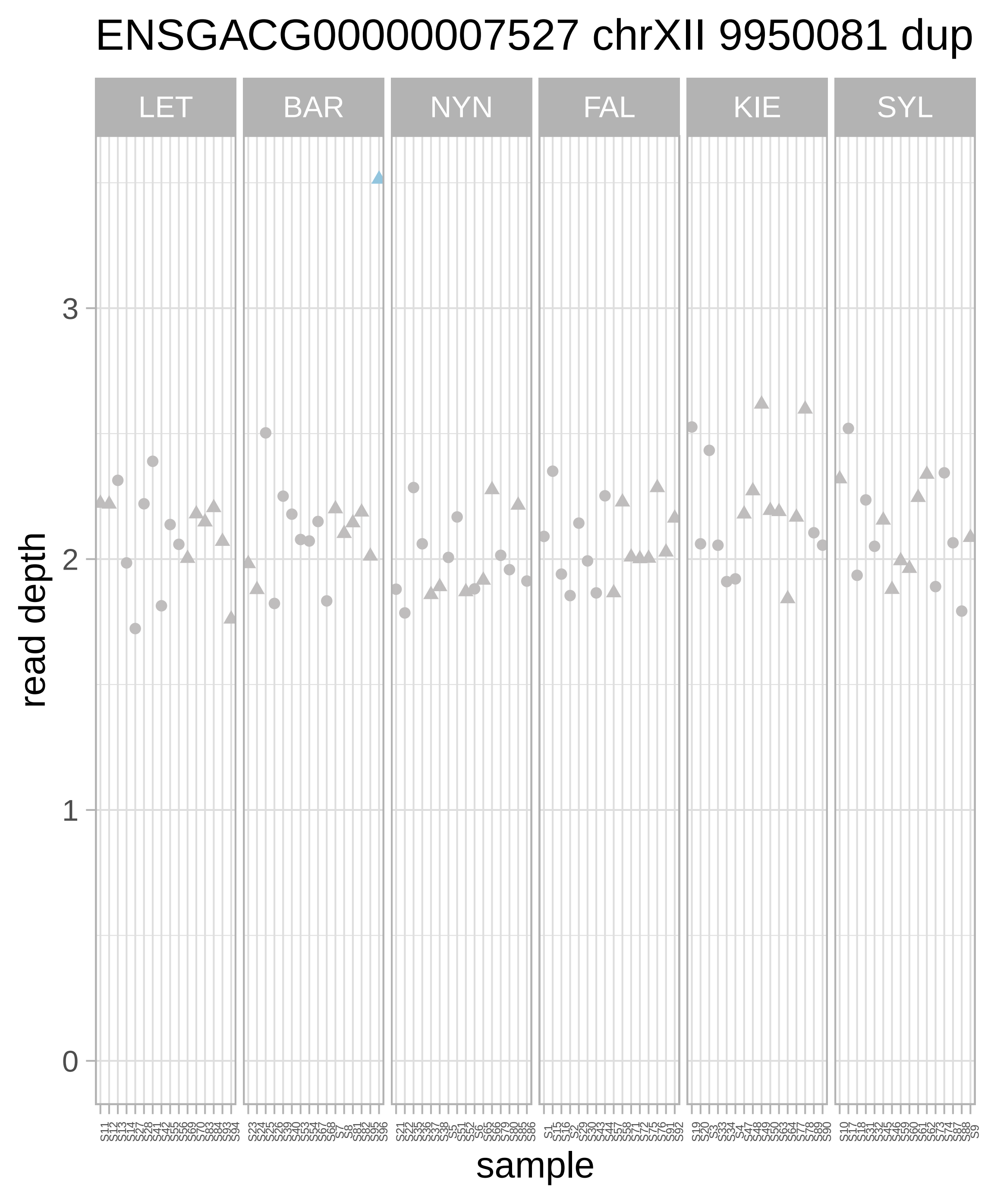

ENSGACG00000007527 chrXII 9950081 dup
LET
BAR
NYN
FAL
KIE
SYL
read depth
0
1
2
3
sample

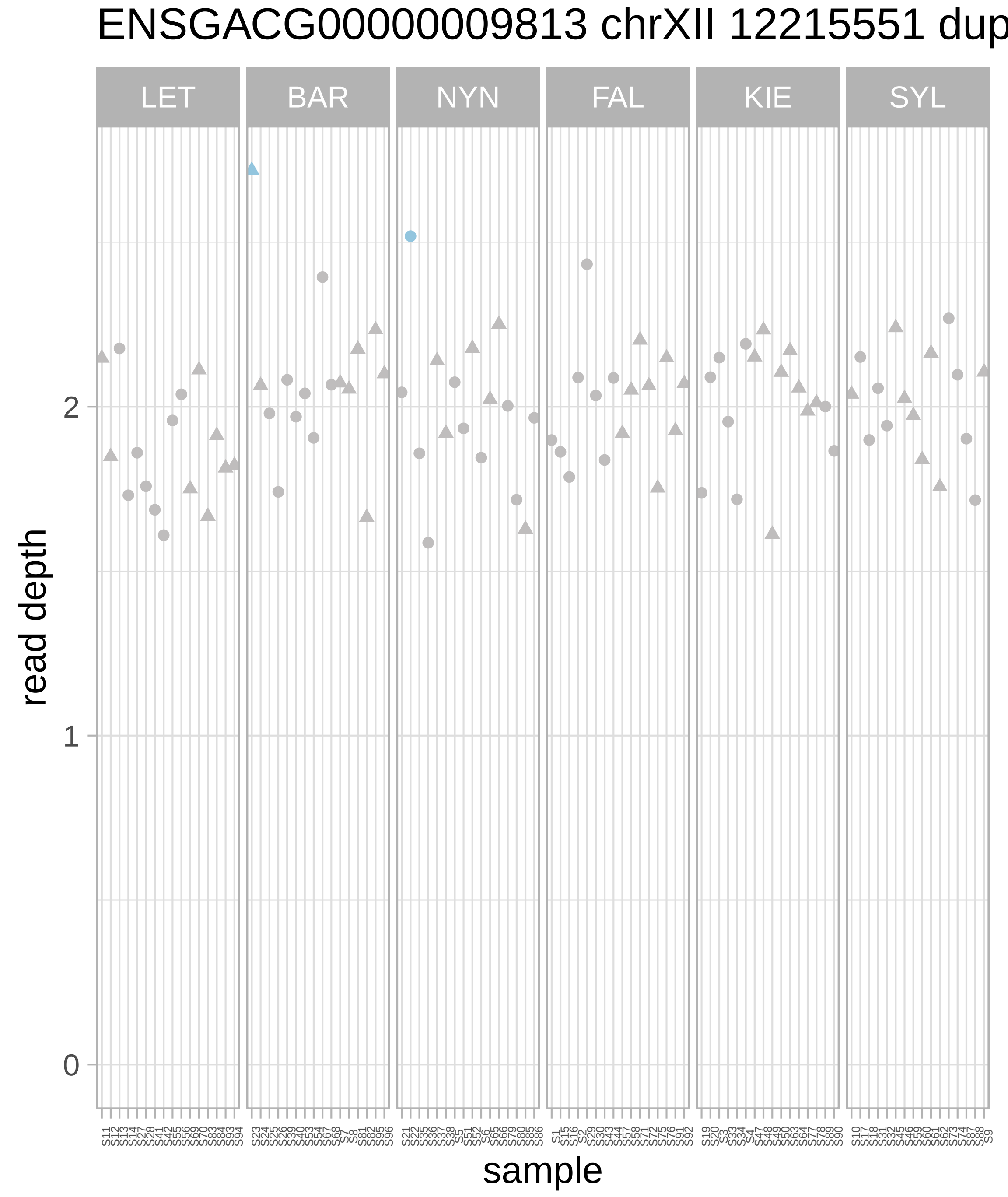
ENSGACG00000009813 chrXII 12215551 dup
LET
BAR
NYN
FAL
KIE
SYL
read depth
0
1
2
S11 S12 S13 S14 S27 S28 S41 S42 S55 S56 S69 S70 S83 S84 S93 S94
S23 S24 S25 S26 S39 S40 S53 S54 S67 S68 S7 S8 S81 S82 S95 S96
S21 S22 S35 S36 S37 S38 S5 S51 S52 S6 S65 S66 S79 S80 S85 S86
S1 S15 S16 S2 S29 S30 S43 S44 S57 S58 S71 S72 S75 S76 S91 S92
S19 S20 S3 S33 S34 S4 S47 S48 S49 S50 S63 S64 S77 S78 S89 S90
S10 S17 S18 S31 S32 S45 S46 S59 S60 S61 S62 S73 S74 S87 S88 S9
sample

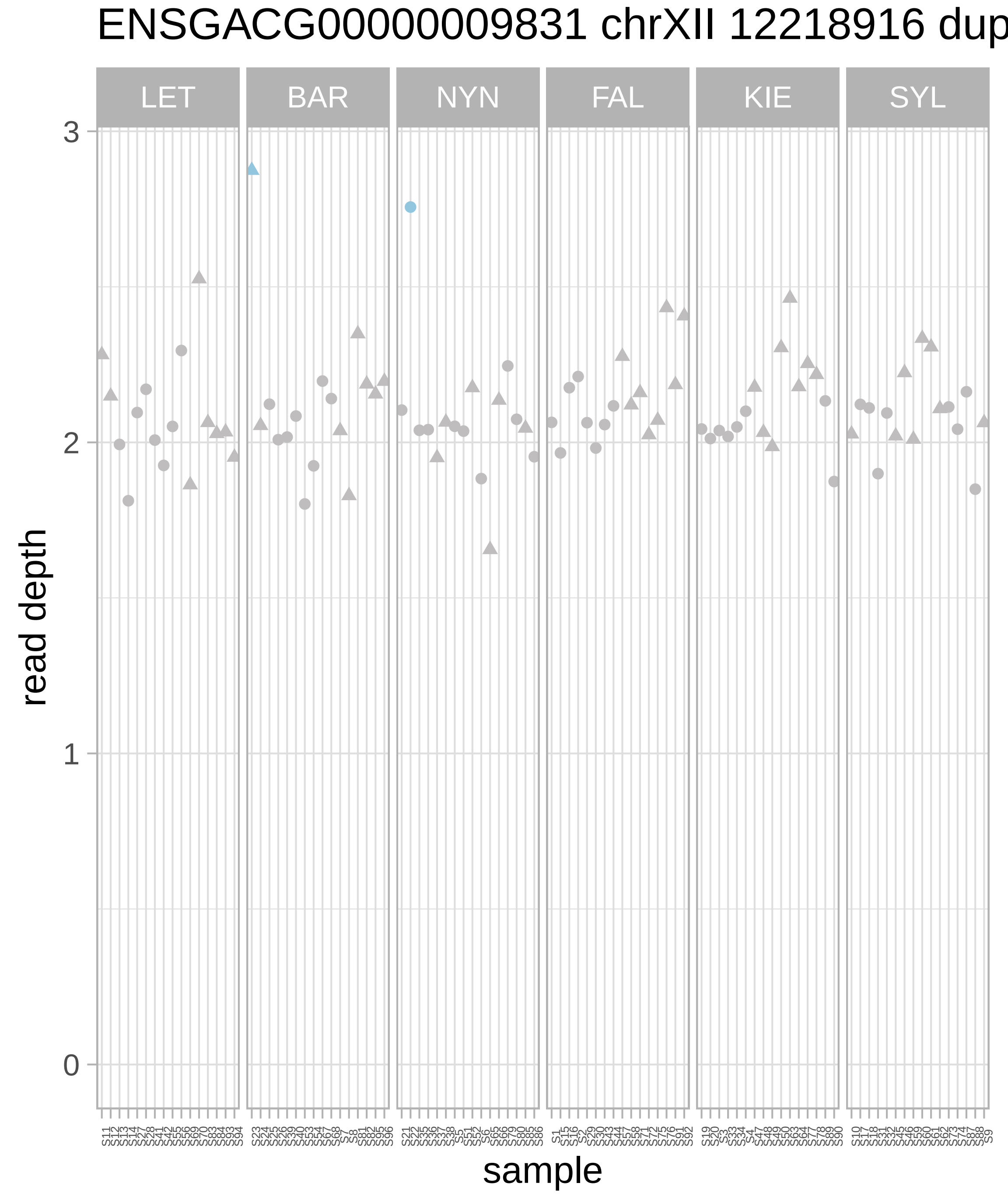

ENSGACG00000009831 chrXII 12218916 dup
LET
BAR
NYN
FAL
KIE
SYL
read depth
0
1
2
3
S11 S12 S13 S14 S27 S28 S41 S42 S55 S56 S69 S70 S83 S84 S93 S94
S23 S24 S25 S26 S39 S40 S53 S54 S67 S68 S7 S8 S81 S82 S95 S96
S21 S22 S35 S36 S37 S38 S5 S51 S52 S6 S65 S66 S79 S80 S85 S86
S1 S15 S16 S2 S29 S30 S43 S44 S57 S58 S71 S72 S75 S76 S91 S92
S19 S20 S3 S33 S34 S4 S47 S48 S49 S50 S63 S64 S77 S78 S89 S90
S10 S17 S18 S31 S32 S45 S46 S59 S60 S61 S62 S73 S74 S87 S88 S9
sample

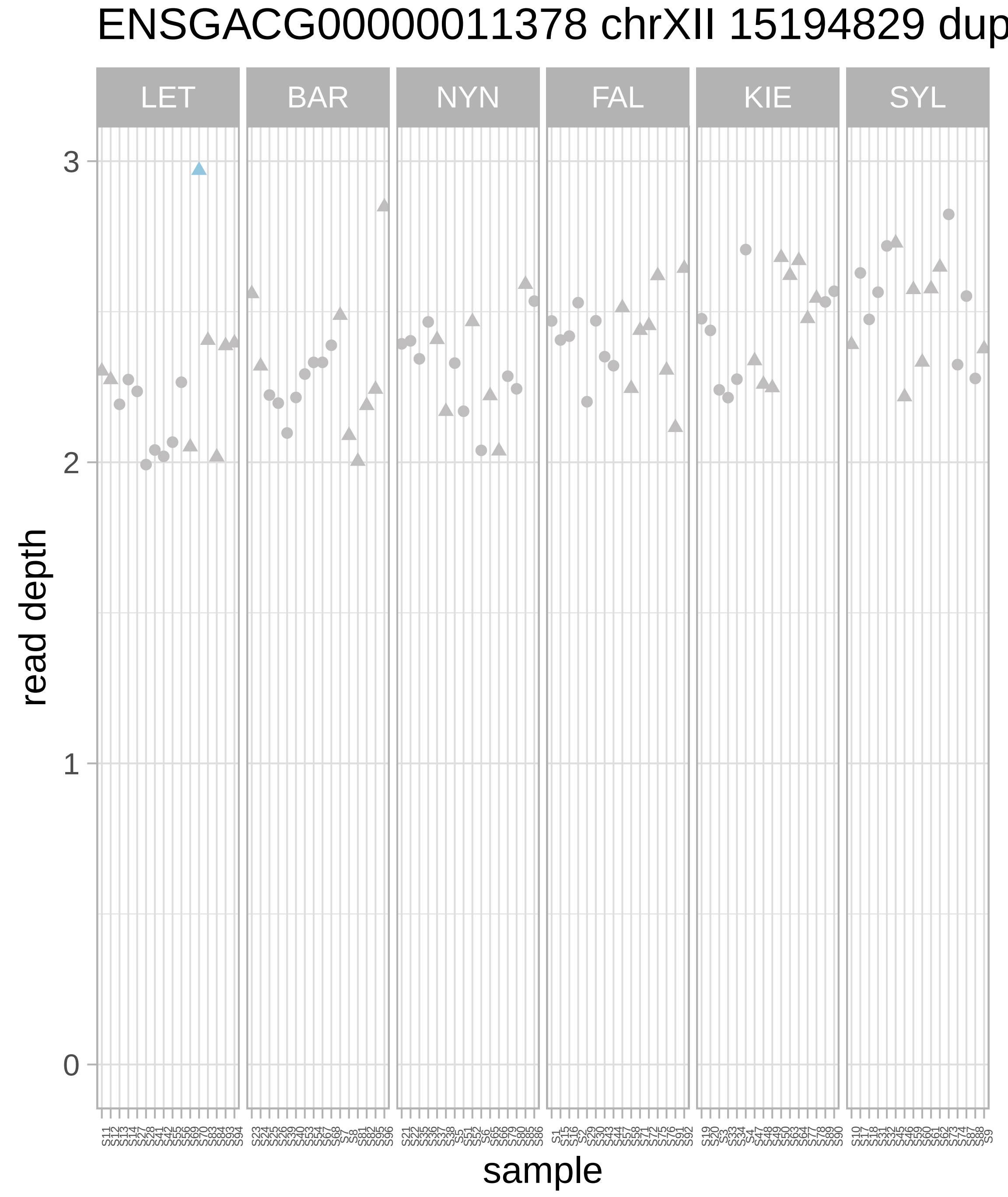
ENSGACG00000011378 chrXII 15194829 dup
LET
BAR
NYN
FAL
KIE
SYL
read depth
0
1
2
3
S11 S12 S13 S14 S27 S28 S41 S42 S55 S56 S69 S70 S83 S84 S93 S94
S23 S24 S25 S26 S39 S40 S53 S54 S67 S68 S7 S8 S81 S82 S95 S96
S21 S22 S35 S36 S37 S38 S5 S51 S52 S6 S65 S66 S79 S80 S85 S86
S1 S15 S16 S2 S29 S30 S43 S44 S57 S58 S71 S72 S75 S76 S91 S92
S19 S20 S3 S33 S34 S4 S47 S48 S49 S50 S63 S64 S77 S78 S89 S90
S10 S17 S18 S31 S32 S45 S46 S59 S60 S61 S62 S73 S74 S87 S88 S9
sample

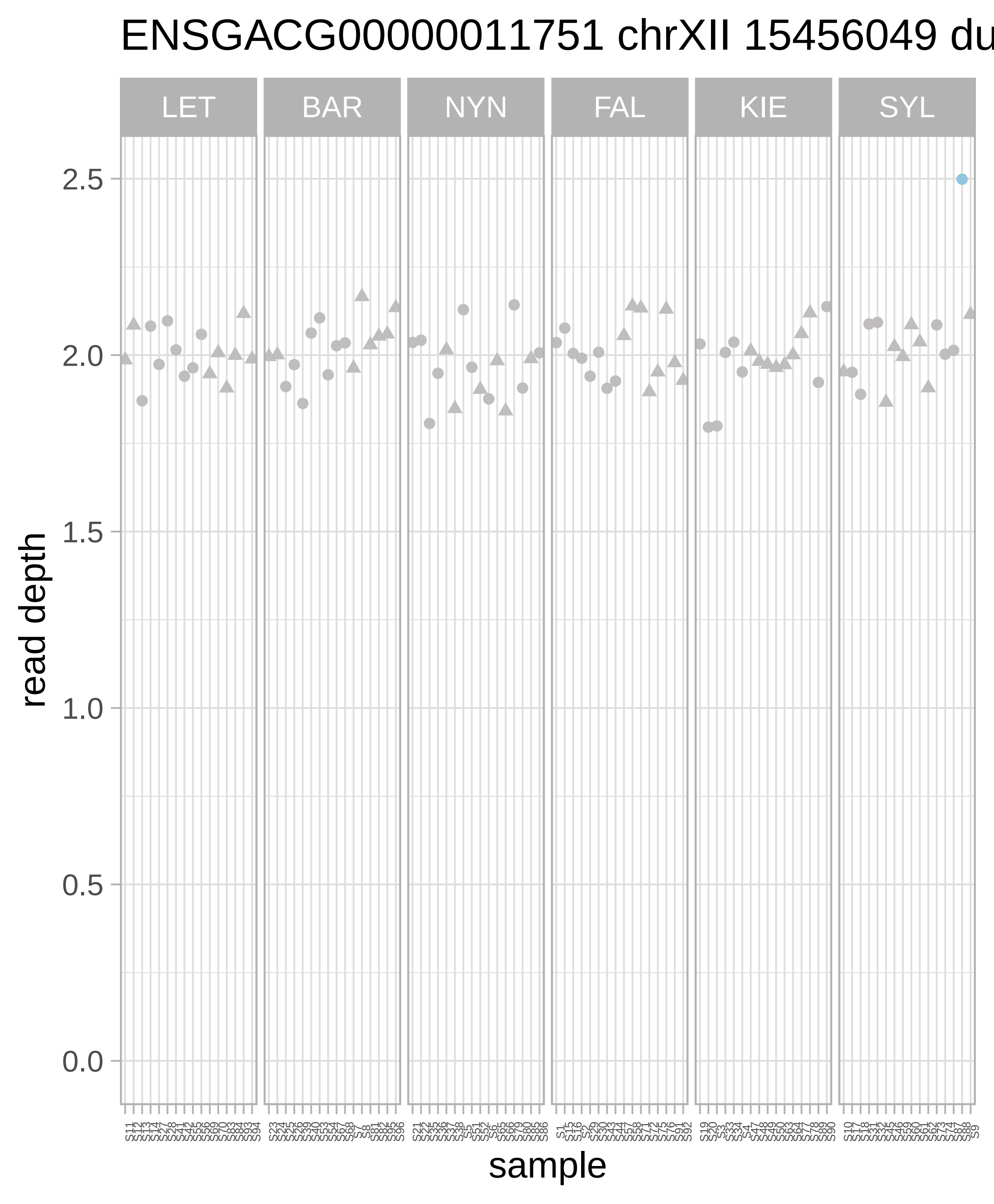
ENSGACG00000011751 chrXII 15456049 du
LET
BAR
NYN
FAL
KIE
SYL
read depth
2.5
2.0
1.5
1.0
0.5
0.0
sample

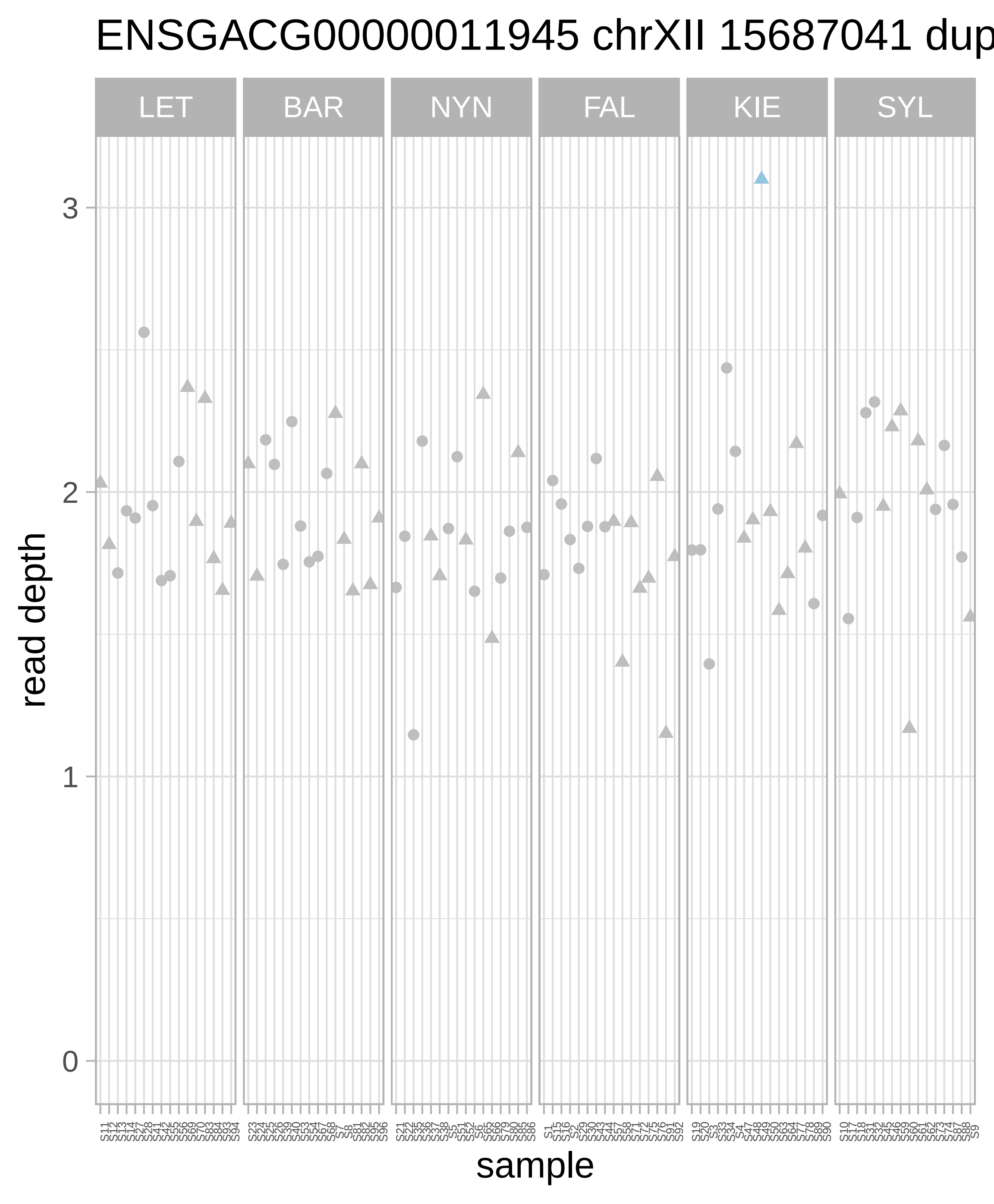
ENSGACG00000011945 chrXII 15687041 dup
LET
BAR
NYN
FAL
KIE
SYL
read depth
0
1
2
3
sample

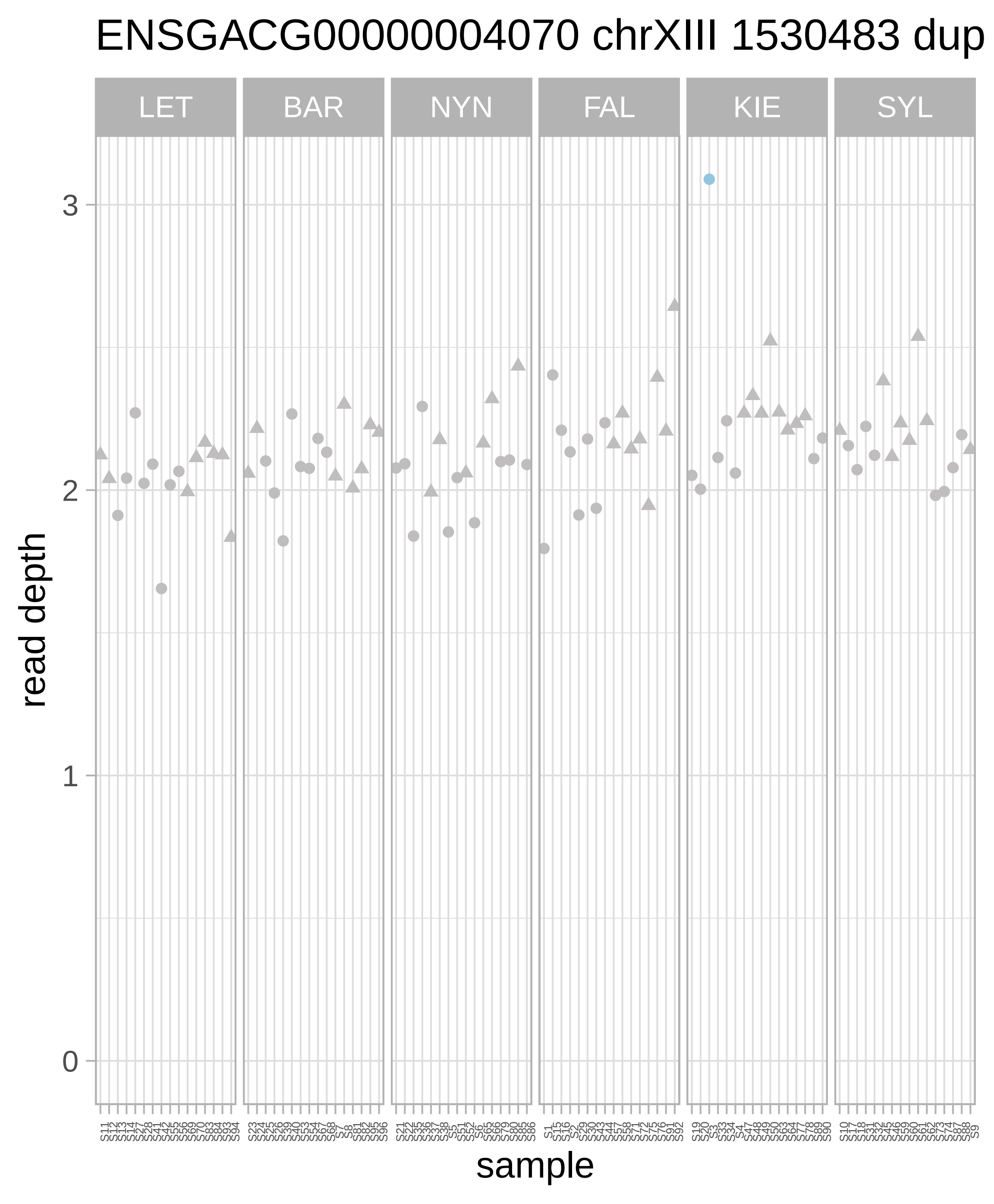
ENSGACG00000004070 chrXIII 1530483 dup
LET
BAR
NYN
FAL
KIE
SYL
read depth
0
1
2
3
sample
S11 S12 S13 S14 S27 S28 S41 S42 S55 S56 S69 S70 S83 S84 S93 S94
S23 S24 S25 S26 S39 S40 S53 S54 S67 S68 S7 S8 S81 S82 S95 S96
S21 S22 S35 S36 S37 S38 S5 S51 S52 S6 S65 S66 S79 S80 S85 S86
S1 S15 S16 S2 S29 S30 S43 S44 S57 S58 S71 S72 S75 S76 S91 S92
S19 S20 S3 S33 S34 S4 S47 S48 S49 S50 S63 S64 S77 S78 S89 S90
S10 S17 S18 S31 S32 S45 S46 S59 S60 S61 S62 S73 S74 S87 S88 S9

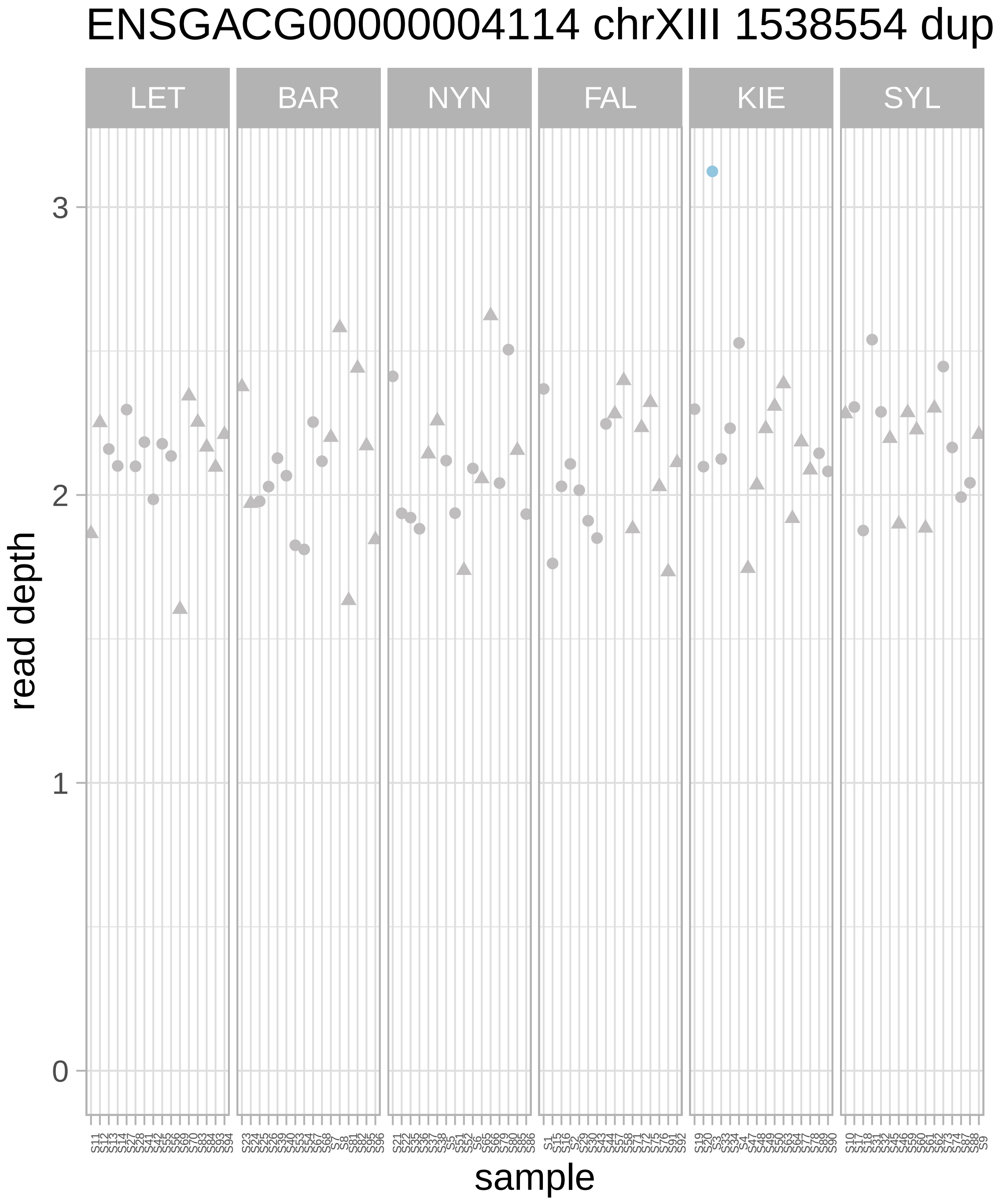
ENSGACG00000004114 chrXIII 1538554 dup
LET
BAR
NYN
FAL
KIE
SYL
read depth
3
2
1
0
S11 S12 S13 S14 S27 S28 S41 S42 S55 S56 S69 S70 S83 S84 S93 S94
S23 S24 S25 S26 S39 S40 S53 S54 S67 S68 S7 S8 S81 S82 S95 S96
S21 S22 S35 S36 S37 S38 S5 S51 S52 S6 S65 S66 S79 S80 S85 S86
S1 S15 S16 S2 S29 S30 S43 S44 S57 S58 S71 S72 S75 S76 S91 S92
S19 S20 S3 S33 S34 S4 S47 S48 S49 S50 S63 S64 S77 S78 S89 S90
S10 S17 S18 S31 S32 S45 S46 S59 S60 S61 S62 S73 S74 S87 S88 S9
sample

ENSGACG00000005324 chrXIII 3199825 dup

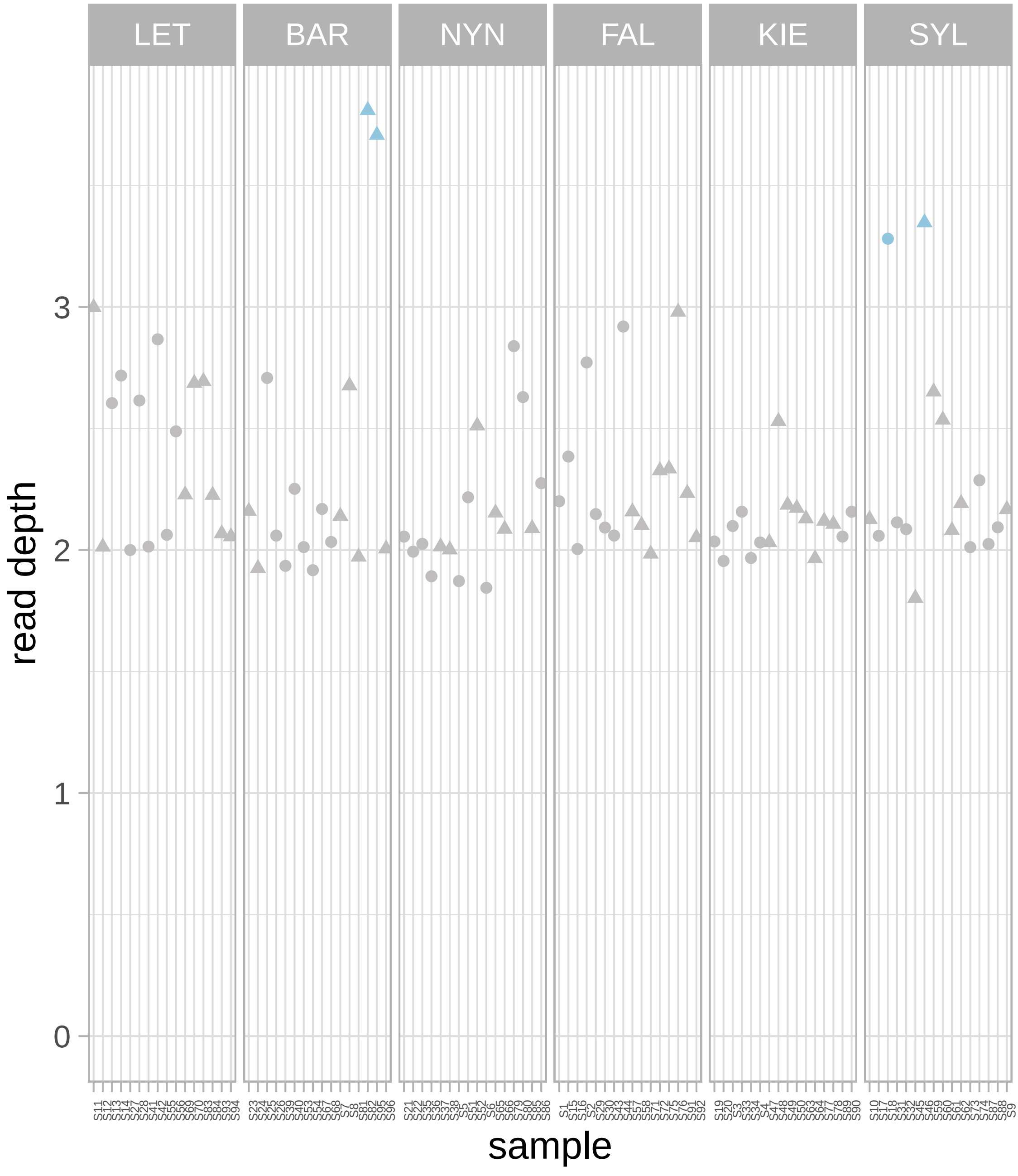

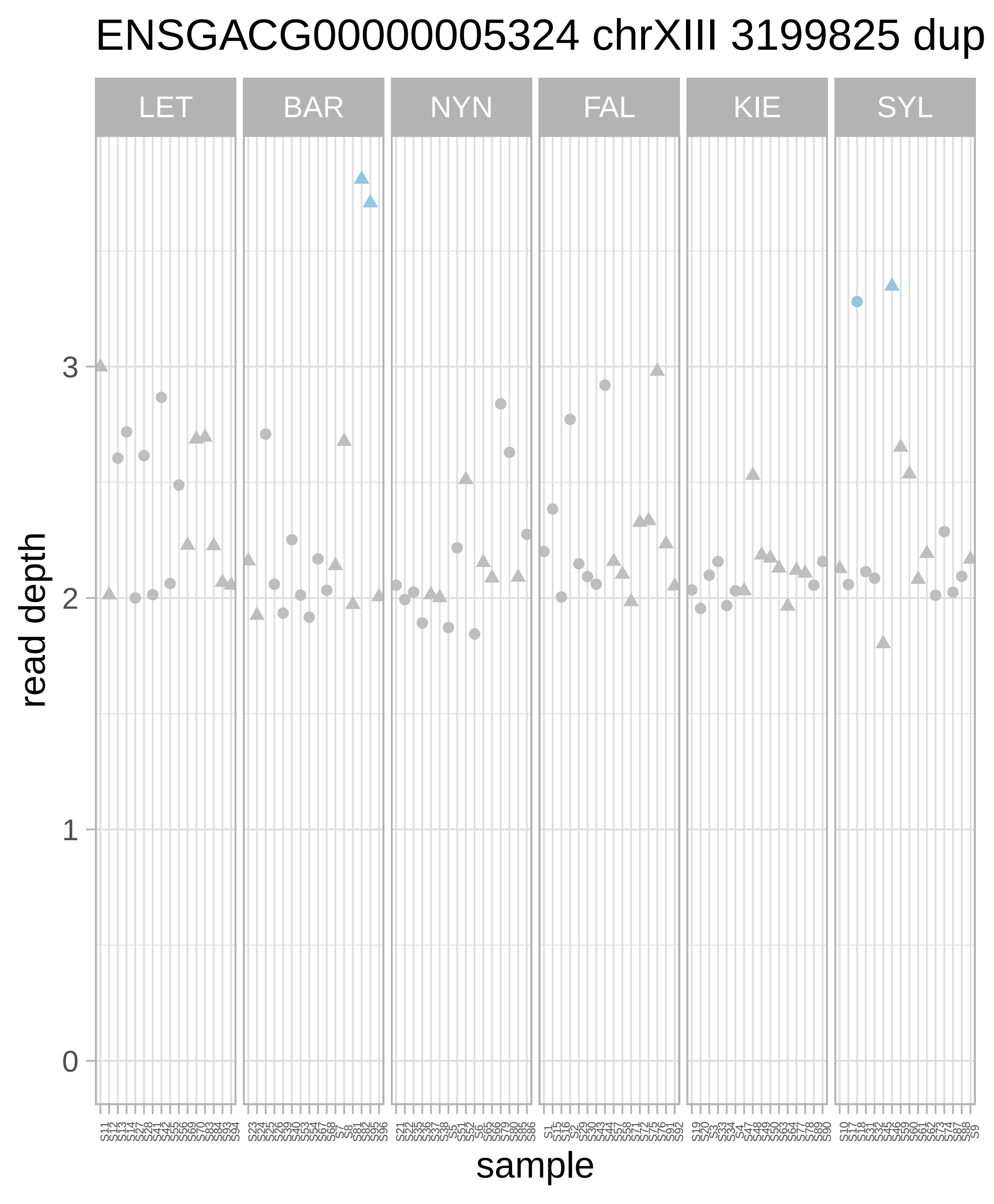

ENSGACG00000005324 chrXIII 3199825 dup
LET
BAR
NYN
FAL
KIE
SYL
read depth
0
1
2
3
sample

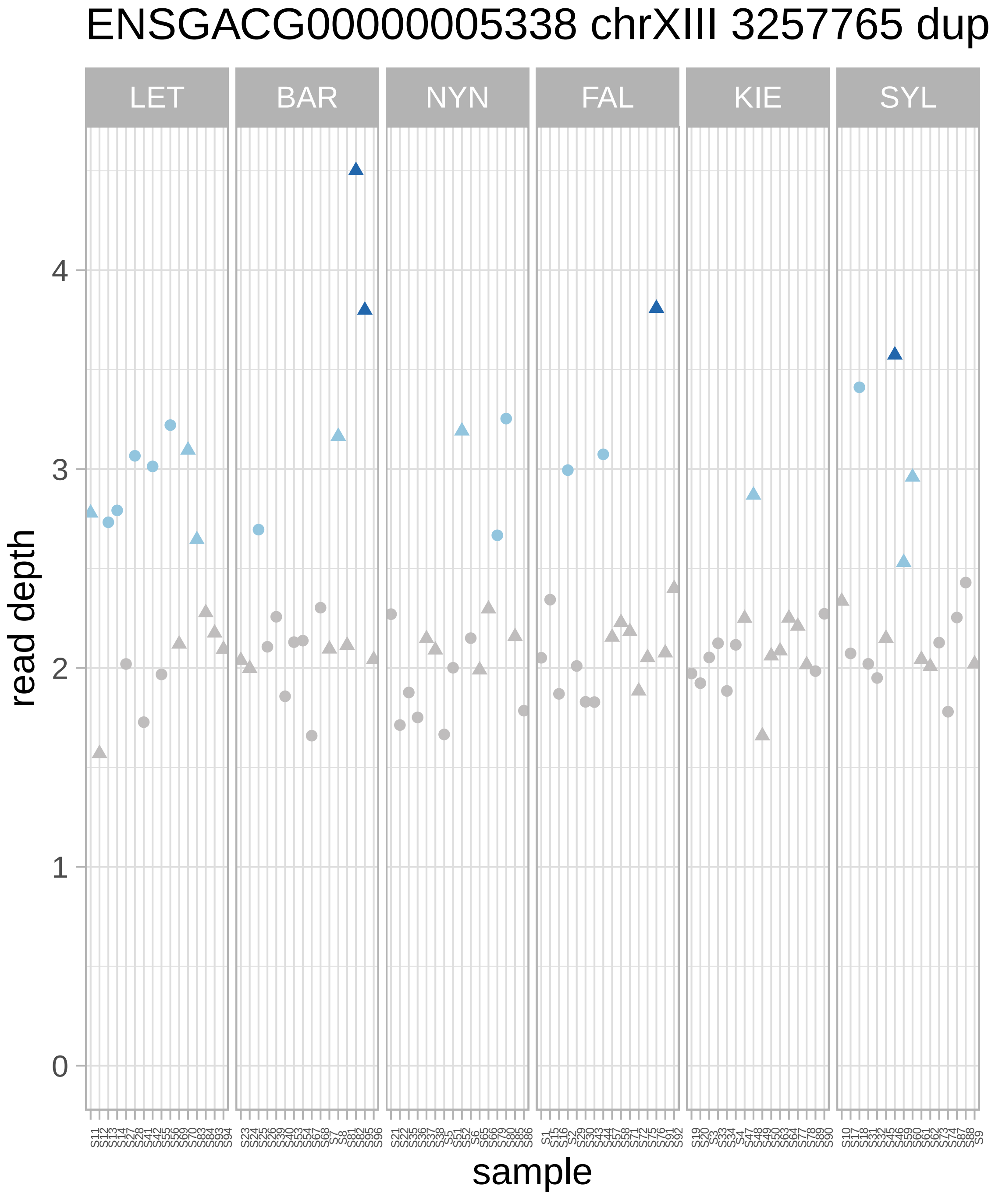

ENSGACG00000005338 chrXIII 3257765 dup
LET
BAR
NYN
FAL
KIE
SYL
read depth
0
1
2
3
4
sample

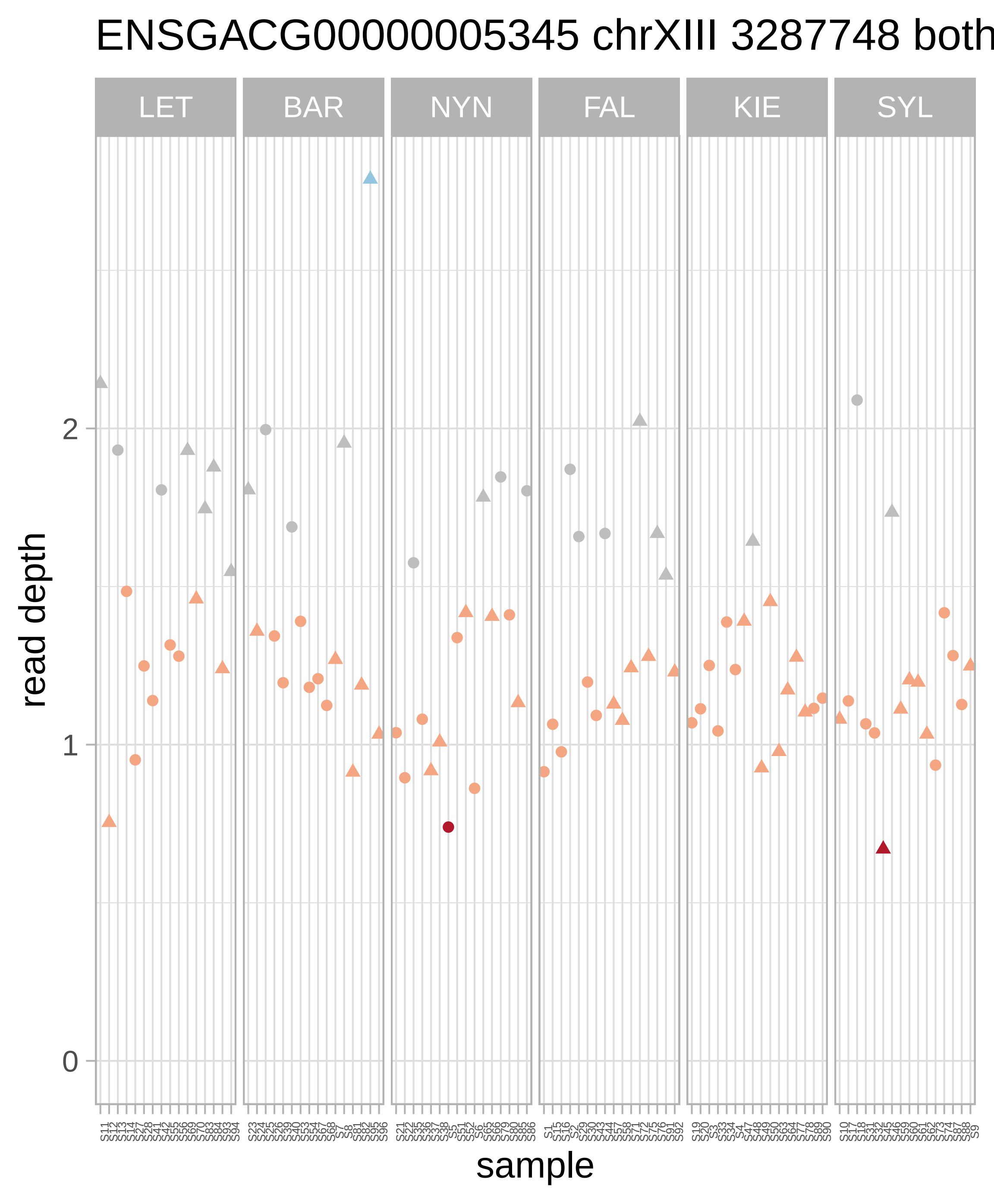
ENSGACG00000005345 chrXIII 3287748 both
LET
BAR
NYN
FAL
KIE
SYL
read depth
2
1
0
sample

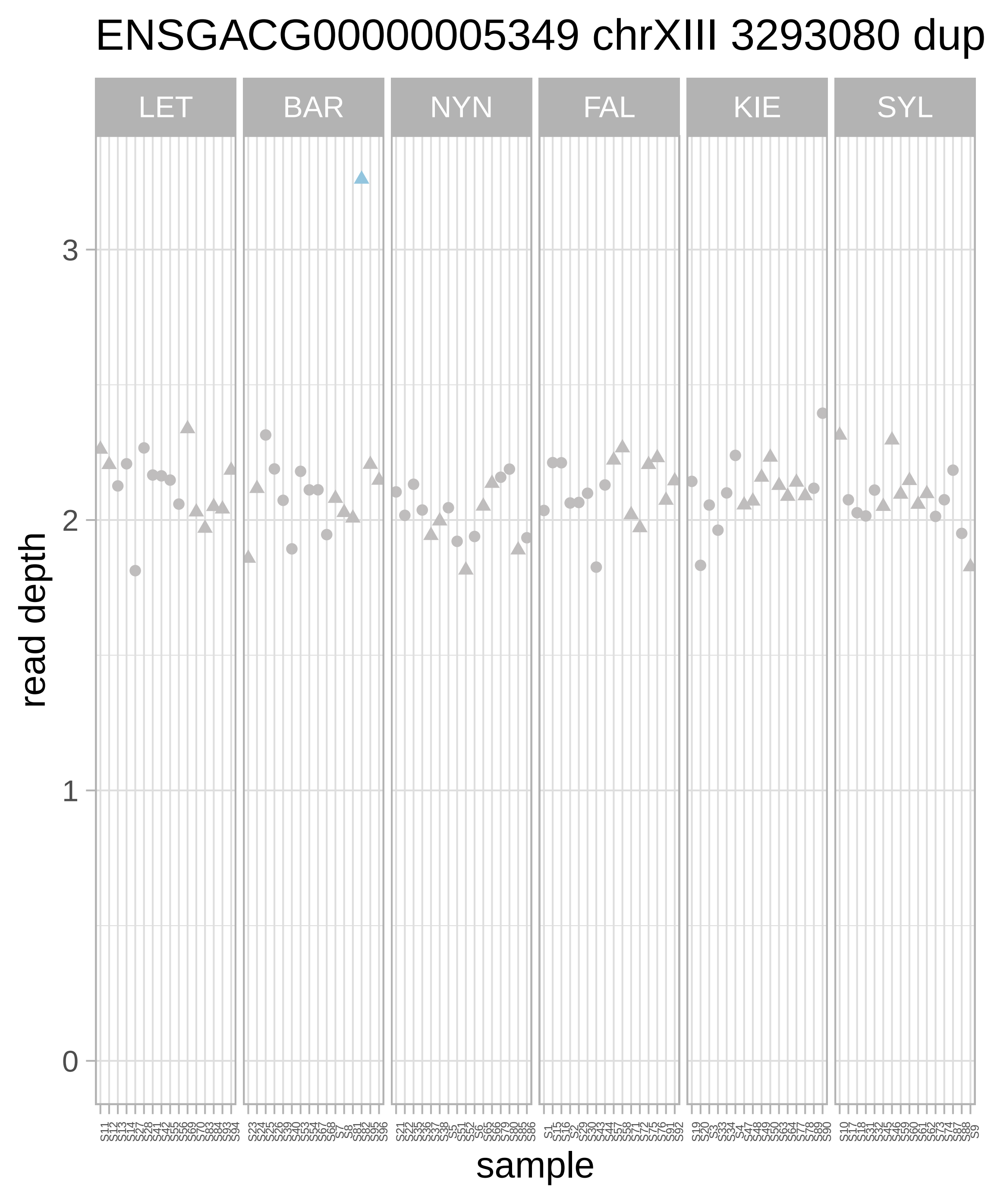
ENSGACG00000005349 chrXIII 3293080 dup
LET
BAR
NYN
FAL
KIE
SYL
read depth
3
2
1
0
sample

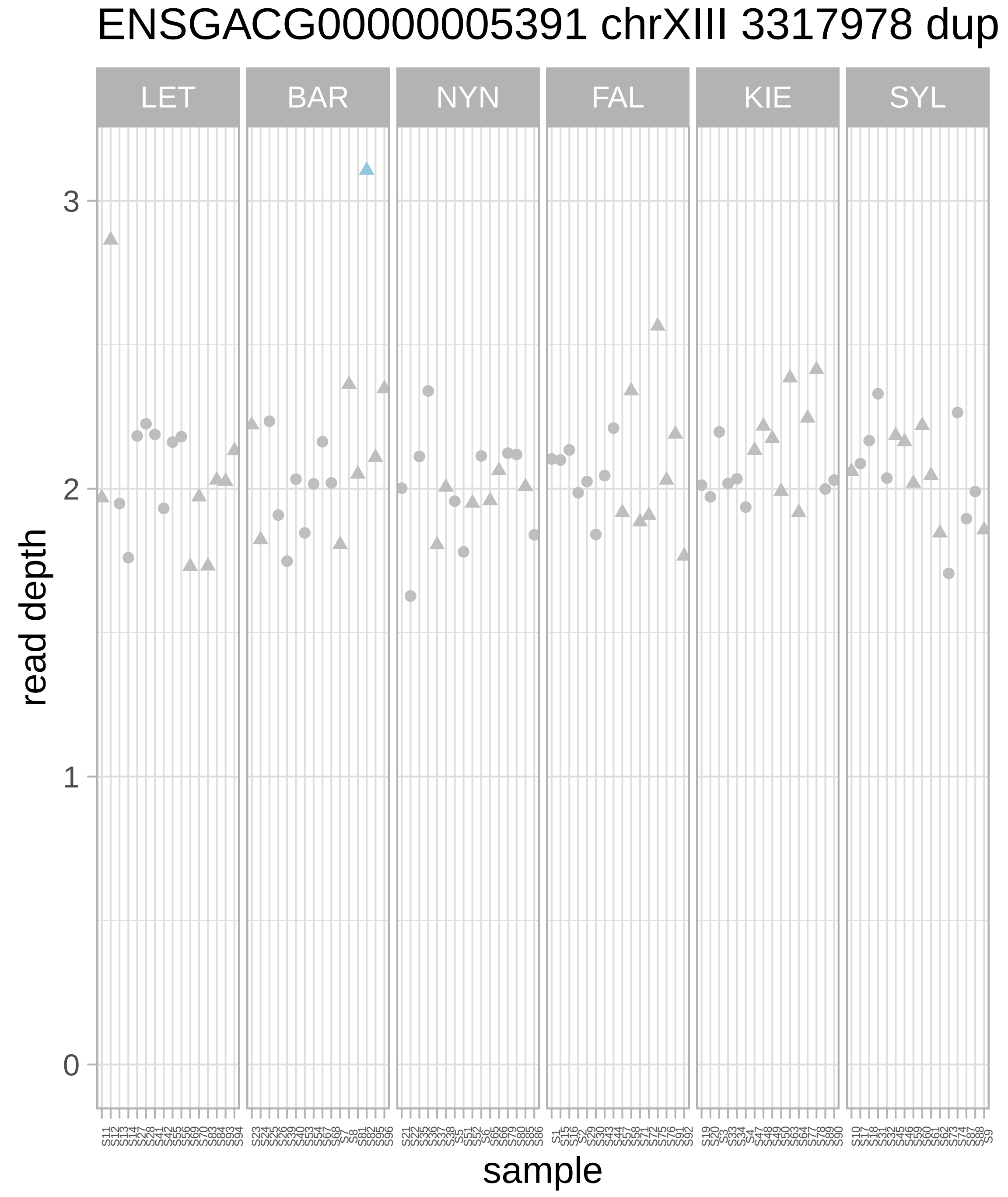
ENSGACG00000005391 chrXIII 3317978 dup
LET
BAR
NYN
FAL
KIE
SYL
read depth
3
2
1
0
sample

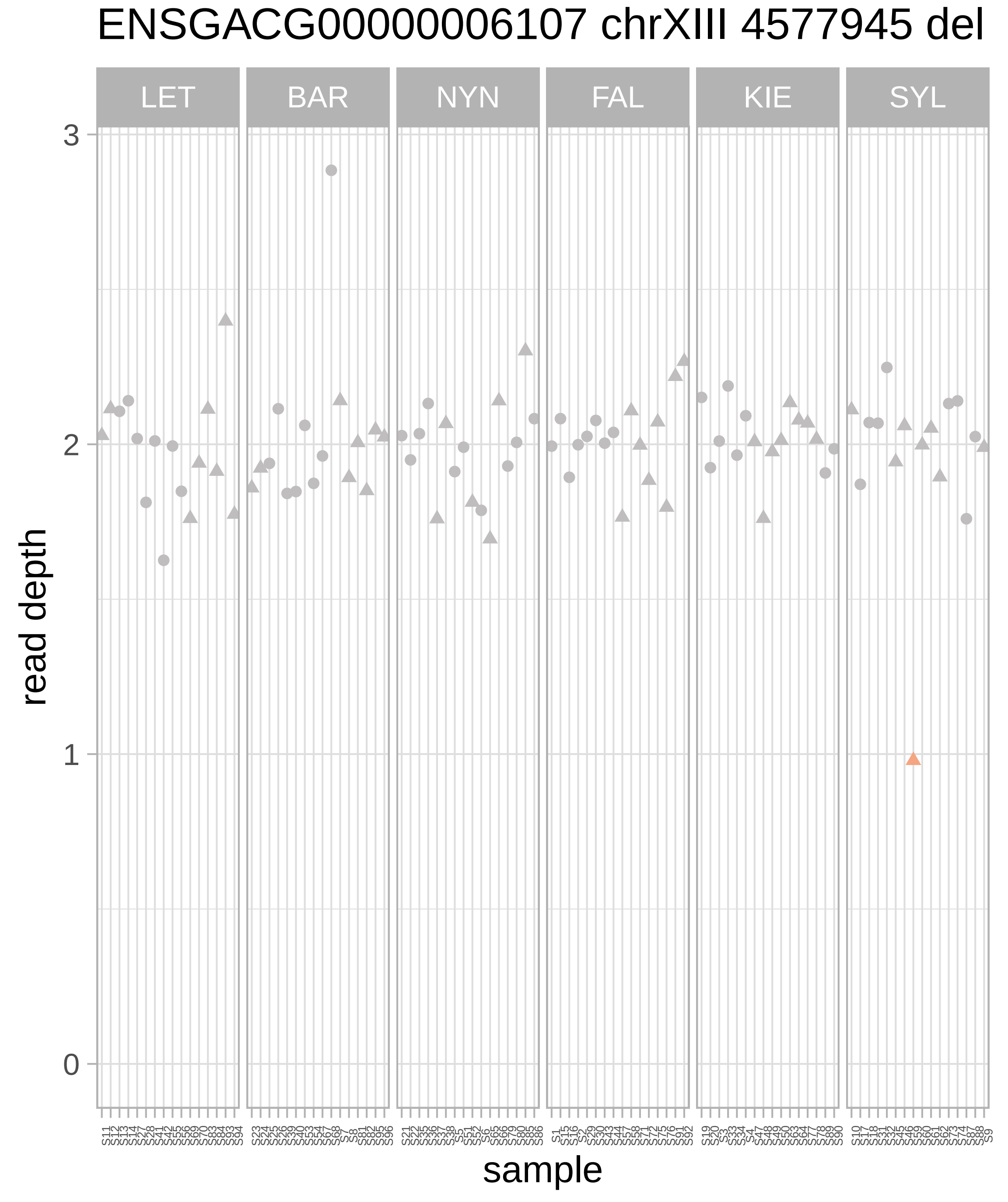
ENSGACG00000006107 chrXIII 4577945 del
LET
BAR
NYN
FAL
KIE
SYL
read depth
3
2
1
0
S11 S12 S13 S14 S27 S28 S41 S42 S55 S56 S69 S70 S83 S84 S93 S94
S23 S24 S25 S26 S39 S40 S53 S54 S67 S68 S7 S8 S81 S82 S95 S96
S21 S22 S35 S36 S37 S38 S5 S51 S52 S6 S65 S66 S79 S80 S85 S86
S1 S15 S16 S2 S29 S30 S43 S44 S57 S58 S71 S72 S75 S76 S91 S92
S19 S20 S3 S33 S34 S4 S47 S48 S49 S50 S63 S64 S77 S78 S89 S90
S10 S17 S18 S31 S32 S45 S46 S59 S60 S61 S62 S73 S74 S87 S88 S9
sample

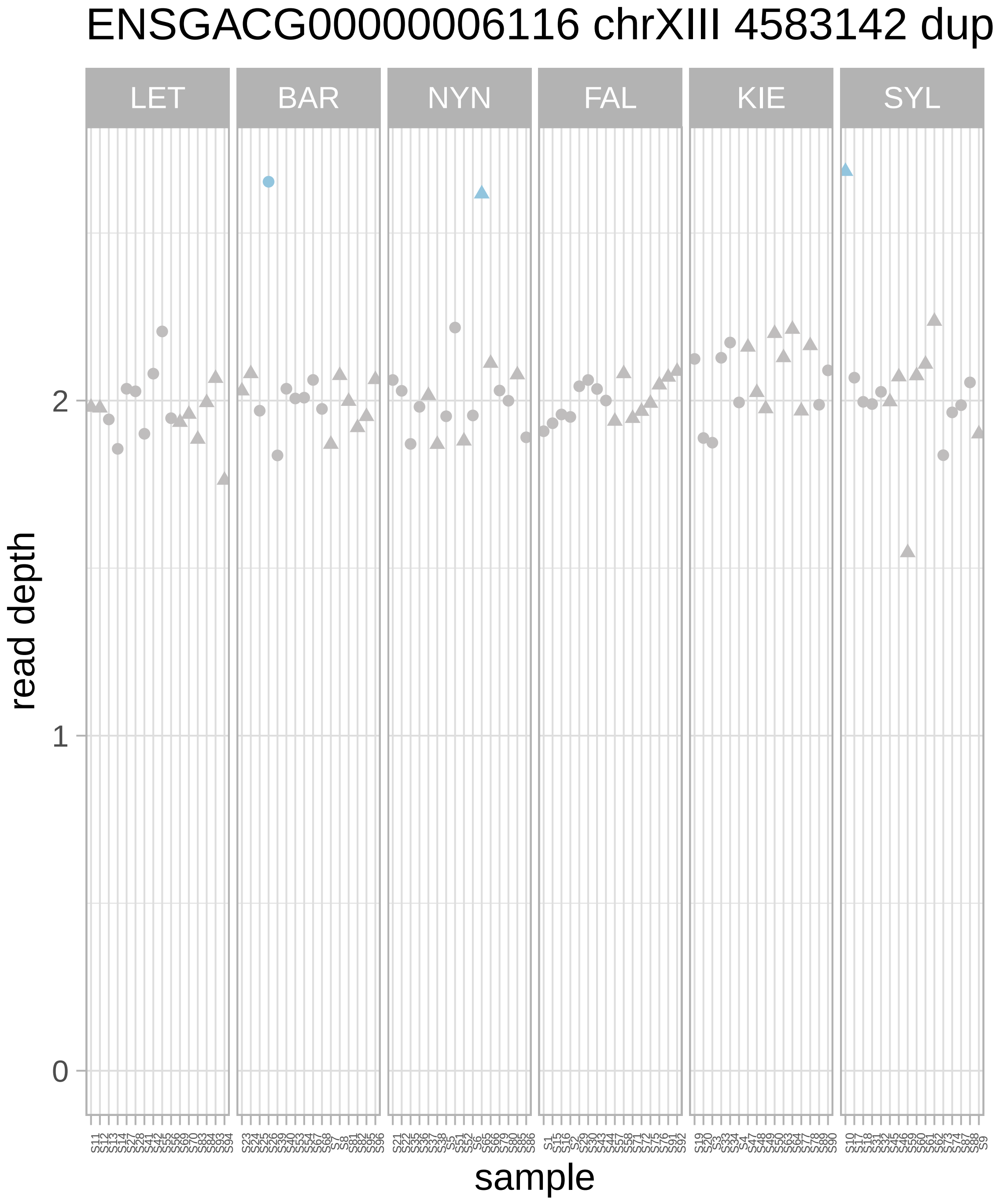
ENSGACG00000006116 chrXIII 4583142 dup
LET
BAR
NYN
FAL
KIE
SYL
read depth
2
1
0
sample

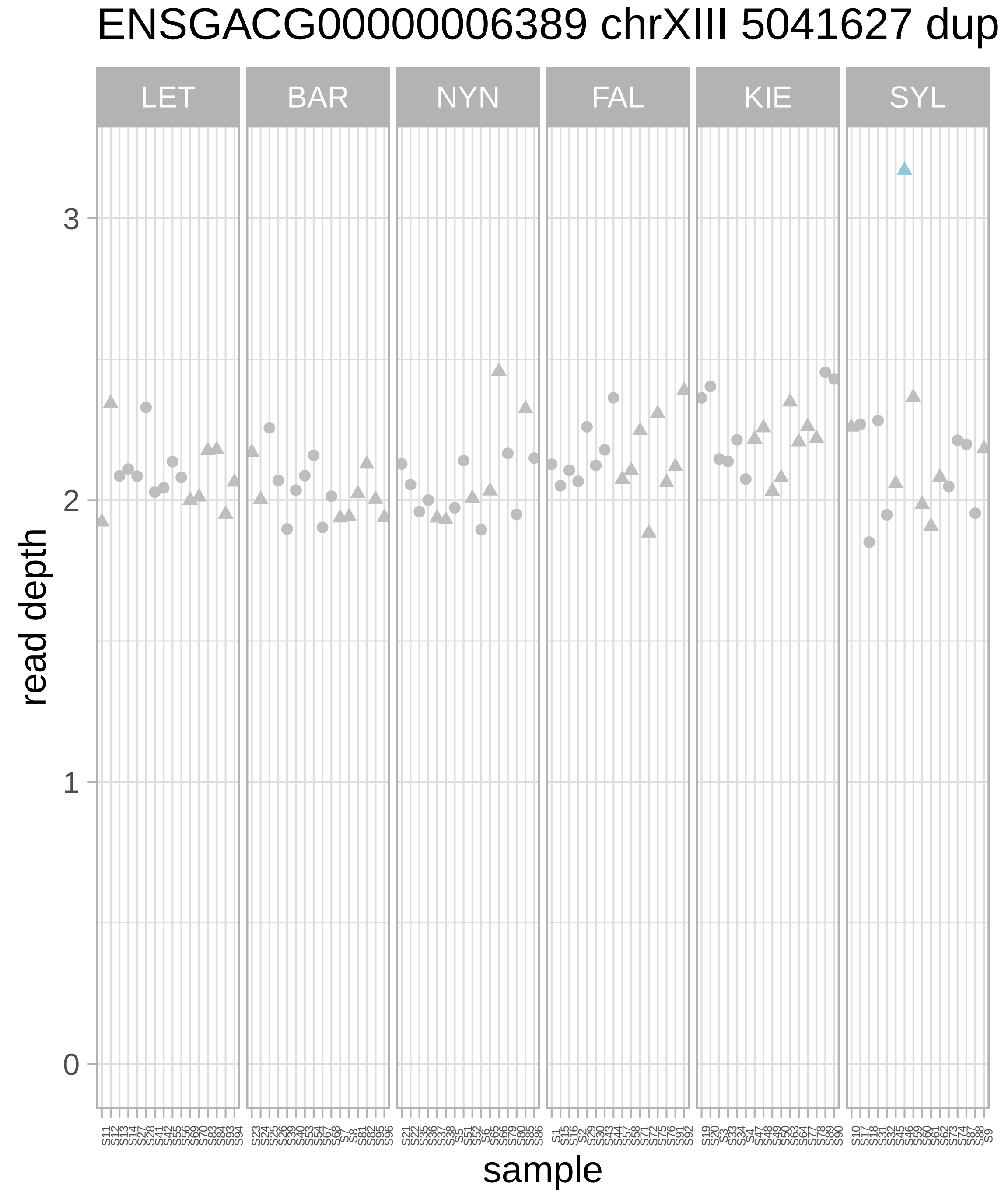
ENSGACG00000006389 chrXIII 5041627 dup
LET
BAR
NYN
FAL
KIE
SYL
read depth
3
2
1
0
sample

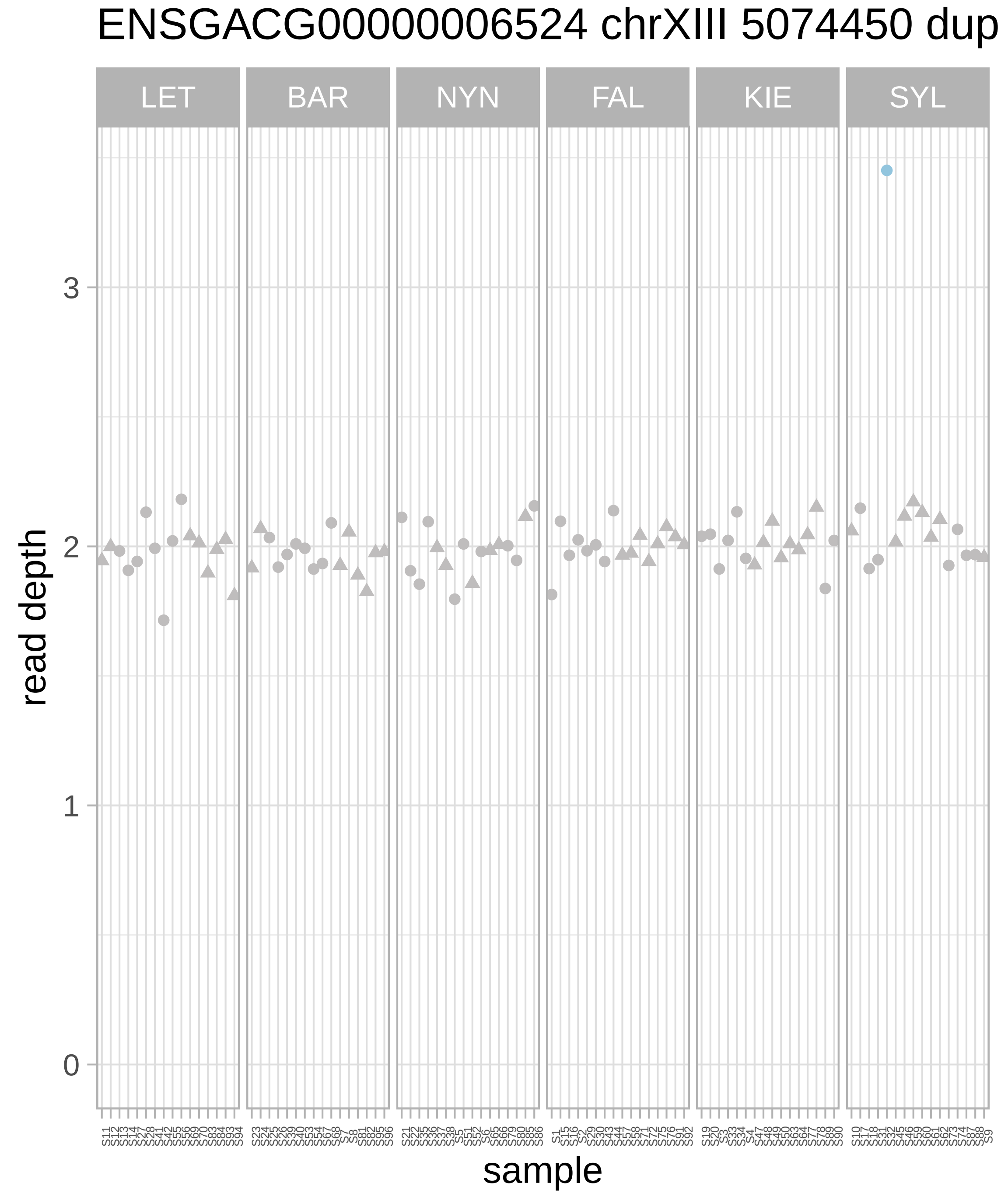
ENSGACG00000006524 chrXIII 5074450 dup
LET
BAR
NYN
FAL
KIE
SYL
read depth
3
2
1
0
S11 S12 S13 S14 S27 S28 S41 S42 S55 S56 S69 S70 S83 S84 S93 S94
S23 S24 S25 S26 S39 S40 S53 S54 S67 S68 S7 S8 S81 S82 S95 S96
S21 S22 S35 S36 S37 S38 S5 S51 S52 S6 S65 S66 S79 S80 S85 S86
S1 S15 S16 S2 S29 S30 S43 S44 S57 S58 S71 S72 S75 S76 S91 S92
S19 S20 S3 S33 S34 S4 S47 S48 S49 S50 S63 S64 S77 S78 S89 S90
S10 S17 S18 S31 S32 S45 S46 S59 S60 S61 S62 S73 S74 S87 S88 S9
sample

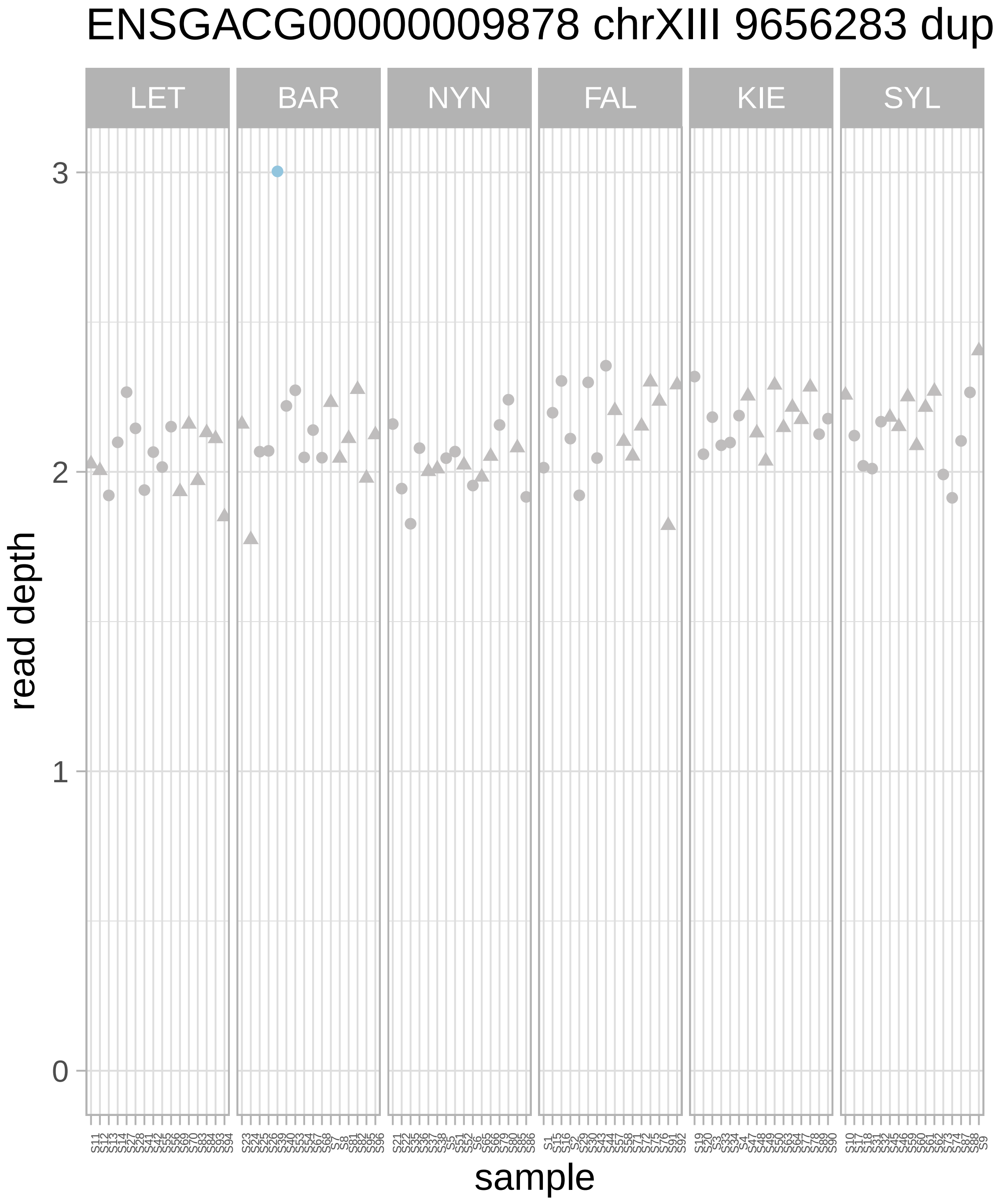
ENSGACG00000009878 chrXIII 9656283 dup
LET
BAR
NYN
FAL
KIE
SYL
read depth
0
1
2
3
sample
S11 S12 S13 S14 S27 S28 S41 S42 S55 S56 S69 S70 S83 S84 S93 S94
S23 S24 S25 S26 S39 S40 S53 S54 S67 S68 S7 S8 S81 S82 S95 S96
S21 S22 S35 S36 S37 S38 S5 S51 S52 S6 S65 S66 S79 S80 S85 S86
S1 S15 S16 S2 S29 S30 S43 S44 S57 S58 S71 S72 S75 S76 S91 S92
S19 S20 S3 S33 S34 S4 S47 S48 S49 S50 S63 S64 S77 S78 S89 S90
S10 S17 S18 S31 S32 S45 S46 S59 S60 S61 S62 S73 S74 S87 S88 S9

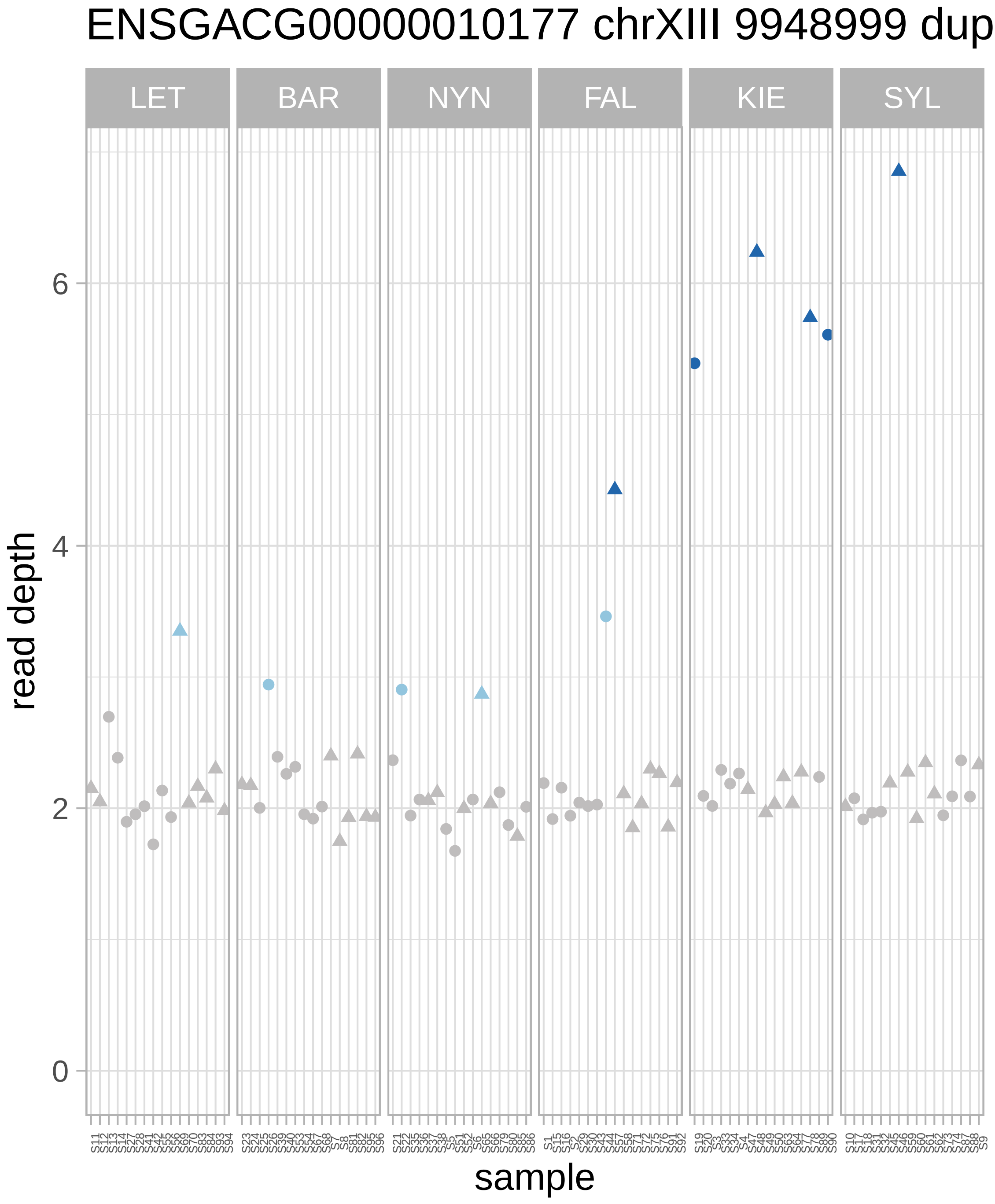
ENSGACG00000010177 chrXIII 9948999 dup
LET
BAR
NYN
FAL
KIE
SYL
read depth
0
2
4
6
S11 S12 S13 S14 S27 S28 S41 S42 S55 S56 S69 S70 S83 S84 S93 S94
S23 S24 S25 S26 S39 S40 S53 S54 S67 S68 S7 S8 S81 S82 S95 S96
S21 S22 S35 S36 S37 S38 S5 S51 S52 S6 S65 S66 S79 S80 S85 S86
S1 S15 S16 S2 S29 S30 S43 S44 S57 S58 S71 S72 S75 S76 S91 S92
S19 S20 S3 S33 S34 S4 S47 S48 S49 S50 S63 S64 S77 S78 S89 S90
S10 S17 S18 S31 S32 S45 S46 S59 S60 S61 S62 S73 S74 S87 S88 S9
sample

ENSGACG00000010182 chrXIII 9952935 dup

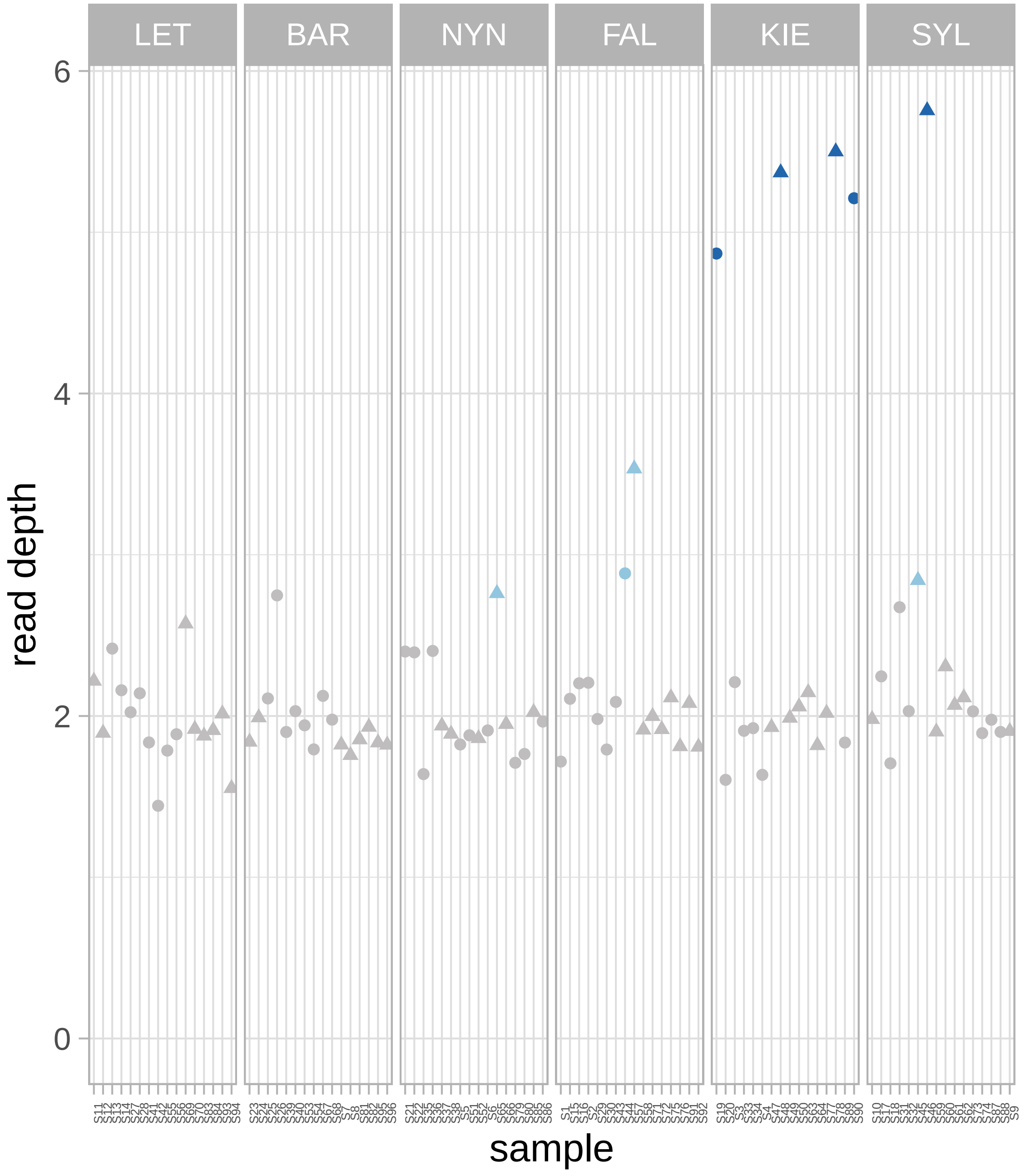

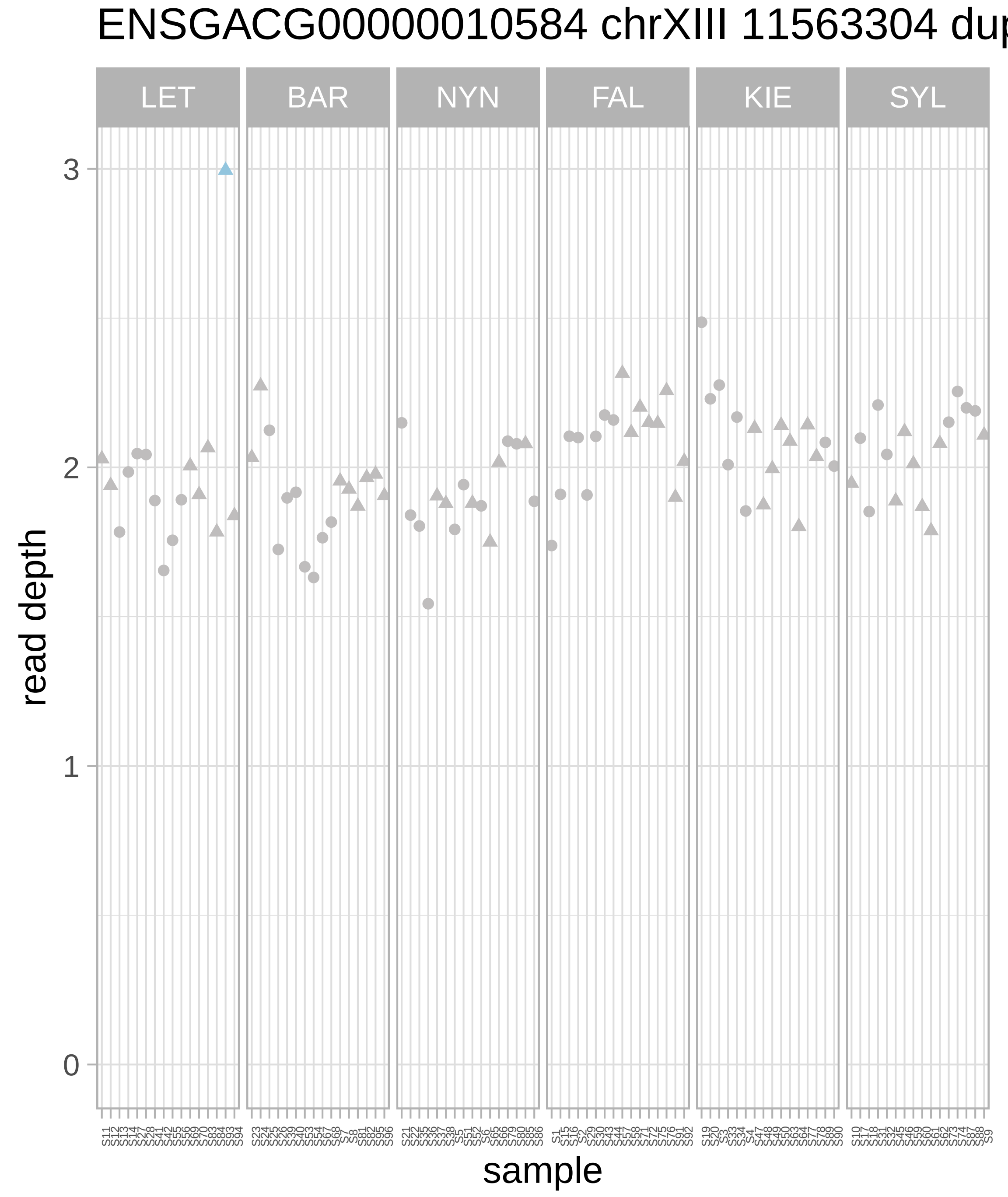
ENSGACG00000010584 chrXIII 11563304 dup
LET
BAR
NYN
FAL
KIE
SYL
3
2
1
0
read depth
sample

ENSGACG00000011195 chrXIII 12419766 dup

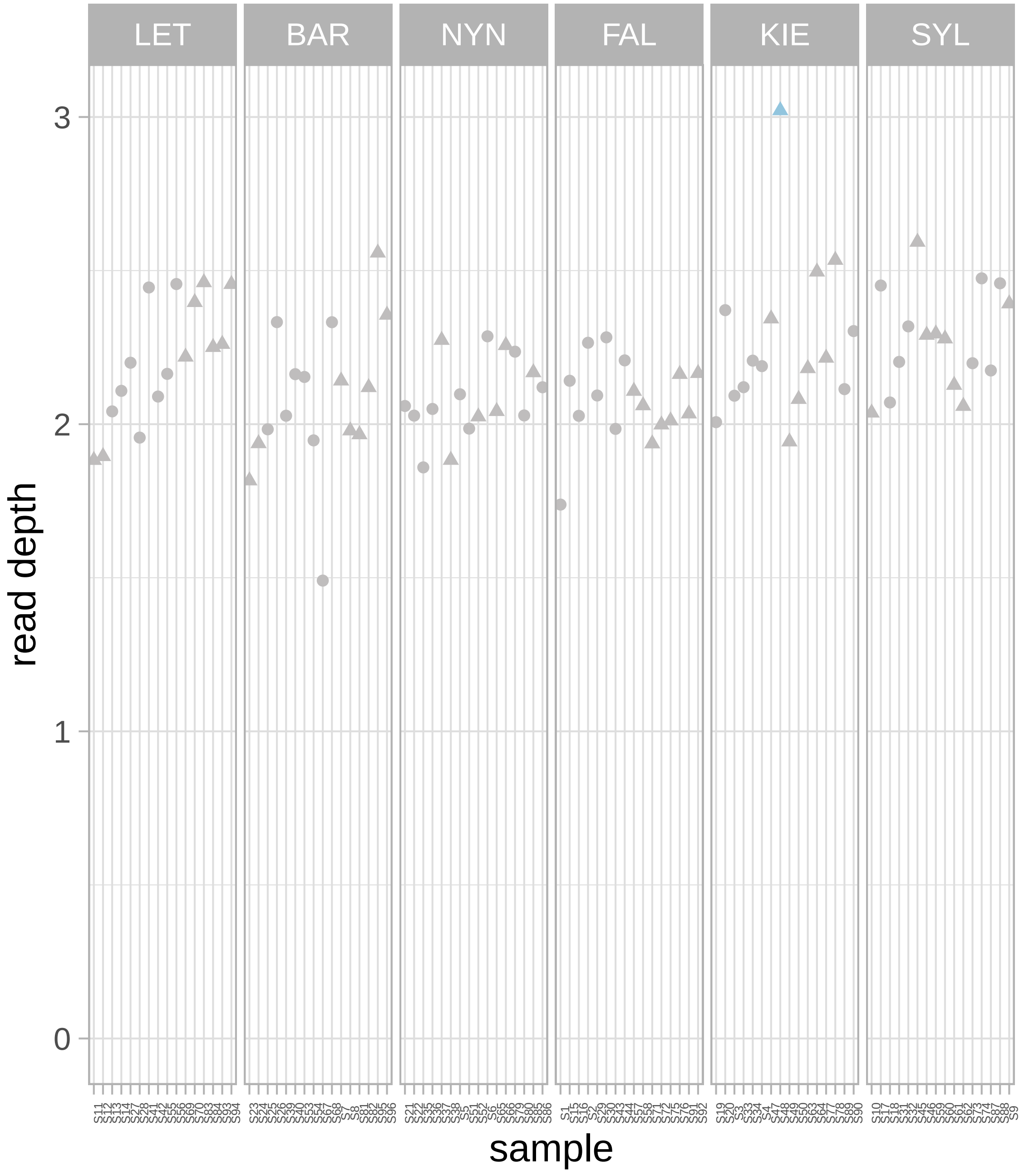

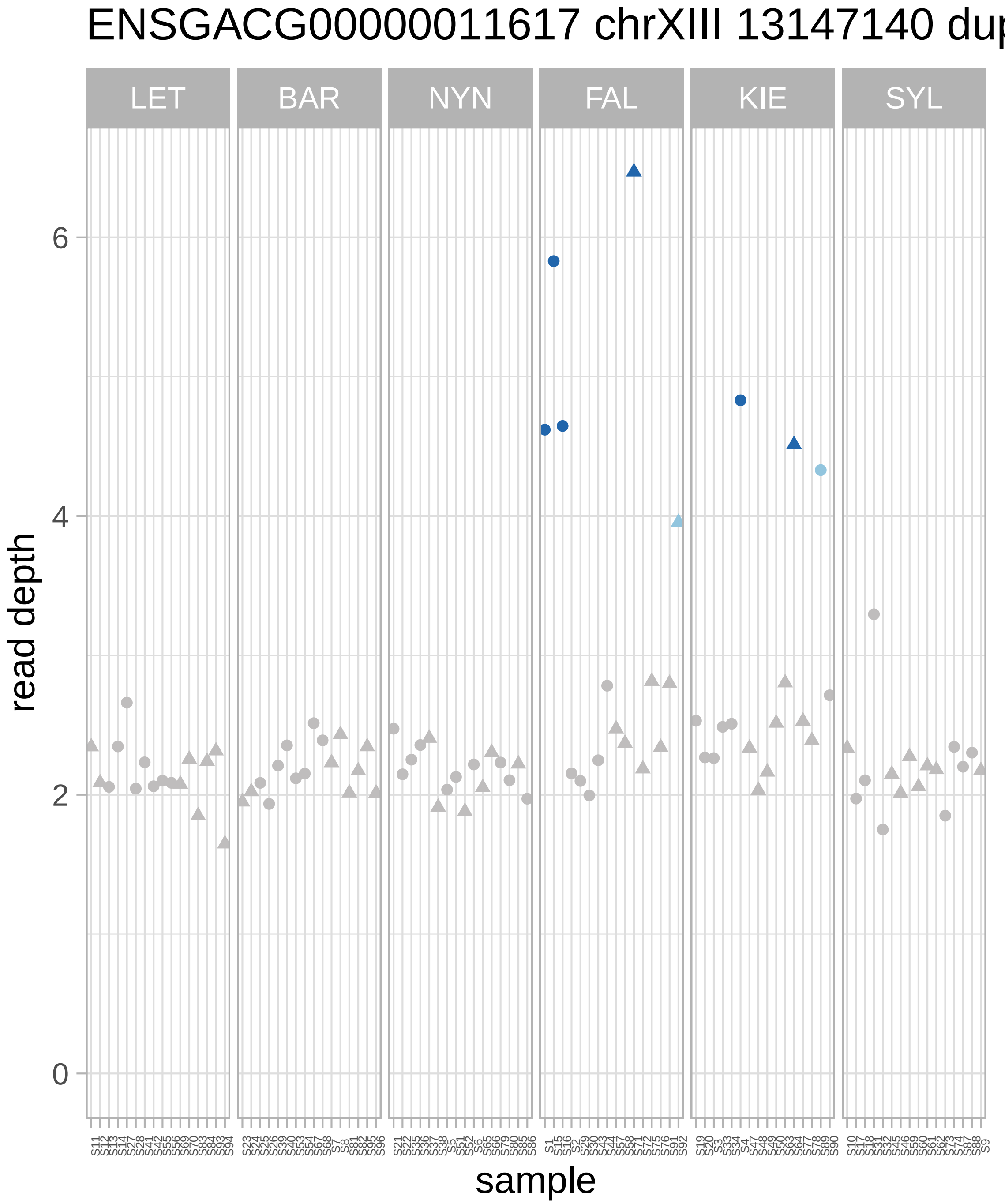
ENSGACG00000011617 chrXIII 13147140 dup
LET
BAR
NYN
FAL
KIE
SYL
read depth
0
2
4
6
sample

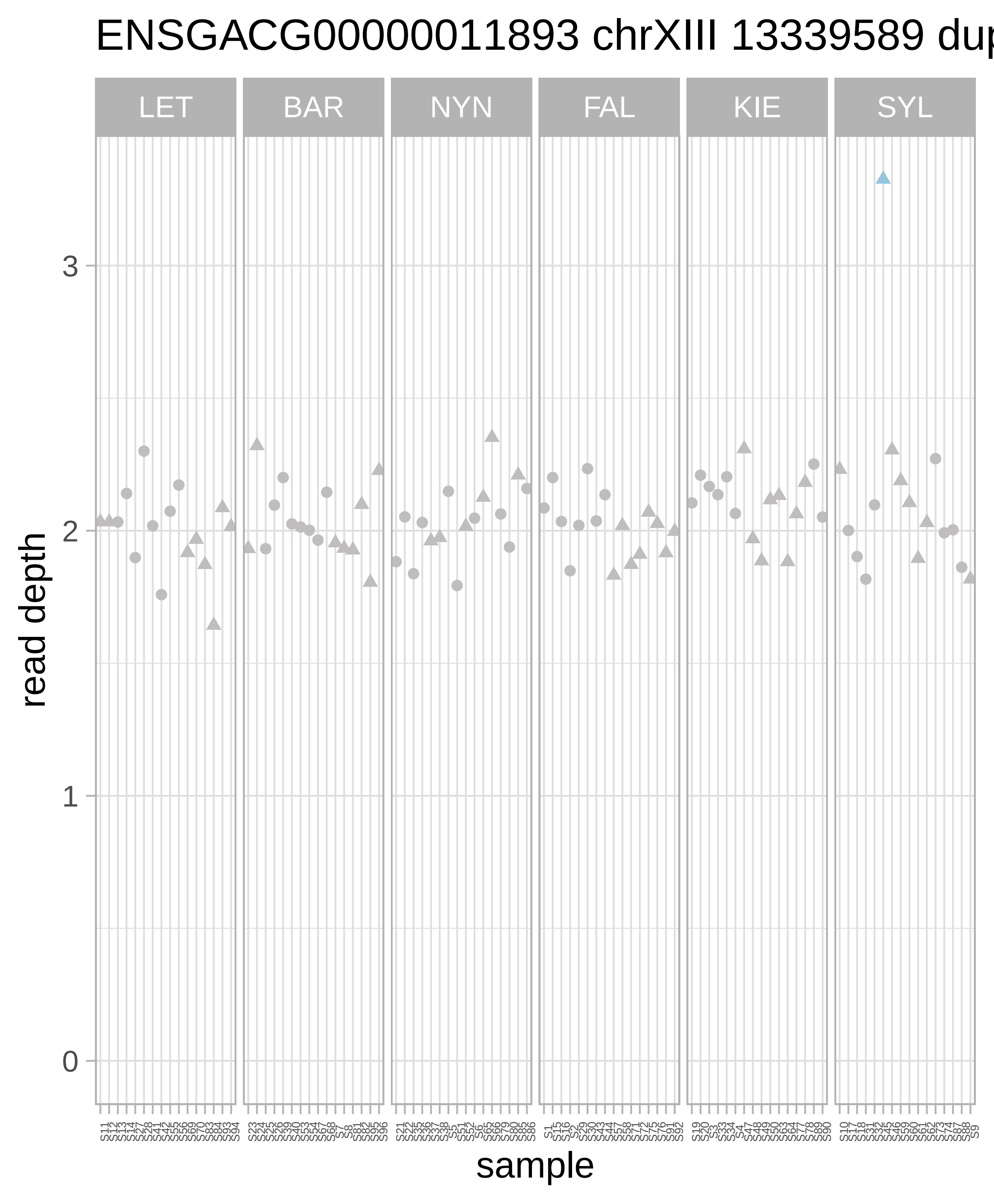
ENSGACG00000011893 chrXIII 13339589 dup
LET
BAR
NYN
FAL
KIE
SYL
read depth
3
2
1
0
sample

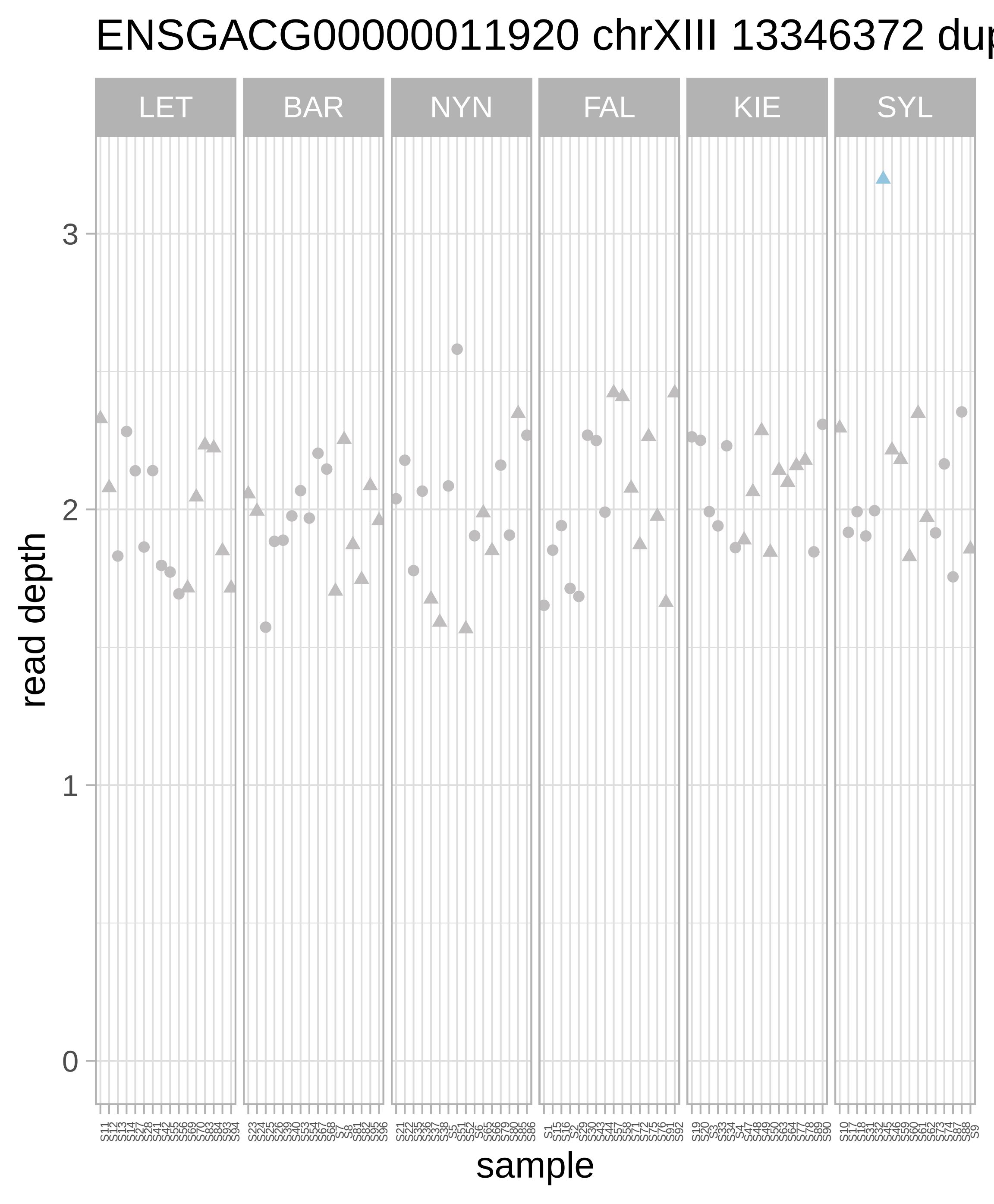
ENSGACG00000011920 chrXIII 13346372 dup
LET
BAR
NYN
FAL
KIE
SYL
read depth
0
1
2
3
sample

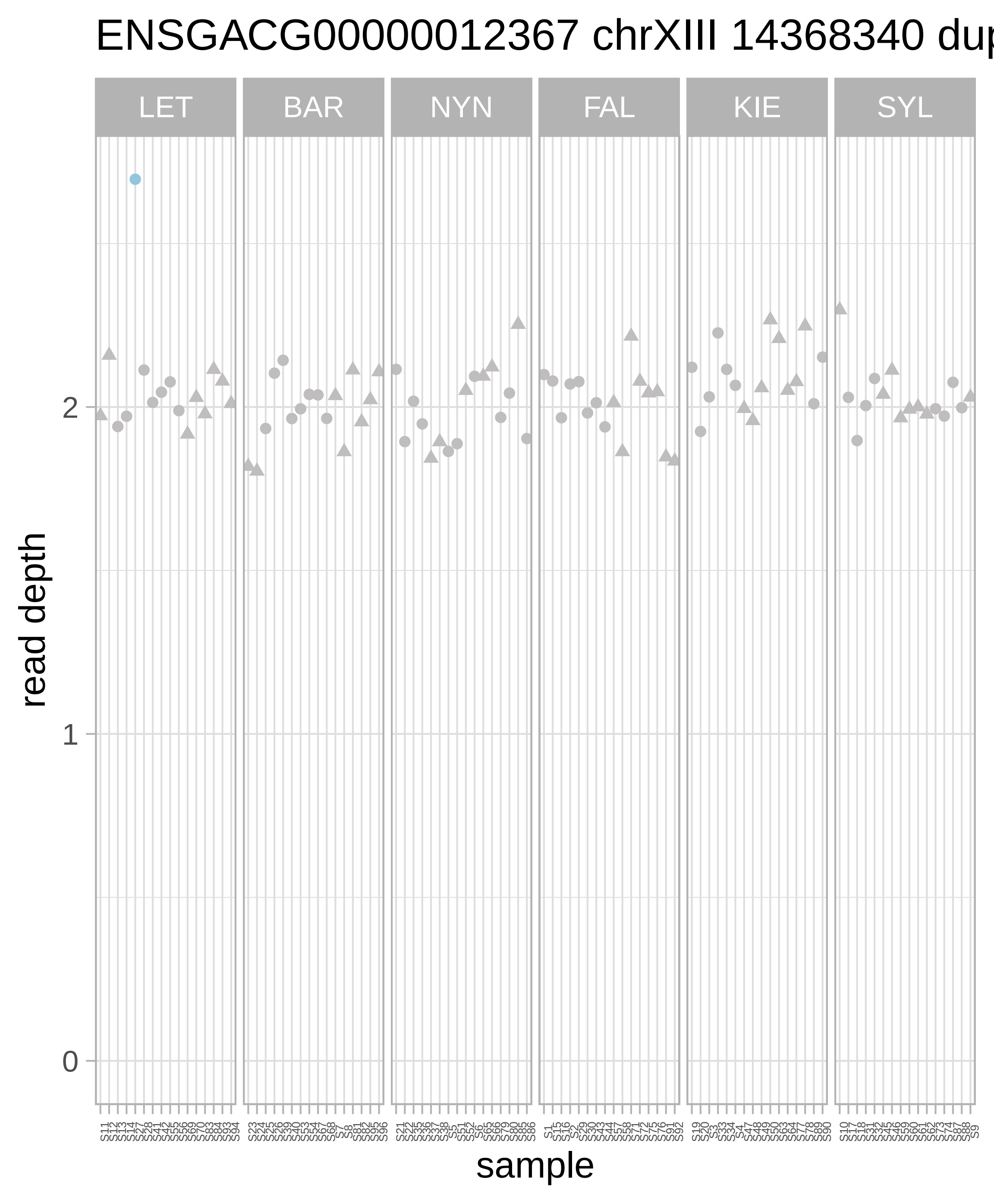
ENSGACG00000012367 chrXIII 14368340 dup
LET
BAR
NYN
FAL
KIE
SYL
read depth
2
1
0
sample

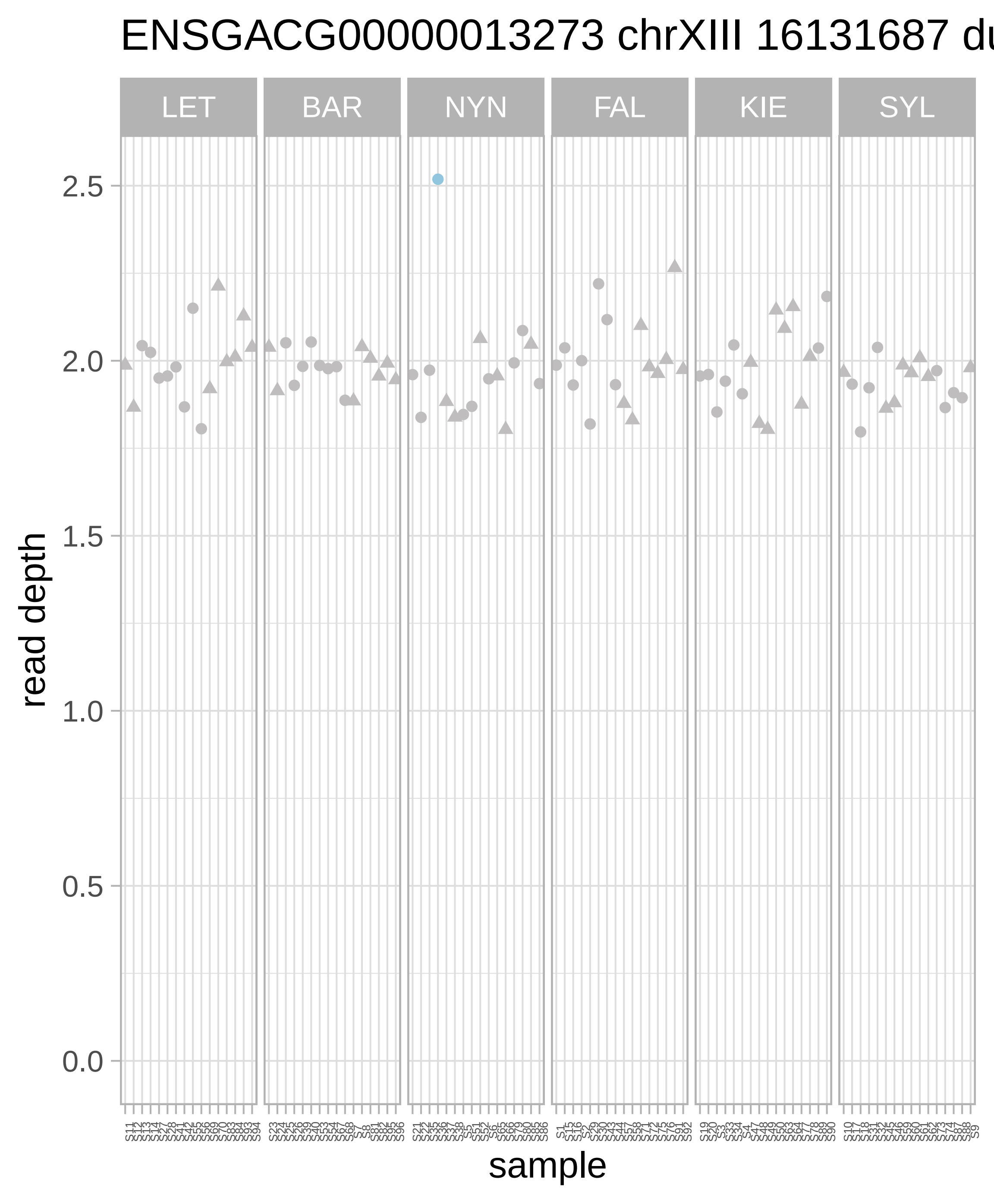
ENSGACG00000013273 chrXIII 16131687 du
LET
BAR
NYN
FAL
KIE
SYL
2.5
2.0
1.5
1.0
0.5
0.0
read depth
sample

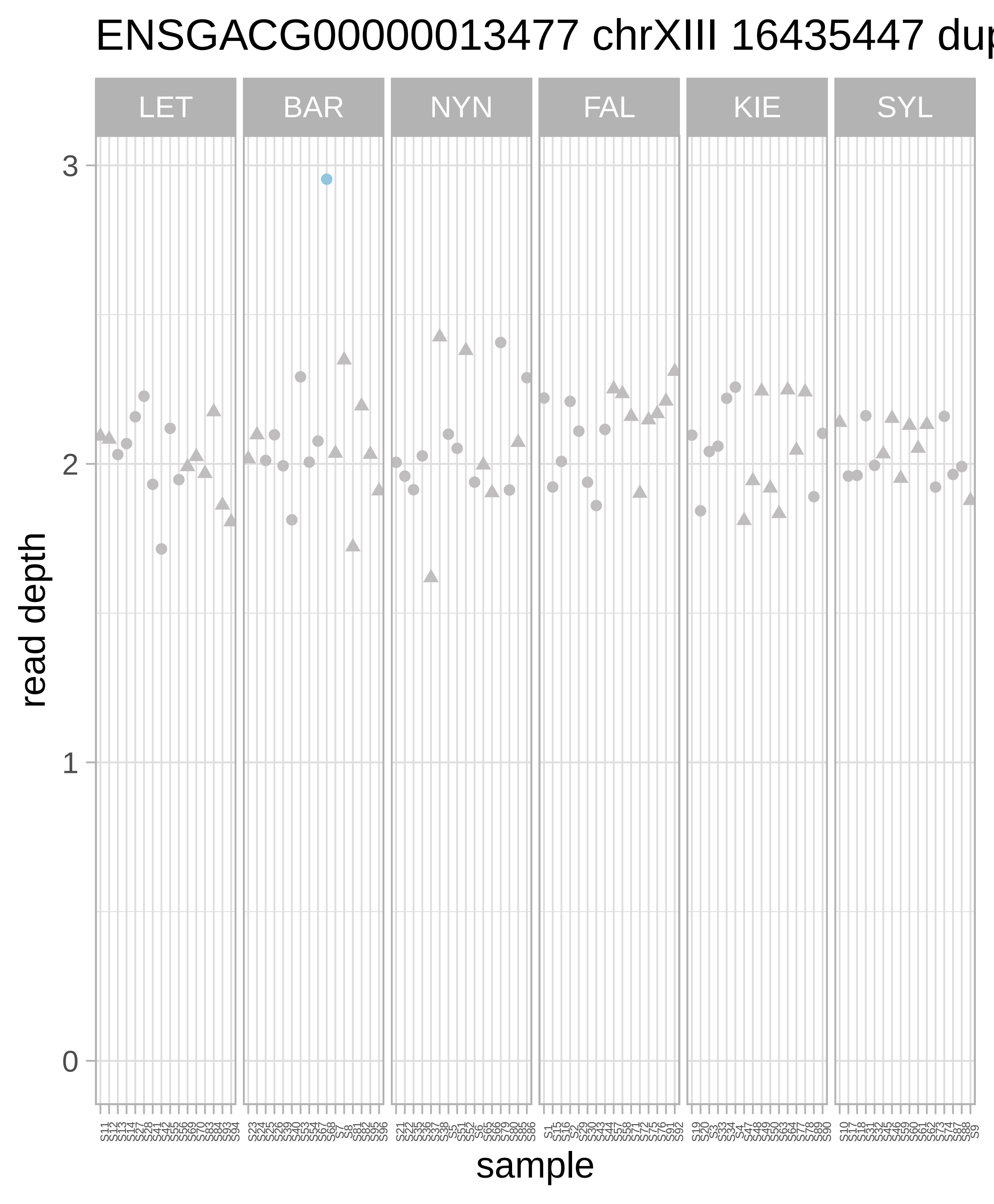
ENSGACG00000013477 chrXIII 16435447 dup
LET
BAR
NYN
FAL
KIE
SYL
read depth
0
1
2
3
sample
S11 S12 S13 S14 S27 S28 S41 S42 S55 S56 S69 S70 S83 S84 S93 S94
S23 S24 S25 S26 S39 S40 S53 S54 S67 S68 S7 S8 S81 S82 S95 S96
S21 S22 S35 S36 S37 S38 S5 S51 S52 S6 S65 S66 S79 S80 S85 S86
S1 S15 S16 S2 S29 S30 S43 S44 S57 S58 S71 S72 S75 S76 S91 S92
S19 S20 S3 S33 S34 S4 S47 S48 S49 S50 S63 S64 S77 S78 S89 S90
S10 S17 S18 S31 S32 S45 S46 S59 S60 S61 S62 S73 S74 S87 S88 S9

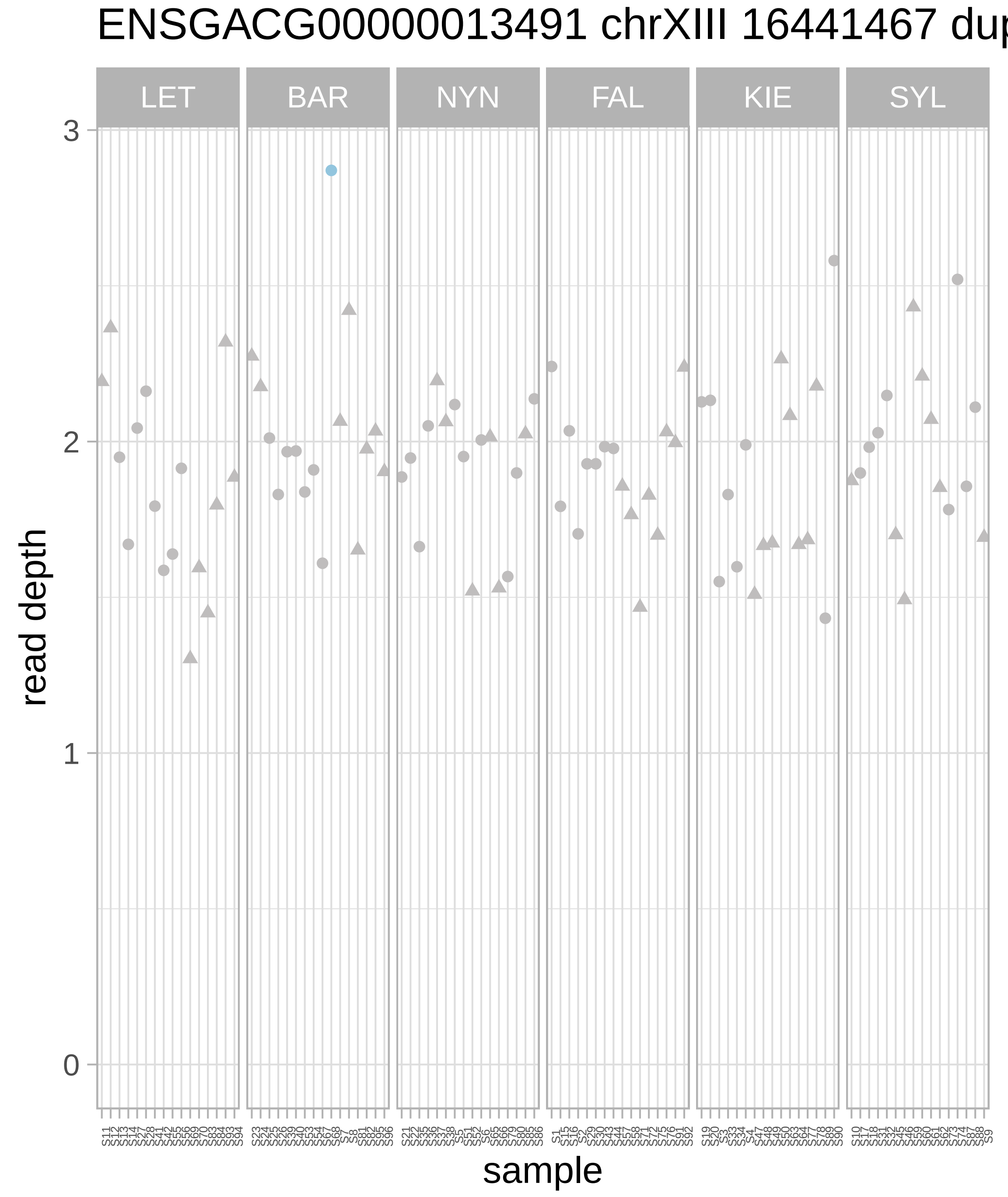
ENSGACG00000013491 chrXIII 16441467 dup
LET
BAR
NYN
FAL
KIE
SYL
read depth
0
1
2
3
sample

ENSGACG00000013652 chrXIII 16756156 du

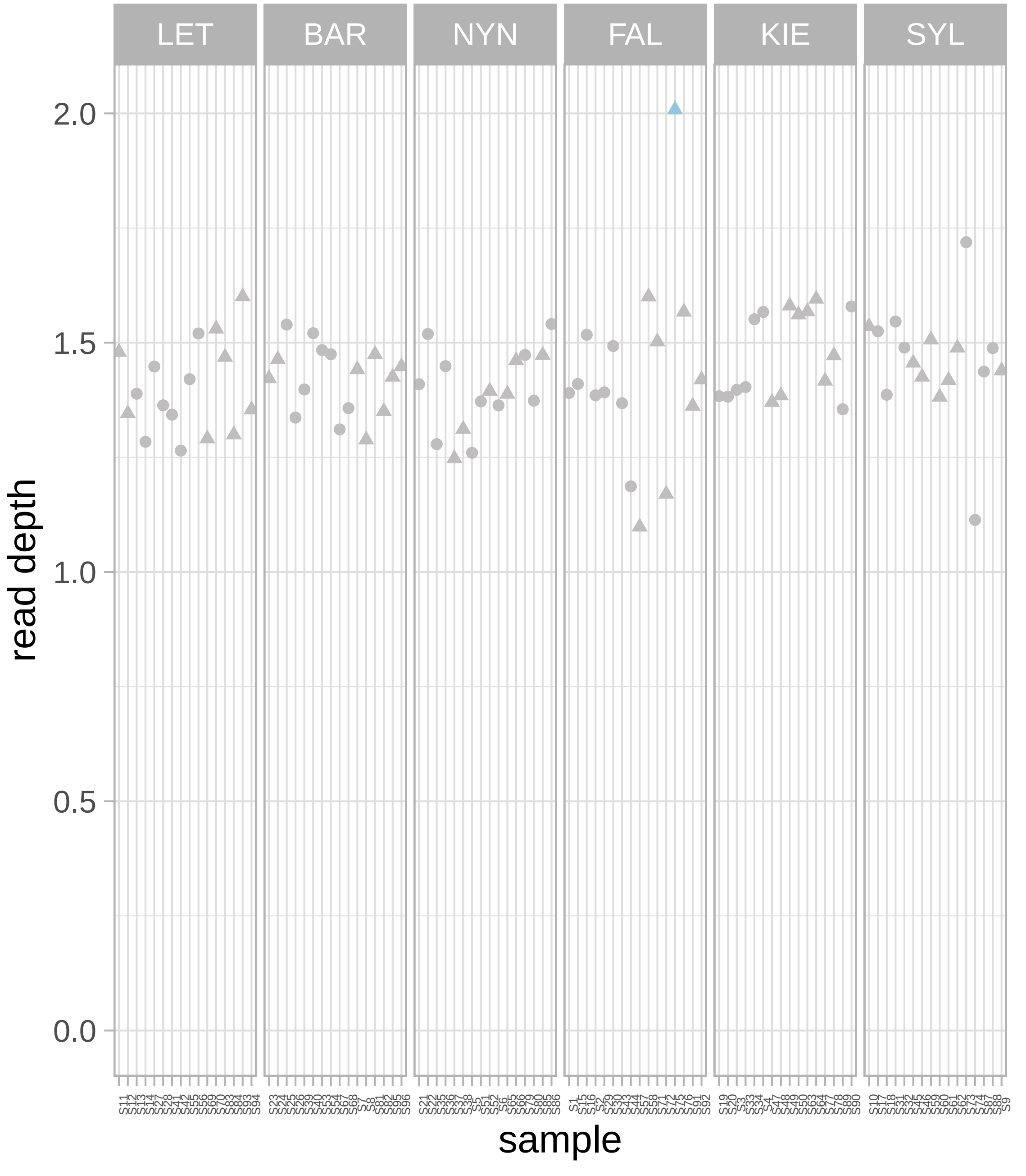

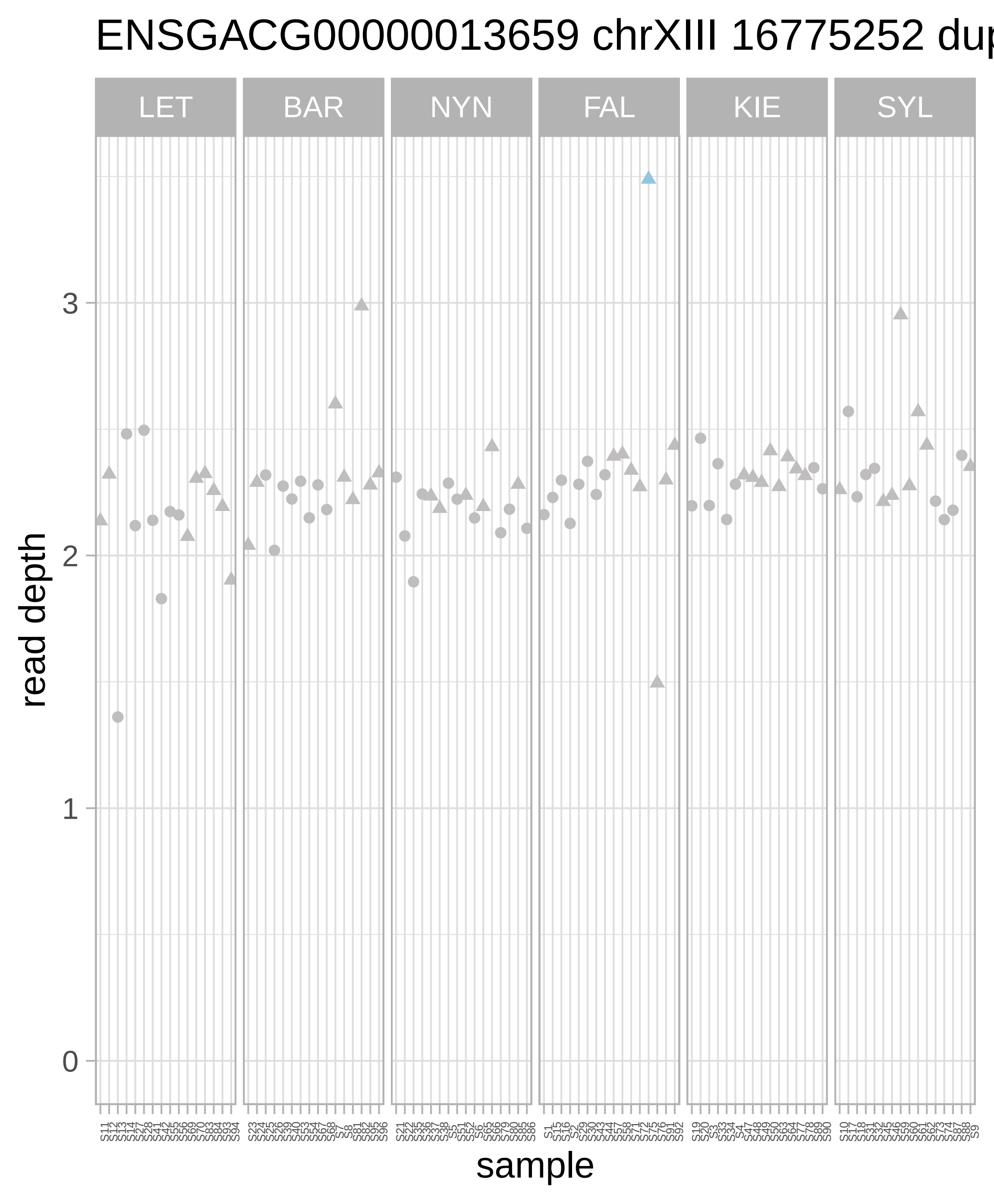

ENSGACG00000013659 chrXIII 16775252 dup
LET
BAR
NYN
FAL
KIE
SYL
read depth
0
1
2
3
sample

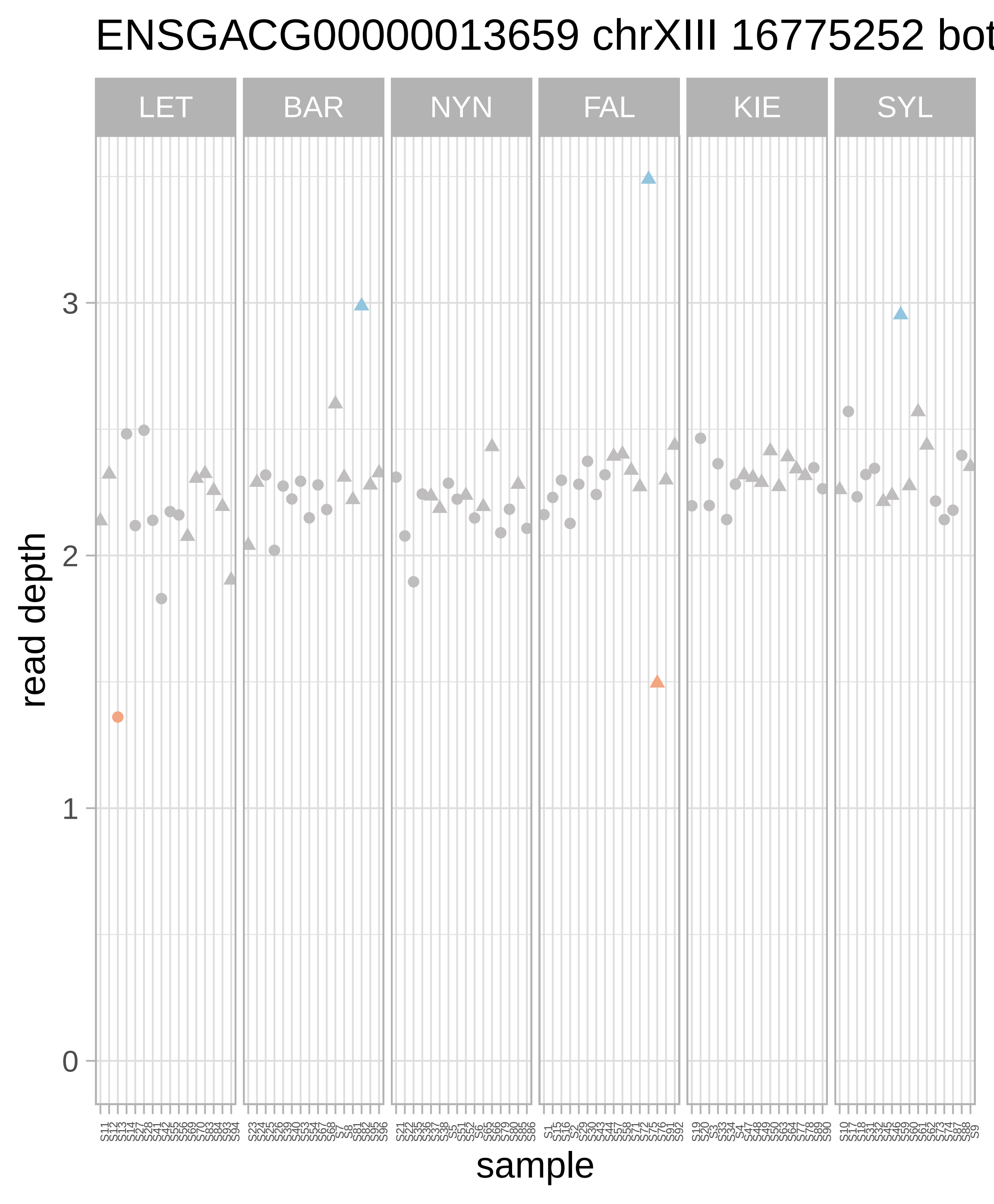
ENSGACG00000013659 chrXIII 16775252 bot
LET
BAR
NYN
FAL
KIE
SYL
read depth
0
1
2
3
sample

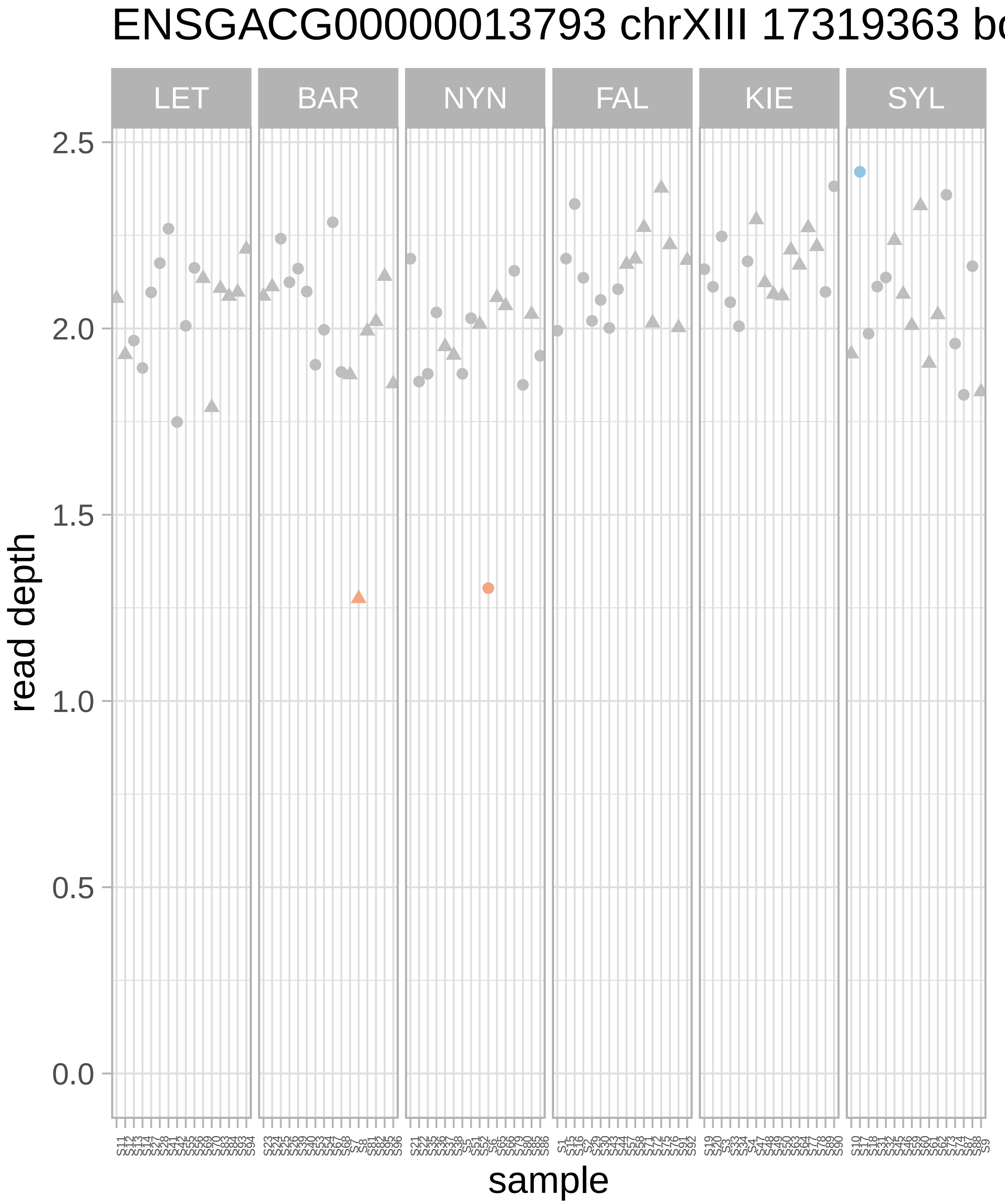
ENSGACG00000013793 chrXIII 17319363 bc
LET
BAR
NYN
FAL
KIE
SYL
read depth
2.5
2.0
1.5
1.0
0.5
0.0
S11 S12 S13 S14 S27 S28 S41 S42 S55 S56 S69 S70 S83 S84 S93 S94
S23 S24 S25 S26 S39 S40 S53 S54 S67 S68 S7 S8 S81 S82 S95 S96
S21 S22 S35 S36 S37 S38 S5 S51 S52 S6 S65 S66 S79 S80 S85 S86
S1 S15 S16 S2 S29 S30 S43 S44 S57 S58 S71 S72 S75 S76 S91 S92
S19 S20 S3 S33 S34 S4 S47 S48 S49 S50 S63 S64 S77 S78 S89 S90
S10 S17 S18 S31 S32 S45 S46 S59 S60 S61 S62 S73 S74 S87 S88 S9
sample

ENSGACG00000013890 chrXIII 17432508 dup

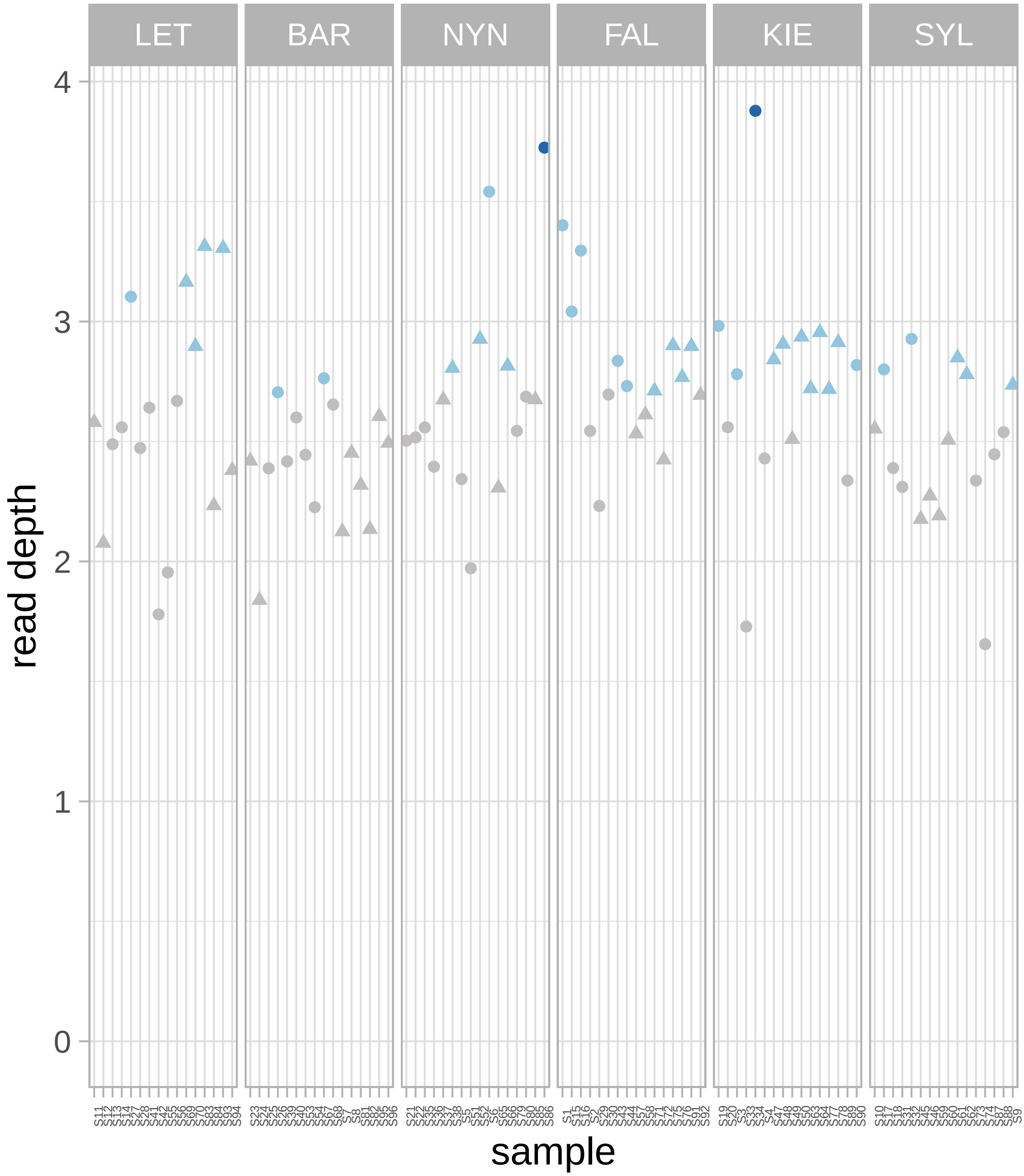

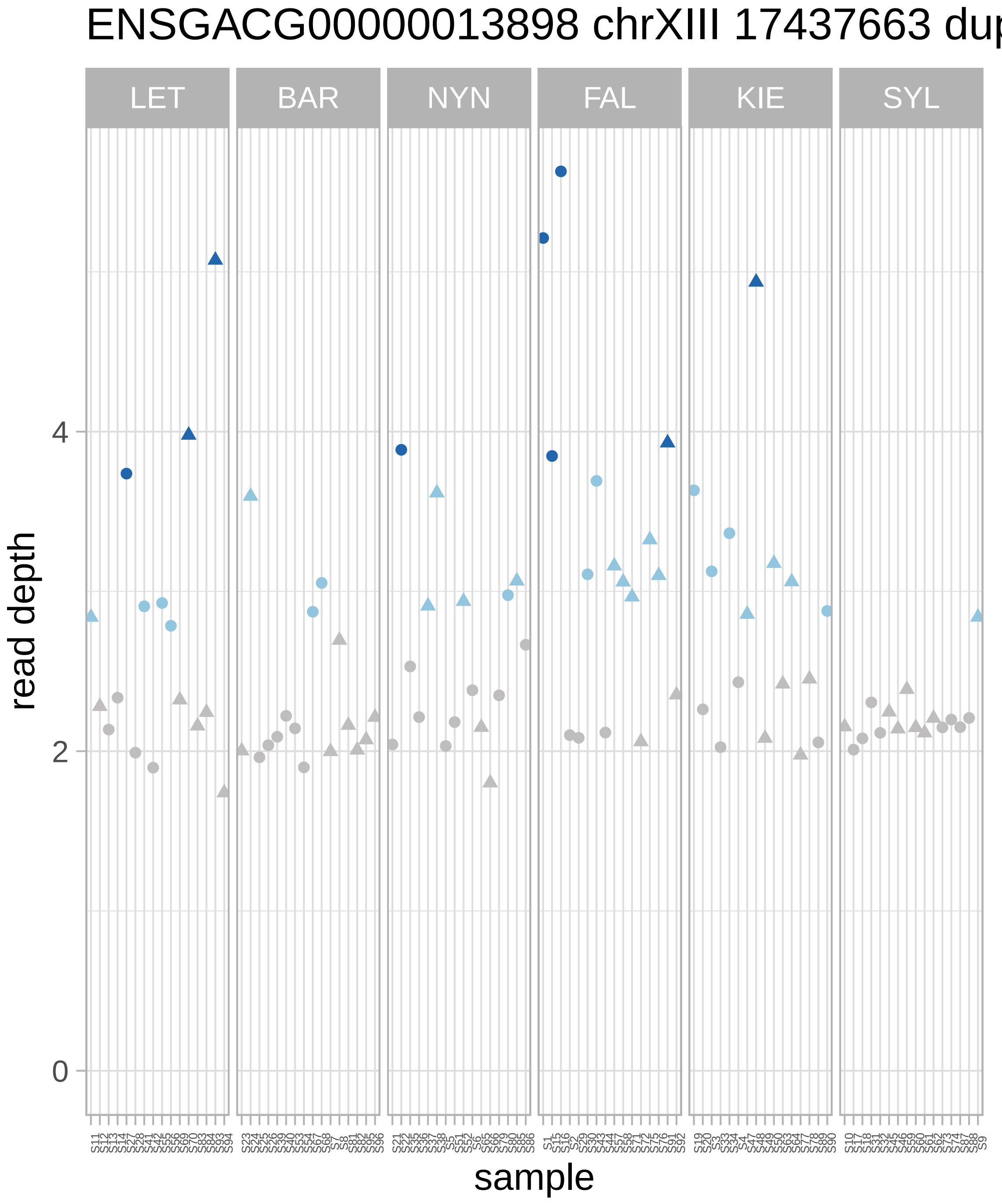
ENSGACG00000013898 chrXIII 17437663 dup
LET
BAR
NYN
FAL
KIE
SYL
read depth
0
2
4
sample
S11 S12 S13 S14 S27 S28 S41 S42 S55 S56 S69 S70 S83 S84 S93 S94
S23 S24 S25 S26 S39 S40 S53 S54 S67 S68 S7 S8 S81 S82 S95 S96
S21 S22 S35 S36 S37 S38 S5 S51 S52 S6 S65 S66 S79 S80 S85 S86
S1 S15 S16 S2 S29 S30 S43 S44 S57 S58 S71 S72 S75 S76 S91 S92
S19 S20 S3 S33 S34 S4 S47 S48 S49 S50 S63 S64 S77 S78 S89 S90
S10 S17 S18 S31 S32 S45 S46 S59 S60 S61 S62 S73 S74 S87 S88 S9

ENSGACG00000014073 chrXIII 18076603 dup

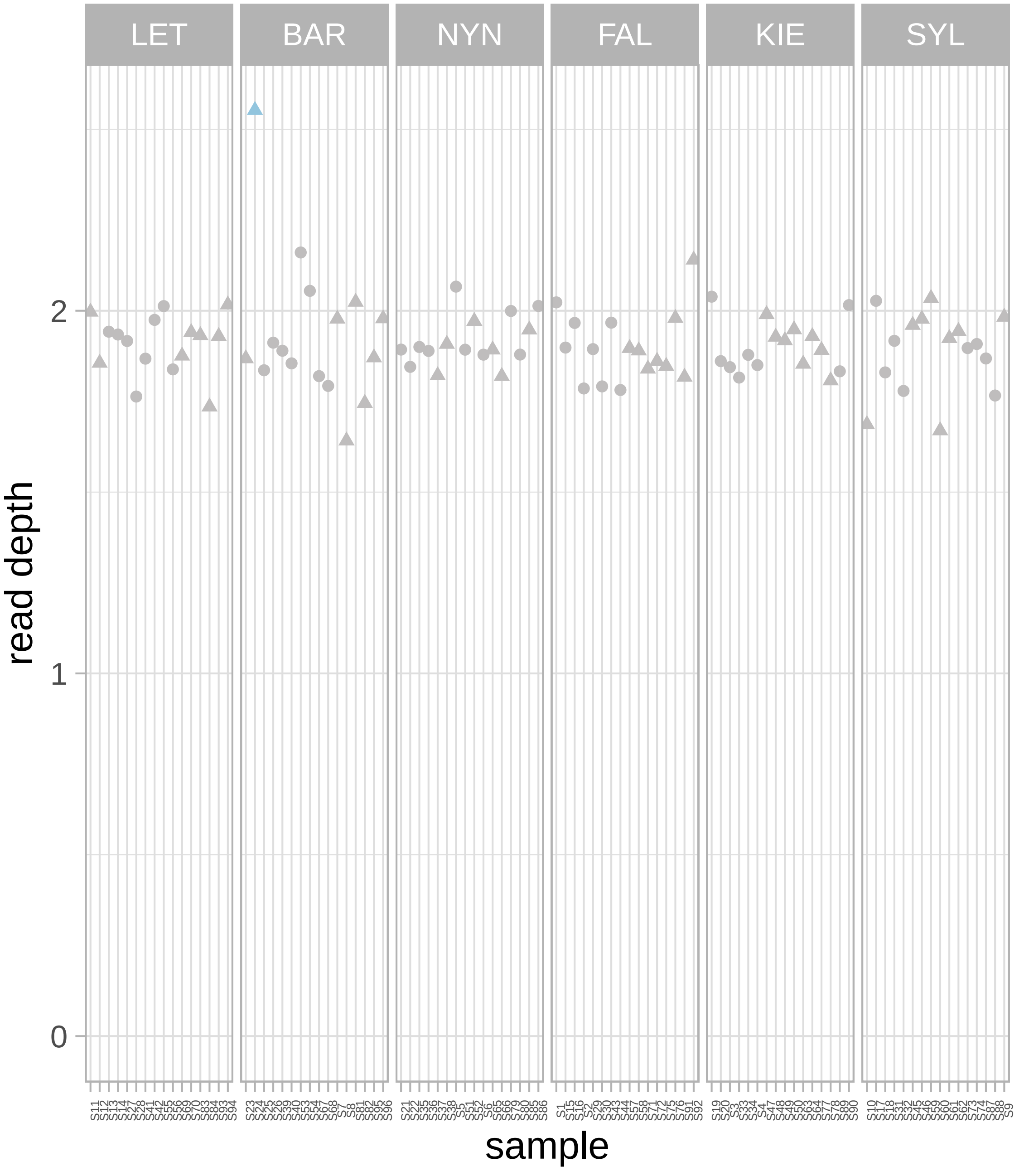

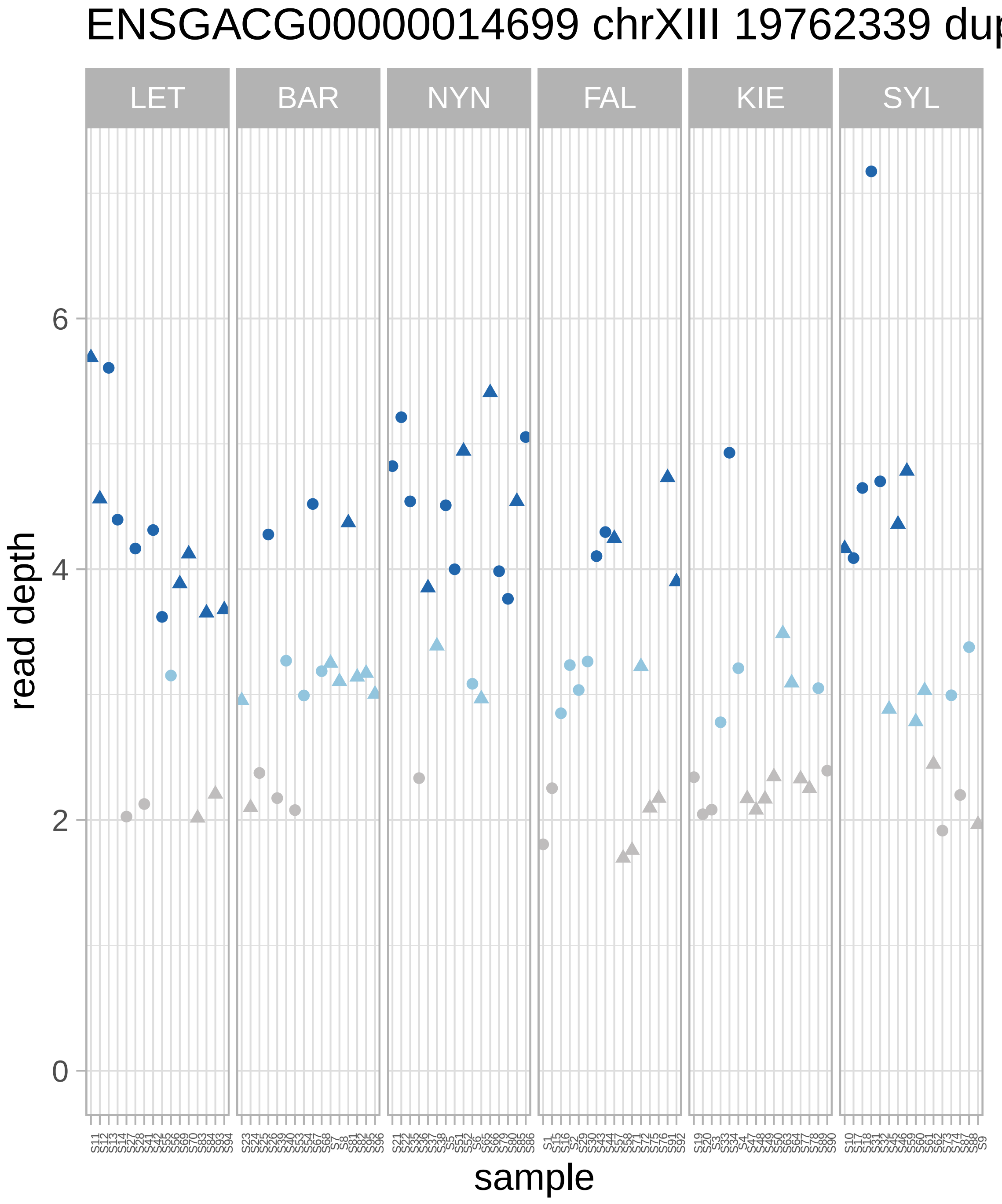
ENSGACG00000014699 chrXIII 19762339 dup
LET
BAR
NYN
FAL
KIE
SYL
read depth
0
2
4
6
sample

ENSGACG00000014767 chrXIII 19955235 dup

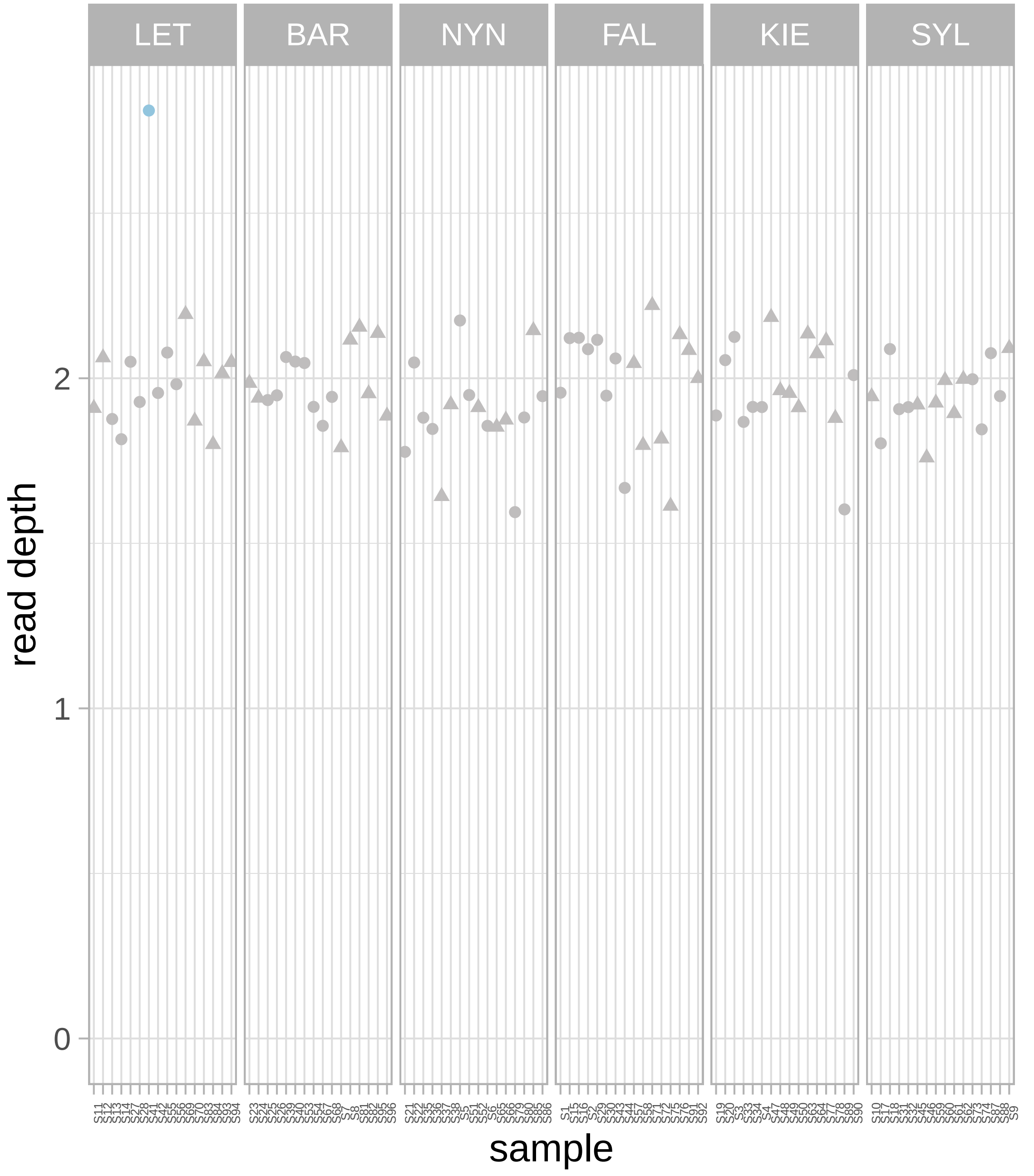

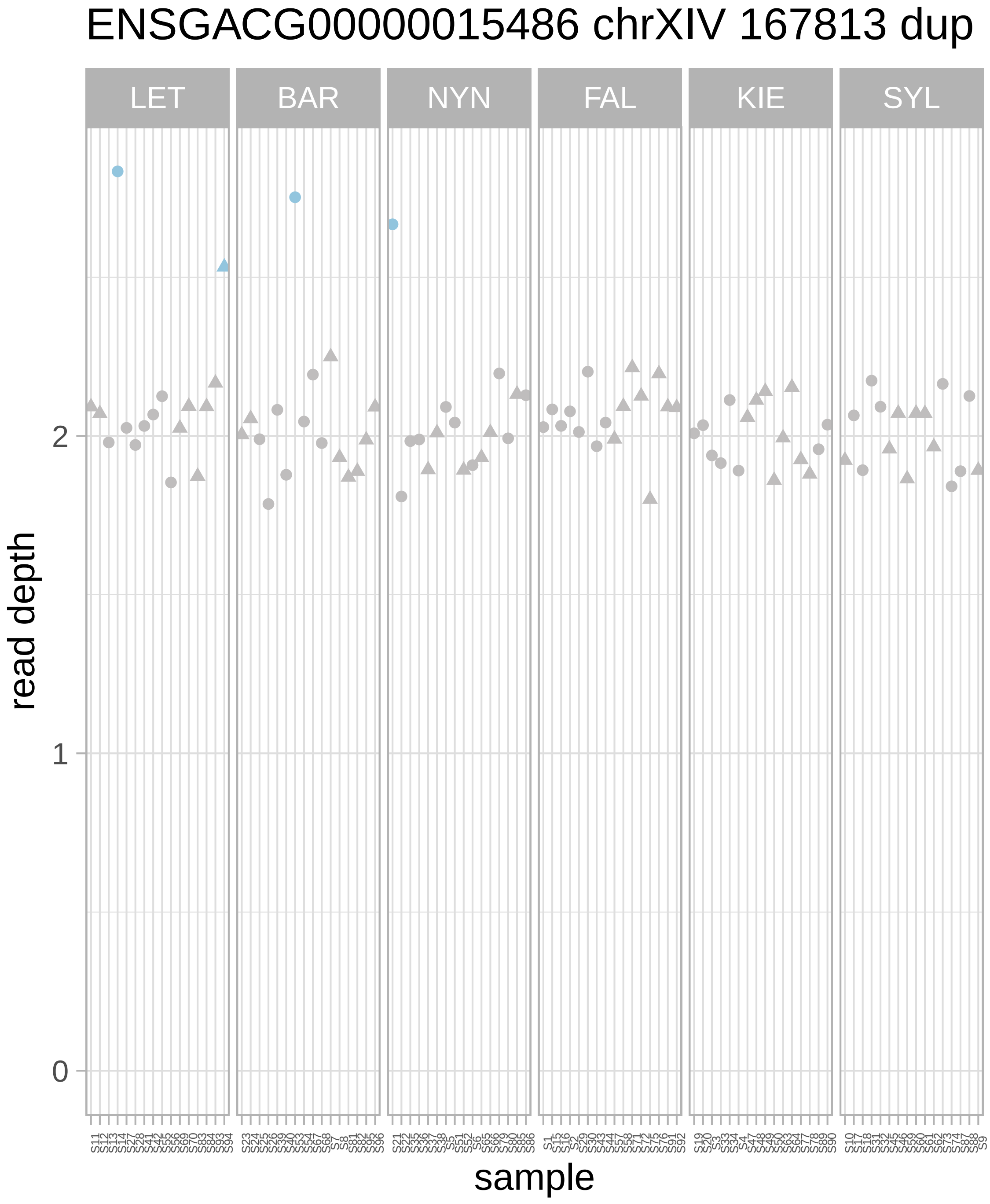
ENSGACG00000015486 chrXIV 167813 dup
LET
BAR
NYN
FAL
KIE
SYL
read depth
0
1
2
S11 S12 S13 S14 S27 S28 S41 S42 S55 S56 S69 S70 S83 S84 S93 S94
S23 S24 S25 S26 S39 S40 S53 S54 S67 S68 S7 S8 S81 S82 S95 S96
S21 S22 S35 S36 S37 S38 S5 S51 S52 S6 S65 S66 S79 S80 S85 S86
S1 S15 S16 S2 S29 S30 S43 S44 S57 S58 S71 S72 S75 S76 S91 S92
S19 S20 S3 S33 S34 S4 S47 S48 S49 S50 S63 S64 S77 S78 S89 S90
S10 S17 S18 S31 S32 S45 S46 S59 S60 S61 S62 S73 S74 S87 S88 S9
sample

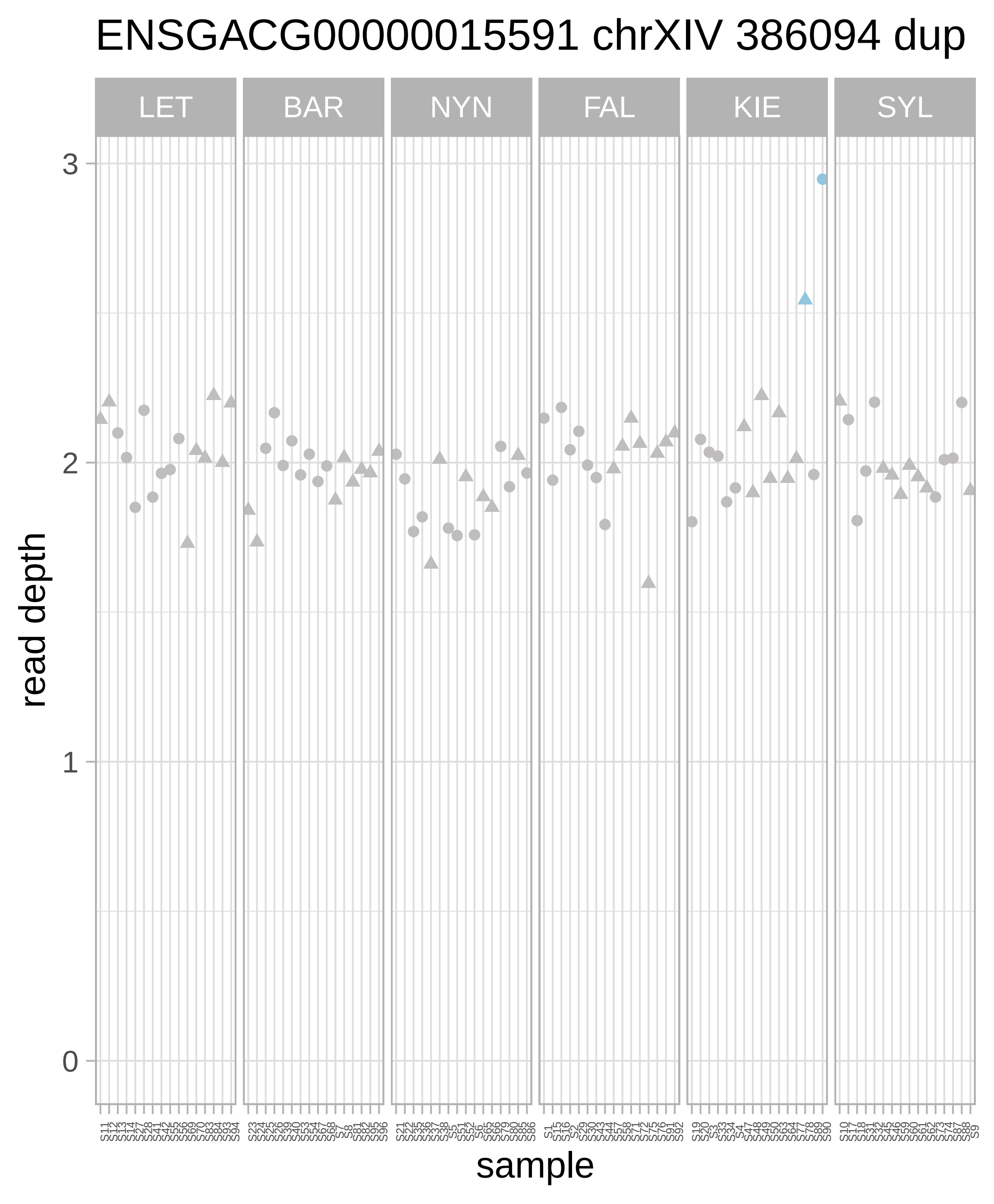
ENSGACG00000015591 chrXIV 386094 dup
LET
BAR
NYN
FAL
KIE
SYL
read depth
0
1
2
3
sample

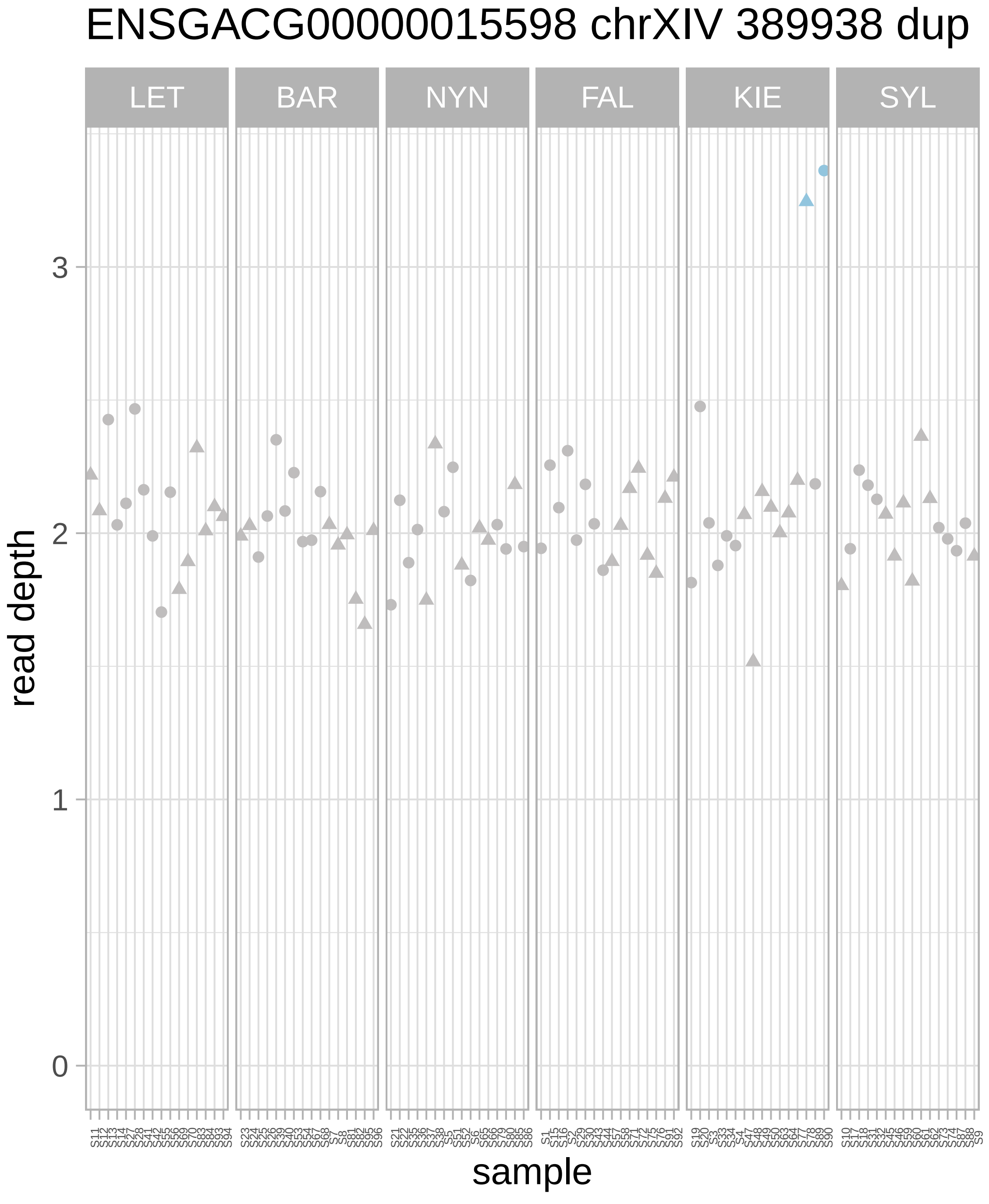
ENSGACG00000015598 chrXIV 389938 dup
LET
BAR
NYN
FAL
KIE
SYL
read depth
3
2
1
0
sample

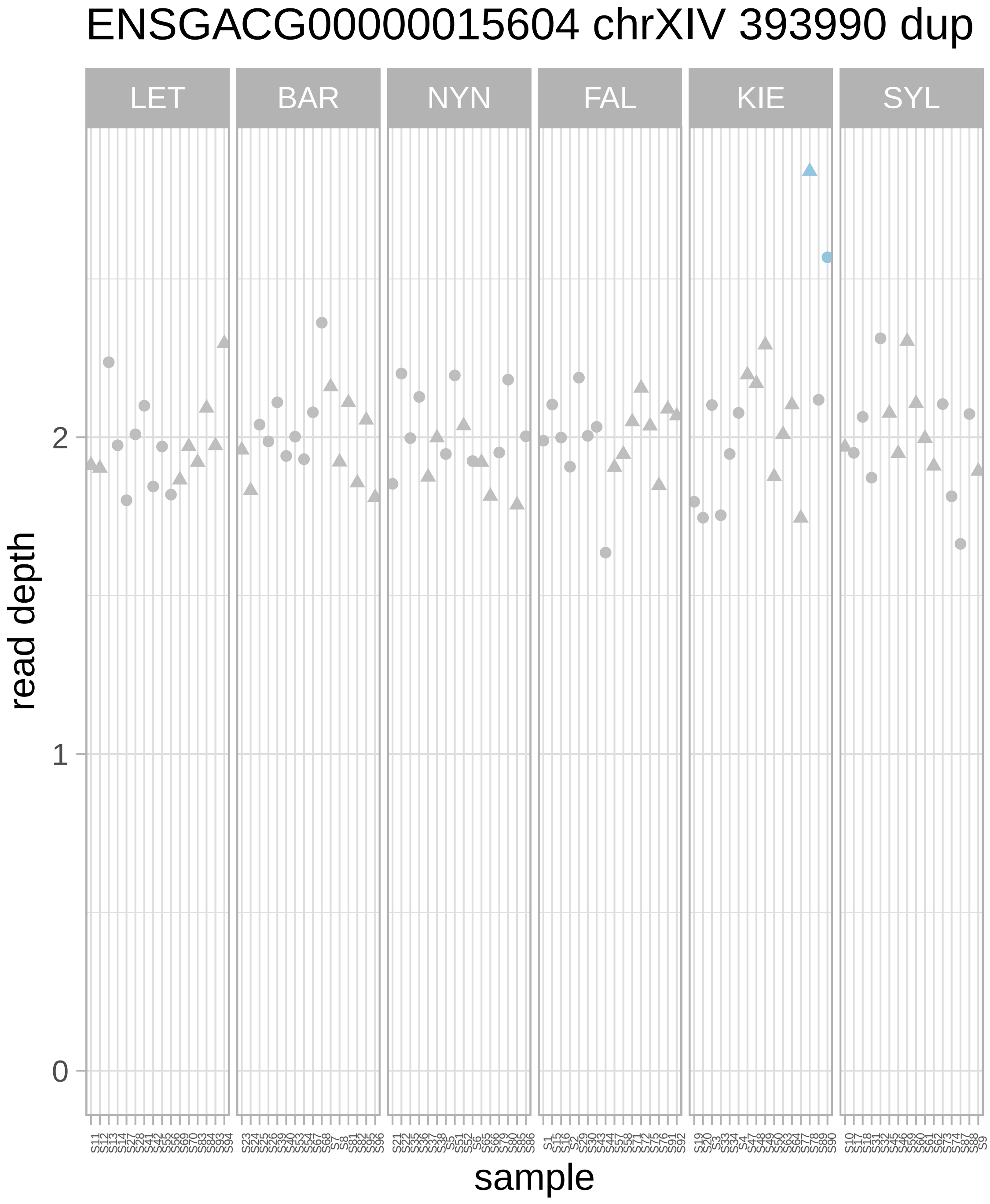
ENSGACG00000015604 chrXIV 393990 dup
LET
BAR
NYN
FAL
KIE
SYL
read depth
0
1
2
sample

ENSGACG00000015938 chrXIV 2606056 dup

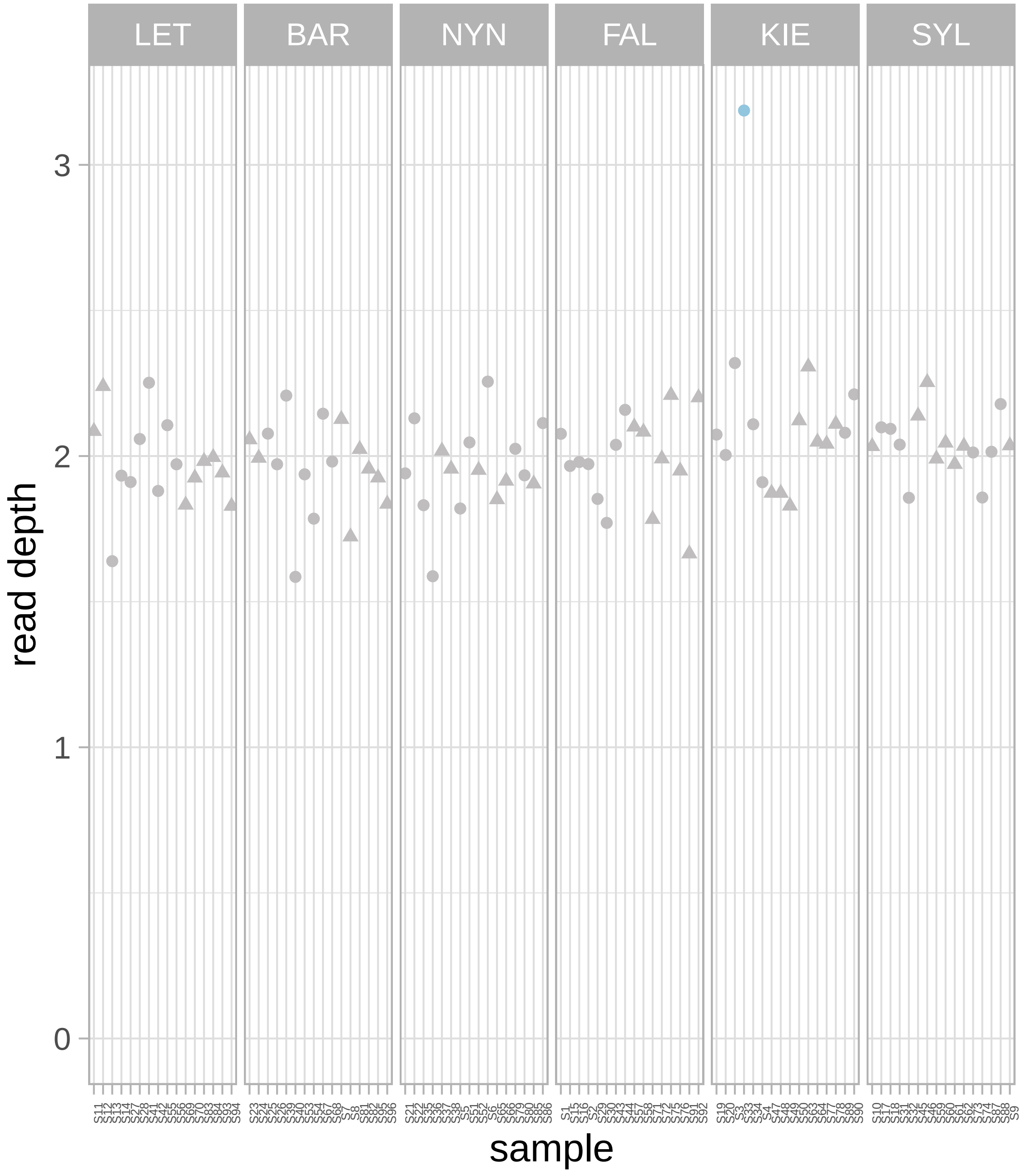

ENSGACG00000016312 chrXIV 3566770 dup

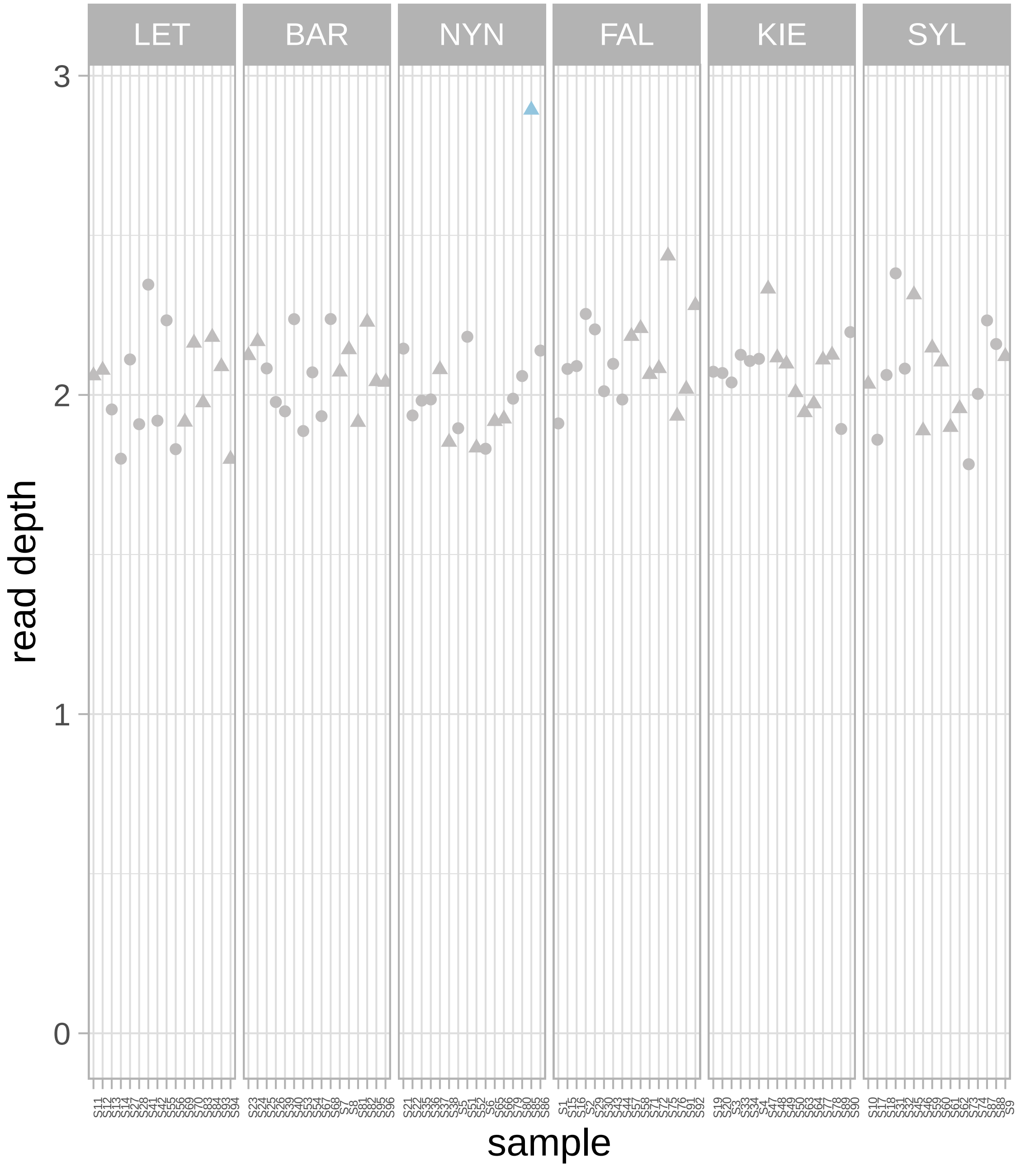

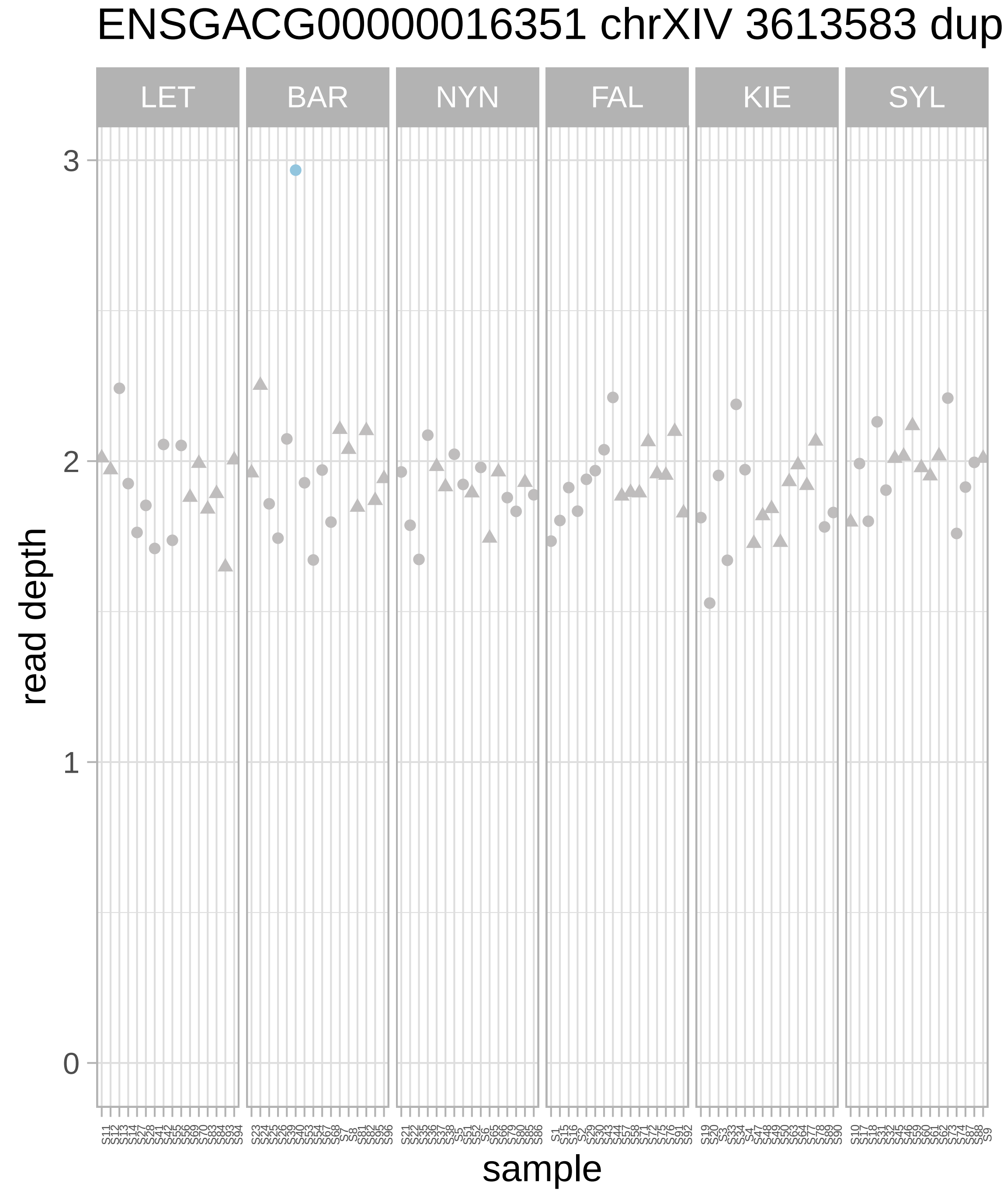
ENSGACG00000016351 chrXIV 3613583 dup
LET
BAR
NYN
FAL
KIE
SYL
read depth
0
1
2
3
S11 S12 S13 S14 S27 S28 S41 S42 S55 S56 S69 S70 S83 S84 S93 S94
S23 S24 S25 S26 S39 S40 S53 S54 S67 S68 S7 S8 S81 S82 S95 S96
S21 S22 S35 S36 S37 S38 S5 S51 S52 S6 S65 S66 S79 S80 S85 S86
S1 S15 S16 S2 S29 S30 S43 S44 S57 S58 S71 S72 S75 S76 S91 S92
S19 S20 S3 S33 S34 S4 S47 S48 S49 S50 S63 S64 S77 S78 S89 S90
S10 S17 S18 S31 S32 S45 S46 S59 S60 S61 S62 S73 S74 S87 S88 S9
sample

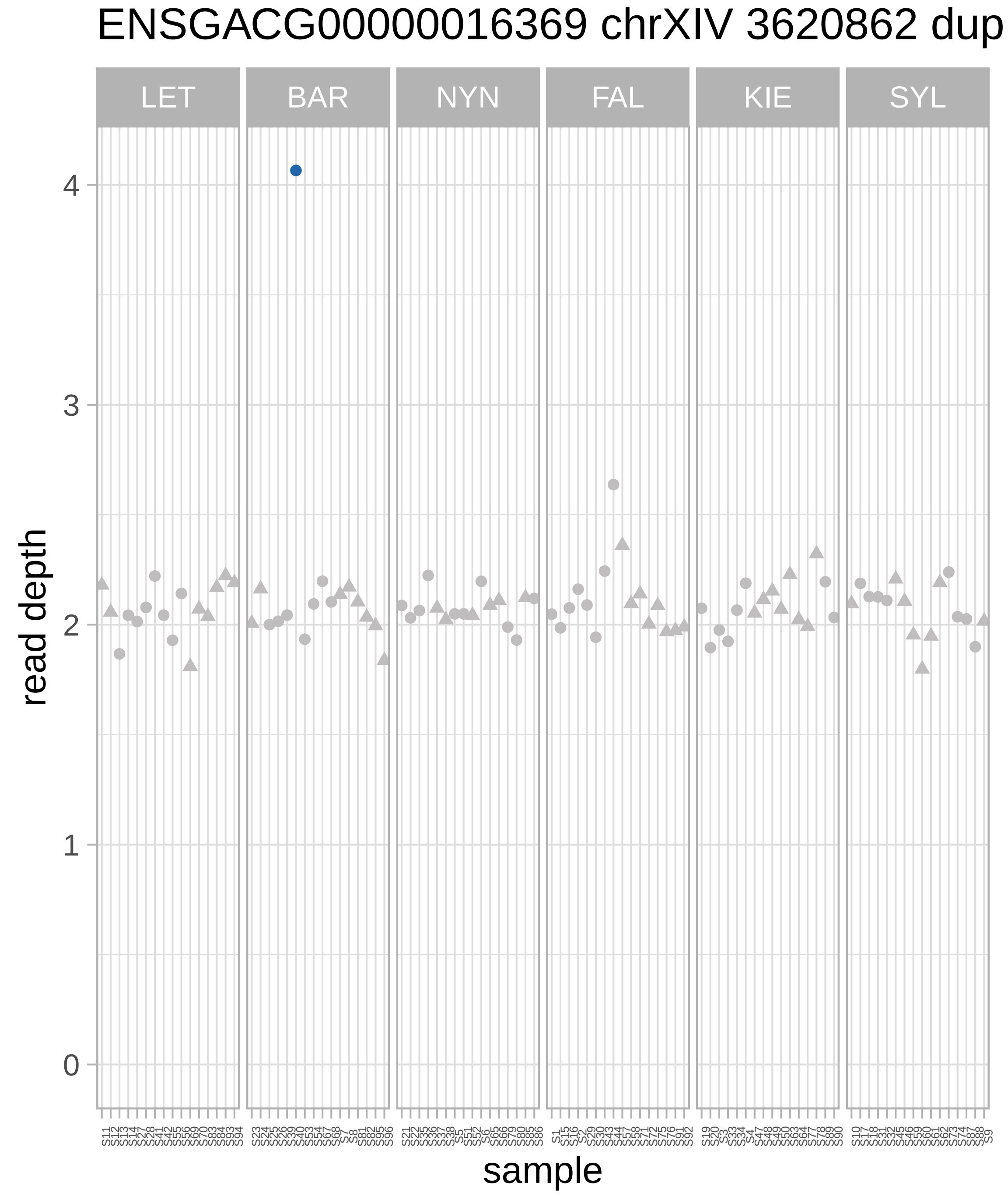
ENSGACG00000016369 chrXIV 3620862 dup
LET
BAR
NYN
FAL
KIE
SYL
read depth
4
3
2
1
0
S11 S12 S13 S14 S27 S28 S41 S42 S55 S56 S69 S70 S83 S84 S93 S94
S23 S24 S25 S26 S39 S40 S53 S54 S67 S68 S7 S8 S81 S82 S95 S96
S21 S22 S35 S36 S37 S38 S5 S51 S52 S6 S65 S66 S79 S80 S85 S86
S1 S15 S16 S2 S29 S30 S43 S44 S57 S58 S71 S72 S75 S76 S91 S92
S19 S20 S3 S33 S34 S4 S47 S48 S49 S50 S63 S64 S77 S78 S89 S90
S10 S17 S18 S31 S32 S45 S46 S59 S60 S61 S62 S73 S74 S87 S88 S9
sample

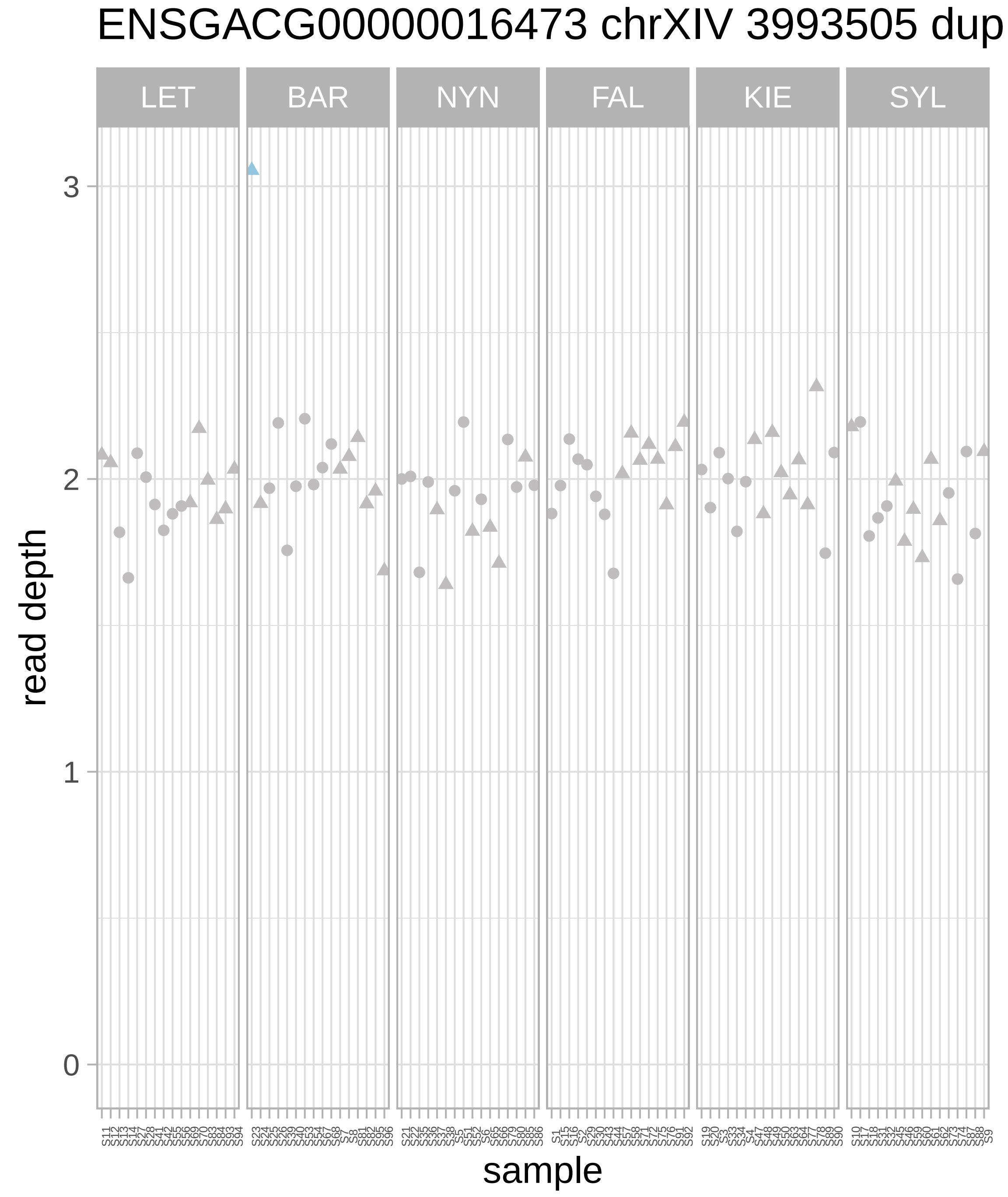
ENSGACG00000016473 chrXIV 3993505 dup
LET
BAR
NYN
FAL
KIE
SYL
read depth
0
1
2
3
sample
S11 S12 S13 S14 S27 S28 S41 S42 S55 S56 S69 S70 S83 S84 S93 S94
S23 S24 S25 S26 S39 S40 S53 S54 S67 S68 S7 S8 S81 S82 S95 S96
S21 S22 S35 S36 S37 S38 S5 S51 S52 S6 S65 S66 S79 S80 S85 S86
S1 S15 S16 S2 S29 S30 S43 S44 S57 S58 S71 S72 S75 S76 S91 S92
S19 S20 S3 S33 S34 S4 S47 S48 S49 S50 S63 S64 S77 S78 S89 S90
S10 S17 S18 S31 S32 S45 S46 S59 S60 S61 S62 S73 S74 S87 S88 S9

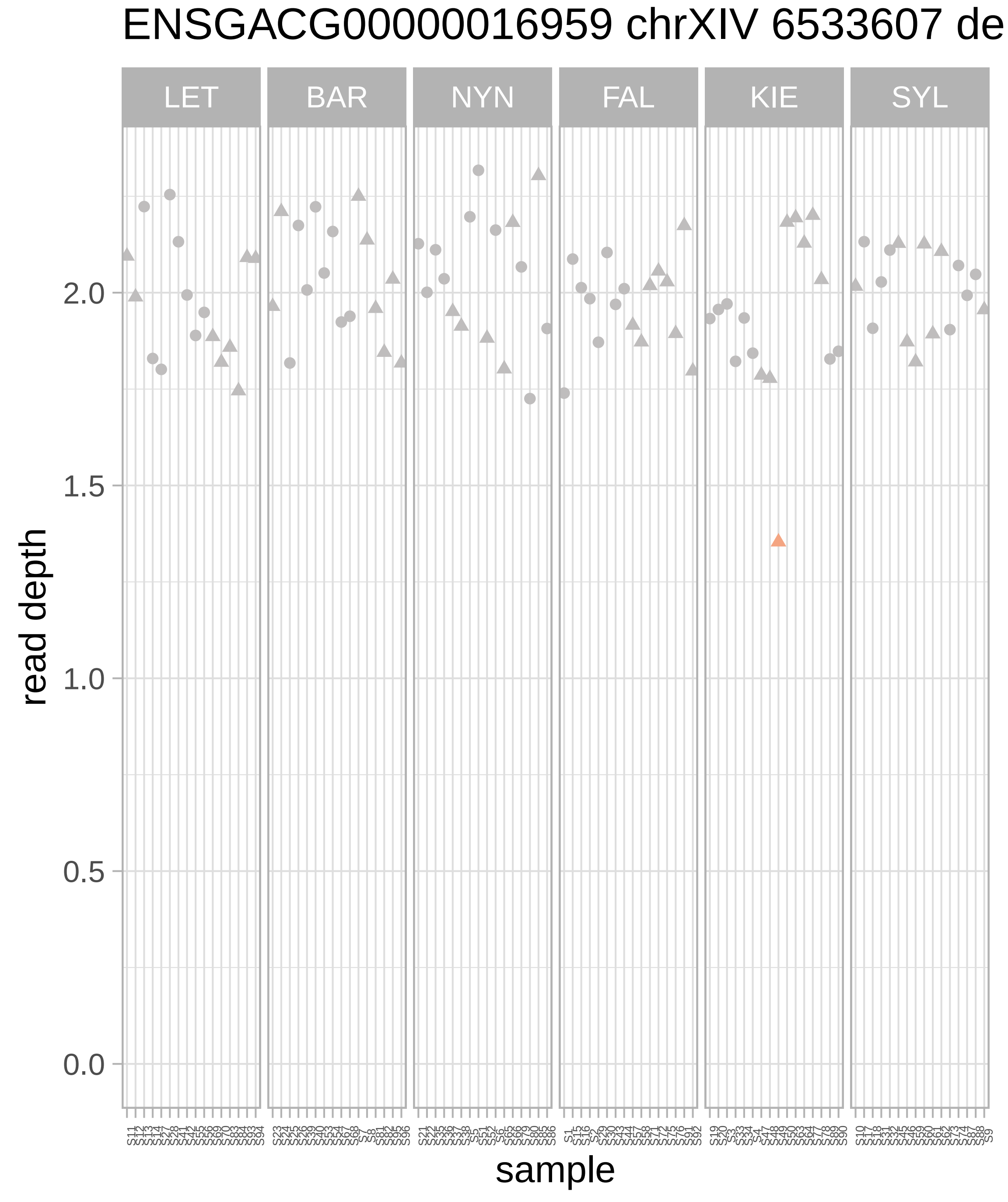
ENSGACG00000016959 chrXIV 6533607 de
LET
BAR
NYN
FAL
KIE
SYL
read depth
2.0
1.5
1.0
0.5
0.0
sample

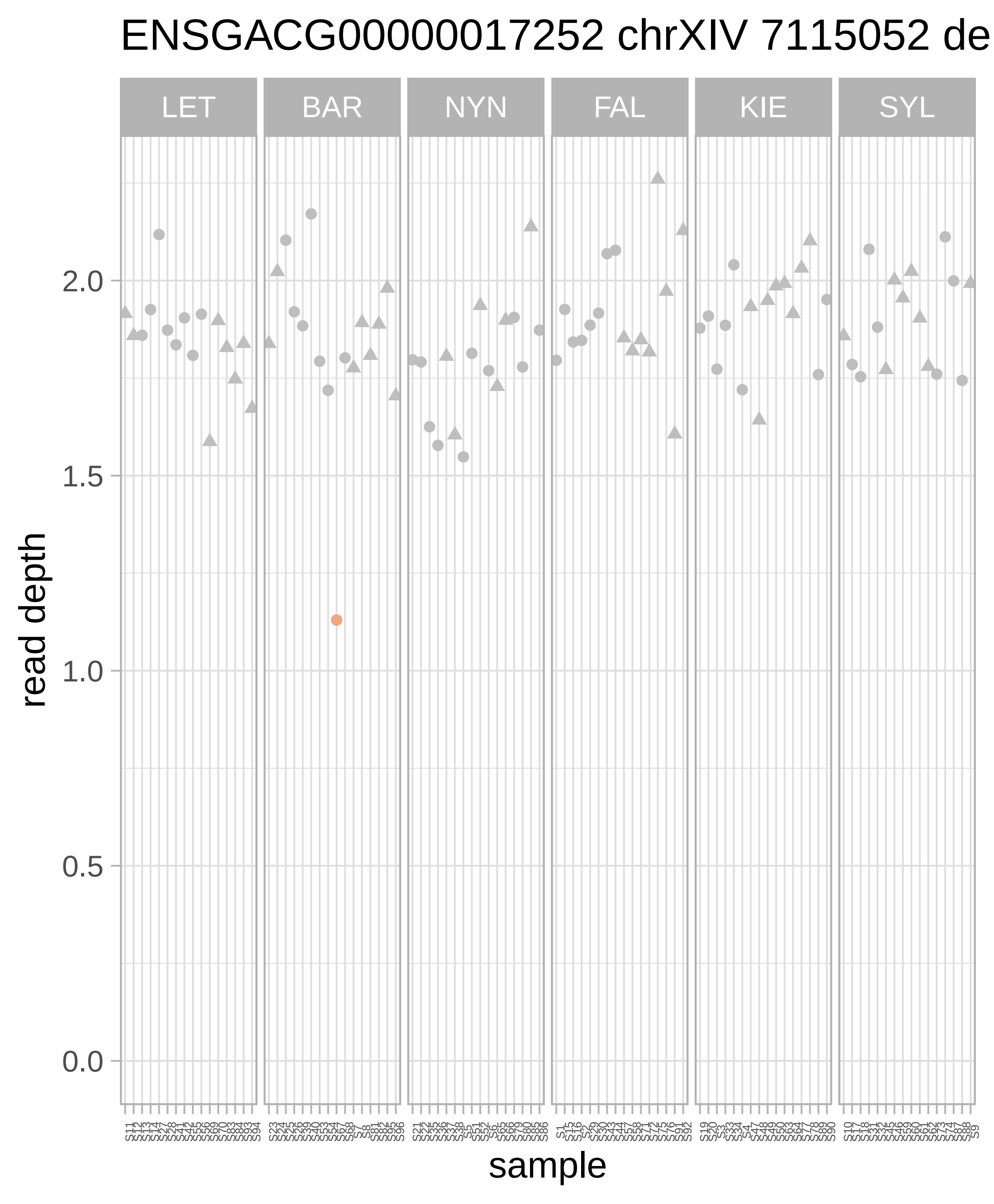
ENSGACG00000017252 chrXIV 7115052 de
LET
BAR
NYN
FAL
KIE
SYL
read depth
2.0
1.5
1.0
0.5
0.0
sample

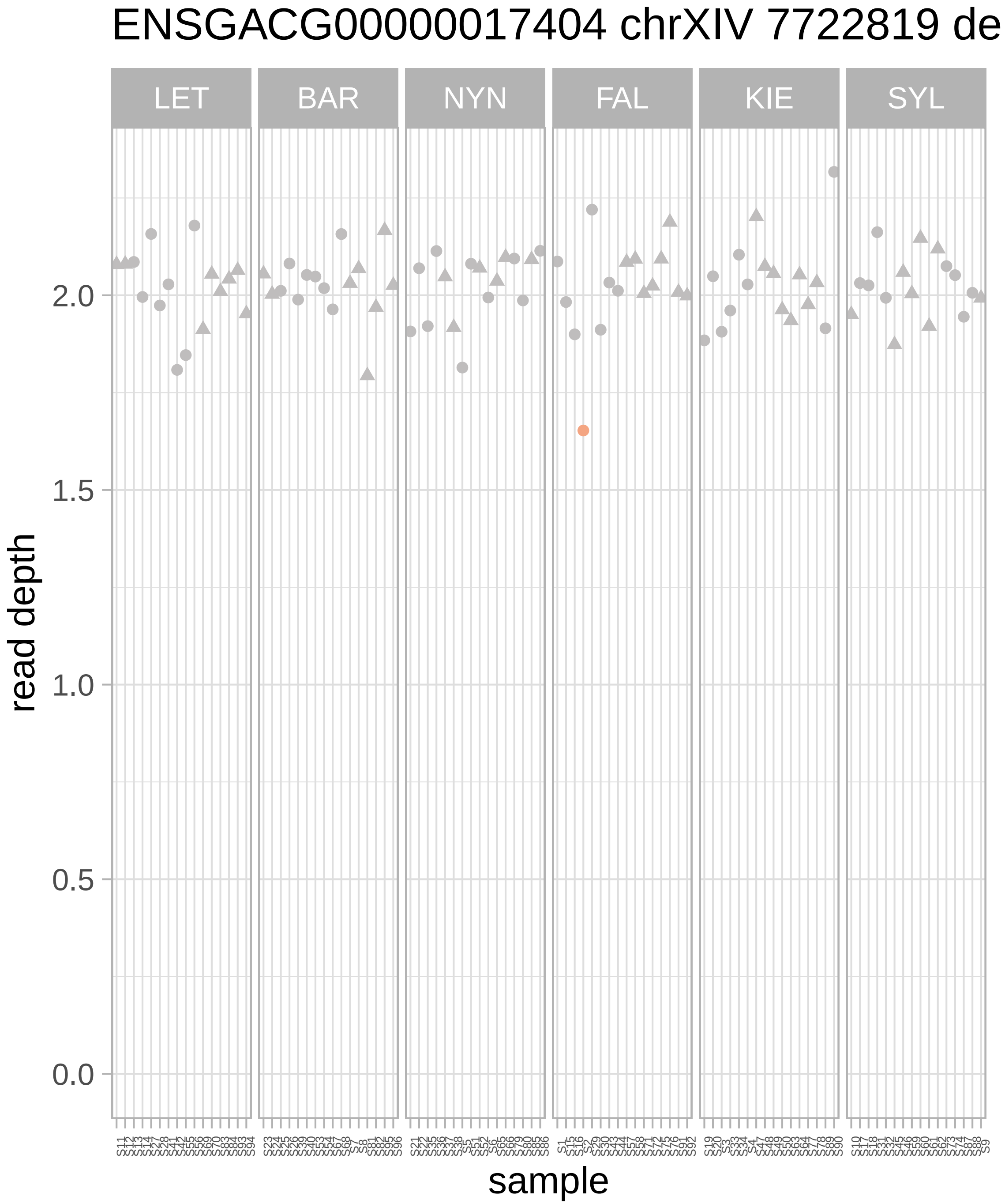
ENSGACG00000017404 chrXIV 7722819 de
LET
BAR
NYN
FAL
KIE
SYL
read depth
0.0
0.5
1.0
1.5
2.0
sample

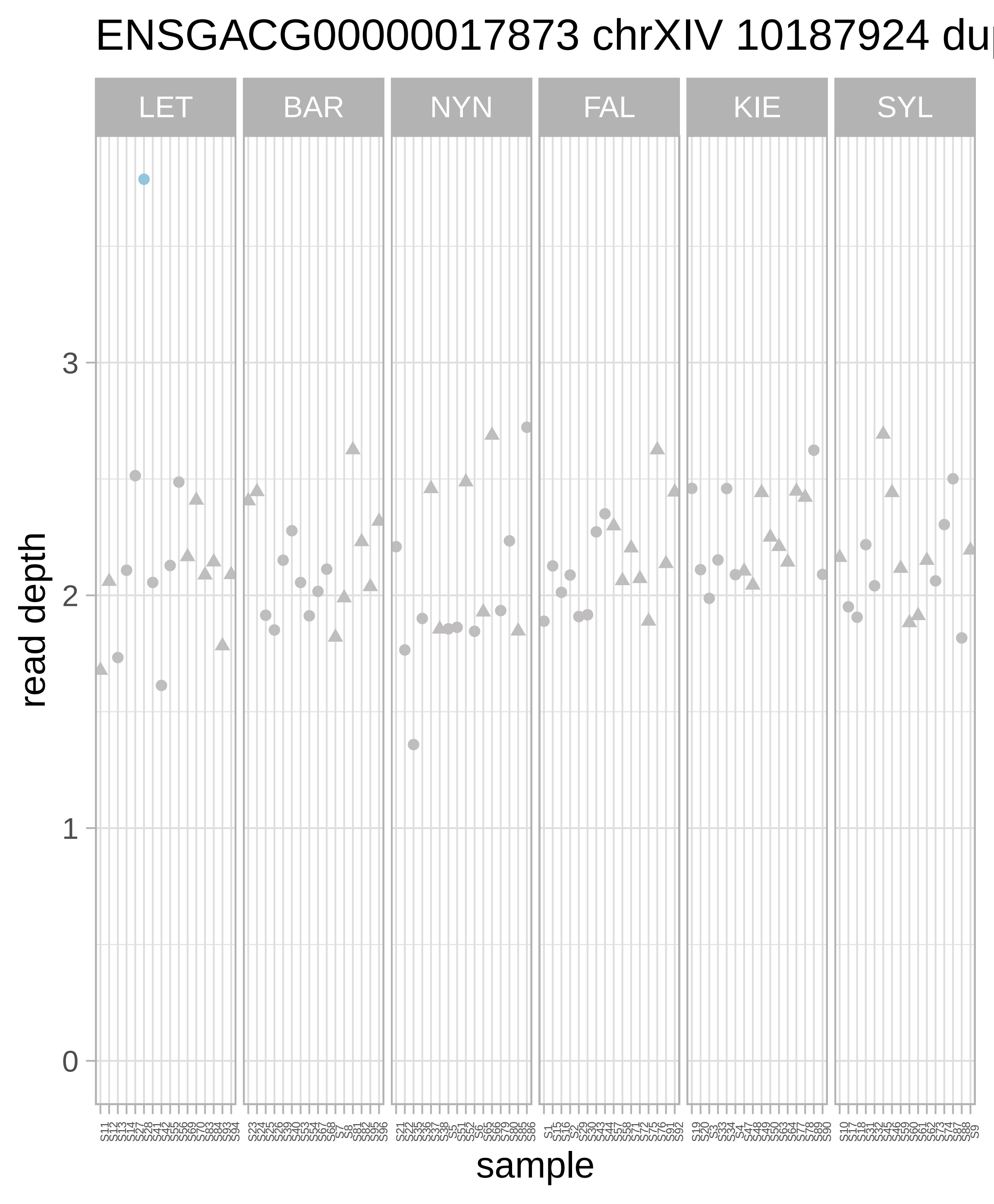
ENSGACG00000017873 chrXIV 10187924 du
LET
BAR
NYN
FAL
KIE
SYL
read depth
3
2
1
0
S11 S12 S13 S14 S27 S28 S41 S42 S55 S56 S69 S70 S83 S84 S93 S94
S23 S24 S25 S26 S39 S40 S53 S54 S67 S68 S7 S8 S81 S82 S95 S96
S21 S22 S35 S36 S37 S38 S5 S51 S52 S6 S65 S66 S79 S80 S85 S86
S1 S15 S16 S2 S29 S30 S43 S44 S57 S58 S71 S72 S75 S76 S91 S92
S19 S20 S3 S33 S34 S4 S47 S48 S49 S50 S63 S64 S77 S78 S89 S90
S10 S17 S18 S31 S32 S45 S46 S59 S60 S61 S62 S73 S74 S87 S88 S9
sample

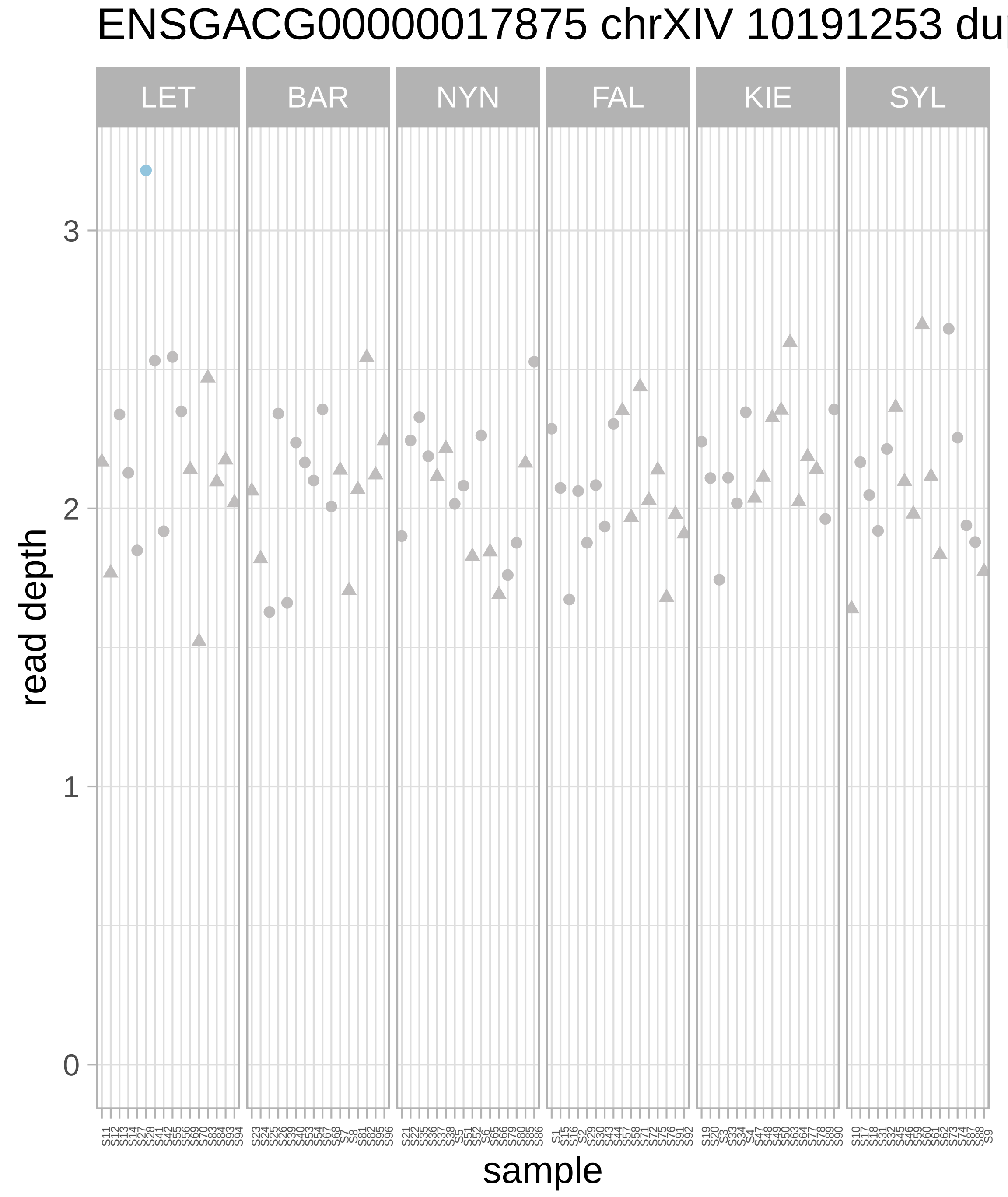
ENSGACG00000017875 chrXIV 10191253 du
LET
BAR
NYN
FAL
KIE
SYL
read depth
0
1
2
3
sample
S11 S12 S13 S14 S27 S28 S41 S42 S55 S56 S69 S70 S83 S84 S93 S94
S23 S24 S25 S26 S39 S40 S53 S54 S67 S68 S7 S8 S81 S82 S95 S96
S21 S22 S35 S36 S37 S38 S5 S51 S52 S6 S65 S66 S79 S80 S85 S86
S1 S15 S16 S2 S29 S30 S43 S44 S57 S58 S71 S72 S75 S76 S91 S92
S19 S20 S3 S33 S34 S4 S47 S48 S49 S50 S63 S64 S77 S78 S89 S90
S10 S17 S18 S31 S32 S45 S46 S59 S60 S61 S62 S73 S74 S87 S88 S9

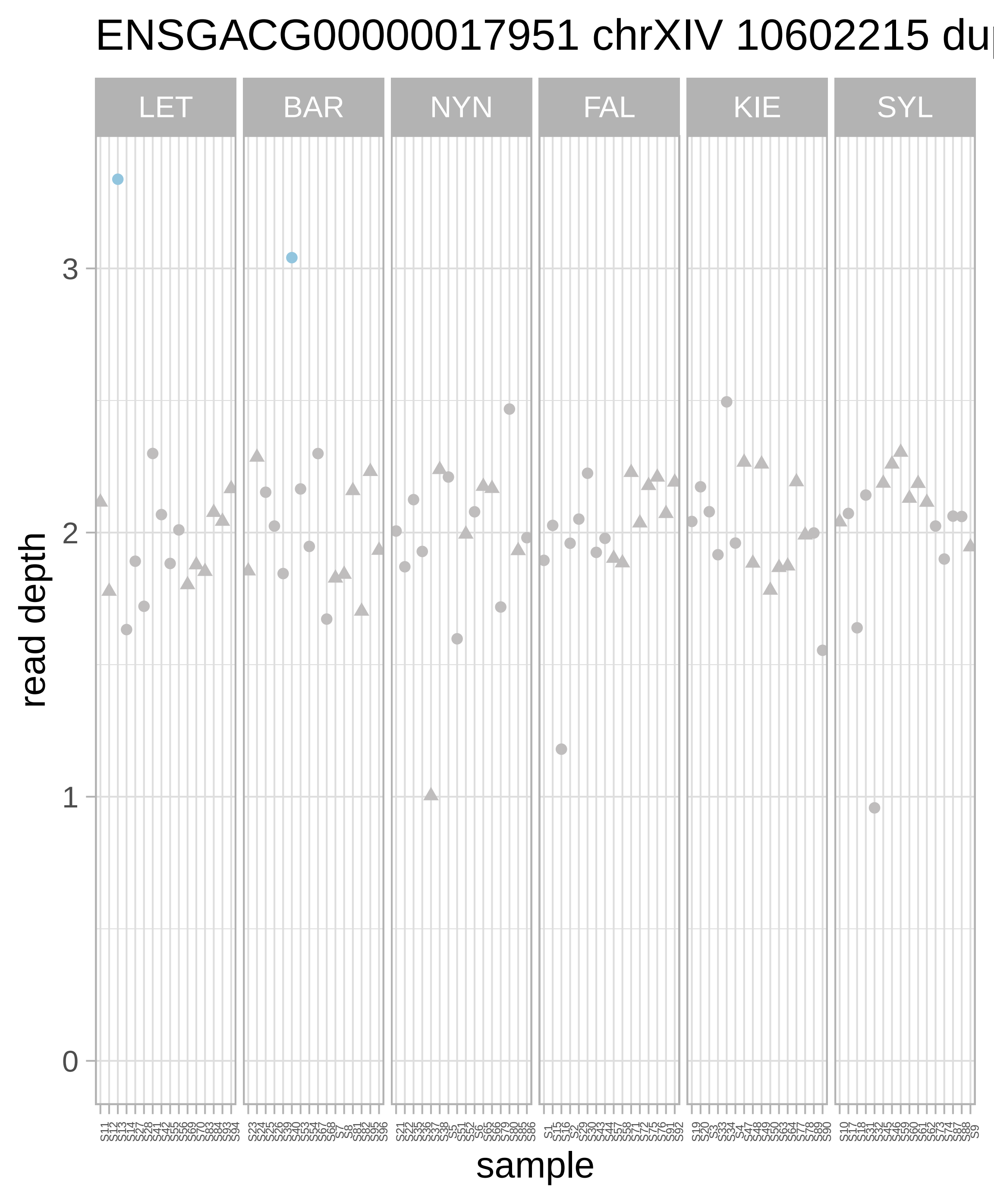

ENSGACG00000017951 chrXIV 10602215 du
LET
BAR
NYN
FAL
KIE
SYL
read depth
0
1
2
3
sample

ENSGACG00000018106 chrXIV 11265127 du

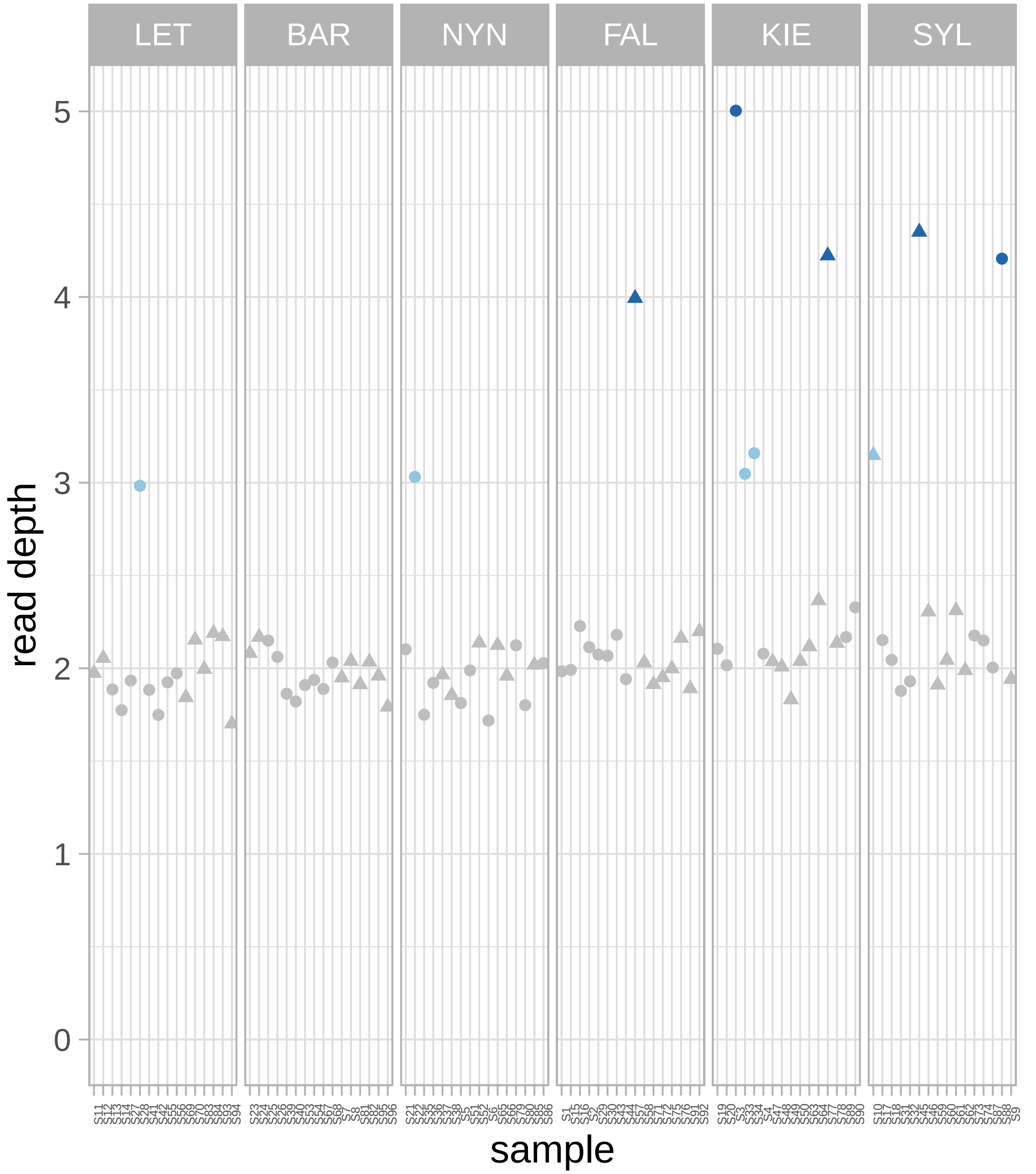

ENSGACG00000018117 chrXIV 11289168 du

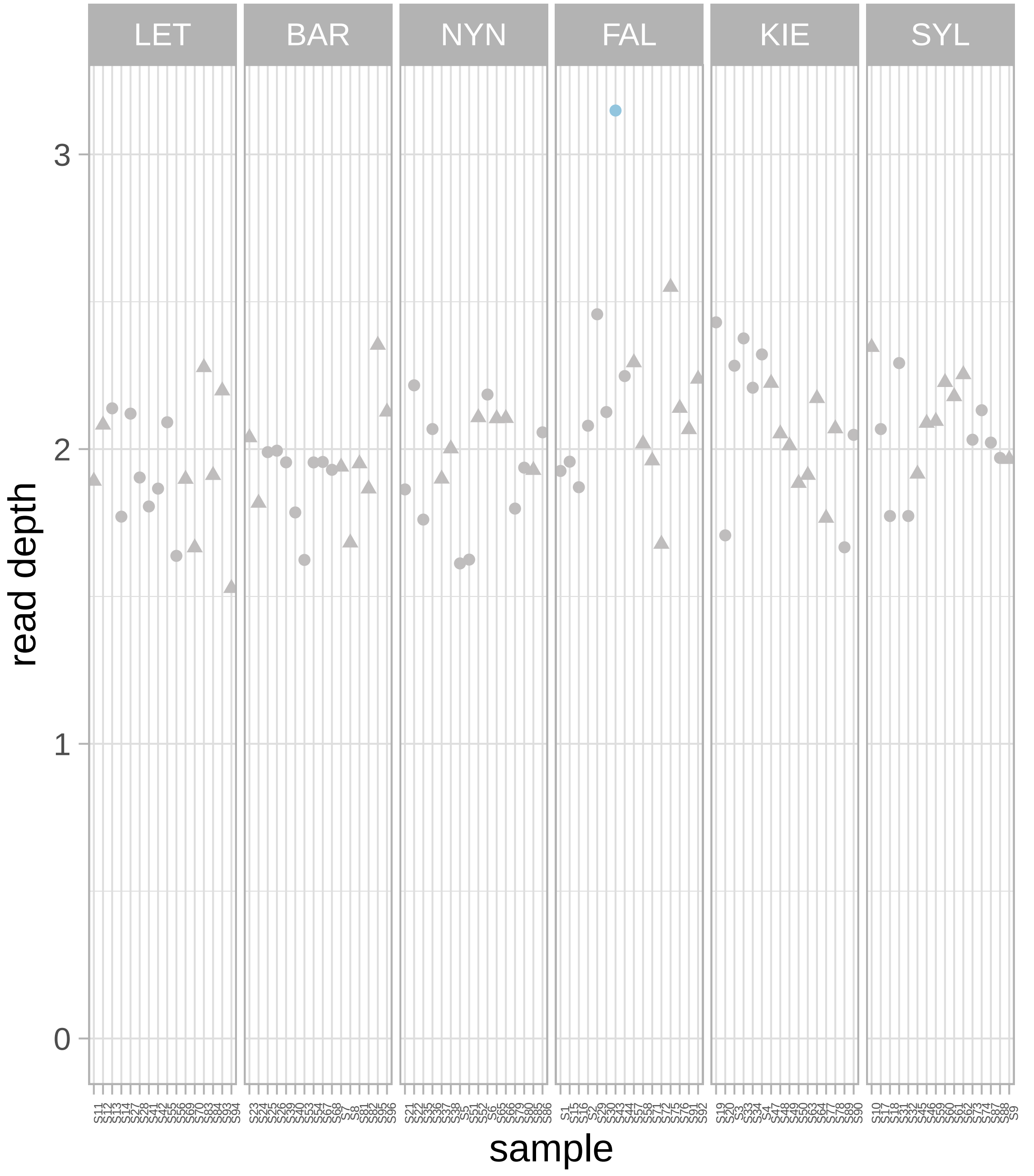

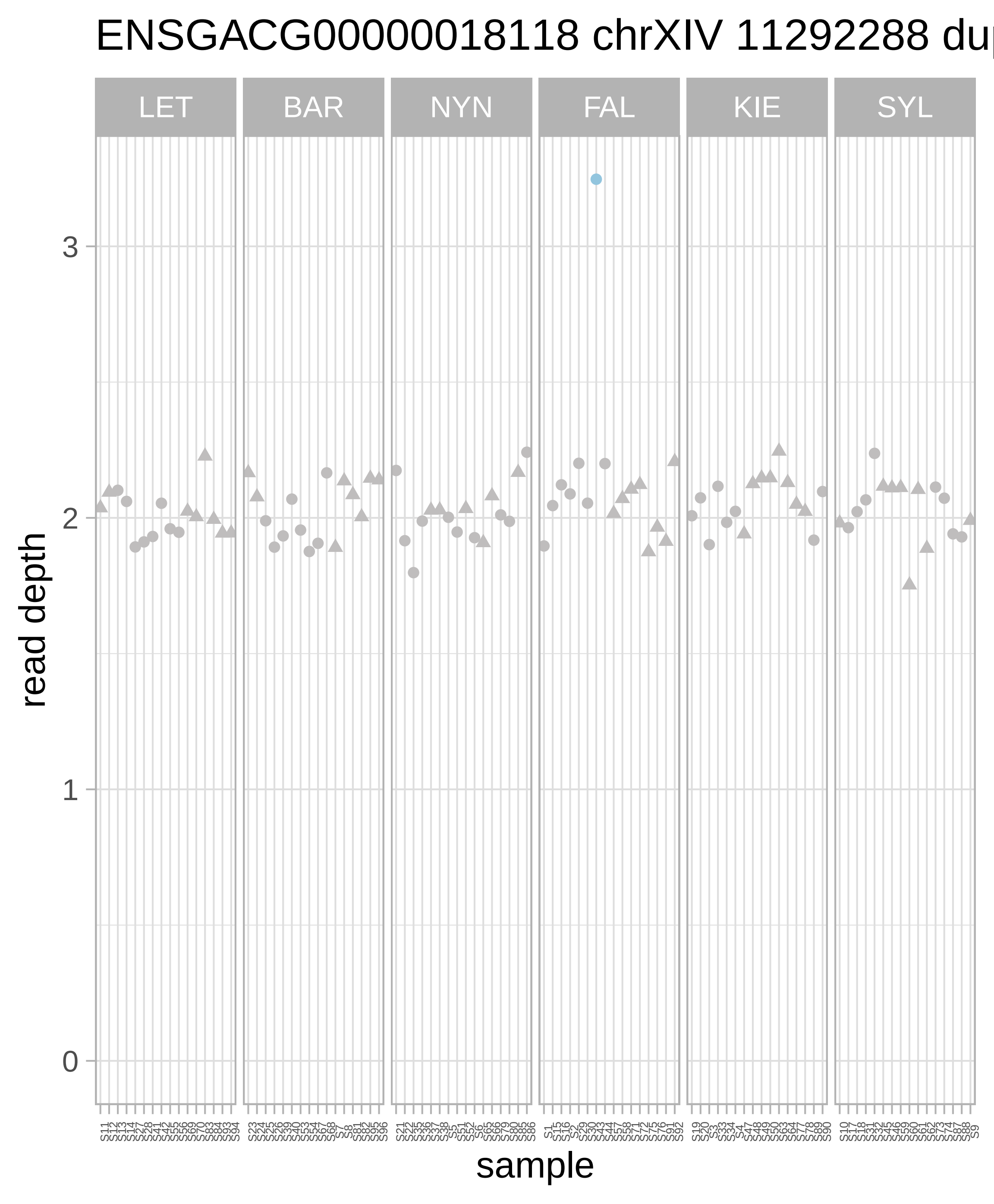
ENSGACG00000018118 chrXIV 11292288 du
LET
BAR
NYN
FAL
KIE
SYL
read depth
3
2
1
0
sample

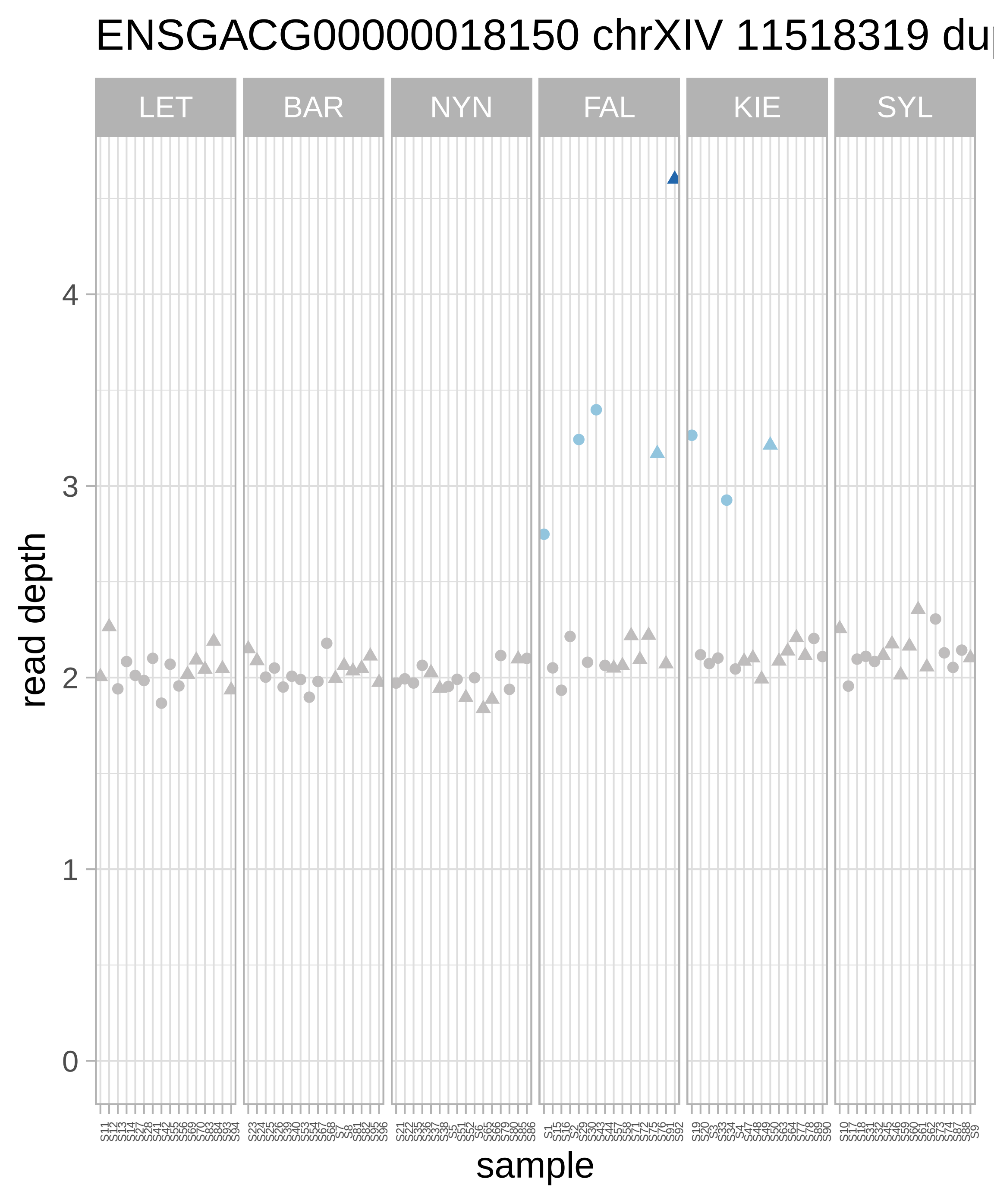

ENSGACG00000018150 chrXIV 11518319 du
LET
BAR
NYN
FAL
KIE
SYL
read depth
0
1
2
3
4
sample

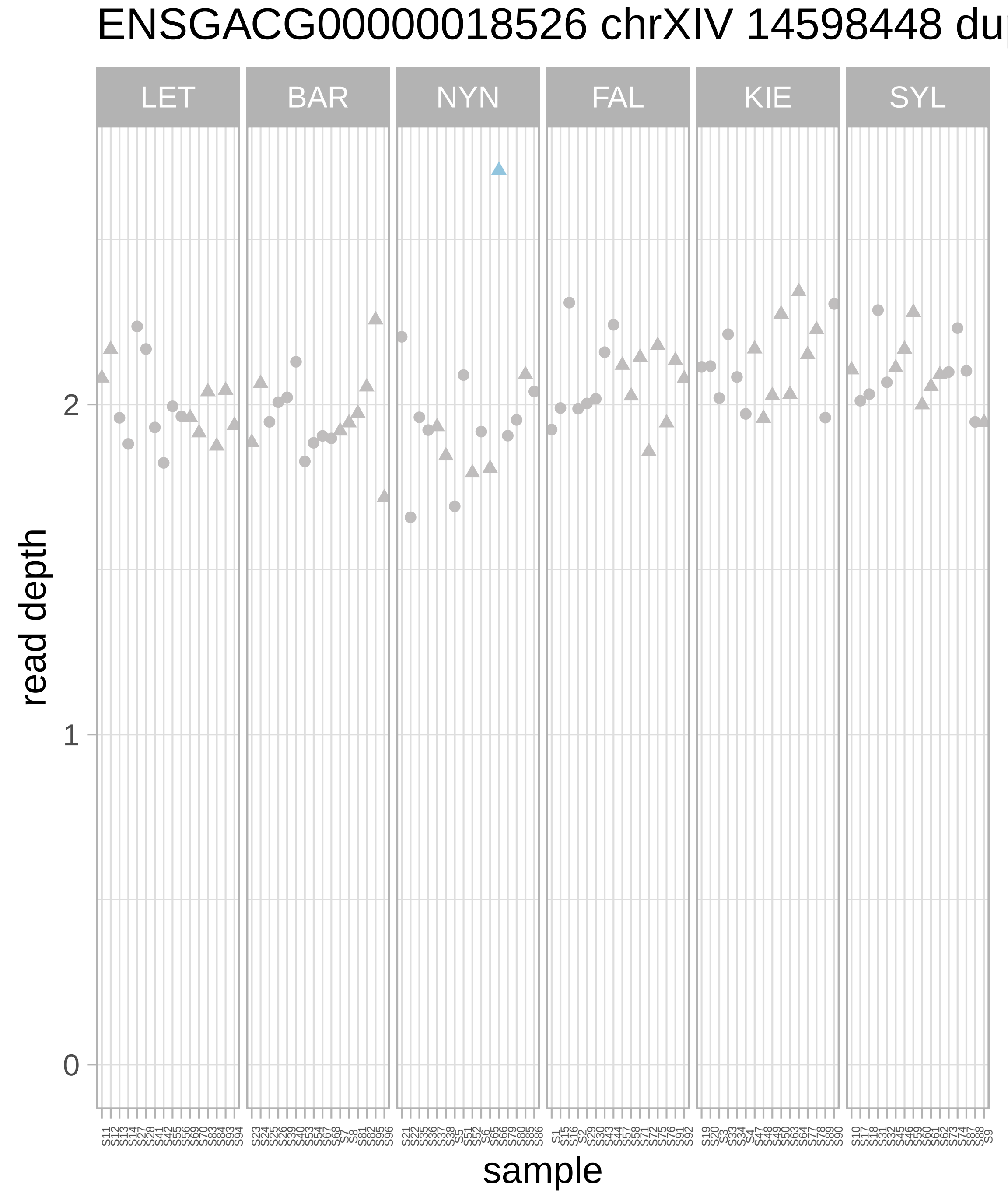
ENSGACG00000018526 chrXIV 14598448 du
LET
BAR
NYN
FAL
KIE
SYL
read depth
0
1
2
sample

ENSGACG00000002356 chrXIX 901299 dup

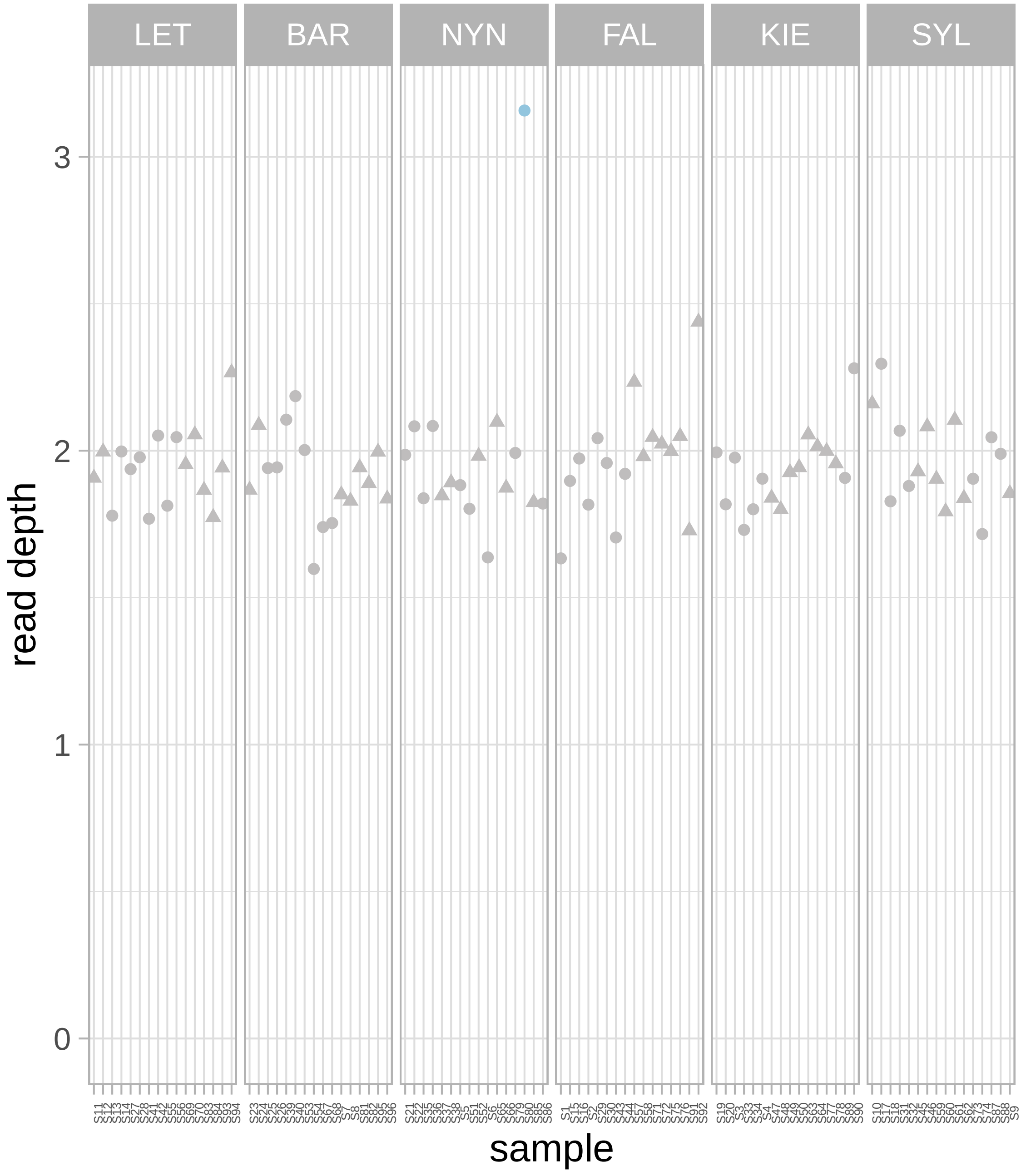

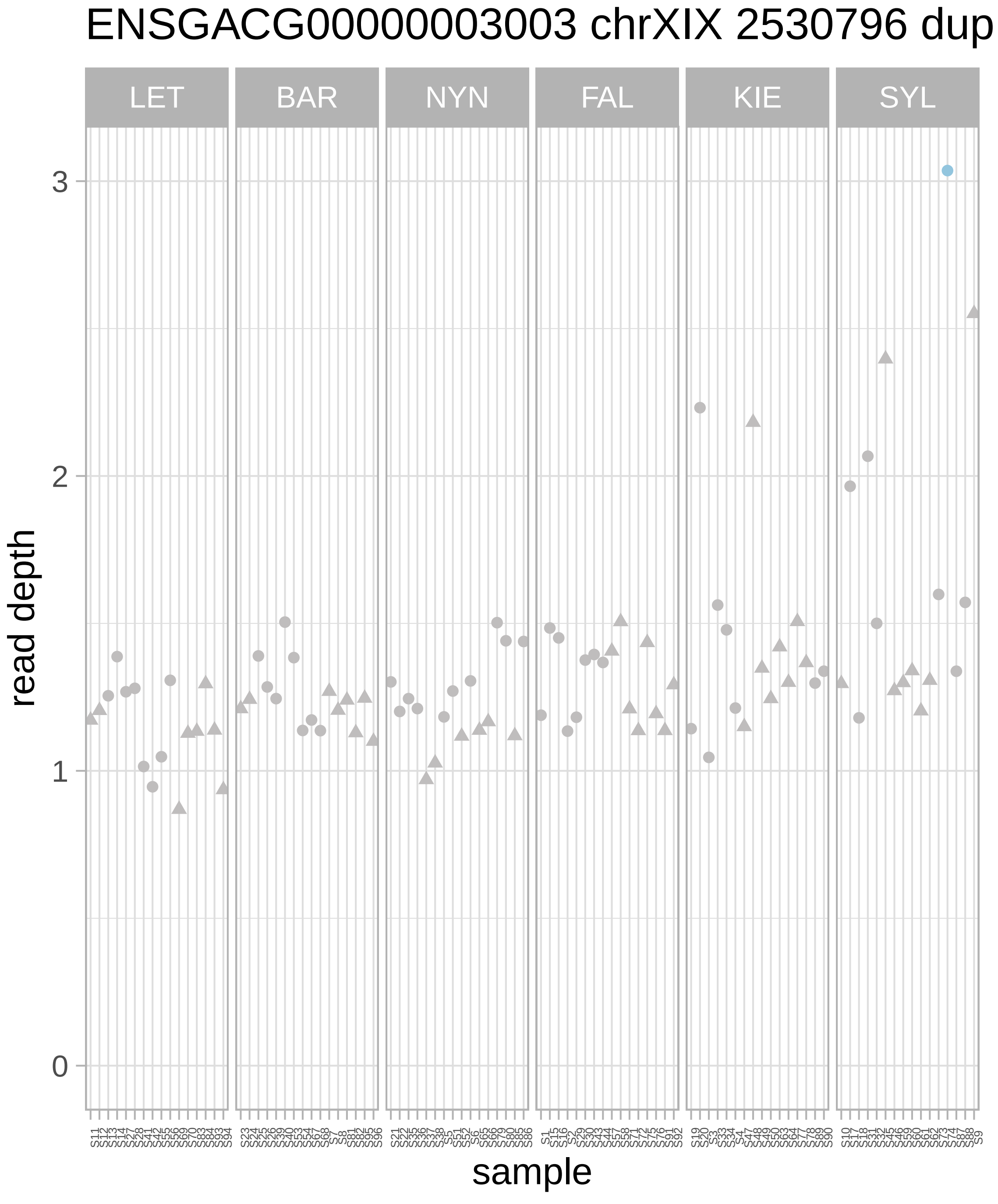

ENSGACG00000003003 chrXIX 2530796 dup
LET
BAR
NYN
FAL
KIE
SYL
read depth
3
2
1
0
S11 S12 S13 S14 S27 S28 S41 S42 S55 S56 S69 S70 S83 S84 S93 S94
S23 S24 S25 S26 S39 S40 S53 S54 S67 S68 S7 S8 S81 S82 S95 S96
S21 S22 S35 S36 S37 S38 S5 S51 S52 S6 S65 S66 S79 S80 S85 S86
S1 S15 S16 S2 S29 S30 S43 S44 S57 S58 S71 S72 S75 S76 S91 S92
S19 S20 S3 S33 S34 S4 S47 S48 S49 S50 S63 S64 S77 S78 S89 S90
S10 S17 S18 S31 S32 S45 S46 S59 S60 S61 S62 S73 S74 S87 S88 S9
sample

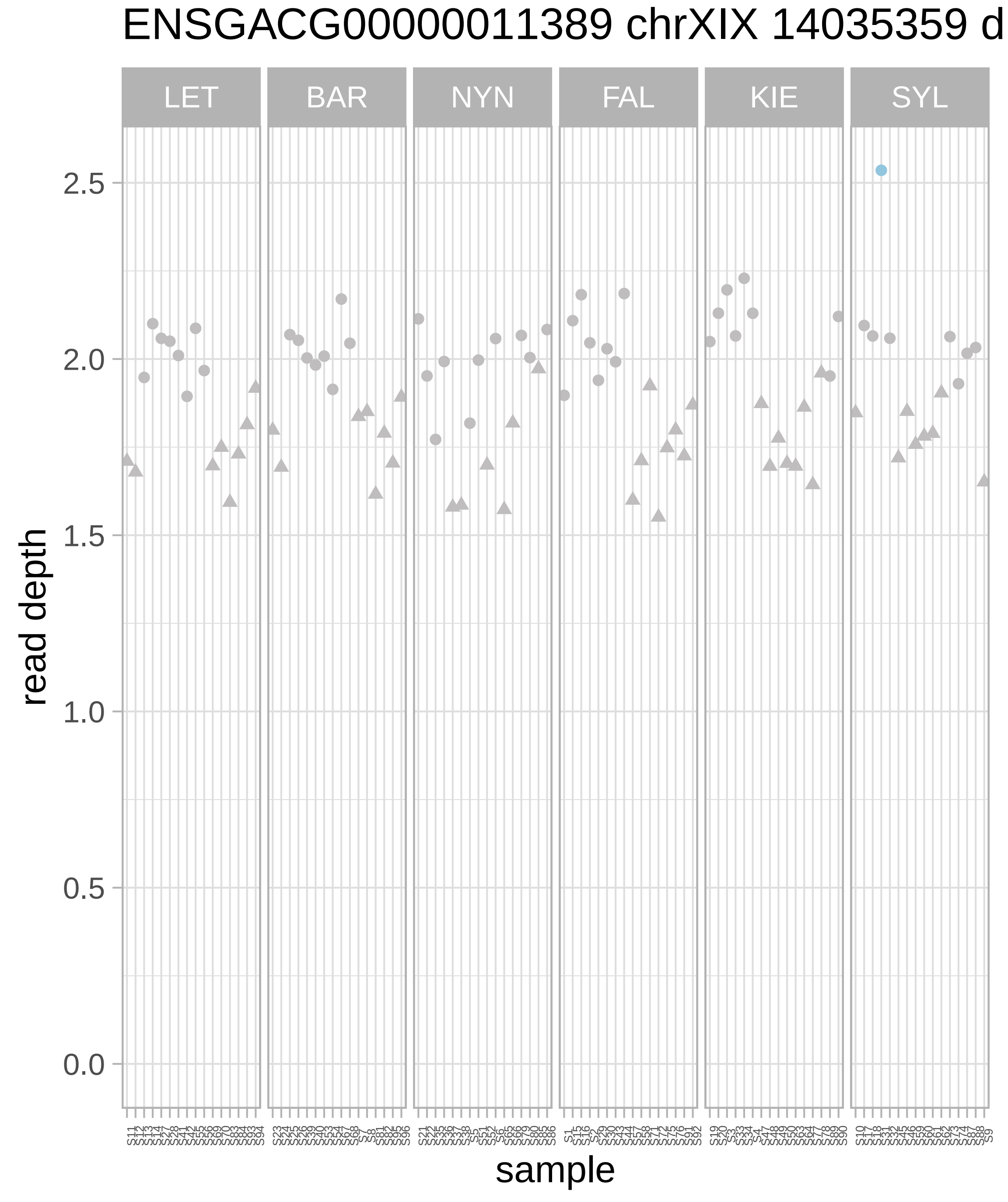
ENSGACG00000011389 chrXIX 14035359 d
LET
BAR
NYN
FAL
KIE
SYL
read depth
2.5
2.0
1.5
1.0
0.5
0.0
S11 S12 S13 S14 S27 S28 S41 S42 S55 S56 S69 S70 S83 S84 S93 S94
S23 S24 S25 S26 S39 S40 S53 S54 S67 S68 S7 S8 S81 S82 S95 S96
S21 S22 S35 S36 S37 S38 S5 S51 S52 S6 S65 S66 S79 S80 S85 S86
S1 S15 S16 S2 S29 S30 S43 S44 S57 S58 S71 S72 S75 S76 S91 S92
S19 S20 S3 S33 S34 S4 S47 S48 S49 S50 S63 S64 S77 S78 S89 S90
S10 S17 S18 S31 S32 S45 S46 S59 S60 S61 S62 S73 S74 S87 S88 S9
sample

ENSGACG00000013886 chrXIX 19640857 dup

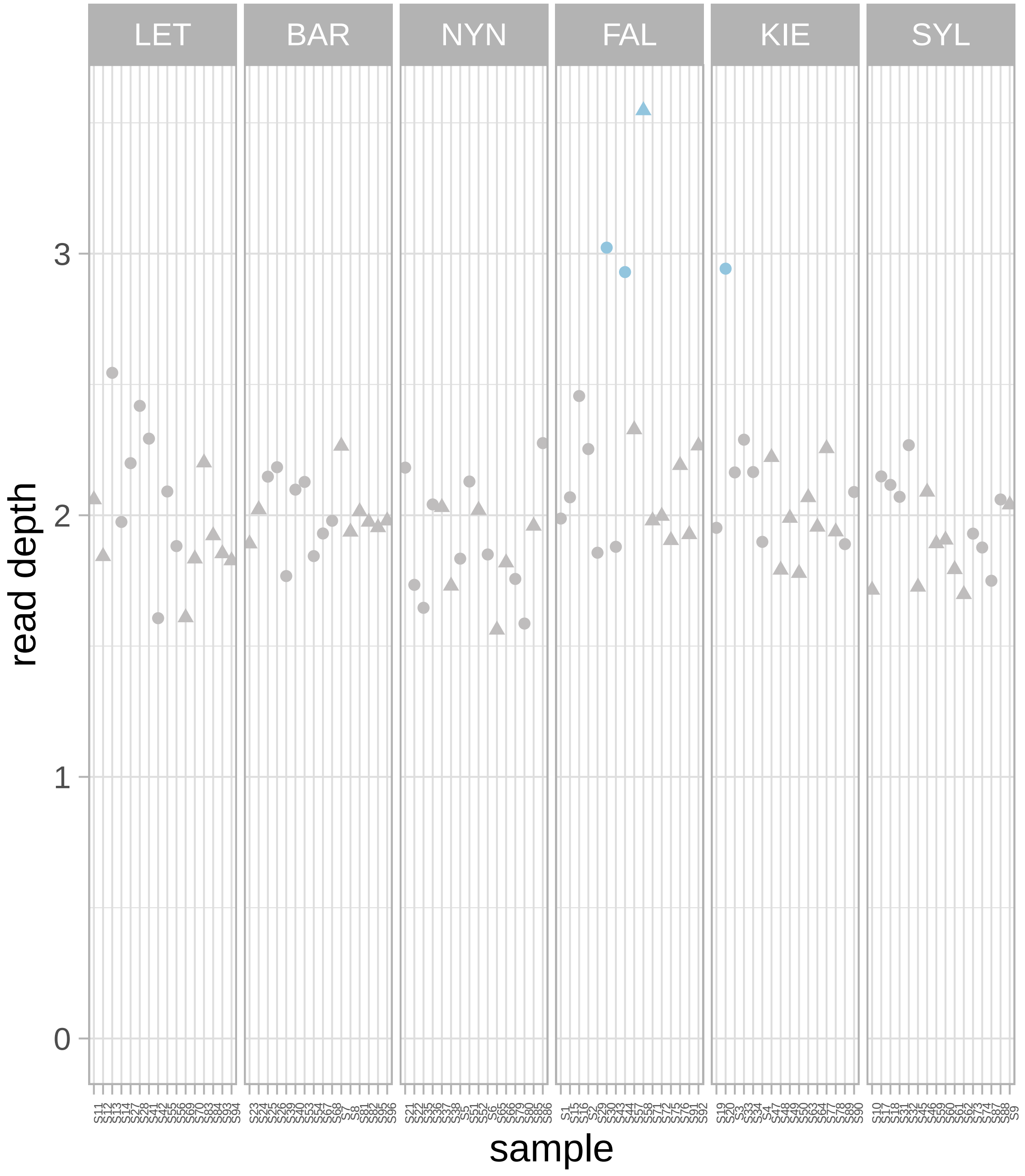

ENSGACG00000004495 chrXV 485619 del

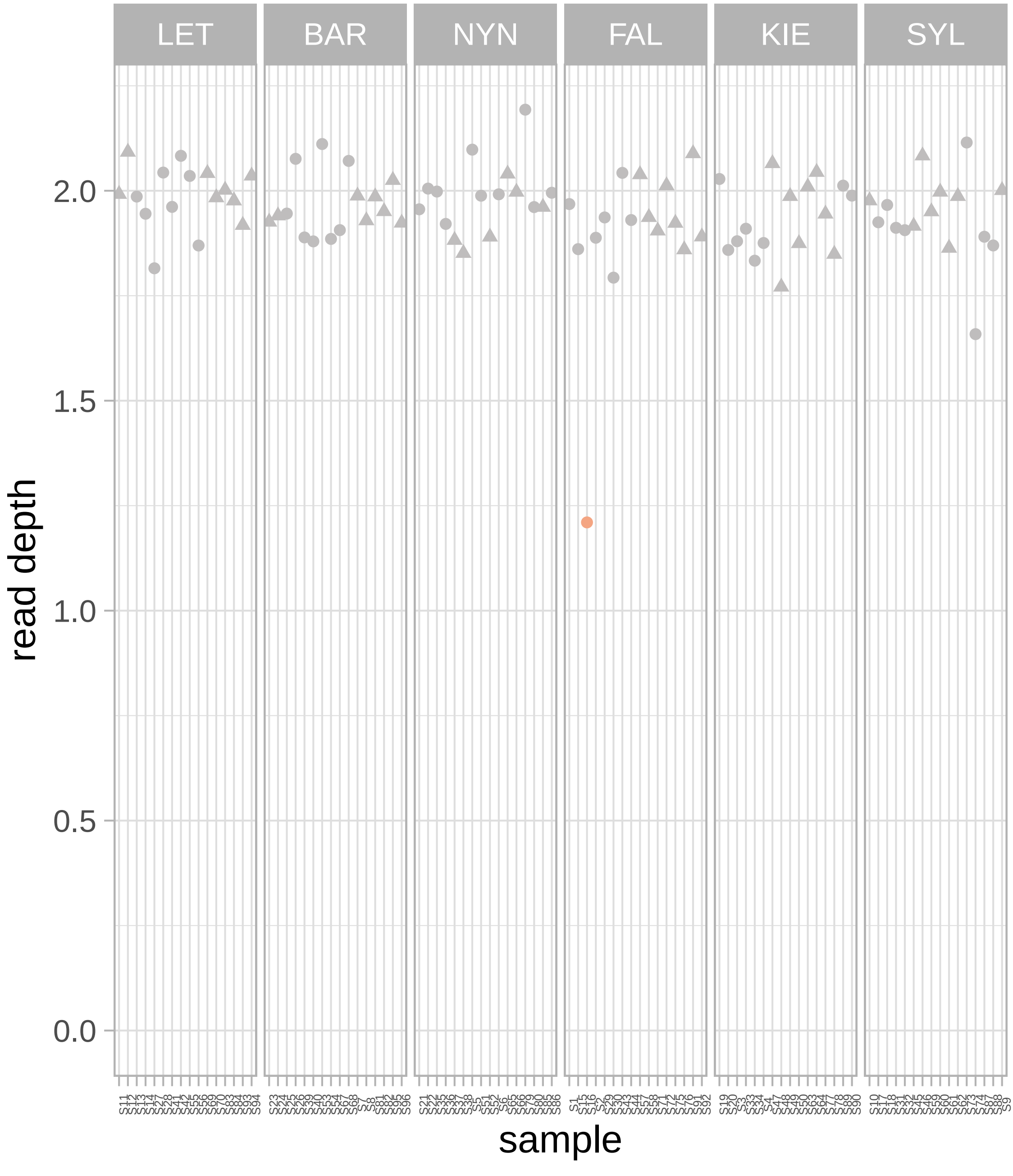

ENSGACG00000004515 chrXV 503553 del

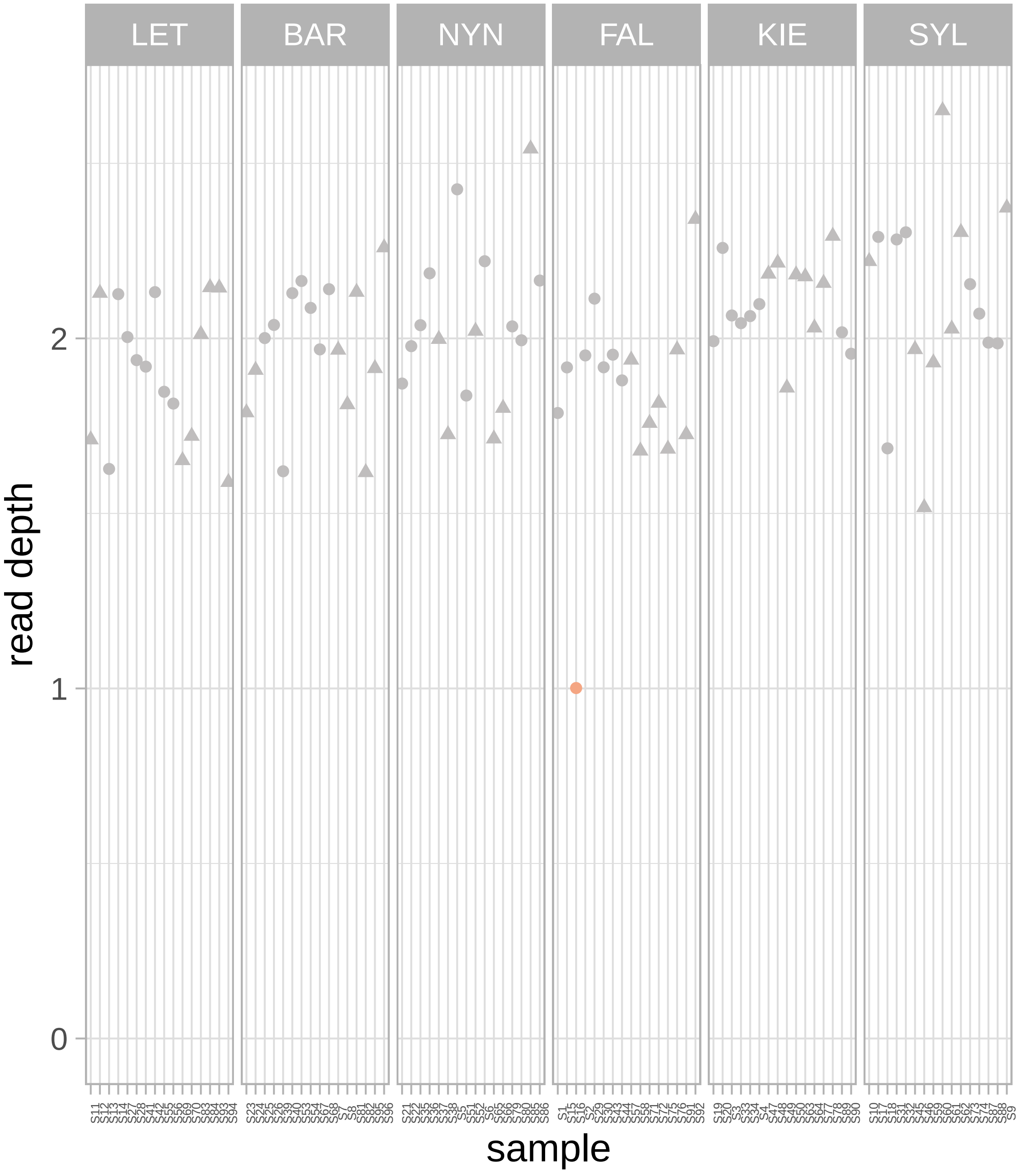

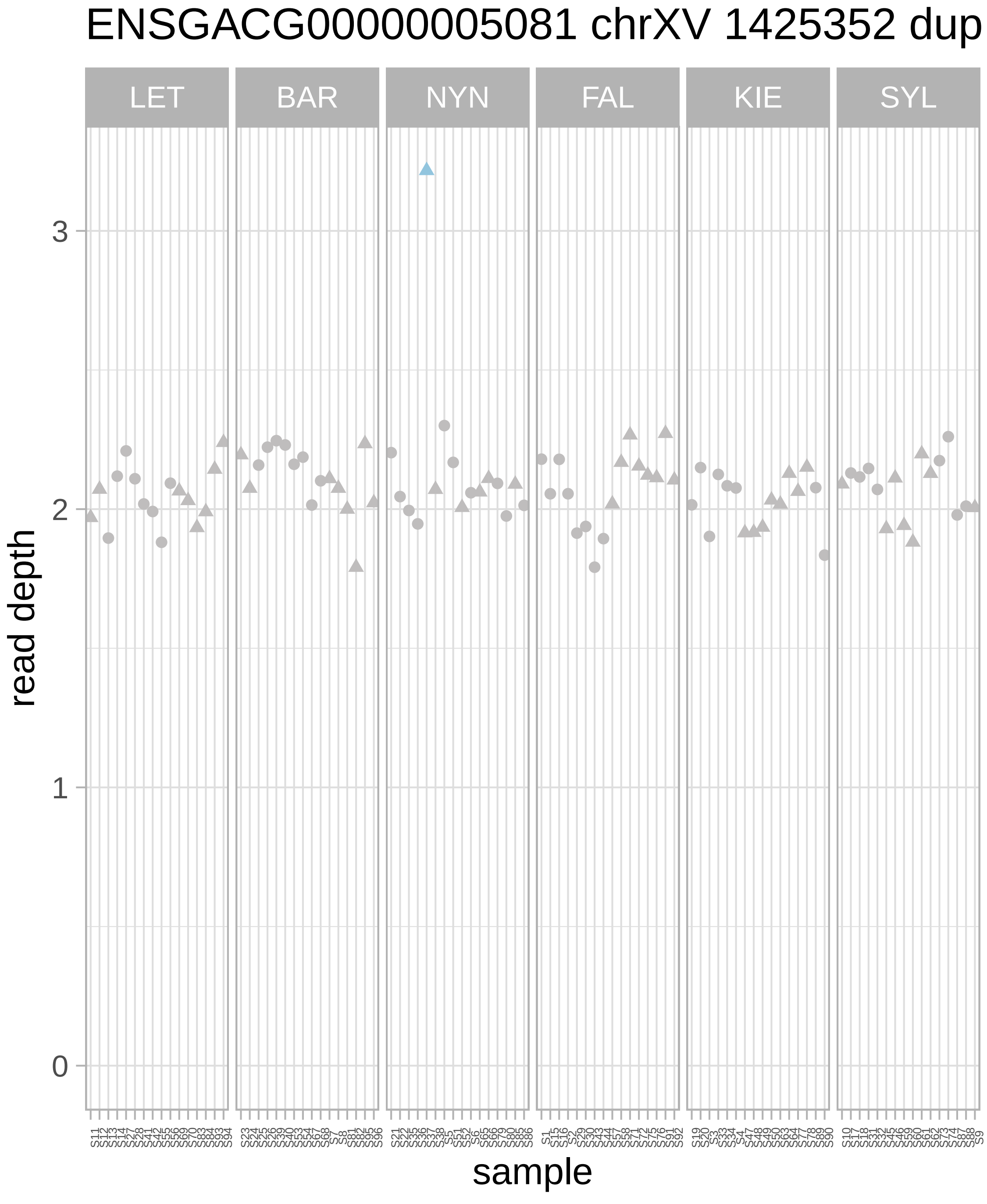
ENSGACG00000005081 chrXV 1425352 dup
LET
BAR
NYN
FAL
KIE
SYL
read depth
0
1
2
3
sample

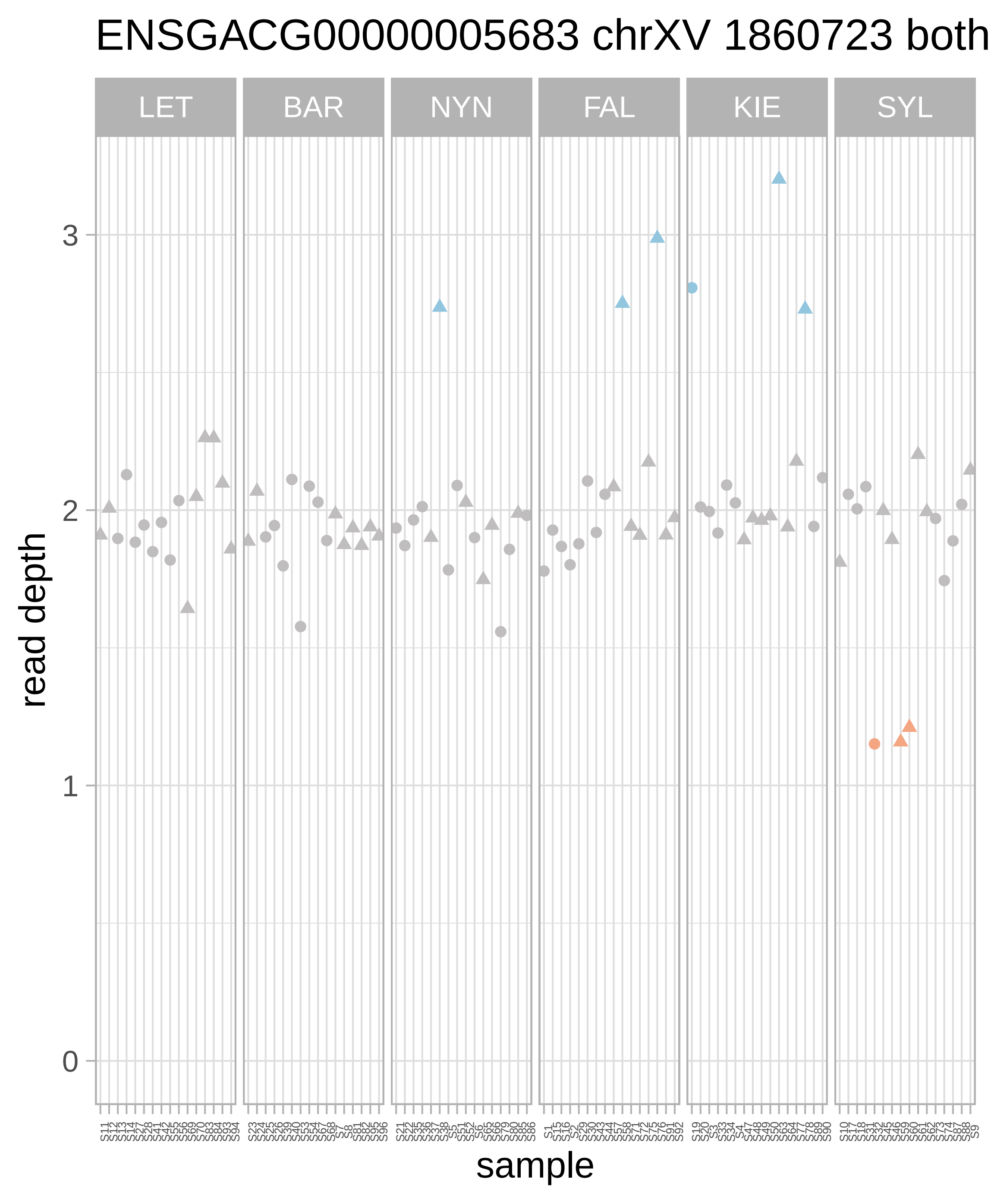

ENSGACG00000005683 chrXV 1860723 both
LET
BAR
NYN
FAL
KIE
SYL
read depth
0
1
2
3
sample

ENSGACG00000005932 chrXV 2183537 dup

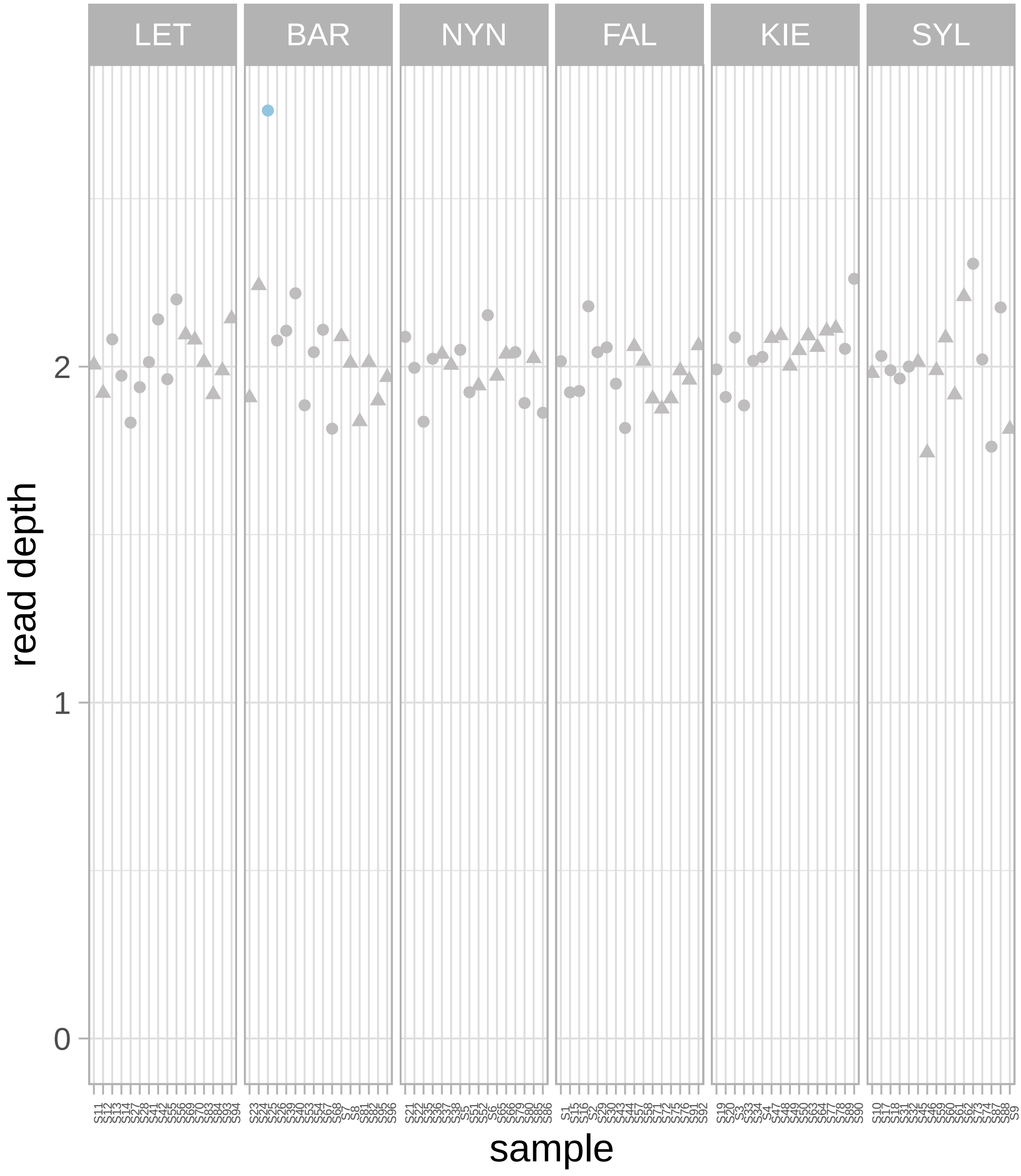

ENSGACG00000006881 chrXV 3525712 dup

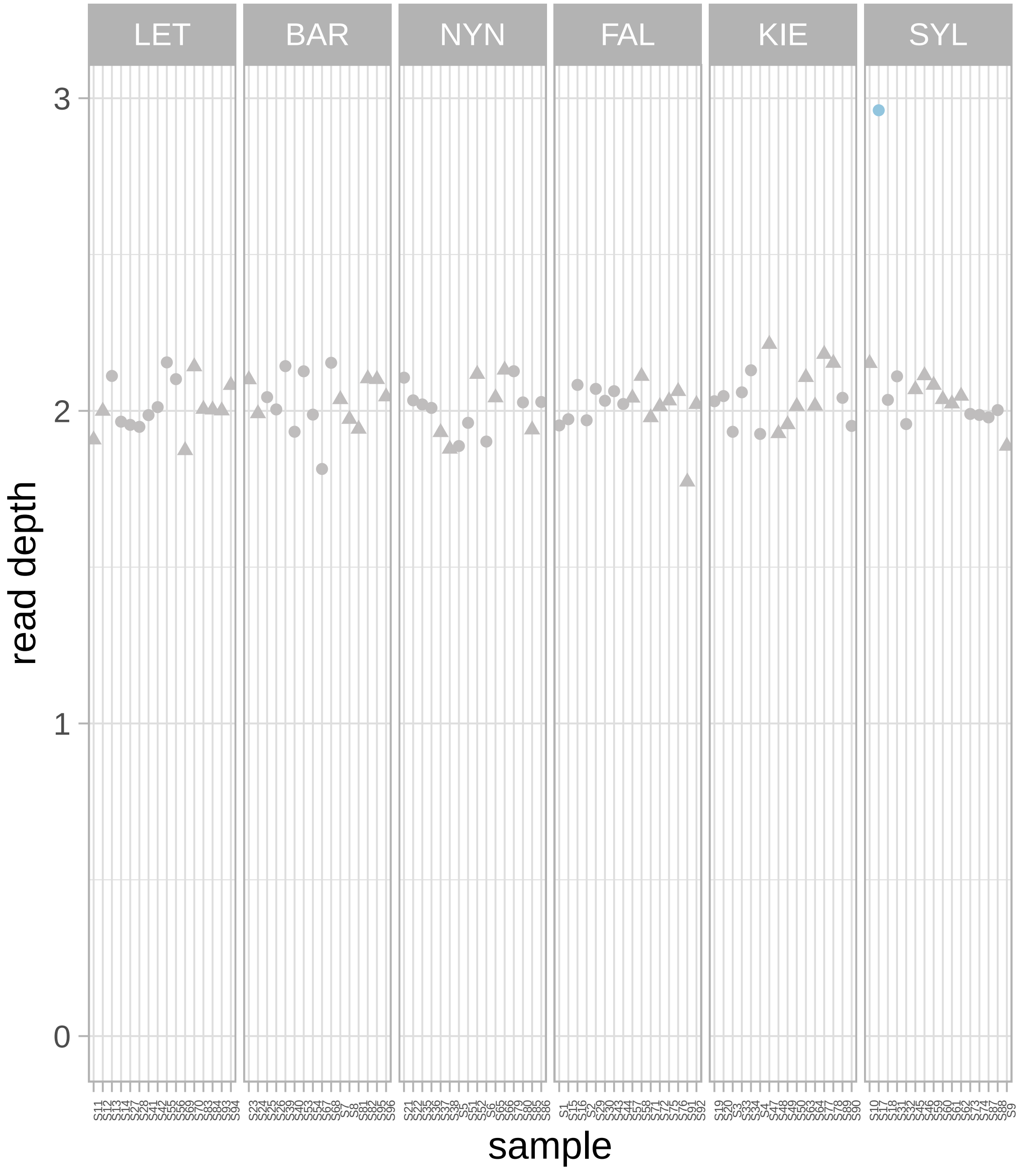

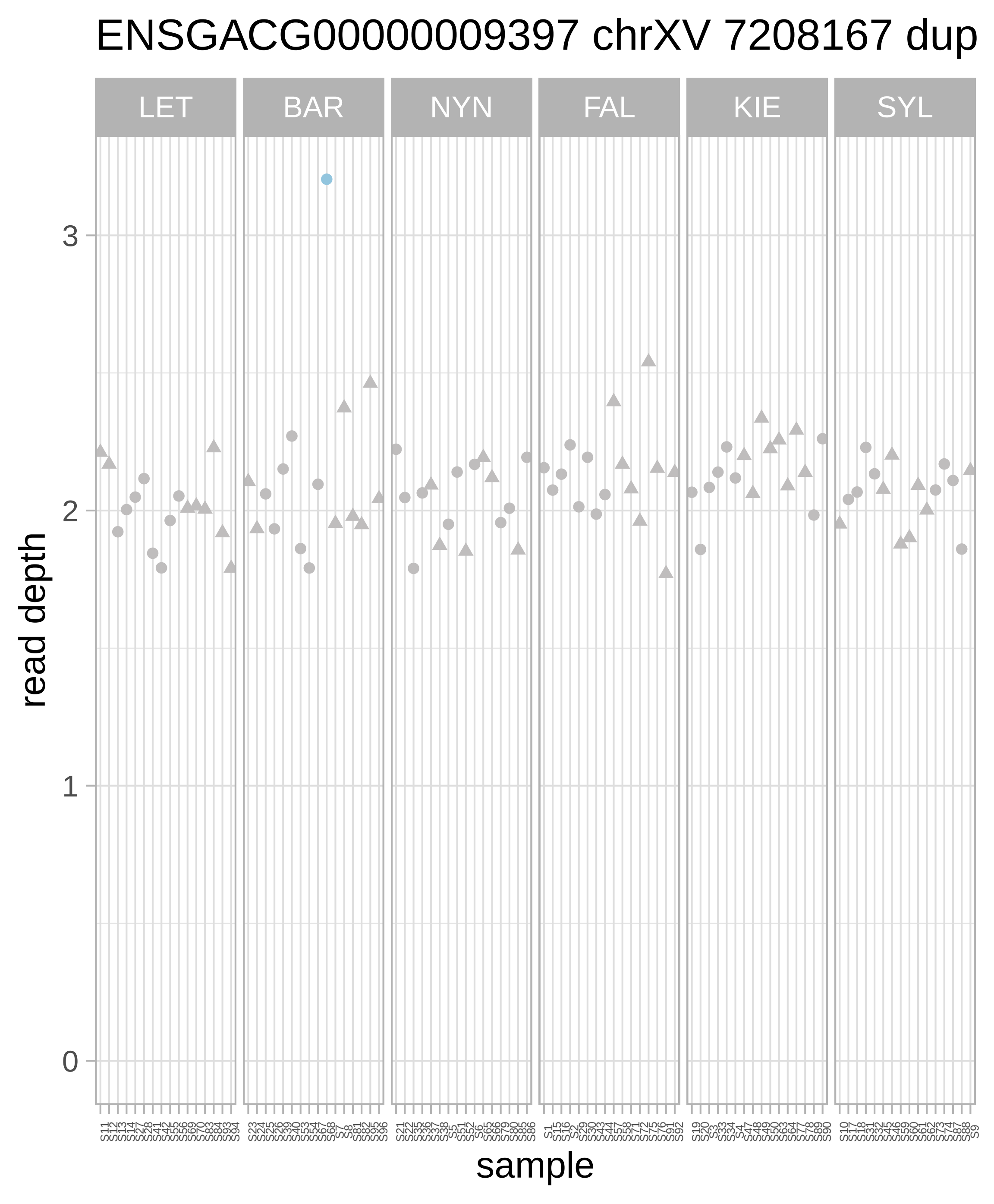

ENSGACG00000009397 chrXV 7208167 dup
LET
BAR
NYN
FAL
KIE
SYL
read depth
0
1
2
3
S11 S12 S13 S14 S27 S28 S41 S42 S55 S56 S69 S70 S83 S84 S93 S94
S23 S24 S25 S26 S39 S40 S53 S54 S67 S68 S7 S8 S81 S82 S95 S96
S21 S22 S35 S36 S37 S38 S5 S51 S52 S6 S65 S66 S79 S80 S85 S86
S1 S15 S16 S2 S29 S30 S43 S44 S57 S58 S71 S72 S75 S76 S91 S92
S19 S20 S3 S33 S34 S4 S47 S48 S49 S50 S63 S64 S77 S78 S89 S90
S10 S17 S18 S31 S32 S45 S46 S59 S60 S61 S62 S73 S74 S87 S88 S9
sample

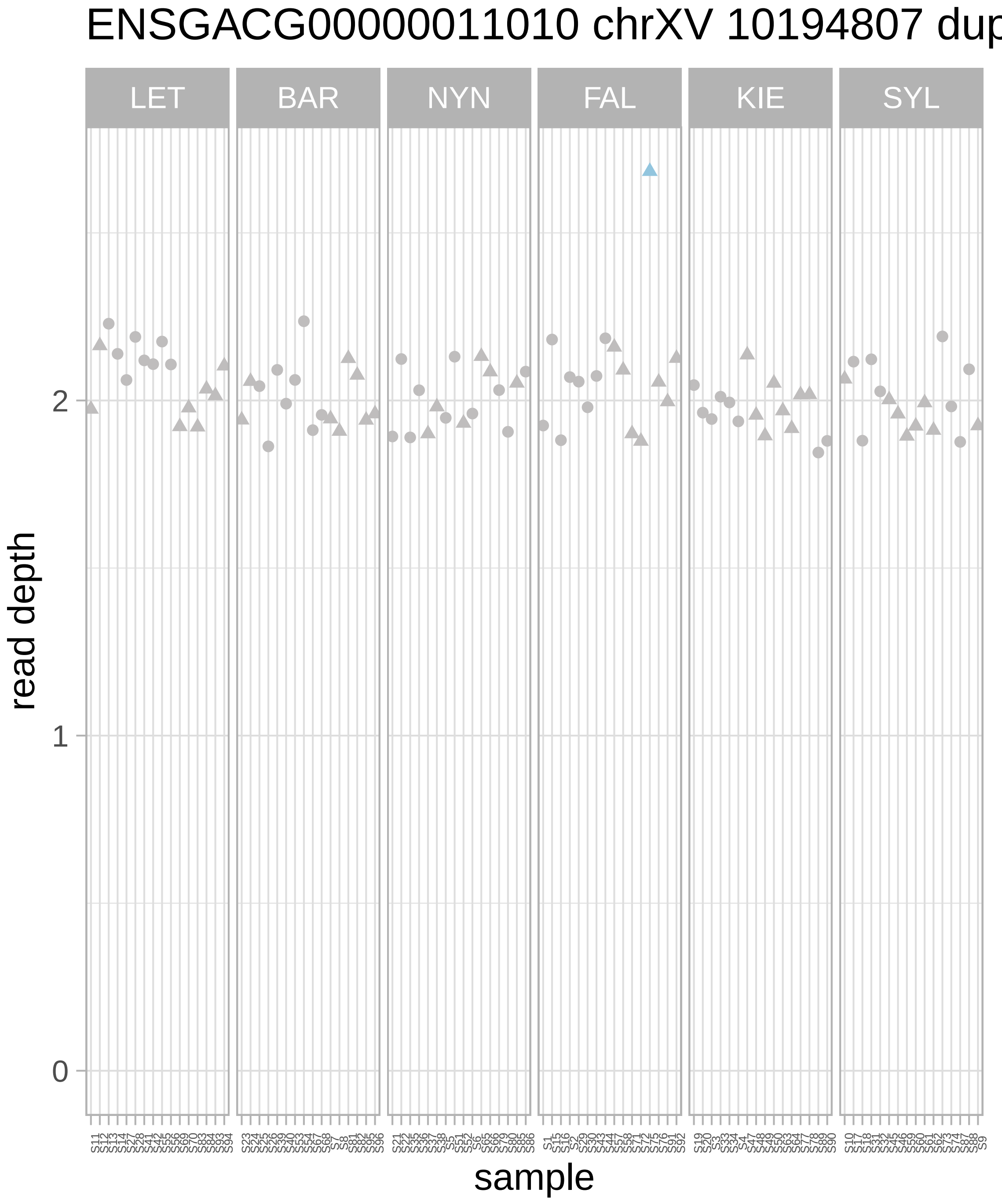
ENSGACG00000011010 chrXV 10194807 dup
LET
BAR
NYN
FAL
KIE
SYL
read depth
0
1
2
sample

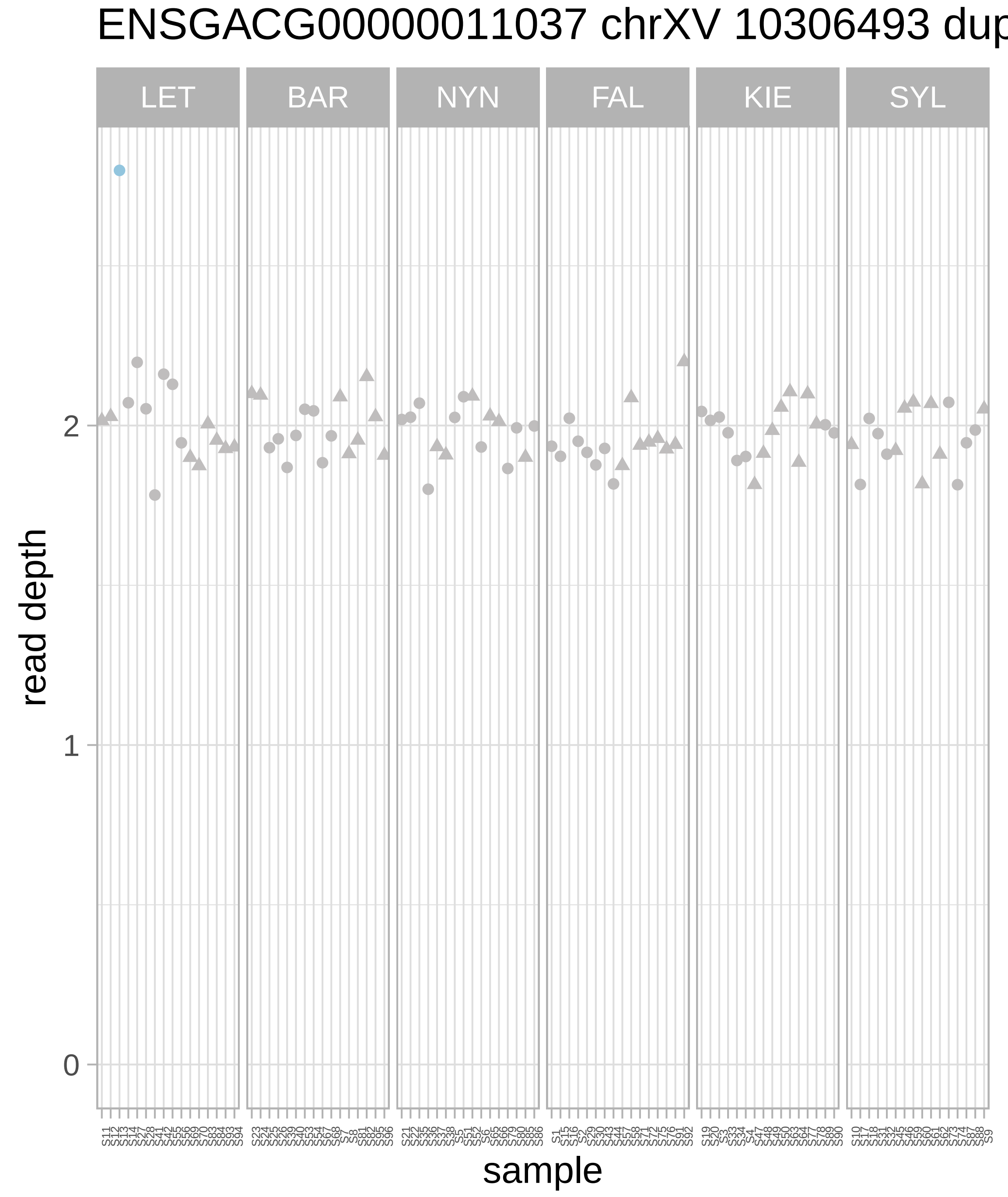
ENSGACG00000011037 chrXV 10306493 dup
LET
BAR
NYN
FAL
KIE
SYL
read depth
2
1
0
sample

ENSGACG00000011756 chrXV 11539450 dup

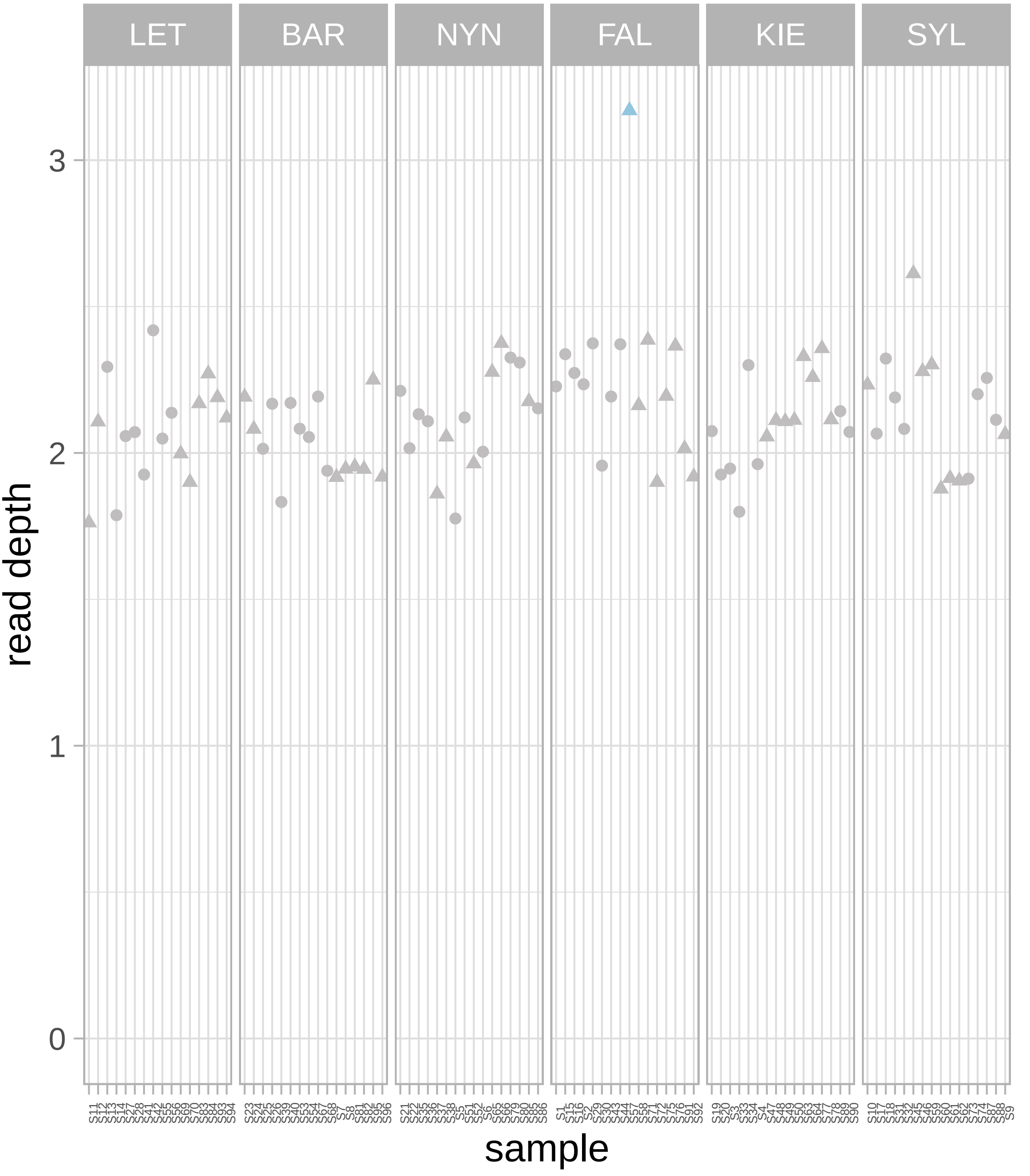

ENSGACG00000011759 chrXV 11545674 dup

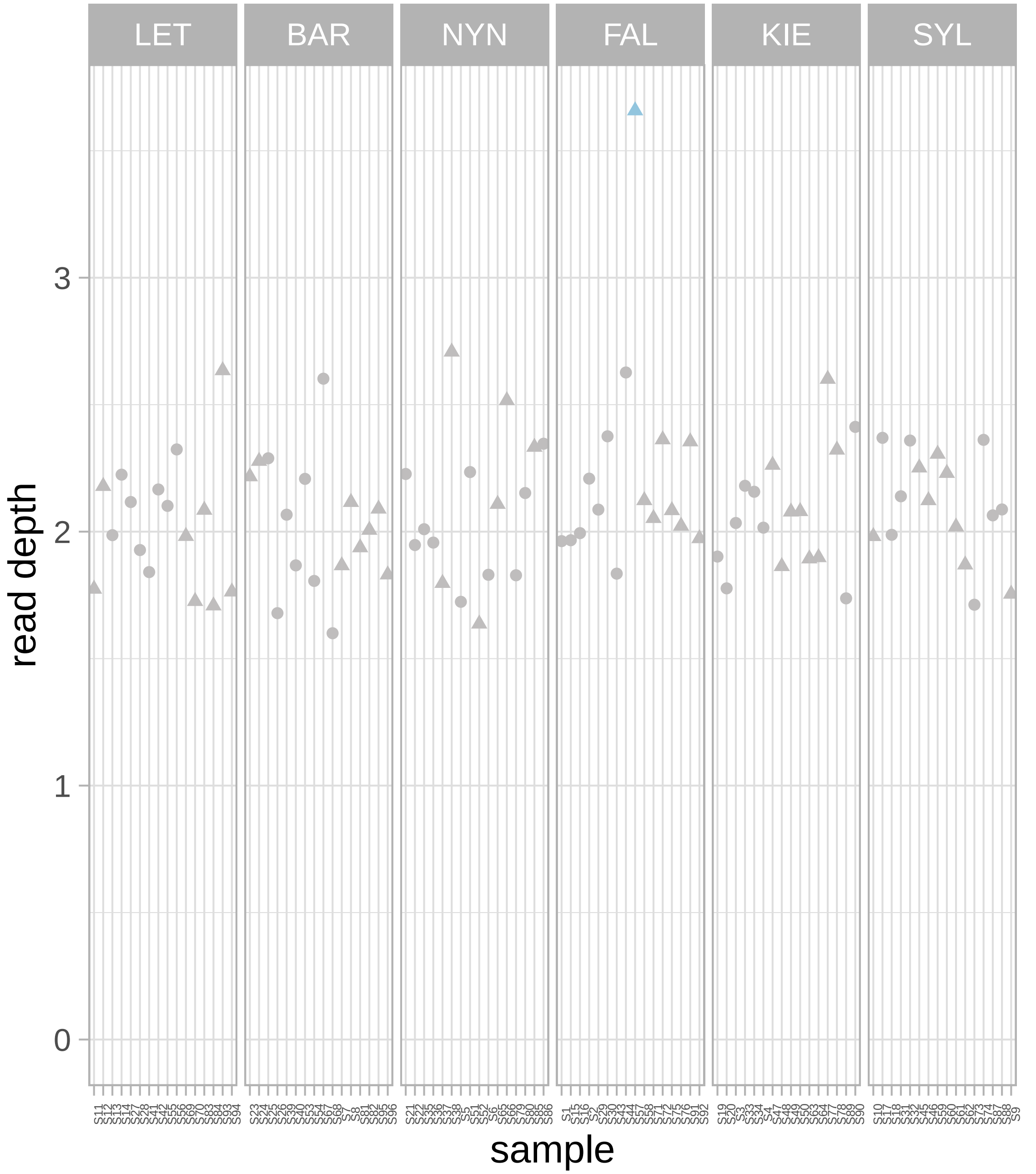

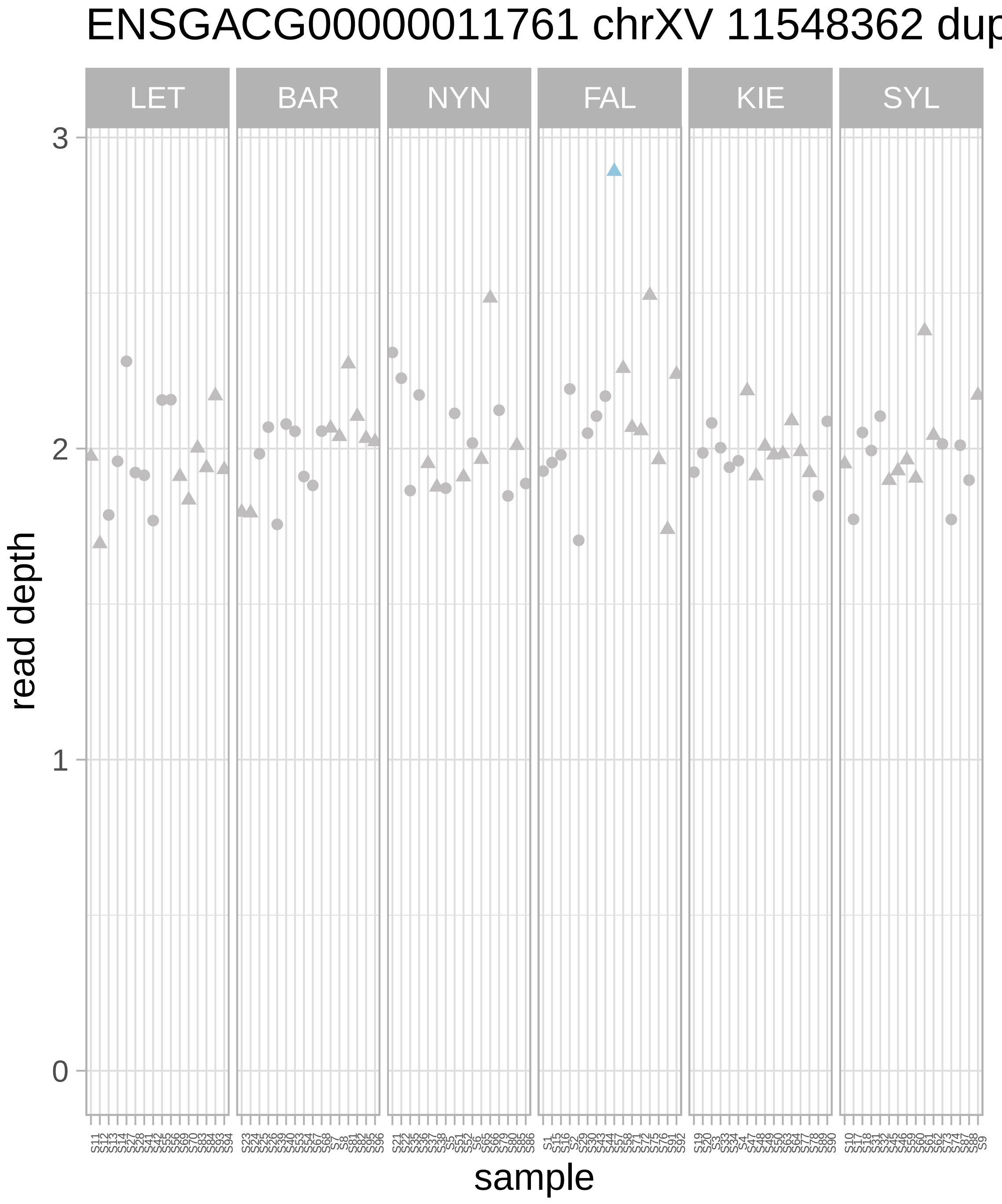
ENSGACG00000011761 chrXV 11548362 dup
LET
BAR
NYN
FAL
KIE
SYL
read depth
3
2
1
0
sample

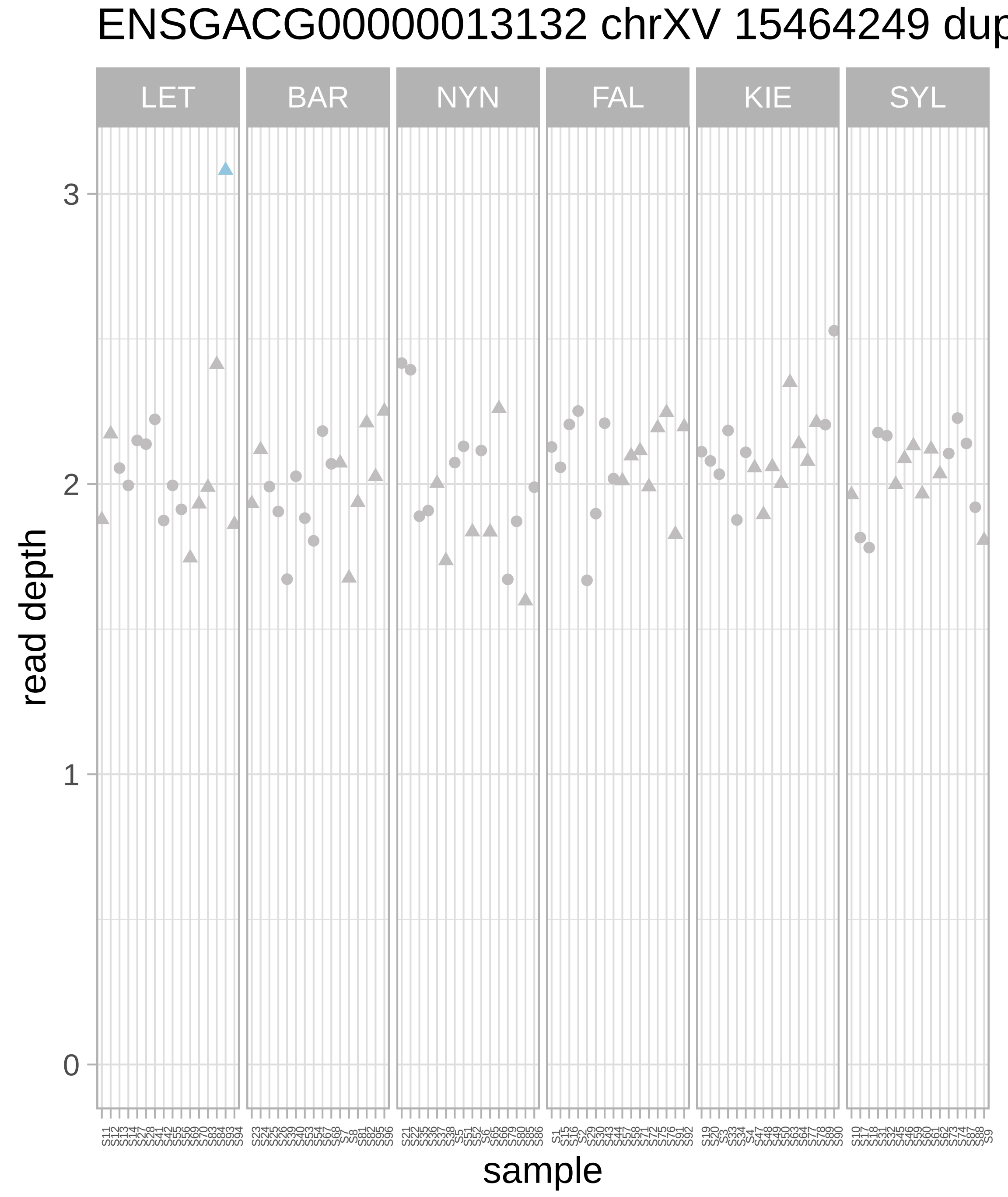
ENSGACG00000013132 chrXV 15464249 dup
LET
BAR
NYN
FAL
KIE
SYL
read depth
0
1
2
3
S11 S12 S13 S14 S27 S28 S41 S42 S55 S56 S69 S70 S83 S84 S93 S94
S23 S24 S25 S26 S39 S40 S53 S54 S67 S68 S7 S8 S81 S82 S95 S96
S21 S22 S35 S36 S37 S38 S5 S51 S52 S6 S65 S66 S79 S80 S85 S86
S1 S15 S16 S2 S29 S30 S43 S44 S57 S58 S71 S72 S75 S76 S91 S92
S19 S20 S3 S33 S34 S4 S47 S48 S49 S50 S63 S64 S77 S78 S89 S90
S10 S17 S18 S31 S32 S45 S46 S59 S60 S61 S62 S73 S74 S87 S88 S9
sample

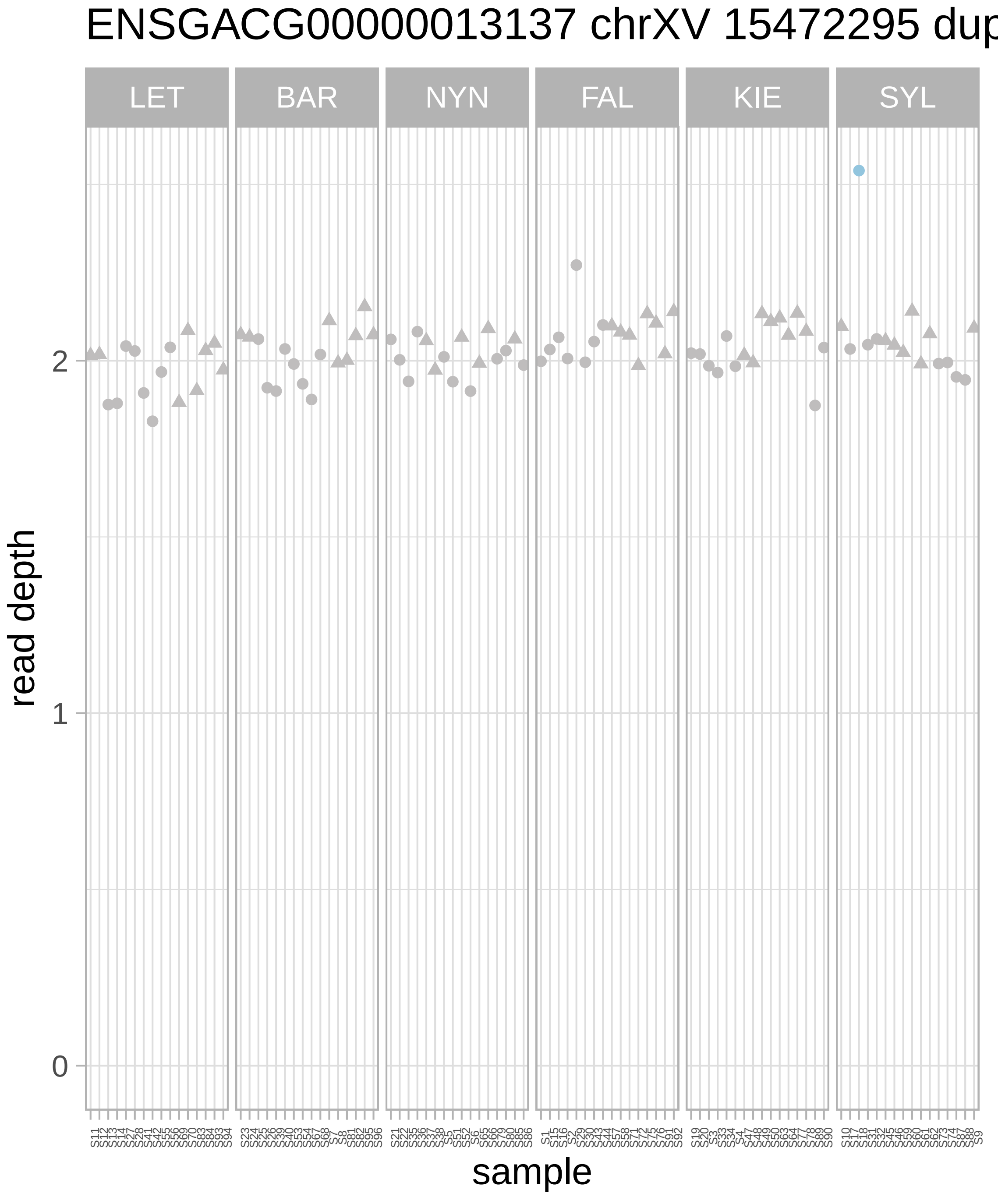

ENSGACG00000013137 chrXV 15472295 dup
LET
BAR
NYN
FAL
KIE
SYL
read depth
0
1
2
sample
S11 S12 S13 S14 S27 S28 S41 S42 S55 S56 S69 S70 S83 S84 S93 S94
S23 S24 S25 S26 S39 S40 S53 S54 S67 S68 S7 S8 S81 S82 S95 S96
S21 S22 S35 S36 S37 S38 S5 S51 S52 S6 S65 S66 S79 S80 S85 S86
S1 S15 S16 S2 S29 S30 S43 S44 S57 S58 S71 S72 S75 S76 S91 S92
S19 S20 S3 S33 S34 S4 S47 S48 S49 S50 S63 S64 S77 S78 S89 S90
S10 S17 S18 S31 S32 S45 S46 S59 S60 S61 S62 S73 S74 S87 S88 S9

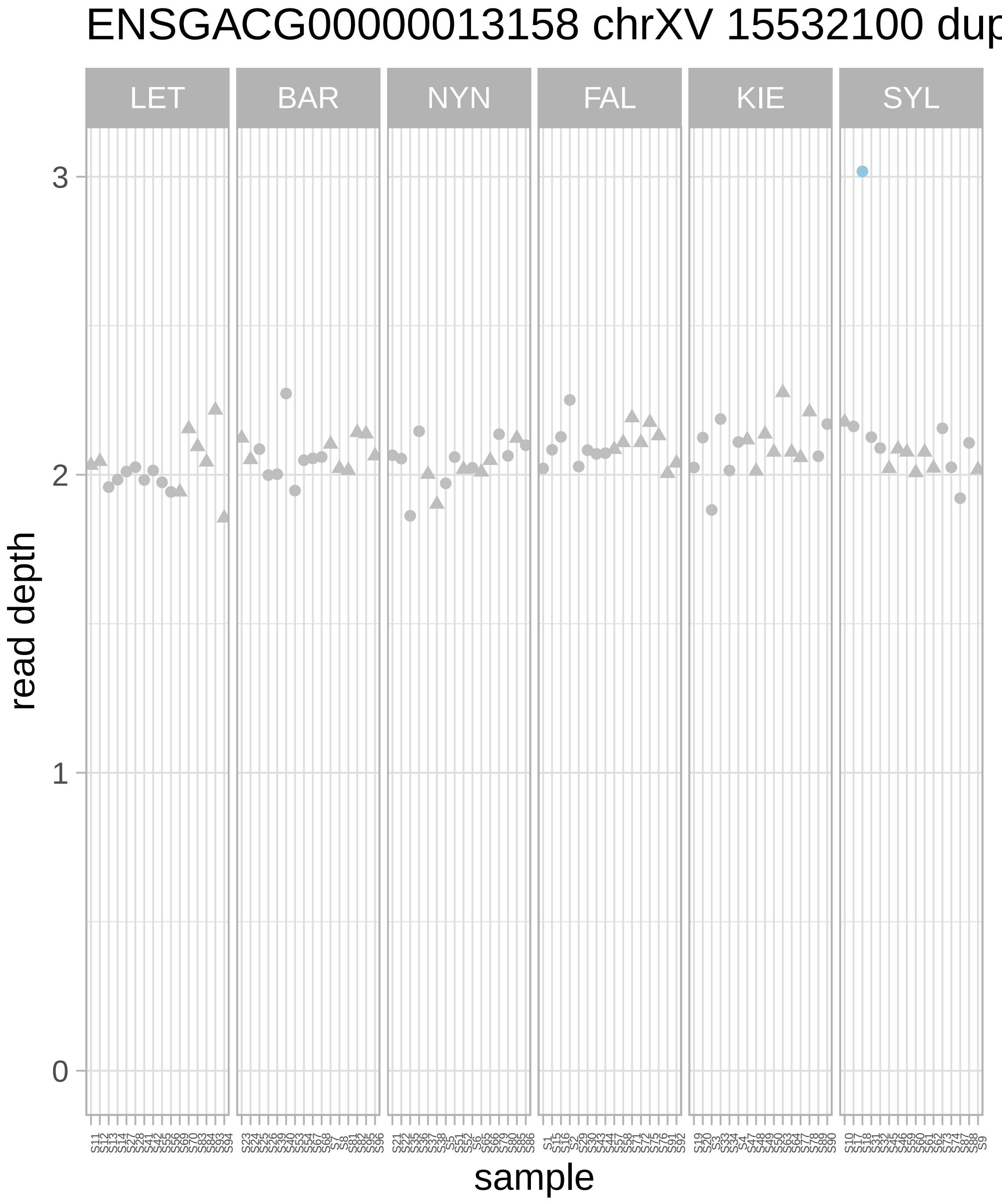
ENSGACG00000013158 chrXV 15532100 dup
LET
BAR
NYN
FAL
KIE
SYL
3
2
1
0
read depth
sample

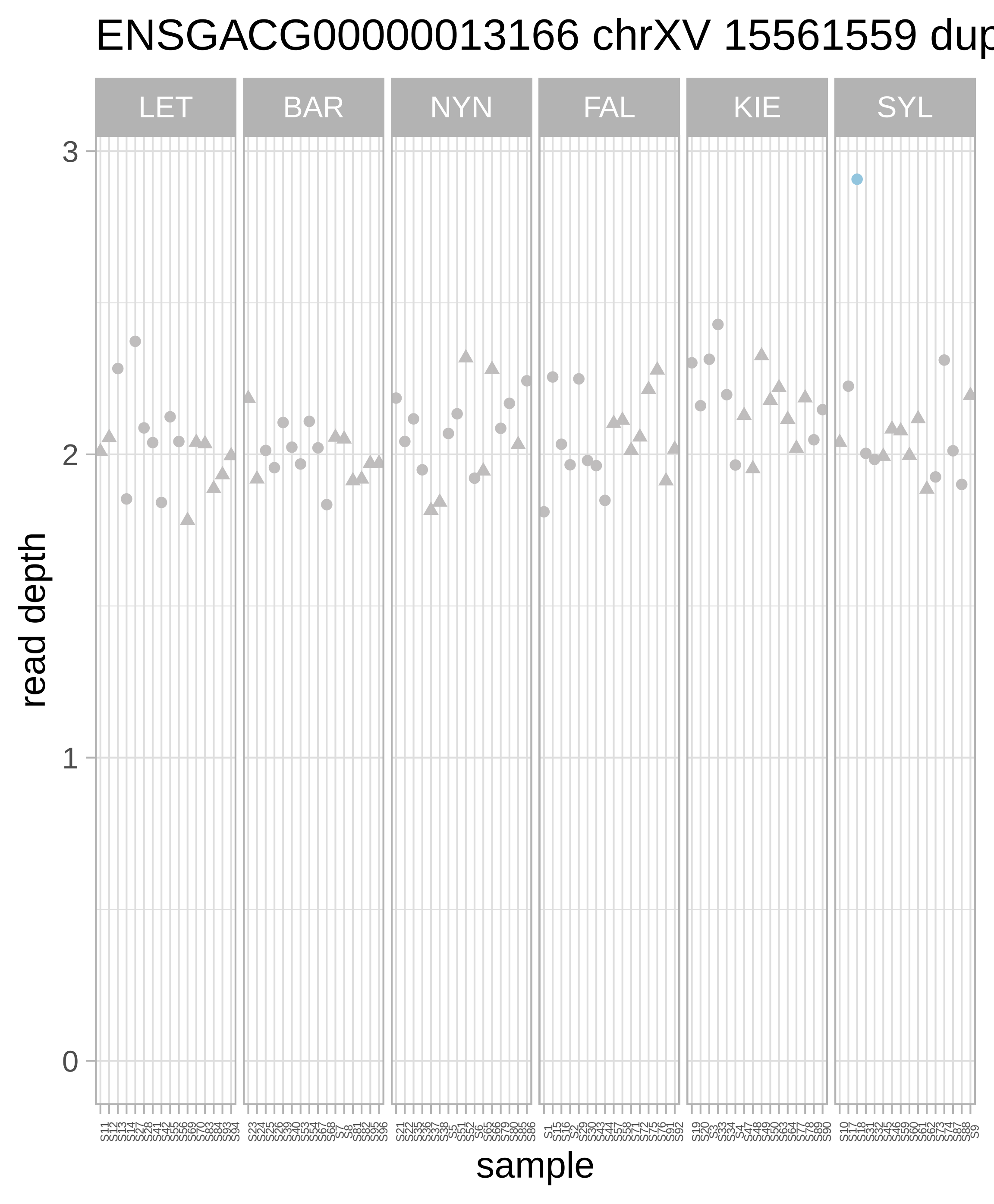

ENSGACG00000013166 chrXV 15561559 dup
LET
BAR
NYN
FAL
KIE
SYL
read depth
0
1
2
3
sample

ENSGACG00000001508 chrXVI 387987 dup

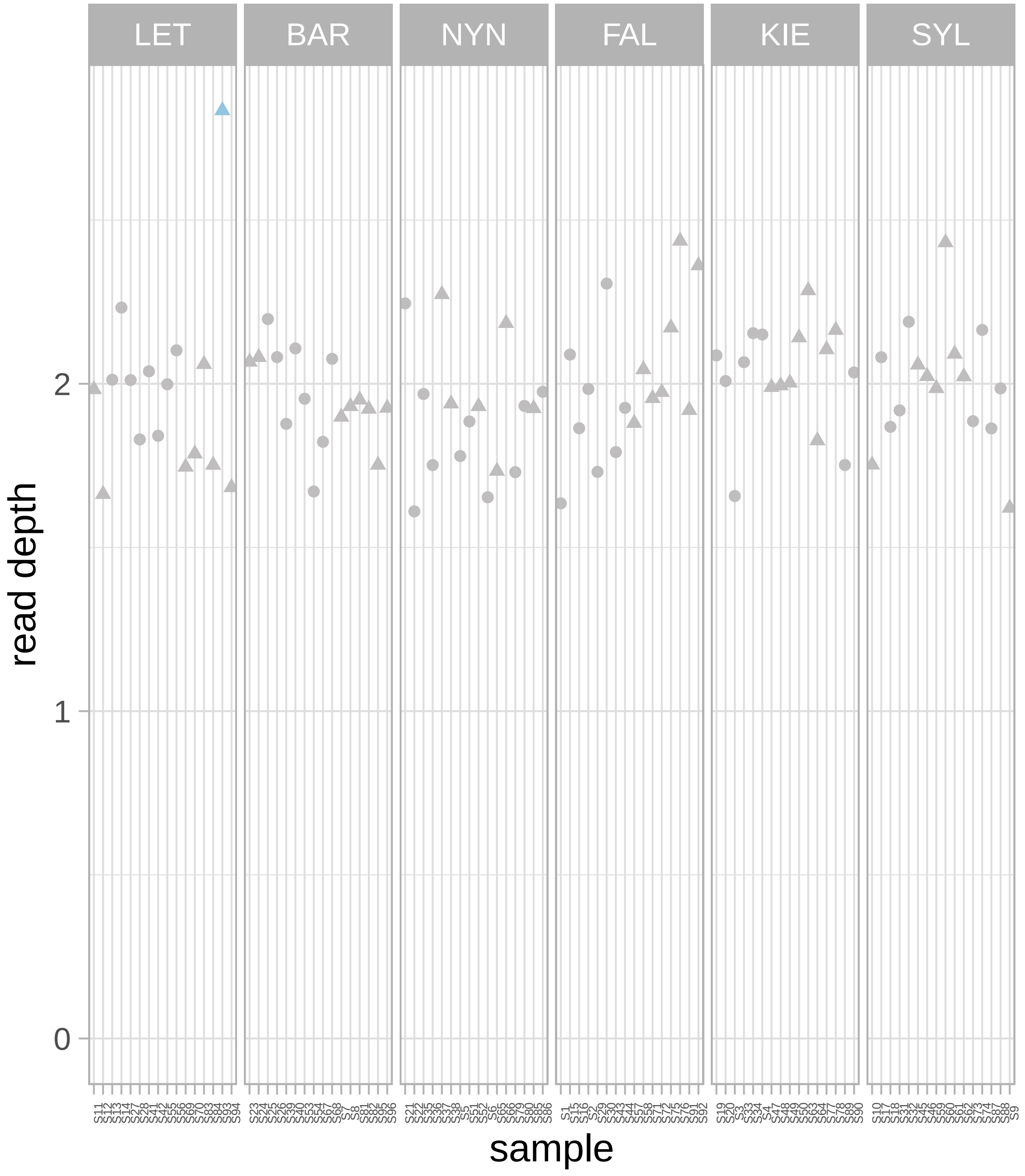

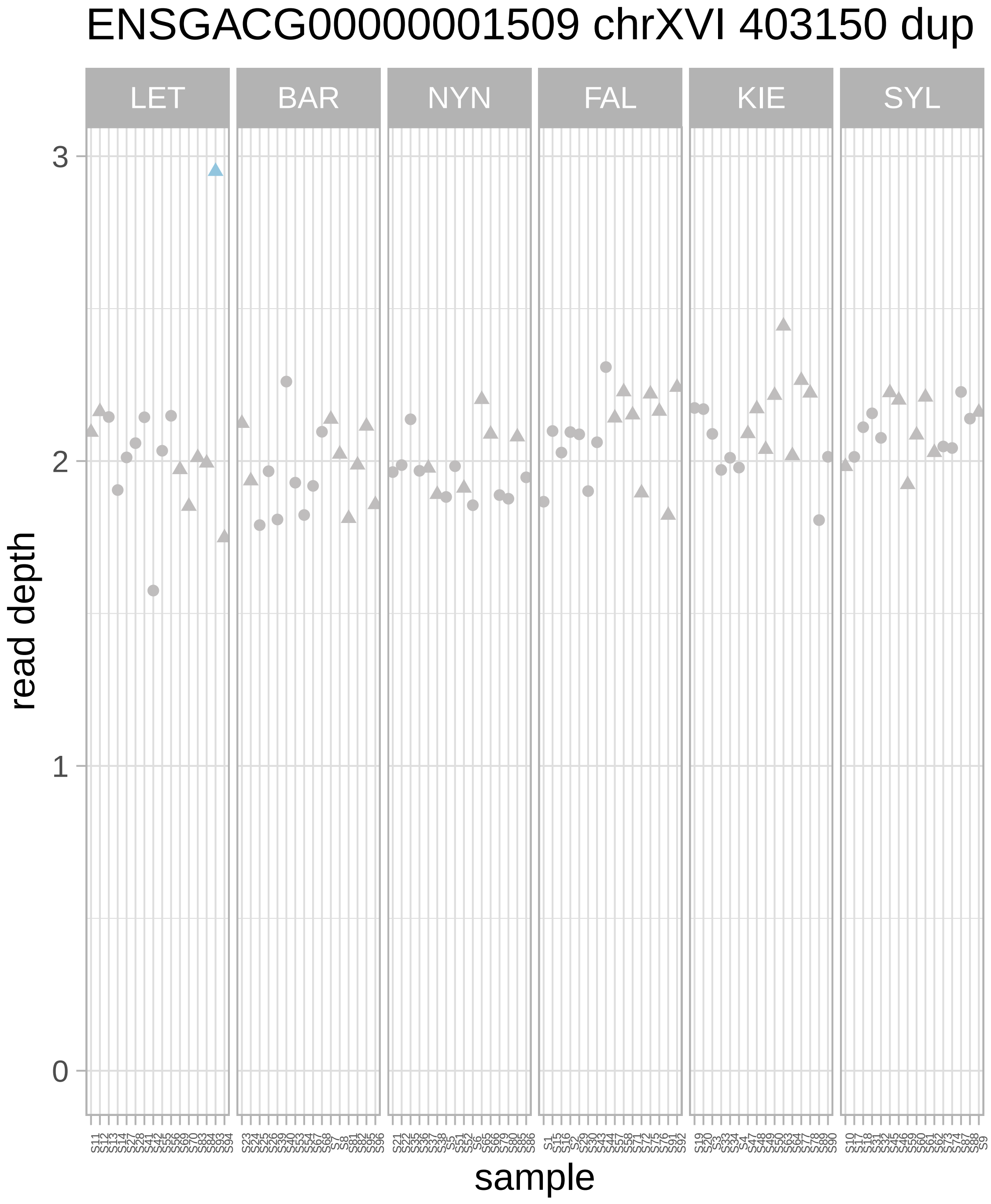
ENSGACG00000001509 chrXVI 403150 dup
LET
BAR
NYN
FAL
KIE
SYL
read depth
0
1
2
3
sample

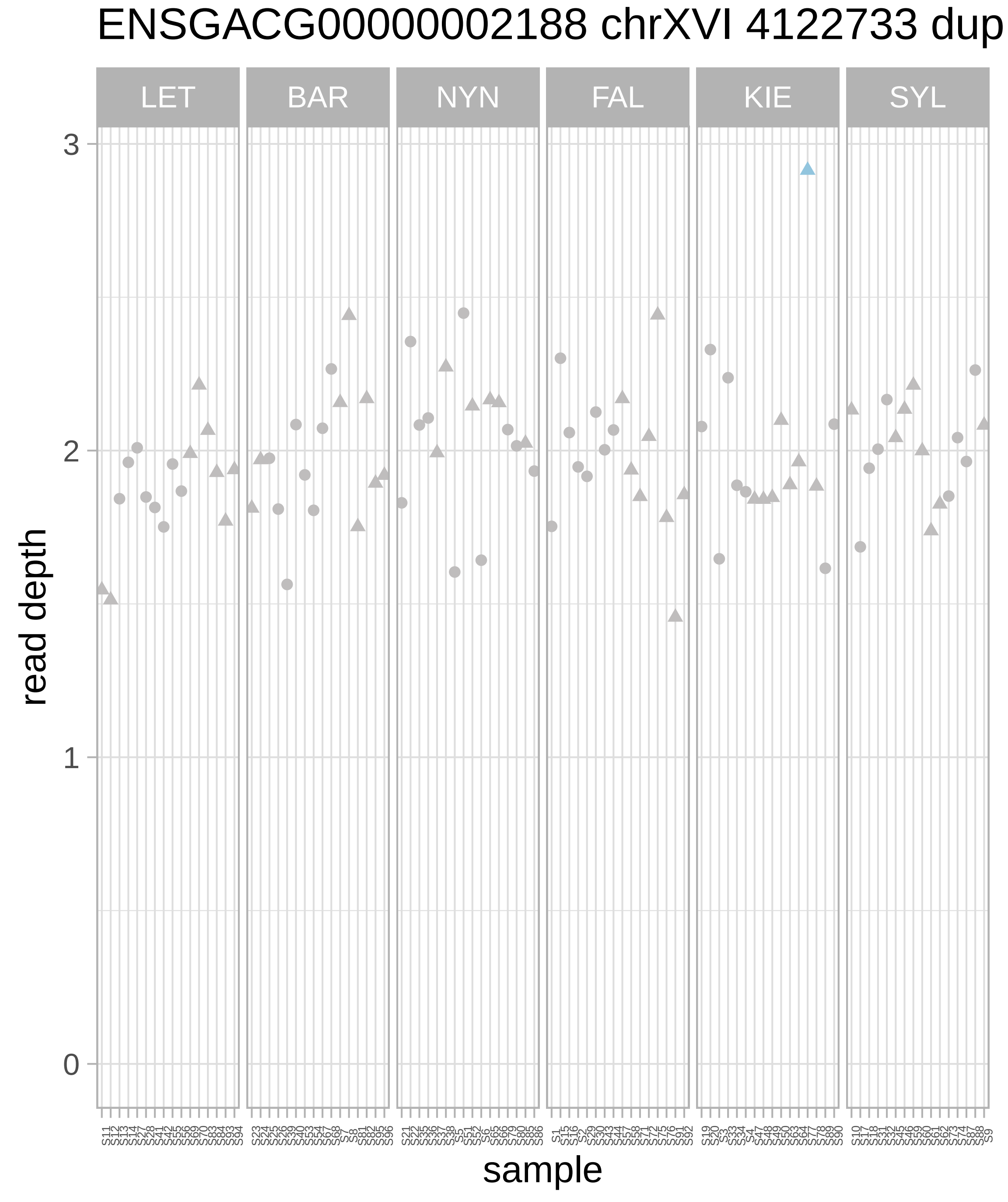
ENSGACG00000002188 chrXVI 4122733 dup
LET
BAR
NYN
FAL
KIE
SYL
3
2
1
0
read depth
S11 S12 S13 S14 S27 S28 S41 S42 S55 S56 S69 S70 S83 S84 S93 S94
S23 S24 S25 S26 S39 S40 S53 S54 S67 S68 S7 S8 S81 S82 S95 S96
S21 S22 S35 S36 S37 S38 S5 S51 S52 S6 S65 S66 S79 S80 S85 S86
S1 S15 S16 S2 S29 S30 S43 S44 S57 S58 S71 S72 S75 S76 S91 S92
S19 S20 S3 S33 S34 S4 S47 S48 S49 S50 S63 S64 S77 S78 S89 S90
S10 S17 S18 S31 S32 S45 S46 S59 S60 S61 S62 S73 S74 S87 S88 S9
sample

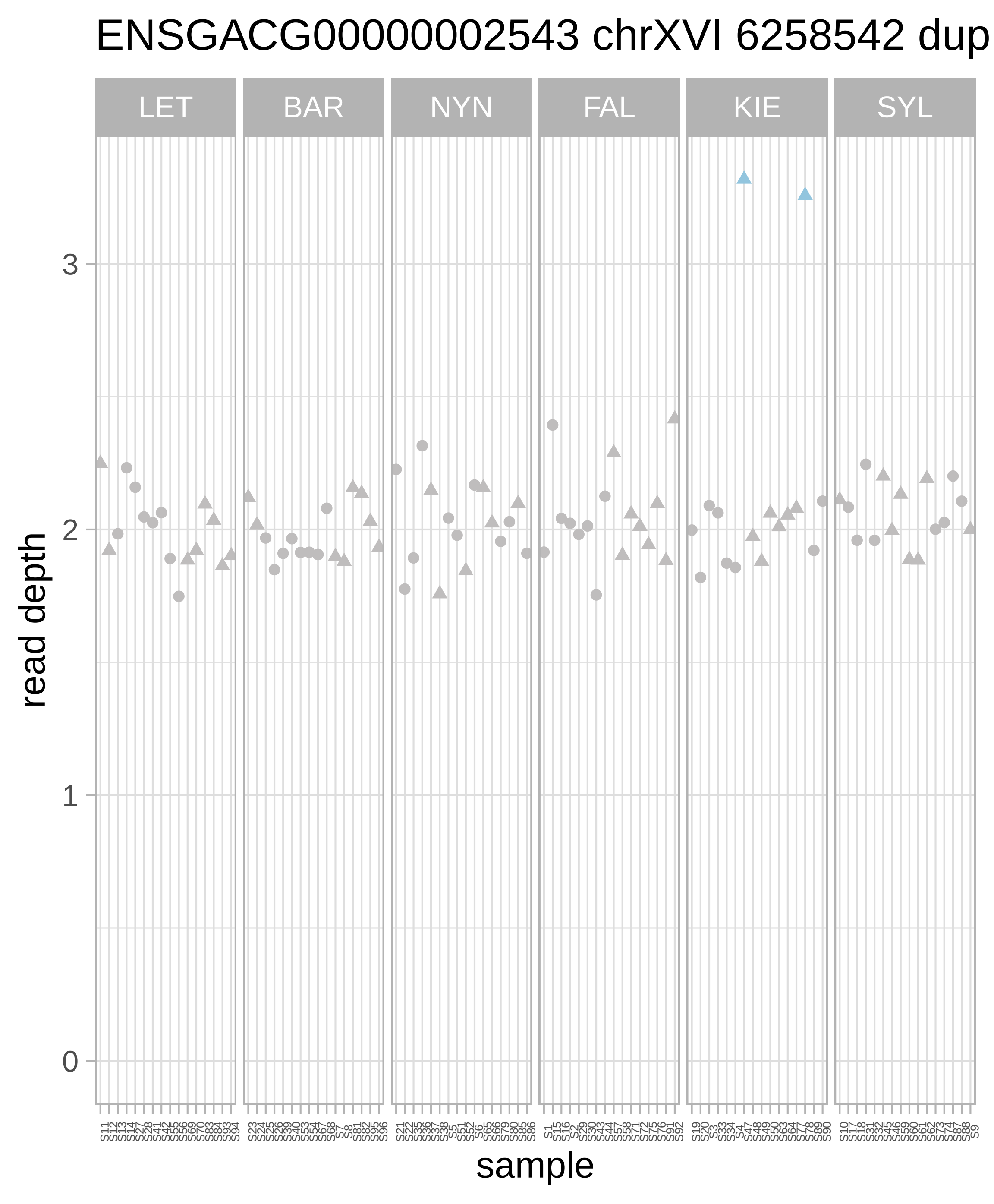
ENSGACG00000002543 chrXVI 6258542 dup
LET
BAR
NYN
FAL
KIE
SYL
read depth
0
1
2
3
sample

ENSGACG00000002735 chrXVI 6467421 dup

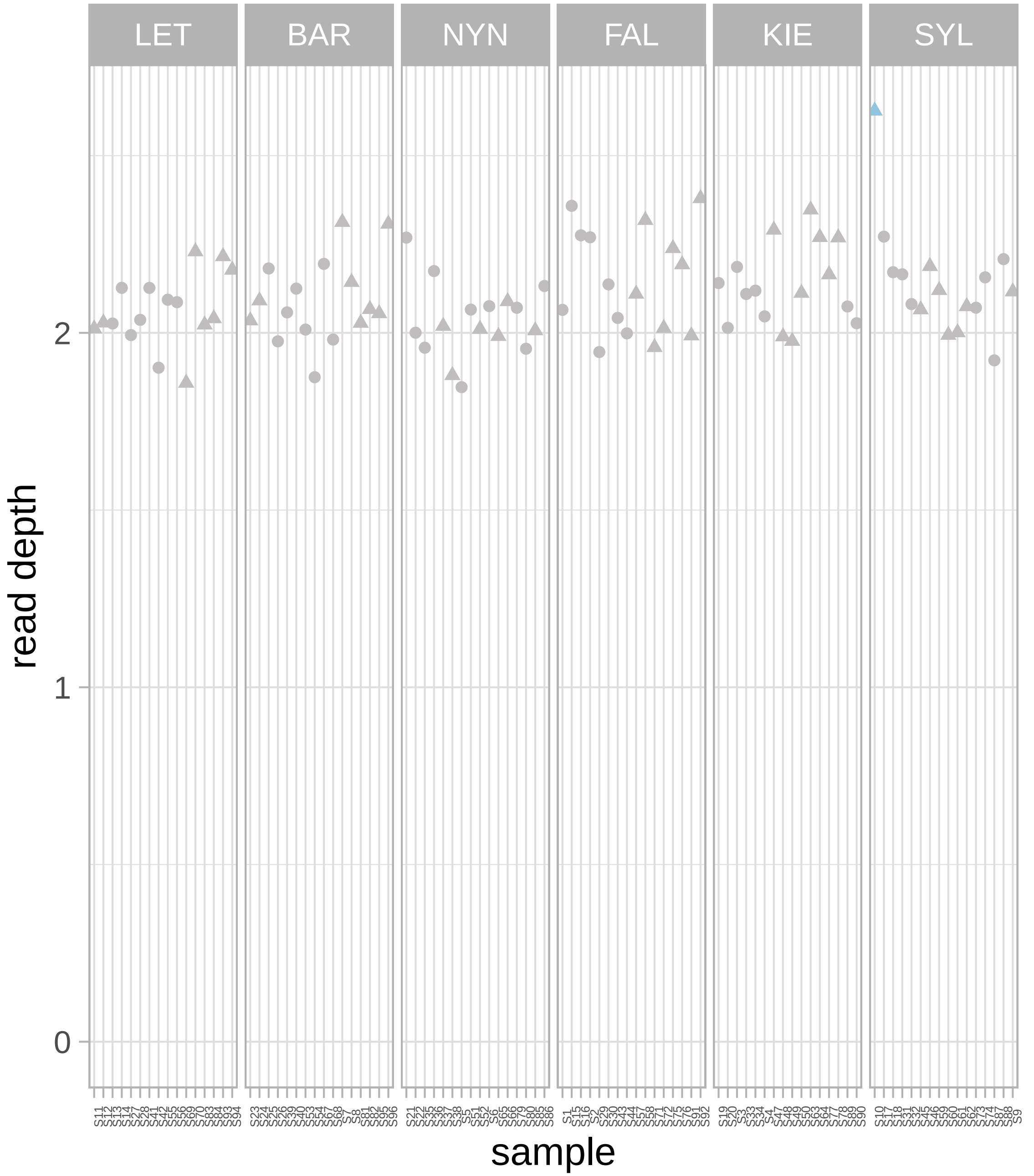

ENSGACG00000002866 chrXVI 7017960 dup

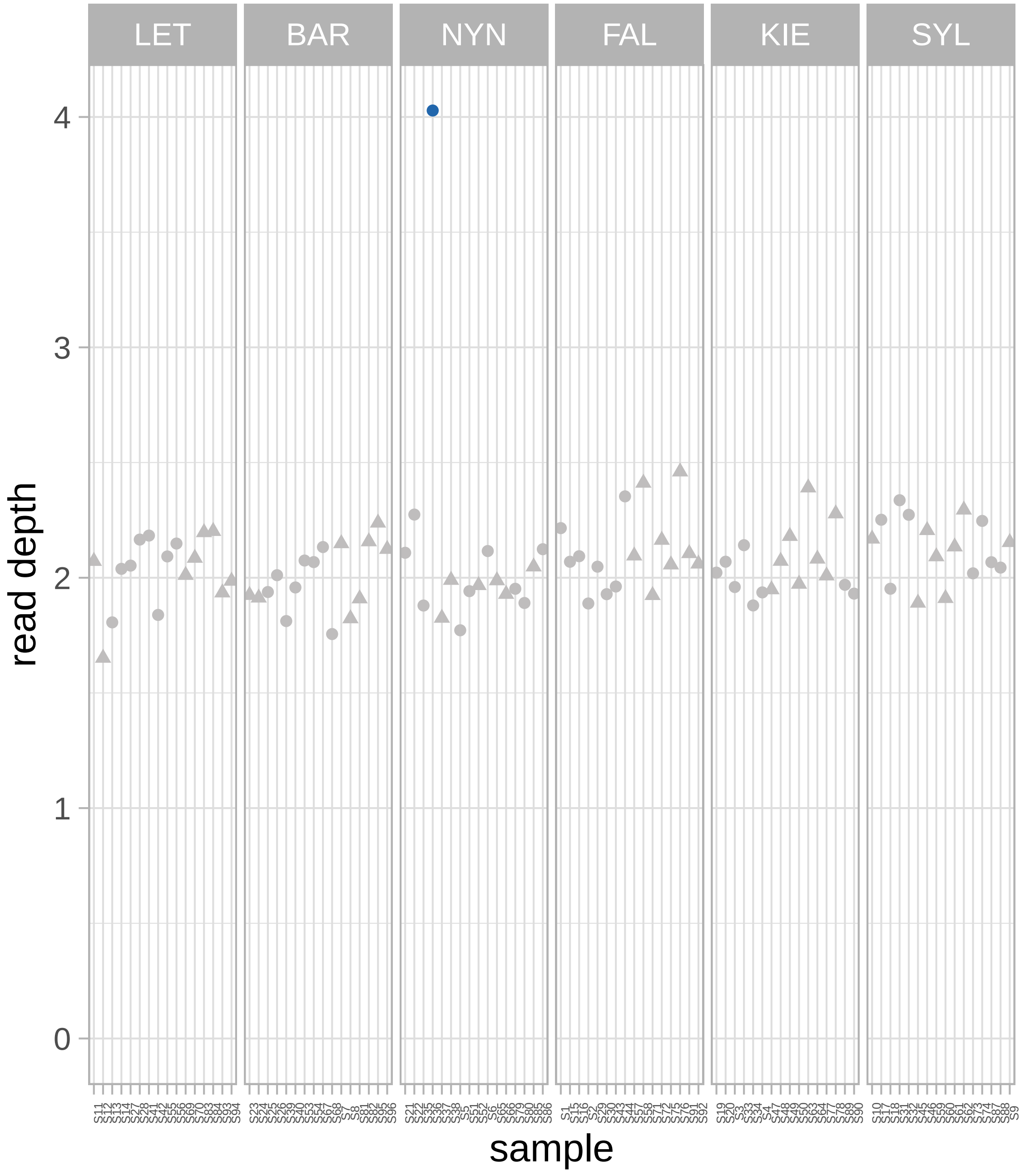

ENSGACG00000003972 chrXVI 8896436 dup

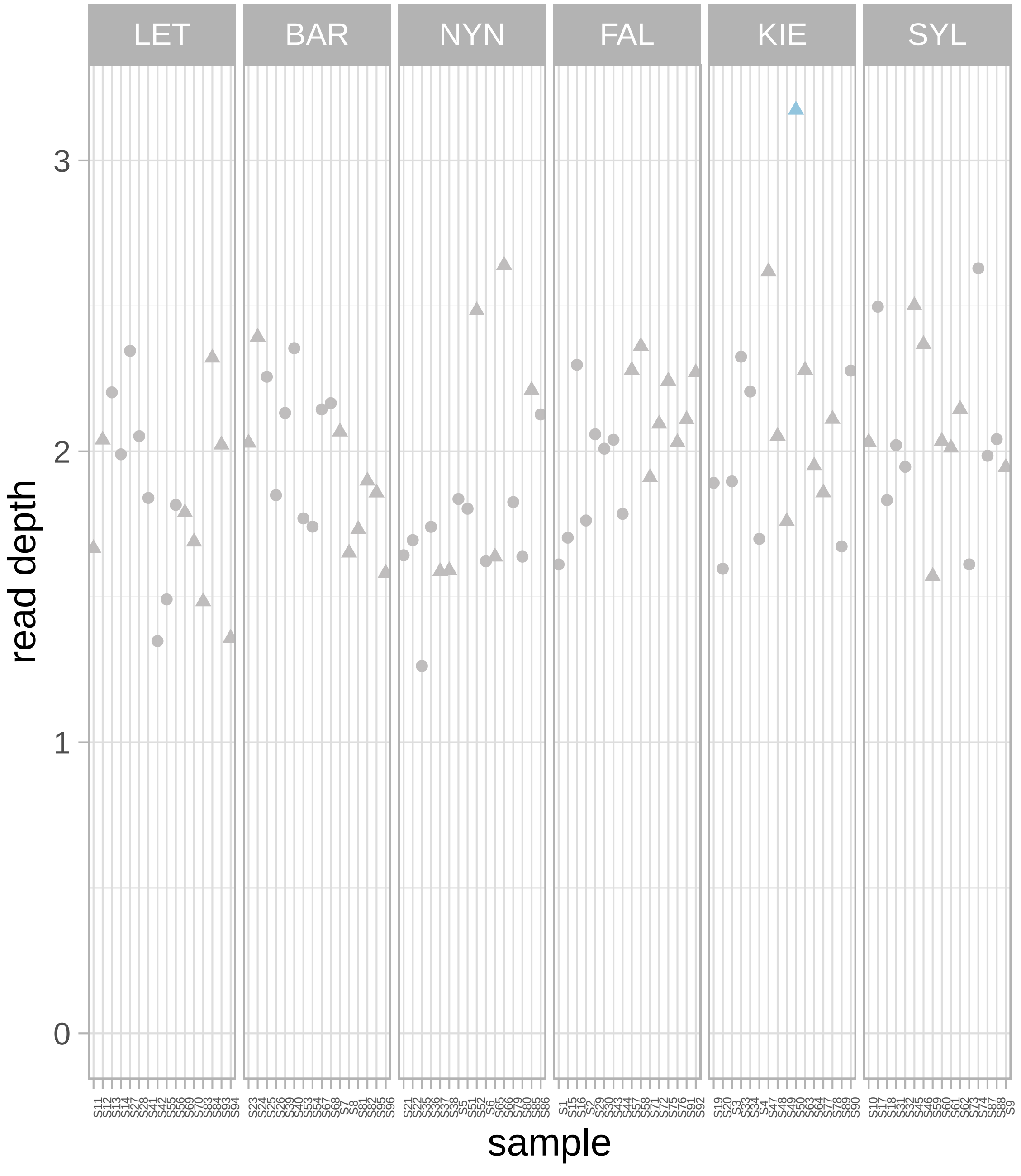

ENSGACG00000004159 chrXVI 9307412 dup

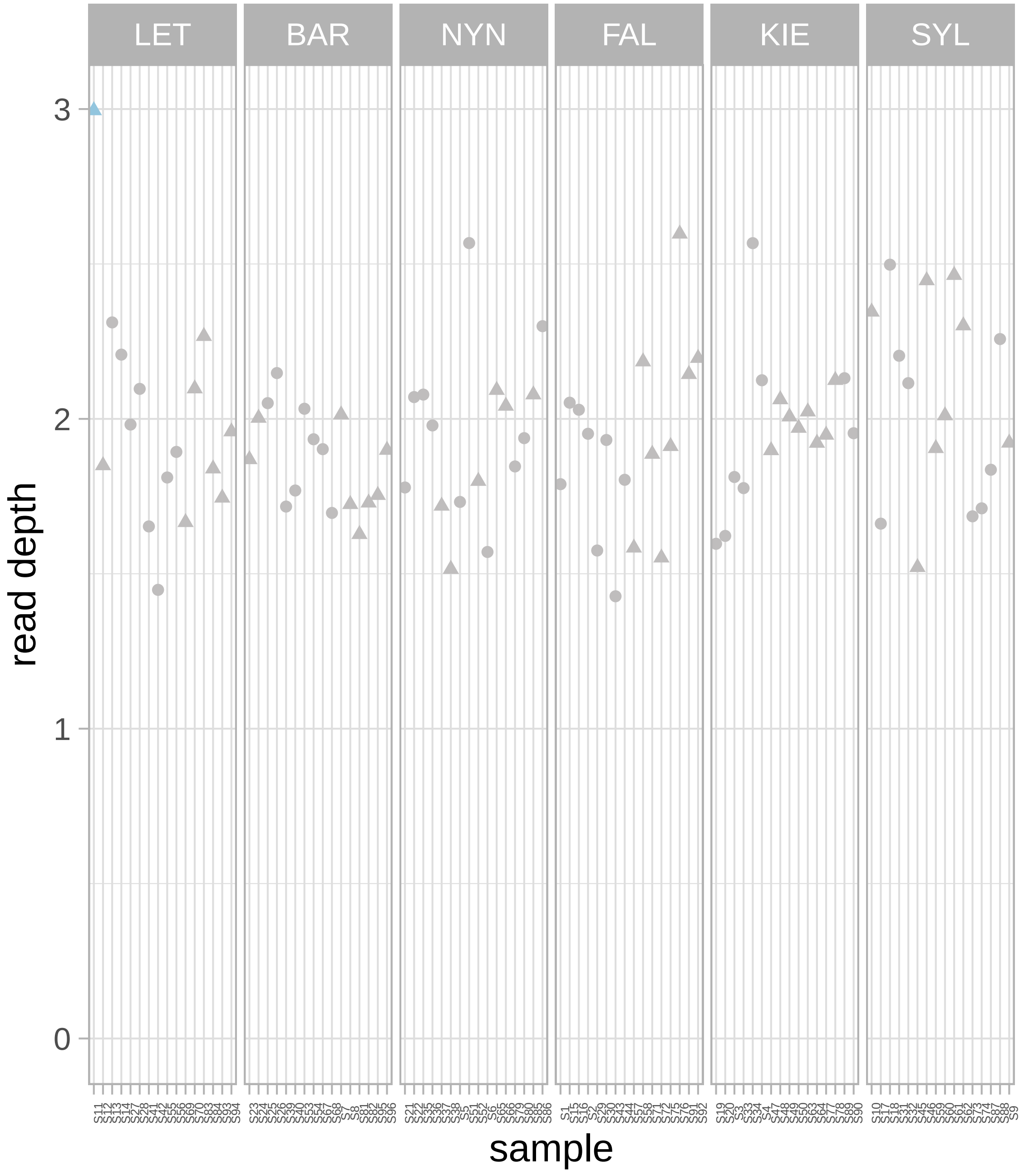

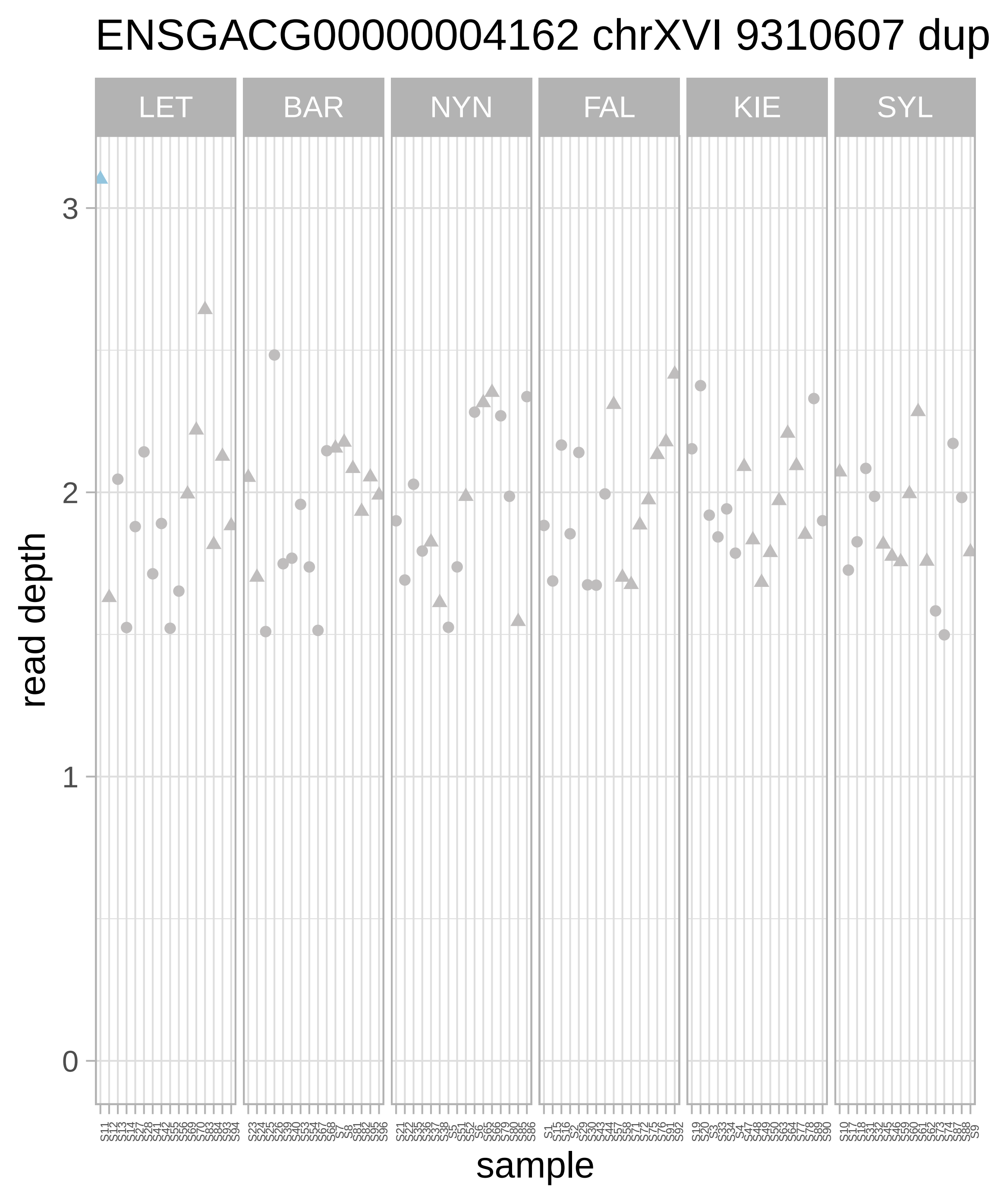

ENSGACG00000004162 chrXVI 9310607 dup
LET
BAR
NYN
FAL
KIE
SYL
read depth
sample
0
1
2
3

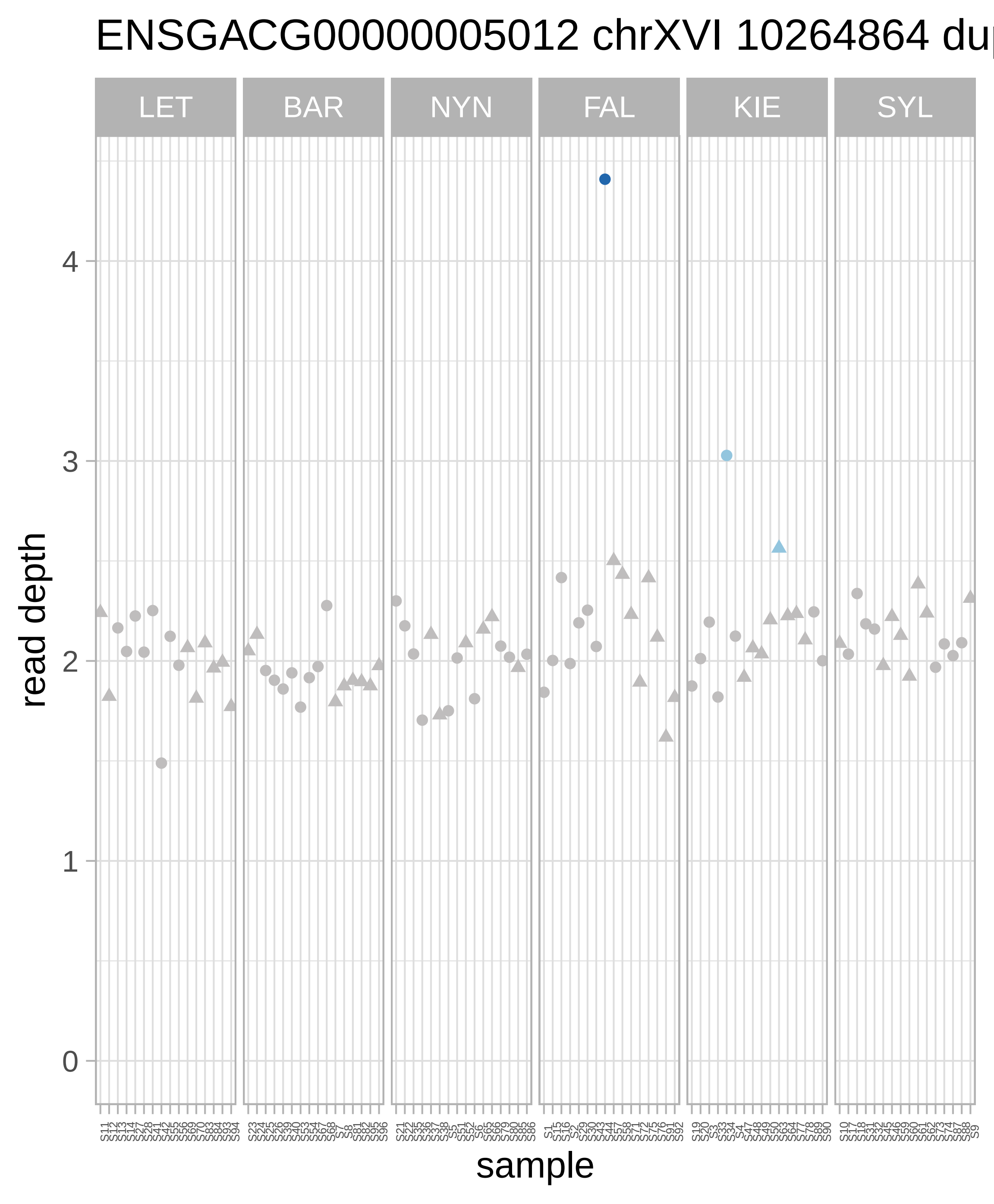
ENSGACG00000005012 chrXVI 10264864 du
LET
BAR
NYN
FAL
KIE
SYL
read depth
0
1
2
3
4
sample

ENSGACG00000007909 chrXVI 15746120 du

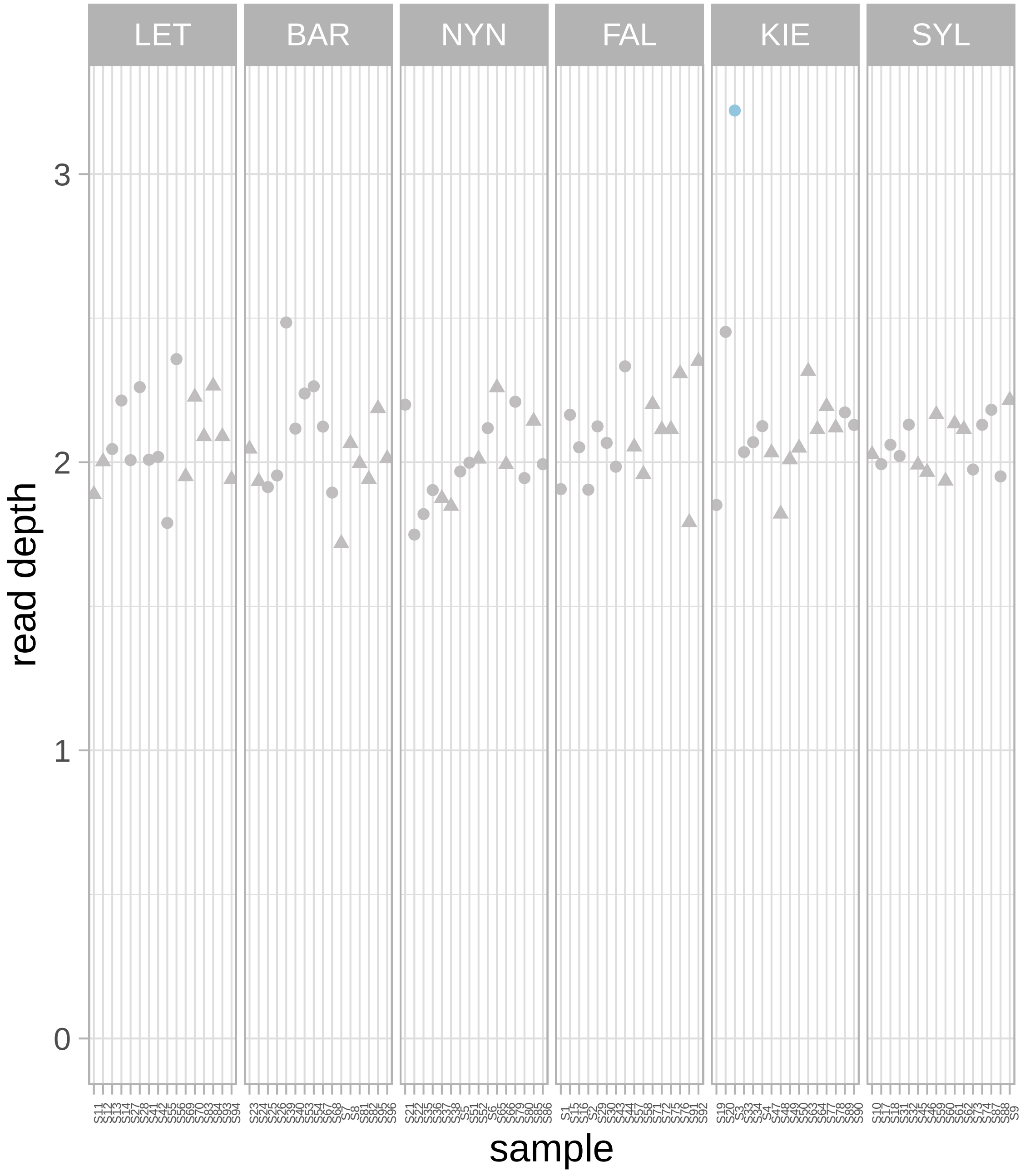

ENSGACG00000004791 chrXVII 2791064 dup

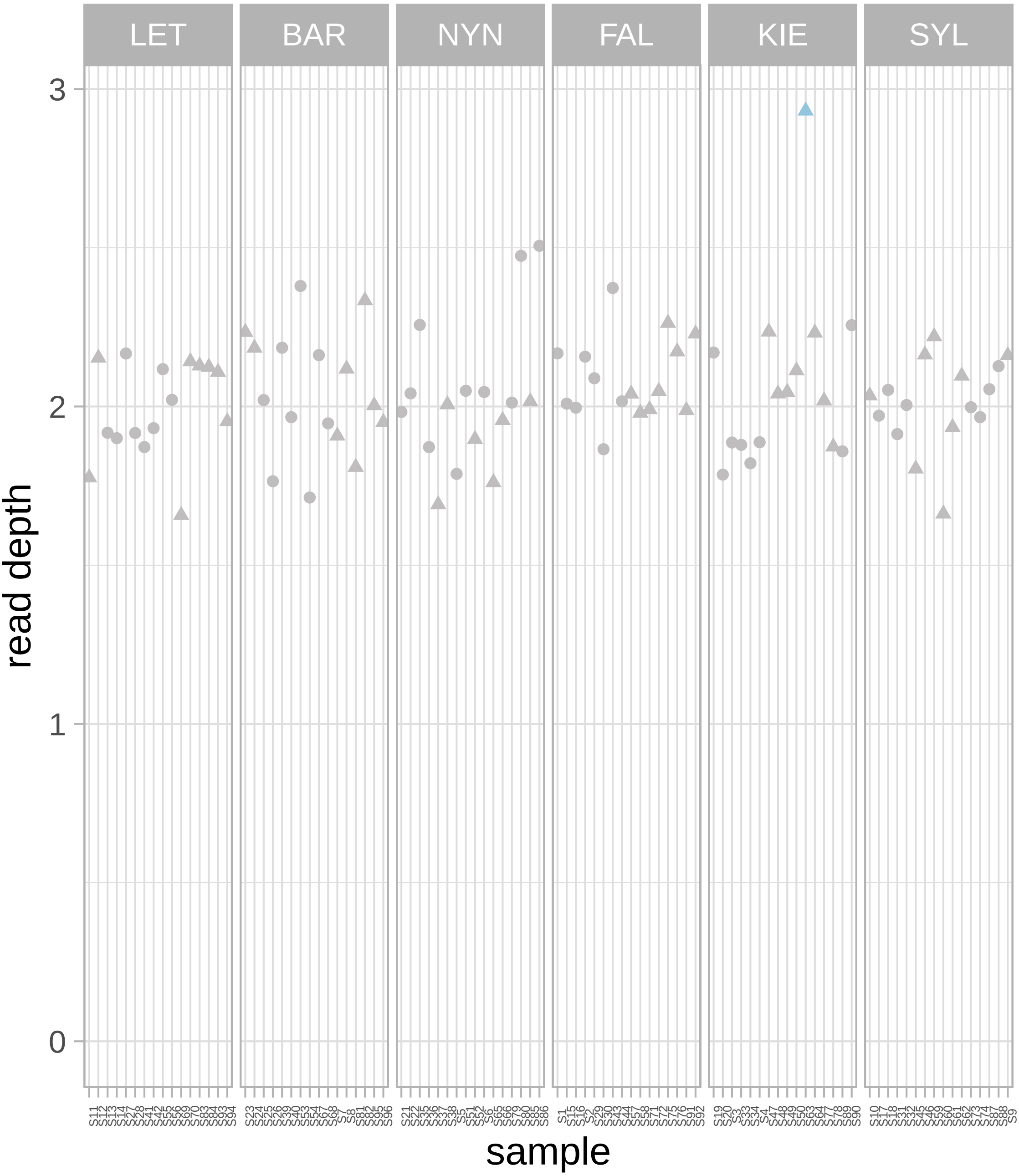

ENSGACG00000006399 chrXVII 5080099 dup

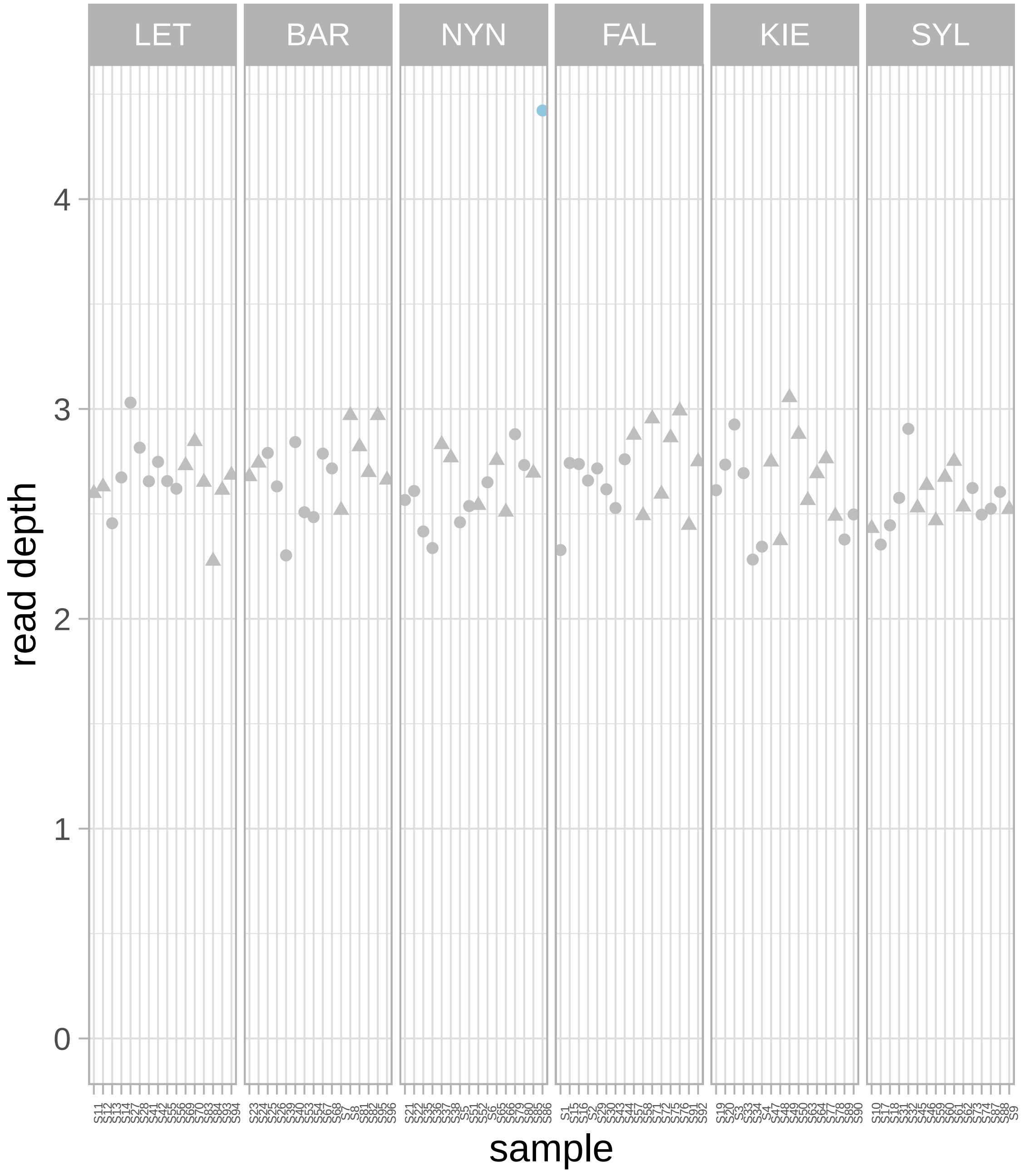

ENSGACG00000006403 chrXVII 5099665 dup

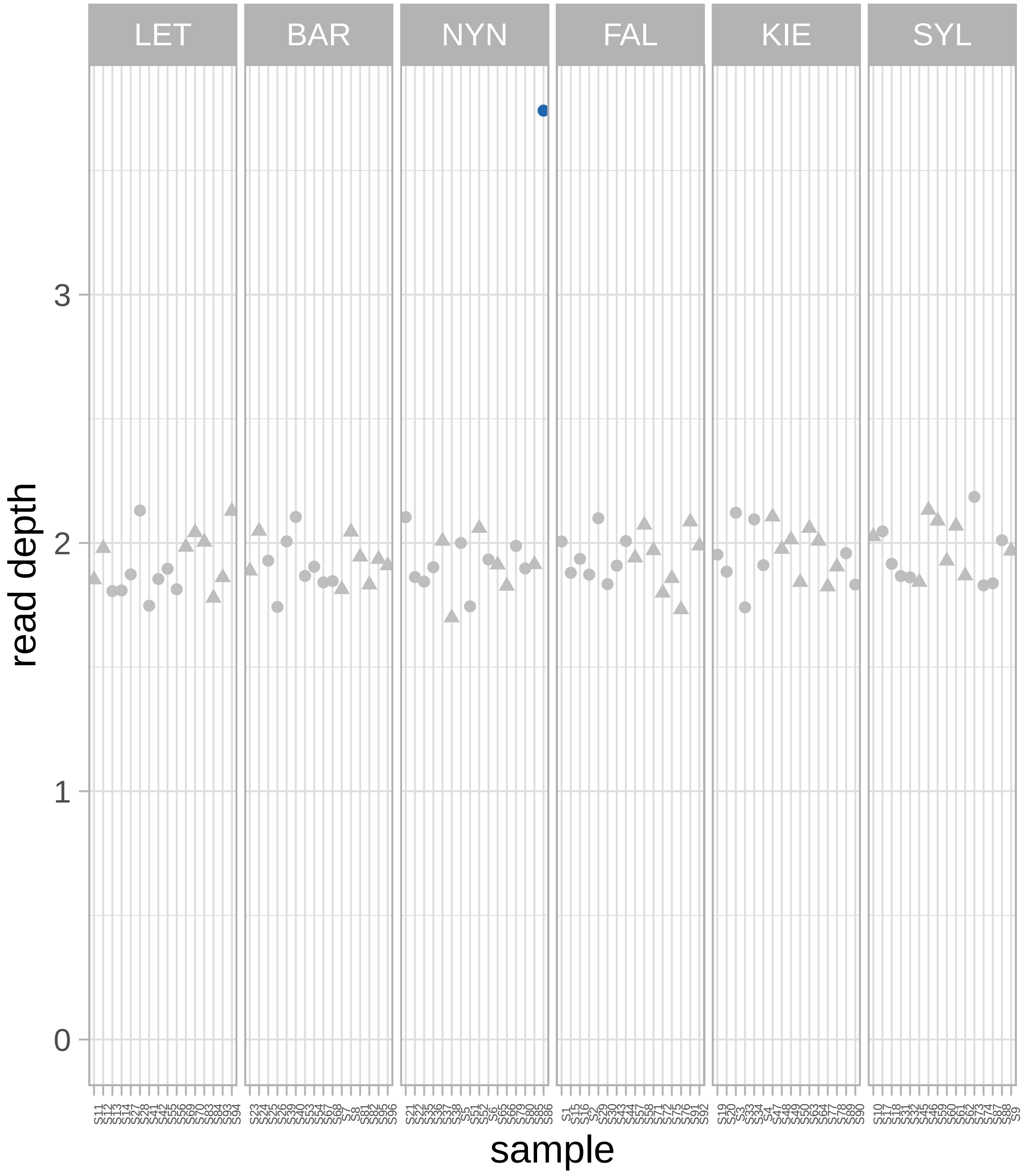

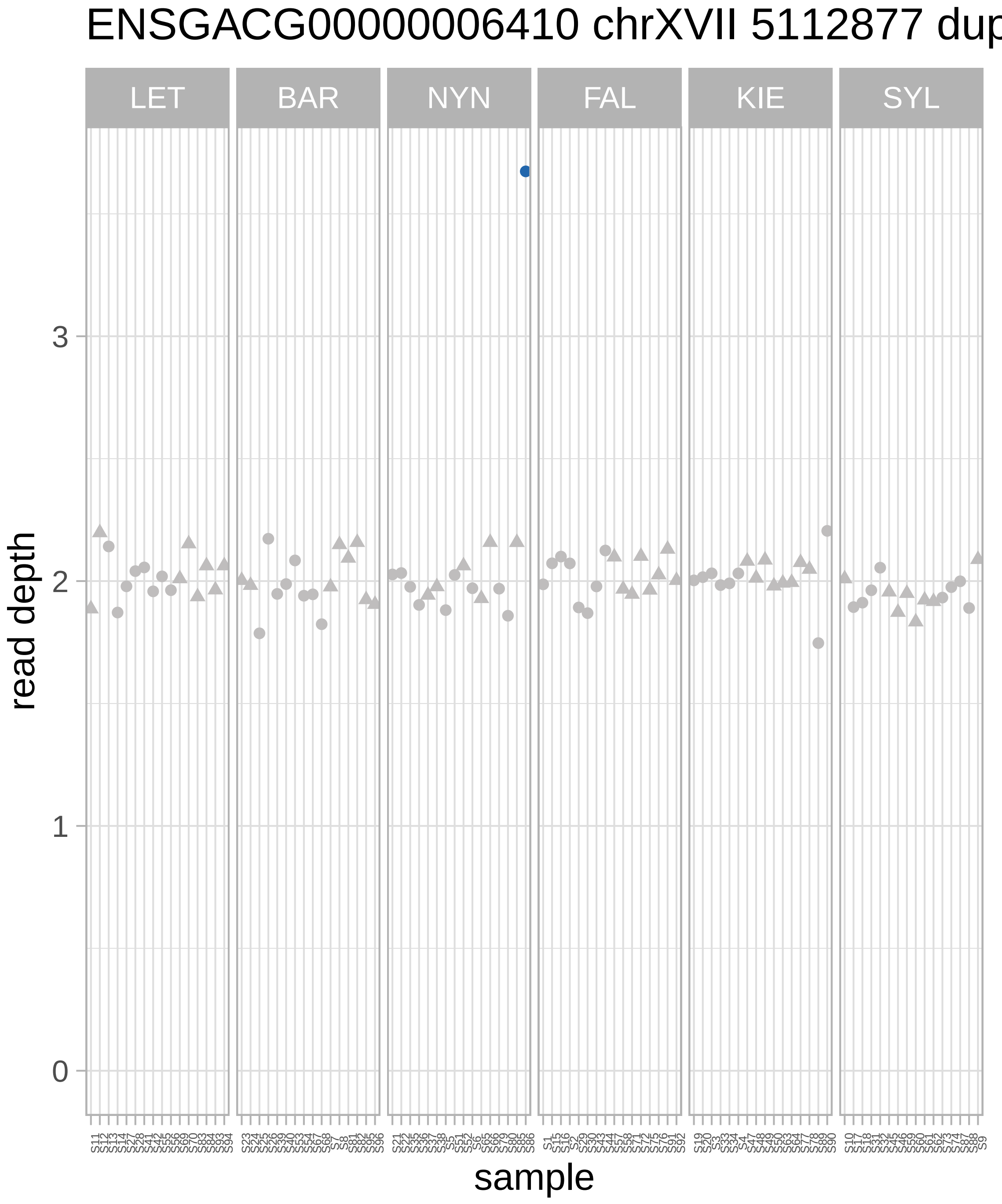
ENSGACG00000006410 chrXVII 5112877 dup
LET
BAR
NYN
FAL
KIE
SYL
read depth
0
1
2
3
sample

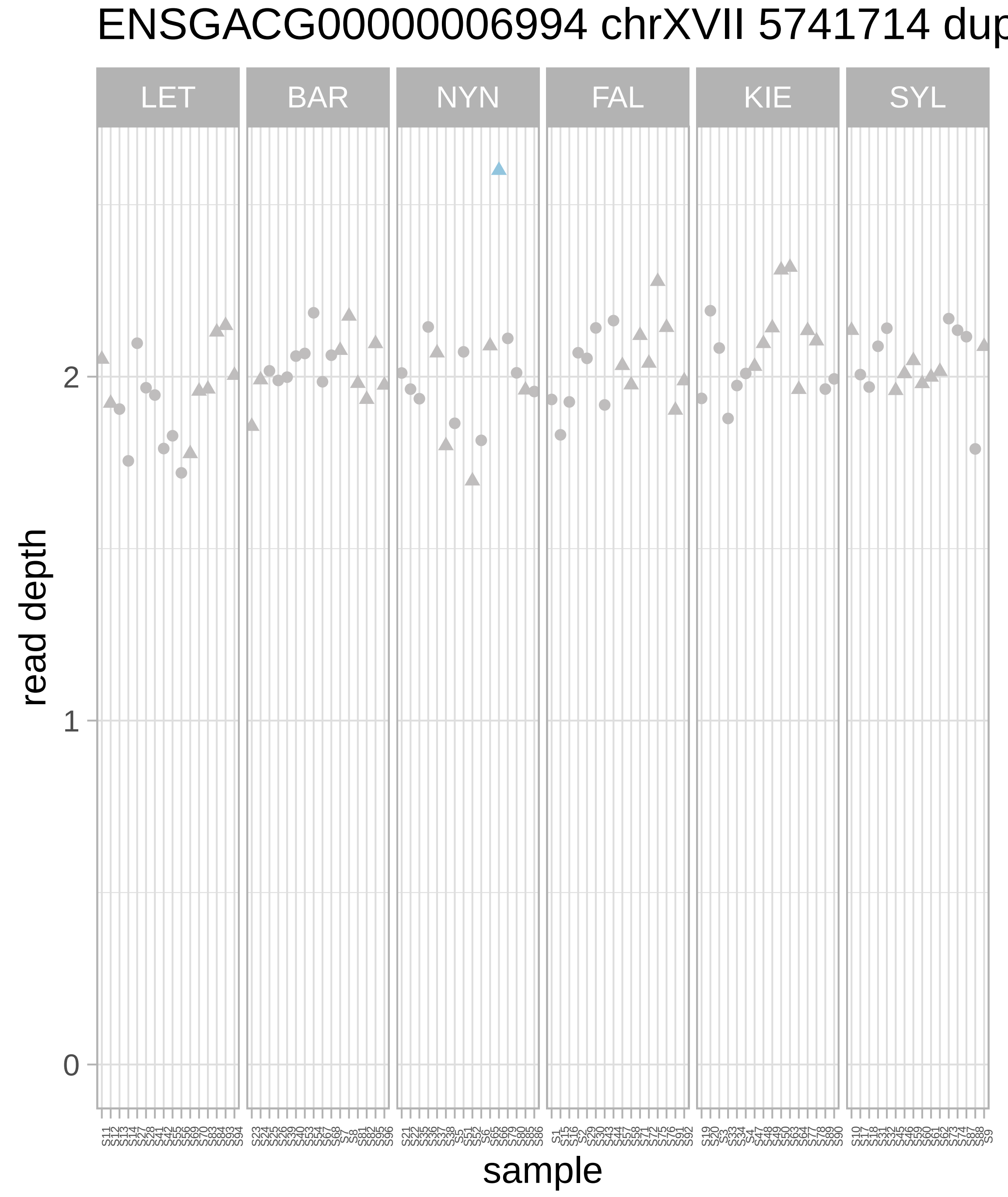

ENSGACG00000006994 chrXVII 5741714 dup
LET
BAR
NYN
FAL
KIE
SYL
read depth
0
1
2
sample

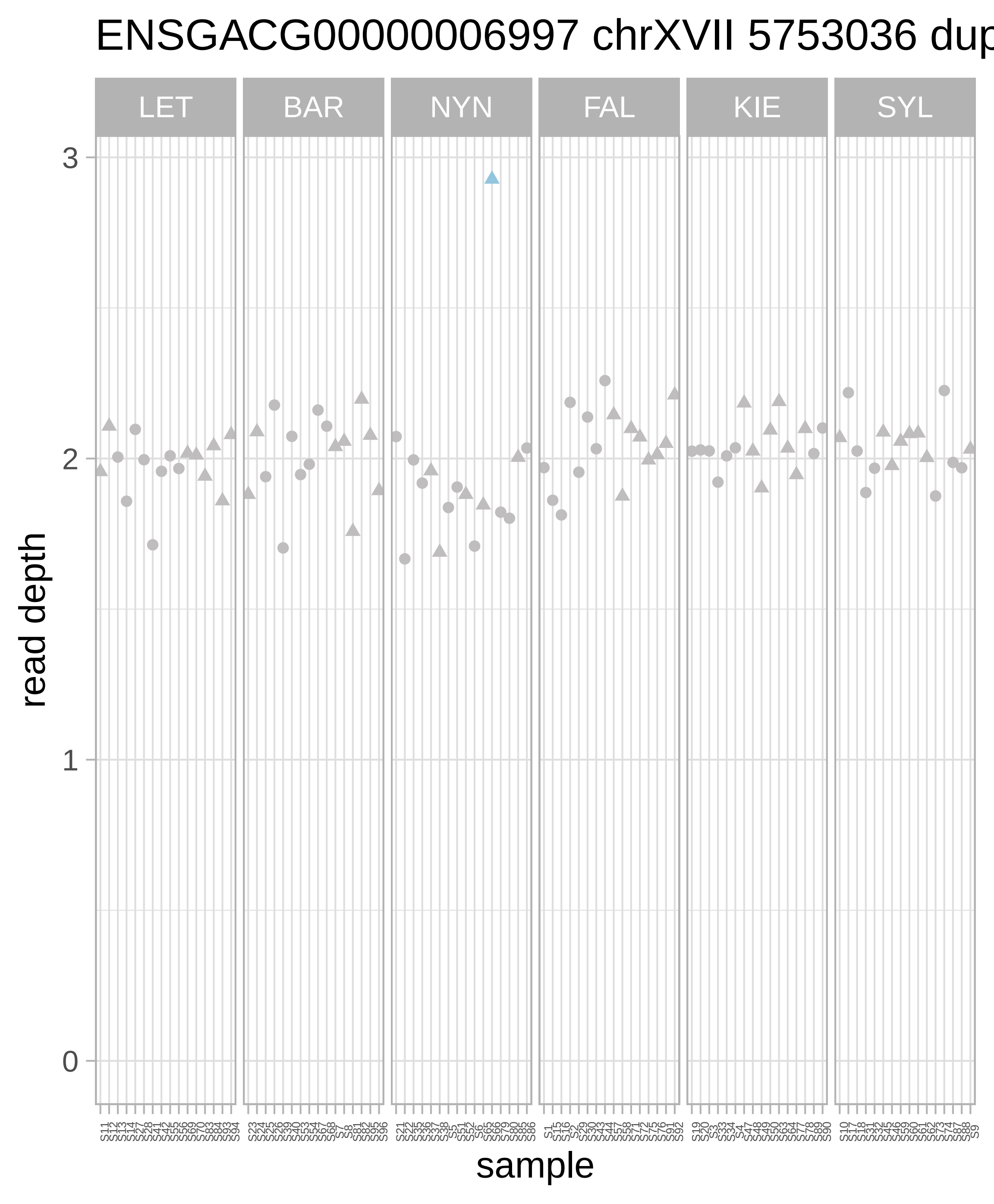
ENSGACG00000006997 chrXVII 5753036 dup
LET
BAR
NYN
FAL
KIE
SYL
3
2
1
0
read depth
sample

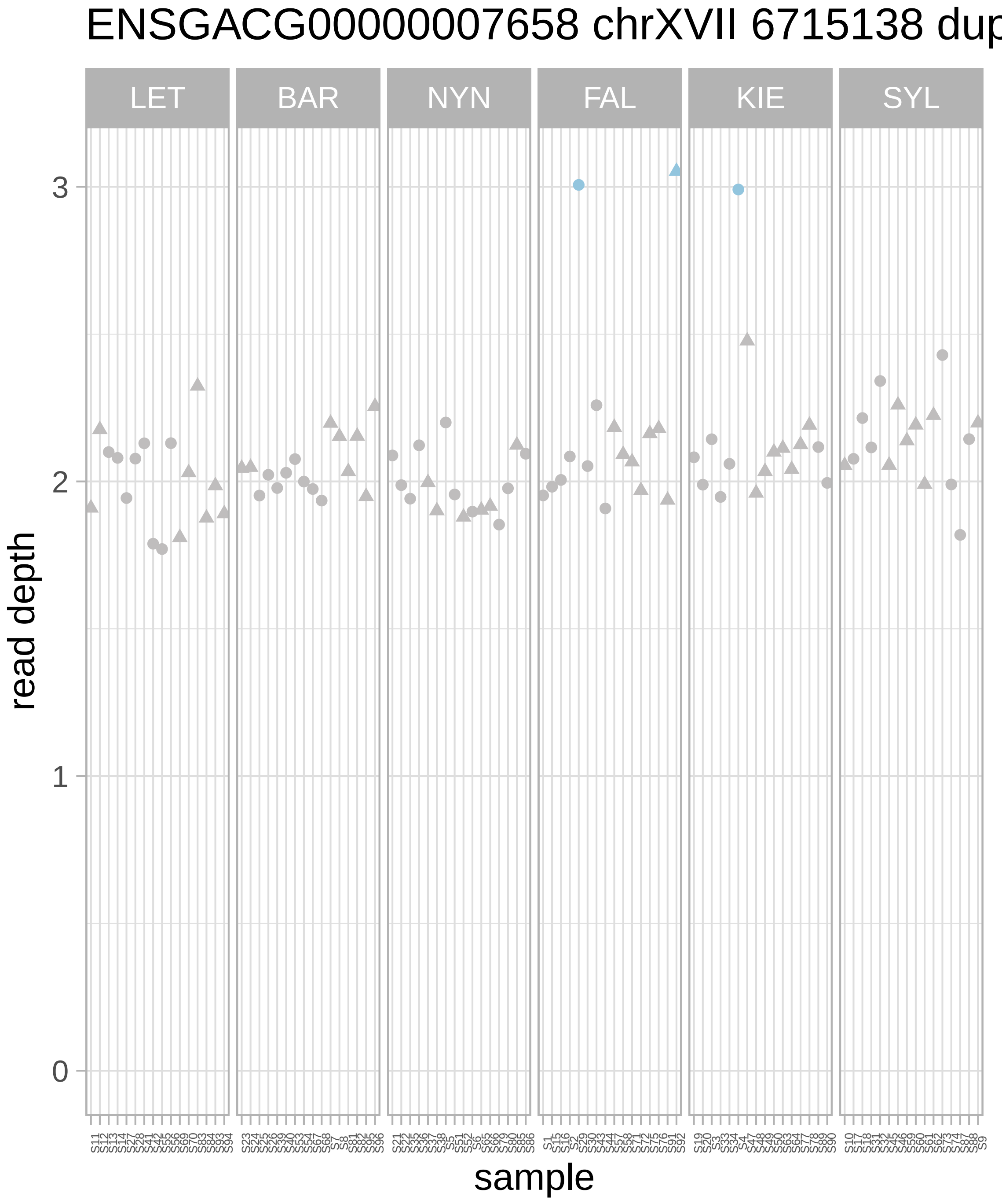
ENSGACG00000007658 chrXVII 6715138 dup
LET
BAR
NYN
FAL
KIE
SYL
read depth
0
1
2
3
S11 S12 S13 S14 S27 S28 S41 S42 S55 S56 S69 S70 S83 S84 S93 S94
S23 S24 S25 S26 S39 S40 S53 S54 S67 S68 S7 S8 S81 S82 S95 S96
S21 S22 S35 S36 S37 S38 S5 S51 S52 S6 S65 S66 S79 S80 S85 S86
S1 S15 S16 S2 S29 S30 S43 S44 S57 S58 S71 S72 S75 S76 S91 S92
S19 S20 S3 S33 S34 S4 S47 S48 S49 S50 S63 S64 S77 S78 S89 S90
S10 S17 S18 S31 S32 S45 S46 S59 S60 S61 S62 S73 S74 S87 S88 S9
sample

ENSGACG00000007667 chrXVII 6721536 dup

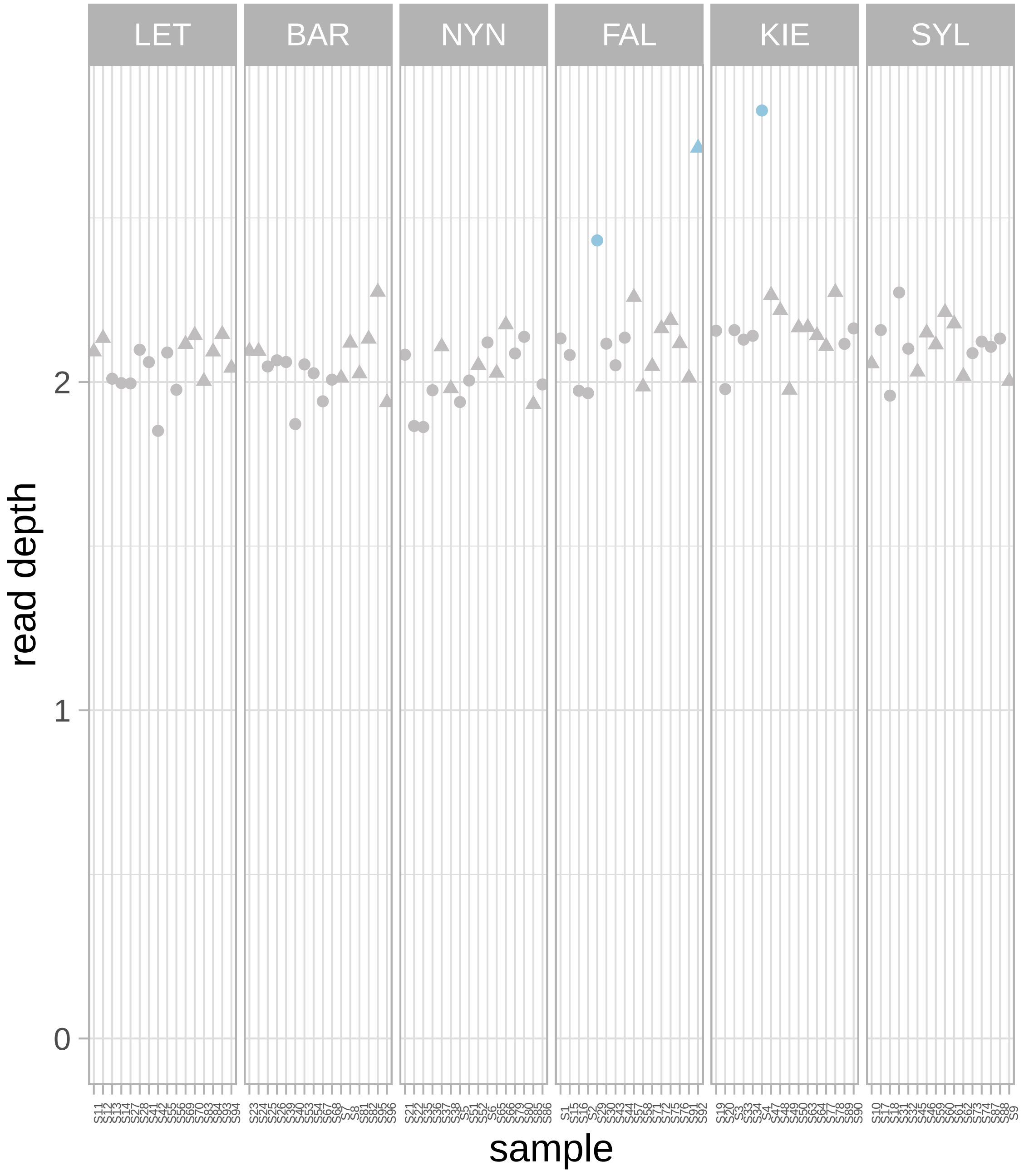

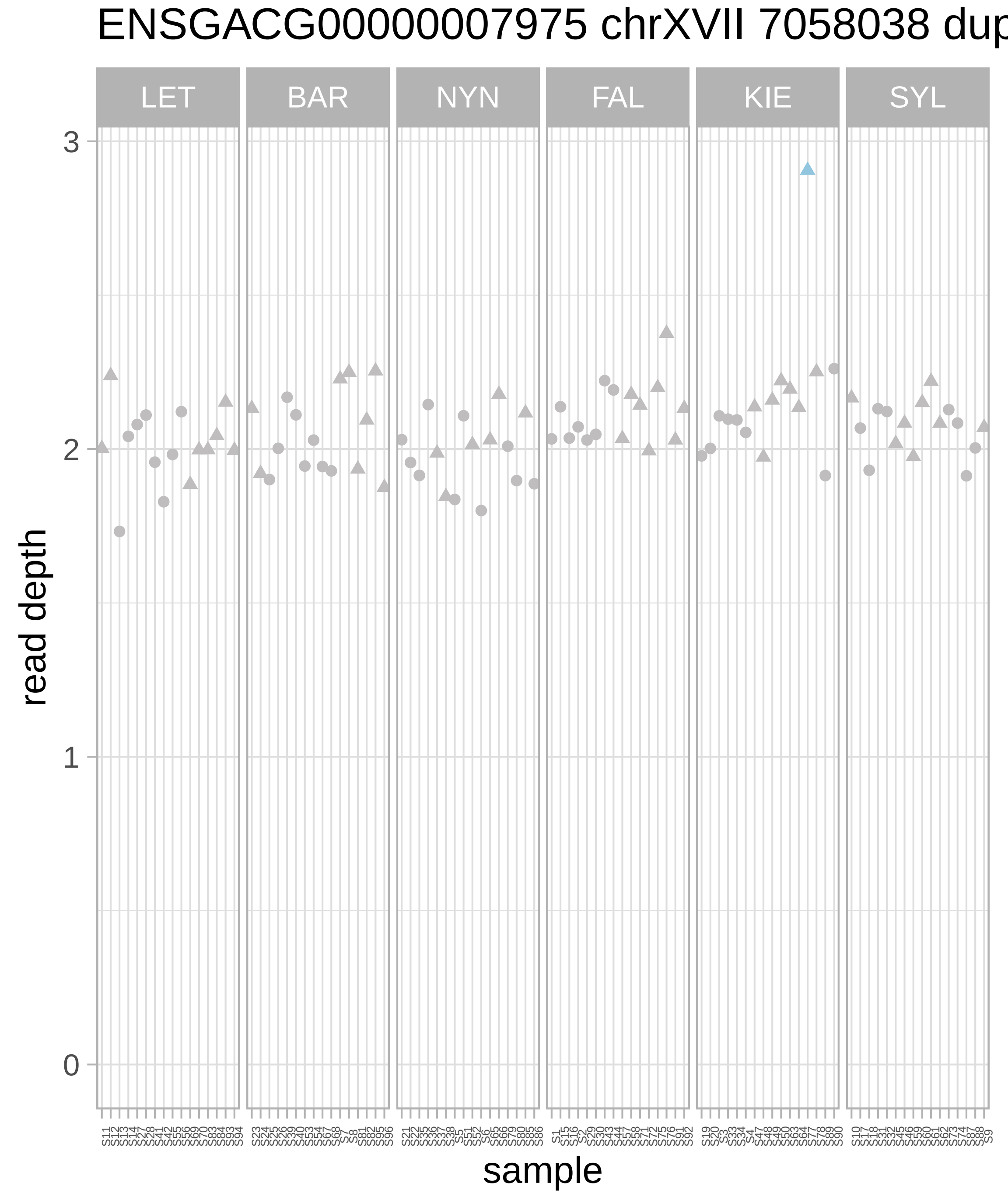
ENSGACG00000007975 chrXVII 7058038 dup
LET
BAR
NYN
FAL
KIE
SYL
read depth
0
1
2
3
S11 S12 S13 S14 S27 S28 S41 S42 S55 S56 S69 S70 S83 S84 S93 S94
S23 S24 S25 S26 S39 S40 S53 S54 S67 S68 S7 S8 S81 S82 S95 S96
S21 S22 S35 S36 S37 S38 S5 S51 S52 S6 S65 S66 S79 S80 S85 S86
S1 S15 S16 S2 S29 S30 S43 S44 S57 S58 S71 S72 S75 S76 S91 S92
S19 S20 S3 S33 S34 S4 S47 S48 S49 S50 S63 S64 S77 S78 S89 S90
S10 S17 S18 S31 S32 S45 S46 S59 S60 S61 S62 S73 S74 S87 S88 S9
sample

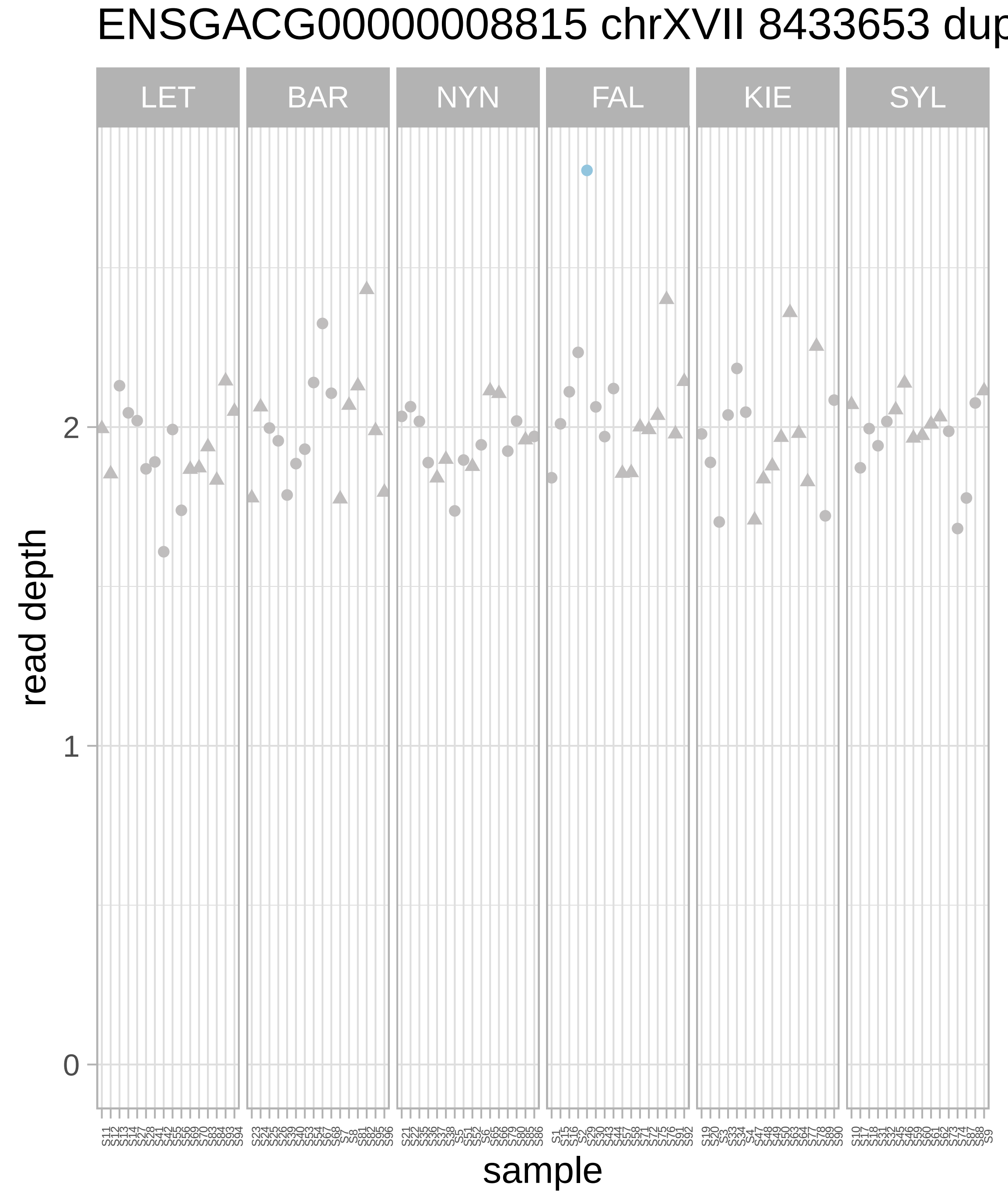
ENSGACG00000008815 chrXVII 8433653 dup
LET
BAR
NYN
FAL
KIE
SYL
read depth
0
1
2
sample

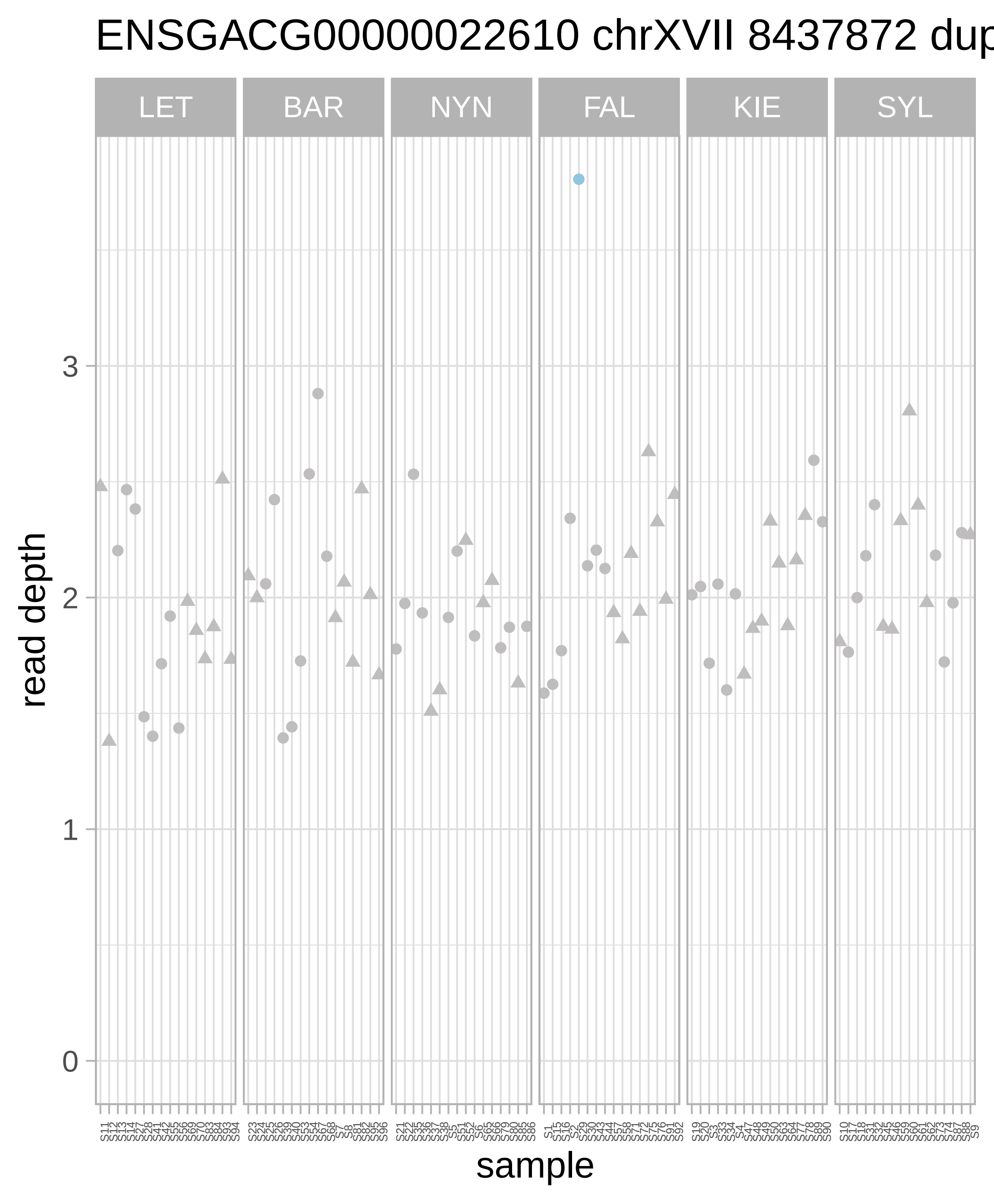
ENSGACG00000022610 chrXVII 8437872 dup
LET
BAR
NYN
FAL
KIE
SYL
read depth
3
2
1
0
S11 S12 S13 S14 S27 S28 S41 S42 S55 S56 S69 S70 S83 S84 S93 S94
S23 S24 S25 S26 S39 S40 S53 S54 S67 S68 S7 S8 S81 S82 S95 S96
S21 S22 S35 S36 S37 S38 S5 S51 S52 S6 S65 S66 S79 S80 S85 S86
S1 S15 S16 S2 S29 S30 S43 S44 S57 S58 S71 S72 S75 S76 S91 S92
S19 S20 S3 S33 S34 S4 S47 S48 S49 S50 S63 S64 S77 S78 S89 S90
S10 S17 S18 S31 S32 S45 S46 S59 S60 S61 S62 S73 S74 S87 S88 S9
sample

ENSGACG00000022710 chrXVII 8437595 dup

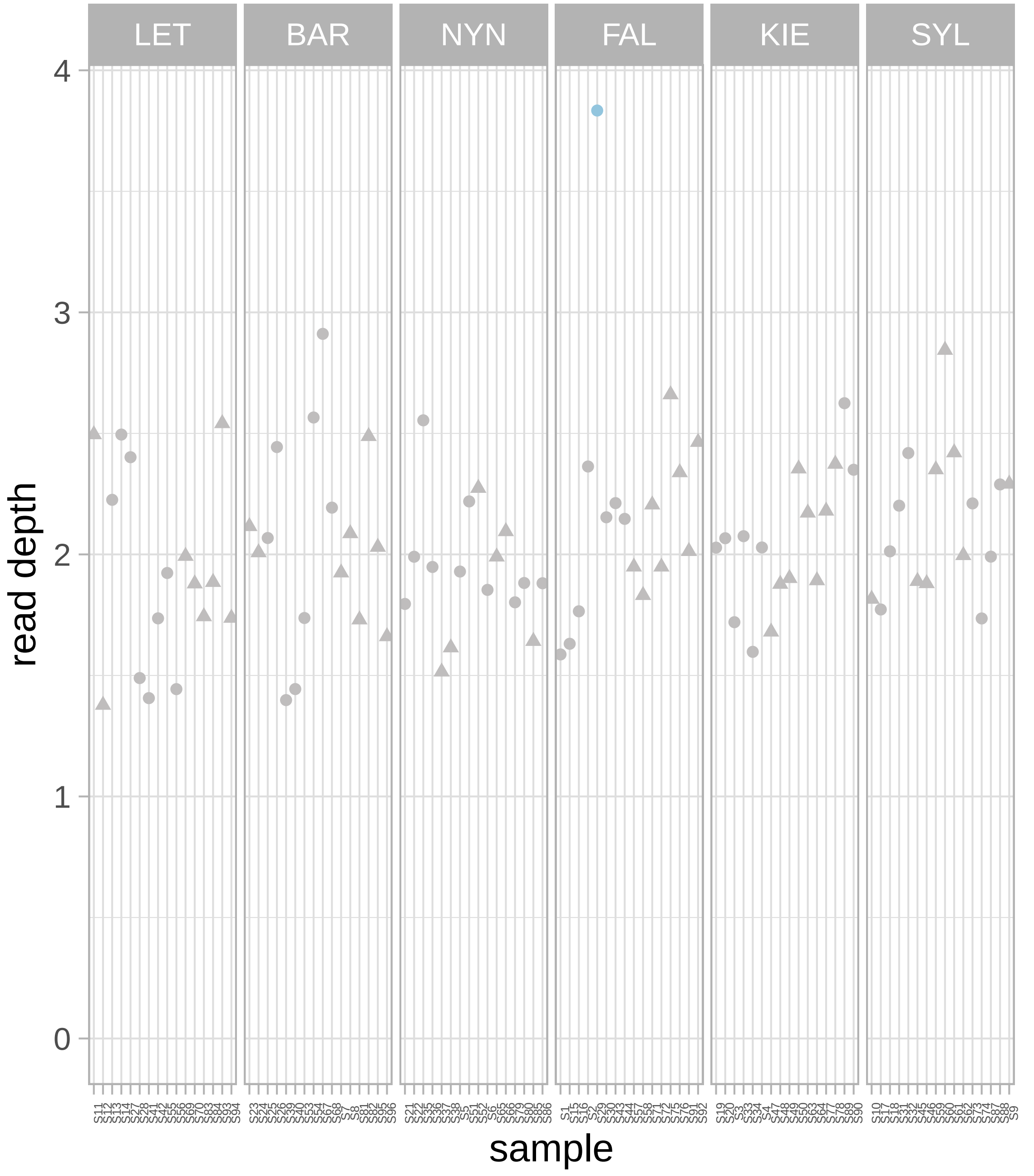

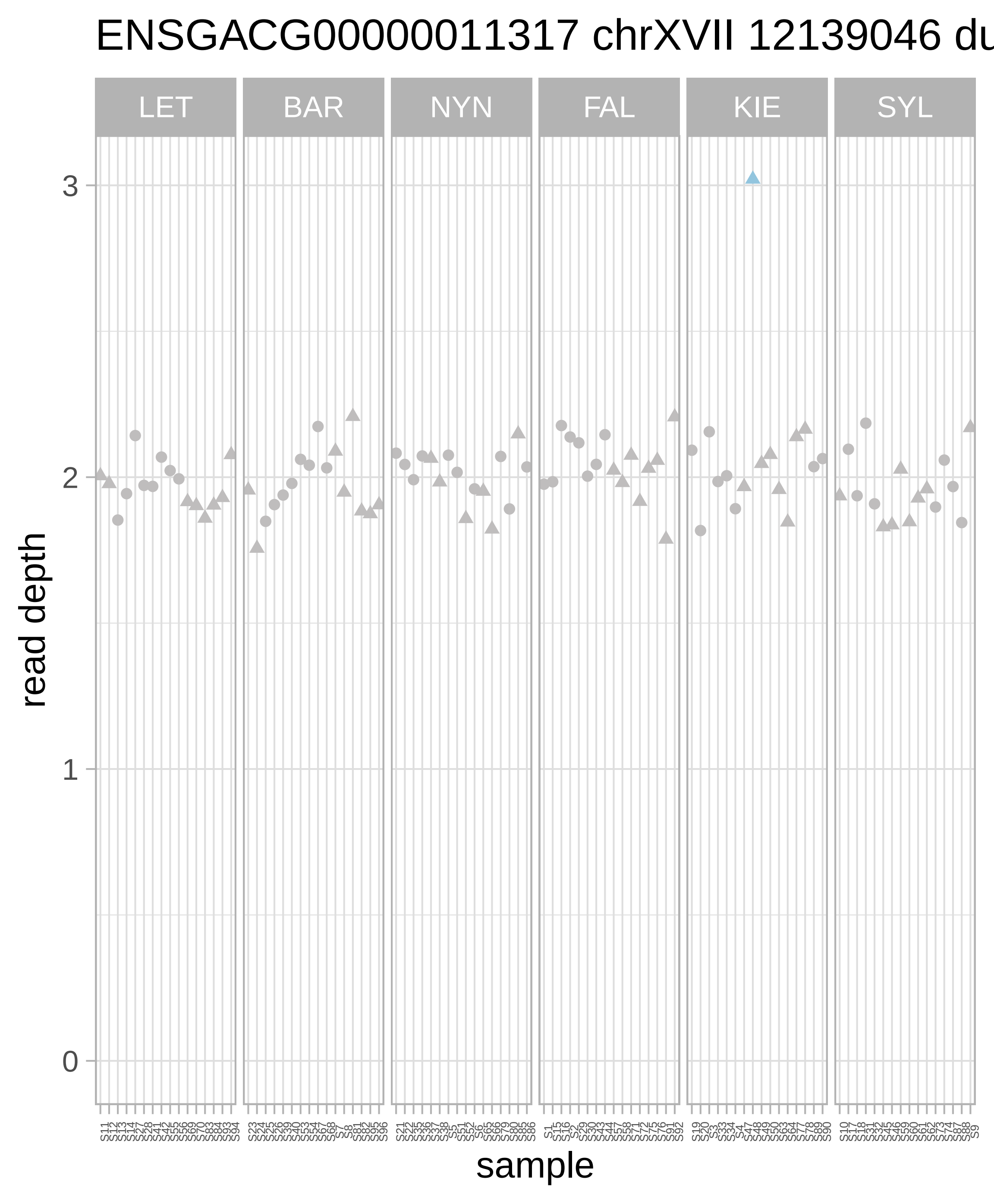
ENSGACG00000011317 chrXVII 12139046 du
LET
BAR
NYN
FAL
KIE
SYL
read depth
0
1
2
3
sample

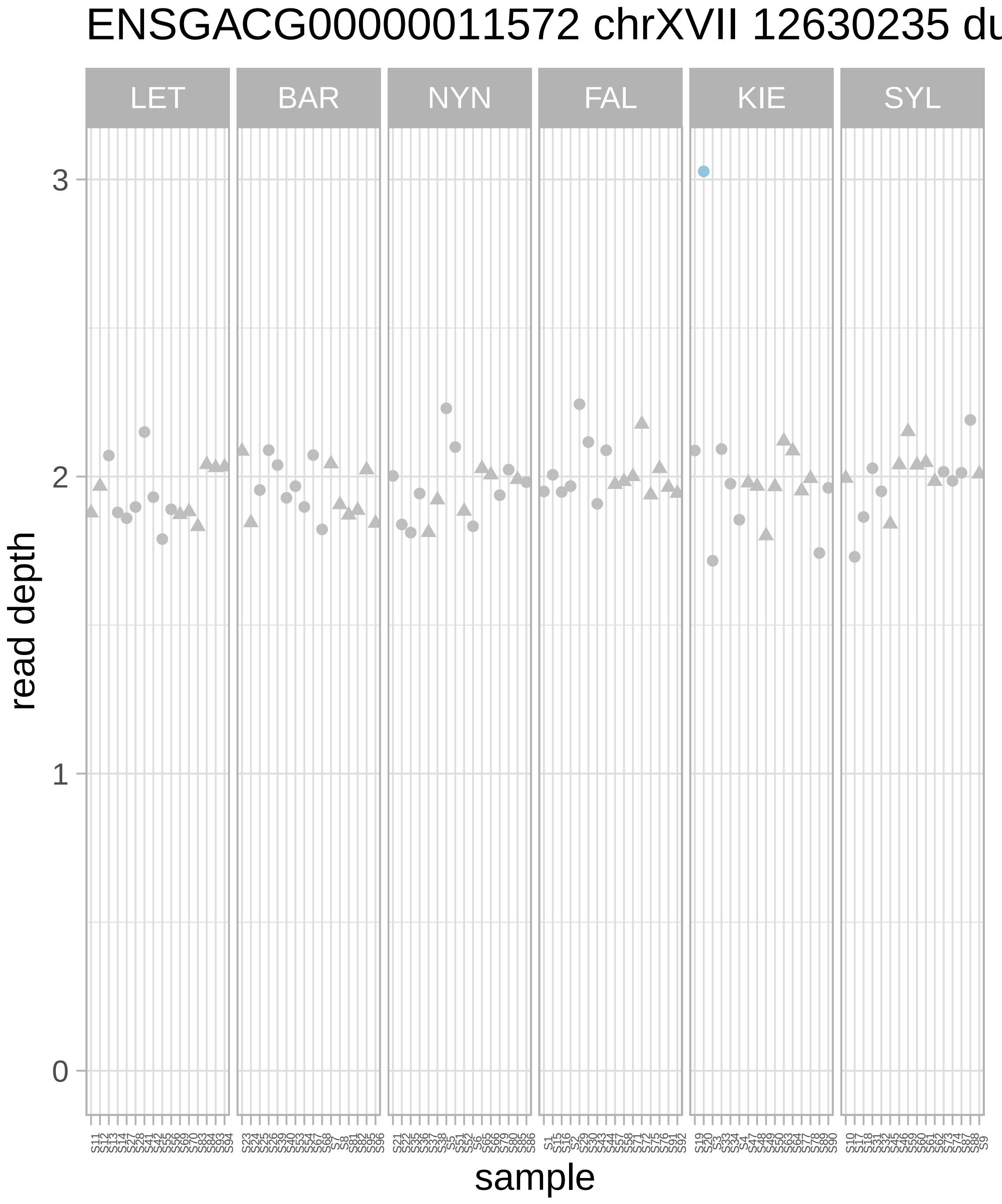
ENSGACG00000011572 chrXVII 12630235 du
LET
BAR
NYN
FAL
KIE
SYL
read depth
0
1
2
3
sample

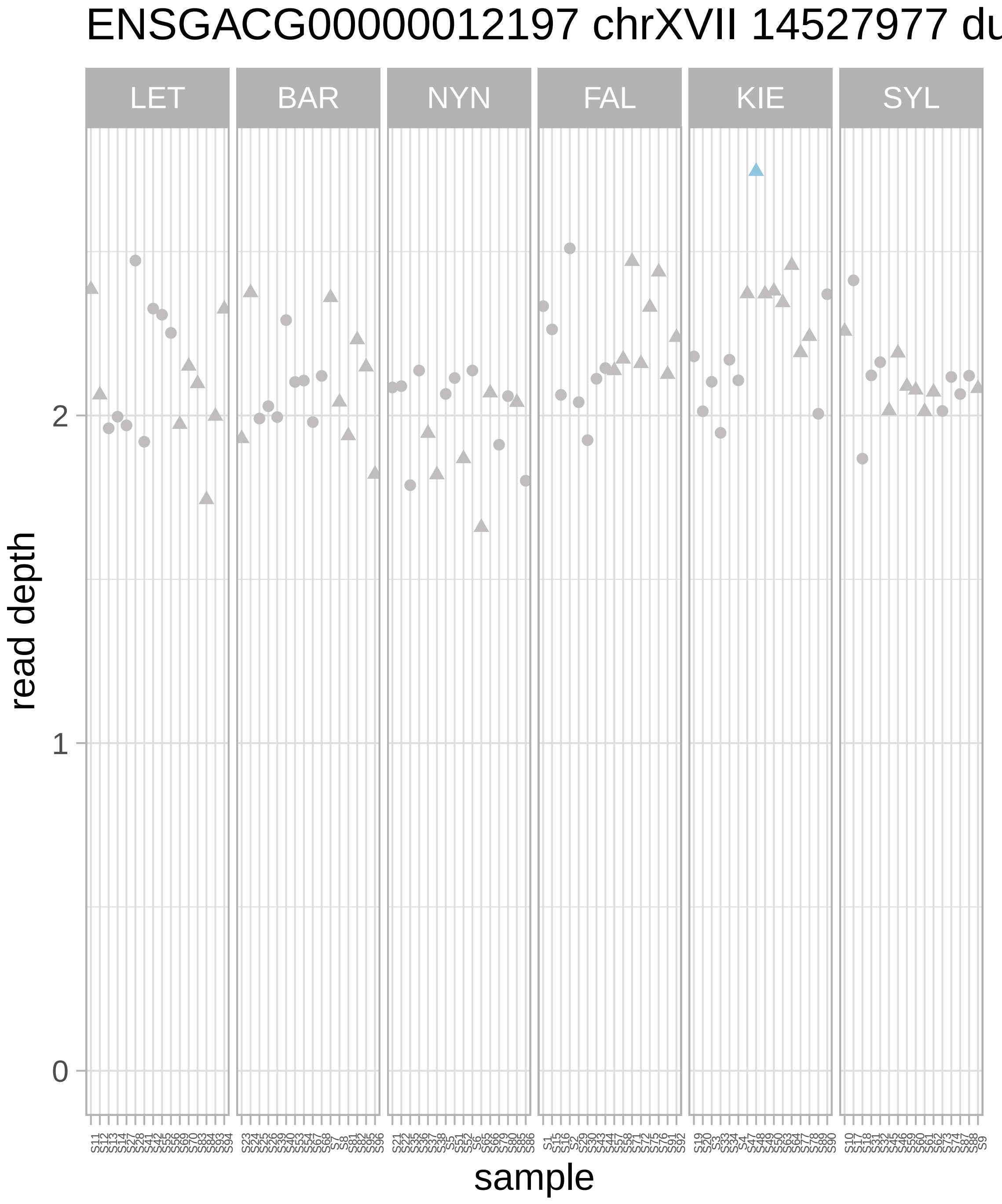
ENSGACG00000012197 chrXVII 14527977 du
LET
BAR
NYN
FAL
KIE
SYL
read depth
2
1
0
sample

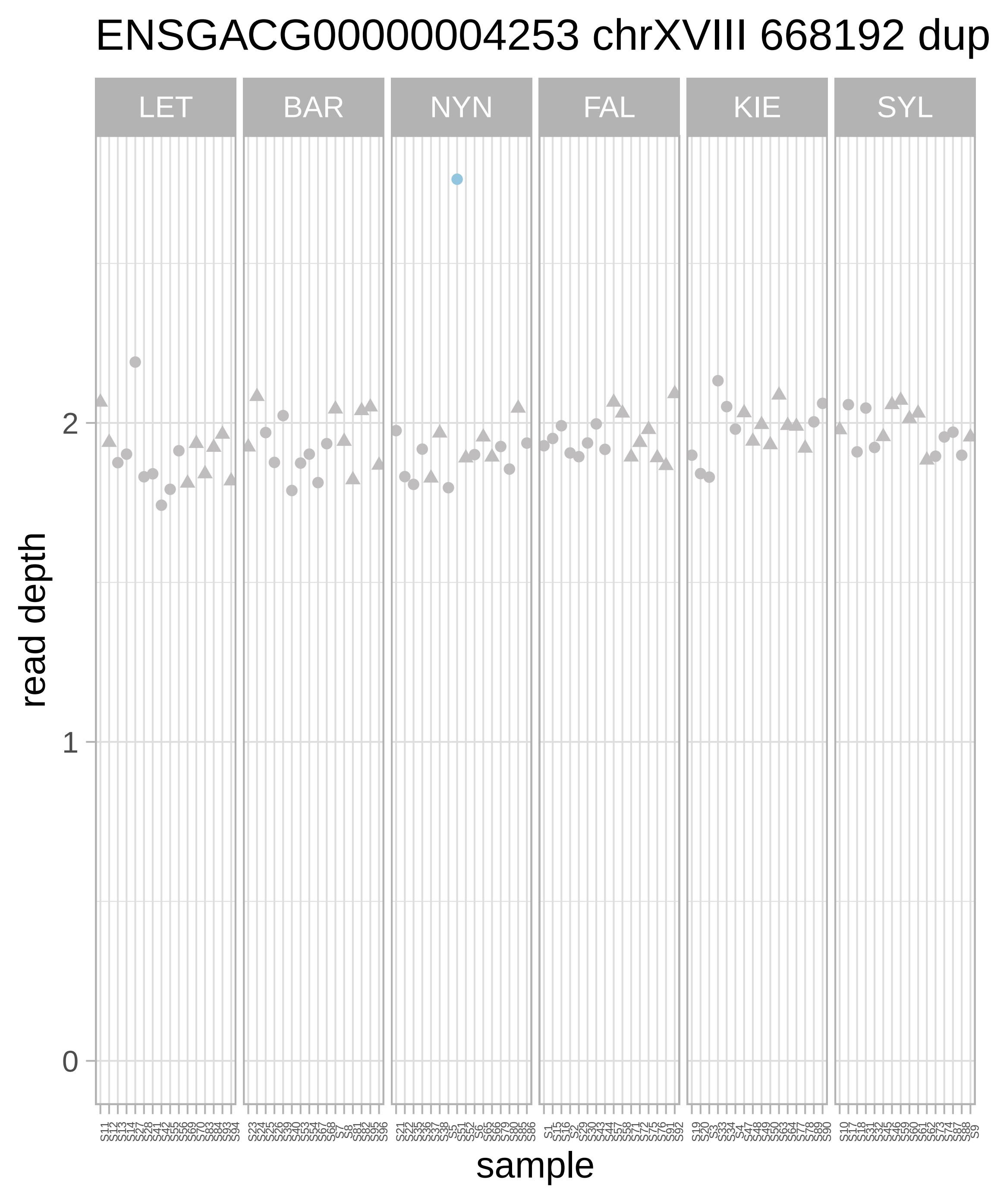
ENSGACG00000004253 chrXVIII 668192 dup
LET
BAR
NYN
FAL
KIE
SYL
read depth
0
1
2
sample

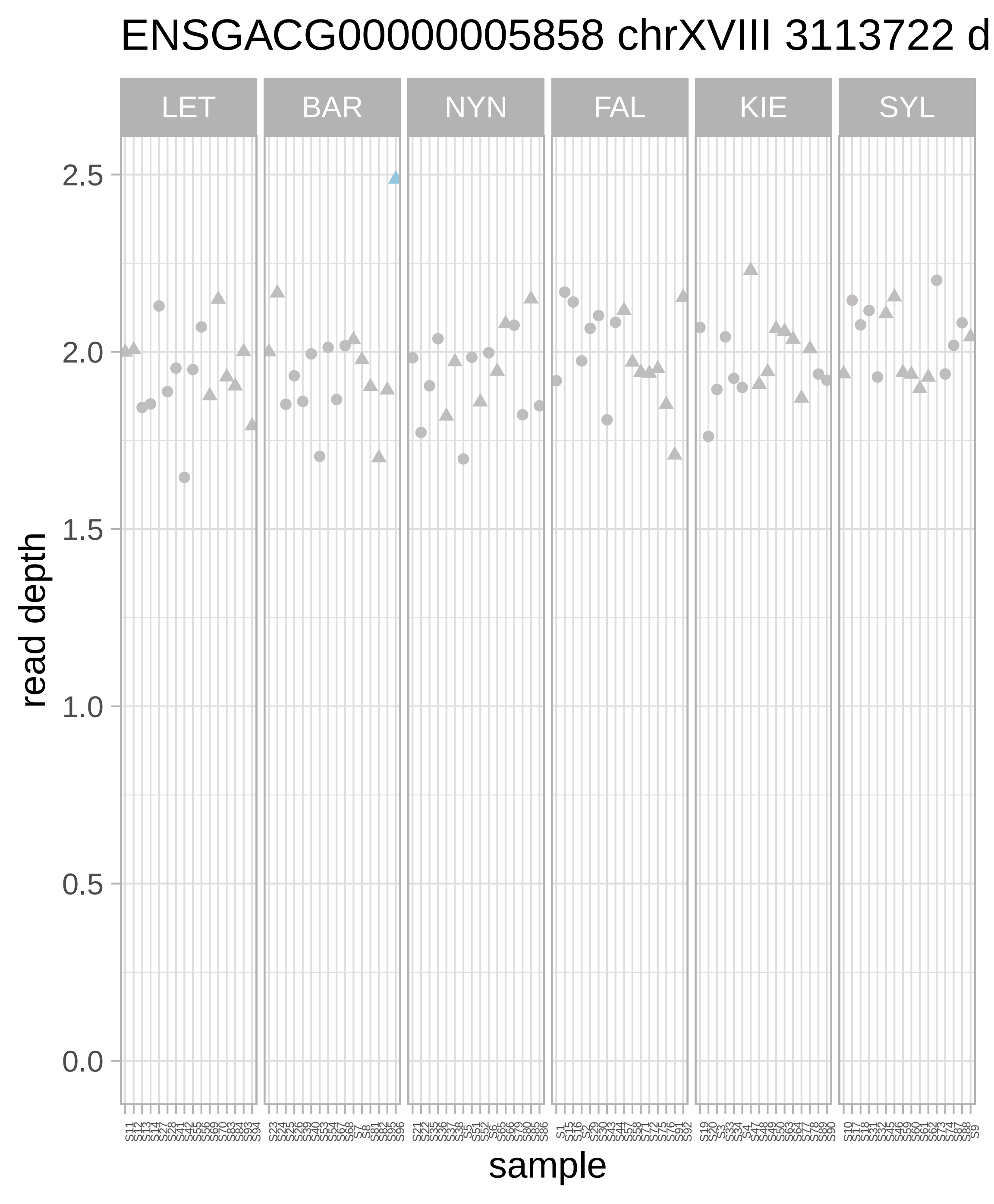
ENSGACG00000005858 chrXVIII 3113722 d
LET
BAR
NYN
FAL
KIE
SYL
read depth
sample
2.5
2.0
1.5
1.0
0.5
0.0

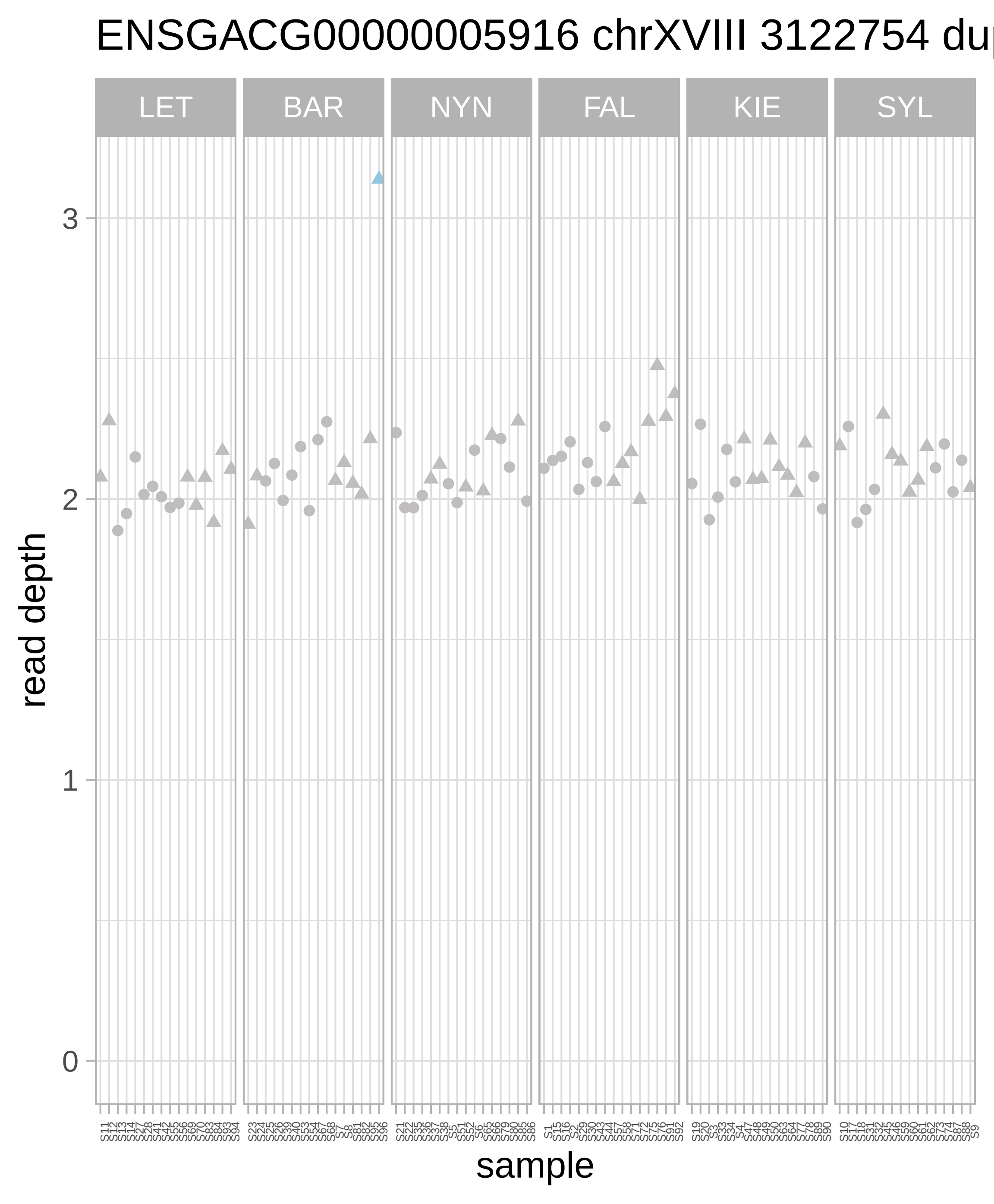

ENSGACG00000005916 chrXVIII 3122754 du
LET
BAR
NYN
FAL
KIE
SYL
read depth
0
1
2
3
sample

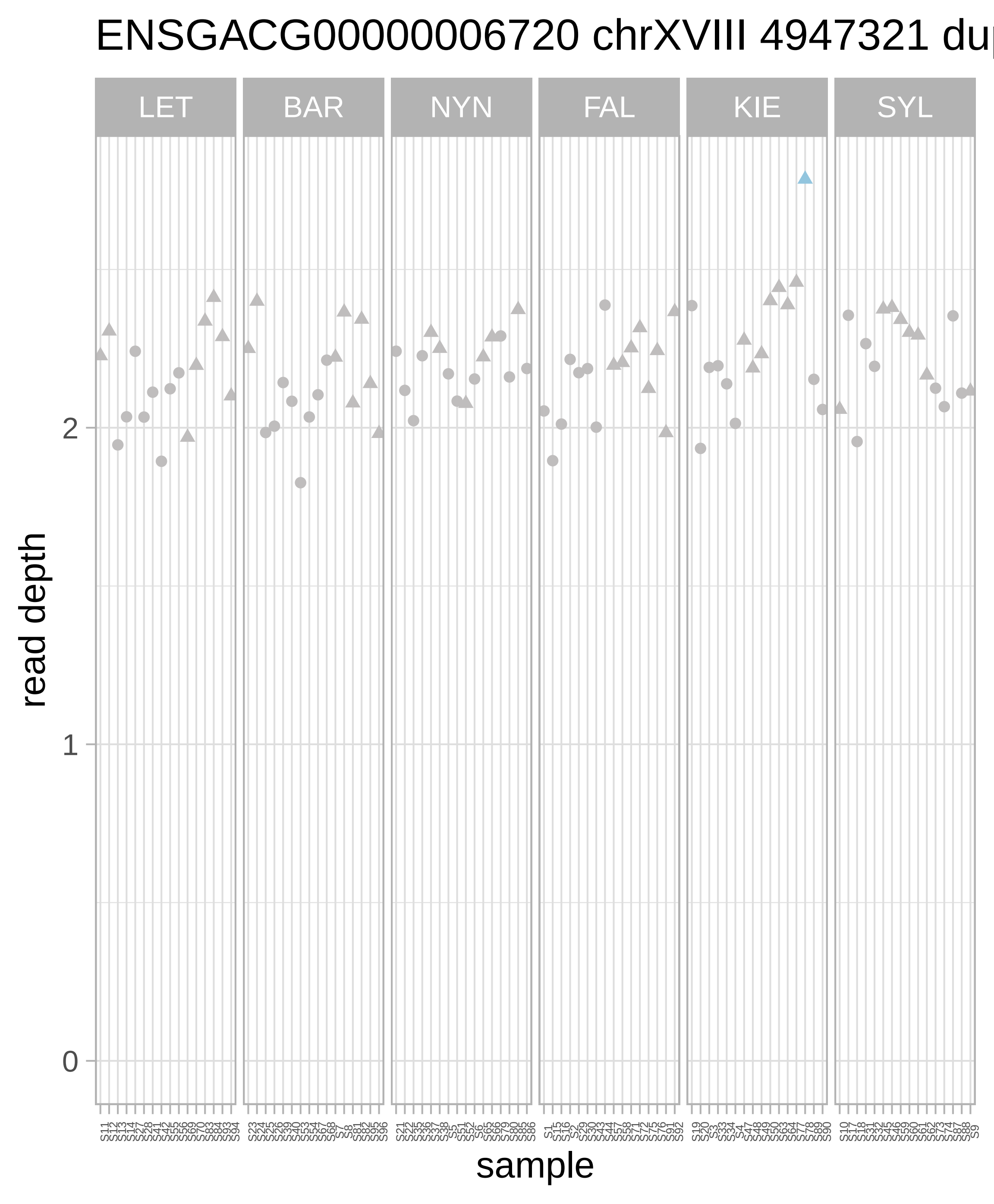
ENSGACG00000006720 chrXVIII 4947321 du
LET
BAR
NYN
FAL
KIE
SYL
read depth
2
1
0
sample

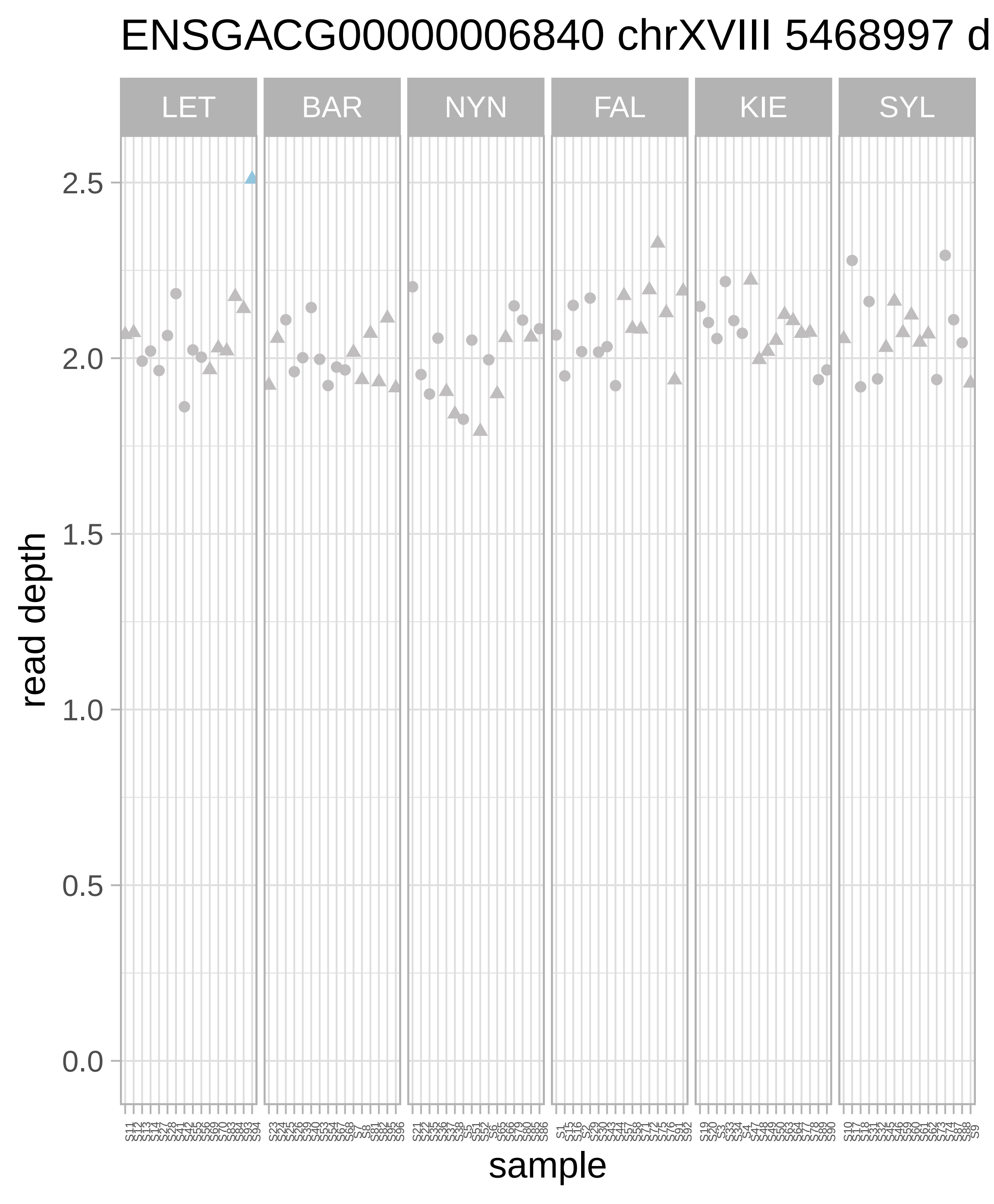
ENSGACG00000006840 chrXVIII 5468997 d
LET
BAR
NYN
FAL
KIE
SYL
read depth
2.5
2.0
1.5
1.0
0.5
0.0
sample

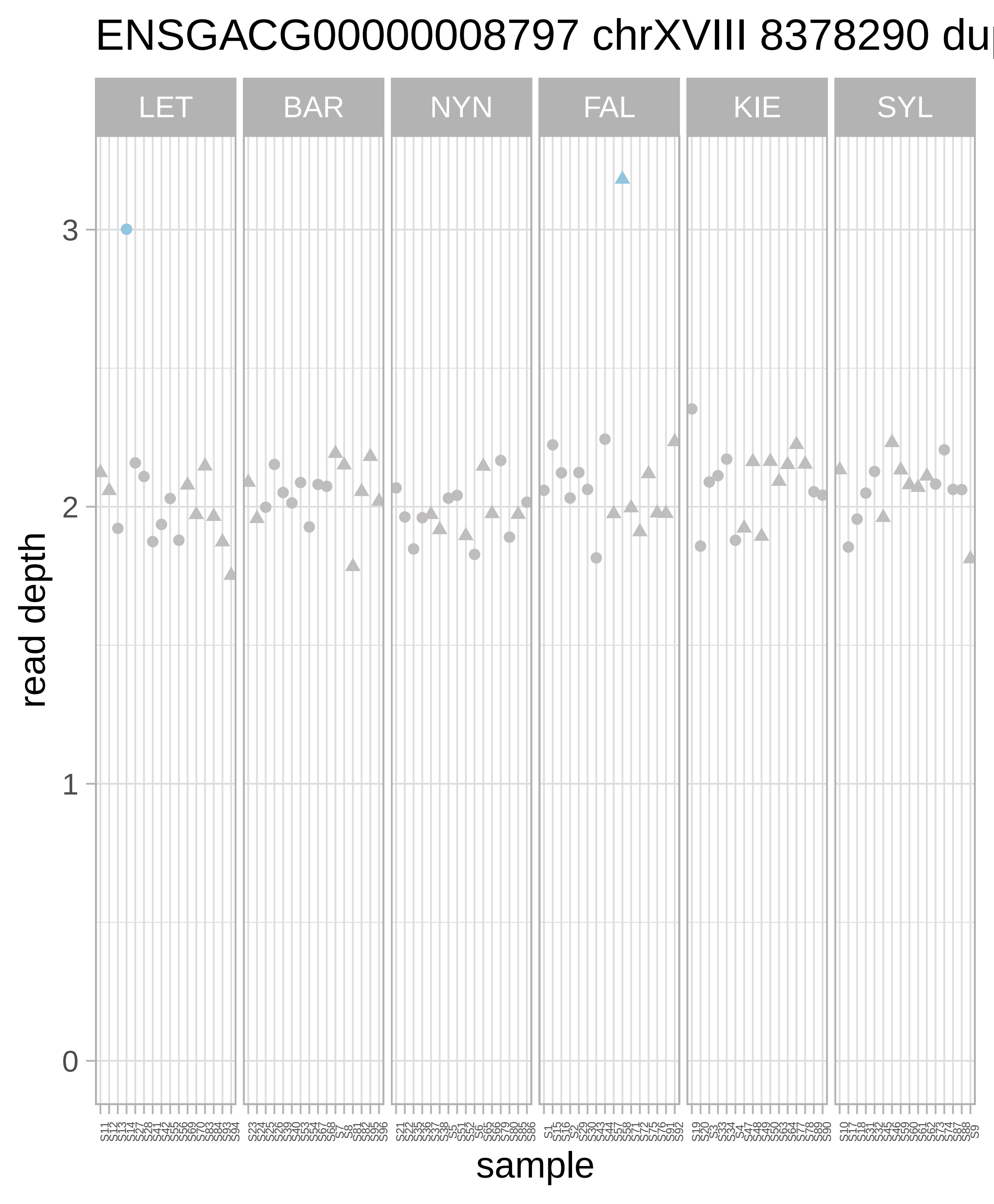
ENSGACG00000008797 chrXVIII 8378290 du
LET
BAR
NYN
FAL
KIE
SYL
read depth
0
1
2
3
sample

ENSGACG00000009500 chrXVIII 9208544 du[illegible]

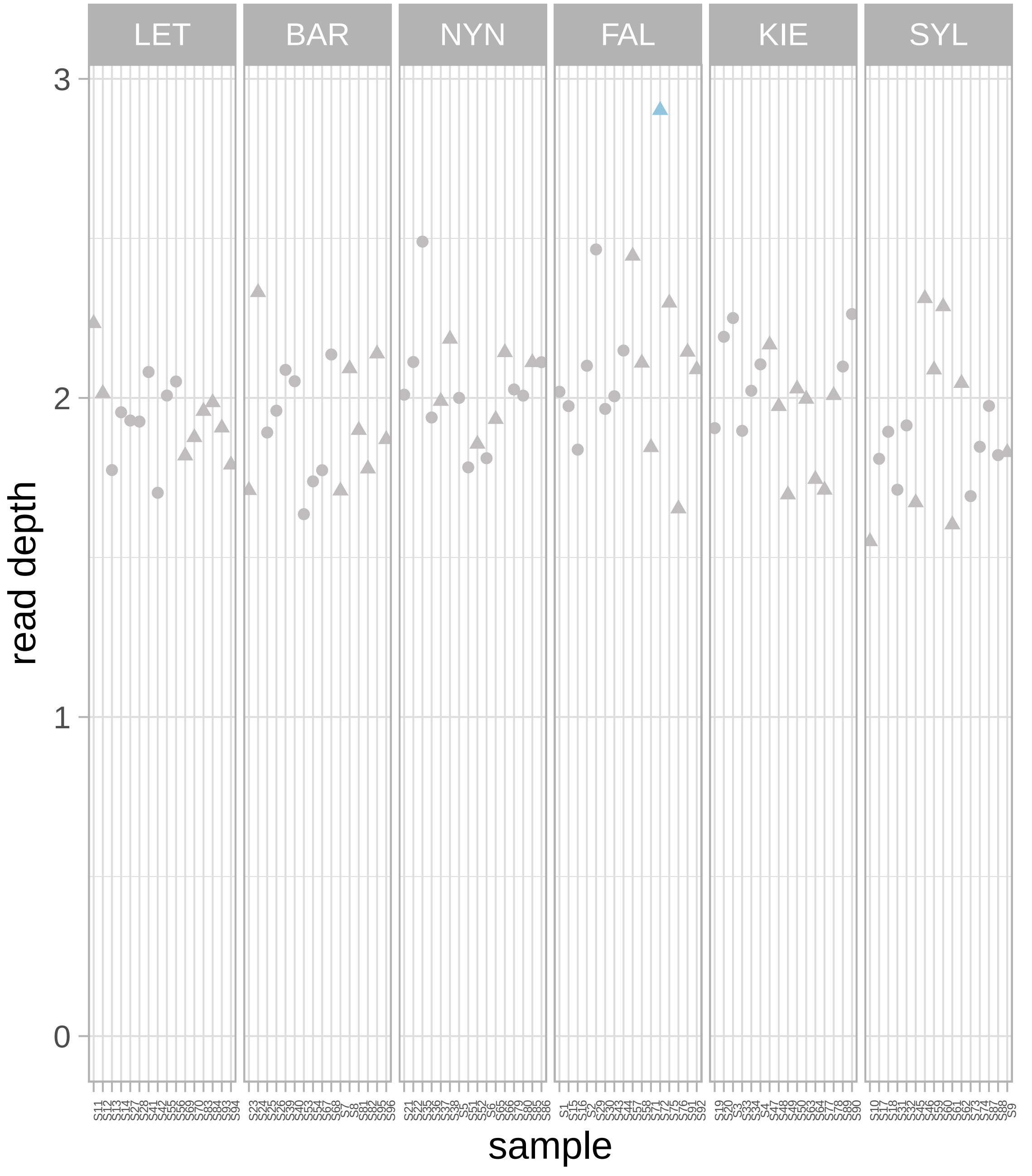

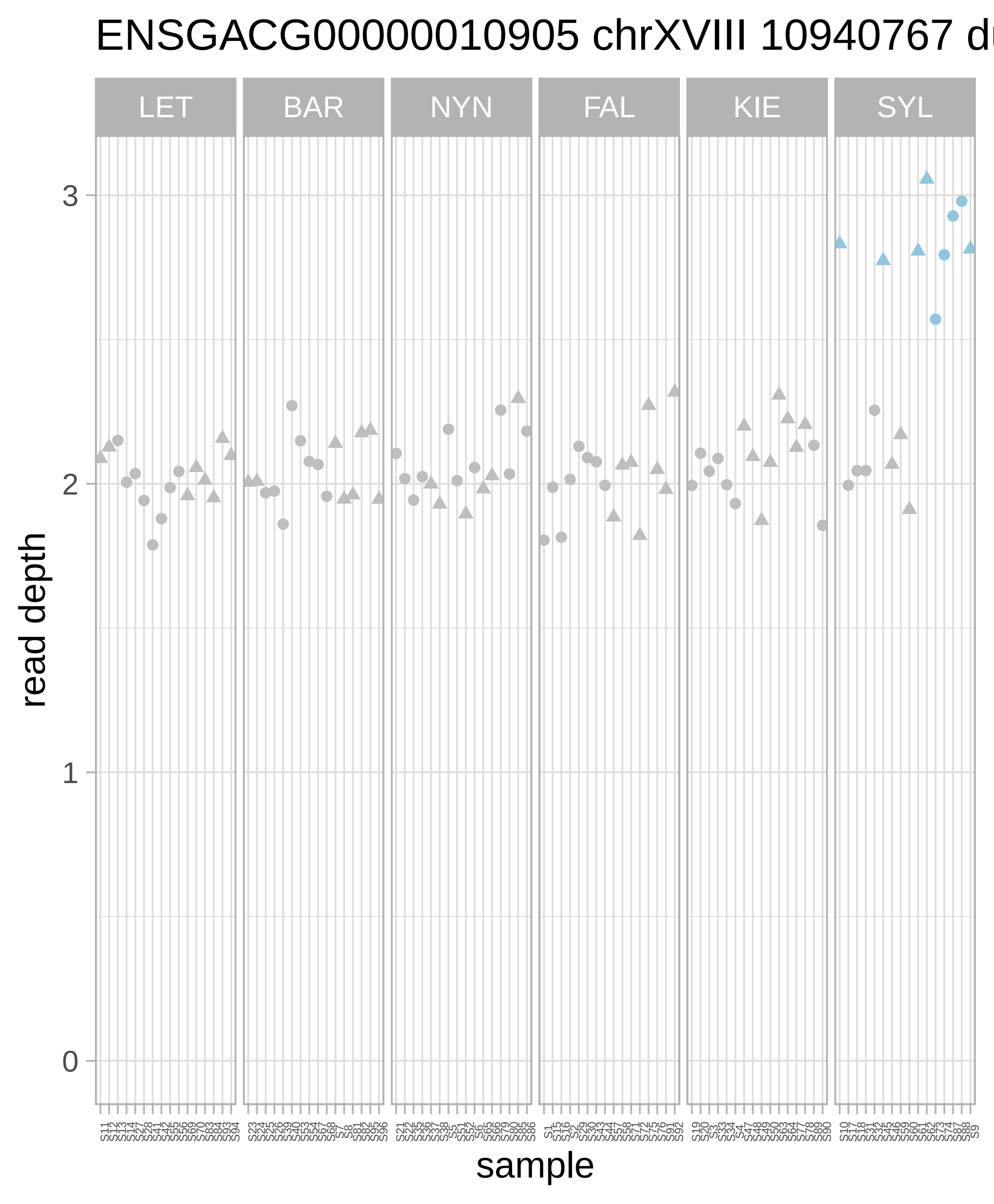
ENSGACG00000010905 chrXVIII 10940767 d
LET
BAR
NYN
FAL
KIE
SYL
3
2
1
0
read depth
sample

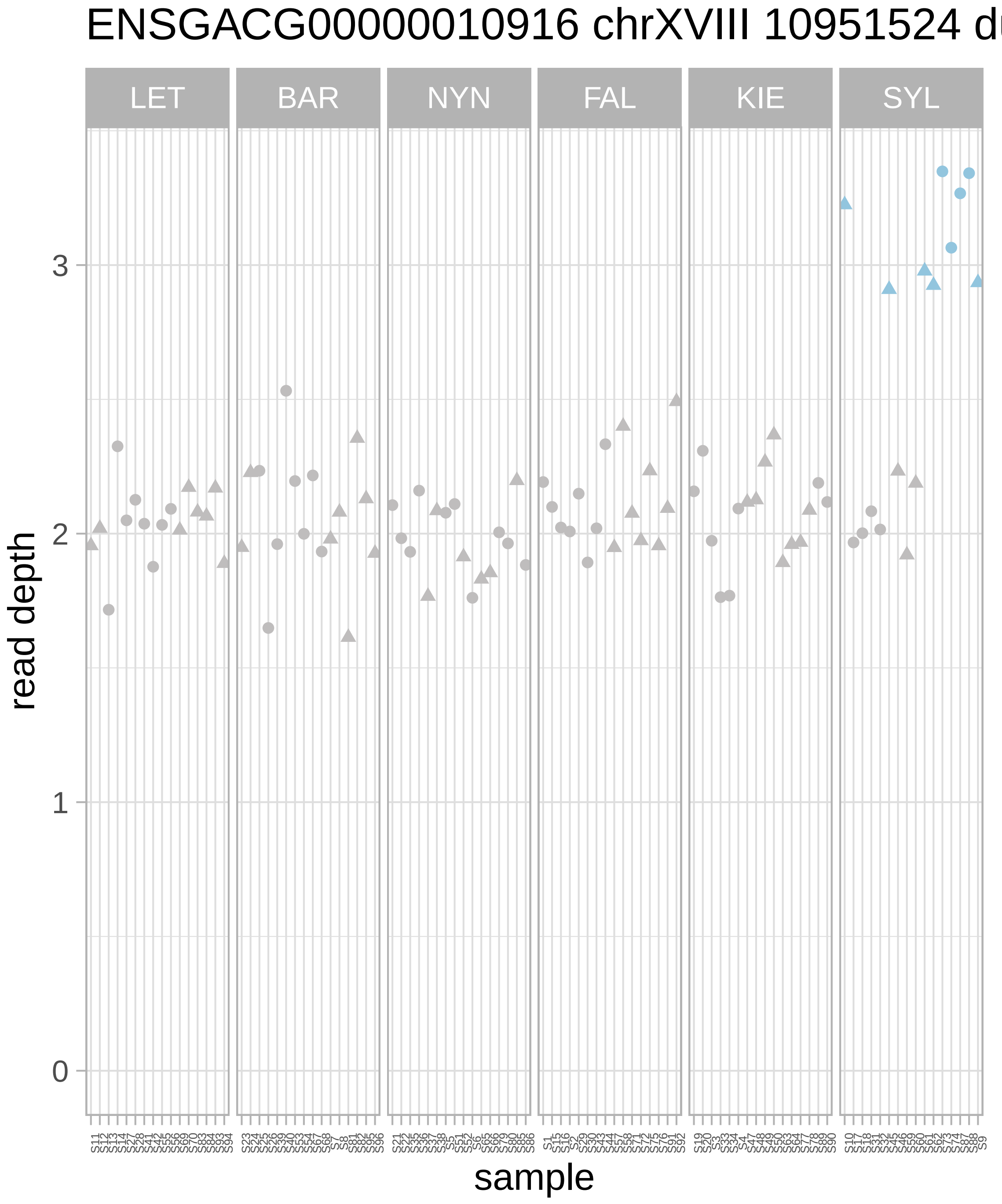
ENSGACG00000010916 chrXVIII 10951524 d
LET
BAR
NYN
FAL
KIE
SYL
read depth
0
1
2
3
sample

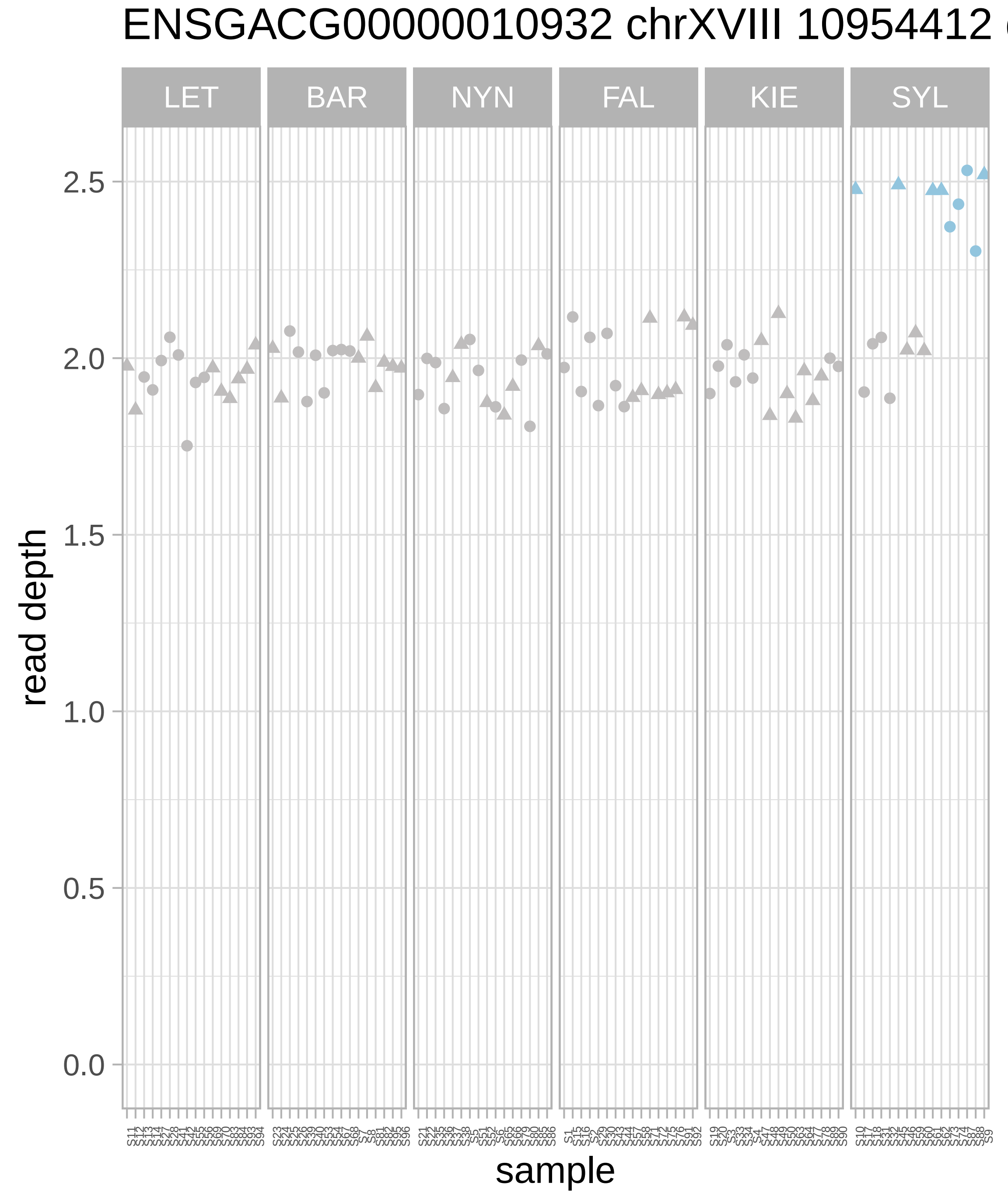
ENSGACG00000010932 chrXVIII 10954412
LET
BAR
NYN
FAL
KIE
SYL
read depth
2.5
2.0
1.5
1.0
0.5
0.0
S11 S12 S13 S14 S27 S28 S41 S42 S55 S56 S69 S70 S83 S84 S93 S94
S23 S24 S25 S26 S39 S40 S53 S54 S67 S68 S7 S8 S81 S82 S95 S96
S21 S22 S35 S36 S37 S38 S5 S51 S52 S6 S65 S66 S79 S80 S85 S86
S1 S15 S16 S2 S29 S30 S43 S44 S57 S58 S71 S72 S75 S76 S91 S92
S19 S20 S3 S33 S34 S4 S47 S48 S49 S50 S63 S64 S77 S78 S89 S90
S10 S17 S18 S31 S32 S45 S46 S59 S60 S61 S62 S73 S74 S87 S88 S9
sample

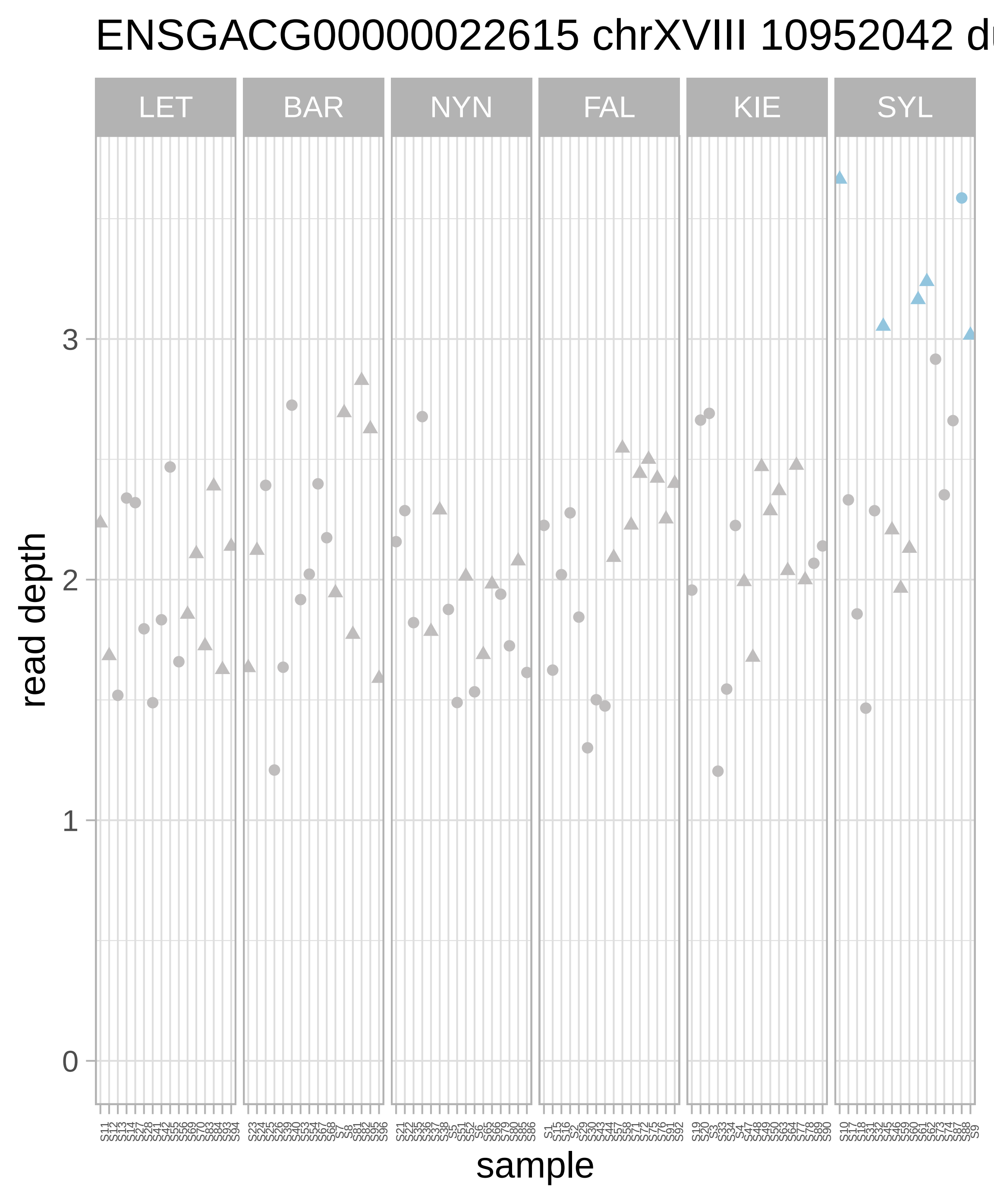
ENSGACG00000022615 chrXVIII 10952042 d
LET
BAR
NYN
FAL
KIE
SYL
read depth
3
2
1
0
S11 S12 S13 S14 S27 S28 S41 S42 S55 S56 S69 S70 S83 S84 S93 S94
S23 S24 S25 S26 S39 S40 S53 S54 S67 S68 S7 S8 S81 S82 S95 S96
S21 S22 S35 S36 S37 S38 S5 S51 S52 S6 S65 S66 S79 S80 S85 S86
S1 S15 S16 S2 S29 S30 S43 S44 S57 S58 S71 S72 S75 S76 S91 S92
S19 S20 S3 S33 S34 S4 S47 S48 S49 S50 S63 S64 S77 S78 S89 S90
S10 S17 S18 S31 S32 S45 S46 S59 S60 S61 S62 S73 S74 S87 S88 S9
sample

ENSGACG00000011113 chrXVIII 11202174 d[illegible]

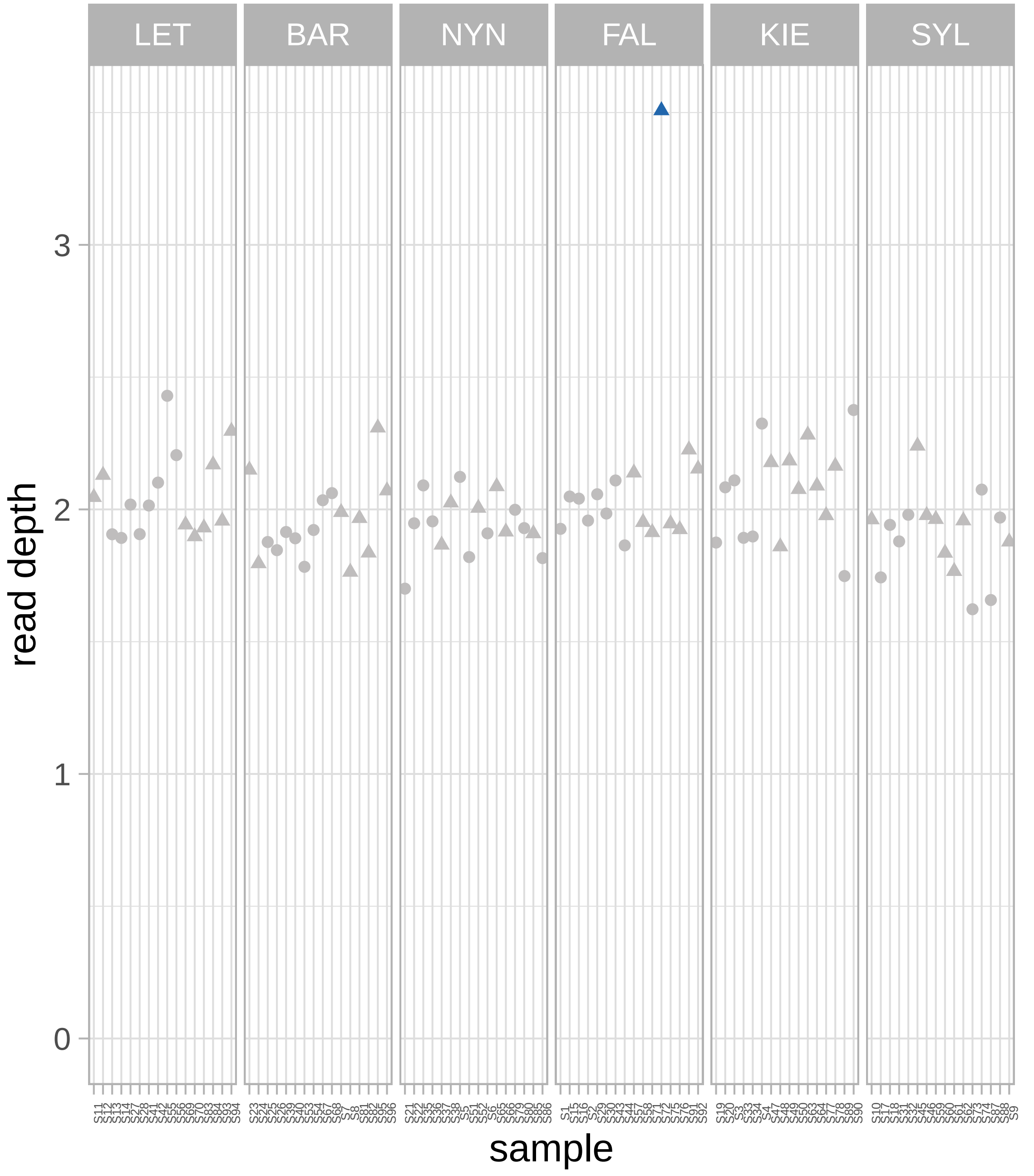

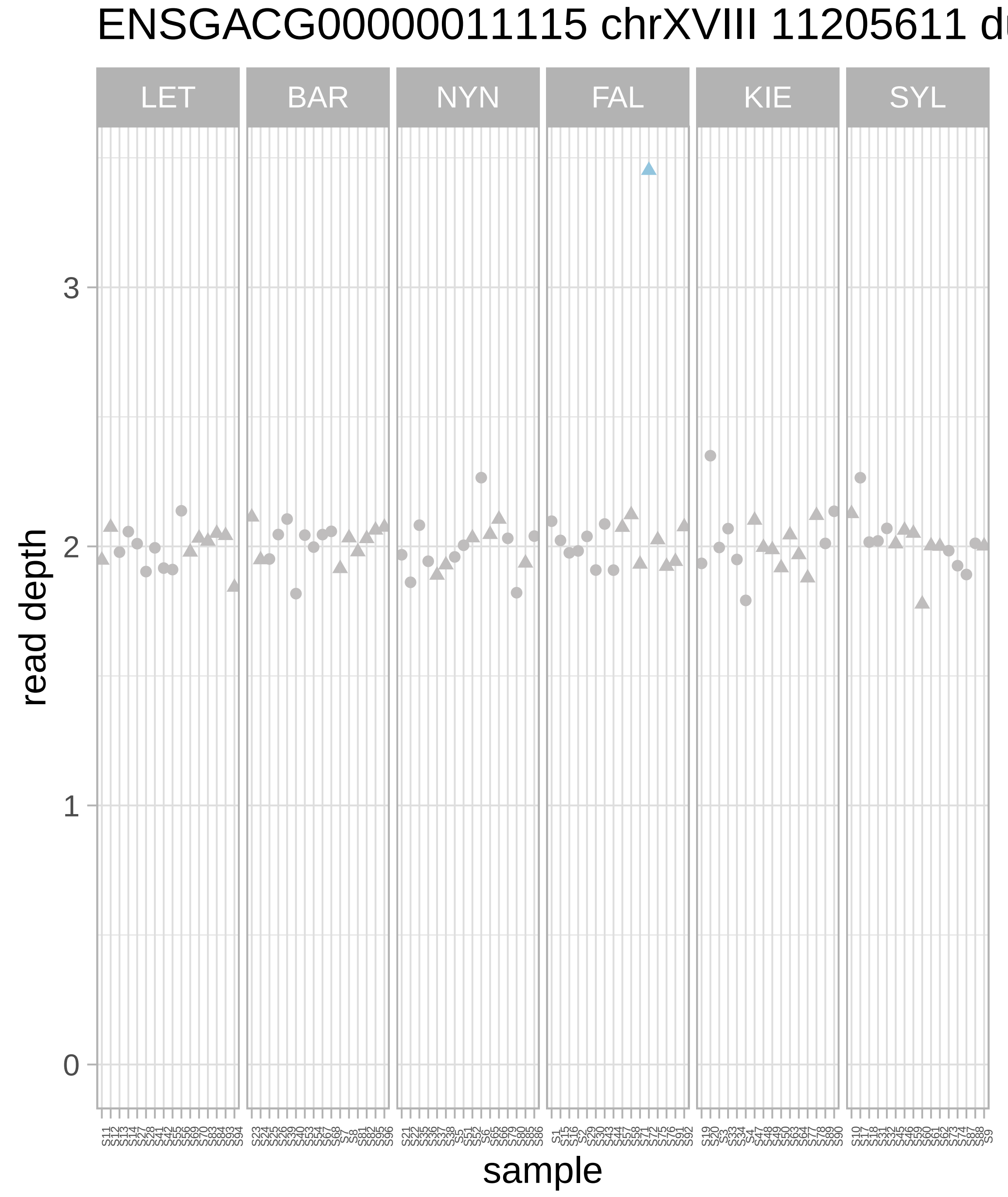
ENSGACG00000011115 chrXVIII 11205611 du
LET
BAR
NYN
FAL
KIE
SYL
read depth
0
1
2
3
S11 S12 S13 S14 S27 S28 S41 S42 S55 S56 S69 S70 S83 S84 S93 S94
S23 S24 S25 S26 S39 S40 S53 S54 S67 S68 S7 S8 S81 S82 S95 S96
S21 S22 S35 S36 S37 S38 S5 S51 S52 S6 S65 S66 S79 S80 S85 S86
S1 S15 S16 S2 S29 S30 S43 S44 S57 S58 S71 S72 S75 S76 S91 S92
S19 S20 S3 S33 S34 S4 S47 S48 S49 S50 S63 S64 S77 S78 S89 S90
S10 S17 S18 S31 S32 S45 S46 S59 S60 S61 S62 S73 S74 S87 S88 S9
sample

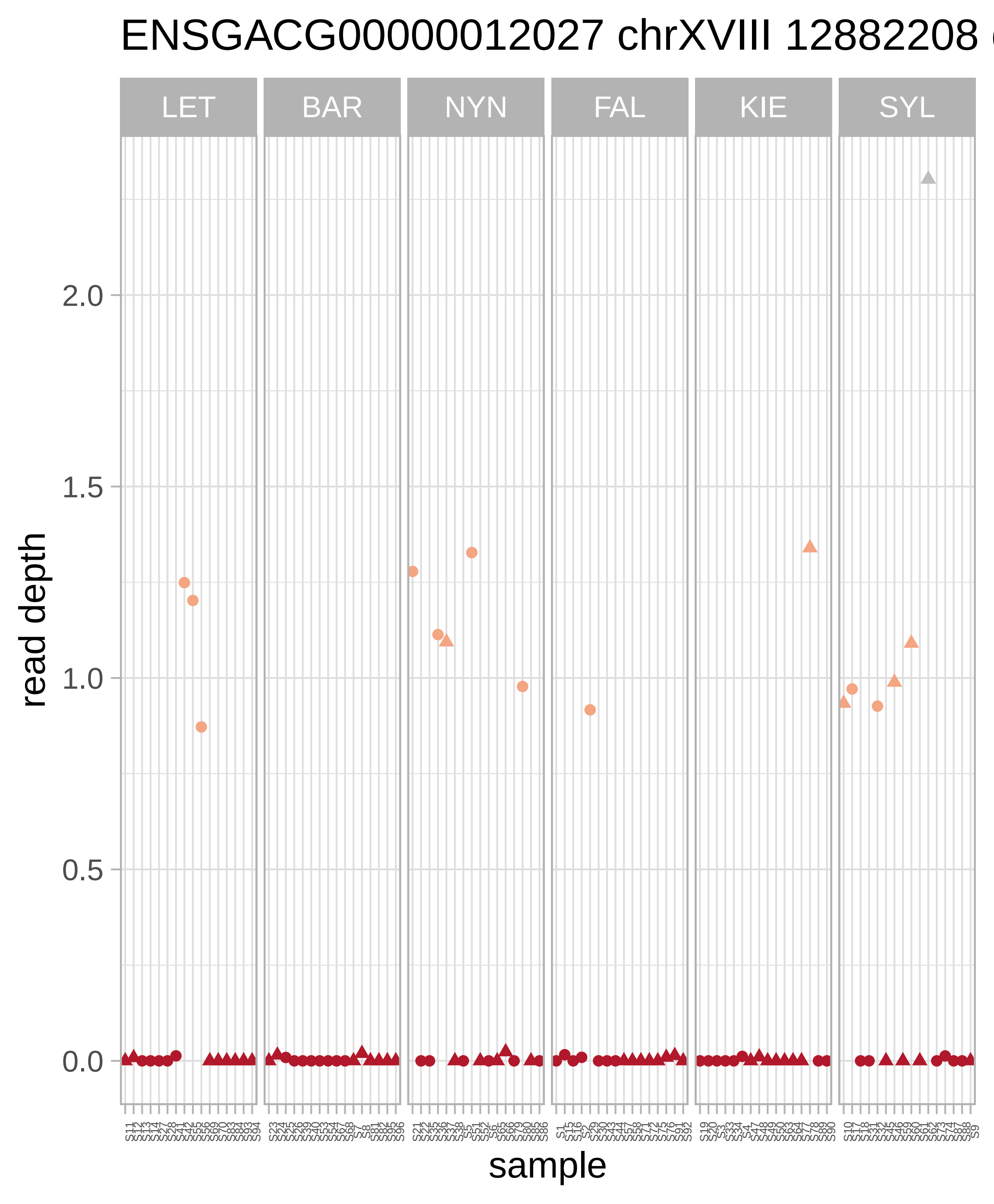
ENSGACG00000012027 chrXVIII 12882208
LET
BAR
NYN
FAL
KIE
SYL
read depth
2.0
1.5
1.0
0.5
0.0
sample

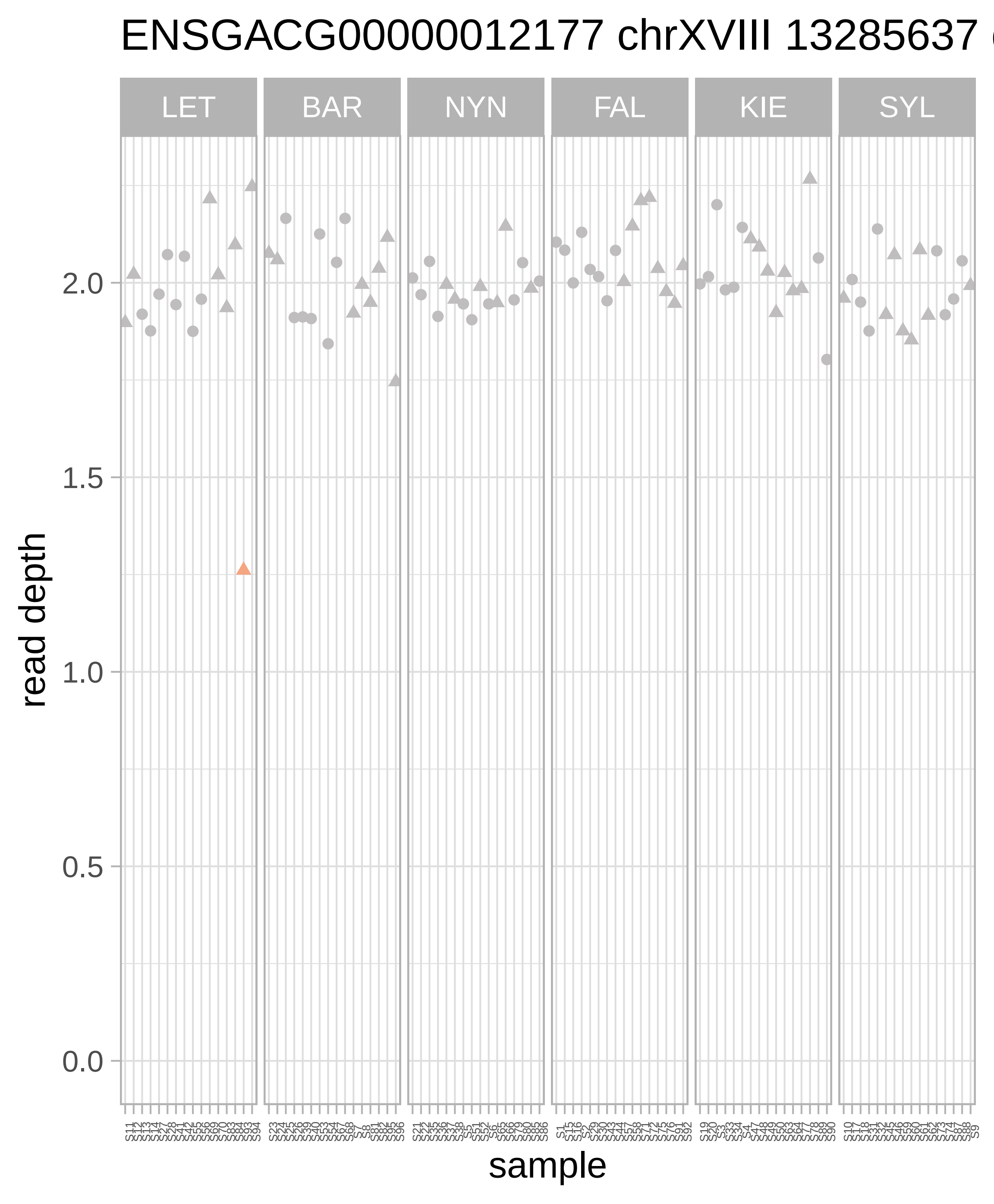
ENSGACG00000012177 chrXVIII 13285637
LET
BAR
NYN
FAL
KIE
SYL
read depth
2.0
1.5
1.0
0.5
0.0
sample

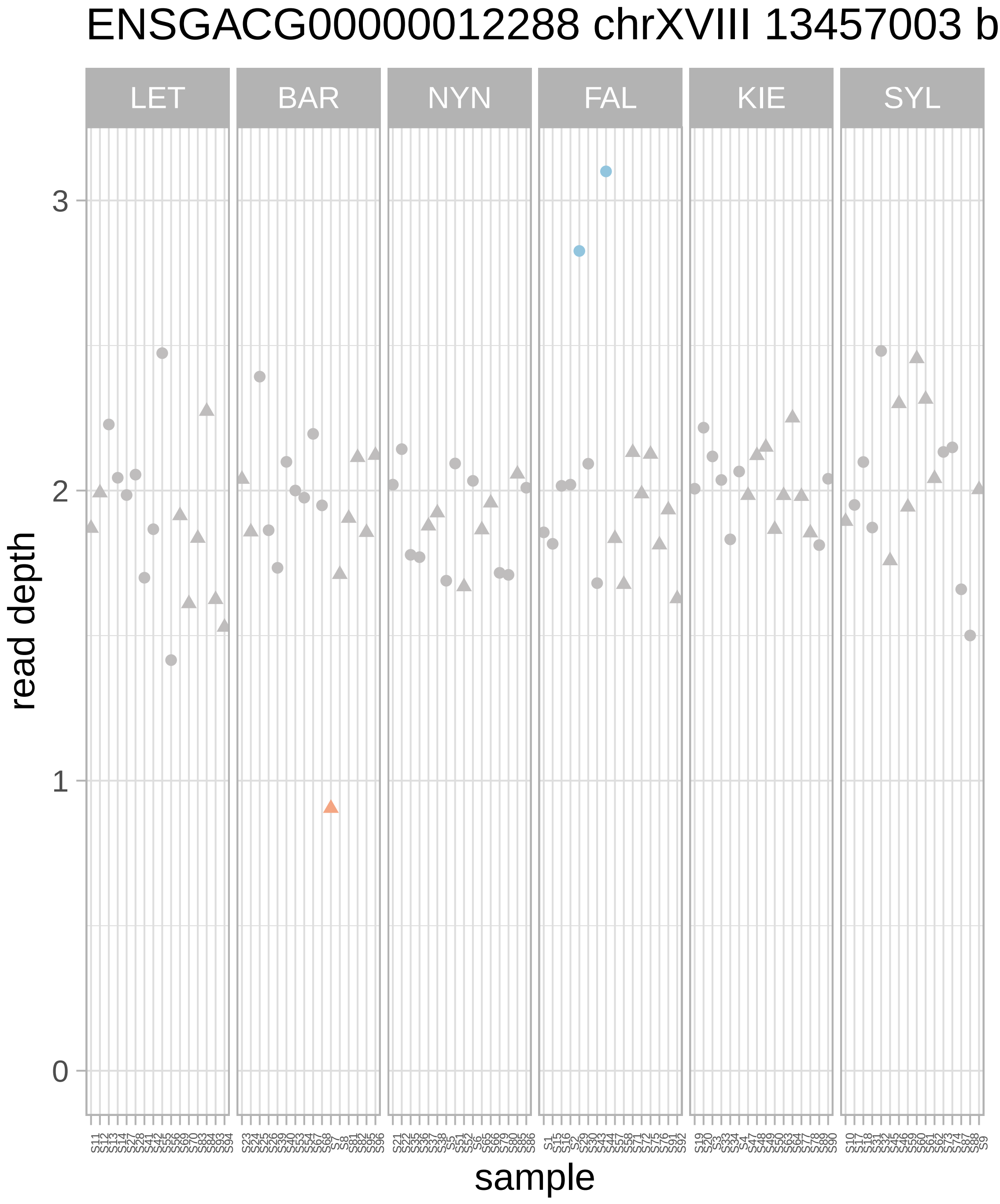
ENSGACG00000012288 chrXVIII 13457003
LET
BAR
NYN
FAL
KIE
SYL
read depth
0
1
2
3
sample

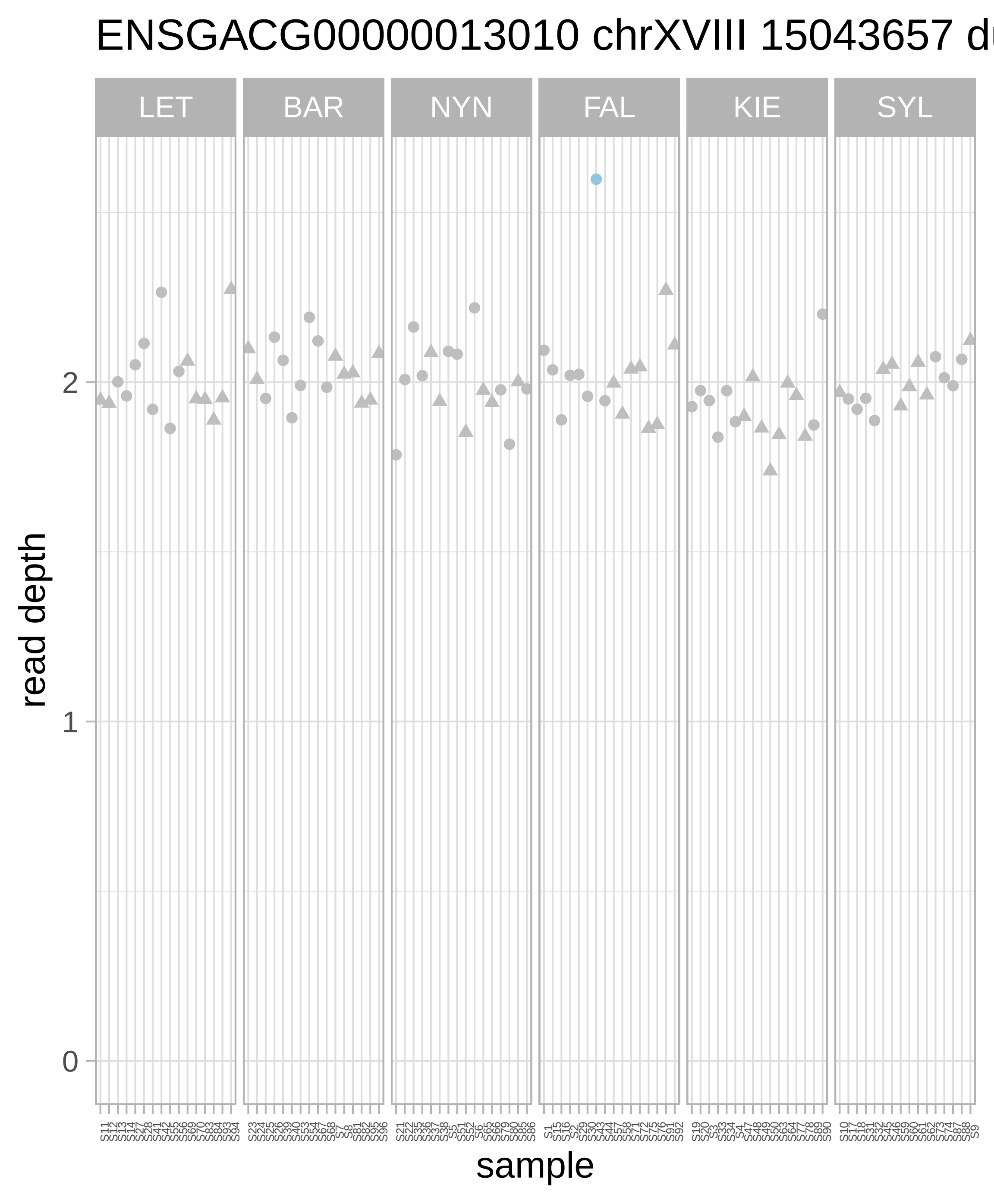

ENSGACG00000013010 chrXVIII 15043657 d
LET
BAR
NYN
FAL
KIE
SYL
read depth
0
1
2
sample

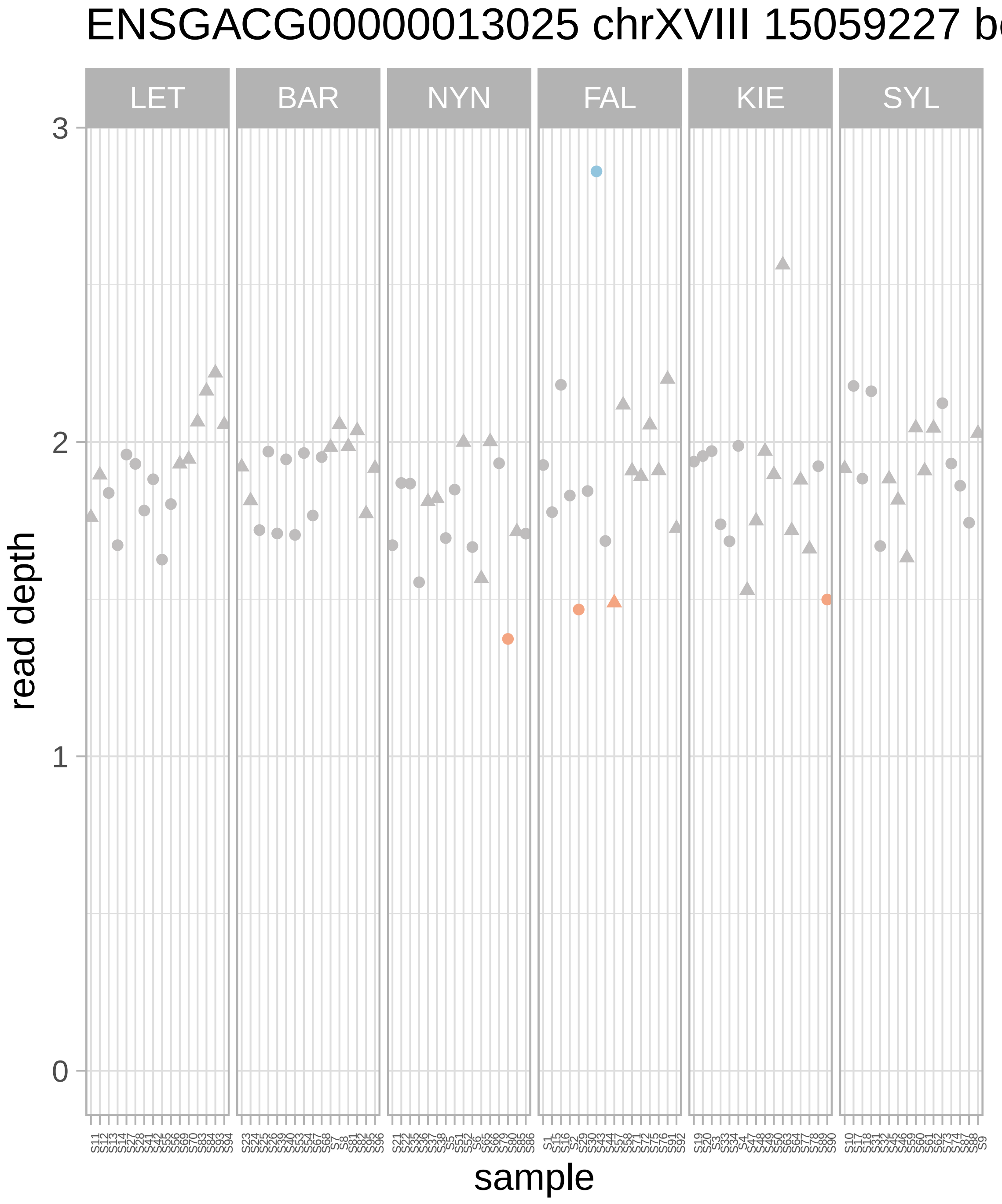
ENSGACG00000013025 chrXVIII 15059227 bo
LET
BAR
NYN
FAL
KIE
SYL
3
2
1
0
read depth
sample

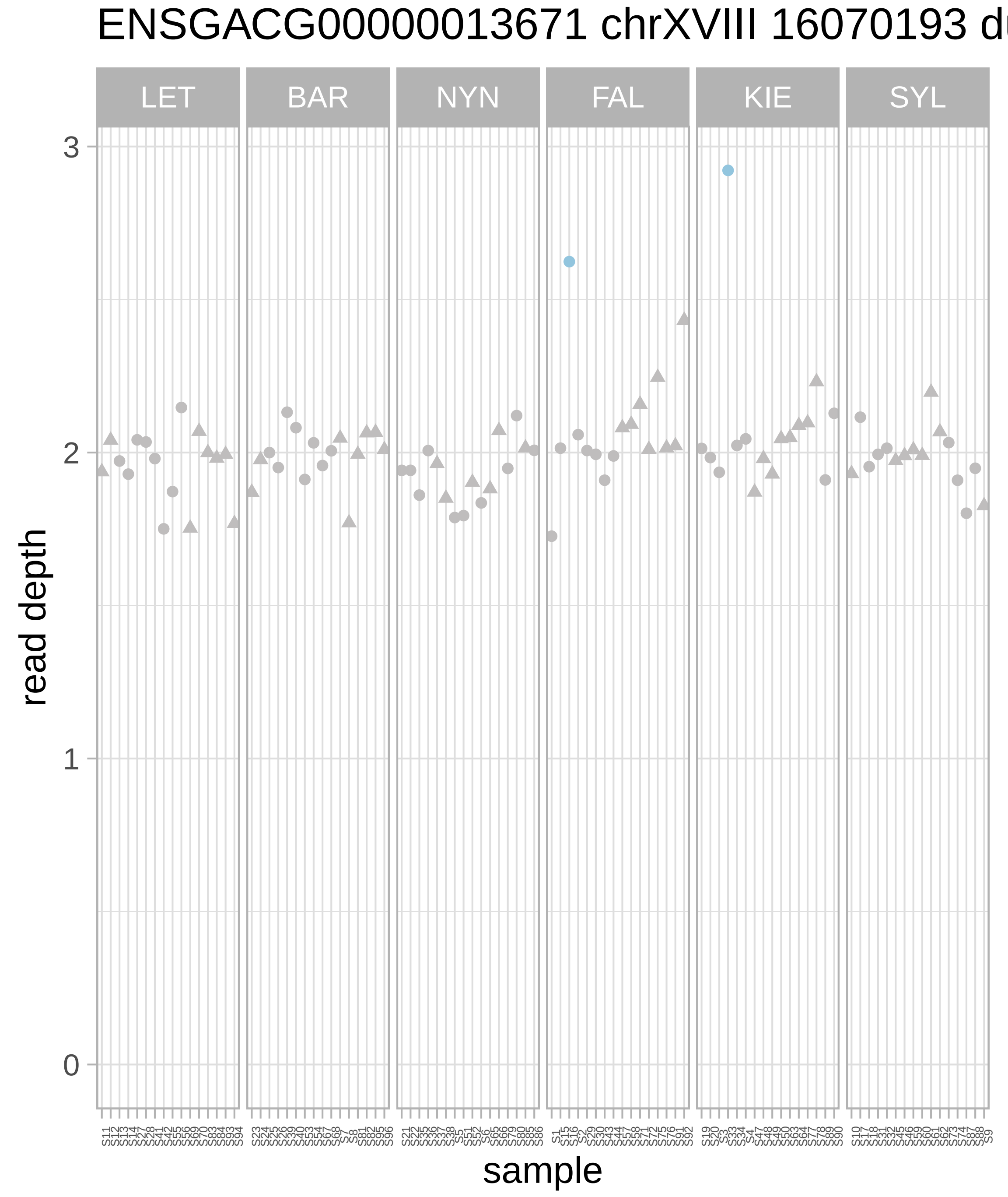
ENSGACG00000013671 chrXVIII 16070193 d
LET
BAR
NYN
FAL
KIE
SYL
3
2
1
0
read depth
S11 S12 S13 S14 S27 S28 S41 S42 S55 S56 S69 S70 S83 S84 S93 S94
S23 S24 S25 S26 S39 S40 S53 S54 S67 S68 S7 S8 S81 S82 S95 S96
S21 S22 S35 S36 S37 S38 S5 S51 S52 S6 S65 S66 S79 S80 S85 S86
S1 S15 S16 S2 S29 S30 S43 S44 S57 S58 S71 S72 S75 S76 S91 S92
S19 S20 S3 S33 S34 S4 S47 S48 S49 S50 S63 S64 S77 S78 S89 S90
S10 S17 S18 S31 S32 S45 S46 S59 S60 S61 S62 S73 S74 S87 S88 S9
sample

ENSGACG00000013674 chrXVIII 16079845 d

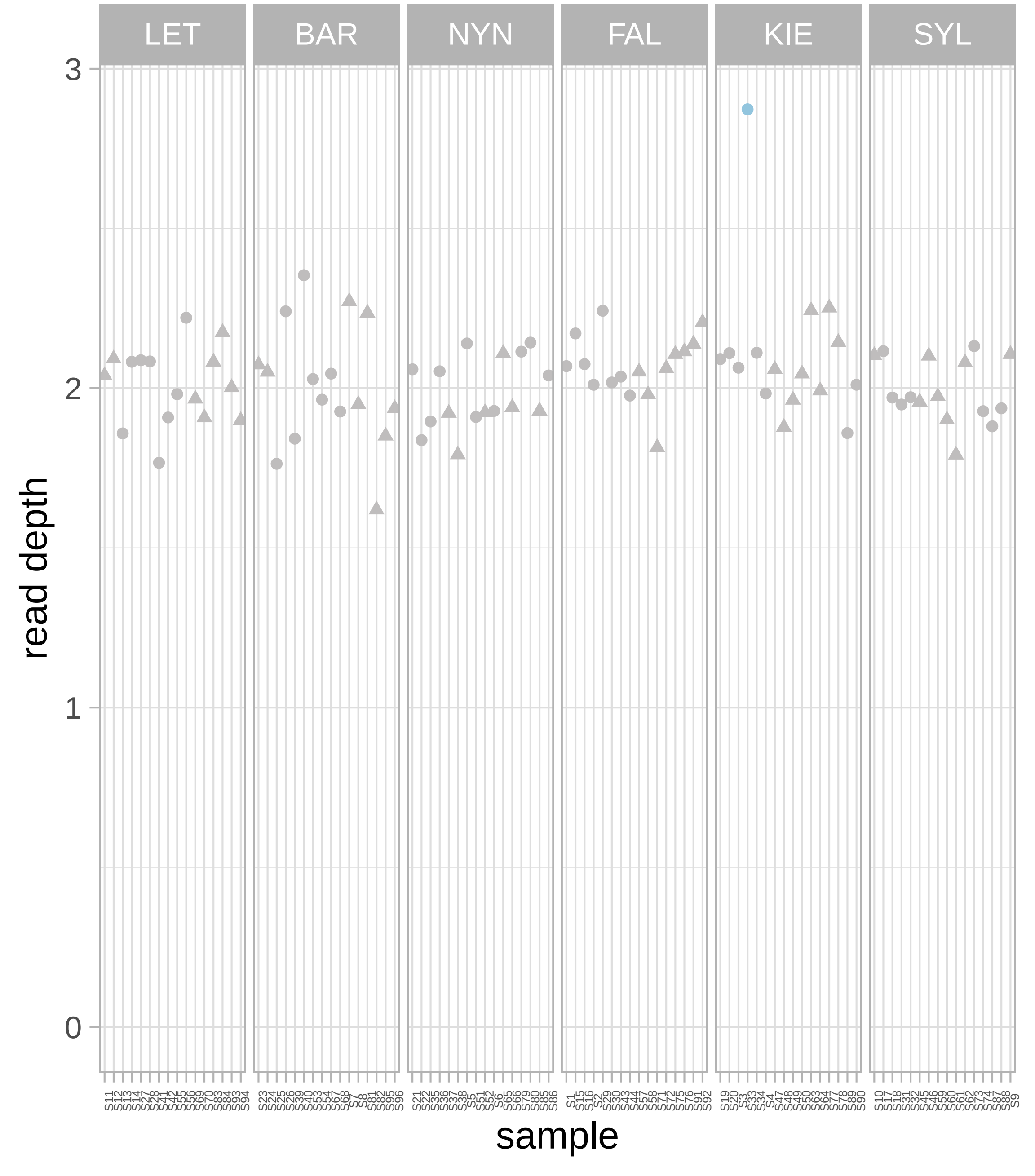

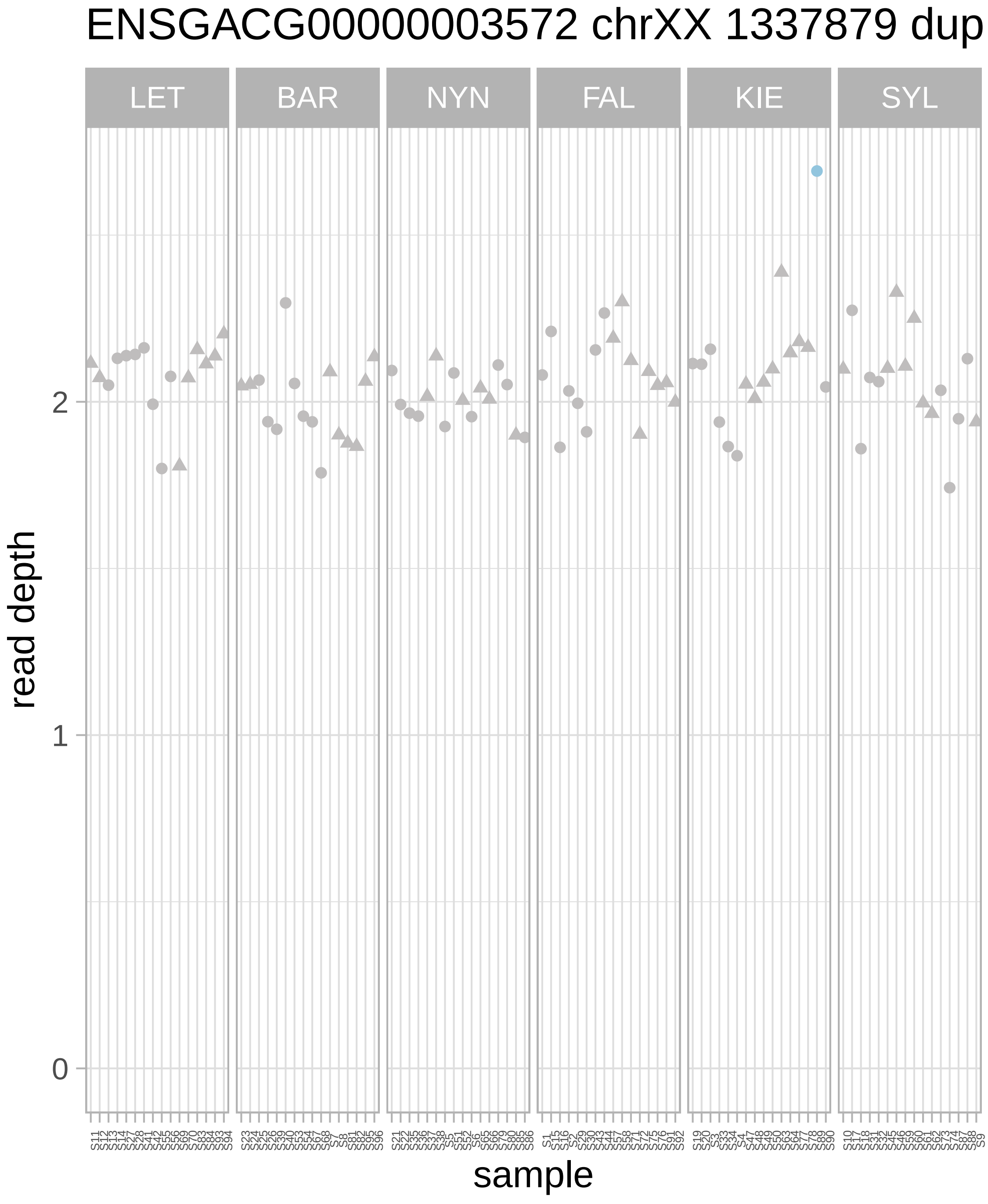

ENSGACG00000003572 chrXX 1337879 dup
LET
BAR
NYN
FAL
KIE
SYL
read depth
0
1
2
sample
S11 S12 S13 S14 S27 S28 S41 S42 S55 S56 S69 S70 S83 S84 S93 S94
S23 S24 S25 S26 S39 S40 S53 S54 S67 S68 S7 S8 S81 S82 S95 S96
S21 S22 S35 S36 S37 S38 S5 S51 S52 S6 S65 S66 S79 S80 S85 S86
S1 S15 S16 S2 S29 S30 S43 S44 S57 S58 S71 S72 S75 S76 S91 S92
S19 S20 S3 S33 S34 S4 S47 S48 S49 S50 S63 S64 S77 S78 S89 S90
S10 S17 S18 S31 S32 S45 S46 S59 S60 S61 S62 S73 S74 S87 S88 S9

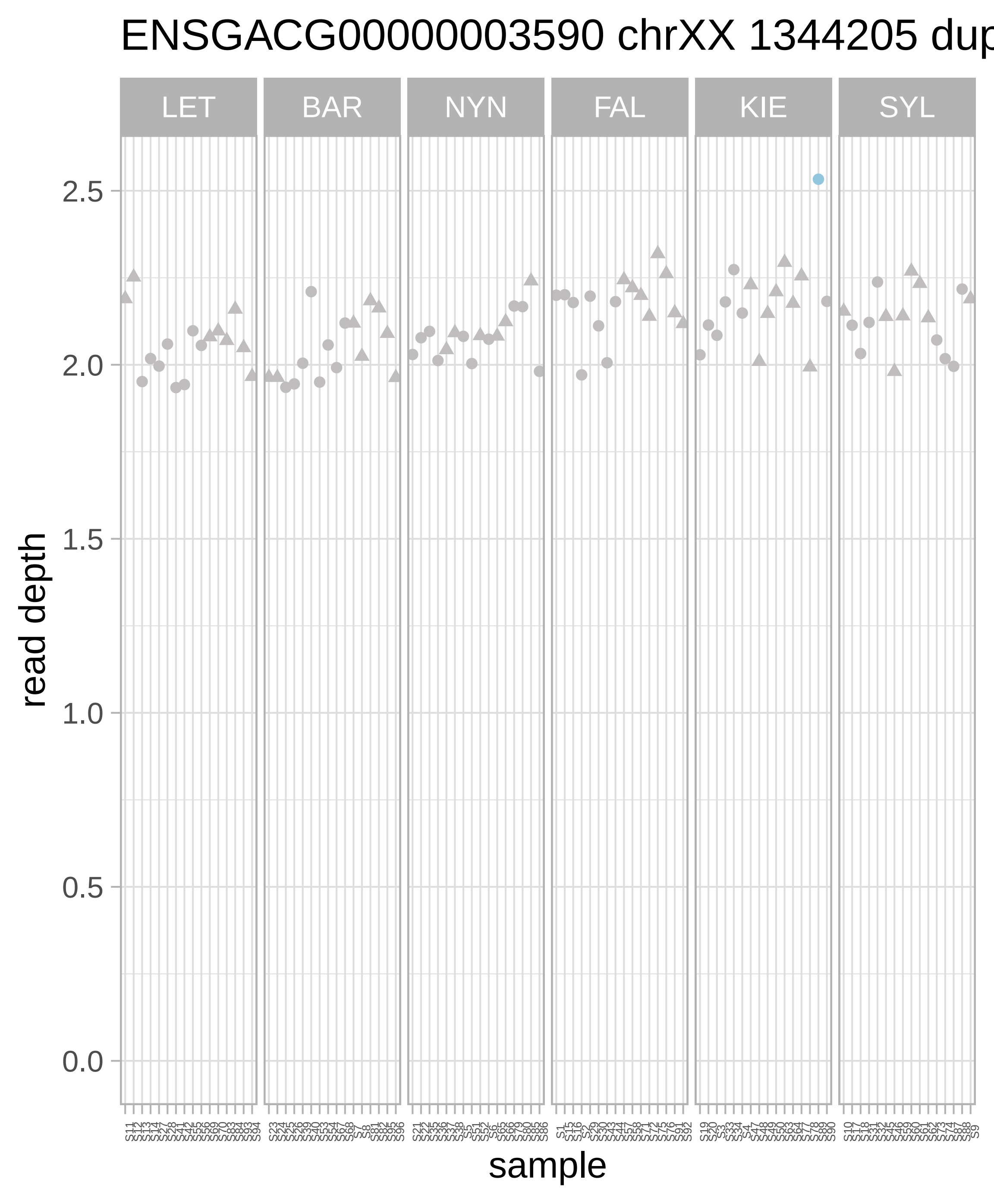

ENSGACG00000003590 chrXX 1344205 dup
LET
BAR
NYN
FAL
KIE
SYL
2.5
2.0
1.5
1.0
0.5
0.0
read depth
sample

ENSGACG00000005839 chrXX 5066709 del

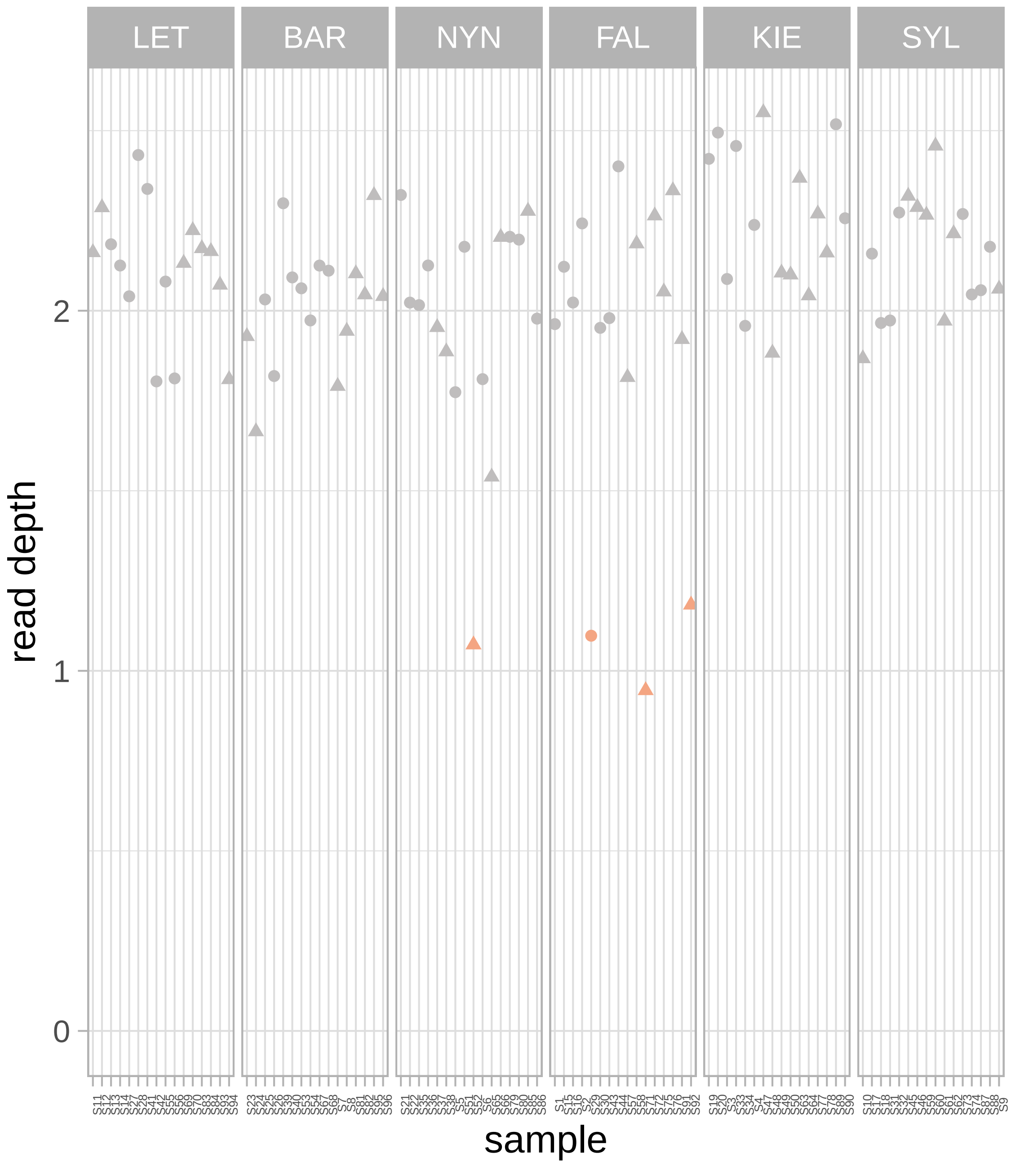

ENSGACG00000007532 chrXX 8774436 dup

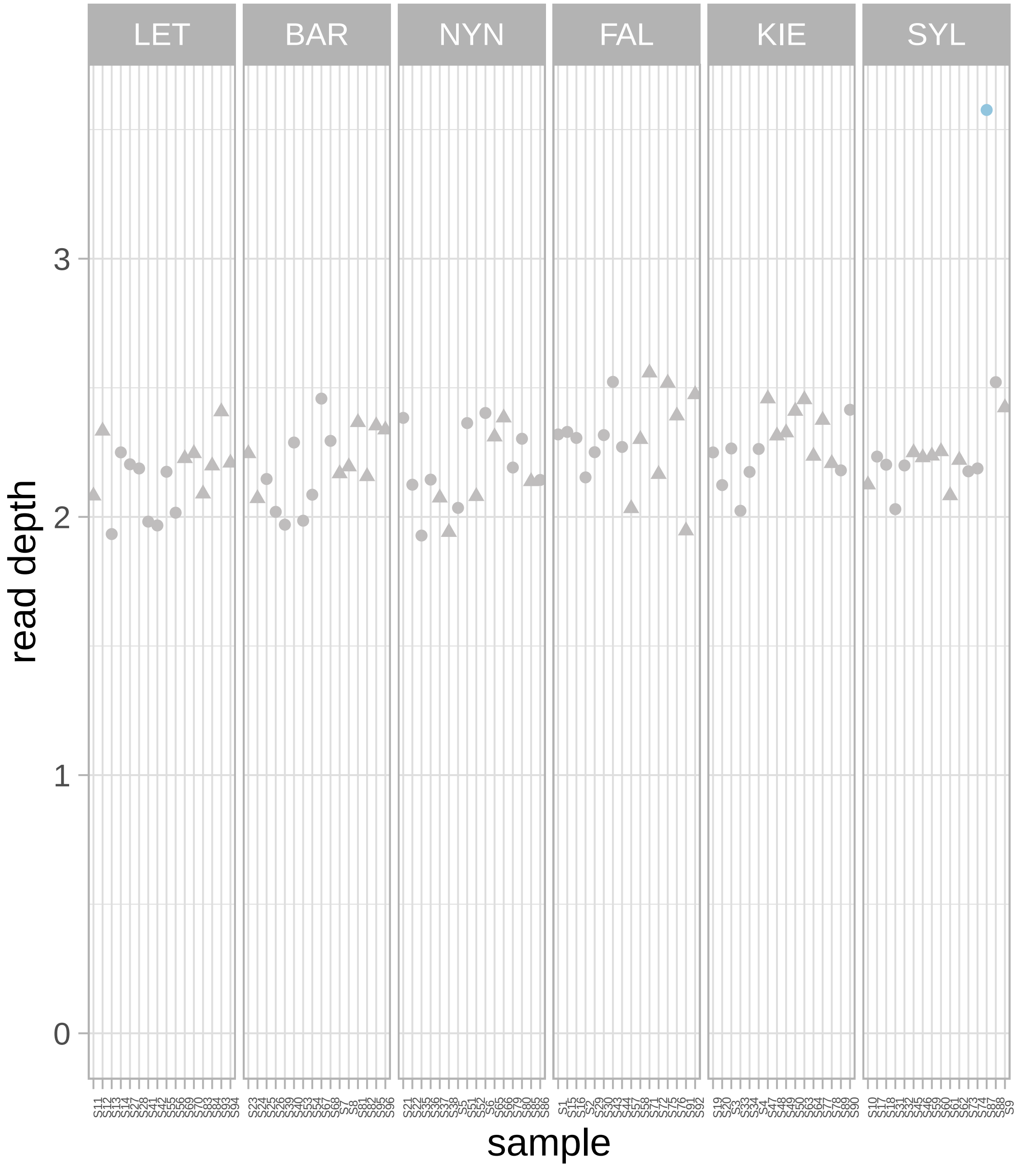

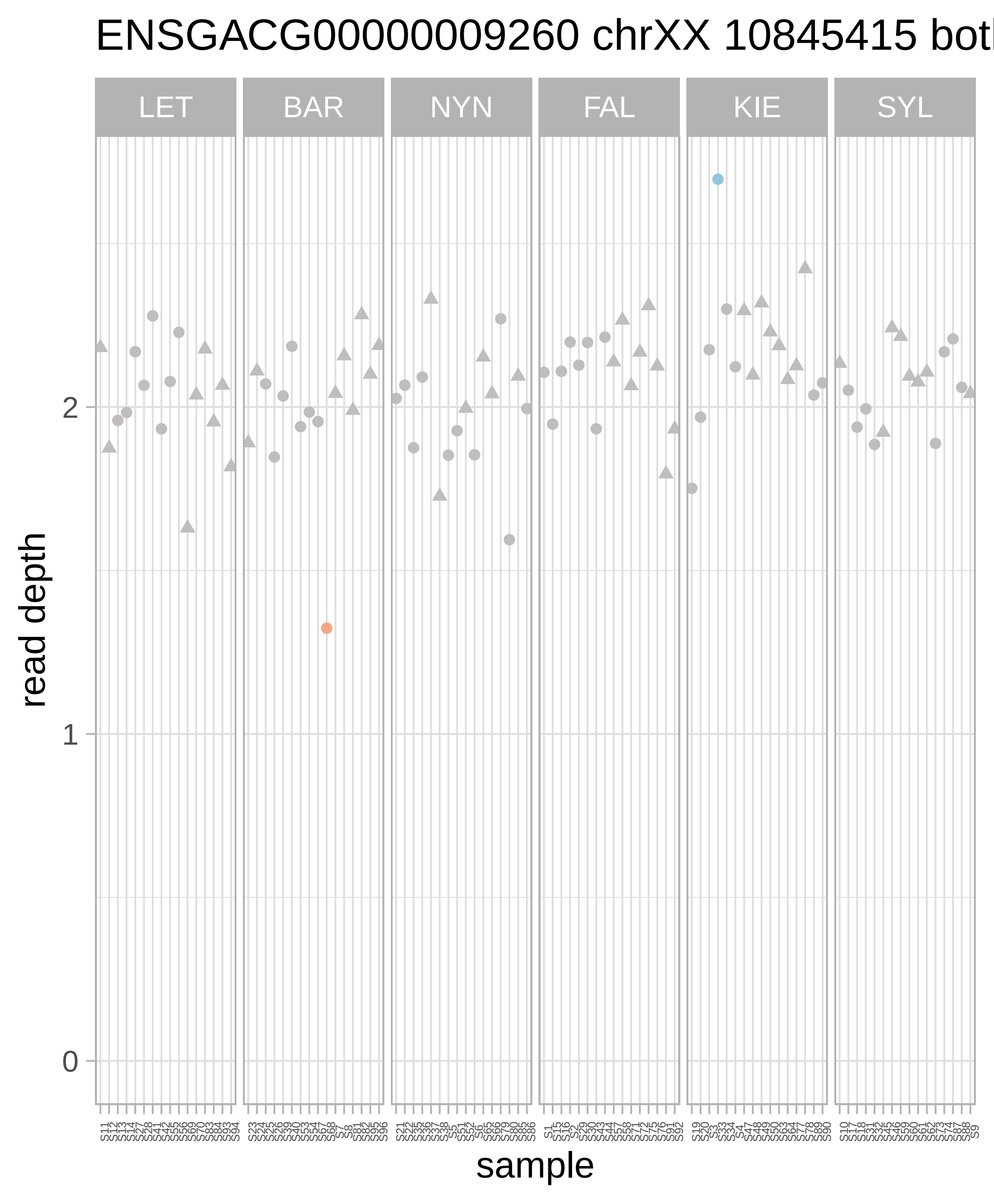

ENSGACG00000009260 chrXX 10845415 bot
LET
BAR
NYN
FAL
KIE
SYL
read depth
0
1
2
sample

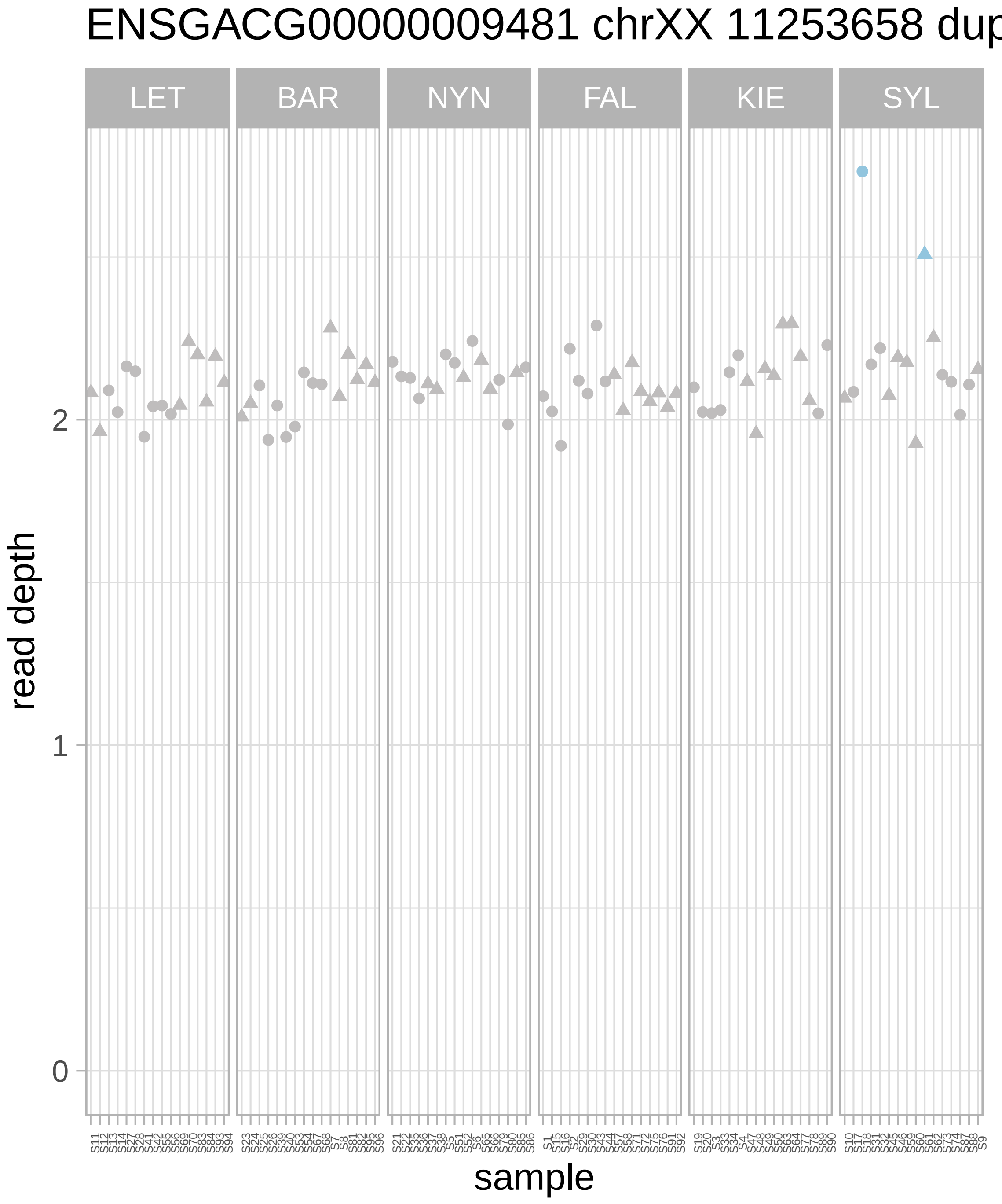
ENSGACG00000009481 chrXX 11253658 dup
LET
BAR
NYN
FAL
KIE
SYL
read depth
0
1
2
sample
S11 S12 S13 S14 S27 S28 S41 S42 S55 S56 S69 S70 S83 S84 S93 S94
S23 S24 S25 S26 S39 S40 S53 S54 S67 S68 S7 S8 S81 S82 S95 S96
S21 S22 S35 S36 S37 S38 S5 S51 S52 S6 S65 S66 S79 S80 S85 S86
S1 S15 S16 S2 S29 S30 S43 S44 S57 S58 S71 S72 S75 S76 S91 S92
S19 S20 S3 S33 S34 S4 S47 S48 S49 S50 S63 S64 S77 S78 S89 S90
S10 S17 S18 S31 S32 S45 S46 S59 S60 S61 S62 S73 S74 S87 S88 S9

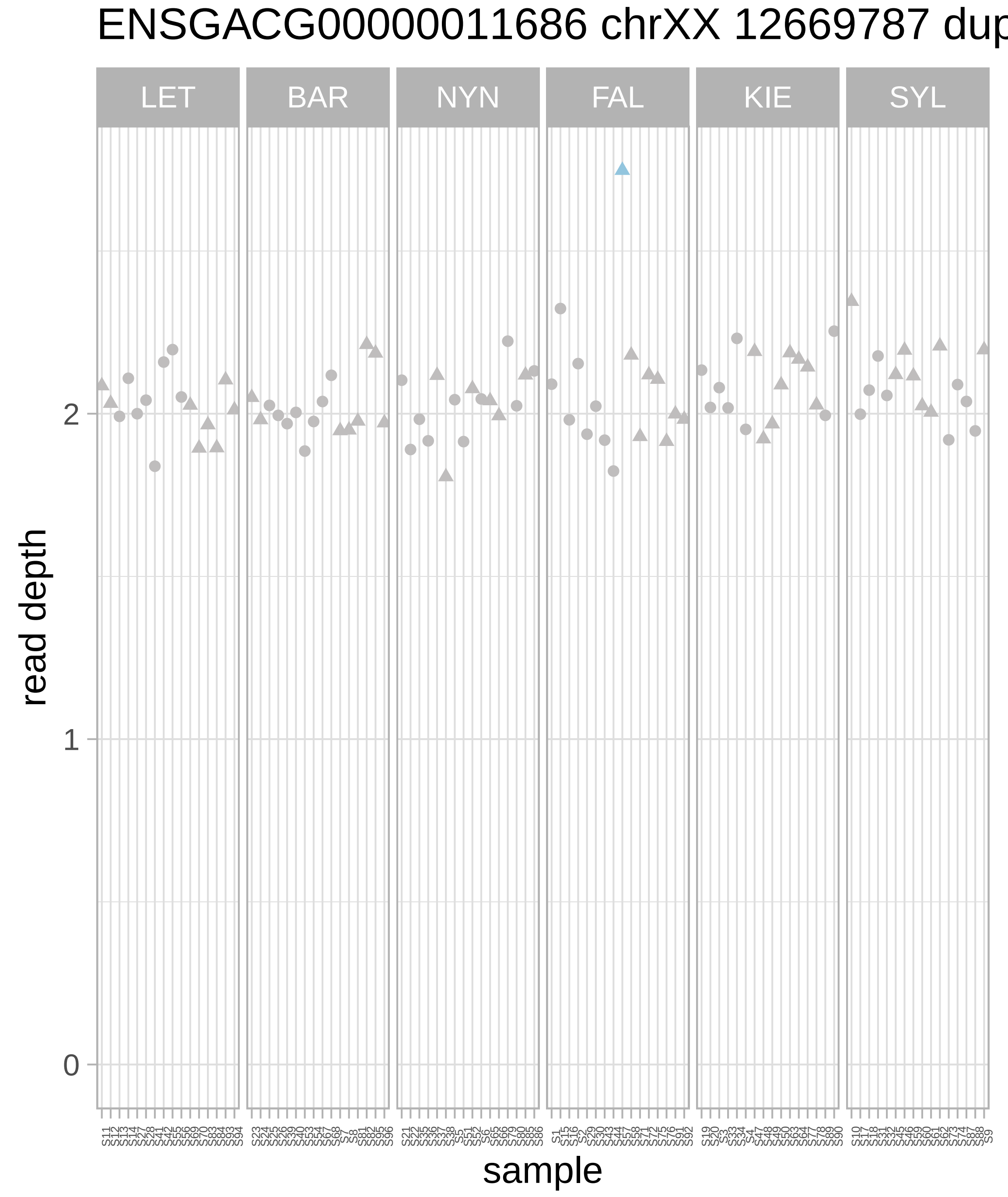
ENSGACG00000011686 chrXX 12669787 dup
LET
BAR
NYN
FAL
KIE
SYL
read depth
0
1
2
S11 S12 S13 S14 S27 S28 S41 S42 S55 S56 S69 S70 S83 S84 S93 S94
S23 S24 S25 S26 S39 S40 S53 S54 S67 S68 S7 S8 S81 S82 S95 S96
S21 S22 S35 S36 S37 S38 S5 S51 S52 S6 S65 S66 S79 S80 S85 S86
S1 S15 S16 S2 S29 S30 S43 S44 S57 S58 S71 S72 S75 S76 S91 S92
S19 S20 S3 S33 S34 S4 S47 S48 S49 S50 S63 S64 S77 S78 S89 S90
S10 S17 S18 S31 S32 S45 S46 S59 S60 S61 S62 S73 S74 S87 S88 S9
sample

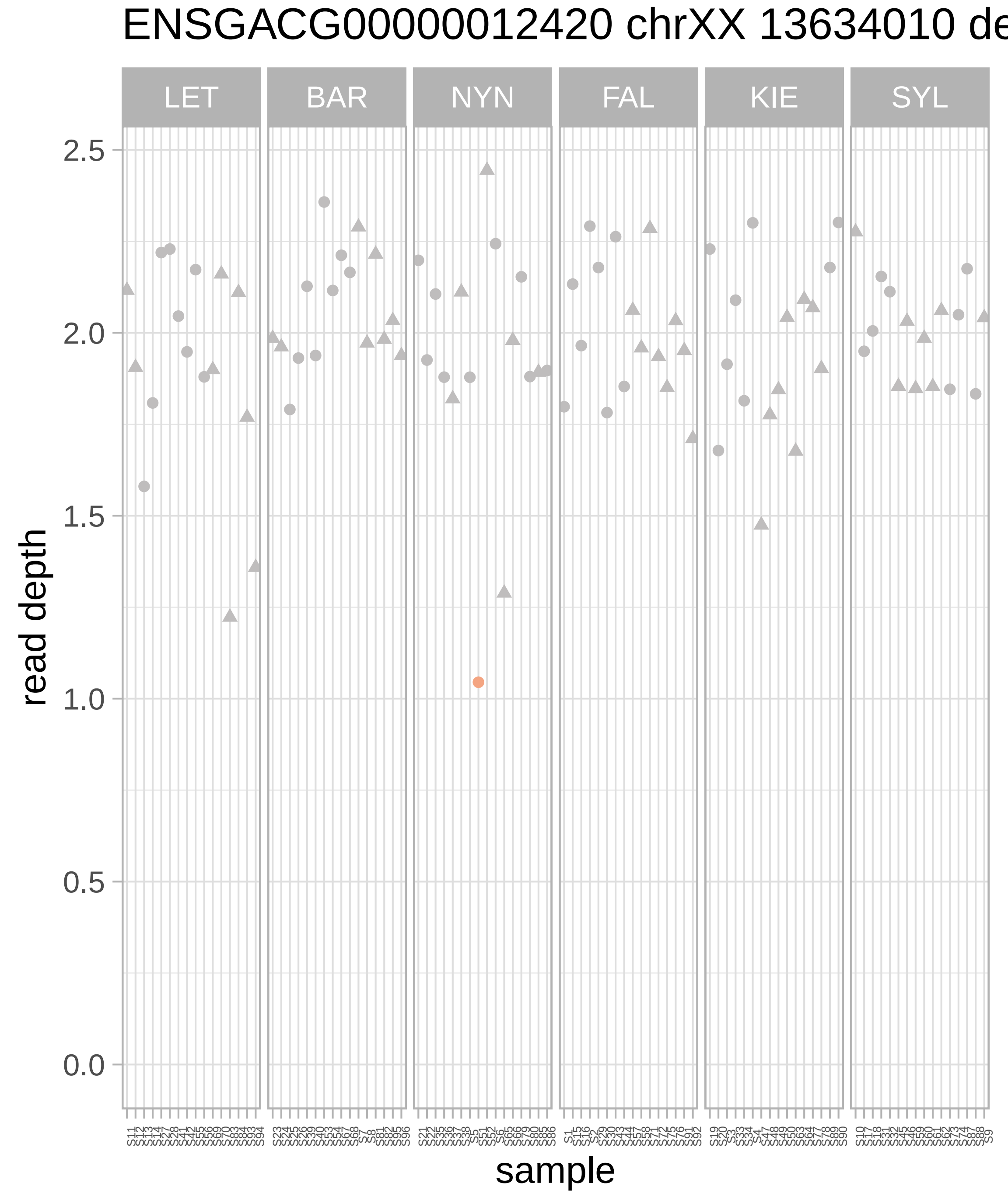

ENSGACG00000012420 chrXX 13634010 de
LET
BAR
NYN
FAL
KIE
SYL
read depth
sample
2.5
2.0
1.5
1.0
0.5
0.0

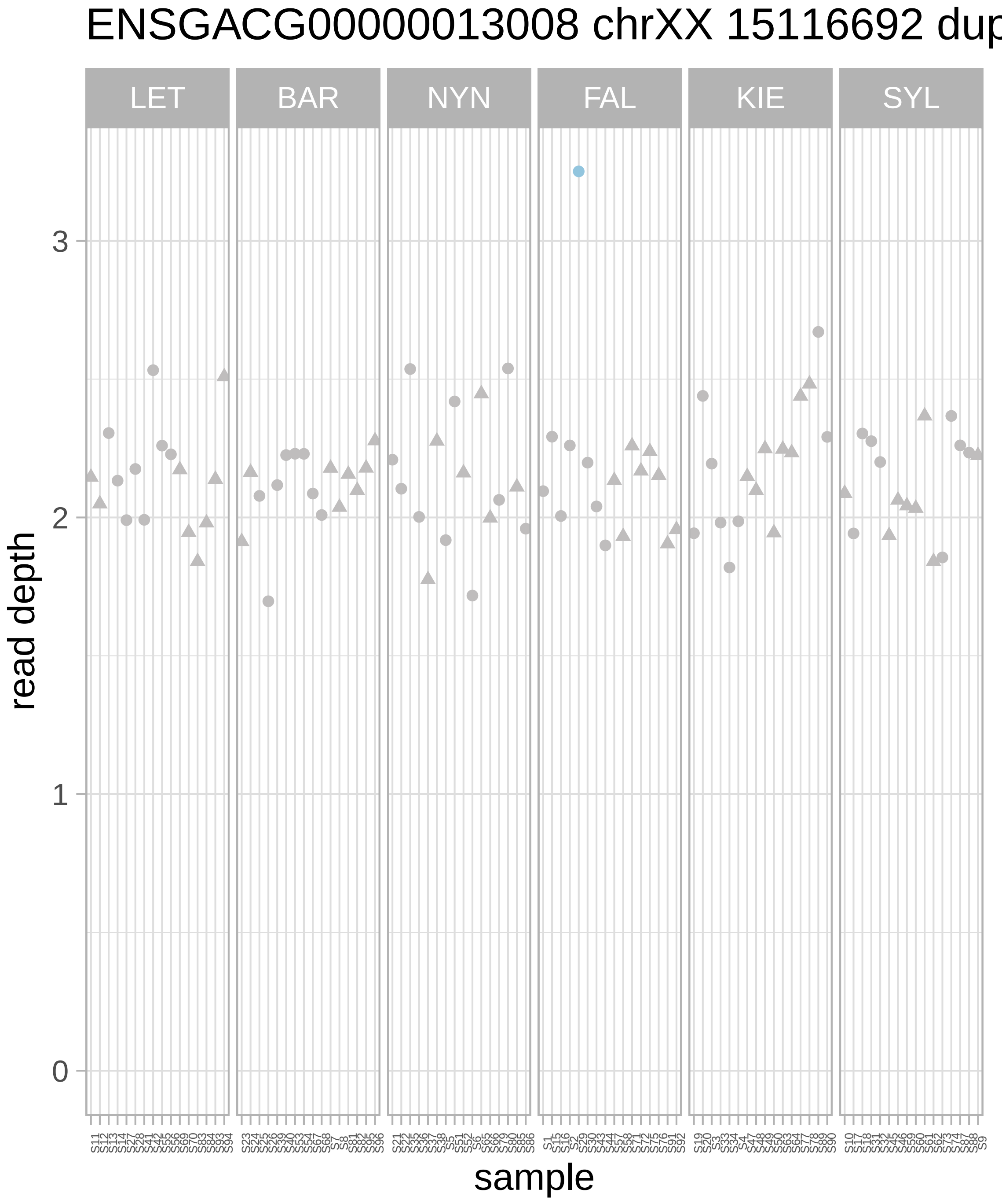
ENSGACG00000013008 chrXX 15116692 dup
LET
BAR
NYN
FAL
KIE
SYL
read depth
0
1
2
3
S11 S12 S13 S14 S27 S28 S41 S42 S55 S56 S69 S70 S83 S84 S93 S94
S23 S24 S25 S26 S39 S40 S53 S54 S67 S68 S7 S8 S81 S82 S95 S96
S21 S22 S35 S36 S37 S38 S5 S51 S52 S6 S65 S66 S79 S80 S85 S86
S1 S15 S16 S2 S29 S30 S43 S44 S57 S58 S71 S72 S75 S76 S91 S92
S19 S20 S3 S33 S34 S4 S47 S48 S49 S50 S63 S64 S77 S78 S89 S90
S10 S17 S18 S31 S32 S45 S46 S59 S60 S61 S62 S73 S74 S87 S88 S9
sample

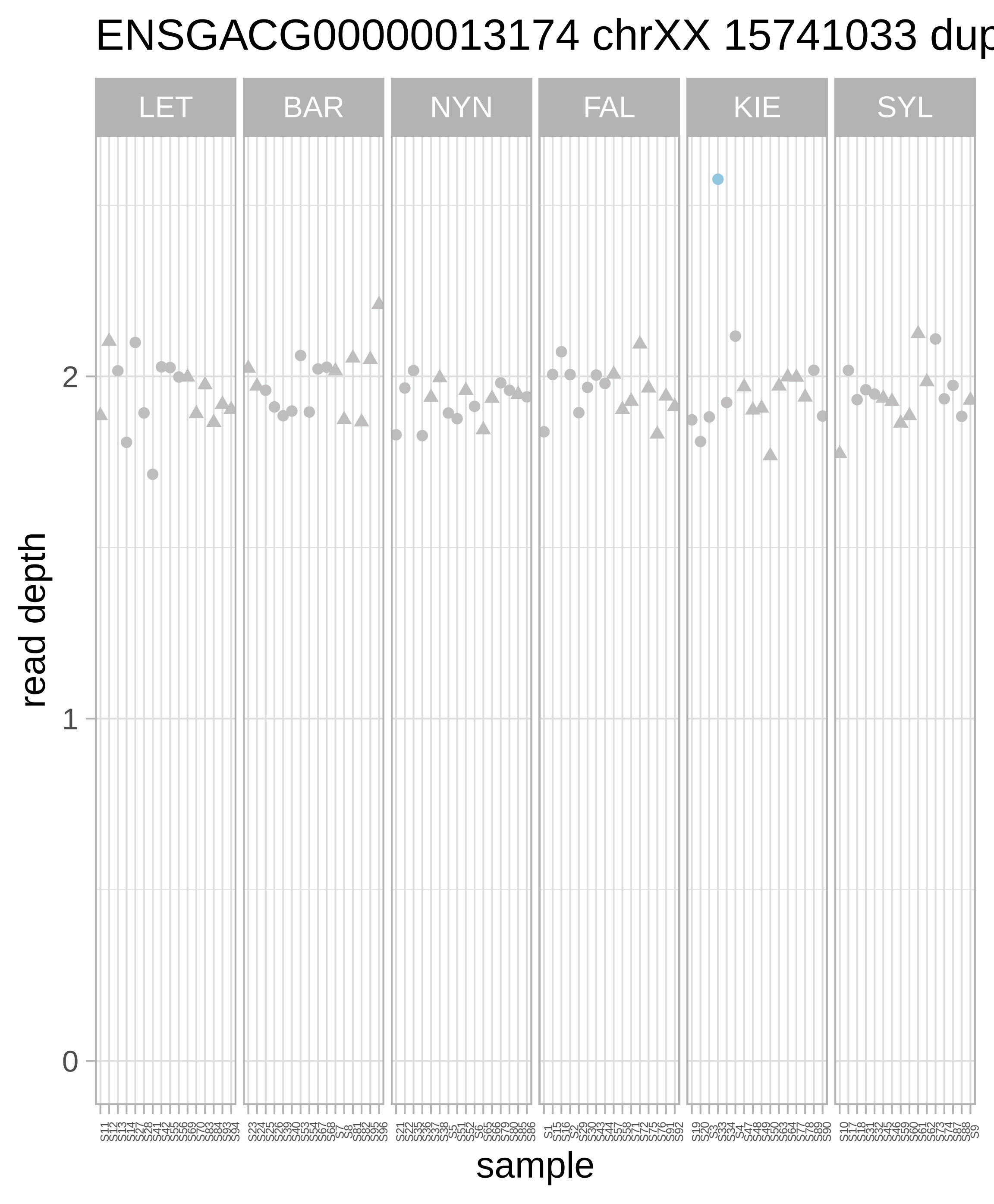
ENSGACG00000013174 chrXX 15741033 dup
LET
BAR
NYN
FAL
KIE
SYL
read depth
0
1
2
sample
S11 S12 S13 S14 S27 S28 S41 S42 S55 S56 S69 S70 S83 S84 S93 S94
S23 S24 S25 S26 S39 S40 S53 S54 S67 S68 S7 S8 S81 S82 S95 S96
S21 S22 S35 S36 S37 S38 S5 S51 S52 S6 S65 S66 S79 S80 S85 S86
S1 S15 S16 S2 S29 S30 S43 S44 S57 S58 S71 S72 S75 S76 S91 S92
S19 S20 S3 S33 S34 S4 S47 S48 S49 S50 S63 S64 S77 S78 S89 S90
S10 S17 S18 S31 S32 S45 S46 S59 S60 S61 S62 S73 S74 S87 S88 S9

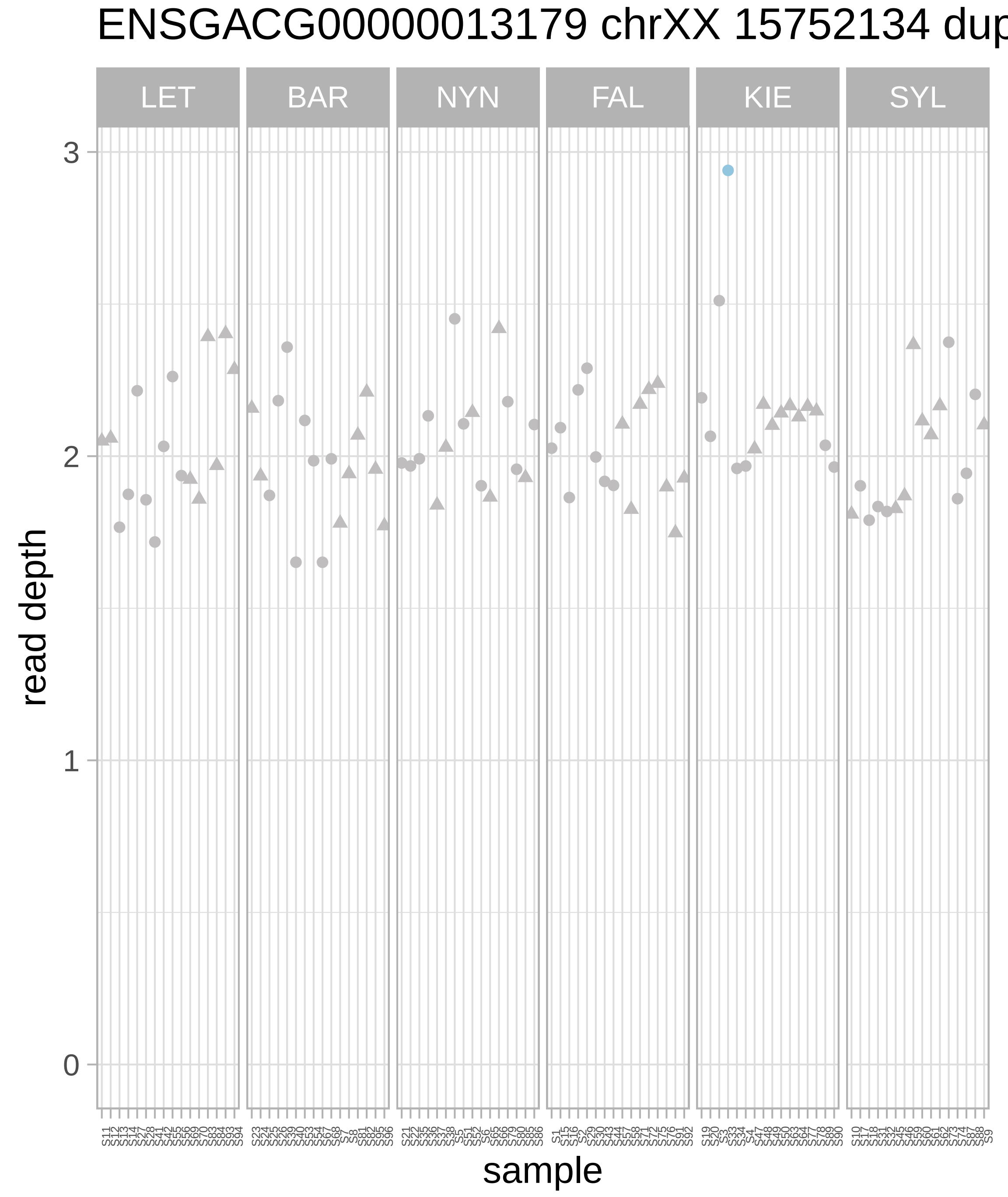

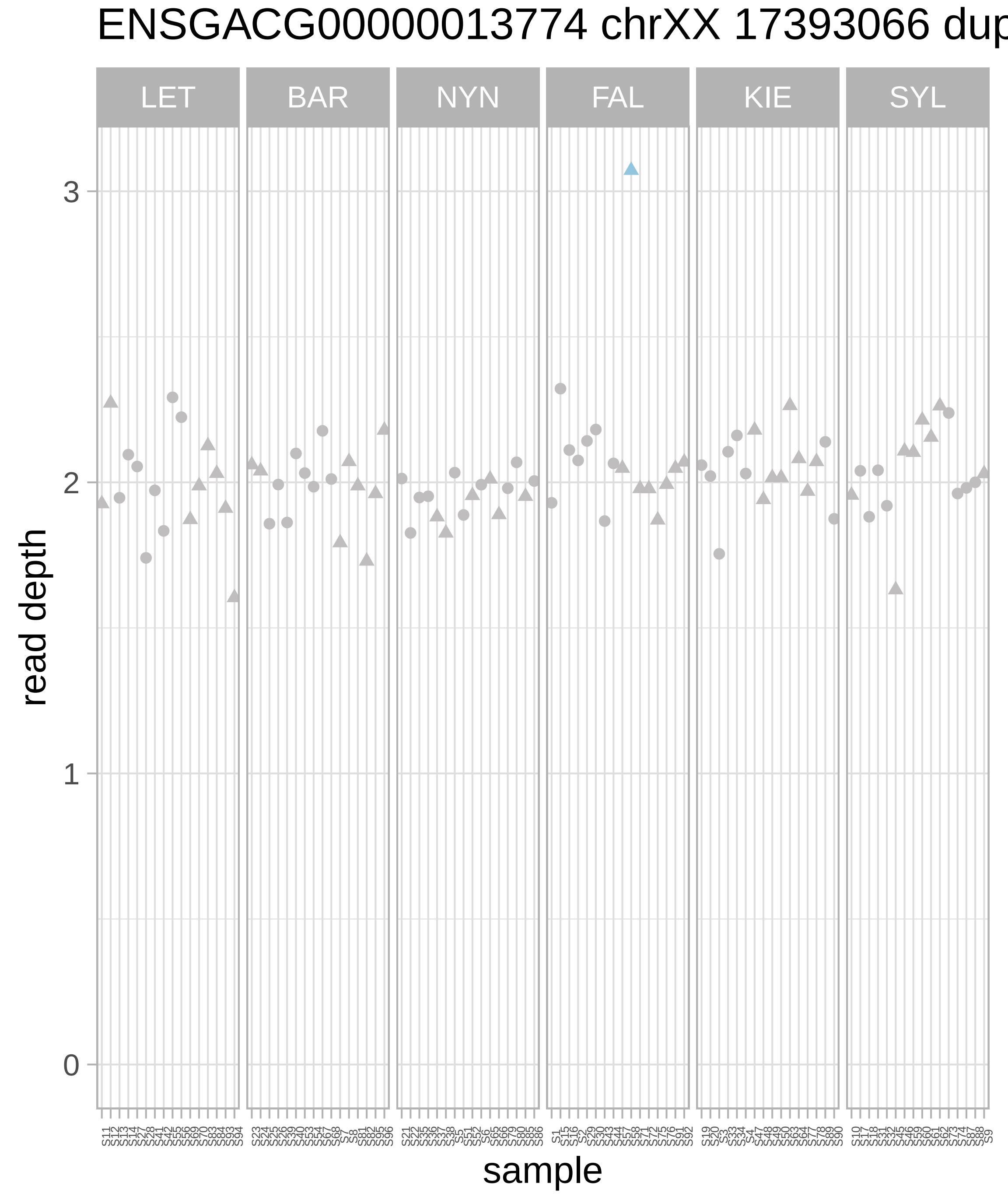
ENSGACG00000013774 chrXX 17393066 dup
LET
BAR
NYN
FAL
KIE
SYL
read depth
0
1
2
3
S11 S12 S13 S14 S27 S28 S41 S42 S55 S56 S69 S70 S83 S84 S93 S94
S23 S24 S25 S26 S39 S40 S53 S54 S67 S68 S7 S8 S81 S82 S95 S96
S21 S22 S35 S36 S37 S38 S5 S51 S52 S6 S65 S66 S79 S80 S85 S86
S1 S15 S16 S2 S29 S30 S43 S44 S57 S58 S71 S72 S75 S76 S91 S92
S19 S20 S3 S33 S34 S4 S47 S48 S49 S50 S63 S64 S77 S78 S89 S90
S10 S17 S18 S31 S32 S45 S46 S59 S60 S61 S62 S73 S74 S87 S88 S9
sample

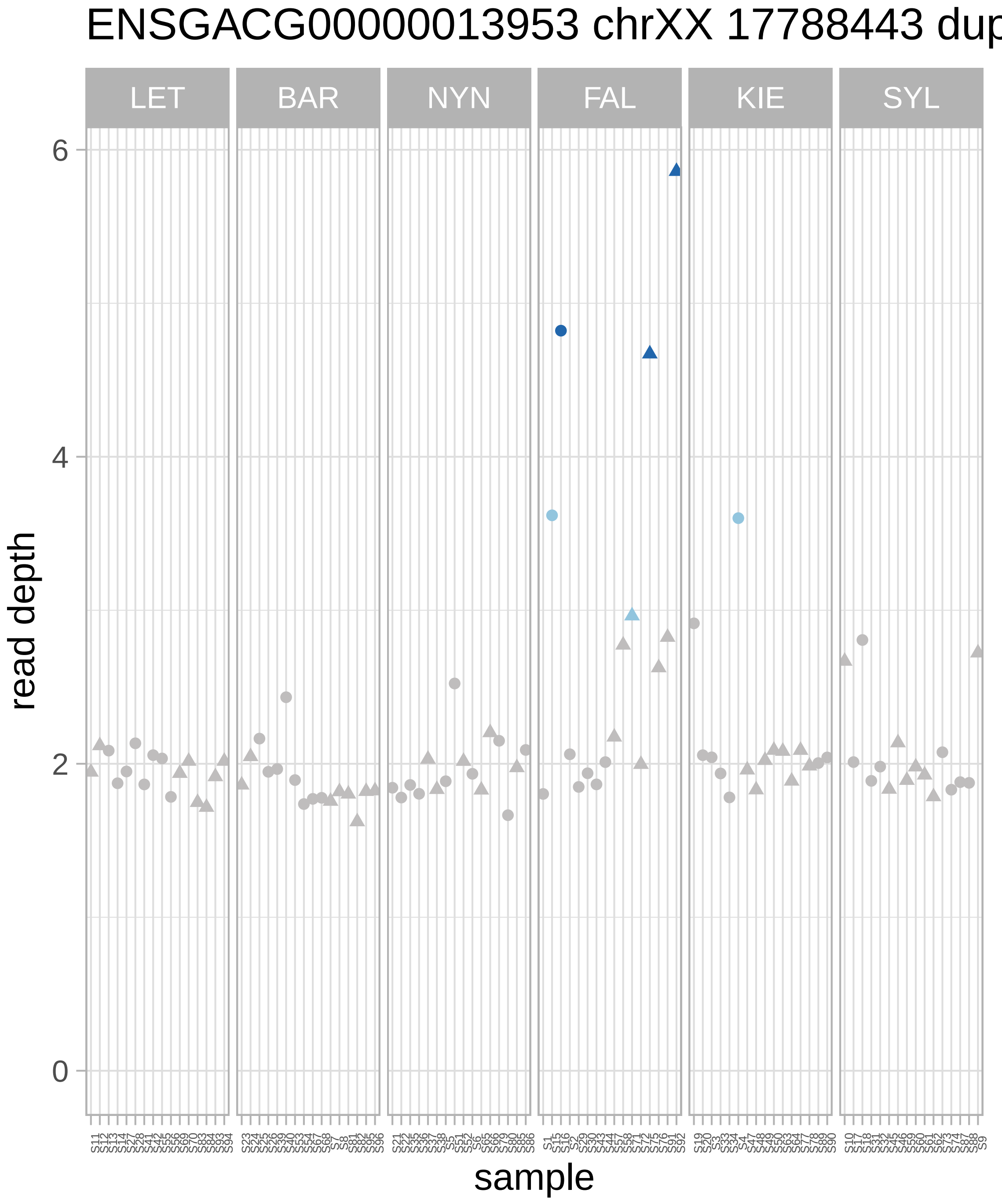
ENSGACG00000013953 chrXX 17788443 dup
LET
BAR
NYN
FAL
KIE
SYL
read depth
0
2
4
6
S11 S12 S13 S14 S27 S28 S41 S42 S55 S56 S69 S70 S83 S84 S93 S94
S23 S24 S25 S26 S39 S40 S53 S54 S67 S68 S7 S8 S81 S82 S95 S96
S21 S22 S35 S36 S37 S38 S5 S51 S52 S6 S65 S66 S79 S80 S85 S86
S1 S15 S16 S2 S29 S30 S43 S44 S57 S58 S71 S72 S75 S76 S91 S92
S19 S20 S3 S33 S34 S4 S47 S48 S49 S50 S63 S64 S77 S78 S89 S90
S10 S17 S18 S31 S32 S45 S46 S59 S60 S61 S62 S73 S74 S87 S88 S9
sample

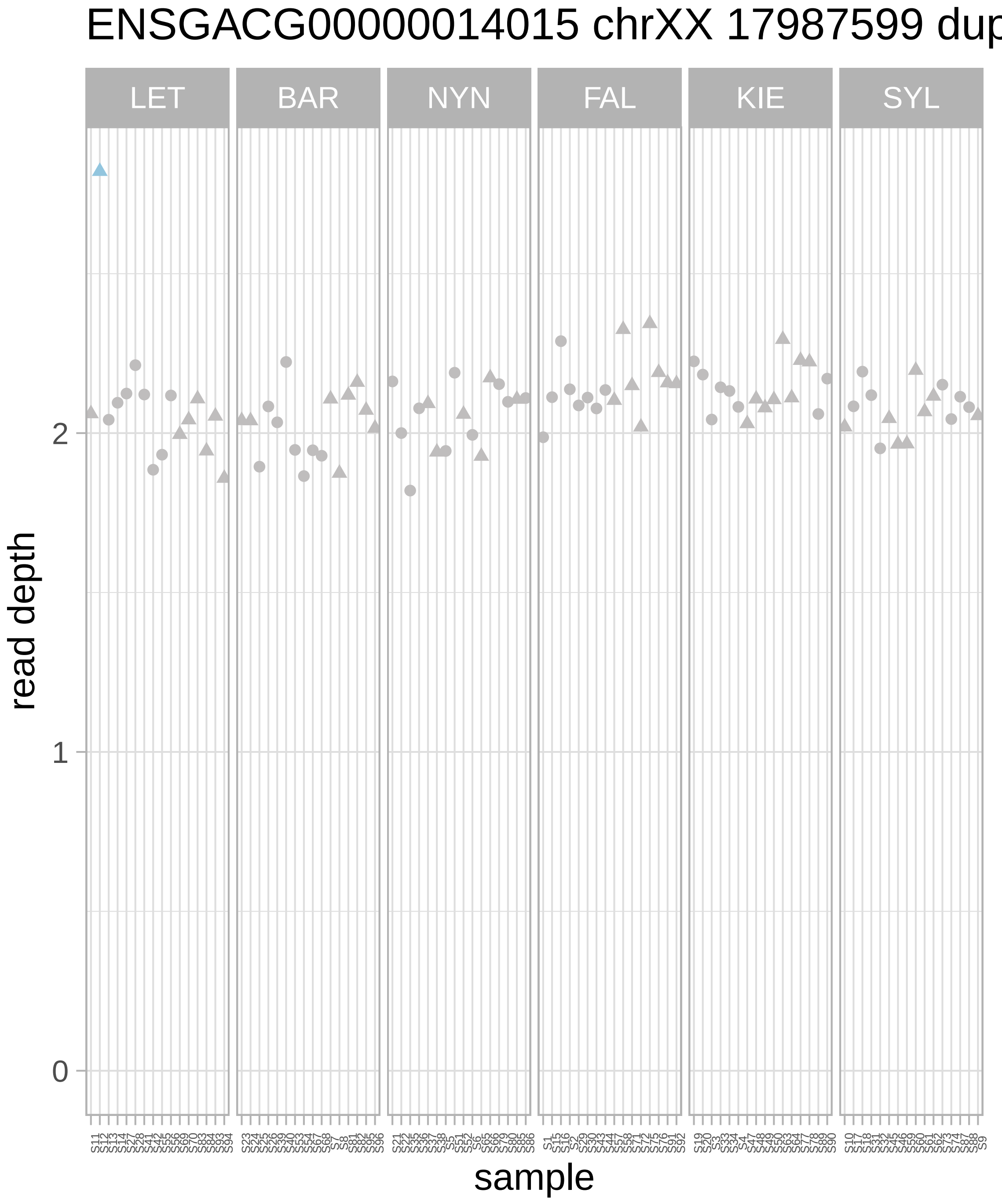
ENSGACG00000014015 chrXX 17987599 dup
LET
BAR
NYN
FAL
KIE
SYL
read depth
2
1
0
sample

ENSGACG00000001694 chrXXI 786253 dup

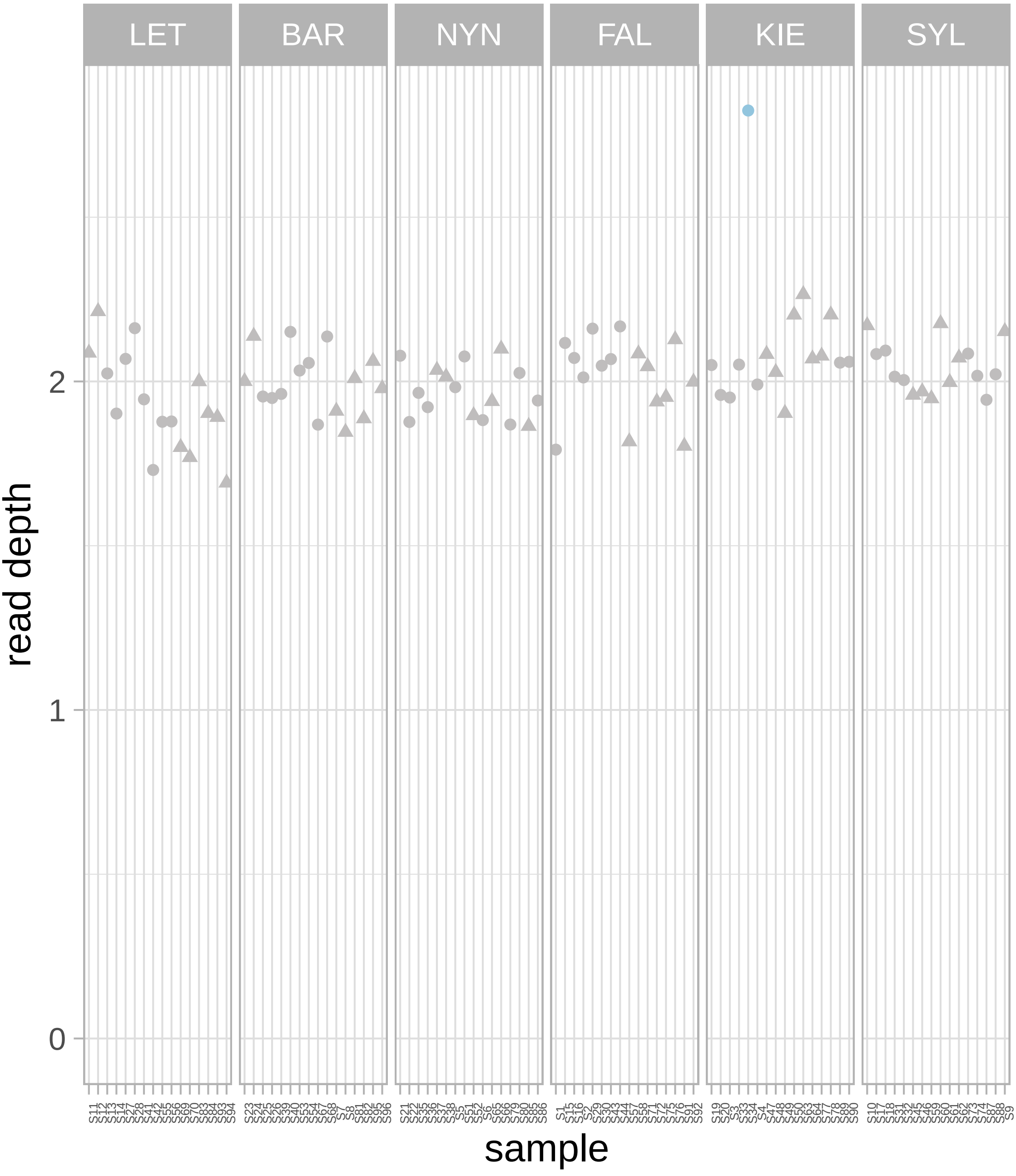

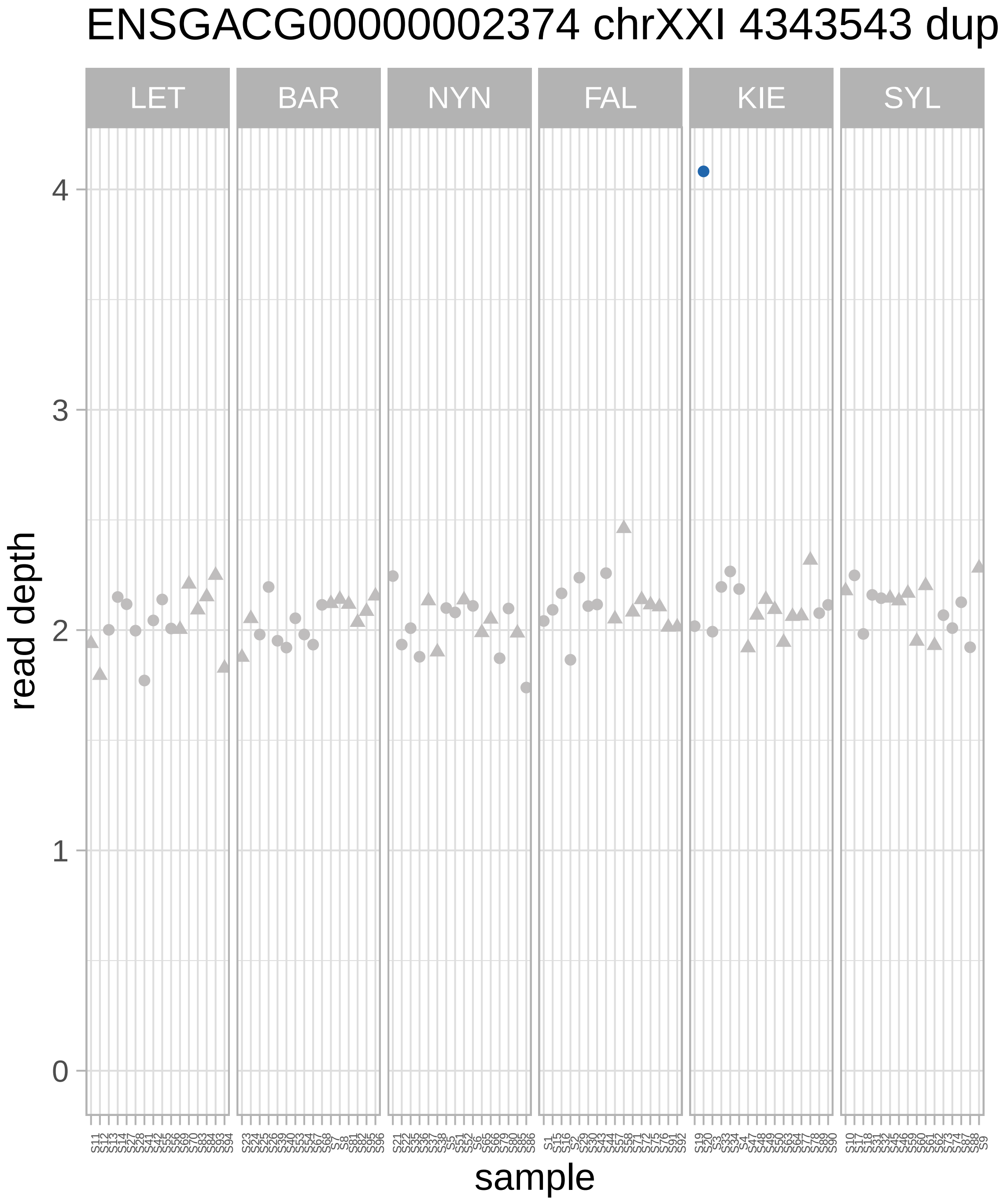
ENSGACG00000002374 chrXXI 4343543 dup
LET
BAR
NYN
FAL
KIE
SYL
read depth
0
1
2
3
4
sample

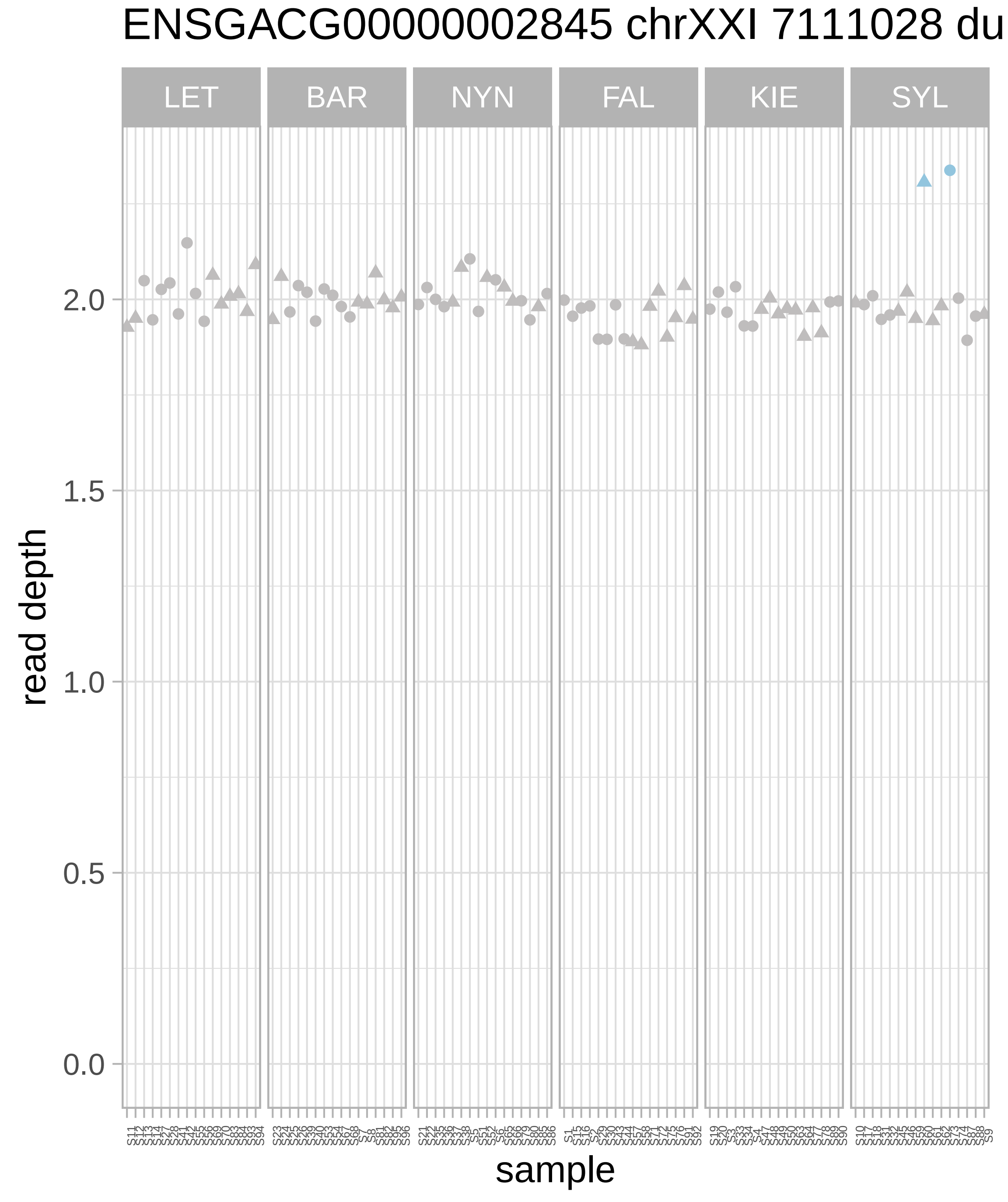
ENSGACG00000002845 chrXXI 7111028 du
LET
BAR
NYN
FAL
KIE
SYL
read depth
2.0
1.5
1.0
0.5
0.0
S11 S12 S13 S14 S27 S28 S41 S42 S55 S56 S69 S70 S83 S84 S93 S94
S23 S24 S25 S26 S39 S40 S53 S54 S67 S68 S7 S8 S81 S82 S95 S96
S21 S22 S35 S36 S37 S38 S5 S51 S52 S6 S65 S66 S79 S80 S85 S86
S1 S15 S16 S2 S29 S30 S43 S44 S57 S58 S71 S72 S75 S76 S91 S92
S19 S20 S3 S33 S34 S4 S47 S48 S49 S50 S63 S64 S77 S78 S89 S90
S10 S17 S18 S31 S32 S45 S46 S59 S60 S61 S62 S73 S74 S87 S88 S9
sample

ENSGACG00000003254 chrXXI 7759787 dup

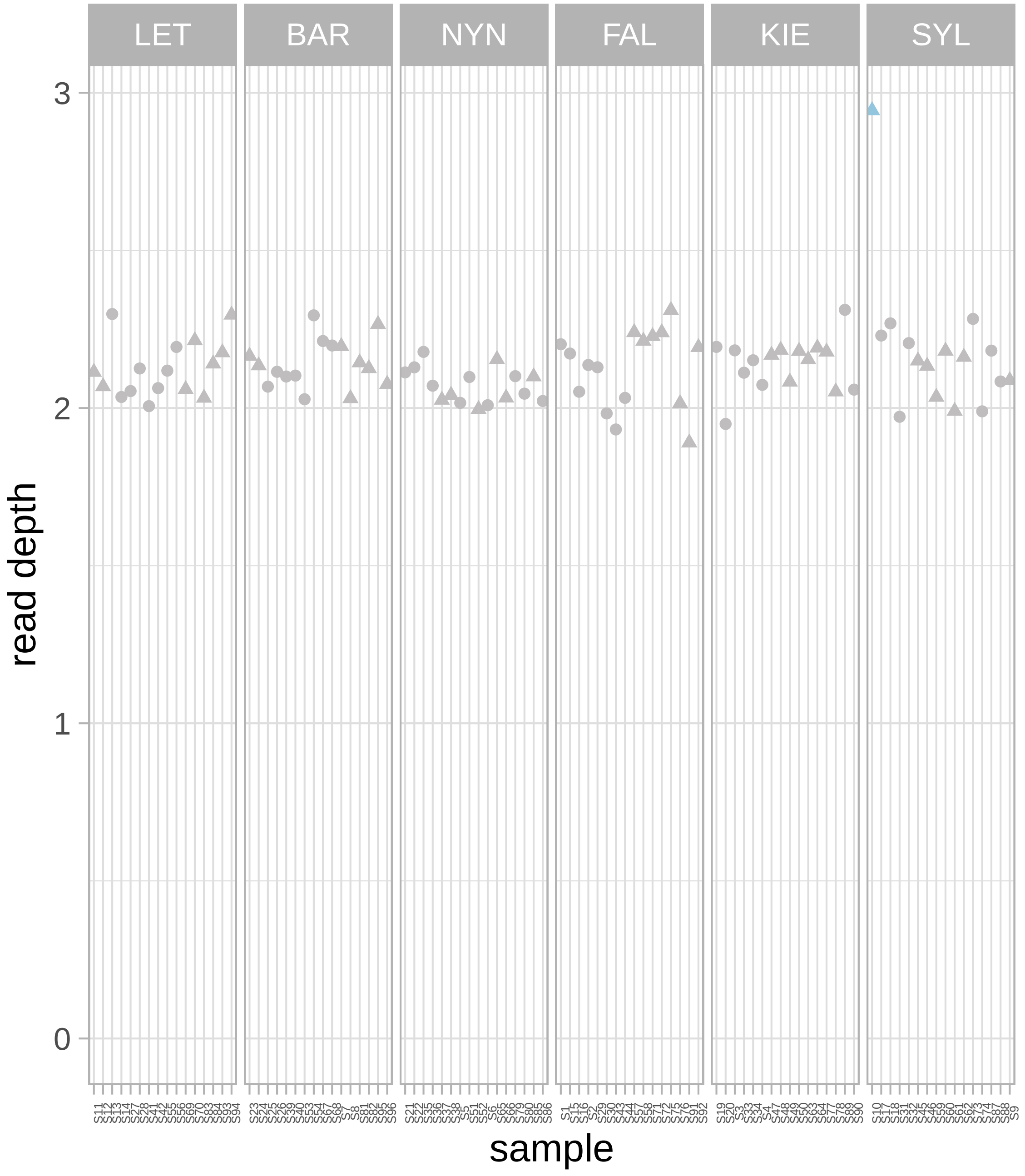

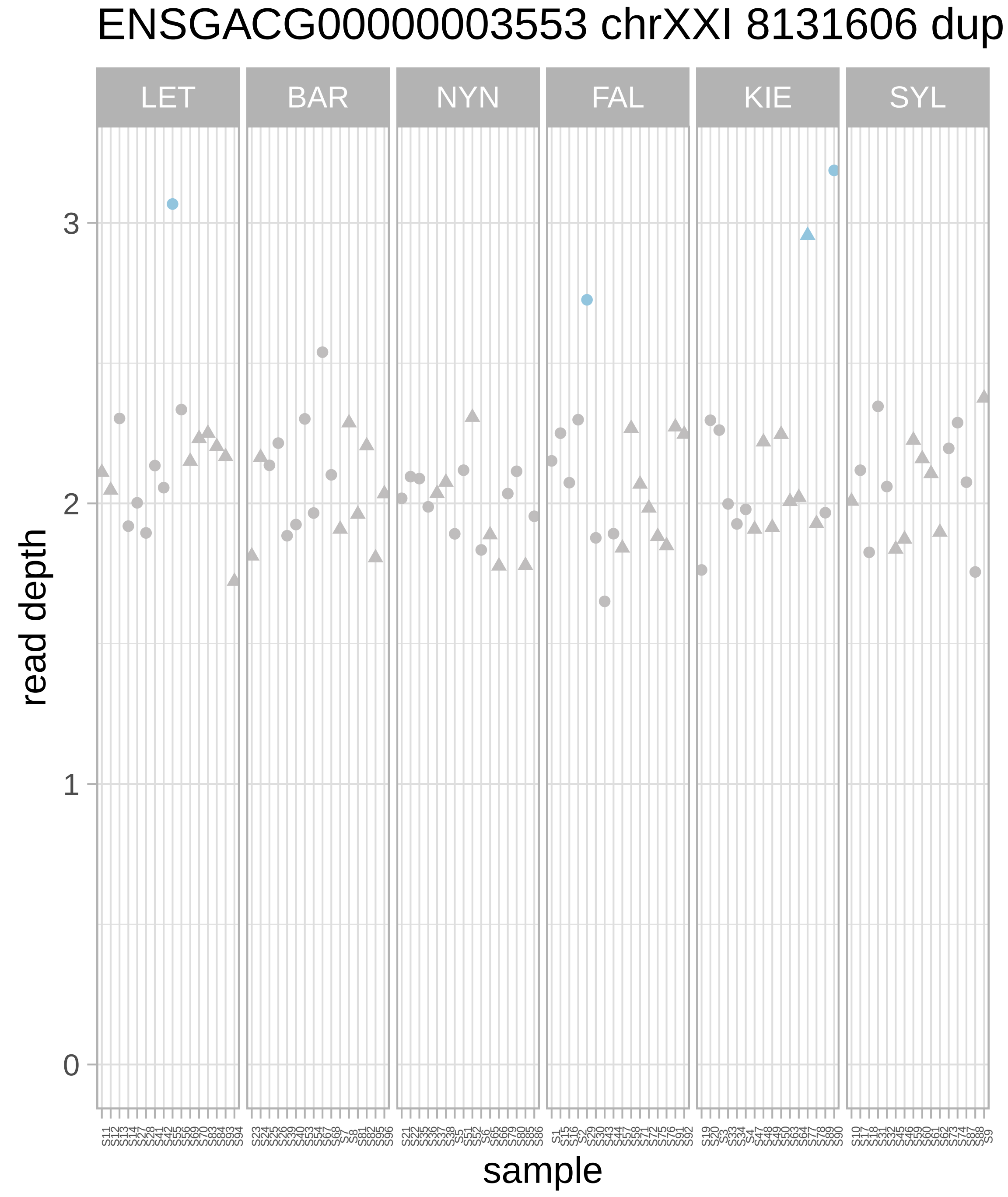

ENSGACG00000003553 chrXXI 8131606 dup
LET
BAR
NYN
FAL
KIE
SYL
read depth
3
2
1
0
sample

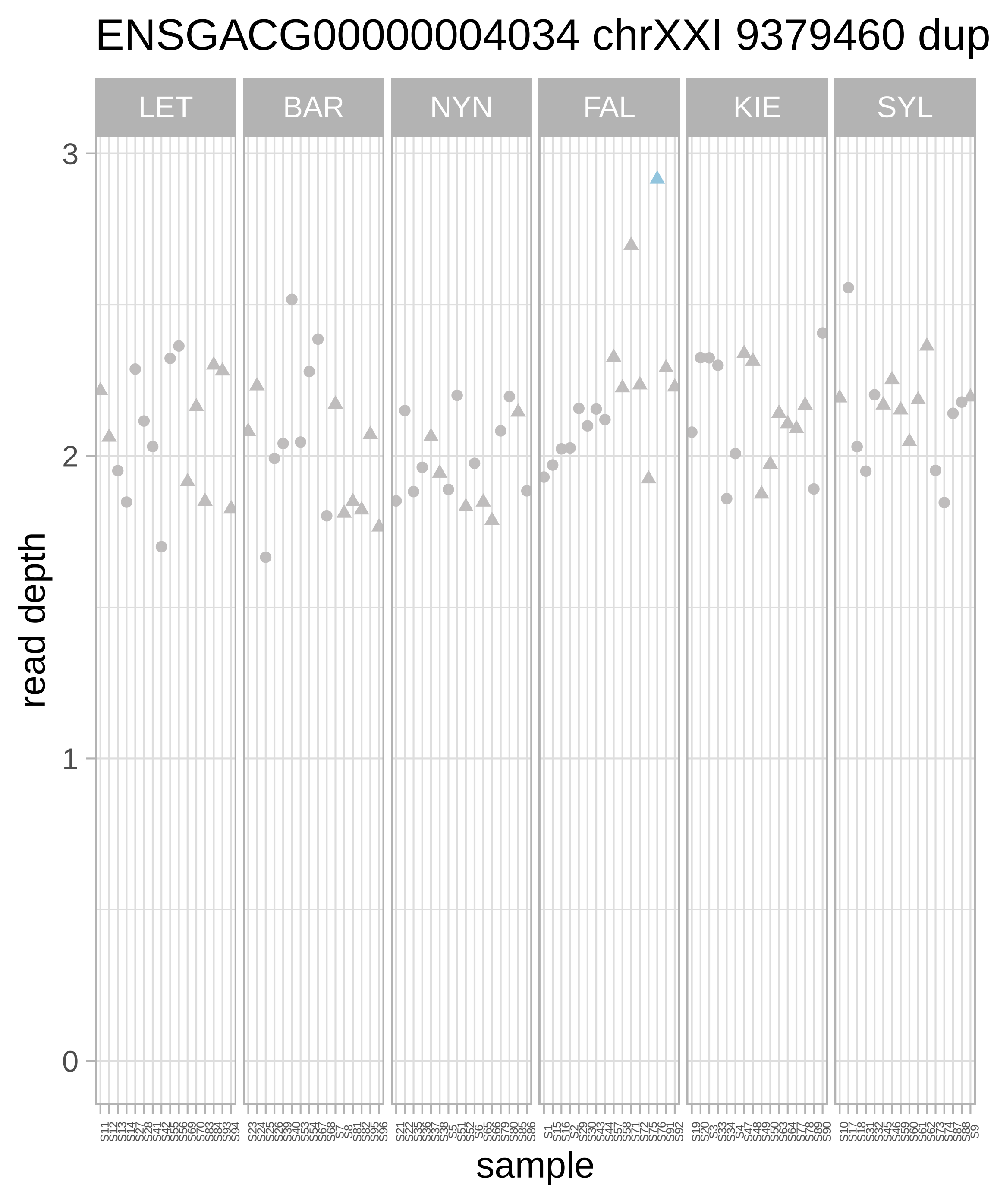
ENSGACG00000004034 chrXXI 9379460 dup
LET
BAR
NYN
FAL
KIE
SYL
read depth
0
1
2
3
sample

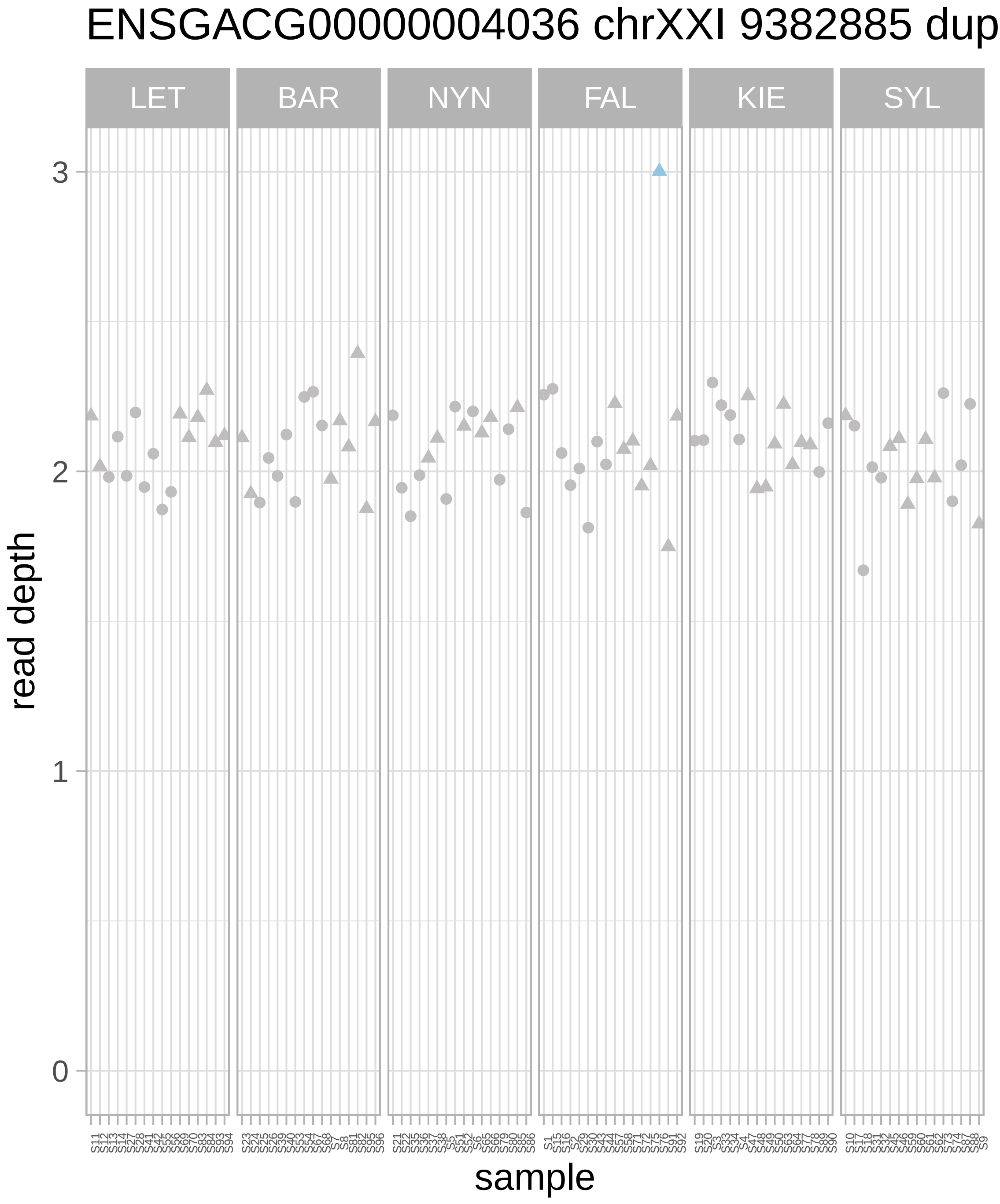
ENSGACG00000004036 chrXXI 9382885 dup
LET
BAR
NYN
FAL
KIE
SYL
read depth
0
1
2
3
sample

ENSGACG00000004052 chrXXI 9389962 dup

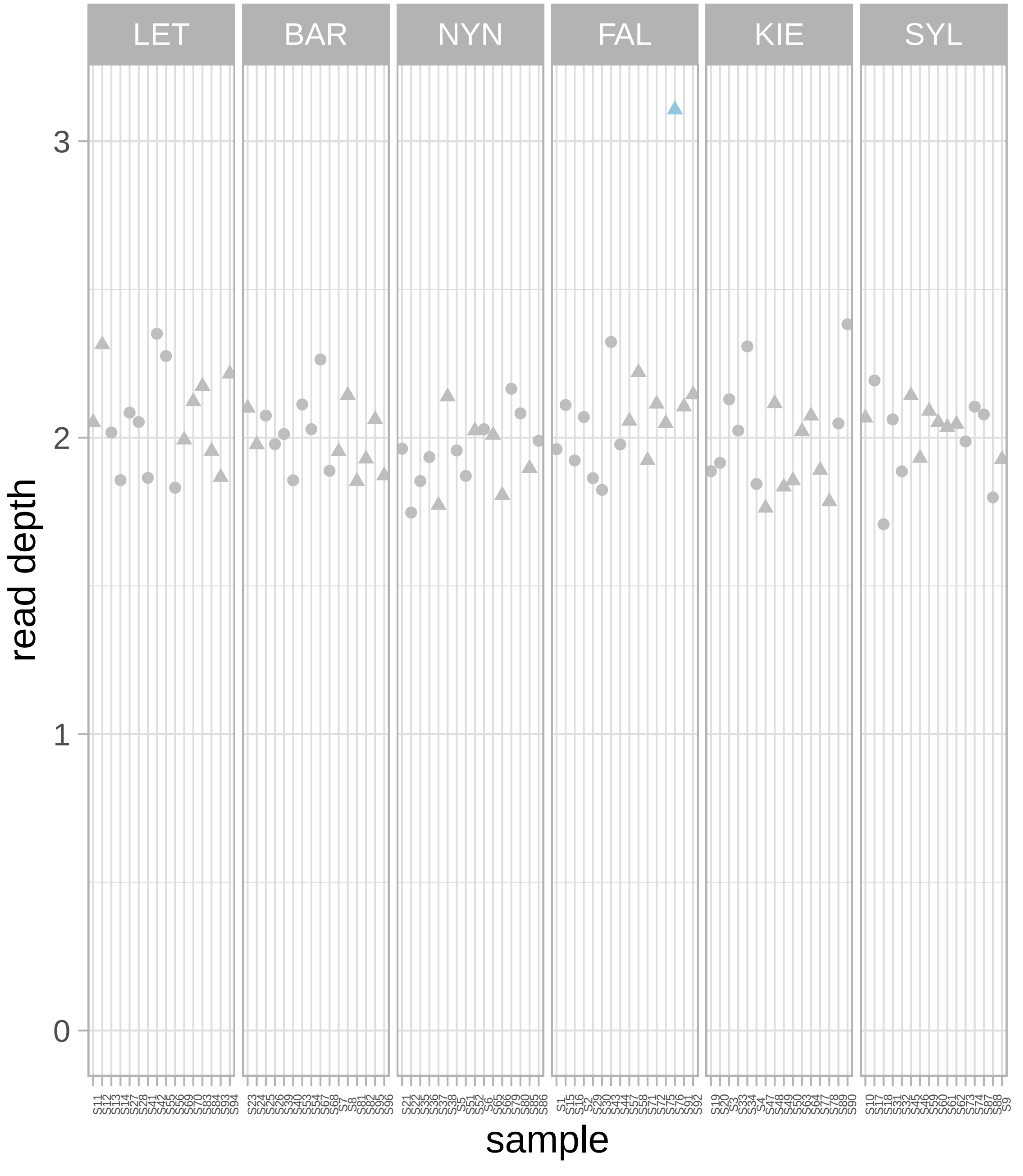

ENSGACG00000004059 chrXXI 9394951 dup

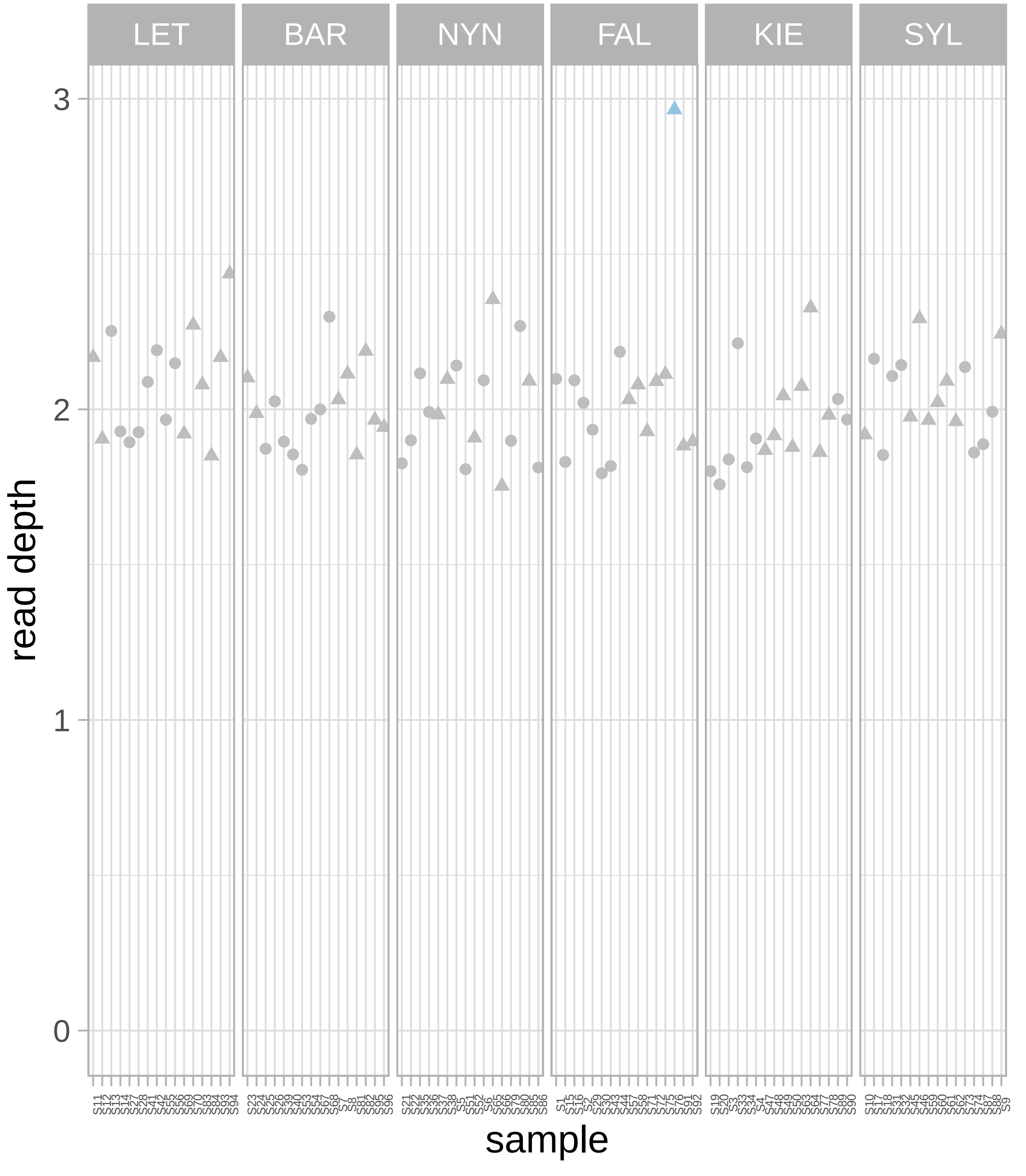

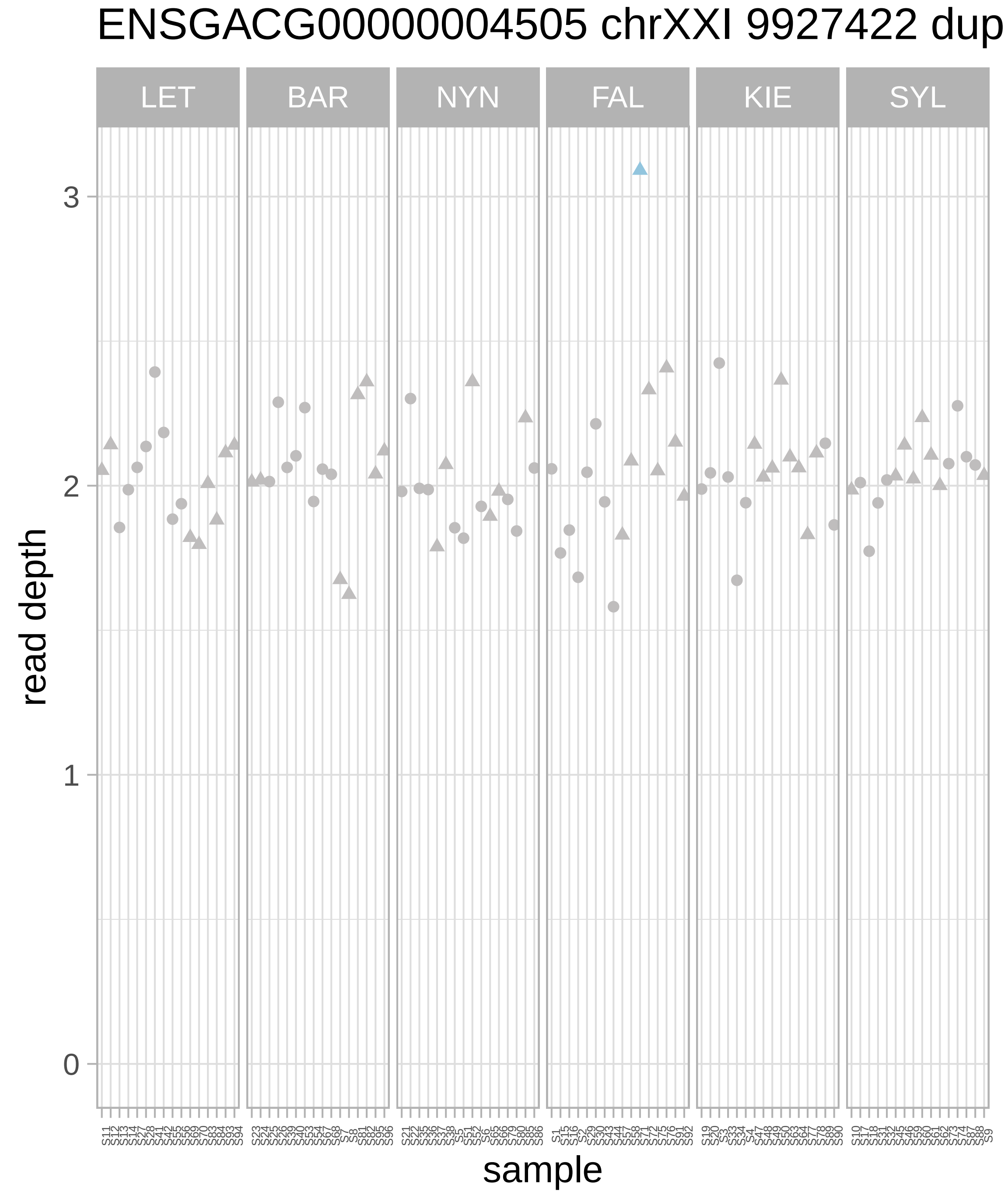
ENSGACG00000004505 chrXXI 9927422 dup
LET
BAR
NYN
FAL
KIE
SYL
read depth
0
1
2
3
sample
S11 S12 S13 S14 S27 S28 S41 S42 S55 S56 S69 S70 S83 S84 S93 S94
S23 S24 S25 S26 S39 S40 S53 S54 S67 S68 S7 S8 S81 S82 S95 S96
S21 S22 S35 S36 S37 S38 S5 S51 S52 S6 S65 S66 S79 S80 S85 S86
S1 S15 S16 S2 S29 S30 S43 S44 S57 S58 S71 S72 S75 S76 S91 S92
S19 S20 S3 S33 S34 S4 S47 S48 S49 S50 S63 S64 S77 S78 S89 S90
S10 S17 S18 S31 S32 S45 S46 S59 S60 S61 S62 S73 S74 S87 S88 S9

ENSGACG00000004998 chrXXI 10874433 du

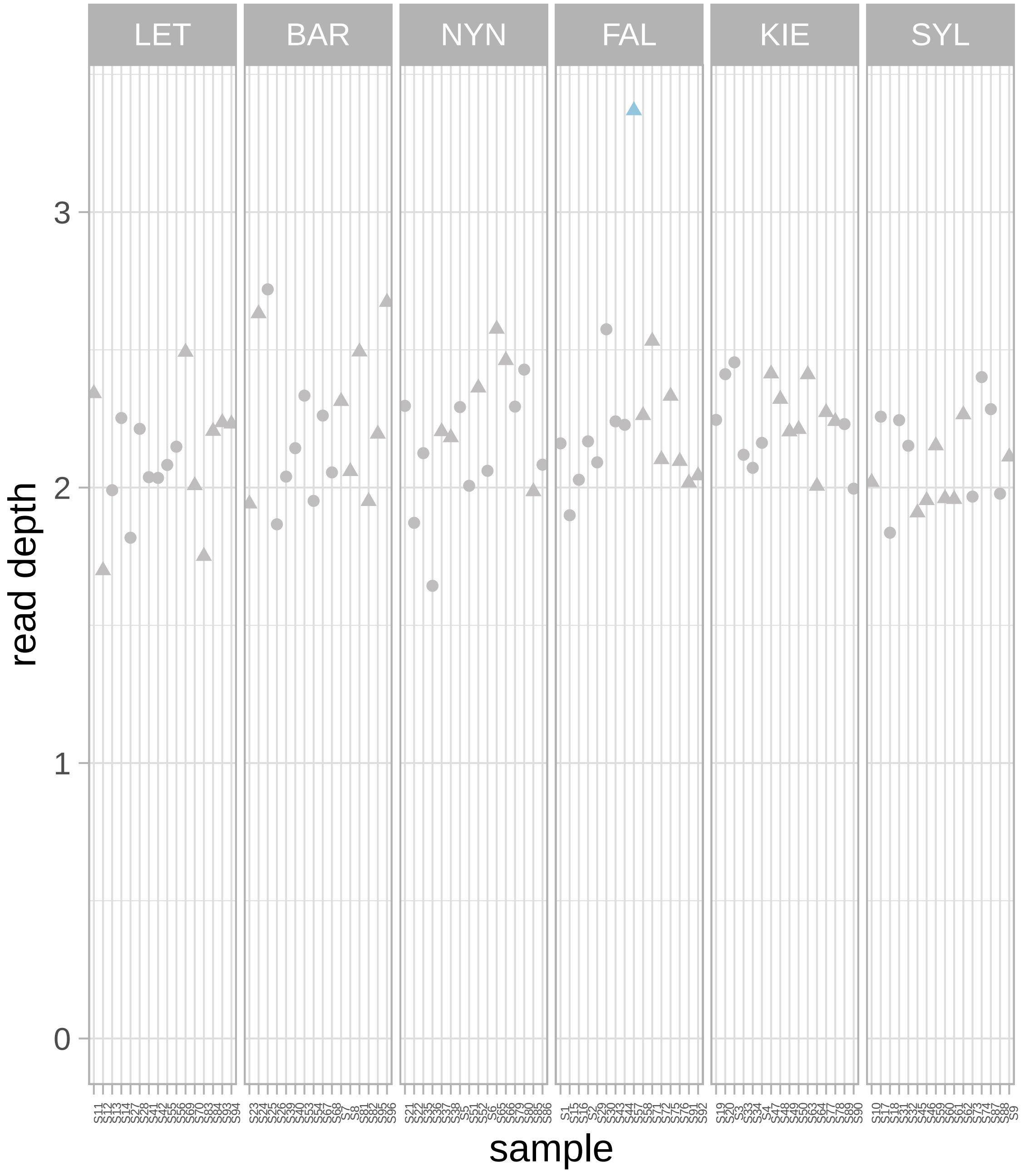

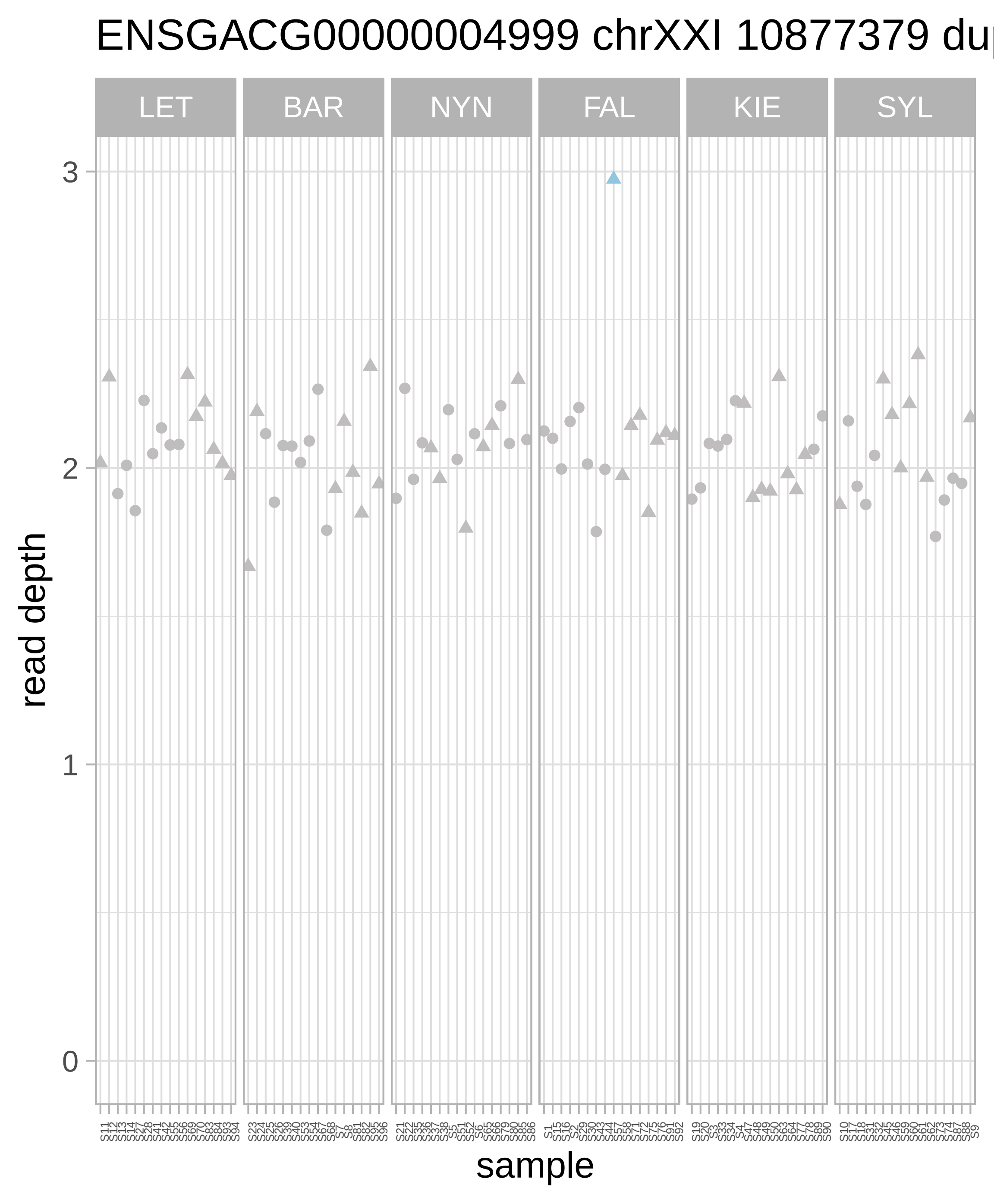
ENSGACG00000004999 chrXXI 10877379 du
LET
BAR
NYN
FAL
KIE
SYL
3
2
1
0
read depth
sample

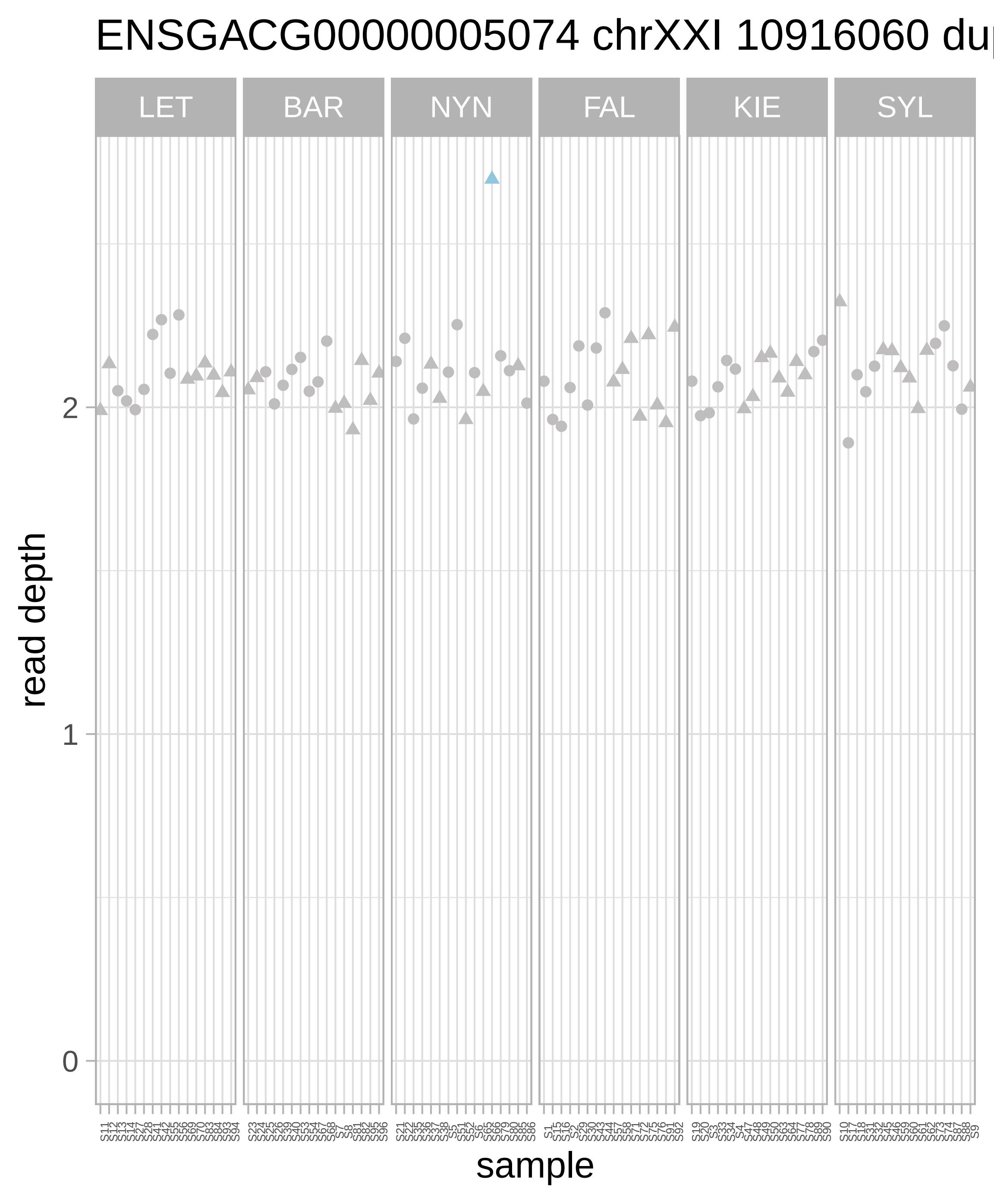

ENSGACG00000005074 chrXXI 10916060 du
LET
BAR
NYN
FAL
KIE
SYL
read depth
0
1
2
sample
